# Supplementary material for: TMPRSS11B promotes an acidified microenvironment and immune suppression in squamous lung cancer
Source: EMBO Rep. 2025 Nov 10;26(24):6346–79. doi: 10.1038/s44319-025-00631-1 (PMC12714794; doi:10.1038/s44319-025-00631-1)
Supplement: Supplementary file 1 — Appendix [file 44319_2025_631_MOESM1_ESM.pdf]

Appendix

TMPRSS11B promotes an acidified microenvironment and immune suppression in squamous lung cancer

Hari Shankar Sunil, Jean Clemenceau, Anthony Grichuk, Isabel Barnfather, Sumanth R. Nakkireddy, Luke Izzo, Qiang Feng, William Hartnett, Bret M. Evers, Lisa Thomas, Indhumathy Subramaniyan, Li Li, William T. Putnam, Steven Hepensteil, Jingfei Zhu, Barrett Updegraff, John D. Minna, Ralph J. DeBerardinis, Tae Hyun Hwang, Jinming Gao, Trudy G. Oliver and Kathryn A. O'Donnell

Table of Content

Appendix Figure S1………………………………….2

Appendix Figure S2………………………………….5

Appendix Figure S3………………………………….6

Appendix Figure S1.

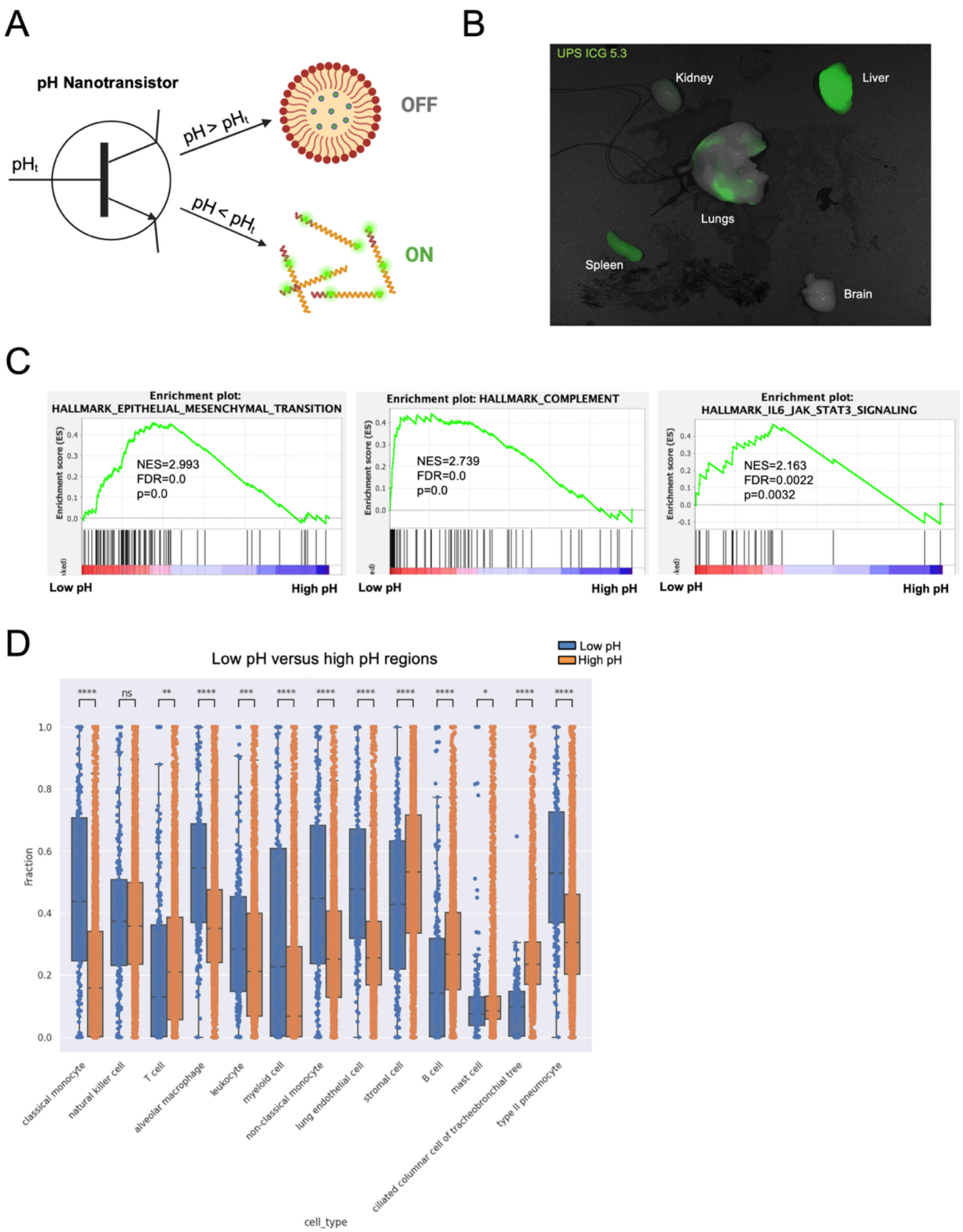

Appendix Figure S1. Low pH/acidic regions in the tumor microenvironment show enrichment for oncogenic signaling and immune cell signatures.

A) Schematic representation of the mechanism of action for the ultra pH sensitive (UPS) nanoparticle, adapted from Feng et al, *Accounts of chemical research*, 2019 (made with BioRender). **B)** Representative fluorescence ICG (UPS 5.3) images of the SNL mouse lung along with liver, kidney, heart, spleen and brain. **C)** Gene set enrichment analysis (GSEA) of the low pH vs. high pH spatial data with normalized enrichment scores (NES), false discovery rate (FDR) and p values for the indicated immune cell gene signatures. The nominal *P* and FDR values were obtained from the "GSEA Preranked" tool (from Broad Institute) using a weighted scoring scheme. Gene sets were evaluated based on the default normalized enrichment score method, and statistical significance was determined by bootstrapping with 1000 permutations. **D)** Quantification of immune cell populations in low pH vs. high pH using cell deconvolution analysis of the spatial data. A two-sided Mann-Whitney-Wilcoxon test was used for the statistical analysis (low pH n=224, high pH n=4050, biological replicates), ****P = 2.60E-33 (Classical monocyte), **P = 0.00022 (T cell), ****P = 3.78E-27 (Alveolar macrophage), ***P = 2.88E-05 (Leukocyte), ****P = 1.59E-09 (Myeloid cell), ****P = 4.58E-18 (Non-classical monocyte), ****P = 1.31E-43 (Lung endothelial cell), ****P = 8.20E-09 (Stromal cell), ****P = 2.32E-14 (B cell), *P = 0.00143 (Mast cell), ****P = 5.48E-71 (Ciliated columnar cell of tracheobronchial tree), ****P = 2.89E-32 (Type II pneumocyte). The box plots represent the distribution of values for each group, extending from the 25th percentile to the 75th percentile; low pH: Classical monocyte (minima=0, median=0.43792, maxima=1, Q1=0.24656, Q3=0.70636), Natural killer cell (minima=0, median=0.37360, maxima=0.92375, Q1=0.23133, Q3=0.50830), T cell (minima=0, median=0.13112, maxima=0.90189, Q1=0.00121, Q3=0.36148), Alveolar macrophages (minima=0, median=0.54638, maxima=1, Q1=0.36868, Q3=0.68845), Leukocyte (minima=0, median=0.28476, maxima=0.90807, Q1=0.14797, Q3=0.45201), Myeloid cell (minima=0, median=0.22636, maxima=1, Q1=0.00249, Q3=0.60839), Non-classical monocyte (minima=0, median=0.44773, maxima=1, Q1=0.23620, Q3=0.68289), Lung endothelial cell (minima=0, median=0.47805, maxima=1, Q1=0.31878, Q3=0.67075), Stromal cell (minima=0, median=0.42868, maxima=1, Q1=0.21909, Q3=0.63408), B cell (minima=0, median=0.14257, maxima=0.79256, Q1=0.00157, Q3=0.31796), Mast cell (minima=0, median=0.07522, maxima=0.26706, Q1=0.03823, Q3=0.12976), Ciliated columnar cell of tracheobronchial tree (minima=0, median=0.09895, maxima=0.36530, Q1=0.00237, Q3=0.14754), Type II pneumocyte (minima=0, median=0.52799, maxima=1, Q1=0.36919, Q3=0.72546); high pH: Classical monocyte (minima=0, median=0.15947, maxima=0.85122, Q1=0.00219, Q3=0.34180), Natural killer cell (minima=0, median=0.35758, maxima=0.89564, Q1=0.23479, Q3=0.49913), T cell (minima=0, median=0.21076, maxima=0.87919, Q1=0.05677, Q3=0.38574), Alveolar macrophages (minima=0, median=0.35095, maxima=0.83039, Q1=0.24057, Q3=0.47650), Leukocyte (minima=0, median=0.21140, maxima=0.89782, Q1=0.06787, Q3=0.39985), Myeloid cell (minima=0, median=0.06771, maxima=0.72710, Q1=0.00127, Q3=0.29160), Non-classical monocyte (minima=0, median=0.25169, maxima=0.82674, Q1=0.12758, Q3=0.40724), Lung endothelial cell (minima=0, median=0.25640, maxima=0.68218, Q1=0.16750, Q3=0.37337), Stromal cell (minima=0, median=0.53181, maxima=1, Q1=0.33560, Q3=0.71715), B cell (minima=0, median=0.26728, maxima=0.77288, Q1=0.15268, Q3=0.40076), Mast cell

(minima=0, median=0.08428, maxima=0.24396, Q1=0.0589, Q3=0.1329), Ciliated columnar cell of tracheobronchial tree (minima=0, median=0.23397, maxima=0.51008, Q1=0.17095, Q3=0.30660), Type II pneumocyte (minima=0, median=0.30479, maxima=0.84486, Q1=0.20332, Q3=0.45993).

Appendix Figure S2.

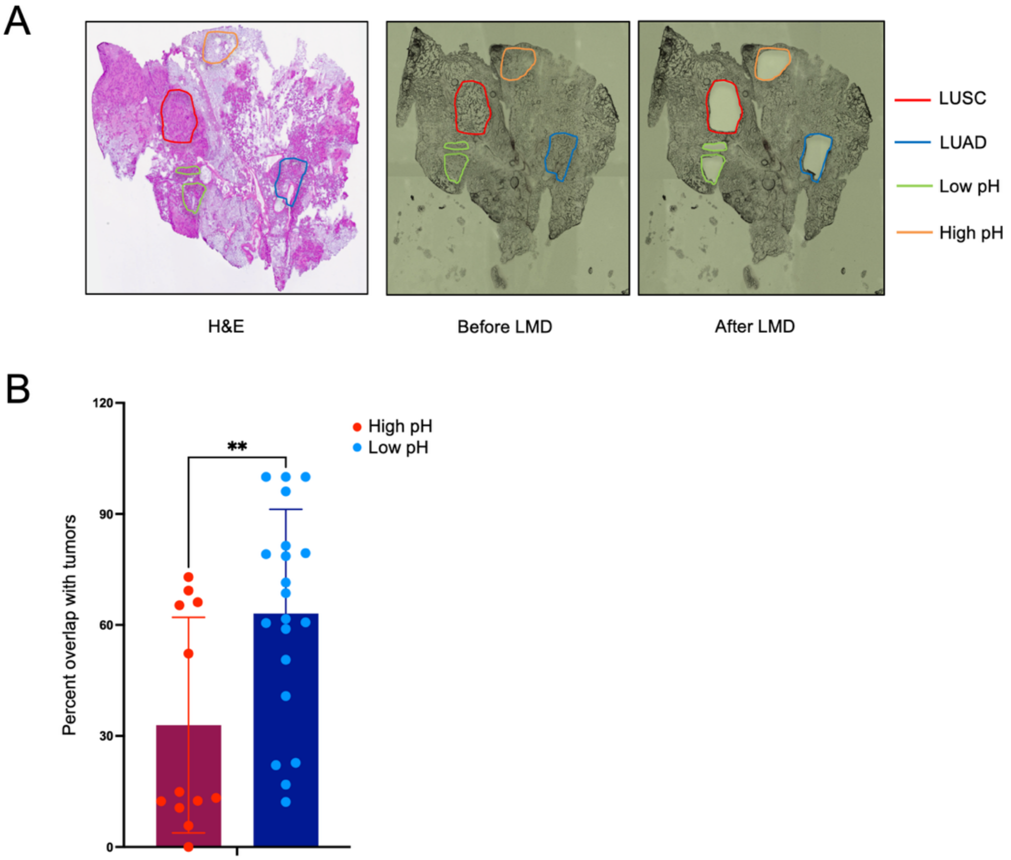

Appendix Figure S2. Laser capture microdissection of the regions of interest (LUSC, LUAD, low pH and high pH) using H&E as reference.

A) Left, Representative H&E image of the SNL lung section (8 months post infection with adeno cre virus) along with corresponding bright field images of the serial section used for LMD, before and after the laser mediated dissection, with annotations. **B)** Quantification of the overlap between low or high pH regions and the tumor regions (low pH n=20, high pH n=12, n=2 mice, biological replicates). Unpaired t test with Welch's correction was used for the analysis, P = 0.0087. Plot represents mean ± SD.

Appendix Figure S3.

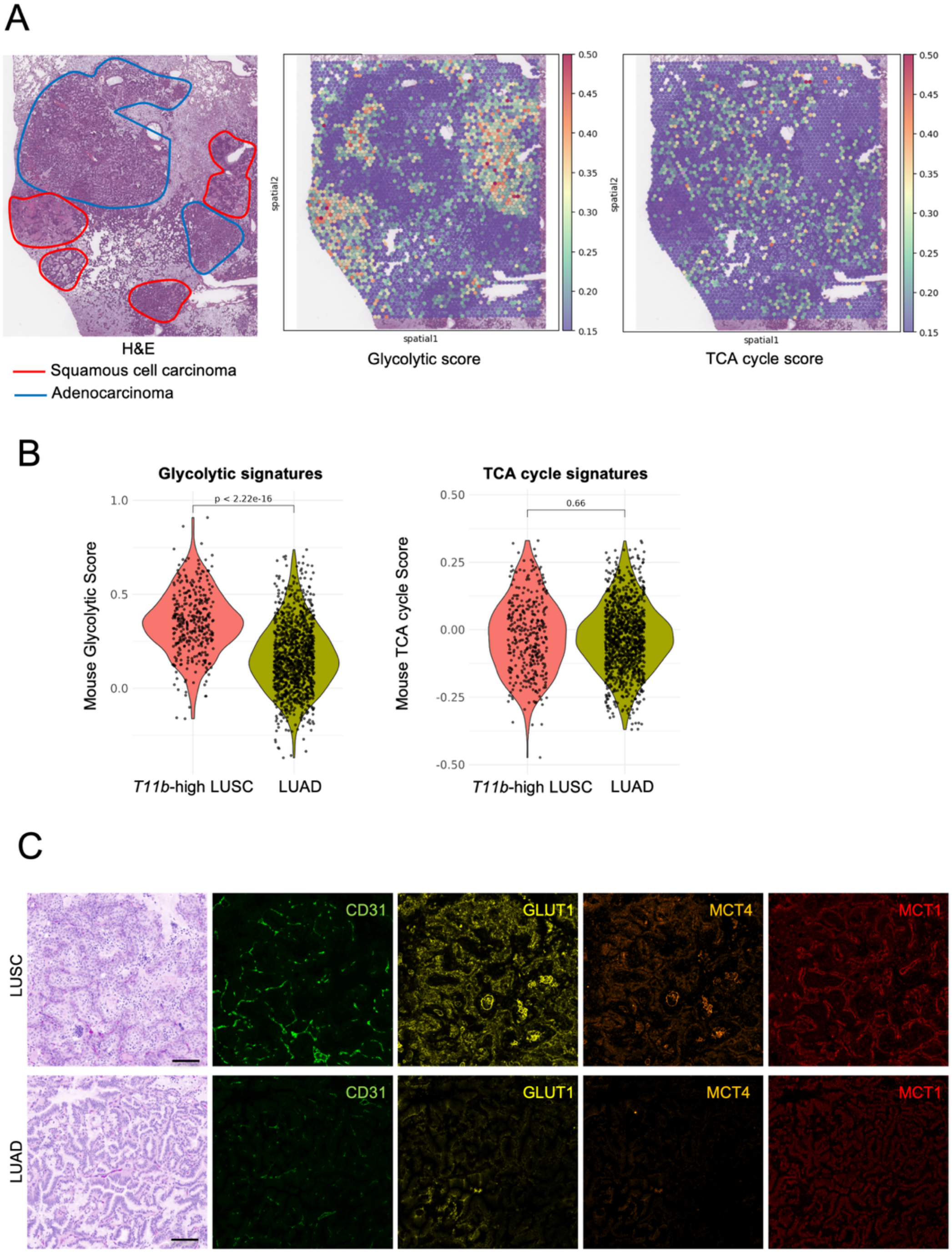

Appendix Figure S3. *Tmprss11b*-high squamous tumors show elevated glycolytic metabolism.

A) H&E image of the lung section from 3A), annotated with regions of LUSC and LUAD, (left) and spatial plots from the transcriptomic data depicting the distribution of the indicated gene signatures (right). **B)** Violin plots representing the enrichment of the indicated gene signatures in *Tmprss11b*-high LUSC and LUAD. A two-tailed Wilcoxon Rank Sum test was used for the statistical analysis (n=1492, biological replicates). $P < 2.22e-16$ (Glycolytic signatures), $P = 0.66$ (TCA cycle signatures). **C)** Representative H&E images and multiplex immunohistochemistry (IHC-F) for CD31, GLUT1, MCT4 and MCT1 in SNL LUSC (top) and mucinous LUAD (bottom). Scale bar, 100μm.
