# Supplementary material for: TMPRSS11B promotes an acidified microenvironment and immune suppression in squamous lung cancer
Source: EMBO Rep. 2025 Nov 10;26(24):6346–79. doi: 10.1038/s44319-025-00631-1 (PMC12714794; doi:10.1038/s44319-025-00631-1)
Supplement: Supplementary file 2 — Table EV1 [file 44319_2025_631_MOESM2_ESM.pdf]

Table EV1: Top 20 differentially expressed genes used for the immune cell deconvolution analysis

	B cell	T cell	alveolar macrophage	ciliated columnar cell of tracheobronchial tree	classical monocyte	leukocyte	lung endothelial cell	mast cell	myeloid cell	natural killer cell	non-classical monocyte	stromal cell	type II pneumocyte
0	Cd74	Rpl13a	Chi3l3	Cbr2	Lyz2	Tyrobp	Ramp2	Tyrobp	Gpx1	Ccl5	Tyrobp	Bgn	Sftpc
1	Cd79a	Uba52	Lyz2	Chchd10	Lyz1	Sepx1	Egfl7	Tmsb4x	Sh3bgrl3	Nkg7	Fcer1g	Plac9	Sftpb
2	H2-Aa	Rps3	Lyz1	Tppp3	Tyrobp	S100a8	Cldn5	Fxyd5	Cd74	AW112010	Gngt2	Gsn	Sftpa1
3	H2-Ab1	Rpl19	Ccl6	Sec14l3	Ctss	Tmsb4x	Ly6a	Srgn	Rps9	Gzma	Lst1	Mgp	Sftpd
4	H2-Eb1	Rplp2	Ftl1	Dynlrb2	Ifitm3	S100a9	Pecam1	Fau	Ctss	Fcer1g	Gpx1	Cd81	Wfdc2
5	Cd79b	Eef1a1	Laptm5	1700016K19Rik	Psap	Cd52	Tspan7	Fcer1g	H2-DMb1	Tyrobp	Lyz2	Col3a1	Cxcl15
6	Rps19	Rplp0	Ctss	1110017D15Rik	Fcer1g	S100a11	Ly6e	Arhgdib	Fau	Klrd1	B2m	Serping1	Slc34a2
7	Rps15a	Rps13	Cd44	Cd24a	Coro1a	Fcer1g	Cav2	Ccl6	Ucp2	Klre1	Coro1a	Rarres2	Cbr2
8	Rps27	Rpl18a	Ucp2	Riiad1	Cd52	Dusp1	Epas1	Rpl38	H2-DMa	Cd52	Cebpb	Sparc	Sfta2
9	Rpl17	Rps15a	1600029D21Rik	Dynll1	Lgals3	Fxyd5	Aqp1	Pfn1	Psap	Pfn1	Ctss	Sod3	Lyz2
10	Rpl18a	Rps18	Cd9	Elof1	Plac8	Lst1	Calcrl	Arpc1b	Rps11	H2afz	Lyz1	Gpx3	Cd74
11	Fau	Rpl18	Abcg1	Fam183b	Ms4a6c	Cebpb	Sdpr	Rps23	Rplp0	Klrb1c	Tmsb4x	Fxyd1	Npc2
12	Rpl13a	Rps14	Ear2	Crip2	Alox5ap	Srgn	H2-D1	Actg1	H2-Ab1	Tmsb4x	Fau	Sparcl1	Napsa
13	Ly6e	Rpsa	Ctsd	1700009P17Rik	S100a4	Ccl6	Eng	Taldo1	H2-Aa	Rac2	Ly6e	Igfbp7	Lyz1
14	Rps18	Rps3a	Sepx1	Wfdc2	Ly6e	Fau	Cav1	Hdc	Cd52	Klrk1	Cyba	Pcolce2	Lamp3
15	Rpl32	Rpl32	Lgals3	Tubb4b	Arpc1b	Ftl1	Cdh5	Rps11	Alox5ap	Id2	Ifitm3	Col1a1	Scd1
16	Rps24	Cd3g	Tmsb4x	Ccdc153	Lst1	Alox5ap	Crip2	Rpl23a	Ctsh	Ncr1	Sfpi1	Col1a2	Spint2
17	Rps13	Cd3d	Mpeg1	Tmem212	Ccl9	Il1b	Acvrl1	Ccl3	Coro1a	Ms4a4b	H2-D1	Serpinh1	Alcam
18	Rps16	Rps6	Cybb	Rsph1	Ifitm6	Slpi	Ctla2a	Rps27	Eef1a1	Ptprcap	Pfn1	Prelp	Chchd10
19	Rps20	Rpl11	Mrc1	Nudc	Gpx1	Pglyrp1	Hpgd	Alox5ap	Erp29	Tmsb10	Lsp1	Mxra8	Lgi3
