# Supplementary material for: TMPRSS11B promotes an acidified microenvironment and immune suppression in squamous lung cancer
Source: EMBO Rep. 2025 Nov 10;26(24):6346–79. doi: 10.1038/s44319-025-00631-1 (PMC12714794; doi:10.1038/s44319-025-00631-1)
Supplement: Supplementary file 3 — Table EV2 [file 44319_2025_631_MOESM3_ESM.pdf]

Table EV2. List of MRMs, compound dependent MS/MS parameters and retention times.

S.No	Compound ID	Q1 mass	Q3 mass	Dwell time (ms)	DP	CE	CXP	RT (min)
3	Lactate	89	43	20	-80	-16	-20	2.1
4	13C3-Lactate	92	45.1	20	-80	-16	-20	2.1
