# Supplementary material for: TMPRSS11B promotes an acidified microenvironment and immune suppression in squamous lung cancer
Source: EMBO Rep. 2025 Nov 10;26(24):6346–79. doi: 10.1038/s44319-025-00631-1 (PMC12714794; doi:10.1038/s44319-025-00631-1)
Supplement: Supplementary file 6 — Source data Fig. 1 [file 44319_2025_631_MOESM6_ESM.zip › Figure 1/1A-B/Read Me.rtf]

T11b_m1_I, T11b_m1_II, T11b_m1_III are different taqman probes spanning different regions of the Tmprss11b mRNA
