# Supplementary material for: TMPRSS11B promotes an acidified microenvironment and immune suppression in squamous lung cancer
Source: EMBO Rep. 2025 Nov 10;26(24):6346–79. doi: 10.1038/s44319-025-00631-1 (PMC12714794; doi:10.1038/s44319-025-00631-1)
Supplement: Supplementary file 6 — Source data Fig. 1 [file 44319_2025_631_MOESM6_ESM.zip › Figure 1/1E/Read Me.rtf]

Tmprss11b shRNA1 and shRNA2 from the manuscript are the Tmprss11b shRNA2 and shRNA3 from the data files, respectively.
