# Supplementary material for: TMPRSS11B promotes an acidified microenvironment and immune suppression in squamous lung cancer
Source: EMBO Rep. 2025 Nov 10;26(24):6346–79. doi: 10.1038/s44319-025-00631-1 (PMC12714794; doi:10.1038/s44319-025-00631-1)
Supplement: Supplementary file 7 — Source data Fig. 2 [file 44319_2025_631_MOESM7_ESM.zip › Figure 2/2A/Read Me.rtf]

The represented results have been obtained from un-published RNA sequencing data from the Oliver lab, Duke University.
