# Supplementary material for: TMPRSS11B promotes an acidified microenvironment and immune suppression in squamous lung cancer
Source: EMBO Rep. 2025 Nov 10;26(24):6346–79. doi: 10.1038/s44319-025-00631-1 (PMC12714794; doi:10.1038/s44319-025-00631-1)
Supplement: Supplementary file 8 — Source data Fig. 3 [file 44319_2025_631_MOESM8_ESM.zip › Figure 3/3D-E/GSEA_Broad Institute_Mh_T11b high vs low LUSC/gsea_report_for_na_neg_1723673606674.html]

Report for na\_neg 1723673606674 [GSEA]

| GS  follow link to MSigDB | GS DETAILS | SIZE | ES | NES | NOM p-val | FDR q-val | FWER p-val | RANK AT MAX | LEADING EDGE || 1 | HALLMARK\_BILE\_ACID\_METABOLISM | Details ... | 28 | -0.35 | -1.53 | 0.048 | 0.530 | 0.650 | 1865 | tags=75%, list=46%, signal=137% |
| 2 | HALLMARK\_SPERMATOGENESIS | Details ... | 19 | -0.40 | -1.53 | 0.053 | 0.266 | 0.652 | 395 | tags=26%, list=10%, signal=29% |
| 3 | HALLMARK\_UNFOLDED\_PROTEIN\_RESPONSE | Details ... | 34 | -0.30 | -1.37 | 0.112 | 0.455 | 0.949 | 1178 | tags=47%, list=29%, signal=66% |
| 4 | HALLMARK\_KRAS\_SIGNALING\_DN | Details ... | 41 | -0.26 | -1.26 | 0.167 | 0.574 | 0.993 | 370 | tags=22%, list=9%, signal=24% |
| 5 | HALLMARK\_ESTROGEN\_RESPONSE\_EARLY | Details ... | 69 | -0.20 | -1.11 | 0.308 | 0.833 | 1.000 | 998 | tags=33%, list=24%, signal=43% |
| 6 | HALLMARK\_ESTROGEN\_RESPONSE\_LATE | Details ... | 65 | -0.18 | -1.00 | 0.445 | 0.997 | 1.000 | 796 | tags=28%, list=19%, signal=34% |
| 7 | HALLMARK\_ANDROGEN\_RESPONSE | Details ... | 35 | -0.20 | -0.95 | 0.548 | 0.995 | 1.000 | 928 | tags=31%, list=23%, signal=40% |
| 8 | HALLMARK\_PROTEIN\_SECRETION | Details ... | 28 | -0.22 | -0.93 | 0.552 | 0.924 | 1.000 | 1603 | tags=54%, list=39%, signal=87% |
| 9 | HALLMARK\_PEROXISOME | Details ... | 23 | -0.23 | -0.92 | 0.578 | 0.841 | 1.000 | 1518 | tags=48%, list=37%, signal=76% |
| 10 | HALLMARK\_E2F\_TARGETS | Details ... | 37 | -0.19 | -0.90 | 0.592 | 0.790 | 1.000 | 1162 | tags=35%, list=28%, signal=49% |
| 11 | HALLMARK\_OXIDATIVE\_PHOSPHORYLATION | Details ... | 47 | -0.16 | -0.81 | 0.738 | 0.872 | 1.000 | 1983 | tags=53%, list=48%, signal=102% |
| 12 | HALLMARK\_MITOTIC\_SPINDLE | Details ... | 44 | -0.16 | -0.79 | 0.753 | 0.827 | 1.000 | 820 | tags=25%, list=20%, signal=31% |
| 13 | HALLMARK\_G2M\_CHECKPOINT | Details ... | 41 | -0.16 | -0.75 | 0.816 | 0.817 | 1.000 | 3455 | tags=100%, list=84%, signal=638% |
Table: Gene sets enriched in phenotype **na**[plain text format]****

  
