# Supplementary material for: TMPRSS11B promotes an acidified microenvironment and immune suppression in squamous lung cancer
Source: EMBO Rep. 2025 Nov 10;26(24):6346–79. doi: 10.1038/s44319-025-00631-1 (PMC12714794; doi:10.1038/s44319-025-00631-1)
Supplement: Supplementary file 8 — Source data Fig. 3 [file 44319_2025_631_MOESM8_ESM.zip › Figure 3/3D-E/GSEA_Broad Institute_Mh_T11b high vs low LUSC/gsea_report_for_na_pos_1723673606674.html]

Report for na\_pos 1723673606674 [GSEA]

| GS  follow link to MSigDB | GS DETAILS | SIZE | ES | NES | NOM p-val | FDR q-val | FWER p-val | RANK AT MAX | LEADING EDGE || 1 | HALLMARK\_EPITHELIAL\_MESENCHYMAL\_TRANSITION | Details ... | 64 | 0.66 | 3.84 | 0.000 | 0.000 | 0.000 | 817 | tags=78%, list=20%, signal=96% |
| 2 | HALLMARK\_COMPLEMENT | Details ... | 63 | 0.60 | 3.40 | 0.000 | 0.000 | 0.000 | 548 | tags=52%, list=13%, signal=60% |
| 3 | HALLMARK\_KRAS\_SIGNALING\_UP | Details ... | 75 | 0.48 | 2.99 | 0.000 | 0.000 | 0.000 | 714 | tags=49%, list=17%, signal=59% |
| 4 | HALLMARK\_APICAL\_JUNCTION | Details ... | 48 | 0.51 | 2.75 | 0.000 | 0.000 | 0.000 | 974 | tags=67%, list=24%, signal=86% |
| 5 | HALLMARK\_COAGULATION | Details ... | 38 | 0.55 | 2.74 | 0.000 | 0.000 | 0.000 | 792 | tags=63%, list=19%, signal=78% |
| 6 | HALLMARK\_IL6\_JAK\_STAT3\_SIGNALING | Details ... | 23 | 0.64 | 2.69 | 0.000 | 0.000 | 0.000 | 562 | tags=65%, list=14%, signal=75% |
| 7 | HALLMARK\_INFLAMMATORY\_RESPONSE | Details ... | 58 | 0.49 | 2.69 | 0.000 | 0.000 | 0.000 | 548 | tags=45%, list=13%, signal=51% |
| 8 | HALLMARK\_TNFA\_SIGNALING\_VIA\_NFKB | Details ... | 78 | 0.42 | 2.53 | 0.000 | 0.000 | 0.000 | 954 | tags=59%, list=23%, signal=75% |
| 9 | HALLMARK\_ALLOGRAFT\_REJECTION | Details ... | 40 | 0.48 | 2.37 | 0.000 | 0.000 | 0.001 | 890 | tags=68%, list=22%, signal=85% |
| 10 | HALLMARK\_HYPOXIA | Details ... | 65 | 0.38 | 2.19 | 0.000 | 0.001 | 0.005 | 776 | tags=49%, list=19%, signal=60% |
| 11 | HALLMARK\_IL2\_STAT5\_SIGNALING | Details ... | 64 | 0.35 | 2.08 | 0.000 | 0.003 | 0.019 | 738 | tags=41%, list=18%, signal=49% |
| 12 | HALLMARK\_P53\_PATHWAY | Details ... | 71 | 0.34 | 2.03 | 0.005 | 0.005 | 0.033 | 834 | tags=44%, list=20%, signal=54% |
| 13 | HALLMARK\_APOPTOSIS | Details ... | 56 | 0.34 | 1.93 | 0.000 | 0.009 | 0.059 | 950 | tags=52%, list=23%, signal=67% |
| 14 | HALLMARK\_MTORC1\_SIGNALING | Details ... | 55 | 0.34 | 1.90 | 0.000 | 0.010 | 0.071 | 949 | tags=51%, list=23%, signal=65% |
| 15 | HALLMARK\_CHOLESTEROL\_HOMEOSTASIS | Details ... | 29 | 0.37 | 1.73 | 0.024 | 0.027 | 0.200 | 885 | tags=55%, list=22%, signal=70% |
| 16 | HALLMARK\_INTERFERON\_GAMMA\_RESPONSE | Details ... | 46 | 0.28 | 1.44 | 0.052 | 0.144 | 0.746 | 954 | tags=52%, list=23%, signal=67% |
| 17 | HALLMARK\_UV\_RESPONSE\_UP | Details ... | 35 | 0.28 | 1.37 | 0.117 | 0.185 | 0.845 | 373 | tags=20%, list=9%, signal=22% |
| 18 | HALLMARK\_TGF\_BETA\_SIGNALING | Details ... | 17 | 0.32 | 1.29 | 0.161 | 0.254 | 0.932 | 1490 | tags=71%, list=36%, signal=111% |
| 19 | HALLMARK\_PI3K\_AKT\_MTOR\_SIGNALING | Details ... | 22 | 0.29 | 1.24 | 0.213 | 0.305 | 0.956 | 942 | tags=50%, list=23%, signal=65% |
| 20 | HALLMARK\_MYOGENESIS | Details ... | 58 | 0.22 | 1.23 | 0.217 | 0.304 | 0.964 | 711 | tags=34%, list=17%, signal=41% |
| 21 | HALLMARK\_GLYCOLYSIS |  | 72 | 0.20 | 1.19 | 0.216 | 0.332 | 0.980 | 724 | tags=32%, list=18%, signal=38% |
| 22 | HALLMARK\_INTERFERON\_ALPHA\_RESPONSE |  | 25 | 0.26 | 1.19 | 0.230 | 0.318 | 0.980 | 1012 | tags=52%, list=25%, signal=69% |
| 23 | HALLMARK\_XENOBIOTIC\_METABOLISM |  | 69 | 0.18 | 1.09 | 0.330 | 0.445 | 0.995 | 89 | tags=9%, list=2%, signal=9% |
| 24 | HALLMARK\_ADIPOGENESIS |  | 71 | 0.18 | 1.06 | 0.352 | 0.469 | 0.999 | 346 | tags=15%, list=8%, signal=17% |
| 25 | HALLMARK\_REACTIVE\_OXYGEN\_SPECIES\_PATHWAY |  | 16 | 0.28 | 1.05 | 0.401 | 0.474 | 1.000 | 2961 | tags=100%, list=72%, signal=361% |
| 26 | HALLMARK\_MYC\_TARGETS\_V1 |  | 31 | 0.21 | 1.00 | 0.470 | 0.534 | 1.000 | 2537 | tags=90%, list=62%, signal=236% |
| 27 | HALLMARK\_UV\_RESPONSE\_DN |  | 52 | 0.17 | 0.94 | 0.557 | 0.604 | 1.000 | 490 | tags=21%, list=12%, signal=24% |
| 28 | HALLMARK\_FATTY\_ACID\_METABOLISM |  | 55 | 0.16 | 0.85 | 0.696 | 0.726 | 1.000 | 283 | tags=11%, list=7%, signal=12% |
| 29 | HALLMARK\_DNA\_REPAIR |  | 36 | 0.15 | 0.74 | 0.793 | 0.850 | 1.000 | 3086 | tags=94%, list=75%, signal=381% |
| 30 | HALLMARK\_HEME\_METABOLISM |  | 47 | 0.12 | 0.64 | 0.905 | 0.911 | 1.000 | 979 | tags=36%, list=24%, signal=47% |
Table: Gene sets enriched in phenotype **na**[plain text format]****

  
