# Supplementary material for: TMPRSS11B promotes an acidified microenvironment and immune suppression in squamous lung cancer
Source: EMBO Rep. 2025 Nov 10;26(24):6346–79. doi: 10.1038/s44319-025-00631-1 (PMC12714794; doi:10.1038/s44319-025-00631-1)
Supplement: Supplementary file 8 — Source data Fig. 3 [file 44319_2025_631_MOESM8_ESM.zip › Figure 3/3D-E/GSEA_Broad Institute_Mh_T11b high vs low LUSC/HALLMARK_ALLOGRAFT_REJECTION.html]

Details for gene set HALLMARK\_ALLOGRAFT\_REJECTION[GSEA]

|  || Dataset | T11b high vs low squamous\_GSEA\_Ranked |
| Phenotype | NoPhenotypeAvailable |
| Upregulated in class | na\_pos |
| GeneSet | HALLMARK\_ALLOGRAFT\_REJECTION |
| Enrichment Score (ES) | 0.47837824 |
| Normalized Enrichment Score (NES) | 2.3744507 |
| Nominal p-value | 0.0 |
| FDR q-value | 2.5641028E-4 |
| FWER p-Value | 0.001 |
Table: GSEA Results Summary

  

Fig 1: Enrichment plot: HALLMARK\_ALLOGRAFT\_REJECTION      
 Profile of the Running ES Score & Positions of GeneSet Members on the Rank Ordered List

  

| SYMBOL | RANK IN GENE LIST | RANK METRIC SCORE | RUNNING ES | CORE ENRICHMENT || 1 | Fcgr2b | 62 | 2.610 | 0.0395 | Yes |
| 2 | Ctss | 63 | 2.582 | 0.0938 | Yes |
| 3 | Itgb2 | 71 | 2.481 | 0.1442 | Yes |
| 4 | Igsf6 | 118 | 1.998 | 0.1748 | Yes |
| 5 | Il1b | 129 | 1.912 | 0.2125 | Yes |
| 6 | Spi1 | 158 | 1.765 | 0.2427 | Yes |
| 7 | Cfp | 171 | 1.717 | 0.2758 | Yes |
| 8 | Mmp9 | 249 | 1.449 | 0.2872 | Yes |
| 9 | Ptprc | 258 | 1.430 | 0.3153 | Yes |
| 10 | Srgn | 270 | 1.392 | 0.3418 | Yes |
| 11 | Capg | 343 | 1.160 | 0.3484 | Yes |
| 12 | Ets1 | 396 | 1.061 | 0.3578 | Yes |
| 13 | Gpr65 | 401 | 1.047 | 0.3789 | Yes |
| 14 | Flna | 471 | 0.946 | 0.3817 | Yes |
| 15 | Tnf | 495 | 0.907 | 0.3951 | Yes |
| 16 | Hcls1 | 503 | 0.897 | 0.4122 | Yes |
| 17 | Cdkn2a | 509 | 0.889 | 0.4296 | Yes |
| 18 | Stab1 | 523 | 0.873 | 0.4448 | Yes |
| 19 | Irf7 | 548 | 0.847 | 0.4566 | Yes |
| 20 | Ifnar2 | 567 | 0.830 | 0.4696 | Yes |
| 21 | Hif1a | 684 | 0.683 | 0.4553 | Yes |
| 22 | Was | 770 | 0.610 | 0.4471 | Yes |
| 23 | Tgfb2 | 800 | 0.593 | 0.4524 | Yes |
| 24 | Lcp2 | 840 | 0.570 | 0.4548 | Yes |
| 25 | Cd74 | 849 | 0.567 | 0.4647 | Yes |
| 26 | B2m | 860 | 0.563 | 0.4741 | Yes |
| 27 | Degs1 | 890 | 0.544 | 0.4784 | Yes |
| 28 | Il15 | 1933 | -0.681 | 0.2354 | No |
| 29 | Ccnd3 | 2040 | -0.700 | 0.2239 | No |
| 30 | Fas | 2311 | -0.764 | 0.1733 | No |
| 31 | F2r | 2690 | -0.872 | 0.0983 | No |
| 32 | Socs5 | 2778 | -0.900 | 0.0957 | No |
| 33 | Ly75 | 2931 | -0.950 | 0.0782 | No |
| 34 | Tap1 | 3068 | -1.004 | 0.0657 | No |
| 35 | Il18 | 3078 | -1.010 | 0.0847 | No |
| 36 | Traf2 | 3325 | -1.127 | 0.0476 | No |
| 37 | Tlr2 | 3387 | -1.156 | 0.0569 | No |
| 38 | Eif3j1 | 3797 | -1.506 | -0.0125 | No |
| 39 | Gcnt1 | 3892 | -1.706 | 0.0001 | No |
| 40 | Ereg | 4031 | -2.297 | 0.0143 | No |
Table: GSEA details [plain text format]

  

Fig 2: HALLMARK\_ALLOGRAFT\_REJECTION: Random ES distribution      
 Gene set null distribution of ES for **HALLMARK\_ALLOGRAFT\_REJECTION**

  
