# Supplementary material for: TMPRSS11B promotes an acidified microenvironment and immune suppression in squamous lung cancer
Source: EMBO Rep. 2025 Nov 10;26(24):6346–79. doi: 10.1038/s44319-025-00631-1 (PMC12714794; doi:10.1038/s44319-025-00631-1)
Supplement: Supplementary file 8 — Source data Fig. 3 [file 44319_2025_631_MOESM8_ESM.zip › Figure 3/3D-E/GSEA_Broad Institute_Mh_T11b high vs low LUSC/HALLMARK_ANDROGEN_RESPONSE.html]

Details for gene set HALLMARK\_ANDROGEN\_RESPONSE[GSEA]

|  || Dataset | T11b high vs low squamous\_GSEA\_Ranked |
| Phenotype | NoPhenotypeAvailable |
| Upregulated in class | na\_neg |
| GeneSet | HALLMARK\_ANDROGEN\_RESPONSE |
| Enrichment Score (ES) | -0.20330438 |
| Normalized Enrichment Score (NES) | -0.950636 |
| Nominal p-value | 0.5477707 |
| FDR q-value | 0.9952117 |
| FWER p-Value | 1.0 |
Table: GSEA Results Summary

  

Fig 1: Enrichment plot: HALLMARK\_ANDROGEN\_RESPONSE      
 Profile of the Running ES Score & Positions of GeneSet Members on the Rank Ordered List

  

| SYMBOL | RANK IN GENE LIST | RANK METRIC SCORE | RUNNING ES | CORE ENRICHMENT || 1 | Plpp1 | 153 | 1.798 | 0.0144 | No |
| 2 | Aldh1a3 | 203 | 1.589 | 0.0485 | No |
| 3 | Mertk | 250 | 1.448 | 0.0792 | No |
| 4 | Ndrg1 | 276 | 1.380 | 0.1130 | No |
| 5 | Ptk2b | 559 | 0.835 | 0.0677 | No |
| 6 | Stk39 | 637 | 0.721 | 0.0697 | No |
| 7 | Sat1 | 817 | 0.584 | 0.0425 | No |
| 8 | Maf | 826 | 0.576 | 0.0572 | No |
| 9 | B2m | 860 | 0.563 | 0.0655 | No |
| 10 | Hmgcr | 885 | 0.549 | 0.0755 | No |
| 11 | Insig1 | 945 | 0.509 | 0.0757 | No |
| 12 | Abcc4 | 1069 | -0.517 | 0.0604 | No |
| 13 | Actn1 | 1186 | -0.537 | 0.0473 | No |
| 14 | Krt8 | 1410 | -0.578 | 0.0091 | No |
| 15 | Pgm3 | 1586 | -0.609 | -0.0164 | No |
| 16 | Tsc22d1 | 1718 | -0.633 | -0.0303 | No |
| 17 | Pias1 | 1789 | -0.646 | -0.0288 | No |
| 18 | Ccnd3 | 2040 | -0.700 | -0.0702 | No |
| 19 | Selenop | 2054 | -0.707 | -0.0528 | No |
| 20 | Slc38a2 | 2121 | -0.719 | -0.0482 | No |
| 21 | Fads1 | 2281 | -0.757 | -0.0655 | No |
| 22 | Sgk1 | 2539 | -0.829 | -0.1048 | No |
| 23 | Elovl5 | 2766 | -0.895 | -0.1346 | No |
| 24 | Xrcc5 | 3034 | -0.988 | -0.1717 | No |
| 25 | Krt19 | 3163 | -1.044 | -0.1730 | Yes |
| 26 | Akap12 | 3203 | -1.066 | -0.1517 | Yes |
| 27 | Lifr | 3297 | -1.111 | -0.1424 | Yes |
| 28 | Ccnd1 | 3303 | -1.112 | -0.1114 | Yes |
| 29 | Spdef | 3388 | -1.157 | -0.0985 | Yes |
| 30 | Tmprss2 | 3501 | -1.211 | -0.0910 | Yes |
| 31 | Gpd1l | 3764 | -1.454 | -0.1134 | Yes |
| 32 | Iqgap2 | 3775 | -1.464 | -0.0734 | Yes |
| 33 | Tnfaip8 | 3837 | -1.581 | -0.0425 | Yes |
| 34 | Slc26a2 | 3936 | -1.780 | -0.0150 | Yes |
| 35 | Homer2 | 3946 | -1.809 | 0.0353 | Yes |
Table: GSEA details [plain text format]

  

Fig 2: HALLMARK\_ANDROGEN\_RESPONSE: Random ES distribution      
 Gene set null distribution of ES for **HALLMARK\_ANDROGEN\_RESPONSE**

  
