# Supplementary material for: TMPRSS11B promotes an acidified microenvironment and immune suppression in squamous lung cancer
Source: EMBO Rep. 2025 Nov 10;26(24):6346–79. doi: 10.1038/s44319-025-00631-1 (PMC12714794; doi:10.1038/s44319-025-00631-1)
Supplement: Supplementary file 8 — Source data Fig. 3 [file 44319_2025_631_MOESM8_ESM.zip › Figure 3/3D-E/GSEA_Broad Institute_Mh_T11b high vs low LUSC/HALLMARK_APICAL_JUNCTION.html]

Details for gene set HALLMARK\_APICAL\_JUNCTION[GSEA]

|  || Dataset | T11b high vs low squamous\_GSEA\_Ranked |
| Phenotype | NoPhenotypeAvailable |
| Upregulated in class | na\_pos |
| GeneSet | HALLMARK\_APICAL\_JUNCTION |
| Enrichment Score (ES) | 0.5115546 |
| Normalized Enrichment Score (NES) | 2.7534955 |
| Nominal p-value | 0.0 |
| FDR q-value | 0.0 |
| FWER p-Value | 0.0 |
Table: GSEA Results Summary

  

Fig 1: Enrichment plot: HALLMARK\_APICAL\_JUNCTION      
 Profile of the Running ES Score & Positions of GeneSet Members on the Rank Ordered List

  

| SYMBOL | RANK IN GENE LIST | RANK METRIC SCORE | RUNNING ES | CORE ENRICHMENT || 1 | Cd274 | 96 | 2.227 | 0.0243 | Yes |
| 2 | Thbs3 | 148 | 1.832 | 0.0513 | Yes |
| 3 | Pik3cb | 149 | 1.832 | 0.0908 | Yes |
| 4 | Tgfbi | 151 | 1.825 | 0.1300 | Yes |
| 5 | Sirpa | 154 | 1.794 | 0.1682 | Yes |
| 6 | Sdc3 | 196 | 1.610 | 0.1928 | Yes |
| 7 | Msn | 219 | 1.522 | 0.2202 | Yes |
| 8 | Mmp9 | 249 | 1.449 | 0.2443 | Yes |
| 9 | Cldn5 | 252 | 1.447 | 0.2751 | Yes |
| 10 | Ptprc | 258 | 1.430 | 0.3047 | Yes |
| 11 | Layn | 259 | 1.428 | 0.3356 | Yes |
| 12 | Tnfrsf11b | 275 | 1.380 | 0.3616 | Yes |
| 13 | Syk | 278 | 1.375 | 0.3908 | Yes |
| 14 | Cdsn | 363 | 1.121 | 0.3943 | Yes |
| 15 | Cdh3 | 417 | 1.023 | 0.4032 | Yes |
| 16 | Nectin1 | 428 | 1.008 | 0.4225 | Yes |
| 17 | Lama3 | 542 | 0.853 | 0.4130 | Yes |
| 18 | Fbn1 | 569 | 0.830 | 0.4245 | Yes |
| 19 | Vwf | 627 | 0.734 | 0.4262 | Yes |
| 20 | Actb | 633 | 0.726 | 0.4406 | Yes |
| 21 | Epb41l2 | 635 | 0.724 | 0.4560 | Yes |
| 22 | Map3k20 | 636 | 0.723 | 0.4716 | Yes |
| 23 | Rsu1 | 731 | 0.645 | 0.4623 | Yes |
| 24 | Jup | 742 | 0.637 | 0.4736 | Yes |
| 25 | Nectin4 | 782 | 0.601 | 0.4769 | Yes |
| 26 | Cldn4 | 811 | 0.589 | 0.4827 | Yes |
| 27 | Col17a1 | 835 | 0.573 | 0.4894 | Yes |
| 28 | Ldlrap1 | 903 | 0.530 | 0.4843 | Yes |
| 29 | Mpzl2 | 939 | 0.512 | 0.4866 | Yes |
| 30 | Bmp1 | 940 | 0.510 | 0.4977 | Yes |
| 31 | Insig1 | 945 | 0.509 | 0.5077 | Yes |
| 32 | Sympk | 974 | -0.502 | 0.5116 | Yes |
| 33 | Actn1 | 1186 | -0.537 | 0.4709 | No |
| 34 | Cdh11 | 1305 | -0.556 | 0.4537 | No |
| 35 | Tial1 | 1359 | -0.567 | 0.4529 | No |
| 36 | Inppl1 | 1372 | -0.569 | 0.4622 | No |
| 37 | Itga3 | 1535 | -0.600 | 0.4351 | No |
| 38 | Col16a1 | 1544 | -0.601 | 0.4461 | No |
| 39 | Vav2 | 1720 | -0.634 | 0.4165 | No |
| 40 | Tsc1 | 1813 | -0.651 | 0.4078 | No |
| 41 | Dhx16 | 1843 | -0.660 | 0.4148 | No |
| 42 | Nectin2 | 1952 | -0.686 | 0.4029 | No |
| 43 | Hadh | 2449 | -0.804 | 0.2975 | No |
| 44 | Mpzl1 | 2667 | -0.864 | 0.2625 | No |
| 45 | Cdh1 | 2989 | -0.975 | 0.2042 | No |
| 46 | Akt2 | 3033 | -0.987 | 0.2148 | No |
| 47 | Parva | 3131 | -1.032 | 0.2131 | No |
| 48 | Sorbs3 | 3256 | -1.096 | 0.2061 | No |
Table: GSEA details [plain text format]

  

Fig 2: HALLMARK\_APICAL\_JUNCTION: Random ES distribution      
 Gene set null distribution of ES for **HALLMARK\_APICAL\_JUNCTION**

  
