# Supplementary material for: TMPRSS11B promotes an acidified microenvironment and immune suppression in squamous lung cancer
Source: EMBO Rep. 2025 Nov 10;26(24):6346–79. doi: 10.1038/s44319-025-00631-1 (PMC12714794; doi:10.1038/s44319-025-00631-1)
Supplement: Supplementary file 8 — Source data Fig. 3 [file 44319_2025_631_MOESM8_ESM.zip › Figure 3/3D-E/GSEA_Broad Institute_Mh_T11b high vs low LUSC/HALLMARK_APOPTOSIS.html]

Details for gene set HALLMARK\_APOPTOSIS[GSEA]

|  || Dataset | T11b high vs low squamous\_GSEA\_Ranked |
| Phenotype | NoPhenotypeAvailable |
| Upregulated in class | na\_pos |
| GeneSet | HALLMARK\_APOPTOSIS |
| Enrichment Score (ES) | 0.3431489 |
| Normalized Enrichment Score (NES) | 1.9329103 |
| Nominal p-value | 0.0 |
| FDR q-value | 0.008607455 |
| FWER p-Value | 0.059 |
Table: GSEA Results Summary

  

Fig 1: Enrichment plot: HALLMARK\_APOPTOSIS      
 Profile of the Running ES Score & Positions of GeneSet Members on the Rank Ordered List

  

| SYMBOL | RANK IN GENE LIST | RANK METRIC SCORE | RUNNING ES | CORE ENRICHMENT || 1 | Plat | 29 | 3.372 | 0.0500 | Yes |
| 2 | Il1a | 44 | 2.812 | 0.0942 | Yes |
| 3 | Hmox1 | 55 | 2.684 | 0.1372 | Yes |
| 4 | Il1b | 129 | 1.912 | 0.1515 | Yes |
| 5 | Emp1 | 133 | 1.901 | 0.1830 | Yes |
| 6 | Rnasel | 179 | 1.692 | 0.2006 | Yes |
| 7 | Cdkn1a | 192 | 1.625 | 0.2251 | Yes |
| 8 | Anxa1 | 290 | 1.344 | 0.2239 | Yes |
| 9 | Gna15 | 326 | 1.197 | 0.2355 | Yes |
| 10 | Irf1 | 372 | 1.111 | 0.2432 | Yes |
| 11 | Atf3 | 373 | 1.107 | 0.2619 | Yes |
| 12 | Lgals3 | 377 | 1.096 | 0.2798 | Yes |
| 13 | Timp2 | 462 | 0.955 | 0.2752 | Yes |
| 14 | Gadd45a | 473 | 0.944 | 0.2887 | Yes |
| 15 | Tnf | 495 | 0.907 | 0.2989 | Yes |
| 16 | Hspb1 | 500 | 0.898 | 0.3131 | Yes |
| 17 | Gadd45b | 522 | 0.873 | 0.3227 | Yes |
| 18 | Cd44 | 562 | 0.834 | 0.3272 | Yes |
| 19 | Birc3 | 607 | 0.758 | 0.3291 | Yes |
| 20 | Cflar | 686 | 0.680 | 0.3213 | Yes |
| 21 | Timp3 | 754 | 0.625 | 0.3153 | Yes |
| 22 | Pdgfrb | 764 | 0.616 | 0.3235 | Yes |
| 23 | Tgfb2 | 800 | 0.593 | 0.3249 | Yes |
| 24 | Sat1 | 817 | 0.584 | 0.3308 | Yes |
| 25 | Bcl2l1 | 878 | 0.552 | 0.3253 | Yes |
| 26 | Casp8 | 920 | 0.520 | 0.3239 | Yes |
| 27 | Pmaip1 | 935 | 0.514 | 0.3292 | Yes |
| 28 | Rela | 943 | 0.509 | 0.3361 | Yes |
| 29 | Mcl1 | 950 | 0.505 | 0.3431 | Yes |
| 30 | Madd | 1256 | -0.548 | 0.2768 | No |
| 31 | Erbb3 | 1362 | -0.568 | 0.2604 | No |
| 32 | Wee1 | 1388 | -0.572 | 0.2639 | No |
| 33 | Pak1 | 1534 | -0.600 | 0.2382 | No |
| 34 | Nedd9 | 1559 | -0.605 | 0.2425 | No |
| 35 | Mgmt | 1660 | -0.622 | 0.2282 | No |
| 36 | Erbb2 | 1855 | -0.662 | 0.1914 | No |
| 37 | Bmf | 2085 | -0.712 | 0.1467 | No |
| 38 | Etf1 | 2182 | -0.735 | 0.1353 | No |
| 39 | Fas | 2311 | -0.764 | 0.1166 | No |
| 40 | Tnfsf10 | 2592 | -0.845 | 0.0615 | No |
| 41 | F2r | 2690 | -0.872 | 0.0522 | No |
| 42 | Sod1 | 2691 | -0.874 | 0.0670 | No |
| 43 | Fdxr | 2703 | -0.876 | 0.0791 | No |
| 44 | Txnip | 2924 | -0.947 | 0.0407 | No |
| 45 | Tap1 | 3068 | -1.004 | 0.0222 | No |
| 46 | Il18 | 3078 | -1.010 | 0.0371 | No |
| 47 | Gstm2 | 3116 | -1.026 | 0.0454 | No |
| 48 | Dnajc3 | 3125 | -1.028 | 0.0608 | No |
| 49 | Psen1 | 3136 | -1.034 | 0.0759 | No |
| 50 | Casp4 | 3233 | -1.084 | 0.0704 | No |
| 51 | Ccnd1 | 3303 | -1.112 | 0.0722 | No |
| 52 | Rock1 | 3378 | -1.149 | 0.0733 | No |
| 53 | Clu | 3405 | -1.162 | 0.0866 | No |
| 54 | Pdcd4 | 3488 | -1.204 | 0.0867 | No |
| 55 | Isg20 | 3663 | -1.348 | 0.0664 | No |
| 56 | Ereg | 4031 | -2.297 | 0.0144 | No |
Table: GSEA details [plain text format]

  

Fig 2: HALLMARK\_APOPTOSIS: Random ES distribution      
 Gene set null distribution of ES for **HALLMARK\_APOPTOSIS**

  
