# Supplementary material for: TMPRSS11B promotes an acidified microenvironment and immune suppression in squamous lung cancer
Source: EMBO Rep. 2025 Nov 10;26(24):6346–79. doi: 10.1038/s44319-025-00631-1 (PMC12714794; doi:10.1038/s44319-025-00631-1)
Supplement: Supplementary file 8 — Source data Fig. 3 [file 44319_2025_631_MOESM8_ESM.zip › Figure 3/3D-E/GSEA_Broad Institute_Mh_T11b high vs low LUSC/HALLMARK_BILE_ACID_METABOLISM.html]

Details for gene set HALLMARK\_BILE\_ACID\_METABOLISM[GSEA]

|  || Dataset | T11b high vs low squamous\_GSEA\_Ranked |
| Phenotype | NoPhenotypeAvailable |
| Upregulated in class | na\_neg |
| GeneSet | HALLMARK\_BILE\_ACID\_METABOLISM |
| Enrichment Score (ES) | -0.34748626 |
| Normalized Enrichment Score (NES) | -1.5339988 |
| Nominal p-value | 0.047933884 |
| FDR q-value | 0.530312 |
| FWER p-Value | 0.65 |
Table: GSEA Results Summary

  

Fig 1: Enrichment plot: HALLMARK\_BILE\_ACID\_METABOLISM      
 Profile of the Running ES Score & Positions of GeneSet Members on the Rank Ordered List

  

| SYMBOL | RANK IN GENE LIST | RANK METRIC SCORE | RUNNING ES | CORE ENRICHMENT || 1 | Abca1 | 172 | 1.716 | 0.0171 | No |
| 2 | Sult2b1 | 497 | 0.901 | -0.0315 | No |
| 3 | Cat | 639 | 0.721 | -0.0412 | No |
| 4 | Rbp1 | 924 | 0.518 | -0.0932 | No |
| 5 | Hsd17b11 | 1184 | -0.536 | -0.1384 | No |
| 6 | Pfkm | 1805 | -0.649 | -0.2685 | No |
| 7 | Abca2 | 2033 | -0.698 | -0.3002 | No |
| 8 | Pex7 | 2226 | -0.745 | -0.3217 | Yes |
| 9 | Hsd17b4 | 2245 | -0.749 | -0.3002 | Yes |
| 10 | Fads1 | 2281 | -0.757 | -0.2826 | Yes |
| 11 | Nedd4 | 2529 | -0.827 | -0.3147 | Yes |
| 12 | Hsd3b7 | 2573 | -0.841 | -0.2962 | Yes |
| 13 | Acsl1 | 2609 | -0.850 | -0.2754 | Yes |
| 14 | Sod1 | 2691 | -0.874 | -0.2651 | Yes |
| 15 | Fdxr | 2703 | -0.876 | -0.2375 | Yes |
| 16 | Amacr | 2833 | -0.917 | -0.2375 | Yes |
| 17 | Pex13 | 2838 | -0.918 | -0.2066 | Yes |
| 18 | Pex11a | 3020 | -0.982 | -0.2172 | Yes |
| 19 | Cyp39a1 | 3040 | -0.990 | -0.1876 | Yes |
| 20 | Nudt12 | 3124 | -1.028 | -0.1724 | Yes |
| 21 | Aldh9a1 | 3166 | -1.046 | -0.1462 | Yes |
| 22 | Abcd3 | 3317 | -1.119 | -0.1444 | Yes |
| 23 | Slc22a18 | 3393 | -1.158 | -0.1227 | Yes |
| 24 | Phyh | 3626 | -1.310 | -0.1345 | Yes |
| 25 | Abca5 | 3717 | -1.399 | -0.1082 | Yes |
| 26 | Idh2 | 3729 | -1.416 | -0.0618 | Yes |
| 27 | Tfcp2l1 | 3955 | -1.839 | -0.0536 | Yes |
| 28 | Aldh1a1 | 4051 | -2.492 | 0.0094 | Yes |
Table: GSEA details [plain text format]

  

Fig 2: HALLMARK\_BILE\_ACID\_METABOLISM: Random ES distribution      
 Gene set null distribution of ES for **HALLMARK\_BILE\_ACID\_METABOLISM**

  
