# Supplementary material for: TMPRSS11B promotes an acidified microenvironment and immune suppression in squamous lung cancer
Source: EMBO Rep. 2025 Nov 10;26(24):6346–79. doi: 10.1038/s44319-025-00631-1 (PMC12714794; doi:10.1038/s44319-025-00631-1)
Supplement: Supplementary file 8 — Source data Fig. 3 [file 44319_2025_631_MOESM8_ESM.zip › Figure 3/3D-E/GSEA_Broad Institute_Mh_T11b high vs low LUSC/HALLMARK_CHOLESTEROL_HOMEOSTASIS.html]

Details for gene set HALLMARK\_CHOLESTEROL\_HOMEOSTASIS[GSEA]

|  || Dataset | T11b high vs low squamous\_GSEA\_Ranked |
| Phenotype | NoPhenotypeAvailable |
| Upregulated in class | na\_pos |
| GeneSet | HALLMARK\_CHOLESTEROL\_HOMEOSTASIS |
| Enrichment Score (ES) | 0.36510095 |
| Normalized Enrichment Score (NES) | 1.7337894 |
| Nominal p-value | 0.023622047 |
| FDR q-value | 0.026906302 |
| FWER p-Value | 0.2 |
Table: GSEA Results Summary

  

Fig 1: Enrichment plot: HALLMARK\_CHOLESTEROL\_HOMEOSTASIS      
 Profile of the Running ES Score & Positions of GeneSet Members on the Rank Ordered List

  

| SYMBOL | RANK IN GENE LIST | RANK METRIC SCORE | RUNNING ES | CORE ENRICHMENT || 1 | Lpl | 43 | 2.812 | 0.0798 | Yes |
| 2 | Lgmn | 141 | 1.877 | 0.1163 | Yes |
| 3 | Gusb | 199 | 1.597 | 0.1536 | Yes |
| 4 | Hsd17b7 | 283 | 1.363 | 0.1770 | Yes |
| 5 | Cxcl16 | 342 | 1.161 | 0.2001 | Yes |
| 6 | Cpeb2 | 366 | 1.117 | 0.2303 | Yes |
| 7 | Atf3 | 373 | 1.107 | 0.2645 | Yes |
| 8 | Lgals3 | 377 | 1.096 | 0.2990 | Yes |
| 9 | Fabp5 | 385 | 1.088 | 0.3322 | Yes |
| 10 | Plaur | 426 | 1.012 | 0.3549 | Yes |
| 11 | Mal2 | 550 | 0.845 | 0.3518 | Yes |
| 12 | Mvk | 669 | 0.695 | 0.3451 | Yes |
| 13 | Cyp51 | 825 | 0.577 | 0.3255 | Yes |
| 14 | Acat2 | 844 | 0.569 | 0.3393 | Yes |
| 15 | Nsdhl | 880 | 0.551 | 0.3484 | Yes |
| 16 | Hmgcr | 885 | 0.549 | 0.3651 | Yes |
| 17 | Gldc | 1118 | -0.526 | 0.3249 | No |
| 18 | Acss2 | 1760 | -0.642 | 0.1877 | No |
| 19 | Abca2 | 2033 | -0.698 | 0.1431 | No |
| 20 | Stx5a | 2066 | -0.712 | 0.1582 | No |
| 21 | Pcyt2 | 2237 | -0.748 | 0.1404 | No |
| 22 | Alcam | 2584 | -0.843 | 0.0823 | No |
| 23 | Jag1 | 2743 | -0.887 | 0.0719 | No |
| 24 | Ech1 | 2819 | -0.911 | 0.0827 | No |
| 25 | Clu | 3405 | -1.162 | -0.0240 | No |
| 26 | Atxn2 | 3471 | -1.197 | -0.0015 | No |
| 27 | Pparg | 3727 | -1.412 | -0.0188 | No |
| 28 | Chka | 3801 | -1.515 | 0.0119 | No |
| 29 | Gstm7 | 3949 | -1.827 | 0.0345 | No |
Table: GSEA details [plain text format]

  

Fig 2: HALLMARK\_CHOLESTEROL\_HOMEOSTASIS: Random ES distribution      
 Gene set null distribution of ES for **HALLMARK\_CHOLESTEROL\_HOMEOSTASIS**

  
