# Supplementary material for: TMPRSS11B promotes an acidified microenvironment and immune suppression in squamous lung cancer
Source: EMBO Rep. 2025 Nov 10;26(24):6346–79. doi: 10.1038/s44319-025-00631-1 (PMC12714794; doi:10.1038/s44319-025-00631-1)
Supplement: Supplementary file 8 — Source data Fig. 3 [file 44319_2025_631_MOESM8_ESM.zip › Figure 3/3D-E/GSEA_Broad Institute_Mh_T11b high vs low LUSC/HALLMARK_COAGULATION.html]

Details for gene set HALLMARK\_COAGULATION[GSEA]

|  || Dataset | T11b high vs low squamous\_GSEA\_Ranked |
| Phenotype | NoPhenotypeAvailable |
| Upregulated in class | na\_pos |
| GeneSet | HALLMARK\_COAGULATION |
| Enrichment Score (ES) | 0.5503548 |
| Normalized Enrichment Score (NES) | 2.738079 |
| Nominal p-value | 0.0 |
| FDR q-value | 0.0 |
| FWER p-Value | 0.0 |
Table: GSEA Results Summary

  

Fig 1: Enrichment plot: HALLMARK\_COAGULATION      
 Profile of the Running ES Score & Positions of GeneSet Members on the Rank Ordered List

  

| SYMBOL | RANK IN GENE LIST | RANK METRIC SCORE | RUNNING ES | CORE ENRICHMENT || 1 | Ctsl | 15 | 3.988 | 0.0740 | Yes |
| 2 | Ctsk | 23 | 3.576 | 0.1420 | Yes |
| 3 | Plat | 29 | 3.372 | 0.2065 | Yes |
| 4 | Serpine1 | 89 | 2.295 | 0.2367 | Yes |
| 5 | Plek | 95 | 2.237 | 0.2791 | Yes |
| 6 | Htra1 | 106 | 2.122 | 0.3180 | Yes |
| 7 | C1qa | 119 | 1.990 | 0.3538 | Yes |
| 8 | Plau | 136 | 1.895 | 0.3868 | Yes |
| 9 | Lgmn | 141 | 1.877 | 0.4224 | Yes |
| 10 | Apoc1 | 164 | 1.742 | 0.4510 | Yes |
| 11 | Ctsb | 177 | 1.695 | 0.4810 | Yes |
| 12 | Mmp9 | 249 | 1.449 | 0.4918 | Yes |
| 13 | Sh2b2 | 261 | 1.425 | 0.5168 | Yes |
| 14 | Anxa1 | 290 | 1.344 | 0.5361 | Yes |
| 15 | Serpinb2 | 527 | 0.873 | 0.4949 | Yes |
| 16 | Fbn1 | 569 | 0.830 | 0.5010 | Yes |
| 17 | Trf | 601 | 0.771 | 0.5084 | Yes |
| 18 | Vwf | 627 | 0.734 | 0.5165 | Yes |
| 19 | Capn2 | 726 | 0.646 | 0.5049 | Yes |
| 20 | Serping1 | 730 | 0.645 | 0.5167 | Yes |
| 21 | Csrp1 | 743 | 0.636 | 0.5262 | Yes |
| 22 | Timp3 | 754 | 0.625 | 0.5359 | Yes |
| 23 | Maff | 776 | 0.605 | 0.5425 | Yes |
| 24 | Sparc | 792 | 0.595 | 0.5504 | Yes |
| 25 | Bmp1 | 940 | 0.510 | 0.5240 | No |
| 26 | Dusp14 | 948 | 0.506 | 0.5322 | No |
| 27 | Gnb2 | 1007 | -0.506 | 0.5277 | No |
| 28 | Hnf4a | 1475 | -0.588 | 0.4239 | No |
| 29 | Ctse | 1793 | -0.647 | 0.3583 | No |
| 30 | Sirt2 | 1889 | -0.671 | 0.3479 | No |
| 31 | Mmp15 | 2387 | -0.786 | 0.2406 | No |
| 32 | Lamp2 | 2707 | -0.877 | 0.1790 | No |
| 33 | Cfi | 2950 | -0.956 | 0.1379 | No |
| 34 | Mst1 | 3084 | -1.012 | 0.1248 | No |
| 35 | Clu | 3405 | -1.162 | 0.0685 | No |
| 36 | Prss23 | 3509 | -1.216 | 0.0668 | No |
| 37 | Hpn | 3768 | -1.456 | 0.0315 | No |
| 38 | Hmgcs2 | 4048 | -2.436 | 0.0101 | No |
Table: GSEA details [plain text format]

  

Fig 2: HALLMARK\_COAGULATION: Random ES distribution      
 Gene set null distribution of ES for **HALLMARK\_COAGULATION**

  
