# Supplementary material for: TMPRSS11B promotes an acidified microenvironment and immune suppression in squamous lung cancer
Source: EMBO Rep. 2025 Nov 10;26(24):6346–79. doi: 10.1038/s44319-025-00631-1 (PMC12714794; doi:10.1038/s44319-025-00631-1)
Supplement: Supplementary file 8 — Source data Fig. 3 [file 44319_2025_631_MOESM8_ESM.zip › Figure 3/3D-E/GSEA_Broad Institute_Mh_T11b high vs low LUSC/HALLMARK_COMPLEMENT.html]

Details for gene set HALLMARK\_COMPLEMENT[GSEA]

|  || Dataset | T11b high vs low squamous\_GSEA\_Ranked |
| Phenotype | NoPhenotypeAvailable |
| Upregulated in class | na\_pos |
| GeneSet | HALLMARK\_COMPLEMENT |
| Enrichment Score (ES) | 0.5986769 |
| Normalized Enrichment Score (NES) | 3.399341 |
| Nominal p-value | 0.0 |
| FDR q-value | 0.0 |
| FWER p-Value | 0.0 |
Table: GSEA Results Summary

  

Fig 1: Enrichment plot: HALLMARK\_COMPLEMENT      
 Profile of the Running ES Score & Positions of GeneSet Members on the Rank Ordered List

  

| SYMBOL | RANK IN GENE LIST | RANK METRIC SCORE | RUNNING ES | CORE ENRICHMENT || 1 | Ctsl | 15 | 3.988 | 0.0450 | Yes |
| 2 | Mmp12 | 25 | 3.512 | 0.0857 | Yes |
| 3 | Plat | 29 | 3.372 | 0.1262 | Yes |
| 4 | Gngt2 | 51 | 2.724 | 0.1543 | Yes |
| 5 | Itgam | 61 | 2.631 | 0.1842 | Yes |
| 6 | Ctss | 63 | 2.582 | 0.2155 | Yes |
| 7 | Fcer1g | 76 | 2.415 | 0.2421 | Yes |
| 8 | S100a9 | 82 | 2.366 | 0.2698 | Yes |
| 9 | Cd36 | 85 | 2.334 | 0.2978 | Yes |
| 10 | Serpine1 | 89 | 2.295 | 0.3251 | Yes |
| 11 | Pla2g7 | 94 | 2.250 | 0.3516 | Yes |
| 12 | Plek | 95 | 2.237 | 0.3790 | Yes |
| 13 | C1qa | 119 | 1.990 | 0.3976 | Yes |
| 14 | Lgmn | 141 | 1.877 | 0.4153 | Yes |
| 15 | Ctsd | 150 | 1.830 | 0.4357 | Yes |
| 16 | Apoc1 | 164 | 1.742 | 0.4538 | Yes |
| 17 | Ctsb | 177 | 1.695 | 0.4715 | Yes |
| 18 | C1qc | 234 | 1.480 | 0.4757 | Yes |
| 19 | Pim1 | 262 | 1.425 | 0.4864 | Yes |
| 20 | Lipa | 296 | 1.318 | 0.4943 | Yes |
| 21 | Ehd1 | 313 | 1.237 | 0.5055 | Yes |
| 22 | Notch4 | 319 | 1.215 | 0.5191 | Yes |
| 23 | F7 | 330 | 1.190 | 0.5312 | Yes |
| 24 | Irf1 | 372 | 1.111 | 0.5346 | Yes |
| 25 | Lgals3 | 377 | 1.096 | 0.5470 | Yes |
| 26 | Col4a2 | 406 | 1.036 | 0.5527 | Yes |
| 27 | Plaur | 426 | 1.012 | 0.5603 | Yes |
| 28 | Cpm | 450 | 0.977 | 0.5666 | Yes |
| 29 | Timp2 | 462 | 0.955 | 0.5755 | Yes |
| 30 | Msrb1 | 472 | 0.945 | 0.5848 | Yes |
| 31 | Cebpb | 504 | 0.897 | 0.5881 | Yes |
| 32 | Serpinb2 | 527 | 0.873 | 0.5933 | Yes |
| 33 | Irf7 | 548 | 0.847 | 0.5987 | Yes |
| 34 | Spock2 | 605 | 0.767 | 0.5941 | No |
| 35 | Serping1 | 730 | 0.645 | 0.5712 | No |
| 36 | Csrp1 | 743 | 0.636 | 0.5760 | No |
| 37 | Was | 770 | 0.610 | 0.5770 | No |
| 38 | Maff | 776 | 0.605 | 0.5832 | No |
| 39 | Lcp2 | 840 | 0.570 | 0.5745 | No |
| 40 | Dusp5 | 861 | 0.563 | 0.5764 | No |
| 41 | Raf1 | 942 | 0.510 | 0.5628 | No |
| 42 | Xpnpep1 | 947 | 0.507 | 0.5680 | No |
| 43 | Calm3 | 961 | -0.500 | 0.5709 | No |
| 44 | Vcpip1 | 1005 | -0.506 | 0.5664 | No |
| 45 | Gnb2 | 1007 | -0.506 | 0.5723 | No |
| 46 | Brpf3 | 1042 | -0.512 | 0.5701 | No |
| 47 | Usp15 | 1403 | -0.575 | 0.4878 | No |
| 48 | Hnf4a | 1475 | -0.588 | 0.4773 | No |
| 49 | Kif2a | 1541 | -0.600 | 0.4685 | No |
| 50 | Prss36 | 1812 | -0.651 | 0.4094 | No |
| 51 | Ltf | 1834 | -0.657 | 0.4123 | No |
| 52 | Usp14 | 1838 | -0.658 | 0.4196 | No |
| 53 | Pla2g4a | 1905 | -0.674 | 0.4114 | No |
| 54 | Ppp4c | 2059 | -0.712 | 0.3821 | No |
| 55 | Usp16 | 2179 | -0.735 | 0.3616 | No |
| 56 | Cp | 2276 | -0.756 | 0.3470 | No |
| 57 | Mmp15 | 2387 | -0.786 | 0.3293 | No |
| 58 | Lamp2 | 2707 | -0.877 | 0.2608 | No |
| 59 | Psen1 | 3136 | -1.034 | 0.1671 | No |
| 60 | Casp4 | 3233 | -1.084 | 0.1565 | No |
| 61 | Clu | 3405 | -1.162 | 0.1283 | No |
| 62 | Gnb4 | 3871 | -1.647 | 0.0330 | No |
| 63 | Gca | 3900 | -1.712 | 0.0469 | No |
Table: GSEA details [plain text format]

  

Fig 2: HALLMARK\_COMPLEMENT: Random ES distribution      
 Gene set null distribution of ES for **HALLMARK\_COMPLEMENT**

  
