# Supplementary material for: TMPRSS11B promotes an acidified microenvironment and immune suppression in squamous lung cancer
Source: EMBO Rep. 2025 Nov 10;26(24):6346–79. doi: 10.1038/s44319-025-00631-1 (PMC12714794; doi:10.1038/s44319-025-00631-1)
Supplement: Supplementary file 8 — Source data Fig. 3 [file 44319_2025_631_MOESM8_ESM.zip › Figure 3/3D-E/GSEA_Broad Institute_Mh_T11b high vs low LUSC/HALLMARK_E2F_TARGETS.html]

Details for gene set HALLMARK\_E2F\_TARGETS[GSEA]

|  || Dataset | T11b high vs low squamous\_GSEA\_Ranked |
| Phenotype | NoPhenotypeAvailable |
| Upregulated in class | na\_neg |
| GeneSet | HALLMARK\_E2F\_TARGETS |
| Enrichment Score (ES) | -0.1862657 |
| Normalized Enrichment Score (NES) | -0.90084326 |
| Nominal p-value | 0.59150326 |
| FDR q-value | 0.78995436 |
| FWER p-Value | 1.0 |
Table: GSEA Results Summary

  

Fig 1: Enrichment plot: HALLMARK\_E2F\_TARGETS      
 Profile of the Running ES Score & Positions of GeneSet Members on the Rank Ordered List

  

| SYMBOL | RANK IN GENE LIST | RANK METRIC SCORE | RUNNING ES | CORE ENRICHMENT || 1 | Cdkn1a | 192 | 1.625 | 0.0022 | No |
| 2 | Melk | 369 | 1.115 | -0.0073 | No |
| 3 | Cdkn2a | 509 | 0.889 | -0.0145 | No |
| 4 | Tfrc | 668 | 0.696 | -0.0322 | No |
| 5 | Mcm3 | 697 | 0.670 | -0.0187 | No |
| 6 | Spc25 | 704 | 0.664 | 0.0001 | No |
| 7 | Cdk1 | 724 | 0.651 | 0.0152 | No |
| 8 | Cks2 | 812 | 0.588 | 0.0117 | No |
| 9 | Mad2l1 | 850 | 0.566 | 0.0198 | No |
| 10 | Atad2 | 958 | 0.501 | 0.0087 | No |
| 11 | Ung | 1258 | -0.549 | -0.0484 | No |
| 12 | Wee1 | 1388 | -0.572 | -0.0628 | No |
| 13 | Orc2 | 1395 | -0.575 | -0.0467 | No |
| 14 | Srsf1 | 1572 | -0.606 | -0.0717 | No |
| 15 | Ilf3 | 1590 | -0.609 | -0.0573 | No |
| 16 | Mlh1 | 1646 | -0.620 | -0.0520 | No |
| 17 | Ubr7 | 1868 | -0.666 | -0.0862 | No |
| 18 | Nap1l1 | 1883 | -0.670 | -0.0692 | No |
| 19 | Mcm4 | 1934 | -0.681 | -0.0608 | No |
| 20 | Pold3 | 2015 | -0.695 | -0.0593 | No |
| 21 | Pnn | 2123 | -0.720 | -0.0638 | No |
| 22 | Pop7 | 2300 | -0.762 | -0.0840 | No |
| 23 | Pds5b | 2511 | -0.822 | -0.1107 | No |
| 24 | Asf1a | 2749 | -0.889 | -0.1421 | No |
| 25 | Pold1 | 2929 | -0.950 | -0.1573 | Yes |
| 26 | Rpa3 | 2973 | -0.965 | -0.1385 | Yes |
| 27 | Rfc2 | 2975 | -0.965 | -0.1093 | Yes |
| 28 | Cdc25a | 2998 | -0.975 | -0.0850 | Yes |
| 29 | Cse1l | 3298 | -1.111 | -0.1249 | Yes |
| 30 | Nbn | 3414 | -1.168 | -0.1177 | Yes |
| 31 | Mthfd2 | 3458 | -1.191 | -0.0920 | Yes |
| 32 | Xpo1 | 3499 | -1.210 | -0.0650 | Yes |
| 33 | Psip1 | 3555 | -1.250 | -0.0405 | Yes |
| 34 | Rbbp7 | 3556 | -1.252 | -0.0023 | Yes |
| 35 | Smc6 | 3654 | -1.342 | 0.0147 | Yes |
| 36 | Paics | 3731 | -1.423 | 0.0393 | Yes |
| 37 | Cnot9 | 3847 | -1.601 | 0.0597 | Yes |
Table: GSEA details [plain text format]

  

Fig 2: HALLMARK\_E2F\_TARGETS: Random ES distribution      
 Gene set null distribution of ES for **HALLMARK\_E2F\_TARGETS**

  
