# Supplementary material for: TMPRSS11B promotes an acidified microenvironment and immune suppression in squamous lung cancer
Source: EMBO Rep. 2025 Nov 10;26(24):6346–79. doi: 10.1038/s44319-025-00631-1 (PMC12714794; doi:10.1038/s44319-025-00631-1)
Supplement: Supplementary file 8 — Source data Fig. 3 [file 44319_2025_631_MOESM8_ESM.zip › Figure 3/3D-E/GSEA_Broad Institute_Mh_T11b high vs low LUSC/HALLMARK_EPITHELIAL_MESENCHYMAL_TRANSITION.html]

Details for gene set HALLMARK\_EPITHELIAL\_MESENCHYMAL\_TRANSITION[GSEA]

|  || Dataset | T11b high vs low squamous\_GSEA\_Ranked |
| Phenotype | NoPhenotypeAvailable |
| Upregulated in class | na\_pos |
| GeneSet | HALLMARK\_EPITHELIAL\_MESENCHYMAL\_TRANSITION |
| Enrichment Score (ES) | 0.66067773 |
| Normalized Enrichment Score (NES) | 3.8383126 |
| Nominal p-value | 0.0 |
| FDR q-value | 0.0 |
| FWER p-Value | 0.0 |
Table: GSEA Results Summary

  

Fig 1: Enrichment plot: HALLMARK\_EPITHELIAL\_MESENCHYMAL\_TRANSITION      
 Profile of the Running ES Score & Positions of GeneSet Members on the Rank Ordered List

  

| SYMBOL | RANK IN GENE LIST | RANK METRIC SCORE | RUNNING ES | CORE ENRICHMENT || 1 | Spp1 | 13 | 4.054 | 0.0468 | Yes |
| 2 | Cxcl5 | 16 | 3.974 | 0.0953 | Yes |
| 3 | Ecm1 | 31 | 3.325 | 0.1329 | Yes |
| 4 | Tnc | 84 | 2.358 | 0.1490 | Yes |
| 5 | Serpine1 | 89 | 2.295 | 0.1764 | Yes |
| 6 | Lgals1 | 98 | 2.178 | 0.2012 | Yes |
| 7 | Htra1 | 106 | 2.122 | 0.2257 | Yes |
| 8 | Vim | 112 | 2.054 | 0.2498 | Yes |
| 9 | Emp3 | 115 | 2.020 | 0.2742 | Yes |
| 10 | Igfbp3 | 124 | 1.955 | 0.2964 | Yes |
| 11 | Tgfbi | 151 | 1.825 | 0.3124 | Yes |
| 12 | Gpc1 | 155 | 1.783 | 0.3337 | Yes |
| 13 | Aplp1 | 161 | 1.747 | 0.3540 | Yes |
| 14 | Gja1 | 186 | 1.653 | 0.3684 | Yes |
| 15 | Col5a2 | 232 | 1.484 | 0.3755 | Yes |
| 16 | Cxcl15 | 238 | 1.470 | 0.3924 | Yes |
| 17 | Tnfrsf11b | 275 | 1.380 | 0.4005 | Yes |
| 18 | Pdlim4 | 279 | 1.375 | 0.4167 | Yes |
| 19 | Pthlh | 299 | 1.307 | 0.4281 | Yes |
| 20 | Col4a1 | 301 | 1.304 | 0.4440 | Yes |
| 21 | Slc6a8 | 315 | 1.227 | 0.4559 | Yes |
| 22 | Itga5 | 320 | 1.212 | 0.4699 | Yes |
| 23 | Crlf1 | 331 | 1.187 | 0.4820 | Yes |
| 24 | Capg | 343 | 1.160 | 0.4936 | Yes |
| 25 | Col5a1 | 348 | 1.150 | 0.5068 | Yes |
| 26 | Lox | 352 | 1.139 | 0.5201 | Yes |
| 27 | Fbln2 | 361 | 1.124 | 0.5320 | Yes |
| 28 | Col3a1 | 383 | 1.092 | 0.5402 | Yes |
| 29 | Col4a2 | 406 | 1.036 | 0.5476 | Yes |
| 30 | Fbln5 | 408 | 1.035 | 0.5601 | Yes |
| 31 | Plaur | 426 | 1.012 | 0.5684 | Yes |
| 32 | Efemp2 | 437 | 0.996 | 0.5782 | Yes |
| 33 | Flna | 471 | 0.946 | 0.5816 | Yes |
| 34 | Gadd45a | 473 | 0.944 | 0.5930 | Yes |
| 35 | Col6a2 | 479 | 0.927 | 0.6032 | Yes |
| 36 | Col1a1 | 483 | 0.921 | 0.6138 | Yes |
| 37 | Fstl1 | 507 | 0.892 | 0.6191 | Yes |
| 38 | Gadd45b | 522 | 0.873 | 0.6264 | Yes |
| 39 | Lama3 | 542 | 0.853 | 0.6322 | Yes |
| 40 | Eln | 549 | 0.845 | 0.6412 | Yes |
| 41 | Cd44 | 562 | 0.834 | 0.6485 | Yes |
| 42 | Fbn1 | 569 | 0.830 | 0.6572 | Yes |
| 43 | Serpinh1 | 621 | 0.740 | 0.6537 | Yes |
| 44 | Plod3 | 679 | 0.687 | 0.6480 | Yes |
| 45 | Col1a2 | 729 | 0.645 | 0.6438 | Yes |
| 46 | Timp3 | 754 | 0.625 | 0.6455 | Yes |
| 47 | Acta2 | 759 | 0.621 | 0.6522 | Yes |
| 48 | Pdgfrb | 764 | 0.616 | 0.6588 | Yes |
| 49 | Sparc | 792 | 0.595 | 0.6594 | Yes |
| 50 | Sat1 | 817 | 0.584 | 0.6607 | Yes |
| 51 | Matn2 | 876 | 0.554 | 0.6531 | No |
| 52 | Wipf1 | 888 | 0.546 | 0.6571 | No |
| 53 | Bmp1 | 940 | 0.510 | 0.6507 | No |
| 54 | Dab2 | 952 | 0.504 | 0.6542 | No |
| 55 | Cdh11 | 1305 | -0.556 | 0.5736 | No |
| 56 | Col16a1 | 1544 | -0.601 | 0.5219 | No |
| 57 | Il15 | 1933 | -0.681 | 0.4340 | No |
| 58 | Fas | 2311 | -0.764 | 0.3498 | No |
| 59 | Pvr | 2314 | -0.766 | 0.3587 | No |
| 60 | Areg | 2629 | -0.856 | 0.2913 | No |
| 61 | Pfn2 | 3511 | -1.217 | 0.0875 | No |
| 62 | Fmod | 3707 | -1.390 | 0.0562 | No |
| 63 | Qsox1 | 3787 | -1.486 | 0.0549 | No |
| 64 | Pcolce | 3854 | -1.610 | 0.0584 | No |
Table: GSEA details [plain text format]

  

Fig 2: HALLMARK\_EPITHELIAL\_MESENCHYMAL\_TRANSITION: Random ES distribution      
 Gene set null distribution of ES for **HALLMARK\_EPITHELIAL\_MESENCHYMAL\_TRANSITION**

  
