# Supplementary material for: TMPRSS11B promotes an acidified microenvironment and immune suppression in squamous lung cancer
Source: EMBO Rep. 2025 Nov 10;26(24):6346–79. doi: 10.1038/s44319-025-00631-1 (PMC12714794; doi:10.1038/s44319-025-00631-1)
Supplement: Supplementary file 8 — Source data Fig. 3 [file 44319_2025_631_MOESM8_ESM.zip › Figure 3/3D-E/GSEA_Broad Institute_Mh_T11b high vs low LUSC/HALLMARK_ESTROGEN_RESPONSE_EARLY.html]

Details for gene set HALLMARK\_ESTROGEN\_RESPONSE\_EARLY[GSEA]

|  || Dataset | T11b high vs low squamous\_GSEA\_Ranked |
| Phenotype | NoPhenotypeAvailable |
| Upregulated in class | na\_neg |
| GeneSet | HALLMARK\_ESTROGEN\_RESPONSE\_EARLY |
| Enrichment Score (ES) | -0.20060904 |
| Normalized Enrichment Score (NES) | -1.1138291 |
| Nominal p-value | 0.30806452 |
| FDR q-value | 0.83336127 |
| FWER p-Value | 1.0 |
Table: GSEA Results Summary

  

Fig 1: Enrichment plot: HALLMARK\_ESTROGEN\_RESPONSE\_EARLY      
 Profile of the Running ES Score & Positions of GeneSet Members on the Rank Ordered List

  

| SYMBOL | RANK IN GENE LIST | RANK METRIC SCORE | RUNNING ES | CORE ENRICHMENT || 1 | Rab31 | 156 | 1.775 | -0.0144 | No |
| 2 | Gja1 | 186 | 1.653 | 0.0012 | No |
| 3 | Mreg | 224 | 1.514 | 0.0128 | No |
| 4 | Gla | 228 | 1.504 | 0.0328 | No |
| 5 | Krt13 | 235 | 1.480 | 0.0516 | No |
| 6 | Klk10 | 281 | 1.373 | 0.0593 | No |
| 7 | Car12 | 306 | 1.274 | 0.0709 | No |
| 8 | Wfs1 | 310 | 1.262 | 0.0875 | No |
| 9 | Cyp26b1 | 316 | 1.222 | 0.1031 | No |
| 10 | Slc7a2 | 370 | 1.114 | 0.1053 | No |
| 11 | Sult2b1 | 497 | 0.901 | 0.0863 | No |
| 12 | Sh3bp5 | 508 | 0.891 | 0.0961 | No |
| 13 | Elf3 | 561 | 0.835 | 0.0947 | No |
| 14 | Cd44 | 562 | 0.834 | 0.1061 | No |
| 15 | Klf4 | 662 | 0.701 | 0.0912 | No |
| 16 | Slc1a1 | 715 | 0.656 | 0.0873 | No |
| 17 | Tubb2b | 756 | 0.624 | 0.0859 | No |
| 18 | Clic3 | 784 | 0.601 | 0.0875 | No |
| 19 | Sfn | 834 | 0.573 | 0.0832 | No |
| 20 | Pmaip1 | 935 | 0.514 | 0.0654 | No |
| 21 | Il6st | 1089 | -0.520 | 0.0345 | No |
| 22 | Svil | 1206 | -0.542 | 0.0131 | No |
| 23 | Bag1 | 1219 | -0.543 | 0.0176 | No |
| 24 | Amfr | 1358 | -0.567 | -0.0090 | No |
| 25 | Krt8 | 1410 | -0.578 | -0.0137 | No |
| 26 | Esrp2 | 1476 | -0.588 | -0.0217 | No |
| 27 | Plaat3 | 1603 | -0.611 | -0.0447 | No |
| 28 | Siah2 | 1781 | -0.644 | -0.0798 | No |
| 29 | Arl3 | 1918 | -0.678 | -0.1043 | No |
| 30 | Flnb | 2016 | -0.695 | -0.1189 | No |
| 31 | Myof | 2057 | -0.709 | -0.1191 | No |
| 32 | Med24 | 2103 | -0.712 | -0.1204 | No |
| 33 | Igf1r | 2178 | -0.734 | -0.1287 | No |
| 34 | Itpk1 | 2209 | -0.740 | -0.1260 | No |
| 35 | Ppif | 2248 | -0.750 | -0.1251 | No |
| 36 | Slc37a1 | 2517 | -0.823 | -0.1805 | No |
| 37 | Akap1 | 2565 | -0.838 | -0.1806 | No |
| 38 | Slc19a2 | 2571 | -0.840 | -0.1703 | No |
| 39 | Tob1 | 2615 | -0.853 | -0.1693 | No |
| 40 | Ptges | 2616 | -0.854 | -0.1575 | No |
| 41 | Areg | 2629 | -0.856 | -0.1487 | No |
| 42 | Xbp1 | 2672 | -0.867 | -0.1472 | No |
| 43 | Celsr1 | 2695 | -0.874 | -0.1407 | No |
| 44 | Asb13 | 2734 | -0.886 | -0.1379 | No |
| 45 | Elovl5 | 2766 | -0.895 | -0.1333 | No |
| 46 | Pex11a | 3020 | -0.982 | -0.1827 | No |
| 47 | Reep1 | 3093 | -1.015 | -0.1866 | Yes |
| 48 | Inpp5f | 3127 | -1.029 | -0.1807 | Yes |
| 49 | Krt19 | 3163 | -1.044 | -0.1750 | Yes |
| 50 | Ttc39a | 3186 | -1.056 | -0.1660 | Yes |
| 51 | Fos | 3189 | -1.058 | -0.1519 | Yes |
| 52 | Tbc1d30 | 3199 | -1.064 | -0.1395 | Yes |
| 53 | Mast4 | 3231 | -1.083 | -0.1323 | Yes |
| 54 | Ugcg | 3258 | -1.098 | -0.1237 | Yes |
| 55 | Ccnd1 | 3303 | -1.112 | -0.1193 | Yes |
| 56 | Rbbp8 | 3346 | -1.138 | -0.1141 | Yes |
| 57 | Prss23 | 3509 | -1.216 | -0.1376 | Yes |
| 58 | Lrig1 | 3543 | -1.241 | -0.1288 | Yes |
| 59 | Ovol2 | 3553 | -1.249 | -0.1138 | Yes |
| 60 | Myb | 3611 | -1.297 | -0.1101 | Yes |
| 61 | Nadsyn1 | 3683 | -1.364 | -0.1090 | Yes |
| 62 | Elf1 | 3720 | -1.405 | -0.0986 | Yes |
| 63 | Hspb8 | 3763 | -1.451 | -0.0891 | Yes |
| 64 | Muc1 | 3926 | -1.769 | -0.1050 | Yes |
| 65 | Slc26a2 | 3936 | -1.780 | -0.0828 | Yes |
| 66 | Tmprss3 | 3966 | -1.914 | -0.0636 | Yes |
| 67 | Stc2 | 3990 | -2.075 | -0.0408 | Yes |
| 68 | Sybu | 3991 | -2.075 | -0.0123 | Yes |
| 69 | Krt15 | 4063 | -2.644 | 0.0065 | Yes |
Table: GSEA details [plain text format]

  

Fig 2: HALLMARK\_ESTROGEN\_RESPONSE\_EARLY: Random ES distribution      
 Gene set null distribution of ES for **HALLMARK\_ESTROGEN\_RESPONSE\_EARLY**

  
