# Supplementary material for: TMPRSS11B promotes an acidified microenvironment and immune suppression in squamous lung cancer
Source: EMBO Rep. 2025 Nov 10;26(24):6346–79. doi: 10.1038/s44319-025-00631-1 (PMC12714794; doi:10.1038/s44319-025-00631-1)
Supplement: Supplementary file 8 — Source data Fig. 3 [file 44319_2025_631_MOESM8_ESM.zip › Figure 3/3D-E/GSEA_Broad Institute_Mh_T11b high vs low LUSC/HALLMARK_ESTROGEN_RESPONSE_LATE.html]

Details for gene set HALLMARK\_ESTROGEN\_RESPONSE\_LATE[GSEA]

|  || Dataset | T11b high vs low squamous\_GSEA\_Ranked |
| Phenotype | NoPhenotypeAvailable |
| Upregulated in class | na\_neg |
| GeneSet | HALLMARK\_ESTROGEN\_RESPONSE\_LATE |
| Enrichment Score (ES) | -0.18052626 |
| Normalized Enrichment Score (NES) | -1.0022943 |
| Nominal p-value | 0.44517186 |
| FDR q-value | 0.9967457 |
| FWER p-Value | 1.0 |
Table: GSEA Results Summary

  

Fig 1: Enrichment plot: HALLMARK\_ESTROGEN\_RESPONSE\_LATE      
 Profile of the Running ES Score & Positions of GeneSet Members on the Rank Ordered List

  

| SYMBOL | RANK IN GENE LIST | RANK METRIC SCORE | RUNNING ES | CORE ENRICHMENT || 1 | S100a9 | 82 | 2.366 | 0.0139 | No |
| 2 | Rab31 | 156 | 1.775 | 0.0214 | No |
| 3 | Gla | 228 | 1.504 | 0.0256 | No |
| 4 | Krt13 | 235 | 1.480 | 0.0455 | No |
| 5 | Cxcl14 | 256 | 1.437 | 0.0613 | No |
| 6 | Ckb | 267 | 1.414 | 0.0793 | No |
| 7 | Klk10 | 281 | 1.373 | 0.0959 | No |
| 8 | Car12 | 306 | 1.274 | 0.1084 | No |
| 9 | Wfs1 | 310 | 1.262 | 0.1259 | No |
| 10 | Cyp26b1 | 316 | 1.222 | 0.1424 | No |
| 11 | Fabp5 | 385 | 1.088 | 0.1412 | No |
| 12 | Pkp3 | 402 | 1.044 | 0.1523 | No |
| 13 | Sult2b1 | 497 | 0.901 | 0.1420 | No |
| 14 | Cd44 | 562 | 0.834 | 0.1382 | No |
| 15 | Klf4 | 662 | 0.701 | 0.1237 | No |
| 16 | Clic3 | 784 | 0.601 | 0.1024 | No |
| 17 | Sfn | 834 | 0.573 | 0.0985 | No |
| 18 | Jak1 | 896 | 0.537 | 0.0911 | No |
| 19 | Il6st | 1089 | -0.520 | 0.0509 | No |
| 20 | Bag1 | 1219 | -0.543 | 0.0267 | No |
| 21 | Unc13b | 1356 | -0.566 | 0.0012 | No |
| 22 | Amfr | 1358 | -0.567 | 0.0091 | No |
| 23 | Tst | 1483 | -0.590 | -0.0132 | No |
| 24 | Etfb | 1565 | -0.605 | -0.0245 | No |
| 25 | Plaat3 | 1603 | -0.611 | -0.0249 | No |
| 26 | Aldh3a2 | 1699 | -0.630 | -0.0394 | No |
| 27 | Siah2 | 1781 | -0.644 | -0.0502 | No |
| 28 | Ltf | 1834 | -0.657 | -0.0536 | No |
| 29 | Arl3 | 1918 | -0.678 | -0.0644 | No |
| 30 | Flnb | 2016 | -0.695 | -0.0784 | No |
| 31 | Myof | 2057 | -0.709 | -0.0781 | No |
| 32 | Mettl3 | 2102 | -0.712 | -0.0787 | No |
| 33 | Itpk1 | 2209 | -0.740 | -0.0943 | No |
| 34 | Ppif | 2248 | -0.750 | -0.0929 | No |
| 35 | Plxnb1 | 2472 | -0.812 | -0.1366 | No |
| 36 | Lsr | 2522 | -0.825 | -0.1368 | No |
| 37 | Sgk1 | 2539 | -0.829 | -0.1288 | No |
| 38 | Tob1 | 2615 | -0.853 | -0.1351 | No |
| 39 | Ptges | 2616 | -0.854 | -0.1227 | No |
| 40 | Areg | 2629 | -0.856 | -0.1133 | No |
| 41 | Xbp1 | 2672 | -0.867 | -0.1112 | No |
| 42 | Dnajc1 | 2741 | -0.887 | -0.1152 | No |
| 43 | Elovl5 | 2766 | -0.895 | -0.1083 | No |
| 44 | Fgfr3 | 2850 | -0.925 | -0.1155 | No |
| 45 | Cdh1 | 2989 | -0.975 | -0.1357 | No |
| 46 | Krt19 | 3163 | -1.044 | -0.1635 | No |
| 47 | Fos | 3189 | -1.058 | -0.1544 | No |
| 48 | Ass1 | 3295 | -1.111 | -0.1645 | Yes |
| 49 | Ccnd1 | 3303 | -1.112 | -0.1501 | Yes |
| 50 | Rbbp8 | 3346 | -1.138 | -0.1441 | Yes |
| 51 | St6galnac2 | 3423 | -1.174 | -0.1459 | Yes |
| 52 | Pdcd4 | 3488 | -1.204 | -0.1444 | Yes |
| 53 | Dnajc12 | 3489 | -1.204 | -0.1270 | Yes |
| 54 | Prss23 | 3509 | -1.216 | -0.1141 | Yes |
| 55 | Ovol2 | 3553 | -1.249 | -0.1067 | Yes |
| 56 | Myb | 3611 | -1.297 | -0.1021 | Yes |
| 57 | Isg20 | 3663 | -1.348 | -0.0953 | Yes |
| 58 | Agr2 | 3714 | -1.397 | -0.0875 | Yes |
| 59 | Idh2 | 3729 | -1.416 | -0.0705 | Yes |
| 60 | Hspb8 | 3763 | -1.451 | -0.0577 | Yes |
| 61 | Dcxr | 3823 | -1.555 | -0.0498 | Yes |
| 62 | Slc26a2 | 3936 | -1.780 | -0.0519 | Yes |
| 63 | Homer2 | 3946 | -1.809 | -0.0279 | Yes |
| 64 | Tmprss3 | 3966 | -1.914 | -0.0049 | Yes |
| 65 | Hmgcs2 | 4048 | -2.436 | 0.0102 | Yes |
Table: GSEA details [plain text format]

  

Fig 2: HALLMARK\_ESTROGEN\_RESPONSE\_LATE: Random ES distribution      
 Gene set null distribution of ES for **HALLMARK\_ESTROGEN\_RESPONSE\_LATE**

  
