# Supplementary material for: TMPRSS11B promotes an acidified microenvironment and immune suppression in squamous lung cancer
Source: EMBO Rep. 2025 Nov 10;26(24):6346–79. doi: 10.1038/s44319-025-00631-1 (PMC12714794; doi:10.1038/s44319-025-00631-1)
Supplement: Supplementary file 8 — Source data Fig. 3 [file 44319_2025_631_MOESM8_ESM.zip › Figure 3/3D-E/GSEA_Broad Institute_Mh_T11b high vs low LUSC/HALLMARK_G2M_CHECKPOINT.html]

Details for gene set HALLMARK\_G2M\_CHECKPOINT[GSEA]

|  || Dataset | T11b high vs low squamous\_GSEA\_Ranked |
| Phenotype | NoPhenotypeAvailable |
| Upregulated in class | na\_neg |
| GeneSet | HALLMARK\_G2M\_CHECKPOINT |
| Enrichment Score (ES) | -0.15707582 |
| Normalized Enrichment Score (NES) | -0.7454116 |
| Nominal p-value | 0.81569964 |
| FDR q-value | 0.81650054 |
| FWER p-Value | 1.0 |
Table: GSEA Results Summary

  

Fig 1: Enrichment plot: HALLMARK\_G2M\_CHECKPOINT      
 Profile of the Running ES Score & Positions of GeneSet Members on the Rank Ordered List

  

| SYMBOL | RANK IN GENE LIST | RANK METRIC SCORE | RUNNING ES | CORE ENRICHMENT || 1 | Map3k20 | 636 | 0.723 | -0.1343 | Yes |
| 2 | Hif1a | 684 | 0.683 | -0.1243 | Yes |
| 3 | Ccna2 | 694 | 0.673 | -0.1053 | Yes |
| 4 | Mcm3 | 697 | 0.670 | -0.0847 | Yes |
| 5 | Cdk1 | 724 | 0.651 | -0.0706 | Yes |
| 6 | Cks2 | 812 | 0.588 | -0.0735 | Yes |
| 7 | Mad2l1 | 850 | 0.566 | -0.0648 | Yes |
| 8 | Cenpa | 892 | 0.542 | -0.0578 | Yes |
| 9 | Uck2 | 1166 | -0.533 | -0.1084 | Yes |
| 10 | Tent4a | 1213 | -0.542 | -0.1027 | Yes |
| 11 | Orc5 | 1240 | -0.546 | -0.0919 | Yes |
| 12 | Smarcc1 | 1250 | -0.548 | -0.0768 | Yes |
| 13 | Odf2 | 1431 | -0.581 | -0.1029 | Yes |
| 14 | Smad3 | 1449 | -0.584 | -0.0887 | Yes |
| 15 | Odc1 | 1542 | -0.601 | -0.0924 | Yes |
| 16 | Numa1 | 1560 | -0.605 | -0.0776 | Yes |
| 17 | Srsf1 | 1572 | -0.606 | -0.0611 | Yes |
| 18 | Mtf2 | 1576 | -0.607 | -0.0427 | Yes |
| 19 | Ilf3 | 1590 | -0.609 | -0.0267 | Yes |
| 20 | Cul1 | 1732 | -0.635 | -0.0415 | Yes |
| 21 | Bub3 | 1742 | -0.637 | -0.0236 | Yes |
| 22 | Ythdc1 | 1786 | -0.646 | -0.0139 | Yes |
| 23 | Lig3 | 1871 | -0.667 | -0.0136 | Yes |
| 24 | Arid4a | 1879 | -0.669 | 0.0058 | Yes |
| 25 | Prpf4b | 1961 | -0.686 | 0.0074 | Yes |
| 26 | Pura | 2032 | -0.698 | 0.0122 | Yes |
| 27 | Nsd2 | 2154 | -0.730 | 0.0053 | Yes |
| 28 | Slc38a1 | 2233 | -0.747 | 0.0096 | Yes |
| 29 | Nek2 | 2485 | -0.816 | -0.0266 | Yes |
| 30 | Pds5b | 2511 | -0.822 | -0.0069 | Yes |
| 31 | Tle3 | 2595 | -0.846 | -0.0007 | Yes |
| 32 | Mnat1 | 2739 | -0.887 | -0.0080 | Yes |
| 33 | Prmt5 | 2797 | -0.905 | 0.0064 | Yes |
| 34 | Cul5 | 2799 | -0.905 | 0.0347 | Yes |
| 35 | Cdc25a | 2998 | -0.975 | 0.0166 | Yes |
| 36 | Kif5b | 3281 | -1.107 | -0.0182 | Yes |
| 37 | Ccnd1 | 3303 | -1.112 | 0.0117 | Yes |
| 38 | Fancc | 3326 | -1.127 | 0.0419 | Yes |
| 39 | Efna5 | 3407 | -1.163 | 0.0588 | Yes |
| 40 | Xpo1 | 3499 | -1.210 | 0.0745 | Yes |
| 41 | Slc12a2 | 4021 | -2.250 | 0.0168 | Yes |
Table: GSEA details [plain text format]

  

Fig 2: HALLMARK\_G2M\_CHECKPOINT: Random ES distribution      
 Gene set null distribution of ES for **HALLMARK\_G2M\_CHECKPOINT**

  
