# Supplementary material for: TMPRSS11B promotes an acidified microenvironment and immune suppression in squamous lung cancer
Source: EMBO Rep. 2025 Nov 10;26(24):6346–79. doi: 10.1038/s44319-025-00631-1 (PMC12714794; doi:10.1038/s44319-025-00631-1)
Supplement: Supplementary file 8 — Source data Fig. 3 [file 44319_2025_631_MOESM8_ESM.zip › Figure 3/3D-E/GSEA_Broad Institute_Mh_T11b high vs low LUSC/HALLMARK_HYPOXIA.html]

Details for gene set HALLMARK\_HYPOXIA[GSEA]

|  || Dataset | T11b high vs low squamous\_GSEA\_Ranked |
| Phenotype | NoPhenotypeAvailable |
| Upregulated in class | na\_pos |
| GeneSet | HALLMARK\_HYPOXIA |
| Enrichment Score (ES) | 0.3766922 |
| Normalized Enrichment Score (NES) | 2.1852589 |
| Nominal p-value | 0.0 |
| FDR q-value | 8.840587E-4 |
| FWER p-Value | 0.005 |
Table: GSEA Results Summary

  

Fig 1: Enrichment plot: HALLMARK\_HYPOXIA      
 Profile of the Running ES Score & Positions of GeneSet Members on the Rank Ordered List

  

| SYMBOL | RANK IN GENE LIST | RANK METRIC SCORE | RUNNING ES | CORE ENRICHMENT || 1 | Hmox1 | 55 | 2.684 | 0.0233 | Yes |
| 2 | Serpine1 | 89 | 2.295 | 0.0468 | Yes |
| 3 | Glrx | 105 | 2.127 | 0.0724 | Yes |
| 4 | Igfbp3 | 124 | 1.955 | 0.0949 | Yes |
| 5 | Tgfbi | 151 | 1.825 | 0.1136 | Yes |
| 6 | Gpc1 | 155 | 1.783 | 0.1375 | Yes |
| 7 | Cdkn1a | 192 | 1.625 | 0.1509 | Yes |
| 8 | Sdc3 | 196 | 1.610 | 0.1724 | Yes |
| 9 | Plin2 | 217 | 1.534 | 0.1885 | Yes |
| 10 | Dusp1 | 239 | 1.468 | 0.2036 | Yes |
| 11 | Pkp1 | 257 | 1.434 | 0.2191 | Yes |
| 12 | Pim1 | 262 | 1.425 | 0.2378 | Yes |
| 13 | Ndrg1 | 276 | 1.380 | 0.2536 | Yes |
| 14 | Ccng2 | 288 | 1.347 | 0.2694 | Yes |
| 15 | Cxcr4 | 289 | 1.347 | 0.2880 | Yes |
| 16 | Car12 | 306 | 1.274 | 0.3016 | Yes |
| 17 | Col5a1 | 348 | 1.150 | 0.3073 | Yes |
| 18 | Lox | 352 | 1.139 | 0.3222 | Yes |
| 19 | Atf3 | 373 | 1.107 | 0.3325 | Yes |
| 20 | Ets1 | 396 | 1.061 | 0.3417 | Yes |
| 21 | Plaur | 426 | 1.012 | 0.3484 | Yes |
| 22 | Mif | 484 | 0.921 | 0.3470 | Yes |
| 23 | Sult2b1 | 497 | 0.901 | 0.3564 | Yes |
| 24 | Ppp1r15a | 552 | 0.843 | 0.3546 | Yes |
| 25 | Gcnt2 | 594 | 0.790 | 0.3553 | Yes |
| 26 | Ndst1 | 609 | 0.756 | 0.3623 | Yes |
| 27 | Rora | 622 | 0.740 | 0.3695 | Yes |
| 28 | Cited2 | 695 | 0.672 | 0.3609 | Yes |
| 29 | Pgk1 | 717 | 0.655 | 0.3647 | Yes |
| 30 | P4ha1 | 720 | 0.654 | 0.3732 | Yes |
| 31 | Anxa2 | 760 | 0.620 | 0.3721 | Yes |
| 32 | Maff | 776 | 0.605 | 0.3767 | Yes |
| 33 | Fam162a | 909 | 0.527 | 0.3512 | No |
| 34 | Ackr3 | 918 | 0.524 | 0.3564 | No |
| 35 | Xpnpep1 | 947 | 0.507 | 0.3564 | No |
| 36 | Ids | 965 | -0.501 | 0.3591 | No |
| 37 | Hdlbp | 984 | -0.503 | 0.3616 | No |
| 38 | Pklr | 1212 | -0.542 | 0.3127 | No |
| 39 | Ilvbl | 1274 | -0.551 | 0.3051 | No |
| 40 | Gaa | 1539 | -0.600 | 0.2478 | No |
| 41 | Pgm1 | 1734 | -0.635 | 0.2084 | No |
| 42 | Siah2 | 1781 | -0.644 | 0.2058 | No |
| 43 | Irs2 | 1906 | -0.674 | 0.1843 | No |
| 44 | Nr3c1 | 2083 | -0.712 | 0.1504 | No |
| 45 | Gpc3 | 2218 | -0.743 | 0.1273 | No |
| 46 | Cp | 2276 | -0.756 | 0.1236 | No |
| 47 | Pgm2 | 2316 | -0.767 | 0.1245 | No |
| 48 | Vldlr | 2403 | -0.792 | 0.1141 | No |
| 49 | Eno3 | 2415 | -0.794 | 0.1223 | No |
| 50 | B3galt6 | 2717 | -0.880 | 0.0596 | No |
| 51 | Cdkn1c | 2722 | -0.882 | 0.0708 | No |
| 52 | Hs3st1 | 2845 | -0.921 | 0.0532 | No |
| 53 | Gpc4 | 3169 | -1.047 | -0.0126 | No |
| 54 | Fos | 3189 | -1.058 | -0.0027 | No |
| 55 | Akap12 | 3203 | -1.066 | 0.0087 | No |
| 56 | Prkca | 3358 | -1.142 | -0.0138 | No |
| 57 | Klhl24 | 3551 | -1.247 | -0.0443 | No |
| 58 | Isg20 | 3663 | -1.348 | -0.0533 | No |
| 59 | Ppargc1a | 3701 | -1.384 | -0.0434 | No |
| 60 | Nedd4l | 3758 | -1.449 | -0.0373 | No |
| 61 | Ankzf1 | 3772 | -1.458 | -0.0204 | No |
| 62 | Dtna | 3836 | -1.581 | -0.0143 | No |
| 63 | Kdelr3 | 3903 | -1.712 | -0.0071 | No |
| 64 | Pam | 3917 | -1.755 | 0.0139 | No |
| 65 | Stc2 | 3990 | -2.075 | 0.0246 | No |
Table: GSEA details [plain text format]

  

Fig 2: HALLMARK\_HYPOXIA: Random ES distribution      
 Gene set null distribution of ES for **HALLMARK\_HYPOXIA**

  
