# Supplementary material for: TMPRSS11B promotes an acidified microenvironment and immune suppression in squamous lung cancer
Source: EMBO Rep. 2025 Nov 10;26(24):6346–79. doi: 10.1038/s44319-025-00631-1 (PMC12714794; doi:10.1038/s44319-025-00631-1)
Supplement: Supplementary file 8 — Source data Fig. 3 [file 44319_2025_631_MOESM8_ESM.zip › Figure 3/3D-E/GSEA_Broad Institute_Mh_T11b high vs low LUSC/HALLMARK_IL2_STAT5_SIGNALING.html]

Details for gene set HALLMARK\_IL2\_STAT5\_SIGNALING[GSEA]

|  || Dataset | T11b high vs low squamous\_GSEA\_Ranked |
| Phenotype | NoPhenotypeAvailable |
| Upregulated in class | na\_pos |
| GeneSet | HALLMARK\_IL2\_STAT5\_SIGNALING |
| Enrichment Score (ES) | 0.34934667 |
| Normalized Enrichment Score (NES) | 2.0784485 |
| Nominal p-value | 0.0 |
| FDR q-value | 0.0029903937 |
| FWER p-Value | 0.019 |
Table: GSEA Results Summary

  

Fig 1: Enrichment plot: HALLMARK\_IL2\_STAT5\_SIGNALING      
 Profile of the Running ES Score & Positions of GeneSet Members on the Rank Ordered List

  

| SYMBOL | RANK IN GENE LIST | RANK METRIC SCORE | RUNNING ES | CORE ENRICHMENT || 1 | Spp1 | 13 | 4.054 | 0.0555 | Yes |
| 2 | Ecm1 | 31 | 3.325 | 0.0994 | Yes |
| 3 | Mxd1 | 69 | 2.527 | 0.1268 | Yes |
| 4 | Emp1 | 133 | 1.901 | 0.1386 | Yes |
| 5 | Ctsz | 138 | 1.884 | 0.1649 | Yes |
| 6 | Plpp1 | 153 | 1.798 | 0.1875 | Yes |
| 7 | Aplp1 | 161 | 1.747 | 0.2110 | Yes |
| 8 | Il1r2 | 188 | 1.630 | 0.2282 | Yes |
| 9 | Plin2 | 217 | 1.534 | 0.2434 | Yes |
| 10 | Ager | 225 | 1.511 | 0.2636 | Yes |
| 11 | Pim1 | 262 | 1.425 | 0.2753 | Yes |
| 12 | Ndrg1 | 276 | 1.380 | 0.2920 | Yes |
| 13 | Capg | 343 | 1.160 | 0.2924 | Yes |
| 14 | Gpr65 | 401 | 1.047 | 0.2934 | Yes |
| 15 | Col6a1 | 415 | 1.025 | 0.3050 | Yes |
| 16 | Tnfrsf1b | 463 | 0.955 | 0.3072 | Yes |
| 17 | Gadd45b | 522 | 0.873 | 0.3054 | Yes |
| 18 | Il3ra | 530 | 0.869 | 0.3162 | Yes |
| 19 | Gsto1 | 543 | 0.850 | 0.3256 | Yes |
| 20 | Cd44 | 562 | 0.834 | 0.3331 | Yes |
| 21 | Rora | 622 | 0.740 | 0.3292 | Yes |
| 22 | Ckap4 | 673 | 0.690 | 0.3268 | Yes |
| 23 | P2rx4 | 707 | 0.663 | 0.3282 | Yes |
| 24 | P4ha1 | 720 | 0.654 | 0.3347 | Yes |
| 25 | Hipk2 | 725 | 0.651 | 0.3431 | Yes |
| 26 | Adam19 | 738 | 0.638 | 0.3493 | Yes |
| 27 | Maff | 776 | 0.605 | 0.3489 | No |
| 28 | Dennd5a | 827 | 0.576 | 0.3448 | No |
| 29 | Prnp | 853 | 0.565 | 0.3468 | No |
| 30 | Bcl2l1 | 878 | 0.552 | 0.3488 | No |
| 31 | Snx14 | 1101 | -0.523 | 0.3013 | No |
| 32 | Uck2 | 1166 | -0.533 | 0.2931 | No |
| 33 | Fah | 1257 | -0.549 | 0.2787 | No |
| 34 | Ptrh2 | 1414 | -0.578 | 0.2483 | No |
| 35 | Odc1 | 1542 | -0.601 | 0.2254 | No |
| 36 | Pdcd2l | 1614 | -0.612 | 0.2167 | No |
| 37 | Ccnd3 | 2040 | -0.700 | 0.1212 | No |
| 38 | Spred2 | 2041 | -0.700 | 0.1314 | No |
| 39 | Pus1 | 2048 | -0.703 | 0.1401 | No |
| 40 | Enpp1 | 2167 | -0.732 | 0.1214 | No |
| 41 | Igf1r | 2178 | -0.734 | 0.1295 | No |
| 42 | Rabgap1l | 2252 | -0.751 | 0.1223 | No |
| 43 | Ptch1 | 2275 | -0.756 | 0.1277 | No |
| 44 | Eno3 | 2415 | -0.794 | 0.1047 | No |
| 45 | Alcam | 2584 | -0.843 | 0.0752 | No |
| 46 | Tnfsf10 | 2592 | -0.845 | 0.0857 | No |
| 47 | Gucy1b1 | 2612 | -0.852 | 0.0933 | No |
| 48 | Dcps | 2660 | -0.864 | 0.0941 | No |
| 49 | Xbp1 | 2672 | -0.867 | 0.1040 | No |
| 50 | Tnfrsf21 | 2673 | -0.868 | 0.1165 | No |
| 51 | Eef1akmt1 | 2719 | -0.882 | 0.1181 | No |
| 52 | Cdkn1c | 2722 | -0.882 | 0.1304 | No |
| 53 | Il18r1 | 2742 | -0.887 | 0.1385 | No |
| 54 | Amacr | 2833 | -0.917 | 0.1294 | No |
| 55 | Spry4 | 2861 | -0.930 | 0.1362 | No |
| 56 | Ahr | 2936 | -0.953 | 0.1316 | No |
| 57 | Etfbkmt | 3079 | -1.010 | 0.1110 | No |
| 58 | Galm | 3177 | -1.050 | 0.1021 | No |
| 59 | Ncoa3 | 3306 | -1.113 | 0.0864 | No |
| 60 | Lrig1 | 3543 | -1.241 | 0.0457 | No |
| 61 | Lclat1 | 3607 | -1.297 | 0.0489 | No |
| 62 | Flt3l | 3704 | -1.390 | 0.0451 | No |
| 63 | Slc1a5 | 3889 | -1.685 | 0.0238 | No |
| 64 | Muc1 | 3926 | -1.769 | 0.0405 | No |
Table: GSEA details [plain text format]

  

Fig 2: HALLMARK\_IL2\_STAT5\_SIGNALING: Random ES distribution      
 Gene set null distribution of ES for **HALLMARK\_IL2\_STAT5\_SIGNALING**

  
