# Supplementary material for: TMPRSS11B promotes an acidified microenvironment and immune suppression in squamous lung cancer
Source: EMBO Rep. 2025 Nov 10;26(24):6346–79. doi: 10.1038/s44319-025-00631-1 (PMC12714794; doi:10.1038/s44319-025-00631-1)
Supplement: Supplementary file 8 — Source data Fig. 3 [file 44319_2025_631_MOESM8_ESM.zip › Figure 3/3D-E/GSEA_Broad Institute_Mh_T11b high vs low LUSC/HALLMARK_IL6_JAK_STAT3_SIGNALING.html]

Details for gene set HALLMARK\_IL6\_JAK\_STAT3\_SIGNALING[GSEA]

|  || Dataset | T11b high vs low squamous\_GSEA\_Ranked |
| Phenotype | NoPhenotypeAvailable |
| Upregulated in class | na\_pos |
| GeneSet | HALLMARK\_IL6\_JAK\_STAT3\_SIGNALING |
| Enrichment Score (ES) | 0.635102 |
| Normalized Enrichment Score (NES) | 2.694919 |
| Nominal p-value | 0.0 |
| FDR q-value | 0.0 |
| FWER p-Value | 0.0 |
Table: GSEA Results Summary

  

Fig 1: Enrichment plot: HALLMARK\_IL6\_JAK\_STAT3\_SIGNALING      
 Profile of the Running ES Score & Positions of GeneSet Members on the Rank Ordered List

  

| SYMBOL | RANK IN GENE LIST | RANK METRIC SCORE | RUNNING ES | CORE ENRICHMENT || 1 | Hmox1 | 55 | 2.684 | 0.0798 | Yes |
| 2 | Cd36 | 85 | 2.334 | 0.1539 | Yes |
| 3 | Csf3r | 113 | 2.052 | 0.2187 | Yes |
| 4 | Il1b | 129 | 1.912 | 0.2815 | Yes |
| 5 | Crlf2 | 182 | 1.670 | 0.3268 | Yes |
| 6 | Csf2ra | 185 | 1.657 | 0.3840 | Yes |
| 7 | Il1r2 | 188 | 1.630 | 0.4402 | Yes |
| 8 | Pim1 | 262 | 1.425 | 0.4718 | Yes |
| 9 | Il17ra | 351 | 1.140 | 0.4899 | Yes |
| 10 | Irf1 | 372 | 1.111 | 0.5236 | Yes |
| 11 | Tnfrsf1b | 463 | 0.955 | 0.5347 | Yes |
| 12 | Il10rb | 467 | 0.949 | 0.5670 | Yes |
| 13 | Tnf | 495 | 0.907 | 0.5919 | Yes |
| 14 | Il3ra | 530 | 0.869 | 0.6137 | Yes |
| 15 | Cd44 | 562 | 0.834 | 0.6351 | Yes |
| 16 | Il6st | 1089 | -0.520 | 0.5239 | No |
| 17 | Cbl | 1164 | -0.533 | 0.5242 | No |
| 18 | Acvrl1 | 1592 | -0.610 | 0.4404 | No |
| 19 | Fas | 2311 | -0.764 | 0.2905 | No |
| 20 | Tnfrsf21 | 2673 | -0.868 | 0.2319 | No |
| 21 | Il18r1 | 2742 | -0.887 | 0.2460 | No |
| 22 | Tlr2 | 3387 | -1.156 | 0.1279 | No |
| 23 | Reg1 | 3588 | -1.278 | 0.1232 | No |
Table: GSEA details [plain text format]

  

Fig 2: HALLMARK\_IL6\_JAK\_STAT3\_SIGNALING: Random ES distribution      
 Gene set null distribution of ES for **HALLMARK\_IL6\_JAK\_STAT3\_SIGNALING**

  
