# Supplementary material for: TMPRSS11B promotes an acidified microenvironment and immune suppression in squamous lung cancer
Source: EMBO Rep. 2025 Nov 10;26(24):6346–79. doi: 10.1038/s44319-025-00631-1 (PMC12714794; doi:10.1038/s44319-025-00631-1)
Supplement: Supplementary file 8 — Source data Fig. 3 [file 44319_2025_631_MOESM8_ESM.zip › Figure 3/3D-E/GSEA_Broad Institute_Mh_T11b high vs low LUSC/HALLMARK_INFLAMMATORY_RESPONSE.html]

Details for gene set HALLMARK\_INFLAMMATORY\_RESPONSE[GSEA]

|  || Dataset | T11b high vs low squamous\_GSEA\_Ranked |
| Phenotype | NoPhenotypeAvailable |
| Upregulated in class | na\_pos |
| GeneSet | HALLMARK\_INFLAMMATORY\_RESPONSE |
| Enrichment Score (ES) | 0.4903665 |
| Normalized Enrichment Score (NES) | 2.687352 |
| Nominal p-value | 0.0 |
| FDR q-value | 0.0 |
| FWER p-Value | 0.0 |
Table: GSEA Results Summary

  

Fig 1: Enrichment plot: HALLMARK\_INFLAMMATORY\_RESPONSE      
 Profile of the Running ES Score & Positions of GeneSet Members on the Rank Ordered List

  

| SYMBOL | RANK IN GENE LIST | RANK METRIC SCORE | RUNNING ES | CORE ENRICHMENT || 1 | Cxcl5 | 16 | 3.974 | 0.0519 | Yes |
| 2 | Il1a | 44 | 2.812 | 0.0847 | Yes |
| 3 | C3ar1 | 46 | 2.795 | 0.1237 | Yes |
| 4 | Cybb | 57 | 2.654 | 0.1585 | Yes |
| 5 | Mxd1 | 69 | 2.527 | 0.1913 | Yes |
| 6 | Msr1 | 78 | 2.391 | 0.2229 | Yes |
| 7 | Serpine1 | 89 | 2.295 | 0.2526 | Yes |
| 8 | Csf3r | 113 | 2.052 | 0.2758 | Yes |
| 9 | Emp3 | 115 | 2.020 | 0.3039 | Yes |
| 10 | Il1b | 129 | 1.912 | 0.3275 | Yes |
| 11 | Abca1 | 172 | 1.716 | 0.3412 | Yes |
| 12 | Ptafr | 191 | 1.626 | 0.3596 | Yes |
| 13 | Cdkn1a | 192 | 1.625 | 0.3824 | Yes |
| 14 | Cxcl15 | 238 | 1.470 | 0.3919 | Yes |
| 15 | Lamp3 | 298 | 1.307 | 0.3956 | Yes |
| 16 | Itga5 | 320 | 1.212 | 0.4075 | Yes |
| 17 | Gna15 | 326 | 1.197 | 0.4230 | Yes |
| 18 | Slc7a2 | 370 | 1.114 | 0.4280 | Yes |
| 19 | Irf1 | 372 | 1.111 | 0.4434 | Yes |
| 20 | Plaur | 426 | 1.012 | 0.4445 | Yes |
| 21 | Tnfrsf1b | 463 | 0.955 | 0.4489 | Yes |
| 22 | Nfkb1 | 486 | 0.918 | 0.4564 | Yes |
| 23 | Stab1 | 523 | 0.873 | 0.4597 | Yes |
| 24 | Rgs1 | 526 | 0.873 | 0.4715 | Yes |
| 25 | Pdpn | 544 | 0.850 | 0.4792 | Yes |
| 26 | Irf7 | 548 | 0.847 | 0.4904 | Yes |
| 27 | Sphk1 | 672 | 0.691 | 0.4696 | No |
| 28 | Hif1a | 684 | 0.683 | 0.4764 | No |
| 29 | P2rx4 | 707 | 0.663 | 0.4803 | No |
| 30 | Lcp2 | 840 | 0.570 | 0.4556 | No |
| 31 | Kif1b | 923 | 0.518 | 0.4425 | No |
| 32 | Raf1 | 942 | 0.510 | 0.4452 | No |
| 33 | Rela | 943 | 0.509 | 0.4524 | No |
| 34 | Nfkbia | 954 | 0.502 | 0.4569 | No |
| 35 | Sri | 1116 | -0.525 | 0.4244 | No |
| 36 | Hbegf | 1222 | -0.544 | 0.4060 | No |
| 37 | Aplnr | 1378 | -0.570 | 0.3755 | No |
| 38 | Ptger4 | 1845 | -0.660 | 0.2692 | No |
| 39 | Ly6e | 1857 | -0.663 | 0.2758 | No |
| 40 | Il15 | 1933 | -0.681 | 0.2668 | No |
| 41 | Gpc3 | 2218 | -0.743 | 0.2068 | No |
| 42 | Scn1b | 2310 | -0.764 | 0.1949 | No |
| 43 | Pvr | 2314 | -0.766 | 0.2050 | No |
| 44 | Slc31a1 | 2408 | -0.793 | 0.1931 | No |
| 45 | Calcrl | 2591 | -0.845 | 0.1598 | No |
| 46 | Tnfsf10 | 2592 | -0.845 | 0.1717 | No |
| 47 | Slc4a4 | 2622 | -0.854 | 0.1765 | No |
| 48 | Il18r1 | 2742 | -0.887 | 0.1594 | No |
| 49 | Ahr | 2936 | -0.953 | 0.1249 | No |
| 50 | Selenos | 2961 | -0.960 | 0.1325 | No |
| 51 | Cd82 | 2976 | -0.965 | 0.1426 | No |
| 52 | Gabbr1 | 2985 | -0.969 | 0.1542 | No |
| 53 | Il18 | 3078 | -1.010 | 0.1456 | No |
| 54 | Psen1 | 3136 | -1.034 | 0.1460 | No |
| 55 | Tlr2 | 3387 | -1.156 | 0.1002 | No |
| 56 | Ccl17 | 3767 | -1.456 | 0.0266 | No |
| 57 | Hpn | 3768 | -1.456 | 0.0471 | No |
| 58 | Ereg | 4031 | -2.297 | 0.0144 | No |
Table: GSEA details [plain text format]

  

Fig 2: HALLMARK\_INFLAMMATORY\_RESPONSE: Random ES distribution      
 Gene set null distribution of ES for **HALLMARK\_INFLAMMATORY\_RESPONSE**

  
