# Supplementary material for: TMPRSS11B promotes an acidified microenvironment and immune suppression in squamous lung cancer
Source: EMBO Rep. 2025 Nov 10;26(24):6346–79. doi: 10.1038/s44319-025-00631-1 (PMC12714794; doi:10.1038/s44319-025-00631-1)
Supplement: Supplementary file 8 — Source data Fig. 3 [file 44319_2025_631_MOESM8_ESM.zip › Figure 3/3D-E/GSEA_Broad Institute_Mh_T11b high vs low LUSC/HALLMARK_INTERFERON_GAMMA_RESPONSE.html]

Details for gene set HALLMARK\_INTERFERON\_GAMMA\_RESPONSE[GSEA]

|  || Dataset | T11b high vs low squamous\_GSEA\_Ranked |
| Phenotype | NoPhenotypeAvailable |
| Upregulated in class | na\_pos |
| GeneSet | HALLMARK\_INTERFERON\_GAMMA\_RESPONSE |
| Enrichment Score (ES) | 0.2849115 |
| Normalized Enrichment Score (NES) | 1.4439138 |
| Nominal p-value | 0.052493438 |
| FDR q-value | 0.144295 |
| FWER p-Value | 0.746 |
Table: GSEA Results Summary

  

Fig 1: Enrichment plot: HALLMARK\_INTERFERON\_GAMMA\_RESPONSE      
 Profile of the Running ES Score & Positions of GeneSet Members on the Rank Ordered List

  

| SYMBOL | RANK IN GENE LIST | RANK METRIC SCORE | RUNNING ES | CORE ENRICHMENT || 1 | Cd274 | 96 | 2.227 | 0.0267 | Yes |
| 2 | Cdkn1a | 192 | 1.625 | 0.0400 | Yes |
| 3 | Ifit3 | 248 | 1.449 | 0.0592 | Yes |
| 4 | Pim1 | 262 | 1.425 | 0.0883 | Yes |
| 5 | Slamf7 | 309 | 1.265 | 0.1055 | Yes |
| 6 | Irf1 | 372 | 1.111 | 0.1154 | Yes |
| 7 | Tnfaip2 | 387 | 1.086 | 0.1365 | Yes |
| 8 | Zbp1 | 391 | 1.078 | 0.1601 | Yes |
| 9 | Ifitm2 | 465 | 0.952 | 0.1636 | Yes |
| 10 | Nfkb1 | 486 | 0.918 | 0.1795 | Yes |
| 11 | Upp1 | 492 | 0.908 | 0.1988 | Yes |
| 12 | Trim25 | 516 | 0.882 | 0.2131 | Yes |
| 13 | Irf7 | 548 | 0.847 | 0.2246 | Yes |
| 14 | Ifnar2 | 567 | 0.830 | 0.2390 | Yes |
| 15 | Parp12 | 571 | 0.828 | 0.2570 | Yes |
| 16 | Oasl1 | 663 | 0.701 | 0.2504 | Yes |
| 17 | Hif1a | 684 | 0.683 | 0.2609 | Yes |
| 18 | Serping1 | 730 | 0.645 | 0.2643 | Yes |
| 19 | Lcp2 | 840 | 0.570 | 0.2503 | Yes |
| 20 | Cd74 | 849 | 0.567 | 0.2612 | Yes |
| 21 | Znfx1 | 851 | 0.566 | 0.2737 | Yes |
| 22 | B2m | 860 | 0.563 | 0.2845 | Yes |
| 23 | Casp8 | 920 | 0.520 | 0.2817 | Yes |
| 24 | Nfkbia | 954 | 0.502 | 0.2849 | Yes |
| 25 | Sri | 1116 | -0.525 | 0.2570 | No |
| 26 | Ogfr | 1770 | -0.642 | 0.1100 | No |
| 27 | Ifi27 | 1778 | -0.644 | 0.1229 | No |
| 28 | Ly6e | 1857 | -0.663 | 0.1186 | No |
| 29 | Auts2 | 1865 | -0.665 | 0.1319 | No |
| 30 | Pla2g4a | 1905 | -0.674 | 0.1376 | No |
| 31 | Il15 | 1933 | -0.681 | 0.1463 | No |
| 32 | Rnf213 | 2130 | -0.724 | 0.1142 | No |
| 33 | Fas | 2311 | -0.764 | 0.0870 | No |
| 34 | Tnfsf10 | 2592 | -0.845 | 0.0369 | No |
| 35 | Epsti1 | 2646 | -0.860 | 0.0433 | No |
| 36 | Txnip | 2924 | -0.947 | -0.0037 | No |
| 37 | Tap1 | 3068 | -1.004 | -0.0164 | No |
| 38 | Rapgef6 | 3087 | -1.012 | 0.0021 | No |
| 39 | Lgals3bp | 3172 | -1.049 | 0.0051 | No |
| 40 | Casp4 | 3233 | -1.084 | 0.0148 | No |
| 41 | Ncoa3 | 3306 | -1.113 | 0.0222 | No |
| 42 | Mthfd2 | 3458 | -1.191 | 0.0118 | No |
| 43 | Nod1 | 3491 | -1.205 | 0.0312 | No |
| 44 | Isg20 | 3663 | -1.348 | 0.0194 | No |
| 45 | St3gal5 | 3960 | -1.876 | -0.0113 | No |
| 46 | Oas2 | 3963 | -1.896 | 0.0312 | No |
Table: GSEA details [plain text format]

  

Fig 2: HALLMARK\_INTERFERON\_GAMMA\_RESPONSE: Random ES distribution      
 Gene set null distribution of ES for **HALLMARK\_INTERFERON\_GAMMA\_RESPONSE**

  
