# Supplementary material for: TMPRSS11B promotes an acidified microenvironment and immune suppression in squamous lung cancer
Source: EMBO Rep. 2025 Nov 10;26(24):6346–79. doi: 10.1038/s44319-025-00631-1 (PMC12714794; doi:10.1038/s44319-025-00631-1)
Supplement: Supplementary file 8 — Source data Fig. 3 [file 44319_2025_631_MOESM8_ESM.zip › Figure 3/3D-E/GSEA_Broad Institute_Mh_T11b high vs low LUSC/HALLMARK_KRAS_SIGNALING_DN.html]

Details for gene set HALLMARK\_KRAS\_SIGNALING\_DN[GSEA]

|  || Dataset | T11b high vs low squamous\_GSEA\_Ranked |
| Phenotype | NoPhenotypeAvailable |
| Upregulated in class | na\_neg |
| GeneSet | HALLMARK\_KRAS\_SIGNALING\_DN |
| Enrichment Score (ES) | -0.26090243 |
| Normalized Enrichment Score (NES) | -1.2557131 |
| Nominal p-value | 0.16744186 |
| FDR q-value | 0.5739127 |
| FWER p-Value | 0.993 |
Table: GSEA Results Summary

  

Fig 1: Enrichment plot: HALLMARK\_KRAS\_SIGNALING\_DN      
 Profile of the Running ES Score & Positions of GeneSet Members on the Rank Ordered List

  

| SYMBOL | RANK IN GENE LIST | RANK METRIC SCORE | RUNNING ES | CORE ENRICHMENT || 1 | Sprr3 | 99 | 2.173 | 0.0186 | No |
| 2 | Slc6a14 | 142 | 1.868 | 0.0453 | No |
| 3 | Krt4 | 206 | 1.581 | 0.0610 | No |
| 4 | Krt13 | 235 | 1.480 | 0.0835 | No |
| 5 | Pkp1 | 257 | 1.434 | 0.1067 | No |
| 6 | Tgm1 | 292 | 1.337 | 0.1248 | No |
| 7 | Alox12b | 305 | 1.276 | 0.1471 | No |
| 8 | Lypd3 | 389 | 1.080 | 0.1480 | No |
| 9 | Serpinb2 | 527 | 0.873 | 0.1315 | No |
| 10 | Slc29a3 | 600 | 0.773 | 0.1290 | No |
| 11 | Krt5 | 709 | 0.661 | 0.1155 | No |
| 12 | Lgals7 | 780 | 0.601 | 0.1101 | No |
| 13 | Tgfb2 | 800 | 0.593 | 0.1171 | No |
| 14 | Kmt2d | 1293 | -0.554 | 0.0066 | No |
| 15 | Gtf3c5 | 1528 | -0.599 | -0.0393 | No |
| 16 | Selenop | 2054 | -0.707 | -0.1550 | No |
| 17 | Fggy | 2369 | -0.779 | -0.2171 | No |
| 18 | Mfsd6 | 2379 | -0.784 | -0.2038 | No |
| 19 | Sgk1 | 2539 | -0.829 | -0.2266 | No |
| 20 | Nr6a1 | 2617 | -0.854 | -0.2287 | No |
| 21 | Prodh | 2664 | -0.864 | -0.2229 | No |
| 22 | Sidt1 | 2815 | -0.910 | -0.2419 | Yes |
| 23 | Fgfr3 | 2850 | -0.925 | -0.2320 | Yes |
| 24 | Camk1d | 2895 | -0.938 | -0.2243 | Yes |
| 25 | Nr4a2 | 2921 | -0.946 | -0.2117 | Yes |
| 26 | Asb7 | 2954 | -0.957 | -0.2006 | Yes |
| 27 | Cyp39a1 | 3040 | -0.990 | -0.2020 | Yes |
| 28 | Thrb | 3105 | -1.020 | -0.1976 | Yes |
| 29 | Coq8a | 3108 | -1.021 | -0.1778 | Yes |
| 30 | Cdkal1 | 3147 | -1.037 | -0.1667 | Yes |
| 31 | Msh5 | 3214 | -1.072 | -0.1617 | Yes |
| 32 | Synpo | 3604 | -1.290 | -0.2323 | Yes |
| 33 | Copz2 | 3721 | -1.407 | -0.2330 | Yes |
| 34 | Gprc5c | 3776 | -1.464 | -0.2173 | Yes |
| 35 | Prkn | 3834 | -1.575 | -0.2002 | Yes |
| 36 | Dtnb | 3928 | -1.771 | -0.1881 | Yes |
| 37 | Tfcp2l1 | 3955 | -1.839 | -0.1581 | Yes |
| 38 | Tent5c | 3985 | -2.032 | -0.1250 | Yes |
| 39 | Tff2 | 3999 | -2.109 | -0.0864 | Yes |
| 40 | Krt15 | 4063 | -2.644 | -0.0495 | Yes |
| 41 | Clps | 4075 | -2.812 | 0.0035 | Yes |
Table: GSEA details [plain text format]

  

Fig 2: HALLMARK\_KRAS\_SIGNALING\_DN: Random ES distribution      
 Gene set null distribution of ES for **HALLMARK\_KRAS\_SIGNALING\_DN**

  
