# Supplementary material for: TMPRSS11B promotes an acidified microenvironment and immune suppression in squamous lung cancer
Source: EMBO Rep. 2025 Nov 10;26(24):6346–79. doi: 10.1038/s44319-025-00631-1 (PMC12714794; doi:10.1038/s44319-025-00631-1)
Supplement: Supplementary file 8 — Source data Fig. 3 [file 44319_2025_631_MOESM8_ESM.zip › Figure 3/3D-E/GSEA_Broad Institute_Mh_T11b high vs low LUSC/HALLMARK_KRAS_SIGNALING_UP.html]

Details for gene set HALLMARK\_KRAS\_SIGNALING\_UP[GSEA]

|  || Dataset | T11b high vs low squamous\_GSEA\_Ranked |
| Phenotype | NoPhenotypeAvailable |
| Upregulated in class | na\_pos |
| GeneSet | HALLMARK\_KRAS\_SIGNALING\_UP |
| Enrichment Score (ES) | 0.47710836 |
| Normalized Enrichment Score (NES) | 2.9919906 |
| Nominal p-value | 0.0 |
| FDR q-value | 0.0 |
| FWER p-Value | 0.0 |
Table: GSEA Results Summary

  

Fig 1: Enrichment plot: HALLMARK\_KRAS\_SIGNALING\_UP      
 Profile of the Running ES Score & Positions of GeneSet Members on the Rank Ordered List

  

| SYMBOL | RANK IN GENE LIST | RANK METRIC SCORE | RUNNING ES | CORE ENRICHMENT || 1 | Ppbp | 5 | 5.111 | 0.0479 | Yes |
| 2 | Arg1 | 9 | 4.329 | 0.0888 | Yes |
| 3 | Spp1 | 13 | 4.054 | 0.1271 | Yes |
| 4 | Gpnmb | 27 | 3.467 | 0.1572 | Yes |
| 5 | Plat | 29 | 3.372 | 0.1894 | Yes |
| 6 | C3ar1 | 46 | 2.795 | 0.2123 | Yes |
| 7 | Ctss | 63 | 2.582 | 0.2332 | Yes |
| 8 | Itgb2 | 71 | 2.481 | 0.2553 | Yes |
| 9 | Cd37 | 74 | 2.458 | 0.2784 | Yes |
| 10 | Fcer1g | 76 | 2.415 | 0.3014 | Yes |
| 11 | Glrx | 105 | 2.127 | 0.3149 | Yes |
| 12 | Mafb | 116 | 2.017 | 0.3318 | Yes |
| 13 | Igfbp3 | 124 | 1.955 | 0.3489 | Yes |
| 14 | Il1b | 129 | 1.912 | 0.3663 | Yes |
| 15 | Emp1 | 133 | 1.901 | 0.3839 | Yes |
| 16 | Plau | 136 | 1.895 | 0.4016 | Yes |
| 17 | Csf2ra | 185 | 1.657 | 0.4056 | Yes |
| 18 | Aldh1a3 | 203 | 1.589 | 0.4166 | Yes |
| 19 | Lcp1 | 231 | 1.490 | 0.4243 | Yes |
| 20 | Prdm1 | 245 | 1.452 | 0.4350 | Yes |
| 21 | Mmp9 | 249 | 1.449 | 0.4482 | Yes |
| 22 | Dock2 | 271 | 1.390 | 0.4563 | Yes |
| 23 | Cxcr4 | 289 | 1.347 | 0.4650 | Yes |
| 24 | Mall | 354 | 1.136 | 0.4600 | Yes |
| 25 | Adam8 | 375 | 1.104 | 0.4657 | Yes |
| 26 | Ets1 | 396 | 1.061 | 0.4709 | Yes |
| 27 | Plaur | 426 | 1.012 | 0.4734 | Yes |
| 28 | Tnfrsf1b | 463 | 0.955 | 0.4736 | Yes |
| 29 | Ppp1r15a | 552 | 0.843 | 0.4598 | Yes |
| 30 | Slpi | 558 | 0.836 | 0.4666 | Yes |
| 31 | Mmd | 588 | 0.798 | 0.4671 | Yes |
| 32 | Birc3 | 607 | 0.758 | 0.4699 | Yes |
| 33 | Klf4 | 662 | 0.701 | 0.4632 | Yes |
| 34 | Gng11 | 677 | 0.687 | 0.4663 | Yes |
| 35 | Ace | 681 | 0.685 | 0.4722 | Yes |
| 36 | Etv5 | 712 | 0.660 | 0.4710 | Yes |
| 37 | Lat2 | 714 | 0.657 | 0.4771 | Yes |
| 38 | Jup | 742 | 0.637 | 0.4765 | No |
| 39 | Eng | 867 | 0.560 | 0.4510 | No |
| 40 | Tspan1 | 886 | 0.549 | 0.4518 | No |
| 41 | Ammecr1 | 900 | 0.534 | 0.4537 | No |
| 42 | Tspan7 | 919 | 0.523 | 0.4543 | No |
| 43 | Mpzl2 | 939 | 0.512 | 0.4544 | No |
| 44 | Cbl | 1164 | -0.533 | 0.4038 | No |
| 45 | Hbegf | 1222 | -0.544 | 0.3948 | No |
| 46 | Fbxo4 | 1247 | -0.547 | 0.3941 | No |
| 47 | Mtmr10 | 1309 | -0.558 | 0.3843 | No |
| 48 | Btbd3 | 1540 | -0.600 | 0.3328 | No |
| 49 | Wdr33 | 1597 | -0.610 | 0.3247 | No |
| 50 | Sdccag8 | 1620 | -0.614 | 0.3251 | No |
| 51 | Galnt3 | 1794 | -0.648 | 0.2883 | No |
| 52 | Anxa10 | 1903 | -0.674 | 0.2678 | No |
| 53 | Rabgap1l | 2252 | -0.751 | 0.1884 | No |
| 54 | Ptbp2 | 2268 | -0.755 | 0.1919 | No |
| 55 | Scn1b | 2310 | -0.764 | 0.1891 | No |
| 56 | Dnmbp | 2448 | -0.803 | 0.1627 | No |
| 57 | Usp12 | 2524 | -0.826 | 0.1519 | No |
| 58 | Tmem176b | 2558 | -0.834 | 0.1518 | No |
| 59 | Akt2 | 3033 | -0.987 | 0.0432 | No |
| 60 | Ano1 | 3053 | -0.995 | 0.0480 | No |
| 61 | Pigr | 3054 | -0.996 | 0.0576 | No |
| 62 | Kcnn4 | 3134 | -1.034 | 0.0479 | No |
| 63 | Akap12 | 3203 | -1.066 | 0.0412 | No |
| 64 | Spry2 | 3254 | -1.095 | 0.0393 | No |
| 65 | Plek2 | 3383 | -1.154 | 0.0185 | No |
| 66 | Adamdec1 | 3477 | -1.202 | 0.0069 | No |
| 67 | Trib2 | 3570 | -1.265 | -0.0038 | No |
| 68 | Tmem176a | 3571 | -1.266 | 0.0083 | No |
| 69 | Cab39l | 3659 | -1.344 | -0.0004 | No |
| 70 | Avl9 | 3693 | -1.374 | 0.0046 | No |
| 71 | Sox9 | 3877 | -1.658 | -0.0250 | No |
| 72 | Cidea | 3931 | -1.773 | -0.0212 | No |
| 73 | F2rl1 | 3967 | -1.914 | -0.0115 | No |
| 74 | Tmem158 | 3977 | -2.002 | 0.0055 | No |
| 75 | Ereg | 4031 | -2.297 | 0.0144 | No |
Table: GSEA details [plain text format]

  

Fig 2: HALLMARK\_KRAS\_SIGNALING\_UP: Random ES distribution      
 Gene set null distribution of ES for **HALLMARK\_KRAS\_SIGNALING\_UP**

  
