# Supplementary material for: TMPRSS11B promotes an acidified microenvironment and immune suppression in squamous lung cancer
Source: EMBO Rep. 2025 Nov 10;26(24):6346–79. doi: 10.1038/s44319-025-00631-1 (PMC12714794; doi:10.1038/s44319-025-00631-1)
Supplement: Supplementary file 8 — Source data Fig. 3 [file 44319_2025_631_MOESM8_ESM.zip › Figure 3/3D-E/GSEA_Broad Institute_Mh_T11b high vs low LUSC/HALLMARK_MITOTIC_SPINDLE.html]

Details for gene set HALLMARK\_MITOTIC\_SPINDLE[GSEA]

|  || Dataset | T11b high vs low squamous\_GSEA\_Ranked |
| Phenotype | NoPhenotypeAvailable |
| Upregulated in class | na\_neg |
| GeneSet | HALLMARK\_MITOTIC\_SPINDLE |
| Enrichment Score (ES) | -0.15920834 |
| Normalized Enrichment Score (NES) | -0.7881239 |
| Nominal p-value | 0.75279105 |
| FDR q-value | 0.8268238 |
| FWER p-Value | 1.0 |
Table: GSEA Results Summary

  

Fig 1: Enrichment plot: HALLMARK\_MITOTIC\_SPINDLE      
 Profile of the Running ES Score & Positions of GeneSet Members on the Rank Ordered List

  

| SYMBOL | RANK IN GENE LIST | RANK METRIC SCORE | RUNNING ES | CORE ENRICHMENT || 1 | Prex1 | 204 | 1.586 | -0.0096 | No |
| 2 | Dock2 | 271 | 1.390 | 0.0099 | No |
| 3 | Flna | 471 | 0.946 | -0.0149 | No |
| 4 | Abr | 538 | 0.860 | -0.0091 | No |
| 5 | Epb41l2 | 635 | 0.724 | -0.0142 | No |
| 6 | Palld | 650 | 0.712 | 0.0007 | No |
| 7 | Cdk1 | 724 | 0.651 | -0.0006 | No |
| 8 | Arhgap27 | 911 | 0.526 | -0.0330 | No |
| 9 | Kif1b | 923 | 0.518 | -0.0224 | No |
| 10 | Cdc42ep2 | 926 | 0.517 | -0.0095 | No |
| 11 | Mark4 | 1085 | -0.519 | -0.0352 | No |
| 12 | Map1s | 1174 | -0.535 | -0.0432 | No |
| 13 | Kif3b | 1241 | -0.546 | -0.0454 | No |
| 14 | Kptn | 1333 | -0.563 | -0.0534 | No |
| 15 | Ezr | 1361 | -0.567 | -0.0455 | No |
| 16 | Sos1 | 1487 | -0.591 | -0.0612 | No |
| 17 | Nedd9 | 1559 | -0.605 | -0.0631 | No |
| 18 | Numa1 | 1560 | -0.605 | -0.0475 | No |
| 19 | Rabgap1 | 1619 | -0.614 | -0.0461 | No |
| 20 | Ckap5 | 1767 | -0.642 | -0.0659 | No |
| 21 | Tsc1 | 1813 | -0.651 | -0.0602 | No |
| 22 | Cttn | 1925 | -0.681 | -0.0701 | No |
| 23 | Flnb | 2016 | -0.695 | -0.0745 | No |
| 24 | Atg4b | 2204 | -0.739 | -0.1017 | No |
| 25 | Arfgef1 | 2351 | -0.774 | -0.1178 | No |
| 26 | Sac3d1 | 2416 | -0.794 | -0.1132 | No |
| 27 | Nek2 | 2485 | -0.816 | -0.1090 | No |
| 28 | Tlk1 | 2491 | -0.817 | -0.0891 | No |
| 29 | Dynll2 | 2574 | -0.841 | -0.0878 | No |
| 30 | Rasa2 | 2847 | -0.924 | -0.1312 | Yes |
| 31 | Mid1 | 2923 | -0.946 | -0.1254 | Yes |
| 32 | Cd2ap | 3029 | -0.985 | -0.1259 | Yes |
| 33 | Rapgef6 | 3087 | -1.012 | -0.1140 | Yes |
| 34 | Tubgcp3 | 3271 | -1.102 | -0.1308 | Yes |
| 35 | Kif5b | 3281 | -1.107 | -0.1046 | Yes |
| 36 | Rock1 | 3378 | -1.149 | -0.0987 | Yes |
| 37 | Mid1ip1 | 3392 | -1.158 | -0.0721 | Yes |
| 38 | Uxt | 3422 | -1.171 | -0.0491 | Yes |
| 39 | Pcm1 | 3485 | -1.203 | -0.0334 | Yes |
| 40 | Arfip2 | 3580 | -1.272 | -0.0239 | Yes |
| 41 | Synpo | 3604 | -1.290 | 0.0037 | Yes |
| 42 | Cep131 | 3673 | -1.356 | 0.0218 | Yes |
| 43 | Pcgf5 | 3728 | -1.416 | 0.0449 | Yes |
| 44 | Sorbs2 | 3904 | -1.712 | 0.0457 | Yes |
Table: GSEA details [plain text format]

  

Fig 2: HALLMARK\_MITOTIC\_SPINDLE: Random ES distribution      
 Gene set null distribution of ES for **HALLMARK\_MITOTIC\_SPINDLE**

  
