# Supplementary material for: TMPRSS11B promotes an acidified microenvironment and immune suppression in squamous lung cancer
Source: EMBO Rep. 2025 Nov 10;26(24):6346–79. doi: 10.1038/s44319-025-00631-1 (PMC12714794; doi:10.1038/s44319-025-00631-1)
Supplement: Supplementary file 8 — Source data Fig. 3 [file 44319_2025_631_MOESM8_ESM.zip › Figure 3/3D-E/GSEA_Broad Institute_Mh_T11b high vs low LUSC/HALLMARK_MTORC1_SIGNALING.html]

Details for gene set HALLMARK\_MTORC1\_SIGNALING[GSEA]

|  || Dataset | T11b high vs low squamous\_GSEA\_Ranked |
| Phenotype | NoPhenotypeAvailable |
| Upregulated in class | na\_pos |
| GeneSet | HALLMARK\_MTORC1\_SIGNALING |
| Enrichment Score (ES) | 0.3426817 |
| Normalized Enrichment Score (NES) | 1.9037918 |
| Nominal p-value | 0.0 |
| FDR q-value | 0.009686839 |
| FWER p-Value | 0.071 |
Table: GSEA Results Summary

  

Fig 1: Enrichment plot: HALLMARK\_MTORC1\_SIGNALING      
 Profile of the Running ES Score & Positions of GeneSet Members on the Rank Ordered List

  

| SYMBOL | RANK IN GENE LIST | RANK METRIC SCORE | RUNNING ES | CORE ENRICHMENT || 1 | Itgb2 | 71 | 2.481 | 0.0298 | Yes |
| 2 | Glrx | 105 | 2.127 | 0.0622 | Yes |
| 3 | Slc7a11 | 126 | 1.940 | 0.0943 | Yes |
| 4 | Lgmn | 141 | 1.877 | 0.1267 | Yes |
| 5 | Cfp | 171 | 1.717 | 0.1523 | Yes |
| 6 | Cdkn1a | 192 | 1.625 | 0.1784 | Yes |
| 7 | Sla | 207 | 1.573 | 0.2050 | Yes |
| 8 | Gla | 228 | 1.504 | 0.2288 | Yes |
| 9 | Map2k3 | 282 | 1.367 | 0.2417 | Yes |
| 10 | Cxcr4 | 289 | 1.347 | 0.2660 | Yes |
| 11 | Coro1a | 390 | 1.079 | 0.2618 | Yes |
| 12 | Nupr1 | 443 | 0.988 | 0.2678 | Yes |
| 13 | Egln3 | 532 | 0.866 | 0.2625 | Yes |
| 14 | Ppp1r15a | 552 | 0.843 | 0.2739 | Yes |
| 15 | Serpinh1 | 621 | 0.740 | 0.2712 | Yes |
| 16 | Tfrc | 668 | 0.696 | 0.2731 | Yes |
| 17 | Ifrd1 | 676 | 0.688 | 0.2845 | Yes |
| 18 | Ssr1 | 710 | 0.661 | 0.2889 | Yes |
| 19 | Pgk1 | 717 | 0.655 | 0.2999 | Yes |
| 20 | P4ha1 | 720 | 0.654 | 0.3119 | Yes |
| 21 | Psat1 | 766 | 0.613 | 0.3125 | Yes |
| 22 | Srd5a1 | 769 | 0.610 | 0.3236 | Yes |
| 23 | Shmt2 | 787 | 0.598 | 0.3308 | Yes |
| 24 | Cyp51 | 825 | 0.577 | 0.3327 | Yes |
| 25 | Hmgcr | 885 | 0.549 | 0.3285 | Yes |
| 26 | Rit1 | 921 | 0.518 | 0.3298 | Yes |
| 27 | Insig1 | 945 | 0.509 | 0.3338 | Yes |
| 28 | Got1 | 949 | 0.506 | 0.3427 | Yes |
| 29 | Rpn1 | 1015 | -0.507 | 0.3363 | No |
| 30 | Canx | 1127 | -0.527 | 0.3188 | No |
| 31 | Ung | 1258 | -0.549 | 0.2971 | No |
| 32 | Eef1e1 | 1296 | -0.554 | 0.2985 | No |
| 33 | Nmt1 | 1368 | -0.569 | 0.2918 | No |
| 34 | Hmbs | 1418 | -0.579 | 0.2907 | No |
| 35 | Pno1 | 1502 | -0.595 | 0.2815 | No |
| 36 | Arpc5l | 1558 | -0.604 | 0.2794 | No |
| 37 | Ppa1 | 1606 | -0.612 | 0.2794 | No |
| 38 | Actr2 | 1729 | -0.634 | 0.2613 | No |
| 39 | Pgm1 | 1734 | -0.635 | 0.2724 | No |
| 40 | Mcm4 | 1934 | -0.681 | 0.2361 | No |
| 41 | Immt | 2168 | -0.733 | 0.1924 | No |
| 42 | Etf1 | 2182 | -0.735 | 0.2032 | No |
| 43 | Fads1 | 2281 | -0.757 | 0.1933 | No |
| 44 | Vldlr | 2403 | -0.792 | 0.1785 | No |
| 45 | Psmc4 | 2525 | -0.826 | 0.1643 | No |
| 46 | Xbp1 | 2672 | -0.867 | 0.1447 | No |
| 47 | Gtf2h1 | 2701 | -0.876 | 0.1544 | No |
| 48 | Fdxr | 2703 | -0.876 | 0.1709 | No |
| 49 | Elovl5 | 2766 | -0.895 | 0.1726 | No |
| 50 | Hspa9 | 2925 | -0.947 | 0.1516 | No |
| 51 | Cdc25a | 2998 | -0.975 | 0.1524 | No |
| 52 | Mthfd2 | 3458 | -1.191 | 0.0614 | No |
| 53 | Sytl2 | 3784 | -1.480 | 0.0091 | No |
| 54 | Slc1a5 | 3889 | -1.685 | 0.0155 | No |
| 55 | Igfbp5 | 3929 | -1.772 | 0.0397 | No |
Table: GSEA details [plain text format]

  

Fig 2: HALLMARK\_MTORC1\_SIGNALING: Random ES distribution      
 Gene set null distribution of ES for **HALLMARK\_MTORC1\_SIGNALING**

  
