# Supplementary material for: TMPRSS11B promotes an acidified microenvironment and immune suppression in squamous lung cancer
Source: EMBO Rep. 2025 Nov 10;26(24):6346–79. doi: 10.1038/s44319-025-00631-1 (PMC12714794; doi:10.1038/s44319-025-00631-1)
Supplement: Supplementary file 8 — Source data Fig. 3 [file 44319_2025_631_MOESM8_ESM.zip › Figure 3/3D-E/GSEA_Broad Institute_Mh_T11b high vs low LUSC/HALLMARK_MYOGENESIS.html]

Details for gene set HALLMARK\_MYOGENESIS[GSEA]

|  || Dataset | T11b high vs low squamous\_GSEA\_Ranked |
| Phenotype | NoPhenotypeAvailable |
| Upregulated in class | na\_pos |
| GeneSet | HALLMARK\_MYOGENESIS |
| Enrichment Score (ES) | 0.21741681 |
| Normalized Enrichment Score (NES) | 1.2259353 |
| Nominal p-value | 0.21674877 |
| FDR q-value | 0.30441874 |
| FWER p-Value | 0.964 |
Table: GSEA Results Summary

  

Fig 1: Enrichment plot: HALLMARK\_MYOGENESIS      
 Profile of the Running ES Score & Positions of GeneSet Members on the Rank Ordered List

  

| SYMBOL | RANK IN GENE LIST | RANK METRIC SCORE | RUNNING ES | CORE ENRICHMENT || 1 | Cd36 | 85 | 2.334 | 0.0203 | Yes |
| 2 | Igfbp3 | 124 | 1.955 | 0.0455 | Yes |
| 3 | Cdkn1a | 192 | 1.625 | 0.0576 | Yes |
| 4 | Ckb | 267 | 1.414 | 0.0643 | Yes |
| 5 | Slc6a8 | 315 | 1.227 | 0.0743 | Yes |
| 6 | Sorbs1 | 325 | 1.205 | 0.0935 | Yes |
| 7 | Igf1 | 376 | 1.100 | 0.1005 | Yes |
| 8 | Col3a1 | 383 | 1.092 | 0.1184 | Yes |
| 9 | Col4a2 | 406 | 1.036 | 0.1313 | Yes |
| 10 | Smtn | 412 | 1.030 | 0.1483 | Yes |
| 11 | Cryab | 422 | 1.017 | 0.1640 | Yes |
| 12 | Col6a2 | 479 | 0.927 | 0.1666 | Yes |
| 13 | Col1a1 | 483 | 0.921 | 0.1821 | Yes |
| 14 | Gadd45b | 522 | 0.873 | 0.1882 | Yes |
| 15 | Fst | 589 | 0.795 | 0.1859 | Yes |
| 16 | Kifc3 | 645 | 0.717 | 0.1849 | Yes |
| 17 | Sphk1 | 672 | 0.691 | 0.1907 | Yes |
| 18 | Ifrd1 | 676 | 0.688 | 0.2022 | Yes |
| 19 | Wwtr1 | 705 | 0.663 | 0.2070 | Yes |
| 20 | Igfbp7 | 711 | 0.661 | 0.2174 | Yes |
| 21 | Sparc | 792 | 0.595 | 0.2081 | No |
| 22 | Ptp4a3 | 813 | 0.588 | 0.2136 | No |
| 23 | Prnp | 853 | 0.565 | 0.2139 | No |
| 24 | Rit1 | 921 | 0.518 | 0.2064 | No |
| 25 | Notch1 | 956 | 0.501 | 0.2069 | No |
| 26 | Mapre3 | 1099 | -0.522 | 0.1809 | No |
| 27 | Svil | 1206 | -0.542 | 0.1642 | No |
| 28 | Bag1 | 1219 | -0.543 | 0.1709 | No |
| 29 | Hbegf | 1222 | -0.544 | 0.1800 | No |
| 30 | Foxo4 | 1340 | -0.564 | 0.1610 | No |
| 31 | Erbb3 | 1362 | -0.568 | 0.1658 | No |
| 32 | Aplnr | 1378 | -0.570 | 0.1722 | No |
| 33 | Pick1 | 1384 | -0.571 | 0.1810 | No |
| 34 | Gaa | 1539 | -0.600 | 0.1535 | No |
| 35 | Eif4a2 | 1758 | -0.641 | 0.1108 | No |
| 36 | Pfkm | 1805 | -0.649 | 0.1108 | No |
| 37 | Rb1 | 1832 | -0.657 | 0.1160 | No |
| 38 | Sirt2 | 1889 | -0.671 | 0.1140 | No |
| 39 | Pde4dip | 2060 | -0.712 | 0.0845 | No |
| 40 | Ocel1 | 2221 | -0.744 | 0.0580 | No |
| 41 | Eno3 | 2415 | -0.794 | 0.0242 | No |
| 42 | Ptgis | 2464 | -0.808 | 0.0266 | No |
| 43 | Hdac5 | 2601 | -0.848 | 0.0078 | No |
| 44 | Acsl1 | 2609 | -0.850 | 0.0212 | No |
| 45 | Akt2 | 3033 | -0.987 | -0.0663 | No |
| 46 | Vipr1 | 3046 | -0.994 | -0.0517 | No |
| 47 | Reep1 | 3093 | -1.015 | -0.0451 | No |
| 48 | Sh2b1 | 3151 | -1.038 | -0.0409 | No |
| 49 | Mapk12 | 3165 | -1.045 | -0.0256 | No |
| 50 | Sorbs3 | 3256 | -1.096 | -0.0285 | No |
| 51 | Spdef | 3388 | -1.157 | -0.0405 | No |
| 52 | Clu | 3405 | -1.162 | -0.0239 | No |
| 53 | Hspb8 | 3763 | -1.451 | -0.0867 | No |
| 54 | Dtna | 3836 | -1.581 | -0.0766 | No |
| 55 | Klf5 | 3839 | -1.583 | -0.0491 | No |
| 56 | Ctf1 | 3934 | -1.776 | -0.0409 | No |
| 57 | Stc2 | 3990 | -2.075 | -0.0178 | No |
| 58 | Myl4 | 4041 | -2.379 | 0.0119 | No |
Table: GSEA details [plain text format]

  

Fig 2: HALLMARK\_MYOGENESIS: Random ES distribution      
 Gene set null distribution of ES for **HALLMARK\_MYOGENESIS**

  
