# Supplementary material for: TMPRSS11B promotes an acidified microenvironment and immune suppression in squamous lung cancer
Source: EMBO Rep. 2025 Nov 10;26(24):6346–79. doi: 10.1038/s44319-025-00631-1 (PMC12714794; doi:10.1038/s44319-025-00631-1)
Supplement: Supplementary file 8 — Source data Fig. 3 [file 44319_2025_631_MOESM8_ESM.zip › Figure 3/3D-E/GSEA_Broad Institute_Mh_T11b high vs low LUSC/HALLMARK_OXIDATIVE_PHOSPHORYLATION.html]

Details for gene set HALLMARK\_OXIDATIVE\_PHOSPHORYLATION[GSEA]

|  || Dataset | T11b high vs low squamous\_GSEA\_Ranked |
| Phenotype | NoPhenotypeAvailable |
| Upregulated in class | na\_neg |
| GeneSet | HALLMARK\_OXIDATIVE\_PHOSPHORYLATION |
| Enrichment Score (ES) | -0.15963352 |
| Normalized Enrichment Score (NES) | -0.8074717 |
| Nominal p-value | 0.738437 |
| FDR q-value | 0.8722413 |
| FWER p-Value | 1.0 |
Table: GSEA Results Summary

  

Fig 1: Enrichment plot: HALLMARK\_OXIDATIVE\_PHOSPHORYLATION      
 Profile of the Running ES Score & Positions of GeneSet Members on the Rank Ordered List

  

| SYMBOL | RANK IN GENE LIST | RANK METRIC SCORE | RUNNING ES | CORE ENRICHMENT || 1 | Tcirg1 | 541 | 0.853 | -0.1107 | No |
| 2 | Atp6v0e | 590 | 0.795 | -0.1011 | No |
| 3 | Atp6v1e1 | 641 | 0.720 | -0.0940 | No |
| 4 | Atp6v1g1 | 716 | 0.655 | -0.0946 | No |
| 5 | Atp6v0b | 884 | 0.550 | -0.1210 | No |
| 6 | Echs1 | 962 | -0.501 | -0.1266 | No |
| 7 | Ndufc2 | 997 | -0.504 | -0.1213 | No |
| 8 | Mtx2 | 1006 | -0.506 | -0.1096 | No |
| 9 | Ndufab1 | 1098 | -0.522 | -0.1180 | No |
| 10 | Opa1 | 1108 | -0.524 | -0.1061 | No |
| 11 | Vdac3 | 1133 | -0.528 | -0.0978 | No |
| 12 | Cox7a2l | 1218 | -0.543 | -0.1039 | No |
| 13 | Ndufv2 | 1275 | -0.552 | -0.1028 | No |
| 14 | Ndufs3 | 1349 | -0.565 | -0.1056 | No |
| 15 | Hsd17b10 | 1448 | -0.584 | -0.1140 | No |
| 16 | Decr1 | 1467 | -0.587 | -0.1026 | No |
| 17 | Hccs | 1477 | -0.589 | -0.0889 | No |
| 18 | Rhot1 | 1519 | -0.597 | -0.0829 | No |
| 19 | Etfb | 1565 | -0.605 | -0.0776 | No |
| 20 | Idh3b | 1568 | -0.606 | -0.0618 | No |
| 21 | Fh1 | 1687 | -0.627 | -0.0740 | No |
| 22 | Cox17 | 1863 | -0.664 | -0.0993 | No |
| 23 | Cox7c | 2108 | -0.712 | -0.1404 | Yes |
| 24 | Immt | 2168 | -0.733 | -0.1352 | Yes |
| 25 | Acaa2 | 2240 | -0.748 | -0.1325 | Yes |
| 26 | Uqcrc2 | 2273 | -0.755 | -0.1200 | Yes |
| 27 | Afg3l2 | 2280 | -0.757 | -0.1010 | Yes |
| 28 | Nqo2 | 2284 | -0.759 | -0.0812 | Yes |
| 29 | Idh3g | 2294 | -0.760 | -0.0629 | Yes |
| 30 | Bckdha | 2457 | -0.806 | -0.0811 | Yes |
| 31 | Acadm | 2482 | -0.814 | -0.0651 | Yes |
| 32 | Acadsb | 2492 | -0.818 | -0.0452 | Yes |
| 33 | Eci1 | 2554 | -0.834 | -0.0377 | Yes |
| 34 | Atp1b1 | 2559 | -0.835 | -0.0161 | Yes |
| 35 | Timm9 | 2596 | -0.846 | -0.0021 | Yes |
| 36 | Ech1 | 2819 | -0.911 | -0.0324 | Yes |
| 37 | Ndufs2 | 2874 | -0.932 | -0.0206 | Yes |
| 38 | Hadha | 2914 | -0.945 | -0.0047 | Yes |
| 39 | Hspa9 | 2925 | -0.947 | 0.0185 | Yes |
| 40 | Por | 2959 | -0.960 | 0.0363 | Yes |
| 41 | Cyb5a | 3121 | -1.027 | 0.0242 | Yes |
| 42 | Acat1 | 3248 | -1.092 | 0.0226 | Yes |
| 43 | Oxa1l | 3276 | -1.104 | 0.0458 | Yes |
| 44 | Aldh6a1 | 3480 | -1.202 | 0.0281 | Yes |
| 45 | Phyh | 3626 | -1.310 | 0.0277 | Yes |
| 46 | Idh2 | 3729 | -1.416 | 0.0407 | Yes |
| 47 | Oat | 3935 | -1.777 | 0.0381 | Yes |
Table: GSEA details [plain text format]

  

Fig 2: HALLMARK\_OXIDATIVE\_PHOSPHORYLATION: Random ES distribution      
 Gene set null distribution of ES for **HALLMARK\_OXIDATIVE\_PHOSPHORYLATION**

  
