# Supplementary material for: TMPRSS11B promotes an acidified microenvironment and immune suppression in squamous lung cancer
Source: EMBO Rep. 2025 Nov 10;26(24):6346–79. doi: 10.1038/s44319-025-00631-1 (PMC12714794; doi:10.1038/s44319-025-00631-1)
Supplement: Supplementary file 8 — Source data Fig. 3 [file 44319_2025_631_MOESM8_ESM.zip › Figure 3/3D-E/GSEA_Broad Institute_Mh_T11b high vs low LUSC/HALLMARK_P53_PATHWAY.html]

Details for gene set HALLMARK\_P53\_PATHWAY[GSEA]

|  || Dataset | T11b high vs low squamous\_GSEA\_Ranked |
| Phenotype | NoPhenotypeAvailable |
| Upregulated in class | na\_pos |
| GeneSet | HALLMARK\_P53\_PATHWAY |
| Enrichment Score (ES) | 0.34348148 |
| Normalized Enrichment Score (NES) | 2.0318408 |
| Nominal p-value | 0.0054054055 |
| FDR q-value | 0.005037428 |
| FWER p-Value | 0.033 |
Table: GSEA Results Summary

  

Fig 1: Enrichment plot: HALLMARK\_P53\_PATHWAY      
 Profile of the Running ES Score & Positions of GeneSet Members on the Rank Ordered List

  

| SYMBOL | RANK IN GENE LIST | RANK METRIC SCORE | RUNNING ES | CORE ENRICHMENT || 1 | Il1a | 44 | 2.812 | 0.0278 | Yes |
| 2 | Hmox1 | 55 | 2.684 | 0.0622 | Yes |
| 3 | S100a10 | 66 | 2.555 | 0.0949 | Yes |
| 4 | Mxd1 | 69 | 2.527 | 0.1293 | Yes |
| 5 | Slc7a11 | 126 | 1.940 | 0.1420 | Yes |
| 6 | Ctsd | 150 | 1.830 | 0.1615 | Yes |
| 7 | Cdkn1a | 192 | 1.625 | 0.1737 | Yes |
| 8 | Ada | 198 | 1.601 | 0.1945 | Yes |
| 9 | Krt17 | 201 | 1.595 | 0.2160 | Yes |
| 10 | Cdkn2b | 211 | 1.562 | 0.2352 | Yes |
| 11 | Clca2 | 246 | 1.451 | 0.2468 | Yes |
| 12 | Epha2 | 251 | 1.447 | 0.2657 | Yes |
| 13 | Ndrg1 | 276 | 1.380 | 0.2787 | Yes |
| 14 | Atf3 | 373 | 1.107 | 0.2701 | Yes |
| 15 | Dram1 | 382 | 1.092 | 0.2831 | Yes |
| 16 | Abcc5 | 400 | 1.047 | 0.2933 | Yes |
| 17 | Nupr1 | 443 | 0.988 | 0.2965 | Yes |
| 18 | Gadd45a | 473 | 0.944 | 0.3023 | Yes |
| 19 | Upp1 | 492 | 0.908 | 0.3103 | Yes |
| 20 | Ier5 | 493 | 0.908 | 0.3228 | Yes |
| 21 | Cdkn2a | 509 | 0.889 | 0.3313 | Yes |
| 22 | Ppp1r15a | 552 | 0.843 | 0.3325 | Yes |
| 23 | Rap2b | 629 | 0.730 | 0.3236 | Yes |
| 24 | Klf4 | 662 | 0.701 | 0.3253 | Yes |
| 25 | Sphk1 | 672 | 0.691 | 0.3326 | Yes |
| 26 | Apaf1 | 690 | 0.680 | 0.3377 | Yes |
| 27 | Def6 | 777 | 0.604 | 0.3247 | Yes |
| 28 | Gm2a | 815 | 0.585 | 0.3235 | Yes |
| 29 | Sat1 | 817 | 0.584 | 0.3313 | Yes |
| 30 | Stom | 820 | 0.581 | 0.3388 | Yes |
| 31 | Sfn | 834 | 0.573 | 0.3435 | Yes |
| 32 | Fam162a | 909 | 0.527 | 0.3323 | No |
| 33 | Tax1bp3 | 953 | 0.503 | 0.3285 | No |
| 34 | Notch1 | 956 | 0.501 | 0.3349 | No |
| 35 | Cgrrf1 | 1083 | -0.519 | 0.3107 | No |
| 36 | Hbegf | 1222 | -0.544 | 0.2839 | No |
| 37 | Prmt2 | 1232 | -0.545 | 0.2892 | No |
| 38 | Sesn1 | 1246 | -0.547 | 0.2935 | No |
| 39 | Hdac3 | 1291 | -0.554 | 0.2902 | No |
| 40 | Slc35d1 | 1376 | -0.570 | 0.2771 | No |
| 41 | Tm7sf3 | 1636 | -0.618 | 0.2212 | No |
| 42 | Tchh | 1693 | -0.628 | 0.2159 | No |
| 43 | Tsc22d1 | 1718 | -0.633 | 0.2186 | No |
| 44 | Rb1 | 1832 | -0.657 | 0.1996 | No |
| 45 | Abhd4 | 1960 | -0.686 | 0.1774 | No |
| 46 | Ccnd3 | 2040 | -0.700 | 0.1674 | No |
| 47 | Ip6k2 | 2137 | -0.726 | 0.1535 | No |
| 48 | Fas | 2311 | -0.764 | 0.1210 | No |
| 49 | Slc19a2 | 2571 | -0.840 | 0.0681 | No |
| 50 | Fbxw7 | 2582 | -0.843 | 0.0772 | No |
| 51 | Tob1 | 2615 | -0.853 | 0.0810 | No |
| 52 | Pmm1 | 2637 | -0.858 | 0.0876 | No |
| 53 | Pidd1 | 2639 | -0.858 | 0.0992 | No |
| 54 | F2r | 2690 | -0.872 | 0.0987 | No |
| 55 | Fdxr | 2703 | -0.876 | 0.1078 | No |
| 56 | Ptpn14 | 2757 | -0.892 | 0.1069 | No |
| 57 | Txnip | 2924 | -0.947 | 0.0787 | No |
| 58 | Nol8 | 2949 | -0.955 | 0.0858 | No |
| 59 | Cd82 | 2976 | -0.965 | 0.0927 | No |
| 60 | Ddb2 | 2996 | -0.975 | 0.1014 | No |
| 61 | Tspyl2 | 3021 | -0.982 | 0.1089 | No |
| 62 | Aen | 3036 | -0.989 | 0.1190 | No |
| 63 | Tap1 | 3068 | -1.004 | 0.1252 | No |
| 64 | Coq8a | 3108 | -1.021 | 0.1295 | No |
| 65 | Ctsf | 3141 | -1.034 | 0.1358 | No |
| 66 | Fos | 3189 | -1.058 | 0.1387 | No |
| 67 | Wwp1 | 3275 | -1.103 | 0.1327 | No |
| 68 | Kif13b | 3318 | -1.119 | 0.1377 | No |
| 69 | Tcn2 | 3391 | -1.158 | 0.1357 | No |
| 70 | Prkab1 | 3413 | -1.165 | 0.1465 | No |
| 71 | Dcxr | 3823 | -1.555 | 0.0662 | No |
Table: GSEA details [plain text format]

  

Fig 2: HALLMARK\_P53\_PATHWAY: Random ES distribution      
 Gene set null distribution of ES for **HALLMARK\_P53\_PATHWAY**

  
