# Supplementary material for: TMPRSS11B promotes an acidified microenvironment and immune suppression in squamous lung cancer
Source: EMBO Rep. 2025 Nov 10;26(24):6346–79. doi: 10.1038/s44319-025-00631-1 (PMC12714794; doi:10.1038/s44319-025-00631-1)
Supplement: Supplementary file 8 — Source data Fig. 3 [file 44319_2025_631_MOESM8_ESM.zip › Figure 3/3D-E/GSEA_Broad Institute_Mh_T11b high vs low LUSC/HALLMARK_PEROXISOME.html]

Details for gene set HALLMARK\_PEROXISOME[GSEA]

|  || Dataset | T11b high vs low squamous\_GSEA\_Ranked |
| Phenotype | NoPhenotypeAvailable |
| Upregulated in class | na\_neg |
| GeneSet | HALLMARK\_PEROXISOME |
| Enrichment Score (ES) | -0.22627303 |
| Normalized Enrichment Score (NES) | -0.91835684 |
| Nominal p-value | 0.5779221 |
| FDR q-value | 0.8407303 |
| FWER p-Value | 1.0 |
Table: GSEA Results Summary

  

Fig 1: Enrichment plot: HALLMARK\_PEROXISOME      
 Profile of the Running ES Score & Positions of GeneSet Members on the Rank Ordered List

  

| SYMBOL | RANK IN GENE LIST | RANK METRIC SCORE | RUNNING ES | CORE ENRICHMENT || 1 | Abcc5 | 400 | 1.047 | -0.0478 | No |
| 2 | Cln8 | 421 | 1.017 | -0.0035 | No |
| 3 | Sult2b1 | 497 | 0.901 | 0.0216 | No |
| 4 | Cat | 639 | 0.721 | 0.0218 | No |
| 5 | Acsl4 | 908 | 0.527 | -0.0187 | No |
| 6 | Cln6 | 966 | -0.501 | -0.0085 | No |
| 7 | Slc25a19 | 973 | -0.501 | 0.0143 | No |
| 8 | Hsd17b11 | 1184 | -0.536 | -0.0114 | No |
| 9 | Nudt19 | 1203 | -0.541 | 0.0103 | No |
| 10 | Smarcc1 | 1250 | -0.548 | 0.0255 | No |
| 11 | Hsd17b4 | 2245 | -0.749 | -0.1827 | No |
| 12 | Fads1 | 2281 | -0.757 | -0.1547 | No |
| 13 | Hsd3b7 | 2573 | -0.841 | -0.1856 | Yes |
| 14 | Acsl1 | 2609 | -0.850 | -0.1532 | Yes |
| 15 | Sod1 | 2691 | -0.874 | -0.1309 | Yes |
| 16 | Elovl5 | 2766 | -0.895 | -0.1058 | Yes |
| 17 | Ech1 | 2819 | -0.911 | -0.0746 | Yes |
| 18 | Pex13 | 2838 | -0.918 | -0.0346 | Yes |
| 19 | Pex11a | 3020 | -0.982 | -0.0317 | Yes |
| 20 | Aldh9a1 | 3166 | -1.046 | -0.0168 | Yes |
| 21 | Abcd3 | 3317 | -1.119 | 0.0005 | Yes |
| 22 | Idh2 | 3729 | -1.416 | -0.0322 | Yes |
| 23 | Aldh1a1 | 4051 | -2.492 | 0.0093 | Yes |
Table: GSEA details [plain text format]

  

Fig 2: HALLMARK\_PEROXISOME: Random ES distribution      
 Gene set null distribution of ES for **HALLMARK\_PEROXISOME**

  
