# Supplementary material for: TMPRSS11B promotes an acidified microenvironment and immune suppression in squamous lung cancer
Source: EMBO Rep. 2025 Nov 10;26(24):6346–79. doi: 10.1038/s44319-025-00631-1 (PMC12714794; doi:10.1038/s44319-025-00631-1)
Supplement: Supplementary file 8 — Source data Fig. 3 [file 44319_2025_631_MOESM8_ESM.zip › Figure 3/3D-E/GSEA_Broad Institute_Mh_T11b high vs low LUSC/HALLMARK_PI3K_AKT_MTOR_SIGNALING.html]

Details for gene set HALLMARK\_PI3K\_AKT\_MTOR\_SIGNALING[GSEA]

|  || Dataset | T11b high vs low squamous\_GSEA\_Ranked |
| Phenotype | NoPhenotypeAvailable |
| Upregulated in class | na\_pos |
| GeneSet | HALLMARK\_PI3K\_AKT\_MTOR\_SIGNALING |
| Enrichment Score (ES) | 0.28762805 |
| Normalized Enrichment Score (NES) | 1.2375903 |
| Nominal p-value | 0.21287128 |
| FDR q-value | 0.30470896 |
| FWER p-Value | 0.956 |
Table: GSEA Results Summary

  

Fig 1: Enrichment plot: HALLMARK\_PI3K\_AKT\_MTOR\_SIGNALING      
 Profile of the Running ES Score & Positions of GeneSet Members on the Rank Ordered List

  

| SYMBOL | RANK IN GENE LIST | RANK METRIC SCORE | RUNNING ES | CORE ENRICHMENT || 1 | Cdkn1a | 192 | 1.625 | 0.0369 | Yes |
| 2 | Sla | 207 | 1.573 | 0.1150 | Yes |
| 3 | Map2k3 | 282 | 1.367 | 0.1676 | Yes |
| 4 | Cxcr4 | 289 | 1.347 | 0.2359 | Yes |
| 5 | Dusp3 | 634 | 0.725 | 0.1888 | Yes |
| 6 | Cdk1 | 724 | 0.651 | 0.2007 | Yes |
| 7 | Sfn | 834 | 0.573 | 0.2036 | Yes |
| 8 | Mknk1 | 881 | 0.551 | 0.2208 | Yes |
| 9 | Cfl1 | 895 | 0.538 | 0.2454 | Yes |
| 10 | Rit1 | 921 | 0.518 | 0.2661 | Yes |
| 11 | Raf1 | 942 | 0.510 | 0.2876 | Yes |
| 12 | Prkag1 | 1326 | -0.563 | 0.2226 | No |
| 13 | Mapkap1 | 1532 | -0.600 | 0.2033 | No |
| 14 | Actr2 | 1729 | -0.634 | 0.1879 | No |
| 15 | Vav3 | 2084 | -0.712 | 0.1378 | No |
| 16 | Gna14 | 2096 | -0.712 | 0.1720 | No |
| 17 | Prkaa2 | 2146 | -0.727 | 0.1976 | No |
| 18 | Itpr2 | 2598 | -0.848 | 0.1306 | No |
| 19 | Ecsit | 2647 | -0.860 | 0.1634 | No |
| 20 | Traf2 | 3325 | -1.127 | 0.0553 | No |
| 21 | Nod1 | 3491 | -1.205 | 0.0772 | No |
| 22 | Cab39l | 3659 | -1.344 | 0.1057 | No |
Table: GSEA details [plain text format]

  

Fig 2: HALLMARK\_PI3K\_AKT\_MTOR\_SIGNALING: Random ES distribution      
 Gene set null distribution of ES for **HALLMARK\_PI3K\_AKT\_MTOR\_SIGNALING**

  
