# Supplementary material for: TMPRSS11B promotes an acidified microenvironment and immune suppression in squamous lung cancer
Source: EMBO Rep. 2025 Nov 10;26(24):6346–79. doi: 10.1038/s44319-025-00631-1 (PMC12714794; doi:10.1038/s44319-025-00631-1)
Supplement: Supplementary file 8 — Source data Fig. 3 [file 44319_2025_631_MOESM8_ESM.zip › Figure 3/3D-E/GSEA_Broad Institute_Mh_T11b high vs low LUSC/HALLMARK_PROTEIN_SECRETION.html]

Details for gene set HALLMARK\_PROTEIN\_SECRETION[GSEA]

|  || Dataset | T11b high vs low squamous\_GSEA\_Ranked |
| Phenotype | NoPhenotypeAvailable |
| Upregulated in class | na\_neg |
| GeneSet | HALLMARK\_PROTEIN\_SECRETION |
| Enrichment Score (ES) | -0.2158746 |
| Normalized Enrichment Score (NES) | -0.92780745 |
| Nominal p-value | 0.5517799 |
| FDR q-value | 0.9240649 |
| FWER p-Value | 1.0 |
Table: GSEA Results Summary

  

Fig 1: Enrichment plot: HALLMARK\_PROTEIN\_SECRETION      
 Profile of the Running ES Score & Positions of GeneSet Members on the Rank Ordered List

  

| SYMBOL | RANK IN GENE LIST | RANK METRIC SCORE | RUNNING ES | CORE ENRICHMENT || 1 | Abca1 | 172 | 1.716 | 0.0212 | No |
| 2 | Gla | 228 | 1.504 | 0.0634 | No |
| 3 | Bnip3 | 365 | 1.118 | 0.0713 | No |
| 4 | Cd63 | 632 | 0.727 | 0.0328 | No |
| 5 | Kif1b | 923 | 0.518 | -0.0194 | No |
| 6 | Mon2 | 1103 | -0.523 | -0.0441 | No |
| 7 | Cav2 | 1299 | -0.555 | -0.0716 | No |
| 8 | Cog2 | 1454 | -0.585 | -0.0878 | No |
| 9 | Rab5a | 1588 | -0.609 | -0.0980 | No |
| 10 | Gbf1 | 1737 | -0.636 | -0.1109 | No |
| 11 | Vamp4 | 1894 | -0.672 | -0.1244 | No |
| 12 | Stam | 1945 | -0.683 | -0.1114 | No |
| 13 | Arfgef1 | 2351 | -0.774 | -0.1824 | No |
| 14 | Golga4 | 2488 | -0.817 | -0.1856 | Yes |
| 15 | Snx2 | 2507 | -0.820 | -0.1597 | Yes |
| 16 | Copb1 | 2636 | -0.857 | -0.1594 | Yes |
| 17 | Sod1 | 2691 | -0.874 | -0.1404 | Yes |
| 18 | Lamp2 | 2707 | -0.877 | -0.1116 | Yes |
| 19 | Ocrl | 2745 | -0.888 | -0.0878 | Yes |
| 20 | Ap3s1 | 2863 | -0.930 | -0.0822 | Yes |
| 21 | Sgms1 | 3032 | -0.985 | -0.0870 | Yes |
| 22 | Arfgap3 | 3188 | -1.056 | -0.0861 | Yes |
| 23 | Tom1l1 | 3232 | -1.084 | -0.0565 | Yes |
| 24 | Dop1a | 3273 | -1.103 | -0.0255 | Yes |
| 25 | Gosr2 | 3316 | -1.118 | 0.0056 | Yes |
| 26 | Ica1 | 3840 | -1.586 | -0.0644 | Yes |
| 27 | Sh3gl2 | 3857 | -1.626 | -0.0081 | Yes |
| 28 | Pam | 3917 | -1.755 | 0.0423 | Yes |
Table: GSEA details [plain text format]

  

Fig 2: HALLMARK\_PROTEIN\_SECRETION: Random ES distribution      
 Gene set null distribution of ES for **HALLMARK\_PROTEIN\_SECRETION**

  
