# Supplementary material for: TMPRSS11B promotes an acidified microenvironment and immune suppression in squamous lung cancer
Source: EMBO Rep. 2025 Nov 10;26(24):6346–79. doi: 10.1038/s44319-025-00631-1 (PMC12714794; doi:10.1038/s44319-025-00631-1)
Supplement: Supplementary file 8 — Source data Fig. 3 [file 44319_2025_631_MOESM8_ESM.zip › Figure 3/3D-E/GSEA_Broad Institute_Mh_T11b high vs low LUSC/HALLMARK_SPERMATOGENESIS.html]

Details for gene set HALLMARK\_SPERMATOGENESIS[GSEA]

|  || Dataset | T11b high vs low squamous\_GSEA\_Ranked |
| Phenotype | NoPhenotypeAvailable |
| Upregulated in class | na\_neg |
| GeneSet | HALLMARK\_SPERMATOGENESIS |
| Enrichment Score (ES) | -0.40079254 |
| Normalized Enrichment Score (NES) | -1.5331686 |
| Nominal p-value | 0.053264603 |
| FDR q-value | 0.26614267 |
| FWER p-Value | 0.652 |
Table: GSEA Results Summary

  

Fig 1: Enrichment plot: HALLMARK\_SPERMATOGENESIS      
 Profile of the Running ES Score & Positions of GeneSet Members on the Rank Ordered List

  

| SYMBOL | RANK IN GENE LIST | RANK METRIC SCORE | RUNNING ES | CORE ENRICHMENT || 1 | Alox15 | 533 | 0.865 | -0.0874 | No |
| 2 | Ace | 681 | 0.685 | -0.0891 | No |
| 3 | Cdk1 | 724 | 0.651 | -0.0667 | No |
| 4 | Mllt10 | 1078 | -0.518 | -0.1273 | No |
| 5 | Vdac3 | 1133 | -0.528 | -0.1141 | No |
| 6 | Tsn | 1281 | -0.553 | -0.1224 | No |
| 7 | Strbp | 1723 | -0.634 | -0.1988 | No |
| 8 | Nphp1 | 1891 | -0.671 | -0.2061 | No |
| 9 | Spata6 | 2214 | -0.742 | -0.2479 | No |
| 10 | Nek2 | 2485 | -0.816 | -0.2732 | No |
| 11 | Mast2 | 2505 | -0.819 | -0.2366 | No |
| 12 | Pias2 | 2526 | -0.827 | -0.2000 | No |
| 13 | Phf7 | 2552 | -0.833 | -0.1642 | No |
| 14 | Zc3h14 | 2693 | -0.874 | -0.1547 | No |
| 15 | Hspa2 | 3696 | -1.378 | -0.3315 | Yes |
| 16 | Ift88 | 3740 | -1.428 | -0.2703 | Yes |
| 17 | Cftr | 3743 | -1.432 | -0.1987 | Yes |
| 18 | Slc12a2 | 4021 | -2.250 | -0.1536 | Yes |
| 19 | Mlf1 | 4085 | -3.382 | 0.0010 | Yes |
Table: GSEA details [plain text format]

  

Fig 2: HALLMARK\_SPERMATOGENESIS: Random ES distribution      
 Gene set null distribution of ES for **HALLMARK\_SPERMATOGENESIS**

  
