# Supplementary material for: TMPRSS11B promotes an acidified microenvironment and immune suppression in squamous lung cancer
Source: EMBO Rep. 2025 Nov 10;26(24):6346–79. doi: 10.1038/s44319-025-00631-1 (PMC12714794; doi:10.1038/s44319-025-00631-1)
Supplement: Supplementary file 8 — Source data Fig. 3 [file 44319_2025_631_MOESM8_ESM.zip › Figure 3/3D-E/GSEA_Broad Institute_Mh_T11b high vs low LUSC/HALLMARK_TGF_BETA_SIGNALING.html]

Details for gene set HALLMARK\_TGF\_BETA\_SIGNALING[GSEA]

|  || Dataset | T11b high vs low squamous\_GSEA\_Ranked |
| Phenotype | NoPhenotypeAvailable |
| Upregulated in class | na\_pos |
| GeneSet | HALLMARK\_TGF\_BETA\_SIGNALING |
| Enrichment Score (ES) | 0.32471457 |
| Normalized Enrichment Score (NES) | 1.2878417 |
| Nominal p-value | 0.16136919 |
| FDR q-value | 0.25372356 |
| FWER p-Value | 0.932 |
Table: GSEA Results Summary

  

Fig 1: Enrichment plot: HALLMARK\_TGF\_BETA\_SIGNALING      
 Profile of the Running ES Score & Positions of GeneSet Members on the Rank Ordered List

  

| SYMBOL | RANK IN GENE LIST | RANK METRIC SCORE | RUNNING ES | CORE ENRICHMENT || 1 | Serpine1 | 89 | 2.295 | 0.1328 | Yes |
| 2 | Rab31 | 156 | 1.775 | 0.2362 | Yes |
| 3 | Ppp1r15a | 552 | 0.843 | 0.1960 | Yes |
| 4 | Wwtr1 | 705 | 0.663 | 0.2034 | Yes |
| 5 | Hipk2 | 725 | 0.651 | 0.2426 | Yes |
| 6 | Ski | 727 | 0.646 | 0.2858 | Yes |
| 7 | Eng | 867 | 0.560 | 0.2894 | Yes |
| 8 | Smurf1 | 905 | 0.529 | 0.3160 | Yes |
| 9 | Smad6 | 1086 | -0.519 | 0.3068 | Yes |
| 10 | Fnta | 1273 | -0.551 | 0.2982 | Yes |
| 11 | Smad3 | 1449 | -0.584 | 0.2946 | Yes |
| 12 | Hdac1 | 1490 | -0.592 | 0.3247 | Yes |
| 13 | Tgif1 | 1899 | -0.673 | 0.2699 | No |
| 14 | Smurf2 | 2298 | -0.761 | 0.2235 | No |
| 15 | Cdkn1c | 2722 | -0.882 | 0.1791 | No |
| 16 | Cdh1 | 2989 | -0.975 | 0.1794 | No |
| 17 | Bmpr1a | 3653 | -1.341 | 0.1070 | No |
Table: GSEA details [plain text format]

  

Fig 2: HALLMARK\_TGF\_BETA\_SIGNALING: Random ES distribution      
 Gene set null distribution of ES for **HALLMARK\_TGF\_BETA\_SIGNALING**

  
