# Supplementary material for: TMPRSS11B promotes an acidified microenvironment and immune suppression in squamous lung cancer
Source: EMBO Rep. 2025 Nov 10;26(24):6346–79. doi: 10.1038/s44319-025-00631-1 (PMC12714794; doi:10.1038/s44319-025-00631-1)
Supplement: Supplementary file 8 — Source data Fig. 3 [file 44319_2025_631_MOESM8_ESM.zip › Figure 3/3D-E/GSEA_Broad Institute_Mh_T11b high vs low LUSC/HALLMARK_TNFA_SIGNALING_VIA_NFKB.html]

Details for gene set HALLMARK\_TNFA\_SIGNALING\_VIA\_NFKB[GSEA]

|  || Dataset | T11b high vs low squamous\_GSEA\_Ranked |
| Phenotype | NoPhenotypeAvailable |
| Upregulated in class | na\_pos |
| GeneSet | HALLMARK\_TNFA\_SIGNALING\_VIA\_NFKB |
| Enrichment Score (ES) | 0.42424306 |
| Normalized Enrichment Score (NES) | 2.5252647 |
| Nominal p-value | 0.0 |
| FDR q-value | 0.0 |
| FWER p-Value | 0.0 |
Table: GSEA Results Summary

  

Fig 1: Enrichment plot: HALLMARK\_TNFA\_SIGNALING\_VIA\_NFKB      
 Profile of the Running ES Score & Positions of GeneSet Members on the Rank Ordered List

  

| SYMBOL | RANK IN GENE LIST | RANK METRIC SCORE | RUNNING ES | CORE ENRICHMENT || 1 | Cxcl5 | 16 | 3.974 | 0.0440 | Yes |
| 2 | Il1a | 44 | 2.812 | 0.0712 | Yes |
| 3 | Mxd1 | 69 | 2.527 | 0.0958 | Yes |
| 4 | Tnc | 84 | 2.358 | 0.1208 | Yes |
| 5 | Serpine1 | 89 | 2.295 | 0.1475 | Yes |
| 6 | Plek | 95 | 2.237 | 0.1733 | Yes |
| 7 | Il1b | 129 | 1.912 | 0.1881 | Yes |
| 8 | Plau | 136 | 1.895 | 0.2095 | Yes |
| 9 | Abca1 | 172 | 1.716 | 0.2215 | Yes |
| 10 | Cdkn1a | 192 | 1.625 | 0.2364 | Yes |
| 11 | Dusp4 | 216 | 1.536 | 0.2493 | Yes |
| 12 | Dusp1 | 239 | 1.468 | 0.2615 | Yes |
| 13 | Map2k3 | 282 | 1.367 | 0.2675 | Yes |
| 14 | Ehd1 | 313 | 1.237 | 0.2750 | Yes |
| 15 | Irf1 | 372 | 1.111 | 0.2740 | Yes |
| 16 | Atf3 | 373 | 1.107 | 0.2873 | Yes |
| 17 | Dram1 | 382 | 1.092 | 0.2985 | Yes |
| 18 | Tnfaip2 | 387 | 1.086 | 0.3106 | Yes |
| 19 | Zc3h12a | 419 | 1.020 | 0.3152 | Yes |
| 20 | Plaur | 426 | 1.012 | 0.3260 | Yes |
| 21 | Gadd45a | 473 | 0.944 | 0.3259 | Yes |
| 22 | Nfkb1 | 486 | 0.918 | 0.3340 | Yes |
| 23 | Plpp3 | 490 | 0.910 | 0.3443 | Yes |
| 24 | Ier5 | 493 | 0.908 | 0.3547 | Yes |
| 25 | Tnf | 495 | 0.907 | 0.3654 | Yes |
| 26 | Cebpb | 504 | 0.897 | 0.3743 | Yes |
| 27 | Gadd45b | 522 | 0.873 | 0.3806 | Yes |
| 28 | Serpinb2 | 527 | 0.873 | 0.3901 | Yes |
| 29 | Ppp1r15a | 552 | 0.843 | 0.3943 | Yes |
| 30 | Cd44 | 562 | 0.834 | 0.4021 | Yes |
| 31 | Birc3 | 607 | 0.758 | 0.4003 | Yes |
| 32 | Tubb2a | 652 | 0.711 | 0.3979 | Yes |
| 33 | Klf4 | 662 | 0.701 | 0.4042 | Yes |
| 34 | Sphk1 | 672 | 0.691 | 0.4103 | Yes |
| 35 | Cflar | 686 | 0.680 | 0.4153 | Yes |
| 36 | Maff | 776 | 0.605 | 0.4004 | Yes |
| 37 | B4galt5 | 778 | 0.602 | 0.4074 | Yes |
| 38 | Sat1 | 817 | 0.584 | 0.4050 | Yes |
| 39 | Dennd5a | 827 | 0.576 | 0.4097 | Yes |
| 40 | Litaf | 856 | 0.564 | 0.4095 | Yes |
| 41 | Dusp5 | 861 | 0.563 | 0.4153 | Yes |
| 42 | Ackr3 | 918 | 0.524 | 0.4077 | Yes |
| 43 | Tank | 933 | 0.514 | 0.4104 | Yes |
| 44 | Rela | 943 | 0.509 | 0.4143 | Yes |
| 45 | Mcl1 | 950 | 0.505 | 0.4189 | Yes |
| 46 | Nfkbia | 954 | 0.502 | 0.4242 | Yes |
| 47 | Il6st | 1089 | -0.520 | 0.3971 | No |
| 48 | Hbegf | 1222 | -0.544 | 0.3708 | No |
| 49 | Klf9 | 1352 | -0.566 | 0.3455 | No |
| 50 | Smad3 | 1449 | -0.584 | 0.3286 | No |
| 51 | Nfat5 | 1506 | -0.597 | 0.3218 | No |
| 52 | Stat5a | 1551 | -0.602 | 0.3182 | No |
| 53 | Tsc22d1 | 1718 | -0.633 | 0.2844 | No |
| 54 | Fut4 | 1801 | -0.649 | 0.2718 | No |
| 55 | Ptger4 | 1845 | -0.660 | 0.2691 | No |
| 56 | Tgif1 | 1899 | -0.673 | 0.2640 | No |
| 57 | Irs2 | 1906 | -0.674 | 0.2706 | No |
| 58 | Fjx1 | 1964 | -0.687 | 0.2647 | No |
| 59 | Cebpd | 2002 | -0.693 | 0.2639 | No |
| 60 | Sgk1 | 2539 | -0.829 | 0.1403 | No |
| 61 | Trip10 | 2549 | -0.832 | 0.1481 | No |
| 62 | Relb | 2560 | -0.835 | 0.1557 | No |
| 63 | Areg | 2629 | -0.856 | 0.1491 | No |
| 64 | Birc2 | 2641 | -0.859 | 0.1567 | No |
| 65 | Jag1 | 2743 | -0.887 | 0.1422 | No |
| 66 | Per1 | 2753 | -0.890 | 0.1508 | No |
| 67 | Nr4a2 | 2921 | -0.946 | 0.1206 | No |
| 68 | Tap1 | 3068 | -1.004 | 0.0963 | No |
| 69 | Il18 | 3078 | -1.010 | 0.1063 | No |
| 70 | Fos | 3189 | -1.058 | 0.0916 | No |
| 71 | Ccnd1 | 3303 | -1.112 | 0.0769 | No |
| 72 | Tlr2 | 3387 | -1.156 | 0.0701 | No |
| 73 | Clcf1 | 3433 | -1.178 | 0.0732 | No |
| 74 | Rel | 3500 | -1.210 | 0.0713 | No |
| 75 | Egr1 | 3532 | -1.234 | 0.0785 | No |
| 76 | Dnajb4 | 3750 | -1.440 | 0.0418 | No |
| 77 | Tnfaip8 | 3837 | -1.581 | 0.0394 | No |
| 78 | F2rl1 | 3967 | -1.914 | 0.0304 | No |
Table: GSEA details [plain text format]

  

Fig 2: HALLMARK\_TNFA\_SIGNALING\_VIA\_NFKB: Random ES distribution      
 Gene set null distribution of ES for **HALLMARK\_TNFA\_SIGNALING\_VIA\_NFKB**

  
