# Supplementary material for: TMPRSS11B promotes an acidified microenvironment and immune suppression in squamous lung cancer
Source: EMBO Rep. 2025 Nov 10;26(24):6346–79. doi: 10.1038/s44319-025-00631-1 (PMC12714794; doi:10.1038/s44319-025-00631-1)
Supplement: Supplementary file 8 — Source data Fig. 3 [file 44319_2025_631_MOESM8_ESM.zip › Figure 3/3D-E/GSEA_Broad Institute_Mh_T11b high vs low LUSC/HALLMARK_UNFOLDED_PROTEIN_RESPONSE.html]

Details for gene set HALLMARK\_UNFOLDED\_PROTEIN\_RESPONSE[GSEA]

|  || Dataset | T11b high vs low squamous\_GSEA\_Ranked |
| Phenotype | NoPhenotypeAvailable |
| Upregulated in class | na\_neg |
| GeneSet | HALLMARK\_UNFOLDED\_PROTEIN\_RESPONSE |
| Enrichment Score (ES) | -0.30106622 |
| Normalized Enrichment Score (NES) | -1.3679417 |
| Nominal p-value | 0.1118421 |
| FDR q-value | 0.45520362 |
| FWER p-Value | 0.949 |
Table: GSEA Results Summary

  

Fig 1: Enrichment plot: HALLMARK\_UNFOLDED\_PROTEIN\_RESPONSE      
 Profile of the Running ES Score & Positions of GeneSet Members on the Rank Ordered List

  

| SYMBOL | RANK IN GENE LIST | RANK METRIC SCORE | RUNNING ES | CORE ENRICHMENT || 1 | Wfs1 | 310 | 1.262 | -0.0374 | No |
| 2 | Atf3 | 373 | 1.107 | -0.0184 | No |
| 3 | Cebpb | 504 | 0.897 | -0.0227 | No |
| 4 | Tubb2a | 652 | 0.711 | -0.0369 | No |
| 5 | Ssr1 | 710 | 0.661 | -0.0306 | No |
| 6 | Psat1 | 766 | 0.613 | -0.0251 | No |
| 7 | Eif2ak3 | 781 | 0.601 | -0.0100 | No |
| 8 | Wipi1 | 941 | 0.510 | -0.0334 | No |
| 9 | Exoc2 | 1289 | -0.554 | -0.1018 | No |
| 10 | Edc4 | 1533 | -0.600 | -0.1432 | No |
| 11 | Cnot6 | 1682 | -0.627 | -0.1603 | No |
| 12 | Eif4a2 | 1758 | -0.641 | -0.1590 | No |
| 13 | Parn | 1811 | -0.651 | -0.1517 | No |
| 14 | Sdad1 | 2025 | -0.697 | -0.1826 | No |
| 15 | Pop4 | 2295 | -0.760 | -0.2254 | No |
| 16 | Mtrex | 2367 | -0.779 | -0.2188 | No |
| 17 | Nfyb | 2441 | -0.801 | -0.2120 | No |
| 18 | Xbp1 | 2672 | -0.867 | -0.2419 | No |
| 19 | Paip1 | 2913 | -0.944 | -0.2718 | Yes |
| 20 | Hspa9 | 2925 | -0.947 | -0.2452 | Yes |
| 21 | Dcp2 | 2964 | -0.961 | -0.2249 | Yes |
| 22 | Bag3 | 3005 | -0.975 | -0.2046 | Yes |
| 23 | Tspyl2 | 3021 | -0.982 | -0.1779 | Yes |
| 24 | Dnajc3 | 3125 | -1.028 | -0.1715 | Yes |
| 25 | Kif5b | 3281 | -1.107 | -0.1754 | Yes |
| 26 | Dnaja4 | 3313 | -1.117 | -0.1485 | Yes |
| 27 | Gosr2 | 3316 | -1.118 | -0.1144 | Yes |
| 28 | Hyou1 | 3322 | -1.127 | -0.0808 | Yes |
| 29 | Mthfd2 | 3458 | -1.191 | -0.0772 | Yes |
| 30 | Pdia5 | 3468 | -1.197 | -0.0424 | Yes |
| 31 | Exosc4 | 3545 | -1.243 | -0.0226 | Yes |
| 32 | Exosc10 | 3563 | -1.256 | 0.0120 | Yes |
| 33 | Kdelr3 | 3903 | -1.712 | -0.0186 | Yes |
| 34 | Stc2 | 3990 | -2.075 | 0.0244 | Yes |
Table: GSEA details [plain text format]

  

Fig 2: HALLMARK\_UNFOLDED\_PROTEIN\_RESPONSE: Random ES distribution      
 Gene set null distribution of ES for **HALLMARK\_UNFOLDED\_PROTEIN\_RESPONSE**

  
