# Supplementary material for: TMPRSS11B promotes an acidified microenvironment and immune suppression in squamous lung cancer
Source: EMBO Rep. 2025 Nov 10;26(24):6346–79. doi: 10.1038/s44319-025-00631-1 (PMC12714794; doi:10.1038/s44319-025-00631-1)
Supplement: Supplementary file 8 — Source data Fig. 3 [file 44319_2025_631_MOESM8_ESM.zip › Figure 3/3D-E/GSEA_Broad Institute_Mh_T11b high vs low LUSC/HALLMARK_UV_RESPONSE_UP.html]

Details for gene set HALLMARK\_UV\_RESPONSE\_UP[GSEA]

|  || Dataset | T11b high vs low squamous\_GSEA\_Ranked |
| Phenotype | NoPhenotypeAvailable |
| Upregulated in class | na\_pos |
| GeneSet | HALLMARK\_UV\_RESPONSE\_UP |
| Enrichment Score (ES) | 0.27937704 |
| Normalized Enrichment Score (NES) | 1.3729025 |
| Nominal p-value | 0.1171875 |
| FDR q-value | 0.18467084 |
| FWER p-Value | 0.845 |
Table: GSEA Results Summary

  

Fig 1: Enrichment plot: HALLMARK\_UV\_RESPONSE\_UP      
 Profile of the Running ES Score & Positions of GeneSet Members on the Rank Ordered List

  

| SYMBOL | RANK IN GENE LIST | RANK METRIC SCORE | RUNNING ES | CORE ENRICHMENT || 1 | Ctsl | 15 | 3.988 | 0.1093 | Yes |
| 2 | Hmox1 | 55 | 2.684 | 0.1758 | Yes |
| 3 | Cdkn2b | 211 | 1.562 | 0.1818 | Yes |
| 4 | Creg1 | 280 | 1.373 | 0.2039 | Yes |
| 5 | Slc6a8 | 315 | 1.227 | 0.2303 | Yes |
| 6 | Irf1 | 372 | 1.111 | 0.2480 | Yes |
| 7 | Atf3 | 373 | 1.107 | 0.2794 | Yes |
| 8 | Dnajb1 | 534 | 0.862 | 0.2644 | No |
| 9 | Tfrc | 668 | 0.696 | 0.2513 | No |
| 10 | Arrb2 | 786 | 0.600 | 0.2394 | No |
| 11 | Maoa | 889 | 0.544 | 0.2297 | No |
| 12 | Nfkbia | 954 | 0.502 | 0.2281 | No |
| 13 | Rpn1 | 1015 | -0.507 | 0.2277 | No |
| 14 | Il6st | 1089 | -0.520 | 0.2245 | No |
| 15 | Urod | 1150 | -0.530 | 0.2247 | No |
| 16 | Tst | 1483 | -0.590 | 0.1595 | No |
| 17 | Tmbim6 | 1599 | -0.611 | 0.1485 | No |
| 18 | Tchh | 1693 | -0.628 | 0.1433 | No |
| 19 | Stard3 | 1751 | -0.640 | 0.1474 | No |
| 20 | Clcn2 | 1999 | -0.693 | 0.1061 | No |
| 21 | Ccnd3 | 2040 | -0.700 | 0.1161 | No |
| 22 | Spr | 2241 | -0.749 | 0.0880 | No |
| 23 | Ppif | 2248 | -0.750 | 0.1078 | No |
| 24 | Cnp | 2305 | -0.763 | 0.1156 | No |
| 25 | Stk25 | 2388 | -0.787 | 0.1177 | No |
| 26 | Bsg | 2530 | -0.827 | 0.1064 | No |
| 27 | Klhdc3 | 2590 | -0.845 | 0.1158 | No |
| 28 | Cdkn1c | 2722 | -0.882 | 0.1084 | No |
| 29 | E2f5 | 2910 | -0.943 | 0.0891 | No |
| 30 | Ppat | 3052 | -0.994 | 0.0825 | No |
| 31 | Tap1 | 3068 | -1.004 | 0.1072 | No |
| 32 | Fos | 3189 | -1.058 | 0.1076 | No |
| 33 | Sult1a1 | 3309 | -1.116 | 0.1099 | No |
| 34 | Hspa2 | 3696 | -1.378 | 0.0537 | No |
| 35 | Chka | 3801 | -1.515 | 0.0710 | No |
Table: GSEA details [plain text format]

  

Fig 2: HALLMARK\_UV\_RESPONSE\_UP: Random ES distribution      
 Gene set null distribution of ES for **HALLMARK\_UV\_RESPONSE\_UP**

  
