# Supplementary material for: TMPRSS11B promotes an acidified microenvironment and immune suppression in squamous lung cancer
Source: EMBO Rep. 2025 Nov 10;26(24):6346–79. doi: 10.1038/s44319-025-00631-1 (PMC12714794; doi:10.1038/s44319-025-00631-1)
Supplement: Supplementary file 9 — Source data Fig. 4 [file 44319_2025_631_MOESM9_ESM.zip › Figure 4/4C/Read Me.rtfd/TXT.rtf]

Lung Adenocarcinoma (TCGA, GDC)TCGA Lung Adenocarcinoma. Source data from GDC and generated in Jul 2024 using ISB-CGC BigQuery tables.Lung Squamous Cell Carcinoma (TCGA, GDC)TCGA Lung Squamous Cell Carcinoma. Source data from GDC and generated in Jul 2024 using ISB-CGC BigQuery tables.The FPKM mRNA values from the the two datasets shown above from cBioPortal were used for the analysis.Read Me.rtf ¬
