# Supplementary material for: TMPRSS11B promotes an acidified microenvironment and immune suppression in squamous lung cancer
Source: EMBO Rep. 2025 Nov 10;26(24):6346–79. doi: 10.1038/s44319-025-00631-1 (PMC12714794; doi:10.1038/s44319-025-00631-1)
Supplement: Supplementary file 9 — Source data Fig. 4 [file 44319_2025_631_MOESM9_ESM.zip › Figure 4/4D/Read Me.rtfd/TXT.rtf]

BEAS 2B cells with inducible expression of KLF4 was used for the experiment. T11B_II and T11B_IV are two different Taqman probes used for the qPCR. Read Me.rtf ¬
