# Supplementary material for: TMPRSS11B promotes an acidified microenvironment and immune suppression in squamous lung cancer
Source: EMBO Rep. 2025 Nov 10;26(24):6346–79. doi: 10.1038/s44319-025-00631-1 (PMC12714794; doi:10.1038/s44319-025-00631-1)
Supplement: Supplementary file 10 — Source data Fig. 5 [file 44319_2025_631_MOESM10_ESM.zip › Figure 5/5C-D/Read Me.rtf]

The “DGE_T11b-high LUSC vs LUAD.xls” file represents the differential gene expression analysis (DEG) results. The .rnk file used for the pre-ranked gene set enrichment analysis (GSEA) has been generated using the average log2FC as the ranking metric.
