# Supplementary material for: TMPRSS11B promotes an acidified microenvironment and immune suppression in squamous lung cancer
Source: EMBO Rep. 2025 Nov 10;26(24):6346–79. doi: 10.1038/s44319-025-00631-1 (PMC12714794; doi:10.1038/s44319-025-00631-1)
Supplement: Supplementary file 10 — Source data Fig. 5 [file 44319_2025_631_MOESM10_ESM.zip › Figure 5/5C-D/GSEA_Broad Institute_M8_T11b-high LUSC vs LUAD/DESCARTES_ORGANOGENESIS_CARDIAC_MUSCLE_LINEAGES.html]

Details for gene set DESCARTES\_ORGANOGENESIS\_CARDIAC\_MUSCLE\_LINEAGES[GSEA]

|  || Dataset | Ranked list\_DGE\_squamousT11b\_vs\_all adenosadeno\_HSE13-NT copy |
| Phenotype | NoPhenotypeAvailable |
| Upregulated in class | na\_neg |
| GeneSet | DESCARTES\_ORGANOGENESIS\_CARDIAC\_MUSCLE\_LINEAGES |
| Enrichment Score (ES) | -0.21646962 |
| Normalized Enrichment Score (NES) | -0.89361095 |
| Nominal p-value | 0.61981565 |
| FDR q-value | 1.0 |
| FWER p-Value | 1.0 |
Table: GSEA Results Summary

  

Fig 1: Enrichment plot: DESCARTES\_ORGANOGENESIS\_CARDIAC\_MUSCLE\_LINEAGES      
 Profile of the Running ES Score & Positions of GeneSet Members on the Rank Ordered List

  

| SYMBOL | RANK IN GENE LIST | RANK METRIC SCORE | RUNNING ES | CORE ENRICHMENT || 1 | Grhl1 | 206 | 2.530 | 0.0246 | No |
| 2 | Rassf5 | 248 | 2.317 | 0.0779 | No |
| 3 | Mif | 323 | 1.991 | 0.1156 | No |
| 4 | Epn3 | 379 | 1.758 | 0.1510 | No |
| 5 | Hspb1 | 460 | 1.530 | 0.1752 | No |
| 6 | Ifitm10 | 584 | 1.233 | 0.1824 | No |
| 7 | Cryab | 800 | 0.869 | 0.1608 | No |
| 8 | Ppp1r13l | 816 | 0.848 | 0.1803 | No |
| 9 | Palld | 927 | 0.725 | 0.1767 | No |
| 10 | Tpm1 | 1262 | -0.514 | 0.1207 | No |
| 11 | Rab1b | 1457 | -0.542 | 0.0947 | No |
| 12 | Rtl8b | 1823 | -0.604 | 0.0347 | No |
| 13 | Rbpms | 2228 | -0.676 | -0.0316 | No |
| 14 | Pnkd | 2487 | -0.726 | -0.0660 | No |
| 15 | Hcfc1r1 | 2783 | -0.787 | -0.1066 | No |
| 16 | Atp1a1 | 3251 | -0.916 | -0.1795 | No |
| 17 | Got1 | 3326 | -0.939 | -0.1699 | No |
| 18 | Fhl2 | 3508 | -1.000 | -0.1810 | No |
| 19 | Mical2 | 3554 | -1.016 | -0.1633 | No |
| 20 | Pkp2 | 3589 | -1.028 | -0.1429 | No |
| 21 | Eno3 | 3938 | -1.207 | -0.1833 | Yes |
| 22 | Bmp2 | 4098 | -1.322 | -0.1812 | Yes |
| 23 | Slc8a1 | 4109 | -1.334 | -0.1476 | Yes |
| 24 | Atp1b1 | 4241 | -1.458 | -0.1360 | Yes |
| 25 | Csrp2 | 4490 | -1.794 | -0.1399 | Yes |
| 26 | Myh7 | 4666 | -2.244 | -0.1165 | Yes |
| 27 | Dmd | 4759 | -2.718 | -0.0631 | Yes |
| 28 | Gata5 | 4777 | -2.834 | 0.0090 | Yes |
Table: GSEA details [plain text format]

  

Fig 2: DESCARTES\_ORGANOGENESIS\_CARDIAC\_MUSCLE\_LINEAGES: Random ES distribution      
 Gene set null distribution of ES for **DESCARTES\_ORGANOGENESIS\_CARDIAC\_MUSCLE\_LINEAGES**

  
