# Supplementary material for: TMPRSS11B promotes an acidified microenvironment and immune suppression in squamous lung cancer
Source: EMBO Rep. 2025 Nov 10;26(24):6346–79. doi: 10.1038/s44319-025-00631-1 (PMC12714794; doi:10.1038/s44319-025-00631-1)
Supplement: Supplementary file 10 — Source data Fig. 5 [file 44319_2025_631_MOESM10_ESM.zip › Figure 5/5C-D/GSEA_Broad Institute_M8_T11b-high LUSC vs LUAD/DESCARTES_ORGANOGENESIS_CHONDROCYTE_PROGENITORS.html]

Details for gene set DESCARTES\_ORGANOGENESIS\_CHONDROCYTE\_PROGENITORS[GSEA]

|  || Dataset | Ranked list\_DGE\_squamousT11b\_vs\_all adenosadeno\_HSE13-NT copy |
| Phenotype | NoPhenotypeAvailable |
| Upregulated in class | na\_neg |
| GeneSet | DESCARTES\_ORGANOGENESIS\_CHONDROCYTE\_PROGENITORS |
| Enrichment Score (ES) | -0.31416836 |
| Normalized Enrichment Score (NES) | -1.0721089 |
| Nominal p-value | 0.378 |
| FDR q-value | 1.0 |
| FWER p-Value | 1.0 |
Table: GSEA Results Summary

  

Fig 1: Enrichment plot: DESCARTES\_ORGANOGENESIS\_CHONDROCYTE\_PROGENITORS      
 Profile of the Running ES Score & Positions of GeneSet Members on the Rank Ordered List

  

| SYMBOL | RANK IN GENE LIST | RANK METRIC SCORE | RUNNING ES | CORE ENRICHMENT || 1 | Flrt2 | 142 | 3.127 | 0.1248 | No |
| 2 | Slc7a11 | 277 | 2.198 | 0.2055 | No |
| 3 | Slc6a20a | 741 | 0.950 | 0.1560 | No |
| 4 | Colec12 | 908 | 0.751 | 0.1586 | No |
| 5 | Lrrk2 | 919 | 0.739 | 0.1929 | No |
| 6 | Bgn | 1714 | -0.584 | 0.0565 | No |
| 7 | Il6st | 2082 | -0.650 | 0.0123 | No |
| 8 | Ifi27 | 2905 | -0.815 | -0.1186 | No |
| 9 | Ghdc | 3526 | -1.007 | -0.1979 | No |
| 10 | Syt12 | 4086 | -1.316 | -0.2492 | Yes |
| 11 | Gmds | 4203 | -1.412 | -0.2036 | Yes |
| 12 | Alcam | 4291 | -1.496 | -0.1479 | Yes |
| 13 | Sidt1 | 4350 | -1.575 | -0.0822 | Yes |
| 14 | Dock5 | 4454 | -1.749 | -0.0173 | Yes |
| 15 | Itgbl1 | 4539 | -1.889 | 0.0585 | Yes |
Table: GSEA details [plain text format]

  

Fig 2: DESCARTES\_ORGANOGENESIS\_CHONDROCYTE\_PROGENITORS: Random ES distribution      
 Gene set null distribution of ES for **DESCARTES\_ORGANOGENESIS\_CHONDROCYTE\_PROGENITORS**

  
