# Supplementary material for: TMPRSS11B promotes an acidified microenvironment and immune suppression in squamous lung cancer
Source: EMBO Rep. 2025 Nov 10;26(24):6346–79. doi: 10.1038/s44319-025-00631-1 (PMC12714794; doi:10.1038/s44319-025-00631-1)
Supplement: Supplementary file 10 — Source data Fig. 5 [file 44319_2025_631_MOESM10_ESM.zip › Figure 5/5C-D/GSEA_Broad Institute_M8_T11b-high LUSC vs LUAD/DESCARTES_ORGANOGENESIS_EPENDYMAL_CELL.html]

Details for gene set DESCARTES\_ORGANOGENESIS\_EPENDYMAL\_CELL[GSEA]

|  || Dataset | Ranked list\_DGE\_squamousT11b\_vs\_all adenosadeno\_HSE13-NT copy |
| Phenotype | NoPhenotypeAvailable |
| Upregulated in class | na\_neg |
| GeneSet | DESCARTES\_ORGANOGENESIS\_EPENDYMAL\_CELL |
| Enrichment Score (ES) | -0.43894556 |
| Normalized Enrichment Score (NES) | -1.5926913 |
| Nominal p-value | 0.03869654 |
| FDR q-value | 0.19906928 |
| FWER p-Value | 0.711 |
Table: GSEA Results Summary

  

Fig 1: Enrichment plot: DESCARTES\_ORGANOGENESIS\_EPENDYMAL\_CELL      
 Profile of the Running ES Score & Positions of GeneSet Members on the Rank Ordered List

  

| SYMBOL | RANK IN GENE LIST | RANK METRIC SCORE | RUNNING ES | CORE ENRICHMENT || 1 | Pthlh | 152 | 3.000 | 0.0760 | No |
| 2 | Dusp14 | 324 | 1.988 | 0.1118 | No |
| 3 | Igfbp2 | 408 | 1.668 | 0.1544 | No |
| 4 | Pef1 | 2385 | -0.703 | -0.2318 | No |
| 5 | Scrn2 | 2803 | -0.791 | -0.2902 | No |
| 6 | Dkk3 | 2880 | -0.810 | -0.2770 | No |
| 7 | Wls | 3401 | -0.961 | -0.3507 | No |
| 8 | Ezr | 3702 | -1.081 | -0.3744 | No |
| 9 | Cfap69 | 4013 | -1.255 | -0.3939 | Yes |
| 10 | Ccdc149 | 4170 | -1.387 | -0.3766 | Yes |
| 11 | Bbs9 | 4205 | -1.412 | -0.3330 | Yes |
| 12 | Zkscan7 | 4209 | -1.415 | -0.2828 | Yes |
| 13 | Slc2a12 | 4293 | -1.498 | -0.2463 | Yes |
| 14 | 1700088E04Rik | 4383 | -1.621 | -0.2066 | Yes |
| 15 | Clmn | 4396 | -1.636 | -0.1504 | Yes |
| 16 | Shroom3 | 4545 | -1.904 | -0.1129 | Yes |
| 17 | Capsl | 4550 | -1.913 | -0.0451 | Yes |
| 18 | Slc5a3 | 4776 | -2.816 | 0.0092 | Yes |
Table: GSEA details [plain text format]

  

Fig 2: DESCARTES\_ORGANOGENESIS\_EPENDYMAL\_CELL: Random ES distribution      
 Gene set null distribution of ES for **DESCARTES\_ORGANOGENESIS\_EPENDYMAL\_CELL**

  
