# Supplementary material for: TMPRSS11B promotes an acidified microenvironment and immune suppression in squamous lung cancer
Source: EMBO Rep. 2025 Nov 10;26(24):6346–79. doi: 10.1038/s44319-025-00631-1 (PMC12714794; doi:10.1038/s44319-025-00631-1)
Supplement: Supplementary file 10 — Source data Fig. 5 [file 44319_2025_631_MOESM10_ESM.zip › Figure 5/5C-D/GSEA_Broad Institute_M8_T11b-high LUSC vs LUAD/DESCARTES_ORGANOGENESIS_HEPATOCYTES.html]

Details for gene set DESCARTES\_ORGANOGENESIS\_HEPATOCYTES[GSEA]

|  || Dataset | Ranked list\_DGE\_squamousT11b\_vs\_all adenosadeno\_HSE13-NT copy |
| Phenotype | NoPhenotypeAvailable |
| Upregulated in class | na\_neg |
| GeneSet | DESCARTES\_ORGANOGENESIS\_HEPATOCYTES |
| Enrichment Score (ES) | -0.4162854 |
| Normalized Enrichment Score (NES) | -2.615865 |
| Nominal p-value | 0.0 |
| FDR q-value | 0.0 |
| FWER p-Value | 0.0 |
Table: GSEA Results Summary

  

Fig 1: Enrichment plot: DESCARTES\_ORGANOGENESIS\_HEPATOCYTES      
 Profile of the Running ES Score & Positions of GeneSet Members on the Rank Ordered List

  

| SYMBOL | RANK IN GENE LIST | RANK METRIC SCORE | RUNNING ES | CORE ENRICHMENT || 1 | Dhrs9 | 86 | 3.898 | -0.0022 | No |
| 2 | Slc25a48 | 192 | 2.648 | -0.0137 | No |
| 3 | Stra6l | 236 | 2.369 | -0.0130 | No |
| 4 | Slc36a2 | 269 | 2.240 | -0.0105 | No |
| 5 | F7 | 279 | 2.188 | -0.0033 | No |
| 6 | Bdh1 | 364 | 1.808 | -0.0138 | No |
| 7 | Itih4 | 375 | 1.767 | -0.0085 | No |
| 8 | Apoc1 | 386 | 1.739 | -0.0034 | No |
| 9 | Hsd17b2 | 393 | 1.711 | 0.0025 | No |
| 10 | Pygl | 404 | 1.673 | 0.0073 | No |
| 11 | Plekhg6 | 436 | 1.593 | 0.0073 | No |
| 12 | Mmp19 | 439 | 1.573 | 0.0135 | No |
| 13 | C3 | 474 | 1.502 | 0.0125 | No |
| 14 | Cd302 | 521 | 1.393 | 0.0084 | No |
| 15 | Tmem37 | 539 | 1.356 | 0.0104 | No |
| 16 | Fah | 583 | 1.236 | 0.0063 | No |
| 17 | Gcat | 639 | 1.111 | -0.0008 | No |
| 18 | Osgin1 | 663 | 1.064 | -0.0013 | No |
| 19 | Ndrg1 | 681 | 1.033 | -0.0007 | No |
| 20 | Trf | 682 | 1.033 | 0.0037 | No |
| 21 | Rnf144b | 732 | 0.962 | -0.0029 | No |
| 22 | Mocos | 767 | 0.908 | -0.0064 | No |
| 23 | Fkbp11 | 864 | 0.807 | -0.0236 | No |
| 24 | Sdr42e1 | 910 | 0.750 | -0.0302 | No |
| 25 | Adk | 931 | 0.720 | -0.0315 | No |
| 26 | Psat1 | 954 | 0.702 | -0.0333 | No |
| 27 | Gstt2 | 1012 | 0.639 | -0.0429 | No |
| 28 | Ikbke | 1027 | 0.626 | -0.0433 | No |
| 29 | Il1r1 | 1040 | 0.614 | -0.0433 | No |
| 30 | Kifc3 | 1046 | 0.611 | -0.0418 | No |
| 31 | Lss | 1052 | 0.608 | -0.0403 | No |
| 32 | Sord | 1146 | 0.514 | -0.0582 | No |
| 33 | Anpep | 1162 | 0.504 | -0.0593 | No |
| 34 | Anxa4 | 1225 | -0.509 | -0.0705 | No |
| 35 | Fpgs | 1242 | -0.511 | -0.0718 | No |
| 36 | Cutc | 1285 | -0.517 | -0.0787 | No |
| 37 | Tmem176a | 1324 | -0.522 | -0.0847 | No |
| 38 | Selenbp1 | 1398 | -0.532 | -0.0981 | No |
| 39 | Shfl | 1443 | -0.540 | -0.1054 | No |
| 40 | Hdlbp | 1479 | -0.548 | -0.1106 | No |
| 41 | Mgst1 | 1519 | -0.554 | -0.1167 | No |
| 42 | Acad11 | 1538 | -0.558 | -0.1182 | No |
| 43 | Tmem205 | 1549 | -0.560 | -0.1180 | No |
| 44 | Pxmp4 | 1621 | -0.572 | -0.1309 | No |
| 45 | Inpp5f | 1783 | -0.597 | -0.1630 | No |
| 46 | Cluh | 1835 | -0.606 | -0.1715 | No |
| 47 | Acaa2 | 1846 | -0.609 | -0.1711 | No |
| 48 | Snd1 | 1866 | -0.613 | -0.1726 | No |
| 49 | Bphl | 1944 | -0.625 | -0.1865 | No |
| 50 | R3hdm2 | 2036 | -0.641 | -0.2034 | No |
| 51 | Gne | 2070 | -0.647 | -0.2078 | No |
| 52 | Grhpr | 2089 | -0.652 | -0.2090 | No |
| 53 | Chka | 2293 | -0.687 | -0.2498 | No |
| 54 | Errfi1 | 2313 | -0.690 | -0.2510 | No |
| 55 | Vwa8 | 2412 | -0.707 | -0.2691 | No |
| 56 | Sdc4 | 2418 | -0.710 | -0.2672 | No |
| 57 | Hip1r | 2473 | -0.723 | -0.2758 | No |
| 58 | Als2cl | 2544 | -0.735 | -0.2878 | No |
| 59 | Agpat2 | 2599 | -0.747 | -0.2963 | No |
| 60 | Ngef | 2671 | -0.763 | -0.3083 | No |
| 61 | Pla2g6 | 2683 | -0.764 | -0.3075 | No |
| 62 | Selenbp2 | 2695 | -0.767 | -0.3067 | No |
| 63 | Preb | 2722 | -0.771 | -0.3090 | No |
| 64 | Fabp5 | 2745 | -0.778 | -0.3105 | No |
| 65 | Ddt | 2787 | -0.788 | -0.3160 | No |
| 66 | Slc25a33 | 2952 | -0.827 | -0.3479 | No |
| 67 | Nostrin | 2973 | -0.835 | -0.3487 | No |
| 68 | Ephx2 | 2986 | -0.838 | -0.3477 | No |
| 69 | Insr | 2987 | -0.838 | -0.3442 | No |
| 70 | Slc39a14 | 3030 | -0.850 | -0.3497 | No |
| 71 | Gjb1 | 3084 | -0.866 | -0.3575 | No |
| 72 | Zscan26 | 3092 | -0.868 | -0.3554 | No |
| 73 | Plekhg3 | 3106 | -0.873 | -0.3545 | No |
| 74 | Sirt3 | 3232 | -0.909 | -0.3776 | No |
| 75 | Gsta3 | 3234 | -0.910 | -0.3740 | No |
| 76 | Slc19a2 | 3267 | -0.920 | -0.3770 | No |
| 77 | Acsl1 | 3337 | -0.941 | -0.3879 | No |
| 78 | Cryz | 3421 | -0.972 | -0.4017 | No |
| 79 | Slc25a15 | 3433 | -0.976 | -0.4000 | No |
| 80 | Chmp4c | 3440 | -0.977 | -0.3972 | No |
| 81 | Asl | 3473 | -0.988 | -0.4000 | No |
| 82 | Usp18 | 3483 | -0.991 | -0.3977 | No |
| 83 | Pex26 | 3486 | -0.992 | -0.3940 | No |
| 84 | Nt5e | 3535 | -1.010 | -0.4001 | No |
| 85 | Ccdc125 | 3548 | -1.014 | -0.3984 | No |
| 86 | Ggcx | 3561 | -1.017 | -0.3968 | No |
| 87 | Soat2 | 3567 | -1.020 | -0.3936 | No |
| 88 | Rnf128 | 3580 | -1.025 | -0.3919 | No |
| 89 | Abcc10 | 3656 | -1.057 | -0.4036 | No |
| 90 | Grk3 | 3691 | -1.074 | -0.4064 | No |
| 91 | Pcyt2 | 3738 | -1.098 | -0.4117 | Yes |
| 92 | Foxa3 | 3746 | -1.101 | -0.4086 | Yes |
| 93 | 2310039H08Rik | 3763 | -1.108 | -0.4074 | Yes |
| 94 | Sh3bgrl2 | 3772 | -1.111 | -0.4045 | Yes |
| 95 | Cfb | 3801 | -1.129 | -0.4058 | Yes |
| 96 | Baiap2l1 | 3803 | -1.130 | -0.4012 | Yes |
| 97 | Sfxn2 | 3810 | -1.134 | -0.3978 | Yes |
| 98 | Ctps2 | 3820 | -1.140 | -0.3949 | Yes |
| 99 | Mcrip2 | 3863 | -1.163 | -0.3991 | Yes |
| 100 | Cyp3a13 | 3865 | -1.163 | -0.3945 | Yes |
| 101 | Galm | 3869 | -1.165 | -0.3902 | Yes |
| 102 | Hkdc1 | 3877 | -1.168 | -0.3868 | Yes |
| 103 | Lactb2 | 3878 | -1.170 | -0.3819 | Yes |
| 104 | 0610040J01Rik | 3911 | -1.192 | -0.3838 | Yes |
| 105 | Hadh | 3948 | -1.215 | -0.3865 | Yes |
| 106 | Nipsnap1 | 3976 | -1.230 | -0.3871 | Yes |
| 107 | Aldh3a2 | 3988 | -1.239 | -0.3843 | Yes |
| 108 | Zfp395 | 4006 | -1.251 | -0.3827 | Yes |
| 109 | Tmem150a | 4015 | -1.256 | -0.3792 | Yes |
| 110 | Abcd3 | 4034 | -1.270 | -0.3777 | Yes |
| 111 | Pgm3 | 4049 | -1.283 | -0.3754 | Yes |
| 112 | Rhpn2 | 4091 | -1.320 | -0.3787 | Yes |
| 113 | Pxmp2 | 4099 | -1.324 | -0.3746 | Yes |
| 114 | Rassf6 | 4140 | -1.362 | -0.3775 | Yes |
| 115 | Ocln | 4148 | -1.367 | -0.3733 | Yes |
| 116 | Mettl26 | 4185 | -1.397 | -0.3752 | Yes |
| 117 | Gstm1 | 4200 | -1.410 | -0.3723 | Yes |
| 118 | Steap2 | 4223 | -1.439 | -0.3710 | Yes |
| 119 | Gldc | 4232 | -1.446 | -0.3667 | Yes |
| 120 | Slc25a10 | 4253 | -1.469 | -0.3649 | Yes |
| 121 | Aqp11 | 4256 | -1.470 | -0.3591 | Yes |
| 122 | Gpd1 | 4257 | -1.470 | -0.3530 | Yes |
| 123 | Gipc2 | 4295 | -1.500 | -0.3547 | Yes |
| 124 | Ttc38 | 4300 | -1.505 | -0.3492 | Yes |
| 125 | Slc22a18 | 4312 | -1.515 | -0.3452 | Yes |
| 126 | Atp8b1 | 4315 | -1.523 | -0.3393 | Yes |
| 127 | Stard10 | 4317 | -1.525 | -0.3331 | Yes |
| 128 | Fgf1 | 4328 | -1.540 | -0.3288 | Yes |
| 129 | Mkrn2os | 4341 | -1.558 | -0.3249 | Yes |
| 130 | Aldh6a1 | 4345 | -1.570 | -0.3189 | Yes |
| 131 | Tmem51 | 4368 | -1.598 | -0.3170 | Yes |
| 132 | Slc35d2 | 4385 | -1.623 | -0.3136 | Yes |
| 133 | Pctp | 4414 | -1.665 | -0.3127 | Yes |
| 134 | Bche | 4424 | -1.680 | -0.3076 | Yes |
| 135 | Phyh | 4469 | -1.768 | -0.3096 | Yes |
| 136 | Chdh | 4474 | -1.777 | -0.3030 | Yes |
| 137 | Cbx7 | 4479 | -1.780 | -0.2965 | Yes |
| 138 | Nadsyn1 | 4491 | -1.796 | -0.2913 | Yes |
| 139 | 2810459M11Rik | 4535 | -1.882 | -0.2927 | Yes |
| 140 | Pdia5 | 4544 | -1.894 | -0.2865 | Yes |
| 141 | Faah | 4548 | -1.910 | -0.2791 | Yes |
| 142 | Pcbd1 | 4552 | -1.919 | -0.2717 | Yes |
| 143 | Tcea3 | 4554 | -1.926 | -0.2639 | Yes |
| 144 | Echdc2 | 4557 | -1.936 | -0.2562 | Yes |
| 145 | Steap1 | 4588 | -2.016 | -0.2542 | Yes |
| 146 | Cideb | 4591 | -2.024 | -0.2461 | Yes |
| 147 | Dop1b | 4611 | -2.077 | -0.2415 | Yes |
| 148 | Dnajc22 | 4615 | -2.083 | -0.2335 | Yes |
| 149 | Pkhd1 | 4626 | -2.104 | -0.2268 | Yes |
| 150 | Akr1c13 | 4632 | -2.122 | -0.2190 | Yes |
| 151 | Hnf1a | 4638 | -2.142 | -0.2111 | Yes |
| 152 | Mgst2 | 4641 | -2.150 | -0.2025 | Yes |
| 153 | Dqx1 | 4649 | -2.185 | -0.1949 | Yes |
| 154 | Cfi | 4653 | -2.192 | -0.1863 | Yes |
| 155 | Aspa | 4655 | -2.196 | -0.1773 | Yes |
| 156 | Slc44a3 | 4658 | -2.202 | -0.1685 | Yes |
| 157 | Hgfac | 4673 | -2.270 | -0.1620 | Yes |
| 158 | Hpn | 4685 | -2.329 | -0.1547 | Yes |
| 159 | Hnf4a | 4686 | -2.337 | -0.1449 | Yes |
| 160 | Pbld2 | 4695 | -2.371 | -0.1367 | Yes |
| 161 | Bcas1 | 4696 | -2.373 | -0.1267 | Yes |
| 162 | Pik3c2g | 4699 | -2.391 | -0.1171 | Yes |
| 163 | Acnat1 | 4702 | -2.400 | -0.1075 | Yes |
| 164 | Tfcp2l1 | 4708 | -2.421 | -0.0984 | Yes |
| 165 | Hmgcs2 | 4714 | -2.435 | -0.0893 | Yes |
| 166 | Iyd | 4739 | -2.572 | -0.0837 | Yes |
| 167 | Akr1c19 | 4742 | -2.597 | -0.0733 | Yes |
| 168 | Ugt2b34 | 4751 | -2.666 | -0.0638 | Yes |
| 169 | Krt20 | 4767 | -2.756 | -0.0555 | Yes |
| 170 | Atp8a2 | 4779 | -2.835 | -0.0460 | Yes |
| 171 | Sec16b | 4780 | -2.854 | -0.0340 | Yes |
| 172 | Sytl5 | 4796 | -3.174 | -0.0240 | Yes |
| 173 | Itih2 | 4803 | -3.281 | -0.0115 | Yes |
| 174 | Baiap2l2 | 4812 | -3.574 | 0.0017 | Yes |
Table: GSEA details [plain text format]

  

Fig 2: DESCARTES\_ORGANOGENESIS\_HEPATOCYTES: Random ES distribution      
 Gene set null distribution of ES for **DESCARTES\_ORGANOGENESIS\_HEPATOCYTES**

  
