# Supplementary material for: TMPRSS11B promotes an acidified microenvironment and immune suppression in squamous lung cancer
Source: EMBO Rep. 2025 Nov 10;26(24):6346–79. doi: 10.1038/s44319-025-00631-1 (PMC12714794; doi:10.1038/s44319-025-00631-1)
Supplement: Supplementary file 10 — Source data Fig. 5 [file 44319_2025_631_MOESM10_ESM.zip › Figure 5/5C-D/GSEA_Broad Institute_M8_T11b-high LUSC vs LUAD/DESCARTES_ORGANOGENESIS_MYOCYTES.html]

Details for gene set DESCARTES\_ORGANOGENESIS\_MYOCYTES[GSEA]

|  || Dataset | Ranked list\_DGE\_squamousT11b\_vs\_all adenosadeno\_HSE13-NT copy |
| Phenotype | NoPhenotypeAvailable |
| Upregulated in class | na\_neg |
| GeneSet | DESCARTES\_ORGANOGENESIS\_MYOCYTES |
| Enrichment Score (ES) | -0.40572447 |
| Normalized Enrichment Score (NES) | -1.467047 |
| Nominal p-value | 0.07692308 |
| FDR q-value | 0.2775065 |
| FWER p-Value | 0.945 |
Table: GSEA Results Summary

  

Fig 1: Enrichment plot: DESCARTES\_ORGANOGENESIS\_MYOCYTES      
 Profile of the Running ES Score & Positions of GeneSet Members on the Rank Ordered List

  

| SYMBOL | RANK IN GENE LIST | RANK METRIC SCORE | RUNNING ES | CORE ENRICHMENT || 1 | Ablim3 | 225 | 2.415 | 0.0387 | No |
| 2 | Frmpd1 | 293 | 2.120 | 0.0998 | No |
| 3 | Tpm2 | 308 | 2.054 | 0.1696 | No |
| 4 | Cdkn1a | 861 | 0.808 | 0.0833 | No |
| 5 | Clcn5 | 1496 | -0.551 | -0.0292 | No |
| 6 | Gramd1b | 2843 | -0.801 | -0.2811 | No |
| 7 | Ncoa1 | 3304 | -0.931 | -0.3439 | No |
| 8 | Cd82 | 3518 | -1.004 | -0.3527 | Yes |
| 9 | Parm1 | 3570 | -1.021 | -0.3272 | Yes |
| 10 | Rnf217 | 3712 | -1.085 | -0.3181 | Yes |
| 11 | Macrod1 | 3732 | -1.095 | -0.2833 | Yes |
| 12 | Ptgis | 4321 | -1.530 | -0.3515 | Yes |
| 13 | Adora1 | 4344 | -1.569 | -0.3006 | Yes |
| 14 | Itgb6 | 4485 | -1.787 | -0.2664 | Yes |
| 15 | Sytl2 | 4596 | -2.034 | -0.2173 | Yes |
| 16 | Fbxo17 | 4656 | -2.197 | -0.1518 | Yes |
| 17 | Adh1 | 4746 | -2.614 | -0.0778 | Yes |
| 18 | Igdcc4 | 4747 | -2.627 | 0.0152 | Yes |
Table: GSEA details [plain text format]

  

Fig 2: DESCARTES\_ORGANOGENESIS\_MYOCYTES: Random ES distribution      
 Gene set null distribution of ES for **DESCARTES\_ORGANOGENESIS\_MYOCYTES**

  
