# Supplementary material for: TMPRSS11B promotes an acidified microenvironment and immune suppression in squamous lung cancer
Source: EMBO Rep. 2025 Nov 10;26(24):6346–79. doi: 10.1038/s44319-025-00631-1 (PMC12714794; doi:10.1038/s44319-025-00631-1)
Supplement: Supplementary file 10 — Source data Fig. 5 [file 44319_2025_631_MOESM10_ESM.zip › Figure 5/5C-D/GSEA_Broad Institute_M8_T11b-high LUSC vs LUAD/DESCARTES_ORGANOGENESIS_SCHWANN_CELL_PRECURSOR.html]

Details for gene set DESCARTES\_ORGANOGENESIS\_SCHWANN\_CELL\_PRECURSOR[GSEA]

|  || Dataset | Ranked list\_DGE\_squamousT11b\_vs\_all adenosadeno\_HSE13-NT copy |
| Phenotype | NoPhenotypeAvailable |
| Upregulated in class | na\_neg |
| GeneSet | DESCARTES\_ORGANOGENESIS\_SCHWANN\_CELL\_PRECURSOR |
| Enrichment Score (ES) | -0.41918522 |
| Normalized Enrichment Score (NES) | -1.6248821 |
| Nominal p-value | 0.026086956 |
| FDR q-value | 0.23419803 |
| FWER p-Value | 0.631 |
Table: GSEA Results Summary

  

Fig 1: Enrichment plot: DESCARTES\_ORGANOGENESIS\_SCHWANN\_CELL\_PRECURSOR      
 Profile of the Running ES Score & Positions of GeneSet Members on the Rank Ordered List

  

| SYMBOL | RANK IN GENE LIST | RANK METRIC SCORE | RUNNING ES | CORE ENRICHMENT || 1 | Afap1l2 | 270 | 2.236 | 0.0058 | No |
| 2 | Sntb1 | 307 | 2.065 | 0.0556 | No |
| 3 | Matn2 | 316 | 2.022 | 0.1100 | No |
| 4 | Megf9 | 380 | 1.758 | 0.1457 | No |
| 5 | Mal | 733 | 0.961 | 0.0990 | No |
| 6 | Fhdc1 | 844 | 0.816 | 0.0987 | No |
| 7 | Entpd2 | 1201 | -0.506 | 0.0386 | No |
| 8 | Erbb3 | 1278 | -0.516 | 0.0370 | No |
| 9 | Ugdh | 1692 | -0.583 | -0.0329 | No |
| 10 | Heyl | 1736 | -0.589 | -0.0255 | No |
| 11 | Wdfy1 | 2153 | -0.662 | -0.0938 | No |
| 12 | Cyp2j6 | 3348 | -0.948 | -0.3164 | No |
| 13 | Rasa2 | 3497 | -0.995 | -0.3197 | No |
| 14 | Serpine2 | 3936 | -1.205 | -0.3775 | Yes |
| 15 | Deptor | 4082 | -1.314 | -0.3712 | Yes |
| 16 | Ednrb | 4313 | -1.516 | -0.3771 | Yes |
| 17 | Bcar3 | 4336 | -1.548 | -0.3387 | Yes |
| 18 | Lmo4 | 4382 | -1.617 | -0.3032 | Yes |
| 19 | Itprid2 | 4401 | -1.648 | -0.2612 | Yes |
| 20 | Adgrg6 | 4542 | -1.892 | -0.2379 | Yes |
| 21 | Dagla | 4546 | -1.906 | -0.1856 | Yes |
| 22 | Tmem117 | 4712 | -2.430 | -0.1526 | Yes |
| 23 | Cdh6 | 4793 | -3.096 | -0.0833 | Yes |
| 24 | Sema3b | 4798 | -3.196 | 0.0046 | Yes |
Table: GSEA details [plain text format]

  

Fig 2: DESCARTES\_ORGANOGENESIS\_SCHWANN\_CELL\_PRECURSOR: Random ES distribution      
 Gene set null distribution of ES for **DESCARTES\_ORGANOGENESIS\_SCHWANN\_CELL\_PRECURSOR**

  
