# Supplementary material for: TMPRSS11B promotes an acidified microenvironment and immune suppression in squamous lung cancer
Source: EMBO Rep. 2025 Nov 10;26(24):6346–79. doi: 10.1038/s44319-025-00631-1 (PMC12714794; doi:10.1038/s44319-025-00631-1)
Supplement: Supplementary file 10 — Source data Fig. 5 [file 44319_2025_631_MOESM10_ESM.zip › Figure 5/5C-D/GSEA_Broad Institute_M8_T11b-high LUSC vs LUAD/DESCARTES_ORGANOGENESIS_SENSORY_NEURONS.html]

Details for gene set DESCARTES\_ORGANOGENESIS\_SENSORY\_NEURONS[GSEA]

|  || Dataset | Ranked list\_DGE\_squamousT11b\_vs\_all adenosadeno\_HSE13-NT copy |
| Phenotype | NoPhenotypeAvailable |
| Upregulated in class | na\_neg |
| GeneSet | DESCARTES\_ORGANOGENESIS\_SENSORY\_NEURONS |
| Enrichment Score (ES) | -0.37501156 |
| Normalized Enrichment Score (NES) | -1.4687128 |
| Nominal p-value | 0.06949807 |
| FDR q-value | 0.34199435 |
| FWER p-Value | 0.944 |
Table: GSEA Results Summary

  

Fig 1: Enrichment plot: DESCARTES\_ORGANOGENESIS\_SENSORY\_NEURONS      
 Profile of the Running ES Score & Positions of GeneSet Members on the Rank Ordered List

  

| SYMBOL | RANK IN GENE LIST | RANK METRIC SCORE | RUNNING ES | CORE ENRICHMENT || 1 | Mreg | 72 | 4.109 | 0.1175 | No |
| 2 | Cadm1 | 633 | 1.126 | 0.0371 | No |
| 3 | Rnf150 | 832 | 0.833 | 0.0227 | No |
| 4 | Serpinb8 | 1074 | 0.582 | -0.0087 | No |
| 5 | Enah | 1159 | 0.506 | -0.0099 | No |
| 6 | Ccser2 | 1267 | -0.515 | -0.0156 | No |
| 7 | Phc2 | 1292 | -0.518 | -0.0039 | No |
| 8 | Csnk1g1 | 1720 | -0.586 | -0.0740 | No |
| 9 | Rapgef6 | 1950 | -0.627 | -0.1015 | No |
| 10 | Rap1gap2 | 2645 | -0.758 | -0.2217 | No |
| 11 | Pde4dip | 3155 | -0.886 | -0.2992 | No |
| 12 | B4galnt3 | 3367 | -0.952 | -0.3125 | No |
| 13 | Ankrd24 | 3443 | -0.978 | -0.2966 | No |
| 14 | Kif13b | 3513 | -1.002 | -0.2786 | No |
| 15 | Synpo | 3888 | -1.180 | -0.3185 | No |
| 16 | Sgpp2 | 4160 | -1.380 | -0.3305 | Yes |
| 17 | Itga7 | 4326 | -1.538 | -0.3153 | Yes |
| 18 | Ccdc92 | 4327 | -1.540 | -0.2656 | Yes |
| 19 | Mapk8ip1 | 4373 | -1.605 | -0.2232 | Yes |
| 20 | Kif19a | 4489 | -1.794 | -0.1894 | Yes |
| 21 | Rgs11 | 4652 | -2.187 | -0.1526 | Yes |
| 22 | Cerkl | 4756 | -2.698 | -0.0870 | Yes |
| 23 | Tesc | 4795 | -3.106 | 0.0052 | Yes |
Table: GSEA details [plain text format]

  

Fig 2: DESCARTES\_ORGANOGENESIS\_SENSORY\_NEURONS: Random ES distribution      
 Gene set null distribution of ES for **DESCARTES\_ORGANOGENESIS\_SENSORY\_NEURONS**

  
