# Supplementary material for: TMPRSS11B promotes an acidified microenvironment and immune suppression in squamous lung cancer
Source: EMBO Rep. 2025 Nov 10;26(24):6346–79. doi: 10.1038/s44319-025-00631-1 (PMC12714794; doi:10.1038/s44319-025-00631-1)
Supplement: Supplementary file 10 — Source data Fig. 5 [file 44319_2025_631_MOESM10_ESM.zip › Figure 5/5C-D/GSEA_Broad Institute_M8_T11b-high LUSC vs LUAD/DESCARTES_ORGANOGENESIS_WHITE_BLOOD_CELLS.html]

Details for gene set DESCARTES\_ORGANOGENESIS\_WHITE\_BLOOD\_CELLS[GSEA]

|  || Dataset | Ranked list\_DGE\_squamousT11b\_vs\_all adenosadeno\_HSE13-NT copy |
| Phenotype | NoPhenotypeAvailable |
| Upregulated in class | na\_pos |
| GeneSet | DESCARTES\_ORGANOGENESIS\_WHITE\_BLOOD\_CELLS |
| Enrichment Score (ES) | 0.56712943 |
| Normalized Enrichment Score (NES) | 3.318962 |
| Nominal p-value | 0.0 |
| FDR q-value | 0.0 |
| FWER p-Value | 0.0 |
Table: GSEA Results Summary

  

Fig 1: Enrichment plot: DESCARTES\_ORGANOGENESIS\_WHITE\_BLOOD\_CELLS      
 Profile of the Running ES Score & Positions of GeneSet Members on the Rank Ordered List

  

| SYMBOL | RANK IN GENE LIST | RANK METRIC SCORE | RUNNING ES | CORE ENRICHMENT || 1 | Clec2g | 45 | 4.739 | 0.0054 | Yes |
| 2 | Ccl9 | 82 | 3.944 | 0.0103 | Yes |
| 3 | Il1a | 119 | 3.497 | 0.0137 | Yes |
| 4 | Csf3r | 124 | 3.373 | 0.0237 | Yes |
| 5 | Itgam | 132 | 3.250 | 0.0326 | Yes |
| 6 | Hk3 | 135 | 3.223 | 0.0425 | Yes |
| 7 | Trem2 | 140 | 3.156 | 0.0518 | Yes |
| 8 | Hmox1 | 144 | 3.102 | 0.0611 | Yes |
| 9 | Cd84 | 159 | 2.936 | 0.0675 | Yes |
| 10 | Cd300lf | 160 | 2.935 | 0.0769 | Yes |
| 11 | Gna15 | 161 | 2.913 | 0.0863 | Yes |
| 12 | Slc37a2 | 168 | 2.843 | 0.0941 | Yes |
| 13 | Slc11a1 | 169 | 2.826 | 0.1032 | Yes |
| 14 | Cybb | 173 | 2.805 | 0.1115 | Yes |
| 15 | Cd68 | 177 | 2.764 | 0.1197 | Yes |
| 16 | Tyrobp | 181 | 2.732 | 0.1279 | Yes |
| 17 | Npl | 184 | 2.724 | 0.1362 | Yes |
| 18 | Ccl6 | 187 | 2.695 | 0.1444 | Yes |
| 19 | Msr1 | 196 | 2.587 | 0.1510 | Yes |
| 20 | Cd36 | 199 | 2.567 | 0.1588 | Yes |
| 21 | C3ar1 | 205 | 2.531 | 0.1658 | Yes |
| 22 | Clec4n | 207 | 2.528 | 0.1737 | Yes |
| 23 | Myo5a | 231 | 2.392 | 0.1764 | Yes |
| 24 | Cyp27a1 | 239 | 2.356 | 0.1825 | Yes |
| 25 | Cd37 | 244 | 2.324 | 0.1891 | Yes |
| 26 | Pstpip1 | 245 | 2.324 | 0.1965 | Yes |
| 27 | Ctss | 247 | 2.317 | 0.2037 | Yes |
| 28 | Spi1 | 250 | 2.309 | 0.2107 | Yes |
| 29 | Cd300c2 | 258 | 2.288 | 0.2166 | Yes |
| 30 | Fcgr3 | 260 | 2.281 | 0.2237 | Yes |
| 31 | Cd53 | 261 | 2.271 | 0.2310 | Yes |
| 32 | Sirpa | 268 | 2.242 | 0.2369 | Yes |
| 33 | Ncf1 | 271 | 2.235 | 0.2436 | Yes |
| 34 | Fcer1g | 272 | 2.235 | 0.2508 | Yes |
| 35 | Ctsd | 274 | 2.219 | 0.2577 | Yes |
| 36 | Fcgr2b | 280 | 2.178 | 0.2636 | Yes |
| 37 | Cd300a | 281 | 2.174 | 0.2706 | Yes |
| 38 | Ptafr | 284 | 2.159 | 0.2771 | Yes |
| 39 | Slc15a3 | 285 | 2.148 | 0.2840 | Yes |
| 40 | Ctsb | 288 | 2.139 | 0.2904 | Yes |
| 41 | Evi2a | 309 | 2.048 | 0.2926 | Yes |
| 42 | C5ar1 | 317 | 2.019 | 0.2976 | Yes |
| 43 | Crlf2 | 325 | 1.984 | 0.3025 | Yes |
| 44 | Cd44 | 329 | 1.976 | 0.3082 | Yes |
| 45 | Cd52 | 332 | 1.963 | 0.3140 | Yes |
| 46 | Ptprc | 338 | 1.926 | 0.3191 | Yes |
| 47 | Myo1f | 340 | 1.918 | 0.3251 | Yes |
| 48 | Mpeg1 | 346 | 1.889 | 0.3301 | Yes |
| 49 | Tnfrsf26 | 347 | 1.879 | 0.3361 | Yes |
| 50 | Jdp2 | 359 | 1.855 | 0.3397 | Yes |
| 51 | Cyth4 | 361 | 1.848 | 0.3454 | Yes |
| 52 | Cd33 | 362 | 1.818 | 0.3512 | Yes |
| 53 | Hexb | 384 | 1.742 | 0.3522 | Yes |
| 54 | Nfam1 | 387 | 1.739 | 0.3574 | Yes |
| 55 | Irf5 | 394 | 1.709 | 0.3616 | Yes |
| 56 | Nckap1l | 395 | 1.708 | 0.3671 | Yes |
| 57 | Havcr2 | 399 | 1.689 | 0.3718 | Yes |
| 58 | Mertk | 402 | 1.676 | 0.3768 | Yes |
| 59 | Lcp2 | 403 | 1.676 | 0.3822 | Yes |
| 60 | Il1rl2 | 406 | 1.672 | 0.3871 | Yes |
| 61 | Dse | 407 | 1.670 | 0.3925 | Yes |
| 62 | Arhgap9 | 415 | 1.652 | 0.3963 | Yes |
| 63 | Def6 | 418 | 1.629 | 0.4011 | Yes |
| 64 | Maf | 421 | 1.624 | 0.4058 | Yes |
| 65 | Fmnl1 | 423 | 1.621 | 0.4108 | Yes |
| 66 | Cebpb | 425 | 1.620 | 0.4158 | Yes |
| 67 | Pik3ap1 | 431 | 1.606 | 0.4199 | Yes |
| 68 | Selplg | 432 | 1.604 | 0.4250 | Yes |
| 69 | Cfp | 433 | 1.599 | 0.4302 | Yes |
| 70 | Apbb1ip | 442 | 1.567 | 0.4335 | Yes |
| 71 | Tnfrsf1b | 443 | 1.566 | 0.4385 | Yes |
| 72 | Lgals3 | 447 | 1.559 | 0.4429 | Yes |
| 73 | St6galnac4 | 449 | 1.551 | 0.4476 | Yes |
| 74 | Fam111a | 455 | 1.540 | 0.4515 | Yes |
| 75 | Lgmn | 458 | 1.534 | 0.4560 | Yes |
| 76 | Plxdc1 | 464 | 1.526 | 0.4598 | Yes |
| 77 | Dock2 | 467 | 1.518 | 0.4642 | Yes |
| 78 | Mrc1 | 471 | 1.505 | 0.4684 | Yes |
| 79 | Gpr65 | 478 | 1.498 | 0.4719 | Yes |
| 80 | Hcls1 | 487 | 1.480 | 0.4749 | Yes |
| 81 | Fxyd5 | 489 | 1.476 | 0.4794 | Yes |
| 82 | Apoe | 490 | 1.475 | 0.4842 | Yes |
| 83 | Lpxn | 492 | 1.463 | 0.4887 | Yes |
| 84 | Ctsz | 493 | 1.463 | 0.4934 | Yes |
| 85 | Pycard | 499 | 1.449 | 0.4969 | Yes |
| 86 | Alox5ap | 500 | 1.445 | 0.5016 | Yes |
| 87 | Gusb | 504 | 1.435 | 0.5055 | Yes |
| 88 | Irf1 | 508 | 1.429 | 0.5095 | Yes |
| 89 | Psap | 510 | 1.415 | 0.5138 | Yes |
| 90 | Pik3cd | 516 | 1.406 | 0.5172 | Yes |
| 91 | Emp3 | 537 | 1.365 | 0.5173 | Yes |
| 92 | Slfn2 | 544 | 1.342 | 0.5203 | Yes |
| 93 | Coro1a | 546 | 1.340 | 0.5244 | Yes |
| 94 | Rab32 | 547 | 1.337 | 0.5287 | Yes |
| 95 | Grn | 554 | 1.328 | 0.5316 | Yes |
| 96 | Vsir | 576 | 1.261 | 0.5311 | Yes |
| 97 | Blnk | 588 | 1.228 | 0.5327 | Yes |
| 98 | Samhd1 | 599 | 1.201 | 0.5344 | Yes |
| 99 | Rasa4 | 600 | 1.200 | 0.5382 | Yes |
| 100 | Sat1 | 614 | 1.180 | 0.5392 | Yes |
| 101 | C1qb | 615 | 1.180 | 0.5430 | Yes |
| 102 | Inpp5d | 625 | 1.161 | 0.5447 | Yes |
| 103 | Apobec1 | 626 | 1.157 | 0.5485 | Yes |
| 104 | Tcirg1 | 638 | 1.112 | 0.5496 | Yes |
| 105 | Glipr1 | 687 | 1.026 | 0.5425 | Yes |
| 106 | Tmem106a | 690 | 1.016 | 0.5453 | Yes |
| 107 | Fnip2 | 696 | 1.010 | 0.5475 | Yes |
| 108 | Mctp1 | 699 | 1.005 | 0.5503 | Yes |
| 109 | Hck | 700 | 1.005 | 0.5535 | Yes |
| 110 | Ptgs1 | 705 | 0.997 | 0.5558 | Yes |
| 111 | C1qa | 710 | 0.990 | 0.5582 | Yes |
| 112 | Csf1r | 747 | 0.942 | 0.5534 | Yes |
| 113 | Fes | 761 | 0.915 | 0.5535 | Yes |
| 114 | Tmem104 | 762 | 0.915 | 0.5564 | Yes |
| 115 | Ctsc | 770 | 0.907 | 0.5578 | Yes |
| 116 | C1qc | 775 | 0.902 | 0.5598 | Yes |
| 117 | Snx8 | 779 | 0.893 | 0.5621 | Yes |
| 118 | Gm2a | 789 | 0.881 | 0.5629 | Yes |
| 119 | B2m | 794 | 0.876 | 0.5649 | Yes |
| 120 | Lyn | 805 | 0.861 | 0.5655 | Yes |
| 121 | Cd74 | 811 | 0.856 | 0.5671 | Yes |
| 122 | Gbp7 | 835 | 0.828 | 0.5648 | No |
| 123 | Il3ra | 859 | 0.808 | 0.5624 | No |
| 124 | Pstpip2 | 866 | 0.805 | 0.5637 | No |
| 125 | Zc3h12a | 881 | 0.786 | 0.5632 | No |
| 126 | Slc46a3 | 885 | 0.773 | 0.5650 | No |
| 127 | Runx3 | 901 | 0.759 | 0.5642 | No |
| 128 | Osbpl8 | 930 | 0.721 | 0.5604 | No |
| 129 | Dnase2a | 932 | 0.720 | 0.5625 | No |
| 130 | Lpar5 | 946 | 0.709 | 0.5620 | No |
| 131 | Rhog | 961 | 0.693 | 0.5611 | No |
| 132 | P2rx7 | 992 | 0.661 | 0.5567 | No |
| 133 | Pdxk | 994 | 0.660 | 0.5586 | No |
| 134 | Casp1 | 1000 | 0.649 | 0.5596 | No |
| 135 | Dtx3l | 1005 | 0.645 | 0.5609 | No |
| 136 | Ctsa | 1010 | 0.641 | 0.5620 | No |
| 137 | H2-D1 | 1021 | 0.632 | 0.5619 | No |
| 138 | Lsp1 | 1023 | 0.629 | 0.5637 | No |
| 139 | Dab2 | 1031 | 0.623 | 0.5642 | No |
| 140 | Ehd4 | 1053 | 0.607 | 0.5616 | No |
| 141 | Litaf | 1060 | 0.599 | 0.5622 | No |
| 142 | Psmb8 | 1067 | 0.589 | 0.5628 | No |
| 143 | Cfh | 1075 | 0.578 | 0.5631 | No |
| 144 | Nlrc5 | 1099 | 0.555 | 0.5599 | No |
| 145 | Psme2b | 1105 | 0.549 | 0.5606 | No |
| 146 | Grk2 | 1109 | 0.547 | 0.5617 | No |
| 147 | Stk10 | 1111 | 0.545 | 0.5632 | No |
| 148 | Sdcbp | 1122 | 0.536 | 0.5628 | No |
| 149 | Dennd4b | 1130 | 0.528 | 0.5629 | No |
| 150 | Ifnar2 | 1147 | 0.514 | 0.5611 | No |
| 151 | Ifngr1 | 1155 | 0.509 | 0.5612 | No |
| 152 | Tpp1 | 1156 | 0.507 | 0.5629 | No |
| 153 | Arhgap17 | 1163 | 0.504 | 0.5632 | No |
| 154 | Hgsnat | 1193 | -0.504 | 0.5585 | No |
| 155 | Il4ra | 1455 | -0.542 | 0.5035 | No |
| 156 | Ppm1h | 1516 | -0.554 | 0.4923 | No |
| 157 | Rnf166 | 1586 | -0.565 | 0.4791 | No |
| 158 | Epsti1 | 1653 | -0.575 | 0.4666 | No |
| 159 | Fuca1 | 1814 | -0.603 | 0.4338 | No |
| 160 | Madd | 1824 | -0.604 | 0.4338 | No |
| 161 | Tbc1d13 | 1852 | -0.611 | 0.4299 | No |
| 162 | Cracr2b | 2009 | -0.637 | 0.3981 | No |
| 163 | Vwa5a | 2066 | -0.646 | 0.3880 | No |
| 164 | Lrch4 | 2161 | -0.664 | 0.3697 | No |
| 165 | Tmem141 | 2198 | -0.670 | 0.3640 | No |
| 166 | Pepd | 2262 | -0.682 | 0.3525 | No |
| 167 | Lrmda | 2275 | -0.684 | 0.3521 | No |
| 168 | Snx24 | 2289 | -0.686 | 0.3515 | No |
| 169 | Cmtm7 | 2316 | -0.692 | 0.3481 | No |
| 170 | Snx2 | 2321 | -0.693 | 0.3494 | No |
| 171 | Trim65 | 2331 | -0.694 | 0.3497 | No |
| 172 | Tep1 | 2366 | -0.700 | 0.3446 | No |
| 173 | Gas6 | 2372 | -0.701 | 0.3457 | No |
| 174 | Mast3 | 2443 | -0.716 | 0.3328 | No |
| 175 | Shisa5 | 2493 | -0.727 | 0.3245 | No |
| 176 | Fchsd2 | 2537 | -0.734 | 0.3175 | No |
| 177 | Apobec3 | 2698 | -0.767 | 0.2853 | No |
| 178 | Ly6e | 2739 | -0.775 | 0.2791 | No |
| 179 | Tbc1d5 | 2824 | -0.795 | 0.2634 | No |
| 180 | Laptm5 | 2847 | -0.801 | 0.2612 | No |
| 181 | Tmcc3 | 2883 | -0.811 | 0.2562 | No |
| 182 | Abcc3 | 2901 | -0.814 | 0.2551 | No |
| 183 | Plekha2 | 2904 | -0.814 | 0.2573 | No |
| 184 | Pld2 | 3009 | -0.845 | 0.2374 | No |
| 185 | Helz2 | 3044 | -0.853 | 0.2327 | No |
| 186 | H2-M3 | 3047 | -0.854 | 0.2351 | No |
| 187 | Tlr4 | 3127 | -0.878 | 0.2207 | No |
| 188 | Paqr7 | 3205 | -0.901 | 0.2069 | No |
| 189 | Ccdc93 | 3214 | -0.903 | 0.2080 | No |
| 190 | Rnf213 | 3273 | -0.921 | 0.1984 | No |
| 191 | Hint2 | 3347 | -0.946 | 0.1856 | No |
| 192 | Fcgrt | 3361 | -0.950 | 0.1858 | No |
| 193 | Naip2 | 3374 | -0.954 | 0.1863 | No |
| 194 | Asah1 | 3425 | -0.972 | 0.1785 | No |
| 195 | Tcn2 | 3584 | -1.026 | 0.1475 | No |
| 196 | Wwp1 | 3720 | -1.087 | 0.1217 | No |
| 197 | Elf4 | 3767 | -1.109 | 0.1153 | No |
| 198 | Aga | 3908 | -1.189 | 0.0887 | No |
| 199 | Rel | 3944 | -1.213 | 0.0850 | No |
| 200 | Manba | 3972 | -1.227 | 0.0830 | No |
| 201 | Gmfg | 4007 | -1.254 | 0.0797 | No |
| 202 | Sp100 | 4053 | -1.285 | 0.0740 | No |
| 203 | Reps2 | 4226 | -1.440 | 0.0413 | No |
| 204 | Nfatc2 | 4277 | -1.484 | 0.0352 | No |
| 205 | Glb1 | 4287 | -1.494 | 0.0380 | No |
| 206 | Neurl3 | 4307 | -1.511 | 0.0388 | No |
| 207 | Arid5a | 4389 | -1.625 | 0.0264 | No |
| 208 | Selenop | 4397 | -1.636 | 0.0301 | No |
| 209 | Snx6 | 4406 | -1.656 | 0.0337 | No |
| 210 | Pparg | 4408 | -1.658 | 0.0388 | No |
| 211 | Gsdmd | 4501 | -1.815 | 0.0247 | No |
| 212 | Cela1 | 4538 | -1.888 | 0.0229 | No |
| 213 | Dock8 | 4572 | -1.967 | 0.0221 | No |
| 214 | Map3k5 | 4574 | -1.973 | 0.0282 | No |
| 215 | Galnt6 | 4645 | -2.159 | 0.0199 | No |
| 216 | Tlr2 | 4716 | -2.445 | 0.0126 | No |
| 217 | Il18 | 4789 | -3.056 | 0.0067 | No |
Table: GSEA details [plain text format]

  

Fig 2: DESCARTES\_ORGANOGENESIS\_WHITE\_BLOOD\_CELLS: Random ES distribution      
 Gene set null distribution of ES for **DESCARTES\_ORGANOGENESIS\_WHITE\_BLOOD\_CELLS**

  
