# Supplementary material for: TMPRSS11B promotes an acidified microenvironment and immune suppression in squamous lung cancer
Source: EMBO Rep. 2025 Nov 10;26(24):6346–79. doi: 10.1038/s44319-025-00631-1 (PMC12714794; doi:10.1038/s44319-025-00631-1)
Supplement: Supplementary file 10 — Source data Fig. 5 [file 44319_2025_631_MOESM10_ESM.zip › Figure 5/5C-D/GSEA_Broad Institute_M8_T11b-high LUSC vs LUAD/gsea_report_for_na_neg_1723674399702.html]

Report for na\_neg 1723674399702 [GSEA]

| GS  follow link to MSigDB | GS DETAILS | SIZE | ES | NES | NOM p-val | FDR q-val | FWER p-val | RANK AT MAX | LEADING EDGE || 1 | DESCARTES\_ORGANOGENESIS\_HEPATOCYTES | Details ... | 174 | -0.42 | -2.62 | 0.000 | 0.000 | 0.000 | 1084 | tags=48%, list=22%, signal=60% |
| 2 | DESCARTES\_ORGANOGENESIS\_SCHWANN\_CELL\_PRECURSOR | Details ... | 24 | -0.42 | -1.62 | 0.026 | 0.234 | 0.631 | 509 | tags=38%, list=11%, signal=42% |
| 3 | DESCARTES\_ORGANOGENESIS\_EPENDYMAL\_CELL | Details ... | 18 | -0.44 | -1.59 | 0.039 | 0.199 | 0.711 | 809 | tags=56%, list=17%, signal=67% |
| 4 | DESCARTES\_ORGANOGENESIS\_SENSORY\_NEURONS | Details ... | 23 | -0.38 | -1.47 | 0.069 | 0.342 | 0.944 | 662 | tags=35%, list=14%, signal=40% |
| 5 | DESCARTES\_ORGANOGENESIS\_MYOCYTES | Details ... | 18 | -0.41 | -1.47 | 0.077 | 0.278 | 0.945 | 501 | tags=39%, list=10%, signal=43% |
| 6 | TABULA\_MURIS\_SENIS\_PANCREAS\_PANCREATIC\_DUCTAL\_CELL\_AGEING | Details ... | 112 | -0.21 | -1.19 | 0.155 | 1.000 | 1.000 | 1146 | tags=30%, list=24%, signal=39% |
| 7 | TABULA\_MURIS\_SENIS\_LARGE\_INTESTINE\_LARGE\_INTESTINE\_GOBLET\_CELL\_AGEING | Details ... | 255 | -0.17 | -1.16 | 0.151 | 1.000 | 1.000 | 1125 | tags=26%, list=23%, signal=32% |
| 8 | ZHANG\_UTERUS\_C13\_EPITHELIAL1\_CELL | Details ... | 109 | -0.19 | -1.14 | 0.239 | 1.000 | 1.000 | 435 | tags=17%, list=9%, signal=18% |
| 9 | TABULA\_MURIS\_SENIS\_LARGE\_INTESTINE\_SECRETORY\_CELL\_AGEING | Details ... | 251 | -0.17 | -1.10 | 0.235 | 1.000 | 1.000 | 1324 | tags=29%, list=27%, signal=38% |
| 10 | DESCARTES\_ORGANOGENESIS\_CHONDROCYTE\_PROGENITORS | Details ... | 15 | -0.31 | -1.07 | 0.378 | 1.000 | 1.000 | 736 | tags=40%, list=15%, signal=47% |
| 11 | TABULA\_MURIS\_SENIS\_LARGE\_INTESTINE\_ENTEROCYTE\_OF\_EPITHELIUM\_OF\_LARGE\_INTESTINE\_AGEING | Details ... | 355 | -0.15 | -1.04 | 0.355 | 1.000 | 1.000 | 1706 | tags=37%, list=35%, signal=53% |
| 12 | TABULA\_MURIS\_SENIS\_HEART\_ATRIAL\_MYOCYTE\_AGEING | Details ... | 49 | -0.21 | -1.04 | 0.380 | 1.000 | 1.000 | 949 | tags=33%, list=20%, signal=40% |
| 13 | TABULA\_MURIS\_SENIS\_LARGE\_INTESTINE\_INTESTINAL\_CRYPT\_STEM\_CELL\_AGEING | Details ... | 103 | -0.17 | -0.99 | 0.486 | 1.000 | 1.000 | 703 | tags=19%, list=15%, signal=22% |
| 14 | ZHANG\_UTERUS\_C2\_REGENERATIVE\_UP | Details ... | 32 | -0.22 | -0.98 | 0.468 | 1.000 | 1.000 | 622 | tags=22%, list=13%, signal=25% |
| 15 | TABULA\_MURIS\_SENIS\_KIDNEY\_EPITHELIAL\_CELL\_OF\_PROXIMAL\_TUBULE\_AGEING | Details ... | 103 | -0.17 | -0.95 | 0.564 | 1.000 | 1.000 | 1096 | tags=27%, list=23%, signal=34% |
| 16 | TABULA\_MURIS\_SENIS\_PANCREAS\_PANCREATIC\_POLYPEPTIDE\_CELL\_AGEING | Details ... | 38 | -0.20 | -0.94 | 0.555 | 1.000 | 1.000 | 2329 | tags=63%, list=48%, signal=121% |
| 17 | ZHANG\_UTERUS\_C14\_ENDOTHELIAL\_MMRN1\_HIGH\_CELL | Details ... | 17 | -0.26 | -0.91 | 0.580 | 1.000 | 1.000 | 425 | tags=24%, list=9%, signal=26% |
| 18 | DESCARTES\_ORGANOGENESIS\_CARDIAC\_MUSCLE\_LINEAGES | Details ... | 28 | -0.22 | -0.89 | 0.620 | 1.000 | 1.000 | 724 | tags=25%, list=15%, signal=29% |
| 19 | TABULA\_MURIS\_SENIS\_BRAIN\_NON\_MYELOID\_NEURON\_AGEING | Details ... | 287 | -0.13 | -0.87 | 0.844 | 1.000 | 1.000 | 1088 | tags=23%, list=23%, signal=28% |
| 20 | TABULA\_MURIS\_SENIS\_PANCREAS\_PANCREATIC\_ALPHA\_CELL\_AGEING | Details ... | 43 | -0.19 | -0.86 | 0.680 | 1.000 | 1.000 | 825 | tags=23%, list=17%, signal=28% |
| 21 | TABULA\_MURIS\_SENIS\_KIDNEY\_KIDNEY\_COLLECTING\_DUCT\_PRINCIPAL\_CELL\_AGEING |  | 135 | -0.14 | -0.85 | 0.772 | 1.000 | 1.000 | 880 | tags=21%, list=18%, signal=26% |
| 22 | DESCARTES\_ORGANOGENESIS\_STROMAL\_CELLS |  | 47 | -0.16 | -0.80 | 0.777 | 1.000 | 1.000 | 2949 | tags=74%, list=61%, signal=190% |
| 23 | TABULA\_MURIS\_SENIS\_LIVER\_HEPATOCYTE\_AGEING |  | 52 | -0.16 | -0.79 | 0.817 | 1.000 | 1.000 | 868 | tags=23%, list=18%, signal=28% |
| 24 | TABULA\_MURIS\_SENIS\_PANCREAS\_PANCREATIC\_DELTA\_CELL\_AGEING |  | 77 | -0.14 | -0.79 | 0.831 | 1.000 | 1.000 | 1540 | tags=38%, list=32%, signal=54% |
| 25 | TABULA\_MURIS\_SENIS\_HEART\_VALVE\_CELL\_AGEING |  | 42 | -0.15 | -0.70 | 0.870 | 1.000 | 1.000 | 917 | tags=24%, list=19%, signal=29% |
| 26 | DESCARTES\_ORGANOGENESIS\_PRIMITIVE\_ERYTHROID\_LINEAGE |  | 153 | -0.11 | -0.69 | 0.964 | 1.000 | 1.000 | 1254 | tags=27%, list=26%, signal=35% |
| 27 | TABULA\_MURIS\_SENIS\_PANCREAS\_PANCREATIC\_BETA\_CELL\_AGEING |  | 73 | -0.13 | -0.67 | 0.943 | 1.000 | 1.000 | 1284 | tags=26%, list=27%, signal=35% |
| 28 | TABULA\_MURIS\_SENIS\_TRACHEA\_ENDOTHELIAL\_CELL\_AGEING |  | 58 | -0.13 | -0.65 | 0.939 | 1.000 | 1.000 | 271 | tags=7%, list=6%, signal=7% |
| 29 | TABULA\_MURIS\_SENIS\_MAMMARY\_GLAND\_ENDOTHELIAL\_CELL\_AGEING |  | 175 | -0.10 | -0.60 | 1.000 | 1.000 | 1.000 | 1329 | tags=25%, list=28%, signal=33% |
| 30 | TABULA\_MURIS\_SENIS\_HEART\_VENTRICULAR\_MYOCYTE\_AGEING |  | 45 | -0.12 | -0.56 | 0.947 | 1.000 | 1.000 | 756 | tags=16%, list=16%, signal=18% |
| 31 | TABULA\_MURIS\_SENIS\_BROWN\_ADIPOSE\_TISSUE\_MESENCHYMAL\_STEM\_CELL\_OF\_ADIPOSE\_AGEING |  | 375 | -0.06 | -0.42 | 1.000 | 0.998 | 1.000 | 1351 | tags=24%, list=28%, signal=31% |
Table: Gene sets enriched in phenotype **na**[plain text format]****

  
