# Supplementary material for: TMPRSS11B promotes an acidified microenvironment and immune suppression in squamous lung cancer
Source: EMBO Rep. 2025 Nov 10;26(24):6346–79. doi: 10.1038/s44319-025-00631-1 (PMC12714794; doi:10.1038/s44319-025-00631-1)
Supplement: Supplementary file 10 — Source data Fig. 5 [file 44319_2025_631_MOESM10_ESM.zip › Figure 5/5C-D/GSEA_Broad Institute_M8_T11b-high LUSC vs LUAD/gsea_report_for_na_pos_1723674399702.html]

Report for na\_pos 1723674399702 [GSEA]

| GS  follow link to MSigDB | GS DETAILS | SIZE | ES | NES | NOM p-val | FDR q-val | FWER p-val | RANK AT MAX | LEADING EDGE || 1 | ZHANG\_UTERUS\_C12\_MONOCYTE | Details ... | 96 | 0.73 | 3.65 | 0.000 | 0.000 | 0.000 | 1067 | tags=88%, list=22%, signal=110% |
| 2 | ZHANG\_UTERUS\_C5\_MACROPHAGE | Details ... | 86 | 0.70 | 3.40 | 0.000 | 0.000 | 0.000 | 861 | tags=76%, list=18%, signal=90% |
| 3 | DESCARTES\_ORGANOGENESIS\_WHITE\_BLOOD\_CELLS | Details ... | 217 | 0.57 | 3.32 | 0.000 | 0.000 | 0.000 | 811 | tags=56%, list=17%, signal=64% |
| 4 | ZHANG\_UTERUS\_C9\_DENDRITIC\_CELL | Details ... | 43 | 0.75 | 3.17 | 0.000 | 0.000 | 0.000 | 1023 | tags=88%, list=21%, signal=111% |
| 5 | TABULA\_MURIS\_SENIS\_MARROW\_GRANULOCYTOPOIETIC\_CELL\_AGEING | Details ... | 43 | 0.73 | 3.09 | 0.000 | 0.000 | 0.000 | 982 | tags=84%, list=20%, signal=104% |
| 6 | TABULA\_MURIS\_SENIS\_LUNG\_ALVEOLAR\_MACROPHAGE\_AGEING | Details ... | 78 | 0.61 | 3.02 | 0.000 | 0.000 | 0.000 | 1090 | tags=72%, list=23%, signal=91% |
| 7 | TABULA\_MURIS\_SENIS\_SPLEEN\_MACROPHAGE\_AGEING | Details ... | 61 | 0.65 | 2.99 | 0.000 | 0.000 | 0.000 | 833 | tags=64%, list=17%, signal=76% |
| 8 | TABULA\_MURIS\_SENIS\_KIDNEY\_MACROPHAGE\_AGEING | Details ... | 54 | 0.64 | 2.88 | 0.000 | 0.000 | 0.000 | 686 | tags=61%, list=14%, signal=70% |
| 9 | TABULA\_MURIS\_SENIS\_MARROW\_PROMONOCYTE\_AGEING | Details ... | 29 | 0.74 | 2.83 | 0.000 | 0.000 | 0.000 | 499 | tags=62%, list=10%, signal=69% |
| 10 | TABULA\_MURIS\_SENIS\_HEART\_AND\_AORTA\_LEUKOCYTE\_AGEING | Details ... | 45 | 0.64 | 2.79 | 0.000 | 0.000 | 0.000 | 609 | tags=53%, list=13%, signal=60% |
| 11 | ZHANG\_UTERUS\_C10\_STROMAL2\_RETNLG\_HIGH\_CELL | Details ... | 30 | 0.72 | 2.79 | 0.000 | 0.000 | 0.000 | 546 | tags=63%, list=11%, signal=71% |
| 12 | TABULA\_MURIS\_SENIS\_TONGUE\_BASAL\_CELL\_OF\_EPIDERMIS\_AGEING | Details ... | 61 | 0.60 | 2.76 | 0.000 | 0.000 | 0.000 | 1068 | tags=66%, list=22%, signal=83% |
| 13 | TABULA\_MURIS\_SENIS\_MARROW\_HEMATOPOIETIC\_PRECURSOR\_CELL\_AGEING | Details ... | 42 | 0.64 | 2.76 | 0.000 | 0.000 | 0.000 | 1023 | tags=76%, list=21%, signal=96% |
| 14 | TABULA\_MURIS\_SENIS\_MARROW\_NAIVE\_T\_CELL\_AGEING | Details ... | 19 | 0.77 | 2.66 | 0.000 | 0.000 | 0.000 | 489 | tags=68%, list=10%, signal=76% |
| 15 | TABULA\_MURIS\_SENIS\_MARROW\_GRANULOCYTE\_AGEING | Details ... | 28 | 0.71 | 2.66 | 0.000 | 0.000 | 0.000 | 601 | tags=64%, list=12%, signal=73% |
| 16 | TABULA\_MURIS\_SENIS\_LUNG\_INTERMEDIATE\_MONOCYTE\_AGEING | Details ... | 102 | 0.51 | 2.59 | 0.000 | 0.000 | 0.000 | 587 | tags=36%, list=12%, signal=40% |
| 17 | TABULA\_MURIS\_SENIS\_AORTA\_PROFESSIONAL\_ANTIGEN\_PRESENTING\_CELL\_AGEING | Details ... | 50 | 0.57 | 2.50 | 0.000 | 0.000 | 0.000 | 887 | tags=60%, list=18%, signal=73% |
| 18 | TABULA\_MURIS\_SENIS\_SPLEEN\_B\_CELL\_AGEING | Details ... | 37 | 0.60 | 2.50 | 0.000 | 0.000 | 0.000 | 822 | tags=62%, list=17%, signal=74% |
| 19 | TABULA\_MURIS\_SENIS\_TONGUE\_KERATINOCYTE\_AGEING | Details ... | 20 | 0.70 | 2.48 | 0.000 | 0.000 | 0.000 | 488 | tags=45%, list=10%, signal=50% |
| 20 | TABULA\_MURIS\_SENIS\_LUNG\_NON\_CLASSICAL\_MONOCYTE\_AGEING | Details ... | 18 | 0.72 | 2.47 | 0.000 | 0.000 | 0.000 | 811 | tags=78%, list=17%, signal=93% |
| 21 | TABULA\_MURIS\_SENIS\_BRAIN\_MYELOID\_MICROGLIAL\_CELL\_AGEING |  | 105 | 0.48 | 2.47 | 0.000 | 0.000 | 0.000 | 861 | tags=47%, list=18%, signal=56% |
| 22 | TABULA\_MURIS\_SENIS\_MARROW\_MACROPHAGE\_AGEING |  | 54 | 0.53 | 2.39 | 0.000 | 0.000 | 0.001 | 770 | tags=46%, list=16%, signal=54% |
| 23 | TABULA\_MURIS\_SENIS\_MAMMARY\_GLAND\_MACROPHAGE\_AGEING |  | 36 | 0.57 | 2.35 | 0.000 | 0.000 | 0.001 | 601 | tags=42%, list=12%, signal=47% |
| 24 | TABULA\_MURIS\_SENIS\_MARROW\_PRECURSOR\_B\_CELL\_AGEING |  | 32 | 0.60 | 2.35 | 0.000 | 0.000 | 0.001 | 871 | tags=59%, list=18%, signal=72% |
| 25 | ZHANG\_UTERUS\_C8\_NK\_CELL |  | 47 | 0.52 | 2.29 | 0.000 | 0.000 | 0.001 | 1067 | tags=60%, list=22%, signal=76% |
| 26 | TABULA\_MURIS\_SENIS\_SPLEEN\_T\_CELL\_AGEING |  | 88 | 0.46 | 2.29 | 0.000 | 0.000 | 0.001 | 1129 | tags=59%, list=23%, signal=76% |
| 27 | TABULA\_MURIS\_SENIS\_TRACHEA\_FIBROBLAST\_AGEING |  | 33 | 0.57 | 2.28 | 0.000 | 0.000 | 0.001 | 861 | tags=52%, list=18%, signal=62% |
| 28 | TABULA\_MURIS\_SENIS\_SPLEEN\_CD4\_POSITIVE\_ALPHA\_BETA\_T\_CELL\_AGEING |  | 65 | 0.49 | 2.26 | 0.000 | 0.000 | 0.004 | 682 | tags=40%, list=14%, signal=46% |
| 29 | TABULA\_MURIS\_SENIS\_SUBCUTANEOUS\_ADIPOSE\_TISSUE\_MYELOID\_CELL\_AGEING |  | 93 | 0.45 | 2.25 | 0.000 | 0.000 | 0.005 | 1127 | tags=55%, list=23%, signal=70% |
| 30 | TABULA\_MURIS\_SENIS\_MARROW\_MONOCYTE\_AGEING |  | 18 | 0.67 | 2.25 | 0.000 | 0.000 | 0.005 | 811 | tags=67%, list=17%, signal=80% |
| 31 | TABULA\_MURIS\_SENIS\_BLADDER\_BLADDER\_UROTHELIAL\_CELL\_AGEING |  | 68 | 0.47 | 2.25 | 0.000 | 0.000 | 0.006 | 1119 | tags=59%, list=23%, signal=76% |
| 32 | TABULA\_MURIS\_SENIS\_BLADDER\_BLADDER\_CELL\_AGEING |  | 114 | 0.44 | 2.24 | 0.000 | 0.000 | 0.006 | 1126 | tags=54%, list=23%, signal=68% |
| 33 | TABULA\_MURIS\_SENIS\_LIMB\_MUSCLE\_MACROPHAGE\_AGEING |  | 38 | 0.54 | 2.24 | 0.000 | 0.000 | 0.006 | 1067 | tags=68%, list=22%, signal=87% |
| 34 | TABULA\_MURIS\_SENIS\_MARROW\_PROERYTHROBLAST\_AGEING |  | 16 | 0.67 | 2.19 | 0.000 | 0.001 | 0.016 | 794 | tags=69%, list=16%, signal=82% |
| 35 | TABULA\_MURIS\_SENIS\_SPLEEN\_CD8\_POSITIVE\_ALPHA\_BETA\_T\_CELL\_AGEING |  | 55 | 0.49 | 2.19 | 0.000 | 0.001 | 0.017 | 1021 | tags=55%, list=21%, signal=68% |
| 36 | TABULA\_MURIS\_SENIS\_LUNG\_CD4\_POSITIVE\_ALPHA\_BETA\_T\_CELL\_AGEING |  | 52 | 0.49 | 2.18 | 0.000 | 0.001 | 0.019 | 1090 | tags=58%, list=23%, signal=74% |
| 37 | TABULA\_MURIS\_SENIS\_GONADAL\_ADIPOSE\_TISSUE\_MYELOID\_CELL\_AGEING |  | 105 | 0.43 | 2.18 | 0.000 | 0.001 | 0.023 | 887 | tags=41%, list=18%, signal=49% |
| 38 | TABULA\_MURIS\_SENIS\_GONADAL\_ADIPOSE\_TISSUE\_B\_CELL\_AGEING |  | 28 | 0.57 | 2.15 | 0.000 | 0.001 | 0.027 | 490 | tags=36%, list=10%, signal=40% |
| 39 | TABULA\_MURIS\_SENIS\_MARROW\_ERYTHROBLAST\_AGEING |  | 32 | 0.54 | 2.14 | 0.000 | 0.001 | 0.028 | 388 | tags=34%, list=8%, signal=37% |
| 40 | TABULA\_MURIS\_SENIS\_SUBCUTANEOUS\_ADIPOSE\_TISSUE\_B\_CELL\_AGEING |  | 53 | 0.48 | 2.12 | 0.000 | 0.001 | 0.034 | 726 | tags=43%, list=15%, signal=51% |
| 41 | TABULA\_MURIS\_SENIS\_MAMMARY\_GLAND\_T\_CELL\_AGEING |  | 159 | 0.39 | 2.12 | 0.000 | 0.001 | 0.035 | 1023 | tags=45%, list=21%, signal=55% |
| 42 | TABULA\_MURIS\_SENIS\_MAMMARY\_GLAND\_LUMINAL\_EPITHELIAL\_CELL\_OF\_MAMMARY\_GLAND\_AGEING |  | 159 | 0.38 | 2.09 | 0.000 | 0.002 | 0.049 | 827 | tags=35%, list=17%, signal=40% |
| 43 | TABULA\_MURIS\_SENIS\_MAMMARY\_GLAND\_B\_CELL\_AGEING |  | 91 | 0.42 | 2.08 | 0.000 | 0.002 | 0.051 | 1090 | tags=52%, list=23%, signal=65% |
| 44 | TABULA\_MURIS\_SENIS\_HEART\_MONOCYTE\_AGEING |  | 48 | 0.48 | 2.08 | 0.000 | 0.002 | 0.051 | 710 | tags=42%, list=15%, signal=48% |
| 45 | TABULA\_MURIS\_SENIS\_LIMB\_MUSCLE\_MESENCHYMAL\_STEM\_CELL\_AGEING |  | 75 | 0.43 | 2.07 | 0.000 | 0.002 | 0.060 | 887 | tags=43%, list=18%, signal=51% |
| 46 | TABULA\_MURIS\_SENIS\_LIMB\_MUSCLE\_T\_CELL\_AGEING |  | 83 | 0.42 | 2.05 | 0.000 | 0.002 | 0.070 | 1043 | tags=51%, list=22%, signal=63% |
| 47 | DESCARTES\_ORGANOGENESIS\_EPITHELIAL\_CELLS |  | 80 | 0.41 | 1.99 | 0.000 | 0.003 | 0.104 | 564 | tags=34%, list=12%, signal=38% |
| 48 | TABULA\_MURIS\_SENIS\_LUNG\_B\_CELL\_AGEING |  | 34 | 0.48 | 1.96 | 0.002 | 0.004 | 0.130 | 1126 | tags=65%, list=23%, signal=84% |
| 49 | TABULA\_MURIS\_SENIS\_LUNG\_CLASSICAL\_MONOCYTE\_AGEING |  | 86 | 0.40 | 1.96 | 0.002 | 0.004 | 0.135 | 976 | tags=38%, list=20%, signal=47% |
| 50 | TABULA\_MURIS\_SENIS\_MARROW\_IMMATURE\_B\_CELL\_AGEING |  | 54 | 0.43 | 1.93 | 0.005 | 0.005 | 0.178 | 1264 | tags=57%, list=26%, signal=77% |
| 51 | TABULA\_MURIS\_SENIS\_KIDNEY\_T\_CELL\_AGEING |  | 20 | 0.54 | 1.90 | 0.010 | 0.006 | 0.216 | 1067 | tags=70%, list=22%, signal=90% |
| 52 | ZHANG\_UTERUS\_C1\_PROLIFERATIVE\_STROMAL1\_MGP\_HIGH\_CELL |  | 101 | 0.37 | 1.89 | 0.000 | 0.007 | 0.231 | 607 | tags=32%, list=13%, signal=35% |
| 53 | TABULA\_MURIS\_SENIS\_SUBCUTANEOUS\_ADIPOSE\_TISSUE\_ENDOTHELIAL\_CELL\_AGEING |  | 65 | 0.40 | 1.89 | 0.004 | 0.007 | 0.232 | 828 | tags=35%, list=17%, signal=42% |
| 54 | ZHANG\_UTERUS\_C3\_PROLIFERATIVE\_STROMAL1\_STROCXCL14\_HIGH\_CELL |  | 22 | 0.51 | 1.89 | 0.008 | 0.006 | 0.232 | 1092 | tags=64%, list=23%, signal=82% |
| 55 | TABULA\_MURIS\_SENIS\_MARROW\_NAIVE\_B\_CELL\_AGEING |  | 87 | 0.38 | 1.89 | 0.000 | 0.006 | 0.235 | 1126 | tags=47%, list=23%, signal=60% |
| 56 | DESCARTES\_ORGANOGENESIS\_MELANOCYTES |  | 19 | 0.54 | 1.86 | 0.008 | 0.008 | 0.288 | 760 | tags=58%, list=16%, signal=68% |
| 57 | TABULA\_MURIS\_SENIS\_MAMMARY\_GLAND\_BASAL\_CELL\_AGEING |  | 141 | 0.34 | 1.84 | 0.000 | 0.009 | 0.333 | 861 | tags=35%, list=18%, signal=41% |
| 58 | TABULA\_MURIS\_SENIS\_MESENTERIC\_ADIPOSE\_TISSUE\_MACROPHAGE\_AGEING |  | 21 | 0.53 | 1.84 | 0.015 | 0.009 | 0.341 | 537 | tags=48%, list=11%, signal=53% |
| 59 | ZHANG\_UTERUS\_C1\_REGENERATIVE\_UP |  | 124 | 0.34 | 1.82 | 0.000 | 0.010 | 0.367 | 887 | tags=41%, list=18%, signal=49% |
| 60 | TABULA\_MURIS\_SENIS\_LIVER\_NK\_CELL\_AGEING |  | 80 | 0.36 | 1.80 | 0.006 | 0.012 | 0.426 | 1326 | tags=56%, list=28%, signal=76% |
| 61 | TABULA\_MURIS\_SENIS\_HEART\_ENDOTHELIAL\_CELL\_OF\_CORONARY\_ARTERY\_AGEING |  | 49 | 0.40 | 1.79 | 0.003 | 0.013 | 0.446 | 1021 | tags=51%, list=21%, signal=64% |
| 62 | TABULA\_MURIS\_SENIS\_LIVER\_ENDOTHELIAL\_CELL\_OF\_HEPATIC\_SINUSOID\_AGEING |  | 129 | 0.33 | 1.78 | 0.008 | 0.013 | 0.465 | 976 | tags=39%, list=20%, signal=47% |
| 63 | TABULA\_MURIS\_SENIS\_BLADDER\_ENDOTHELIAL\_CELL\_AGEING |  | 161 | 0.31 | 1.74 | 0.001 | 0.019 | 0.595 | 1168 | tags=45%, list=24%, signal=58% |
| 64 | TABULA\_MURIS\_SENIS\_SKIN\_EPIDERMAL\_CELL\_AGEING |  | 225 | 0.30 | 1.74 | 0.000 | 0.019 | 0.596 | 840 | tags=27%, list=17%, signal=31% |
| 65 | TABULA\_MURIS\_SENIS\_SUBCUTANEOUS\_ADIPOSE\_TISSUE\_MESENCHYMAL\_STEM\_CELL\_OF\_ADIPOSE\_AGEING |  | 100 | 0.34 | 1.74 | 0.003 | 0.019 | 0.601 | 861 | tags=37%, list=18%, signal=44% |
| 66 | TABULA\_MURIS\_SENIS\_SPLEEN\_MATURE\_NK\_T\_CELL\_AGEING |  | 20 | 0.51 | 1.73 | 0.022 | 0.019 | 0.609 | 537 | tags=40%, list=11%, signal=45% |
| 67 | DESCARTES\_ORGANOGENESIS\_MEGAKARYOCYTES |  | 46 | 0.40 | 1.73 | 0.014 | 0.019 | 0.612 | 952 | tags=43%, list=20%, signal=54% |
| 68 | TABULA\_MURIS\_SENIS\_MESENTERIC\_ADIPOSE\_TISSUE\_B\_CELL\_AGEING |  | 26 | 0.47 | 1.72 | 0.028 | 0.020 | 0.635 | 811 | tags=42%, list=17%, signal=51% |
| 69 | TABULA\_MURIS\_SENIS\_BROWN\_ADIPOSE\_TISSUE\_T\_CELL\_AGEING |  | 35 | 0.42 | 1.70 | 0.011 | 0.022 | 0.678 | 607 | tags=37%, list=13%, signal=42% |
| 70 | TABULA\_MURIS\_SENIS\_LIMB\_MUSCLE\_B\_CELL\_AGEING |  | 70 | 0.35 | 1.68 | 0.018 | 0.025 | 0.737 | 1090 | tags=46%, list=23%, signal=58% |
| 71 | TABULA\_MURIS\_SENIS\_THYMUS\_DN4\_THYMOCYTE\_AGEING |  | 60 | 0.36 | 1.66 | 0.012 | 0.029 | 0.776 | 901 | tags=38%, list=19%, signal=47% |
| 72 | TABULA\_MURIS\_SENIS\_HEART\_AND\_AORTA\_FIBROBLAST\_OF\_CARDIAC\_TISSUE\_AGEING |  | 96 | 0.32 | 1.65 | 0.006 | 0.031 | 0.804 | 1115 | tags=48%, list=23%, signal=61% |
| 73 | ZHANG\_UTERUS\_C4\_MYOFIBROBLAST |  | 151 | 0.29 | 1.61 | 0.006 | 0.038 | 0.869 | 1112 | tags=41%, list=23%, signal=52% |
| 74 | TABULA\_MURIS\_SENIS\_LIMB\_MUSCLE\_SKELETAL\_MUSCLE\_SATELLITE\_CELL\_AGEING |  | 38 | 0.39 | 1.59 | 0.033 | 0.045 | 0.910 | 1326 | tags=58%, list=28%, signal=79% |
| 75 | ZHANG\_UTERUS\_C15\_B\_CELL |  | 20 | 0.46 | 1.58 | 0.047 | 0.045 | 0.918 | 708 | tags=45%, list=15%, signal=53% |
| 76 | TABULA\_MURIS\_SENIS\_MESENTERIC\_ADIPOSE\_TISSUE\_CD4\_POSITIVE\_ALPHA\_BETA\_T\_CELL\_AGEING |  | 30 | 0.40 | 1.58 | 0.050 | 0.046 | 0.927 | 887 | tags=40%, list=18%, signal=49% |
| 77 | TABULA\_MURIS\_SENIS\_GONADAL\_ADIPOSE\_TISSUE\_MESENCHYMAL\_STEM\_CELL\_OF\_ADIPOSE\_AGEING |  | 231 | 0.27 | 1.54 | 0.007 | 0.060 | 0.963 | 871 | tags=28%, list=18%, signal=33% |
| 78 | TABULA\_MURIS\_SENIS\_LUNG\_NK\_CELL\_AGEING |  | 15 | 0.47 | 1.51 | 0.081 | 0.068 | 0.978 | 1067 | tags=60%, list=22%, signal=77% |
| 79 | TABULA\_MURIS\_SENIS\_SKIN\_BASAL\_CELL\_OF\_EPIDERMIS\_AGEING |  | 303 | 0.25 | 1.51 | 0.001 | 0.069 | 0.980 | 897 | tags=26%, list=19%, signal=30% |
| 80 | TABULA\_MURIS\_SENIS\_LUNG\_FIBROBLAST\_OF\_LUNG\_AGEING |  | 94 | 0.30 | 1.51 | 0.021 | 0.068 | 0.980 | 979 | tags=35%, list=20%, signal=43% |
| 81 | TABULA\_MURIS\_SENIS\_HEART\_FIBROBLAST\_OF\_CARDIAC\_TISSUE\_AGEING |  | 88 | 0.30 | 1.49 | 0.032 | 0.076 | 0.992 | 833 | tags=38%, list=17%, signal=45% |
| 82 | TABULA\_MURIS\_SENIS\_KIDNEY\_FENESTRATED\_CELL\_AGEING |  | 33 | 0.37 | 1.47 | 0.066 | 0.083 | 0.994 | 811 | tags=36%, list=17%, signal=43% |
| 83 | ZHANG\_UTERUS\_C2\_SECRETORY\_STROMAL3\_RAMP3\_HIGH\_CELL |  | 75 | 0.30 | 1.46 | 0.046 | 0.085 | 0.995 | 587 | tags=27%, list=12%, signal=30% |
| 84 | TABULA\_MURIS\_SENIS\_BROWN\_ADIPOSE\_TISSUE\_B\_CELL\_AGEING |  | 121 | 0.28 | 1.43 | 0.052 | 0.099 | 0.999 | 876 | tags=31%, list=18%, signal=37% |
| 85 | TABULA\_MURIS\_SENIS\_TRACHEA\_MACROPHAGE\_AGEING |  | 19 | 0.41 | 1.41 | 0.112 | 0.112 | 1.000 | 751 | tags=37%, list=16%, signal=43% |
| 86 | ZHANG\_UTERUS\_C6\_ENDOTHELIAL\_PLVAP\_HIGH\_CELL |  | 57 | 0.31 | 1.40 | 0.094 | 0.121 | 1.000 | 1326 | tags=56%, list=28%, signal=77% |
| 87 | ZHANG\_UTERUS\_C0\_SECRETORY\_STROMAL3\_NPPC\_HIGH\_CELL |  | 123 | 0.26 | 1.37 | 0.050 | 0.135 | 1.000 | 1112 | tags=40%, list=23%, signal=50% |
| 88 | TABULA\_MURIS\_SENIS\_PANCREAS\_LEUKOCYTE\_AGEING |  | 15 | 0.43 | 1.35 | 0.139 | 0.150 | 1.000 | 643 | tags=40%, list=13%, signal=46% |
| 89 | TABULA\_MURIS\_SENIS\_LIVER\_MYELOID\_LEUKOCYTE\_AGEING |  | 16 | 0.41 | 1.33 | 0.158 | 0.164 | 1.000 | 1127 | tags=63%, list=23%, signal=81% |
| 90 | TABULA\_MURIS\_SENIS\_MARROW\_NK\_CELL\_AGEING |  | 149 | 0.24 | 1.32 | 0.079 | 0.174 | 1.000 | 822 | tags=23%, list=17%, signal=27% |
| 91 | TABULA\_MURIS\_SENIS\_MARROW\_LATE\_PRO\_B\_CELL\_AGEING |  | 62 | 0.28 | 1.30 | 0.148 | 0.189 | 1.000 | 1270 | tags=48%, list=26%, signal=65% |
| 92 | TABULA\_MURIS\_SENIS\_DIAPHRAGM\_SKELETAL\_MUSCLE\_SATELLITE\_CELL\_AGEING |  | 48 | 0.29 | 1.28 | 0.174 | 0.209 | 1.000 | 997 | tags=38%, list=21%, signal=47% |
| 93 | TABULA\_MURIS\_SENIS\_BROWN\_ADIPOSE\_TISSUE\_MYELOID\_CELL\_AGEING |  | 91 | 0.25 | 1.27 | 0.150 | 0.220 | 1.000 | 811 | tags=27%, list=17%, signal=32% |
| 94 | TABULA\_MURIS\_SENIS\_AORTA\_AORTIC\_ENDOTHELIAL\_CELL\_AGEING |  | 104 | 0.25 | 1.26 | 0.135 | 0.226 | 1.000 | 834 | tags=28%, list=17%, signal=33% |
| 95 | DESCARTES\_ORGANOGENESIS\_ENDOTHELIAL\_CELLS |  | 83 | 0.26 | 1.26 | 0.148 | 0.225 | 1.000 | 1118 | tags=45%, list=23%, signal=57% |
| 96 | TABULA\_MURIS\_SENIS\_LIMB\_MUSCLE\_ENDOTHELIAL\_CELL\_AGEING |  | 50 | 0.28 | 1.24 | 0.189 | 0.245 | 1.000 | 1067 | tags=50%, list=22%, signal=64% |
| 97 | TABULA\_MURIS\_SENIS\_KIDNEY\_KIDNEY\_MESANGIAL\_CELL\_AGEING |  | 17 | 0.36 | 1.22 | 0.247 | 0.264 | 1.000 | 1021 | tags=53%, list=21%, signal=67% |
| 98 | TABULA\_MURIS\_SENIS\_HEART\_AND\_AORTA\_ENDOTHELIAL\_CELL\_OF\_CORONARY\_ARTERY\_AGEING |  | 98 | 0.24 | 1.21 | 0.201 | 0.279 | 1.000 | 1118 | tags=42%, list=23%, signal=53% |
| 99 | ZHANG\_UTERUS\_C7\_EPITHELIAL2\_CELL |  | 217 | 0.21 | 1.20 | 0.170 | 0.278 | 1.000 | 971 | tags=28%, list=20%, signal=34% |
| 100 | TABULA\_MURIS\_SENIS\_KIDNEY\_KIDNEY\_LOOP\_OF\_HENLE\_THICK\_ASCENDING\_LIMB\_EPITHELIAL\_CELL\_AGEING |  | 74 | 0.24 | 1.16 | 0.245 | 0.339 | 1.000 | 1029 | tags=38%, list=21%, signal=47% |
| 101 | TABULA\_MURIS\_SENIS\_SPLEEN\_NK\_CELL\_AGEING |  | 16 | 0.35 | 1.11 | 0.312 | 0.404 | 1.000 | 532 | tags=31%, list=11%, signal=35% |
| 102 | TABULA\_MURIS\_SENIS\_SPLEEN\_MEGAKARYOCYTE\_ERYTHROID\_PROGENITOR\_CELL\_AGEING |  | 21 | 0.32 | 1.10 | 0.346 | 0.416 | 1.000 | 1171 | tags=62%, list=24%, signal=81% |
| 103 | TABULA\_MURIS\_SENIS\_LUNG\_ADVENTITIAL\_CELL\_AGEING |  | 68 | 0.24 | 1.10 | 0.316 | 0.417 | 1.000 | 1120 | tags=37%, list=23%, signal=47% |
| 104 | TABULA\_MURIS\_SENIS\_KIDNEY\_PODOCYTE\_AGEING |  | 72 | 0.23 | 1.08 | 0.328 | 0.443 | 1.000 | 855 | tags=33%, list=18%, signal=40% |
| 105 | TABULA\_MURIS\_SENIS\_LIVER\_MATURE\_NK\_T\_CELL\_AGEING |  | 38 | 0.26 | 1.08 | 0.341 | 0.449 | 1.000 | 1904 | tags=63%, list=39%, signal=104% |
| 106 | TABULA\_MURIS\_SENIS\_LUNG\_BRONCHIAL\_SMOOTH\_MUSCLE\_CELL\_AGEING |  | 56 | 0.24 | 1.07 | 0.361 | 0.458 | 1.000 | 1072 | tags=46%, list=22%, signal=59% |
| 107 | TABULA\_MURIS\_SENIS\_MAMMARY\_GLAND\_STROMAL\_CELL\_AGEING |  | 123 | 0.19 | 1.03 | 0.398 | 0.515 | 1.000 | 607 | tags=18%, list=13%, signal=20% |
| 108 | TABULA\_MURIS\_SENIS\_SPLEEN\_GRANULOCYTE\_AGEING |  | 56 | 0.23 | 1.02 | 0.412 | 0.528 | 1.000 | 1293 | tags=43%, list=27%, signal=58% |
| 109 | TABULA\_MURIS\_SENIS\_SPLEEN\_PROERYTHROBLAST\_AGEING |  | 262 | 0.17 | 1.02 | 0.413 | 0.532 | 1.000 | 3148 | tags=86%, list=65%, signal=235% |
| 110 | TABULA\_MURIS\_SENIS\_KIDNEY\_KIDNEY\_DISTAL\_CONVOLUTED\_TUBULE\_EPITHELIAL\_CELL\_AGEING |  | 31 | 0.25 | 0.99 | 0.463 | 0.576 | 1.000 | 827 | tags=32%, list=17%, signal=39% |
| 111 | TABULA\_MURIS\_SENIS\_DIAPHRAGM\_MESENCHYMAL\_STEM\_CELL\_AGEING |  | 87 | 0.19 | 0.95 | 0.532 | 0.653 | 1.000 | 1326 | tags=45%, list=28%, signal=61% |
| 112 | TABULA\_MURIS\_SENIS\_KIDNEY\_BRUSH\_CELL\_AGEING |  | 18 | 0.28 | 0.95 | 0.514 | 0.654 | 1.000 | 811 | tags=33%, list=17%, signal=40% |
| 113 | TABULA\_MURIS\_SENIS\_SKIN\_BULGE\_KERATINOCYTE\_AGEING |  | 277 | 0.16 | 0.94 | 0.600 | 0.670 | 1.000 | 846 | tags=21%, list=18%, signal=24% |
| 114 | TABULA\_MURIS\_SENIS\_KIDNEY\_KIDNEY\_PROXIMAL\_CONVOLUTED\_TUBULE\_EPITHELIAL\_CELL\_AGEING |  | 55 | 0.20 | 0.93 | 0.547 | 0.667 | 1.000 | 819 | tags=31%, list=17%, signal=37% |
| 115 | TABULA\_MURIS\_SENIS\_SUBCUTANEOUS\_ADIPOSE\_TISSUE\_EPITHELIAL\_CELL\_AGEING |  | 221 | 0.16 | 0.90 | 0.669 | 0.732 | 1.000 | 922 | tags=24%, list=19%, signal=28% |
| 116 | TABULA\_MURIS\_SENIS\_MESENTERIC\_ADIPOSE\_TISSUE\_MESENCHYMAL\_STEM\_CELL\_OF\_ADIPOSE\_AGEING |  | 226 | 0.15 | 0.86 | 0.745 | 0.805 | 1.000 | 1021 | tags=28%, list=21%, signal=34% |
| 117 | TABULA\_MURIS\_SENIS\_MARROW\_GRANULOCYTE\_MONOCYTE\_PROGENITOR\_CELL\_AGEING |  | 61 | 0.18 | 0.82 | 0.718 | 0.858 | 1.000 | 2764 | tags=80%, list=57%, signal=186% |
| 118 | TABULA\_MURIS\_SENIS\_BRAIN\_NON\_MYELOID\_ASTROCYTE\_AGEING |  | 158 | 0.15 | 0.81 | 0.810 | 0.866 | 1.000 | 846 | tags=22%, list=18%, signal=25% |
| 119 | TABULA\_MURIS\_SENIS\_THYMUS\_THYMOCYTE\_AGEING |  | 126 | 0.15 | 0.79 | 0.820 | 0.893 | 1.000 | 3301 | tags=88%, list=68%, signal=272% |
| 120 | TABULA\_MURIS\_SENIS\_HEART\_AND\_AORTA\_SMOOTH\_MUSCLE\_CELL\_AGEING |  | 25 | 0.22 | 0.79 | 0.711 | 0.886 | 1.000 | 1021 | tags=40%, list=21%, signal=50% |
| 121 | TABULA\_MURIS\_SENIS\_BRAIN\_NON\_MYELOID\_BRAIN\_PERICYTE\_AGEING |  | 229 | 0.13 | 0.76 | 0.916 | 0.923 | 1.000 | 861 | tags=20%, list=18%, signal=23% |
| 122 | TABULA\_MURIS\_SENIS\_GONADAL\_ADIPOSE\_TISSUE\_ENDOTHELIAL\_CELL\_AGEING |  | 342 | 0.13 | 0.76 | 0.941 | 0.916 | 1.000 | 902 | tags=18%, list=19%, signal=21% |
| 123 | TABULA\_MURIS\_SENIS\_BRAIN\_NON\_MYELOID\_OLIGODENDROCYTE\_PRECURSOR\_CELL\_AGEING |  | 130 | 0.14 | 0.74 | 0.883 | 0.933 | 1.000 | 682 | tags=15%, list=14%, signal=17% |
| 124 | TABULA\_MURIS\_SENIS\_HEART\_ENDOCARDIAL\_CELL\_AGEING |  | 20 | 0.21 | 0.73 | 0.765 | 0.933 | 1.000 | 1047 | tags=35%, list=22%, signal=45% |
| 125 | TABULA\_MURIS\_SENIS\_MARROW\_MATURE\_ALPHA\_BETA\_T\_CELL\_AGEING |  | 169 | 0.13 | 0.72 | 0.917 | 0.937 | 1.000 | 1744 | tags=50%, list=36%, signal=76% |
| 126 | TABULA\_MURIS\_SENIS\_LIMB\_MUSCLE\_SMOOTH\_MUSCLE\_CELL\_AGEING |  | 32 | 0.18 | 0.69 | 0.826 | 0.960 | 1.000 | 1075 | tags=41%, list=22%, signal=52% |
| 127 | TABULA\_MURIS\_SENIS\_AORTA\_FIBROBLAST\_OF\_CARDIAC\_TISSUE\_AGEING |  | 251 | 0.12 | 0.68 | 0.975 | 0.960 | 1.000 | 966 | tags=22%, list=20%, signal=26% |
| 128 | TABULA\_MURIS\_SENIS\_MARROW\_HEMATOPOIETIC\_STEM\_CELL\_AGEING |  | 219 | 0.11 | 0.62 | 0.993 | 0.996 | 1.000 | 1327 | tags=34%, list=28%, signal=45% |
| 129 | TABULA\_MURIS\_SENIS\_BROWN\_ADIPOSE\_TISSUE\_ENDOTHELIAL\_CELL\_AGEING |  | 74 | 0.13 | 0.62 | 0.948 | 0.991 | 1.000 | 800 | tags=20%, list=17%, signal=24% |
| 130 | TABULA\_MURIS\_SENIS\_HEART\_AND\_AORTA\_CARDIOMYOCYTE\_AGEING |  | 19 | 0.18 | 0.61 | 0.904 | 0.990 | 1.000 | 465 | tags=16%, list=10%, signal=17% |
| 131 | TABULA\_MURIS\_SENIS\_BRAIN\_NON\_MYELOID\_ENDOTHELIAL\_CELL\_AGEING |  | 108 | 0.11 | 0.57 | 0.981 | 0.998 | 1.000 | 1302 | tags=36%, list=27%, signal=48% |
| 132 | TABULA\_MURIS\_SENIS\_MESENTERIC\_ADIPOSE\_TISSUE\_ENDOTHELIAL\_CELL\_AGEING |  | 30 | 0.12 | 0.47 | 0.975 | 1.000 | 1.000 | 1844 | tags=57%, list=38%, signal=91% |
| 133 | TABULA\_MURIS\_SENIS\_PANCREAS\_ENDOTHELIAL\_CELL\_AGEING |  | 88 | 0.09 | 0.45 | 0.998 | 1.000 | 1.000 | 155 | tags=3%, list=3%, signal=3% |
| 134 | TABULA\_MURIS\_SENIS\_TRACHEA\_T\_CELL\_AGEING |  | 137 | 0.08 | 0.45 | 1.000 | 1.000 | 1.000 | 3915 | tags=94%, list=81%, signal=487% |
| 135 | TABULA\_MURIS\_SENIS\_BRAIN\_NON\_MYELOID\_OLIGODENDROCYTE\_AGEING |  | 331 | 0.06 | 0.38 | 1.000 | 0.999 | 1.000 | 4215 | tags=97%, list=87%, signal=721% |
Table: Gene sets enriched in phenotype **na**[plain text format]****

  
