# Supplementary material for: TMPRSS11B promotes an acidified microenvironment and immune suppression in squamous lung cancer
Source: EMBO Rep. 2025 Nov 10;26(24):6346–79. doi: 10.1038/s44319-025-00631-1 (PMC12714794; doi:10.1038/s44319-025-00631-1)
Supplement: Supplementary file 10 — Source data Fig. 5 [file 44319_2025_631_MOESM10_ESM.zip › Figure 5/5C-D/GSEA_Broad Institute_M8_T11b-high LUSC vs LUAD/TABULA_MURIS_SENIS_AORTA_PROFESSIONAL_ANTIGEN_PRESENTING_CELL_AGEING.html]

Details for gene set TABULA\_MURIS\_SENIS\_AORTA\_PROFESSIONAL\_ANTIGEN\_PRESENTING\_CELL\_AGEING[GSEA]

|  || Dataset | Ranked list\_DGE\_squamousT11b\_vs\_all adenosadeno\_HSE13-NT copy |
| Phenotype | NoPhenotypeAvailable |
| Upregulated in class | na\_pos |
| GeneSet | TABULA\_MURIS\_SENIS\_AORTA\_PROFESSIONAL\_ANTIGEN\_PRESENTING\_CELL\_AGEING |
| Enrichment Score (ES) | 0.567125 |
| Normalized Enrichment Score (NES) | 2.5049465 |
| Nominal p-value | 0.0 |
| FDR q-value | 0.0 |
| FWER p-Value | 0.0 |
Table: GSEA Results Summary

  

Fig 1: Enrichment plot: TABULA\_MURIS\_SENIS\_AORTA\_PROFESSIONAL\_ANTIGEN\_PRESENTING\_CELL\_AGEING      
 Profile of the Running ES Score & Positions of GeneSet Members on the Rank Ordered List

  

| SYMBOL | RANK IN GENE LIST | RANK METRIC SCORE | RUNNING ES | CORE ENRICHMENT || 1 | Krt14 | 31 | 5.553 | 0.0756 | Yes |
| 2 | Cd68 | 177 | 2.764 | 0.0860 | Yes |
| 3 | Tyrobp | 181 | 2.732 | 0.1258 | Yes |
| 4 | Srgn | 185 | 2.715 | 0.1653 | Yes |
| 5 | Wfdc17 | 241 | 2.337 | 0.1883 | Yes |
| 6 | Ctss | 247 | 2.317 | 0.2215 | Yes |
| 7 | Fcer1g | 272 | 2.235 | 0.2495 | Yes |
| 8 | Ly6a | 278 | 2.197 | 0.2809 | Yes |
| 9 | Cd52 | 332 | 1.963 | 0.2988 | Yes |
| 10 | Dusp1 | 339 | 1.923 | 0.3260 | Yes |
| 11 | Cebpb | 425 | 1.620 | 0.3321 | Yes |
| 12 | Fxyd5 | 489 | 1.476 | 0.3407 | Yes |
| 13 | Apoe | 490 | 1.475 | 0.3625 | Yes |
| 14 | Lpxn | 492 | 1.463 | 0.3839 | Yes |
| 15 | Ctsz | 493 | 1.463 | 0.4055 | Yes |
| 16 | Alox5ap | 500 | 1.445 | 0.4256 | Yes |
| 17 | Tgfbi | 502 | 1.443 | 0.4467 | Yes |
| 18 | Psap | 510 | 1.415 | 0.4662 | Yes |
| 19 | Coro1a | 546 | 1.340 | 0.4786 | Yes |
| 20 | Grn | 554 | 1.328 | 0.4968 | Yes |
| 21 | Capg | 574 | 1.263 | 0.5115 | Yes |
| 22 | Arhgdib | 672 | 1.048 | 0.5067 | Yes |
| 23 | Trf | 682 | 1.033 | 0.5200 | Yes |
| 24 | Ctsc | 770 | 0.907 | 0.5152 | Yes |
| 25 | H2-Ab1 | 778 | 0.895 | 0.5270 | Yes |
| 26 | Gm2a | 789 | 0.881 | 0.5379 | Yes |
| 27 | B2m | 794 | 0.876 | 0.5500 | Yes |
| 28 | Cd74 | 811 | 0.856 | 0.5593 | Yes |
| 29 | Cdkn1a | 861 | 0.808 | 0.5610 | Yes |
| 30 | Nfkbia | 887 | 0.772 | 0.5671 | Yes |
| 31 | H2-Eb1 | 976 | 0.681 | 0.5587 | No |
| 32 | H2-D1 | 1021 | 0.632 | 0.5589 | No |
| 33 | Psmb8 | 1067 | 0.589 | 0.5581 | No |
| 34 | Pcbp1 | 1248 | -0.513 | 0.5280 | No |
| 35 | Tmsb4x | 1258 | -0.514 | 0.5337 | No |
| 36 | Cpne1 | 1265 | -0.515 | 0.5400 | No |
| 37 | Timp3 | 1480 | -0.548 | 0.5033 | No |
| 38 | Calm1 | 1839 | -0.607 | 0.4372 | No |
| 39 | Snx2 | 2321 | -0.693 | 0.3466 | No |
| 40 | Sdc4 | 2418 | -0.710 | 0.3370 | No |
| 41 | Shisa5 | 2493 | -0.727 | 0.3322 | No |
| 42 | Ly6e | 2739 | -0.775 | 0.2923 | No |
| 43 | Papss2 | 3416 | -0.969 | 0.1650 | No |
| 44 | Fos | 3575 | -1.023 | 0.1470 | No |
| 45 | Krt15 | 3685 | -1.071 | 0.1400 | No |
| 46 | Dcn | 3919 | -1.196 | 0.1088 | No |
| 47 | Oas1a | 3951 | -1.216 | 0.1203 | No |
| 48 | Btg2 | 4032 | -1.269 | 0.1223 | No |
| 49 | Tgif1 | 4179 | -1.391 | 0.1122 | No |
| 50 | Mgp | 4278 | -1.484 | 0.1136 | No |
Table: GSEA details [plain text format]

  

Fig 2: TABULA\_MURIS\_SENIS\_AORTA\_PROFESSIONAL\_ANTIGEN\_PRESENTING\_CELL\_AGEING: Random ES distribution      
 Gene set null distribution of ES for **TABULA\_MURIS\_SENIS\_AORTA\_PROFESSIONAL\_ANTIGEN\_PRESENTING\_CELL\_AGEING**

  
