# Supplementary material for: TMPRSS11B promotes an acidified microenvironment and immune suppression in squamous lung cancer
Source: EMBO Rep. 2025 Nov 10;26(24):6346–79. doi: 10.1038/s44319-025-00631-1 (PMC12714794; doi:10.1038/s44319-025-00631-1)
Supplement: Supplementary file 10 — Source data Fig. 5 [file 44319_2025_631_MOESM10_ESM.zip › Figure 5/5C-D/GSEA_Broad Institute_M8_T11b-high LUSC vs LUAD/TABULA_MURIS_SENIS_BRAIN_NON_MYELOID_NEURON_AGEING.html]

Details for gene set TABULA\_MURIS\_SENIS\_BRAIN\_NON\_MYELOID\_NEURON\_AGEING[GSEA]

|  || Dataset | Ranked list\_DGE\_squamousT11b\_vs\_all adenosadeno\_HSE13-NT copy |
| Phenotype | NoPhenotypeAvailable |
| Upregulated in class | na\_neg |
| GeneSet | TABULA\_MURIS\_SENIS\_BRAIN\_NON\_MYELOID\_NEURON\_AGEING |
| Enrichment Score (ES) | -0.12943582 |
| Normalized Enrichment Score (NES) | -0.8747668 |
| Nominal p-value | 0.8442623 |
| FDR q-value | 1.0 |
| FWER p-Value | 1.0 |
Table: GSEA Results Summary

  

Fig 1: Enrichment plot: TABULA\_MURIS\_SENIS\_BRAIN\_NON\_MYELOID\_NEURON\_AGEING      
 Profile of the Running ES Score & Positions of GeneSet Members on the Rank Ordered List

  

| SYMBOL | RANK IN GENE LIST | RANK METRIC SCORE | RUNNING ES | CORE ENRICHMENT || 1 | Pou3f1 | 107 | 3.639 | -0.0109 | No |
| 2 | Mamdc2 | 145 | 3.094 | -0.0082 | No |
| 3 | Kctd1 | 194 | 2.626 | -0.0096 | No |
| 4 | Spns2 | 197 | 2.574 | -0.0010 | No |
| 5 | Nectin1 | 233 | 2.387 | -0.0004 | No |
| 6 | Sirpa | 268 | 2.242 | -0.0000 | No |
| 7 | Sult2b1 | 282 | 2.172 | 0.0047 | No |
| 8 | Fth1 | 289 | 2.129 | 0.0109 | No |
| 9 | Vim | 302 | 2.105 | 0.0156 | No |
| 10 | Smpdl3b | 417 | 1.636 | -0.0038 | No |
| 11 | Klf4 | 448 | 1.555 | -0.0050 | No |
| 12 | 5031439G07Rik | 480 | 1.493 | -0.0066 | No |
| 13 | Apoe | 490 | 1.475 | -0.0034 | No |
| 14 | Jag2 | 498 | 1.452 | 0.0001 | No |
| 15 | Pltp | 523 | 1.391 | -0.0003 | No |
| 16 | S100a16 | 569 | 1.288 | -0.0057 | No |
| 17 | Capg | 574 | 1.263 | -0.0022 | No |
| 18 | Ifitm10 | 584 | 1.233 | 0.0002 | No |
| 19 | Fam171a2 | 590 | 1.227 | 0.0033 | No |
| 20 | Rnf149 | 664 | 1.063 | -0.0090 | No |
| 21 | Trf | 682 | 1.033 | -0.0092 | No |
| 22 | Plekho1 | 683 | 1.033 | -0.0055 | No |
| 23 | Cat | 712 | 0.989 | -0.0083 | No |
| 24 | Iffo2 | 725 | 0.974 | -0.0075 | No |
| 25 | Wwtr1 | 730 | 0.963 | -0.0050 | No |
| 26 | Ctsc | 770 | 0.907 | -0.0104 | No |
| 27 | B2m | 794 | 0.876 | -0.0124 | No |
| 28 | Cd74 | 811 | 0.856 | -0.0130 | No |
| 29 | B4galt5 | 825 | 0.839 | -0.0129 | No |
| 30 | Ptprs | 836 | 0.828 | -0.0122 | No |
| 31 | Pitpnm2 | 839 | 0.825 | -0.0098 | No |
| 32 | Plpp3 | 846 | 0.814 | -0.0082 | No |
| 33 | Pstpip2 | 866 | 0.805 | -0.0096 | No |
| 34 | Fem1c | 878 | 0.790 | -0.0093 | No |
| 35 | Cldn5 | 902 | 0.758 | -0.0117 | No |
| 36 | Flna | 905 | 0.755 | -0.0095 | No |
| 37 | Sptbn2 | 924 | 0.734 | -0.0109 | No |
| 38 | H2-Eb1 | 976 | 0.681 | -0.0197 | No |
| 39 | Atox1 | 997 | 0.651 | -0.0219 | No |
| 40 | Uchl3 | 1004 | 0.646 | -0.0209 | No |
| 41 | H2-D1 | 1021 | 0.632 | -0.0222 | No |
| 42 | Col3a1 | 1034 | 0.619 | -0.0227 | No |
| 43 | Ppic | 1039 | 0.615 | -0.0215 | No |
| 44 | Arhgap27 | 1048 | 0.610 | -0.0211 | No |
| 45 | Icam1 | 1070 | 0.586 | -0.0237 | No |
| 46 | Cfh | 1075 | 0.578 | -0.0225 | No |
| 47 | Txnrd1 | 1081 | 0.571 | -0.0216 | No |
| 48 | Arhgap17 | 1163 | 0.504 | -0.0377 | No |
| 49 | Kdm6b | 1191 | -0.504 | -0.0419 | No |
| 50 | Pgp | 1217 | -0.508 | -0.0456 | No |
| 51 | Tmem88 | 1221 | -0.508 | -0.0445 | No |
| 52 | Gigyf1 | 1233 | -0.510 | -0.0452 | No |
| 53 | Tmsb4x | 1258 | -0.514 | -0.0487 | No |
| 54 | Grcc10 | 1270 | -0.515 | -0.0493 | No |
| 55 | Pafah2 | 1284 | -0.517 | -0.0503 | No |
| 56 | Mon2 | 1296 | -0.519 | -0.0510 | No |
| 57 | Pfdn2 | 1298 | -0.519 | -0.0494 | No |
| 58 | Pabpn1 | 1301 | -0.519 | -0.0480 | No |
| 59 | Epg5 | 1334 | -0.523 | -0.0532 | No |
| 60 | Mllt1 | 1341 | -0.524 | -0.0527 | No |
| 61 | Pacs1 | 1342 | -0.524 | -0.0509 | No |
| 62 | Klc2 | 1357 | -0.527 | -0.0521 | No |
| 63 | Adgra2 | 1366 | -0.529 | -0.0520 | No |
| 64 | Mapk3 | 1369 | -0.529 | -0.0506 | No |
| 65 | Ltbp1 | 1378 | -0.530 | -0.0505 | No |
| 66 | Gtpbp6 | 1379 | -0.530 | -0.0487 | No |
| 67 | Sfxn3 | 1414 | -0.534 | -0.0543 | No |
| 68 | Tle5 | 1425 | -0.536 | -0.0546 | No |
| 69 | Dapk3 | 1439 | -0.539 | -0.0556 | No |
| 70 | Cep164 | 1441 | -0.539 | -0.0539 | No |
| 71 | Prmt3 | 1445 | -0.541 | -0.0527 | No |
| 72 | Glt8d1 | 1530 | -0.557 | -0.0693 | No |
| 73 | Lrrc14 | 1545 | -0.560 | -0.0704 | No |
| 74 | Acp6 | 1547 | -0.560 | -0.0687 | No |
| 75 | Dctn6 | 1596 | -0.567 | -0.0773 | No |
| 76 | Tppp3 | 1617 | -0.571 | -0.0797 | No |
| 77 | Pxmp4 | 1621 | -0.572 | -0.0783 | No |
| 78 | Iqsec2 | 1623 | -0.572 | -0.0766 | No |
| 79 | Mt1 | 1658 | -0.576 | -0.0820 | No |
| 80 | Sf3b5 | 1659 | -0.576 | -0.0800 | No |
| 81 | Cebpd | 1668 | -0.578 | -0.0798 | No |
| 82 | Kctd17 | 1672 | -0.579 | -0.0784 | No |
| 83 | Dhx37 | 1680 | -0.580 | -0.0779 | No |
| 84 | Rtf1 | 1697 | -0.583 | -0.0794 | No |
| 85 | Bgn | 1714 | -0.584 | -0.0809 | No |
| 86 | Dennd1b | 1717 | -0.585 | -0.0793 | No |
| 87 | Pus10 | 1718 | -0.585 | -0.0772 | No |
| 88 | Ddx10 | 1723 | -0.587 | -0.0760 | No |
| 89 | Zranb1 | 1740 | -0.590 | -0.0775 | No |
| 90 | Zgpat | 1741 | -0.590 | -0.0754 | No |
| 91 | Wwox | 1812 | -0.603 | -0.0888 | No |
| 92 | Zfp362 | 1817 | -0.603 | -0.0875 | No |
| 93 | Madd | 1824 | -0.604 | -0.0868 | No |
| 94 | Map1s | 1830 | -0.605 | -0.0857 | No |
| 95 | Acaa2 | 1846 | -0.609 | -0.0869 | No |
| 96 | Nr2c2ap | 1856 | -0.612 | -0.0868 | No |
| 97 | Tmem175 | 1863 | -0.613 | -0.0859 | No |
| 98 | Gga1 | 1882 | -0.616 | -0.0877 | No |
| 99 | Ado | 1886 | -0.617 | -0.0862 | No |
| 100 | Ankrd54 | 1890 | -0.617 | -0.0847 | No |
| 101 | Ccdc9 | 1892 | -0.617 | -0.0828 | No |
| 102 | 1700109H08Rik | 1927 | -0.622 | -0.0881 | No |
| 103 | Qdpr | 1949 | -0.627 | -0.0906 | No |
| 104 | Chd8 | 1955 | -0.628 | -0.0895 | No |
| 105 | Wdr59 | 1966 | -0.630 | -0.0895 | No |
| 106 | Ccar2 | 1982 | -0.633 | -0.0906 | No |
| 107 | Ppp1r35 | 2003 | -0.637 | -0.0927 | No |
| 108 | Cracr2b | 2009 | -0.637 | -0.0916 | No |
| 109 | Zfand3 | 2010 | -0.638 | -0.0894 | No |
| 110 | Csnk1g2 | 2018 | -0.639 | -0.0887 | No |
| 111 | Per1 | 2020 | -0.639 | -0.0867 | No |
| 112 | Gpatch8 | 2061 | -0.646 | -0.0932 | No |
| 113 | Pon2 | 2062 | -0.646 | -0.0910 | No |
| 114 | Tsr1 | 2069 | -0.647 | -0.0900 | No |
| 115 | Gtf3c5 | 2090 | -0.652 | -0.0922 | No |
| 116 | Mpv17l2 | 2093 | -0.653 | -0.0903 | No |
| 117 | Tmem101 | 2118 | -0.657 | -0.0933 | No |
| 118 | Spg7 | 2140 | -0.661 | -0.0956 | No |
| 119 | Trio | 2167 | -0.665 | -0.0990 | No |
| 120 | Slc9a6 | 2186 | -0.668 | -0.1007 | No |
| 121 | Acbd6 | 2195 | -0.669 | -0.1001 | No |
| 122 | Tex261 | 2206 | -0.672 | -0.0999 | No |
| 123 | Gnb2 | 2210 | -0.673 | -0.0982 | No |
| 124 | Keap1 | 2212 | -0.673 | -0.0961 | No |
| 125 | Ing4 | 2236 | -0.678 | -0.0988 | No |
| 126 | Mettl1 | 2254 | -0.681 | -0.1002 | No |
| 127 | Kpna4 | 2268 | -0.683 | -0.1006 | No |
| 128 | Chka | 2293 | -0.687 | -0.1035 | No |
| 129 | Pnpla6 | 2295 | -0.687 | -0.1013 | No |
| 130 | Zfp692 | 2298 | -0.688 | -0.0994 | No |
| 131 | Zyx | 2334 | -0.694 | -0.1047 | No |
| 132 | Fam98c | 2335 | -0.695 | -0.1022 | No |
| 133 | Sap130 | 2336 | -0.695 | -0.0998 | No |
| 134 | Ehmt2 | 2392 | -0.705 | -0.1095 | No |
| 135 | Rab11fip2 | 2408 | -0.707 | -0.1103 | No |
| 136 | Stau1 | 2415 | -0.709 | -0.1091 | No |
| 137 | Sdc4 | 2418 | -0.710 | -0.1071 | No |
| 138 | Nacc2 | 2424 | -0.711 | -0.1057 | No |
| 139 | Elp2 | 2430 | -0.713 | -0.1043 | No |
| 140 | Mast3 | 2443 | -0.716 | -0.1044 | No |
| 141 | Mfn1 | 2472 | -0.722 | -0.1081 | No |
| 142 | Hip1r | 2473 | -0.723 | -0.1056 | No |
| 143 | Tbc1d1 | 2474 | -0.723 | -0.1030 | No |
| 144 | Enoph1 | 2476 | -0.723 | -0.1007 | No |
| 145 | Aldoc | 2488 | -0.726 | -0.1006 | No |
| 146 | Rmdn1 | 2495 | -0.727 | -0.0994 | No |
| 147 | Nt5c | 2556 | -0.737 | -0.1100 | No |
| 148 | Chd3 | 2557 | -0.738 | -0.1074 | No |
| 149 | Smad7 | 2594 | -0.746 | -0.1128 | No |
| 150 | Fig4 | 2614 | -0.752 | -0.1143 | No |
| 151 | Yeats2 | 2641 | -0.757 | -0.1174 | No |
| 152 | Tfdp2 | 2658 | -0.760 | -0.1183 | No |
| 153 | Akap8 | 2662 | -0.762 | -0.1163 | No |
| 154 | Zfpl1 | 2674 | -0.763 | -0.1160 | No |
| 155 | Polr2i | 2682 | -0.764 | -0.1149 | No |
| 156 | Msra | 2696 | -0.767 | -0.1151 | No |
| 157 | Tmem208 | 2697 | -0.767 | -0.1124 | No |
| 158 | Exoc6b | 2706 | -0.768 | -0.1115 | No |
| 159 | Mxd4 | 2713 | -0.770 | -0.1101 | No |
| 160 | Pnisr | 2718 | -0.771 | -0.1083 | No |
| 161 | Megf8 | 2724 | -0.772 | -0.1067 | No |
| 162 | Ctc1 | 2733 | -0.774 | -0.1057 | No |
| 163 | Zcchc14 | 2737 | -0.775 | -0.1037 | No |
| 164 | Fabp5 | 2745 | -0.778 | -0.1025 | No |
| 165 | Rdh13 | 2754 | -0.780 | -0.1015 | No |
| 166 | Tmc7 | 2761 | -0.782 | -0.1001 | No |
| 167 | Cyp20a1 | 2765 | -0.783 | -0.0980 | No |
| 168 | Lamc1 | 2789 | -0.788 | -0.1003 | No |
| 169 | Phf2 | 2802 | -0.791 | -0.1002 | No |
| 170 | Dxo | 2830 | -0.797 | -0.1034 | No |
| 171 | Eif2b4 | 2839 | -0.800 | -0.1024 | No |
| 172 | N4bp2 | 2846 | -0.801 | -0.1009 | No |
| 173 | Trrap | 2854 | -0.803 | -0.0996 | No |
| 174 | Gpr137 | 2889 | -0.813 | -0.1043 | No |
| 175 | C1qtnf4 | 2892 | -0.813 | -0.1019 | No |
| 176 | Hook2 | 2944 | -0.824 | -0.1102 | No |
| 177 | Fh1 | 2972 | -0.834 | -0.1132 | No |
| 178 | Ift43 | 2992 | -0.838 | -0.1145 | No |
| 179 | Rbm7 | 2994 | -0.839 | -0.1118 | No |
| 180 | Rpf1 | 3015 | -0.846 | -0.1132 | No |
| 181 | Nfkbil1 | 3026 | -0.849 | -0.1125 | No |
| 182 | Arhgef40 | 3029 | -0.849 | -0.1099 | No |
| 183 | Gadd45gip1 | 3033 | -0.850 | -0.1076 | No |
| 184 | Ube2o | 3056 | -0.857 | -0.1095 | No |
| 185 | Foxj3 | 3076 | -0.864 | -0.1106 | No |
| 186 | Vps26c | 3081 | -0.865 | -0.1085 | No |
| 187 | L3mbtl2 | 3085 | -0.866 | -0.1061 | No |
| 188 | Adcy9 | 3086 | -0.866 | -0.1031 | No |
| 189 | Haghl | 3099 | -0.871 | -0.1027 | No |
| 190 | Ring1 | 3102 | -0.871 | -0.1001 | No |
| 191 | Tgfbr2 | 3104 | -0.872 | -0.0972 | No |
| 192 | Cirbp | 3148 | -0.884 | -0.1036 | No |
| 193 | Lrrc42 | 3152 | -0.886 | -0.1012 | No |
| 194 | Dbp | 3176 | -0.892 | -0.1031 | No |
| 195 | Spint2 | 3189 | -0.895 | -0.1026 | No |
| 196 | Kdm6a | 3210 | -0.902 | -0.1039 | No |
| 197 | C2cd3 | 3215 | -0.903 | -0.1016 | No |
| 198 | Tsc1 | 3220 | -0.905 | -0.0993 | No |
| 199 | Gfpt1 | 3227 | -0.907 | -0.0975 | No |
| 200 | D11Wsu47e | 3258 | -0.918 | -0.1009 | No |
| 201 | Commd9 | 3259 | -0.918 | -0.0977 | No |
| 202 | Zfp324 | 3261 | -0.918 | -0.0947 | No |
| 203 | Gramd4 | 3265 | -0.920 | -0.0921 | No |
| 204 | Phldb2 | 3270 | -0.920 | -0.0898 | No |
| 205 | Ppa2 | 3287 | -0.925 | -0.0901 | No |
| 206 | Akr1e1 | 3295 | -0.929 | -0.0884 | No |
| 207 | Cant1 | 3316 | -0.936 | -0.0895 | No |
| 208 | B9d2 | 3338 | -0.941 | -0.0908 | No |
| 209 | Fcgrt | 3361 | -0.950 | -0.0923 | No |
| 210 | Taco1 | 3404 | -0.962 | -0.0982 | No |
| 211 | Kiz | 3407 | -0.962 | -0.0953 | No |
| 212 | Ap2b1 | 3413 | -0.966 | -0.0930 | No |
| 213 | Sh3yl1 | 3461 | -0.985 | -0.1000 | No |
| 214 | Mecr | 3462 | -0.985 | -0.0965 | No |
| 215 | Armc9 | 3496 | -0.995 | -0.1003 | No |
| 216 | Vps13d | 3536 | -1.011 | -0.1054 | No |
| 217 | Maz | 3596 | -1.032 | -0.1148 | No |
| 218 | Polr3e | 3608 | -1.035 | -0.1136 | No |
| 219 | Pik3r4 | 3618 | -1.038 | -0.1119 | No |
| 220 | Dnajc1 | 3626 | -1.042 | -0.1098 | No |
| 221 | Krt15 | 3685 | -1.071 | -0.1188 | No |
| 222 | Dock7 | 3734 | -1.095 | -0.1256 | Yes |
| 223 | Pcyt2 | 3738 | -1.098 | -0.1224 | Yes |
| 224 | Mettl8 | 3745 | -1.100 | -0.1199 | Yes |
| 225 | Taf7 | 3751 | -1.102 | -0.1171 | Yes |
| 226 | Zfp821 | 3773 | -1.113 | -0.1179 | Yes |
| 227 | Pick1 | 3793 | -1.123 | -0.1181 | Yes |
| 228 | Iqcc | 3807 | -1.132 | -0.1170 | Yes |
| 229 | Tab1 | 3819 | -1.139 | -0.1155 | Yes |
| 230 | Trim68 | 3845 | -1.154 | -0.1169 | Yes |
| 231 | Cdc42ep1 | 3847 | -1.155 | -0.1131 | Yes |
| 232 | Tgoln1 | 3875 | -1.168 | -0.1150 | Yes |
| 233 | Dusp23 | 3901 | -1.183 | -0.1164 | Yes |
| 234 | Dynll2 | 3904 | -1.185 | -0.1126 | Yes |
| 235 | Bri3 | 3905 | -1.186 | -0.1085 | Yes |
| 236 | Dcn | 3919 | -1.196 | -0.1072 | Yes |
| 237 | Hadh | 3948 | -1.215 | -0.1091 | Yes |
| 238 | Qsox1 | 3954 | -1.217 | -0.1059 | Yes |
| 239 | Serpinb6a | 3957 | -1.219 | -0.1021 | Yes |
| 240 | Sgce | 3962 | -1.222 | -0.0987 | Yes |
| 241 | Pycr1 | 3965 | -1.224 | -0.0949 | Yes |
| 242 | Hsd17b11 | 3980 | -1.233 | -0.0936 | Yes |
| 243 | Bcl2 | 3987 | -1.239 | -0.0906 | Yes |
| 244 | Med19 | 3991 | -1.241 | -0.0869 | Yes |
| 245 | Prrc1 | 3995 | -1.245 | -0.0832 | Yes |
| 246 | Btg2 | 4032 | -1.269 | -0.0867 | Yes |
| 247 | Zfp787 | 4105 | -1.333 | -0.0979 | Yes |
| 248 | Cyp4f16 | 4130 | -1.354 | -0.0985 | Yes |
| 249 | Tyro3 | 4132 | -1.355 | -0.0940 | Yes |
| 250 | Hdac5 | 4145 | -1.366 | -0.0918 | Yes |
| 251 | Zc3h6 | 4155 | -1.373 | -0.0890 | Yes |
| 252 | Kansl1l | 4165 | -1.383 | -0.0862 | Yes |
| 253 | Gstm1 | 4200 | -1.410 | -0.0887 | Yes |
| 254 | Cystm1 | 4215 | -1.424 | -0.0868 | Yes |
| 255 | AU040320 | 4246 | -1.464 | -0.0883 | Yes |
| 256 | Ncmap | 4254 | -1.469 | -0.0847 | Yes |
| 257 | Sec14l1 | 4263 | -1.472 | -0.0813 | Yes |
| 258 | Pcgf2 | 4270 | -1.479 | -0.0775 | Yes |
| 259 | Ppcs | 4272 | -1.480 | -0.0725 | Yes |
| 260 | Spr | 4273 | -1.481 | -0.0673 | Yes |
| 261 | Mgp | 4278 | -1.484 | -0.0630 | Yes |
| 262 | Auts2 | 4305 | -1.508 | -0.0635 | Yes |
| 263 | Stard10 | 4317 | -1.525 | -0.0605 | Yes |
| 264 | Ptgis | 4321 | -1.530 | -0.0558 | Yes |
| 265 | Basp1 | 4338 | -1.550 | -0.0539 | Yes |
| 266 | Atp8a1 | 4339 | -1.555 | -0.0485 | Yes |
| 267 | Sidt1 | 4350 | -1.575 | -0.0452 | Yes |
| 268 | Senp6 | 4370 | -1.600 | -0.0438 | Yes |
| 269 | Mapk8ip1 | 4373 | -1.605 | -0.0386 | Yes |
| 270 | Lmo4 | 4382 | -1.617 | -0.0347 | Yes |
| 271 | Selenop | 4397 | -1.636 | -0.0321 | Yes |
| 272 | Syt7 | 4409 | -1.661 | -0.0287 | Yes |
| 273 | Slc35d1 | 4438 | -1.718 | -0.0288 | Yes |
| 274 | Sorbs3 | 4439 | -1.719 | -0.0228 | Yes |
| 275 | Lypd8 | 4470 | -1.770 | -0.0232 | Yes |
| 276 | Krt8 | 4476 | -1.778 | -0.0181 | Yes |
| 277 | Pde8b | 4504 | -1.823 | -0.0177 | Yes |
| 278 | Cul9 | 4559 | -1.940 | -0.0228 | Yes |
| 279 | Ufsp1 | 4657 | -2.202 | -0.0365 | Yes |
| 280 | Epb41l4b | 4659 | -2.204 | -0.0290 | Yes |
| 281 | Lurap1l | 4679 | -2.307 | -0.0251 | Yes |
| 282 | Serp2 | 4693 | -2.366 | -0.0197 | Yes |
| 283 | Hmgcs2 | 4714 | -2.435 | -0.0156 | Yes |
| 284 | Adh1 | 4746 | -2.614 | -0.0132 | Yes |
| 285 | Dmd | 4759 | -2.718 | -0.0064 | Yes |
| 286 | Ttyh1 | 4766 | -2.750 | 0.0019 | Yes |
| 287 | Ppp1r1b | 4771 | -2.786 | 0.0108 | Yes |
Table: GSEA details [plain text format]

  

Fig 2: TABULA\_MURIS\_SENIS\_BRAIN\_NON\_MYELOID\_NEURON\_AGEING: Random ES distribution      
 Gene set null distribution of ES for **TABULA\_MURIS\_SENIS\_BRAIN\_NON\_MYELOID\_NEURON\_AGEING**

  
