# Supplementary material for: TMPRSS11B promotes an acidified microenvironment and immune suppression in squamous lung cancer
Source: EMBO Rep. 2025 Nov 10;26(24):6346–79. doi: 10.1038/s44319-025-00631-1 (PMC12714794; doi:10.1038/s44319-025-00631-1)
Supplement: Supplementary file 10 — Source data Fig. 5 [file 44319_2025_631_MOESM10_ESM.zip › Figure 5/5C-D/GSEA_Broad Institute_M8_T11b-high LUSC vs LUAD/TABULA_MURIS_SENIS_HEART_AND_AORTA_LEUKOCYTE_AGEING.html]

Details for gene set TABULA\_MURIS\_SENIS\_HEART\_AND\_AORTA\_LEUKOCYTE\_AGEING[GSEA]

|  || Dataset | Ranked list\_DGE\_squamousT11b\_vs\_all adenosadeno\_HSE13-NT copy |
| Phenotype | NoPhenotypeAvailable |
| Upregulated in class | na\_pos |
| GeneSet | TABULA\_MURIS\_SENIS\_HEART\_AND\_AORTA\_LEUKOCYTE\_AGEING |
| Enrichment Score (ES) | 0.6418905 |
| Normalized Enrichment Score (NES) | 2.791916 |
| Nominal p-value | 0.0 |
| FDR q-value | 0.0 |
| FWER p-Value | 0.0 |
Table: GSEA Results Summary

  

Fig 1: Enrichment plot: TABULA\_MURIS\_SENIS\_HEART\_AND\_AORTA\_LEUKOCYTE\_AGEING      
 Profile of the Running ES Score & Positions of GeneSet Members on the Rank Ordered List

  

| SYMBOL | RANK IN GENE LIST | RANK METRIC SCORE | RUNNING ES | CORE ENRICHMENT || 1 | Ecm1 | 27 | 5.794 | 0.0760 | Yes |
| 2 | Tnfaip2 | 83 | 3.933 | 0.1200 | Yes |
| 3 | Clec4d | 131 | 3.254 | 0.1560 | Yes |
| 4 | Itgb2 | 158 | 2.937 | 0.1920 | Yes |
| 5 | Srgn | 185 | 2.715 | 0.2248 | Yes |
| 6 | Ccl6 | 187 | 2.695 | 0.2626 | Yes |
| 7 | Anxa1 | 218 | 2.455 | 0.2909 | Yes |
| 8 | Il1b | 240 | 2.351 | 0.3197 | Yes |
| 9 | Wfdc17 | 241 | 2.337 | 0.3526 | Yes |
| 10 | Plek | 252 | 2.303 | 0.3830 | Yes |
| 11 | Ly6a | 278 | 2.197 | 0.4087 | Yes |
| 12 | Fcgr2b | 280 | 2.178 | 0.4392 | Yes |
| 13 | Pim1 | 319 | 2.011 | 0.4596 | Yes |
| 14 | Pirb | 330 | 1.965 | 0.4852 | Yes |
| 15 | Mpeg1 | 346 | 1.889 | 0.5087 | Yes |
| 16 | S100a4 | 353 | 1.867 | 0.5338 | Yes |
| 17 | Lgals3 | 447 | 1.559 | 0.5363 | Yes |
| 18 | Klf4 | 448 | 1.555 | 0.5582 | Yes |
| 19 | Hspb1 | 460 | 1.530 | 0.5775 | Yes |
| 20 | Sirpb1c | 515 | 1.407 | 0.5860 | Yes |
| 21 | Metrnl | 520 | 1.394 | 0.6048 | Yes |
| 22 | Acp5 | 536 | 1.366 | 0.6209 | Yes |
| 23 | Xdh | 540 | 1.354 | 0.6394 | Yes |
| 24 | Ier5 | 609 | 1.186 | 0.6419 | Yes |
| 25 | Ninj1 | 833 | 0.831 | 0.6069 | No |
| 26 | Fabp4 | 914 | 0.747 | 0.6007 | No |
| 27 | Ier3 | 967 | 0.691 | 0.5995 | No |
| 28 | Mcl1 | 969 | 0.689 | 0.6090 | No |
| 29 | Msrb1 | 982 | 0.669 | 0.6160 | No |
| 30 | Icam1 | 1070 | 0.586 | 0.6060 | No |
| 31 | Nupr1 | 1092 | 0.562 | 0.6095 | No |
| 32 | Trib1 | 1112 | 0.544 | 0.6132 | No |
| 33 | Stat3 | 1393 | -0.531 | 0.5621 | No |
| 34 | Mgst1 | 1519 | -0.554 | 0.5437 | No |
| 35 | Bgn | 1714 | -0.584 | 0.5114 | No |
| 36 | Tpt1 | 1787 | -0.598 | 0.5047 | No |
| 37 | Pnrc1 | 1935 | -0.624 | 0.4827 | No |
| 38 | Socs3 | 2116 | -0.657 | 0.4543 | No |
| 39 | Nfkbiz | 2566 | -0.740 | 0.3707 | No |
| 40 | S100a6 | 3412 | -0.966 | 0.2074 | No |
| 41 | Fos | 3575 | -1.023 | 0.1879 | No |
| 42 | Id3 | 3653 | -1.055 | 0.1867 | No |
| 43 | Dcn | 3919 | -1.196 | 0.1480 | No |
| 44 | Nr4a1 | 3978 | -1.233 | 0.1533 | No |
| 45 | Lmo4 | 4382 | -1.617 | 0.0917 | No |
Table: GSEA details [plain text format]

  

Fig 2: TABULA\_MURIS\_SENIS\_HEART\_AND\_AORTA\_LEUKOCYTE\_AGEING: Random ES distribution      
 Gene set null distribution of ES for **TABULA\_MURIS\_SENIS\_HEART\_AND\_AORTA\_LEUKOCYTE\_AGEING**

  
