# Supplementary material for: TMPRSS11B promotes an acidified microenvironment and immune suppression in squamous lung cancer
Source: EMBO Rep. 2025 Nov 10;26(24):6346–79. doi: 10.1038/s44319-025-00631-1 (PMC12714794; doi:10.1038/s44319-025-00631-1)
Supplement: Supplementary file 10 — Source data Fig. 5 [file 44319_2025_631_MOESM10_ESM.zip › Figure 5/5C-D/GSEA_Broad Institute_M8_T11b-high LUSC vs LUAD/TABULA_MURIS_SENIS_HEART_ATRIAL_MYOCYTE_AGEING.html]

Details for gene set TABULA\_MURIS\_SENIS\_HEART\_ATRIAL\_MYOCYTE\_AGEING[GSEA]

|  || Dataset | Ranked list\_DGE\_squamousT11b\_vs\_all adenosadeno\_HSE13-NT copy |
| Phenotype | NoPhenotypeAvailable |
| Upregulated in class | na\_neg |
| GeneSet | TABULA\_MURIS\_SENIS\_HEART\_ATRIAL\_MYOCYTE\_AGEING |
| Enrichment Score (ES) | -0.21297488 |
| Normalized Enrichment Score (NES) | -1.0389252 |
| Nominal p-value | 0.38010204 |
| FDR q-value | 1.0 |
| FWER p-Value | 1.0 |
Table: GSEA Results Summary

  

Fig 1: Enrichment plot: TABULA\_MURIS\_SENIS\_HEART\_ATRIAL\_MYOCYTE\_AGEING      
 Profile of the Running ES Score & Positions of GeneSet Members on the Rank Ordered List

  

| SYMBOL | RANK IN GENE LIST | RANK METRIC SCORE | RUNNING ES | CORE ENRICHMENT || 1 | Abca12 | 19 | 6.246 | 0.0960 | No |
| 2 | Ctss | 247 | 2.317 | 0.0855 | No |
| 3 | Ly6a | 278 | 2.197 | 0.1143 | No |
| 4 | Selplg | 432 | 1.604 | 0.1079 | No |
| 5 | Fxyd5 | 489 | 1.476 | 0.1198 | No |
| 6 | Coro1a | 546 | 1.340 | 0.1295 | No |
| 7 | Capg | 574 | 1.263 | 0.1441 | No |
| 8 | C1qb | 615 | 1.180 | 0.1546 | No |
| 9 | Arhgdib | 672 | 1.048 | 0.1596 | No |
| 10 | Ctnnbip1 | 677 | 1.039 | 0.1754 | No |
| 11 | H2-Ab1 | 778 | 0.895 | 0.1687 | No |
| 12 | Cd74 | 811 | 0.856 | 0.1757 | No |
| 13 | H2-Eb1 | 976 | 0.681 | 0.1523 | No |
| 14 | Mkrn1 | 1093 | 0.561 | 0.1369 | No |
| 15 | Car2 | 1171 | -0.500 | 0.1288 | No |
| 16 | Ppme1 | 1268 | -0.515 | 0.1169 | No |
| 17 | Tle5 | 1425 | -0.536 | 0.0928 | No |
| 18 | Vezf1 | 1535 | -0.558 | 0.0789 | No |
| 19 | Pdlim4 | 1851 | -0.610 | 0.0226 | No |
| 20 | Fech | 1931 | -0.623 | 0.0161 | No |
| 21 | Rnf216 | 1957 | -0.628 | 0.0209 | No |
| 22 | Aph1a | 2074 | -0.648 | 0.0069 | No |
| 23 | Rbpms | 2228 | -0.676 | -0.0143 | No |
| 24 | Prpf19 | 2264 | -0.683 | -0.0107 | No |
| 25 | Hnrnpd | 2328 | -0.694 | -0.0128 | No |
| 26 | Med25 | 2784 | -0.787 | -0.0956 | No |
| 27 | Lmtk2 | 2800 | -0.790 | -0.0861 | No |
| 28 | Gfpt1 | 3227 | -0.907 | -0.1608 | No |
| 29 | Zfp579 | 3451 | -0.981 | -0.1919 | No |
| 30 | Pnpo | 3495 | -0.995 | -0.1850 | No |
| 31 | Samd8 | 3502 | -0.998 | -0.1703 | No |
| 32 | Id3 | 3653 | -1.055 | -0.1848 | No |
| 33 | Macrod1 | 3732 | -1.095 | -0.1836 | No |
| 34 | Zmym2 | 3873 | -1.166 | -0.1943 | Yes |
| 35 | Dynll2 | 3904 | -1.185 | -0.1816 | Yes |
| 36 | Bri3 | 3905 | -1.186 | -0.1627 | Yes |
| 37 | Vamp2 | 3923 | -1.199 | -0.1470 | Yes |
| 38 | Pxmp2 | 4099 | -1.324 | -0.1625 | Yes |
| 39 | Nr2f2 | 4183 | -1.396 | -0.1576 | Yes |
| 40 | Bpgm | 4184 | -1.396 | -0.1353 | Yes |
| 41 | Ccnd1 | 4229 | -1.443 | -0.1214 | Yes |
| 42 | Spr | 4273 | -1.481 | -0.1067 | Yes |
| 43 | Amacr | 4274 | -1.482 | -0.0830 | Yes |
| 44 | Syt7 | 4409 | -1.661 | -0.0845 | Yes |
| 45 | Zfp704 | 4443 | -1.725 | -0.0638 | Yes |
| 46 | Gca | 4505 | -1.825 | -0.0474 | Yes |
| 47 | Lamb3 | 4582 | -1.998 | -0.0314 | Yes |
| 48 | Ccnd2 | 4672 | -2.265 | -0.0138 | Yes |
| 49 | Ppp1r1b | 4771 | -2.786 | 0.0103 | Yes |
Table: GSEA details [plain text format]

  

Fig 2: TABULA\_MURIS\_SENIS\_HEART\_ATRIAL\_MYOCYTE\_AGEING: Random ES distribution      
 Gene set null distribution of ES for **TABULA\_MURIS\_SENIS\_HEART\_ATRIAL\_MYOCYTE\_AGEING**

  
