# Supplementary material for: TMPRSS11B promotes an acidified microenvironment and immune suppression in squamous lung cancer
Source: EMBO Rep. 2025 Nov 10;26(24):6346–79. doi: 10.1038/s44319-025-00631-1 (PMC12714794; doi:10.1038/s44319-025-00631-1)
Supplement: Supplementary file 10 — Source data Fig. 5 [file 44319_2025_631_MOESM10_ESM.zip › Figure 5/5C-D/GSEA_Broad Institute_M8_T11b-high LUSC vs LUAD/TABULA_MURIS_SENIS_KIDNEY_EPITHELIAL_CELL_OF_PROXIMAL_TUBULE_AGEING.html]

Details for gene set TABULA\_MURIS\_SENIS\_KIDNEY\_EPITHELIAL\_CELL\_OF\_PROXIMAL\_TUBULE\_AGEING[GSEA]

|  || Dataset | Ranked list\_DGE\_squamousT11b\_vs\_all adenosadeno\_HSE13-NT copy |
| Phenotype | NoPhenotypeAvailable |
| Upregulated in class | na\_neg |
| GeneSet | TABULA\_MURIS\_SENIS\_KIDNEY\_EPITHELIAL\_CELL\_OF\_PROXIMAL\_TUBULE\_AGEING |
| Enrichment Score (ES) | -0.16503964 |
| Normalized Enrichment Score (NES) | -0.9539953 |
| Nominal p-value | 0.56368566 |
| FDR q-value | 1.0 |
| FWER p-Value | 1.0 |
Table: GSEA Results Summary

  

Fig 1: Enrichment plot: TABULA\_MURIS\_SENIS\_KIDNEY\_EPITHELIAL\_CELL\_OF\_PROXIMAL\_TUBULE\_AGEING      
 Profile of the Running ES Score & Positions of GeneSet Members on the Rank Ordered List

  

| SYMBOL | RANK IN GENE LIST | RANK METRIC SCORE | RUNNING ES | CORE ENRICHMENT || 1 | Npl | 184 | 2.724 | -0.0146 | No |
| 2 | Scd1 | 215 | 2.464 | 0.0011 | No |
| 3 | S100a10 | 263 | 2.263 | 0.0115 | No |
| 4 | Fth1 | 289 | 2.129 | 0.0253 | No |
| 5 | Nqo1 | 301 | 2.106 | 0.0418 | No |
| 6 | Apoe | 490 | 1.475 | 0.0152 | No |
| 7 | Acot1 | 512 | 1.413 | 0.0234 | No |
| 8 | Cd302 | 521 | 1.393 | 0.0342 | No |
| 9 | Tmem37 | 539 | 1.356 | 0.0427 | No |
| 10 | Hebp1 | 562 | 1.299 | 0.0497 | No |
| 11 | Cstb | 587 | 1.229 | 0.0556 | No |
| 12 | Sat1 | 614 | 1.180 | 0.0607 | No |
| 13 | Txn1 | 743 | 0.944 | 0.0420 | No |
| 14 | Mgll | 752 | 0.929 | 0.0487 | No |
| 15 | B2m | 794 | 0.876 | 0.0478 | No |
| 16 | Cd74 | 811 | 0.856 | 0.0521 | No |
| 17 | Nuak2 | 819 | 0.843 | 0.0582 | No |
| 18 | Fam162a | 858 | 0.810 | 0.0574 | No |
| 19 | Uba52 | 896 | 0.762 | 0.0564 | No |
| 20 | Adk | 931 | 0.720 | 0.0556 | No |
| 21 | Txndc17 | 1025 | 0.628 | 0.0416 | No |
| 22 | Tmbim4 | 1103 | 0.550 | 0.0302 | No |
| 23 | Ndufb6 | 1150 | 0.512 | 0.0250 | No |
| 24 | Gsta4 | 1227 | -0.509 | 0.0135 | No |
| 25 | Eef1d | 1235 | -0.510 | 0.0166 | No |
| 26 | Grcc10 | 1270 | -0.515 | 0.0140 | No |
| 27 | Anapc13 | 1293 | -0.518 | 0.0139 | No |
| 28 | Mgst1 | 1519 | -0.554 | -0.0288 | No |
| 29 | Tmem205 | 1549 | -0.560 | -0.0299 | No |
| 30 | Wfdc2 | 1725 | -0.587 | -0.0617 | No |
| 31 | Gemin7 | 1747 | -0.591 | -0.0609 | No |
| 32 | Tpt1 | 1787 | -0.598 | -0.0638 | No |
| 33 | Micos13 | 1804 | -0.601 | -0.0618 | No |
| 34 | Eif3f | 1833 | -0.606 | -0.0623 | No |
| 35 | Acaa2 | 1846 | -0.609 | -0.0594 | No |
| 36 | Eif1 | 1859 | -0.612 | -0.0564 | No |
| 37 | Pnrc1 | 1935 | -0.624 | -0.0667 | No |
| 38 | Cyb5a | 1942 | -0.625 | -0.0624 | No |
| 39 | Bphl | 1944 | -0.625 | -0.0570 | No |
| 40 | Mpst | 1962 | -0.629 | -0.0550 | No |
| 41 | Vkorc1 | 2063 | -0.646 | -0.0704 | No |
| 42 | Srrm2 | 2159 | -0.663 | -0.0846 | No |
| 43 | Tex261 | 2206 | -0.672 | -0.0883 | No |
| 44 | Fmc1 | 2232 | -0.677 | -0.0875 | No |
| 45 | Ndufa7 | 2344 | -0.696 | -0.1048 | No |
| 46 | Laptm4b | 2349 | -0.697 | -0.0994 | No |
| 47 | Polr2e | 2361 | -0.699 | -0.0955 | No |
| 48 | Gas6 | 2372 | -0.701 | -0.0913 | No |
| 49 | Aldh2 | 2388 | -0.704 | -0.0882 | No |
| 50 | Guk1 | 2516 | -0.730 | -0.1086 | No |
| 51 | Krtcap2 | 2586 | -0.744 | -0.1165 | No |
| 52 | Fbp2 | 2651 | -0.759 | -0.1233 | No |
| 53 | Atraid | 2778 | -0.786 | -0.1429 | No |
| 54 | Hcfc1r1 | 2783 | -0.787 | -0.1367 | No |
| 55 | Ddt | 2787 | -0.788 | -0.1303 | No |
| 56 | Iah1 | 2825 | -0.796 | -0.1310 | No |
| 57 | Gstk1 | 2827 | -0.797 | -0.1241 | No |
| 58 | Sephs2 | 2836 | -0.798 | -0.1186 | No |
| 59 | Ifi27 | 2905 | -0.815 | -0.1257 | No |
| 60 | Tmco1 | 2982 | -0.837 | -0.1343 | No |
| 61 | Hmgb1 | 2990 | -0.838 | -0.1283 | No |
| 62 | S100a1 | 3077 | -0.864 | -0.1388 | No |
| 63 | Tmem147 | 3100 | -0.871 | -0.1357 | No |
| 64 | Hsd17b10 | 3144 | -0.883 | -0.1369 | No |
| 65 | Sgk1 | 3151 | -0.885 | -0.1302 | No |
| 66 | Chchd7 | 3229 | -0.909 | -0.1384 | No |
| 67 | Gsta3 | 3234 | -0.910 | -0.1311 | No |
| 68 | Ddrgk1 | 3282 | -0.924 | -0.1327 | No |
| 69 | Ppa2 | 3287 | -0.925 | -0.1253 | No |
| 70 | Gstm5 | 3294 | -0.927 | -0.1183 | No |
| 71 | Acsl1 | 3337 | -0.941 | -0.1187 | No |
| 72 | Hint2 | 3347 | -0.946 | -0.1121 | No |
| 73 | Tmem59 | 3397 | -0.960 | -0.1139 | No |
| 74 | Cryz | 3421 | -0.972 | -0.1101 | No |
| 75 | Cbr1 | 3607 | -1.035 | -0.1400 | No |
| 76 | Sri | 3726 | -1.092 | -0.1553 | Yes |
| 77 | Gm5617 | 3729 | -1.094 | -0.1459 | Yes |
| 78 | Srsf3 | 3744 | -1.100 | -0.1390 | Yes |
| 79 | 2310039H08Rik | 3763 | -1.108 | -0.1329 | Yes |
| 80 | Cfb | 3801 | -1.129 | -0.1306 | Yes |
| 81 | Npnt | 3802 | -1.129 | -0.1205 | Yes |
| 82 | Ascc1 | 3821 | -1.140 | -0.1141 | Yes |
| 83 | Krcc1 | 3922 | -1.198 | -0.1245 | Yes |
| 84 | Btg2 | 4032 | -1.269 | -0.1363 | Yes |
| 85 | Ncoa7 | 4063 | -1.292 | -0.1310 | Yes |
| 86 | Pts | 4078 | -1.307 | -0.1223 | Yes |
| 87 | Pxmp2 | 4099 | -1.324 | -0.1146 | Yes |
| 88 | Tstd1 | 4139 | -1.362 | -0.1107 | Yes |
| 89 | Gstm1 | 4200 | -1.410 | -0.1108 | Yes |
| 90 | Akr7a5 | 4207 | -1.413 | -0.0994 | Yes |
| 91 | Ccdc107 | 4218 | -1.430 | -0.0887 | Yes |
| 92 | Ccnd1 | 4229 | -1.443 | -0.0779 | Yes |
| 93 | Dnajc12 | 4234 | -1.449 | -0.0657 | Yes |
| 94 | Cmbl | 4433 | -1.707 | -0.0924 | Yes |
| 95 | Ggact | 4441 | -1.723 | -0.0784 | Yes |
| 96 | Csrp2 | 4490 | -1.794 | -0.0725 | Yes |
| 97 | Rida | 4525 | -1.856 | -0.0631 | Yes |
| 98 | Cela1 | 4538 | -1.888 | -0.0487 | Yes |
| 99 | Tcea3 | 4554 | -1.926 | -0.0346 | Yes |
| 100 | Sult1c2 | 4612 | -2.077 | -0.0281 | Yes |
| 101 | Cyp4b1 | 4689 | -2.344 | -0.0232 | Yes |
| 102 | Iyd | 4739 | -2.572 | -0.0105 | Yes |
| 103 | Ddc | 4790 | -3.065 | 0.0064 | Yes |
Table: GSEA details [plain text format]

  

Fig 2: TABULA\_MURIS\_SENIS\_KIDNEY\_EPITHELIAL\_CELL\_OF\_PROXIMAL\_TUBULE\_AGEING: Random ES distribution      
 Gene set null distribution of ES for **TABULA\_MURIS\_SENIS\_KIDNEY\_EPITHELIAL\_CELL\_OF\_PROXIMAL\_TUBULE\_AGEING**

  
