# Supplementary material for: TMPRSS11B promotes an acidified microenvironment and immune suppression in squamous lung cancer
Source: EMBO Rep. 2025 Nov 10;26(24):6346–79. doi: 10.1038/s44319-025-00631-1 (PMC12714794; doi:10.1038/s44319-025-00631-1)
Supplement: Supplementary file 10 — Source data Fig. 5 [file 44319_2025_631_MOESM10_ESM.zip › Figure 5/5C-D/GSEA_Broad Institute_M8_T11b-high LUSC vs LUAD/TABULA_MURIS_SENIS_KIDNEY_MACROPHAGE_AGEING.html]

Details for gene set TABULA\_MURIS\_SENIS\_KIDNEY\_MACROPHAGE\_AGEING[GSEA]

|  || Dataset | Ranked list\_DGE\_squamousT11b\_vs\_all adenosadeno\_HSE13-NT copy |
| Phenotype | NoPhenotypeAvailable |
| Upregulated in class | na\_pos |
| GeneSet | TABULA\_MURIS\_SENIS\_KIDNEY\_MACROPHAGE\_AGEING |
| Enrichment Score (ES) | 0.6421502 |
| Normalized Enrichment Score (NES) | 2.8822973 |
| Nominal p-value | 0.0 |
| FDR q-value | 0.0 |
| FWER p-Value | 0.0 |
Table: GSEA Results Summary

  

Fig 1: Enrichment plot: TABULA\_MURIS\_SENIS\_KIDNEY\_MACROPHAGE\_AGEING      
 Profile of the Running ES Score & Positions of GeneSet Members on the Rank Ordered List

  

| SYMBOL | RANK IN GENE LIST | RANK METRIC SCORE | RUNNING ES | CORE ENRICHMENT || 1 | Ccl9 | 82 | 3.944 | 0.0308 | Yes |
| 2 | Tnfaip2 | 83 | 3.933 | 0.0787 | Yes |
| 3 | Itgam | 132 | 3.250 | 0.1082 | Yes |
| 4 | Trem2 | 140 | 3.156 | 0.1452 | Yes |
| 5 | Cd84 | 159 | 2.936 | 0.1771 | Yes |
| 6 | Ccl6 | 187 | 2.695 | 0.2043 | Yes |
| 7 | Pla2g7 | 195 | 2.599 | 0.2345 | Yes |
| 8 | Lyz1 | 254 | 2.301 | 0.2503 | Yes |
| 9 | Gngt2 | 273 | 2.235 | 0.2737 | Yes |
| 10 | Ctsd | 274 | 2.219 | 0.3008 | Yes |
| 11 | Ly6a | 278 | 2.197 | 0.3269 | Yes |
| 12 | Cd300a | 281 | 2.174 | 0.3529 | Yes |
| 13 | Vim | 302 | 2.105 | 0.3744 | Yes |
| 14 | Cd44 | 329 | 1.976 | 0.3930 | Yes |
| 15 | S100a4 | 353 | 1.867 | 0.4109 | Yes |
| 16 | Rap2b | 398 | 1.689 | 0.4222 | Yes |
| 17 | Cebpb | 425 | 1.620 | 0.4365 | Yes |
| 18 | Cfp | 433 | 1.599 | 0.4545 | Yes |
| 19 | Lgals3 | 447 | 1.559 | 0.4707 | Yes |
| 20 | Klf4 | 448 | 1.555 | 0.4897 | Yes |
| 21 | Alox5ap | 500 | 1.445 | 0.4966 | Yes |
| 22 | Sirpb1c | 515 | 1.407 | 0.5108 | Yes |
| 23 | Pltp | 523 | 1.391 | 0.5262 | Yes |
| 24 | Acp5 | 536 | 1.366 | 0.5403 | Yes |
| 25 | Emp3 | 537 | 1.365 | 0.5570 | Yes |
| 26 | Grn | 554 | 1.328 | 0.5698 | Yes |
| 27 | Capg | 574 | 1.263 | 0.5812 | Yes |
| 28 | Cstb | 587 | 1.229 | 0.5936 | Yes |
| 29 | Prdx5 | 601 | 1.198 | 0.6055 | Yes |
| 30 | Sat1 | 614 | 1.180 | 0.6173 | Yes |
| 31 | Zeb2 | 641 | 1.108 | 0.6254 | Yes |
| 32 | Il2rg | 648 | 1.091 | 0.6374 | Yes |
| 33 | Atp1b3 | 686 | 1.028 | 0.6422 | Yes |
| 34 | Txn1 | 743 | 0.944 | 0.6419 | No |
| 35 | Ninj1 | 833 | 0.831 | 0.6334 | No |
| 36 | Ier3 | 967 | 0.691 | 0.6139 | No |
| 37 | Mcl1 | 969 | 0.689 | 0.6220 | No |
| 38 | Msrb1 | 982 | 0.669 | 0.6277 | No |
| 39 | Pgk1 | 1029 | 0.624 | 0.6256 | No |
| 40 | Ahnak | 1090 | 0.565 | 0.6199 | No |
| 41 | Sod2 | 1120 | 0.540 | 0.6204 | No |
| 42 | Ptpn1 | 1126 | 0.534 | 0.6259 | No |
| 43 | Cmip | 1518 | -0.554 | 0.5506 | No |
| 44 | Agpat4 | 2099 | -0.654 | 0.4369 | No |
| 45 | Socs3 | 2116 | -0.657 | 0.4415 | No |
| 46 | Aldh2 | 2388 | -0.704 | 0.3932 | No |
| 47 | Ly6e | 2739 | -0.775 | 0.3292 | No |
| 48 | Idh1 | 3183 | -0.894 | 0.2472 | No |
| 49 | S100a6 | 3412 | -0.966 | 0.2111 | No |
| 50 | Fos | 3575 | -1.023 | 0.1896 | No |
| 51 | Ezr | 3702 | -1.081 | 0.1763 | No |
| 52 | Gmfg | 4007 | -1.254 | 0.1278 | No |
| 53 | Lmo4 | 4382 | -1.617 | 0.0691 | No |
| 54 | Rida | 4525 | -1.856 | 0.0619 | No |
Table: GSEA details [plain text format]

  

Fig 2: TABULA\_MURIS\_SENIS\_KIDNEY\_MACROPHAGE\_AGEING: Random ES distribution      
 Gene set null distribution of ES for **TABULA\_MURIS\_SENIS\_KIDNEY\_MACROPHAGE\_AGEING**

  
