# Supplementary material for: TMPRSS11B promotes an acidified microenvironment and immune suppression in squamous lung cancer
Source: EMBO Rep. 2025 Nov 10;26(24):6346–79. doi: 10.1038/s44319-025-00631-1 (PMC12714794; doi:10.1038/s44319-025-00631-1)
Supplement: Supplementary file 10 — Source data Fig. 5 [file 44319_2025_631_MOESM10_ESM.zip › Figure 5/5C-D/GSEA_Broad Institute_M8_T11b-high LUSC vs LUAD/TABULA_MURIS_SENIS_LARGE_INTESTINE_ENTEROCYTE_OF_EPITHELIUM_OF_LARGE_INTESTINE_AGEING.html]

Details for gene set TABULA\_MURIS\_SENIS\_LARGE\_INTESTINE\_ENTEROCYTE\_OF\_EPITHELIUM\_OF\_LARGE\_INTESTINE\_AGEING[GSEA]

|  || Dataset | Ranked list\_DGE\_squamousT11b\_vs\_all adenosadeno\_HSE13-NT copy |
| Phenotype | NoPhenotypeAvailable |
| Upregulated in class | na\_neg |
| GeneSet | TABULA\_MURIS\_SENIS\_LARGE\_INTESTINE\_ENTEROCYTE\_OF\_EPITHELIUM\_OF\_LARGE\_INTESTINE\_AGEING |
| Enrichment Score (ES) | -0.15090135 |
| Normalized Enrichment Score (NES) | -1.0414219 |
| Nominal p-value | 0.35454544 |
| FDR q-value | 1.0 |
| FWER p-Value | 1.0 |
Table: GSEA Results Summary

  

Fig 1: Enrichment plot: TABULA\_MURIS\_SENIS\_LARGE\_INTESTINE\_ENTEROCYTE\_OF\_EPITHELIUM\_OF\_LARGE\_INTESTINE\_AGEING      
 Profile of the Running ES Score & Positions of GeneSet Members on the Rank Ordered List

  

| SYMBOL | RANK IN GENE LIST | RANK METRIC SCORE | RUNNING ES | CORE ENRICHMENT || 1 | Ppfia3 | 40 | 4.955 | 0.0047 | No |
| 2 | Gsdmc2 | 41 | 4.896 | 0.0182 | No |
| 3 | Ctsb | 288 | 2.139 | -0.0310 | No |
| 4 | Fth1 | 289 | 2.129 | -0.0251 | No |
| 5 | Nqo1 | 301 | 2.106 | -0.0218 | No |
| 6 | Mif | 323 | 1.991 | -0.0210 | No |
| 7 | Prap1 | 328 | 1.979 | -0.0164 | No |
| 8 | Pglyrp1 | 345 | 1.894 | -0.0148 | No |
| 9 | Bdh1 | 364 | 1.808 | -0.0138 | No |
| 10 | Pgam1 | 381 | 1.756 | -0.0126 | No |
| 11 | Hsd17b2 | 393 | 1.711 | -0.0103 | No |
| 12 | Gsto1 | 397 | 1.701 | -0.0063 | No |
| 13 | Lgals3 | 447 | 1.559 | -0.0130 | No |
| 14 | AA467197 | 452 | 1.544 | -0.0096 | No |
| 15 | Ctsz | 493 | 1.463 | -0.0145 | No |
| 16 | Pycard | 499 | 1.449 | -0.0116 | No |
| 17 | Psap | 510 | 1.415 | -0.0100 | No |
| 18 | Creg1 | 533 | 1.369 | -0.0111 | No |
| 19 | Acp5 | 536 | 1.366 | -0.0078 | No |
| 20 | S100a16 | 569 | 1.288 | -0.0114 | No |
| 21 | Capg | 574 | 1.263 | -0.0088 | No |
| 22 | Esd | 579 | 1.253 | -0.0063 | No |
| 23 | Cstb | 587 | 1.229 | -0.0045 | No |
| 24 | Prdx5 | 601 | 1.198 | -0.0041 | No |
| 25 | Sat1 | 614 | 1.180 | -0.0035 | No |
| 26 | Rab24 | 621 | 1.168 | -0.0016 | No |
| 27 | Hras | 719 | 0.981 | -0.0206 | No |
| 28 | Rtn4 | 726 | 0.971 | -0.0193 | No |
| 29 | Cks2 | 729 | 0.967 | -0.0171 | No |
| 30 | Blvrb | 731 | 0.962 | -0.0147 | No |
| 31 | Ece1 | 739 | 0.953 | -0.0136 | No |
| 32 | Txn1 | 743 | 0.944 | -0.0117 | No |
| 33 | Prelid1 | 751 | 0.932 | -0.0107 | No |
| 34 | B2m | 794 | 0.876 | -0.0177 | No |
| 35 | Gadd45b | 804 | 0.862 | -0.0173 | No |
| 36 | Prnp | 806 | 0.860 | -0.0151 | No |
| 37 | Npc2 | 822 | 0.841 | -0.0162 | No |
| 38 | Gipc1 | 840 | 0.823 | -0.0177 | No |
| 39 | Fam162a | 858 | 0.810 | -0.0193 | No |
| 40 | Ostf1 | 871 | 0.799 | -0.0198 | No |
| 41 | Nfkbia | 887 | 0.772 | -0.0210 | No |
| 42 | Ap2a2 | 891 | 0.767 | -0.0196 | No |
| 43 | Stap2 | 894 | 0.764 | -0.0179 | No |
| 44 | Uba52 | 896 | 0.762 | -0.0160 | No |
| 45 | Atp6v0e | 942 | 0.714 | -0.0241 | No |
| 46 | Atox1 | 997 | 0.651 | -0.0344 | No |
| 47 | Casp1 | 1000 | 0.649 | -0.0331 | No |
| 48 | Stard5 | 1003 | 0.646 | -0.0318 | No |
| 49 | Gstt2 | 1012 | 0.639 | -0.0318 | No |
| 50 | Elovl1 | 1018 | 0.635 | -0.0312 | No |
| 51 | H2-D1 | 1021 | 0.632 | -0.0299 | No |
| 52 | Txndc17 | 1025 | 0.628 | -0.0288 | No |
| 53 | Pgk1 | 1029 | 0.624 | -0.0277 | No |
| 54 | Nudt19 | 1030 | 0.624 | -0.0260 | No |
| 55 | Snrpf | 1089 | 0.566 | -0.0375 | No |
| 56 | Rab11a | 1095 | 0.560 | -0.0370 | No |
| 57 | Tmbim4 | 1103 | 0.550 | -0.0371 | No |
| 58 | Fdx1 | 1106 | 0.548 | -0.0360 | No |
| 59 | Pnp | 1110 | 0.546 | -0.0352 | No |
| 60 | Atp6v1g1 | 1116 | 0.543 | -0.0348 | No |
| 61 | Sod2 | 1120 | 0.540 | -0.0340 | No |
| 62 | Tmem134 | 1128 | 0.530 | -0.0341 | No |
| 63 | Ndufb6 | 1150 | 0.512 | -0.0374 | No |
| 64 | Arpc4 | 1169 | 0.501 | -0.0400 | No |
| 65 | Sar1b | 1170 | -0.500 | -0.0387 | No |
| 66 | Car2 | 1171 | -0.500 | -0.0373 | No |
| 67 | Acaa1a | 1177 | -0.501 | -0.0370 | No |
| 68 | Tpd52l2 | 1188 | -0.503 | -0.0379 | No |
| 69 | Pgp | 1217 | -0.508 | -0.0427 | No |
| 70 | Erg28 | 1231 | -0.510 | -0.0442 | No |
| 71 | Eef1d | 1235 | -0.510 | -0.0435 | No |
| 72 | Zfand2b | 1236 | -0.510 | -0.0421 | No |
| 73 | Brk1 | 1247 | -0.512 | -0.0429 | No |
| 74 | Tm2d2 | 1250 | -0.513 | -0.0420 | No |
| 75 | Nol7 | 1257 | -0.514 | -0.0419 | No |
| 76 | Tmsb4x | 1258 | -0.514 | -0.0405 | No |
| 77 | Grcc10 | 1270 | -0.515 | -0.0415 | No |
| 78 | Map1lc3a | 1273 | -0.516 | -0.0405 | No |
| 79 | Anapc13 | 1293 | -0.518 | -0.0434 | No |
| 80 | Pfdn2 | 1298 | -0.519 | -0.0428 | No |
| 81 | Arl2 | 1302 | -0.519 | -0.0421 | No |
| 82 | Gstp2 | 1318 | -0.521 | -0.0440 | No |
| 83 | Mri1 | 1320 | -0.521 | -0.0428 | No |
| 84 | Tmem11 | 1327 | -0.522 | -0.0427 | No |
| 85 | Sdhaf4 | 1333 | -0.523 | -0.0424 | No |
| 86 | Hnrnpc | 1347 | -0.525 | -0.0438 | No |
| 87 | Unc50 | 1386 | -0.530 | -0.0509 | No |
| 88 | Timm44 | 1402 | -0.532 | -0.0528 | No |
| 89 | Tle5 | 1425 | -0.536 | -0.0562 | No |
| 90 | Ndufv2 | 1437 | -0.539 | -0.0572 | No |
| 91 | Dapk3 | 1439 | -0.539 | -0.0559 | No |
| 92 | Socs2 | 1448 | -0.541 | -0.0562 | No |
| 93 | Srek1ip1 | 1478 | -0.548 | -0.0612 | No |
| 94 | Nipsnap3b | 1548 | -0.560 | -0.0751 | No |
| 95 | Tmem205 | 1549 | -0.560 | -0.0736 | No |
| 96 | Hdac1 | 1550 | -0.560 | -0.0720 | No |
| 97 | Nat9 | 1562 | -0.562 | -0.0729 | No |
| 98 | Rpp21 | 1595 | -0.567 | -0.0785 | No |
| 99 | Ndufs4 | 1608 | -0.570 | -0.0797 | No |
| 100 | Abhd6 | 1614 | -0.571 | -0.0792 | No |
| 101 | Bcap31 | 1622 | -0.572 | -0.0792 | No |
| 102 | Anapc16 | 1632 | -0.573 | -0.0796 | No |
| 103 | Trappc5 | 1635 | -0.573 | -0.0785 | No |
| 104 | Ypel3 | 1644 | -0.575 | -0.0787 | No |
| 105 | Hsp90aa1 | 1647 | -0.575 | -0.0776 | No |
| 106 | Mt1 | 1658 | -0.576 | -0.0782 | No |
| 107 | Sf3b5 | 1659 | -0.576 | -0.0766 | No |
| 108 | Dpy30 | 1664 | -0.577 | -0.0759 | No |
| 109 | Sra1 | 1670 | -0.579 | -0.0755 | No |
| 110 | Hagh | 1673 | -0.579 | -0.0743 | No |
| 111 | Cyb5r3 | 1675 | -0.579 | -0.0729 | No |
| 112 | Pebp1 | 1685 | -0.581 | -0.0733 | No |
| 113 | Sugt1 | 1693 | -0.583 | -0.0733 | No |
| 114 | Mvb12a | 1728 | -0.588 | -0.0793 | No |
| 115 | Emc10 | 1744 | -0.590 | -0.0810 | No |
| 116 | Gemin7 | 1747 | -0.591 | -0.0798 | No |
| 117 | Suclg1 | 1762 | -0.593 | -0.0813 | No |
| 118 | Nudc | 1766 | -0.594 | -0.0804 | No |
| 119 | Cops6 | 1777 | -0.596 | -0.0810 | No |
| 120 | Tpt1 | 1787 | -0.598 | -0.0813 | No |
| 121 | Aprt | 1790 | -0.598 | -0.0801 | No |
| 122 | Micos13 | 1804 | -0.601 | -0.0814 | No |
| 123 | BC031181 | 1805 | -0.601 | -0.0797 | No |
| 124 | Fuca1 | 1814 | -0.603 | -0.0799 | No |
| 125 | Eif3f | 1833 | -0.606 | -0.0822 | No |
| 126 | Spink4 | 1838 | -0.607 | -0.0814 | No |
| 127 | Calm1 | 1839 | -0.607 | -0.0798 | No |
| 128 | Acaa2 | 1846 | -0.609 | -0.0794 | No |
| 129 | Txnl4a | 1868 | -0.613 | -0.0824 | No |
| 130 | Coa3 | 1871 | -0.614 | -0.0812 | No |
| 131 | Emg1 | 1891 | -0.617 | -0.0838 | No |
| 132 | Vdac3 | 1895 | -0.618 | -0.0827 | No |
| 133 | Tmem54 | 1924 | -0.622 | -0.0873 | No |
| 134 | Dnaja1 | 1926 | -0.622 | -0.0858 | No |
| 135 | Pigx | 1936 | -0.624 | -0.0861 | No |
| 136 | Cyb5a | 1942 | -0.625 | -0.0855 | No |
| 137 | Qdpr | 1949 | -0.627 | -0.0851 | No |
| 138 | Polr1c | 1959 | -0.629 | -0.0854 | No |
| 139 | Mpst | 1962 | -0.629 | -0.0841 | No |
| 140 | Rp9 | 1991 | -0.634 | -0.0886 | No |
| 141 | Tmem223 | 1999 | -0.636 | -0.0884 | No |
| 142 | Polr2k | 2071 | -0.648 | -0.1025 | No |
| 143 | Mpv17l2 | 2093 | -0.653 | -0.1054 | No |
| 144 | Ndufs2 | 2103 | -0.655 | -0.1056 | No |
| 145 | Mcee | 2106 | -0.655 | -0.1043 | No |
| 146 | Raly | 2113 | -0.656 | -0.1038 | No |
| 147 | Sin3b | 2139 | -0.661 | -0.1076 | No |
| 148 | Ubl7 | 2146 | -0.662 | -0.1071 | No |
| 149 | Txn2 | 2163 | -0.664 | -0.1089 | No |
| 150 | Alad | 2165 | -0.664 | -0.1073 | No |
| 151 | H13 | 2183 | -0.667 | -0.1092 | No |
| 152 | Tex261 | 2206 | -0.672 | -0.1123 | No |
| 153 | Gnb2 | 2210 | -0.673 | -0.1111 | No |
| 154 | Gstt3 | 2225 | -0.675 | -0.1124 | No |
| 155 | Fmc1 | 2232 | -0.677 | -0.1119 | No |
| 156 | Arl6ip5 | 2311 | -0.690 | -0.1274 | No |
| 157 | Fam98c | 2335 | -0.695 | -0.1307 | No |
| 158 | Spag7 | 2338 | -0.695 | -0.1292 | No |
| 159 | Ndufa7 | 2344 | -0.696 | -0.1284 | No |
| 160 | Churc1 | 2345 | -0.696 | -0.1265 | No |
| 161 | Ywhaq | 2346 | -0.697 | -0.1246 | No |
| 162 | Polr2e | 2361 | -0.699 | -0.1258 | No |
| 163 | Dnlz | 2368 | -0.700 | -0.1252 | No |
| 164 | 2610528J11Rik | 2396 | -0.705 | -0.1293 | No |
| 165 | Sdc4 | 2418 | -0.710 | -0.1320 | No |
| 166 | Yipf3 | 2423 | -0.711 | -0.1310 | No |
| 167 | Naxd | 2426 | -0.712 | -0.1294 | No |
| 168 | Rab4b | 2428 | -0.712 | -0.1277 | No |
| 169 | Smagp | 2447 | -0.717 | -0.1298 | No |
| 170 | Idh3g | 2455 | -0.719 | -0.1293 | No |
| 171 | Dmbt1 | 2466 | -0.721 | -0.1296 | No |
| 172 | Hmgcl | 2468 | -0.721 | -0.1278 | No |
| 173 | Pnkd | 2487 | -0.726 | -0.1299 | No |
| 174 | Acads | 2492 | -0.727 | -0.1288 | No |
| 175 | Shisa5 | 2493 | -0.727 | -0.1267 | No |
| 176 | Polr2c | 2515 | -0.730 | -0.1294 | No |
| 177 | Guk1 | 2516 | -0.730 | -0.1274 | No |
| 178 | Tmem33 | 2536 | -0.734 | -0.1297 | No |
| 179 | Cib1 | 2552 | -0.737 | -0.1310 | No |
| 180 | Calm3 | 2555 | -0.737 | -0.1294 | No |
| 181 | Nt5c | 2556 | -0.737 | -0.1274 | No |
| 182 | Kxd1 | 2559 | -0.739 | -0.1258 | No |
| 183 | Smim14 | 2579 | -0.744 | -0.1280 | No |
| 184 | Krtcap2 | 2586 | -0.744 | -0.1273 | No |
| 185 | Selenos | 2616 | -0.752 | -0.1317 | No |
| 186 | Ndufa9 | 2646 | -0.758 | -0.1361 | No |
| 187 | Fbp2 | 2651 | -0.759 | -0.1349 | No |
| 188 | Zfpl1 | 2674 | -0.763 | -0.1377 | No |
| 189 | Polr2i | 2682 | -0.764 | -0.1372 | No |
| 190 | Sod1 | 2688 | -0.765 | -0.1362 | No |
| 191 | Ppa1 | 2690 | -0.766 | -0.1343 | No |
| 192 | Msra | 2696 | -0.767 | -0.1333 | No |
| 193 | Tmem208 | 2697 | -0.767 | -0.1312 | No |
| 194 | Tmem171 | 2705 | -0.768 | -0.1306 | No |
| 195 | Ccs | 2710 | -0.769 | -0.1294 | No |
| 196 | 2510002D24Rik | 2723 | -0.771 | -0.1300 | No |
| 197 | Cnpy2 | 2726 | -0.772 | -0.1283 | No |
| 198 | Nans | 2757 | -0.781 | -0.1329 | No |
| 199 | Bsg | 2764 | -0.783 | -0.1320 | No |
| 200 | Atraid | 2778 | -0.786 | -0.1328 | No |
| 201 | Hcfc1r1 | 2783 | -0.787 | -0.1315 | No |
| 202 | Ddt | 2787 | -0.788 | -0.1300 | No |
| 203 | Bola1 | 2805 | -0.791 | -0.1316 | No |
| 204 | Ciao2a | 2807 | -0.791 | -0.1297 | No |
| 205 | Iah1 | 2825 | -0.796 | -0.1313 | No |
| 206 | Gstk1 | 2827 | -0.797 | -0.1293 | No |
| 207 | Smim22 | 2845 | -0.801 | -0.1309 | No |
| 208 | Cox16 | 2870 | -0.808 | -0.1341 | No |
| 209 | Tex264 | 2874 | -0.809 | -0.1325 | No |
| 210 | Pmm1 | 2876 | -0.809 | -0.1305 | No |
| 211 | Ifi27 | 2905 | -0.815 | -0.1345 | No |
| 212 | 2210016L21Rik | 2913 | -0.817 | -0.1338 | No |
| 213 | Timm8b | 2968 | -0.831 | -0.1436 | No |
| 214 | Fh1 | 2972 | -0.834 | -0.1420 | No |
| 215 | Tmco1 | 2982 | -0.837 | -0.1417 | No |
| 216 | Gtf3c6 | 2983 | -0.837 | -0.1394 | No |
| 217 | Nsa2 | 2989 | -0.838 | -0.1382 | No |
| 218 | Hmgb1 | 2990 | -0.838 | -0.1359 | No |
| 219 | Gadd45gip1 | 3033 | -0.850 | -0.1430 | No |
| 220 | Dpm1 | 3067 | -0.859 | -0.1480 | No |
| 221 | S100a1 | 3077 | -0.864 | -0.1476 | No |
| 222 | Idh3b | 3079 | -0.865 | -0.1455 | No |
| 223 | Tmem147 | 3100 | -0.871 | -0.1475 | No |
| 224 | Eif4e2 | 3116 | -0.876 | -0.1485 | Yes |
| 225 | Yipf1 | 3121 | -0.877 | -0.1470 | Yes |
| 226 | Smim24 | 3124 | -0.878 | -0.1450 | Yes |
| 227 | Hsd17b10 | 3144 | -0.883 | -0.1468 | Yes |
| 228 | Clybl | 3145 | -0.883 | -0.1444 | Yes |
| 229 | Cirbp | 3148 | -0.884 | -0.1424 | Yes |
| 230 | Gna11 | 3165 | -0.889 | -0.1435 | Yes |
| 231 | Vasp | 3174 | -0.891 | -0.1428 | Yes |
| 232 | Idh1 | 3183 | -0.894 | -0.1422 | Yes |
| 233 | Fkbp1a | 3188 | -0.895 | -0.1406 | Yes |
| 234 | Spint2 | 3189 | -0.895 | -0.1381 | Yes |
| 235 | Zfp706 | 3191 | -0.896 | -0.1359 | Yes |
| 236 | Mif4gd | 3208 | -0.902 | -0.1370 | Yes |
| 237 | Chchd7 | 3229 | -0.909 | -0.1390 | Yes |
| 238 | Adh5 | 3239 | -0.911 | -0.1385 | Yes |
| 239 | Uqcc3 | 3246 | -0.914 | -0.1373 | Yes |
| 240 | Ndufb8 | 3274 | -0.922 | -0.1408 | Yes |
| 241 | Surf1 | 3279 | -0.924 | -0.1391 | Yes |
| 242 | Ddrgk1 | 3282 | -0.924 | -0.1370 | Yes |
| 243 | Ppa2 | 3287 | -0.925 | -0.1354 | Yes |
| 244 | Gstm5 | 3294 | -0.927 | -0.1342 | Yes |
| 245 | Akr1e1 | 3295 | -0.929 | -0.1316 | Yes |
| 246 | Naxe | 3301 | -0.931 | -0.1302 | Yes |
| 247 | Tmed4 | 3329 | -0.939 | -0.1336 | Yes |
| 248 | Fkbp4 | 3332 | -0.940 | -0.1315 | Yes |
| 249 | Sqor | 3336 | -0.941 | -0.1295 | Yes |
| 250 | Hint2 | 3347 | -0.946 | -0.1292 | Yes |
| 251 | Mdp1 | 3355 | -0.949 | -0.1281 | Yes |
| 252 | Fcgrt | 3361 | -0.950 | -0.1266 | Yes |
| 253 | Mlec | 3380 | -0.956 | -0.1280 | Yes |
| 254 | Tmem59 | 3397 | -0.960 | -0.1290 | Yes |
| 255 | Sdhd | 3399 | -0.960 | -0.1265 | Yes |
| 256 | Acot13 | 3414 | -0.967 | -0.1270 | Yes |
| 257 | Lgr4 | 3436 | -0.977 | -0.1290 | Yes |
| 258 | Dnajc4 | 3459 | -0.984 | -0.1312 | Yes |
| 259 | Ces1d | 3471 | -0.987 | -0.1310 | Yes |
| 260 | Asl | 3473 | -0.988 | -0.1285 | Yes |
| 261 | Rnf186 | 3474 | -0.988 | -0.1257 | Yes |
| 262 | Zmat5 | 3484 | -0.991 | -0.1250 | Yes |
| 263 | Nudt14 | 3503 | -0.998 | -0.1263 | Yes |
| 264 | Aamdc | 3547 | -1.013 | -0.1331 | Yes |
| 265 | Cenpx | 3601 | -1.035 | -0.1422 | Yes |
| 266 | Cbr1 | 3607 | -1.035 | -0.1404 | Yes |
| 267 | Mt2 | 3612 | -1.035 | -0.1385 | Yes |
| 268 | Thap4 | 3631 | -1.044 | -0.1396 | Yes |
| 269 | Lgals9 | 3638 | -1.047 | -0.1381 | Yes |
| 270 | Galk1 | 3646 | -1.051 | -0.1367 | Yes |
| 271 | Plpp2 | 3671 | -1.064 | -0.1392 | Yes |
| 272 | Tsc22d1 | 3676 | -1.066 | -0.1371 | Yes |
| 273 | Decr1 | 3683 | -1.070 | -0.1355 | Yes |
| 274 | Krtcap3 | 3697 | -1.078 | -0.1355 | Yes |
| 275 | Ech1 | 3705 | -1.083 | -0.1340 | Yes |
| 276 | Sri | 3726 | -1.092 | -0.1355 | Yes |
| 277 | Macrod1 | 3732 | -1.095 | -0.1336 | Yes |
| 278 | Srsf3 | 3744 | -1.100 | -0.1330 | Yes |
| 279 | 2310039H08Rik | 3763 | -1.108 | -0.1340 | Yes |
| 280 | Fahd1 | 3787 | -1.119 | -0.1361 | Yes |
| 281 | Pigr | 3833 | -1.149 | -0.1430 | Yes |
| 282 | Mcrip2 | 3863 | -1.163 | -0.1463 | Yes |
| 283 | Bag1 | 3870 | -1.165 | -0.1444 | Yes |
| 284 | Saysd1 | 3876 | -1.168 | -0.1423 | Yes |
| 285 | Gnpnat1 | 3884 | -1.177 | -0.1406 | Yes |
| 286 | Dynll2 | 3904 | -1.185 | -0.1416 | Yes |
| 287 | Bri3 | 3905 | -1.186 | -0.1383 | Yes |
| 288 | Pigp | 3918 | -1.195 | -0.1377 | Yes |
| 289 | Ppdpf | 3930 | -1.203 | -0.1369 | Yes |
| 290 | Mea1 | 3947 | -1.215 | -0.1371 | Yes |
| 291 | Hadh | 3948 | -1.215 | -0.1338 | Yes |
| 292 | Dcxr | 3966 | -1.224 | -0.1342 | Yes |
| 293 | Mpnd | 3971 | -1.227 | -0.1317 | Yes |
| 294 | Wbp1 | 3986 | -1.239 | -0.1314 | Yes |
| 295 | Cdc42ep5 | 4009 | -1.255 | -0.1329 | Yes |
| 296 | Pts | 4078 | -1.307 | -0.1445 | Yes |
| 297 | Prelid2 | 4107 | -1.334 | -0.1471 | Yes |
| 298 | Sult1a1 | 4112 | -1.339 | -0.1443 | Yes |
| 299 | Pllp | 4119 | -1.348 | -0.1419 | Yes |
| 300 | Tstd1 | 4139 | -1.362 | -0.1424 | Yes |
| 301 | Gtf2a2 | 4156 | -1.374 | -0.1422 | Yes |
| 302 | Mettl26 | 4185 | -1.397 | -0.1447 | Yes |
| 303 | Gstm1 | 4200 | -1.410 | -0.1439 | Yes |
| 304 | Gmds | 4203 | -1.412 | -0.1405 | Yes |
| 305 | Akr7a5 | 4207 | -1.413 | -0.1372 | Yes |
| 306 | Cystm1 | 4215 | -1.424 | -0.1349 | Yes |
| 307 | Ccdc107 | 4218 | -1.430 | -0.1314 | Yes |
| 308 | Vsig2 | 4221 | -1.434 | -0.1279 | Yes |
| 309 | Aqp11 | 4256 | -1.470 | -0.1314 | Yes |
| 310 | Gpd1 | 4257 | -1.470 | -0.1274 | Yes |
| 311 | Cisd3 | 4271 | -1.480 | -0.1262 | Yes |
| 312 | Ppcs | 4272 | -1.480 | -0.1221 | Yes |
| 313 | Spr | 4273 | -1.481 | -0.1181 | Yes |
| 314 | Fermt1 | 4276 | -1.484 | -0.1144 | Yes |
| 315 | Tmem45b | 4279 | -1.487 | -0.1108 | Yes |
| 316 | Gipc2 | 4295 | -1.500 | -0.1100 | Yes |
| 317 | Slc22a18 | 4312 | -1.515 | -0.1094 | Yes |
| 318 | Ociad2 | 4322 | -1.535 | -0.1072 | Yes |
| 319 | Mkrn2os | 4341 | -1.558 | -0.1069 | Yes |
| 320 | Cgref1 | 4354 | -1.583 | -0.1052 | Yes |
| 321 | Pafah1b3 | 4356 | -1.584 | -0.1011 | Yes |
| 322 | Cnnm4 | 4367 | -1.597 | -0.0989 | Yes |
| 323 | Lmo4 | 4382 | -1.617 | -0.0976 | Yes |
| 324 | Espn | 4392 | -1.628 | -0.0951 | Yes |
| 325 | Bad | 4410 | -1.663 | -0.0943 | Yes |
| 326 | Cmbl | 4433 | -1.707 | -0.0946 | Yes |
| 327 | Tspan8 | 4446 | -1.740 | -0.0924 | Yes |
| 328 | Rab4a | 4465 | -1.760 | -0.0916 | Yes |
| 329 | Khk | 4487 | -1.787 | -0.0914 | Yes |
| 330 | Csrp2 | 4490 | -1.794 | -0.0869 | Yes |
| 331 | Krt19 | 4510 | -1.837 | -0.0861 | Yes |
| 332 | Abhd14b | 4513 | -1.840 | -0.0815 | Yes |
| 333 | Cela1 | 4538 | -1.888 | -0.0816 | Yes |
| 334 | Gm3336 | 4540 | -1.890 | -0.0766 | Yes |
| 335 | Klf5 | 4543 | -1.893 | -0.0719 | Yes |
| 336 | Tcea3 | 4554 | -1.926 | -0.0688 | Yes |
| 337 | Cideb | 4591 | -2.024 | -0.0713 | Yes |
| 338 | Car9 | 4594 | -2.031 | -0.0661 | Yes |
| 339 | Sult1b1 | 4616 | -2.083 | -0.0651 | Yes |
| 340 | Akr1c13 | 4632 | -2.122 | -0.0626 | Yes |
| 341 | Fa2h | 4634 | -2.134 | -0.0569 | Yes |
| 342 | Ifi27l2b | 4639 | -2.143 | -0.0519 | Yes |
| 343 | Mgst2 | 4641 | -2.150 | -0.0462 | Yes |
| 344 | Tmem98 | 4651 | -2.187 | -0.0422 | Yes |
| 345 | Degs2 | 4665 | -2.227 | -0.0390 | Yes |
| 346 | Agr2 | 4671 | -2.265 | -0.0338 | Yes |
| 347 | Lurap1l | 4679 | -2.307 | -0.0291 | Yes |
| 348 | Hmgcs2 | 4714 | -2.435 | -0.0300 | Yes |
| 349 | Ces1f | 4723 | -2.490 | -0.0249 | Yes |
| 350 | Akr1c12 | 4726 | -2.499 | -0.0184 | Yes |
| 351 | Akr1c19 | 4742 | -2.597 | -0.0146 | Yes |
| 352 | Adh1 | 4746 | -2.614 | -0.0081 | Yes |
| 353 | Lgals4 | 4748 | -2.635 | -0.0011 | Yes |
| 354 | Paqr5 | 4787 | -3.012 | -0.0013 | Yes |
| 355 | Il18 | 4789 | -3.056 | 0.0069 | Yes |
Table: GSEA details [plain text format]

  

Fig 2: TABULA\_MURIS\_SENIS\_LARGE\_INTESTINE\_ENTEROCYTE\_OF\_EPITHELIUM\_OF\_LARGE\_INTESTINE\_AGEING: Random ES distribution      
 Gene set null distribution of ES for **TABULA\_MURIS\_SENIS\_LARGE\_INTESTINE\_ENTEROCYTE\_OF\_EPITHELIUM\_OF\_LARGE\_INTESTINE\_AGEING**

  
