# Supplementary material for: TMPRSS11B promotes an acidified microenvironment and immune suppression in squamous lung cancer
Source: EMBO Rep. 2025 Nov 10;26(24):6346–79. doi: 10.1038/s44319-025-00631-1 (PMC12714794; doi:10.1038/s44319-025-00631-1)
Supplement: Supplementary file 10 — Source data Fig. 5 [file 44319_2025_631_MOESM10_ESM.zip › Figure 5/5C-D/GSEA_Broad Institute_M8_T11b-high LUSC vs LUAD/TABULA_MURIS_SENIS_LARGE_INTESTINE_INTESTINAL_CRYPT_STEM_CELL_AGEING.html]

Details for gene set TABULA\_MURIS\_SENIS\_LARGE\_INTESTINE\_INTESTINAL\_CRYPT\_STEM\_CELL\_AGEING[GSEA]

|  || Dataset | Ranked list\_DGE\_squamousT11b\_vs\_all adenosadeno\_HSE13-NT copy |
| Phenotype | NoPhenotypeAvailable |
| Upregulated in class | na\_neg |
| GeneSet | TABULA\_MURIS\_SENIS\_LARGE\_INTESTINE\_INTESTINAL\_CRYPT\_STEM\_CELL\_AGEING |
| Enrichment Score (ES) | -0.17471626 |
| Normalized Enrichment Score (NES) | -0.9880003 |
| Nominal p-value | 0.48554912 |
| FDR q-value | 1.0 |
| FWER p-Value | 1.0 |
Table: GSEA Results Summary

  

Fig 1: Enrichment plot: TABULA\_MURIS\_SENIS\_LARGE\_INTESTINE\_INTESTINAL\_CRYPT\_STEM\_CELL\_AGEING      
 Profile of the Running ES Score & Positions of GeneSet Members on the Rank Ordered List

  

| SYMBOL | RANK IN GENE LIST | RANK METRIC SCORE | RUNNING ES | CORE ENRICHMENT || 1 | Lypd3 | 15 | 6.488 | 0.0533 | No |
| 2 | Mfge8 | 208 | 2.523 | 0.0346 | No |
| 3 | S100a14 | 253 | 2.301 | 0.0453 | No |
| 4 | Fth1 | 289 | 2.129 | 0.0564 | No |
| 5 | Pglyrp1 | 345 | 1.894 | 0.0612 | No |
| 6 | Rgs1 | 350 | 1.874 | 0.0767 | No |
| 7 | Ctsz | 493 | 1.463 | 0.0593 | No |
| 8 | S100a16 | 569 | 1.288 | 0.0547 | No |
| 9 | Trf | 682 | 1.033 | 0.0399 | No |
| 10 | Mal | 733 | 0.961 | 0.0377 | No |
| 11 | Ece1 | 739 | 0.953 | 0.0449 | No |
| 12 | Gadd45b | 804 | 0.862 | 0.0389 | No |
| 13 | Ninj1 | 833 | 0.831 | 0.0402 | No |
| 14 | Gipc1 | 840 | 0.823 | 0.0461 | No |
| 15 | Rgcc | 943 | 0.713 | 0.0307 | No |
| 16 | Ier3 | 967 | 0.691 | 0.0318 | No |
| 17 | Pkm | 970 | 0.686 | 0.0373 | No |
| 18 | H2-D1 | 1021 | 0.632 | 0.0323 | No |
| 19 | Stmn1 | 1124 | 0.536 | 0.0153 | No |
| 20 | Arpc4 | 1169 | 0.501 | 0.0103 | No |
| 21 | Kdm6b | 1191 | -0.504 | 0.0103 | No |
| 22 | Sgf29 | 1192 | -0.504 | 0.0147 | No |
| 23 | Pgp | 1217 | -0.508 | 0.0140 | No |
| 24 | Gsta4 | 1227 | -0.509 | 0.0165 | No |
| 25 | Eef1d | 1235 | -0.510 | 0.0195 | No |
| 26 | Mri1 | 1320 | -0.521 | 0.0062 | No |
| 27 | Timm44 | 1402 | -0.532 | -0.0063 | No |
| 28 | Tle5 | 1425 | -0.536 | -0.0063 | No |
| 29 | Socs2 | 1448 | -0.541 | -0.0063 | No |
| 30 | Mgst1 | 1519 | -0.554 | -0.0163 | No |
| 31 | Nt5c3b | 1651 | -0.575 | -0.0390 | No |
| 32 | Pebp1 | 1685 | -0.581 | -0.0410 | No |
| 33 | Vps72 | 1737 | -0.589 | -0.0467 | No |
| 34 | Emc10 | 1744 | -0.590 | -0.0428 | No |
| 35 | Eif3f | 1833 | -0.606 | -0.0562 | No |
| 36 | Calm1 | 1839 | -0.607 | -0.0519 | No |
| 37 | Emg1 | 1891 | -0.617 | -0.0574 | No |
| 38 | Cd248 | 2110 | -0.655 | -0.0979 | No |
| 39 | Raly | 2113 | -0.656 | -0.0926 | No |
| 40 | Ubl7 | 2146 | -0.662 | -0.0936 | No |
| 41 | Txn2 | 2163 | -0.664 | -0.0912 | No |
| 42 | Cdpf1 | 2197 | -0.670 | -0.0924 | No |
| 43 | Tex261 | 2206 | -0.672 | -0.0882 | No |
| 44 | Ptgr1 | 2287 | -0.685 | -0.0992 | No |
| 45 | Fam98c | 2335 | -0.695 | -0.1031 | No |
| 46 | 2610528J11Rik | 2396 | -0.705 | -0.1097 | No |
| 47 | Sdc4 | 2418 | -0.710 | -0.1080 | No |
| 48 | Naxd | 2426 | -0.712 | -0.1033 | No |
| 49 | Smagp | 2447 | -0.717 | -0.1013 | No |
| 50 | Osgep | 2499 | -0.728 | -0.1057 | No |
| 51 | Guk1 | 2516 | -0.730 | -0.1028 | No |
| 52 | Nt5c | 2556 | -0.737 | -0.1046 | No |
| 53 | Zfpl1 | 2674 | -0.763 | -0.1228 | No |
| 54 | 2510002D24Rik | 2723 | -0.771 | -0.1262 | No |
| 55 | Aarsd1 | 2738 | -0.775 | -0.1225 | No |
| 56 | Ly6e | 2739 | -0.775 | -0.1157 | No |
| 57 | Bsg | 2764 | -0.783 | -0.1140 | No |
| 58 | Sfxn1 | 2864 | -0.806 | -0.1279 | No |
| 59 | Pmm1 | 2876 | -0.809 | -0.1232 | No |
| 60 | 2210016L21Rik | 2913 | -0.817 | -0.1237 | No |
| 61 | Hmgb1 | 2990 | -0.838 | -0.1326 | No |
| 62 | Gadd45gip1 | 3033 | -0.850 | -0.1341 | No |
| 63 | Gjb1 | 3084 | -0.866 | -0.1371 | No |
| 64 | Dcps | 3130 | -0.879 | -0.1390 | No |
| 65 | Hsd17b10 | 3144 | -0.883 | -0.1341 | No |
| 66 | Cirbp | 3148 | -0.884 | -0.1270 | No |
| 67 | Spint2 | 3189 | -0.895 | -0.1277 | No |
| 68 | Ddrgk1 | 3282 | -0.924 | -0.1391 | No |
| 69 | Tmed4 | 3329 | -0.939 | -0.1407 | No |
| 70 | Fam241b | 3349 | -0.948 | -0.1365 | No |
| 71 | Lsr | 3431 | -0.976 | -0.1452 | No |
| 72 | Mecr | 3462 | -0.985 | -0.1429 | No |
| 73 | Asl | 3473 | -0.988 | -0.1365 | No |
| 74 | Rnf186 | 3474 | -0.988 | -0.1279 | No |
| 75 | Zmat5 | 3484 | -0.991 | -0.1211 | No |
| 76 | Cdk5rap3 | 3593 | -1.030 | -0.1351 | No |
| 77 | Krtcap3 | 3697 | -1.078 | -0.1475 | No |
| 78 | Foxa3 | 3746 | -1.101 | -0.1481 | No |
| 79 | Fahd1 | 3787 | -1.119 | -0.1468 | No |
| 80 | Smco4 | 3805 | -1.131 | -0.1406 | No |
| 81 | Dynll2 | 3904 | -1.185 | -0.1510 | No |
| 82 | Bri3 | 3905 | -1.186 | -0.1407 | No |
| 83 | Cdc42ep5 | 4009 | -1.255 | -0.1516 | No |
| 84 | Pllp | 4119 | -1.348 | -0.1630 | Yes |
| 85 | Qtrt1 | 4153 | -1.373 | -0.1580 | Yes |
| 86 | Tmem9 | 4164 | -1.381 | -0.1481 | Yes |
| 87 | Ptov1 | 4177 | -1.389 | -0.1386 | Yes |
| 88 | Mettl26 | 4185 | -1.397 | -0.1279 | Yes |
| 89 | Hes6 | 4191 | -1.404 | -0.1167 | Yes |
| 90 | Akr7a5 | 4207 | -1.413 | -0.1076 | Yes |
| 91 | Vsig2 | 4221 | -1.434 | -0.0979 | Yes |
| 92 | Ccnd1 | 4229 | -1.443 | -0.0868 | Yes |
| 93 | Spr | 4273 | -1.481 | -0.0830 | Yes |
| 94 | Fermt1 | 4276 | -1.484 | -0.0705 | Yes |
| 95 | Pafah1b3 | 4356 | -1.584 | -0.0735 | Yes |
| 96 | Cela1 | 4538 | -1.888 | -0.0954 | Yes |
| 97 | Tff3 | 4571 | -1.966 | -0.0850 | Yes |
| 98 | Sdsl | 4661 | -2.212 | -0.0846 | Yes |
| 99 | Akr1c12 | 4726 | -2.499 | -0.0764 | Yes |
| 100 | Adh1 | 4746 | -2.614 | -0.0577 | Yes |
| 101 | Krt20 | 4767 | -2.756 | -0.0380 | Yes |
| 102 | Ppp1r1b | 4771 | -2.786 | -0.0143 | Yes |
| 103 | Clu | 4775 | -2.816 | 0.0095 | Yes |
Table: GSEA details [plain text format]

  

Fig 2: TABULA\_MURIS\_SENIS\_LARGE\_INTESTINE\_INTESTINAL\_CRYPT\_STEM\_CELL\_AGEING: Random ES distribution      
 Gene set null distribution of ES for **TABULA\_MURIS\_SENIS\_LARGE\_INTESTINE\_INTESTINAL\_CRYPT\_STEM\_CELL\_AGEING**

  
