# Supplementary material for: TMPRSS11B promotes an acidified microenvironment and immune suppression in squamous lung cancer
Source: EMBO Rep. 2025 Nov 10;26(24):6346–79. doi: 10.1038/s44319-025-00631-1 (PMC12714794; doi:10.1038/s44319-025-00631-1)
Supplement: Supplementary file 10 — Source data Fig. 5 [file 44319_2025_631_MOESM10_ESM.zip › Figure 5/5C-D/GSEA_Broad Institute_M8_T11b-high LUSC vs LUAD/TABULA_MURIS_SENIS_LARGE_INTESTINE_LARGE_INTESTINE_GOBLET_CELL_AGEING.html]

Details for gene set TABULA\_MURIS\_SENIS\_LARGE\_INTESTINE\_LARGE\_INTESTINE\_GOBLET\_CELL\_AGEING[GSEA]

|  || Dataset | Ranked list\_DGE\_squamousT11b\_vs\_all adenosadeno\_HSE13-NT copy |
| Phenotype | NoPhenotypeAvailable |
| Upregulated in class | na\_neg |
| GeneSet | TABULA\_MURIS\_SENIS\_LARGE\_INTESTINE\_LARGE\_INTESTINE\_GOBLET\_CELL\_AGEING |
| Enrichment Score (ES) | -0.17035066 |
| Normalized Enrichment Score (NES) | -1.1595203 |
| Nominal p-value | 0.15129152 |
| FDR q-value | 1.0 |
| FWER p-Value | 1.0 |
Table: GSEA Results Summary

  

Fig 1: Enrichment plot: TABULA\_MURIS\_SENIS\_LARGE\_INTESTINE\_LARGE\_INTESTINE\_GOBLET\_CELL\_AGEING      
 Profile of the Running ES Score & Positions of GeneSet Members on the Rank Ordered List

  

| SYMBOL | RANK IN GENE LIST | RANK METRIC SCORE | RUNNING ES | CORE ENRICHMENT || 1 | Plet1 | 190 | 2.674 | -0.0310 | No |
| 2 | S100a14 | 253 | 2.301 | -0.0355 | No |
| 3 | Ctsb | 288 | 2.139 | -0.0345 | No |
| 4 | Fth1 | 289 | 2.129 | -0.0261 | No |
| 5 | Mif | 323 | 1.991 | -0.0255 | No |
| 6 | Dusp1 | 339 | 1.923 | -0.0211 | No |
| 7 | Pglyrp1 | 345 | 1.894 | -0.0148 | No |
| 8 | Gsto1 | 397 | 1.701 | -0.0192 | No |
| 9 | Urah | 476 | 1.500 | -0.0304 | No |
| 10 | Ctsz | 493 | 1.463 | -0.0281 | No |
| 11 | Pycard | 499 | 1.449 | -0.0234 | No |
| 12 | Psap | 510 | 1.415 | -0.0200 | No |
| 13 | Acp5 | 536 | 1.366 | -0.0201 | No |
| 14 | Hilpda | 552 | 1.330 | -0.0181 | No |
| 15 | S100a16 | 569 | 1.288 | -0.0166 | No |
| 16 | Prdx5 | 601 | 1.198 | -0.0186 | No |
| 17 | Ctnnbip1 | 677 | 1.039 | -0.0309 | No |
| 18 | Txn1 | 743 | 0.944 | -0.0414 | No |
| 19 | Prelid1 | 751 | 0.932 | -0.0393 | No |
| 20 | B2m | 794 | 0.876 | -0.0450 | No |
| 21 | Gadd45b | 804 | 0.862 | -0.0436 | No |
| 22 | Npc2 | 822 | 0.841 | -0.0440 | No |
| 23 | Gipc1 | 840 | 0.823 | -0.0444 | No |
| 24 | Phldb3 | 851 | 0.812 | -0.0434 | No |
| 25 | Fkbp11 | 864 | 0.807 | -0.0429 | No |
| 26 | Nfkbia | 887 | 0.772 | -0.0446 | No |
| 27 | Stap2 | 894 | 0.764 | -0.0429 | No |
| 28 | Ier3 | 967 | 0.691 | -0.0560 | No |
| 29 | Pkm | 970 | 0.686 | -0.0537 | No |
| 30 | Atox1 | 997 | 0.651 | -0.0568 | No |
| 31 | Gstt2 | 1012 | 0.639 | -0.0574 | No |
| 32 | Elovl1 | 1018 | 0.635 | -0.0559 | No |
| 33 | H2-D1 | 1021 | 0.632 | -0.0539 | No |
| 34 | Txndc17 | 1025 | 0.628 | -0.0521 | No |
| 35 | Ehd4 | 1053 | 0.607 | -0.0556 | No |
| 36 | Nupr1 | 1092 | 0.562 | -0.0617 | No |
| 37 | Tmbim4 | 1103 | 0.550 | -0.0617 | No |
| 38 | Atp6v1g1 | 1116 | 0.543 | -0.0622 | No |
| 39 | Sod2 | 1120 | 0.540 | -0.0607 | No |
| 40 | Ndufb6 | 1150 | 0.512 | -0.0650 | No |
| 41 | Ppp1r2 | 1160 | 0.505 | -0.0650 | No |
| 42 | Gng11 | 1167 | 0.502 | -0.0643 | No |
| 43 | Arpc4 | 1169 | 0.501 | -0.0626 | No |
| 44 | Sar1b | 1170 | -0.500 | -0.0606 | No |
| 45 | Car2 | 1171 | -0.500 | -0.0586 | No |
| 46 | Tpd52l2 | 1188 | -0.503 | -0.0601 | No |
| 47 | Pgp | 1217 | -0.508 | -0.0642 | No |
| 48 | Eef1d | 1235 | -0.510 | -0.0659 | No |
| 49 | Zfand2b | 1236 | -0.510 | -0.0639 | No |
| 50 | Brk1 | 1247 | -0.512 | -0.0641 | No |
| 51 | Tm2d2 | 1250 | -0.513 | -0.0625 | No |
| 52 | Tmsb4x | 1258 | -0.514 | -0.0620 | No |
| 53 | Grcc10 | 1270 | -0.515 | -0.0624 | No |
| 54 | Map1lc3a | 1273 | -0.516 | -0.0608 | No |
| 55 | Pfdn2 | 1298 | -0.519 | -0.0640 | No |
| 56 | Gstp2 | 1318 | -0.521 | -0.0661 | No |
| 57 | Mri1 | 1320 | -0.521 | -0.0642 | No |
| 58 | Tmem176a | 1324 | -0.522 | -0.0628 | No |
| 59 | Tmem176b | 1326 | -0.522 | -0.0610 | No |
| 60 | Tmem11 | 1327 | -0.522 | -0.0589 | No |
| 61 | Unc50 | 1386 | -0.530 | -0.0695 | No |
| 62 | Tle5 | 1425 | -0.536 | -0.0757 | No |
| 63 | Ndufv2 | 1437 | -0.539 | -0.0760 | No |
| 64 | Plaat3 | 1505 | -0.552 | -0.0885 | No |
| 65 | Dtymk | 1507 | -0.552 | -0.0866 | No |
| 66 | Nectin2 | 1531 | -0.558 | -0.0894 | No |
| 67 | Tmem205 | 1549 | -0.560 | -0.0909 | No |
| 68 | Ndufs4 | 1608 | -0.570 | -0.1013 | No |
| 69 | Anapc16 | 1632 | -0.573 | -0.1041 | No |
| 70 | Ypel3 | 1644 | -0.575 | -0.1043 | No |
| 71 | Sf3b5 | 1659 | -0.576 | -0.1050 | No |
| 72 | Hagh | 1673 | -0.579 | -0.1056 | No |
| 73 | Cyb5r3 | 1675 | -0.579 | -0.1035 | No |
| 74 | Pebp1 | 1685 | -0.581 | -0.1032 | No |
| 75 | Mvb12a | 1728 | -0.588 | -0.1101 | No |
| 76 | Vps72 | 1737 | -0.589 | -0.1095 | No |
| 77 | Emc10 | 1744 | -0.590 | -0.1085 | No |
| 78 | Tcf7l1 | 1746 | -0.590 | -0.1064 | No |
| 79 | Suclg1 | 1762 | -0.593 | -0.1073 | No |
| 80 | Tpt1 | 1787 | -0.598 | -0.1102 | No |
| 81 | Micos13 | 1804 | -0.601 | -0.1113 | No |
| 82 | BC031181 | 1805 | -0.601 | -0.1090 | No |
| 83 | Fuca1 | 1814 | -0.603 | -0.1083 | No |
| 84 | Eif3f | 1833 | -0.606 | -0.1099 | No |
| 85 | Calm1 | 1839 | -0.607 | -0.1086 | No |
| 86 | Txnl4a | 1868 | -0.613 | -0.1123 | No |
| 87 | Coa3 | 1871 | -0.614 | -0.1103 | No |
| 88 | Emg1 | 1891 | -0.617 | -0.1120 | No |
| 89 | Cyb5a | 1942 | -0.625 | -0.1205 | No |
| 90 | Qdpr | 1949 | -0.627 | -0.1193 | No |
| 91 | Cracr2b | 2009 | -0.637 | -0.1297 | No |
| 92 | Vkorc1 | 2063 | -0.646 | -0.1388 | No |
| 93 | Mpv17l2 | 2093 | -0.653 | -0.1426 | No |
| 94 | Ndufs2 | 2103 | -0.655 | -0.1419 | No |
| 95 | Lrrc26 | 2107 | -0.655 | -0.1400 | No |
| 96 | Tmbim6 | 2111 | -0.655 | -0.1381 | No |
| 97 | Sin3b | 2139 | -0.661 | -0.1414 | No |
| 98 | Ubl7 | 2146 | -0.662 | -0.1401 | No |
| 99 | Txn2 | 2163 | -0.664 | -0.1409 | No |
| 100 | Alad | 2165 | -0.664 | -0.1385 | No |
| 101 | H13 | 2183 | -0.667 | -0.1396 | No |
| 102 | Cdpf1 | 2197 | -0.670 | -0.1398 | No |
| 103 | Tmem141 | 2198 | -0.670 | -0.1372 | No |
| 104 | Tex261 | 2206 | -0.672 | -0.1361 | No |
| 105 | Gnb2 | 2210 | -0.673 | -0.1341 | No |
| 106 | Bet1l | 2249 | -0.680 | -0.1397 | No |
| 107 | Ptgr1 | 2287 | -0.685 | -0.1451 | No |
| 108 | Sil1 | 2329 | -0.694 | -0.1513 | No |
| 109 | Fam98c | 2335 | -0.695 | -0.1497 | No |
| 110 | Spag7 | 2338 | -0.695 | -0.1474 | No |
| 111 | Ndufa7 | 2344 | -0.696 | -0.1457 | No |
| 112 | Polr2e | 2361 | -0.699 | -0.1464 | No |
| 113 | Dnlz | 2368 | -0.700 | -0.1450 | No |
| 114 | Aldh2 | 2388 | -0.704 | -0.1464 | No |
| 115 | 2610528J11Rik | 2396 | -0.705 | -0.1451 | No |
| 116 | Yipf3 | 2423 | -0.711 | -0.1480 | No |
| 117 | Naxd | 2426 | -0.712 | -0.1456 | No |
| 118 | Smagp | 2447 | -0.717 | -0.1472 | No |
| 119 | Hmgcl | 2468 | -0.721 | -0.1487 | No |
| 120 | Shisa5 | 2493 | -0.727 | -0.1511 | No |
| 121 | Faap20 | 2505 | -0.729 | -0.1506 | No |
| 122 | Guk1 | 2516 | -0.730 | -0.1499 | No |
| 123 | S100a13 | 2519 | -0.731 | -0.1475 | No |
| 124 | Idh2 | 2529 | -0.733 | -0.1465 | No |
| 125 | B3gat3 | 2535 | -0.734 | -0.1447 | No |
| 126 | Cib1 | 2552 | -0.737 | -0.1453 | No |
| 127 | Calm3 | 2555 | -0.737 | -0.1428 | No |
| 128 | Smim14 | 2579 | -0.744 | -0.1449 | No |
| 129 | Krtcap2 | 2586 | -0.744 | -0.1433 | No |
| 130 | Snw1 | 2591 | -0.745 | -0.1412 | No |
| 131 | Selenos | 2616 | -0.752 | -0.1435 | No |
| 132 | Fbp2 | 2651 | -0.759 | -0.1480 | No |
| 133 | Zfpl1 | 2674 | -0.763 | -0.1498 | No |
| 134 | Ppa1 | 2690 | -0.766 | -0.1500 | No |
| 135 | Tmem208 | 2697 | -0.767 | -0.1483 | No |
| 136 | Tmem109 | 2708 | -0.769 | -0.1475 | No |
| 137 | Ccs | 2710 | -0.769 | -0.1446 | No |
| 138 | 2510002D24Rik | 2723 | -0.771 | -0.1442 | No |
| 139 | Cnpy2 | 2726 | -0.772 | -0.1416 | No |
| 140 | Aarsd1 | 2738 | -0.775 | -0.1410 | No |
| 141 | Ly6e | 2739 | -0.775 | -0.1379 | No |
| 142 | Nans | 2757 | -0.781 | -0.1385 | No |
| 143 | Bsg | 2764 | -0.783 | -0.1367 | No |
| 144 | Atraid | 2778 | -0.786 | -0.1365 | No |
| 145 | Ddt | 2787 | -0.788 | -0.1351 | No |
| 146 | Bola1 | 2805 | -0.791 | -0.1357 | No |
| 147 | Ciao2a | 2807 | -0.791 | -0.1328 | No |
| 148 | Smim22 | 2845 | -0.801 | -0.1377 | No |
| 149 | Sfxn1 | 2864 | -0.806 | -0.1385 | No |
| 150 | Slc50a1 | 2865 | -0.806 | -0.1353 | No |
| 151 | Tm2d3 | 2872 | -0.808 | -0.1334 | No |
| 152 | Tex264 | 2874 | -0.809 | -0.1305 | No |
| 153 | Itm2c | 2893 | -0.813 | -0.1312 | No |
| 154 | Ifi27 | 2905 | -0.815 | -0.1304 | No |
| 155 | Mob2 | 2969 | -0.832 | -0.1409 | No |
| 156 | Gadd45gip1 | 3033 | -0.850 | -0.1513 | No |
| 157 | Fam3b | 3069 | -0.860 | -0.1556 | No |
| 158 | S100a1 | 3077 | -0.864 | -0.1537 | No |
| 159 | Gjb1 | 3084 | -0.866 | -0.1516 | No |
| 160 | Tmem147 | 3100 | -0.871 | -0.1514 | No |
| 161 | Isg20 | 3115 | -0.875 | -0.1510 | No |
| 162 | Yipf1 | 3121 | -0.877 | -0.1487 | No |
| 163 | Hsd17b10 | 3144 | -0.883 | -0.1500 | No |
| 164 | Cirbp | 3148 | -0.884 | -0.1472 | No |
| 165 | Spint2 | 3189 | -0.895 | -0.1524 | No |
| 166 | Uqcc3 | 3246 | -0.914 | -0.1610 | No |
| 167 | Commd9 | 3259 | -0.918 | -0.1600 | No |
| 168 | Ndufb8 | 3274 | -0.922 | -0.1595 | No |
| 169 | Surf1 | 3279 | -0.924 | -0.1567 | No |
| 170 | Ddrgk1 | 3282 | -0.924 | -0.1535 | No |
| 171 | Gstm5 | 3294 | -0.927 | -0.1522 | No |
| 172 | Naxe | 3301 | -0.931 | -0.1499 | No |
| 173 | Atp2c2 | 3322 | -0.938 | -0.1505 | No |
| 174 | Tmed4 | 3329 | -0.939 | -0.1481 | No |
| 175 | Hint2 | 3347 | -0.946 | -0.1481 | No |
| 176 | Tmem59 | 3397 | -0.960 | -0.1550 | No |
| 177 | Sdhd | 3399 | -0.960 | -0.1515 | No |
| 178 | 3110040N11Rik | 3409 | -0.963 | -0.1496 | No |
| 179 | Acot13 | 3414 | -0.967 | -0.1467 | No |
| 180 | Mecr | 3462 | -0.985 | -0.1531 | No |
| 181 | Zmat5 | 3484 | -0.991 | -0.1538 | No |
| 182 | Nudt14 | 3503 | -0.998 | -0.1538 | No |
| 183 | Txndc12 | 3516 | -1.003 | -0.1524 | No |
| 184 | Cd82 | 3518 | -1.004 | -0.1487 | No |
| 185 | Gadd45g | 3538 | -1.011 | -0.1488 | No |
| 186 | Cenpx | 3601 | -1.035 | -0.1583 | No |
| 187 | Lgals9 | 3638 | -1.047 | -0.1621 | No |
| 188 | Tsc22d1 | 3676 | -1.066 | -0.1660 | No |
| 189 | Krtcap3 | 3697 | -1.078 | -0.1661 | Yes |
| 190 | Pdrg1 | 3698 | -1.078 | -0.1618 | Yes |
| 191 | Ech1 | 3705 | -1.083 | -0.1589 | Yes |
| 192 | Sri | 3726 | -1.092 | -0.1589 | Yes |
| 193 | 2310039H08Rik | 3763 | -1.108 | -0.1624 | Yes |
| 194 | Cmtm8 | 3784 | -1.118 | -0.1624 | Yes |
| 195 | Smco4 | 3805 | -1.131 | -0.1623 | Yes |
| 196 | Foxp4 | 3822 | -1.140 | -0.1613 | Yes |
| 197 | Arfip2 | 3827 | -1.141 | -0.1577 | Yes |
| 198 | Mcrip2 | 3863 | -1.163 | -0.1607 | Yes |
| 199 | Bag1 | 3870 | -1.165 | -0.1574 | Yes |
| 200 | Dnajc3 | 3895 | -1.181 | -0.1580 | Yes |
| 201 | Dynll2 | 3904 | -1.185 | -0.1551 | Yes |
| 202 | Bri3 | 3905 | -1.186 | -0.1504 | Yes |
| 203 | Aga | 3908 | -1.189 | -0.1461 | Yes |
| 204 | Ppdpf | 3930 | -1.203 | -0.1460 | Yes |
| 205 | Mea1 | 3947 | -1.215 | -0.1447 | Yes |
| 206 | Qsox1 | 3954 | -1.217 | -0.1412 | Yes |
| 207 | Dcxr | 3966 | -1.224 | -0.1388 | Yes |
| 208 | Wbp1 | 3986 | -1.239 | -0.1380 | Yes |
| 209 | Cdc42ep5 | 4009 | -1.255 | -0.1379 | Yes |
| 210 | Tcf7l2 | 4036 | -1.272 | -0.1386 | Yes |
| 211 | Pts | 4078 | -1.307 | -0.1424 | Yes |
| 212 | Pllp | 4119 | -1.348 | -0.1458 | Yes |
| 213 | Npdc1 | 4121 | -1.350 | -0.1407 | Yes |
| 214 | Tstd1 | 4139 | -1.362 | -0.1390 | Yes |
| 215 | Gtf2a2 | 4156 | -1.374 | -0.1371 | Yes |
| 216 | Tmem9 | 4164 | -1.381 | -0.1332 | Yes |
| 217 | Ptov1 | 4177 | -1.389 | -0.1303 | Yes |
| 218 | Mettl26 | 4185 | -1.397 | -0.1263 | Yes |
| 219 | Hes6 | 4191 | -1.404 | -0.1219 | Yes |
| 220 | Gstm1 | 4200 | -1.410 | -0.1180 | Yes |
| 221 | Gmds | 4203 | -1.412 | -0.1129 | Yes |
| 222 | Akr7a5 | 4207 | -1.413 | -0.1080 | Yes |
| 223 | Ccdc107 | 4218 | -1.430 | -0.1045 | Yes |
| 224 | Vsig2 | 4221 | -1.434 | -0.0993 | Yes |
| 225 | Ccnd1 | 4229 | -1.443 | -0.0951 | Yes |
| 226 | Ppif | 4262 | -1.472 | -0.0963 | Yes |
| 227 | Cisd3 | 4271 | -1.480 | -0.0922 | Yes |
| 228 | Spr | 4273 | -1.481 | -0.0866 | Yes |
| 229 | Fermt1 | 4276 | -1.484 | -0.0811 | Yes |
| 230 | Tmem45b | 4279 | -1.487 | -0.0757 | Yes |
| 231 | Gipc2 | 4295 | -1.500 | -0.0731 | Yes |
| 232 | Ociad2 | 4322 | -1.535 | -0.0727 | Yes |
| 233 | Bcat2 | 4335 | -1.547 | -0.0692 | Yes |
| 234 | Mkrn2os | 4341 | -1.558 | -0.0641 | Yes |
| 235 | Cgref1 | 4354 | -1.583 | -0.0605 | Yes |
| 236 | Pafah1b3 | 4356 | -1.584 | -0.0545 | Yes |
| 237 | Syt7 | 4409 | -1.661 | -0.0593 | Yes |
| 238 | Bad | 4410 | -1.663 | -0.0527 | Yes |
| 239 | Ppp1r14d | 4420 | -1.672 | -0.0481 | Yes |
| 240 | Gstm2 | 4466 | -1.761 | -0.0510 | Yes |
| 241 | Krt19 | 4510 | -1.837 | -0.0531 | Yes |
| 242 | Cela1 | 4538 | -1.888 | -0.0516 | Yes |
| 243 | Klf5 | 4543 | -1.893 | -0.0450 | Yes |
| 244 | Pcbd1 | 4552 | -1.919 | -0.0391 | Yes |
| 245 | Tcea3 | 4554 | -1.926 | -0.0317 | Yes |
| 246 | Mpi | 4570 | -1.966 | -0.0273 | Yes |
| 247 | Prss32 | 4584 | -2.008 | -0.0222 | Yes |
| 248 | Smim6 | 4618 | -2.085 | -0.0212 | Yes |
| 249 | Ifi27l2b | 4639 | -2.143 | -0.0171 | Yes |
| 250 | Mgst2 | 4641 | -2.150 | -0.0088 | Yes |
| 251 | Degs2 | 4665 | -2.227 | -0.0050 | Yes |
| 252 | Agr2 | 4671 | -2.265 | 0.0028 | Yes |
| 253 | Ccnd2 | 4672 | -2.265 | 0.0118 | Yes |
| 254 | Creb3l1 | 4682 | -2.320 | 0.0190 | Yes |
| 255 | Ppp1r1b | 4771 | -2.786 | 0.0107 | Yes |
Table: GSEA details [plain text format]

  

Fig 2: TABULA\_MURIS\_SENIS\_LARGE\_INTESTINE\_LARGE\_INTESTINE\_GOBLET\_CELL\_AGEING: Random ES distribution      
 Gene set null distribution of ES for **TABULA\_MURIS\_SENIS\_LARGE\_INTESTINE\_LARGE\_INTESTINE\_GOBLET\_CELL\_AGEING**

  
