# Supplementary material for: TMPRSS11B promotes an acidified microenvironment and immune suppression in squamous lung cancer
Source: EMBO Rep. 2025 Nov 10;26(24):6346–79. doi: 10.1038/s44319-025-00631-1 (PMC12714794; doi:10.1038/s44319-025-00631-1)
Supplement: Supplementary file 10 — Source data Fig. 5 [file 44319_2025_631_MOESM10_ESM.zip › Figure 5/5C-D/GSEA_Broad Institute_M8_T11b-high LUSC vs LUAD/TABULA_MURIS_SENIS_LARGE_INTESTINE_SECRETORY_CELL_AGEING.html]

Details for gene set TABULA\_MURIS\_SENIS\_LARGE\_INTESTINE\_SECRETORY\_CELL\_AGEING[GSEA]

|  || Dataset | Ranked list\_DGE\_squamousT11b\_vs\_all adenosadeno\_HSE13-NT copy |
| Phenotype | NoPhenotypeAvailable |
| Upregulated in class | na\_neg |
| GeneSet | TABULA\_MURIS\_SENIS\_LARGE\_INTESTINE\_SECRETORY\_CELL\_AGEING |
| Enrichment Score (ES) | -0.16540597 |
| Normalized Enrichment Score (NES) | -1.1032928 |
| Nominal p-value | 0.23461539 |
| FDR q-value | 1.0 |
| FWER p-Value | 1.0 |
Table: GSEA Results Summary

  

Fig 1: Enrichment plot: TABULA\_MURIS\_SENIS\_LARGE\_INTESTINE\_SECRETORY\_CELL\_AGEING      
 Profile of the Running ES Score & Positions of GeneSet Members on the Rank Ordered List

  

| SYMBOL | RANK IN GENE LIST | RANK METRIC SCORE | RUNNING ES | CORE ENRICHMENT || 1 | Mxd1 | 163 | 2.892 | -0.0240 | No |
| 2 | S100a14 | 253 | 2.301 | -0.0342 | No |
| 3 | Dusp5 | 275 | 2.206 | -0.0299 | No |
| 4 | Ctsb | 288 | 2.139 | -0.0239 | No |
| 5 | Csf2ra | 299 | 2.108 | -0.0176 | No |
| 6 | Dusp1 | 339 | 1.923 | -0.0184 | No |
| 7 | Bdh1 | 364 | 1.808 | -0.0164 | No |
| 8 | St6galnac4 | 449 | 1.551 | -0.0285 | No |
| 9 | Dnajb1 | 453 | 1.542 | -0.0229 | No |
| 10 | Psap | 510 | 1.415 | -0.0295 | No |
| 11 | Snrk | 572 | 1.264 | -0.0377 | No |
| 12 | Ehd1 | 637 | 1.118 | -0.0472 | No |
| 13 | Rnf149 | 664 | 1.063 | -0.0486 | No |
| 14 | Fam110a | 676 | 1.039 | -0.0469 | No |
| 15 | Ctnnbip1 | 677 | 1.039 | -0.0427 | No |
| 16 | Trf | 682 | 1.033 | -0.0394 | No |
| 17 | Ece1 | 739 | 0.953 | -0.0478 | No |
| 18 | Dusp3 | 765 | 0.910 | -0.0496 | No |
| 19 | Csrnp1 | 769 | 0.907 | -0.0466 | No |
| 20 | B4galnt1 | 792 | 0.878 | -0.0479 | No |
| 21 | Gadd45b | 804 | 0.862 | -0.0468 | No |
| 22 | Prnp | 806 | 0.860 | -0.0436 | No |
| 23 | Gipc1 | 840 | 0.823 | -0.0475 | No |
| 24 | Fam83g | 880 | 0.787 | -0.0528 | No |
| 25 | Nucb2 | 893 | 0.764 | -0.0524 | No |
| 26 | Stap2 | 894 | 0.764 | -0.0493 | No |
| 27 | Incenp | 937 | 0.718 | -0.0556 | No |
| 28 | Ybx3 | 945 | 0.710 | -0.0543 | No |
| 29 | Pkm | 970 | 0.686 | -0.0568 | No |
| 30 | Rrm1 | 971 | 0.685 | -0.0540 | No |
| 31 | Pgk1 | 1029 | 0.624 | -0.0640 | No |
| 32 | Ehd4 | 1053 | 0.607 | -0.0665 | No |
| 33 | Mcm5 | 1087 | 0.567 | -0.0715 | No |
| 34 | Ptpn1 | 1126 | 0.534 | -0.0776 | No |
| 35 | Ppp1r2 | 1160 | 0.505 | -0.0828 | No |
| 36 | Arpc4 | 1169 | 0.501 | -0.0825 | No |
| 37 | Cgn | 1175 | -0.501 | -0.0816 | No |
| 38 | Kdm6b | 1191 | -0.504 | -0.0829 | No |
| 39 | Nap1l4 | 1199 | -0.505 | -0.0824 | No |
| 40 | Nfia | 1238 | -0.510 | -0.0886 | No |
| 41 | Cln6 | 1254 | -0.513 | -0.0898 | No |
| 42 | Map1lc3a | 1273 | -0.516 | -0.0917 | No |
| 43 | Vps37b | 1274 | -0.516 | -0.0896 | No |
| 44 | Fam3a | 1343 | -0.524 | -0.1024 | No |
| 45 | Ruvbl2 | 1351 | -0.526 | -0.1018 | No |
| 46 | Zfp414 | 1362 | -0.528 | -0.1019 | No |
| 47 | Ddx56 | 1365 | -0.528 | -0.1002 | No |
| 48 | Akt1 | 1371 | -0.529 | -0.0991 | No |
| 49 | Arl2bp | 1394 | -0.532 | -0.1018 | No |
| 50 | Hnrnpk | 1409 | -0.533 | -0.1027 | No |
| 51 | Ppp1r16a | 1423 | -0.536 | -0.1034 | No |
| 52 | Tle5 | 1425 | -0.536 | -0.1015 | No |
| 53 | Dapk3 | 1439 | -0.539 | -0.1021 | No |
| 54 | Clcc1 | 1446 | -0.541 | -0.1013 | No |
| 55 | Cbx1 | 1451 | -0.541 | -0.1000 | No |
| 56 | Rab1b | 1457 | -0.542 | -0.0989 | No |
| 57 | Fbxw5 | 1464 | -0.544 | -0.0980 | No |
| 58 | Ncstn | 1466 | -0.545 | -0.0960 | No |
| 59 | Exoc7 | 1475 | -0.548 | -0.0956 | No |
| 60 | Ecsit | 1504 | -0.552 | -0.0995 | No |
| 61 | Dtymk | 1507 | -0.552 | -0.0977 | No |
| 62 | Nectin2 | 1531 | -0.558 | -0.1005 | No |
| 63 | Lmf2 | 1537 | -0.558 | -0.0993 | No |
| 64 | Gpsm1 | 1543 | -0.559 | -0.0982 | No |
| 65 | Rnf166 | 1586 | -0.565 | -0.1051 | No |
| 66 | Rmdn3 | 1606 | -0.569 | -0.1069 | No |
| 67 | Brix1 | 1609 | -0.570 | -0.1051 | No |
| 68 | Nt5c3b | 1651 | -0.575 | -0.1117 | No |
| 69 | Pebp1 | 1685 | -0.581 | -0.1166 | No |
| 70 | Rbm26 | 1710 | -0.583 | -0.1195 | No |
| 71 | Mcat | 1732 | -0.589 | -0.1217 | No |
| 72 | Vps72 | 1737 | -0.589 | -0.1202 | No |
| 73 | Emc10 | 1744 | -0.590 | -0.1192 | No |
| 74 | Tcf7l1 | 1746 | -0.590 | -0.1170 | No |
| 75 | Srprb | 1752 | -0.592 | -0.1157 | No |
| 76 | Rgmb | 1768 | -0.594 | -0.1166 | No |
| 77 | Rusc1 | 1780 | -0.597 | -0.1166 | No |
| 78 | Traf3ip2 | 1798 | -0.599 | -0.1179 | No |
| 79 | Tmem161a | 1810 | -0.603 | -0.1179 | No |
| 80 | Tmed5 | 1831 | -0.605 | -0.1198 | No |
| 81 | Eif3f | 1833 | -0.606 | -0.1176 | No |
| 82 | Snx17 | 1836 | -0.607 | -0.1156 | No |
| 83 | Gga1 | 1882 | -0.616 | -0.1229 | No |
| 84 | Sap18 | 1885 | -0.617 | -0.1209 | No |
| 85 | AW209491 | 1889 | -0.617 | -0.1191 | No |
| 86 | Dnaja1 | 1926 | -0.622 | -0.1244 | No |
| 87 | Eml2 | 1930 | -0.623 | -0.1226 | No |
| 88 | Rad23a | 1946 | -0.626 | -0.1233 | No |
| 89 | Qdpr | 1949 | -0.627 | -0.1213 | No |
| 90 | Mpst | 1962 | -0.629 | -0.1213 | No |
| 91 | Pak1 | 1990 | -0.634 | -0.1247 | No |
| 92 | Ppp1r35 | 2003 | -0.637 | -0.1248 | No |
| 93 | Cracr2b | 2009 | -0.637 | -0.1233 | No |
| 94 | Csnk1g2 | 2018 | -0.639 | -0.1225 | No |
| 95 | Itpk1 | 2025 | -0.640 | -0.1212 | No |
| 96 | Snx15 | 2038 | -0.642 | -0.1212 | No |
| 97 | Tfg | 2048 | -0.643 | -0.1206 | No |
| 98 | Traf4 | 2080 | -0.650 | -0.1248 | No |
| 99 | Mtfr1 | 2086 | -0.651 | -0.1233 | No |
| 100 | Tmbim6 | 2111 | -0.655 | -0.1259 | No |
| 101 | Raly | 2113 | -0.656 | -0.1234 | No |
| 102 | Farsb | 2115 | -0.656 | -0.1210 | No |
| 103 | Ivd | 2126 | -0.658 | -0.1205 | No |
| 104 | Ei24 | 2132 | -0.660 | -0.1190 | No |
| 105 | Ubl7 | 2146 | -0.662 | -0.1192 | No |
| 106 | Txn2 | 2163 | -0.664 | -0.1200 | No |
| 107 | Eif2a | 2172 | -0.665 | -0.1191 | No |
| 108 | Polr3d | 2182 | -0.666 | -0.1183 | No |
| 109 | Tex261 | 2206 | -0.672 | -0.1207 | No |
| 110 | Keap1 | 2212 | -0.673 | -0.1190 | No |
| 111 | Prpf19 | 2264 | -0.683 | -0.1275 | No |
| 112 | Dus1l | 2267 | -0.683 | -0.1251 | No |
| 113 | 2410002F23Rik | 2280 | -0.685 | -0.1250 | No |
| 114 | Gnptg | 2327 | -0.694 | -0.1323 | No |
| 115 | Hnrnpd | 2328 | -0.694 | -0.1295 | No |
| 116 | Sil1 | 2329 | -0.694 | -0.1267 | No |
| 117 | Rnf44 | 2367 | -0.700 | -0.1320 | No |
| 118 | Psmd3 | 2373 | -0.701 | -0.1302 | No |
| 119 | Pcnp | 2376 | -0.701 | -0.1278 | No |
| 120 | 2610528J11Rik | 2396 | -0.705 | -0.1291 | No |
| 121 | Tmem39a | 2405 | -0.707 | -0.1281 | No |
| 122 | Klhl22 | 2421 | -0.710 | -0.1285 | No |
| 123 | Snx1 | 2431 | -0.713 | -0.1276 | No |
| 124 | Klf16 | 2435 | -0.714 | -0.1253 | No |
| 125 | Pnkp | 2454 | -0.718 | -0.1264 | No |
| 126 | Sh2b1 | 2460 | -0.719 | -0.1246 | No |
| 127 | Shisa5 | 2493 | -0.727 | -0.1287 | No |
| 128 | B3gat3 | 2535 | -0.734 | -0.1347 | No |
| 129 | Pycr2 | 2540 | -0.735 | -0.1326 | No |
| 130 | Calm3 | 2555 | -0.737 | -0.1327 | No |
| 131 | Nfkbiz | 2566 | -0.740 | -0.1319 | No |
| 132 | Coasy | 2567 | -0.740 | -0.1289 | No |
| 133 | Dedd | 2577 | -0.743 | -0.1279 | No |
| 134 | Akap8 | 2662 | -0.762 | -0.1432 | No |
| 135 | Zfpl1 | 2674 | -0.763 | -0.1425 | No |
| 136 | Usp22 | 2679 | -0.764 | -0.1403 | No |
| 137 | Elavl1 | 2687 | -0.765 | -0.1388 | No |
| 138 | Apobec3 | 2698 | -0.767 | -0.1379 | No |
| 139 | Tmem109 | 2708 | -0.769 | -0.1367 | No |
| 140 | Preb | 2722 | -0.771 | -0.1365 | No |
| 141 | Rdh13 | 2754 | -0.780 | -0.1401 | No |
| 142 | Polr3gl | 2763 | -0.783 | -0.1387 | No |
| 143 | Bsg | 2764 | -0.783 | -0.1355 | No |
| 144 | Med25 | 2784 | -0.787 | -0.1365 | No |
| 145 | Actr1b | 2790 | -0.789 | -0.1344 | No |
| 146 | Bzw2 | 2815 | -0.793 | -0.1365 | No |
| 147 | Dxo | 2830 | -0.797 | -0.1364 | No |
| 148 | Eif2b4 | 2839 | -0.800 | -0.1349 | No |
| 149 | Plpbp | 2852 | -0.803 | -0.1343 | No |
| 150 | Sfxn1 | 2864 | -0.806 | -0.1334 | No |
| 151 | Itm2c | 2893 | -0.813 | -0.1363 | No |
| 152 | Tysnd1 | 2914 | -0.817 | -0.1374 | No |
| 153 | Arid4b | 2924 | -0.818 | -0.1360 | No |
| 154 | Tbc1d17 | 2949 | -0.825 | -0.1380 | No |
| 155 | Mllt6 | 2954 | -0.827 | -0.1355 | No |
| 156 | Msi2 | 2967 | -0.831 | -0.1348 | No |
| 157 | Pak4 | 2974 | -0.835 | -0.1327 | No |
| 158 | Mrtfb | 3016 | -0.846 | -0.1383 | No |
| 159 | Ep400 | 3020 | -0.846 | -0.1355 | No |
| 160 | Gadd45gip1 | 3033 | -0.850 | -0.1347 | No |
| 161 | Efcab14 | 3075 | -0.863 | -0.1402 | No |
| 162 | Gjb1 | 3084 | -0.866 | -0.1385 | No |
| 163 | Dcps | 3130 | -0.879 | -0.1448 | No |
| 164 | Cirbp | 3148 | -0.884 | -0.1450 | No |
| 165 | Gna11 | 3165 | -0.889 | -0.1449 | No |
| 166 | Vasp | 3174 | -0.891 | -0.1430 | No |
| 167 | Paip1 | 3260 | -0.918 | -0.1579 | No |
| 168 | Ddrgk1 | 3282 | -0.924 | -0.1588 | No |
| 169 | Akr1e1 | 3295 | -0.929 | -0.1577 | No |
| 170 | Ddhd2 | 3312 | -0.935 | -0.1574 | No |
| 171 | Cant1 | 3316 | -0.936 | -0.1543 | No |
| 172 | Tmed4 | 3329 | -0.939 | -0.1531 | No |
| 173 | Fkbp4 | 3332 | -0.940 | -0.1498 | No |
| 174 | Fam241b | 3349 | -0.948 | -0.1495 | No |
| 175 | Arfgef3 | 3352 | -0.949 | -0.1461 | No |
| 176 | Btbd2 | 3353 | -0.949 | -0.1423 | No |
| 177 | Bcar1 | 3434 | -0.976 | -0.1558 | No |
| 178 | Rita1 | 3476 | -0.988 | -0.1608 | No |
| 179 | Scn1b | 3498 | -0.996 | -0.1614 | Yes |
| 180 | Slc1a5 | 3499 | -0.997 | -0.1574 | Yes |
| 181 | Txndc12 | 3516 | -1.003 | -0.1568 | Yes |
| 182 | Cd82 | 3518 | -1.004 | -0.1530 | Yes |
| 183 | Gadd45g | 3538 | -1.011 | -0.1531 | Yes |
| 184 | Pkp2 | 3589 | -1.028 | -0.1599 | Yes |
| 185 | Cdk5rap3 | 3593 | -1.030 | -0.1564 | Yes |
| 186 | Maz | 3596 | -1.032 | -0.1527 | Yes |
| 187 | Polr3e | 3608 | -1.035 | -0.1509 | Yes |
| 188 | Atg4b | 3634 | -1.045 | -0.1522 | Yes |
| 189 | Slc30a6 | 3660 | -1.059 | -0.1534 | Yes |
| 190 | Tmem263 | 3665 | -1.061 | -0.1500 | Yes |
| 191 | Abcb8 | 3666 | -1.062 | -0.1457 | Yes |
| 192 | Tsc22d1 | 3676 | -1.066 | -0.1434 | Yes |
| 193 | Macrod1 | 3732 | -1.095 | -0.1510 | Yes |
| 194 | Foxa3 | 3746 | -1.101 | -0.1494 | Yes |
| 195 | Tnk1 | 3781 | -1.116 | -0.1523 | Yes |
| 196 | Pick1 | 3793 | -1.123 | -0.1502 | Yes |
| 197 | Cfb | 3801 | -1.129 | -0.1472 | Yes |
| 198 | Smco4 | 3805 | -1.131 | -0.1433 | Yes |
| 199 | Arfip2 | 3827 | -1.141 | -0.1433 | Yes |
| 200 | Dynll2 | 3904 | -1.185 | -0.1551 | Yes |
| 201 | Bri3 | 3905 | -1.186 | -0.1504 | Yes |
| 202 | Aga | 3908 | -1.189 | -0.1460 | Yes |
| 203 | Mid1ip1 | 3910 | -1.190 | -0.1414 | Yes |
| 204 | Eri3 | 3914 | -1.192 | -0.1373 | Yes |
| 205 | Vamp2 | 3923 | -1.199 | -0.1342 | Yes |
| 206 | Gpr180 | 3941 | -1.210 | -0.1330 | Yes |
| 207 | Qsox1 | 3954 | -1.217 | -0.1308 | Yes |
| 208 | Inava | 3981 | -1.234 | -0.1315 | Yes |
| 209 | Prkab1 | 4029 | -1.267 | -0.1367 | Yes |
| 210 | Tcf7l2 | 4036 | -1.272 | -0.1328 | Yes |
| 211 | Slc9a1 | 4037 | -1.273 | -0.1277 | Yes |
| 212 | Pheta1 | 4057 | -1.288 | -0.1267 | Yes |
| 213 | Lap3 | 4064 | -1.292 | -0.1228 | Yes |
| 214 | Zfp787 | 4105 | -1.333 | -0.1262 | Yes |
| 215 | Lzts2 | 4118 | -1.345 | -0.1234 | Yes |
| 216 | Foxp1 | 4122 | -1.350 | -0.1186 | Yes |
| 217 | Tmub1 | 4149 | -1.368 | -0.1188 | Yes |
| 218 | Tmem9 | 4164 | -1.381 | -0.1163 | Yes |
| 219 | Vmac | 4167 | -1.384 | -0.1111 | Yes |
| 220 | Ptov1 | 4177 | -1.389 | -0.1075 | Yes |
| 221 | Btbd6 | 4202 | -1.411 | -0.1071 | Yes |
| 222 | Vsig2 | 4221 | -1.434 | -0.1052 | Yes |
| 223 | Ccnd1 | 4229 | -1.443 | -0.1009 | Yes |
| 224 | Slc25a10 | 4253 | -1.469 | -0.1000 | Yes |
| 225 | Gpd1 | 4257 | -1.470 | -0.0948 | Yes |
| 226 | Cnp | 4258 | -1.471 | -0.0888 | Yes |
| 227 | Ppif | 4262 | -1.472 | -0.0836 | Yes |
| 228 | Ppcs | 4272 | -1.480 | -0.0796 | Yes |
| 229 | Spr | 4273 | -1.481 | -0.0736 | Yes |
| 230 | Ttc38 | 4300 | -1.505 | -0.0732 | Yes |
| 231 | Cnnm4 | 4367 | -1.597 | -0.0812 | Yes |
| 232 | Rbm38 | 4407 | -1.656 | -0.0831 | Yes |
| 233 | Syt7 | 4409 | -1.661 | -0.0766 | Yes |
| 234 | Sox9 | 4412 | -1.664 | -0.0704 | Yes |
| 235 | Pdk2 | 4428 | -1.695 | -0.0668 | Yes |
| 236 | Nav2 | 4458 | -1.753 | -0.0661 | Yes |
| 237 | Krt19 | 4510 | -1.837 | -0.0698 | Yes |
| 238 | Klf5 | 4543 | -1.893 | -0.0692 | Yes |
| 239 | Tcea3 | 4554 | -1.926 | -0.0636 | Yes |
| 240 | Mpi | 4570 | -1.966 | -0.0590 | Yes |
| 241 | Cideb | 4591 | -2.024 | -0.0552 | Yes |
| 242 | Tcf4 | 4599 | -2.046 | -0.0485 | Yes |
| 243 | Ica1 | 4624 | -2.099 | -0.0453 | Yes |
| 244 | Lurap1l | 4679 | -2.307 | -0.0478 | Yes |
| 245 | Fut2 | 4680 | -2.318 | -0.0385 | Yes |
| 246 | Hid1 | 4703 | -2.401 | -0.0336 | Yes |
| 247 | Foxa2 | 4720 | -2.472 | -0.0272 | Yes |
| 248 | Tox | 4745 | -2.612 | -0.0219 | Yes |
| 249 | Ppp1r1b | 4771 | -2.786 | -0.0161 | Yes |
| 250 | D630039A03Rik | 4784 | -2.979 | -0.0067 | Yes |
| 251 | Baiap2l2 | 4812 | -3.574 | 0.0018 | Yes |
Table: GSEA details [plain text format]

  

Fig 2: TABULA\_MURIS\_SENIS\_LARGE\_INTESTINE\_SECRETORY\_CELL\_AGEING: Random ES distribution      
 Gene set null distribution of ES for **TABULA\_MURIS\_SENIS\_LARGE\_INTESTINE\_SECRETORY\_CELL\_AGEING**

  
