# Supplementary material for: TMPRSS11B promotes an acidified microenvironment and immune suppression in squamous lung cancer
Source: EMBO Rep. 2025 Nov 10;26(24):6346–79. doi: 10.1038/s44319-025-00631-1 (PMC12714794; doi:10.1038/s44319-025-00631-1)
Supplement: Supplementary file 10 — Source data Fig. 5 [file 44319_2025_631_MOESM10_ESM.zip › Figure 5/5C-D/GSEA_Broad Institute_M8_T11b-high LUSC vs LUAD/TABULA_MURIS_SENIS_LUNG_ALVEOLAR_MACROPHAGE_AGEING.html]

Details for gene set TABULA\_MURIS\_SENIS\_LUNG\_ALVEOLAR\_MACROPHAGE\_AGEING[GSEA]

|  || Dataset | Ranked list\_DGE\_squamousT11b\_vs\_all adenosadeno\_HSE13-NT copy |
| Phenotype | NoPhenotypeAvailable |
| Upregulated in class | na\_pos |
| GeneSet | TABULA\_MURIS\_SENIS\_LUNG\_ALVEOLAR\_MACROPHAGE\_AGEING |
| Enrichment Score (ES) | 0.6052836 |
| Normalized Enrichment Score (NES) | 3.0157342 |
| Nominal p-value | 0.0 |
| FDR q-value | 0.0 |
| FWER p-Value | 0.0 |
Table: GSEA Results Summary

  

Fig 1: Enrichment plot: TABULA\_MURIS\_SENIS\_LUNG\_ALVEOLAR\_MACROPHAGE\_AGEING      
 Profile of the Running ES Score & Positions of GeneSet Members on the Rank Ordered List

  

| SYMBOL | RANK IN GENE LIST | RANK METRIC SCORE | RUNNING ES | CORE ENRICHMENT || 1 | Spp1 | 69 | 4.139 | 0.0198 | Yes |
| 2 | Ctsl | 76 | 4.064 | 0.0523 | Yes |
| 3 | Ccl9 | 82 | 3.944 | 0.0839 | Yes |
| 4 | S100a8 | 93 | 3.788 | 0.1133 | Yes |
| 5 | S100a9 | 110 | 3.624 | 0.1400 | Yes |
| 6 | Ctsk | 111 | 3.616 | 0.1700 | Yes |
| 7 | Trem2 | 140 | 3.156 | 0.1902 | Yes |
| 8 | Lpl | 149 | 3.044 | 0.2138 | Yes |
| 9 | Cybb | 173 | 2.805 | 0.2322 | Yes |
| 10 | Tyrobp | 181 | 2.732 | 0.2534 | Yes |
| 11 | Srgn | 185 | 2.715 | 0.2753 | Yes |
| 12 | Cd274 | 213 | 2.481 | 0.2902 | Yes |
| 13 | Scd1 | 215 | 2.464 | 0.3105 | Yes |
| 14 | Slpi | 220 | 2.439 | 0.3299 | Yes |
| 15 | Il1b | 240 | 2.351 | 0.3454 | Yes |
| 16 | Wfdc17 | 241 | 2.337 | 0.3648 | Yes |
| 17 | Ctss | 247 | 2.317 | 0.3829 | Yes |
| 18 | S100a10 | 263 | 2.263 | 0.3985 | Yes |
| 19 | Fcer1g | 272 | 2.235 | 0.4154 | Yes |
| 20 | Ctsb | 288 | 2.139 | 0.4300 | Yes |
| 21 | Pirb | 330 | 1.965 | 0.4377 | Yes |
| 22 | Cd52 | 332 | 1.963 | 0.4537 | Yes |
| 23 | S100a4 | 353 | 1.867 | 0.4650 | Yes |
| 24 | Hexb | 384 | 1.742 | 0.4731 | Yes |
| 25 | Apoc1 | 386 | 1.739 | 0.4874 | Yes |
| 26 | Fcgr4 | 405 | 1.672 | 0.4974 | Yes |
| 27 | Msn | 419 | 1.628 | 0.5082 | Yes |
| 28 | Lgals3 | 447 | 1.559 | 0.5155 | Yes |
| 29 | C3 | 474 | 1.502 | 0.5224 | Yes |
| 30 | Apoe | 490 | 1.475 | 0.5315 | Yes |
| 31 | Pycard | 499 | 1.449 | 0.5419 | Yes |
| 32 | Psap | 510 | 1.415 | 0.5515 | Yes |
| 33 | Creg1 | 533 | 1.369 | 0.5582 | Yes |
| 34 | Emp3 | 537 | 1.365 | 0.5689 | Yes |
| 35 | Prdx5 | 601 | 1.198 | 0.5656 | Yes |
| 36 | Timp2 | 607 | 1.189 | 0.5744 | Yes |
| 37 | Sat1 | 614 | 1.180 | 0.5829 | Yes |
| 38 | Rtn4 | 726 | 0.971 | 0.5676 | Yes |
| 39 | Blvrb | 731 | 0.962 | 0.5747 | Yes |
| 40 | Csf1r | 747 | 0.942 | 0.5793 | Yes |
| 41 | H2-Ab1 | 778 | 0.895 | 0.5804 | Yes |
| 42 | B2m | 794 | 0.876 | 0.5846 | Yes |
| 43 | Cd74 | 811 | 0.856 | 0.5883 | Yes |
| 44 | Npc2 | 822 | 0.841 | 0.5932 | Yes |
| 45 | Uba52 | 896 | 0.762 | 0.5841 | Yes |
| 46 | Fabp4 | 914 | 0.747 | 0.5867 | Yes |
| 47 | Dynll1 | 941 | 0.717 | 0.5872 | Yes |
| 48 | Ier3 | 967 | 0.691 | 0.5876 | Yes |
| 49 | Pkm | 970 | 0.686 | 0.5929 | Yes |
| 50 | H2-Eb1 | 976 | 0.681 | 0.5975 | Yes |
| 51 | Igf1 | 978 | 0.674 | 0.6029 | Yes |
| 52 | H2-D1 | 1021 | 0.632 | 0.5993 | Yes |
| 53 | Pgk1 | 1029 | 0.624 | 0.6030 | Yes |
| 54 | Litaf | 1060 | 0.599 | 0.6016 | Yes |
| 55 | Psmb8 | 1067 | 0.589 | 0.6052 | Yes |
| 56 | Ahnak | 1090 | 0.565 | 0.6053 | Yes |
| 57 | Grcc10 | 1270 | -0.515 | 0.5718 | No |
| 58 | Mgst1 | 1519 | -0.554 | 0.5241 | No |
| 59 | Chpt1 | 1556 | -0.561 | 0.5212 | No |
| 60 | Micos13 | 1804 | -0.601 | 0.4741 | No |
| 61 | Calm1 | 1839 | -0.607 | 0.4720 | No |
| 62 | Pnrc1 | 1935 | -0.624 | 0.4571 | No |
| 63 | Aldh2 | 2388 | -0.704 | 0.3677 | No |
| 64 | Krtcap2 | 2586 | -0.744 | 0.3323 | No |
| 65 | Sod1 | 2688 | -0.765 | 0.3174 | No |
| 66 | Ly6e | 2739 | -0.775 | 0.3133 | No |
| 67 | S100a1 | 3077 | -0.864 | 0.2494 | No |
| 68 | Cd2ap | 3167 | -0.889 | 0.2380 | No |
| 69 | Uqcc3 | 3246 | -0.914 | 0.2291 | No |
| 70 | S100a6 | 3412 | -0.966 | 0.2024 | No |
| 71 | Ggh | 3487 | -0.992 | 0.1950 | No |
| 72 | Tanc2 | 3785 | -1.119 | 0.1417 | No |
| 73 | Serpinb6a | 3957 | -1.219 | 0.1157 | No |
| 74 | Foxp1 | 4122 | -1.350 | 0.0924 | No |
| 75 | Gstm1 | 4200 | -1.410 | 0.0878 | No |
| 76 | Alcam | 4291 | -1.496 | 0.0813 | No |
| 77 | Cmbl | 4433 | -1.707 | 0.0657 | No |
| 78 | Cd24a | 4541 | -1.891 | 0.0588 | No |
Table: GSEA details [plain text format]

  

Fig 2: TABULA\_MURIS\_SENIS\_LUNG\_ALVEOLAR\_MACROPHAGE\_AGEING: Random ES distribution      
 Gene set null distribution of ES for **TABULA\_MURIS\_SENIS\_LUNG\_ALVEOLAR\_MACROPHAGE\_AGEING**

  
