# Supplementary material for: TMPRSS11B promotes an acidified microenvironment and immune suppression in squamous lung cancer
Source: EMBO Rep. 2025 Nov 10;26(24):6346–79. doi: 10.1038/s44319-025-00631-1 (PMC12714794; doi:10.1038/s44319-025-00631-1)
Supplement: Supplementary file 10 — Source data Fig. 5 [file 44319_2025_631_MOESM10_ESM.zip › Figure 5/5C-D/GSEA_Broad Institute_M8_T11b-high LUSC vs LUAD/TABULA_MURIS_SENIS_LUNG_INTERMEDIATE_MONOCYTE_AGEING.html]

Details for gene set TABULA\_MURIS\_SENIS\_LUNG\_INTERMEDIATE\_MONOCYTE\_AGEING[GSEA]

|  || Dataset | Ranked list\_DGE\_squamousT11b\_vs\_all adenosadeno\_HSE13-NT copy |
| Phenotype | NoPhenotypeAvailable |
| Upregulated in class | na\_pos |
| GeneSet | TABULA\_MURIS\_SENIS\_LUNG\_INTERMEDIATE\_MONOCYTE\_AGEING |
| Enrichment Score (ES) | 0.5072102 |
| Normalized Enrichment Score (NES) | 2.5893676 |
| Nominal p-value | 0.0 |
| FDR q-value | 0.0 |
| FWER p-Value | 0.0 |
Table: GSEA Results Summary

  

Fig 1: Enrichment plot: TABULA\_MURIS\_SENIS\_LUNG\_INTERMEDIATE\_MONOCYTE\_AGEING      
 Profile of the Running ES Score & Positions of GeneSet Members on the Rank Ordered List

  

| SYMBOL | RANK IN GENE LIST | RANK METRIC SCORE | RUNNING ES | CORE ENRICHMENT || 1 | Ctsl | 76 | 4.064 | 0.0146 | Yes |
| 2 | Ccl9 | 82 | 3.944 | 0.0434 | Yes |
| 3 | Tnfaip2 | 83 | 3.933 | 0.0732 | Yes |
| 4 | S100a8 | 93 | 3.788 | 0.0999 | Yes |
| 5 | S100a9 | 110 | 3.624 | 0.1239 | Yes |
| 6 | Clec4d | 131 | 3.254 | 0.1443 | Yes |
| 7 | Trem2 | 140 | 3.156 | 0.1665 | Yes |
| 8 | Cybb | 173 | 2.805 | 0.1809 | Yes |
| 9 | Csf2rb | 176 | 2.766 | 0.2014 | Yes |
| 10 | Fermt3 | 193 | 2.642 | 0.2180 | Yes |
| 11 | Slpi | 220 | 2.439 | 0.2310 | Yes |
| 12 | Il1b | 240 | 2.351 | 0.2447 | Yes |
| 13 | Wfdc17 | 241 | 2.337 | 0.2624 | Yes |
| 14 | Plek | 252 | 2.303 | 0.2777 | Yes |
| 15 | S100a10 | 263 | 2.263 | 0.2927 | Yes |
| 16 | Ctsd | 274 | 2.219 | 0.3074 | Yes |
| 17 | Ly6a | 278 | 2.197 | 0.3234 | Yes |
| 18 | Fth1 | 289 | 2.129 | 0.3374 | Yes |
| 19 | Mif | 323 | 1.991 | 0.3454 | Yes |
| 20 | Pirb | 330 | 1.965 | 0.3590 | Yes |
| 21 | Cd52 | 332 | 1.963 | 0.3737 | Yes |
| 22 | Bcl2a1b | 349 | 1.876 | 0.3845 | Yes |
| 23 | Hp | 388 | 1.736 | 0.3895 | Yes |
| 24 | Lgals1 | 400 | 1.684 | 0.3999 | Yes |
| 25 | Fcgr4 | 405 | 1.672 | 0.4117 | Yes |
| 26 | Ifitm1 | 427 | 1.619 | 0.4195 | Yes |
| 27 | Lgals3 | 447 | 1.559 | 0.4273 | Yes |
| 28 | Fam111a | 455 | 1.540 | 0.4375 | Yes |
| 29 | Syk | 459 | 1.531 | 0.4484 | Yes |
| 30 | C3 | 474 | 1.502 | 0.4568 | Yes |
| 31 | Emb | 484 | 1.488 | 0.4662 | Yes |
| 32 | Apoe | 490 | 1.475 | 0.4763 | Yes |
| 33 | Pycard | 499 | 1.449 | 0.4855 | Yes |
| 34 | Creg1 | 533 | 1.369 | 0.4889 | Yes |
| 35 | Acp5 | 536 | 1.366 | 0.4988 | Yes |
| 36 | Esd | 579 | 1.253 | 0.4994 | Yes |
| 37 | Cstb | 587 | 1.229 | 0.5072 | Yes |
| 38 | Hck | 700 | 1.005 | 0.4911 | No |
| 39 | Blvrb | 731 | 0.962 | 0.4920 | No |
| 40 | Txn1 | 743 | 0.944 | 0.4968 | No |
| 41 | Prelid1 | 751 | 0.932 | 0.5024 | No |
| 42 | Siva1 | 892 | 0.765 | 0.4785 | No |
| 43 | Uba52 | 896 | 0.762 | 0.4836 | No |
| 44 | Ier3 | 967 | 0.691 | 0.4740 | No |
| 45 | Pkm | 970 | 0.686 | 0.4788 | No |
| 46 | Atox1 | 997 | 0.651 | 0.4782 | No |
| 47 | Pgk1 | 1029 | 0.624 | 0.4763 | No |
| 48 | Rassf4 | 1055 | 0.604 | 0.4756 | No |
| 49 | Grina | 1115 | 0.543 | 0.4672 | No |
| 50 | Sod2 | 1120 | 0.540 | 0.4705 | No |
| 51 | Ptpn1 | 1126 | 0.534 | 0.4734 | No |
| 52 | Rilpl2 | 1127 | 0.533 | 0.4775 | No |
| 53 | Ndufb6 | 1150 | 0.512 | 0.4767 | No |
| 54 | Smc1a | 1189 | -0.503 | 0.4724 | No |
| 55 | Eef1d | 1235 | -0.510 | 0.4668 | No |
| 56 | Tm2d2 | 1250 | -0.513 | 0.4677 | No |
| 57 | Nol7 | 1257 | -0.514 | 0.4703 | No |
| 58 | Grcc10 | 1270 | -0.515 | 0.4717 | No |
| 59 | Imp3 | 1311 | -0.520 | 0.4671 | No |
| 60 | Itgb1 | 1391 | -0.531 | 0.4544 | No |
| 61 | P4hb | 1469 | -0.546 | 0.4422 | No |
| 62 | Rpp21 | 1595 | -0.567 | 0.4200 | No |
| 63 | Xpa | 1601 | -0.568 | 0.4232 | No |
| 64 | Usp34 | 1678 | -0.579 | 0.4115 | No |
| 65 | Rtf1 | 1697 | -0.583 | 0.4121 | No |
| 66 | Aprt | 1790 | -0.598 | 0.3971 | No |
| 67 | Micos13 | 1804 | -0.601 | 0.3989 | No |
| 68 | Emg1 | 1891 | -0.617 | 0.3854 | No |
| 69 | Dnaja1 | 1926 | -0.622 | 0.3829 | No |
| 70 | Cyb5a | 1942 | -0.625 | 0.3844 | No |
| 71 | Rp9 | 1991 | -0.634 | 0.3791 | No |
| 72 | Agpat4 | 2099 | -0.654 | 0.3613 | No |
| 73 | Srrm2 | 2159 | -0.663 | 0.3538 | No |
| 74 | Atxn7l3b | 2363 | -0.700 | 0.3161 | No |
| 75 | S100a13 | 2519 | -0.731 | 0.2888 | No |
| 76 | Krtcap2 | 2586 | -0.744 | 0.2804 | No |
| 77 | Sod1 | 2688 | -0.765 | 0.2648 | No |
| 78 | Tmem208 | 2697 | -0.767 | 0.2689 | No |
| 79 | Cnpy2 | 2726 | -0.772 | 0.2688 | No |
| 80 | Spata13 | 2747 | -0.778 | 0.2705 | No |
| 81 | Ddt | 2787 | -0.788 | 0.2682 | No |
| 82 | Ciao2a | 2807 | -0.791 | 0.2701 | No |
| 83 | Filip1l | 2887 | -0.812 | 0.2595 | No |
| 84 | Ifi27 | 2905 | -0.815 | 0.2621 | No |
| 85 | Kif5b | 2964 | -0.830 | 0.2561 | No |
| 86 | Rere | 2991 | -0.838 | 0.2569 | No |
| 87 | Tgm2 | 2996 | -0.839 | 0.2624 | No |
| 88 | Tnrc6b | 3055 | -0.857 | 0.2566 | No |
| 89 | Son | 3098 | -0.871 | 0.2543 | No |
| 90 | Prorsd1 | 3226 | -0.907 | 0.2342 | No |
| 91 | Uqcc3 | 3246 | -0.914 | 0.2371 | No |
| 92 | Ndufb8 | 3274 | -0.922 | 0.2384 | No |
| 93 | Rbm39 | 3394 | -0.958 | 0.2204 | No |
| 94 | S100a6 | 3412 | -0.966 | 0.2241 | No |
| 95 | Dnm2 | 3545 | -1.013 | 0.2038 | No |
| 96 | Cenpx | 3601 | -1.035 | 0.2000 | No |
| 97 | Id3 | 3653 | -1.055 | 0.1972 | No |
| 98 | Tnfaip8 | 3897 | -1.181 | 0.1546 | No |
| 99 | Rel | 3944 | -1.213 | 0.1540 | No |
| 100 | Smim11 | 3961 | -1.222 | 0.1599 | No |
| 101 | Gmfg | 4007 | -1.254 | 0.1598 | No |
| 102 | Lmo4 | 4382 | -1.617 | 0.0928 | No |
Table: GSEA details [plain text format]

  

Fig 2: TABULA\_MURIS\_SENIS\_LUNG\_INTERMEDIATE\_MONOCYTE\_AGEING: Random ES distribution      
 Gene set null distribution of ES for **TABULA\_MURIS\_SENIS\_LUNG\_INTERMEDIATE\_MONOCYTE\_AGEING**

  
