# Supplementary material for: TMPRSS11B promotes an acidified microenvironment and immune suppression in squamous lung cancer
Source: EMBO Rep. 2025 Nov 10;26(24):6346–79. doi: 10.1038/s44319-025-00631-1 (PMC12714794; doi:10.1038/s44319-025-00631-1)
Supplement: Supplementary file 10 — Source data Fig. 5 [file 44319_2025_631_MOESM10_ESM.zip › Figure 5/5C-D/GSEA_Broad Institute_M8_T11b-high LUSC vs LUAD/TABULA_MURIS_SENIS_LUNG_NON_CLASSICAL_MONOCYTE_AGEING.html]

Details for gene set TABULA\_MURIS\_SENIS\_LUNG\_NON\_CLASSICAL\_MONOCYTE\_AGEING[GSEA]

|  || Dataset | Ranked list\_DGE\_squamousT11b\_vs\_all adenosadeno\_HSE13-NT copy |
| Phenotype | NoPhenotypeAvailable |
| Upregulated in class | na\_pos |
| GeneSet | TABULA\_MURIS\_SENIS\_LUNG\_NON\_CLASSICAL\_MONOCYTE\_AGEING |
| Enrichment Score (ES) | 0.72283965 |
| Normalized Enrichment Score (NES) | 2.471832 |
| Nominal p-value | 0.0 |
| FDR q-value | 0.0 |
| FWER p-Value | 0.0 |
Table: GSEA Results Summary

  

Fig 1: Enrichment plot: TABULA\_MURIS\_SENIS\_LUNG\_NON\_CLASSICAL\_MONOCYTE\_AGEING      
 Profile of the Running ES Score & Positions of GeneSet Members on the Rank Ordered List

  

| SYMBOL | RANK IN GENE LIST | RANK METRIC SCORE | RUNNING ES | CORE ENRICHMENT || 1 | Cybb | 173 | 2.805 | 0.0588 | Yes |
| 2 | Pla2g7 | 195 | 2.599 | 0.1423 | Yes |
| 3 | Slpi | 220 | 2.439 | 0.2197 | Yes |
| 4 | Il1b | 240 | 2.351 | 0.2952 | Yes |
| 5 | Fcer1g | 272 | 2.235 | 0.3644 | Yes |
| 6 | Ly6a | 278 | 2.197 | 0.4376 | Yes |
| 7 | Fth1 | 289 | 2.129 | 0.5075 | Yes |
| 8 | Cd52 | 332 | 1.963 | 0.5651 | Yes |
| 9 | Hp | 388 | 1.736 | 0.6123 | Yes |
| 10 | Fcgr4 | 405 | 1.672 | 0.6655 | Yes |
| 11 | Apoe | 490 | 1.475 | 0.6979 | Yes |
| 12 | Txn1 | 743 | 0.944 | 0.6774 | Yes |
| 13 | H2-Ab1 | 778 | 0.895 | 0.7006 | Yes |
| 14 | Cd74 | 811 | 0.856 | 0.7228 | Yes |
| 15 | H2-Eb1 | 976 | 0.681 | 0.7117 | No |
| 16 | Cfh | 1075 | 0.578 | 0.7108 | No |
| 17 | Filip1l | 2887 | -0.812 | 0.3612 | No |
| 18 | Rel | 3944 | -1.213 | 0.1824 | No |
Table: GSEA details [plain text format]

  

Fig 2: TABULA\_MURIS\_SENIS\_LUNG\_NON\_CLASSICAL\_MONOCYTE\_AGEING: Random ES distribution      
 Gene set null distribution of ES for **TABULA\_MURIS\_SENIS\_LUNG\_NON\_CLASSICAL\_MONOCYTE\_AGEING**

  
