# Supplementary material for: TMPRSS11B promotes an acidified microenvironment and immune suppression in squamous lung cancer
Source: EMBO Rep. 2025 Nov 10;26(24):6346–79. doi: 10.1038/s44319-025-00631-1 (PMC12714794; doi:10.1038/s44319-025-00631-1)
Supplement: Supplementary file 10 — Source data Fig. 5 [file 44319_2025_631_MOESM10_ESM.zip › Figure 5/5C-D/GSEA_Broad Institute_M8_T11b-high LUSC vs LUAD/TABULA_MURIS_SENIS_MARROW_GRANULOCYTE_AGEING.html]

Details for gene set TABULA\_MURIS\_SENIS\_MARROW\_GRANULOCYTE\_AGEING[GSEA]

|  || Dataset | Ranked list\_DGE\_squamousT11b\_vs\_all adenosadeno\_HSE13-NT copy |
| Phenotype | NoPhenotypeAvailable |
| Upregulated in class | na\_pos |
| GeneSet | TABULA\_MURIS\_SENIS\_MARROW\_GRANULOCYTE\_AGEING |
| Enrichment Score (ES) | 0.7130401 |
| Normalized Enrichment Score (NES) | 2.6599073 |
| Nominal p-value | 0.0 |
| FDR q-value | 0.0 |
| FWER p-Value | 0.0 |
Table: GSEA Results Summary

  

Fig 1: Enrichment plot: TABULA\_MURIS\_SENIS\_MARROW\_GRANULOCYTE\_AGEING      
 Profile of the Running ES Score & Positions of GeneSet Members on the Rank Ordered List

  

| SYMBOL | RANK IN GENE LIST | RANK METRIC SCORE | RUNNING ES | CORE ENRICHMENT || 1 | S100a8 | 93 | 3.788 | 0.0633 | Yes |
| 2 | S100a9 | 110 | 3.624 | 0.1391 | Yes |
| 3 | Tyrobp | 181 | 2.732 | 0.1842 | Yes |
| 4 | Ccl6 | 187 | 2.695 | 0.2420 | Yes |
| 5 | Slpi | 220 | 2.439 | 0.2886 | Yes |
| 6 | Spi1 | 250 | 2.309 | 0.3330 | Yes |
| 7 | Orm1 | 262 | 2.265 | 0.3802 | Yes |
| 8 | Fcer1g | 272 | 2.235 | 0.4271 | Yes |
| 9 | Lcn2 | 283 | 2.163 | 0.4723 | Yes |
| 10 | Cd52 | 332 | 1.963 | 0.5051 | Yes |
| 11 | Pglyrp1 | 345 | 1.894 | 0.5440 | Yes |
| 12 | Hp | 388 | 1.736 | 0.5731 | Yes |
| 13 | Lgals3 | 447 | 1.559 | 0.5951 | Yes |
| 14 | Fxyd5 | 489 | 1.476 | 0.6188 | Yes |
| 15 | Alox5ap | 500 | 1.445 | 0.6482 | Yes |
| 16 | Emp3 | 537 | 1.365 | 0.6705 | Yes |
| 17 | Coro1a | 546 | 1.340 | 0.6981 | Yes |
| 18 | Prdx5 | 601 | 1.198 | 0.7130 | Yes |
| 19 | B2m | 794 | 0.876 | 0.6921 | No |
| 20 | Pkm | 970 | 0.686 | 0.6706 | No |
| 21 | H2-D1 | 1021 | 0.632 | 0.6739 | No |
| 22 | Lsp1 | 1023 | 0.629 | 0.6875 | No |
| 23 | Tmsb4x | 1258 | -0.514 | 0.6499 | No |
| 24 | Cebpd | 1668 | -0.578 | 0.5772 | No |
| 25 | Calm1 | 1839 | -0.607 | 0.5550 | No |
| 26 | Lrg1 | 2920 | -0.817 | 0.3475 | No |
| 27 | S100a6 | 3412 | -0.966 | 0.2662 | No |
| 28 | Gmfg | 4007 | -1.254 | 0.1696 | No |
Table: GSEA details [plain text format]

  

Fig 2: TABULA\_MURIS\_SENIS\_MARROW\_GRANULOCYTE\_AGEING: Random ES distribution      
 Gene set null distribution of ES for **TABULA\_MURIS\_SENIS\_MARROW\_GRANULOCYTE\_AGEING**

  
