# Supplementary material for: TMPRSS11B promotes an acidified microenvironment and immune suppression in squamous lung cancer
Source: EMBO Rep. 2025 Nov 10;26(24):6346–79. doi: 10.1038/s44319-025-00631-1 (PMC12714794; doi:10.1038/s44319-025-00631-1)
Supplement: Supplementary file 10 — Source data Fig. 5 [file 44319_2025_631_MOESM10_ESM.zip › Figure 5/5C-D/GSEA_Broad Institute_M8_T11b-high LUSC vs LUAD/TABULA_MURIS_SENIS_MARROW_GRANULOCYTOPOIETIC_CELL_AGEING.html]

Details for gene set TABULA\_MURIS\_SENIS\_MARROW\_GRANULOCYTOPOIETIC\_CELL\_AGEING[GSEA]

|  || Dataset | Ranked list\_DGE\_squamousT11b\_vs\_all adenosadeno\_HSE13-NT copy |
| Phenotype | NoPhenotypeAvailable |
| Upregulated in class | na\_pos |
| GeneSet | TABULA\_MURIS\_SENIS\_MARROW\_GRANULOCYTOPOIETIC\_CELL\_AGEING |
| Enrichment Score (ES) | 0.7304758 |
| Normalized Enrichment Score (NES) | 3.087987 |
| Nominal p-value | 0.0 |
| FDR q-value | 0.0 |
| FWER p-Value | 0.0 |
Table: GSEA Results Summary

  

Fig 1: Enrichment plot: TABULA\_MURIS\_SENIS\_MARROW\_GRANULOCYTOPOIETIC\_CELL\_AGEING      
 Profile of the Running ES Score & Positions of GeneSet Members on the Rank Ordered List

  

| SYMBOL | RANK IN GENE LIST | RANK METRIC SCORE | RUNNING ES | CORE ENRICHMENT || 1 | S100a8 | 93 | 3.788 | 0.0381 | Yes |
| 2 | S100a9 | 110 | 3.624 | 0.0898 | Yes |
| 3 | Cybb | 173 | 2.805 | 0.1194 | Yes |
| 4 | Tyrobp | 181 | 2.732 | 0.1594 | Yes |
| 5 | Anxa1 | 218 | 2.455 | 0.1891 | Yes |
| 6 | Slpi | 220 | 2.439 | 0.2260 | Yes |
| 7 | Spi1 | 250 | 2.309 | 0.2550 | Yes |
| 8 | Lyz1 | 254 | 2.301 | 0.2893 | Yes |
| 9 | Orm1 | 262 | 2.265 | 0.3222 | Yes |
| 10 | Ltf | 264 | 2.250 | 0.3562 | Yes |
| 11 | Fcer1g | 272 | 2.235 | 0.3887 | Yes |
| 12 | Lcn2 | 283 | 2.163 | 0.4194 | Yes |
| 13 | Fth1 | 289 | 2.129 | 0.4507 | Yes |
| 14 | Cd52 | 332 | 1.963 | 0.4717 | Yes |
| 15 | Pglyrp1 | 345 | 1.894 | 0.4980 | Yes |
| 16 | Gpsm3 | 360 | 1.854 | 0.5232 | Yes |
| 17 | Hp | 388 | 1.736 | 0.5439 | Yes |
| 18 | Lgals3 | 447 | 1.559 | 0.5555 | Yes |
| 19 | AA467197 | 452 | 1.544 | 0.5781 | Yes |
| 20 | Fxyd5 | 489 | 1.476 | 0.5929 | Yes |
| 21 | Apoe | 490 | 1.475 | 0.6153 | Yes |
| 22 | Alox5ap | 500 | 1.445 | 0.6354 | Yes |
| 23 | Emp3 | 537 | 1.365 | 0.6486 | Yes |
| 24 | Prdx5 | 601 | 1.198 | 0.6536 | Yes |
| 25 | Arhgdib | 672 | 1.048 | 0.6549 | Yes |
| 26 | Spc25 | 673 | 1.047 | 0.6708 | Yes |
| 27 | Cks2 | 729 | 0.967 | 0.6740 | Yes |
| 28 | Blvrb | 731 | 0.962 | 0.6884 | Yes |
| 29 | Cdca3 | 749 | 0.932 | 0.6990 | Yes |
| 30 | Cdk1 | 820 | 0.842 | 0.6971 | Yes |
| 31 | 1810037I17Rik | 857 | 0.810 | 0.7019 | Yes |
| 32 | Nusap1 | 907 | 0.752 | 0.7030 | Yes |
| 33 | Cdca8 | 929 | 0.722 | 0.7096 | Yes |
| 34 | Rgcc | 943 | 0.713 | 0.7177 | Yes |
| 35 | H2-Eb1 | 976 | 0.681 | 0.7214 | Yes |
| 36 | Msrb1 | 982 | 0.669 | 0.7305 | Yes |
| 37 | Grina | 1115 | 0.543 | 0.7111 | No |
| 38 | Car2 | 1171 | -0.500 | 0.7072 | No |
| 39 | Pgp | 1217 | -0.508 | 0.7055 | No |
| 40 | Cebpd | 1668 | -0.578 | 0.6201 | No |
| 41 | Bsg | 2764 | -0.783 | 0.4028 | No |
| 42 | Lrg1 | 2920 | -0.817 | 0.3828 | No |
| 43 | S100a6 | 3412 | -0.966 | 0.2947 | No |
Table: GSEA details [plain text format]

  

Fig 2: TABULA\_MURIS\_SENIS\_MARROW\_GRANULOCYTOPOIETIC\_CELL\_AGEING: Random ES distribution      
 Gene set null distribution of ES for **TABULA\_MURIS\_SENIS\_MARROW\_GRANULOCYTOPOIETIC\_CELL\_AGEING**

  
