# Supplementary material for: TMPRSS11B promotes an acidified microenvironment and immune suppression in squamous lung cancer
Source: EMBO Rep. 2025 Nov 10;26(24):6346–79. doi: 10.1038/s44319-025-00631-1 (PMC12714794; doi:10.1038/s44319-025-00631-1)
Supplement: Supplementary file 10 — Source data Fig. 5 [file 44319_2025_631_MOESM10_ESM.zip › Figure 5/5C-D/GSEA_Broad Institute_M8_T11b-high LUSC vs LUAD/TABULA_MURIS_SENIS_MARROW_HEMATOPOIETIC_PRECURSOR_CELL_AGEING.html]

Details for gene set TABULA\_MURIS\_SENIS\_MARROW\_HEMATOPOIETIC\_PRECURSOR\_CELL\_AGEING[GSEA]

|  || Dataset | Ranked list\_DGE\_squamousT11b\_vs\_all adenosadeno\_HSE13-NT copy |
| Phenotype | NoPhenotypeAvailable |
| Upregulated in class | na\_pos |
| GeneSet | TABULA\_MURIS\_SENIS\_MARROW\_HEMATOPOIETIC\_PRECURSOR\_CELL\_AGEING |
| Enrichment Score (ES) | 0.64473647 |
| Normalized Enrichment Score (NES) | 2.7626686 |
| Nominal p-value | 0.0 |
| FDR q-value | 0.0 |
| FWER p-Value | 0.0 |
Table: GSEA Results Summary

  

Fig 1: Enrichment plot: TABULA\_MURIS\_SENIS\_MARROW\_HEMATOPOIETIC\_PRECURSOR\_CELL\_AGEING      
 Profile of the Running ES Score & Positions of GeneSet Members on the Rank Ordered List

  

| SYMBOL | RANK IN GENE LIST | RANK METRIC SCORE | RUNNING ES | CORE ENRICHMENT || 1 | S100a8 | 93 | 3.788 | 0.0372 | Yes |
| 2 | S100a9 | 110 | 3.624 | 0.0882 | Yes |
| 3 | Itgb2 | 158 | 2.937 | 0.1223 | Yes |
| 4 | Tyrobp | 181 | 2.732 | 0.1586 | Yes |
| 5 | Ccl6 | 187 | 2.695 | 0.1979 | Yes |
| 6 | Anxa1 | 218 | 2.455 | 0.2284 | Yes |
| 7 | Slpi | 220 | 2.439 | 0.2647 | Yes |
| 8 | Lyz1 | 254 | 2.301 | 0.2922 | Yes |
| 9 | Ltf | 264 | 2.250 | 0.3240 | Yes |
| 10 | Ly6a | 278 | 2.197 | 0.3542 | Yes |
| 11 | Lcn2 | 283 | 2.163 | 0.3857 | Yes |
| 12 | Ctsb | 288 | 2.139 | 0.4169 | Yes |
| 13 | Cd52 | 332 | 1.963 | 0.4373 | Yes |
| 14 | Pglyrp1 | 345 | 1.894 | 0.4631 | Yes |
| 15 | Bcl2a1b | 349 | 1.876 | 0.4906 | Yes |
| 16 | S100a4 | 353 | 1.867 | 0.5179 | Yes |
| 17 | Gpsm3 | 360 | 1.854 | 0.5444 | Yes |
| 18 | Hp | 388 | 1.736 | 0.5648 | Yes |
| 19 | Lgals3 | 447 | 1.559 | 0.5760 | Yes |
| 20 | Alox5ap | 500 | 1.445 | 0.5867 | Yes |
| 21 | Slfn2 | 544 | 1.342 | 0.5978 | Yes |
| 22 | Vsir | 576 | 1.261 | 0.6102 | Yes |
| 23 | Prdx5 | 601 | 1.198 | 0.6231 | Yes |
| 24 | Sat1 | 614 | 1.180 | 0.6383 | Yes |
| 25 | H2-Ab1 | 778 | 0.895 | 0.6175 | Yes |
| 26 | B2m | 794 | 0.876 | 0.6275 | Yes |
| 27 | Cd74 | 811 | 0.856 | 0.6370 | Yes |
| 28 | Ostf1 | 871 | 0.799 | 0.6366 | Yes |
| 29 | H2-Eb1 | 976 | 0.681 | 0.6250 | Yes |
| 30 | Msrb1 | 982 | 0.669 | 0.6340 | Yes |
| 31 | H2-D1 | 1021 | 0.632 | 0.6355 | Yes |
| 32 | Lsp1 | 1023 | 0.629 | 0.6447 | Yes |
| 33 | Grina | 1115 | 0.543 | 0.6338 | No |
| 34 | Tmsb4x | 1258 | -0.514 | 0.6118 | No |
| 35 | Tmbim6 | 2111 | -0.655 | 0.4433 | No |
| 36 | Shisa5 | 2493 | -0.727 | 0.3745 | No |
| 37 | Itm2c | 2893 | -0.813 | 0.3032 | No |
| 38 | Vasp | 3174 | -0.891 | 0.2579 | No |
| 39 | S100a6 | 3412 | -0.966 | 0.2228 | No |
| 40 | Cd82 | 3518 | -1.004 | 0.2159 | No |
| 41 | 4930523C07Rik | 4528 | -1.860 | 0.0326 | No |
| 42 | Cd24a | 4541 | -1.891 | 0.0584 | No |
Table: GSEA details [plain text format]

  

Fig 2: TABULA\_MURIS\_SENIS\_MARROW\_HEMATOPOIETIC\_PRECURSOR\_CELL\_AGEING: Random ES distribution      
 Gene set null distribution of ES for **TABULA\_MURIS\_SENIS\_MARROW\_HEMATOPOIETIC\_PRECURSOR\_CELL\_AGEING**

  
