# Supplementary material for: TMPRSS11B promotes an acidified microenvironment and immune suppression in squamous lung cancer
Source: EMBO Rep. 2025 Nov 10;26(24):6346–79. doi: 10.1038/s44319-025-00631-1 (PMC12714794; doi:10.1038/s44319-025-00631-1)
Supplement: Supplementary file 10 — Source data Fig. 5 [file 44319_2025_631_MOESM10_ESM.zip › Figure 5/5C-D/GSEA_Broad Institute_M8_T11b-high LUSC vs LUAD/TABULA_MURIS_SENIS_MARROW_NAIVE_T_CELL_AGEING.html]

Details for gene set TABULA\_MURIS\_SENIS\_MARROW\_NAIVE\_T\_CELL\_AGEING[GSEA]

|  || Dataset | Ranked list\_DGE\_squamousT11b\_vs\_all adenosadeno\_HSE13-NT copy |
| Phenotype | NoPhenotypeAvailable |
| Upregulated in class | na\_pos |
| GeneSet | TABULA\_MURIS\_SENIS\_MARROW\_NAIVE\_T\_CELL\_AGEING |
| Enrichment Score (ES) | 0.7743935 |
| Normalized Enrichment Score (NES) | 2.6618109 |
| Nominal p-value | 0.0 |
| FDR q-value | 0.0 |
| FWER p-Value | 0.0 |
Table: GSEA Results Summary

  

Fig 1: Enrichment plot: TABULA\_MURIS\_SENIS\_MARROW\_NAIVE\_T\_CELL\_AGEING      
 Profile of the Running ES Score & Positions of GeneSet Members on the Rank Ordered List

  

| SYMBOL | RANK IN GENE LIST | RANK METRIC SCORE | RUNNING ES | CORE ENRICHMENT || 1 | S100a8 | 93 | 3.788 | 0.0827 | Yes |
| 2 | S100a9 | 110 | 3.624 | 0.1771 | Yes |
| 3 | Dmkn | 112 | 3.602 | 0.2739 | Yes |
| 4 | Tyrobp | 181 | 2.732 | 0.3334 | Yes |
| 5 | Ccl6 | 187 | 2.695 | 0.4050 | Yes |
| 6 | Slpi | 220 | 2.439 | 0.4640 | Yes |
| 7 | Ltf | 264 | 2.250 | 0.5157 | Yes |
| 8 | Fcer1g | 272 | 2.235 | 0.5745 | Yes |
| 9 | Lcn2 | 283 | 2.163 | 0.6307 | Yes |
| 10 | Cd52 | 332 | 1.963 | 0.6736 | Yes |
| 11 | Pglyrp1 | 345 | 1.894 | 0.7222 | Yes |
| 12 | Lgals3 | 447 | 1.559 | 0.7432 | Yes |
| 13 | Fxyd5 | 489 | 1.476 | 0.7744 | Yes |
| 14 | Cd74 | 811 | 0.856 | 0.7306 | No |
| 15 | H2-D1 | 1021 | 0.632 | 0.7041 | No |
| 16 | Atp6v1g1 | 1116 | 0.543 | 0.6992 | No |
| 17 | Lrg1 | 2920 | -0.817 | 0.3457 | No |
| 18 | Son | 3098 | -0.871 | 0.3323 | No |
| 19 | S100a6 | 3412 | -0.966 | 0.2932 | No |
Table: GSEA details [plain text format]

  

Fig 2: TABULA\_MURIS\_SENIS\_MARROW\_NAIVE\_T\_CELL\_AGEING: Random ES distribution      
 Gene set null distribution of ES for **TABULA\_MURIS\_SENIS\_MARROW\_NAIVE\_T\_CELL\_AGEING**

  
