# Supplementary material for: TMPRSS11B promotes an acidified microenvironment and immune suppression in squamous lung cancer
Source: EMBO Rep. 2025 Nov 10;26(24):6346–79. doi: 10.1038/s44319-025-00631-1 (PMC12714794; doi:10.1038/s44319-025-00631-1)
Supplement: Supplementary file 10 — Source data Fig. 5 [file 44319_2025_631_MOESM10_ESM.zip › Figure 5/5C-D/GSEA_Broad Institute_M8_T11b-high LUSC vs LUAD/TABULA_MURIS_SENIS_MARROW_PROMONOCYTE_AGEING.html]

Details for gene set TABULA\_MURIS\_SENIS\_MARROW\_PROMONOCYTE\_AGEING[GSEA]

|  || Dataset | Ranked list\_DGE\_squamousT11b\_vs\_all adenosadeno\_HSE13-NT copy |
| Phenotype | NoPhenotypeAvailable |
| Upregulated in class | na\_pos |
| GeneSet | TABULA\_MURIS\_SENIS\_MARROW\_PROMONOCYTE\_AGEING |
| Enrichment Score (ES) | 0.7385114 |
| Normalized Enrichment Score (NES) | 2.8322394 |
| Nominal p-value | 0.0 |
| FDR q-value | 0.0 |
| FWER p-Value | 0.0 |
Table: GSEA Results Summary

  

Fig 1: Enrichment plot: TABULA\_MURIS\_SENIS\_MARROW\_PROMONOCYTE\_AGEING      
 Profile of the Running ES Score & Positions of GeneSet Members on the Rank Ordered List

  

| SYMBOL | RANK IN GENE LIST | RANK METRIC SCORE | RUNNING ES | CORE ENRICHMENT || 1 | Ctsl | 76 | 4.064 | 0.0600 | Yes |
| 2 | S100a8 | 93 | 3.788 | 0.1274 | Yes |
| 3 | S100a9 | 110 | 3.624 | 0.1918 | Yes |
| 4 | Dmkn | 112 | 3.602 | 0.2589 | Yes |
| 5 | Trem2 | 140 | 3.156 | 0.3122 | Yes |
| 6 | Srgn | 185 | 2.715 | 0.3537 | Yes |
| 7 | Wfdc17 | 241 | 2.337 | 0.3859 | Yes |
| 8 | Spi1 | 250 | 2.309 | 0.4273 | Yes |
| 9 | Ltf | 264 | 2.250 | 0.4666 | Yes |
| 10 | Gngt2 | 273 | 2.235 | 0.5067 | Yes |
| 11 | Lcn2 | 283 | 2.163 | 0.5452 | Yes |
| 12 | Fth1 | 289 | 2.129 | 0.5839 | Yes |
| 13 | Pim1 | 319 | 2.011 | 0.6154 | Yes |
| 14 | Mif | 323 | 1.991 | 0.6520 | Yes |
| 15 | Pglyrp1 | 345 | 1.894 | 0.6830 | Yes |
| 16 | Hp | 388 | 1.736 | 0.7066 | Yes |
| 17 | Apoe | 490 | 1.475 | 0.7131 | Yes |
| 18 | Pycard | 499 | 1.449 | 0.7385 | Yes |
| 19 | H2-Ab1 | 778 | 0.895 | 0.6972 | No |
| 20 | Cd74 | 811 | 0.856 | 0.7065 | No |
| 21 | Psmb8 | 1067 | 0.589 | 0.6643 | No |
| 22 | Nupr1 | 1092 | 0.562 | 0.6698 | No |
| 23 | Ptpn1 | 1126 | 0.534 | 0.6729 | No |
| 24 | Tmem176a | 1324 | -0.522 | 0.6415 | No |
| 25 | Tmem176b | 1326 | -0.522 | 0.6511 | No |
| 26 | Calm1 | 1839 | -0.607 | 0.5556 | No |
| 27 | Selenos | 2616 | -0.752 | 0.4077 | No |
| 28 | Cirbp | 3148 | -0.884 | 0.3134 | No |
| 29 | Cd24a | 4541 | -1.891 | 0.0582 | No |
Table: GSEA details [plain text format]

  

Fig 2: TABULA\_MURIS\_SENIS\_MARROW\_PROMONOCYTE\_AGEING: Random ES distribution      
 Gene set null distribution of ES for **TABULA\_MURIS\_SENIS\_MARROW\_PROMONOCYTE\_AGEING**

  
