# Supplementary material for: TMPRSS11B promotes an acidified microenvironment and immune suppression in squamous lung cancer
Source: EMBO Rep. 2025 Nov 10;26(24):6346–79. doi: 10.1038/s44319-025-00631-1 (PMC12714794; doi:10.1038/s44319-025-00631-1)
Supplement: Supplementary file 10 — Source data Fig. 5 [file 44319_2025_631_MOESM10_ESM.zip › Figure 5/5C-D/GSEA_Broad Institute_M8_T11b-high LUSC vs LUAD/TABULA_MURIS_SENIS_PANCREAS_PANCREATIC_ALPHA_CELL_AGEING.html]

Details for gene set TABULA\_MURIS\_SENIS\_PANCREAS\_PANCREATIC\_ALPHA\_CELL\_AGEING[GSEA]

|  || Dataset | Ranked list\_DGE\_squamousT11b\_vs\_all adenosadeno\_HSE13-NT copy |
| Phenotype | NoPhenotypeAvailable |
| Upregulated in class | na\_neg |
| GeneSet | TABULA\_MURIS\_SENIS\_PANCREAS\_PANCREATIC\_ALPHA\_CELL\_AGEING |
| Enrichment Score (ES) | -0.1859539 |
| Normalized Enrichment Score (NES) | -0.8609171 |
| Nominal p-value | 0.68 |
| FDR q-value | 1.0 |
| FWER p-Value | 1.0 |
Table: GSEA Results Summary

  

Fig 1: Enrichment plot: TABULA\_MURIS\_SENIS\_PANCREAS\_PANCREATIC\_ALPHA\_CELL\_AGEING      
 Profile of the Running ES Score & Positions of GeneSet Members on the Rank Ordered List

  

| SYMBOL | RANK IN GENE LIST | RANK METRIC SCORE | RUNNING ES | CORE ENRICHMENT || 1 | Rnase1 | 102 | 3.680 | 0.0483 | Yes |
| 2 | Trem2 | 140 | 3.156 | 0.1003 | Yes |
| 3 | Fth1 | 289 | 2.129 | 0.1097 | Yes |
| 4 | Dusp1 | 339 | 1.923 | 0.1358 | Yes |
| 5 | Klf4 | 448 | 1.555 | 0.1426 | Yes |
| 6 | Ppp1r15a | 543 | 1.345 | 0.1484 | Yes |
| 7 | Atf3 | 643 | 1.106 | 0.1487 | Yes |
| 8 | C1qa | 710 | 0.990 | 0.1536 | Yes |
| 9 | Ece1 | 739 | 0.953 | 0.1658 | Yes |
| 10 | Cldn4 | 764 | 0.911 | 0.1780 | Yes |
| 11 | Cdkn1a | 861 | 0.808 | 0.1732 | No |
| 12 | Kdm6b | 1191 | -0.504 | 0.1139 | No |
| 13 | Pcbp1 | 1248 | -0.513 | 0.1119 | No |
| 14 | Map1lc3a | 1273 | -0.516 | 0.1166 | No |
| 15 | Mapre3 | 1300 | -0.519 | 0.1210 | No |
| 16 | Prkaca | 1381 | -0.530 | 0.1143 | No |
| 17 | Tle5 | 1425 | -0.536 | 0.1154 | No |
| 18 | Hsp90aa1 | 1647 | -0.575 | 0.0800 | No |
| 19 | Pebp1 | 1685 | -0.581 | 0.0833 | No |
| 20 | Rbm26 | 1710 | -0.583 | 0.0893 | No |
| 21 | Emc10 | 1744 | -0.590 | 0.0936 | No |
| 22 | Arglu1 | 2318 | -0.692 | -0.0132 | No |
| 23 | Sdc4 | 2418 | -0.710 | -0.0205 | No |
| 24 | Calm3 | 2555 | -0.737 | -0.0350 | No |
| 25 | Selenos | 2616 | -0.752 | -0.0334 | No |
| 26 | Sod1 | 2688 | -0.765 | -0.0337 | No |
| 27 | Bsg | 2764 | -0.783 | -0.0346 | No |
| 28 | Itm2c | 2893 | -0.813 | -0.0460 | No |
| 29 | Ifi27 | 2905 | -0.815 | -0.0329 | No |
| 30 | Cirbp | 3148 | -0.884 | -0.0668 | No |
| 31 | Rbm39 | 3394 | -0.958 | -0.0999 | No |
| 32 | Fos | 3575 | -1.023 | -0.1182 | No |
| 33 | Tsc22d1 | 3676 | -1.066 | -0.1190 | No |
| 34 | Xbp1 | 3997 | -1.246 | -0.1624 | No |
| 35 | Btg2 | 4032 | -1.269 | -0.1455 | No |
| 36 | Tob1 | 4038 | -1.273 | -0.1224 | No |
| 37 | Ptov1 | 4177 | -1.389 | -0.1250 | No |
| 38 | Lgals3bp | 4189 | -1.402 | -0.1008 | No |
| 39 | Krt8 | 4476 | -1.778 | -0.1270 | No |
| 40 | Cela1 | 4538 | -1.888 | -0.1040 | No |
| 41 | Ccnd2 | 4672 | -2.265 | -0.0889 | No |
| 42 | Ddc | 4790 | -3.065 | -0.0554 | No |
| 43 | Reg1 | 4802 | -3.247 | 0.0038 | No |
Table: GSEA details [plain text format]

  

Fig 2: TABULA\_MURIS\_SENIS\_PANCREAS\_PANCREATIC\_ALPHA\_CELL\_AGEING: Random ES distribution      
 Gene set null distribution of ES for **TABULA\_MURIS\_SENIS\_PANCREAS\_PANCREATIC\_ALPHA\_CELL\_AGEING**

  
