# Supplementary material for: TMPRSS11B promotes an acidified microenvironment and immune suppression in squamous lung cancer
Source: EMBO Rep. 2025 Nov 10;26(24):6346–79. doi: 10.1038/s44319-025-00631-1 (PMC12714794; doi:10.1038/s44319-025-00631-1)
Supplement: Supplementary file 10 — Source data Fig. 5 [file 44319_2025_631_MOESM10_ESM.zip › Figure 5/5C-D/GSEA_Broad Institute_M8_T11b-high LUSC vs LUAD/TABULA_MURIS_SENIS_PANCREAS_PANCREATIC_DUCTAL_CELL_AGEING.html]

Details for gene set TABULA\_MURIS\_SENIS\_PANCREAS\_PANCREATIC\_DUCTAL\_CELL\_AGEING[GSEA]

|  || Dataset | Ranked list\_DGE\_squamousT11b\_vs\_all adenosadeno\_HSE13-NT copy |
| Phenotype | NoPhenotypeAvailable |
| Upregulated in class | na\_neg |
| GeneSet | TABULA\_MURIS\_SENIS\_PANCREAS\_PANCREATIC\_DUCTAL\_CELL\_AGEING |
| Enrichment Score (ES) | -0.20896935 |
| Normalized Enrichment Score (NES) | -1.1918238 |
| Nominal p-value | 0.15451895 |
| FDR q-value | 1.0 |
| FWER p-Value | 1.0 |
Table: GSEA Results Summary

  

Fig 1: Enrichment plot: TABULA\_MURIS\_SENIS\_PANCREAS\_PANCREATIC\_DUCTAL\_CELL\_AGEING      
 Profile of the Running ES Score & Positions of GeneSet Members on the Rank Ordered List

  

| SYMBOL | RANK IN GENE LIST | RANK METRIC SCORE | RUNNING ES | CORE ENRICHMENT || 1 | Plet1 | 190 | 2.674 | -0.0178 | No |
| 2 | Ly6a | 278 | 2.197 | -0.0178 | No |
| 3 | Lcn2 | 283 | 2.163 | -0.0004 | No |
| 4 | Fth1 | 289 | 2.129 | 0.0164 | No |
| 5 | Csf2ra | 299 | 2.108 | 0.0323 | No |
| 6 | C3 | 474 | 1.502 | 0.0080 | No |
| 7 | Znrf3 | 528 | 1.375 | 0.0083 | No |
| 8 | Tmem37 | 539 | 1.356 | 0.0176 | No |
| 9 | S100a16 | 569 | 1.288 | 0.0223 | No |
| 10 | Prdx5 | 601 | 1.198 | 0.0258 | No |
| 11 | Sat1 | 614 | 1.180 | 0.0332 | No |
| 12 | Osgin1 | 663 | 1.064 | 0.0319 | No |
| 13 | B2m | 794 | 0.876 | 0.0117 | No |
| 14 | Cryab | 800 | 0.869 | 0.0180 | No |
| 15 | Gadd45b | 804 | 0.862 | 0.0246 | No |
| 16 | Nucb2 | 893 | 0.764 | 0.0123 | No |
| 17 | H2-Q6 | 999 | 0.650 | -0.0045 | No |
| 18 | H2-D1 | 1021 | 0.632 | -0.0036 | No |
| 19 | Psmb8 | 1067 | 0.589 | -0.0082 | No |
| 20 | Rbp1 | 1091 | 0.565 | -0.0084 | No |
| 21 | Nupr1 | 1092 | 0.562 | -0.0036 | No |
| 22 | Kdm6b | 1191 | -0.504 | -0.0202 | No |
| 23 | Cfap298 | 1200 | -0.506 | -0.0176 | No |
| 24 | Ift27 | 1244 | -0.511 | -0.0224 | No |
| 25 | Tmsb4x | 1258 | -0.514 | -0.0209 | No |
| 26 | Map1lc3a | 1273 | -0.516 | -0.0195 | No |
| 27 | Eapp | 1305 | -0.519 | -0.0217 | No |
| 28 | Wiz | 1322 | -0.522 | -0.0207 | No |
| 29 | Tmem176a | 1324 | -0.522 | -0.0165 | No |
| 30 | Tmem176b | 1326 | -0.522 | -0.0124 | No |
| 31 | Zfp414 | 1362 | -0.528 | -0.0153 | No |
| 32 | Mapk3 | 1369 | -0.529 | -0.0122 | No |
| 33 | Abl1 | 1424 | -0.536 | -0.0191 | No |
| 34 | Tle5 | 1425 | -0.536 | -0.0146 | No |
| 35 | Dapk3 | 1439 | -0.539 | -0.0128 | No |
| 36 | Mgst1 | 1519 | -0.554 | -0.0249 | No |
| 37 | Nectin2 | 1531 | -0.558 | -0.0226 | No |
| 38 | Bcap31 | 1622 | -0.572 | -0.0369 | No |
| 39 | Myo10 | 1627 | -0.573 | -0.0329 | No |
| 40 | Pebp1 | 1685 | -0.581 | -0.0401 | No |
| 41 | Rtf1 | 1697 | -0.583 | -0.0375 | No |
| 42 | Rbm26 | 1710 | -0.583 | -0.0352 | No |
| 43 | Vps72 | 1737 | -0.589 | -0.0357 | No |
| 44 | Cic | 1825 | -0.604 | -0.0491 | No |
| 45 | Nr2c2ap | 1856 | -0.612 | -0.0503 | No |
| 46 | Eif1 | 1859 | -0.612 | -0.0456 | No |
| 47 | Ppp1r35 | 2003 | -0.637 | -0.0706 | No |
| 48 | Ergic1 | 2053 | -0.644 | -0.0756 | No |
| 49 | Atn1 | 2079 | -0.650 | -0.0754 | No |
| 50 | Ubl7 | 2146 | -0.662 | -0.0839 | No |
| 51 | Txn2 | 2163 | -0.664 | -0.0817 | No |
| 52 | Pdcd2 | 2179 | -0.666 | -0.0793 | No |
| 53 | Kpna4 | 2268 | -0.683 | -0.0922 | No |
| 54 | Spag7 | 2338 | -0.695 | -0.1010 | No |
| 55 | Aldh2 | 2388 | -0.704 | -0.1055 | No |
| 56 | 2610528J11Rik | 2396 | -0.705 | -0.1010 | No |
| 57 | Sdc4 | 2418 | -0.710 | -0.0995 | No |
| 58 | Smagp | 2447 | -0.717 | -0.0994 | No |
| 59 | Selenos | 2616 | -0.752 | -0.1288 | No |
| 60 | Ndufa9 | 2646 | -0.758 | -0.1285 | No |
| 61 | Sod1 | 2688 | -0.765 | -0.1308 | No |
| 62 | Ppa1 | 2690 | -0.766 | -0.1246 | No |
| 63 | Polr3gl | 2763 | -0.783 | -0.1333 | No |
| 64 | Bsg | 2764 | -0.783 | -0.1267 | No |
| 65 | Smim19 | 2821 | -0.795 | -0.1319 | No |
| 66 | Ifi27 | 2905 | -0.815 | -0.1426 | No |
| 67 | Gadd45gip1 | 3033 | -0.850 | -0.1624 | No |
| 68 | Gjb1 | 3084 | -0.866 | -0.1658 | No |
| 69 | Zscan26 | 3092 | -0.868 | -0.1599 | No |
| 70 | Cirbp | 3148 | -0.884 | -0.1642 | No |
| 71 | Gsta3 | 3234 | -0.910 | -0.1746 | No |
| 72 | Paip1 | 3260 | -0.918 | -0.1721 | No |
| 73 | Tmem59 | 3397 | -0.960 | -0.1929 | No |
| 74 | Kdsr | 3402 | -0.961 | -0.1857 | No |
| 75 | Ssbp3 | 3427 | -0.974 | -0.1826 | No |
| 76 | Rita1 | 3476 | -0.988 | -0.1845 | No |
| 77 | Fos | 3575 | -1.023 | -0.1967 | No |
| 78 | Fnbp1l | 3600 | -1.034 | -0.1930 | No |
| 79 | Tsc22d1 | 3676 | -1.066 | -0.2000 | Yes |
| 80 | Cavin1 | 3689 | -1.073 | -0.1935 | Yes |
| 81 | Thap3 | 3727 | -1.093 | -0.1922 | Yes |
| 82 | Macrod1 | 3732 | -1.095 | -0.1838 | Yes |
| 83 | Marf1 | 3764 | -1.108 | -0.1810 | Yes |
| 84 | Smco4 | 3805 | -1.131 | -0.1800 | Yes |
| 85 | Dynll2 | 3904 | -1.185 | -0.1908 | Yes |
| 86 | Bri3 | 3905 | -1.186 | -0.1808 | Yes |
| 87 | Spry2 | 3925 | -1.200 | -0.1748 | Yes |
| 88 | Qsox1 | 3954 | -1.217 | -0.1705 | Yes |
| 89 | Cdc42ep5 | 4009 | -1.255 | -0.1714 | Yes |
| 90 | Tcf7l2 | 4036 | -1.272 | -0.1662 | Yes |
| 91 | Chd6 | 4079 | -1.311 | -0.1641 | Yes |
| 92 | Zfp787 | 4105 | -1.333 | -0.1582 | Yes |
| 93 | Tmem9 | 4164 | -1.381 | -0.1588 | Yes |
| 94 | Nr2f2 | 4183 | -1.396 | -0.1509 | Yes |
| 95 | Lgals3bp | 4189 | -1.402 | -0.1402 | Yes |
| 96 | Fuz | 4198 | -1.408 | -0.1300 | Yes |
| 97 | Gstm1 | 4200 | -1.410 | -0.1183 | Yes |
| 98 | Spr | 4273 | -1.481 | -0.1212 | Yes |
| 99 | Lmo4 | 4382 | -1.617 | -0.1305 | Yes |
| 100 | Tm4sf1 | 4421 | -1.676 | -0.1244 | Yes |
| 101 | Zfp704 | 4443 | -1.725 | -0.1144 | Yes |
| 102 | Nav2 | 4458 | -1.753 | -0.1026 | Yes |
| 103 | Gstm2 | 4466 | -1.761 | -0.0892 | Yes |
| 104 | Cbx7 | 4479 | -1.780 | -0.0768 | Yes |
| 105 | Igsf5 | 4549 | -1.912 | -0.0753 | Yes |
| 106 | Capsl | 4550 | -1.913 | -0.0592 | Yes |
| 107 | Echdc2 | 4557 | -1.936 | -0.0442 | Yes |
| 108 | Lrrc75a | 4567 | -1.957 | -0.0296 | Yes |
| 109 | Ccnd2 | 4672 | -2.265 | -0.0326 | Yes |
| 110 | Lurap1l | 4679 | -2.307 | -0.0145 | Yes |
| 111 | Arhgap44 | 4744 | -2.611 | -0.0061 | Yes |
| 112 | Adh1 | 4746 | -2.614 | 0.0157 | Yes |
Table: GSEA details [plain text format]

  

Fig 2: TABULA\_MURIS\_SENIS\_PANCREAS\_PANCREATIC\_DUCTAL\_CELL\_AGEING: Random ES distribution      
 Gene set null distribution of ES for **TABULA\_MURIS\_SENIS\_PANCREAS\_PANCREATIC\_DUCTAL\_CELL\_AGEING**

  
