# Supplementary material for: TMPRSS11B promotes an acidified microenvironment and immune suppression in squamous lung cancer
Source: EMBO Rep. 2025 Nov 10;26(24):6346–79. doi: 10.1038/s44319-025-00631-1 (PMC12714794; doi:10.1038/s44319-025-00631-1)
Supplement: Supplementary file 10 — Source data Fig. 5 [file 44319_2025_631_MOESM10_ESM.zip › Figure 5/5C-D/GSEA_Broad Institute_M8_T11b-high LUSC vs LUAD/TABULA_MURIS_SENIS_PANCREAS_PANCREATIC_POLYPEPTIDE_CELL_AGEING.html]

Details for gene set TABULA\_MURIS\_SENIS\_PANCREAS\_PANCREATIC\_POLYPEPTIDE\_CELL\_AGEING[GSEA]

|  || Dataset | Ranked list\_DGE\_squamousT11b\_vs\_all adenosadeno\_HSE13-NT copy |
| Phenotype | NoPhenotypeAvailable |
| Upregulated in class | na\_neg |
| GeneSet | TABULA\_MURIS\_SENIS\_PANCREAS\_PANCREATIC\_POLYPEPTIDE\_CELL\_AGEING |
| Enrichment Score (ES) | -0.20308311 |
| Normalized Enrichment Score (NES) | -0.9417457 |
| Nominal p-value | 0.5550351 |
| FDR q-value | 1.0 |
| FWER p-Value | 1.0 |
Table: GSEA Results Summary

  

Fig 1: Enrichment plot: TABULA\_MURIS\_SENIS\_PANCREAS\_PANCREATIC\_POLYPEPTIDE\_CELL\_AGEING      
 Profile of the Running ES Score & Positions of GeneSet Members on the Rank Ordered List

  

| SYMBOL | RANK IN GENE LIST | RANK METRIC SCORE | RUNNING ES | CORE ENRICHMENT || 1 | Dusp1 | 339 | 1.923 | -0.0146 | No |
| 2 | Dnajb1 | 453 | 1.542 | 0.0070 | No |
| 3 | Prelid1 | 751 | 0.932 | -0.0278 | No |
| 4 | Cfap298 | 1200 | -0.506 | -0.1067 | No |
| 5 | Map1lc3a | 1273 | -0.516 | -0.1066 | No |
| 6 | Klc3 | 1412 | -0.534 | -0.1198 | No |
| 7 | Tle5 | 1425 | -0.536 | -0.1066 | No |
| 8 | Trappc5 | 1635 | -0.573 | -0.1335 | No |
| 9 | Pebp1 | 1685 | -0.581 | -0.1268 | No |
| 10 | Emc10 | 1744 | -0.590 | -0.1216 | No |
| 11 | Ctnnbl1 | 1854 | -0.611 | -0.1265 | No |
| 12 | Rab12 | 1922 | -0.622 | -0.1223 | No |
| 13 | Pnrc1 | 1935 | -0.624 | -0.1065 | No |
| 14 | Tex261 | 2206 | -0.672 | -0.1433 | No |
| 15 | Shisa5 | 2493 | -0.727 | -0.1818 | Yes |
| 16 | Mmadhc | 2569 | -0.741 | -0.1758 | Yes |
| 17 | Selenos | 2616 | -0.752 | -0.1634 | Yes |
| 18 | Ppa1 | 2690 | -0.766 | -0.1562 | Yes |
| 19 | Bsg | 2764 | -0.783 | -0.1485 | Yes |
| 20 | Nudt16 | 2959 | -0.829 | -0.1648 | Yes |
| 21 | Ssb | 2962 | -0.829 | -0.1409 | Yes |
| 22 | Lrig1 | 3028 | -0.849 | -0.1296 | Yes |
| 23 | Sdr39u1 | 3244 | -0.912 | -0.1479 | Yes |
| 24 | Alkbh6 | 3283 | -0.924 | -0.1288 | Yes |
| 25 | Ppa2 | 3287 | -0.925 | -0.1023 | Yes |
| 26 | Ilkap | 3324 | -0.938 | -0.0823 | Yes |
| 27 | Papss2 | 3416 | -0.969 | -0.0730 | Yes |
| 28 | Lsr | 3431 | -0.976 | -0.0473 | Yes |
| 29 | Pofut1 | 3507 | -1.000 | -0.0337 | Yes |
| 30 | Fos | 3575 | -1.023 | -0.0178 | Yes |
| 31 | Cbr1 | 3607 | -1.035 | 0.0061 | Yes |
| 32 | Agtrap | 3609 | -1.035 | 0.0362 | Yes |
| 33 | Tsc22d1 | 3676 | -1.066 | 0.0536 | Yes |
| 34 | Foxa3 | 3746 | -1.101 | 0.0714 | Yes |
| 35 | Erlec1 | 3766 | -1.108 | 0.0999 | Yes |
| 36 | Gal3st1 | 3958 | -1.220 | 0.0957 | Yes |
| 37 | Bace2 | 4135 | -1.360 | 0.0987 | Yes |
| 38 | Casz1 | 4308 | -1.511 | 0.1070 | Yes |
Table: GSEA details [plain text format]

  

Fig 2: TABULA\_MURIS\_SENIS\_PANCREAS\_PANCREATIC\_POLYPEPTIDE\_CELL\_AGEING: Random ES distribution      
 Gene set null distribution of ES for **TABULA\_MURIS\_SENIS\_PANCREAS\_PANCREATIC\_POLYPEPTIDE\_CELL\_AGEING**

  
