# Supplementary material for: TMPRSS11B promotes an acidified microenvironment and immune suppression in squamous lung cancer
Source: EMBO Rep. 2025 Nov 10;26(24):6346–79. doi: 10.1038/s44319-025-00631-1 (PMC12714794; doi:10.1038/s44319-025-00631-1)
Supplement: Supplementary file 10 — Source data Fig. 5 [file 44319_2025_631_MOESM10_ESM.zip › Figure 5/5C-D/GSEA_Broad Institute_M8_T11b-high LUSC vs LUAD/TABULA_MURIS_SENIS_SPLEEN_B_CELL_AGEING.html]

Details for gene set TABULA\_MURIS\_SENIS\_SPLEEN\_B\_CELL\_AGEING[GSEA]

|  || Dataset | Ranked list\_DGE\_squamousT11b\_vs\_all adenosadeno\_HSE13-NT copy |
| Phenotype | NoPhenotypeAvailable |
| Upregulated in class | na\_pos |
| GeneSet | TABULA\_MURIS\_SENIS\_SPLEEN\_B\_CELL\_AGEING |
| Enrichment Score (ES) | 0.60437614 |
| Normalized Enrichment Score (NES) | 2.4969926 |
| Nominal p-value | 0.0 |
| FDR q-value | 0.0 |
| FWER p-Value | 0.0 |
Table: GSEA Results Summary

  

Fig 1: Enrichment plot: TABULA\_MURIS\_SENIS\_SPLEEN\_B\_CELL\_AGEING      
 Profile of the Running ES Score & Positions of GeneSet Members on the Rank Ordered List

  

| SYMBOL | RANK IN GENE LIST | RANK METRIC SCORE | RUNNING ES | CORE ENRICHMENT || 1 | S100a8 | 93 | 3.788 | 0.0517 | Yes |
| 2 | S100a9 | 110 | 3.624 | 0.1164 | Yes |
| 3 | Cybb | 173 | 2.805 | 0.1561 | Yes |
| 4 | Srgn | 185 | 2.715 | 0.2047 | Yes |
| 5 | Ly6a | 278 | 2.197 | 0.2267 | Yes |
| 6 | Fcgr2b | 280 | 2.178 | 0.2674 | Yes |
| 7 | Evi2a | 309 | 2.048 | 0.3000 | Yes |
| 8 | Cd44 | 329 | 1.976 | 0.3331 | Yes |
| 9 | Bcl2a1b | 349 | 1.876 | 0.3644 | Yes |
| 10 | Lgals1 | 400 | 1.684 | 0.3856 | Yes |
| 11 | Syk | 459 | 1.531 | 0.4022 | Yes |
| 12 | Lat2 | 477 | 1.499 | 0.4268 | Yes |
| 13 | Fxyd5 | 489 | 1.476 | 0.4522 | Yes |
| 14 | Apoe | 490 | 1.475 | 0.4799 | Yes |
| 15 | Pycard | 499 | 1.449 | 0.5054 | Yes |
| 16 | Psap | 510 | 1.415 | 0.5299 | Yes |
| 17 | Emp3 | 537 | 1.365 | 0.5501 | Yes |
| 18 | Capg | 574 | 1.263 | 0.5663 | Yes |
| 19 | Sat1 | 614 | 1.180 | 0.5803 | Yes |
| 20 | Txn1 | 743 | 0.944 | 0.5712 | Yes |
| 21 | H2-Ab1 | 778 | 0.895 | 0.5809 | Yes |
| 22 | B2m | 794 | 0.876 | 0.5942 | Yes |
| 23 | Npc2 | 822 | 0.841 | 0.6044 | Yes |
| 24 | H2-Eb1 | 976 | 0.681 | 0.5852 | No |
| 25 | H2-D1 | 1021 | 0.632 | 0.5878 | No |
| 26 | Psmb8 | 1067 | 0.589 | 0.5895 | No |
| 27 | Ahnak | 1090 | 0.565 | 0.5955 | No |
| 28 | Ptpn1 | 1126 | 0.534 | 0.5982 | No |
| 29 | Itgb1 | 1391 | -0.531 | 0.5530 | No |
| 30 | Tpt1 | 1787 | -0.598 | 0.4817 | No |
| 31 | Fam3c | 2384 | -0.703 | 0.3703 | No |
| 32 | Itm2c | 2893 | -0.813 | 0.2793 | No |
| 33 | Cirbp | 3148 | -0.884 | 0.2428 | No |
| 34 | Fos | 3575 | -1.023 | 0.1730 | No |
| 35 | Tnfaip8 | 3897 | -1.181 | 0.1281 | No |
| 36 | Zbtb20 | 4171 | -1.387 | 0.0970 | No |
| 37 | Tcf4 | 4599 | -2.046 | 0.0462 | No |
Table: GSEA details [plain text format]

  

Fig 2: TABULA\_MURIS\_SENIS\_SPLEEN\_B\_CELL\_AGEING: Random ES distribution      
 Gene set null distribution of ES for **TABULA\_MURIS\_SENIS\_SPLEEN\_B\_CELL\_AGEING**

  
