# Supplementary material for: TMPRSS11B promotes an acidified microenvironment and immune suppression in squamous lung cancer
Source: EMBO Rep. 2025 Nov 10;26(24):6346–79. doi: 10.1038/s44319-025-00631-1 (PMC12714794; doi:10.1038/s44319-025-00631-1)
Supplement: Supplementary file 10 — Source data Fig. 5 [file 44319_2025_631_MOESM10_ESM.zip › Figure 5/5C-D/GSEA_Broad Institute_M8_T11b-high LUSC vs LUAD/TABULA_MURIS_SENIS_SPLEEN_MACROPHAGE_AGEING.html]

Details for gene set TABULA\_MURIS\_SENIS\_SPLEEN\_MACROPHAGE\_AGEING[GSEA]

|  || Dataset | Ranked list\_DGE\_squamousT11b\_vs\_all adenosadeno\_HSE13-NT copy |
| Phenotype | NoPhenotypeAvailable |
| Upregulated in class | na\_pos |
| GeneSet | TABULA\_MURIS\_SENIS\_SPLEEN\_MACROPHAGE\_AGEING |
| Enrichment Score (ES) | 0.650869 |
| Normalized Enrichment Score (NES) | 2.9857597 |
| Nominal p-value | 0.0 |
| FDR q-value | 0.0 |
| FWER p-Value | 0.0 |
Table: GSEA Results Summary

  

Fig 1: Enrichment plot: TABULA\_MURIS\_SENIS\_SPLEEN\_MACROPHAGE\_AGEING      
 Profile of the Running ES Score & Positions of GeneSet Members on the Rank Ordered List

  

| SYMBOL | RANK IN GENE LIST | RANK METRIC SCORE | RUNNING ES | CORE ENRICHMENT || 1 | Ppbp | 28 | 5.754 | 0.0613 | Yes |
| 2 | S100a8 | 93 | 3.788 | 0.0921 | Yes |
| 3 | S100a9 | 110 | 3.624 | 0.1311 | Yes |
| 4 | Cybb | 173 | 2.805 | 0.1508 | Yes |
| 5 | Slpi | 220 | 2.439 | 0.1697 | Yes |
| 6 | Il1b | 240 | 2.351 | 0.1931 | Yes |
| 7 | Wfdc17 | 241 | 2.337 | 0.2204 | Yes |
| 8 | Ctss | 247 | 2.317 | 0.2464 | Yes |
| 9 | Lyz1 | 254 | 2.301 | 0.2720 | Yes |
| 10 | Cd300c2 | 258 | 2.288 | 0.2981 | Yes |
| 11 | Fcer1g | 272 | 2.235 | 0.3215 | Yes |
| 12 | Ctsd | 274 | 2.219 | 0.3472 | Yes |
| 13 | Ly6a | 278 | 2.197 | 0.3723 | Yes |
| 14 | Ctsb | 288 | 2.139 | 0.3954 | Yes |
| 15 | Fth1 | 289 | 2.129 | 0.4202 | Yes |
| 16 | Mpeg1 | 346 | 1.889 | 0.4305 | Yes |
| 17 | Lgals1 | 400 | 1.684 | 0.4391 | Yes |
| 18 | Ifitm1 | 427 | 1.619 | 0.4525 | Yes |
| 19 | Cfp | 433 | 1.599 | 0.4701 | Yes |
| 20 | Lgmn | 458 | 1.534 | 0.4830 | Yes |
| 21 | Apoe | 490 | 1.475 | 0.4937 | Yes |
| 22 | Creg1 | 533 | 1.369 | 0.5009 | Yes |
| 23 | Acp5 | 536 | 1.366 | 0.5164 | Yes |
| 24 | Grn | 554 | 1.328 | 0.5284 | Yes |
| 25 | Cstb | 587 | 1.229 | 0.5360 | Yes |
| 26 | Prdx5 | 601 | 1.198 | 0.5473 | Yes |
| 27 | Sat1 | 614 | 1.180 | 0.5585 | Yes |
| 28 | C1qb | 615 | 1.180 | 0.5723 | Yes |
| 29 | Rgs10 | 660 | 1.068 | 0.5755 | Yes |
| 30 | Trf | 682 | 1.033 | 0.5832 | Yes |
| 31 | Atp1b3 | 686 | 1.028 | 0.5946 | Yes |
| 32 | C1qa | 710 | 0.990 | 0.6013 | Yes |
| 33 | Blvrb | 731 | 0.962 | 0.6083 | Yes |
| 34 | Txn1 | 743 | 0.944 | 0.6171 | Yes |
| 35 | Csf1r | 747 | 0.942 | 0.6274 | Yes |
| 36 | Ctsc | 770 | 0.907 | 0.6334 | Yes |
| 37 | C1qc | 775 | 0.902 | 0.6431 | Yes |
| 38 | Npc2 | 822 | 0.841 | 0.6433 | Yes |
| 39 | Ninj1 | 833 | 0.831 | 0.6509 | Yes |
| 40 | Flna | 905 | 0.755 | 0.6448 | No |
| 41 | Atp6v0e | 942 | 0.714 | 0.6456 | No |
| 42 | Msrb1 | 982 | 0.669 | 0.6452 | No |
| 43 | Slc3a2 | 1041 | 0.613 | 0.6402 | No |
| 44 | Litaf | 1060 | 0.599 | 0.6434 | No |
| 45 | Tmbim4 | 1103 | 0.550 | 0.6410 | No |
| 46 | Ptpn1 | 1126 | 0.534 | 0.6426 | No |
| 47 | Gng11 | 1167 | 0.502 | 0.6400 | No |
| 48 | Tmem176a | 1324 | -0.522 | 0.6134 | No |
| 49 | Tmem176b | 1326 | -0.522 | 0.6193 | No |
| 50 | App | 1359 | -0.528 | 0.6187 | No |
| 51 | P4hb | 1469 | -0.546 | 0.6022 | No |
| 52 | Plaat3 | 1505 | -0.552 | 0.6013 | No |
| 53 | Rpp21 | 1595 | -0.567 | 0.5892 | No |
| 54 | Mt1 | 1658 | -0.576 | 0.5829 | No |
| 55 | Cmtm7 | 2316 | -0.692 | 0.4529 | No |
| 56 | Aldh2 | 2388 | -0.704 | 0.4463 | No |
| 57 | Smagp | 2447 | -0.717 | 0.4424 | No |
| 58 | Sod1 | 2688 | -0.765 | 0.4010 | No |
| 59 | Fabp5 | 2745 | -0.778 | 0.3983 | No |
| 60 | Basp1 | 4338 | -1.550 | 0.0819 | No |
| 61 | Selenop | 4397 | -1.636 | 0.0889 | No |
Table: GSEA details [plain text format]

  

Fig 2: TABULA\_MURIS\_SENIS\_SPLEEN\_MACROPHAGE\_AGEING: Random ES distribution      
 Gene set null distribution of ES for **TABULA\_MURIS\_SENIS\_SPLEEN\_MACROPHAGE\_AGEING**

  
