# Supplementary material for: TMPRSS11B promotes an acidified microenvironment and immune suppression in squamous lung cancer
Source: EMBO Rep. 2025 Nov 10;26(24):6346–79. doi: 10.1038/s44319-025-00631-1 (PMC12714794; doi:10.1038/s44319-025-00631-1)
Supplement: Supplementary file 10 — Source data Fig. 5 [file 44319_2025_631_MOESM10_ESM.zip › Figure 5/5C-D/GSEA_Broad Institute_M8_T11b-high LUSC vs LUAD/TABULA_MURIS_SENIS_TONGUE_BASAL_CELL_OF_EPIDERMIS_AGEING.html]

Details for gene set TABULA\_MURIS\_SENIS\_TONGUE\_BASAL\_CELL\_OF\_EPIDERMIS\_AGEING[GSEA]

|  || Dataset | Ranked list\_DGE\_squamousT11b\_vs\_all adenosadeno\_HSE13-NT copy |
| Phenotype | NoPhenotypeAvailable |
| Upregulated in class | na\_pos |
| GeneSet | TABULA\_MURIS\_SENIS\_TONGUE\_BASAL\_CELL\_OF\_EPIDERMIS\_AGEING |
| Enrichment Score (ES) | 0.60171324 |
| Normalized Enrichment Score (NES) | 2.7639241 |
| Nominal p-value | 0.0 |
| FDR q-value | 0.0 |
| FWER p-Value | 0.0 |
Table: GSEA Results Summary

  

Fig 1: Enrichment plot: TABULA\_MURIS\_SENIS\_TONGUE\_BASAL\_CELL\_OF\_EPIDERMIS\_AGEING      
 Profile of the Running ES Score & Positions of GeneSet Members on the Rank Ordered List

  

| SYMBOL | RANK IN GENE LIST | RANK METRIC SCORE | RUNNING ES | CORE ENRICHMENT || 1 | Krtdap | 1 | 7.386 | 0.0785 | Yes |
| 2 | Krt6b | 3 | 7.285 | 0.1559 | Yes |
| 3 | Tgm3 | 4 | 7.271 | 0.2333 | Yes |
| 4 | Lypd3 | 15 | 6.488 | 0.3004 | Yes |
| 5 | Krt16 | 22 | 6.108 | 0.3642 | Yes |
| 6 | Cysrt1 | 81 | 3.947 | 0.3940 | Yes |
| 7 | Plet1 | 190 | 2.674 | 0.3998 | Yes |
| 8 | Slpi | 220 | 2.439 | 0.4197 | Yes |
| 9 | S100a14 | 253 | 2.301 | 0.4375 | Yes |
| 10 | Tubb6 | 357 | 1.861 | 0.4357 | Yes |
| 11 | Apoc1 | 386 | 1.739 | 0.4483 | Yes |
| 12 | Gsto1 | 397 | 1.701 | 0.4643 | Yes |
| 13 | Tacstd2 | 437 | 1.575 | 0.4729 | Yes |
| 14 | Lgals3 | 447 | 1.559 | 0.4876 | Yes |
| 15 | Apoe | 490 | 1.475 | 0.4945 | Yes |
| 16 | Zfand5 | 577 | 1.260 | 0.4899 | Yes |
| 17 | Ccna2 | 582 | 1.243 | 0.5023 | Yes |
| 18 | Cenpa | 604 | 1.193 | 0.5106 | Yes |
| 19 | Ehf | 698 | 1.007 | 0.5018 | Yes |
| 20 | Cks2 | 729 | 0.967 | 0.5058 | Yes |
| 21 | Cdca3 | 749 | 0.932 | 0.5117 | Yes |
| 22 | Cldn4 | 764 | 0.911 | 0.5185 | Yes |
| 23 | Phlda1 | 766 | 0.908 | 0.5280 | Yes |
| 24 | B2m | 794 | 0.876 | 0.5316 | Yes |
| 25 | Gadd45b | 804 | 0.862 | 0.5389 | Yes |
| 26 | Spc24 | 813 | 0.853 | 0.5463 | Yes |
| 27 | Cdk1 | 820 | 0.842 | 0.5540 | Yes |
| 28 | Cxcl16 | 855 | 0.811 | 0.5555 | Yes |
| 29 | Nfkbia | 887 | 0.772 | 0.5572 | Yes |
| 30 | Stx11 | 889 | 0.770 | 0.5652 | Yes |
| 31 | Nusap1 | 907 | 0.752 | 0.5697 | Yes |
| 32 | Ovol1 | 925 | 0.730 | 0.5739 | Yes |
| 33 | Cdca8 | 929 | 0.722 | 0.5809 | Yes |
| 34 | Incenp | 937 | 0.718 | 0.5871 | Yes |
| 35 | Ier3 | 967 | 0.691 | 0.5884 | Yes |
| 36 | Racgap1 | 968 | 0.689 | 0.5957 | Yes |
| 37 | H2-D1 | 1021 | 0.632 | 0.5915 | Yes |
| 38 | Pgk1 | 1029 | 0.624 | 0.5967 | Yes |
| 39 | Tk1 | 1045 | 0.611 | 0.6001 | Yes |
| 40 | Dut | 1068 | 0.589 | 0.6017 | Yes |
| 41 | Stmn1 | 1124 | 0.536 | 0.5959 | No |
| 42 | Smc4 | 1134 | 0.523 | 0.5996 | No |
| 43 | Gsta4 | 1227 | -0.509 | 0.5856 | No |
| 44 | Selenbp1 | 1398 | -0.532 | 0.5556 | No |
| 45 | Tle5 | 1425 | -0.536 | 0.5558 | No |
| 46 | Mgst1 | 1519 | -0.554 | 0.5422 | No |
| 47 | Pebp1 | 1685 | -0.581 | 0.5137 | No |
| 48 | Nudc | 1766 | -0.594 | 0.5033 | No |
| 49 | Tpt1 | 1787 | -0.598 | 0.5054 | No |
| 50 | Pnrc1 | 1935 | -0.624 | 0.4812 | No |
| 51 | Ptgr1 | 2287 | -0.685 | 0.4147 | No |
| 52 | Sod1 | 2688 | -0.765 | 0.3389 | No |
| 53 | Ppa1 | 2690 | -0.766 | 0.3468 | No |
| 54 | Fabp5 | 2745 | -0.778 | 0.3438 | No |
| 55 | Pmm1 | 2876 | -0.809 | 0.3251 | No |
| 56 | Ifi27 | 2905 | -0.815 | 0.3279 | No |
| 57 | Aldh3a1 | 3207 | -0.901 | 0.2742 | No |
| 58 | Tsc22d1 | 3676 | -1.066 | 0.1873 | No |
| 59 | Gstm1 | 4200 | -1.410 | 0.0924 | No |
| 60 | Adh7 | 4375 | -1.609 | 0.0730 | No |
| 61 | Shroom3 | 4545 | -1.904 | 0.0578 | No |
Table: GSEA details [plain text format]

  

Fig 2: TABULA\_MURIS\_SENIS\_TONGUE\_BASAL\_CELL\_OF\_EPIDERMIS\_AGEING: Random ES distribution      
 Gene set null distribution of ES for **TABULA\_MURIS\_SENIS\_TONGUE\_BASAL\_CELL\_OF\_EPIDERMIS\_AGEING**

  
