# Supplementary material for: TMPRSS11B promotes an acidified microenvironment and immune suppression in squamous lung cancer
Source: EMBO Rep. 2025 Nov 10;26(24):6346–79. doi: 10.1038/s44319-025-00631-1 (PMC12714794; doi:10.1038/s44319-025-00631-1)
Supplement: Supplementary file 10 — Source data Fig. 5 [file 44319_2025_631_MOESM10_ESM.zip › Figure 5/5C-D/GSEA_Broad Institute_M8_T11b-high LUSC vs LUAD/TABULA_MURIS_SENIS_TONGUE_KERATINOCYTE_AGEING.html]

Details for gene set TABULA\_MURIS\_SENIS\_TONGUE\_KERATINOCYTE\_AGEING[GSEA]

|  || Dataset | Ranked list\_DGE\_squamousT11b\_vs\_all adenosadeno\_HSE13-NT copy |
| Phenotype | NoPhenotypeAvailable |
| Upregulated in class | na\_pos |
| GeneSet | TABULA\_MURIS\_SENIS\_TONGUE\_KERATINOCYTE\_AGEING |
| Enrichment Score (ES) | 0.7025601 |
| Normalized Enrichment Score (NES) | 2.4771996 |
| Nominal p-value | 0.0 |
| FDR q-value | 0.0 |
| FWER p-Value | 0.0 |
Table: GSEA Results Summary

  

Fig 1: Enrichment plot: TABULA\_MURIS\_SENIS\_TONGUE\_KERATINOCYTE\_AGEING      
 Profile of the Running ES Score & Positions of GeneSet Members on the Rank Ordered List

  

| SYMBOL | RANK IN GENE LIST | RANK METRIC SCORE | RUNNING ES | CORE ENRICHMENT || 1 | Krtdap | 1 | 7.386 | 0.1431 | Yes |
| 2 | Krt6b | 3 | 7.285 | 0.2842 | Yes |
| 3 | Tgm3 | 4 | 7.271 | 0.4253 | Yes |
| 4 | Krt16 | 22 | 6.108 | 0.5402 | Yes |
| 5 | Krt14 | 31 | 5.553 | 0.6463 | Yes |
| 6 | Slpi | 220 | 2.439 | 0.6545 | Yes |
| 7 | Nqo1 | 301 | 2.106 | 0.6787 | Yes |
| 8 | Apoc1 | 386 | 1.739 | 0.6949 | Yes |
| 9 | Ly6g6c | 488 | 1.479 | 0.7026 | Yes |
| 10 | Phlda1 | 766 | 0.908 | 0.6625 | No |
| 11 | B2m | 794 | 0.876 | 0.6738 | No |
| 12 | H2-D1 | 1021 | 0.632 | 0.6390 | No |
| 13 | Gsta4 | 1227 | -0.509 | 0.6062 | No |
| 14 | Tpt1 | 1787 | -0.598 | 0.5014 | No |
| 15 | Ptgr1 | 2287 | -0.685 | 0.4107 | No |
| 16 | Aldh3a1 | 3207 | -0.901 | 0.2368 | No |
| 17 | Ces1d | 3471 | -0.987 | 0.2012 | No |
| 18 | Pdzk1ip1 | 3668 | -1.063 | 0.1810 | No |
| 19 | Gstm1 | 4200 | -1.410 | 0.0977 | No |
| 20 | Adh7 | 4375 | -1.609 | 0.0927 | No |
Table: GSEA details [plain text format]

  

Fig 2: TABULA\_MURIS\_SENIS\_TONGUE\_KERATINOCYTE\_AGEING: Random ES distribution      
 Gene set null distribution of ES for **TABULA\_MURIS\_SENIS\_TONGUE\_KERATINOCYTE\_AGEING**

  
