# Supplementary material for: TMPRSS11B promotes an acidified microenvironment and immune suppression in squamous lung cancer
Source: EMBO Rep. 2025 Nov 10;26(24):6346–79. doi: 10.1038/s44319-025-00631-1 (PMC12714794; doi:10.1038/s44319-025-00631-1)
Supplement: Supplementary file 10 — Source data Fig. 5 [file 44319_2025_631_MOESM10_ESM.zip › Figure 5/5C-D/GSEA_Broad Institute_M8_T11b-high LUSC vs LUAD/ZHANG_UTERUS_C10_STROMAL2_RETNLG_HIGH_CELL.html]

Details for gene set ZHANG\_UTERUS\_C10\_STROMAL2\_RETNLG\_HIGH\_CELL[GSEA]

|  || Dataset | Ranked list\_DGE\_squamousT11b\_vs\_all adenosadeno\_HSE13-NT copy |
| Phenotype | NoPhenotypeAvailable |
| Upregulated in class | na\_pos |
| GeneSet | ZHANG\_UTERUS\_C10\_STROMAL2\_RETNLG\_HIGH\_CELL |
| Enrichment Score (ES) | 0.7220886 |
| Normalized Enrichment Score (NES) | 2.7879689 |
| Nominal p-value | 0.0 |
| FDR q-value | 0.0 |
| FWER p-Value | 0.0 |
Table: GSEA Results Summary

  

Fig 1: Enrichment plot: ZHANG\_UTERUS\_C10\_STROMAL2\_RETNLG\_HIGH\_CELL      
 Profile of the Running ES Score & Positions of GeneSet Members on the Rank Ordered List

  

| SYMBOL | RANK IN GENE LIST | RANK METRIC SCORE | RUNNING ES | CORE ENRICHMENT || 1 | Cxcl3 | 18 | 6.265 | 0.1026 | Yes |
| 2 | Serpinb2 | 89 | 3.864 | 0.1536 | Yes |
| 3 | Adam8 | 138 | 3.205 | 0.1980 | Yes |
| 4 | Cybb | 173 | 2.805 | 0.2386 | Yes |
| 5 | Csf2rb | 176 | 2.766 | 0.2851 | Yes |
| 6 | Tyrobp | 181 | 2.732 | 0.3307 | Yes |
| 7 | Srgn | 185 | 2.715 | 0.3761 | Yes |
| 8 | Ccl6 | 187 | 2.695 | 0.4217 | Yes |
| 9 | Pla2g7 | 195 | 2.599 | 0.4643 | Yes |
| 10 | Il1b | 240 | 2.351 | 0.4951 | Yes |
| 11 | Plek | 252 | 2.303 | 0.5319 | Yes |
| 12 | Slc7a11 | 277 | 2.198 | 0.5642 | Yes |
| 13 | Il1rn | 287 | 2.146 | 0.5988 | Yes |
| 14 | Fth1 | 289 | 2.129 | 0.6347 | Yes |
| 15 | Cd52 | 332 | 1.963 | 0.6593 | Yes |
| 16 | Rgs1 | 350 | 1.874 | 0.6876 | Yes |
| 17 | Cebpb | 425 | 1.620 | 0.6996 | Yes |
| 18 | Alox5ap | 500 | 1.445 | 0.7087 | Yes |
| 19 | Coro1a | 546 | 1.340 | 0.7221 | Yes |
| 20 | Mcl1 | 969 | 0.689 | 0.6457 | No |
| 21 | Msrb1 | 982 | 0.669 | 0.6546 | No |
| 22 | Trib1 | 1112 | 0.544 | 0.6369 | No |
| 23 | Grina | 1115 | 0.543 | 0.6457 | No |
| 24 | Tmsb4x | 1258 | -0.514 | 0.6248 | No |
| 25 | Laptm5 | 2847 | -0.801 | 0.3069 | No |
| 26 | Tgm2 | 2996 | -0.839 | 0.2903 | No |
| 27 | Vasp | 3174 | -0.891 | 0.2685 | No |
| 28 | Thbs1 | 3344 | -0.945 | 0.2492 | No |
| 29 | Basp1 | 4338 | -1.550 | 0.0683 | No |
| 30 | Cd24a | 4541 | -1.891 | 0.0582 | No |
Table: GSEA details [plain text format]

  

Fig 2: ZHANG\_UTERUS\_C10\_STROMAL2\_RETNLG\_HIGH\_CELL: Random ES distribution      
 Gene set null distribution of ES for **ZHANG\_UTERUS\_C10\_STROMAL2\_RETNLG\_HIGH\_CELL**

  
