# Supplementary material for: TMPRSS11B promotes an acidified microenvironment and immune suppression in squamous lung cancer
Source: EMBO Rep. 2025 Nov 10;26(24):6346–79. doi: 10.1038/s44319-025-00631-1 (PMC12714794; doi:10.1038/s44319-025-00631-1)
Supplement: Supplementary file 10 — Source data Fig. 5 [file 44319_2025_631_MOESM10_ESM.zip › Figure 5/5C-D/GSEA_Broad Institute_M8_T11b-high LUSC vs LUAD/ZHANG_UTERUS_C12_MONOCYTE.html]

Details for gene set ZHANG\_UTERUS\_C12\_MONOCYTE[GSEA]

|  || Dataset | Ranked list\_DGE\_squamousT11b\_vs\_all adenosadeno\_HSE13-NT copy |
| Phenotype | NoPhenotypeAvailable |
| Upregulated in class | na\_pos |
| GeneSet | ZHANG\_UTERUS\_C12\_MONOCYTE |
| Enrichment Score (ES) | 0.7308463 |
| Normalized Enrichment Score (NES) | 3.6548057 |
| Nominal p-value | 0.0 |
| FDR q-value | 0.0 |
| FWER p-Value | 0.0 |
Table: GSEA Results Summary

  

Fig 1: Enrichment plot: ZHANG\_UTERUS\_C12\_MONOCYTE      
 Profile of the Running ES Score & Positions of GeneSet Members on the Rank Ordered List

  

| SYMBOL | RANK IN GENE LIST | RANK METRIC SCORE | RUNNING ES | CORE ENRICHMENT || 1 | Spp1 | 69 | 4.139 | 0.0143 | Yes |
| 2 | Ccl9 | 82 | 3.944 | 0.0393 | Yes |
| 3 | Hmox1 | 144 | 3.102 | 0.0481 | Yes |
| 4 | Itgb2 | 158 | 2.937 | 0.0659 | Yes |
| 5 | Cybb | 173 | 2.805 | 0.0825 | Yes |
| 6 | Csf2rb | 176 | 2.766 | 0.1014 | Yes |
| 7 | Cd68 | 177 | 2.764 | 0.1207 | Yes |
| 8 | Tyrobp | 181 | 2.732 | 0.1392 | Yes |
| 9 | Srgn | 185 | 2.715 | 0.1575 | Yes |
| 10 | Ccl6 | 187 | 2.695 | 0.1761 | Yes |
| 11 | Il1b | 240 | 2.351 | 0.1816 | Yes |
| 12 | Wfdc17 | 241 | 2.337 | 0.1979 | Yes |
| 13 | Ctss | 247 | 2.317 | 0.2130 | Yes |
| 14 | Spi1 | 250 | 2.309 | 0.2287 | Yes |
| 15 | Plek | 252 | 2.303 | 0.2446 | Yes |
| 16 | Fcgr3 | 260 | 2.281 | 0.2591 | Yes |
| 17 | Cd53 | 261 | 2.271 | 0.2749 | Yes |
| 18 | Fcer1g | 272 | 2.235 | 0.2884 | Yes |
| 19 | Dusp5 | 275 | 2.206 | 0.3034 | Yes |
| 20 | Fcgr2b | 280 | 2.178 | 0.3178 | Yes |
| 21 | Ctsb | 288 | 2.139 | 0.3313 | Yes |
| 22 | Fth1 | 289 | 2.129 | 0.3461 | Yes |
| 23 | Csf2ra | 299 | 2.108 | 0.3590 | Yes |
| 24 | Mafb | 303 | 2.104 | 0.3730 | Yes |
| 25 | C5ar1 | 317 | 2.019 | 0.3844 | Yes |
| 26 | Pim1 | 319 | 2.011 | 0.3982 | Yes |
| 27 | Cd44 | 329 | 1.976 | 0.4101 | Yes |
| 28 | Cd52 | 332 | 1.963 | 0.4234 | Yes |
| 29 | Ptprc | 338 | 1.926 | 0.4358 | Yes |
| 30 | Mpeg1 | 346 | 1.889 | 0.4476 | Yes |
| 31 | Rgs1 | 350 | 1.874 | 0.4600 | Yes |
| 32 | S100a4 | 353 | 1.867 | 0.4726 | Yes |
| 33 | Emilin2 | 354 | 1.867 | 0.4857 | Yes |
| 34 | Irf5 | 394 | 1.709 | 0.4894 | Yes |
| 35 | Cfp | 433 | 1.599 | 0.4925 | Yes |
| 36 | Lgals3 | 447 | 1.559 | 0.5006 | Yes |
| 37 | Lgmn | 458 | 1.534 | 0.5092 | Yes |
| 38 | Lat2 | 477 | 1.499 | 0.5159 | Yes |
| 39 | Fxyd5 | 489 | 1.476 | 0.5239 | Yes |
| 40 | Apoe | 490 | 1.475 | 0.5342 | Yes |
| 41 | Ctsz | 493 | 1.463 | 0.5440 | Yes |
| 42 | Alox5ap | 500 | 1.445 | 0.5528 | Yes |
| 43 | Tgfbi | 502 | 1.443 | 0.5627 | Yes |
| 44 | Plin2 | 507 | 1.431 | 0.5719 | Yes |
| 45 | Psap | 510 | 1.415 | 0.5813 | Yes |
| 46 | Rab8b | 532 | 1.369 | 0.5864 | Yes |
| 47 | Emp3 | 537 | 1.365 | 0.5951 | Yes |
| 48 | Slfn2 | 544 | 1.342 | 0.6032 | Yes |
| 49 | Coro1a | 546 | 1.340 | 0.6124 | Yes |
| 50 | Sdc3 | 550 | 1.335 | 0.6211 | Yes |
| 51 | Grn | 554 | 1.328 | 0.6297 | Yes |
| 52 | Esd | 579 | 1.253 | 0.6334 | Yes |
| 53 | Cstb | 587 | 1.229 | 0.6405 | Yes |
| 54 | Samhd1 | 599 | 1.201 | 0.6466 | Yes |
| 55 | Prdx5 | 601 | 1.198 | 0.6547 | Yes |
| 56 | Kctd12 | 613 | 1.183 | 0.6607 | Yes |
| 57 | Sat1 | 614 | 1.180 | 0.6689 | Yes |
| 58 | C1qb | 615 | 1.180 | 0.6772 | Yes |
| 59 | Irf7 | 617 | 1.174 | 0.6852 | Yes |
| 60 | Ifrd1 | 671 | 1.048 | 0.6813 | Yes |
| 61 | Arhgdib | 672 | 1.048 | 0.6886 | Yes |
| 62 | Csf1r | 747 | 0.942 | 0.6795 | Yes |
| 63 | Ctsc | 770 | 0.907 | 0.6812 | Yes |
| 64 | C1qc | 775 | 0.902 | 0.6867 | Yes |
| 65 | H2-Ab1 | 778 | 0.895 | 0.6925 | Yes |
| 66 | B2m | 794 | 0.876 | 0.6954 | Yes |
| 67 | Cd74 | 811 | 0.856 | 0.6980 | Yes |
| 68 | Npc2 | 822 | 0.841 | 0.7018 | Yes |
| 69 | Ninj1 | 833 | 0.831 | 0.7055 | Yes |
| 70 | Lcp1 | 849 | 0.813 | 0.7080 | Yes |
| 71 | Cdkn1a | 861 | 0.808 | 0.7113 | Yes |
| 72 | Fem1c | 878 | 0.790 | 0.7134 | Yes |
| 73 | Ftl1-ps1 | 879 | 0.787 | 0.7189 | Yes |
| 74 | Mcl1 | 969 | 0.689 | 0.7049 | Yes |
| 75 | Pkm | 970 | 0.686 | 0.7097 | Yes |
| 76 | H2-Eb1 | 976 | 0.681 | 0.7134 | Yes |
| 77 | Msrb1 | 982 | 0.669 | 0.7170 | Yes |
| 78 | Atox1 | 997 | 0.651 | 0.7186 | Yes |
| 79 | Ctsa | 1010 | 0.641 | 0.7205 | Yes |
| 80 | H2-D1 | 1021 | 0.632 | 0.7228 | Yes |
| 81 | Ddit3 | 1038 | 0.616 | 0.7238 | Yes |
| 82 | Slc3a2 | 1041 | 0.613 | 0.7276 | Yes |
| 83 | Litaf | 1060 | 0.599 | 0.7280 | Yes |
| 84 | Psmb8 | 1067 | 0.589 | 0.7308 | Yes |
| 85 | Sdcbp | 1122 | 0.536 | 0.7232 | No |
| 86 | Ptpn1 | 1126 | 0.534 | 0.7263 | No |
| 87 | Rilpl2 | 1127 | 0.533 | 0.7300 | No |
| 88 | Tmsb4x | 1258 | -0.514 | 0.7061 | No |
| 89 | Vps37b | 1274 | -0.516 | 0.7065 | No |
| 90 | Calm1 | 1839 | -0.607 | 0.5914 | No |
| 91 | Por | 1937 | -0.624 | 0.5752 | No |
| 92 | Ly6e | 2739 | -0.775 | 0.4111 | No |
| 93 | Laptm5 | 2847 | -0.801 | 0.3941 | No |
| 94 | Got1 | 3326 | -0.939 | 0.2994 | No |
| 95 | Thbs1 | 3344 | -0.945 | 0.3025 | No |
| 96 | Tgif1 | 4179 | -1.391 | 0.1357 | No |
Table: GSEA details [plain text format]

  

Fig 2: ZHANG\_UTERUS\_C12\_MONOCYTE: Random ES distribution      
 Gene set null distribution of ES for **ZHANG\_UTERUS\_C12\_MONOCYTE**

  
