# Supplementary material for: TMPRSS11B promotes an acidified microenvironment and immune suppression in squamous lung cancer
Source: EMBO Rep. 2025 Nov 10;26(24):6346–79. doi: 10.1038/s44319-025-00631-1 (PMC12714794; doi:10.1038/s44319-025-00631-1)
Supplement: Supplementary file 10 — Source data Fig. 5 [file 44319_2025_631_MOESM10_ESM.zip › Figure 5/5C-D/GSEA_Broad Institute_M8_T11b-high LUSC vs LUAD/ZHANG_UTERUS_C13_EPITHELIAL1_CELL.html]

Details for gene set ZHANG\_UTERUS\_C13\_EPITHELIAL1\_CELL[GSEA]

|  || Dataset | Ranked list\_DGE\_squamousT11b\_vs\_all adenosadeno\_HSE13-NT copy |
| Phenotype | NoPhenotypeAvailable |
| Upregulated in class | na\_neg |
| GeneSet | ZHANG\_UTERUS\_C13\_EPITHELIAL1\_CELL |
| Enrichment Score (ES) | -0.19160017 |
| Normalized Enrichment Score (NES) | -1.1380994 |
| Nominal p-value | 0.2392473 |
| FDR q-value | 1.0 |
| FWER p-Value | 1.0 |
Table: GSEA Results Summary

  

Fig 1: Enrichment plot: ZHANG\_UTERUS\_C13\_EPITHELIAL1\_CELL      
 Profile of the Running ES Score & Positions of GeneSet Members on the Rank Ordered List

  

| SYMBOL | RANK IN GENE LIST | RANK METRIC SCORE | RUNNING ES | CORE ENRICHMENT || 1 | Sprr2f | 24 | 6.058 | 0.0419 | No |
| 2 | Serpinb11 | 106 | 3.658 | 0.0531 | No |
| 3 | Plat | 188 | 2.690 | 0.0568 | No |
| 4 | Plet1 | 190 | 2.674 | 0.0774 | No |
| 5 | Ptn | 221 | 2.429 | 0.0898 | No |
| 6 | Ltf | 264 | 2.250 | 0.0984 | No |
| 7 | Lcn2 | 283 | 2.163 | 0.1113 | No |
| 8 | Mif | 323 | 1.991 | 0.1185 | No |
| 9 | Gsto1 | 397 | 1.701 | 0.1162 | No |
| 10 | Ifitm1 | 427 | 1.619 | 0.1226 | No |
| 11 | C3 | 474 | 1.502 | 0.1245 | No |
| 12 | Tgfbi | 502 | 1.443 | 0.1300 | No |
| 13 | Txn1 | 743 | 0.944 | 0.0864 | No |
| 14 | S100g | 796 | 0.873 | 0.0821 | No |
| 15 | Spc24 | 813 | 0.853 | 0.0854 | No |
| 16 | 1810037I17Rik | 857 | 0.810 | 0.0825 | No |
| 17 | Siva1 | 892 | 0.765 | 0.0812 | No |
| 18 | Dut | 1068 | 0.589 | 0.0487 | No |
| 19 | Snrpf | 1089 | 0.566 | 0.0488 | No |
| 20 | Stmn1 | 1124 | 0.536 | 0.0458 | No |
| 21 | Ndufb6 | 1150 | 0.512 | 0.0444 | No |
| 22 | Anpep | 1162 | 0.504 | 0.0460 | No |
| 23 | Anxa4 | 1225 | -0.509 | 0.0368 | No |
| 24 | Brk1 | 1247 | -0.512 | 0.0363 | No |
| 25 | Anapc13 | 1293 | -0.518 | 0.0308 | No |
| 26 | Pfdn2 | 1298 | -0.519 | 0.0340 | No |
| 27 | Tmem176a | 1324 | -0.522 | 0.0327 | No |
| 28 | Tmem176b | 1326 | -0.522 | 0.0366 | No |
| 29 | Hnrnpc | 1347 | -0.525 | 0.0364 | No |
| 30 | Mphosph8 | 1399 | -0.532 | 0.0297 | No |
| 31 | Tle5 | 1425 | -0.536 | 0.0285 | No |
| 32 | Ndufv2 | 1437 | -0.539 | 0.0304 | No |
| 33 | Dtymk | 1507 | -0.552 | 0.0200 | No |
| 34 | Mgst1 | 1519 | -0.554 | 0.0220 | No |
| 35 | Mt1 | 1658 | -0.576 | -0.0028 | No |
| 36 | Sf3b5 | 1659 | -0.576 | 0.0016 | No |
| 37 | Dpy30 | 1664 | -0.577 | 0.0053 | No |
| 38 | Cyb5r3 | 1675 | -0.579 | 0.0077 | No |
| 39 | Sftpd | 1715 | -0.584 | 0.0039 | No |
| 40 | Wfdc2 | 1725 | -0.587 | 0.0066 | No |
| 41 | Suclg1 | 1762 | -0.593 | 0.0035 | No |
| 42 | Tpt1 | 1787 | -0.598 | 0.0031 | No |
| 43 | Micos13 | 1804 | -0.601 | 0.0043 | No |
| 44 | Pdlim4 | 1851 | -0.610 | -0.0007 | No |
| 45 | Coa3 | 1871 | -0.614 | 0.0000 | No |
| 46 | Rad23a | 1946 | -0.626 | -0.0108 | No |
| 47 | Acadl | 2030 | -0.641 | -0.0235 | No |
| 48 | Alad | 2165 | -0.664 | -0.0467 | No |
| 49 | Rbbp7 | 2243 | -0.679 | -0.0578 | No |
| 50 | Ivns1abp | 2272 | -0.684 | -0.0584 | No |
| 51 | Ndufa7 | 2344 | -0.696 | -0.0681 | No |
| 52 | Hat1 | 2351 | -0.697 | -0.0640 | No |
| 53 | Polr2e | 2361 | -0.699 | -0.0605 | No |
| 54 | Cib1 | 2552 | -0.737 | -0.0951 | No |
| 55 | Krtcap2 | 2586 | -0.744 | -0.0963 | No |
| 56 | Stx18 | 2623 | -0.753 | -0.0981 | No |
| 57 | Polr2i | 2682 | -0.764 | -0.1045 | No |
| 58 | 2510002D24Rik | 2723 | -0.771 | -0.1070 | No |
| 59 | Bola1 | 2805 | -0.791 | -0.1180 | No |
| 60 | Ciao2a | 2807 | -0.791 | -0.1121 | No |
| 61 | Iah1 | 2825 | -0.796 | -0.1095 | No |
| 62 | Smim22 | 2845 | -0.801 | -0.1073 | No |
| 63 | Sfxn1 | 2864 | -0.806 | -0.1049 | No |
| 64 | Timm8b | 2968 | -0.831 | -0.1203 | No |
| 65 | Rpa3 | 2978 | -0.836 | -0.1157 | No |
| 66 | Gtf3c6 | 2983 | -0.837 | -0.1101 | No |
| 67 | Id1 | 3000 | -0.840 | -0.1070 | No |
| 68 | Yipf1 | 3121 | -0.877 | -0.1256 | No |
| 69 | Spint2 | 3189 | -0.895 | -0.1329 | No |
| 70 | Atp1a1 | 3251 | -0.916 | -0.1387 | No |
| 71 | Ndufb8 | 3274 | -0.922 | -0.1362 | No |
| 72 | Gstm5 | 3294 | -0.927 | -0.1331 | No |
| 73 | Fkbp4 | 3332 | -0.940 | -0.1336 | No |
| 74 | Slc1a5 | 3499 | -0.997 | -0.1611 | No |
| 75 | Nudt14 | 3503 | -0.998 | -0.1540 | No |
| 76 | Kctd14 | 3533 | -1.008 | -0.1523 | No |
| 77 | Aamdc | 3547 | -1.013 | -0.1472 | No |
| 78 | Bcam | 3563 | -1.018 | -0.1425 | No |
| 79 | Rnf128 | 3580 | -1.025 | -0.1380 | No |
| 80 | Cenpx | 3601 | -1.035 | -0.1342 | No |
| 81 | Mt2 | 3612 | -1.035 | -0.1283 | No |
| 82 | Srsf3 | 3744 | -1.100 | -0.1475 | No |
| 83 | Pigr | 3833 | -1.149 | -0.1573 | No |
| 84 | Dynll2 | 3904 | -1.185 | -0.1629 | No |
| 85 | Mid1ip1 | 3910 | -1.190 | -0.1548 | No |
| 86 | Adi1 | 3913 | -1.192 | -0.1459 | No |
| 87 | Dcxr | 3966 | -1.224 | -0.1475 | No |
| 88 | Gtf2a2 | 4156 | -1.374 | -0.1769 | No |
| 89 | Gstm1 | 4200 | -1.410 | -0.1751 | No |
| 90 | Cystm1 | 4215 | -1.424 | -0.1670 | No |
| 91 | Fermt1 | 4276 | -1.484 | -0.1683 | No |
| 92 | Tmem158 | 4387 | -1.624 | -0.1790 | Yes |
| 93 | Sox9 | 4412 | -1.664 | -0.1712 | Yes |
| 94 | Tspan8 | 4446 | -1.740 | -0.1647 | Yes |
| 95 | Gstm2 | 4466 | -1.761 | -0.1550 | Yes |
| 96 | Krt8 | 4476 | -1.778 | -0.1432 | Yes |
| 97 | Krt19 | 4510 | -1.837 | -0.1359 | Yes |
| 98 | Sult1d1 | 4523 | -1.850 | -0.1241 | Yes |
| 99 | Cd24a | 4541 | -1.891 | -0.1130 | Yes |
| 100 | Paics | 4553 | -1.923 | -0.1004 | Yes |
| 101 | Muc1 | 4556 | -1.935 | -0.0859 | Yes |
| 102 | Echdc2 | 4557 | -1.936 | -0.0708 | Yes |
| 103 | Cbx6 | 4569 | -1.964 | -0.0579 | Yes |
| 104 | Cited4 | 4587 | -2.016 | -0.0459 | Yes |
| 105 | Bcat1 | 4607 | -2.061 | -0.0339 | Yes |
| 106 | Cfi | 4653 | -2.192 | -0.0265 | Yes |
| 107 | Cxcl17 | 4691 | -2.359 | -0.0160 | Yes |
| 108 | Gstm7 | 4760 | -2.718 | -0.0093 | Yes |
| 109 | Clu | 4775 | -2.816 | 0.0096 | Yes |
Table: GSEA details [plain text format]

  

Fig 2: ZHANG\_UTERUS\_C13\_EPITHELIAL1\_CELL: Random ES distribution      
 Gene set null distribution of ES for **ZHANG\_UTERUS\_C13\_EPITHELIAL1\_CELL**

  
