# Supplementary material for: TMPRSS11B promotes an acidified microenvironment and immune suppression in squamous lung cancer
Source: EMBO Rep. 2025 Nov 10;26(24):6346–79. doi: 10.1038/s44319-025-00631-1 (PMC12714794; doi:10.1038/s44319-025-00631-1)
Supplement: Supplementary file 10 — Source data Fig. 5 [file 44319_2025_631_MOESM10_ESM.zip › Figure 5/5C-D/GSEA_Broad Institute_M8_T11b-high LUSC vs LUAD/ZHANG_UTERUS_C14_ENDOTHELIAL_MMRN1_HIGH_CELL.html]

Details for gene set ZHANG\_UTERUS\_C14\_ENDOTHELIAL\_MMRN1\_HIGH\_CELL[GSEA]

|  || Dataset | Ranked list\_DGE\_squamousT11b\_vs\_all adenosadeno\_HSE13-NT copy |
| Phenotype | NoPhenotypeAvailable |
| Upregulated in class | na\_neg |
| GeneSet | ZHANG\_UTERUS\_C14\_ENDOTHELIAL\_MMRN1\_HIGH\_CELL |
| Enrichment Score (ES) | -0.26001838 |
| Normalized Enrichment Score (NES) | -0.906963 |
| Nominal p-value | 0.5795678 |
| FDR q-value | 1.0 |
| FWER p-Value | 1.0 |
Table: GSEA Results Summary

  

Fig 1: Enrichment plot: ZHANG\_UTERUS\_C14\_ENDOTHELIAL\_MMRN1\_HIGH\_CELL      
 Profile of the Running ES Score & Positions of GeneSet Members on the Rank Ordered List

  

| SYMBOL | RANK IN GENE LIST | RANK METRIC SCORE | RUNNING ES | CORE ENRICHMENT || 1 | Gngt2 | 273 | 2.235 | 0.0537 | Yes |
| 2 | Fth1 | 289 | 2.129 | 0.1558 | Yes |
| 3 | Jup | 565 | 1.293 | 0.1625 | Yes |
| 4 | Cldn5 | 902 | 0.758 | 0.1300 | Yes |
| 5 | Nrp2 | 1001 | 0.649 | 0.1417 | Yes |
| 6 | Fgl2 | 1019 | 0.634 | 0.1695 | Yes |
| 7 | Rab11a | 1095 | 0.560 | 0.1816 | Yes |
| 8 | Gng11 | 1167 | 0.502 | 0.1916 | Yes |
| 9 | Timp3 | 1480 | -0.548 | 0.1538 | No |
| 10 | Cyb5r3 | 1675 | -0.579 | 0.1420 | No |
| 11 | Aplp2 | 3500 | -0.997 | -0.1884 | No |
| 12 | Fxyd6 | 3769 | -1.109 | -0.1893 | No |
| 13 | Serpine2 | 3936 | -1.205 | -0.1643 | No |
| 14 | Selenop | 4397 | -1.636 | -0.1791 | No |
| 15 | Tm4sf1 | 4421 | -1.676 | -0.1010 | No |
| 16 | Txnip | 4471 | -1.771 | -0.0237 | No |
| 17 | Cavin2 | 4560 | -1.944 | 0.0541 | No |
Table: GSEA details [plain text format]

  

Fig 2: ZHANG\_UTERUS\_C14\_ENDOTHELIAL\_MMRN1\_HIGH\_CELL: Random ES distribution      
 Gene set null distribution of ES for **ZHANG\_UTERUS\_C14\_ENDOTHELIAL\_MMRN1\_HIGH\_CELL**

  
