# Supplementary material for: TMPRSS11B promotes an acidified microenvironment and immune suppression in squamous lung cancer
Source: EMBO Rep. 2025 Nov 10;26(24):6346–79. doi: 10.1038/s44319-025-00631-1 (PMC12714794; doi:10.1038/s44319-025-00631-1)
Supplement: Supplementary file 10 — Source data Fig. 5 [file 44319_2025_631_MOESM10_ESM.zip › Figure 5/5C-D/GSEA_Broad Institute_M8_T11b-high LUSC vs LUAD/ZHANG_UTERUS_C2_REGENERATIVE_UP.html]

Details for gene set ZHANG\_UTERUS\_C2\_REGENERATIVE\_UP[GSEA]

|  || Dataset | Ranked list\_DGE\_squamousT11b\_vs\_all adenosadeno\_HSE13-NT copy |
| Phenotype | NoPhenotypeAvailable |
| Upregulated in class | na\_neg |
| GeneSet | ZHANG\_UTERUS\_C2\_REGENERATIVE\_UP |
| Enrichment Score (ES) | -0.22215362 |
| Normalized Enrichment Score (NES) | -0.983163 |
| Nominal p-value | 0.46848738 |
| FDR q-value | 1.0 |
| FWER p-Value | 1.0 |
Table: GSEA Results Summary

  

Fig 1: Enrichment plot: ZHANG\_UTERUS\_C2\_REGENERATIVE\_UP      
 Profile of the Running ES Score & Positions of GeneSet Members on the Rank Ordered List

  

| SYMBOL | RANK IN GENE LIST | RANK METRIC SCORE | RUNNING ES | CORE ENRICHMENT || 1 | Serpinb11 | 106 | 3.658 | 0.0696 | No |
| 2 | Ltf | 264 | 2.250 | 0.0932 | No |
| 3 | Mif | 323 | 1.991 | 0.1310 | No |
| 4 | Ifitm1 | 427 | 1.619 | 0.1501 | No |
| 5 | Tgfbi | 502 | 1.443 | 0.1708 | No |
| 6 | S100g | 796 | 0.873 | 0.1315 | No |
| 7 | Siva1 | 892 | 0.765 | 0.1309 | No |
| 8 | Dut | 1068 | 0.589 | 0.1091 | No |
| 9 | Rbp1 | 1091 | 0.565 | 0.1187 | No |
| 10 | Stmn1 | 1124 | 0.536 | 0.1254 | No |
| 11 | Tmem176a | 1324 | -0.522 | 0.0969 | No |
| 12 | Tmem176b | 1326 | -0.522 | 0.1098 | No |
| 13 | Mgst1 | 1519 | -0.554 | 0.0836 | No |
| 14 | Mt1 | 1658 | -0.576 | 0.0692 | No |
| 15 | Tpt1 | 1787 | -0.598 | 0.0575 | No |
| 16 | Ivns1abp | 2272 | -0.684 | -0.0264 | No |
| 17 | Gas6 | 2372 | -0.701 | -0.0295 | No |
| 18 | Krtcap2 | 2586 | -0.744 | -0.0554 | No |
| 19 | Stx18 | 2623 | -0.753 | -0.0440 | No |
| 20 | Iah1 | 2825 | -0.796 | -0.0660 | No |
| 21 | Gstm5 | 3294 | -0.927 | -0.1405 | No |
| 22 | Kctd14 | 3533 | -1.008 | -0.1649 | No |
| 23 | Mt2 | 3612 | -1.035 | -0.1552 | No |
| 24 | Id3 | 3653 | -1.055 | -0.1371 | No |
| 25 | Pigr | 3833 | -1.149 | -0.1457 | No |
| 26 | Gstm1 | 4200 | -1.410 | -0.1868 | Yes |
| 27 | Mgp | 4278 | -1.484 | -0.1657 | Yes |
| 28 | Gstm2 | 4466 | -1.761 | -0.1606 | Yes |
| 29 | Sult1d1 | 4523 | -1.850 | -0.1259 | Yes |
| 30 | Echdc2 | 4557 | -1.936 | -0.0842 | Yes |
| 31 | Gstm7 | 4760 | -2.718 | -0.0583 | Yes |
| 32 | Clu | 4775 | -2.816 | 0.0094 | Yes |
Table: GSEA details [plain text format]

  

Fig 2: ZHANG\_UTERUS\_C2\_REGENERATIVE\_UP: Random ES distribution      
 Gene set null distribution of ES for **ZHANG\_UTERUS\_C2\_REGENERATIVE\_UP**

  
