# Supplementary material for: TMPRSS11B promotes an acidified microenvironment and immune suppression in squamous lung cancer
Source: EMBO Rep. 2025 Nov 10;26(24):6346–79. doi: 10.1038/s44319-025-00631-1 (PMC12714794; doi:10.1038/s44319-025-00631-1)
Supplement: Supplementary file 10 — Source data Fig. 5 [file 44319_2025_631_MOESM10_ESM.zip › Figure 5/5C-D/GSEA_Broad Institute_M8_T11b-high LUSC vs LUAD/ZHANG_UTERUS_C5_MACROPHAGE.html]

Details for gene set ZHANG\_UTERUS\_C5\_MACROPHAGE[GSEA]

|  || Dataset | Ranked list\_DGE\_squamousT11b\_vs\_all adenosadeno\_HSE13-NT copy |
| Phenotype | NoPhenotypeAvailable |
| Upregulated in class | na\_pos |
| GeneSet | ZHANG\_UTERUS\_C5\_MACROPHAGE |
| Enrichment Score (ES) | 0.6977248 |
| Normalized Enrichment Score (NES) | 3.4042451 |
| Nominal p-value | 0.0 |
| FDR q-value | 0.0 |
| FWER p-Value | 0.0 |
Table: GSEA Results Summary

  

Fig 1: Enrichment plot: ZHANG\_UTERUS\_C5\_MACROPHAGE      
 Profile of the Running ES Score & Positions of GeneSet Members on the Rank Ordered List

  

| SYMBOL | RANK IN GENE LIST | RANK METRIC SCORE | RUNNING ES | CORE ENRICHMENT || 1 | Ccl9 | 82 | 3.944 | 0.0132 | Yes |
| 2 | Trem2 | 140 | 3.156 | 0.0256 | Yes |
| 3 | Hmox1 | 144 | 3.102 | 0.0489 | Yes |
| 4 | Lpl | 149 | 3.044 | 0.0716 | Yes |
| 5 | Cybb | 173 | 2.805 | 0.0885 | Yes |
| 6 | Cd68 | 177 | 2.764 | 0.1092 | Yes |
| 7 | Tyrobp | 181 | 2.732 | 0.1297 | Yes |
| 8 | Ccl6 | 187 | 2.695 | 0.1495 | Yes |
| 9 | C3ar1 | 205 | 2.531 | 0.1655 | Yes |
| 10 | Il1b | 240 | 2.351 | 0.1765 | Yes |
| 11 | Wfdc17 | 241 | 2.337 | 0.1945 | Yes |
| 12 | Ctss | 247 | 2.317 | 0.2114 | Yes |
| 13 | Spi1 | 250 | 2.309 | 0.2288 | Yes |
| 14 | Plek | 252 | 2.303 | 0.2464 | Yes |
| 15 | Fcgr3 | 260 | 2.281 | 0.2626 | Yes |
| 16 | Cd53 | 261 | 2.271 | 0.2802 | Yes |
| 17 | Sirpa | 268 | 2.242 | 0.2962 | Yes |
| 18 | Fcer1g | 272 | 2.235 | 0.3129 | Yes |
| 19 | Ctsd | 274 | 2.219 | 0.3299 | Yes |
| 20 | Fcgr2b | 280 | 2.178 | 0.3456 | Yes |
| 21 | Il1rn | 287 | 2.146 | 0.3610 | Yes |
| 22 | Ctsb | 288 | 2.139 | 0.3775 | Yes |
| 23 | Fth1 | 289 | 2.129 | 0.3940 | Yes |
| 24 | Mafb | 303 | 2.104 | 0.4075 | Yes |
| 25 | Cd52 | 332 | 1.963 | 0.4168 | Yes |
| 26 | Mpeg1 | 346 | 1.889 | 0.4287 | Yes |
| 27 | Bcl2a1b | 349 | 1.876 | 0.4427 | Yes |
| 28 | Rgs1 | 350 | 1.874 | 0.4572 | Yes |
| 29 | Hexb | 384 | 1.742 | 0.4637 | Yes |
| 30 | Cfp | 433 | 1.599 | 0.4660 | Yes |
| 31 | Lgals3 | 447 | 1.559 | 0.4753 | Yes |
| 32 | Lgmn | 458 | 1.534 | 0.4850 | Yes |
| 33 | Mrc1 | 471 | 1.505 | 0.4941 | Yes |
| 34 | Lipa | 473 | 1.504 | 0.5056 | Yes |
| 35 | Apoe | 490 | 1.475 | 0.5136 | Yes |
| 36 | Ctsz | 493 | 1.463 | 0.5245 | Yes |
| 37 | Alox5ap | 500 | 1.445 | 0.5344 | Yes |
| 38 | Plin2 | 507 | 1.431 | 0.5442 | Yes |
| 39 | Psap | 510 | 1.415 | 0.5547 | Yes |
| 40 | Pltp | 523 | 1.391 | 0.5629 | Yes |
| 41 | Creg1 | 533 | 1.369 | 0.5716 | Yes |
| 42 | Coro1a | 546 | 1.340 | 0.5795 | Yes |
| 43 | Sdc3 | 550 | 1.335 | 0.5892 | Yes |
| 44 | Grn | 554 | 1.328 | 0.5988 | Yes |
| 45 | Cstb | 587 | 1.229 | 0.6015 | Yes |
| 46 | Ier5 | 609 | 1.186 | 0.6063 | Yes |
| 47 | Kctd12 | 613 | 1.183 | 0.6148 | Yes |
| 48 | Sat1 | 614 | 1.180 | 0.6239 | Yes |
| 49 | C1qb | 615 | 1.180 | 0.6331 | Yes |
| 50 | Atf3 | 643 | 1.106 | 0.6359 | Yes |
| 51 | Rgs10 | 660 | 1.068 | 0.6408 | Yes |
| 52 | Trf | 682 | 1.033 | 0.6443 | Yes |
| 53 | C1qa | 710 | 0.990 | 0.6463 | Yes |
| 54 | Blvrb | 731 | 0.962 | 0.6495 | Yes |
| 55 | Csf1r | 747 | 0.942 | 0.6536 | Yes |
| 56 | Ctsc | 770 | 0.907 | 0.6560 | Yes |
| 57 | C1qc | 775 | 0.902 | 0.6621 | Yes |
| 58 | H2-Ab1 | 778 | 0.895 | 0.6686 | Yes |
| 59 | B2m | 794 | 0.876 | 0.6722 | Yes |
| 60 | Cd74 | 811 | 0.856 | 0.6755 | Yes |
| 61 | Npc2 | 822 | 0.841 | 0.6799 | Yes |
| 62 | Ninj1 | 833 | 0.831 | 0.6842 | Yes |
| 63 | Lcp1 | 849 | 0.813 | 0.6873 | Yes |
| 64 | Cxcl16 | 855 | 0.811 | 0.6925 | Yes |
| 65 | Cdkn1a | 861 | 0.808 | 0.6977 | Yes |
| 66 | Ier3 | 967 | 0.691 | 0.6809 | No |
| 67 | Mcl1 | 969 | 0.689 | 0.6860 | No |
| 68 | H2-Eb1 | 976 | 0.681 | 0.6900 | No |
| 69 | Ctsa | 1010 | 0.641 | 0.6880 | No |
| 70 | H2-D1 | 1021 | 0.632 | 0.6908 | No |
| 71 | Dab2 | 1031 | 0.623 | 0.6937 | No |
| 72 | Ehd4 | 1053 | 0.607 | 0.6939 | No |
| 73 | Psmb8 | 1067 | 0.589 | 0.6958 | No |
| 74 | Sdcbp | 1122 | 0.536 | 0.6885 | No |
| 75 | Tmsb4x | 1258 | -0.514 | 0.6640 | No |
| 76 | Tmem176a | 1324 | -0.522 | 0.6543 | No |
| 77 | Tmem176b | 1326 | -0.522 | 0.6581 | No |
| 78 | Calm1 | 1839 | -0.607 | 0.5547 | No |
| 79 | Snx2 | 2321 | -0.693 | 0.4584 | No |
| 80 | Nfkbiz | 2566 | -0.740 | 0.4126 | No |
| 81 | Ly6e | 2739 | -0.775 | 0.3823 | No |
| 82 | Laptm5 | 2847 | -0.801 | 0.3659 | No |
| 83 | Asah1 | 3425 | -0.972 | 0.2516 | No |
| 84 | Lgals3bp | 4189 | -1.402 | 0.1013 | No |
| 85 | Selenop | 4397 | -1.636 | 0.0702 | No |
| 86 | Tlr2 | 4716 | -2.445 | 0.0220 | No |
Table: GSEA details [plain text format]

  

Fig 2: ZHANG\_UTERUS\_C5\_MACROPHAGE: Random ES distribution      
 Gene set null distribution of ES for **ZHANG\_UTERUS\_C5\_MACROPHAGE**

  
