# Supplementary material for: TMPRSS11B promotes an acidified microenvironment and immune suppression in squamous lung cancer
Source: EMBO Rep. 2025 Nov 10;26(24):6346–79. doi: 10.1038/s44319-025-00631-1 (PMC12714794; doi:10.1038/s44319-025-00631-1)
Supplement: Supplementary file 10 — Source data Fig. 5 [file 44319_2025_631_MOESM10_ESM.zip › Figure 5/5C-D/GSEA_Broad Institute_M8_T11b-high LUSC vs LUAD/ZHANG_UTERUS_C9_DENDRITIC_CELL.html]

Details for gene set ZHANG\_UTERUS\_C9\_DENDRITIC\_CELL[GSEA]

|  || Dataset | Ranked list\_DGE\_squamousT11b\_vs\_all adenosadeno\_HSE13-NT copy |
| Phenotype | NoPhenotypeAvailable |
| Upregulated in class | na\_pos |
| GeneSet | ZHANG\_UTERUS\_C9\_DENDRITIC\_CELL |
| Enrichment Score (ES) | 0.7473314 |
| Normalized Enrichment Score (NES) | 3.1662974 |
| Nominal p-value | 0.0 |
| FDR q-value | 0.0 |
| FWER p-Value | 0.0 |
Table: GSEA Results Summary

  

Fig 1: Enrichment plot: ZHANG\_UTERUS\_C9\_DENDRITIC\_CELL      
 Profile of the Running ES Score & Positions of GeneSet Members on the Rank Ordered List

  

| SYMBOL | RANK IN GENE LIST | RANK METRIC SCORE | RUNNING ES | CORE ENRICHMENT || 1 | Il1r2 | 32 | 5.303 | 0.0739 | Yes |
| 2 | Tyrobp | 181 | 2.732 | 0.0845 | Yes |
| 3 | Srgn | 185 | 2.715 | 0.1252 | Yes |
| 4 | Ccl6 | 187 | 2.695 | 0.1659 | Yes |
| 5 | Il1b | 240 | 2.351 | 0.1908 | Yes |
| 6 | Wfdc17 | 241 | 2.337 | 0.2263 | Yes |
| 7 | Ctss | 247 | 2.317 | 0.2605 | Yes |
| 8 | Spi1 | 250 | 2.309 | 0.2952 | Yes |
| 9 | Plek | 252 | 2.303 | 0.3300 | Yes |
| 10 | Cd53 | 261 | 2.271 | 0.3629 | Yes |
| 11 | Fcer1g | 272 | 2.235 | 0.3948 | Yes |
| 12 | Fth1 | 289 | 2.129 | 0.4238 | Yes |
| 13 | Pim1 | 319 | 2.011 | 0.4483 | Yes |
| 14 | Cd52 | 332 | 1.963 | 0.4757 | Yes |
| 15 | Mpeg1 | 346 | 1.889 | 0.5017 | Yes |
| 16 | Bcl2a1b | 349 | 1.876 | 0.5298 | Yes |
| 17 | Rgs1 | 350 | 1.874 | 0.5583 | Yes |
| 18 | Lgals3 | 447 | 1.559 | 0.5619 | Yes |
| 19 | Lgmn | 458 | 1.534 | 0.5831 | Yes |
| 20 | Fxyd5 | 489 | 1.476 | 0.5993 | Yes |
| 21 | Ctsz | 493 | 1.463 | 0.6209 | Yes |
| 22 | Alox5ap | 500 | 1.445 | 0.6416 | Yes |
| 23 | Psap | 510 | 1.415 | 0.6612 | Yes |
| 24 | Coro1a | 546 | 1.340 | 0.6743 | Yes |
| 25 | Arhgdib | 672 | 1.048 | 0.6641 | Yes |
| 26 | Ctsc | 770 | 0.907 | 0.6575 | Yes |
| 27 | H2-Ab1 | 778 | 0.895 | 0.6697 | Yes |
| 28 | Gm2a | 789 | 0.881 | 0.6810 | Yes |
| 29 | B2m | 794 | 0.876 | 0.6935 | Yes |
| 30 | Cd74 | 811 | 0.856 | 0.7031 | Yes |
| 31 | Lcp1 | 849 | 0.813 | 0.7078 | Yes |
| 32 | Cxcl16 | 855 | 0.811 | 0.7191 | Yes |
| 33 | Cdkn1a | 861 | 0.808 | 0.7303 | Yes |
| 34 | Mcl1 | 969 | 0.689 | 0.7184 | Yes |
| 35 | H2-Eb1 | 976 | 0.681 | 0.7275 | Yes |
| 36 | Atox1 | 997 | 0.651 | 0.7332 | Yes |
| 37 | H2-D1 | 1021 | 0.632 | 0.7380 | Yes |
| 38 | Lsp1 | 1023 | 0.629 | 0.7473 | Yes |
| 39 | Psmb8 | 1067 | 0.589 | 0.7473 | No |
| 40 | Rilpl2 | 1127 | 0.533 | 0.7431 | No |
| 41 | Tmsb4x | 1258 | -0.514 | 0.7237 | No |
| 42 | Calm1 | 1839 | -0.607 | 0.6115 | No |
| 43 | Laptm5 | 2847 | -0.801 | 0.4129 | No |
Table: GSEA details [plain text format]

  

Fig 2: ZHANG\_UTERUS\_C9\_DENDRITIC\_CELL: Random ES distribution      
 Gene set null distribution of ES for **ZHANG\_UTERUS\_C9\_DENDRITIC\_CELL**

  
