# Supplementary material for: TMPRSS11B promotes an acidified microenvironment and immune suppression in squamous lung cancer
Source: EMBO Rep. 2025 Nov 10;26(24):6346–79. doi: 10.1038/s44319-025-00631-1 (PMC12714794; doi:10.1038/s44319-025-00631-1)
Supplement: Supplementary file 11 — Source data Fig. 6 [file 44319_2025_631_MOESM11_ESM.zip › Figure 6/6D-E/GSEA Broad Institute_low pH vs rest of the regions (high pH)/DESCARTES_ORGANOGENESIS_ENDOTHELIAL_CELLS.html]

Details for gene set DESCARTES\_ORGANOGENESIS\_ENDOTHELIAL\_CELLS[GSEA]

|  || Dataset | Lactate high vs low\_Ranked |
| Phenotype | NoPhenotypeAvailable |
| Upregulated in class | na\_pos |
| GeneSet | DESCARTES\_ORGANOGENESIS\_ENDOTHELIAL\_CELLS |
| Enrichment Score (ES) | 0.44752273 |
| Normalized Enrichment Score (NES) | 3.0885189 |
| Nominal p-value | 0.0 |
| FDR q-value | 0.0 |
| FWER p-Value | 0.0 |
Table: GSEA Results Summary

  

Fig 1: Enrichment plot: DESCARTES\_ORGANOGENESIS\_ENDOTHELIAL\_CELLS      
 Profile of the Running ES Score & Positions of GeneSet Members on the Rank Ordered List

  

| SYMBOL | RANK IN GENE LIST | RANK METRIC SCORE | RUNNING ES | CORE ENRICHMENT || 1 | Cav1 | 107 | 1.564 | -0.0221 | Yes |
| 2 | Cd93 | 186 | 1.398 | -0.0359 | Yes |
| 3 | Clec1a | 203 | 1.369 | -0.0287 | Yes |
| 4 | Ptprm | 223 | 1.346 | -0.0228 | Yes |
| 5 | Cldn5 | 227 | 1.338 | -0.0115 | Yes |
| 6 | Npr1 | 245 | 1.299 | -0.0053 | Yes |
| 7 | Esam | 253 | 1.285 | 0.0041 | Yes |
| 8 | Acer2 | 265 | 1.264 | 0.0120 | Yes |
| 9 | BC028528 | 279 | 1.246 | 0.0190 | Yes |
| 10 | Msn | 280 | 1.243 | 0.0305 | Yes |
| 11 | Col4a1 | 286 | 1.235 | 0.0402 | Yes |
| 12 | Lrrc8c | 287 | 1.235 | 0.0515 | Yes |
| 13 | Col15a1 | 289 | 1.234 | 0.0626 | Yes |
| 14 | Myct1 | 292 | 1.229 | 0.0732 | Yes |
| 15 | Rin3 | 309 | 1.214 | 0.0789 | Yes |
| 16 | Adam19 | 312 | 1.209 | 0.0894 | Yes |
| 17 | Gimap8 | 324 | 1.194 | 0.0967 | Yes |
| 18 | Col4a2 | 331 | 1.182 | 0.1055 | Yes |
| 19 | Ptprb | 337 | 1.179 | 0.1147 | Yes |
| 20 | Icam2 | 355 | 1.156 | 0.1195 | Yes |
| 21 | Itpkb | 366 | 1.145 | 0.1266 | Yes |
| 22 | Gja4 | 399 | 1.101 | 0.1259 | Yes |
| 23 | Gimap6 | 403 | 1.097 | 0.1349 | Yes |
| 24 | Egfl7 | 406 | 1.093 | 0.1443 | Yes |
| 25 | Klf2 | 412 | 1.087 | 0.1526 | Yes |
| 26 | Cyyr1 | 413 | 1.086 | 0.1627 | Yes |
| 27 | Rasip1 | 427 | 1.073 | 0.1681 | Yes |
| 28 | Plxnd1 | 430 | 1.069 | 0.1773 | Yes |
| 29 | Sox18 | 433 | 1.067 | 0.1864 | Yes |
| 30 | Slfn5 | 434 | 1.066 | 0.1963 | Yes |
| 31 | Arhgef15 | 439 | 1.057 | 0.2046 | Yes |
| 32 | She | 444 | 1.051 | 0.2129 | Yes |
| 33 | Sox17 | 450 | 1.047 | 0.2209 | Yes |
| 34 | Cdh5 | 455 | 1.041 | 0.2291 | Yes |
| 35 | Sparc | 460 | 1.038 | 0.2373 | Yes |
| 36 | Acvrl1 | 462 | 1.038 | 0.2465 | Yes |
| 37 | Clec14a | 471 | 1.031 | 0.2533 | Yes |
| 38 | Mmrn2 | 473 | 1.025 | 0.2624 | Yes |
| 39 | Adgre5 | 493 | 0.998 | 0.2651 | Yes |
| 40 | Erg | 500 | 0.992 | 0.2722 | Yes |
| 41 | Tie1 | 504 | 0.985 | 0.2803 | Yes |
| 42 | Plvap | 512 | 0.977 | 0.2869 | Yes |
| 43 | Sema3g | 514 | 0.977 | 0.2956 | Yes |
| 44 | Kank3 | 523 | 0.968 | 0.3018 | Yes |
| 45 | Jcad | 535 | 0.960 | 0.3069 | Yes |
| 46 | Ushbp1 | 545 | 0.954 | 0.3126 | Yes |
| 47 | Vash1 | 556 | 0.946 | 0.3179 | Yes |
| 48 | Sh2d3c | 564 | 0.940 | 0.3242 | Yes |
| 49 | Fgd5 | 584 | 0.913 | 0.3261 | Yes |
| 50 | Ccm2l | 593 | 0.901 | 0.3317 | Yes |
| 51 | Ehd2 | 597 | 0.893 | 0.3389 | Yes |
| 52 | Afap1l1 | 605 | 0.888 | 0.3446 | Yes |
| 53 | Gimap1 | 614 | 0.876 | 0.3500 | Yes |
| 54 | S1pr1 | 618 | 0.873 | 0.3570 | Yes |
| 55 | Flt1 | 628 | 0.869 | 0.3619 | Yes |
| 56 | Calcrl | 631 | 0.867 | 0.3693 | Yes |
| 57 | Dysf | 632 | 0.865 | 0.3772 | Yes |
| 58 | Pecam1 | 658 | 0.845 | 0.3765 | Yes |
| 59 | Ramp2 | 670 | 0.833 | 0.3804 | Yes |
| 60 | Ets1 | 671 | 0.831 | 0.3881 | Yes |
| 61 | Epas1 | 680 | 0.827 | 0.3930 | Yes |
| 62 | Snrk | 687 | 0.821 | 0.3985 | Yes |
| 63 | Adgrl4 | 692 | 0.817 | 0.4046 | Yes |
| 64 | Tmem88 | 712 | 0.801 | 0.4055 | Yes |
| 65 | Eng | 733 | 0.776 | 0.4059 | Yes |
| 66 | Kdr | 748 | 0.765 | 0.4081 | Yes |
| 67 | Osmr | 751 | 0.762 | 0.4145 | Yes |
| 68 | Notch4 | 766 | 0.746 | 0.4166 | Yes |
| 69 | Shank3 | 768 | 0.742 | 0.4230 | Yes |
| 70 | Grap | 780 | 0.725 | 0.4260 | Yes |
| 71 | Fmnl3 | 792 | 0.713 | 0.4288 | Yes |
| 72 | Adcy4 | 822 | 0.688 | 0.4252 | Yes |
| 73 | Pced1b | 826 | 0.685 | 0.4305 | Yes |
| 74 | Cd34 | 835 | 0.679 | 0.4340 | Yes |
| 75 | Arhgap29 | 840 | 0.678 | 0.4389 | Yes |
| 76 | Prcp | 842 | 0.676 | 0.4448 | Yes |
| 77 | Sh3bp5 | 881 | 0.642 | 0.4377 | Yes |
| 78 | Prex2 | 882 | 0.642 | 0.4437 | Yes |
| 79 | Rapgef5 | 903 | 0.627 | 0.4426 | Yes |
| 80 | Pkn3 | 939 | 0.605 | 0.4362 | Yes |
| 81 | Arap3 | 941 | 0.604 | 0.4415 | Yes |
| 82 | Sox7 | 942 | 0.603 | 0.4470 | Yes |
| 83 | Ecscr | 970 | 0.587 | 0.4432 | Yes |
| 84 | Adam15 | 974 | 0.580 | 0.4475 | Yes |
| 85 | Pear1 | 1005 | 0.560 | 0.4424 | No |
| 86 | Hdac7 | 1031 | 0.548 | 0.4390 | No |
| 87 | Col18a1 | 1034 | 0.547 | 0.4433 | No |
| 88 | Rhoj | 1049 | 0.537 | 0.4435 | No |
| 89 | Adgrf5 | 1082 | 0.518 | 0.4373 | No |
| 90 | Plaur | 1098 | 0.504 | 0.4368 | No |
| 91 | Stap2 | 1127 | -0.505 | 0.4319 | No |
| 92 | Ece1 | 1216 | -0.525 | 0.4067 | No |
| 93 | Bcl2 | 1390 | -0.561 | 0.3528 | No |
| 94 | Asah2 | 1525 | -0.592 | 0.3125 | No |
| 95 | Nr4a1 | 1632 | -0.627 | 0.2821 | No |
| 96 | Cgnl1 | 1635 | -0.628 | 0.2872 | No |
| 97 | Mast4 | 1762 | -0.675 | 0.2504 | No |
| 98 | S100a16 | 1801 | -0.687 | 0.2437 | No |
| 99 | Hbegf | 1860 | -0.709 | 0.2304 | No |
| 100 | AU021092 | 1873 | -0.712 | 0.2329 | No |
| 101 | Pgm2 | 1899 | -0.721 | 0.2310 | No |
| 102 | Epha2 | 1997 | -0.758 | 0.2049 | No |
| 103 | Itpr3 | 1998 | -0.759 | 0.2119 | No |
| 104 | Flnb | 2221 | -0.871 | 0.1441 | No |
| 105 | Ppp1r13b | 2383 | -0.994 | 0.0983 | No |
| 106 | Bik | 2425 | -1.027 | 0.0937 | No |
| 107 | Tnfrsf11b | 2433 | -1.037 | 0.1009 | No |
| 108 | Myo6 | 2548 | -1.149 | 0.0725 | No |
| 109 | Galnt4 | 2722 | -1.412 | 0.0265 | No |
| 110 | Foxq1 | 2872 | -1.867 | -0.0072 | No |
| 111 | Kcne3 | 2945 | -2.310 | -0.0105 | No |
| 112 | Enpp3 | 3037 | -4.586 | 0.0007 | No |
Table: GSEA details [plain text format]

  

Fig 2: DESCARTES\_ORGANOGENESIS\_ENDOTHELIAL\_CELLS: Random ES distribution      
 Gene set null distribution of ES for **DESCARTES\_ORGANOGENESIS\_ENDOTHELIAL\_CELLS**

  
