# Supplementary material for: TMPRSS11B promotes an acidified microenvironment and immune suppression in squamous lung cancer
Source: EMBO Rep. 2025 Nov 10;26(24):6346–79. doi: 10.1038/s44319-025-00631-1 (PMC12714794; doi:10.1038/s44319-025-00631-1)
Supplement: Supplementary file 11 — Source data Fig. 6 [file 44319_2025_631_MOESM11_ESM.zip › Figure 6/6D-E/GSEA Broad Institute_low pH vs rest of the regions (high pH)/DESCARTES_ORGANOGENESIS_EPITHELIAL_CELLS.html]

Details for gene set DESCARTES\_ORGANOGENESIS\_EPITHELIAL\_CELLS[GSEA]

|  || Dataset | Lactate high vs low\_Ranked |
| Phenotype | NoPhenotypeAvailable |
| Upregulated in class | na\_neg |
| GeneSet | DESCARTES\_ORGANOGENESIS\_EPITHELIAL\_CELLS |
| Enrichment Score (ES) | -0.67664874 |
| Normalized Enrichment Score (NES) | -3.589764 |
| Nominal p-value | 0.0 |
| FDR q-value | 0.0 |
| FWER p-Value | 0.0 |
Table: GSEA Results Summary

  

Fig 1: Enrichment plot: DESCARTES\_ORGANOGENESIS\_EPITHELIAL\_CELLS      
 Profile of the Running ES Score & Positions of GeneSet Members on the Rank Ordered List

  

| SYMBOL | RANK IN GENE LIST | RANK METRIC SCORE | RUNNING ES | CORE ENRICHMENT || 1 | Susd4 | 74 | 1.644 | -0.0123 | No |
| 2 | Mfge8 | 118 | 1.535 | -0.0149 | No |
| 3 | Sema3e | 317 | 1.206 | -0.0725 | No |
| 4 | Scnn1b | 528 | 0.965 | -0.1360 | No |
| 5 | Adrb1 | 807 | 0.702 | -0.2245 | No |
| 6 | Galnt18 | 926 | 0.612 | -0.2596 | No |
| 7 | Vstm5 | 1285 | -0.538 | -0.3765 | No |
| 8 | Eps8l1 | 1307 | -0.542 | -0.3794 | No |
| 9 | Llgl2 | 1344 | -0.551 | -0.3873 | No |
| 10 | Cldn4 | 1430 | -0.572 | -0.4116 | No |
| 11 | Ovol2 | 1648 | -0.632 | -0.4800 | No |
| 12 | Patj | 1689 | -0.648 | -0.4885 | No |
| 13 | Lsr | 1792 | -0.685 | -0.5177 | No |
| 14 | Dgka | 1813 | -0.694 | -0.5191 | No |
| 15 | Prss8 | 1985 | -0.752 | -0.5710 | No |
| 16 | Sh3rf2 | 2048 | -0.784 | -0.5859 | No |
| 17 | Tacstd2 | 2079 | -0.799 | -0.5898 | No |
| 18 | Unc5cl | 2100 | -0.806 | -0.5904 | No |
| 19 | Kdf1 | 2184 | -0.851 | -0.6118 | No |
| 20 | Kcnk1 | 2305 | -0.929 | -0.6452 | No |
| 21 | Ap1m2 | 2386 | -0.995 | -0.6645 | No |
| 22 | Fam83b | 2423 | -1.025 | -0.6687 | Yes |
| 23 | Ripk4 | 2443 | -1.046 | -0.6670 | Yes |
| 24 | Itgb4 | 2445 | -1.047 | -0.6592 | Yes |
| 25 | Marveld3 | 2450 | -1.053 | -0.6524 | Yes |
| 26 | Spint1 | 2505 | -1.098 | -0.6621 | Yes |
| 27 | Tmem30b | 2520 | -1.116 | -0.6582 | Yes |
| 28 | Esrp2 | 2535 | -1.132 | -0.6541 | Yes |
| 29 | Spint2 | 2539 | -1.139 | -0.6463 | Yes |
| 30 | Tmprss2 | 2557 | -1.159 | -0.6431 | Yes |
| 31 | Osbpl3 | 2566 | -1.169 | -0.6367 | Yes |
| 32 | Pof1b | 2567 | -1.169 | -0.6276 | Yes |
| 33 | Cblc | 2582 | -1.186 | -0.6232 | Yes |
| 34 | St14 | 2628 | -1.245 | -0.6287 | Yes |
| 35 | Cldn7 | 2642 | -1.269 | -0.6232 | Yes |
| 36 | Grhl2 | 2652 | -1.288 | -0.6163 | Yes |
| 37 | Cdh1 | 2655 | -1.294 | -0.6069 | Yes |
| 38 | Irf6 | 2657 | -1.295 | -0.5972 | Yes |
| 39 | Lad1 | 2675 | -1.325 | -0.5927 | Yes |
| 40 | Cldn23 | 2686 | -1.345 | -0.5856 | Yes |
| 41 | Esrp1 | 2699 | -1.358 | -0.5791 | Yes |
| 42 | Cdcp1 | 2704 | -1.374 | -0.5698 | Yes |
| 43 | Pkp3 | 2706 | -1.377 | -0.5595 | Yes |
| 44 | Lypd6b | 2728 | -1.421 | -0.5555 | Yes |
| 45 | Nipal2 | 2731 | -1.438 | -0.5450 | Yes |
| 46 | Tmem45b | 2732 | -1.438 | -0.5339 | Yes |
| 47 | Frem2 | 2739 | -1.457 | -0.5246 | Yes |
| 48 | Epha1 | 2746 | -1.471 | -0.5152 | Yes |
| 49 | Krt7 | 2749 | -1.487 | -0.5043 | Yes |
| 50 | Plcd3 | 2758 | -1.508 | -0.4953 | Yes |
| 51 | Arhgef16 | 2780 | -1.546 | -0.4904 | Yes |
| 52 | Nectin4 | 2786 | -1.558 | -0.4800 | Yes |
| 53 | Fermt1 | 2795 | -1.579 | -0.4705 | Yes |
| 54 | Arhgef19 | 2804 | -1.597 | -0.4608 | Yes |
| 55 | Ikzf2 | 2807 | -1.599 | -0.4491 | Yes |
| 56 | Epcam | 2813 | -1.616 | -0.4382 | Yes |
| 57 | Lnx1 | 2818 | -1.629 | -0.4269 | Yes |
| 58 | Klf5 | 2823 | -1.644 | -0.4155 | Yes |
| 59 | Exph5 | 2840 | -1.715 | -0.4076 | Yes |
| 60 | Galnt3 | 2845 | -1.733 | -0.3955 | Yes |
| 61 | Adam28 | 2848 | -1.739 | -0.3827 | Yes |
| 62 | Ppl | 2852 | -1.754 | -0.3701 | Yes |
| 63 | Wfdc2 | 2861 | -1.816 | -0.3587 | Yes |
| 64 | Wnt4 | 2873 | -1.869 | -0.3479 | Yes |
| 65 | Ttll10 | 2877 | -1.875 | -0.3344 | Yes |
| 66 | Cers3 | 2891 | -1.961 | -0.3235 | Yes |
| 67 | Ildr1 | 2900 | -2.016 | -0.3106 | Yes |
| 68 | Crybg1 | 2901 | -2.026 | -0.2949 | Yes |
| 69 | Vtcn1 | 2908 | -2.071 | -0.2808 | Yes |
| 70 | Krt15 | 2935 | -2.256 | -0.2721 | Yes |
| 71 | Paqr5 | 2944 | -2.303 | -0.2569 | Yes |
| 72 | Plch2 | 2966 | -2.523 | -0.2444 | Yes |
| 73 | Atp2c2 | 2980 | -2.830 | -0.2269 | Yes |
| 74 | Krt14 | 2985 | -2.898 | -0.2057 | Yes |
| 75 | Gpr87 | 2992 | -3.022 | -0.1843 | Yes |
| 76 | Pkp1 | 2996 | -3.083 | -0.1614 | Yes |
| 77 | Gabrp | 2998 | -3.126 | -0.1375 | Yes |
| 78 | Krt5 | 3013 | -3.435 | -0.1155 | Yes |
| 79 | Gjb2 | 3016 | -3.482 | -0.0892 | Yes |
| 80 | Fat2 | 3018 | -3.571 | -0.0618 | Yes |
| 81 | Trp63 | 3034 | -4.244 | -0.0339 | Yes |
| 82 | Col17a1 | 3036 | -4.544 | 0.0010 | Yes |
Table: GSEA details [plain text format]

  

Fig 2: DESCARTES\_ORGANOGENESIS\_EPITHELIAL\_CELLS: Random ES distribution      
 Gene set null distribution of ES for **DESCARTES\_ORGANOGENESIS\_EPITHELIAL\_CELLS**

  
