# Supplementary material for: TMPRSS11B promotes an acidified microenvironment and immune suppression in squamous lung cancer
Source: EMBO Rep. 2025 Nov 10;26(24):6346–79. doi: 10.1038/s44319-025-00631-1 (PMC12714794; doi:10.1038/s44319-025-00631-1)
Supplement: Supplementary file 11 — Source data Fig. 6 [file 44319_2025_631_MOESM11_ESM.zip › Figure 6/6D-E/GSEA Broad Institute_low pH vs rest of the regions (high pH)/DESCARTES_ORGANOGENESIS_HEPATOCYTES.html]

Details for gene set DESCARTES\_ORGANOGENESIS\_HEPATOCYTES[GSEA]

|  || Dataset | Lactate high vs low\_Ranked |
| Phenotype | NoPhenotypeAvailable |
| Upregulated in class | na\_neg |
| GeneSet | DESCARTES\_ORGANOGENESIS\_HEPATOCYTES |
| Enrichment Score (ES) | -0.30653775 |
| Normalized Enrichment Score (NES) | -1.7393923 |
| Nominal p-value | 0.006002401 |
| FDR q-value | 0.0468591 |
| FWER p-Value | 0.685 |
Table: GSEA Results Summary

  

Fig 1: Enrichment plot: DESCARTES\_ORGANOGENESIS\_HEPATOCYTES      
 Profile of the Running ES Score & Positions of GeneSet Members on the Rank Ordered List

  

| SYMBOL | RANK IN GENE LIST | RANK METRIC SCORE | RUNNING ES | CORE ENRICHMENT || 1 | F7 | 10 | 2.132 | 0.0130 | No |
| 2 | Apoc1 | 40 | 1.811 | 0.0170 | No |
| 3 | Stra6l | 72 | 1.653 | 0.0191 | No |
| 4 | Lonrf3 | 91 | 1.607 | 0.0253 | No |
| 5 | Slc36a2 | 134 | 1.510 | 0.0225 | No |
| 6 | Mmp19 | 148 | 1.475 | 0.0294 | No |
| 7 | Qpct | 149 | 1.474 | 0.0408 | No |
| 8 | Tmem37 | 152 | 1.461 | 0.0513 | No |
| 9 | Pcolce2 | 184 | 1.402 | 0.0515 | No |
| 10 | Anpep | 212 | 1.361 | 0.0527 | No |
| 11 | Fgg | 221 | 1.352 | 0.0604 | No |
| 12 | Gdf15 | 231 | 1.329 | 0.0675 | No |
| 13 | Cd302 | 283 | 1.237 | 0.0596 | No |
| 14 | Adk | 356 | 1.156 | 0.0438 | No |
| 15 | Fn1 | 520 | 0.973 | -0.0046 | No |
| 16 | Trf | 541 | 0.957 | -0.0041 | No |
| 17 | Hc | 562 | 0.941 | -0.0037 | No |
| 18 | Phyhd1 | 620 | 0.872 | -0.0165 | No |
| 19 | Pygl | 633 | 0.865 | -0.0139 | No |
| 20 | Slc7a2 | 795 | 0.711 | -0.0636 | No |
| 21 | Fmo2 | 845 | 0.674 | -0.0752 | No |
| 22 | G0s2 | 878 | 0.645 | -0.0812 | No |
| 23 | Grb10 | 894 | 0.634 | -0.0815 | No |
| 24 | Emp2 | 915 | 0.618 | -0.0836 | No |
| 25 | Cys1 | 919 | 0.617 | -0.0799 | No |
| 26 | Fkbp5 | 935 | 0.606 | -0.0803 | No |
| 27 | C3 | 948 | 0.602 | -0.0798 | No |
| 28 | Ly75 | 1042 | 0.542 | -0.1075 | No |
| 29 | C1ra | 1069 | 0.527 | -0.1123 | No |
| 30 | Sfmbt1 | 1073 | 0.523 | -0.1094 | No |
| 31 | Grk3 | 1095 | 0.505 | -0.1127 | No |
| 32 | Pecr | 1110 | -0.502 | -0.1136 | No |
| 33 | Nipsnap2 | 1112 | -0.502 | -0.1101 | No |
| 34 | Osgin1 | 1138 | -0.506 | -0.1147 | No |
| 35 | Cpm | 1180 | -0.515 | -0.1248 | No |
| 36 | Gpt2 | 1185 | -0.515 | -0.1222 | No |
| 37 | Dop1b | 1271 | -0.536 | -0.1472 | No |
| 38 | Got2 | 1317 | -0.545 | -0.1585 | No |
| 39 | Slc25a10 | 1343 | -0.551 | -0.1628 | No |
| 40 | Acad11 | 1452 | -0.575 | -0.1953 | No |
| 41 | Aldh4a1 | 1475 | -0.580 | -0.1984 | No |
| 42 | Phyh | 1502 | -0.587 | -0.2028 | No |
| 43 | Ocln | 1510 | -0.589 | -0.2007 | No |
| 44 | Nt5e | 1516 | -0.591 | -0.1978 | No |
| 45 | Slc22a18 | 1519 | -0.591 | -0.1940 | No |
| 46 | Dap | 1535 | -0.595 | -0.1945 | No |
| 47 | Fabp5 | 1603 | -0.617 | -0.2127 | No |
| 48 | Usp18 | 1621 | -0.622 | -0.2138 | No |
| 49 | Chdh | 1650 | -0.633 | -0.2185 | No |
| 50 | Ephx2 | 1664 | -0.637 | -0.2181 | No |
| 51 | Tmem51 | 1721 | -0.662 | -0.2321 | No |
| 52 | Echdc2 | 1769 | -0.679 | -0.2430 | No |
| 53 | Als2cl | 1771 | -0.679 | -0.2381 | No |
| 54 | Tmem205 | 1833 | -0.702 | -0.2536 | No |
| 55 | Galm | 1846 | -0.705 | -0.2523 | No |
| 56 | Synj2 | 1849 | -0.707 | -0.2476 | No |
| 57 | Hadh | 1869 | -0.711 | -0.2486 | No |
| 58 | Siah2 | 1909 | -0.724 | -0.2564 | No |
| 59 | Slc25a15 | 1921 | -0.728 | -0.2545 | No |
| 60 | Akr1c13 | 1950 | -0.740 | -0.2584 | No |
| 61 | Zfp395 | 1977 | -0.750 | -0.2616 | No |
| 62 | Hip1r | 2058 | -0.789 | -0.2829 | No |
| 63 | Slc35d2 | 2060 | -0.789 | -0.2772 | No |
| 64 | Aqp11 | 2068 | -0.794 | -0.2735 | No |
| 65 | Bcas1 | 2102 | -0.806 | -0.2786 | No |
| 66 | Mettl26 | 2118 | -0.815 | -0.2774 | No |
| 67 | Atp8b1 | 2164 | -0.842 | -0.2864 | No |
| 68 | Abcc10 | 2187 | -0.853 | -0.2874 | No |
| 69 | Plekhg3 | 2244 | -0.887 | -0.2997 | Yes |
| 70 | Lss | 2249 | -0.890 | -0.2942 | Yes |
| 71 | Sh3bgrl2 | 2257 | -0.897 | -0.2897 | Yes |
| 72 | Grhpr | 2258 | -0.898 | -0.2828 | Yes |
| 73 | Hnf4a | 2271 | -0.910 | -0.2799 | Yes |
| 74 | Steap2 | 2281 | -0.915 | -0.2760 | Yes |
| 75 | Herpud1 | 2283 | -0.915 | -0.2693 | Yes |
| 76 | Stard10 | 2320 | -0.937 | -0.2744 | Yes |
| 77 | Kyat1 | 2321 | -0.937 | -0.2672 | Yes |
| 78 | Crot | 2333 | -0.945 | -0.2637 | Yes |
| 79 | Chmp4c | 2343 | -0.954 | -0.2594 | Yes |
| 80 | Sh3d19 | 2360 | -0.974 | -0.2574 | Yes |
| 81 | Gpd1 | 2364 | -0.978 | -0.2509 | Yes |
| 82 | 2310039H08Rik | 2388 | -0.997 | -0.2511 | Yes |
| 83 | Pgm3 | 2398 | -1.006 | -0.2465 | Yes |
| 84 | Faah | 2427 | -1.027 | -0.2481 | Yes |
| 85 | Cryz | 2436 | -1.039 | -0.2429 | Yes |
| 86 | Sdr42e1 | 2486 | -1.079 | -0.2514 | Yes |
| 87 | Baiap2l2 | 2487 | -1.081 | -0.2431 | Yes |
| 88 | Mgst2 | 2501 | -1.095 | -0.2391 | Yes |
| 89 | Rassf6 | 2502 | -1.096 | -0.2306 | Yes |
| 90 | Tfcp2l1 | 2528 | -1.125 | -0.2306 | Yes |
| 91 | Pex26 | 2545 | -1.145 | -0.2272 | Yes |
| 92 | Grb7 | 2550 | -1.150 | -0.2197 | Yes |
| 93 | Nadsyn1 | 2576 | -1.177 | -0.2193 | Yes |
| 94 | 2810459M11Rik | 2581 | -1.185 | -0.2115 | Yes |
| 95 | 0610040J01Rik | 2596 | -1.207 | -0.2070 | Yes |
| 96 | Pik3c2g | 2614 | -1.226 | -0.2034 | Yes |
| 97 | Mcrip2 | 2640 | -1.267 | -0.2022 | Yes |
| 98 | Hpn | 2653 | -1.291 | -0.1964 | Yes |
| 99 | Ngef | 2666 | -1.312 | -0.1904 | Yes |
| 100 | Slc39a4 | 2673 | -1.321 | -0.1823 | Yes |
| 101 | Acsl1 | 2687 | -1.346 | -0.1764 | Yes |
| 102 | Sec16b | 2691 | -1.349 | -0.1670 | Yes |
| 103 | Slc44a3 | 2725 | -1.415 | -0.1674 | Yes |
| 104 | Dgat2 | 2727 | -1.420 | -0.1569 | Yes |
| 105 | Rhpn2 | 2740 | -1.458 | -0.1498 | Yes |
| 106 | Ugt2b34 | 2756 | -1.498 | -0.1434 | Yes |
| 107 | Tst | 2769 | -1.524 | -0.1357 | Yes |
| 108 | Cfb | 2772 | -1.529 | -0.1247 | Yes |
| 109 | Psat1 | 2790 | -1.568 | -0.1184 | Yes |
| 110 | Sytl5 | 2817 | -1.626 | -0.1148 | Yes |
| 111 | Gpr39 | 2825 | -1.650 | -0.1045 | Yes |
| 112 | Krt20 | 2839 | -1.707 | -0.0958 | Yes |
| 113 | Pdia5 | 2847 | -1.737 | -0.0849 | Yes |
| 114 | Aspa | 2862 | -1.819 | -0.0757 | Yes |
| 115 | Shmt1 | 2871 | -1.860 | -0.0641 | Yes |
| 116 | Plekhg6 | 2882 | -1.907 | -0.0528 | Yes |
| 117 | Hgfac | 2903 | -2.055 | -0.0439 | Yes |
| 118 | Bdh1 | 2937 | -2.259 | -0.0378 | Yes |
| 119 | Acnat1 | 2975 | -2.693 | -0.0298 | Yes |
| 120 | Cfi | 2977 | -2.744 | -0.0090 | Yes |
| 121 | Itih2 | 3027 | -3.881 | 0.0041 | Yes |
Table: GSEA details [plain text format]

  

Fig 2: DESCARTES\_ORGANOGENESIS\_HEPATOCYTES: Random ES distribution      
 Gene set null distribution of ES for **DESCARTES\_ORGANOGENESIS\_HEPATOCYTES**

  
