# Supplementary material for: TMPRSS11B promotes an acidified microenvironment and immune suppression in squamous lung cancer
Source: EMBO Rep. 2025 Nov 10;26(24):6346–79. doi: 10.1038/s44319-025-00631-1 (PMC12714794; doi:10.1038/s44319-025-00631-1)
Supplement: Supplementary file 11 — Source data Fig. 6 [file 44319_2025_631_MOESM11_ESM.zip › Figure 6/6D-E/GSEA Broad Institute_low pH vs rest of the regions (high pH)/DESCARTES_ORGANOGENESIS_WHITE_BLOOD_CELLS.html]

Details for gene set DESCARTES\_ORGANOGENESIS\_WHITE\_BLOOD\_CELLS[GSEA]

|  || Dataset | Lactate high vs low\_Ranked |
| Phenotype | NoPhenotypeAvailable |
| Upregulated in class | na\_pos |
| GeneSet | DESCARTES\_ORGANOGENESIS\_WHITE\_BLOOD\_CELLS |
| Enrichment Score (ES) | 0.6219268 |
| Normalized Enrichment Score (NES) | 4.947049 |
| Nominal p-value | 0.0 |
| FDR q-value | 0.0 |
| FWER p-Value | 0.0 |
Table: GSEA Results Summary

  

Fig 1: Enrichment plot: DESCARTES\_ORGANOGENESIS\_WHITE\_BLOOD\_CELLS      
 Profile of the Running ES Score & Positions of GeneSet Members on the Rank Ordered List

  

| SYMBOL | RANK IN GENE LIST | RANK METRIC SCORE | RUNNING ES | CORE ENRICHMENT || 1 | C1qb | 2 | 2.291 | 0.0082 | Yes |
| 2 | C3ar1 | 4 | 2.207 | 0.0164 | Yes |
| 3 | Slc37a2 | 5 | 2.178 | 0.0249 | Yes |
| 4 | Apoe | 6 | 2.177 | 0.0334 | Yes |
| 5 | Hk3 | 11 | 2.105 | 0.0401 | Yes |
| 6 | Ctss | 14 | 2.088 | 0.0476 | Yes |
| 7 | Trem2 | 15 | 2.053 | 0.0555 | Yes |
| 8 | Hmox1 | 16 | 2.051 | 0.0635 | Yes |
| 9 | Cd180 | 18 | 2.000 | 0.0709 | Yes |
| 10 | C1qc | 20 | 1.988 | 0.0783 | Yes |
| 11 | Myo1f | 23 | 1.937 | 0.0851 | Yes |
| 12 | Mertk | 24 | 1.934 | 0.0927 | Yes |
| 13 | Cd300c2 | 26 | 1.896 | 0.0997 | Yes |
| 14 | Ms4a6d | 27 | 1.895 | 0.1071 | Yes |
| 15 | Lgmn | 28 | 1.878 | 0.1144 | Yes |
| 16 | C1qa | 29 | 1.862 | 0.1216 | Yes |
| 17 | Cd300a | 32 | 1.849 | 0.1281 | Yes |
| 18 | Cd84 | 35 | 1.825 | 0.1345 | Yes |
| 19 | Cd68 | 36 | 1.820 | 0.1416 | Yes |
| 20 | Ms4a6c | 37 | 1.819 | 0.1486 | Yes |
| 21 | Fcgr1 | 38 | 1.818 | 0.1557 | Yes |
| 22 | Mpeg1 | 42 | 1.799 | 0.1616 | Yes |
| 23 | Slc15a3 | 44 | 1.795 | 0.1683 | Yes |
| 24 | Ctsb | 46 | 1.778 | 0.1748 | Yes |
| 25 | Slc11a1 | 48 | 1.770 | 0.1814 | Yes |
| 26 | Cd37 | 49 | 1.763 | 0.1882 | Yes |
| 27 | Napsa | 50 | 1.758 | 0.1950 | Yes |
| 28 | Cd36 | 52 | 1.748 | 0.2015 | Yes |
| 29 | Sirpa | 56 | 1.702 | 0.2070 | Yes |
| 30 | Psap | 59 | 1.694 | 0.2129 | Yes |
| 31 | Rftn1 | 61 | 1.690 | 0.2191 | Yes |
| 32 | Pik3r5 | 63 | 1.685 | 0.2253 | Yes |
| 33 | Ly86 | 66 | 1.677 | 0.2312 | Yes |
| 34 | Ms4a7 | 79 | 1.623 | 0.2332 | Yes |
| 35 | Evi2a | 81 | 1.620 | 0.2392 | Yes |
| 36 | Tyrobp | 83 | 1.618 | 0.2451 | Yes |
| 37 | Spi1 | 86 | 1.616 | 0.2507 | Yes |
| 38 | Fcer1g | 95 | 1.597 | 0.2541 | Yes |
| 39 | C5ar1 | 96 | 1.596 | 0.2603 | Yes |
| 40 | Myo1g | 99 | 1.578 | 0.2657 | Yes |
| 41 | Lpxn | 100 | 1.576 | 0.2718 | Yes |
| 42 | Cd83 | 101 | 1.575 | 0.2780 | Yes |
| 43 | Fcgr2b | 102 | 1.572 | 0.2841 | Yes |
| 44 | Fcgr3 | 108 | 1.562 | 0.2884 | Yes |
| 45 | Arhgap9 | 110 | 1.552 | 0.2941 | Yes |
| 46 | St6galnac4 | 111 | 1.551 | 0.3001 | Yes |
| 47 | Sash3 | 114 | 1.546 | 0.3054 | Yes |
| 48 | Arhgap25 | 117 | 1.536 | 0.3107 | Yes |
| 49 | Cybb | 119 | 1.535 | 0.3163 | Yes |
| 50 | Nfam1 | 120 | 1.532 | 0.3222 | Yes |
| 51 | Rasa4 | 122 | 1.531 | 0.3278 | Yes |
| 52 | Gusb | 123 | 1.530 | 0.3338 | Yes |
| 53 | Tmem106a | 126 | 1.522 | 0.3390 | Yes |
| 54 | Lair1 | 127 | 1.521 | 0.3449 | Yes |
| 55 | Pstpip1 | 129 | 1.519 | 0.3505 | Yes |
| 56 | Cyth4 | 130 | 1.517 | 0.3564 | Yes |
| 57 | Ctsd | 136 | 1.507 | 0.3605 | Yes |
| 58 | Cd53 | 139 | 1.488 | 0.3655 | Yes |
| 59 | Pik3cd | 144 | 1.476 | 0.3699 | Yes |
| 60 | Cd48 | 146 | 1.476 | 0.3752 | Yes |
| 61 | Gpr65 | 151 | 1.461 | 0.3795 | Yes |
| 62 | Hcls1 | 156 | 1.451 | 0.3837 | Yes |
| 63 | Npl | 163 | 1.441 | 0.3872 | Yes |
| 64 | Myo5a | 165 | 1.435 | 0.3924 | Yes |
| 65 | Slco2b1 | 166 | 1.426 | 0.3980 | Yes |
| 66 | Cfp | 168 | 1.424 | 0.4032 | Yes |
| 67 | Vsir | 170 | 1.421 | 0.4083 | Yes |
| 68 | Tnfrsf1b | 171 | 1.412 | 0.4138 | Yes |
| 69 | Ccr5 | 173 | 1.410 | 0.4190 | Yes |
| 70 | Nckap1l | 182 | 1.403 | 0.4216 | Yes |
| 71 | Plcl2 | 188 | 1.395 | 0.4252 | Yes |
| 72 | Emp3 | 191 | 1.389 | 0.4299 | Yes |
| 73 | Ptgs1 | 192 | 1.388 | 0.4353 | Yes |
| 74 | Tnfrsf26 | 193 | 1.384 | 0.4407 | Yes |
| 75 | Hexb | 197 | 1.379 | 0.4450 | Yes |
| 76 | Pla2g15 | 199 | 1.378 | 0.4500 | Yes |
| 77 | Ncf1 | 201 | 1.373 | 0.4550 | Yes |
| 78 | Cd300lf | 205 | 1.368 | 0.4593 | Yes |
| 79 | Glipr1 | 208 | 1.364 | 0.4639 | Yes |
| 80 | Nrros | 209 | 1.362 | 0.4692 | Yes |
| 81 | Slc29a3 | 219 | 1.353 | 0.4712 | Yes |
| 82 | Dock2 | 229 | 1.333 | 0.4732 | Yes |
| 83 | Fnip2 | 230 | 1.329 | 0.4784 | Yes |
| 84 | Cd52 | 233 | 1.323 | 0.4828 | Yes |
| 85 | Tmem86a | 236 | 1.319 | 0.4873 | Yes |
| 86 | Ctsz | 242 | 1.303 | 0.4906 | Yes |
| 87 | Csf1r | 249 | 1.292 | 0.4935 | Yes |
| 88 | Msr1 | 252 | 1.288 | 0.4978 | Yes |
| 89 | Ptprc | 255 | 1.283 | 0.5021 | Yes |
| 90 | Plxdc1 | 259 | 1.275 | 0.5059 | Yes |
| 91 | Pip4k2a | 260 | 1.275 | 0.5109 | Yes |
| 92 | Prkcb | 268 | 1.263 | 0.5133 | Yes |
| 93 | Gm2a | 274 | 1.259 | 0.5165 | Yes |
| 94 | Ccr2 | 288 | 1.234 | 0.5167 | Yes |
| 95 | Apobec1 | 291 | 1.230 | 0.5207 | Yes |
| 96 | Ctsa | 302 | 1.219 | 0.5219 | Yes |
| 97 | Man2b1 | 305 | 1.216 | 0.5260 | Yes |
| 98 | Kcnab2 | 319 | 1.204 | 0.5260 | Yes |
| 99 | Apbb1ip | 322 | 1.197 | 0.5300 | Yes |
| 100 | Vav1 | 325 | 1.192 | 0.5339 | Yes |
| 101 | Abhd12 | 330 | 1.182 | 0.5371 | Yes |
| 102 | Selplg | 332 | 1.182 | 0.5413 | Yes |
| 103 | Mctp1 | 335 | 1.180 | 0.5452 | Yes |
| 104 | Lgals3 | 344 | 1.170 | 0.5469 | Yes |
| 105 | Crlf2 | 352 | 1.164 | 0.5490 | Yes |
| 106 | Tfec | 358 | 1.154 | 0.5517 | Yes |
| 107 | Grn | 365 | 1.146 | 0.5540 | Yes |
| 108 | Inpp5d | 367 | 1.144 | 0.5581 | Yes |
| 109 | Dse | 368 | 1.143 | 0.5626 | Yes |
| 110 | Adap2 | 372 | 1.137 | 0.5659 | Yes |
| 111 | Nlrc5 | 374 | 1.134 | 0.5700 | Yes |
| 112 | Alox5ap | 375 | 1.133 | 0.5744 | Yes |
| 113 | Cd74 | 376 | 1.133 | 0.5788 | Yes |
| 114 | Fxyd5 | 377 | 1.133 | 0.5832 | Yes |
| 115 | Havcr2 | 384 | 1.119 | 0.5854 | Yes |
| 116 | Tmem104 | 392 | 1.108 | 0.5873 | Yes |
| 117 | Parvg | 393 | 1.107 | 0.5916 | Yes |
| 118 | Ptafr | 395 | 1.106 | 0.5955 | Yes |
| 119 | B2m | 402 | 1.097 | 0.5977 | Yes |
| 120 | Ccl9 | 411 | 1.089 | 0.5991 | Yes |
| 121 | Dock10 | 440 | 1.057 | 0.5933 | Yes |
| 122 | Stard8 | 442 | 1.053 | 0.5970 | Yes |
| 123 | Lcp2 | 449 | 1.047 | 0.5990 | Yes |
| 124 | Lsp1 | 459 | 1.039 | 0.5998 | Yes |
| 125 | Unc93b1 | 464 | 1.037 | 0.6024 | Yes |
| 126 | Il10rb | 469 | 1.031 | 0.6050 | Yes |
| 127 | Dnase2a | 472 | 1.026 | 0.6083 | Yes |
| 128 | Hexa | 476 | 1.021 | 0.6112 | Yes |
| 129 | Tpp1 | 482 | 1.010 | 0.6134 | Yes |
| 130 | Mrc1 | 502 | 0.988 | 0.6105 | Yes |
| 131 | Samhd1 | 505 | 0.985 | 0.6136 | Yes |
| 132 | Tcirg1 | 509 | 0.983 | 0.6164 | Yes |
| 133 | Coro1a | 522 | 0.970 | 0.6159 | Yes |
| 134 | Ctsc | 525 | 0.966 | 0.6189 | Yes |
| 135 | Tifab | 552 | 0.949 | 0.6134 | Yes |
| 136 | Cyba | 554 | 0.947 | 0.6167 | Yes |
| 137 | Dennd4b | 557 | 0.944 | 0.6197 | Yes |
| 138 | Hck | 581 | 0.918 | 0.6151 | Yes |
| 139 | Ehd4 | 595 | 0.901 | 0.6140 | Yes |
| 140 | Il3ra | 609 | 0.883 | 0.6129 | Yes |
| 141 | Blvra | 612 | 0.878 | 0.6156 | Yes |
| 142 | Irf5 | 624 | 0.871 | 0.6151 | Yes |
| 143 | Laptm5 | 659 | 0.844 | 0.6063 | Yes |
| 144 | Irf8 | 663 | 0.840 | 0.6085 | Yes |
| 145 | Actr3 | 665 | 0.838 | 0.6114 | Yes |
| 146 | Cerk | 676 | 0.830 | 0.6111 | Yes |
| 147 | Tbc1d4 | 682 | 0.826 | 0.6125 | Yes |
| 148 | Rnase4 | 691 | 0.817 | 0.6129 | Yes |
| 149 | Il6ra | 700 | 0.810 | 0.6132 | Yes |
| 150 | Arrb2 | 718 | 0.796 | 0.6103 | Yes |
| 151 | H2-D1 | 722 | 0.794 | 0.6123 | Yes |
| 152 | Snx30 | 739 | 0.771 | 0.6096 | Yes |
| 153 | Ifngr1 | 740 | 0.770 | 0.6126 | Yes |
| 154 | Tmem268 | 745 | 0.767 | 0.6142 | Yes |
| 155 | Slc38a6 | 761 | 0.752 | 0.6118 | Yes |
| 156 | Abr | 764 | 0.747 | 0.6140 | Yes |
| 157 | Pik3ap1 | 765 | 0.747 | 0.6169 | Yes |
| 158 | Ccl6 | 772 | 0.736 | 0.6177 | Yes |
| 159 | Cebpb | 773 | 0.736 | 0.6205 | Yes |
| 160 | Cst3 | 782 | 0.723 | 0.6205 | Yes |
| 161 | Rab20 | 811 | 0.700 | 0.6133 | Yes |
| 162 | Abcd1 | 815 | 0.697 | 0.6150 | Yes |
| 163 | Grb2 | 816 | 0.695 | 0.6177 | Yes |
| 164 | H2-K1 | 818 | 0.692 | 0.6200 | Yes |
| 165 | Fgd2 | 824 | 0.686 | 0.6209 | Yes |
| 166 | Plgrkt | 836 | 0.679 | 0.6196 | Yes |
| 167 | Psmb8 | 838 | 0.678 | 0.6219 | Yes |
| 168 | Dab2 | 867 | 0.651 | 0.6145 | No |
| 169 | Gmip | 871 | 0.650 | 0.6160 | No |
| 170 | Ctsh | 890 | 0.637 | 0.6121 | No |
| 171 | Blnk | 895 | 0.634 | 0.6132 | No |
| 172 | Fam111a | 901 | 0.629 | 0.6138 | No |
| 173 | Slfn2 | 908 | 0.624 | 0.6141 | No |
| 174 | Csf3r | 924 | 0.615 | 0.6112 | No |
| 175 | Stk17b | 930 | 0.609 | 0.6118 | No |
| 176 | Fes | 931 | 0.609 | 0.6142 | No |
| 177 | Rhog | 932 | 0.609 | 0.6166 | No |
| 178 | Sdcbp | 945 | 0.602 | 0.6146 | No |
| 179 | Coro7 | 946 | 0.602 | 0.6170 | No |
| 180 | P2rx7 | 951 | 0.599 | 0.6179 | No |
| 181 | H2-M3 | 955 | 0.598 | 0.6192 | No |
| 182 | Ifnar2 | 958 | 0.597 | 0.6208 | No |
| 183 | Paqr7 | 963 | 0.592 | 0.6217 | No |
| 184 | Prkcd | 984 | 0.574 | 0.6168 | No |
| 185 | Lrmda | 999 | 0.564 | 0.6141 | No |
| 186 | Hfe | 1001 | 0.562 | 0.6159 | No |
| 187 | Erp29 | 1003 | 0.562 | 0.6177 | No |
| 188 | Nr1h3 | 1011 | 0.557 | 0.6174 | No |
| 189 | Cfh | 1030 | 0.548 | 0.6132 | No |
| 190 | Nagpa | 1032 | 0.548 | 0.6149 | No |
| 191 | Lamp1 | 1051 | 0.536 | 0.6107 | No |
| 192 | Iqgap1 | 1058 | 0.534 | 0.6106 | No |
| 193 | Atg7 | 1067 | 0.529 | 0.6098 | No |
| 194 | Cd44 | 1072 | 0.523 | 0.6104 | No |
| 195 | Litaf | 1084 | 0.517 | 0.6086 | No |
| 196 | Ptpn6 | 1096 | 0.505 | 0.6066 | No |
| 197 | Madd | 1135 | -0.506 | 0.5951 | No |
| 198 | Zfp710 | 1427 | -0.571 | 0.4943 | No |
| 199 | Tcn2 | 1491 | -0.583 | 0.4743 | No |
| 200 | Casp4 | 1495 | -0.585 | 0.4755 | No |
| 201 | Rnf213 | 1561 | -0.604 | 0.4548 | No |
| 202 | Dtx3l | 1644 | -0.631 | 0.4282 | No |
| 203 | Pycard | 2022 | -0.771 | 0.2977 | No |
| 204 | Jdp2 | 2042 | -0.780 | 0.2940 | No |
| 205 | Ighm | 2137 | -0.822 | 0.2640 | No |
| 206 | Snx6 | 2190 | -0.854 | 0.2489 | No |
| 207 | Map3k5 | 2330 | -0.942 | 0.2033 | No |
| 208 | Galnt6 | 2353 | -0.967 | 0.1993 | No |
| 209 | Ly6e | 2392 | -1.001 | 0.1897 | No |
| 210 | Cyp27a1 | 2463 | -1.060 | 0.1690 | No |
| 211 | Engase | 2512 | -1.108 | 0.1564 | No |
| 212 | Il18 | 2586 | -1.191 | 0.1351 | No |
| 213 | Ptpn22 | 2611 | -1.223 | 0.1314 | No |
| 214 | Plac8 | 2695 | -1.353 | 0.1073 | No |
| 215 | Tlr2 | 2819 | -1.629 | 0.0701 | No |
| 216 | Cela1 | 2888 | -1.928 | 0.0535 | No |
Table: GSEA details [plain text format]

  

Fig 2: DESCARTES\_ORGANOGENESIS\_WHITE\_BLOOD\_CELLS: Random ES distribution      
 Gene set null distribution of ES for **DESCARTES\_ORGANOGENESIS\_WHITE\_BLOOD\_CELLS**

  
