# Supplementary material for: TMPRSS11B promotes an acidified microenvironment and immune suppression in squamous lung cancer
Source: EMBO Rep. 2025 Nov 10;26(24):6346–79. doi: 10.1038/s44319-025-00631-1 (PMC12714794; doi:10.1038/s44319-025-00631-1)
Supplement: Supplementary file 11 — Source data Fig. 6 [file 44319_2025_631_MOESM11_ESM.zip › Figure 6/6D-E/GSEA Broad Institute_low pH vs rest of the regions (high pH)/gsea_report_for_na_neg_1723659072606.html]

Report for na\_neg 1723659072606 [GSEA]

| GS  follow link to MSigDB | GS DETAILS | SIZE | ES | NES | NOM p-val | FDR q-val | FWER p-val | RANK AT MAX | LEADING EDGE || 1 | DESCARTES\_ORGANOGENESIS\_EPITHELIAL\_CELLS | Details ... | 82 | -0.68 | -3.59 | 0.000 | 0.000 | 0.000 | 618 | tags=74%, list=20%, signal=91% |
| 2 | ZHANG\_UTERUS\_C7\_EPITHELIAL2\_CELL | Details ... | 166 | -0.40 | -2.41 | 0.000 | 0.000 | 0.000 | 805 | tags=47%, list=26%, signal=60% |
| 3 | TABULA\_MURIS\_SENIS\_TONGUE\_KERATINOCYTE\_AGEING | Details ... | 18 | -0.61 | -2.17 | 0.000 | 0.001 | 0.008 | 232 | tags=44%, list=8%, signal=48% |
| 4 | ZHANG\_UTERUS\_C13\_EPITHELIAL1\_CELL | Details ... | 80 | -0.39 | -2.02 | 0.000 | 0.007 | 0.089 | 946 | tags=54%, list=31%, signal=76% |
| 5 | TABULA\_MURIS\_SENIS\_TONGUE\_BASAL\_CELL\_OF\_EPIDERMIS\_AGEING | Details ... | 39 | -0.42 | -1.89 | 0.008 | 0.018 | 0.278 | 279 | tags=28%, list=9%, signal=31% |
| 6 | ZHANG\_UTERUS\_C1\_REGENERATIVE\_UP | Details ... | 95 | -0.34 | -1.87 | 0.000 | 0.018 | 0.320 | 755 | tags=44%, list=25%, signal=57% |
| 7 | DESCARTES\_ORGANOGENESIS\_HEPATOCYTES | Details ... | 121 | -0.31 | -1.74 | 0.006 | 0.047 | 0.685 | 797 | tags=44%, list=26%, signal=57% |
| 8 | TABULA\_MURIS\_SENIS\_MAMMARY\_GLAND\_LUMINAL\_EPITHELIAL\_CELL\_OF\_MAMMARY\_GLAND\_AGEING | Details ... | 116 | -0.30 | -1.72 | 0.004 | 0.048 | 0.740 | 308 | tags=20%, list=10%, signal=21% |
| 9 | TABULA\_MURIS\_SENIS\_GONADAL\_ADIPOSE\_TISSUE\_B\_CELL\_AGEING | Details ... | 26 | -0.41 | -1.59 | 0.045 | 0.105 | 0.965 | 106 | tags=19%, list=3%, signal=20% |
| 10 | ZHANG\_UTERUS\_C2\_REGENERATIVE\_UP | Details ... | 23 | -0.41 | -1.55 | 0.054 | 0.122 | 0.989 | 443 | tags=35%, list=15%, signal=40% |
| 11 | TABULA\_MURIS\_SENIS\_LARGE\_INTESTINE\_INTESTINAL\_CRYPT\_STEM\_CELL\_AGEING | Details ... | 89 | -0.26 | -1.40 | 0.080 | 0.273 | 1.000 | 805 | tags=36%, list=26%, signal=47% |
| 12 | TABULA\_MURIS\_SENIS\_LARGE\_INTESTINE\_ENTEROCYTE\_OF\_EPITHELIUM\_OF\_LARGE\_INTESTINE\_AGEING | Details ... | 215 | -0.21 | -1.33 | 0.069 | 0.365 | 1.000 | 1091 | tags=43%, list=36%, signal=63% |
| 13 | TABULA\_MURIS\_SENIS\_LARGE\_INTESTINE\_LARGE\_INTESTINE\_GOBLET\_CELL\_AGEING | Details ... | 169 | -0.22 | -1.30 | 0.102 | 0.395 | 1.000 | 1067 | tags=43%, list=35%, signal=62% |
| 14 | TABULA\_MURIS\_SENIS\_SKIN\_EPIDERMAL\_CELL\_AGEING | Details ... | 159 | -0.22 | -1.28 | 0.115 | 0.390 | 1.000 | 575 | tags=24%, list=19%, signal=28% |
| 15 | TABULA\_MURIS\_SENIS\_SKIN\_BASAL\_CELL\_OF\_EPIDERMIS\_AGEING | Details ... | 210 | -0.20 | -1.25 | 0.127 | 0.433 | 1.000 | 542 | tags=21%, list=18%, signal=24% |
| 16 | TABULA\_MURIS\_SENIS\_MARROW\_NAIVE\_T\_CELL\_AGEING | Details ... | 20 | -0.33 | -1.23 | 0.242 | 0.439 | 1.000 | 78 | tags=15%, list=3%, signal=15% |
| 17 | TABULA\_MURIS\_SENIS\_PANCREAS\_PANCREATIC\_POLYPEPTIDE\_CELL\_AGEING | Details ... | 28 | -0.30 | -1.19 | 0.239 | 0.483 | 1.000 | 663 | tags=36%, list=22%, signal=45% |
| 18 | TABULA\_MURIS\_SENIS\_MARROW\_MACROPHAGE\_AGEING | Details ... | 41 | -0.26 | -1.16 | 0.287 | 0.516 | 1.000 | 135 | tags=12%, list=4%, signal=13% |
| 19 | TABULA\_MURIS\_SENIS\_MARROW\_ERYTHROBLAST\_AGEING | Details ... | 27 | -0.27 | -1.10 | 0.371 | 0.623 | 1.000 | 338 | tags=19%, list=11%, signal=21% |
| 20 | TABULA\_MURIS\_SENIS\_BLADDER\_BLADDER\_UROTHELIAL\_CELL\_AGEING | Details ... | 55 | -0.23 | -1.08 | 0.348 | 0.624 | 1.000 | 545 | tags=27%, list=18%, signal=33% |
| 21 | DESCARTES\_ORGANOGENESIS\_SENSORY\_NEURONS |  | 17 | -0.30 | -1.08 | 0.364 | 0.604 | 1.000 | 764 | tags=41%, list=25%, signal=55% |
| 22 | TABULA\_MURIS\_SENIS\_SPLEEN\_GRANULOCYTE\_AGEING |  | 38 | -0.22 | -0.98 | 0.497 | 0.775 | 1.000 | 93 | tags=8%, list=3%, signal=8% |
| 23 | TABULA\_MURIS\_SENIS\_HEART\_VENTRICULAR\_MYOCYTE\_AGEING |  | 28 | -0.23 | -0.95 | 0.514 | 0.807 | 1.000 | 426 | tags=18%, list=14%, signal=21% |
| 24 | TABULA\_MURIS\_SENIS\_KIDNEY\_KIDNEY\_COLLECTING\_DUCT\_PRINCIPAL\_CELL\_AGEING |  | 96 | -0.17 | -0.95 | 0.528 | 0.792 | 1.000 | 550 | tags=22%, list=18%, signal=26% |
| 25 | TABULA\_MURIS\_SENIS\_SUBCUTANEOUS\_ADIPOSE\_TISSUE\_EPITHELIAL\_CELL\_AGEING |  | 139 | -0.16 | -0.93 | 0.591 | 0.799 | 1.000 | 767 | tags=28%, list=25%, signal=36% |
| 26 | DESCARTES\_ORGANOGENESIS\_STROMAL\_CELLS |  | 29 | -0.22 | -0.89 | 0.603 | 0.838 | 1.000 | 1924 | tags=86%, list=63%, signal=233% |
| 27 | TABULA\_MURIS\_SENIS\_LARGE\_INTESTINE\_SECRETORY\_CELL\_AGEING |  | 146 | -0.15 | -0.88 | 0.669 | 0.824 | 1.000 | 1914 | tags=79%, list=63%, signal=202% |
| 28 | TABULA\_MURIS\_SENIS\_MARROW\_PROERYTHROBLAST\_AGEING |  | 16 | -0.25 | -0.86 | 0.627 | 0.827 | 1.000 | 6 | tags=6%, list=0%, signal=6% |
| 29 | DESCARTES\_ORGANOGENESIS\_PRIMITIVE\_ERYTHROID\_LINEAGE |  | 106 | -0.13 | -0.73 | 0.865 | 1.000 | 1.000 | 695 | tags=25%, list=23%, signal=31% |
| 30 | DESCARTES\_ORGANOGENESIS\_SCHWANN\_CELL\_PRECURSOR |  | 16 | -0.21 | -0.70 | 0.815 | 1.000 | 1.000 | 875 | tags=38%, list=29%, signal=52% |
| 31 | TABULA\_MURIS\_SENIS\_BLADDER\_ENDOTHELIAL\_CELL\_AGEING |  | 115 | -0.12 | -0.69 | 0.900 | 0.983 | 1.000 | 545 | tags=18%, list=18%, signal=21% |
| 32 | TABULA\_MURIS\_SENIS\_KIDNEY\_EPITHELIAL\_CELL\_OF\_PROXIMAL\_TUBULE\_AGEING |  | 67 | -0.13 | -0.67 | 0.906 | 0.980 | 1.000 | 354 | tags=15%, list=12%, signal=17% |
| 33 | TABULA\_MURIS\_SENIS\_PANCREAS\_PANCREATIC\_DELTA\_CELL\_AGEING |  | 47 | -0.12 | -0.59 | 0.938 | 1.000 | 1.000 | 1016 | tags=36%, list=33%, signal=53% |
| 34 | TABULA\_MURIS\_SENIS\_PANCREAS\_PANCREATIC\_DUCTAL\_CELL\_AGEING |  | 73 | -0.09 | -0.45 | 0.994 | 1.000 | 1.000 | 880 | tags=27%, list=29%, signal=38% |
| 35 | DESCARTES\_ORGANOGENESIS\_CARDIAC\_MUSCLE\_LINEAGES |  | 19 | -0.12 | -0.45 | 0.988 | 0.992 | 1.000 | 752 | tags=26%, list=25%, signal=35% |
Table: Gene sets enriched in phenotype **na**[plain text format]****

  
