# Supplementary material for: TMPRSS11B promotes an acidified microenvironment and immune suppression in squamous lung cancer
Source: EMBO Rep. 2025 Nov 10;26(24):6346–79. doi: 10.1038/s44319-025-00631-1 (PMC12714794; doi:10.1038/s44319-025-00631-1)
Supplement: Supplementary file 11 — Source data Fig. 6 [file 44319_2025_631_MOESM11_ESM.zip › Figure 6/6D-E/GSEA Broad Institute_low pH vs rest of the regions (high pH)/gsea_report_for_na_pos_1723659072606.html]

Report for na\_pos 1723659072606 [GSEA]

| GS  follow link to MSigDB | GS DETAILS | SIZE | ES | NES | NOM p-val | FDR q-val | FWER p-val | RANK AT MAX | LEADING EDGE || 1 | DESCARTES\_ORGANOGENESIS\_WHITE\_BLOOD\_CELLS | Details ... | 216 | 0.62 | 4.95 | 0.000 | 0.000 | 0.000 | 838 | tags=77%, list=28%, signal=99% |
| 2 | ZHANG\_UTERUS\_C5\_MACROPHAGE | Details ... | 100 | 0.68 | 4.70 | 0.000 | 0.000 | 0.000 | 554 | tags=73%, list=18%, signal=86% |
| 3 | ZHANG\_UTERUS\_C12\_MONOCYTE | Details ... | 104 | 0.64 | 4.37 | 0.000 | 0.000 | 0.000 | 665 | tags=73%, list=22%, signal=90% |
| 4 | ZHANG\_UTERUS\_C9\_DENDRITIC\_CELL | Details ... | 53 | 0.70 | 4.10 | 0.000 | 0.000 | 0.000 | 861 | tags=94%, list=28%, signal=129% |
| 5 | TABULA\_MURIS\_SENIS\_LUNG\_ALVEOLAR\_MACROPHAGE\_AGEING | Details ... | 63 | 0.61 | 3.81 | 0.000 | 0.000 | 0.000 | 465 | tags=63%, list=15%, signal=73% |
| 6 | TABULA\_MURIS\_SENIS\_BRAIN\_MYELOID\_MICROGLIAL\_CELL\_AGEING | Details ... | 103 | 0.51 | 3.56 | 0.000 | 0.000 | 0.000 | 1006 | tags=81%, list=33%, signal=116% |
| 7 | TABULA\_MURIS\_SENIS\_BROWN\_ADIPOSE\_TISSUE\_B\_CELL\_AGEING | Details ... | 84 | 0.51 | 3.48 | 0.000 | 0.000 | 0.000 | 838 | tags=69%, list=28%, signal=93% |
| 8 | TABULA\_MURIS\_SENIS\_KIDNEY\_MACROPHAGE\_AGEING | Details ... | 49 | 0.57 | 3.43 | 0.000 | 0.000 | 0.000 | 493 | tags=59%, list=16%, signal=70% |
| 9 | TABULA\_MURIS\_SENIS\_MAMMARY\_GLAND\_T\_CELL\_AGEING | Details ... | 126 | 0.43 | 3.22 | 0.000 | 0.000 | 0.000 | 868 | tags=63%, list=29%, signal=85% |
| 10 | DESCARTES\_ORGANOGENESIS\_ENDOTHELIAL\_CELLS | Details ... | 112 | 0.45 | 3.09 | 0.000 | 0.000 | 0.000 | 974 | tags=75%, list=32%, signal=106% |
| 11 | ZHANG\_UTERUS\_C8\_NK\_CELL | Details ... | 52 | 0.52 | 3.06 | 0.000 | 0.000 | 0.000 | 1040 | tags=85%, list=34%, signal=126% |
| 12 | TABULA\_MURIS\_SENIS\_SPLEEN\_MACROPHAGE\_AGEING | Details ... | 50 | 0.52 | 3.02 | 0.000 | 0.000 | 0.000 | 724 | tags=62%, list=24%, signal=80% |
| 13 | TABULA\_MURIS\_SENIS\_LUNG\_B\_CELL\_AGEING | Details ... | 38 | 0.55 | 2.99 | 0.000 | 0.000 | 0.000 | 980 | tags=82%, list=32%, signal=119% |
| 14 | TABULA\_MURIS\_SENIS\_BROWN\_ADIPOSE\_TISSUE\_ENDOTHELIAL\_CELL\_AGEING | Details ... | 62 | 0.48 | 2.91 | 0.000 | 0.000 | 0.000 | 985 | tags=71%, list=32%, signal=103% |
| 15 | ZHANG\_UTERUS\_C6\_ENDOTHELIAL\_PLVAP\_HIGH\_CELL | Details ... | 58 | 0.46 | 2.86 | 0.000 | 0.000 | 0.000 | 748 | tags=67%, list=25%, signal=87% |
| 16 | TABULA\_MURIS\_SENIS\_HEART\_FIBROBLAST\_OF\_CARDIAC\_TISSUE\_AGEING | Details ... | 73 | 0.44 | 2.86 | 0.000 | 0.000 | 0.000 | 860 | tags=60%, list=28%, signal=82% |
| 17 | TABULA\_MURIS\_SENIS\_SPLEEN\_B\_CELL\_AGEING | Details ... | 41 | 0.51 | 2.84 | 0.000 | 0.000 | 0.000 | 1072 | tags=83%, list=35%, signal=126% |
| 18 | TABULA\_MURIS\_SENIS\_MAMMARY\_GLAND\_B\_CELL\_AGEING | Details ... | 97 | 0.41 | 2.84 | 0.000 | 0.000 | 0.000 | 862 | tags=62%, list=28%, signal=84% |
| 19 | TABULA\_MURIS\_SENIS\_LUNG\_BRONCHIAL\_SMOOTH\_MUSCLE\_CELL\_AGEING | Details ... | 53 | 0.47 | 2.82 | 0.000 | 0.000 | 0.000 | 976 | tags=77%, list=32%, signal=112% |
| 20 | TABULA\_MURIS\_SENIS\_HEART\_MONOCYTE\_AGEING | Details ... | 49 | 0.49 | 2.77 | 0.000 | 0.000 | 0.000 | 567 | tags=53%, list=19%, signal=64% |
| 21 | TABULA\_MURIS\_SENIS\_AORTA\_AORTIC\_ENDOTHELIAL\_CELL\_AGEING |  | 85 | 0.40 | 2.73 | 0.000 | 0.000 | 0.000 | 626 | tags=53%, list=21%, signal=65% |
| 22 | TABULA\_MURIS\_SENIS\_HEART\_AND\_AORTA\_FIBROBLAST\_OF\_CARDIAC\_TISSUE\_AGEING |  | 86 | 0.40 | 2.72 | 0.000 | 0.000 | 0.000 | 870 | tags=59%, list=29%, signal=81% |
| 23 | TABULA\_MURIS\_SENIS\_AORTA\_PROFESSIONAL\_ANTIGEN\_PRESENTING\_CELL\_AGEING |  | 54 | 0.45 | 2.71 | 0.000 | 0.000 | 0.000 | 569 | tags=56%, list=19%, signal=67% |
| 24 | TABULA\_MURIS\_SENIS\_LIVER\_ENDOTHELIAL\_CELL\_OF\_HEPATIC\_SINUSOID\_AGEING |  | 92 | 0.40 | 2.68 | 0.000 | 0.000 | 0.000 | 881 | tags=55%, list=29%, signal=76% |
| 25 | TABULA\_MURIS\_SENIS\_LUNG\_INTERMEDIATE\_MONOCYTE\_AGEING |  | 76 | 0.41 | 2.68 | 0.000 | 0.000 | 0.000 | 581 | tags=43%, list=19%, signal=52% |
| 26 | TABULA\_MURIS\_SENIS\_BROWN\_ADIPOSE\_TISSUE\_MYELOID\_CELL\_AGEING |  | 68 | 0.43 | 2.67 | 0.000 | 0.000 | 0.000 | 782 | tags=57%, list=26%, signal=75% |
| 27 | ZHANG\_UTERUS\_C10\_STROMAL2\_RETNLG\_HIGH\_CELL |  | 24 | 0.57 | 2.66 | 0.000 | 0.000 | 0.000 | 878 | tags=88%, list=29%, signal=122% |
| 28 | TABULA\_MURIS\_SENIS\_HEART\_ENDOTHELIAL\_CELL\_OF\_CORONARY\_ARTERY\_AGEING |  | 45 | 0.47 | 2.66 | 0.000 | 0.000 | 0.000 | 569 | tags=53%, list=19%, signal=65% |
| 29 | ZHANG\_UTERUS\_C1\_PROLIFERATIVE\_STROMAL1\_MGP\_HIGH\_CELL |  | 102 | 0.37 | 2.62 | 0.000 | 0.000 | 0.000 | 879 | tags=61%, list=29%, signal=83% |
| 30 | TABULA\_MURIS\_SENIS\_LUNG\_NON\_CLASSICAL\_MONOCYTE\_AGEING |  | 18 | 0.64 | 2.60 | 0.000 | 0.000 | 0.000 | 656 | tags=78%, list=22%, signal=99% |
| 31 | TABULA\_MURIS\_SENIS\_KIDNEY\_KIDNEY\_CORTEX\_ARTERY\_CELL\_AGEING |  | 18 | 0.61 | 2.59 | 0.000 | 0.000 | 0.000 | 818 | tags=83%, list=27%, signal=113% |
| 32 | TABULA\_MURIS\_SENIS\_MESENTERIC\_ADIPOSE\_TISSUE\_MACROPHAGE\_AGEING |  | 24 | 0.57 | 2.58 | 0.000 | 0.000 | 0.000 | 612 | tags=67%, list=20%, signal=83% |
| 33 | TABULA\_MURIS\_SENIS\_SUBCUTANEOUS\_ADIPOSE\_TISSUE\_B\_CELL\_AGEING |  | 47 | 0.45 | 2.58 | 0.000 | 0.000 | 0.000 | 617 | tags=53%, list=20%, signal=66% |
| 34 | TABULA\_MURIS\_SENIS\_LIMB\_MUSCLE\_ENDOTHELIAL\_CELL\_AGEING |  | 44 | 0.44 | 2.54 | 0.000 | 0.000 | 0.000 | 961 | tags=75%, list=32%, signal=108% |
| 35 | TABULA\_MURIS\_SENIS\_MAMMARY\_GLAND\_ENDOTHELIAL\_CELL\_AGEING |  | 123 | 0.34 | 2.50 | 0.000 | 0.000 | 0.000 | 974 | tags=59%, list=32%, signal=83% |
| 36 | TABULA\_MURIS\_SENIS\_MESENTERIC\_ADIPOSE\_TISSUE\_CD4\_POSITIVE\_ALPHA\_BETA\_T\_CELL\_AGEING |  | 25 | 0.52 | 2.48 | 0.000 | 0.000 | 0.000 | 460 | tags=52%, list=15%, signal=61% |
| 37 | TABULA\_MURIS\_SENIS\_MARROW\_GRANULOCYTE\_AGEING |  | 27 | 0.50 | 2.48 | 0.000 | 0.000 | 0.000 | 818 | tags=74%, list=27%, signal=100% |
| 38 | TABULA\_MURIS\_SENIS\_LIMB\_MUSCLE\_MACROPHAGE\_AGEING |  | 39 | 0.46 | 2.48 | 0.000 | 0.000 | 0.000 | 554 | tags=56%, list=18%, signal=68% |
| 39 | TABULA\_MURIS\_SENIS\_BRAIN\_MYELOID\_MACROPHAGE\_AGEING |  | 16 | 0.61 | 2.43 | 0.000 | 0.000 | 0.000 | 973 | tags=94%, list=32%, signal=137% |
| 40 | TABULA\_MURIS\_SENIS\_SPLEEN\_T\_CELL\_AGEING |  | 60 | 0.40 | 2.42 | 0.000 | 0.000 | 0.001 | 965 | tags=67%, list=32%, signal=96% |
| 41 | TABULA\_MURIS\_SENIS\_LUNG\_CD4\_POSITIVE\_ALPHA\_BETA\_T\_CELL\_AGEING |  | 40 | 0.43 | 2.41 | 0.000 | 0.000 | 0.002 | 880 | tags=68%, list=29%, signal=94% |
| 42 | TABULA\_MURIS\_SENIS\_LUNG\_FIBROBLAST\_OF\_LUNG\_AGEING |  | 73 | 0.37 | 2.39 | 0.000 | 0.000 | 0.002 | 1087 | tags=67%, list=36%, signal=102% |
| 43 | TABULA\_MURIS\_SENIS\_BROWN\_ADIPOSE\_TISSUE\_T\_CELL\_AGEING |  | 31 | 0.48 | 2.39 | 0.000 | 0.000 | 0.002 | 574 | tags=55%, list=19%, signal=67% |
| 44 | TABULA\_MURIS\_SENIS\_MESENTERIC\_ADIPOSE\_TISSUE\_B\_CELL\_AGEING |  | 32 | 0.45 | 2.35 | 0.000 | 0.000 | 0.002 | 567 | tags=56%, list=19%, signal=68% |
| 45 | TABULA\_MURIS\_SENIS\_MESENTERIC\_ADIPOSE\_TISSUE\_ENDOTHELIAL\_CELL\_AGEING |  | 22 | 0.53 | 2.33 | 0.000 | 0.000 | 0.002 | 1070 | tags=86%, list=35%, signal=132% |
| 46 | TABULA\_MURIS\_SENIS\_SUBCUTANEOUS\_ADIPOSE\_TISSUE\_MESENCHYMAL\_STEM\_CELL\_OF\_ADIPOSE\_AGEING |  | 90 | 0.35 | 2.32 | 0.000 | 0.000 | 0.002 | 1076 | tags=69%, list=35%, signal=103% |
| 47 | TABULA\_MURIS\_SENIS\_GONADAL\_ADIPOSE\_TISSUE\_MESENCHYMAL\_STEM\_CELL\_OF\_ADIPOSE\_AGEING |  | 182 | 0.30 | 2.30 | 0.000 | 0.000 | 0.003 | 1084 | tags=62%, list=36%, signal=91% |
| 48 | TABULA\_MURIS\_SENIS\_MARROW\_NAIVE\_B\_CELL\_AGEING |  | 56 | 0.38 | 2.30 | 0.000 | 0.000 | 0.003 | 857 | tags=57%, list=28%, signal=78% |
| 49 | TABULA\_MURIS\_SENIS\_MAMMARY\_GLAND\_STROMAL\_CELL\_AGEING |  | 95 | 0.33 | 2.29 | 0.000 | 0.000 | 0.003 | 879 | tags=57%, list=29%, signal=77% |
| 50 | TABULA\_MURIS\_SENIS\_MARROW\_NK\_CELL\_AGEING |  | 91 | 0.33 | 2.28 | 0.000 | 0.000 | 0.003 | 732 | tags=44%, list=24%, signal=56% |
| 51 | TABULA\_MURIS\_SENIS\_SUBCUTANEOUS\_ADIPOSE\_TISSUE\_MYELOID\_CELL\_AGEING |  | 71 | 0.35 | 2.25 | 0.000 | 0.000 | 0.004 | 1004 | tags=66%, list=33%, signal=97% |
| 52 | TABULA\_MURIS\_SENIS\_HEART\_AND\_AORTA\_ENDOTHELIAL\_CELL\_OF\_CORONARY\_ARTERY\_AGEING |  | 77 | 0.35 | 2.23 | 0.000 | 0.000 | 0.006 | 926 | tags=61%, list=30%, signal=86% |
| 53 | TABULA\_MURIS\_SENIS\_HEART\_VALVE\_CELL\_AGEING |  | 34 | 0.42 | 2.23 | 0.000 | 0.000 | 0.007 | 992 | tags=71%, list=33%, signal=104% |
| 54 | ZHANG\_UTERUS\_C2\_SECRETORY\_STROMAL3\_RAMP3\_HIGH\_CELL |  | 70 | 0.35 | 2.23 | 0.000 | 0.000 | 0.007 | 743 | tags=50%, list=24%, signal=65% |
| 55 | TABULA\_MURIS\_SENIS\_MARROW\_GRANULOCYTOPOIETIC\_CELL\_AGEING |  | 39 | 0.41 | 2.22 | 0.000 | 0.001 | 0.009 | 726 | tags=67%, list=24%, signal=86% |
| 56 | ZHANG\_UTERUS\_C0\_SECRETORY\_STROMAL3\_NPPC\_HIGH\_CELL |  | 102 | 0.31 | 2.19 | 0.000 | 0.001 | 0.014 | 743 | tags=45%, list=24%, signal=58% |
| 57 | TABULA\_MURIS\_SENIS\_MARROW\_HEMATOPOIETIC\_PRECURSOR\_CELL\_AGEING |  | 39 | 0.40 | 2.17 | 0.000 | 0.001 | 0.016 | 908 | tags=77%, list=30%, signal=108% |
| 58 | TABULA\_MURIS\_SENIS\_MARROW\_GRANULOCYTE\_MONOCYTE\_PROGENITOR\_CELL\_AGEING |  | 34 | 0.41 | 2.16 | 0.000 | 0.001 | 0.016 | 503 | tags=44%, list=17%, signal=52% |
| 59 | TABULA\_MURIS\_SENIS\_LIVER\_MATURE\_NK\_T\_CELL\_AGEING |  | 27 | 0.45 | 2.14 | 0.000 | 0.001 | 0.020 | 902 | tags=63%, list=30%, signal=89% |
| 60 | ZHANG\_UTERUS\_C14\_ENDOTHELIAL\_MMRN1\_HIGH\_CELL |  | 17 | 0.52 | 2.11 | 0.003 | 0.001 | 0.026 | 970 | tags=82%, list=32%, signal=120% |
| 61 | TABULA\_MURIS\_SENIS\_KIDNEY\_FENESTRATED\_CELL\_AGEING |  | 27 | 0.43 | 2.11 | 0.000 | 0.001 | 0.026 | 503 | tags=52%, list=17%, signal=62% |
| 62 | TABULA\_MURIS\_SENIS\_HEART\_AND\_AORTA\_LEUKOCYTE\_AGEING |  | 40 | 0.39 | 2.10 | 0.000 | 0.001 | 0.027 | 870 | tags=60%, list=29%, signal=83% |
| 63 | TABULA\_MURIS\_SENIS\_LIMB\_MUSCLE\_SMOOTH\_MUSCLE\_CELL\_AGEING |  | 22 | 0.47 | 2.10 | 0.000 | 0.001 | 0.028 | 1079 | tags=82%, list=35%, signal=126% |
| 64 | TABULA\_MURIS\_SENIS\_LUNG\_ADVENTITIAL\_CELL\_AGEING |  | 54 | 0.36 | 2.05 | 0.000 | 0.002 | 0.047 | 1087 | tags=67%, list=36%, signal=102% |
| 65 | TABULA\_MURIS\_SENIS\_MESENTERIC\_ADIPOSE\_TISSUE\_MESENCHYMAL\_STEM\_CELL\_OF\_ADIPOSE\_AGEING |  | 166 | 0.26 | 2.05 | 0.000 | 0.002 | 0.049 | 1091 | tags=57%, list=36%, signal=84% |
| 66 | TABULA\_MURIS\_SENIS\_GONADAL\_ADIPOSE\_TISSUE\_MYELOID\_CELL\_AGEING |  | 87 | 0.30 | 2.04 | 0.000 | 0.002 | 0.050 | 1003 | tags=64%, list=33%, signal=93% |
| 67 | TABULA\_MURIS\_SENIS\_THYMUS\_DN4\_THYMOCYTE\_AGEING |  | 46 | 0.35 | 2.02 | 0.000 | 0.003 | 0.058 | 973 | tags=65%, list=32%, signal=94% |
| 68 | TABULA\_MURIS\_SENIS\_LUNG\_CLASSICAL\_MONOCYTE\_AGEING |  | 57 | 0.33 | 2.02 | 0.000 | 0.003 | 0.061 | 574 | tags=37%, list=19%, signal=45% |
| 69 | ZHANG\_UTERUS\_C4\_MYOFIBROBLAST |  | 106 | 0.29 | 2.02 | 0.000 | 0.003 | 0.061 | 859 | tags=50%, list=28%, signal=67% |
| 70 | ZHANG\_UTERUS\_C3\_PROLIFERATIVE\_STROMAL1\_STROCXCL14\_HIGH\_CELL |  | 27 | 0.42 | 2.01 | 0.000 | 0.003 | 0.066 | 860 | tags=74%, list=28%, signal=102% |
| 71 | TABULA\_MURIS\_SENIS\_KIDNEY\_KIDNEY\_PROXIMAL\_CONVOLUTED\_TUBULE\_EPITHELIAL\_CELL\_AGEING |  | 41 | 0.36 | 1.99 | 0.003 | 0.004 | 0.079 | 603 | tags=49%, list=20%, signal=60% |
| 72 | TABULA\_MURIS\_SENIS\_LIMB\_MUSCLE\_B\_CELL\_AGEING |  | 53 | 0.32 | 1.94 | 0.000 | 0.005 | 0.109 | 859 | tags=51%, list=28%, signal=70% |
| 73 | TABULA\_MURIS\_SENIS\_SPLEEN\_MATURE\_NK\_T\_CELL\_AGEING |  | 18 | 0.46 | 1.93 | 0.015 | 0.006 | 0.119 | 574 | tags=50%, list=19%, signal=61% |
| 74 | TABULA\_MURIS\_SENIS\_LIVER\_NK\_CELL\_AGEING |  | 58 | 0.31 | 1.92 | 0.008 | 0.006 | 0.124 | 714 | tags=47%, list=23%, signal=60% |
| 75 | TABULA\_MURIS\_SENIS\_SPLEEN\_CD4\_POSITIVE\_ALPHA\_BETA\_T\_CELL\_AGEING |  | 54 | 0.31 | 1.90 | 0.004 | 0.006 | 0.140 | 907 | tags=61%, list=30%, signal=86% |
| 76 | TABULA\_MURIS\_SENIS\_DIAPHRAGM\_MESENCHYMAL\_STEM\_CELL\_AGEING |  | 57 | 0.31 | 1.86 | 0.009 | 0.008 | 0.184 | 1087 | tags=61%, list=36%, signal=94% |
| 77 | TABULA\_MURIS\_SENIS\_MARROW\_MONOCYTE\_AGEING |  | 16 | 0.46 | 1.85 | 0.003 | 0.009 | 0.195 | 465 | tags=69%, list=15%, signal=81% |
| 78 | TABULA\_MURIS\_SENIS\_KIDNEY\_PODOCYTE\_AGEING |  | 60 | 0.29 | 1.84 | 0.008 | 0.009 | 0.207 | 1070 | tags=62%, list=35%, signal=93% |
| 79 | TABULA\_MURIS\_SENIS\_MAMMARY\_GLAND\_MACROPHAGE\_AGEING |  | 33 | 0.35 | 1.81 | 0.013 | 0.011 | 0.240 | 804 | tags=55%, list=26%, signal=73% |
| 80 | TABULA\_MURIS\_SENIS\_GONADAL\_ADIPOSE\_TISSUE\_ENDOTHELIAL\_CELL\_AGEING |  | 228 | 0.22 | 1.79 | 0.000 | 0.012 | 0.275 | 1003 | tags=50%, list=33%, signal=68% |
| 81 | DESCARTES\_ORGANOGENESIS\_MELANOCYTES |  | 16 | 0.44 | 1.79 | 0.016 | 0.013 | 0.283 | 316 | tags=44%, list=10%, signal=49% |
| 82 | TABULA\_MURIS\_SENIS\_MARROW\_PRECURSOR\_B\_CELL\_AGEING |  | 31 | 0.35 | 1.79 | 0.006 | 0.012 | 0.283 | 547 | tags=48%, list=18%, signal=58% |
| 83 | TABULA\_MURIS\_SENIS\_LIMB\_MUSCLE\_MESENCHYMAL\_STEM\_CELL\_AGEING |  | 60 | 0.29 | 1.77 | 0.016 | 0.013 | 0.305 | 1070 | tags=65%, list=35%, signal=98% |
| 84 | TABULA\_MURIS\_SENIS\_TRACHEA\_ENDOTHELIAL\_CELL\_AGEING |  | 46 | 0.28 | 1.62 | 0.010 | 0.033 | 0.601 | 1077 | tags=63%, list=35%, signal=96% |
| 85 | TABULA\_MURIS\_SENIS\_PANCREAS\_ENDOTHELIAL\_CELL\_AGEING |  | 56 | 0.26 | 1.62 | 0.032 | 0.033 | 0.603 | 1088 | tags=61%, list=36%, signal=93% |
| 86 | TABULA\_MURIS\_SENIS\_BRAIN\_NON\_MYELOID\_ENDOTHELIAL\_CELL\_AGEING |  | 82 | 0.24 | 1.60 | 0.010 | 0.038 | 0.659 | 905 | tags=49%, list=30%, signal=68% |
| 87 | TABULA\_MURIS\_SENIS\_SUBCUTANEOUS\_ADIPOSE\_TISSUE\_ENDOTHELIAL\_CELL\_AGEING |  | 58 | 0.26 | 1.57 | 0.017 | 0.044 | 0.718 | 1031 | tags=62%, list=34%, signal=92% |
| 88 | TABULA\_MURIS\_SENIS\_MARROW\_IMMATURE\_B\_CELL\_AGEING |  | 37 | 0.29 | 1.56 | 0.031 | 0.044 | 0.723 | 973 | tags=62%, list=32%, signal=90% |
| 89 | TABULA\_MURIS\_SENIS\_SPLEEN\_CD8\_POSITIVE\_ALPHA\_BETA\_T\_CELL\_AGEING |  | 58 | 0.26 | 1.56 | 0.012 | 0.044 | 0.726 | 973 | tags=60%, list=32%, signal=87% |
| 90 | TABULA\_MURIS\_SENIS\_MARROW\_PROMONOCYTE\_AGEING |  | 27 | 0.32 | 1.56 | 0.033 | 0.044 | 0.735 | 554 | tags=48%, list=18%, signal=58% |
| 91 | TABULA\_MURIS\_SENIS\_LIMB\_MUSCLE\_SKELETAL\_MUSCLE\_SATELLITE\_CELL\_AGEING |  | 31 | 0.31 | 1.55 | 0.026 | 0.046 | 0.753 | 848 | tags=52%, list=28%, signal=71% |
| 92 | TABULA\_MURIS\_SENIS\_TRACHEA\_MACROPHAGE\_AGEING |  | 16 | 0.37 | 1.52 | 0.078 | 0.054 | 0.816 | 973 | tags=63%, list=32%, signal=91% |
| 93 | TABULA\_MURIS\_SENIS\_LIMB\_MUSCLE\_T\_CELL\_AGEING |  | 69 | 0.24 | 1.50 | 0.041 | 0.059 | 0.844 | 1076 | tags=58%, list=35%, signal=88% |
| 94 | TABULA\_MURIS\_SENIS\_HEART\_ATRIAL\_MYOCYTE\_AGEING |  | 30 | 0.30 | 1.49 | 0.044 | 0.061 | 0.853 | 522 | tags=37%, list=17%, signal=44% |
| 95 | TABULA\_MURIS\_SENIS\_KIDNEY\_KIDNEY\_MESANGIAL\_CELL\_AGEING |  | 18 | 0.36 | 1.47 | 0.080 | 0.069 | 0.889 | 818 | tags=61%, list=27%, signal=83% |
| 96 | TABULA\_MURIS\_SENIS\_BRAIN\_NON\_MYELOID\_OLIGODENDROCYTE\_PRECURSOR\_CELL\_AGEING |  | 88 | 0.21 | 1.45 | 0.026 | 0.077 | 0.917 | 1020 | tags=53%, list=34%, signal=78% |
| 97 | TABULA\_MURIS\_SENIS\_AORTA\_FIBROBLAST\_OF\_CARDIAC\_TISSUE\_AGEING |  | 170 | 0.19 | 1.44 | 0.008 | 0.078 | 0.920 | 900 | tags=42%, list=30%, signal=56% |
| 98 | TABULA\_MURIS\_SENIS\_KIDNEY\_BRUSH\_CELL\_AGEING |  | 18 | 0.34 | 1.44 | 0.083 | 0.078 | 0.921 | 465 | tags=44%, list=15%, signal=52% |
| 99 | TABULA\_MURIS\_SENIS\_BROWN\_ADIPOSE\_TISSUE\_MESENCHYMAL\_STEM\_CELL\_OF\_ADIPOSE\_AGEING |  | 215 | 0.18 | 1.43 | 0.021 | 0.082 | 0.932 | 1077 | tags=49%, list=35%, signal=70% |
| 100 | TABULA\_MURIS\_SENIS\_SPLEEN\_MEGAKARYOCYTE\_ERYTHROID\_PROGENITOR\_CELL\_AGEING |  | 16 | 0.33 | 1.33 | 0.149 | 0.136 | 0.988 | 667 | tags=50%, list=22%, signal=64% |
| 101 | TABULA\_MURIS\_SENIS\_BRAIN\_NON\_MYELOID\_BRAIN\_PERICYTE\_AGEING |  | 142 | 0.17 | 1.29 | 0.105 | 0.168 | 0.996 | 1025 | tags=49%, list=34%, signal=70% |
| 102 | TABULA\_MURIS\_SENIS\_PANCREAS\_LEUKOCYTE\_AGEING |  | 16 | 0.32 | 1.27 | 0.171 | 0.179 | 0.998 | 973 | tags=75%, list=32%, signal=110% |
| 103 | TABULA\_MURIS\_SENIS\_SPLEEN\_NK\_CELL\_AGEING |  | 16 | 0.30 | 1.24 | 0.189 | 0.203 | 1.000 | 489 | tags=38%, list=16%, signal=44% |
| 104 | TABULA\_MURIS\_SENIS\_MARROW\_HEMATOPOIETIC\_STEM\_CELL\_AGEING |  | 147 | 0.17 | 1.24 | 0.098 | 0.204 | 1.000 | 888 | tags=38%, list=29%, signal=51% |
| 105 | TABULA\_MURIS\_SENIS\_MARROW\_MATURE\_ALPHA\_BETA\_T\_CELL\_AGEING |  | 101 | 0.17 | 1.23 | 0.101 | 0.212 | 1.000 | 503 | tags=24%, list=17%, signal=28% |
| 106 | TABULA\_MURIS\_SENIS\_LIVER\_HEPATOCYTE\_AGEING |  | 34 | 0.23 | 1.22 | 0.197 | 0.221 | 1.000 | 388 | tags=26%, list=13%, signal=30% |
| 107 | TABULA\_MURIS\_SENIS\_DIAPHRAGM\_SKELETAL\_MUSCLE\_SATELLITE\_CELL\_AGEING |  | 29 | 0.25 | 1.22 | 0.184 | 0.222 | 1.000 | 1006 | tags=55%, list=33%, signal=82% |
| 108 | ZHANG\_UTERUS\_C15\_B\_CELL |  | 18 | 0.28 | 1.20 | 0.249 | 0.238 | 1.000 | 380 | tags=33%, list=13%, signal=38% |
| 109 | TABULA\_MURIS\_SENIS\_KIDNEY\_KIDNEY\_DISTAL\_CONVOLUTED\_TUBULE\_EPITHELIAL\_CELL\_AGEING |  | 26 | 0.24 | 1.18 | 0.224 | 0.254 | 1.000 | 402 | tags=27%, list=13%, signal=31% |
| 110 | TABULA\_MURIS\_SENIS\_TRACHEA\_FIBROBLAST\_AGEING |  | 33 | 0.22 | 1.13 | 0.274 | 0.322 | 1.000 | 865 | tags=48%, list=28%, signal=67% |
| 111 | TABULA\_MURIS\_SENIS\_BRAIN\_NON\_MYELOID\_NEURON\_AGEING |  | 188 | 0.14 | 1.12 | 0.202 | 0.323 | 1.000 | 818 | tags=35%, list=27%, signal=45% |
| 112 | TABULA\_MURIS\_SENIS\_KIDNEY\_KIDNEY\_LOOP\_OF\_HENLE\_THICK\_ASCENDING\_LIMB\_EPITHELIAL\_CELL\_AGEING |  | 60 | 0.18 | 1.12 | 0.293 | 0.328 | 1.000 | 503 | tags=27%, list=17%, signal=31% |
| 113 | TABULA\_MURIS\_SENIS\_BRAIN\_NON\_MYELOID\_ASTROCYTE\_AGEING |  | 108 | 0.16 | 1.11 | 0.269 | 0.344 | 1.000 | 1077 | tags=54%, list=35%, signal=80% |
| 114 | TABULA\_MURIS\_SENIS\_MAMMARY\_GLAND\_BASAL\_CELL\_AGEING |  | 109 | 0.15 | 1.06 | 0.359 | 0.404 | 1.000 | 870 | tags=41%, list=29%, signal=56% |
| 115 | TABULA\_MURIS\_SENIS\_PANCREAS\_PANCREATIC\_BETA\_CELL\_AGEING |  | 48 | 0.18 | 1.04 | 0.380 | 0.428 | 1.000 | 856 | tags=42%, list=28%, signal=57% |
| 116 | TABULA\_MURIS\_SENIS\_KIDNEY\_T\_CELL\_AGEING |  | 20 | 0.25 | 1.03 | 0.383 | 0.451 | 1.000 | 860 | tags=50%, list=28%, signal=69% |
| 117 | TABULA\_MURIS\_SENIS\_PANCREAS\_PANCREATIC\_ALPHA\_CELL\_AGEING |  | 36 | 0.20 | 1.03 | 0.431 | 0.450 | 1.000 | 569 | tags=28%, list=19%, signal=34% |
| 118 | TABULA\_MURIS\_SENIS\_BRAIN\_NON\_MYELOID\_OLIGODENDROCYTE\_AGEING |  | 174 | 0.13 | 1.02 | 0.410 | 0.455 | 1.000 | 2169 | tags=89%, list=71%, signal=291% |
| 119 | DESCARTES\_ORGANOGENESIS\_MEGAKARYOCYTES |  | 35 | 0.19 | 1.01 | 0.460 | 0.467 | 1.000 | 626 | tags=34%, list=21%, signal=43% |
| 120 | TABULA\_MURIS\_SENIS\_SPLEEN\_PROERYTHROBLAST\_AGEING |  | 132 | 0.13 | 1.00 | 0.474 | 0.488 | 1.000 | 2396 | tags=95%, list=79%, signal=431% |
| 121 | TABULA\_MURIS\_SENIS\_TRACHEA\_T\_CELL\_AGEING |  | 68 | 0.16 | 0.98 | 0.449 | 0.511 | 1.000 | 973 | tags=41%, list=32%, signal=59% |
| 122 | TABULA\_MURIS\_SENIS\_BLADDER\_BLADDER\_CELL\_AGEING |  | 85 | 0.14 | 0.95 | 0.560 | 0.553 | 1.000 | 1297 | tags=62%, list=43%, signal=106% |
| 123 | TABULA\_MURIS\_SENIS\_SKIN\_BULGE\_KERATINOCYTE\_AGEING |  | 183 | 0.12 | 0.95 | 0.563 | 0.561 | 1.000 | 567 | tags=24%, list=19%, signal=28% |
| 124 | TABULA\_MURIS\_SENIS\_MARROW\_LATE\_PRO\_B\_CELL\_AGEING |  | 40 | 0.17 | 0.94 | 0.570 | 0.572 | 1.000 | 1087 | tags=52%, list=36%, signal=81% |
| 125 | TABULA\_MURIS\_SENIS\_THYMUS\_THYMOCYTE\_AGEING |  | 81 | 0.14 | 0.92 | 0.618 | 0.588 | 1.000 | 2508 | tags=98%, list=83%, signal=542% |
| 126 | TABULA\_MURIS\_SENIS\_HEART\_AND\_AORTA\_SMOOTH\_MUSCLE\_CELL\_AGEING |  | 19 | 0.17 | 0.72 | 0.816 | 0.868 | 1.000 | 820 | tags=42%, list=27%, signal=57% |
Table: Gene sets enriched in phenotype **na**[plain text format]****

  
