# Supplementary material for: TMPRSS11B promotes an acidified microenvironment and immune suppression in squamous lung cancer
Source: EMBO Rep. 2025 Nov 10;26(24):6346–79. doi: 10.1038/s44319-025-00631-1 (PMC12714794; doi:10.1038/s44319-025-00631-1)
Supplement: Supplementary file 11 — Source data Fig. 6 [file 44319_2025_631_MOESM11_ESM.zip › Figure 6/6D-E/GSEA Broad Institute_low pH vs rest of the regions (high pH)/index.html]

Index for xtools.gsea.GseaPreranked my\_analysis.GseaPreranked.1723659072606

### GSEA Report for Dataset Lactate high vs low\_Ranked

#### Enrichment in phenotype: **na**

- 126 / 161 gene sets are upregulated in phenotype **na\_pos**- 108 gene sets are significant at FDR < 25%- 81 gene sets are significantly enriched at nominal pvalue < 1%- 96 gene sets are significantly enriched at nominal pvalue < 5%- Snapshot of enrichment results- Detailed enrichment results in html format- Detailed enrichment results in TSV format (tab delimited text)- Guide to interpret results

#### Enrichment in phenotype: **na**

- 35 / 161 gene sets are upregulated in phenotype **na\_neg**- 10 gene sets are significantly enriched at FDR < 25%- 8 gene sets are significantly enriched at nominal pvalue < 1%- 9 gene sets are significantly enriched at nominal pvalue < 5%- Snapshot of enrichment results- Detailed enrichment results in html format- Detailed enrichment results in TSV format (tab delimited text)- Guide to interpret results

#### Dataset details

- The dataset has 3040 features (genes)- No probe set => gene symbol collapsing was requested, so all 3040 features were used

#### Gene set details

- Gene set size filters (min=15, max=500) resulted in filtering out 72 / 233 gene sets- The remaining 161 gene sets were used in the analysis- List of gene sets used and their sizes (restricted to features in the specified dataset)

#### Gene markers for the **na\_pos** *versus* **na\_neg** comparison

- The dataset has 3040 features (genes)- Detailed rank ordered gene list for all features in the dataset

#### Global statistics and plots

- Plot of p-values *vs.* NES- Global ES histogram

#### Other

- Parameters used for this analysis

#### Comments

- Timestamp used as the random seed: 1723659072606

#### Citing GSEA and MSigDB

To cite your use of the GSEA software please reference the following:

- Subramanian, A., Tamayo, P., et al. (2005, PNAS). - Mootha, V. K., Lindgren, C. M., et al. (2003, Nature Genetics).

For use of the Molecular Signatures Database (MSigDB), to cite please reference   
one or more of the following as appropriate, along with the source for the gene set as listed on the gene set page:

- Liberzon A, et al. (Bioinformatics, 2011). - Liberzon A, et al. (Cell Systems 2015).

---

Report: my\_analysis.GseaPreranked.1723659072606.rpt   by user: S434287

xtools.gsea.GseaPreranked [Wed, Aug 14, '24 1 PM 11]

Website: www.gsea-msigdb.org/gsea
Questions & Suggestions: Contact page
