# Supplementary material for: TMPRSS11B promotes an acidified microenvironment and immune suppression in squamous lung cancer
Source: EMBO Rep. 2025 Nov 10;26(24):6346–79. doi: 10.1038/s44319-025-00631-1 (PMC12714794; doi:10.1038/s44319-025-00631-1)
Supplement: Supplementary file 11 — Source data Fig. 6 [file 44319_2025_631_MOESM11_ESM.zip › Figure 6/6D-E/GSEA Broad Institute_low pH vs rest of the regions (high pH)/TABULA_MURIS_SENIS_BLADDER_BLADDER_UROTHELIAL_CELL_AGEING.html]

Details for gene set TABULA\_MURIS\_SENIS\_BLADDER\_BLADDER\_UROTHELIAL\_CELL\_AGEING[GSEA]

|  || Dataset | Lactate high vs low\_Ranked |
| Phenotype | NoPhenotypeAvailable |
| Upregulated in class | na\_neg |
| GeneSet | TABULA\_MURIS\_SENIS\_BLADDER\_BLADDER\_UROTHELIAL\_CELL\_AGEING |
| Enrichment Score (ES) | -0.2271744 |
| Normalized Enrichment Score (NES) | -1.0835311 |
| Nominal p-value | 0.34816754 |
| FDR q-value | 0.6237359 |
| FWER p-Value | 1.0 |
Table: GSEA Results Summary

  

Fig 1: Enrichment plot: TABULA\_MURIS\_SENIS\_BLADDER\_BLADDER\_UROTHELIAL\_CELL\_AGEING      
 Profile of the Running ES Score & Positions of GeneSet Members on the Rank Ordered List

  

| SYMBOL | RANK IN GENE LIST | RANK METRIC SCORE | RUNNING ES | CORE ENRICHMENT || 1 | Gpnmb | 3 | 2.213 | 0.0355 | No |
| 2 | Hmox1 | 16 | 2.051 | 0.0653 | No |
| 3 | Ctsb | 46 | 1.778 | 0.0849 | No |
| 4 | Nrn1 | 68 | 1.672 | 0.1055 | No |
| 5 | Plin2 | 90 | 1.607 | 0.1250 | No |
| 6 | Mfge8 | 118 | 1.535 | 0.1412 | No |
| 7 | Lgals3 | 344 | 1.170 | 0.0852 | No |
| 8 | B2m | 402 | 1.097 | 0.0842 | No |
| 9 | Fth1 | 503 | 0.986 | 0.0669 | No |
| 10 | Creg1 | 517 | 0.973 | 0.0786 | No |
| 11 | Cyba | 554 | 0.947 | 0.0822 | No |
| 12 | H2-D1 | 722 | 0.794 | 0.0393 | No |
| 13 | Cdkn1a | 749 | 0.765 | 0.0433 | No |
| 14 | H2-K1 | 818 | 0.692 | 0.0319 | No |
| 15 | Plpp3 | 938 | 0.605 | 0.0020 | No |
| 16 | Cfl1 | 973 | 0.581 | 0.0002 | No |
| 17 | Cstb | 1056 | 0.535 | -0.0184 | No |
| 18 | Plaur | 1098 | 0.504 | -0.0239 | No |
| 19 | Sdcbp2 | 1160 | -0.511 | -0.0359 | No |
| 20 | Krt8 | 1193 | -0.519 | -0.0380 | No |
| 21 | Eif3f | 1225 | -0.527 | -0.0397 | No |
| 22 | Hmgcr | 1281 | -0.538 | -0.0493 | No |
| 23 | Msmo1 | 1304 | -0.541 | -0.0477 | No |
| 24 | Rac1 | 1404 | -0.564 | -0.0716 | No |
| 25 | Eif6 | 1550 | -0.601 | -0.1102 | No |
| 26 | Bcl3 | 1709 | -0.657 | -0.1523 | No |
| 27 | Ppa1 | 1719 | -0.661 | -0.1445 | No |
| 28 | Eef1d | 1738 | -0.668 | -0.1395 | No |
| 29 | S100a16 | 1801 | -0.687 | -0.1489 | No |
| 30 | Nop56 | 1822 | -0.696 | -0.1441 | No |
| 31 | Aldh3a1 | 1866 | -0.710 | -0.1468 | No |
| 32 | Fdft1 | 1952 | -0.741 | -0.1631 | No |
| 33 | Hsp90aa1 | 1965 | -0.745 | -0.1548 | No |
| 34 | Xbp1 | 1976 | -0.750 | -0.1458 | No |
| 35 | Cbr3 | 2160 | -0.834 | -0.1933 | No |
| 36 | Lmo4 | 2166 | -0.842 | -0.1811 | No |
| 37 | Phlda1 | 2291 | -0.920 | -0.2075 | No |
| 38 | Cdc42ep5 | 2317 | -0.935 | -0.2004 | No |
| 39 | Vps37b | 2349 | -0.962 | -0.1949 | No |
| 40 | Smox | 2377 | -0.990 | -0.1876 | No |
| 41 | Srsf3 | 2496 | -1.090 | -0.2092 | Yes |
| 42 | Avpi1 | 2499 | -1.093 | -0.1918 | Yes |
| 43 | S100a14 | 2580 | -1.185 | -0.1991 | Yes |
| 44 | Gpx2 | 2598 | -1.211 | -0.1848 | Yes |
| 45 | Cldn7 | 2642 | -1.269 | -0.1783 | Yes |
| 46 | Urah | 2658 | -1.303 | -0.1618 | Yes |
| 47 | Krt23 | 2683 | -1.340 | -0.1477 | Yes |
| 48 | Asns | 2703 | -1.371 | -0.1315 | Yes |
| 49 | Areg | 2775 | -1.538 | -0.1299 | Yes |
| 50 | Aqp3 | 2802 | -1.594 | -0.1123 | Yes |
| 51 | Klf5 | 2823 | -1.644 | -0.0919 | Yes |
| 52 | Sprr1a | 2841 | -1.716 | -0.0693 | Yes |
| 53 | Cbr2 | 2865 | -1.827 | -0.0468 | Yes |
| 54 | Prss22 | 3002 | -3.154 | -0.0404 | Yes |
| 55 | Lypd3 | 3005 | -3.177 | 0.0114 | Yes |
Table: GSEA details [plain text format]

  

Fig 2: TABULA\_MURIS\_SENIS\_BLADDER\_BLADDER\_UROTHELIAL\_CELL\_AGEING: Random ES distribution      
 Gene set null distribution of ES for **TABULA\_MURIS\_SENIS\_BLADDER\_BLADDER\_UROTHELIAL\_CELL\_AGEING**

  
