# Supplementary material for: TMPRSS11B promotes an acidified microenvironment and immune suppression in squamous lung cancer
Source: EMBO Rep. 2025 Nov 10;26(24):6346–79. doi: 10.1038/s44319-025-00631-1 (PMC12714794; doi:10.1038/s44319-025-00631-1)
Supplement: Supplementary file 11 — Source data Fig. 6 [file 44319_2025_631_MOESM11_ESM.zip › Figure 6/6D-E/GSEA Broad Institute_low pH vs rest of the regions (high pH)/TABULA_MURIS_SENIS_BRAIN_MYELOID_MICROGLIAL_CELL_AGEING.html]

Details for gene set TABULA\_MURIS\_SENIS\_BRAIN\_MYELOID\_MICROGLIAL\_CELL\_AGEING[GSEA]

|  || Dataset | Lactate high vs low\_Ranked |
| Phenotype | NoPhenotypeAvailable |
| Upregulated in class | na\_pos |
| GeneSet | TABULA\_MURIS\_SENIS\_BRAIN\_MYELOID\_MICROGLIAL\_CELL\_AGEING |
| Enrichment Score (ES) | 0.5088713 |
| Normalized Enrichment Score (NES) | 3.5570679 |
| Nominal p-value | 0.0 |
| FDR q-value | 0.0 |
| FWER p-Value | 0.0 |
Table: GSEA Results Summary

  

Fig 1: Enrichment plot: TABULA\_MURIS\_SENIS\_BRAIN\_MYELOID\_MICROGLIAL\_CELL\_AGEING      
 Profile of the Running ES Score & Positions of GeneSet Members on the Rank Ordered List

  

| SYMBOL | RANK IN GENE LIST | RANK METRIC SCORE | RUNNING ES | CORE ENRICHMENT || 1 | C1qb | 2 | 2.291 | 0.0191 | Yes |
| 2 | Apoe | 6 | 2.177 | 0.0368 | Yes |
| 3 | Ctsl | 8 | 2.153 | 0.0551 | Yes |
| 4 | Fabp4 | 12 | 2.100 | 0.0722 | Yes |
| 5 | Ctss | 14 | 2.088 | 0.0898 | Yes |
| 6 | Trem2 | 15 | 2.053 | 0.1075 | Yes |
| 7 | C1qc | 20 | 1.988 | 0.1233 | Yes |
| 8 | Cd300c2 | 26 | 1.896 | 0.1380 | Yes |
| 9 | C1qa | 29 | 1.862 | 0.1534 | Yes |
| 10 | Lpl | 34 | 1.833 | 0.1678 | Yes |
| 11 | Cd68 | 36 | 1.820 | 0.1832 | Yes |
| 12 | Ctsb | 46 | 1.778 | 0.1954 | Yes |
| 13 | Cd37 | 49 | 1.763 | 0.2100 | Yes |
| 14 | Lat2 | 64 | 1.685 | 0.2197 | Yes |
| 15 | Tyrobp | 83 | 1.618 | 0.2276 | Yes |
| 16 | Spi1 | 86 | 1.616 | 0.2408 | Yes |
| 17 | Plekho1 | 92 | 1.606 | 0.2530 | Yes |
| 18 | Fcer1g | 95 | 1.597 | 0.2661 | Yes |
| 19 | Fcgr2b | 102 | 1.572 | 0.2776 | Yes |
| 20 | Vim | 128 | 1.521 | 0.2822 | Yes |
| 21 | Csf2ra | 143 | 1.478 | 0.2902 | Yes |
| 22 | Cd48 | 146 | 1.476 | 0.3022 | Yes |
| 23 | Gpsm3 | 194 | 1.384 | 0.2982 | Yes |
| 24 | Npc2 | 202 | 1.371 | 0.3076 | Yes |
| 25 | Hebp1 | 224 | 1.345 | 0.3120 | Yes |
| 26 | Cd52 | 233 | 1.323 | 0.3207 | Yes |
| 27 | Tmem86a | 236 | 1.319 | 0.3314 | Yes |
| 28 | Ctsz | 242 | 1.303 | 0.3410 | Yes |
| 29 | Arhgap45 | 243 | 1.301 | 0.3522 | Yes |
| 30 | Tmem100 | 258 | 1.277 | 0.3584 | Yes |
| 31 | Dpysl2 | 298 | 1.225 | 0.3557 | Yes |
| 32 | Timp2 | 334 | 1.180 | 0.3540 | Yes |
| 33 | Abi3 | 339 | 1.175 | 0.3628 | Yes |
| 34 | Crlf2 | 352 | 1.164 | 0.3687 | Yes |
| 35 | Cd74 | 376 | 1.133 | 0.3707 | Yes |
| 36 | B2m | 402 | 1.097 | 0.3716 | Yes |
| 37 | H2-Ab1 | 404 | 1.094 | 0.3807 | Yes |
| 38 | H2-DMa | 418 | 1.083 | 0.3856 | Yes |
| 39 | H2-Eb1 | 425 | 1.075 | 0.3928 | Yes |
| 40 | Arhgdib | 445 | 1.049 | 0.3954 | Yes |
| 41 | Cotl1 | 447 | 1.049 | 0.4041 | Yes |
| 42 | Lsp1 | 459 | 1.039 | 0.4093 | Yes |
| 43 | Sparc | 460 | 1.038 | 0.4183 | Yes |
| 44 | H2-Aa | 465 | 1.035 | 0.4259 | Yes |
| 45 | Cxcl16 | 479 | 1.013 | 0.4302 | Yes |
| 46 | Tubb6 | 480 | 1.013 | 0.4389 | Yes |
| 47 | Tspan7 | 487 | 1.004 | 0.4455 | Yes |
| 48 | Fth1 | 503 | 0.986 | 0.4489 | Yes |
| 49 | Serping1 | 519 | 0.973 | 0.4522 | Yes |
| 50 | Coro1a | 522 | 0.970 | 0.4599 | Yes |
| 51 | Cyba | 554 | 0.947 | 0.4575 | Yes |
| 52 | Trim35 | 583 | 0.914 | 0.4559 | Yes |
| 53 | Gpx1 | 603 | 0.888 | 0.4571 | Yes |
| 54 | Tspan4 | 625 | 0.870 | 0.4574 | Yes |
| 55 | Irf8 | 663 | 0.840 | 0.4521 | Yes |
| 56 | Slc43a3 | 675 | 0.830 | 0.4555 | Yes |
| 57 | Arrb2 | 718 | 0.796 | 0.4480 | Yes |
| 58 | H2-D1 | 722 | 0.794 | 0.4539 | Yes |
| 59 | Cd63 | 724 | 0.793 | 0.4604 | Yes |
| 60 | Grina | 726 | 0.791 | 0.4669 | Yes |
| 61 | Ppp1r18 | 732 | 0.776 | 0.4718 | Yes |
| 62 | Bst2 | 736 | 0.772 | 0.4775 | Yes |
| 63 | Anxa5 | 743 | 0.769 | 0.4821 | Yes |
| 64 | Cdkn1a | 749 | 0.765 | 0.4870 | Yes |
| 65 | Cst3 | 782 | 0.723 | 0.4823 | Yes |
| 66 | Ddah2 | 800 | 0.706 | 0.4826 | Yes |
| 67 | H2-K1 | 818 | 0.692 | 0.4828 | Yes |
| 68 | Rgs10 | 837 | 0.678 | 0.4825 | Yes |
| 69 | Psmb8 | 838 | 0.678 | 0.4884 | Yes |
| 70 | Calm2 | 857 | 0.664 | 0.4880 | Yes |
| 71 | Bgn | 870 | 0.650 | 0.4895 | Yes |
| 72 | Atp6v0e | 888 | 0.638 | 0.4892 | Yes |
| 73 | Ctsh | 890 | 0.637 | 0.4944 | Yes |
| 74 | Mgp | 898 | 0.630 | 0.4974 | Yes |
| 75 | Syngr1 | 911 | 0.622 | 0.4987 | Yes |
| 76 | H2-M3 | 955 | 0.598 | 0.4892 | Yes |
| 77 | Atp6v0c | 960 | 0.595 | 0.4930 | Yes |
| 78 | Cfl1 | 973 | 0.581 | 0.4939 | Yes |
| 79 | Dok1 | 977 | 0.577 | 0.4979 | Yes |
| 80 | Camk1 | 985 | 0.574 | 0.5004 | Yes |
| 81 | Arl8a | 992 | 0.568 | 0.5033 | Yes |
| 82 | Erp29 | 1003 | 0.562 | 0.5047 | Yes |
| 83 | Sh3bgrl3 | 1006 | 0.560 | 0.5089 | Yes |
| 84 | Gpx3 | 1070 | 0.527 | 0.4920 | No |
| 85 | Rack1 | 1195 | -0.519 | 0.4542 | No |
| 86 | Selenos | 1201 | -0.520 | 0.4570 | No |
| 87 | Eif3f | 1225 | -0.527 | 0.4537 | No |
| 88 | Eef1b2 | 1352 | -0.553 | 0.4156 | No |
| 89 | Zfp710 | 1427 | -0.571 | 0.3953 | No |
| 90 | Mbp | 1464 | -0.577 | 0.3880 | No |
| 91 | Bsg | 1560 | -0.604 | 0.3609 | No |
| 92 | Rbm26 | 1633 | -0.628 | 0.3418 | No |
| 93 | Eef1d | 1738 | -0.668 | 0.3121 | No |
| 94 | Tmed3 | 1790 | -0.685 | 0.3007 | No |
| 95 | Slamf9 | 1929 | -0.731 | 0.2600 | No |
| 96 | Basp1 | 2191 | -0.855 | 0.1785 | No |
| 97 | Ly6a | 2366 | -0.979 | 0.1277 | No |
| 98 | Ly6e | 2392 | -1.001 | 0.1278 | No |
| 99 | Dcn | 2701 | -1.362 | 0.0347 | No |
| 100 | Clu | 2876 | -1.874 | -0.0084 | No |
| 101 | Upk1b | 2917 | -2.131 | -0.0036 | No |
| 102 | Krt15 | 2935 | -2.256 | 0.0101 | No |
| 103 | Krt14 | 2985 | -2.898 | 0.0184 | No |
Table: GSEA details [plain text format]

  

Fig 2: TABULA\_MURIS\_SENIS\_BRAIN\_MYELOID\_MICROGLIAL\_CELL\_AGEING: Random ES distribution      
 Gene set null distribution of ES for **TABULA\_MURIS\_SENIS\_BRAIN\_MYELOID\_MICROGLIAL\_CELL\_AGEING**

  
