# Supplementary material for: TMPRSS11B promotes an acidified microenvironment and immune suppression in squamous lung cancer
Source: EMBO Rep. 2025 Nov 10;26(24):6346–79. doi: 10.1038/s44319-025-00631-1 (PMC12714794; doi:10.1038/s44319-025-00631-1)
Supplement: Supplementary file 11 — Source data Fig. 6 [file 44319_2025_631_MOESM11_ESM.zip › Figure 6/6D-E/GSEA Broad Institute_low pH vs rest of the regions (high pH)/TABULA_MURIS_SENIS_BROWN_ADIPOSE_TISSUE_B_CELL_AGEING.html]

Details for gene set TABULA\_MURIS\_SENIS\_BROWN\_ADIPOSE\_TISSUE\_B\_CELL\_AGEING[GSEA]

|  || Dataset | Lactate high vs low\_Ranked |
| Phenotype | NoPhenotypeAvailable |
| Upregulated in class | na\_pos |
| GeneSet | TABULA\_MURIS\_SENIS\_BROWN\_ADIPOSE\_TISSUE\_B\_CELL\_AGEING |
| Enrichment Score (ES) | 0.5109468 |
| Normalized Enrichment Score (NES) | 3.4761415 |
| Nominal p-value | 0.0 |
| FDR q-value | 0.0 |
| FWER p-Value | 0.0 |
Table: GSEA Results Summary

  

Fig 1: Enrichment plot: TABULA\_MURIS\_SENIS\_BROWN\_ADIPOSE\_TISSUE\_B\_CELL\_AGEING      
 Profile of the Running ES Score & Positions of GeneSet Members on the Rank Ordered List

  

| SYMBOL | RANK IN GENE LIST | RANK METRIC SCORE | RUNNING ES | CORE ENRICHMENT || 1 | C1qb | 2 | 2.291 | 0.0245 | Yes |
| 2 | Apoe | 6 | 2.177 | 0.0474 | Yes |
| 3 | Itm2a | 7 | 2.155 | 0.0711 | Yes |
| 4 | Ctsl | 8 | 2.153 | 0.0948 | Yes |
| 5 | Fabp4 | 12 | 2.100 | 0.1168 | Yes |
| 6 | Cd68 | 36 | 1.820 | 0.1291 | Yes |
| 7 | Ctsb | 46 | 1.778 | 0.1456 | Yes |
| 8 | Cd36 | 52 | 1.748 | 0.1631 | Yes |
| 9 | Psap | 59 | 1.694 | 0.1797 | Yes |
| 10 | Plekho1 | 92 | 1.606 | 0.1865 | Yes |
| 11 | Fcer1g | 95 | 1.597 | 0.2034 | Yes |
| 12 | Cav1 | 107 | 1.564 | 0.2169 | Yes |
| 13 | Mfge8 | 118 | 1.535 | 0.2304 | Yes |
| 14 | Lair1 | 127 | 1.521 | 0.2444 | Yes |
| 15 | Vim | 128 | 1.521 | 0.2611 | Yes |
| 16 | Pltp | 133 | 1.513 | 0.2764 | Yes |
| 17 | Ctsd | 136 | 1.507 | 0.2923 | Yes |
| 18 | Vsir | 170 | 1.421 | 0.2967 | Yes |
| 19 | Hexb | 197 | 1.379 | 0.3031 | Yes |
| 20 | Ctsz | 242 | 1.303 | 0.3025 | Yes |
| 21 | Man2b1 | 305 | 1.216 | 0.2949 | Yes |
| 22 | Thbd | 306 | 1.215 | 0.3083 | Yes |
| 23 | Mgll | 316 | 1.208 | 0.3185 | Yes |
| 24 | Apbb1ip | 322 | 1.197 | 0.3299 | Yes |
| 25 | Abhd12 | 330 | 1.182 | 0.3406 | Yes |
| 26 | Selplg | 332 | 1.182 | 0.3532 | Yes |
| 27 | Timp2 | 334 | 1.180 | 0.3659 | Yes |
| 28 | Abi3 | 339 | 1.175 | 0.3774 | Yes |
| 29 | Lgals3 | 344 | 1.170 | 0.3889 | Yes |
| 30 | Crlf2 | 352 | 1.164 | 0.3993 | Yes |
| 31 | Grn | 365 | 1.146 | 0.4079 | Yes |
| 32 | B2m | 402 | 1.097 | 0.4078 | Yes |
| 33 | Klf2 | 412 | 1.087 | 0.4167 | Yes |
| 34 | Cotl1 | 447 | 1.049 | 0.4167 | Yes |
| 35 | Tubb6 | 480 | 1.013 | 0.4170 | Yes |
| 36 | Icam1 | 492 | 0.999 | 0.4243 | Yes |
| 37 | Plod1 | 497 | 0.995 | 0.4339 | Yes |
| 38 | Tie1 | 504 | 0.985 | 0.4427 | Yes |
| 39 | Serping1 | 519 | 0.973 | 0.4486 | Yes |
| 40 | Cyba | 554 | 0.947 | 0.4475 | Yes |
| 41 | Trim35 | 583 | 0.914 | 0.4481 | Yes |
| 42 | Ehd2 | 597 | 0.893 | 0.4535 | Yes |
| 43 | Sipa1 | 611 | 0.880 | 0.4588 | Yes |
| 44 | Tgfbi | 651 | 0.851 | 0.4549 | Yes |
| 45 | Irf8 | 663 | 0.840 | 0.4605 | Yes |
| 46 | Slc43a3 | 675 | 0.830 | 0.4659 | Yes |
| 47 | Dhrs3 | 703 | 0.807 | 0.4656 | Yes |
| 48 | Bst2 | 736 | 0.772 | 0.4633 | Yes |
| 49 | Ifngr1 | 740 | 0.770 | 0.4707 | Yes |
| 50 | Anxa5 | 743 | 0.769 | 0.4785 | Yes |
| 51 | Cdkn1a | 749 | 0.765 | 0.4852 | Yes |
| 52 | Cst3 | 782 | 0.723 | 0.4823 | Yes |
| 53 | Tln1 | 804 | 0.703 | 0.4830 | Yes |
| 54 | Grb2 | 816 | 0.695 | 0.4869 | Yes |
| 55 | H2-K1 | 818 | 0.692 | 0.4941 | Yes |
| 56 | Prnp | 820 | 0.690 | 0.5014 | Yes |
| 57 | Epb41l2 | 827 | 0.683 | 0.5069 | Yes |
| 58 | Psmb8 | 838 | 0.678 | 0.5109 | Yes |
| 59 | Ctsh | 890 | 0.637 | 0.5007 | No |
| 60 | Slc48a1 | 916 | 0.618 | 0.4990 | No |
| 61 | Plpp3 | 938 | 0.605 | 0.4986 | No |
| 62 | Serpine2 | 967 | 0.590 | 0.4956 | No |
| 63 | Cfl1 | 973 | 0.581 | 0.5003 | No |
| 64 | Tppp3 | 1219 | -0.525 | 0.4232 | No |
| 65 | Senp6 | 1364 | -0.556 | 0.3806 | No |
| 66 | Pmaip1 | 1451 | -0.575 | 0.3578 | No |
| 67 | Mbp | 1464 | -0.577 | 0.3601 | No |
| 68 | Cnn3 | 1470 | -0.579 | 0.3648 | No |
| 69 | Mt2 | 1478 | -0.581 | 0.3688 | No |
| 70 | Bsg | 1560 | -0.604 | 0.3480 | No |
| 71 | Tmem59 | 1563 | -0.605 | 0.3540 | No |
| 72 | Tmed3 | 1790 | -0.685 | 0.2851 | No |
| 73 | Pttg1ip | 1884 | -0.716 | 0.2615 | No |
| 74 | Atp1a1 | 1953 | -0.741 | 0.2466 | No |
| 75 | Xbp1 | 1976 | -0.750 | 0.2474 | No |
| 76 | Ccdc88c | 2115 | -0.814 | 0.2097 | No |
| 77 | Dnajc3 | 2133 | -0.820 | 0.2129 | No |
| 78 | Ly6a | 2366 | -0.979 | 0.1452 | No |
| 79 | Psip1 | 2410 | -1.012 | 0.1418 | No |
| 80 | Myo6 | 2548 | -1.149 | 0.1081 | No |
| 81 | Igfbp5 | 2556 | -1.159 | 0.1185 | No |
| 82 | Fahd1 | 2588 | -1.195 | 0.1211 | No |
| 83 | Dcn | 2701 | -1.362 | 0.0982 | No |
| 84 | Egr1 | 2733 | -1.440 | 0.1035 | No |
Table: GSEA details [plain text format]

  

Fig 2: TABULA\_MURIS\_SENIS\_BROWN\_ADIPOSE\_TISSUE\_B\_CELL\_AGEING: Random ES distribution      
 Gene set null distribution of ES for **TABULA\_MURIS\_SENIS\_BROWN\_ADIPOSE\_TISSUE\_B\_CELL\_AGEING**

  
