# Supplementary material for: TMPRSS11B promotes an acidified microenvironment and immune suppression in squamous lung cancer
Source: EMBO Rep. 2025 Nov 10;26(24):6346–79. doi: 10.1038/s44319-025-00631-1 (PMC12714794; doi:10.1038/s44319-025-00631-1)
Supplement: Supplementary file 11 — Source data Fig. 6 [file 44319_2025_631_MOESM11_ESM.zip › Figure 6/6D-E/GSEA Broad Institute_low pH vs rest of the regions (high pH)/TABULA_MURIS_SENIS_BROWN_ADIPOSE_TISSUE_ENDOTHELIAL_CELL_AGEING.html]

Details for gene set TABULA\_MURIS\_SENIS\_BROWN\_ADIPOSE\_TISSUE\_ENDOTHELIAL\_CELL\_AGEING[GSEA]

|  || Dataset | Lactate high vs low\_Ranked |
| Phenotype | NoPhenotypeAvailable |
| Upregulated in class | na\_pos |
| GeneSet | TABULA\_MURIS\_SENIS\_BROWN\_ADIPOSE\_TISSUE\_ENDOTHELIAL\_CELL\_AGEING |
| Enrichment Score (ES) | 0.47514853 |
| Normalized Enrichment Score (NES) | 2.9136746 |
| Nominal p-value | 0.0 |
| FDR q-value | 0.0 |
| FWER p-Value | 0.0 |
Table: GSEA Results Summary

  

Fig 1: Enrichment plot: TABULA\_MURIS\_SENIS\_BROWN\_ADIPOSE\_TISSUE\_ENDOTHELIAL\_CELL\_AGEING      
 Profile of the Running ES Score & Positions of GeneSet Members on the Rank Ordered List

  

| SYMBOL | RANK IN GENE LIST | RANK METRIC SCORE | RUNNING ES | CORE ENRICHMENT || 1 | Apoe | 6 | 2.177 | 0.0339 | Yes |
| 2 | Itm2a | 7 | 2.155 | 0.0695 | Yes |
| 3 | Lgmn | 28 | 1.878 | 0.0937 | Yes |
| 4 | Ctsb | 46 | 1.778 | 0.1173 | Yes |
| 5 | Tnfsf12 | 115 | 1.544 | 0.1200 | Yes |
| 6 | Vim | 128 | 1.521 | 0.1411 | Yes |
| 7 | Pltp | 133 | 1.513 | 0.1647 | Yes |
| 8 | Plek | 135 | 1.509 | 0.1893 | Yes |
| 9 | Cryab | 158 | 1.449 | 0.2058 | Yes |
| 10 | Hexb | 197 | 1.379 | 0.2158 | Yes |
| 11 | Ptprm | 223 | 1.346 | 0.2296 | Yes |
| 12 | Cldn5 | 227 | 1.338 | 0.2506 | Yes |
| 13 | Dnmt3a | 239 | 1.310 | 0.2686 | Yes |
| 14 | Npr1 | 245 | 1.299 | 0.2883 | Yes |
| 15 | Csf1r | 249 | 1.292 | 0.3086 | Yes |
| 16 | Acer2 | 265 | 1.264 | 0.3245 | Yes |
| 17 | Thbd | 306 | 1.215 | 0.3311 | Yes |
| 18 | Abi3 | 339 | 1.175 | 0.3397 | Yes |
| 19 | Egfl7 | 406 | 1.093 | 0.3356 | Yes |
| 20 | Klf2 | 412 | 1.087 | 0.3518 | Yes |
| 21 | Prex1 | 435 | 1.066 | 0.3620 | Yes |
| 22 | Cdkn1c | 446 | 1.049 | 0.3760 | Yes |
| 23 | H2-Aa | 465 | 1.035 | 0.3870 | Yes |
| 24 | Plvap | 512 | 0.977 | 0.3877 | Yes |
| 25 | Trf | 541 | 0.957 | 0.3941 | Yes |
| 26 | Serpinh1 | 546 | 0.953 | 0.4085 | Yes |
| 27 | Laptm5 | 659 | 0.844 | 0.3848 | Yes |
| 28 | Ramp2 | 670 | 0.833 | 0.3952 | Yes |
| 29 | H2-D1 | 722 | 0.794 | 0.3911 | Yes |
| 30 | Cd63 | 724 | 0.793 | 0.4039 | Yes |
| 31 | Ifngr1 | 740 | 0.770 | 0.4116 | Yes |
| 32 | Hsd3b7 | 754 | 0.760 | 0.4198 | Yes |
| 33 | Sh3kbp1 | 762 | 0.752 | 0.4298 | Yes |
| 34 | Cst3 | 782 | 0.723 | 0.4354 | Yes |
| 35 | Grb2 | 816 | 0.695 | 0.4358 | Yes |
| 36 | H2-K1 | 818 | 0.692 | 0.4468 | Yes |
| 37 | Calm2 | 857 | 0.664 | 0.4450 | Yes |
| 38 | Igfbp7 | 860 | 0.655 | 0.4552 | Yes |
| 39 | Arhgef2 | 866 | 0.652 | 0.4642 | Yes |
| 40 | Mgp | 898 | 0.630 | 0.4642 | Yes |
| 41 | Ogn | 950 | 0.601 | 0.4570 | Yes |
| 42 | Atp6v0c | 960 | 0.595 | 0.4638 | Yes |
| 43 | Cfl1 | 973 | 0.581 | 0.4694 | Yes |
| 44 | Camk1 | 985 | 0.574 | 0.4751 | Yes |
| 45 | Hdac7 | 1031 | 0.548 | 0.4691 | No |
| 46 | Myl12a | 1087 | 0.516 | 0.4591 | No |
| 47 | Brd3 | 1118 | -0.503 | 0.4574 | No |
| 48 | Lgalsl | 1184 | -0.515 | 0.4440 | No |
| 49 | Ece1 | 1216 | -0.525 | 0.4423 | No |
| 50 | Dctn1 | 1291 | -0.539 | 0.4263 | No |
| 51 | Slc44a1 | 1403 | -0.564 | 0.3984 | No |
| 52 | Zbtb7a | 1424 | -0.569 | 0.4010 | No |
| 53 | Mt2 | 1478 | -0.581 | 0.3928 | No |
| 54 | Kmt2e | 1514 | -0.590 | 0.3908 | No |
| 55 | Ier2 | 1527 | -0.593 | 0.3965 | No |
| 56 | Tmem59 | 1563 | -0.605 | 0.3948 | No |
| 57 | Usp22 | 1740 | -0.668 | 0.3467 | No |
| 58 | Tmed3 | 1790 | -0.685 | 0.3415 | No |
| 59 | Cfap20 | 2145 | -0.825 | 0.2363 | No |
| 60 | Lmo4 | 2166 | -0.842 | 0.2434 | No |
| 61 | Atp9a | 2504 | -1.097 | 0.1484 | No |
| 62 | Clu | 2876 | -1.874 | 0.0547 | No |
Table: GSEA details [plain text format]

  

Fig 2: TABULA\_MURIS\_SENIS\_BROWN\_ADIPOSE\_TISSUE\_ENDOTHELIAL\_CELL\_AGEING: Random ES distribution      
 Gene set null distribution of ES for **TABULA\_MURIS\_SENIS\_BROWN\_ADIPOSE\_TISSUE\_ENDOTHELIAL\_CELL\_AGEING**

  
