# Supplementary material for: TMPRSS11B promotes an acidified microenvironment and immune suppression in squamous lung cancer
Source: EMBO Rep. 2025 Nov 10;26(24):6346–79. doi: 10.1038/s44319-025-00631-1 (PMC12714794; doi:10.1038/s44319-025-00631-1)
Supplement: Supplementary file 11 — Source data Fig. 6 [file 44319_2025_631_MOESM11_ESM.zip › Figure 6/6D-E/GSEA Broad Institute_low pH vs rest of the regions (high pH)/TABULA_MURIS_SENIS_GONADAL_ADIPOSE_TISSUE_B_CELL_AGEING.html]

Details for gene set TABULA\_MURIS\_SENIS\_GONADAL\_ADIPOSE\_TISSUE\_B\_CELL\_AGEING[GSEA]

|  || Dataset | Lactate high vs low\_Ranked |
| Phenotype | NoPhenotypeAvailable |
| Upregulated in class | na\_neg |
| GeneSet | TABULA\_MURIS\_SENIS\_GONADAL\_ADIPOSE\_TISSUE\_B\_CELL\_AGEING |
| Enrichment Score (ES) | -0.40508035 |
| Normalized Enrichment Score (NES) | -1.5932275 |
| Nominal p-value | 0.04531722 |
| FDR q-value | 0.10486804 |
| FWER p-Value | 0.965 |
Table: GSEA Results Summary

  

Fig 1: Enrichment plot: TABULA\_MURIS\_SENIS\_GONADAL\_ADIPOSE\_TISSUE\_B\_CELL\_AGEING      
 Profile of the Running ES Score & Positions of GeneSet Members on the Rank Ordered List

  

| SYMBOL | RANK IN GENE LIST | RANK METRIC SCORE | RUNNING ES | CORE ENRICHMENT || 1 | Apoe | 6 | 2.177 | 0.0558 | No |
| 2 | Lgals1 | 45 | 1.781 | 0.0905 | No |
| 3 | Fcgr2b | 102 | 1.572 | 0.1137 | No |
| 4 | Klf2 | 412 | 1.087 | 0.0400 | No |
| 5 | Serpina3g | 437 | 1.063 | 0.0603 | No |
| 6 | Cotl1 | 447 | 1.049 | 0.0851 | No |
| 7 | Sparc | 460 | 1.038 | 0.1087 | No |
| 8 | H2-Aa | 465 | 1.035 | 0.1349 | No |
| 9 | Cyba | 554 | 0.947 | 0.1308 | No |
| 10 | Cdkn1a | 749 | 0.765 | 0.0868 | No |
| 11 | Cst3 | 782 | 0.723 | 0.0954 | No |
| 12 | Psmb8 | 838 | 0.678 | 0.0951 | No |
| 13 | Cfl1 | 973 | 0.581 | 0.0661 | No |
| 14 | Gpx3 | 1070 | 0.527 | 0.0483 | No |
| 15 | Anxa1 | 1347 | -0.552 | -0.0287 | No |
| 16 | Ddx27 | 1392 | -0.561 | -0.0284 | No |
| 17 | Cops6 | 1432 | -0.572 | -0.0261 | No |
| 18 | Ccnd2 | 2242 | -0.887 | -0.2710 | No |
| 19 | Ly6a | 2366 | -0.979 | -0.2858 | No |
| 20 | Pafah1b3 | 2606 | -1.218 | -0.3328 | No |
| 21 | Dcn | 2701 | -1.362 | -0.3278 | No |
| 22 | Krt15 | 2935 | -2.256 | -0.3452 | Yes |
| 23 | Krt14 | 2985 | -2.898 | -0.2845 | Yes |
| 24 | Krt5 | 3013 | -3.435 | -0.2022 | Yes |
| 25 | Lgals7 | 3028 | -3.903 | -0.1032 | Yes |
| 26 | Krt6a | 3030 | -4.012 | 0.0030 | Yes |
Table: GSEA details [plain text format]

  

Fig 2: TABULA\_MURIS\_SENIS\_GONADAL\_ADIPOSE\_TISSUE\_B\_CELL\_AGEING: Random ES distribution      
 Gene set null distribution of ES for **TABULA\_MURIS\_SENIS\_GONADAL\_ADIPOSE\_TISSUE\_B\_CELL\_AGEING**

  
