# Supplementary material for: TMPRSS11B promotes an acidified microenvironment and immune suppression in squamous lung cancer
Source: EMBO Rep. 2025 Nov 10;26(24):6346–79. doi: 10.1038/s44319-025-00631-1 (PMC12714794; doi:10.1038/s44319-025-00631-1)
Supplement: Supplementary file 11 — Source data Fig. 6 [file 44319_2025_631_MOESM11_ESM.zip › Figure 6/6D-E/GSEA Broad Institute_low pH vs rest of the regions (high pH)/TABULA_MURIS_SENIS_HEART_FIBROBLAST_OF_CARDIAC_TISSUE_AGEING.html]

Details for gene set TABULA\_MURIS\_SENIS\_HEART\_FIBROBLAST\_OF\_CARDIAC\_TISSUE\_AGEING[GSEA]

|  || Dataset | Lactate high vs low\_Ranked |
| Phenotype | NoPhenotypeAvailable |
| Upregulated in class | na\_pos |
| GeneSet | TABULA\_MURIS\_SENIS\_HEART\_FIBROBLAST\_OF\_CARDIAC\_TISSUE\_AGEING |
| Enrichment Score (ES) | 0.43523386 |
| Normalized Enrichment Score (NES) | 2.8570826 |
| Nominal p-value | 0.0 |
| FDR q-value | 0.0 |
| FWER p-Value | 0.0 |
Table: GSEA Results Summary

  

Fig 1: Enrichment plot: TABULA\_MURIS\_SENIS\_HEART\_FIBROBLAST\_OF\_CARDIAC\_TISSUE\_AGEING      
 Profile of the Running ES Score & Positions of GeneSet Members on the Rank Ordered List

  

| SYMBOL | RANK IN GENE LIST | RANK METRIC SCORE | RUNNING ES | CORE ENRICHMENT || 1 | C1qb | 2 | 2.291 | 0.0292 | Yes |
| 2 | Gpnmb | 3 | 2.213 | 0.0580 | Yes |
| 3 | Apoe | 6 | 2.177 | 0.0857 | Yes |
| 4 | C1qc | 20 | 1.988 | 0.1073 | Yes |
| 5 | Mafb | 22 | 1.943 | 0.1323 | Yes |
| 6 | Lgmn | 28 | 1.878 | 0.1551 | Yes |
| 7 | C1qa | 29 | 1.862 | 0.1793 | Yes |
| 8 | Mmp3 | 30 | 1.861 | 0.2036 | Yes |
| 9 | Ctsb | 46 | 1.778 | 0.2217 | Yes |
| 10 | Cmtm3 | 70 | 1.669 | 0.2357 | Yes |
| 11 | Tnfsf12 | 115 | 1.544 | 0.2410 | Yes |
| 12 | Vim | 128 | 1.521 | 0.2568 | Yes |
| 13 | Pltp | 133 | 1.513 | 0.2752 | Yes |
| 14 | Cryab | 158 | 1.449 | 0.2860 | Yes |
| 15 | Atf3 | 161 | 1.444 | 0.3041 | Yes |
| 16 | Npc2 | 202 | 1.371 | 0.3085 | Yes |
| 17 | Dpysl2 | 298 | 1.225 | 0.2925 | Yes |
| 18 | Thbd | 306 | 1.215 | 0.3060 | Yes |
| 19 | Cd74 | 376 | 1.133 | 0.2975 | Yes |
| 20 | Fxyd5 | 377 | 1.133 | 0.3122 | Yes |
| 21 | B2m | 402 | 1.097 | 0.3185 | Yes |
| 22 | H2-Ab1 | 404 | 1.094 | 0.3324 | Yes |
| 23 | Arhgdib | 445 | 1.049 | 0.3326 | Yes |
| 24 | Cdkn1c | 446 | 1.049 | 0.3463 | Yes |
| 25 | Cotl1 | 447 | 1.049 | 0.3599 | Yes |
| 26 | H2-Aa | 465 | 1.035 | 0.3677 | Yes |
| 27 | Nsg1 | 477 | 1.019 | 0.3773 | Yes |
| 28 | Icam1 | 492 | 0.999 | 0.3856 | Yes |
| 29 | Fth1 | 503 | 0.986 | 0.3951 | Yes |
| 30 | Trf | 541 | 0.957 | 0.3951 | Yes |
| 31 | Ccn5 | 542 | 0.955 | 0.4075 | Yes |
| 32 | Cyba | 554 | 0.947 | 0.4162 | Yes |
| 33 | Tspan4 | 625 | 0.870 | 0.4039 | Yes |
| 34 | Dhrs3 | 703 | 0.807 | 0.3885 | Yes |
| 35 | Rem1 | 706 | 0.806 | 0.3983 | Yes |
| 36 | H2-D1 | 722 | 0.794 | 0.4036 | Yes |
| 37 | Cd63 | 724 | 0.793 | 0.4136 | Yes |
| 38 | Grina | 726 | 0.791 | 0.4236 | Yes |
| 39 | Bst2 | 736 | 0.772 | 0.4306 | Yes |
| 40 | Rras | 788 | 0.717 | 0.4228 | Yes |
| 41 | H2-K1 | 818 | 0.692 | 0.4220 | Yes |
| 42 | Prnp | 820 | 0.690 | 0.4307 | Yes |
| 43 | Psmb8 | 838 | 0.678 | 0.4338 | Yes |
| 44 | Igfbp7 | 860 | 0.655 | 0.4352 | Yes |
| 45 | Ndufa4l2 | 900 | 0.629 | 0.4303 | No |
| 46 | C3 | 948 | 0.602 | 0.4223 | No |
| 47 | Cfl1 | 973 | 0.581 | 0.4218 | No |
| 48 | Arl8a | 992 | 0.568 | 0.4231 | No |
| 49 | Ninj1 | 1025 | 0.551 | 0.4195 | No |
| 50 | H2-T23 | 1045 | 0.540 | 0.4202 | No |
| 51 | Gabarapl1 | 1061 | 0.533 | 0.4221 | No |
| 52 | Serpina3n | 1063 | 0.532 | 0.4286 | No |
| 53 | Gpx3 | 1070 | 0.527 | 0.4335 | No |
| 54 | Rack1 | 1195 | -0.519 | 0.3985 | No |
| 55 | Eif3f | 1225 | -0.527 | 0.3956 | No |
| 56 | H3f3b | 1262 | -0.534 | 0.3904 | No |
| 57 | Ier2 | 1527 | -0.593 | 0.3091 | No |
| 58 | Bsg | 1560 | -0.604 | 0.3062 | No |
| 59 | Nr4a1 | 1632 | -0.627 | 0.2905 | No |
| 60 | Rbm26 | 1633 | -0.628 | 0.2986 | No |
| 61 | Aox3 | 1755 | -0.673 | 0.2666 | No |
| 62 | S100a16 | 1801 | -0.687 | 0.2604 | No |
| 63 | Lmo4 | 2166 | -0.842 | 0.1487 | No |
| 64 | Sox9 | 2201 | -0.860 | 0.1485 | No |
| 65 | Elof1 | 2236 | -0.885 | 0.1485 | No |
| 66 | Hes1 | 2255 | -0.896 | 0.1541 | No |
| 67 | Ly6a | 2366 | -0.979 | 0.1298 | No |
| 68 | Fxyd6 | 2370 | -0.984 | 0.1416 | No |
| 69 | Nfkbiz | 2444 | -1.047 | 0.1307 | No |
| 70 | Fos | 2491 | -1.083 | 0.1293 | No |
| 71 | Adh1 | 2495 | -1.089 | 0.1425 | No |
| 72 | Egr1 | 2733 | -1.440 | 0.0814 | No |
| 73 | Ces1d | 2822 | -1.643 | 0.0731 | No |
Table: GSEA details [plain text format]

  

Fig 2: TABULA\_MURIS\_SENIS\_HEART\_FIBROBLAST\_OF\_CARDIAC\_TISSUE\_AGEING: Random ES distribution      
 Gene set null distribution of ES for **TABULA\_MURIS\_SENIS\_HEART\_FIBROBLAST\_OF\_CARDIAC\_TISSUE\_AGEING**

  
