# Supplementary material for: TMPRSS11B promotes an acidified microenvironment and immune suppression in squamous lung cancer
Source: EMBO Rep. 2025 Nov 10;26(24):6346–79. doi: 10.1038/s44319-025-00631-1 (PMC12714794; doi:10.1038/s44319-025-00631-1)
Supplement: Supplementary file 11 — Source data Fig. 6 [file 44319_2025_631_MOESM11_ESM.zip › Figure 6/6D-E/GSEA Broad Institute_low pH vs rest of the regions (high pH)/TABULA_MURIS_SENIS_HEART_MONOCYTE_AGEING.html]

Details for gene set TABULA\_MURIS\_SENIS\_HEART\_MONOCYTE\_AGEING[GSEA]

|  || Dataset | Lactate high vs low\_Ranked |
| Phenotype | NoPhenotypeAvailable |
| Upregulated in class | na\_pos |
| GeneSet | TABULA\_MURIS\_SENIS\_HEART\_MONOCYTE\_AGEING |
| Enrichment Score (ES) | 0.48517105 |
| Normalized Enrichment Score (NES) | 2.7749767 |
| Nominal p-value | 0.0 |
| FDR q-value | 0.0 |
| FWER p-Value | 0.0 |
Table: GSEA Results Summary

  

Fig 1: Enrichment plot: TABULA\_MURIS\_SENIS\_HEART\_MONOCYTE\_AGEING      
 Profile of the Running ES Score & Positions of GeneSet Members on the Rank Ordered List

  

| SYMBOL | RANK IN GENE LIST | RANK METRIC SCORE | RUNNING ES | CORE ENRICHMENT || 1 | C1qa | 29 | 1.862 | 0.0277 | Yes |
| 2 | Gngt2 | 58 | 1.698 | 0.0525 | Yes |
| 3 | Lyz1 | 78 | 1.627 | 0.0789 | Yes |
| 4 | Spi1 | 86 | 1.616 | 0.1090 | Yes |
| 5 | Plekho1 | 92 | 1.606 | 0.1397 | Yes |
| 6 | H2-M2 | 113 | 1.548 | 0.1641 | Yes |
| 7 | Vim | 128 | 1.521 | 0.1900 | Yes |
| 8 | Csf2ra | 143 | 1.478 | 0.2150 | Yes |
| 9 | Mmp12 | 218 | 1.354 | 0.2175 | Yes |
| 10 | Ctsz | 242 | 1.303 | 0.2360 | Yes |
| 11 | Dpysl2 | 298 | 1.225 | 0.2422 | Yes |
| 12 | Cd72 | 327 | 1.189 | 0.2568 | Yes |
| 13 | Abi3 | 339 | 1.175 | 0.2767 | Yes |
| 14 | Lgals3 | 344 | 1.170 | 0.2989 | Yes |
| 15 | H2-DMb1 | 348 | 1.167 | 0.3214 | Yes |
| 16 | Crlf2 | 352 | 1.164 | 0.3438 | Yes |
| 17 | Fxyd5 | 377 | 1.133 | 0.3585 | Yes |
| 18 | Il2rg | 380 | 1.128 | 0.3805 | Yes |
| 19 | H2-Ab1 | 404 | 1.094 | 0.3948 | Yes |
| 20 | H2-Eb1 | 425 | 1.075 | 0.4097 | Yes |
| 21 | Cotl1 | 447 | 1.049 | 0.4238 | Yes |
| 22 | Sparc | 460 | 1.038 | 0.4407 | Yes |
| 23 | H2-Aa | 465 | 1.035 | 0.4602 | Yes |
| 24 | Acp5 | 483 | 1.008 | 0.4747 | Yes |
| 25 | Cyba | 554 | 0.947 | 0.4704 | Yes |
| 26 | Dpt | 567 | 0.935 | 0.4852 | Yes |
| 27 | Metrnl | 714 | 0.799 | 0.4524 | No |
| 28 | Arrb2 | 718 | 0.796 | 0.4674 | No |
| 29 | Grina | 726 | 0.791 | 0.4810 | No |
| 30 | H2-K1 | 818 | 0.692 | 0.4645 | No |
| 31 | Psmb8 | 838 | 0.678 | 0.4718 | No |
| 32 | Mgp | 898 | 0.630 | 0.4647 | No |
| 33 | Atp6v0c | 960 | 0.595 | 0.4563 | No |
| 34 | Tnfsf13 | 969 | 0.588 | 0.4654 | No |
| 35 | Cfl1 | 973 | 0.581 | 0.4761 | No |
| 36 | Erp29 | 1003 | 0.562 | 0.4777 | No |
| 37 | Gpx3 | 1070 | 0.527 | 0.4663 | No |
| 38 | Eif3f | 1225 | -0.527 | 0.4254 | No |
| 39 | H3f3b | 1262 | -0.534 | 0.4241 | No |
| 40 | Anxa1 | 1347 | -0.552 | 0.4071 | No |
| 41 | Zfp710 | 1427 | -0.571 | 0.3921 | No |
| 42 | Ier2 | 1527 | -0.593 | 0.3710 | No |
| 43 | Bsg | 1560 | -0.604 | 0.3724 | No |
| 44 | Tmed3 | 1790 | -0.685 | 0.3096 | No |
| 45 | Cnnm4 | 2021 | -0.770 | 0.2482 | No |
| 46 | S100a6 | 2175 | -0.847 | 0.2140 | No |
| 47 | Basp1 | 2191 | -0.855 | 0.2262 | No |
| 48 | Dcn | 2701 | -1.362 | 0.0834 | No |
| 49 | Ecm1 | 2738 | -1.455 | 0.1006 | No |
Table: GSEA details [plain text format]

  

Fig 2: TABULA\_MURIS\_SENIS\_HEART\_MONOCYTE\_AGEING: Random ES distribution      
 Gene set null distribution of ES for **TABULA\_MURIS\_SENIS\_HEART\_MONOCYTE\_AGEING**

  
