# Supplementary material for: TMPRSS11B promotes an acidified microenvironment and immune suppression in squamous lung cancer
Source: EMBO Rep. 2025 Nov 10;26(24):6346–79. doi: 10.1038/s44319-025-00631-1 (PMC12714794; doi:10.1038/s44319-025-00631-1)
Supplement: Supplementary file 11 — Source data Fig. 6 [file 44319_2025_631_MOESM11_ESM.zip › Figure 6/6D-E/GSEA Broad Institute_low pH vs rest of the regions (high pH)/TABULA_MURIS_SENIS_KIDNEY_MACROPHAGE_AGEING.html]

Details for gene set TABULA\_MURIS\_SENIS\_KIDNEY\_MACROPHAGE\_AGEING[GSEA]

|  || Dataset | Lactate high vs low\_Ranked |
| Phenotype | NoPhenotypeAvailable |
| Upregulated in class | na\_pos |
| GeneSet | TABULA\_MURIS\_SENIS\_KIDNEY\_MACROPHAGE\_AGEING |
| Enrichment Score (ES) | 0.5732997 |
| Normalized Enrichment Score (NES) | 3.4255316 |
| Nominal p-value | 0.0 |
| FDR q-value | 0.0 |
| FWER p-Value | 0.0 |
Table: GSEA Results Summary

  

Fig 1: Enrichment plot: TABULA\_MURIS\_SENIS\_KIDNEY\_MACROPHAGE\_AGEING      
 Profile of the Running ES Score & Positions of GeneSet Members on the Rank Ordered List

  

| SYMBOL | RANK IN GENE LIST | RANK METRIC SCORE | RUNNING ES | CORE ENRICHMENT || 1 | Trem2 | 15 | 2.053 | 0.0304 | Yes |
| 2 | Clec12a | 19 | 1.996 | 0.0638 | Yes |
| 3 | Ms4a6d | 27 | 1.895 | 0.0942 | Yes |
| 4 | Cd300a | 32 | 1.849 | 0.1248 | Yes |
| 5 | Cd84 | 35 | 1.825 | 0.1556 | Yes |
| 6 | Ms4a6c | 37 | 1.819 | 0.1866 | Yes |
| 7 | Napsa | 50 | 1.758 | 0.2130 | Yes |
| 8 | Gngt2 | 58 | 1.698 | 0.2399 | Yes |
| 9 | Pla2g7 | 65 | 1.683 | 0.2670 | Yes |
| 10 | Lyz1 | 78 | 1.627 | 0.2910 | Yes |
| 11 | Ms4a7 | 79 | 1.623 | 0.3190 | Yes |
| 12 | Vim | 128 | 1.521 | 0.3292 | Yes |
| 13 | Pltp | 133 | 1.513 | 0.3540 | Yes |
| 14 | Ctsd | 136 | 1.507 | 0.3793 | Yes |
| 15 | Zeb2 | 157 | 1.451 | 0.3977 | Yes |
| 16 | Cfp | 168 | 1.424 | 0.4189 | Yes |
| 17 | Lrp1 | 183 | 1.402 | 0.4384 | Yes |
| 18 | Emp3 | 191 | 1.389 | 0.4600 | Yes |
| 19 | Fgr | 296 | 1.227 | 0.4464 | Yes |
| 20 | Rap2b | 338 | 1.178 | 0.4531 | Yes |
| 21 | Lgals3 | 344 | 1.170 | 0.4716 | Yes |
| 22 | Grn | 365 | 1.146 | 0.4847 | Yes |
| 23 | Alox5ap | 375 | 1.133 | 0.5012 | Yes |
| 24 | Il2rg | 380 | 1.128 | 0.5193 | Yes |
| 25 | Ccl9 | 411 | 1.089 | 0.5281 | Yes |
| 26 | Klf2 | 412 | 1.087 | 0.5469 | Yes |
| 27 | Sirpb1c | 463 | 1.037 | 0.5480 | Yes |
| 28 | Acp5 | 483 | 1.008 | 0.5591 | Yes |
| 29 | Adgre5 | 493 | 0.998 | 0.5733 | Yes |
| 30 | Cyba | 554 | 0.947 | 0.5696 | No |
| 31 | Msrb1 | 629 | 0.868 | 0.5598 | No |
| 32 | Txn1 | 656 | 0.846 | 0.5657 | No |
| 33 | Ccl6 | 772 | 0.736 | 0.5400 | No |
| 34 | Cebpb | 773 | 0.736 | 0.5527 | No |
| 35 | Mcl1 | 861 | 0.655 | 0.5349 | No |
| 36 | Ptpn1 | 940 | 0.604 | 0.5192 | No |
| 37 | Ninj1 | 1025 | 0.551 | 0.5007 | No |
| 38 | Cstb | 1056 | 0.535 | 0.4999 | No |
| 39 | Gpx3 | 1070 | 0.527 | 0.5046 | No |
| 40 | Cd44 | 1072 | 0.523 | 0.5133 | No |
| 41 | Agpat4 | 1086 | 0.517 | 0.5179 | No |
| 42 | Pgk1 | 1680 | -0.642 | 0.3307 | No |
| 43 | Lmo4 | 2166 | -0.842 | 0.1831 | No |
| 44 | S100a6 | 2175 | -0.847 | 0.1950 | No |
| 45 | Ezr | 2297 | -0.926 | 0.1705 | No |
| 46 | Ly6a | 2366 | -0.979 | 0.1647 | No |
| 47 | Ly6e | 2392 | -1.001 | 0.1736 | No |
| 48 | Fos | 2491 | -1.083 | 0.1595 | No |
| 49 | Plac8 | 2695 | -1.353 | 0.1150 | No |
Table: GSEA details [plain text format]

  

Fig 2: TABULA\_MURIS\_SENIS\_KIDNEY\_MACROPHAGE\_AGEING: Random ES distribution      
 Gene set null distribution of ES for **TABULA\_MURIS\_SENIS\_KIDNEY\_MACROPHAGE\_AGEING**

  
