# Supplementary material for: TMPRSS11B promotes an acidified microenvironment and immune suppression in squamous lung cancer
Source: EMBO Rep. 2025 Nov 10;26(24):6346–79. doi: 10.1038/s44319-025-00631-1 (PMC12714794; doi:10.1038/s44319-025-00631-1)
Supplement: Supplementary file 11 — Source data Fig. 6 [file 44319_2025_631_MOESM11_ESM.zip › Figure 6/6D-E/GSEA Broad Institute_low pH vs rest of the regions (high pH)/TABULA_MURIS_SENIS_LARGE_INTESTINE_ENTEROCYTE_OF_EPITHELIUM_OF_LARGE_INTESTINE_AGEING.html]

Details for gene set TABULA\_MURIS\_SENIS\_LARGE\_INTESTINE\_ENTEROCYTE\_OF\_EPITHELIUM\_OF\_LARGE\_INTESTINE\_AGEING[GSEA]

|  || Dataset | Lactate high vs low\_Ranked |
| Phenotype | NoPhenotypeAvailable |
| Upregulated in class | na\_neg |
| GeneSet | TABULA\_MURIS\_SENIS\_LARGE\_INTESTINE\_ENTEROCYTE\_OF\_EPITHELIUM\_OF\_LARGE\_INTESTINE\_AGEING |
| Enrichment Score (ES) | -0.2136998 |
| Normalized Enrichment Score (NES) | -1.3296487 |
| Nominal p-value | 0.06942889 |
| FDR q-value | 0.36509648 |
| FWER p-Value | 1.0 |
Table: GSEA Results Summary

  

Fig 1: Enrichment plot: TABULA\_MURIS\_SENIS\_LARGE\_INTESTINE\_ENTEROCYTE\_OF\_EPITHELIUM\_OF\_LARGE\_INTESTINE\_AGEING      
 Profile of the Running ES Score & Positions of GeneSet Members on the Rank Ordered List

  

| SYMBOL | RANK IN GENE LIST | RANK METRIC SCORE | RUNNING ES | CORE ENRICHMENT || 1 | Mmp3 | 30 | 1.861 | -0.0010 | No |
| 2 | Ctsb | 46 | 1.778 | 0.0029 | No |
| 3 | Psap | 59 | 1.694 | 0.0075 | No |
| 4 | Npc2 | 202 | 1.371 | -0.0357 | No |
| 5 | Ctsz | 242 | 1.303 | -0.0427 | No |
| 6 | Arg2 | 303 | 1.219 | -0.0576 | No |
| 7 | Lgals3 | 344 | 1.170 | -0.0657 | No |
| 8 | B2m | 402 | 1.097 | -0.0802 | No |
| 9 | Hexa | 476 | 1.021 | -0.1008 | No |
| 10 | Acp5 | 483 | 1.008 | -0.0976 | No |
| 11 | Tmem252 | 484 | 1.008 | -0.0924 | No |
| 12 | Fth1 | 503 | 0.986 | -0.0937 | No |
| 13 | Creg1 | 517 | 0.973 | -0.0932 | No |
| 14 | Cyba | 554 | 0.947 | -0.1011 | No |
| 15 | Ccdc141 | 598 | 0.893 | -0.1117 | No |
| 16 | Gpx1 | 603 | 0.888 | -0.1085 | No |
| 17 | Txn1 | 656 | 0.846 | -0.1225 | No |
| 18 | Smim24 | 657 | 0.845 | -0.1181 | No |
| 19 | Rnase4 | 691 | 0.817 | -0.1255 | No |
| 20 | H2-D1 | 722 | 0.794 | -0.1320 | No |
| 21 | Cd63 | 724 | 0.793 | -0.1283 | No |
| 22 | Cst3 | 782 | 0.723 | -0.1447 | No |
| 23 | Ramac | 797 | 0.709 | -0.1460 | No |
| 24 | Prnp | 820 | 0.690 | -0.1502 | No |
| 25 | Plgrkt | 836 | 0.679 | -0.1520 | No |
| 26 | Calm2 | 857 | 0.664 | -0.1556 | No |
| 27 | Ostf1 | 862 | 0.654 | -0.1536 | No |
| 28 | Ms4a8a | 864 | 0.654 | -0.1506 | No |
| 29 | Pgam1 | 880 | 0.643 | -0.1526 | No |
| 30 | Atp6v0e | 888 | 0.638 | -0.1517 | No |
| 31 | Iscu | 905 | 0.626 | -0.1542 | No |
| 32 | Stard5 | 917 | 0.618 | -0.1548 | No |
| 33 | Cfl1 | 973 | 0.581 | -0.1713 | No |
| 34 | Erp29 | 1003 | 0.562 | -0.1786 | No |
| 35 | Sh3bgrl3 | 1006 | 0.560 | -0.1764 | No |
| 36 | Atp6v1g1 | 1020 | 0.553 | -0.1782 | No |
| 37 | Cstb | 1056 | 0.535 | -0.1878 | No |
| 38 | Srp14 | 1077 | 0.519 | -0.1922 | No |
| 39 | Myl12a | 1087 | 0.516 | -0.1927 | No |
| 40 | Fam98c | 1103 | -0.500 | -0.1954 | No |
| 41 | Gmds | 1104 | -0.501 | -0.1928 | No |
| 42 | Nipsnap2 | 1112 | -0.502 | -0.1927 | No |
| 43 | Stap2 | 1127 | -0.505 | -0.1950 | No |
| 44 | Ndufaf2 | 1147 | -0.508 | -0.1991 | No |
| 45 | Sdcbp2 | 1160 | -0.511 | -0.2007 | No |
| 46 | Zfpl1 | 1161 | -0.511 | -0.1981 | No |
| 47 | Calm3 | 1189 | -0.517 | -0.2049 | No |
| 48 | Coq9 | 1192 | -0.518 | -0.2030 | No |
| 49 | Rack1 | 1195 | -0.519 | -0.2010 | No |
| 50 | Selenos | 1201 | -0.520 | -0.2000 | No |
| 51 | Idh3g | 1205 | -0.521 | -0.1984 | No |
| 52 | Idh3b | 1206 | -0.521 | -0.1957 | No |
| 53 | Ece1 | 1216 | -0.525 | -0.1962 | No |
| 54 | Eif3f | 1225 | -0.527 | -0.1963 | No |
| 55 | 2510002D24Rik | 1230 | -0.528 | -0.1949 | No |
| 56 | Fam162a | 1258 | -0.533 | -0.2017 | No |
| 57 | Pigp | 1260 | -0.533 | -0.1993 | No |
| 58 | H3f3b | 1262 | -0.534 | -0.1969 | No |
| 59 | Bag1 | 1264 | -0.534 | -0.1945 | No |
| 60 | Erg28 | 1269 | -0.535 | -0.1931 | No |
| 61 | Ndufs2 | 1297 | -0.540 | -0.1999 | No |
| 62 | Abhd14b | 1298 | -0.540 | -0.1971 | No |
| 63 | Clpp | 1300 | -0.540 | -0.1946 | No |
| 64 | S100a11 | 1339 | -0.550 | -0.2052 | No |
| 65 | Pdcd6 | 1350 | -0.553 | -0.2059 | No |
| 66 | Eef1b2 | 1352 | -0.553 | -0.2034 | No |
| 67 | Dbndd2 | 1375 | -0.558 | -0.2083 | No |
| 68 | BC031181 | 1376 | -0.558 | -0.2054 | No |
| 69 | Tax1bp3 | 1383 | -0.560 | -0.2046 | No |
| 70 | Surf1 | 1396 | -0.562 | -0.2059 | No |
| 71 | Rac1 | 1404 | -0.564 | -0.2055 | No |
| 72 | Nudt14 | 1414 | -0.566 | -0.2057 | No |
| 73 | Mpst | 1415 | -0.566 | -0.2028 | No |
| 74 | Cops6 | 1432 | -0.572 | -0.2055 | No |
| 75 | Acp1 | 1433 | -0.572 | -0.2025 | No |
| 76 | Uqcc3 | 1456 | -0.576 | -0.2073 | No |
| 77 | Ostc | 1457 | -0.576 | -0.2043 | No |
| 78 | Dpm1 | 1459 | -0.576 | -0.2017 | No |
| 79 | Bola1 | 1463 | -0.577 | -0.1998 | No |
| 80 | Mt2 | 1478 | -0.581 | -0.2017 | No |
| 81 | Eef1g | 1501 | -0.586 | -0.2065 | No |
| 82 | Slc22a18 | 1519 | -0.591 | -0.2094 | No |
| 83 | Zfand2b | 1522 | -0.592 | -0.2071 | No |
| 84 | Ndufa9 | 1524 | -0.592 | -0.2043 | No |
| 85 | Ier2 | 1527 | -0.593 | -0.2020 | No |
| 86 | 1810009A15Rik | 1530 | -0.593 | -0.1996 | No |
| 87 | Eif6 | 1550 | -0.601 | -0.2032 | No |
| 88 | Hmgb1 | 1558 | -0.603 | -0.2026 | No |
| 89 | Bsg | 1560 | -0.604 | -0.1998 | No |
| 90 | Tmem59 | 1563 | -0.605 | -0.1973 | No |
| 91 | Glo1 | 1573 | -0.608 | -0.1974 | No |
| 92 | Kdelr2 | 1577 | -0.609 | -0.1953 | No |
| 93 | Mdp1 | 1596 | -0.615 | -0.1985 | No |
| 94 | Hdac1 | 1598 | -0.616 | -0.1956 | No |
| 95 | Aimp1 | 1609 | -0.618 | -0.1960 | No |
| 96 | Ndufv2 | 1610 | -0.618 | -0.1927 | No |
| 97 | Emg1 | 1616 | -0.620 | -0.1913 | No |
| 98 | Timm44 | 1618 | -0.621 | -0.1884 | No |
| 99 | Pigx | 1620 | -0.622 | -0.1856 | No |
| 100 | Ccdc107 | 1625 | -0.623 | -0.1837 | No |
| 101 | Smim20 | 1628 | -0.625 | -0.1812 | No |
| 102 | Sqor | 1649 | -0.632 | -0.1850 | No |
| 103 | Nudt22 | 1651 | -0.633 | -0.1821 | No |
| 104 | Yipf1 | 1662 | -0.637 | -0.1823 | No |
| 105 | 2610528J11Rik | 1670 | -0.639 | -0.1815 | No |
| 106 | Pgk1 | 1680 | -0.642 | -0.1813 | No |
| 107 | Mea1 | 1688 | -0.648 | -0.1804 | No |
| 108 | Vdac3 | 1705 | -0.657 | -0.1827 | No |
| 109 | Ppa1 | 1719 | -0.661 | -0.1839 | No |
| 110 | Eef1d | 1738 | -0.668 | -0.1868 | No |
| 111 | Tmem147 | 1757 | -0.673 | -0.1897 | No |
| 112 | Atg101 | 1782 | -0.681 | -0.1946 | No |
| 113 | Tmed3 | 1790 | -0.685 | -0.1936 | No |
| 114 | Thap4 | 1797 | -0.686 | -0.1921 | No |
| 115 | S100a16 | 1801 | -0.687 | -0.1896 | No |
| 116 | Alkbh7 | 1821 | -0.696 | -0.1927 | No |
| 117 | Cib1 | 1832 | -0.701 | -0.1926 | No |
| 118 | Tmem205 | 1833 | -0.702 | -0.1890 | No |
| 119 | Hadh | 1869 | -0.711 | -0.1977 | No |
| 120 | Eci1 | 1871 | -0.711 | -0.1944 | No |
| 121 | Pmm1 | 1922 | -0.729 | -0.2083 | No |
| 122 | Fh1 | 1923 | -0.730 | -0.2045 | No |
| 123 | Akr1c13 | 1950 | -0.740 | -0.2099 | Yes |
| 124 | Hnrnpc | 1958 | -0.742 | -0.2085 | Yes |
| 125 | Rpp21 | 1964 | -0.745 | -0.2064 | Yes |
| 126 | Hsp90aa1 | 1965 | -0.745 | -0.2025 | Yes |
| 127 | Trappc5 | 1980 | -0.750 | -0.2036 | Yes |
| 128 | Gclm | 2000 | -0.761 | -0.2064 | Yes |
| 129 | Smagp | 2010 | -0.766 | -0.2056 | Yes |
| 130 | Pts | 2017 | -0.768 | -0.2037 | Yes |
| 131 | Cnnm4 | 2021 | -0.770 | -0.2008 | Yes |
| 132 | Pycard | 2022 | -0.771 | -0.1968 | Yes |
| 133 | Rab25 | 2032 | -0.776 | -0.1959 | Yes |
| 134 | Cmbl | 2054 | -0.787 | -0.1993 | Yes |
| 135 | Aqp11 | 2068 | -0.794 | -0.1998 | Yes |
| 136 | Mcu | 2075 | -0.797 | -0.1978 | Yes |
| 137 | Plpp2 | 2091 | -0.804 | -0.1989 | Yes |
| 138 | Gtf2a2 | 2095 | -0.804 | -0.1958 | Yes |
| 139 | Lurap1l | 2107 | -0.808 | -0.1955 | Yes |
| 140 | Tmem98 | 2109 | -0.809 | -0.1917 | Yes |
| 141 | Fkbp4 | 2117 | -0.814 | -0.1899 | Yes |
| 142 | Mettl26 | 2118 | -0.815 | -0.1857 | Yes |
| 143 | Car9 | 2120 | -0.815 | -0.1818 | Yes |
| 144 | Nudt19 | 2129 | -0.818 | -0.1804 | Yes |
| 145 | Arpc5l | 2141 | -0.824 | -0.1800 | Yes |
| 146 | Tstd1 | 2153 | -0.828 | -0.1796 | Yes |
| 147 | Cbr3 | 2160 | -0.834 | -0.1774 | Yes |
| 148 | Macrod1 | 2161 | -0.838 | -0.1731 | Yes |
| 149 | Lmo4 | 2166 | -0.842 | -0.1701 | Yes |
| 150 | Spag7 | 2169 | -0.844 | -0.1665 | Yes |
| 151 | Rp9 | 2194 | -0.856 | -0.1705 | Yes |
| 152 | Gtf3c6 | 2196 | -0.857 | -0.1664 | Yes |
| 153 | Akr1e1 | 2206 | -0.861 | -0.1651 | Yes |
| 154 | Elof1 | 2236 | -0.885 | -0.1708 | Yes |
| 155 | Akr7a5 | 2240 | -0.886 | -0.1673 | Yes |
| 156 | Cisd3 | 2243 | -0.887 | -0.1634 | Yes |
| 157 | Ifi27l2b | 2250 | -0.894 | -0.1609 | Yes |
| 158 | Krtcap3 | 2265 | -0.902 | -0.1612 | Yes |
| 159 | Hcfc1r1 | 2289 | -0.918 | -0.1645 | Yes |
| 160 | Srek1ip1 | 2300 | -0.928 | -0.1633 | Yes |
| 161 | Cdc42ep5 | 2317 | -0.935 | -0.1641 | Yes |
| 162 | Lgals4 | 2334 | -0.945 | -0.1648 | Yes |
| 163 | Fmc1 | 2341 | -0.954 | -0.1620 | Yes |
| 164 | Nans | 2346 | -0.959 | -0.1585 | Yes |
| 165 | Gale | 2351 | -0.965 | -0.1549 | Yes |
| 166 | Gpd1 | 2364 | -0.978 | -0.1540 | Yes |
| 167 | Cldn3 | 2379 | -0.991 | -0.1539 | Yes |
| 168 | Ap1m2 | 2386 | -0.995 | -0.1508 | Yes |
| 169 | 2310039H08Rik | 2388 | -0.997 | -0.1460 | Yes |
| 170 | Tspan1 | 2419 | -1.017 | -0.1513 | Yes |
| 171 | Pllp | 2430 | -1.030 | -0.1495 | Yes |
| 172 | Dcxr | 2434 | -1.037 | -0.1452 | Yes |
| 173 | Krt19 | 2476 | -1.067 | -0.1542 | Yes |
| 174 | Adh1 | 2495 | -1.089 | -0.1549 | Yes |
| 175 | Srsf3 | 2496 | -1.090 | -0.1493 | Yes |
| 176 | Mgst2 | 2501 | -1.095 | -0.1450 | Yes |
| 177 | Gnpnat1 | 2514 | -1.111 | -0.1435 | Yes |
| 178 | Bad | 2522 | -1.117 | -0.1402 | Yes |
| 179 | Spint2 | 2539 | -1.139 | -0.1399 | Yes |
| 180 | Gstt3 | 2579 | -1.184 | -0.1476 | Yes |
| 181 | Cgref1 | 2584 | -1.189 | -0.1428 | Yes |
| 182 | Il18 | 2586 | -1.191 | -0.1370 | Yes |
| 183 | Fahd1 | 2588 | -1.195 | -0.1312 | Yes |
| 184 | Smim22 | 2591 | -1.201 | -0.1257 | Yes |
| 185 | Pafah1b3 | 2606 | -1.218 | -0.1243 | Yes |
| 186 | Mcrip2 | 2640 | -1.267 | -0.1294 | Yes |
| 187 | Cldn7 | 2642 | -1.269 | -0.1232 | Yes |
| 188 | Ppcs | 2643 | -1.272 | -0.1166 | Yes |
| 189 | Clybl | 2681 | -1.337 | -0.1227 | Yes |
| 190 | Espn | 2688 | -1.346 | -0.1179 | Yes |
| 191 | Plac8 | 2695 | -1.353 | -0.1130 | Yes |
| 192 | AA467197 | 2702 | -1.364 | -0.1080 | Yes |
| 193 | Gstm5 | 2715 | -1.392 | -0.1051 | Yes |
| 194 | Gm3336 | 2730 | -1.426 | -0.1026 | Yes |
| 195 | Tmem45b | 2732 | -1.438 | -0.0955 | Yes |
| 196 | Noxo1 | 2734 | -1.440 | -0.0884 | Yes |
| 197 | Fa2h | 2735 | -1.446 | -0.0809 | Yes |
| 198 | Fbp2 | 2748 | -1.486 | -0.0774 | Yes |
| 199 | Krt7 | 2749 | -1.487 | -0.0697 | Yes |
| 200 | Tst | 2769 | -1.524 | -0.0686 | Yes |
| 201 | Gstp2 | 2788 | -1.561 | -0.0668 | Yes |
| 202 | Fermt1 | 2795 | -1.579 | -0.0608 | Yes |
| 203 | Ces1d | 2822 | -1.643 | -0.0614 | Yes |
| 204 | Klf5 | 2823 | -1.644 | -0.0529 | Yes |
| 205 | Adam28 | 2848 | -1.739 | -0.0524 | Yes |
| 206 | Cela1 | 2888 | -1.928 | -0.0562 | Yes |
| 207 | Pigr | 2910 | -2.092 | -0.0528 | Yes |
| 208 | Gsto1 | 2912 | -2.103 | -0.0422 | Yes |
| 209 | Fgfbp1 | 2921 | -2.159 | -0.0339 | Yes |
| 210 | Bdh1 | 2937 | -2.259 | -0.0275 | Yes |
| 211 | Agr2 | 2938 | -2.260 | -0.0157 | Yes |
| 212 | Paqr5 | 2944 | -2.303 | -0.0056 | Yes |
| 213 | Dmbt1 | 2949 | -2.353 | 0.0052 | Yes |
| 214 | Pglyrp1 | 2963 | -2.492 | 0.0136 | Yes |
| 215 | Ces1f | 2965 | -2.505 | 0.0262 | Yes |
Table: GSEA details [plain text format]

  

Fig 2: TABULA\_MURIS\_SENIS\_LARGE\_INTESTINE\_ENTEROCYTE\_OF\_EPITHELIUM\_OF\_LARGE\_INTESTINE\_AGEING: Random ES distribution      
 Gene set null distribution of ES for **TABULA\_MURIS\_SENIS\_LARGE\_INTESTINE\_ENTEROCYTE\_OF\_EPITHELIUM\_OF\_LARGE\_INTESTINE\_AGEING**

  
