# Supplementary material for: TMPRSS11B promotes an acidified microenvironment and immune suppression in squamous lung cancer
Source: EMBO Rep. 2025 Nov 10;26(24):6346–79. doi: 10.1038/s44319-025-00631-1 (PMC12714794; doi:10.1038/s44319-025-00631-1)
Supplement: Supplementary file 11 — Source data Fig. 6 [file 44319_2025_631_MOESM11_ESM.zip › Figure 6/6D-E/GSEA Broad Institute_low pH vs rest of the regions (high pH)/TABULA_MURIS_SENIS_LARGE_INTESTINE_INTESTINAL_CRYPT_STEM_CELL_AGEING.html]

Details for gene set TABULA\_MURIS\_SENIS\_LARGE\_INTESTINE\_INTESTINAL\_CRYPT\_STEM\_CELL\_AGEING[GSEA]

|  || Dataset | Lactate high vs low\_Ranked |
| Phenotype | NoPhenotypeAvailable |
| Upregulated in class | na\_neg |
| GeneSet | TABULA\_MURIS\_SENIS\_LARGE\_INTESTINE\_INTESTINAL\_CRYPT\_STEM\_CELL\_AGEING |
| Enrichment Score (ES) | -0.2620746 |
| Normalized Enrichment Score (NES) | -1.4004151 |
| Nominal p-value | 0.08010013 |
| FDR q-value | 0.27324712 |
| FWER p-Value | 1.0 |
Table: GSEA Results Summary

  

Fig 1: Enrichment plot: TABULA\_MURIS\_SENIS\_LARGE\_INTESTINE\_INTESTINAL\_CRYPT\_STEM\_CELL\_AGEING      
 Profile of the Running ES Score & Positions of GeneSet Members on the Rank Ordered List

  

| SYMBOL | RANK IN GENE LIST | RANK METRIC SCORE | RUNNING ES | CORE ENRICHMENT || 1 | Rgs1 | 105 | 1.567 | -0.0174 | No |
| 2 | Mfge8 | 118 | 1.535 | -0.0037 | No |
| 3 | Ctsz | 242 | 1.303 | -0.0303 | No |
| 4 | Rgcc | 346 | 1.169 | -0.0517 | No |
| 5 | Cdo1 | 362 | 1.150 | -0.0434 | No |
| 6 | Cotl1 | 447 | 1.049 | -0.0598 | No |
| 7 | Fth1 | 503 | 0.986 | -0.0670 | No |
| 8 | Trf | 541 | 0.957 | -0.0684 | No |
| 9 | Cyba | 554 | 0.947 | -0.0615 | No |
| 10 | Rnase4 | 691 | 0.817 | -0.0982 | No |
| 11 | Col1a2 | 704 | 0.807 | -0.0929 | No |
| 12 | H2-D1 | 722 | 0.794 | -0.0894 | No |
| 13 | Cd63 | 724 | 0.793 | -0.0806 | No |
| 14 | Cd248 | 801 | 0.705 | -0.0982 | No |
| 15 | H2-K1 | 818 | 0.692 | -0.0956 | No |
| 16 | Calm2 | 857 | 0.664 | -0.1008 | No |
| 17 | Ctsh | 890 | 0.637 | -0.1043 | No |
| 18 | Cfl1 | 973 | 0.581 | -0.1253 | No |
| 19 | Ninj1 | 1025 | 0.551 | -0.1362 | No |
| 20 | Ptpn6 | 1096 | 0.505 | -0.1541 | No |
| 21 | Fam98c | 1103 | -0.500 | -0.1503 | No |
| 22 | Sdcbp2 | 1160 | -0.511 | -0.1634 | No |
| 23 | Zfpl1 | 1161 | -0.511 | -0.1575 | No |
| 24 | Fbl | 1188 | -0.517 | -0.1603 | No |
| 25 | Rack1 | 1195 | -0.519 | -0.1563 | No |
| 26 | Ptov1 | 1210 | -0.523 | -0.1550 | No |
| 27 | Ece1 | 1216 | -0.525 | -0.1506 | No |
| 28 | Eif3f | 1225 | -0.527 | -0.1472 | No |
| 29 | 2510002D24Rik | 1230 | -0.528 | -0.1425 | No |
| 30 | H3f3b | 1262 | -0.534 | -0.1468 | No |
| 31 | Cdk5rap3 | 1327 | -0.548 | -0.1621 | No |
| 32 | Eef1b2 | 1352 | -0.553 | -0.1638 | No |
| 33 | Dcps | 1357 | -0.554 | -0.1588 | No |
| 34 | Sfxn1 | 1362 | -0.555 | -0.1537 | No |
| 35 | Ostc | 1457 | -0.576 | -0.1789 | No |
| 36 | Eef1g | 1501 | -0.586 | -0.1867 | No |
| 37 | Ier2 | 1527 | -0.593 | -0.1883 | No |
| 38 | Eif6 | 1550 | -0.601 | -0.1888 | No |
| 39 | Hmgb1 | 1558 | -0.603 | -0.1842 | No |
| 40 | Bsg | 1560 | -0.604 | -0.1775 | No |
| 41 | Emg1 | 1616 | -0.620 | -0.1890 | No |
| 42 | Timm44 | 1618 | -0.621 | -0.1821 | No |
| 43 | Nudt22 | 1651 | -0.633 | -0.1856 | No |
| 44 | 2610528J11Rik | 1670 | -0.639 | -0.1843 | No |
| 45 | Mal | 1710 | -0.658 | -0.1899 | No |
| 46 | Eef1d | 1738 | -0.668 | -0.1913 | No |
| 47 | Atg101 | 1782 | -0.681 | -0.1980 | No |
| 48 | Tmed3 | 1790 | -0.685 | -0.1925 | No |
| 49 | Lsr | 1792 | -0.685 | -0.1849 | No |
| 50 | S100a16 | 1801 | -0.687 | -0.1796 | No |
| 51 | Nop56 | 1822 | -0.696 | -0.1783 | No |
| 52 | Pmm1 | 1922 | -0.729 | -0.2034 | No |
| 53 | Smagp | 2010 | -0.766 | -0.2241 | No |
| 54 | Endog | 2012 | -0.767 | -0.2155 | No |
| 55 | Cdpf1 | 2052 | -0.786 | -0.2196 | No |
| 56 | Mettl26 | 2118 | -0.815 | -0.2322 | No |
| 57 | Apex1 | 2125 | -0.816 | -0.2248 | No |
| 58 | Elof1 | 2236 | -0.885 | -0.2518 | Yes |
| 59 | Akr7a5 | 2240 | -0.886 | -0.2426 | Yes |
| 60 | Hes1 | 2255 | -0.896 | -0.2369 | Yes |
| 61 | Krtcap3 | 2265 | -0.902 | -0.2296 | Yes |
| 62 | Pold2 | 2301 | -0.928 | -0.2307 | Yes |
| 63 | Cdc42ep5 | 2317 | -0.935 | -0.2249 | Yes |
| 64 | Ptgr1 | 2332 | -0.943 | -0.2187 | Yes |
| 65 | Gale | 2351 | -0.965 | -0.2137 | Yes |
| 66 | Cldn3 | 2379 | -0.991 | -0.2113 | Yes |
| 67 | Ly6e | 2392 | -1.001 | -0.2038 | Yes |
| 68 | Pllp | 2430 | -1.030 | -0.2044 | Yes |
| 69 | Sdsl | 2438 | -1.042 | -0.1947 | Yes |
| 70 | Mecr | 2441 | -1.044 | -0.1833 | Yes |
| 71 | Adh1 | 2495 | -1.089 | -0.1887 | Yes |
| 72 | Spint2 | 2539 | -1.139 | -0.1900 | Yes |
| 73 | S100a14 | 2580 | -1.185 | -0.1899 | Yes |
| 74 | Fahd1 | 2588 | -1.195 | -0.1784 | Yes |
| 75 | Pafah1b3 | 2606 | -1.218 | -0.1701 | Yes |
| 76 | Noxo1 | 2734 | -1.440 | -0.1964 | Yes |
| 77 | Krt7 | 2749 | -1.487 | -0.1839 | Yes |
| 78 | Tst | 2769 | -1.524 | -0.1727 | Yes |
| 79 | Fermt1 | 2795 | -1.579 | -0.1629 | Yes |
| 80 | Gsta4 | 2809 | -1.609 | -0.1487 | Yes |
| 81 | Krt20 | 2839 | -1.707 | -0.1387 | Yes |
| 82 | Qtrt1 | 2857 | -1.782 | -0.1239 | Yes |
| 83 | Clu | 2876 | -1.874 | -0.1083 | Yes |
| 84 | Ppp1r1b | 2878 | -1.876 | -0.0869 | Yes |
| 85 | Cela1 | 2888 | -1.928 | -0.0676 | Yes |
| 86 | Fgfbp1 | 2921 | -2.159 | -0.0534 | Yes |
| 87 | Kcne3 | 2945 | -2.310 | -0.0345 | Yes |
| 88 | Pglyrp1 | 2963 | -2.492 | -0.0114 | Yes |
| 89 | Lypd3 | 3005 | -3.177 | 0.0115 | Yes |
Table: GSEA details [plain text format]

  

Fig 2: TABULA\_MURIS\_SENIS\_LARGE\_INTESTINE\_INTESTINAL\_CRYPT\_STEM\_CELL\_AGEING: Random ES distribution      
 Gene set null distribution of ES for **TABULA\_MURIS\_SENIS\_LARGE\_INTESTINE\_INTESTINAL\_CRYPT\_STEM\_CELL\_AGEING**

  
