# Supplementary material for: TMPRSS11B promotes an acidified microenvironment and immune suppression in squamous lung cancer
Source: EMBO Rep. 2025 Nov 10;26(24):6346–79. doi: 10.1038/s44319-025-00631-1 (PMC12714794; doi:10.1038/s44319-025-00631-1)
Supplement: Supplementary file 11 — Source data Fig. 6 [file 44319_2025_631_MOESM11_ESM.zip › Figure 6/6D-E/GSEA Broad Institute_low pH vs rest of the regions (high pH)/TABULA_MURIS_SENIS_LARGE_INTESTINE_LARGE_INTESTINE_GOBLET_CELL_AGEING.html]

Details for gene set TABULA\_MURIS\_SENIS\_LARGE\_INTESTINE\_LARGE\_INTESTINE\_GOBLET\_CELL\_AGEING[GSEA]

|  || Dataset | Lactate high vs low\_Ranked |
| Phenotype | NoPhenotypeAvailable |
| Upregulated in class | na\_neg |
| GeneSet | TABULA\_MURIS\_SENIS\_LARGE\_INTESTINE\_LARGE\_INTESTINE\_GOBLET\_CELL\_AGEING |
| Enrichment Score (ES) | -0.21644391 |
| Normalized Enrichment Score (NES) | -1.2975113 |
| Nominal p-value | 0.10215664 |
| FDR q-value | 0.3948916 |
| FWER p-Value | 1.0 |
Table: GSEA Results Summary

  

Fig 1: Enrichment plot: TABULA\_MURIS\_SENIS\_LARGE\_INTESTINE\_LARGE\_INTESTINE\_GOBLET\_CELL\_AGEING      
 Profile of the Running ES Score & Positions of GeneSet Members on the Rank Ordered List

  

| SYMBOL | RANK IN GENE LIST | RANK METRIC SCORE | RUNNING ES | CORE ENRICHMENT || 1 | Ctsb | 46 | 1.778 | -0.0047 | No |
| 2 | Psap | 59 | 1.694 | 0.0018 | No |
| 3 | Npc2 | 202 | 1.371 | -0.0389 | No |
| 4 | Ctsz | 242 | 1.303 | -0.0442 | No |
| 5 | Cdo1 | 362 | 1.150 | -0.0784 | No |
| 6 | Cbfa2t3 | 394 | 1.107 | -0.0821 | No |
| 7 | B2m | 402 | 1.097 | -0.0776 | No |
| 8 | Crip1 | 431 | 1.069 | -0.0806 | No |
| 9 | Cotl1 | 447 | 1.049 | -0.0791 | No |
| 10 | Rasl12 | 468 | 1.033 | -0.0795 | No |
| 11 | Acp5 | 483 | 1.008 | -0.0780 | No |
| 12 | Fth1 | 503 | 0.986 | -0.0784 | No |
| 13 | Cyba | 554 | 0.947 | -0.0898 | No |
| 14 | Dusp1 | 569 | 0.935 | -0.0887 | No |
| 15 | Aqp1 | 572 | 0.930 | -0.0835 | No |
| 16 | Ehd4 | 595 | 0.901 | -0.0855 | No |
| 17 | Gpx1 | 603 | 0.888 | -0.0823 | No |
| 18 | Hilpda | 636 | 0.860 | -0.0879 | No |
| 19 | Txn1 | 656 | 0.846 | -0.0892 | No |
| 20 | Gng11 | 662 | 0.840 | -0.0856 | No |
| 21 | Irf8 | 663 | 0.840 | -0.0803 | No |
| 22 | Rnase4 | 691 | 0.817 | -0.0845 | No |
| 23 | H2-D1 | 722 | 0.794 | -0.0899 | No |
| 24 | Cd63 | 724 | 0.793 | -0.0852 | No |
| 25 | Dhrs7 | 752 | 0.762 | -0.0898 | No |
| 26 | Cst3 | 782 | 0.723 | -0.0953 | No |
| 27 | Ramac | 797 | 0.709 | -0.0956 | No |
| 28 | Tcf7l1 | 810 | 0.700 | -0.0954 | No |
| 29 | H2-K1 | 818 | 0.692 | -0.0934 | No |
| 30 | Calm2 | 857 | 0.664 | -0.1025 | No |
| 31 | Ctsh | 890 | 0.637 | -0.1096 | No |
| 32 | Iscu | 905 | 0.626 | -0.1105 | No |
| 33 | Cfl1 | 973 | 0.581 | -0.1301 | No |
| 34 | Erp29 | 1003 | 0.562 | -0.1366 | No |
| 35 | Sh3bgrl3 | 1006 | 0.560 | -0.1338 | No |
| 36 | Atp6v1g1 | 1020 | 0.553 | -0.1348 | No |
| 37 | H2-T23 | 1045 | 0.540 | -0.1397 | No |
| 38 | Fam98c | 1103 | -0.500 | -0.1564 | No |
| 39 | Gmds | 1104 | -0.501 | -0.1532 | No |
| 40 | Stap2 | 1127 | -0.505 | -0.1577 | No |
| 41 | Sdcbp2 | 1160 | -0.511 | -0.1656 | No |
| 42 | Zfpl1 | 1161 | -0.511 | -0.1623 | No |
| 43 | Fbl | 1188 | -0.517 | -0.1681 | No |
| 44 | Calm3 | 1189 | -0.517 | -0.1648 | No |
| 45 | Coq9 | 1192 | -0.518 | -0.1622 | No |
| 46 | Selenos | 1201 | -0.520 | -0.1617 | No |
| 47 | Ptov1 | 1210 | -0.523 | -0.1612 | No |
| 48 | Eif3f | 1225 | -0.527 | -0.1627 | No |
| 49 | 2510002D24Rik | 1230 | -0.528 | -0.1608 | No |
| 50 | H3f3b | 1262 | -0.534 | -0.1682 | No |
| 51 | Bag1 | 1264 | -0.534 | -0.1651 | No |
| 52 | Ndufs2 | 1297 | -0.540 | -0.1728 | No |
| 53 | S100a11 | 1339 | -0.550 | -0.1836 | No |
| 54 | Pdcd6 | 1350 | -0.553 | -0.1836 | No |
| 55 | Sil1 | 1353 | -0.553 | -0.1808 | No |
| 56 | Sfxn1 | 1362 | -0.555 | -0.1800 | No |
| 57 | BC031181 | 1376 | -0.558 | -0.1810 | No |
| 58 | Surf1 | 1396 | -0.562 | -0.1841 | No |
| 59 | Arfip2 | 1400 | -0.562 | -0.1816 | No |
| 60 | Rac1 | 1404 | -0.564 | -0.1790 | No |
| 61 | Nudt14 | 1414 | -0.566 | -0.1786 | No |
| 62 | Acp1 | 1433 | -0.572 | -0.1812 | No |
| 63 | Uqcc3 | 1456 | -0.576 | -0.1852 | No |
| 64 | Ostc | 1457 | -0.576 | -0.1815 | No |
| 65 | Bola1 | 1463 | -0.577 | -0.1796 | No |
| 66 | Nectin2 | 1479 | -0.581 | -0.1812 | No |
| 67 | Mob2 | 1489 | -0.583 | -0.1806 | No |
| 68 | Eef1g | 1501 | -0.586 | -0.1807 | No |
| 69 | Zfand2b | 1522 | -0.592 | -0.1839 | No |
| 70 | Ier2 | 1527 | -0.593 | -0.1815 | No |
| 71 | Dap | 1535 | -0.595 | -0.1802 | No |
| 72 | Commd9 | 1540 | -0.597 | -0.1778 | No |
| 73 | Eif6 | 1550 | -0.601 | -0.1771 | No |
| 74 | Bsg | 1560 | -0.604 | -0.1764 | No |
| 75 | Tmem59 | 1563 | -0.605 | -0.1733 | No |
| 76 | Foxp4 | 1604 | -0.617 | -0.1833 | No |
| 77 | Ndufv2 | 1610 | -0.618 | -0.1811 | No |
| 78 | Emg1 | 1616 | -0.620 | -0.1789 | No |
| 79 | Ccdc107 | 1625 | -0.623 | -0.1777 | No |
| 80 | Smim20 | 1628 | -0.625 | -0.1745 | No |
| 81 | Nhp2 | 1637 | -0.629 | -0.1733 | No |
| 82 | Mpi | 1642 | -0.631 | -0.1706 | No |
| 83 | Nudt22 | 1651 | -0.633 | -0.1694 | No |
| 84 | Yipf1 | 1662 | -0.637 | -0.1689 | No |
| 85 | 2610528J11Rik | 1670 | -0.639 | -0.1672 | No |
| 86 | Mea1 | 1688 | -0.648 | -0.1690 | No |
| 87 | Ppa1 | 1719 | -0.661 | -0.1753 | No |
| 88 | Pdzd11 | 1720 | -0.662 | -0.1711 | No |
| 89 | Eef1d | 1738 | -0.668 | -0.1728 | No |
| 90 | Tmem147 | 1757 | -0.673 | -0.1748 | No |
| 91 | Atg101 | 1782 | -0.681 | -0.1788 | No |
| 92 | Tmed3 | 1790 | -0.685 | -0.1769 | No |
| 93 | S100a16 | 1801 | -0.687 | -0.1760 | No |
| 94 | Cib1 | 1832 | -0.701 | -0.1820 | No |
| 95 | Tmem205 | 1833 | -0.702 | -0.1776 | No |
| 96 | Gstm2 | 1848 | -0.706 | -0.1780 | No |
| 97 | Fdft1 | 1952 | -0.741 | -0.2091 | No |
| 98 | Rab3d | 1974 | -0.749 | -0.2117 | Yes |
| 99 | Prss8 | 1985 | -0.752 | -0.2104 | Yes |
| 100 | Serinc2 | 1986 | -0.754 | -0.2056 | Yes |
| 101 | Smagp | 2010 | -0.766 | -0.2088 | Yes |
| 102 | Endog | 2012 | -0.767 | -0.2042 | Yes |
| 103 | Pts | 2017 | -0.768 | -0.2007 | Yes |
| 104 | Pycard | 2022 | -0.771 | -0.1972 | Yes |
| 105 | Rab25 | 2032 | -0.776 | -0.1955 | Yes |
| 106 | Cdpf1 | 2052 | -0.786 | -0.1971 | Yes |
| 107 | Gtf2a2 | 2095 | -0.804 | -0.2066 | Yes |
| 108 | Mettl26 | 2118 | -0.815 | -0.2091 | Yes |
| 109 | Tm2d3 | 2122 | -0.816 | -0.2050 | Yes |
| 110 | Cmtm8 | 2124 | -0.816 | -0.2001 | Yes |
| 111 | Dnajc3 | 2133 | -0.820 | -0.1977 | Yes |
| 112 | Tstd1 | 2153 | -0.828 | -0.1991 | Yes |
| 113 | Spag7 | 2169 | -0.844 | -0.1989 | Yes |
| 114 | Ppif | 2171 | -0.845 | -0.1939 | Yes |
| 115 | Gmppb | 2217 | -0.868 | -0.2041 | Yes |
| 116 | Elof1 | 2236 | -0.885 | -0.2047 | Yes |
| 117 | Akr7a5 | 2240 | -0.886 | -0.2002 | Yes |
| 118 | Ccnd2 | 2242 | -0.887 | -0.1949 | Yes |
| 119 | Cisd3 | 2243 | -0.887 | -0.1892 | Yes |
| 120 | Ifi27l2b | 2250 | -0.894 | -0.1857 | Yes |
| 121 | Krtcap3 | 2265 | -0.902 | -0.1848 | Yes |
| 122 | Cdc42ep5 | 2317 | -0.935 | -0.1966 | Yes |
| 123 | Ptgr1 | 2332 | -0.943 | -0.1955 | Yes |
| 124 | Nans | 2346 | -0.959 | -0.1940 | Yes |
| 125 | Gale | 2351 | -0.965 | -0.1892 | Yes |
| 126 | Bcat2 | 2363 | -0.978 | -0.1869 | Yes |
| 127 | Cldn3 | 2379 | -0.991 | -0.1858 | Yes |
| 128 | 2310039H08Rik | 2388 | -0.997 | -0.1822 | Yes |
| 129 | Ly6e | 2392 | -1.001 | -0.1769 | Yes |
| 130 | Tspan1 | 2419 | -1.017 | -0.1795 | Yes |
| 131 | Pllp | 2430 | -1.030 | -0.1765 | Yes |
| 132 | Dcxr | 2434 | -1.037 | -0.1709 | Yes |
| 133 | Mecr | 2441 | -1.044 | -0.1664 | Yes |
| 134 | Krt19 | 2476 | -1.067 | -0.1715 | Yes |
| 135 | Mgst2 | 2501 | -1.095 | -0.1729 | Yes |
| 136 | Smim6 | 2506 | -1.098 | -0.1673 | Yes |
| 137 | Bad | 2522 | -1.117 | -0.1654 | Yes |
| 138 | Spint2 | 2539 | -1.139 | -0.1638 | Yes |
| 139 | Nupr1 | 2565 | -1.167 | -0.1651 | Yes |
| 140 | S100a14 | 2580 | -1.185 | -0.1624 | Yes |
| 141 | Cgref1 | 2584 | -1.189 | -0.1559 | Yes |
| 142 | Smim22 | 2591 | -1.201 | -0.1504 | Yes |
| 143 | Gpx2 | 2598 | -1.211 | -0.1448 | Yes |
| 144 | Pafah1b3 | 2606 | -1.218 | -0.1395 | Yes |
| 145 | Mcrip2 | 2640 | -1.267 | -0.1429 | Yes |
| 146 | Urah | 2658 | -1.303 | -0.1406 | Yes |
| 147 | Phldb3 | 2684 | -1.340 | -0.1408 | Yes |
| 148 | Gstm5 | 2715 | -1.392 | -0.1424 | Yes |
| 149 | Tmem45b | 2732 | -1.438 | -0.1388 | Yes |
| 150 | Noxo1 | 2734 | -1.440 | -0.1300 | Yes |
| 151 | Fbp2 | 2748 | -1.486 | -0.1251 | Yes |
| 152 | Plet1 | 2762 | -1.513 | -0.1201 | Yes |
| 153 | Tst | 2769 | -1.524 | -0.1125 | Yes |
| 154 | Lrrc26 | 2777 | -1.542 | -0.1051 | Yes |
| 155 | Gstp2 | 2788 | -1.561 | -0.0987 | Yes |
| 156 | Fermt1 | 2795 | -1.579 | -0.0908 | Yes |
| 157 | Fxyd3 | 2815 | -1.624 | -0.0871 | Yes |
| 158 | Klf5 | 2823 | -1.644 | -0.0791 | Yes |
| 159 | Isg20 | 2853 | -1.758 | -0.0780 | Yes |
| 160 | Creb3l1 | 2875 | -1.872 | -0.0734 | Yes |
| 161 | Ppp1r1b | 2878 | -1.876 | -0.0622 | Yes |
| 162 | Qsox1 | 2881 | -1.902 | -0.0508 | Yes |
| 163 | Cela1 | 2888 | -1.928 | -0.0407 | Yes |
| 164 | Gsto1 | 2912 | -2.103 | -0.0353 | Yes |
| 165 | Agr2 | 2938 | -2.260 | -0.0297 | Yes |
| 166 | Kcne3 | 2945 | -2.310 | -0.0171 | Yes |
| 167 | Prss32 | 2952 | -2.366 | -0.0042 | Yes |
| 168 | Pglyrp1 | 2963 | -2.492 | 0.0082 | Yes |
| 169 | Atp2c2 | 2980 | -2.830 | 0.0206 | Yes |
Table: GSEA details [plain text format]

  

Fig 2: TABULA\_MURIS\_SENIS\_LARGE\_INTESTINE\_LARGE\_INTESTINE\_GOBLET\_CELL\_AGEING: Random ES distribution      
 Gene set null distribution of ES for **TABULA\_MURIS\_SENIS\_LARGE\_INTESTINE\_LARGE\_INTESTINE\_GOBLET\_CELL\_AGEING**

  
