# Supplementary material for: TMPRSS11B promotes an acidified microenvironment and immune suppression in squamous lung cancer
Source: EMBO Rep. 2025 Nov 10;26(24):6346–79. doi: 10.1038/s44319-025-00631-1 (PMC12714794; doi:10.1038/s44319-025-00631-1)
Supplement: Supplementary file 11 — Source data Fig. 6 [file 44319_2025_631_MOESM11_ESM.zip › Figure 6/6D-E/GSEA Broad Institute_low pH vs rest of the regions (high pH)/TABULA_MURIS_SENIS_LUNG_ALVEOLAR_MACROPHAGE_AGEING.html]

Details for gene set TABULA\_MURIS\_SENIS\_LUNG\_ALVEOLAR\_MACROPHAGE\_AGEING[GSEA]

|  || Dataset | Lactate high vs low\_Ranked |
| Phenotype | NoPhenotypeAvailable |
| Upregulated in class | na\_pos |
| GeneSet | TABULA\_MURIS\_SENIS\_LUNG\_ALVEOLAR\_MACROPHAGE\_AGEING |
| Enrichment Score (ES) | 0.60844404 |
| Normalized Enrichment Score (NES) | 3.8142753 |
| Nominal p-value | 0.0 |
| FDR q-value | 0.0 |
| FWER p-Value | 0.0 |
Table: GSEA Results Summary

  

Fig 1: Enrichment plot: TABULA\_MURIS\_SENIS\_LUNG\_ALVEOLAR\_MACROPHAGE\_AGEING      
 Profile of the Running ES Score & Positions of GeneSet Members on the Rank Ordered List

  

| SYMBOL | RANK IN GENE LIST | RANK METRIC SCORE | RUNNING ES | CORE ENRICHMENT || 1 | Apoe | 6 | 2.177 | 0.0256 | Yes |
| 2 | Ctsl | 8 | 2.153 | 0.0527 | Yes |
| 3 | Fabp4 | 12 | 2.100 | 0.0784 | Yes |
| 4 | Ctss | 14 | 2.088 | 0.1046 | Yes |
| 5 | Trem2 | 15 | 2.053 | 0.1306 | Yes |
| 6 | Lpl | 34 | 1.833 | 0.1479 | Yes |
| 7 | Ms4a6c | 37 | 1.819 | 0.1703 | Yes |
| 8 | Apoc1 | 40 | 1.811 | 0.1927 | Yes |
| 9 | Ctsb | 46 | 1.778 | 0.2136 | Yes |
| 10 | Napsa | 50 | 1.758 | 0.2349 | Yes |
| 11 | Pirb | 57 | 1.699 | 0.2545 | Yes |
| 12 | Psap | 59 | 1.694 | 0.2757 | Yes |
| 13 | Igf1 | 60 | 1.691 | 0.2972 | Yes |
| 14 | Ctsk | 71 | 1.668 | 0.3150 | Yes |
| 15 | Tyrobp | 83 | 1.618 | 0.3319 | Yes |
| 16 | Fcer1g | 95 | 1.597 | 0.3485 | Yes |
| 17 | Cd274 | 104 | 1.569 | 0.3657 | Yes |
| 18 | Cybb | 119 | 1.535 | 0.3806 | Yes |
| 19 | Fcgr4 | 155 | 1.453 | 0.3873 | Yes |
| 20 | Emp3 | 191 | 1.389 | 0.3932 | Yes |
| 21 | Hexb | 197 | 1.379 | 0.4090 | Yes |
| 22 | Npc2 | 202 | 1.371 | 0.4251 | Yes |
| 23 | Spp1 | 232 | 1.327 | 0.4322 | Yes |
| 24 | Cd52 | 233 | 1.323 | 0.4490 | Yes |
| 25 | Csf1r | 249 | 1.292 | 0.4604 | Yes |
| 26 | Msn | 280 | 1.243 | 0.4661 | Yes |
| 27 | Hcst | 282 | 1.242 | 0.4816 | Yes |
| 28 | Timp2 | 334 | 1.180 | 0.4794 | Yes |
| 29 | Lgals3 | 344 | 1.170 | 0.4913 | Yes |
| 30 | Wfdc17 | 345 | 1.169 | 0.5061 | Yes |
| 31 | H2-DMb1 | 348 | 1.167 | 0.5203 | Yes |
| 32 | Cd74 | 376 | 1.133 | 0.5256 | Yes |
| 33 | Scd1 | 388 | 1.111 | 0.5360 | Yes |
| 34 | B2m | 402 | 1.097 | 0.5456 | Yes |
| 35 | H2-Ab1 | 404 | 1.094 | 0.5592 | Yes |
| 36 | Ccl9 | 411 | 1.089 | 0.5710 | Yes |
| 37 | Klf2 | 412 | 1.087 | 0.5848 | Yes |
| 38 | H2-Eb1 | 425 | 1.075 | 0.5945 | Yes |
| 39 | Crip1 | 431 | 1.069 | 0.6064 | Yes |
| 40 | H2-Aa | 465 | 1.035 | 0.6084 | Yes |
| 41 | Creg1 | 517 | 0.973 | 0.6037 | No |
| 42 | Cyba | 554 | 0.947 | 0.6036 | No |
| 43 | H2-D1 | 722 | 0.794 | 0.5576 | No |
| 44 | Cd63 | 724 | 0.793 | 0.5674 | No |
| 45 | Cst3 | 782 | 0.723 | 0.5574 | No |
| 46 | Tln1 | 804 | 0.703 | 0.5593 | No |
| 47 | H2-K1 | 818 | 0.692 | 0.5637 | No |
| 48 | Psmb8 | 838 | 0.678 | 0.5660 | No |
| 49 | C3 | 948 | 0.602 | 0.5370 | No |
| 50 | S100a10 | 965 | 0.592 | 0.5391 | No |
| 51 | Litaf | 1084 | 0.517 | 0.5061 | No |
| 52 | S100a11 | 1339 | -0.550 | 0.4278 | No |
| 53 | Uqcc3 | 1456 | -0.576 | 0.3961 | No |
| 54 | Pgk1 | 1680 | -0.642 | 0.3294 | No |
| 55 | Slpi | 2011 | -0.766 | 0.2283 | No |
| 56 | Pycard | 2022 | -0.771 | 0.2347 | No |
| 57 | Cmbl | 2054 | -0.787 | 0.2343 | No |
| 58 | Cd2ap | 2149 | -0.827 | 0.2132 | No |
| 59 | S100a6 | 2175 | -0.847 | 0.2156 | No |
| 60 | Ly6e | 2392 | -1.001 | 0.1557 | No |
| 61 | Ggh | 2480 | -1.071 | 0.1401 | No |
| 62 | Plac8 | 2695 | -1.353 | 0.0854 | No |
| 63 | Cd24a | 2948 | -2.343 | 0.0306 | No |
Table: GSEA details [plain text format]

  

Fig 2: TABULA\_MURIS\_SENIS\_LUNG\_ALVEOLAR\_MACROPHAGE\_AGEING: Random ES distribution      
 Gene set null distribution of ES for **TABULA\_MURIS\_SENIS\_LUNG\_ALVEOLAR\_MACROPHAGE\_AGEING**

  
