# Supplementary material for: TMPRSS11B promotes an acidified microenvironment and immune suppression in squamous lung cancer
Source: EMBO Rep. 2025 Nov 10;26(24):6346–79. doi: 10.1038/s44319-025-00631-1 (PMC12714794; doi:10.1038/s44319-025-00631-1)
Supplement: Supplementary file 11 — Source data Fig. 6 [file 44319_2025_631_MOESM11_ESM.zip › Figure 6/6D-E/GSEA Broad Institute_low pH vs rest of the regions (high pH)/TABULA_MURIS_SENIS_LUNG_BRONCHIAL_SMOOTH_MUSCLE_CELL_AGEING.html]

Details for gene set TABULA\_MURIS\_SENIS\_LUNG\_BRONCHIAL\_SMOOTH\_MUSCLE\_CELL\_AGEING[GSEA]

|  || Dataset | Lactate high vs low\_Ranked |
| Phenotype | NoPhenotypeAvailable |
| Upregulated in class | na\_pos |
| GeneSet | TABULA\_MURIS\_SENIS\_LUNG\_BRONCHIAL\_SMOOTH\_MUSCLE\_CELL\_AGEING |
| Enrichment Score (ES) | 0.47171798 |
| Normalized Enrichment Score (NES) | 2.8205643 |
| Nominal p-value | 0.0 |
| FDR q-value | 0.0 |
| FWER p-Value | 0.0 |
Table: GSEA Results Summary

  

Fig 1: Enrichment plot: TABULA\_MURIS\_SENIS\_LUNG\_BRONCHIAL\_SMOOTH\_MUSCLE\_CELL\_AGEING      
 Profile of the Running ES Score & Positions of GeneSet Members on the Rank Ordered List

  

| SYMBOL | RANK IN GENE LIST | RANK METRIC SCORE | RUNNING ES | CORE ENRICHMENT || 1 | Cryab | 158 | 1.449 | -0.0242 | Yes |
| 2 | Atf3 | 161 | 1.444 | 0.0037 | Yes |
| 3 | Emp3 | 191 | 1.389 | 0.0215 | Yes |
| 4 | Cldn5 | 227 | 1.338 | 0.0363 | Yes |
| 5 | Ptprb | 337 | 1.179 | 0.0232 | Yes |
| 6 | Vwf | 343 | 1.170 | 0.0447 | Yes |
| 7 | H2-DMb1 | 348 | 1.167 | 0.0664 | Yes |
| 8 | Adk | 356 | 1.156 | 0.0870 | Yes |
| 9 | Cd74 | 376 | 1.133 | 0.1031 | Yes |
| 10 | Fxyd5 | 377 | 1.133 | 0.1255 | Yes |
| 11 | Gja4 | 399 | 1.101 | 0.1403 | Yes |
| 12 | B2m | 402 | 1.097 | 0.1613 | Yes |
| 13 | H2-Ab1 | 404 | 1.094 | 0.1827 | Yes |
| 14 | Klf2 | 412 | 1.087 | 0.2018 | Yes |
| 15 | H2-DMa | 418 | 1.083 | 0.2216 | Yes |
| 16 | H2-Eb1 | 425 | 1.075 | 0.2409 | Yes |
| 17 | Crip1 | 431 | 1.069 | 0.2604 | Yes |
| 18 | Sox17 | 450 | 1.047 | 0.2751 | Yes |
| 19 | H2-Aa | 465 | 1.035 | 0.2909 | Yes |
| 20 | Mmrn2 | 473 | 1.025 | 0.3089 | Yes |
| 21 | Nsg1 | 477 | 1.019 | 0.3281 | Yes |
| 22 | Tmem252 | 484 | 1.008 | 0.3460 | Yes |
| 23 | Cxcl12 | 488 | 1.003 | 0.3649 | Yes |
| 24 | Aqp1 | 572 | 0.930 | 0.3555 | Yes |
| 25 | Flt1 | 628 | 0.869 | 0.3543 | Yes |
| 26 | Hilpda | 636 | 0.860 | 0.3690 | Yes |
| 27 | Vegfa | 650 | 0.852 | 0.3815 | Yes |
| 28 | H2-Q4 | 681 | 0.826 | 0.3878 | Yes |
| 29 | Syne1 | 710 | 0.803 | 0.3943 | Yes |
| 30 | Akap12 | 713 | 0.801 | 0.4095 | Yes |
| 31 | H2-D1 | 722 | 0.794 | 0.4226 | Yes |
| 32 | Adamts1 | 723 | 0.793 | 0.4383 | Yes |
| 33 | Cdkn1a | 749 | 0.765 | 0.4450 | Yes |
| 34 | Jam2 | 798 | 0.707 | 0.4430 | Yes |
| 35 | H2-K1 | 818 | 0.692 | 0.4503 | Yes |
| 36 | Psmb8 | 838 | 0.678 | 0.4574 | Yes |
| 37 | Spag9 | 906 | 0.626 | 0.4474 | Yes |
| 38 | Sox7 | 942 | 0.603 | 0.4476 | Yes |
| 39 | Cfl1 | 973 | 0.581 | 0.4491 | Yes |
| 40 | Adam15 | 974 | 0.580 | 0.4606 | Yes |
| 41 | Lrrc32 | 976 | 0.580 | 0.4717 | Yes |
| 42 | H2-T23 | 1045 | 0.540 | 0.4596 | No |
| 43 | Nr4a1 | 1632 | -0.627 | 0.2759 | No |
| 44 | Gstm2 | 1848 | -0.706 | 0.2179 | No |
| 45 | Hsp90aa1 | 1965 | -0.745 | 0.1938 | No |
| 46 | Spag7 | 2169 | -0.844 | 0.1426 | No |
| 47 | Ccnd2 | 2242 | -0.887 | 0.1360 | No |
| 48 | Klf3 | 2248 | -0.890 | 0.1520 | No |
| 49 | Upp1 | 2304 | -0.929 | 0.1519 | No |
| 50 | Ly6a | 2366 | -0.979 | 0.1509 | No |
| 51 | Palmd | 2518 | -1.115 | 0.1224 | No |
| 52 | Prss23 | 2623 | -1.238 | 0.1121 | No |
| 53 | Plac8 | 2695 | -1.353 | 0.1152 | No |
Table: GSEA details [plain text format]

  

Fig 2: TABULA\_MURIS\_SENIS\_LUNG\_BRONCHIAL\_SMOOTH\_MUSCLE\_CELL\_AGEING: Random ES distribution      
 Gene set null distribution of ES for **TABULA\_MURIS\_SENIS\_LUNG\_BRONCHIAL\_SMOOTH\_MUSCLE\_CELL\_AGEING**

  
