# Supplementary material for: TMPRSS11B promotes an acidified microenvironment and immune suppression in squamous lung cancer
Source: EMBO Rep. 2025 Nov 10;26(24):6346–79. doi: 10.1038/s44319-025-00631-1 (PMC12714794; doi:10.1038/s44319-025-00631-1)
Supplement: Supplementary file 11 — Source data Fig. 6 [file 44319_2025_631_MOESM11_ESM.zip › Figure 6/6D-E/GSEA Broad Institute_low pH vs rest of the regions (high pH)/TABULA_MURIS_SENIS_LUNG_B_CELL_AGEING.html]

Details for gene set TABULA\_MURIS\_SENIS\_LUNG\_B\_CELL\_AGEING[GSEA]

|  || Dataset | Lactate high vs low\_Ranked |
| Phenotype | NoPhenotypeAvailable |
| Upregulated in class | na\_pos |
| GeneSet | TABULA\_MURIS\_SENIS\_LUNG\_B\_CELL\_AGEING |
| Enrichment Score (ES) | 0.55319935 |
| Normalized Enrichment Score (NES) | 2.9933257 |
| Nominal p-value | 0.0 |
| FDR q-value | 0.0 |
| FWER p-Value | 0.0 |
Table: GSEA Results Summary

  

Fig 1: Enrichment plot: TABULA\_MURIS\_SENIS\_LUNG\_B\_CELL\_AGEING      
 Profile of the Running ES Score & Positions of GeneSet Members on the Rank Ordered List

  

| SYMBOL | RANK IN GENE LIST | RANK METRIC SCORE | RUNNING ES | CORE ENRICHMENT || 1 | Lgals1 | 45 | 1.781 | 0.0317 | Yes |
| 2 | Napsa | 50 | 1.758 | 0.0764 | Yes |
| 3 | Evi2a | 81 | 1.620 | 0.1089 | Yes |
| 4 | Fcgr2b | 102 | 1.572 | 0.1434 | Yes |
| 5 | Vim | 128 | 1.521 | 0.1750 | Yes |
| 6 | Plek | 135 | 1.509 | 0.2125 | Yes |
| 7 | Emp3 | 191 | 1.389 | 0.2306 | Yes |
| 8 | Bcl2a1b | 241 | 1.307 | 0.2485 | Yes |
| 9 | Cd72 | 327 | 1.189 | 0.2513 | Yes |
| 10 | Lgals3 | 344 | 1.170 | 0.2767 | Yes |
| 11 | Fxyd5 | 377 | 1.133 | 0.2957 | Yes |
| 12 | B2m | 402 | 1.097 | 0.3164 | Yes |
| 13 | H2-Ab1 | 404 | 1.094 | 0.3448 | Yes |
| 14 | Anxa6 | 417 | 1.083 | 0.3692 | Yes |
| 15 | H2-Eb1 | 425 | 1.075 | 0.3950 | Yes |
| 16 | Crip1 | 431 | 1.069 | 0.4214 | Yes |
| 17 | Serpina3g | 437 | 1.063 | 0.4475 | Yes |
| 18 | H2-Aa | 465 | 1.035 | 0.4657 | Yes |
| 19 | Adgre5 | 493 | 0.998 | 0.4829 | Yes |
| 20 | Kctd12 | 532 | 0.963 | 0.4954 | Yes |
| 21 | Cyba | 554 | 0.947 | 0.5132 | Yes |
| 22 | Gns | 617 | 0.874 | 0.5155 | Yes |
| 23 | H2-D1 | 722 | 0.794 | 0.5016 | Yes |
| 24 | Grb2 | 816 | 0.695 | 0.4889 | Yes |
| 25 | H2-K1 | 818 | 0.692 | 0.5067 | Yes |
| 26 | Psmb8 | 838 | 0.678 | 0.5181 | Yes |
| 27 | Calm2 | 857 | 0.664 | 0.5295 | Yes |
| 28 | Ctsh | 890 | 0.637 | 0.5356 | Yes |
| 29 | Ptpn1 | 940 | 0.604 | 0.5351 | Yes |
| 30 | Mtss1 | 953 | 0.599 | 0.5468 | Yes |
| 31 | Ankrd44 | 980 | 0.576 | 0.5532 | Yes |
| 32 | Cd44 | 1072 | 0.523 | 0.5366 | No |
| 33 | Sik1 | 1302 | -0.541 | 0.4745 | No |
| 34 | Ier2 | 1527 | -0.593 | 0.4154 | No |
| 35 | Mtdh | 1735 | -0.667 | 0.3639 | No |
| 36 | Tnfaip8 | 2050 | -0.785 | 0.2799 | No |
| 37 | Ccnd2 | 2242 | -0.887 | 0.2395 | No |
| 38 | Ly6a | 2366 | -0.979 | 0.2242 | No |
Table: GSEA details [plain text format]

  

Fig 2: TABULA\_MURIS\_SENIS\_LUNG\_B\_CELL\_AGEING: Random ES distribution      
 Gene set null distribution of ES for **TABULA\_MURIS\_SENIS\_LUNG\_B\_CELL\_AGEING**

  
