# Supplementary material for: TMPRSS11B promotes an acidified microenvironment and immune suppression in squamous lung cancer
Source: EMBO Rep. 2025 Nov 10;26(24):6346–79. doi: 10.1038/s44319-025-00631-1 (PMC12714794; doi:10.1038/s44319-025-00631-1)
Supplement: Supplementary file 11 — Source data Fig. 6 [file 44319_2025_631_MOESM11_ESM.zip › Figure 6/6D-E/GSEA Broad Institute_low pH vs rest of the regions (high pH)/TABULA_MURIS_SENIS_MAMMARY_GLAND_B_CELL_AGEING.html]

Details for gene set TABULA\_MURIS\_SENIS\_MAMMARY\_GLAND\_B\_CELL\_AGEING[GSEA]

|  || Dataset | Lactate high vs low\_Ranked |
| Phenotype | NoPhenotypeAvailable |
| Upregulated in class | na\_pos |
| GeneSet | TABULA\_MURIS\_SENIS\_MAMMARY\_GLAND\_B\_CELL\_AGEING |
| Enrichment Score (ES) | 0.41217494 |
| Normalized Enrichment Score (NES) | 2.8357594 |
| Nominal p-value | 0.0 |
| FDR q-value | 0.0 |
| FWER p-Value | 0.0 |
Table: GSEA Results Summary

  

Fig 1: Enrichment plot: TABULA\_MURIS\_SENIS\_MAMMARY\_GLAND\_B\_CELL\_AGEING      
 Profile of the Running ES Score & Positions of GeneSet Members on the Rank Ordered List

  

| SYMBOL | RANK IN GENE LIST | RANK METRIC SCORE | RUNNING ES | CORE ENRICHMENT || 1 | Ctss | 14 | 2.088 | 0.0161 | Yes |
| 2 | Lgals1 | 45 | 1.781 | 0.0238 | Yes |
| 3 | Ctsb | 46 | 1.778 | 0.0415 | Yes |
| 4 | Napsa | 50 | 1.758 | 0.0581 | Yes |
| 5 | Psap | 59 | 1.694 | 0.0723 | Yes |
| 6 | Ly86 | 66 | 1.677 | 0.0871 | Yes |
| 7 | Hvcn1 | 73 | 1.650 | 0.1015 | Yes |
| 8 | Evi2a | 81 | 1.620 | 0.1154 | Yes |
| 9 | Cd22 | 88 | 1.612 | 0.1295 | Yes |
| 10 | Cybb | 119 | 1.535 | 0.1346 | Yes |
| 11 | Vim | 128 | 1.521 | 0.1471 | Yes |
| 12 | Atf3 | 161 | 1.444 | 0.1507 | Yes |
| 13 | Gpsm3 | 194 | 1.384 | 0.1537 | Yes |
| 14 | Syk | 200 | 1.373 | 0.1657 | Yes |
| 15 | Npc2 | 202 | 1.371 | 0.1791 | Yes |
| 16 | Evl | 204 | 1.369 | 0.1924 | Yes |
| 17 | Cd52 | 233 | 1.323 | 0.1961 | Yes |
| 18 | Ptprc | 255 | 1.283 | 0.2018 | Yes |
| 19 | Gm2a | 274 | 1.259 | 0.2083 | Yes |
| 20 | Celf2 | 276 | 1.256 | 0.2206 | Yes |
| 21 | Ikzf1 | 285 | 1.236 | 0.2302 | Yes |
| 22 | Ctsa | 302 | 1.219 | 0.2370 | Yes |
| 23 | Cd72 | 327 | 1.189 | 0.2407 | Yes |
| 24 | Rgcc | 346 | 1.169 | 0.2463 | Yes |
| 25 | H2-DMb1 | 348 | 1.167 | 0.2576 | Yes |
| 26 | Crlf2 | 352 | 1.164 | 0.2682 | Yes |
| 27 | Tmem243 | 370 | 1.140 | 0.2739 | Yes |
| 28 | Fxyd5 | 377 | 1.133 | 0.2831 | Yes |
| 29 | B2m | 402 | 1.097 | 0.2860 | Yes |
| 30 | Gimap6 | 403 | 1.097 | 0.2969 | Yes |
| 31 | Klf2 | 412 | 1.087 | 0.3051 | Yes |
| 32 | Lcp1 | 416 | 1.084 | 0.3149 | Yes |
| 33 | Anxa6 | 417 | 1.083 | 0.3258 | Yes |
| 34 | Crip1 | 431 | 1.069 | 0.3320 | Yes |
| 35 | Arhgdib | 445 | 1.049 | 0.3381 | Yes |
| 36 | Cotl1 | 447 | 1.049 | 0.3483 | Yes |
| 37 | Lsp1 | 459 | 1.039 | 0.3549 | Yes |
| 38 | Unc93b1 | 464 | 1.037 | 0.3639 | Yes |
| 39 | Acp5 | 483 | 1.008 | 0.3679 | Yes |
| 40 | Fermt3 | 506 | 0.985 | 0.3703 | Yes |
| 41 | Coro1a | 522 | 0.970 | 0.3749 | Yes |
| 42 | Kctd12 | 532 | 0.963 | 0.3815 | Yes |
| 43 | Cyba | 554 | 0.947 | 0.3838 | Yes |
| 44 | Gpx1 | 603 | 0.888 | 0.3764 | Yes |
| 45 | Dok3 | 613 | 0.876 | 0.3821 | Yes |
| 46 | Txn1 | 656 | 0.846 | 0.3763 | Yes |
| 47 | Actr3 | 665 | 0.838 | 0.3819 | Yes |
| 48 | H2-D1 | 722 | 0.794 | 0.3708 | Yes |
| 49 | Cnn2 | 727 | 0.789 | 0.3774 | Yes |
| 50 | Anxa5 | 743 | 0.769 | 0.3800 | Yes |
| 51 | Tsc22d3 | 756 | 0.757 | 0.3835 | Yes |
| 52 | Cst3 | 782 | 0.723 | 0.3822 | Yes |
| 53 | Tln1 | 804 | 0.703 | 0.3821 | Yes |
| 54 | Grb2 | 816 | 0.695 | 0.3853 | Yes |
| 55 | H2-K1 | 818 | 0.692 | 0.3919 | Yes |
| 56 | Lbh | 821 | 0.689 | 0.3981 | Yes |
| 57 | Psmb8 | 838 | 0.678 | 0.3995 | Yes |
| 58 | Actb | 856 | 0.664 | 0.4003 | Yes |
| 59 | Calm2 | 857 | 0.664 | 0.4070 | Yes |
| 60 | Ostf1 | 862 | 0.654 | 0.4122 | Yes |
| 61 | Ctsh | 890 | 0.637 | 0.4094 | No |
| 62 | Ltb | 902 | 0.628 | 0.4119 | No |
| 63 | Cfl1 | 973 | 0.581 | 0.3939 | No |
| 64 | Sh3bgrl3 | 1006 | 0.560 | 0.3887 | No |
| 65 | H2-T23 | 1045 | 0.540 | 0.3812 | No |
| 66 | Gpx3 | 1070 | 0.527 | 0.3783 | No |
| 67 | Cd44 | 1072 | 0.523 | 0.3832 | No |
| 68 | Myl12a | 1087 | 0.516 | 0.3836 | No |
| 69 | Ptpn6 | 1096 | 0.505 | 0.3859 | No |
| 70 | Calm3 | 1189 | -0.517 | 0.3598 | No |
| 71 | Selenos | 1201 | -0.520 | 0.3613 | No |
| 72 | H3f3b | 1262 | -0.534 | 0.3462 | No |
| 73 | P4hb | 1282 | -0.538 | 0.3452 | No |
| 74 | Sik1 | 1302 | -0.541 | 0.3441 | No |
| 75 | Bcl2 | 1390 | -0.561 | 0.3202 | No |
| 76 | Mt2 | 1478 | -0.581 | 0.2964 | No |
| 77 | Txndc5 | 1761 | -0.675 | 0.2073 | No |
| 78 | Tmed3 | 1790 | -0.685 | 0.2047 | No |
| 79 | Siah2 | 1909 | -0.724 | 0.1718 | No |
| 80 | Xbp1 | 1976 | -0.750 | 0.1569 | No |
| 81 | Slpi | 2011 | -0.766 | 0.1530 | No |
| 82 | Pycard | 2022 | -0.771 | 0.1573 | No |
| 83 | Tnfaip8 | 2050 | -0.785 | 0.1560 | No |
| 84 | Dnajc3 | 2133 | -0.820 | 0.1363 | No |
| 85 | Arpc5l | 2141 | -0.824 | 0.1422 | No |
| 86 | S100a6 | 2175 | -0.847 | 0.1394 | No |
| 87 | Ralgps2 | 2235 | -0.884 | 0.1282 | No |
| 88 | Pmf1 | 2267 | -0.905 | 0.1268 | No |
| 89 | Herpud1 | 2283 | -0.915 | 0.1308 | No |
| 90 | Ly6a | 2366 | -0.979 | 0.1128 | No |
| 91 | Jchain | 2385 | -0.995 | 0.1166 | No |
| 92 | Spint2 | 2539 | -1.139 | 0.0760 | No |
| 93 | Pafah1b3 | 2606 | -1.218 | 0.0658 | No |
| 94 | Dcn | 2701 | -1.362 | 0.0474 | No |
| 95 | Egr1 | 2733 | -1.440 | 0.0513 | No |
| 96 | Ly6d | 2942 | -2.300 | 0.0036 | No |
| 97 | Krt14 | 2985 | -2.898 | 0.0183 | No |
Table: GSEA details [plain text format]

  

Fig 2: TABULA\_MURIS\_SENIS\_MAMMARY\_GLAND\_B\_CELL\_AGEING: Random ES distribution      
 Gene set null distribution of ES for **TABULA\_MURIS\_SENIS\_MAMMARY\_GLAND\_B\_CELL\_AGEING**

  
