# Supplementary material for: TMPRSS11B promotes an acidified microenvironment and immune suppression in squamous lung cancer
Source: EMBO Rep. 2025 Nov 10;26(24):6346–79. doi: 10.1038/s44319-025-00631-1 (PMC12714794; doi:10.1038/s44319-025-00631-1)
Supplement: Supplementary file 11 — Source data Fig. 6 [file 44319_2025_631_MOESM11_ESM.zip › Figure 6/6D-E/GSEA Broad Institute_low pH vs rest of the regions (high pH)/TABULA_MURIS_SENIS_MAMMARY_GLAND_LUMINAL_EPITHELIAL_CELL_OF_MAMMARY_GLAND_AGEING.html]

Details for gene set TABULA\_MURIS\_SENIS\_MAMMARY\_GLAND\_LUMINAL\_EPITHELIAL\_CELL\_OF\_MAMMARY\_GLAND\_AGEING[GSEA]

|  || Dataset | Lactate high vs low\_Ranked |
| Phenotype | NoPhenotypeAvailable |
| Upregulated in class | na\_neg |
| GeneSet | TABULA\_MURIS\_SENIS\_MAMMARY\_GLAND\_LUMINAL\_EPITHELIAL\_CELL\_OF\_MAMMARY\_GLAND\_AGEING |
| Enrichment Score (ES) | -0.30294815 |
| Normalized Enrichment Score (NES) | -1.7186339 |
| Nominal p-value | 0.0036945813 |
| FDR q-value | 0.048055615 |
| FWER p-Value | 0.74 |
Table: GSEA Results Summary

  

Fig 1: Enrichment plot: TABULA\_MURIS\_SENIS\_MAMMARY\_GLAND\_LUMINAL\_EPITHELIAL\_CELL\_OF\_MAMMARY\_GLAND\_AGEING      
 Profile of the Running ES Score & Positions of GeneSet Members on the Rank Ordered List

  

| SYMBOL | RANK IN GENE LIST | RANK METRIC SCORE | RUNNING ES | CORE ENRICHMENT || 1 | Apoe | 6 | 2.177 | 0.0140 | No |
| 2 | Mfge8 | 118 | 1.535 | -0.0127 | No |
| 3 | Dpep2 | 132 | 1.513 | -0.0060 | No |
| 4 | Pxdc1 | 150 | 1.467 | -0.0010 | No |
| 5 | Emb | 159 | 1.449 | 0.0070 | No |
| 6 | Atf3 | 161 | 1.444 | 0.0173 | No |
| 7 | Cxcl15 | 190 | 1.391 | 0.0180 | No |
| 8 | Anpep | 212 | 1.361 | 0.0208 | No |
| 9 | Cd52 | 233 | 1.323 | 0.0237 | No |
| 10 | Gm2a | 274 | 1.259 | 0.0193 | No |
| 11 | Gja1 | 314 | 1.208 | 0.0149 | No |
| 12 | Timp2 | 334 | 1.180 | 0.0171 | No |
| 13 | Cd200 | 336 | 1.180 | 0.0254 | No |
| 14 | Lgals3 | 344 | 1.170 | 0.0317 | No |
| 15 | Grn | 365 | 1.146 | 0.0333 | No |
| 16 | Cd74 | 376 | 1.133 | 0.0382 | No |
| 17 | H2-Ab1 | 404 | 1.094 | 0.0370 | No |
| 18 | Klf2 | 412 | 1.087 | 0.0426 | No |
| 19 | H2-Eb1 | 425 | 1.075 | 0.0465 | No |
| 20 | Crip1 | 431 | 1.069 | 0.0526 | No |
| 21 | Ntn1 | 441 | 1.054 | 0.0573 | No |
| 22 | H2-Aa | 465 | 1.035 | 0.0571 | No |
| 23 | Icam1 | 492 | 0.999 | 0.0555 | No |
| 24 | Fth1 | 503 | 0.986 | 0.0594 | No |
| 25 | Trf | 541 | 0.957 | 0.0538 | No |
| 26 | Dusp1 | 569 | 0.935 | 0.0514 | No |
| 27 | Ltbp3 | 601 | 0.890 | 0.0474 | No |
| 28 | Hilpda | 636 | 0.860 | 0.0421 | No |
| 29 | Ogfrl1 | 684 | 0.824 | 0.0321 | No |
| 30 | Dhrs3 | 703 | 0.807 | 0.0319 | No |
| 31 | H2-D1 | 722 | 0.794 | 0.0316 | No |
| 32 | Wsb1 | 730 | 0.780 | 0.0349 | No |
| 33 | Cdkn1a | 749 | 0.765 | 0.0344 | No |
| 34 | Cebpb | 773 | 0.736 | 0.0320 | No |
| 35 | Cst3 | 782 | 0.723 | 0.0346 | No |
| 36 | Oaz2 | 789 | 0.716 | 0.0378 | No |
| 37 | Slc7a2 | 795 | 0.711 | 0.0413 | No |
| 38 | B4galnt1 | 812 | 0.699 | 0.0410 | No |
| 39 | H2-K1 | 818 | 0.692 | 0.0444 | No |
| 40 | Calm2 | 857 | 0.664 | 0.0363 | No |
| 41 | Pim1 | 859 | 0.656 | 0.0408 | No |
| 42 | Igfbp7 | 860 | 0.655 | 0.0456 | No |
| 43 | Mcl1 | 861 | 0.655 | 0.0504 | No |
| 44 | Timp3 | 959 | 0.596 | 0.0217 | No |
| 45 | Cfl1 | 973 | 0.581 | 0.0215 | No |
| 46 | Rhoj | 1049 | 0.537 | -0.0002 | No |
| 47 | Gabarapl1 | 1061 | 0.533 | -0.0000 | No |
| 48 | Gpx3 | 1070 | 0.527 | 0.0011 | No |
| 49 | Arl4c | 1105 | -0.501 | -0.0068 | No |
| 50 | Ptov1 | 1210 | -0.523 | -0.0385 | No |
| 51 | Ece1 | 1216 | -0.525 | -0.0364 | No |
| 52 | Tppp3 | 1219 | -0.525 | -0.0332 | No |
| 53 | Eif3f | 1225 | -0.527 | -0.0310 | No |
| 54 | Nfix | 1256 | -0.533 | -0.0373 | No |
| 55 | Maff | 1259 | -0.533 | -0.0341 | No |
| 56 | H3f3b | 1262 | -0.534 | -0.0308 | No |
| 57 | Fosl2 | 1283 | -0.538 | -0.0337 | No |
| 58 | Sik1 | 1302 | -0.541 | -0.0359 | No |
| 59 | Anxa1 | 1347 | -0.552 | -0.0469 | No |
| 60 | Ythdc1 | 1358 | -0.554 | -0.0462 | No |
| 61 | Fam110a | 1393 | -0.561 | -0.0537 | No |
| 62 | Btg2 | 1440 | -0.573 | -0.0652 | No |
| 63 | Ier2 | 1527 | -0.593 | -0.0903 | No |
| 64 | Bsg | 1560 | -0.604 | -0.0968 | No |
| 65 | Pbx1 | 1574 | -0.609 | -0.0967 | No |
| 66 | Nr4a1 | 1632 | -0.627 | -0.1116 | No |
| 67 | Abhd16a | 1687 | -0.647 | -0.1253 | No |
| 68 | Tnfrsf21 | 1702 | -0.654 | -0.1253 | No |
| 69 | Bcl3 | 1709 | -0.657 | -0.1225 | No |
| 70 | Aldoc | 1714 | -0.659 | -0.1190 | No |
| 71 | Tmed3 | 1790 | -0.685 | -0.1396 | No |
| 72 | Hbegf | 1860 | -0.709 | -0.1580 | No |
| 73 | Tob1 | 1942 | -0.737 | -0.1802 | No |
| 74 | Atp1a1 | 1953 | -0.741 | -0.1782 | No |
| 75 | Xbp1 | 1976 | -0.750 | -0.1802 | No |
| 76 | Mlph | 1995 | -0.757 | -0.1808 | No |
| 77 | Epha2 | 1997 | -0.758 | -0.1755 | No |
| 78 | Smagp | 2010 | -0.766 | -0.1740 | No |
| 79 | Slpi | 2011 | -0.766 | -0.1683 | No |
| 80 | Dnajb1 | 2127 | -0.818 | -0.2017 | No |
| 81 | Atp1b1 | 2139 | -0.823 | -0.1993 | No |
| 82 | Rp9 | 2194 | -0.856 | -0.2115 | No |
| 83 | Pkp4 | 2224 | -0.874 | -0.2150 | No |
| 84 | Pim3 | 2306 | -0.930 | -0.2358 | No |
| 85 | Ly6e | 2392 | -1.001 | -0.2575 | No |
| 86 | Nfkbiz | 2444 | -1.047 | -0.2673 | No |
| 87 | Irx5 | 2467 | -1.063 | -0.2669 | No |
| 88 | Fos | 2491 | -1.083 | -0.2668 | No |
| 89 | Elf3 | 2533 | -1.130 | -0.2725 | No |
| 90 | Iffo2 | 2536 | -1.136 | -0.2648 | No |
| 91 | Igfbp5 | 2556 | -1.159 | -0.2628 | No |
| 92 | Ell2 | 2637 | -1.264 | -0.2808 | No |
| 93 | Dcn | 2701 | -1.362 | -0.2923 | Yes |
| 94 | Egr1 | 2733 | -1.440 | -0.2923 | Yes |
| 95 | Ecm1 | 2738 | -1.455 | -0.2830 | Yes |
| 96 | Pam | 2741 | -1.459 | -0.2729 | Yes |
| 97 | Dsp | 2776 | -1.540 | -0.2732 | Yes |
| 98 | Klc3 | 2821 | -1.633 | -0.2762 | Yes |
| 99 | Klf5 | 2823 | -1.644 | -0.2644 | Yes |
| 100 | Camk2n1 | 2835 | -1.685 | -0.2558 | Yes |
| 101 | Kit | 2838 | -1.701 | -0.2439 | Yes |
| 102 | Sprr1a | 2841 | -1.716 | -0.2320 | Yes |
| 103 | Wfdc2 | 2861 | -1.816 | -0.2251 | Yes |
| 104 | Cbr2 | 2865 | -1.827 | -0.2127 | Yes |
| 105 | Foxa1 | 2870 | -1.858 | -0.2003 | Yes |
| 106 | Gsto1 | 2912 | -2.103 | -0.1989 | Yes |
| 107 | Stc2 | 2919 | -2.148 | -0.1851 | Yes |
| 108 | Krt15 | 2935 | -2.256 | -0.1736 | Yes |
| 109 | Ly6d | 2942 | -2.300 | -0.1587 | Yes |
| 110 | Krt14 | 2985 | -2.898 | -0.1517 | Yes |
| 111 | Krt17 | 3006 | -3.210 | -0.1349 | Yes |
| 112 | Krt5 | 3013 | -3.435 | -0.1116 | Yes |
| 113 | Cpe | 3023 | -3.802 | -0.0867 | Yes |
| 114 | Lgals7 | 3028 | -3.903 | -0.0593 | Yes |
| 115 | Krt6a | 3030 | -4.012 | -0.0301 | Yes |
| 116 | Ltf | 3035 | -4.454 | 0.0014 | Yes |
Table: GSEA details [plain text format]

  

Fig 2: TABULA\_MURIS\_SENIS\_MAMMARY\_GLAND\_LUMINAL\_EPITHELIAL\_CELL\_OF\_MAMMARY\_GLAND\_AGEING: Random ES distribution      
 Gene set null distribution of ES for **TABULA\_MURIS\_SENIS\_MAMMARY\_GLAND\_LUMINAL\_EPITHELIAL\_CELL\_OF\_MAMMARY\_GLAND\_AGEING**

  
