# Supplementary material for: TMPRSS11B promotes an acidified microenvironment and immune suppression in squamous lung cancer
Source: EMBO Rep. 2025 Nov 10;26(24):6346–79. doi: 10.1038/s44319-025-00631-1 (PMC12714794; doi:10.1038/s44319-025-00631-1)
Supplement: Supplementary file 11 — Source data Fig. 6 [file 44319_2025_631_MOESM11_ESM.zip › Figure 6/6D-E/GSEA Broad Institute_low pH vs rest of the regions (high pH)/TABULA_MURIS_SENIS_MAMMARY_GLAND_T_CELL_AGEING.html]

Details for gene set TABULA\_MURIS\_SENIS\_MAMMARY\_GLAND\_T\_CELL\_AGEING[GSEA]

|  || Dataset | Lactate high vs low\_Ranked |
| Phenotype | NoPhenotypeAvailable |
| Upregulated in class | na\_pos |
| GeneSet | TABULA\_MURIS\_SENIS\_MAMMARY\_GLAND\_T\_CELL\_AGEING |
| Enrichment Score (ES) | 0.43421224 |
| Normalized Enrichment Score (NES) | 3.2245588 |
| Nominal p-value | 0.0 |
| FDR q-value | 0.0 |
| FWER p-Value | 0.0 |
Table: GSEA Results Summary

  

Fig 1: Enrichment plot: TABULA\_MURIS\_SENIS\_MAMMARY\_GLAND\_T\_CELL\_AGEING      
 Profile of the Running ES Score & Positions of GeneSet Members on the Rank Ordered List

  

| SYMBOL | RANK IN GENE LIST | RANK METRIC SCORE | RUNNING ES | CORE ENRICHMENT || 1 | Apoe | 6 | 2.177 | 0.0147 | Yes |
| 2 | Ctss | 14 | 2.088 | 0.0283 | Yes |
| 3 | Lgals1 | 45 | 1.781 | 0.0317 | Yes |
| 4 | Ctsb | 46 | 1.778 | 0.0454 | Yes |
| 5 | Cd37 | 49 | 1.763 | 0.0582 | Yes |
| 6 | Psap | 59 | 1.694 | 0.0682 | Yes |
| 7 | Tyrobp | 83 | 1.618 | 0.0727 | Yes |
| 8 | Fcer1g | 95 | 1.597 | 0.0812 | Yes |
| 9 | Myo1g | 99 | 1.578 | 0.0923 | Yes |
| 10 | Lpxn | 100 | 1.576 | 0.1044 | Yes |
| 11 | Rgs1 | 105 | 1.567 | 0.1151 | Yes |
| 12 | Arhgap9 | 110 | 1.552 | 0.1256 | Yes |
| 13 | Vim | 128 | 1.521 | 0.1315 | Yes |
| 14 | Ctsd | 136 | 1.507 | 0.1407 | Yes |
| 15 | Itgb2 | 138 | 1.498 | 0.1518 | Yes |
| 16 | Cd48 | 146 | 1.476 | 0.1608 | Yes |
| 17 | Pxdc1 | 150 | 1.467 | 0.1710 | Yes |
| 18 | Hcls1 | 156 | 1.451 | 0.1804 | Yes |
| 19 | Atf3 | 161 | 1.444 | 0.1902 | Yes |
| 20 | Tnfrsf1b | 171 | 1.412 | 0.1979 | Yes |
| 21 | Emp3 | 191 | 1.389 | 0.2021 | Yes |
| 22 | Gpsm3 | 194 | 1.384 | 0.2120 | Yes |
| 23 | Evl | 204 | 1.369 | 0.2195 | Yes |
| 24 | Glipr1 | 208 | 1.364 | 0.2289 | Yes |
| 25 | Cd52 | 233 | 1.323 | 0.2308 | Yes |
| 26 | Bcl2a1b | 241 | 1.307 | 0.2385 | Yes |
| 27 | Ptprc | 255 | 1.283 | 0.2439 | Yes |
| 28 | Sla | 256 | 1.280 | 0.2537 | Yes |
| 29 | Gm2a | 274 | 1.259 | 0.2576 | Yes |
| 30 | Celf2 | 276 | 1.256 | 0.2669 | Yes |
| 31 | Msn | 280 | 1.243 | 0.2754 | Yes |
| 32 | Hcst | 282 | 1.242 | 0.2846 | Yes |
| 33 | Arhgap31 | 284 | 1.237 | 0.2937 | Yes |
| 34 | Ccr2 | 288 | 1.234 | 0.3022 | Yes |
| 35 | Selplg | 332 | 1.182 | 0.2965 | Yes |
| 36 | Timp2 | 334 | 1.180 | 0.3052 | Yes |
| 37 | Lgals3 | 344 | 1.170 | 0.3111 | Yes |
| 38 | Crlf2 | 352 | 1.164 | 0.3177 | Yes |
| 39 | Fxyd5 | 377 | 1.133 | 0.3181 | Yes |
| 40 | Il2rg | 380 | 1.128 | 0.3261 | Yes |
| 41 | B2m | 402 | 1.097 | 0.3274 | Yes |
| 42 | Lcp1 | 416 | 1.084 | 0.3312 | Yes |
| 43 | Anxa6 | 417 | 1.083 | 0.3395 | Yes |
| 44 | Fgl2 | 428 | 1.073 | 0.3444 | Yes |
| 45 | Crip1 | 431 | 1.069 | 0.3519 | Yes |
| 46 | Arhgdib | 445 | 1.049 | 0.3555 | Yes |
| 47 | Cotl1 | 447 | 1.049 | 0.3632 | Yes |
| 48 | Lsp1 | 459 | 1.039 | 0.3674 | Yes |
| 49 | H2-Aa | 465 | 1.035 | 0.3737 | Yes |
| 50 | Adgre5 | 493 | 0.998 | 0.3721 | Yes |
| 51 | Coro1a | 522 | 0.970 | 0.3699 | Yes |
| 52 | Kctd12 | 532 | 0.963 | 0.3742 | Yes |
| 53 | Cyba | 554 | 0.947 | 0.3743 | Yes |
| 54 | Dusp1 | 569 | 0.935 | 0.3767 | Yes |
| 55 | Ctla2a | 574 | 0.924 | 0.3824 | Yes |
| 56 | Gpx1 | 603 | 0.888 | 0.3796 | Yes |
| 57 | Hilpda | 636 | 0.860 | 0.3752 | Yes |
| 58 | Txn1 | 656 | 0.846 | 0.3752 | Yes |
| 59 | Laptm5 | 659 | 0.844 | 0.3810 | Yes |
| 60 | Actr3 | 665 | 0.838 | 0.3857 | Yes |
| 61 | Esyt1 | 678 | 0.829 | 0.3880 | Yes |
| 62 | Flna | 707 | 0.805 | 0.3846 | Yes |
| 63 | H2-D1 | 722 | 0.794 | 0.3859 | Yes |
| 64 | Cnn2 | 727 | 0.789 | 0.3906 | Yes |
| 65 | Ppp1r18 | 732 | 0.776 | 0.3952 | Yes |
| 66 | Anxa5 | 743 | 0.769 | 0.3976 | Yes |
| 67 | Cdkn1a | 749 | 0.765 | 0.4018 | Yes |
| 68 | Cebpb | 773 | 0.736 | 0.3996 | Yes |
| 69 | Wipf1 | 781 | 0.724 | 0.4027 | Yes |
| 70 | Cst3 | 782 | 0.723 | 0.4083 | Yes |
| 71 | Tln1 | 804 | 0.703 | 0.4065 | Yes |
| 72 | B4galnt1 | 812 | 0.699 | 0.4095 | Yes |
| 73 | H2-K1 | 818 | 0.692 | 0.4131 | Yes |
| 74 | Lbh | 821 | 0.689 | 0.4177 | Yes |
| 75 | Psmb8 | 838 | 0.678 | 0.4174 | Yes |
| 76 | Cxcr4 | 847 | 0.673 | 0.4198 | Yes |
| 77 | Actb | 856 | 0.664 | 0.4222 | Yes |
| 78 | Calm2 | 857 | 0.664 | 0.4273 | Yes |
| 79 | Ostf1 | 862 | 0.654 | 0.4309 | Yes |
| 80 | Ubl3 | 868 | 0.651 | 0.4342 | Yes |
| 81 | Atp6v0e | 888 | 0.638 | 0.4326 | No |
| 82 | Ltb | 902 | 0.628 | 0.4330 | No |
| 83 | S100a10 | 965 | 0.592 | 0.4162 | No |
| 84 | Cfl1 | 973 | 0.581 | 0.4183 | No |
| 85 | Serpinb9 | 982 | 0.575 | 0.4200 | No |
| 86 | F2r | 1002 | 0.562 | 0.4178 | No |
| 87 | Sh3bgrl3 | 1006 | 0.560 | 0.4210 | No |
| 88 | H2-T23 | 1045 | 0.540 | 0.4122 | No |
| 89 | Lamp1 | 1051 | 0.536 | 0.4146 | No |
| 90 | Iqgap1 | 1058 | 0.534 | 0.4166 | No |
| 91 | Gpx3 | 1070 | 0.527 | 0.4169 | No |
| 92 | Cd44 | 1072 | 0.523 | 0.4205 | No |
| 93 | Myl12a | 1087 | 0.516 | 0.4197 | No |
| 94 | Ptpn6 | 1096 | 0.505 | 0.4208 | No |
| 95 | Calm3 | 1189 | -0.517 | 0.3932 | No |
| 96 | Pigp | 1260 | -0.533 | 0.3733 | No |
| 97 | H3f3b | 1262 | -0.534 | 0.3771 | No |
| 98 | S100a11 | 1339 | -0.550 | 0.3552 | No |
| 99 | BC031181 | 1376 | -0.558 | 0.3472 | No |
| 100 | Bcl2 | 1390 | -0.561 | 0.3470 | No |
| 101 | Btg2 | 1440 | -0.573 | 0.3346 | No |
| 102 | Mt2 | 1478 | -0.581 | 0.3264 | No |
| 103 | Ier2 | 1527 | -0.593 | 0.3144 | No |
| 104 | Dap | 1535 | -0.595 | 0.3166 | No |
| 105 | Hmgb1 | 1558 | -0.603 | 0.3137 | No |
| 106 | Bsg | 1560 | -0.604 | 0.3180 | No |
| 107 | Ccdc107 | 1625 | -0.623 | 0.3008 | No |
| 108 | Nr4a1 | 1632 | -0.627 | 0.3036 | No |
| 109 | Pycard | 2022 | -0.771 | 0.1760 | No |
| 110 | Tnfaip8 | 2050 | -0.785 | 0.1728 | No |
| 111 | Lmo4 | 2166 | -0.842 | 0.1398 | No |
| 112 | S100a6 | 2175 | -0.847 | 0.1435 | No |
| 113 | Ccnd2 | 2242 | -0.887 | 0.1277 | No |
| 114 | Ly6a | 2366 | -0.979 | 0.0930 | No |
| 115 | Ly6e | 2392 | -1.001 | 0.0921 | No |
| 116 | Nfkbiz | 2444 | -1.047 | 0.0827 | No |
| 117 | Fos | 2491 | -1.083 | 0.0752 | No |
| 118 | Avpi1 | 2499 | -1.093 | 0.0812 | No |
| 119 | Glrx | 2526 | -1.119 | 0.0809 | No |
| 120 | Spint2 | 2539 | -1.139 | 0.0855 | No |
| 121 | Nr4a2 | 2700 | -1.361 | 0.0411 | No |
| 122 | Dcn | 2701 | -1.362 | 0.0516 | No |
| 123 | Pkp3 | 2706 | -1.377 | 0.0608 | No |
| 124 | Egr1 | 2733 | -1.440 | 0.0629 | No |
| 125 | Pglyrp1 | 2963 | -2.492 | 0.0035 | No |
| 126 | Krt14 | 2985 | -2.898 | 0.0185 | No |
Table: GSEA details [plain text format]

  

Fig 2: TABULA\_MURIS\_SENIS\_MAMMARY\_GLAND\_T\_CELL\_AGEING: Random ES distribution      
 Gene set null distribution of ES for **TABULA\_MURIS\_SENIS\_MAMMARY\_GLAND\_T\_CELL\_AGEING**

  
