# Supplementary material for: TMPRSS11B promotes an acidified microenvironment and immune suppression in squamous lung cancer
Source: EMBO Rep. 2025 Nov 10;26(24):6346–79. doi: 10.1038/s44319-025-00631-1 (PMC12714794; doi:10.1038/s44319-025-00631-1)
Supplement: Supplementary file 11 — Source data Fig. 6 [file 44319_2025_631_MOESM11_ESM.zip › Figure 6/6D-E/GSEA Broad Institute_low pH vs rest of the regions (high pH)/TABULA_MURIS_SENIS_MARROW_ERYTHROBLAST_AGEING.html]

Details for gene set TABULA\_MURIS\_SENIS\_MARROW\_ERYTHROBLAST\_AGEING[GSEA]

|  || Dataset | Lactate high vs low\_Ranked |
| Phenotype | NoPhenotypeAvailable |
| Upregulated in class | na\_neg |
| GeneSet | TABULA\_MURIS\_SENIS\_MARROW\_ERYTHROBLAST\_AGEING |
| Enrichment Score (ES) | -0.2730938 |
| Normalized Enrichment Score (NES) | -1.0986705 |
| Nominal p-value | 0.37142858 |
| FDR q-value | 0.62330824 |
| FWER p-Value | 1.0 |
Table: GSEA Results Summary

  

Fig 1: Enrichment plot: TABULA\_MURIS\_SENIS\_MARROW\_ERYTHROBLAST\_AGEING      
 Profile of the Running ES Score & Positions of GeneSet Members on the Rank Ordered List

  

| SYMBOL | RANK IN GENE LIST | RANK METRIC SCORE | RUNNING ES | CORE ENRICHMENT || 1 | Tyrobp | 83 | 1.618 | 0.0273 | No |
| 2 | Fcer1g | 95 | 1.597 | 0.0778 | No |
| 3 | Cd52 | 233 | 1.323 | 0.0772 | No |
| 4 | Crip1 | 431 | 1.069 | 0.0480 | No |
| 5 | Arhgdib | 445 | 1.049 | 0.0793 | No |
| 6 | Cyba | 554 | 0.947 | 0.0755 | No |
| 7 | Lcn2 | 711 | 0.802 | 0.0509 | No |
| 8 | H2-D1 | 722 | 0.794 | 0.0745 | No |
| 9 | Pim1 | 859 | 0.656 | 0.0516 | No |
| 10 | Arl6ip1 | 961 | 0.595 | 0.0383 | No |
| 11 | Sh3bgrl3 | 1006 | 0.560 | 0.0427 | No |
| 12 | Pbrm1 | 1114 | -0.502 | 0.0242 | No |
| 13 | Rack1 | 1195 | -0.519 | 0.0152 | No |
| 14 | Snrpa1 | 1335 | -0.549 | -0.0123 | No |
| 15 | S100a11 | 1339 | -0.550 | 0.0054 | No |
| 16 | Eef1b2 | 1352 | -0.553 | 0.0201 | No |
| 17 | Zbtb7a | 1424 | -0.569 | 0.0159 | No |
| 18 | Eef1g | 1501 | -0.586 | 0.0105 | No |
| 19 | Hsp90aa1 | 1965 | -0.745 | -0.1179 | No |
| 20 | Slpi | 2011 | -0.766 | -0.1069 | No |
| 21 | S100a6 | 2175 | -0.847 | -0.1322 | No |
| 22 | Jchain | 2385 | -0.995 | -0.1679 | No |
| 23 | Asns | 2703 | -1.371 | -0.2266 | Yes |
| 24 | Gstm5 | 2715 | -1.392 | -0.1831 | Yes |
| 25 | Prxl2a | 2805 | -1.599 | -0.1584 | Yes |
| 26 | Pglyrp1 | 2963 | -2.492 | -0.1261 | Yes |
| 27 | Ltf | 3035 | -4.454 | 0.0013 | Yes |
Table: GSEA details [plain text format]

  

Fig 2: TABULA\_MURIS\_SENIS\_MARROW\_ERYTHROBLAST\_AGEING: Random ES distribution      
 Gene set null distribution of ES for **TABULA\_MURIS\_SENIS\_MARROW\_ERYTHROBLAST\_AGEING**

  
