# Supplementary material for: TMPRSS11B promotes an acidified microenvironment and immune suppression in squamous lung cancer
Source: EMBO Rep. 2025 Nov 10;26(24):6346–79. doi: 10.1038/s44319-025-00631-1 (PMC12714794; doi:10.1038/s44319-025-00631-1)
Supplement: Supplementary file 11 — Source data Fig. 6 [file 44319_2025_631_MOESM11_ESM.zip › Figure 6/6D-E/GSEA Broad Institute_low pH vs rest of the regions (high pH)/TABULA_MURIS_SENIS_MARROW_MACROPHAGE_AGEING.html]

Details for gene set TABULA\_MURIS\_SENIS\_MARROW\_MACROPHAGE\_AGEING[GSEA]

|  || Dataset | Lactate high vs low\_Ranked |
| Phenotype | NoPhenotypeAvailable |
| Upregulated in class | na\_neg |
| GeneSet | TABULA\_MURIS\_SENIS\_MARROW\_MACROPHAGE\_AGEING |
| Enrichment Score (ES) | -0.25604546 |
| Normalized Enrichment Score (NES) | -1.1629422 |
| Nominal p-value | 0.28650904 |
| FDR q-value | 0.5163786 |
| FWER p-Value | 1.0 |
Table: GSEA Results Summary

  

Fig 1: Enrichment plot: TABULA\_MURIS\_SENIS\_MARROW\_MACROPHAGE\_AGEING      
 Profile of the Running ES Score & Positions of GeneSet Members on the Rank Ordered List

  

| SYMBOL | RANK IN GENE LIST | RANK METRIC SCORE | RUNNING ES | CORE ENRICHMENT || 1 | Psap | 59 | 1.694 | 0.0166 | No |
| 2 | Spi1 | 86 | 1.616 | 0.0425 | No |
| 3 | Fcer1g | 95 | 1.597 | 0.0740 | No |
| 4 | Emilin2 | 141 | 1.485 | 0.0908 | No |
| 5 | Emp3 | 191 | 1.389 | 0.1042 | No |
| 6 | Cd52 | 233 | 1.323 | 0.1188 | No |
| 7 | Lgals3 | 344 | 1.170 | 0.1071 | No |
| 8 | H2-DMb1 | 348 | 1.167 | 0.1311 | No |
| 9 | Crip1 | 431 | 1.069 | 0.1267 | No |
| 10 | Arhgdib | 445 | 1.049 | 0.1448 | No |
| 11 | Coro1a | 522 | 0.970 | 0.1402 | No |
| 12 | Ctsc | 525 | 0.966 | 0.1602 | No |
| 13 | Cyba | 554 | 0.947 | 0.1711 | No |
| 14 | Msrb1 | 629 | 0.868 | 0.1650 | No |
| 15 | Tgfbi | 651 | 0.851 | 0.1762 | No |
| 16 | Lcn2 | 711 | 0.802 | 0.1737 | No |
| 17 | Psmb8 | 838 | 0.678 | 0.1462 | No |
| 18 | Pim1 | 859 | 0.656 | 0.1536 | No |
| 19 | Slfn2 | 908 | 0.624 | 0.1510 | No |
| 20 | Ptpn1 | 940 | 0.604 | 0.1535 | No |
| 21 | Cfl1 | 973 | 0.581 | 0.1553 | No |
| 22 | Ptpn6 | 1096 | 0.505 | 0.1254 | No |
| 23 | Acp1 | 1433 | -0.572 | 0.0256 | No |
| 24 | Eif6 | 1550 | -0.601 | -0.0002 | No |
| 25 | Bsg | 1560 | -0.604 | 0.0097 | No |
| 26 | Emg1 | 1616 | -0.620 | 0.0047 | No |
| 27 | Nhp2 | 1637 | -0.629 | 0.0115 | No |
| 28 | Mtdh | 1735 | -0.667 | -0.0066 | No |
| 29 | Tmed3 | 1790 | -0.685 | -0.0100 | No |
| 30 | Siva1 | 1794 | -0.685 | 0.0037 | No |
| 31 | Hsp90aa1 | 1965 | -0.745 | -0.0370 | No |
| 32 | Slpi | 2011 | -0.766 | -0.0357 | No |
| 33 | Lmo4 | 2166 | -0.842 | -0.0690 | No |
| 34 | S100a6 | 2175 | -0.847 | -0.0535 | No |
| 35 | Elof1 | 2236 | -0.885 | -0.0546 | No |
| 36 | Jchain | 2385 | -0.995 | -0.0827 | No |
| 37 | Ifitm1 | 2906 | -2.063 | -0.2119 | Yes |
| 38 | Cd24a | 2948 | -2.343 | -0.1754 | Yes |
| 39 | Pglyrp1 | 2963 | -2.492 | -0.1268 | Yes |
| 40 | Dmkn | 2971 | -2.623 | -0.0730 | Yes |
| 41 | Ltf | 3035 | -4.454 | 0.0013 | Yes |
Table: GSEA details [plain text format]

  

Fig 2: TABULA\_MURIS\_SENIS\_MARROW\_MACROPHAGE\_AGEING: Random ES distribution      
 Gene set null distribution of ES for **TABULA\_MURIS\_SENIS\_MARROW\_MACROPHAGE\_AGEING**

  
