# Supplementary material for: TMPRSS11B promotes an acidified microenvironment and immune suppression in squamous lung cancer
Source: EMBO Rep. 2025 Nov 10;26(24):6346–79. doi: 10.1038/s44319-025-00631-1 (PMC12714794; doi:10.1038/s44319-025-00631-1)
Supplement: Supplementary file 11 — Source data Fig. 6 [file 44319_2025_631_MOESM11_ESM.zip › Figure 6/6D-E/GSEA Broad Institute_low pH vs rest of the regions (high pH)/TABULA_MURIS_SENIS_MARROW_NAIVE_T_CELL_AGEING.html]

Details for gene set TABULA\_MURIS\_SENIS\_MARROW\_NAIVE\_T\_CELL\_AGEING[GSEA]

|  || Dataset | Lactate high vs low\_Ranked |
| Phenotype | NoPhenotypeAvailable |
| Upregulated in class | na\_neg |
| GeneSet | TABULA\_MURIS\_SENIS\_MARROW\_NAIVE\_T\_CELL\_AGEING |
| Enrichment Score (ES) | -0.33025205 |
| Normalized Enrichment Score (NES) | -1.2299395 |
| Nominal p-value | 0.24179104 |
| FDR q-value | 0.4390114 |
| FWER p-Value | 1.0 |
Table: GSEA Results Summary

  

Fig 1: Enrichment plot: TABULA\_MURIS\_SENIS\_MARROW\_NAIVE\_T\_CELL\_AGEING      
 Profile of the Running ES Score & Positions of GeneSet Members on the Rank Ordered List

  

| SYMBOL | RANK IN GENE LIST | RANK METRIC SCORE | RUNNING ES | CORE ENRICHMENT || 1 | Tyrobp | 83 | 1.618 | 0.0325 | Yes |
| 2 | Fcer1g | 95 | 1.597 | 0.0881 | Yes |
| 3 | Cd52 | 233 | 1.323 | 0.0917 | Yes |
| 4 | Hcst | 282 | 1.242 | 0.1219 | Yes |
| 5 | Lgals3 | 344 | 1.170 | 0.1450 | Yes |
| 6 | Cd74 | 376 | 1.133 | 0.1768 | Yes |
| 7 | Fxyd5 | 377 | 1.133 | 0.2188 | Yes |
| 8 | Crip1 | 431 | 1.069 | 0.2409 | Yes |
| 9 | Cyba | 554 | 0.947 | 0.2356 | Yes |
| 10 | Lcn2 | 711 | 0.802 | 0.2137 | Yes |
| 11 | H2-D1 | 722 | 0.794 | 0.2398 | Yes |
| 12 | Ccl6 | 772 | 0.736 | 0.2508 | Yes |
| 13 | Atp6v1g1 | 1020 | 0.553 | 0.1895 | No |
| 14 | Atg101 | 1782 | -0.681 | -0.0372 | No |
| 15 | Slpi | 2011 | -0.766 | -0.0843 | No |
| 16 | S100a6 | 2175 | -0.847 | -0.1069 | No |
| 17 | Jchain | 2385 | -0.995 | -0.1392 | No |
| 18 | Pglyrp1 | 2963 | -2.492 | -0.2379 | No |
| 19 | Dmkn | 2971 | -2.623 | -0.1429 | No |
| 20 | Ltf | 3035 | -4.454 | 0.0013 | No |
Table: GSEA details [plain text format]

  

Fig 2: TABULA\_MURIS\_SENIS\_MARROW\_NAIVE\_T\_CELL\_AGEING: Random ES distribution      
 Gene set null distribution of ES for **TABULA\_MURIS\_SENIS\_MARROW\_NAIVE\_T\_CELL\_AGEING**

  
