# Supplementary material for: TMPRSS11B promotes an acidified microenvironment and immune suppression in squamous lung cancer
Source: EMBO Rep. 2025 Nov 10;26(24):6346–79. doi: 10.1038/s44319-025-00631-1 (PMC12714794; doi:10.1038/s44319-025-00631-1)
Supplement: Supplementary file 11 — Source data Fig. 6 [file 44319_2025_631_MOESM11_ESM.zip › Figure 6/6D-E/GSEA Broad Institute_low pH vs rest of the regions (high pH)/TABULA_MURIS_SENIS_PANCREAS_PANCREATIC_POLYPEPTIDE_CELL_AGEING.html]

Details for gene set TABULA\_MURIS\_SENIS\_PANCREAS\_PANCREATIC\_POLYPEPTIDE\_CELL\_AGEING[GSEA]

|  || Dataset | Lactate high vs low\_Ranked |
| Phenotype | NoPhenotypeAvailable |
| Upregulated in class | na\_neg |
| GeneSet | TABULA\_MURIS\_SENIS\_PANCREAS\_PANCREATIC\_POLYPEPTIDE\_CELL\_AGEING |
| Enrichment Score (ES) | -0.2971969 |
| Normalized Enrichment Score (NES) | -1.1937295 |
| Nominal p-value | 0.23857144 |
| FDR q-value | 0.48275393 |
| FWER p-Value | 1.0 |
Table: GSEA Results Summary

  

Fig 1: Enrichment plot: TABULA\_MURIS\_SENIS\_PANCREAS\_PANCREATIC\_POLYPEPTIDE\_CELL\_AGEING      
 Profile of the Running ES Score & Positions of GeneSet Members on the Rank Ordered List

  

| SYMBOL | RANK IN GENE LIST | RANK METRIC SCORE | RUNNING ES | CORE ENRICHMENT || 1 | Tceal9 | 409 | 1.090 | -0.0933 | No |
| 2 | Cotl1 | 447 | 1.049 | -0.0647 | No |
| 3 | Dusp1 | 569 | 0.935 | -0.0685 | No |
| 4 | Calm2 | 857 | 0.664 | -0.1379 | No |
| 5 | Cfl1 | 973 | 0.581 | -0.1534 | No |
| 6 | Rab12 | 978 | 0.576 | -0.1323 | No |
| 7 | Selenos | 1201 | -0.520 | -0.1858 | No |
| 8 | Ilkap | 1246 | -0.531 | -0.1797 | No |
| 9 | H3f3b | 1262 | -0.534 | -0.1639 | No |
| 10 | Sdr39u1 | 1295 | -0.540 | -0.1535 | No |
| 11 | F8a | 1337 | -0.550 | -0.1457 | No |
| 12 | Ier2 | 1527 | -0.593 | -0.1853 | No |
| 13 | Bsg | 1560 | -0.604 | -0.1724 | No |
| 14 | Ppa1 | 1719 | -0.661 | -0.1991 | No |
| 15 | Lsr | 1792 | -0.685 | -0.1964 | No |
| 16 | Trappc5 | 1980 | -0.750 | -0.2292 | No |
| 17 | Mmadhc | 2088 | -0.803 | -0.2334 | No |
| 18 | Dnajb1 | 2127 | -0.818 | -0.2142 | No |
| 19 | Erlec1 | 2378 | -0.991 | -0.2586 | Yes |
| 20 | Gal3st1 | 2470 | -1.064 | -0.2473 | Yes |
| 21 | Fos | 2491 | -1.083 | -0.2118 | Yes |
| 22 | Zbtb7c | 2555 | -1.152 | -0.1878 | Yes |
| 23 | Casz1 | 2607 | -1.220 | -0.1572 | Yes |
| 24 | Mphosph6 | 2620 | -1.232 | -0.1132 | Yes |
| 25 | Bace2 | 2625 | -1.240 | -0.0662 | Yes |
| 26 | Cfap298 | 2744 | -1.464 | -0.0483 | Yes |
| 27 | Klc3 | 2821 | -1.633 | -0.0099 | Yes |
| 28 | Lrig1 | 2913 | -2.103 | 0.0418 | Yes |
Table: GSEA details [plain text format]

  

Fig 2: TABULA\_MURIS\_SENIS\_PANCREAS\_PANCREATIC\_POLYPEPTIDE\_CELL\_AGEING: Random ES distribution      
 Gene set null distribution of ES for **TABULA\_MURIS\_SENIS\_PANCREAS\_PANCREATIC\_POLYPEPTIDE\_CELL\_AGEING**

  
