# Supplementary material for: TMPRSS11B promotes an acidified microenvironment and immune suppression in squamous lung cancer
Source: EMBO Rep. 2025 Nov 10;26(24):6346–79. doi: 10.1038/s44319-025-00631-1 (PMC12714794; doi:10.1038/s44319-025-00631-1)
Supplement: Supplementary file 11 — Source data Fig. 6 [file 44319_2025_631_MOESM11_ESM.zip › Figure 6/6D-E/GSEA Broad Institute_low pH vs rest of the regions (high pH)/TABULA_MURIS_SENIS_SKIN_BASAL_CELL_OF_EPIDERMIS_AGEING.html]

Details for gene set TABULA\_MURIS\_SENIS\_SKIN\_BASAL\_CELL\_OF\_EPIDERMIS\_AGEING[GSEA]

|  || Dataset | Lactate high vs low\_Ranked |
| Phenotype | NoPhenotypeAvailable |
| Upregulated in class | na\_neg |
| GeneSet | TABULA\_MURIS\_SENIS\_SKIN\_BASAL\_CELL\_OF\_EPIDERMIS\_AGEING |
| Enrichment Score (ES) | -0.20307966 |
| Normalized Enrichment Score (NES) | -1.2468038 |
| Nominal p-value | 0.12666667 |
| FDR q-value | 0.43293586 |
| FWER p-Value | 1.0 |
Table: GSEA Results Summary

  

Fig 1: Enrichment plot: TABULA\_MURIS\_SENIS\_SKIN\_BASAL\_CELL\_OF\_EPIDERMIS\_AGEING      
 Profile of the Running ES Score & Positions of GeneSet Members on the Rank Ordered List

  

| SYMBOL | RANK IN GENE LIST | RANK METRIC SCORE | RUNNING ES | CORE ENRICHMENT || 1 | Apoe | 6 | 2.177 | 0.0084 | No |
| 2 | Apoc1 | 40 | 1.811 | 0.0054 | No |
| 3 | Napsa | 50 | 1.758 | 0.0107 | No |
| 4 | Ly86 | 66 | 1.677 | 0.0135 | No |
| 5 | Fcgr2b | 102 | 1.572 | 0.0087 | No |
| 6 | Cryab | 158 | 1.449 | -0.0038 | No |
| 7 | Vsir | 170 | 1.421 | -0.0008 | No |
| 8 | Npc2 | 202 | 1.371 | -0.0052 | No |
| 9 | Ctsz | 242 | 1.303 | -0.0127 | No |
| 10 | Dpysl2 | 298 | 1.225 | -0.0262 | No |
| 11 | Mgll | 316 | 1.208 | -0.0264 | No |
| 12 | Sftpa1 | 326 | 1.190 | -0.0238 | No |
| 13 | Lgals3 | 344 | 1.170 | -0.0242 | No |
| 14 | Cd74 | 376 | 1.133 | -0.0297 | No |
| 15 | Fxyd5 | 377 | 1.133 | -0.0243 | No |
| 16 | B2m | 402 | 1.097 | -0.0275 | No |
| 17 | H2-Ab1 | 404 | 1.094 | -0.0225 | No |
| 18 | Tceal9 | 409 | 1.090 | -0.0187 | No |
| 19 | Klf2 | 412 | 1.087 | -0.0142 | No |
| 20 | H2-DMa | 418 | 1.083 | -0.0107 | No |
| 21 | H2-Eb1 | 425 | 1.075 | -0.0077 | No |
| 22 | Arhgdib | 445 | 1.049 | -0.0093 | No |
| 23 | Cotl1 | 447 | 1.049 | -0.0046 | No |
| 24 | Sftpc | 453 | 1.044 | -0.0014 | No |
| 25 | Sparc | 460 | 1.038 | 0.0015 | No |
| 26 | Il11ra1 | 461 | 1.038 | 0.0065 | No |
| 27 | H2-Aa | 465 | 1.035 | 0.0104 | No |
| 28 | Fth1 | 503 | 0.986 | 0.0021 | No |
| 29 | Coro1a | 522 | 0.970 | 0.0004 | No |
| 30 | Rcsd1 | 555 | 0.947 | -0.0063 | No |
| 31 | Cib2 | 585 | 0.912 | -0.0122 | No |
| 32 | Clic3 | 594 | 0.901 | -0.0107 | No |
| 33 | Gpx1 | 603 | 0.888 | -0.0092 | No |
| 34 | Gimap1 | 614 | 0.876 | -0.0085 | No |
| 35 | Hilpda | 636 | 0.860 | -0.0118 | No |
| 36 | Metrnl | 714 | 0.799 | -0.0352 | No |
| 37 | Cd63 | 724 | 0.793 | -0.0345 | No |
| 38 | Cdkn1a | 749 | 0.765 | -0.0393 | No |
| 39 | Grap | 780 | 0.725 | -0.0465 | No |
| 40 | Cst3 | 782 | 0.723 | -0.0433 | No |
| 41 | Rras | 788 | 0.717 | -0.0416 | No |
| 42 | Rgs4 | 791 | 0.714 | -0.0389 | No |
| 43 | Grb2 | 816 | 0.695 | -0.0440 | No |
| 44 | H2-K1 | 818 | 0.692 | -0.0411 | No |
| 45 | Rgs10 | 837 | 0.678 | -0.0442 | No |
| 46 | Psmb8 | 838 | 0.678 | -0.0409 | No |
| 47 | Calm2 | 857 | 0.664 | -0.0440 | No |
| 48 | Igfbp7 | 860 | 0.655 | -0.0416 | No |
| 49 | Ostf1 | 862 | 0.654 | -0.0388 | No |
| 50 | Pdlim2 | 865 | 0.653 | -0.0364 | No |
| 51 | Bgn | 870 | 0.650 | -0.0346 | No |
| 52 | Atp6v0e | 888 | 0.638 | -0.0376 | No |
| 53 | Ctsh | 890 | 0.637 | -0.0349 | No |
| 54 | Ndufa4l2 | 900 | 0.629 | -0.0350 | No |
| 55 | Ltb | 902 | 0.628 | -0.0323 | No |
| 56 | Iscu | 905 | 0.626 | -0.0300 | No |
| 57 | Dtnbp1 | 910 | 0.623 | -0.0284 | No |
| 58 | Emp2 | 915 | 0.618 | -0.0269 | No |
| 59 | Cebpa | 923 | 0.615 | -0.0264 | No |
| 60 | Plpp3 | 938 | 0.605 | -0.0284 | No |
| 61 | Ptpn1 | 940 | 0.604 | -0.0259 | No |
| 62 | Fam89b | 947 | 0.602 | -0.0251 | No |
| 63 | Cfl1 | 973 | 0.581 | -0.0311 | No |
| 64 | Erp29 | 1003 | 0.562 | -0.0387 | No |
| 65 | Sh3bgrl3 | 1006 | 0.560 | -0.0367 | No |
| 66 | Atp6v1g1 | 1020 | 0.553 | -0.0386 | No |
| 67 | Ninj1 | 1025 | 0.551 | -0.0374 | No |
| 68 | Slc6a8 | 1041 | 0.543 | -0.0401 | No |
| 69 | Cstb | 1056 | 0.535 | -0.0424 | No |
| 70 | Srp14 | 1077 | 0.519 | -0.0470 | No |
| 71 | Myl12a | 1087 | 0.516 | -0.0477 | No |
| 72 | Lin37 | 1151 | -0.509 | -0.0675 | No |
| 73 | Zfpl1 | 1161 | -0.511 | -0.0682 | No |
| 74 | Mettl5 | 1166 | -0.512 | -0.0672 | No |
| 75 | Gid8 | 1178 | -0.514 | -0.0686 | No |
| 76 | Fbl | 1188 | -0.517 | -0.0693 | No |
| 77 | Rack1 | 1195 | -0.519 | -0.0689 | No |
| 78 | Selenos | 1201 | -0.520 | -0.0682 | No |
| 79 | Ece1 | 1216 | -0.525 | -0.0706 | No |
| 80 | Eif3f | 1225 | -0.527 | -0.0709 | No |
| 81 | 2510002D24Rik | 1230 | -0.528 | -0.0697 | No |
| 82 | Ift27 | 1244 | -0.531 | -0.0718 | No |
| 83 | Nfix | 1256 | -0.533 | -0.0731 | No |
| 84 | Fam162a | 1258 | -0.533 | -0.0709 | No |
| 85 | Bag1 | 1264 | -0.534 | -0.0701 | No |
| 86 | Rarg | 1287 | -0.538 | -0.0753 | No |
| 87 | Clpp | 1300 | -0.540 | -0.0769 | No |
| 88 | Bmyc | 1305 | -0.542 | -0.0757 | No |
| 89 | S100a11 | 1339 | -0.550 | -0.0847 | No |
| 90 | Eef1b2 | 1352 | -0.553 | -0.0863 | No |
| 91 | Senp6 | 1364 | -0.556 | -0.0875 | No |
| 92 | Dbndd2 | 1375 | -0.558 | -0.0883 | No |
| 93 | BC031181 | 1376 | -0.558 | -0.0856 | No |
| 94 | Ddr1 | 1387 | -0.561 | -0.0865 | No |
| 95 | Fam110a | 1393 | -0.561 | -0.0855 | No |
| 96 | Bod1 | 1398 | -0.562 | -0.0843 | No |
| 97 | Thap3 | 1413 | -0.566 | -0.0865 | No |
| 98 | Nudt14 | 1414 | -0.566 | -0.0837 | No |
| 99 | Mpst | 1415 | -0.566 | -0.0810 | No |
| 100 | Uqcc3 | 1456 | -0.576 | -0.0924 | No |
| 101 | Ostc | 1457 | -0.576 | -0.0896 | No |
| 102 | Dpm1 | 1459 | -0.576 | -0.0872 | No |
| 103 | Mbp | 1464 | -0.577 | -0.0858 | No |
| 104 | Dexi | 1471 | -0.580 | -0.0851 | No |
| 105 | Zfand2b | 1522 | -0.592 | -0.1000 | No |
| 106 | Ier2 | 1527 | -0.593 | -0.0985 | No |
| 107 | Commd9 | 1540 | -0.597 | -0.0999 | No |
| 108 | Uchl3 | 1551 | -0.602 | -0.1005 | No |
| 109 | Bsg | 1560 | -0.604 | -0.1004 | No |
| 110 | Fabp5 | 1603 | -0.617 | -0.1123 | No |
| 111 | Aimp2 | 1605 | -0.617 | -0.1097 | No |
| 112 | Aimp1 | 1609 | -0.618 | -0.1078 | No |
| 113 | Ndufv2 | 1610 | -0.618 | -0.1048 | No |
| 114 | Zfp503 | 1619 | -0.622 | -0.1046 | No |
| 115 | Ccdc107 | 1625 | -0.623 | -0.1034 | No |
| 116 | Smim20 | 1628 | -0.625 | -0.1011 | No |
| 117 | Rbm26 | 1633 | -0.628 | -0.0995 | No |
| 118 | Nhp2 | 1637 | -0.629 | -0.0975 | No |
| 119 | 2610528J11Rik | 1670 | -0.639 | -0.1058 | No |
| 120 | Mea1 | 1688 | -0.648 | -0.1086 | No |
| 121 | Eef1d | 1738 | -0.668 | -0.1227 | No |
| 122 | Nop16 | 1748 | -0.672 | -0.1227 | No |
| 123 | Tmem147 | 1757 | -0.673 | -0.1223 | No |
| 124 | Echdc2 | 1769 | -0.679 | -0.1229 | No |
| 125 | Atg101 | 1782 | -0.681 | -0.1238 | No |
| 126 | Tmed3 | 1790 | -0.685 | -0.1230 | No |
| 127 | Siva1 | 1794 | -0.685 | -0.1208 | No |
| 128 | S100a16 | 1801 | -0.687 | -0.1196 | No |
| 129 | Cib1 | 1832 | -0.701 | -0.1268 | No |
| 130 | Tmem205 | 1833 | -0.702 | -0.1234 | No |
| 131 | Hbegf | 1860 | -0.709 | -0.1292 | No |
| 132 | Eci1 | 1871 | -0.711 | -0.1293 | No |
| 133 | Pmm1 | 1922 | -0.729 | -0.1435 | No |
| 134 | Akr1c13 | 1950 | -0.740 | -0.1495 | No |
| 135 | Smagp | 2010 | -0.766 | -0.1666 | No |
| 136 | Slpi | 2011 | -0.766 | -0.1629 | No |
| 137 | Endog | 2012 | -0.767 | -0.1592 | No |
| 138 | Pycard | 2022 | -0.771 | -0.1587 | No |
| 139 | Pgrmc2 | 2031 | -0.775 | -0.1578 | No |
| 140 | Rab25 | 2032 | -0.776 | -0.1541 | No |
| 141 | Pop7 | 2037 | -0.777 | -0.1517 | No |
| 142 | Gtf2a2 | 2095 | -0.804 | -0.1680 | No |
| 143 | Tm2d3 | 2122 | -0.816 | -0.1733 | No |
| 144 | Cmtm8 | 2124 | -0.816 | -0.1697 | No |
| 145 | Apex1 | 2125 | -0.816 | -0.1657 | No |
| 146 | Arpc5l | 2141 | -0.824 | -0.1671 | No |
| 147 | Cbr3 | 2160 | -0.834 | -0.1694 | No |
| 148 | Macrod1 | 2161 | -0.838 | -0.1654 | No |
| 149 | Lmo4 | 2166 | -0.842 | -0.1627 | No |
| 150 | Spag7 | 2169 | -0.844 | -0.1594 | No |
| 151 | Rp9 | 2194 | -0.856 | -0.1637 | No |
| 152 | Gtf3c6 | 2196 | -0.857 | -0.1600 | No |
| 153 | Elof1 | 2236 | -0.885 | -0.1695 | No |
| 154 | Nectin1 | 2239 | -0.886 | -0.1659 | No |
| 155 | Ccnd2 | 2242 | -0.887 | -0.1624 | No |
| 156 | Grhpr | 2258 | -0.898 | -0.1633 | No |
| 157 | Pmf1 | 2267 | -0.905 | -0.1618 | No |
| 158 | Hcfc1r1 | 2289 | -0.918 | -0.1648 | No |
| 159 | Psph | 2315 | -0.934 | -0.1691 | No |
| 160 | Cdc42ep5 | 2317 | -0.935 | -0.1650 | No |
| 161 | Nans | 2346 | -0.959 | -0.1703 | No |
| 162 | Cldn3 | 2379 | -0.991 | -0.1768 | No |
| 163 | 2310039H08Rik | 2388 | -0.997 | -0.1748 | No |
| 164 | Ly6e | 2392 | -1.001 | -0.1711 | No |
| 165 | Ssbp3 | 2456 | -1.057 | -0.1882 | No |
| 166 | Avpi1 | 2499 | -1.093 | -0.1978 | Yes |
| 167 | Acbd4 | 2507 | -1.099 | -0.1950 | Yes |
| 168 | Bad | 2522 | -1.117 | -0.1946 | Yes |
| 169 | Iffo2 | 2536 | -1.136 | -0.1937 | Yes |
| 170 | Spint2 | 2539 | -1.139 | -0.1889 | Yes |
| 171 | Zfp703 | 2541 | -1.141 | -0.1838 | Yes |
| 172 | Nupr1 | 2565 | -1.167 | -0.1863 | Yes |
| 173 | S100a14 | 2580 | -1.185 | -0.1855 | Yes |
| 174 | Fahd1 | 2588 | -1.195 | -0.1822 | Yes |
| 175 | Pafah1b3 | 2606 | -1.218 | -0.1824 | Yes |
| 176 | Casz1 | 2607 | -1.220 | -0.1765 | Yes |
| 177 | Wdr74 | 2617 | -1.230 | -0.1738 | Yes |
| 178 | Urah | 2658 | -1.303 | -0.1816 | Yes |
| 179 | Ppp1r13l | 2692 | -1.350 | -0.1868 | Yes |
| 180 | Plac8 | 2695 | -1.353 | -0.1810 | Yes |
| 181 | Gpatch4 | 2696 | -1.354 | -0.1745 | Yes |
| 182 | Dcn | 2701 | -1.362 | -0.1693 | Yes |
| 183 | Gstm5 | 2715 | -1.392 | -0.1672 | Yes |
| 184 | Tmem45b | 2732 | -1.438 | -0.1659 | Yes |
| 185 | Cfap298 | 2744 | -1.464 | -0.1628 | Yes |
| 186 | Krt7 | 2749 | -1.487 | -0.1570 | Yes |
| 187 | Plet1 | 2762 | -1.513 | -0.1540 | Yes |
| 188 | Rac3 | 2783 | -1.553 | -0.1536 | Yes |
| 189 | Fermt1 | 2795 | -1.579 | -0.1498 | Yes |
| 190 | Pdzk1ip1 | 2812 | -1.614 | -0.1477 | Yes |
| 191 | Fxyd3 | 2815 | -1.624 | -0.1406 | Yes |
| 192 | Klc3 | 2821 | -1.633 | -0.1345 | Yes |
| 193 | Klf5 | 2823 | -1.644 | -0.1269 | Yes |
| 194 | Sprr1a | 2841 | -1.716 | -0.1247 | Yes |
| 195 | Wfdc2 | 2861 | -1.816 | -0.1226 | Yes |
| 196 | Wnt4 | 2873 | -1.869 | -0.1175 | Yes |
| 197 | Hebp2 | 2874 | -1.871 | -0.1085 | Yes |
| 198 | Cela1 | 2888 | -1.928 | -0.1038 | Yes |
| 199 | Gsto1 | 2912 | -2.103 | -0.1018 | Yes |
| 200 | Fgfbp1 | 2921 | -2.159 | -0.0943 | Yes |
| 201 | Ly6g6c | 2922 | -2.162 | -0.0838 | Yes |
| 202 | Evpl | 2925 | -2.172 | -0.0741 | Yes |
| 203 | Tmem45a | 2934 | -2.244 | -0.0661 | Yes |
| 204 | Ly6d | 2942 | -2.300 | -0.0575 | Yes |
| 205 | Zfp750 | 2969 | -2.612 | -0.0541 | Yes |
| 206 | Dmkn | 2971 | -2.623 | -0.0418 | Yes |
| 207 | Krtdap | 2997 | -3.095 | -0.0358 | Yes |
| 208 | Lypd3 | 3005 | -3.177 | -0.0229 | Yes |
| 209 | Krt17 | 3006 | -3.210 | -0.0075 | Yes |
| 210 | Lgals7 | 3028 | -3.903 | 0.0039 | Yes |
Table: GSEA details [plain text format]

  

Fig 2: TABULA\_MURIS\_SENIS\_SKIN\_BASAL\_CELL\_OF\_EPIDERMIS\_AGEING: Random ES distribution      
 Gene set null distribution of ES for **TABULA\_MURIS\_SENIS\_SKIN\_BASAL\_CELL\_OF\_EPIDERMIS\_AGEING**

  
