# Supplementary material for: TMPRSS11B promotes an acidified microenvironment and immune suppression in squamous lung cancer
Source: EMBO Rep. 2025 Nov 10;26(24):6346–79. doi: 10.1038/s44319-025-00631-1 (PMC12714794; doi:10.1038/s44319-025-00631-1)
Supplement: Supplementary file 11 — Source data Fig. 6 [file 44319_2025_631_MOESM11_ESM.zip › Figure 6/6D-E/GSEA Broad Institute_low pH vs rest of the regions (high pH)/TABULA_MURIS_SENIS_SKIN_EPIDERMAL_CELL_AGEING.html]

Details for gene set TABULA\_MURIS\_SENIS\_SKIN\_EPIDERMAL\_CELL\_AGEING[GSEA]

|  || Dataset | Lactate high vs low\_Ranked |
| Phenotype | NoPhenotypeAvailable |
| Upregulated in class | na\_neg |
| GeneSet | TABULA\_MURIS\_SENIS\_SKIN\_EPIDERMAL\_CELL\_AGEING |
| Enrichment Score (ES) | -0.21699953 |
| Normalized Enrichment Score (NES) | -1.2841402 |
| Nominal p-value | 0.1152968 |
| FDR q-value | 0.3901891 |
| FWER p-Value | 1.0 |
Table: GSEA Results Summary

  

Fig 1: Enrichment plot: TABULA\_MURIS\_SENIS\_SKIN\_EPIDERMAL\_CELL\_AGEING      
 Profile of the Running ES Score & Positions of GeneSet Members on the Rank Ordered List

  

| SYMBOL | RANK IN GENE LIST | RANK METRIC SCORE | RUNNING ES | CORE ENRICHMENT || 1 | Cd37 | 49 | 1.763 | -0.0061 | No |
| 2 | Evi2a | 81 | 1.620 | -0.0068 | No |
| 3 | Tyrobp | 83 | 1.618 | 0.0029 | No |
| 4 | Fcer1g | 95 | 1.597 | 0.0089 | No |
| 5 | Rgs1 | 105 | 1.567 | 0.0155 | No |
| 6 | Vim | 128 | 1.521 | 0.0173 | No |
| 7 | Cd53 | 139 | 1.488 | 0.0230 | No |
| 8 | Col6a3 | 175 | 1.408 | 0.0196 | No |
| 9 | Npc2 | 202 | 1.371 | 0.0191 | No |
| 10 | Cldn5 | 227 | 1.338 | 0.0190 | No |
| 11 | Ctsz | 242 | 1.303 | 0.0222 | No |
| 12 | Ptprc | 255 | 1.283 | 0.0260 | No |
| 13 | Lgals3 | 344 | 1.170 | 0.0027 | No |
| 14 | Cd74 | 376 | 1.133 | -0.0010 | No |
| 15 | Fstl3 | 379 | 1.129 | 0.0053 | No |
| 16 | B2m | 402 | 1.097 | 0.0044 | No |
| 17 | H2-Ab1 | 404 | 1.094 | 0.0109 | No |
| 18 | Egfl7 | 406 | 1.093 | 0.0173 | No |
| 19 | H2-DMa | 418 | 1.083 | 0.0202 | No |
| 20 | Slc10a6 | 424 | 1.075 | 0.0251 | No |
| 21 | H2-Eb1 | 425 | 1.075 | 0.0318 | No |
| 22 | Sparc | 460 | 1.038 | 0.0264 | No |
| 23 | Unc93b1 | 464 | 1.037 | 0.0318 | No |
| 24 | H2-Aa | 465 | 1.035 | 0.0382 | No |
| 25 | Fermt3 | 506 | 0.985 | 0.0304 | No |
| 26 | Rassf4 | 560 | 0.942 | 0.0178 | No |
| 27 | Lypd8 | 561 | 0.941 | 0.0237 | No |
| 28 | Dhrs3 | 703 | 0.807 | -0.0203 | No |
| 29 | Metrnl | 714 | 0.799 | -0.0188 | No |
| 30 | H2-D1 | 722 | 0.794 | -0.0163 | No |
| 31 | Cd63 | 724 | 0.793 | -0.0117 | No |
| 32 | Grina | 726 | 0.791 | -0.0072 | No |
| 33 | Cdkn1a | 749 | 0.765 | -0.0101 | No |
| 34 | Ccl6 | 772 | 0.736 | -0.0132 | No |
| 35 | Oaz2 | 789 | 0.716 | -0.0143 | No |
| 36 | Notch3 | 809 | 0.700 | -0.0165 | No |
| 37 | H2-K1 | 818 | 0.692 | -0.0150 | No |
| 38 | Plgrkt | 836 | 0.679 | -0.0167 | No |
| 39 | Psmb8 | 838 | 0.678 | -0.0129 | No |
| 40 | Calm2 | 857 | 0.664 | -0.0150 | No |
| 41 | Igfbp7 | 860 | 0.655 | -0.0116 | No |
| 42 | Pdlim2 | 865 | 0.653 | -0.0090 | No |
| 43 | Mgp | 898 | 0.630 | -0.0162 | No |
| 44 | Ndufa4l2 | 900 | 0.629 | -0.0126 | No |
| 45 | Ltb | 902 | 0.628 | -0.0091 | No |
| 46 | Dtnbp1 | 910 | 0.623 | -0.0077 | No |
| 47 | Cebpa | 923 | 0.615 | -0.0080 | No |
| 48 | Ptpn1 | 940 | 0.604 | -0.0098 | No |
| 49 | Fam89b | 947 | 0.602 | -0.0082 | No |
| 50 | Cfl1 | 973 | 0.581 | -0.0133 | No |
| 51 | Erp29 | 1003 | 0.562 | -0.0198 | No |
| 52 | Ninj1 | 1025 | 0.551 | -0.0237 | No |
| 53 | Slc6a8 | 1041 | 0.543 | -0.0256 | No |
| 54 | Gabarapl1 | 1061 | 0.533 | -0.0289 | No |
| 55 | Brd3 | 1118 | -0.503 | -0.0452 | No |
| 56 | Ctcf | 1124 | -0.504 | -0.0438 | No |
| 57 | Sdcbp2 | 1160 | -0.511 | -0.0528 | No |
| 58 | Zfpl1 | 1161 | -0.511 | -0.0496 | No |
| 59 | Mettl5 | 1166 | -0.512 | -0.0478 | No |
| 60 | Fbl | 1188 | -0.517 | -0.0519 | No |
| 61 | Rack1 | 1195 | -0.519 | -0.0508 | No |
| 62 | Ptov1 | 1210 | -0.523 | -0.0524 | No |
| 63 | Ece1 | 1216 | -0.525 | -0.0509 | No |
| 64 | Eif3f | 1225 | -0.527 | -0.0504 | No |
| 65 | 2510002D24Rik | 1230 | -0.528 | -0.0485 | No |
| 66 | Ilkap | 1246 | -0.531 | -0.0504 | No |
| 67 | Nfix | 1256 | -0.533 | -0.0503 | No |
| 68 | Bag1 | 1264 | -0.534 | -0.0494 | No |
| 69 | Fosl2 | 1283 | -0.538 | -0.0523 | No |
| 70 | Rarg | 1287 | -0.538 | -0.0500 | No |
| 71 | Abhd14b | 1298 | -0.540 | -0.0501 | No |
| 72 | Eef1b2 | 1352 | -0.553 | -0.0651 | No |
| 73 | Utp3 | 1354 | -0.553 | -0.0620 | No |
| 74 | Senp6 | 1364 | -0.556 | -0.0617 | No |
| 75 | Ddr1 | 1387 | -0.561 | -0.0659 | No |
| 76 | Bod1 | 1398 | -0.562 | -0.0659 | No |
| 77 | Thap3 | 1413 | -0.566 | -0.0672 | No |
| 78 | Mpst | 1415 | -0.566 | -0.0641 | No |
| 79 | Zfp710 | 1427 | -0.571 | -0.0643 | No |
| 80 | Casc3 | 1435 | -0.572 | -0.0632 | No |
| 81 | Ostc | 1457 | -0.576 | -0.0669 | No |
| 82 | Mbp | 1464 | -0.577 | -0.0654 | No |
| 83 | Dexi | 1471 | -0.580 | -0.0639 | No |
| 84 | Nectin2 | 1479 | -0.581 | -0.0628 | No |
| 85 | Zfand2b | 1522 | -0.592 | -0.0737 | No |
| 86 | Ier2 | 1527 | -0.593 | -0.0714 | No |
| 87 | Commd9 | 1540 | -0.597 | -0.0719 | No |
| 88 | Bsg | 1560 | -0.604 | -0.0747 | No |
| 89 | Aimp1 | 1609 | -0.618 | -0.0876 | No |
| 90 | Timm44 | 1618 | -0.621 | -0.0865 | No |
| 91 | Nr4a1 | 1632 | -0.627 | -0.0871 | No |
| 92 | Rbm26 | 1633 | -0.628 | -0.0832 | No |
| 93 | 2610528J11Rik | 1670 | -0.639 | -0.0918 | No |
| 94 | Abhd16a | 1687 | -0.647 | -0.0933 | No |
| 95 | Mal | 1710 | -0.658 | -0.0969 | No |
| 96 | Echdc2 | 1769 | -0.679 | -0.1128 | No |
| 97 | Atg101 | 1782 | -0.681 | -0.1127 | No |
| 98 | Tmed3 | 1790 | -0.685 | -0.1109 | No |
| 99 | S100a16 | 1801 | -0.687 | -0.1102 | No |
| 100 | Spin1 | 1815 | -0.695 | -0.1104 | No |
| 101 | Tmem205 | 1833 | -0.702 | -0.1119 | No |
| 102 | Hyou1 | 1969 | -0.747 | -0.1542 | No |
| 103 | Tmem106c | 1973 | -0.748 | -0.1506 | No |
| 104 | Smagp | 2010 | -0.766 | -0.1583 | No |
| 105 | Slpi | 2011 | -0.766 | -0.1536 | No |
| 106 | Dedd | 2018 | -0.768 | -0.1509 | No |
| 107 | Cited2 | 2055 | -0.788 | -0.1585 | No |
| 108 | Macrod1 | 2161 | -0.838 | -0.1898 | No |
| 109 | Lmo4 | 2166 | -0.842 | -0.1859 | No |
| 110 | Spag7 | 2169 | -0.844 | -0.1814 | No |
| 111 | Rp9 | 2194 | -0.856 | -0.1844 | No |
| 112 | Ybx3 | 2212 | -0.865 | -0.1850 | No |
| 113 | Elof1 | 2236 | -0.885 | -0.1875 | No |
| 114 | Grhpr | 2258 | -0.898 | -0.1892 | No |
| 115 | Pmf1 | 2267 | -0.905 | -0.1864 | No |
| 116 | Pim3 | 2306 | -0.930 | -0.1938 | No |
| 117 | 2410002F23Rik | 2324 | -0.938 | -0.1939 | No |
| 118 | Tacc2 | 2335 | -0.948 | -0.1915 | No |
| 119 | Nans | 2346 | -0.959 | -0.1890 | No |
| 120 | Epb41l4b | 2382 | -0.993 | -0.1950 | No |
| 121 | Nfkbiz | 2444 | -1.047 | -0.2097 | No |
| 122 | Cobl | 2466 | -1.061 | -0.2104 | Yes |
| 123 | Irx5 | 2467 | -1.063 | -0.2038 | Yes |
| 124 | Fos | 2491 | -1.083 | -0.2051 | Yes |
| 125 | Acbd4 | 2507 | -1.099 | -0.2035 | Yes |
| 126 | Bad | 2522 | -1.117 | -0.2015 | Yes |
| 127 | Elf3 | 2533 | -1.130 | -0.1979 | Yes |
| 128 | Iffo2 | 2536 | -1.136 | -0.1916 | Yes |
| 129 | Spint2 | 2539 | -1.139 | -0.1852 | Yes |
| 130 | Zfp703 | 2541 | -1.141 | -0.1785 | Yes |
| 131 | Nupr1 | 2565 | -1.167 | -0.1793 | Yes |
| 132 | Plcb3 | 2573 | -1.174 | -0.1744 | Yes |
| 133 | S100a14 | 2580 | -1.185 | -0.1692 | Yes |
| 134 | Casz1 | 2607 | -1.220 | -0.1706 | Yes |
| 135 | Mob3b | 2610 | -1.223 | -0.1638 | Yes |
| 136 | Tmem79 | 2651 | -1.286 | -0.1697 | Yes |
| 137 | Ppp1r13l | 2692 | -1.350 | -0.1752 | Yes |
| 138 | Plac8 | 2695 | -1.353 | -0.1675 | Yes |
| 139 | Gpatch4 | 2696 | -1.354 | -0.1591 | Yes |
| 140 | Nr4a2 | 2700 | -1.361 | -0.1517 | Yes |
| 141 | Dcn | 2701 | -1.362 | -0.1433 | Yes |
| 142 | Egr1 | 2733 | -1.440 | -0.1451 | Yes |
| 143 | Rassf7 | 2752 | -1.491 | -0.1422 | Yes |
| 144 | Bicdl2 | 2774 | -1.535 | -0.1399 | Yes |
| 145 | Klc3 | 2821 | -1.633 | -0.1458 | Yes |
| 146 | Klf5 | 2823 | -1.644 | -0.1359 | Yes |
| 147 | Anxa8 | 2833 | -1.679 | -0.1287 | Yes |
| 148 | Ly6g6e | 2837 | -1.699 | -0.1192 | Yes |
| 149 | Foxq1 | 2872 | -1.867 | -0.1194 | Yes |
| 150 | Hebp2 | 2874 | -1.871 | -0.1082 | Yes |
| 151 | Fgfbp1 | 2921 | -2.159 | -0.1108 | Yes |
| 152 | Evpl | 2925 | -2.172 | -0.0984 | Yes |
| 153 | Smim5 | 2962 | -2.450 | -0.0957 | Yes |
| 154 | Krt4 | 2983 | -2.885 | -0.0848 | Yes |
| 155 | Krt14 | 2985 | -2.898 | -0.0672 | Yes |
| 156 | Lypd3 | 3005 | -3.177 | -0.0541 | Yes |
| 157 | Krt17 | 3006 | -3.210 | -0.0342 | Yes |
| 158 | Krt5 | 3013 | -3.435 | -0.0150 | Yes |
| 159 | Krt13 | 3025 | -3.823 | 0.0049 | Yes |
Table: GSEA details [plain text format]

  

Fig 2: TABULA\_MURIS\_SENIS\_SKIN\_EPIDERMAL\_CELL\_AGEING: Random ES distribution      
 Gene set null distribution of ES for **TABULA\_MURIS\_SENIS\_SKIN\_EPIDERMAL\_CELL\_AGEING**

  
