# Supplementary material for: TMPRSS11B promotes an acidified microenvironment and immune suppression in squamous lung cancer
Source: EMBO Rep. 2025 Nov 10;26(24):6346–79. doi: 10.1038/s44319-025-00631-1 (PMC12714794; doi:10.1038/s44319-025-00631-1)
Supplement: Supplementary file 11 — Source data Fig. 6 [file 44319_2025_631_MOESM11_ESM.zip › Figure 6/6D-E/GSEA Broad Institute_low pH vs rest of the regions (high pH)/TABULA_MURIS_SENIS_SPLEEN_B_CELL_AGEING.html]

Details for gene set TABULA\_MURIS\_SENIS\_SPLEEN\_B\_CELL\_AGEING[GSEA]

|  || Dataset | Lactate high vs low\_Ranked |
| Phenotype | NoPhenotypeAvailable |
| Upregulated in class | na\_pos |
| GeneSet | TABULA\_MURIS\_SENIS\_SPLEEN\_B\_CELL\_AGEING |
| Enrichment Score (ES) | 0.51295936 |
| Normalized Enrichment Score (NES) | 2.843751 |
| Nominal p-value | 0.0 |
| FDR q-value | 0.0 |
| FWER p-Value | 0.0 |
Table: GSEA Results Summary

  

Fig 1: Enrichment plot: TABULA\_MURIS\_SENIS\_SPLEEN\_B\_CELL\_AGEING      
 Profile of the Running ES Score & Positions of GeneSet Members on the Rank Ordered List

  

| SYMBOL | RANK IN GENE LIST | RANK METRIC SCORE | RUNNING ES | CORE ENRICHMENT || 1 | Apoe | 6 | 2.177 | 0.0489 | Yes |
| 2 | Lgals1 | 45 | 1.781 | 0.0779 | Yes |
| 3 | Psap | 59 | 1.694 | 0.1132 | Yes |
| 4 | Lat2 | 64 | 1.685 | 0.1513 | Yes |
| 5 | Evi2a | 81 | 1.620 | 0.1838 | Yes |
| 6 | Fcgr2b | 102 | 1.572 | 0.2139 | Yes |
| 7 | Cybb | 119 | 1.535 | 0.2445 | Yes |
| 8 | Emp3 | 191 | 1.389 | 0.2533 | Yes |
| 9 | Syk | 200 | 1.373 | 0.2828 | Yes |
| 10 | Npc2 | 202 | 1.371 | 0.3145 | Yes |
| 11 | Bcl2a1b | 241 | 1.307 | 0.3324 | Yes |
| 12 | Cd72 | 327 | 1.189 | 0.3319 | Yes |
| 13 | H2-DMb1 | 348 | 1.167 | 0.3525 | Yes |
| 14 | Fxyd5 | 377 | 1.133 | 0.3697 | Yes |
| 15 | B2m | 402 | 1.097 | 0.3873 | Yes |
| 16 | H2-Ab1 | 404 | 1.094 | 0.4126 | Yes |
| 17 | H2-Eb1 | 425 | 1.075 | 0.4311 | Yes |
| 18 | Crip1 | 431 | 1.069 | 0.4544 | Yes |
| 19 | H2-Aa | 465 | 1.035 | 0.4676 | Yes |
| 20 | Gns | 617 | 0.874 | 0.4377 | Yes |
| 21 | Txn1 | 656 | 0.846 | 0.4448 | Yes |
| 22 | Irf8 | 663 | 0.840 | 0.4625 | Yes |
| 23 | H2-Q4 | 681 | 0.826 | 0.4761 | Yes |
| 24 | H2-D1 | 722 | 0.794 | 0.4813 | Yes |
| 25 | Cst3 | 782 | 0.723 | 0.4786 | Yes |
| 26 | Grb2 | 816 | 0.695 | 0.4839 | Yes |
| 27 | H2-K1 | 818 | 0.692 | 0.4997 | Yes |
| 28 | Psmb8 | 838 | 0.678 | 0.5092 | Yes |
| 29 | Ctsh | 890 | 0.637 | 0.5071 | Yes |
| 30 | Ptpn1 | 940 | 0.604 | 0.5049 | Yes |
| 31 | Sh3bgrl3 | 1006 | 0.560 | 0.4963 | Yes |
| 32 | Sh3glb1 | 1036 | 0.545 | 0.4994 | Yes |
| 33 | H2-T23 | 1045 | 0.540 | 0.5094 | Yes |
| 34 | Cd44 | 1072 | 0.523 | 0.5130 | Yes |
| 35 | Txndc5 | 1761 | -0.675 | 0.2993 | No |
| 36 | Nap1l1 | 1905 | -0.723 | 0.2686 | No |
| 37 | Pycard | 2022 | -0.771 | 0.2479 | No |
| 38 | Tnfaip8 | 2050 | -0.785 | 0.2573 | No |
| 39 | Ly6a | 2366 | -0.979 | 0.1751 | No |
| 40 | Jchain | 2385 | -0.995 | 0.1924 | No |
| 41 | Fos | 2491 | -1.083 | 0.1827 | No |
Table: GSEA details [plain text format]

  

Fig 2: TABULA\_MURIS\_SENIS\_SPLEEN\_B\_CELL\_AGEING: Random ES distribution      
 Gene set null distribution of ES for **TABULA\_MURIS\_SENIS\_SPLEEN\_B\_CELL\_AGEING**

  
