# Supplementary material for: TMPRSS11B promotes an acidified microenvironment and immune suppression in squamous lung cancer
Source: EMBO Rep. 2025 Nov 10;26(24):6346–79. doi: 10.1038/s44319-025-00631-1 (PMC12714794; doi:10.1038/s44319-025-00631-1)
Supplement: Supplementary file 11 — Source data Fig. 6 [file 44319_2025_631_MOESM11_ESM.zip › Figure 6/6D-E/GSEA Broad Institute_low pH vs rest of the regions (high pH)/TABULA_MURIS_SENIS_SPLEEN_MACROPHAGE_AGEING.html]

Details for gene set TABULA\_MURIS\_SENIS\_SPLEEN\_MACROPHAGE\_AGEING[GSEA]

|  || Dataset | Lactate high vs low\_Ranked |
| Phenotype | NoPhenotypeAvailable |
| Upregulated in class | na\_pos |
| GeneSet | TABULA\_MURIS\_SENIS\_SPLEEN\_MACROPHAGE\_AGEING |
| Enrichment Score (ES) | 0.51894623 |
| Normalized Enrichment Score (NES) | 3.019665 |
| Nominal p-value | 0.0 |
| FDR q-value | 0.0 |
| FWER p-Value | 0.0 |
Table: GSEA Results Summary

  

Fig 1: Enrichment plot: TABULA\_MURIS\_SENIS\_SPLEEN\_MACROPHAGE\_AGEING      
 Profile of the Running ES Score & Positions of GeneSet Members on the Rank Ordered List

  

| SYMBOL | RANK IN GENE LIST | RANK METRIC SCORE | RUNNING ES | CORE ENRICHMENT || 1 | C1qb | 2 | 2.291 | 0.0392 | Yes |
| 2 | Apoe | 6 | 2.177 | 0.0760 | Yes |
| 3 | Ctss | 14 | 2.088 | 0.1100 | Yes |
| 4 | C1qc | 20 | 1.988 | 0.1429 | Yes |
| 5 | Cd300c2 | 26 | 1.896 | 0.1742 | Yes |
| 6 | Lgmn | 28 | 1.878 | 0.2065 | Yes |
| 7 | C1qa | 29 | 1.862 | 0.2389 | Yes |
| 8 | Mpeg1 | 42 | 1.799 | 0.2661 | Yes |
| 9 | Lgals1 | 45 | 1.781 | 0.2964 | Yes |
| 10 | Ctsb | 46 | 1.778 | 0.3273 | Yes |
| 11 | Lyz1 | 78 | 1.627 | 0.3453 | Yes |
| 12 | Fcer1g | 95 | 1.597 | 0.3677 | Yes |
| 13 | Cybb | 119 | 1.535 | 0.3867 | Yes |
| 14 | Ctsd | 136 | 1.507 | 0.4075 | Yes |
| 15 | Cfp | 168 | 1.424 | 0.4219 | Yes |
| 16 | Npc2 | 202 | 1.371 | 0.4347 | Yes |
| 17 | Csf1r | 249 | 1.292 | 0.4418 | Yes |
| 18 | Wfdc17 | 345 | 1.169 | 0.4304 | Yes |
| 19 | Grn | 365 | 1.146 | 0.4439 | Yes |
| 20 | Hexa | 476 | 1.021 | 0.4249 | Yes |
| 21 | Acp5 | 483 | 1.008 | 0.4404 | Yes |
| 22 | Fth1 | 503 | 0.986 | 0.4512 | Yes |
| 23 | Creg1 | 517 | 0.973 | 0.4638 | Yes |
| 24 | Ctsc | 525 | 0.966 | 0.4782 | Yes |
| 25 | Trf | 541 | 0.957 | 0.4899 | Yes |
| 26 | Ctla2a | 574 | 0.924 | 0.4952 | Yes |
| 27 | Msrb1 | 629 | 0.868 | 0.4923 | Yes |
| 28 | Txn1 | 656 | 0.846 | 0.4983 | Yes |
| 29 | Gng11 | 662 | 0.840 | 0.5112 | Yes |
| 30 | Flna | 707 | 0.805 | 0.5105 | Yes |
| 31 | Cd63 | 724 | 0.793 | 0.5189 | Yes |
| 32 | Tln1 | 804 | 0.703 | 0.5048 | No |
| 33 | Rgs10 | 837 | 0.678 | 0.5058 | No |
| 34 | Atp6v0e | 888 | 0.638 | 0.5002 | No |
| 35 | Ptpn1 | 940 | 0.604 | 0.4937 | No |
| 36 | Ninj1 | 1025 | 0.551 | 0.4752 | No |
| 37 | Sh3glb1 | 1036 | 0.545 | 0.4813 | No |
| 38 | Lamp1 | 1051 | 0.536 | 0.4859 | No |
| 39 | Cstb | 1056 | 0.535 | 0.4939 | No |
| 40 | Litaf | 1084 | 0.517 | 0.4939 | No |
| 41 | P4hb | 1282 | -0.538 | 0.4373 | No |
| 42 | Fabp5 | 1603 | -0.617 | 0.3410 | No |
| 43 | Tmed3 | 1790 | -0.685 | 0.2907 | No |
| 44 | Rpp21 | 1964 | -0.745 | 0.2458 | No |
| 45 | Smagp | 2010 | -0.766 | 0.2441 | No |
| 46 | Slpi | 2011 | -0.766 | 0.2574 | No |
| 47 | Basp1 | 2191 | -0.855 | 0.2124 | No |
| 48 | Ly6a | 2366 | -0.979 | 0.1712 | No |
| 49 | Jchain | 2385 | -0.995 | 0.1825 | No |
| 50 | Ifitm1 | 2906 | -2.063 | 0.0445 | No |
Table: GSEA details [plain text format]

  

Fig 2: TABULA\_MURIS\_SENIS\_SPLEEN\_MACROPHAGE\_AGEING: Random ES distribution      
 Gene set null distribution of ES for **TABULA\_MURIS\_SENIS\_SPLEEN\_MACROPHAGE\_AGEING**

  
