# Supplementary material for: TMPRSS11B promotes an acidified microenvironment and immune suppression in squamous lung cancer
Source: EMBO Rep. 2025 Nov 10;26(24):6346–79. doi: 10.1038/s44319-025-00631-1 (PMC12714794; doi:10.1038/s44319-025-00631-1)
Supplement: Supplementary file 11 — Source data Fig. 6 [file 44319_2025_631_MOESM11_ESM.zip › Figure 6/6D-E/GSEA Broad Institute_low pH vs rest of the regions (high pH)/TABULA_MURIS_SENIS_TONGUE_BASAL_CELL_OF_EPIDERMIS_AGEING.html]

Details for gene set TABULA\_MURIS\_SENIS\_TONGUE\_BASAL\_CELL\_OF\_EPIDERMIS\_AGEING[GSEA]

|  || Dataset | Lactate high vs low\_Ranked |
| Phenotype | NoPhenotypeAvailable |
| Upregulated in class | na\_neg |
| GeneSet | TABULA\_MURIS\_SENIS\_TONGUE\_BASAL\_CELL\_OF\_EPIDERMIS\_AGEING |
| Enrichment Score (ES) | -0.42003742 |
| Normalized Enrichment Score (NES) | -1.8912519 |
| Nominal p-value | 0.008264462 |
| FDR q-value | 0.018335763 |
| FWER p-Value | 0.278 |
Table: GSEA Results Summary

  

Fig 1: Enrichment plot: TABULA\_MURIS\_SENIS\_TONGUE\_BASAL\_CELL\_OF\_EPIDERMIS\_AGEING      
 Profile of the Running ES Score & Positions of GeneSet Members on the Rank Ordered List

  

| SYMBOL | RANK IN GENE LIST | RANK METRIC SCORE | RUNNING ES | CORE ENRICHMENT || 1 | Apoe | 6 | 2.177 | 0.0418 | No |
| 2 | Apoc1 | 40 | 1.811 | 0.0673 | No |
| 3 | Lgals3 | 344 | 1.170 | -0.0102 | No |
| 4 | B2m | 402 | 1.097 | -0.0071 | No |
| 5 | Cxcl16 | 479 | 1.013 | -0.0120 | No |
| 6 | Tubb6 | 480 | 1.013 | 0.0084 | No |
| 7 | Cenpa | 655 | 0.847 | -0.0326 | No |
| 8 | H2-D1 | 722 | 0.794 | -0.0386 | No |
| 9 | H2-K1 | 818 | 0.692 | -0.0563 | No |
| 10 | Arl6ip1 | 961 | 0.595 | -0.0917 | No |
| 11 | Ccna2 | 1094 | 0.505 | -0.1255 | No |
| 12 | Plaur | 1098 | 0.504 | -0.1163 | No |
| 13 | Rack1 | 1195 | -0.519 | -0.1379 | No |
| 14 | Eef1b2 | 1352 | -0.553 | -0.1787 | No |
| 15 | Cldn4 | 1430 | -0.572 | -0.1929 | No |
| 16 | Fabp5 | 1603 | -0.617 | -0.2378 | No |
| 17 | Nhp2 | 1637 | -0.629 | -0.2361 | No |
| 18 | Pgk1 | 1680 | -0.642 | -0.2372 | No |
| 19 | Ppa1 | 1719 | -0.661 | -0.2366 | No |
| 20 | Aldh3a1 | 1866 | -0.710 | -0.2709 | No |
| 21 | Tyms | 1908 | -0.724 | -0.2700 | No |
| 22 | Pmm1 | 1922 | -0.729 | -0.2597 | No |
| 23 | Slpi | 2011 | -0.766 | -0.2736 | No |
| 24 | Tacstd2 | 2079 | -0.799 | -0.2798 | No |
| 25 | Phlda1 | 2291 | -0.920 | -0.3316 | No |
| 26 | Ptgr1 | 2332 | -0.943 | -0.3260 | No |
| 27 | S100a14 | 2580 | -1.185 | -0.3844 | No |
| 28 | Gpx2 | 2598 | -1.211 | -0.3657 | No |
| 29 | Plet1 | 2762 | -1.513 | -0.3896 | Yes |
| 30 | Ovol1 | 2764 | -1.518 | -0.3594 | Yes |
| 31 | Gsta4 | 2809 | -1.609 | -0.3416 | Yes |
| 32 | Epcam | 2813 | -1.616 | -0.3101 | Yes |
| 33 | Gsto1 | 2912 | -2.103 | -0.3005 | Yes |
| 34 | Ehf | 2933 | -2.240 | -0.2620 | Yes |
| 35 | Ly6d | 2942 | -2.300 | -0.2184 | Yes |
| 36 | Adh7 | 2943 | -2.301 | -0.1721 | Yes |
| 37 | Krtdap | 2997 | -3.095 | -0.1275 | Yes |
| 38 | Lypd3 | 3005 | -3.177 | -0.0659 | Yes |
| 39 | Krt6b | 3024 | -3.819 | 0.0050 | Yes |
Table: GSEA details [plain text format]

  

Fig 2: TABULA\_MURIS\_SENIS\_TONGUE\_BASAL\_CELL\_OF\_EPIDERMIS\_AGEING: Random ES distribution      
 Gene set null distribution of ES for **TABULA\_MURIS\_SENIS\_TONGUE\_BASAL\_CELL\_OF\_EPIDERMIS\_AGEING**

  
