# Supplementary material for: TMPRSS11B promotes an acidified microenvironment and immune suppression in squamous lung cancer
Source: EMBO Rep. 2025 Nov 10;26(24):6346–79. doi: 10.1038/s44319-025-00631-1 (PMC12714794; doi:10.1038/s44319-025-00631-1)
Supplement: Supplementary file 11 — Source data Fig. 6 [file 44319_2025_631_MOESM11_ESM.zip › Figure 6/6D-E/GSEA Broad Institute_low pH vs rest of the regions (high pH)/TABULA_MURIS_SENIS_TONGUE_KERATINOCYTE_AGEING.html]

Details for gene set TABULA\_MURIS\_SENIS\_TONGUE\_KERATINOCYTE\_AGEING[GSEA]

|  || Dataset | Lactate high vs low\_Ranked |
| Phenotype | NoPhenotypeAvailable |
| Upregulated in class | na\_neg |
| GeneSet | TABULA\_MURIS\_SENIS\_TONGUE\_KERATINOCYTE\_AGEING |
| Enrichment Score (ES) | -0.61113256 |
| Normalized Enrichment Score (NES) | -2.172181 |
| Nominal p-value | 0.0 |
| FDR q-value | 7.614597E-4 |
| FWER p-Value | 0.008 |
Table: GSEA Results Summary

  

Fig 1: Enrichment plot: TABULA\_MURIS\_SENIS\_TONGUE\_KERATINOCYTE\_AGEING      
 Profile of the Running ES Score & Positions of GeneSet Members on the Rank Ordered List

  

| SYMBOL | RANK IN GENE LIST | RANK METRIC SCORE | RUNNING ES | CORE ENRICHMENT || 1 | Apoc1 | 40 | 1.811 | 0.0516 | No |
| 2 | B2m | 402 | 1.097 | -0.0286 | No |
| 3 | H2-D1 | 722 | 0.794 | -0.1058 | No |
| 4 | H2-K1 | 818 | 0.692 | -0.1125 | No |
| 5 | Rack1 | 1195 | -0.519 | -0.2183 | No |
| 6 | Eef1b2 | 1352 | -0.553 | -0.2501 | No |
| 7 | Aldh3a1 | 1866 | -0.710 | -0.3945 | No |
| 8 | Slpi | 2011 | -0.766 | -0.4147 | No |
| 9 | Phlda1 | 2291 | -0.920 | -0.4741 | No |
| 10 | Ptgr1 | 2332 | -0.943 | -0.4536 | No |
| 11 | Gsta4 | 2809 | -1.609 | -0.5536 | Yes |
| 12 | Pdzk1ip1 | 2812 | -1.614 | -0.4965 | Yes |
| 13 | Ces1d | 2822 | -1.643 | -0.4407 | Yes |
| 14 | Ly6g6c | 2922 | -2.162 | -0.3960 | Yes |
| 15 | Adh7 | 2943 | -2.301 | -0.3203 | Yes |
| 16 | Krt14 | 2985 | -2.898 | -0.2302 | Yes |
| 17 | Krtdap | 2997 | -3.095 | -0.1231 | Yes |
| 18 | Krt6b | 3024 | -3.819 | 0.0050 | Yes |
Table: GSEA details [plain text format]

  

Fig 2: TABULA\_MURIS\_SENIS\_TONGUE\_KERATINOCYTE\_AGEING: Random ES distribution      
 Gene set null distribution of ES for **TABULA\_MURIS\_SENIS\_TONGUE\_KERATINOCYTE\_AGEING**

  
