# Supplementary material for: TMPRSS11B promotes an acidified microenvironment and immune suppression in squamous lung cancer
Source: EMBO Rep. 2025 Nov 10;26(24):6346–79. doi: 10.1038/s44319-025-00631-1 (PMC12714794; doi:10.1038/s44319-025-00631-1)
Supplement: Supplementary file 11 — Source data Fig. 6 [file 44319_2025_631_MOESM11_ESM.zip › Figure 6/6D-E/GSEA Broad Institute_low pH vs rest of the regions (high pH)/ZHANG_UTERUS_C12_MONOCYTE.html]

Details for gene set ZHANG\_UTERUS\_C12\_MONOCYTE[GSEA]

|  || Dataset | Lactate high vs low\_Ranked |
| Phenotype | NoPhenotypeAvailable |
| Upregulated in class | na\_pos |
| GeneSet | ZHANG\_UTERUS\_C12\_MONOCYTE |
| Enrichment Score (ES) | 0.6372185 |
| Normalized Enrichment Score (NES) | 4.365721 |
| Nominal p-value | 0.0 |
| FDR q-value | 0.0 |
| FWER p-Value | 0.0 |
Table: GSEA Results Summary

  

Fig 1: Enrichment plot: ZHANG\_UTERUS\_C12\_MONOCYTE      
 Profile of the Running ES Score & Positions of GeneSet Members on the Rank Ordered List

  

| SYMBOL | RANK IN GENE LIST | RANK METRIC SCORE | RUNNING ES | CORE ENRICHMENT || 1 | C1qb | 2 | 2.291 | 0.0178 | Yes |
| 2 | Apoe | 6 | 2.177 | 0.0344 | Yes |
| 3 | Ctss | 14 | 2.088 | 0.0489 | Yes |
| 4 | Hmox1 | 16 | 2.051 | 0.0651 | Yes |
| 5 | C1qc | 20 | 1.988 | 0.0801 | Yes |
| 6 | Mafb | 22 | 1.943 | 0.0955 | Yes |
| 7 | Ms4a6d | 27 | 1.895 | 0.1094 | Yes |
| 8 | Lgmn | 28 | 1.878 | 0.1246 | Yes |
| 9 | Cd68 | 36 | 1.820 | 0.1369 | Yes |
| 10 | Ms4a6c | 37 | 1.819 | 0.1516 | Yes |
| 11 | Fcgr1 | 38 | 1.818 | 0.1663 | Yes |
| 12 | Mpeg1 | 42 | 1.799 | 0.1798 | Yes |
| 13 | Ctsb | 46 | 1.778 | 0.1931 | Yes |
| 14 | Psap | 59 | 1.694 | 0.2027 | Yes |
| 15 | Lat2 | 64 | 1.685 | 0.2149 | Yes |
| 16 | Ly86 | 66 | 1.677 | 0.2281 | Yes |
| 17 | Tyrobp | 83 | 1.618 | 0.2358 | Yes |
| 18 | Spi1 | 86 | 1.616 | 0.2481 | Yes |
| 19 | Plin2 | 90 | 1.607 | 0.2601 | Yes |
| 20 | Fcer1g | 95 | 1.597 | 0.2716 | Yes |
| 21 | C5ar1 | 96 | 1.596 | 0.2845 | Yes |
| 22 | Cd83 | 101 | 1.575 | 0.2959 | Yes |
| 23 | Fcgr2b | 102 | 1.572 | 0.3086 | Yes |
| 24 | Rgs1 | 105 | 1.567 | 0.3205 | Yes |
| 25 | Sdc3 | 106 | 1.564 | 0.3332 | Yes |
| 26 | Fcgr3 | 108 | 1.562 | 0.3454 | Yes |
| 27 | Cybb | 119 | 1.535 | 0.3544 | Yes |
| 28 | Plek | 135 | 1.509 | 0.3615 | Yes |
| 29 | Itgb2 | 138 | 1.498 | 0.3729 | Yes |
| 30 | Cd53 | 139 | 1.488 | 0.3849 | Yes |
| 31 | Emilin2 | 141 | 1.485 | 0.3966 | Yes |
| 32 | Csf2ra | 143 | 1.478 | 0.4082 | Yes |
| 33 | Cfp | 168 | 1.424 | 0.4115 | Yes |
| 34 | Emp3 | 191 | 1.389 | 0.4152 | Yes |
| 35 | Npc2 | 202 | 1.371 | 0.4229 | Yes |
| 36 | Ftl1-ps1 | 211 | 1.361 | 0.4312 | Yes |
| 37 | Spp1 | 232 | 1.327 | 0.4351 | Yes |
| 38 | Cd52 | 233 | 1.323 | 0.4458 | Yes |
| 39 | Ctsz | 242 | 1.303 | 0.4536 | Yes |
| 40 | Csf1r | 249 | 1.292 | 0.4619 | Yes |
| 41 | Ptprc | 255 | 1.283 | 0.4706 | Yes |
| 42 | Ccr2 | 288 | 1.234 | 0.4697 | Yes |
| 43 | Ctsa | 302 | 1.219 | 0.4751 | Yes |
| 44 | Csf2rb | 318 | 1.205 | 0.4797 | Yes |
| 45 | Lgals3 | 344 | 1.170 | 0.4806 | Yes |
| 46 | Wfdc17 | 345 | 1.169 | 0.4901 | Yes |
| 47 | H2-DMb1 | 348 | 1.167 | 0.4988 | Yes |
| 48 | Grn | 365 | 1.146 | 0.5026 | Yes |
| 49 | Alox5ap | 375 | 1.133 | 0.5087 | Yes |
| 50 | Cd74 | 376 | 1.133 | 0.5179 | Yes |
| 51 | Fxyd5 | 377 | 1.133 | 0.5270 | Yes |
| 52 | B2m | 402 | 1.097 | 0.5277 | Yes |
| 53 | H2-Ab1 | 404 | 1.094 | 0.5362 | Yes |
| 54 | Ccl9 | 411 | 1.089 | 0.5430 | Yes |
| 55 | Lcp1 | 416 | 1.084 | 0.5503 | Yes |
| 56 | H2-DMa | 418 | 1.083 | 0.5587 | Yes |
| 57 | H2-Eb1 | 425 | 1.075 | 0.5654 | Yes |
| 58 | Crip1 | 431 | 1.069 | 0.5723 | Yes |
| 59 | Arhgdib | 445 | 1.049 | 0.5764 | Yes |
| 60 | Cotl1 | 447 | 1.049 | 0.5845 | Yes |
| 61 | Unc93b1 | 464 | 1.037 | 0.5874 | Yes |
| 62 | H2-Aa | 465 | 1.035 | 0.5958 | Yes |
| 63 | Rab8b | 489 | 1.002 | 0.5960 | Yes |
| 64 | Fth1 | 503 | 0.986 | 0.5996 | Yes |
| 65 | Samhd1 | 505 | 0.985 | 0.6072 | Yes |
| 66 | Coro1a | 522 | 0.970 | 0.6096 | Yes |
| 67 | Atp6v1c1 | 524 | 0.967 | 0.6170 | Yes |
| 68 | Ctsc | 525 | 0.966 | 0.6248 | Yes |
| 69 | Kctd12 | 532 | 0.963 | 0.6306 | Yes |
| 70 | Cyba | 554 | 0.947 | 0.6311 | Yes |
| 71 | Rilpl2 | 580 | 0.918 | 0.6300 | Yes |
| 72 | Irf5 | 624 | 0.871 | 0.6224 | Yes |
| 73 | Msrb1 | 629 | 0.868 | 0.6280 | Yes |
| 74 | Tgfbi | 651 | 0.851 | 0.6277 | Yes |
| 75 | Laptm5 | 659 | 0.844 | 0.6322 | Yes |
| 76 | Actr3 | 665 | 0.838 | 0.6372 | Yes |
| 77 | H2-D1 | 722 | 0.794 | 0.6246 | No |
| 78 | Cdkn1a | 749 | 0.765 | 0.6219 | No |
| 79 | Ccl6 | 772 | 0.736 | 0.6203 | No |
| 80 | Cst3 | 782 | 0.723 | 0.6231 | No |
| 81 | Rab20 | 811 | 0.700 | 0.6192 | No |
| 82 | Psmb8 | 838 | 0.678 | 0.6158 | No |
| 83 | Pim1 | 859 | 0.656 | 0.6143 | No |
| 84 | Mcl1 | 861 | 0.655 | 0.6193 | No |
| 85 | Ubl3 | 868 | 0.651 | 0.6225 | No |
| 86 | Slfn2 | 908 | 0.624 | 0.6142 | No |
| 87 | Ptpn1 | 940 | 0.604 | 0.6086 | No |
| 88 | Sdcbp | 945 | 0.602 | 0.6121 | No |
| 89 | Picalm | 993 | 0.568 | 0.6006 | No |
| 90 | Sh3bgrl3 | 1006 | 0.560 | 0.6011 | No |
| 91 | Ninj1 | 1025 | 0.551 | 0.5994 | No |
| 92 | Cstb | 1056 | 0.535 | 0.5935 | No |
| 93 | Iqgap1 | 1058 | 0.534 | 0.5975 | No |
| 94 | Cd44 | 1072 | 0.523 | 0.5973 | No |
| 95 | Litaf | 1084 | 0.517 | 0.5977 | No |
| 96 | Plaur | 1098 | 0.504 | 0.5973 | No |
| 97 | Tgif1 | 1220 | -0.526 | 0.5604 | No |
| 98 | H3f3b | 1262 | -0.534 | 0.5507 | No |
| 99 | Gda | 2280 | -0.913 | 0.2117 | No |
| 100 | Vps37b | 2349 | -0.962 | 0.1963 | No |
| 101 | Ly6e | 2392 | -1.001 | 0.1901 | No |
| 102 | Por | 2395 | -1.005 | 0.1975 | No |
| 103 | Irf7 | 2638 | -1.265 | 0.1253 | No |
| 104 | Plac8 | 2695 | -1.353 | 0.1172 | No |
Table: GSEA details [plain text format]

  

Fig 2: ZHANG\_UTERUS\_C12\_MONOCYTE: Random ES distribution      
 Gene set null distribution of ES for **ZHANG\_UTERUS\_C12\_MONOCYTE**

  
