# Supplementary material for: TMPRSS11B promotes an acidified microenvironment and immune suppression in squamous lung cancer
Source: EMBO Rep. 2025 Nov 10;26(24):6346–79. doi: 10.1038/s44319-025-00631-1 (PMC12714794; doi:10.1038/s44319-025-00631-1)
Supplement: Supplementary file 11 — Source data Fig. 6 [file 44319_2025_631_MOESM11_ESM.zip › Figure 6/6D-E/GSEA Broad Institute_low pH vs rest of the regions (high pH)/ZHANG_UTERUS_C13_EPITHELIAL1_CELL.html]

Details for gene set ZHANG\_UTERUS\_C13\_EPITHELIAL1\_CELL[GSEA]

|  || Dataset | Lactate high vs low\_Ranked |
| Phenotype | NoPhenotypeAvailable |
| Upregulated in class | na\_neg |
| GeneSet | ZHANG\_UTERUS\_C13\_EPITHELIAL1\_CELL |
| Enrichment Score (ES) | -0.386368 |
| Normalized Enrichment Score (NES) | -2.0159814 |
| Nominal p-value | 0.0 |
| FDR q-value | 0.0065632467 |
| FWER p-Value | 0.089 |
Table: GSEA Results Summary

  

Fig 1: Enrichment plot: ZHANG\_UTERUS\_C13\_EPITHELIAL1\_CELL      
 Profile of the Running ES Score & Positions of GeneSet Members on the Rank Ordered List

  

| SYMBOL | RANK IN GENE LIST | RANK METRIC SCORE | RUNNING ES | CORE ENRICHMENT || 1 | Egfl6 | 131 | 1.514 | -0.0275 | No |
| 2 | Anpep | 212 | 1.361 | -0.0394 | No |
| 3 | Arg2 | 303 | 1.219 | -0.0563 | No |
| 4 | Cdo1 | 362 | 1.150 | -0.0632 | No |
| 5 | Tceal9 | 409 | 1.090 | -0.0666 | No |
| 6 | Sox17 | 450 | 1.047 | -0.0685 | No |
| 7 | S100g | 563 | 0.941 | -0.0959 | No |
| 8 | Tgfbi | 651 | 0.851 | -0.1159 | No |
| 9 | Txn1 | 656 | 0.846 | -0.1079 | No |
| 10 | Lcn2 | 711 | 0.802 | -0.1172 | No |
| 11 | C3 | 948 | 0.602 | -0.1903 | No |
| 12 | Arl6ip1 | 961 | 0.595 | -0.1877 | No |
| 13 | Ndufaf2 | 1147 | -0.508 | -0.2446 | No |
| 14 | Krt8 | 1193 | -0.519 | -0.2541 | No |
| 15 | 2510002D24Rik | 1230 | -0.528 | -0.2604 | No |
| 16 | Eef1b2 | 1352 | -0.553 | -0.2951 | No |
| 17 | Sfxn1 | 1362 | -0.555 | -0.2920 | No |
| 18 | Idh3a | 1408 | -0.565 | -0.3009 | No |
| 19 | Nudt14 | 1414 | -0.566 | -0.2964 | No |
| 20 | Acp1 | 1433 | -0.572 | -0.2961 | No |
| 21 | Bola1 | 1463 | -0.577 | -0.2995 | No |
| 22 | Mt2 | 1478 | -0.581 | -0.2978 | No |
| 23 | Ndufv2 | 1610 | -0.618 | -0.3352 | No |
| 24 | Nhp2 | 1637 | -0.629 | -0.3370 | No |
| 25 | Yipf1 | 1662 | -0.637 | -0.3381 | No |
| 26 | Paics | 1667 | -0.638 | -0.3323 | No |
| 27 | Knop1 | 1707 | -0.657 | -0.3382 | No |
| 28 | Echdc2 | 1769 | -0.679 | -0.3513 | No |
| 29 | Siva1 | 1794 | -0.685 | -0.3518 | No |
| 30 | Cbx6 | 1799 | -0.686 | -0.3456 | No |
| 31 | Cib1 | 1832 | -0.701 | -0.3486 | No |
| 32 | Gstm2 | 1848 | -0.706 | -0.3459 | No |
| 33 | Nap1l1 | 1905 | -0.723 | -0.3568 | No |
| 34 | Tyms | 1908 | -0.724 | -0.3494 | No |
| 35 | Atp1a1 | 1953 | -0.741 | -0.3561 | No |
| 36 | Hnrnpc | 1958 | -0.742 | -0.3492 | No |
| 37 | Gclm | 2000 | -0.761 | -0.3546 | No |
| 38 | Gtf2a2 | 2095 | -0.804 | -0.3775 | Yes |
| 39 | Fkbp4 | 2117 | -0.814 | -0.3755 | Yes |
| 40 | Apex1 | 2125 | -0.816 | -0.3688 | Yes |
| 41 | Arpc5l | 2141 | -0.824 | -0.3648 | Yes |
| 42 | Plat | 2156 | -0.832 | -0.3603 | Yes |
| 43 | Gtf3c6 | 2196 | -0.857 | -0.3640 | Yes |
| 44 | Sox9 | 2201 | -0.860 | -0.3558 | Yes |
| 45 | Aldh1a1 | 2205 | -0.861 | -0.3473 | Yes |
| 46 | Pmf1 | 2267 | -0.905 | -0.3578 | Yes |
| 47 | Acadl | 2329 | -0.941 | -0.3680 | Yes |
| 48 | Cited4 | 2358 | -0.972 | -0.3667 | Yes |
| 49 | Slc1a5 | 2375 | -0.989 | -0.3611 | Yes |
| 50 | Cldn3 | 2379 | -0.991 | -0.3512 | Yes |
| 51 | Dcxr | 2434 | -1.037 | -0.3579 | Yes |
| 52 | Id1 | 2455 | -1.056 | -0.3530 | Yes |
| 53 | Krt19 | 2476 | -1.067 | -0.3479 | Yes |
| 54 | Srsf3 | 2496 | -1.090 | -0.3422 | Yes |
| 55 | Spint2 | 2539 | -1.139 | -0.3438 | Yes |
| 56 | Smim22 | 2591 | -1.201 | -0.3477 | Yes |
| 57 | Gpx2 | 2598 | -1.211 | -0.3363 | Yes |
| 58 | Rbbp7 | 2609 | -1.222 | -0.3262 | Yes |
| 59 | Cldn7 | 2642 | -1.269 | -0.3229 | Yes |
| 60 | Rdh10 | 2645 | -1.273 | -0.3095 | Yes |
| 61 | Slc39a4 | 2673 | -1.321 | -0.3039 | Yes |
| 62 | Gstm5 | 2715 | -1.392 | -0.3024 | Yes |
| 63 | Dgat2 | 2727 | -1.420 | -0.2903 | Yes |
| 64 | Plet1 | 2762 | -1.513 | -0.2851 | Yes |
| 65 | Cxcl17 | 2779 | -1.545 | -0.2733 | Yes |
| 66 | Muc1 | 2794 | -1.579 | -0.2606 | Yes |
| 67 | Fermt1 | 2795 | -1.579 | -0.2431 | Yes |
| 68 | Epcam | 2813 | -1.616 | -0.2309 | Yes |
| 69 | Fxyd3 | 2815 | -1.624 | -0.2132 | Yes |
| 70 | Wfdc2 | 2861 | -1.816 | -0.2083 | Yes |
| 71 | Cbr2 | 2865 | -1.827 | -0.1891 | Yes |
| 72 | Clu | 2876 | -1.874 | -0.1717 | Yes |
| 73 | Ifitm1 | 2906 | -2.063 | -0.1586 | Yes |
| 74 | Pigr | 2910 | -2.092 | -0.1364 | Yes |
| 75 | Gsto1 | 2912 | -2.103 | -0.1134 | Yes |
| 76 | Kctd14 | 2928 | -2.194 | -0.0942 | Yes |
| 77 | Tmem158 | 2930 | -2.218 | -0.0699 | Yes |
| 78 | Cd24a | 2948 | -2.343 | -0.0497 | Yes |
| 79 | Cfi | 2977 | -2.744 | -0.0288 | Yes |
| 80 | Ltf | 3035 | -4.454 | 0.0014 | Yes |
Table: GSEA details [plain text format]

  

Fig 2: ZHANG\_UTERUS\_C13\_EPITHELIAL1\_CELL: Random ES distribution      
 Gene set null distribution of ES for **ZHANG\_UTERUS\_C13\_EPITHELIAL1\_CELL**

  
