# Supplementary material for: TMPRSS11B promotes an acidified microenvironment and immune suppression in squamous lung cancer
Source: EMBO Rep. 2025 Nov 10;26(24):6346–79. doi: 10.1038/s44319-025-00631-1 (PMC12714794; doi:10.1038/s44319-025-00631-1)
Supplement: Supplementary file 11 — Source data Fig. 6 [file 44319_2025_631_MOESM11_ESM.zip › Figure 6/6D-E/GSEA Broad Institute_low pH vs rest of the regions (high pH)/ZHANG_UTERUS_C1_REGENERATIVE_UP.html]

Details for gene set ZHANG\_UTERUS\_C1\_REGENERATIVE\_UP[GSEA]

|  || Dataset | Lactate high vs low\_Ranked |
| Phenotype | NoPhenotypeAvailable |
| Upregulated in class | na\_neg |
| GeneSet | ZHANG\_UTERUS\_C1\_REGENERATIVE\_UP |
| Enrichment Score (ES) | -0.34490868 |
| Normalized Enrichment Score (NES) | -1.868492 |
| Nominal p-value | 0.0 |
| FDR q-value | 0.018494694 |
| FWER p-Value | 0.32 |
Table: GSEA Results Summary

  

Fig 1: Enrichment plot: ZHANG\_UTERUS\_C1\_REGENERATIVE\_UP      
 Profile of the Running ES Score & Positions of GeneSet Members on the Rank Ordered List

  

| SYMBOL | RANK IN GENE LIST | RANK METRIC SCORE | RUNNING ES | CORE ENRICHMENT || 1 | Hmox1 | 16 | 2.051 | 0.0147 | No |
| 2 | Lgals1 | 45 | 1.781 | 0.0227 | No |
| 3 | Vim | 128 | 1.521 | 0.0097 | No |
| 4 | Atf3 | 161 | 1.444 | 0.0130 | No |
| 5 | Ftl1-ps1 | 211 | 1.361 | 0.0098 | No |
| 6 | Col6a1 | 246 | 1.297 | 0.0109 | No |
| 7 | Lgals3 | 344 | 1.170 | -0.0105 | No |
| 8 | Anxa6 | 417 | 1.083 | -0.0244 | No |
| 9 | Ctsc | 525 | 0.966 | -0.0512 | No |
| 10 | Dusp1 | 569 | 0.935 | -0.0566 | No |
| 11 | Gpx1 | 603 | 0.888 | -0.0591 | No |
| 12 | Col1a1 | 643 | 0.856 | -0.0640 | No |
| 13 | Col1a2 | 704 | 0.807 | -0.0764 | No |
| 14 | Wsb1 | 730 | 0.780 | -0.0773 | No |
| 15 | Cdkn1a | 749 | 0.765 | -0.0759 | No |
| 16 | Rarres2 | 848 | 0.672 | -0.1026 | No |
| 17 | Pim1 | 859 | 0.656 | -0.0995 | No |
| 18 | Blnk | 895 | 0.634 | -0.1052 | No |
| 19 | Sh3glb1 | 1036 | 0.545 | -0.1474 | No |
| 20 | Cald1 | 1066 | 0.529 | -0.1520 | No |
| 21 | Plaur | 1098 | 0.504 | -0.1576 | No |
| 22 | Dpp3 | 1120 | -0.504 | -0.1598 | No |
| 23 | Fbl | 1188 | -0.517 | -0.1775 | No |
| 24 | Krt8 | 1193 | -0.519 | -0.1738 | No |
| 25 | Tgif1 | 1220 | -0.526 | -0.1774 | No |
| 26 | Net1 | 1229 | -0.528 | -0.1750 | No |
| 27 | Maff | 1259 | -0.533 | -0.1796 | No |
| 28 | Msmo1 | 1304 | -0.541 | -0.1892 | No |
| 29 | Wasl | 1306 | -0.542 | -0.1842 | No |
| 30 | Nip7 | 1334 | -0.549 | -0.1880 | No |
| 31 | Snrpa1 | 1335 | -0.549 | -0.1826 | No |
| 32 | Anxa1 | 1347 | -0.552 | -0.1809 | No |
| 33 | Cldn4 | 1430 | -0.572 | -0.2032 | No |
| 34 | Btg2 | 1440 | -0.573 | -0.2006 | No |
| 35 | Pmaip1 | 1451 | -0.575 | -0.1984 | No |
| 36 | Tmprss4 | 1517 | -0.591 | -0.2146 | No |
| 37 | Nr4a1 | 1632 | -0.627 | -0.2472 | No |
| 38 | Trip10 | 1641 | -0.630 | -0.2437 | No |
| 39 | Pgk1 | 1680 | -0.642 | -0.2503 | No |
| 40 | Txndc5 | 1761 | -0.675 | -0.2709 | No |
| 41 | Lin7c | 1853 | -0.708 | -0.2948 | No |
| 42 | Hbegf | 1860 | -0.709 | -0.2899 | No |
| 43 | Cttn | 1883 | -0.716 | -0.2904 | No |
| 44 | Dalrd3 | 1928 | -0.731 | -0.2981 | No |
| 45 | G6pdx | 1934 | -0.733 | -0.2926 | No |
| 46 | Clint1 | 1996 | -0.758 | -0.3059 | No |
| 47 | Mxd1 | 2005 | -0.763 | -0.3011 | No |
| 48 | Wdr77 | 2045 | -0.782 | -0.3067 | No |
| 49 | Tacstd2 | 2079 | -0.799 | -0.3101 | No |
| 50 | Cd2ap | 2149 | -0.827 | -0.3254 | No |
| 51 | S100a6 | 2175 | -0.847 | -0.3256 | No |
| 52 | Pkp4 | 2224 | -0.874 | -0.3333 | No |
| 53 | Hmgcs1 | 2251 | -0.894 | -0.3334 | No |
| 54 | Pdxdc1 | 2286 | -0.916 | -0.3359 | Yes |
| 55 | Phlda1 | 2291 | -0.920 | -0.3283 | Yes |
| 56 | Kcnk1 | 2305 | -0.929 | -0.3236 | Yes |
| 57 | Dhcr24 | 2316 | -0.935 | -0.3178 | Yes |
| 58 | Cdc42ep5 | 2317 | -0.935 | -0.3086 | Yes |
| 59 | Ly6a | 2366 | -0.979 | -0.3153 | Yes |
| 60 | F3 | 2372 | -0.985 | -0.3073 | Yes |
| 61 | Smox | 2377 | -0.990 | -0.2990 | Yes |
| 62 | Jchain | 2385 | -0.995 | -0.2916 | Yes |
| 63 | Gpd1l | 2397 | -1.006 | -0.2855 | Yes |
| 64 | Tspan1 | 2419 | -1.017 | -0.2826 | Yes |
| 65 | Fam107b | 2420 | -1.020 | -0.2726 | Yes |
| 66 | Nfkbiz | 2444 | -1.047 | -0.2702 | Yes |
| 67 | Krt19 | 2476 | -1.067 | -0.2702 | Yes |
| 68 | Adh1 | 2495 | -1.089 | -0.2656 | Yes |
| 69 | Igha | 2511 | -1.107 | -0.2599 | Yes |
| 70 | Elf3 | 2533 | -1.130 | -0.2559 | Yes |
| 71 | Cnksr1 | 2544 | -1.145 | -0.2481 | Yes |
| 72 | Golm1 | 2590 | -1.199 | -0.2516 | Yes |
| 73 | Bace2 | 2625 | -1.240 | -0.2510 | Yes |
| 74 | Slc44a4 | 2633 | -1.255 | -0.2411 | Yes |
| 75 | Lad1 | 2675 | -1.325 | -0.2420 | Yes |
| 76 | Rnd3 | 2679 | -1.331 | -0.2299 | Yes |
| 77 | Cldn23 | 2686 | -1.345 | -0.2188 | Yes |
| 78 | Plac8 | 2695 | -1.353 | -0.2082 | Yes |
| 79 | Krt7 | 2749 | -1.487 | -0.2116 | Yes |
| 80 | Cfb | 2772 | -1.529 | -0.2041 | Yes |
| 81 | Prxl2a | 2805 | -1.599 | -0.1993 | Yes |
| 82 | Gsta4 | 2809 | -1.609 | -0.1845 | Yes |
| 83 | Epcam | 2813 | -1.616 | -0.1697 | Yes |
| 84 | Klf5 | 2823 | -1.644 | -0.1566 | Yes |
| 85 | Capn5 | 2830 | -1.667 | -0.1423 | Yes |
| 86 | Sprr1a | 2841 | -1.716 | -0.1289 | Yes |
| 87 | Galnt3 | 2845 | -1.733 | -0.1129 | Yes |
| 88 | Mboat2 | 2864 | -1.824 | -0.1011 | Yes |
| 89 | Cbr2 | 2865 | -1.827 | -0.0832 | Yes |
| 90 | Qsox1 | 2881 | -1.902 | -0.0696 | Yes |
| 91 | Mfsd4a | 2899 | -2.010 | -0.0557 | Yes |
| 92 | Muc4 | 2941 | -2.290 | -0.0471 | Yes |
| 93 | Ceacam1 | 2954 | -2.374 | -0.0279 | Yes |
| 94 | Atp2c2 | 2980 | -2.830 | -0.0086 | Yes |
| 95 | Ramp3 | 2984 | -2.886 | 0.0187 | Yes |
Table: GSEA details [plain text format]

  

Fig 2: ZHANG\_UTERUS\_C1\_REGENERATIVE\_UP: Random ES distribution      
 Gene set null distribution of ES for **ZHANG\_UTERUS\_C1\_REGENERATIVE\_UP**

  
