# Supplementary material for: TMPRSS11B promotes an acidified microenvironment and immune suppression in squamous lung cancer
Source: EMBO Rep. 2025 Nov 10;26(24):6346–79. doi: 10.1038/s44319-025-00631-1 (PMC12714794; doi:10.1038/s44319-025-00631-1)
Supplement: Supplementary file 11 — Source data Fig. 6 [file 44319_2025_631_MOESM11_ESM.zip › Figure 6/6D-E/GSEA Broad Institute_low pH vs rest of the regions (high pH)/ZHANG_UTERUS_C2_REGENERATIVE_UP.html]

Details for gene set ZHANG\_UTERUS\_C2\_REGENERATIVE\_UP[GSEA]

|  || Dataset | Lactate high vs low\_Ranked |
| Phenotype | NoPhenotypeAvailable |
| Upregulated in class | na\_neg |
| GeneSet | ZHANG\_UTERUS\_C2\_REGENERATIVE\_UP |
| Enrichment Score (ES) | -0.41233346 |
| Normalized Enrichment Score (NES) | -1.5527589 |
| Nominal p-value | 0.05351682 |
| FDR q-value | 0.12224496 |
| FWER p-Value | 0.989 |
Table: GSEA Results Summary

  

Fig 1: Enrichment plot: ZHANG\_UTERUS\_C2\_REGENERATIVE\_UP      
 Profile of the Running ES Score & Positions of GeneSet Members on the Rank Ordered List

  

| SYMBOL | RANK IN GENE LIST | RANK METRIC SCORE | RUNNING ES | CORE ENRICHMENT || 1 | Egfl6 | 131 | 1.514 | 0.0076 | No |
| 2 | Arg2 | 303 | 1.219 | -0.0079 | No |
| 3 | Cdo1 | 362 | 1.150 | 0.0116 | No |
| 4 | Tceal9 | 409 | 1.090 | 0.0331 | No |
| 5 | Sox17 | 450 | 1.047 | 0.0552 | No |
| 6 | S100g | 563 | 0.941 | 0.0498 | No |
| 7 | Tgfbi | 651 | 0.851 | 0.0496 | No |
| 8 | Igfbp7 | 860 | 0.655 | 0.0028 | No |
| 9 | Mgp | 898 | 0.630 | 0.0118 | No |
| 10 | Eef1b2 | 1352 | -0.553 | -0.1197 | No |
| 11 | Mt2 | 1478 | -0.581 | -0.1416 | No |
| 12 | Echdc2 | 1769 | -0.679 | -0.2148 | No |
| 13 | Siva1 | 1794 | -0.685 | -0.1997 | No |
| 14 | Gstm2 | 1848 | -0.706 | -0.1934 | No |
| 15 | Aldh1a1 | 2205 | -0.861 | -0.2824 | No |
| 16 | Gpx2 | 2598 | -1.211 | -0.3715 | Yes |
| 17 | Rbp1 | 2599 | -1.213 | -0.3306 | Yes |
| 18 | Gstm5 | 2715 | -1.392 | -0.3218 | Yes |
| 19 | Clu | 2876 | -1.874 | -0.3116 | Yes |
| 20 | Ifitm1 | 2906 | -2.063 | -0.2516 | Yes |
| 21 | Pigr | 2910 | -2.092 | -0.1821 | Yes |
| 22 | Kctd14 | 2928 | -2.194 | -0.1137 | Yes |
| 23 | Ltf | 3035 | -4.454 | 0.0013 | Yes |
Table: GSEA details [plain text format]

  

Fig 2: ZHANG\_UTERUS\_C2\_REGENERATIVE\_UP: Random ES distribution      
 Gene set null distribution of ES for **ZHANG\_UTERUS\_C2\_REGENERATIVE\_UP**

  
