# Supplementary material for: TMPRSS11B promotes an acidified microenvironment and immune suppression in squamous lung cancer
Source: EMBO Rep. 2025 Nov 10;26(24):6346–79. doi: 10.1038/s44319-025-00631-1 (PMC12714794; doi:10.1038/s44319-025-00631-1)
Supplement: Supplementary file 11 — Source data Fig. 6 [file 44319_2025_631_MOESM11_ESM.zip › Figure 6/6D-E/GSEA Broad Institute_low pH vs rest of the regions (high pH)/ZHANG_UTERUS_C5_MACROPHAGE.html]

Details for gene set ZHANG\_UTERUS\_C5\_MACROPHAGE[GSEA]

|  || Dataset | Lactate high vs low\_Ranked |
| Phenotype | NoPhenotypeAvailable |
| Upregulated in class | na\_pos |
| GeneSet | ZHANG\_UTERUS\_C5\_MACROPHAGE |
| Enrichment Score (ES) | 0.67589813 |
| Normalized Enrichment Score (NES) | 4.700584 |
| Nominal p-value | 0.0 |
| FDR q-value | 0.0 |
| FWER p-Value | 0.0 |
Table: GSEA Results Summary

  

Fig 1: Enrichment plot: ZHANG\_UTERUS\_C5\_MACROPHAGE      
 Profile of the Running ES Score & Positions of GeneSet Members on the Rank Ordered List

  

| SYMBOL | RANK IN GENE LIST | RANK METRIC SCORE | RUNNING ES | CORE ENRICHMENT || 1 | C1qb | 2 | 2.291 | 0.0175 | Yes |
| 2 | C3ar1 | 4 | 2.207 | 0.0346 | Yes |
| 3 | Apoe | 6 | 2.177 | 0.0515 | Yes |
| 4 | Ctss | 14 | 2.088 | 0.0656 | Yes |
| 5 | Trem2 | 15 | 2.053 | 0.0819 | Yes |
| 6 | Hmox1 | 16 | 2.051 | 0.0981 | Yes |
| 7 | Clec12a | 19 | 1.996 | 0.1132 | Yes |
| 8 | C1qc | 20 | 1.988 | 0.1289 | Yes |
| 9 | Mafb | 22 | 1.943 | 0.1440 | Yes |
| 10 | Ms4a6d | 27 | 1.895 | 0.1576 | Yes |
| 11 | Lgmn | 28 | 1.878 | 0.1725 | Yes |
| 12 | C1qa | 29 | 1.862 | 0.1872 | Yes |
| 13 | Lpl | 34 | 1.833 | 0.2004 | Yes |
| 14 | Cd68 | 36 | 1.820 | 0.2144 | Yes |
| 15 | Ms4a6c | 37 | 1.819 | 0.2288 | Yes |
| 16 | Fcgr1 | 38 | 1.818 | 0.2432 | Yes |
| 17 | Mpeg1 | 42 | 1.799 | 0.2564 | Yes |
| 18 | Ctsb | 46 | 1.778 | 0.2695 | Yes |
| 19 | Lipa | 54 | 1.721 | 0.2807 | Yes |
| 20 | Sirpa | 56 | 1.702 | 0.2938 | Yes |
| 21 | Psap | 59 | 1.694 | 0.3066 | Yes |
| 22 | Ly86 | 66 | 1.677 | 0.3178 | Yes |
| 23 | Ms4a7 | 79 | 1.623 | 0.3266 | Yes |
| 24 | Tyrobp | 83 | 1.618 | 0.3384 | Yes |
| 25 | Spi1 | 86 | 1.616 | 0.3505 | Yes |
| 26 | Plin2 | 90 | 1.607 | 0.3622 | Yes |
| 27 | Fcer1g | 95 | 1.597 | 0.3734 | Yes |
| 28 | Cd83 | 101 | 1.575 | 0.3842 | Yes |
| 29 | Fcgr2b | 102 | 1.572 | 0.3966 | Yes |
| 30 | Rgs1 | 105 | 1.567 | 0.4084 | Yes |
| 31 | Sdc3 | 106 | 1.564 | 0.4207 | Yes |
| 32 | Fcgr3 | 108 | 1.562 | 0.4328 | Yes |
| 33 | Cybb | 119 | 1.535 | 0.4415 | Yes |
| 34 | Pltp | 133 | 1.513 | 0.4491 | Yes |
| 35 | Plek | 135 | 1.509 | 0.4607 | Yes |
| 36 | Ctsd | 136 | 1.507 | 0.4726 | Yes |
| 37 | Cd53 | 139 | 1.488 | 0.4837 | Yes |
| 38 | Atf3 | 161 | 1.444 | 0.4880 | Yes |
| 39 | Cfp | 168 | 1.424 | 0.4972 | Yes |
| 40 | Hexb | 197 | 1.379 | 0.4986 | Yes |
| 41 | Npc2 | 202 | 1.371 | 0.5081 | Yes |
| 42 | Cd52 | 233 | 1.323 | 0.5084 | Yes |
| 43 | Bcl2a1b | 241 | 1.307 | 0.5163 | Yes |
| 44 | Ctsz | 242 | 1.303 | 0.5266 | Yes |
| 45 | Csf1r | 249 | 1.292 | 0.5348 | Yes |
| 46 | Ctsa | 302 | 1.219 | 0.5268 | Yes |
| 47 | Lgals3 | 344 | 1.170 | 0.5221 | Yes |
| 48 | Wfdc17 | 345 | 1.169 | 0.5313 | Yes |
| 49 | H2-DMb1 | 348 | 1.167 | 0.5399 | Yes |
| 50 | Grn | 365 | 1.146 | 0.5435 | Yes |
| 51 | Alox5ap | 375 | 1.133 | 0.5494 | Yes |
| 52 | Cd74 | 376 | 1.133 | 0.5584 | Yes |
| 53 | B2m | 402 | 1.097 | 0.5586 | Yes |
| 54 | H2-Ab1 | 404 | 1.094 | 0.5669 | Yes |
| 55 | Ccl9 | 411 | 1.089 | 0.5735 | Yes |
| 56 | Klf2 | 412 | 1.087 | 0.5821 | Yes |
| 57 | Lcp1 | 416 | 1.084 | 0.5897 | Yes |
| 58 | H2-DMa | 418 | 1.083 | 0.5979 | Yes |
| 59 | Snx5 | 421 | 1.078 | 0.6057 | Yes |
| 60 | H2-Eb1 | 425 | 1.075 | 0.6132 | Yes |
| 61 | Cotl1 | 447 | 1.049 | 0.6144 | Yes |
| 62 | Unc93b1 | 464 | 1.037 | 0.6171 | Yes |
| 63 | H2-Aa | 465 | 1.035 | 0.6253 | Yes |
| 64 | Hexa | 476 | 1.021 | 0.6300 | Yes |
| 65 | Cxcl16 | 479 | 1.013 | 0.6373 | Yes |
| 66 | Mrc1 | 502 | 0.988 | 0.6377 | Yes |
| 67 | Fth1 | 503 | 0.986 | 0.6455 | Yes |
| 68 | Creg1 | 517 | 0.973 | 0.6488 | Yes |
| 69 | Coro1a | 522 | 0.970 | 0.6551 | Yes |
| 70 | Ctsc | 525 | 0.966 | 0.6621 | Yes |
| 71 | Kctd12 | 532 | 0.963 | 0.6676 | Yes |
| 72 | Trf | 541 | 0.957 | 0.6725 | Yes |
| 73 | Cyba | 554 | 0.947 | 0.6759 | Yes |
| 74 | Ehd4 | 595 | 0.901 | 0.6694 | No |
| 75 | Ccl8 | 622 | 0.872 | 0.6675 | No |
| 76 | Laptm5 | 659 | 0.844 | 0.6619 | No |
| 77 | Actr3 | 665 | 0.838 | 0.6668 | No |
| 78 | H2-D1 | 722 | 0.794 | 0.6541 | No |
| 79 | Cdkn1a | 749 | 0.765 | 0.6513 | No |
| 80 | Ccl6 | 772 | 0.736 | 0.6496 | No |
| 81 | Cst3 | 782 | 0.723 | 0.6523 | No |
| 82 | H2-K1 | 818 | 0.692 | 0.6459 | No |
| 83 | Rgs10 | 837 | 0.678 | 0.6451 | No |
| 84 | Psmb8 | 838 | 0.678 | 0.6505 | No |
| 85 | Actb | 856 | 0.664 | 0.6500 | No |
| 86 | Mcl1 | 861 | 0.655 | 0.6538 | No |
| 87 | Dab2 | 867 | 0.651 | 0.6572 | No |
| 88 | Ctsh | 890 | 0.637 | 0.6548 | No |
| 89 | Sdcbp | 945 | 0.602 | 0.6412 | No |
| 90 | Cfl1 | 973 | 0.581 | 0.6366 | No |
| 91 | Sh3bgrl3 | 1006 | 0.560 | 0.6302 | No |
| 92 | Ninj1 | 1025 | 0.551 | 0.6284 | No |
| 93 | Lamp1 | 1051 | 0.536 | 0.6241 | No |
| 94 | Cstb | 1056 | 0.535 | 0.6270 | No |
| 95 | H3f3b | 1262 | -0.534 | 0.5615 | No |
| 96 | Il1rn | 1858 | -0.709 | 0.3647 | No |
| 97 | Ighm | 2137 | -0.822 | 0.2767 | No |
| 98 | Ly6e | 2392 | -1.001 | 0.1982 | No |
| 99 | Nfkbiz | 2444 | -1.047 | 0.1891 | No |
| 100 | Tlr2 | 2819 | -1.629 | 0.0748 | No |
Table: GSEA details [plain text format]

  

Fig 2: ZHANG\_UTERUS\_C5\_MACROPHAGE: Random ES distribution      
 Gene set null distribution of ES for **ZHANG\_UTERUS\_C5\_MACROPHAGE**

  
