# Supplementary material for: TMPRSS11B promotes an acidified microenvironment and immune suppression in squamous lung cancer
Source: EMBO Rep. 2025 Nov 10;26(24):6346–79. doi: 10.1038/s44319-025-00631-1 (PMC12714794; doi:10.1038/s44319-025-00631-1)
Supplement: Supplementary file 11 — Source data Fig. 6 [file 44319_2025_631_MOESM11_ESM.zip › Figure 6/6D-E/GSEA Broad Institute_low pH vs rest of the regions (high pH)/ZHANG_UTERUS_C6_ENDOTHELIAL_PLVAP_HIGH_CELL.html]

Details for gene set ZHANG\_UTERUS\_C6\_ENDOTHELIAL\_PLVAP\_HIGH\_CELL[GSEA]

|  || Dataset | Lactate high vs low\_Ranked |
| Phenotype | NoPhenotypeAvailable |
| Upregulated in class | na\_pos |
| GeneSet | ZHANG\_UTERUS\_C6\_ENDOTHELIAL\_PLVAP\_HIGH\_CELL |
| Enrichment Score (ES) | 0.46364877 |
| Normalized Enrichment Score (NES) | 2.8594785 |
| Nominal p-value | 0.0 |
| FDR q-value | 0.0 |
| FWER p-Value | 0.0 |
Table: GSEA Results Summary

  

Fig 1: Enrichment plot: ZHANG\_UTERUS\_C6\_ENDOTHELIAL\_PLVAP\_HIGH\_CELL      
 Profile of the Running ES Score & Positions of GeneSet Members on the Rank Ordered List

  

| SYMBOL | RANK IN GENE LIST | RANK METRIC SCORE | RUNNING ES | CORE ENRICHMENT || 1 | Cav1 | 107 | 1.564 | -0.0092 | Yes |
| 2 | Vim | 128 | 1.521 | 0.0101 | Yes |
| 3 | Pltp | 133 | 1.513 | 0.0346 | Yes |
| 4 | Atf3 | 161 | 1.444 | 0.0502 | Yes |
| 5 | Cd93 | 186 | 1.398 | 0.0660 | Yes |
| 6 | Esam | 253 | 1.285 | 0.0658 | Yes |
| 7 | Msn | 280 | 1.243 | 0.0783 | Yes |
| 8 | Thbd | 306 | 1.215 | 0.0907 | Yes |
| 9 | Cd200 | 336 | 1.180 | 0.1011 | Yes |
| 10 | Ptprb | 337 | 1.179 | 0.1213 | Yes |
| 11 | H2-Q7 | 347 | 1.168 | 0.1382 | Yes |
| 12 | Fxyd5 | 377 | 1.133 | 0.1478 | Yes |
| 13 | B2m | 402 | 1.097 | 0.1585 | Yes |
| 14 | Gimap6 | 403 | 1.097 | 0.1772 | Yes |
| 15 | Egfl7 | 406 | 1.093 | 0.1952 | Yes |
| 16 | Klf2 | 412 | 1.087 | 0.2121 | Yes |
| 17 | Podxl | 420 | 1.081 | 0.2282 | Yes |
| 18 | Rasip1 | 427 | 1.073 | 0.2445 | Yes |
| 19 | Slfn5 | 434 | 1.066 | 0.2607 | Yes |
| 20 | Cdh5 | 455 | 1.041 | 0.2718 | Yes |
| 21 | Mmrn2 | 473 | 1.025 | 0.2836 | Yes |
| 22 | Tmem252 | 484 | 1.008 | 0.2974 | Yes |
| 23 | Icam1 | 492 | 0.999 | 0.3121 | Yes |
| 24 | Plvap | 512 | 0.977 | 0.3225 | Yes |
| 25 | Sparcl1 | 566 | 0.938 | 0.3207 | Yes |
| 26 | Aqp1 | 572 | 0.930 | 0.3349 | Yes |
| 27 | Ctla2a | 574 | 0.924 | 0.3504 | Yes |
| 28 | Ehd4 | 595 | 0.901 | 0.3590 | Yes |
| 29 | Flt1 | 628 | 0.869 | 0.3631 | Yes |
| 30 | Pecam1 | 658 | 0.845 | 0.3678 | Yes |
| 31 | Gng11 | 662 | 0.840 | 0.3812 | Yes |
| 32 | Ramp2 | 670 | 0.833 | 0.3931 | Yes |
| 33 | Epas1 | 680 | 0.827 | 0.4042 | Yes |
| 34 | Tmem88 | 712 | 0.801 | 0.4075 | Yes |
| 35 | H2-D1 | 722 | 0.794 | 0.4180 | Yes |
| 36 | Adamts1 | 723 | 0.793 | 0.4315 | Yes |
| 37 | Eng | 733 | 0.776 | 0.4418 | Yes |
| 38 | Scarb1 | 738 | 0.772 | 0.4536 | Yes |
| 39 | Kdr | 748 | 0.765 | 0.4636 | Yes |
| 40 | H2-K1 | 818 | 0.692 | 0.4523 | No |
| 41 | Psmb8 | 838 | 0.678 | 0.4575 | No |
| 42 | Igfbp7 | 860 | 0.655 | 0.4617 | No |
| 43 | Plpp3 | 938 | 0.605 | 0.4462 | No |
| 44 | Timp3 | 959 | 0.596 | 0.4497 | No |
| 45 | Ecscr | 970 | 0.587 | 0.4563 | No |
| 46 | Adgrf5 | 1082 | 0.518 | 0.4280 | No |
| 47 | Hspb1 | 1190 | -0.517 | 0.4009 | No |
| 48 | S100a16 | 1801 | -0.687 | 0.2081 | No |
| 49 | Hsp90aa1 | 1965 | -0.745 | 0.1661 | No |
| 50 | Hes1 | 2255 | -0.896 | 0.0845 | No |
| 51 | Pim3 | 2306 | -0.930 | 0.0836 | No |
| 52 | Ly6a | 2366 | -0.979 | 0.0806 | No |
| 53 | Ly6e | 2392 | -1.001 | 0.0893 | No |
| 54 | Nfkbiz | 2444 | -1.047 | 0.0900 | No |
| 55 | Id1 | 2455 | -1.056 | 0.1047 | No |
| 56 | Hspa1b | 2761 | -1.513 | 0.0283 | No |
| 57 | Clu | 2876 | -1.874 | 0.0220 | No |
| 58 | Tshz2 | 2879 | -1.890 | 0.0537 | No |
Table: GSEA details [plain text format]

  

Fig 2: ZHANG\_UTERUS\_C6\_ENDOTHELIAL\_PLVAP\_HIGH\_CELL: Random ES distribution      
 Gene set null distribution of ES for **ZHANG\_UTERUS\_C6\_ENDOTHELIAL\_PLVAP\_HIGH\_CELL**

  
