# Supplementary material for: TMPRSS11B promotes an acidified microenvironment and immune suppression in squamous lung cancer
Source: EMBO Rep. 2025 Nov 10;26(24):6346–79. doi: 10.1038/s44319-025-00631-1 (PMC12714794; doi:10.1038/s44319-025-00631-1)
Supplement: Supplementary file 11 — Source data Fig. 6 [file 44319_2025_631_MOESM11_ESM.zip › Figure 6/6D-E/GSEA Broad Institute_low pH vs rest of the regions (high pH)/ZHANG_UTERUS_C7_EPITHELIAL2_CELL.html]

Details for gene set ZHANG\_UTERUS\_C7\_EPITHELIAL2\_CELL[GSEA]

|  || Dataset | Lactate high vs low\_Ranked |
| Phenotype | NoPhenotypeAvailable |
| Upregulated in class | na\_neg |
| GeneSet | ZHANG\_UTERUS\_C7\_EPITHELIAL2\_CELL |
| Enrichment Score (ES) | -0.40216342 |
| Normalized Enrichment Score (NES) | -2.4079368 |
| Nominal p-value | 0.0 |
| FDR q-value | 0.0 |
| FWER p-Value | 0.0 |
Table: GSEA Results Summary

  

Fig 1: Enrichment plot: ZHANG\_UTERUS\_C7\_EPITHELIAL2\_CELL      
 Profile of the Running ES Score & Positions of GeneSet Members on the Rank Ordered List

  

| SYMBOL | RANK IN GENE LIST | RANK METRIC SCORE | RUNNING ES | CORE ENRICHMENT || 1 | Fam20c | 169 | 1.421 | -0.0509 | No |
| 2 | Anpep | 212 | 1.361 | -0.0579 | No |
| 3 | Spp1 | 232 | 1.327 | -0.0571 | No |
| 4 | Snx5 | 421 | 1.078 | -0.1165 | No |
| 5 | Crip1 | 431 | 1.069 | -0.1136 | No |
| 6 | Cyba | 554 | 0.947 | -0.1508 | No |
| 7 | S100g | 563 | 0.941 | -0.1483 | No |
| 8 | Gpx1 | 603 | 0.888 | -0.1569 | No |
| 9 | Cenpa | 655 | 0.847 | -0.1699 | No |
| 10 | Txn1 | 656 | 0.846 | -0.1652 | No |
| 11 | Lcn2 | 711 | 0.802 | -0.1795 | No |
| 12 | Ostf1 | 862 | 0.654 | -0.2280 | No |
| 13 | Ubl3 | 868 | 0.651 | -0.2261 | No |
| 14 | C3 | 948 | 0.602 | -0.2502 | No |
| 15 | S100a10 | 965 | 0.592 | -0.2525 | No |
| 16 | Plaur | 1098 | 0.504 | -0.2956 | No |
| 17 | Gmds | 1104 | -0.501 | -0.2946 | No |
| 18 | Pycr2 | 1146 | -0.508 | -0.3060 | No |
| 19 | Fbl | 1188 | -0.517 | -0.3174 | No |
| 20 | Calm3 | 1189 | -0.517 | -0.3145 | No |
| 21 | Krt8 | 1193 | -0.519 | -0.3126 | No |
| 22 | Rack1 | 1195 | -0.519 | -0.3101 | No |
| 23 | Idh3b | 1206 | -0.521 | -0.3106 | No |
| 24 | Net1 | 1229 | -0.528 | -0.3153 | No |
| 25 | Armc10 | 1265 | -0.534 | -0.3245 | No |
| 26 | Msmo1 | 1304 | -0.541 | -0.3347 | No |
| 27 | Bmyc | 1305 | -0.542 | -0.3317 | No |
| 28 | Snrpa1 | 1335 | -0.549 | -0.3387 | No |
| 29 | S100a11 | 1339 | -0.550 | -0.3367 | No |
| 30 | Anxa1 | 1347 | -0.552 | -0.3360 | No |
| 31 | Pdcd6 | 1350 | -0.553 | -0.3336 | No |
| 32 | Ythdc1 | 1358 | -0.554 | -0.3330 | No |
| 33 | Sfxn1 | 1362 | -0.555 | -0.3309 | No |
| 34 | Kif5b | 1372 | -0.558 | -0.3309 | No |
| 35 | Stip1 | 1374 | -0.558 | -0.3282 | No |
| 36 | Idh3a | 1408 | -0.565 | -0.3365 | No |
| 37 | Pno1 | 1423 | -0.568 | -0.3382 | No |
| 38 | Cldn4 | 1430 | -0.572 | -0.3371 | No |
| 39 | Acp1 | 1433 | -0.572 | -0.3346 | No |
| 40 | Anxa7 | 1434 | -0.572 | -0.3314 | No |
| 41 | Ostc | 1457 | -0.576 | -0.3358 | No |
| 42 | Eef1g | 1501 | -0.586 | -0.3475 | No |
| 43 | Tmprss4 | 1517 | -0.591 | -0.3494 | No |
| 44 | Ndufa9 | 1524 | -0.592 | -0.3482 | No |
| 45 | Eif6 | 1550 | -0.601 | -0.3535 | No |
| 46 | Uchl3 | 1551 | -0.602 | -0.3502 | No |
| 47 | Hmgb1 | 1558 | -0.603 | -0.3489 | No |
| 48 | Yrdc | 1600 | -0.616 | -0.3597 | No |
| 49 | Ndufv2 | 1610 | -0.618 | -0.3594 | No |
| 50 | Emg1 | 1616 | -0.620 | -0.3577 | No |
| 51 | Nhp2 | 1637 | -0.629 | -0.3611 | No |
| 52 | Ppm1g | 1660 | -0.637 | -0.3652 | No |
| 53 | Pgk1 | 1680 | -0.642 | -0.3682 | No |
| 54 | Ppa1 | 1719 | -0.661 | -0.3777 | No |
| 55 | Brix1 | 1731 | -0.666 | -0.3779 | No |
| 56 | Eef1d | 1738 | -0.668 | -0.3762 | No |
| 57 | Hspa9 | 1743 | -0.670 | -0.3739 | No |
| 58 | Zc3h15 | 1767 | -0.678 | -0.3781 | No |
| 59 | Lsr | 1792 | -0.685 | -0.3826 | No |
| 60 | Cib1 | 1832 | -0.701 | -0.3922 | No |
| 61 | Hbegf | 1860 | -0.709 | -0.3977 | Yes |
| 62 | Cops4 | 1870 | -0.711 | -0.3968 | Yes |
| 63 | Cttn | 1883 | -0.716 | -0.3970 | Yes |
| 64 | Ruvbl2 | 1892 | -0.719 | -0.3958 | Yes |
| 65 | Tmc4 | 1901 | -0.721 | -0.3945 | Yes |
| 66 | Nap1l1 | 1905 | -0.723 | -0.3915 | Yes |
| 67 | Tyms | 1908 | -0.724 | -0.3882 | Yes |
| 68 | Rpn1 | 1925 | -0.730 | -0.3897 | Yes |
| 69 | G6pdx | 1934 | -0.733 | -0.3884 | Yes |
| 70 | 2200002D01Rik | 1948 | -0.739 | -0.3887 | Yes |
| 71 | Atp1a1 | 1953 | -0.741 | -0.3860 | Yes |
| 72 | Hnrnpc | 1958 | -0.742 | -0.3832 | Yes |
| 73 | Serinc2 | 1986 | -0.754 | -0.3884 | Yes |
| 74 | Clint1 | 1996 | -0.758 | -0.3873 | Yes |
| 75 | Gclm | 2000 | -0.761 | -0.3841 | Yes |
| 76 | Pycard | 2022 | -0.771 | -0.3871 | Yes |
| 77 | Rab25 | 2032 | -0.776 | -0.3859 | Yes |
| 78 | Nop58 | 2073 | -0.796 | -0.3954 | Yes |
| 79 | Tacstd2 | 2079 | -0.799 | -0.3926 | Yes |
| 80 | Plpp2 | 2091 | -0.804 | -0.3920 | Yes |
| 81 | Cyp51 | 2098 | -0.806 | -0.3896 | Yes |
| 82 | Fkbp4 | 2117 | -0.814 | -0.3913 | Yes |
| 83 | Txnl1 | 2119 | -0.815 | -0.3871 | Yes |
| 84 | Apex1 | 2125 | -0.816 | -0.3843 | Yes |
| 85 | Cd2ap | 2149 | -0.827 | -0.3876 | Yes |
| 86 | Nsun2 | 2172 | -0.845 | -0.3906 | Yes |
| 87 | Gtf3c6 | 2196 | -0.857 | -0.3938 | Yes |
| 88 | Ybx3 | 2212 | -0.865 | -0.3942 | Yes |
| 89 | Elof1 | 2236 | -0.885 | -0.3972 | Yes |
| 90 | Rpf1 | 2238 | -0.886 | -0.3926 | Yes |
| 91 | Commd2 | 2246 | -0.888 | -0.3901 | Yes |
| 92 | Krtcap3 | 2265 | -0.902 | -0.3913 | Yes |
| 93 | Phlda1 | 2291 | -0.920 | -0.3949 | Yes |
| 94 | Ezr | 2297 | -0.926 | -0.3914 | Yes |
| 95 | Kcnk1 | 2305 | -0.929 | -0.3887 | Yes |
| 96 | G3bp1 | 2307 | -0.930 | -0.3838 | Yes |
| 97 | Ddx1 | 2311 | -0.933 | -0.3797 | Yes |
| 98 | Dhcr24 | 2316 | -0.935 | -0.3758 | Yes |
| 99 | Cdc42ep5 | 2317 | -0.935 | -0.3706 | Yes |
| 100 | Jup | 2337 | -0.950 | -0.3719 | Yes |
| 101 | Fmc1 | 2341 | -0.954 | -0.3676 | Yes |
| 102 | Gale | 2351 | -0.965 | -0.3653 | Yes |
| 103 | Ckmt1 | 2359 | -0.974 | -0.3623 | Yes |
| 104 | Ly6a | 2366 | -0.979 | -0.3590 | Yes |
| 105 | F3 | 2372 | -0.985 | -0.3552 | Yes |
| 106 | Slc1a5 | 2375 | -0.989 | -0.3504 | Yes |
| 107 | Smox | 2377 | -0.990 | -0.3452 | Yes |
| 108 | Cldn3 | 2379 | -0.991 | -0.3400 | Yes |
| 109 | Ap1m2 | 2386 | -0.995 | -0.3365 | Yes |
| 110 | Por | 2395 | -1.005 | -0.3337 | Yes |
| 111 | Tspan1 | 2419 | -1.017 | -0.3360 | Yes |
| 112 | Fam107b | 2420 | -1.020 | -0.3303 | Yes |
| 113 | Dcxr | 2434 | -1.037 | -0.3290 | Yes |
| 114 | Krt19 | 2476 | -1.067 | -0.3373 | Yes |
| 115 | Srsf3 | 2496 | -1.090 | -0.3378 | Yes |
| 116 | Avpi1 | 2499 | -1.093 | -0.3324 | Yes |
| 117 | Bzw2 | 2508 | -1.100 | -0.3291 | Yes |
| 118 | Elf3 | 2533 | -1.130 | -0.3311 | Yes |
| 119 | Spint2 | 2539 | -1.139 | -0.3265 | Yes |
| 120 | Golm1 | 2590 | -1.199 | -0.3372 | Yes |
| 121 | Smim22 | 2591 | -1.201 | -0.3305 | Yes |
| 122 | Las1l | 2604 | -1.217 | -0.3278 | Yes |
| 123 | Rbbp7 | 2609 | -1.222 | -0.3224 | Yes |
| 124 | Bace2 | 2625 | -1.240 | -0.3207 | Yes |
| 125 | St14 | 2628 | -1.245 | -0.3144 | Yes |
| 126 | Slc44a4 | 2633 | -1.255 | -0.3088 | Yes |
| 127 | Cldn7 | 2642 | -1.269 | -0.3045 | Yes |
| 128 | Cdh1 | 2655 | -1.294 | -0.3014 | Yes |
| 129 | Urah | 2658 | -1.303 | -0.2948 | Yes |
| 130 | Pigyl | 2660 | -1.307 | -0.2879 | Yes |
| 131 | Slc39a4 | 2673 | -1.321 | -0.2847 | Yes |
| 132 | Lad1 | 2675 | -1.325 | -0.2776 | Yes |
| 133 | Cldn23 | 2686 | -1.345 | -0.2736 | Yes |
| 134 | Tmem45b | 2732 | -1.438 | -0.2812 | Yes |
| 135 | Prkab1 | 2736 | -1.451 | -0.2741 | Yes |
| 136 | Cfap298 | 2744 | -1.464 | -0.2684 | Yes |
| 137 | Krt7 | 2749 | -1.487 | -0.2615 | Yes |
| 138 | Plet1 | 2762 | -1.513 | -0.2572 | Yes |
| 139 | Cfb | 2772 | -1.529 | -0.2518 | Yes |
| 140 | Cxcl17 | 2779 | -1.545 | -0.2452 | Yes |
| 141 | Psat1 | 2790 | -1.568 | -0.2399 | Yes |
| 142 | Muc1 | 2794 | -1.579 | -0.2322 | Yes |
| 143 | Fermt1 | 2795 | -1.579 | -0.2233 | Yes |
| 144 | Prxl2a | 2805 | -1.599 | -0.2175 | Yes |
| 145 | Gsta4 | 2809 | -1.609 | -0.2096 | Yes |
| 146 | Pdzk1ip1 | 2812 | -1.614 | -0.2012 | Yes |
| 147 | Epcam | 2813 | -1.616 | -0.1922 | Yes |
| 148 | Fxyd3 | 2815 | -1.624 | -0.1835 | Yes |
| 149 | Prom1 | 2828 | -1.661 | -0.1784 | Yes |
| 150 | Sprr1a | 2841 | -1.716 | -0.1730 | Yes |
| 151 | Wfdc2 | 2861 | -1.816 | -0.1694 | Yes |
| 152 | Cbr2 | 2865 | -1.827 | -0.1603 | Yes |
| 153 | Qsox1 | 2881 | -1.902 | -0.1548 | Yes |
| 154 | Fut2 | 2889 | -1.930 | -0.1465 | Yes |
| 155 | Mfsd4a | 2899 | -2.010 | -0.1384 | Yes |
| 156 | Ifitm1 | 2906 | -2.063 | -0.1289 | Yes |
| 157 | Gsto1 | 2912 | -2.103 | -0.1189 | Yes |
| 158 | Muc4 | 2941 | -2.290 | -0.1159 | Yes |
| 159 | Cd24a | 2948 | -2.343 | -0.1049 | Yes |
| 160 | Ceacam1 | 2954 | -2.374 | -0.0933 | Yes |
| 161 | Pglyrp1 | 2963 | -2.492 | -0.0822 | Yes |
| 162 | Atp2c2 | 2980 | -2.830 | -0.0719 | Yes |
| 163 | Gjb2 | 3016 | -3.482 | -0.0647 | Yes |
| 164 | Car12 | 3022 | -3.775 | -0.0453 | Yes |
| 165 | Ltf | 3035 | -4.454 | -0.0246 | Yes |
| 166 | Enpp3 | 3037 | -4.586 | 0.0007 | Yes |
Table: GSEA details [plain text format]

  

Fig 2: ZHANG\_UTERUS\_C7\_EPITHELIAL2\_CELL: Random ES distribution      
 Gene set null distribution of ES for **ZHANG\_UTERUS\_C7\_EPITHELIAL2\_CELL**

  
