# Supplementary material for: TMPRSS11B promotes an acidified microenvironment and immune suppression in squamous lung cancer
Source: EMBO Rep. 2025 Nov 10;26(24):6346–79. doi: 10.1038/s44319-025-00631-1 (PMC12714794; doi:10.1038/s44319-025-00631-1)
Supplement: Supplementary file 11 — Source data Fig. 6 [file 44319_2025_631_MOESM11_ESM.zip › Figure 6/6D-E/GSEA Broad Institute_low pH vs rest of the regions (high pH)/ZHANG_UTERUS_C8_NK_CELL.html]

Details for gene set ZHANG\_UTERUS\_C8\_NK\_CELL[GSEA]

|  || Dataset | Lactate high vs low\_Ranked |
| Phenotype | NoPhenotypeAvailable |
| Upregulated in class | na\_pos |
| GeneSet | ZHANG\_UTERUS\_C8\_NK\_CELL |
| Enrichment Score (ES) | 0.5231407 |
| Normalized Enrichment Score (NES) | 3.0571165 |
| Nominal p-value | 0.0 |
| FDR q-value | 0.0 |
| FWER p-Value | 0.0 |
Table: GSEA Results Summary

  

Fig 1: Enrichment plot: ZHANG\_UTERUS\_C8\_NK\_CELL      
 Profile of the Running ES Score & Positions of GeneSet Members on the Rank Ordered List

  

| SYMBOL | RANK IN GENE LIST | RANK METRIC SCORE | RUNNING ES | CORE ENRICHMENT || 1 | Tyrobp | 83 | 1.618 | 0.0046 | Yes |
| 2 | Fcer1g | 95 | 1.597 | 0.0329 | Yes |
| 3 | Rgs1 | 105 | 1.567 | 0.0612 | Yes |
| 4 | Cd53 | 139 | 1.488 | 0.0799 | Yes |
| 5 | Evl | 204 | 1.369 | 0.0859 | Yes |
| 6 | Spp1 | 232 | 1.327 | 0.1034 | Yes |
| 7 | Cd52 | 233 | 1.323 | 0.1299 | Yes |
| 8 | Ptprc | 255 | 1.283 | 0.1486 | Yes |
| 9 | H2-Q6 | 272 | 1.260 | 0.1684 | Yes |
| 10 | Hcst | 282 | 1.242 | 0.1903 | Yes |
| 11 | Ccr2 | 288 | 1.234 | 0.2133 | Yes |
| 12 | Selplg | 332 | 1.182 | 0.2225 | Yes |
| 13 | H2-Q7 | 347 | 1.168 | 0.2412 | Yes |
| 14 | Fxyd5 | 377 | 1.133 | 0.2542 | Yes |
| 15 | Il2rg | 380 | 1.128 | 0.2761 | Yes |
| 16 | B2m | 402 | 1.097 | 0.2910 | Yes |
| 17 | Gimap6 | 403 | 1.097 | 0.3129 | Yes |
| 18 | Lcp1 | 416 | 1.084 | 0.3306 | Yes |
| 19 | Crip1 | 431 | 1.069 | 0.3473 | Yes |
| 20 | Arhgdib | 445 | 1.049 | 0.3640 | Yes |
| 21 | Cotl1 | 447 | 1.049 | 0.3846 | Yes |
| 22 | Coro1a | 522 | 0.970 | 0.3793 | Yes |
| 23 | Cyba | 554 | 0.947 | 0.3879 | Yes |
| 24 | Hilpda | 636 | 0.860 | 0.3780 | Yes |
| 25 | Cenpa | 655 | 0.847 | 0.3889 | Yes |
| 26 | Laptm5 | 659 | 0.844 | 0.4048 | Yes |
| 27 | Irf8 | 663 | 0.840 | 0.4206 | Yes |
| 28 | Actr3 | 665 | 0.838 | 0.4370 | Yes |
| 29 | Ets1 | 671 | 0.831 | 0.4520 | Yes |
| 30 | H2-D1 | 722 | 0.794 | 0.4511 | Yes |
| 31 | Ifngr1 | 740 | 0.770 | 0.4608 | Yes |
| 32 | H2-K1 | 818 | 0.692 | 0.4489 | Yes |
| 33 | Psmb8 | 838 | 0.678 | 0.4561 | Yes |
| 34 | Calm2 | 857 | 0.664 | 0.4634 | Yes |
| 35 | Ostf1 | 862 | 0.654 | 0.4751 | Yes |
| 36 | Bcl2l11 | 885 | 0.641 | 0.4806 | Yes |
| 37 | Stk17b | 930 | 0.609 | 0.4781 | Yes |
| 38 | S100a10 | 965 | 0.592 | 0.4785 | Yes |
| 39 | Cfl1 | 973 | 0.581 | 0.4878 | Yes |
| 40 | Serpinb9 | 982 | 0.575 | 0.4967 | Yes |
| 41 | Dgat1 | 987 | 0.571 | 0.5067 | Yes |
| 42 | Sh3bgrl3 | 1006 | 0.560 | 0.5119 | Yes |
| 43 | Mbnl1 | 1018 | 0.553 | 0.5193 | Yes |
| 44 | Jak1 | 1040 | 0.543 | 0.5231 | Yes |
| 45 | Litaf | 1084 | 0.517 | 0.5191 | No |
| 46 | H3f3b | 1262 | -0.534 | 0.4705 | No |
| 47 | Fosl2 | 1283 | -0.538 | 0.4746 | No |
| 48 | Vps37b | 2349 | -0.962 | 0.1374 | No |
| 49 | Ly6e | 2392 | -1.001 | 0.1434 | No |
| 50 | Fam107b | 2420 | -1.020 | 0.1548 | No |
| 51 | Ptpn22 | 2611 | -1.223 | 0.1157 | No |
| 52 | Nr4a2 | 2700 | -1.361 | 0.1135 | No |
Table: GSEA details [plain text format]

  

Fig 2: ZHANG\_UTERUS\_C8\_NK\_CELL: Random ES distribution      
 Gene set null distribution of ES for **ZHANG\_UTERUS\_C8\_NK\_CELL**

  
