# Supplementary material for: TMPRSS11B promotes an acidified microenvironment and immune suppression in squamous lung cancer
Source: EMBO Rep. 2025 Nov 10;26(24):6346–79. doi: 10.1038/s44319-025-00631-1 (PMC12714794; doi:10.1038/s44319-025-00631-1)
Supplement: Supplementary file 11 — Source data Fig. 6 [file 44319_2025_631_MOESM11_ESM.zip › Figure 6/6D-E/GSEA Broad Institute_low pH vs rest of the regions (high pH)/ZHANG_UTERUS_C9_DENDRITIC_CELL.html]

Details for gene set ZHANG\_UTERUS\_C9\_DENDRITIC\_CELL[GSEA]

|  || Dataset | Lactate high vs low\_Ranked |
| Phenotype | NoPhenotypeAvailable |
| Upregulated in class | na\_pos |
| GeneSet | ZHANG\_UTERUS\_C9\_DENDRITIC\_CELL |
| Enrichment Score (ES) | 0.6999241 |
| Normalized Enrichment Score (NES) | 4.101417 |
| Nominal p-value | 0.0 |
| FDR q-value | 0.0 |
| FWER p-Value | 0.0 |
Table: GSEA Results Summary

  

Fig 1: Enrichment plot: ZHANG\_UTERUS\_C9\_DENDRITIC\_CELL      
 Profile of the Running ES Score & Positions of GeneSet Members on the Rank Ordered List

  

| SYMBOL | RANK IN GENE LIST | RANK METRIC SCORE | RUNNING ES | CORE ENRICHMENT || 1 | Ctss | 14 | 2.088 | 0.0298 | Yes |
| 2 | Lgmn | 28 | 1.878 | 0.0565 | Yes |
| 3 | Ms4a6c | 37 | 1.819 | 0.0839 | Yes |
| 4 | Mpeg1 | 42 | 1.799 | 0.1122 | Yes |
| 5 | Napsa | 50 | 1.758 | 0.1389 | Yes |
| 6 | Psap | 59 | 1.694 | 0.1643 | Yes |
| 7 | Tyrobp | 83 | 1.618 | 0.1833 | Yes |
| 8 | Spi1 | 86 | 1.616 | 0.2093 | Yes |
| 9 | Fcer1g | 95 | 1.597 | 0.2330 | Yes |
| 10 | Cd83 | 101 | 1.575 | 0.2574 | Yes |
| 11 | Rgs1 | 105 | 1.567 | 0.2822 | Yes |
| 12 | Plek | 135 | 1.509 | 0.2975 | Yes |
| 13 | Cd53 | 139 | 1.488 | 0.3210 | Yes |
| 14 | Cd52 | 233 | 1.323 | 0.3118 | Yes |
| 15 | Bcl2a1b | 241 | 1.307 | 0.3310 | Yes |
| 16 | Ctsz | 242 | 1.303 | 0.3525 | Yes |
| 17 | Gm2a | 274 | 1.259 | 0.3630 | Yes |
| 18 | Lgals3 | 344 | 1.170 | 0.3592 | Yes |
| 19 | Wfdc17 | 345 | 1.169 | 0.3785 | Yes |
| 20 | H2-DMb1 | 348 | 1.167 | 0.3971 | Yes |
| 21 | Alox5ap | 375 | 1.133 | 0.4071 | Yes |
| 22 | Cd74 | 376 | 1.133 | 0.4258 | Yes |
| 23 | Fxyd5 | 377 | 1.133 | 0.4446 | Yes |
| 24 | B2m | 402 | 1.097 | 0.4546 | Yes |
| 25 | H2-Ab1 | 404 | 1.094 | 0.4724 | Yes |
| 26 | Lcp1 | 416 | 1.084 | 0.4866 | Yes |
| 27 | H2-DMa | 418 | 1.083 | 0.5042 | Yes |
| 28 | H2-Eb1 | 425 | 1.075 | 0.5199 | Yes |
| 29 | Crip1 | 431 | 1.069 | 0.5359 | Yes |
| 30 | Arhgdib | 445 | 1.049 | 0.5489 | Yes |
| 31 | Cotl1 | 447 | 1.049 | 0.5659 | Yes |
| 32 | Lsp1 | 459 | 1.039 | 0.5794 | Yes |
| 33 | Unc93b1 | 464 | 1.037 | 0.5952 | Yes |
| 34 | H2-Aa | 465 | 1.035 | 0.6123 | Yes |
| 35 | Cxcl16 | 479 | 1.013 | 0.6246 | Yes |
| 36 | Fth1 | 503 | 0.986 | 0.6332 | Yes |
| 37 | Coro1a | 522 | 0.970 | 0.6432 | Yes |
| 38 | Ctsc | 525 | 0.966 | 0.6585 | Yes |
| 39 | Cyba | 554 | 0.947 | 0.6648 | Yes |
| 40 | Rilpl2 | 580 | 0.918 | 0.6716 | Yes |
| 41 | Laptm5 | 659 | 0.844 | 0.6594 | Yes |
| 42 | H2-D1 | 722 | 0.794 | 0.6518 | Yes |
| 43 | Cdkn1a | 749 | 0.765 | 0.6557 | Yes |
| 44 | Ccl6 | 772 | 0.736 | 0.6605 | Yes |
| 45 | Cst3 | 782 | 0.723 | 0.6694 | Yes |
| 46 | H2-K1 | 818 | 0.692 | 0.6691 | Yes |
| 47 | Psmb8 | 838 | 0.678 | 0.6740 | Yes |
| 48 | Actb | 856 | 0.664 | 0.6793 | Yes |
| 49 | Pim1 | 859 | 0.656 | 0.6894 | Yes |
| 50 | Mcl1 | 861 | 0.655 | 0.6999 | Yes |
| 51 | Cfl1 | 973 | 0.581 | 0.6724 | No |
| 52 | Picalm | 993 | 0.568 | 0.6754 | No |
| 53 | Sh3bgrl3 | 1006 | 0.560 | 0.6806 | No |
Table: GSEA details [plain text format]

  

Fig 2: ZHANG\_UTERUS\_C9\_DENDRITIC\_CELL: Random ES distribution      
 Gene set null distribution of ES for **ZHANG\_UTERUS\_C9\_DENDRITIC\_CELL**

  
