# Supplementary material for: TMPRSS11B promotes an acidified microenvironment and immune suppression in squamous lung cancer
Source: EMBO Rep. 2025 Nov 10;26(24):6346–79. doi: 10.1038/s44319-025-00631-1 (PMC12714794; doi:10.1038/s44319-025-00631-1)
Supplement: Supplementary file 12 — Source data Fig. 7 [file 44319_2025_631_MOESM12_ESM.zip › Figure 7/Read Me.rtf]

HHSE51_NT and HHSE53_NT represent the mouse IDs. The harvested lungs from each mouse was split into two halves for snap freezing and downstream analysis, denoted as L1 and L2. Also, for each half,  the LMD sections were obtained at an interval of 500mm on the z-plane, with the closest serial section for each being used for the corresponding H&Es. Hence the LMD IDs of L1_1/8/15 (or L2_1/8/15) and corresponding H&E IDs of L1_2/9/16 (or L2_2/9/16).
