# Supplementary material for: TMPRSS11B promotes an acidified microenvironment and immune suppression in squamous lung cancer
Source: EMBO Rep. 2025 Nov 10;26(24):6346–79. doi: 10.1038/s44319-025-00631-1 (PMC12714794; doi:10.1038/s44319-025-00631-1)

1. **100bp ladder**
2. **Control sgRNA (Sur_1 primers)**
3. T11b sgRNA 3 (Sur_1 primers)
4. Control sgRNA (Sur_2 primers)
5. T11b sgRNA 3 (Sur_2 primers)
6. **T11b sgRNA 1 (Sur_1 primers)**
7. Control sgRNA (Sur_3 primers)
8. T11b sgRNA 1 (Sur_3 primers)
9. Control sgRNA (Sur_4 primers)
10. T11b sgRNA 2 (Sur_4 primers)
11. Control sgRNA (Sur_5 primers)
12. T11b sgRNA 2 (Sur_5 primers)
13. Negative (no DNA) control
14. Negative (no DNA) control
15. 100bp ladder

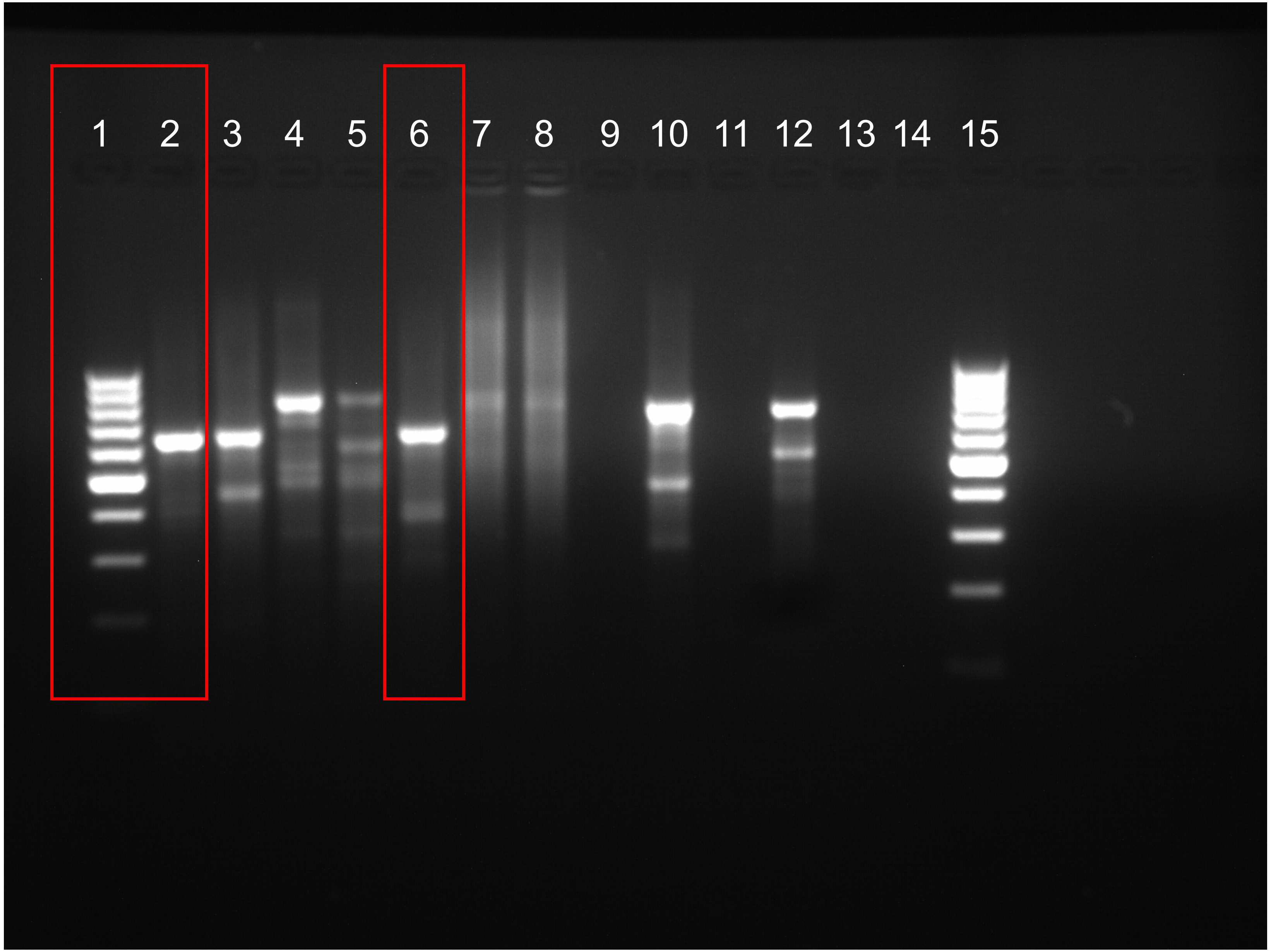

1. **100bp ladder**
2. Control sgRNA (Sur_1 primers)
3. T11b sgRNA 3 (Sur_1 primers)
4. Control sgRNA (Sur_2 primers)
5. T11b sgRNA 3 (Sur_2 primers)
6. Control sgRNA (Sur_1 primers)
7. T11b sgRNA 1 (Sur_1 primers)
8. **Control sgRNA (Sur_4 primers)**
9. **T11b sgRNA 2 (Sur_4 primers)**
10. Control sgRNA (Sur_5 primers)
11. T11b sgRNA 2 (Sur_5 primers)
12. Negative (no enzyme) control
13. Negative (no enzyme) control
14. Negative (no enzyme) control
15. Negative (no enzyme) control
16. Positive control (enzyme + DNA from kit)
17. Negative control (no enzyme + DNA from kit)
18. 100bp ladder

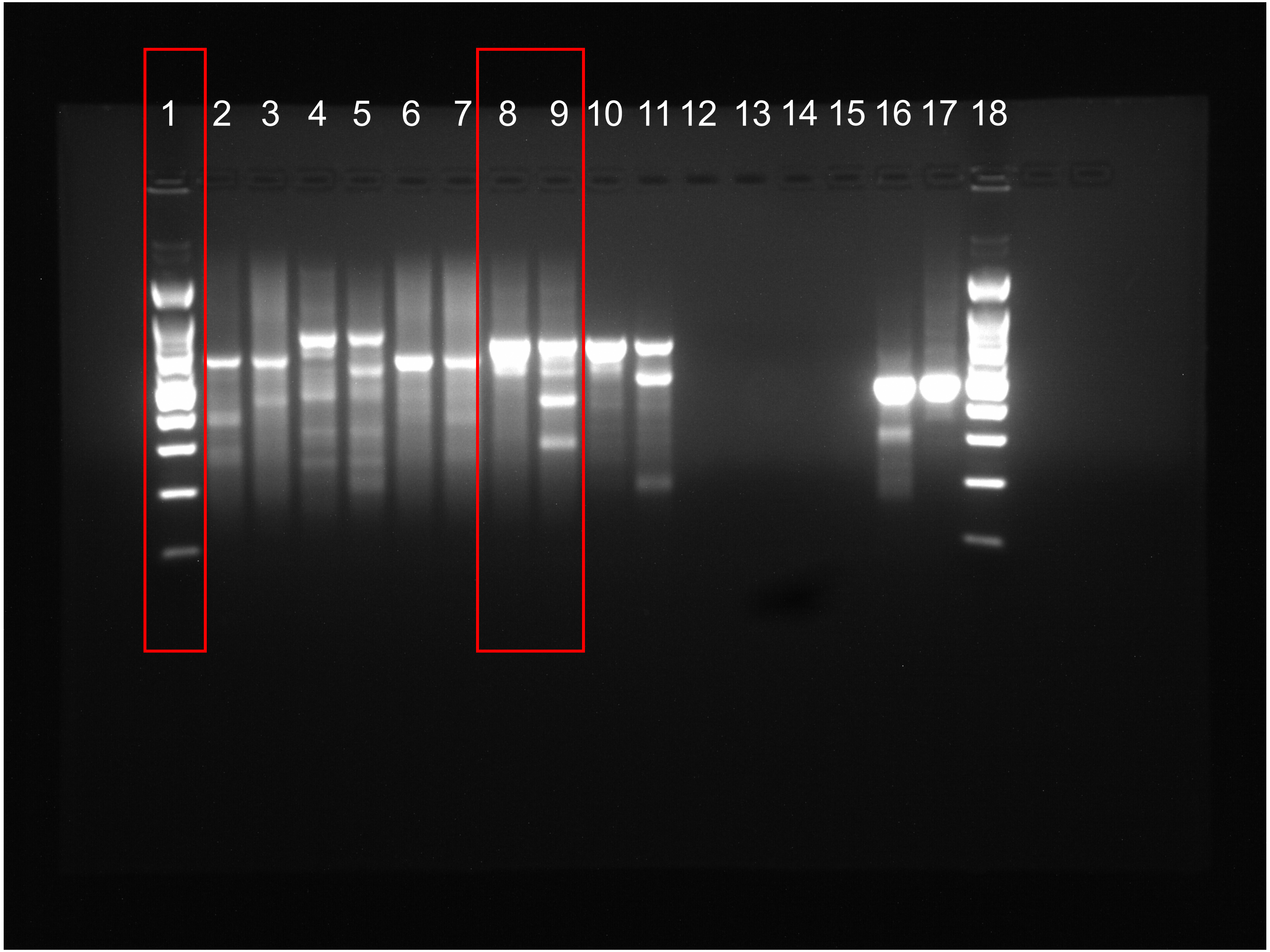

Supplement: Supplementary file 13 — Figure EV1 Source Data [file 44319_2025_631_MOESM13_ESM.zip › Figure EV1/EV1A/Surveyor assay gels (labelled).pdf]
