# Supplementary material for: TMPRSS11B promotes an acidified microenvironment and immune suppression in squamous lung cancer
Source: EMBO Rep. 2025 Nov 10;26(24):6346–79. doi: 10.1038/s44319-025-00631-1 (PMC12714794; doi:10.1038/s44319-025-00631-1)
Supplement: Supplementary file 14 — Figure EV2 Source Data [file 44319_2025_631_MOESM14_ESM.zip › Figure EV2/EV2D-E/GSEA_Broad Institute_Mh_T11b-high LUSC vs LUAD/gsea_report_for_na_neg_1723673850052.html]

Report for na\_neg 1723673850052 [GSEA]

| GS  follow link to MSigDB | GS DETAILS | SIZE | ES | NES | NOM p-val | FDR q-val | FWER p-val | RANK AT MAX | LEADING EDGE || 1 | HALLMARK\_MYOGENESIS | Details ... | 64 | -0.29 | -1.48 | 0.041 | 0.562 | 0.564 | 506 | tags=22%, list=10%, signal=24% |
| 2 | HALLMARK\_ANDROGEN\_RESPONSE | Details ... | 42 | -0.30 | -1.37 | 0.089 | 0.523 | 0.795 | 1190 | tags=48%, list=25%, signal=63% |
| 3 | HALLMARK\_BILE\_ACID\_METABOLISM | Details ... | 41 | -0.23 | -1.04 | 0.396 | 1.000 | 1.000 | 842 | tags=29%, list=17%, signal=35% |
| 4 | HALLMARK\_PROTEIN\_SECRETION | Details ... | 30 | -0.25 | -1.04 | 0.418 | 1.000 | 1.000 | 634 | tags=23%, list=13%, signal=27% |
| 5 | HALLMARK\_ADIPOGENESIS | Details ... | 80 | -0.18 | -0.96 | 0.505 | 1.000 | 1.000 | 1323 | tags=38%, list=27%, signal=51% |
| 6 | HALLMARK\_TGF\_BETA\_SIGNALING | Details ... | 27 | -0.23 | -0.95 | 0.514 | 1.000 | 1.000 | 2259 | tags=63%, list=47%, signal=118% |
| 7 | HALLMARK\_OXIDATIVE\_PHOSPHORYLATION | Details ... | 48 | -0.19 | -0.91 | 0.595 | 1.000 | 1.000 | 2976 | tags=73%, list=62%, signal=189% |
| 8 | HALLMARK\_SPERMATOGENESIS | Details ... | 16 | -0.20 | -0.69 | 0.852 | 1.000 | 1.000 | 2183 | tags=56%, list=45%, signal=102% |
| 9 | HALLMARK\_DNA\_REPAIR | Details ... | 53 | -0.14 | -0.68 | 0.910 | 1.000 | 1.000 | 3246 | tags=81%, list=67%, signal=246% |
| 10 | HALLMARK\_MITOTIC\_SPINDLE | Details ... | 66 | -0.12 | -0.63 | 0.956 | 1.000 | 1.000 | 1655 | tags=36%, list=34%, signal=55% |
| 11 | HALLMARK\_UV\_RESPONSE\_DN | Details ... | 70 | -0.12 | -0.63 | 0.932 | 1.000 | 1.000 | 1825 | tags=39%, list=38%, signal=61% |
| 12 | HALLMARK\_G2M\_CHECKPOINT | Details ... | 61 | -0.12 | -0.62 | 0.958 | 1.000 | 1.000 | 4240 | tags=100%, list=88%, signal=819% |
| 13 | HALLMARK\_UNFOLDED\_PROTEIN\_RESPONSE | Details ... | 39 | -0.13 | -0.59 | 0.972 | 0.964 | 1.000 | 2100 | tags=46%, list=44%, signal=81% |
Table: Gene sets enriched in phenotype **na**[plain text format]****

  
