# Supplementary material for: TMPRSS11B promotes an acidified microenvironment and immune suppression in squamous lung cancer
Source: EMBO Rep. 2025 Nov 10;26(24):6346–79. doi: 10.1038/s44319-025-00631-1 (PMC12714794; doi:10.1038/s44319-025-00631-1)
Supplement: Supplementary file 14 — Figure EV2 Source Data [file 44319_2025_631_MOESM14_ESM.zip › Figure EV2/EV2D-E/GSEA_Broad Institute_Mh_T11b-high LUSC vs LUAD/gsea_report_for_na_pos_1723673850052.html]

Report for na\_pos 1723673850052 [GSEA]

| GS  follow link to MSigDB | GS DETAILS | SIZE | ES | NES | NOM p-val | FDR q-val | FWER p-val | RANK AT MAX | LEADING EDGE || 1 | HALLMARK\_COMPLEMENT | Details ... | 71 | 0.52 | 2.45 | 0.000 | 0.000 | 0.000 | 805 | tags=49%, list=17%, signal=58% |
| 2 | HALLMARK\_KRAS\_SIGNALING\_UP | Details ... | 94 | 0.45 | 2.23 | 0.000 | 0.000 | 0.000 | 477 | tags=31%, list=10%, signal=34% |
| 3 | HALLMARK\_INFLAMMATORY\_RESPONSE | Details ... | 67 | 0.47 | 2.23 | 0.000 | 0.000 | 0.000 | 1070 | tags=63%, list=22%, signal=79% |
| 4 | HALLMARK\_KRAS\_SIGNALING\_DN | Details ... | 47 | 0.51 | 2.18 | 0.000 | 0.000 | 0.000 | 222 | tags=26%, list=5%, signal=27% |
| 5 | HALLMARK\_IL6\_JAK\_STAT3\_SIGNALING | Details ... | 27 | 0.55 | 2.08 | 0.000 | 0.001 | 0.003 | 508 | tags=44%, list=11%, signal=49% |
| 6 | HALLMARK\_EPITHELIAL\_MESENCHYMAL\_TRANSITION | Details ... | 74 | 0.42 | 2.03 | 0.003 | 0.002 | 0.012 | 687 | tags=38%, list=14%, signal=43% |
| 7 | HALLMARK\_P53\_PATHWAY | Details ... | 95 | 0.39 | 1.98 | 0.000 | 0.004 | 0.024 | 861 | tags=41%, list=18%, signal=49% |
| 8 | HALLMARK\_ALLOGRAFT\_REJECTION | Details ... | 49 | 0.45 | 1.95 | 0.000 | 0.005 | 0.033 | 940 | tags=55%, list=19%, signal=68% |
| 9 | HALLMARK\_TNFA\_SIGNALING\_VIA\_NFKB | Details ... | 90 | 0.38 | 1.90 | 0.000 | 0.008 | 0.060 | 1129 | tags=56%, list=23%, signal=71% |
| 10 | HALLMARK\_ESTROGEN\_RESPONSE\_LATE | Details ... | 84 | 0.36 | 1.76 | 0.012 | 0.027 | 0.200 | 737 | tags=33%, list=15%, signal=39% |
| 11 | HALLMARK\_COAGULATION | Details ... | 44 | 0.41 | 1.75 | 0.012 | 0.025 | 0.206 | 526 | tags=34%, list=11%, signal=38% |
| 12 | HALLMARK\_ANGIOGENESIS | Details ... | 16 | 0.52 | 1.72 | 0.019 | 0.030 | 0.256 | 498 | tags=38%, list=10%, signal=42% |
| 13 | HALLMARK\_APICAL\_JUNCTION | Details ... | 65 | 0.36 | 1.71 | 0.007 | 0.031 | 0.286 | 903 | tags=42%, list=19%, signal=50% |
| 14 | HALLMARK\_IL2\_STAT5\_SIGNALING | Details ... | 82 | 0.35 | 1.69 | 0.002 | 0.032 | 0.317 | 583 | tags=26%, list=12%, signal=29% |
| 15 | HALLMARK\_ESTROGEN\_RESPONSE\_EARLY | Details ... | 96 | 0.31 | 1.59 | 0.013 | 0.059 | 0.538 | 753 | tags=29%, list=16%, signal=34% |
| 16 | HALLMARK\_MTORC1\_SIGNALING | Details ... | 52 | 0.35 | 1.58 | 0.037 | 0.061 | 0.565 | 1164 | tags=60%, list=24%, signal=78% |
| 17 | HALLMARK\_INTERFERON\_GAMMA\_RESPONSE | Details ... | 63 | 0.34 | 1.57 | 0.033 | 0.060 | 0.582 | 1147 | tags=54%, list=24%, signal=70% |
| 18 | HALLMARK\_HYPOXIA | Details ... | 77 | 0.31 | 1.51 | 0.039 | 0.086 | 0.724 | 863 | tags=35%, list=18%, signal=42% |
| 19 | HALLMARK\_APICAL\_SURFACE | Details ... | 21 | 0.39 | 1.39 | 0.119 | 0.155 | 0.925 | 805 | tags=38%, list=17%, signal=46% |
| 20 | HALLMARK\_APOPTOSIS | Details ... | 69 | 0.27 | 1.25 | 0.176 | 0.289 | 0.995 | 1000 | tags=36%, list=21%, signal=45% |
| 21 | HALLMARK\_XENOBIOTIC\_METABOLISM |  | 89 | 0.25 | 1.23 | 0.172 | 0.305 | 0.998 | 731 | tags=26%, list=15%, signal=30% |
| 22 | HALLMARK\_REACTIVE\_OXYGEN\_SPECIES\_PATHWAY |  | 21 | 0.35 | 1.21 | 0.240 | 0.312 | 0.998 | 1120 | tags=52%, list=23%, signal=68% |
| 23 | HALLMARK\_E2F\_TARGETS |  | 45 | 0.28 | 1.18 | 0.248 | 0.333 | 0.999 | 2353 | tags=82%, list=49%, signal=159% |
| 24 | HALLMARK\_UV\_RESPONSE\_UP |  | 57 | 0.21 | 0.97 | 0.501 | 0.677 | 1.000 | 1177 | tags=39%, list=24%, signal=50% |
| 25 | HALLMARK\_INTERFERON\_ALPHA\_RESPONSE |  | 37 | 0.23 | 0.93 | 0.554 | 0.710 | 1.000 | 1104 | tags=43%, list=23%, signal=56% |
| 26 | HALLMARK\_PI3K\_AKT\_MTOR\_SIGNALING |  | 23 | 0.25 | 0.91 | 0.555 | 0.723 | 1.000 | 1178 | tags=48%, list=24%, signal=63% |
| 27 | HALLMARK\_CHOLESTEROL\_HOMEOSTASIS |  | 34 | 0.22 | 0.91 | 0.583 | 0.704 | 1.000 | 1054 | tags=44%, list=22%, signal=56% |
| 28 | HALLMARK\_MYC\_TARGETS\_V1 |  | 29 | 0.24 | 0.90 | 0.588 | 0.697 | 1.000 | 2712 | tags=86%, list=56%, signal=196% |
| 29 | HALLMARK\_GLYCOLYSIS |  | 90 | 0.14 | 0.68 | 0.913 | 0.984 | 1.000 | 1051 | tags=31%, list=22%, signal=39% |
| 30 | HALLMARK\_FATTY\_ACID\_METABOLISM |  | 71 | 0.12 | 0.59 | 0.964 | 1.000 | 1.000 | 450 | tags=10%, list=9%, signal=11% |
| 31 | HALLMARK\_PEROXISOME |  | 35 | 0.14 | 0.58 | 0.928 | 0.986 | 1.000 | 1177 | tags=37%, list=24%, signal=49% |
| 32 | HALLMARK\_HEME\_METABOLISM |  | 76 | 0.12 | 0.56 | 0.985 | 0.967 | 1.000 | 876 | tags=22%, list=18%, signal=27% |
Table: Gene sets enriched in phenotype **na**[plain text format]****

  
