# Supplementary material for: TMPRSS11B promotes an acidified microenvironment and immune suppression in squamous lung cancer
Source: EMBO Rep. 2025 Nov 10;26(24):6346–79. doi: 10.1038/s44319-025-00631-1 (PMC12714794; doi:10.1038/s44319-025-00631-1)
Supplement: Supplementary file 14 — Figure EV2 Source Data [file 44319_2025_631_MOESM14_ESM.zip › Figure EV2/EV2D-E/GSEA_Broad Institute_Mh_T11b-high LUSC vs LUAD/HALLMARK_ADIPOGENESIS.html]

Details for gene set HALLMARK\_ADIPOGENESIS[GSEA]

|  || Dataset | Ranked list\_DGE\_squamousT11b\_vs\_all adenosadeno\_HSE13-NT copy |
| Phenotype | NoPhenotypeAvailable |
| Upregulated in class | na\_neg |
| GeneSet | HALLMARK\_ADIPOGENESIS |
| Enrichment Score (ES) | -0.177787 |
| Normalized Enrichment Score (NES) | -0.96480465 |
| Nominal p-value | 0.5054054 |
| FDR q-value | 1.0 |
| FWER p-Value | 1.0 |
Table: GSEA Results Summary

  

Fig 1: Enrichment plot: HALLMARK\_ADIPOGENESIS      
 Profile of the Running ES Score & Positions of GeneSet Members on the Rank Ordered List

  

| SYMBOL | RANK IN GENE LIST | RANK METRIC SCORE | RUNNING ES | CORE ENRICHMENT || 1 | Lpl | 149 | 3.044 | 0.0021 | No |
| 2 | Abca1 | 172 | 2.809 | 0.0284 | No |
| 3 | Cd36 | 199 | 2.567 | 0.0512 | No |
| 4 | Orm1 | 262 | 2.265 | 0.0630 | No |
| 5 | Sorbs1 | 389 | 1.734 | 0.0556 | No |
| 6 | C3 | 474 | 1.502 | 0.0544 | No |
| 7 | Apoe | 490 | 1.475 | 0.0675 | No |
| 8 | Plin2 | 507 | 1.431 | 0.0799 | No |
| 9 | Cd302 | 521 | 1.393 | 0.0924 | No |
| 10 | Fah | 583 | 1.236 | 0.0932 | No |
| 11 | Gpat4 | 619 | 1.171 | 0.0987 | No |
| 12 | Stom | 675 | 1.042 | 0.0986 | No |
| 13 | Atp1b3 | 686 | 1.028 | 0.1078 | No |
| 14 | Cat | 712 | 0.989 | 0.1134 | No |
| 15 | Mgll | 752 | 0.929 | 0.1154 | No |
| 16 | Rab34 | 798 | 0.872 | 0.1155 | No |
| 17 | Ccng2 | 808 | 0.859 | 0.1231 | No |
| 18 | Sowahc | 868 | 0.805 | 0.1195 | No |
| 19 | Fabp4 | 914 | 0.747 | 0.1182 | No |
| 20 | Col4a1 | 975 | 0.682 | 0.1131 | No |
| 21 | Nabp1 | 1119 | 0.540 | 0.0889 | No |
| 22 | Tank | 1129 | 0.529 | 0.0928 | No |
| 23 | Ifngr1 | 1155 | 0.509 | 0.0931 | No |
| 24 | Hibch | 1218 | -0.508 | 0.0856 | No |
| 25 | Rmdn3 | 1606 | -0.569 | 0.0103 | No |
| 26 | Rnf11 | 1636 | -0.573 | 0.0105 | No |
| 27 | Suclg1 | 1762 | -0.593 | -0.0094 | No |
| 28 | Acaa2 | 1846 | -0.609 | -0.0201 | No |
| 29 | Por | 1937 | -0.624 | -0.0323 | No |
| 30 | Qdpr | 1949 | -0.627 | -0.0277 | No |
| 31 | Acadl | 2030 | -0.641 | -0.0375 | No |
| 32 | Gpd2 | 2056 | -0.645 | -0.0357 | No |
| 33 | Adipor2 | 2276 | -0.684 | -0.0743 | No |
| 34 | Aldh2 | 2388 | -0.704 | -0.0900 | No |
| 35 | Idh3g | 2455 | -0.719 | -0.0960 | No |
| 36 | Acads | 2492 | -0.727 | -0.0956 | No |
| 37 | Itsn1 | 2604 | -0.749 | -0.1108 | No |
| 38 | Cpt2 | 2653 | -0.759 | -0.1125 | No |
| 39 | Sod1 | 2688 | -0.765 | -0.1113 | No |
| 40 | Preb | 2722 | -0.771 | -0.1097 | No |
| 41 | Reep6 | 2730 | -0.773 | -0.1027 | No |
| 42 | Ddt | 2787 | -0.788 | -0.1058 | No |
| 43 | Bckdha | 2848 | -0.802 | -0.1096 | No |
| 44 | Mccc1 | 2867 | -0.806 | -0.1046 | No |
| 45 | Ephx2 | 2986 | -0.838 | -0.1202 | No |
| 46 | G3bp2 | 3060 | -0.858 | -0.1262 | No |
| 47 | Stat5a | 3062 | -0.858 | -0.1169 | No |
| 48 | Idh1 | 3183 | -0.894 | -0.1324 | No |
| 49 | Fzd4 | 3311 | -0.934 | -0.1489 | No |
| 50 | Sqor | 3336 | -0.941 | -0.1436 | No |
| 51 | Slc1a5 | 3499 | -0.997 | -0.1668 | Yes |
| 52 | Aplp2 | 3500 | -0.997 | -0.1558 | Yes |
| 53 | Col15a1 | 3582 | -1.026 | -0.1616 | Yes |
| 54 | Immt | 3616 | -1.038 | -0.1572 | Yes |
| 55 | Abcb8 | 3666 | -1.062 | -0.1558 | Yes |
| 56 | Decr1 | 3683 | -1.070 | -0.1474 | Yes |
| 57 | Cavin1 | 3689 | -1.073 | -0.1366 | Yes |
| 58 | Ech1 | 3705 | -1.083 | -0.1279 | Yes |
| 59 | Pdcd4 | 3741 | -1.099 | -0.1231 | Yes |
| 60 | Lifr | 3748 | -1.101 | -0.1123 | Yes |
| 61 | Lpcat3 | 3806 | -1.131 | -0.1119 | Yes |
| 62 | Acadm | 3866 | -1.164 | -0.1115 | Yes |
| 63 | Pim3 | 3892 | -1.181 | -0.1038 | Yes |
| 64 | Chuk | 3945 | -1.214 | -0.1013 | Yes |
| 65 | Hadh | 3948 | -1.215 | -0.0884 | Yes |
| 66 | Slc25a1 | 3974 | -1.228 | -0.0801 | Yes |
| 67 | Tob1 | 4038 | -1.273 | -0.0794 | Yes |
| 68 | Atl2 | 4069 | -1.299 | -0.0714 | Yes |
| 69 | Sult1a1 | 4112 | -1.339 | -0.0656 | Yes |
| 70 | Dbt | 4252 | -1.468 | -0.0787 | Yes |
| 71 | Slc25a10 | 4253 | -1.469 | -0.0625 | Yes |
| 72 | Itga7 | 4326 | -1.538 | -0.0608 | Yes |
| 73 | Adcy6 | 4381 | -1.614 | -0.0544 | Yes |
| 74 | Pparg | 4408 | -1.658 | -0.0416 | Yes |
| 75 | Cmbl | 4433 | -1.707 | -0.0279 | Yes |
| 76 | Phyh | 4469 | -1.768 | -0.0158 | Yes |
| 77 | Phldb1 | 4534 | -1.878 | -0.0086 | Yes |
| 78 | Sncg | 4551 | -1.917 | 0.0091 | Yes |
| 79 | Cavin2 | 4560 | -1.944 | 0.0288 | Yes |
| 80 | Cyp4b1 | 4689 | -2.344 | 0.0276 | Yes |
Table: GSEA details [plain text format]

  

Fig 2: HALLMARK\_ADIPOGENESIS: Random ES distribution      
 Gene set null distribution of ES for **HALLMARK\_ADIPOGENESIS**

  
