# Supplementary material for: TMPRSS11B promotes an acidified microenvironment and immune suppression in squamous lung cancer
Source: EMBO Rep. 2025 Nov 10;26(24):6346–79. doi: 10.1038/s44319-025-00631-1 (PMC12714794; doi:10.1038/s44319-025-00631-1)
Supplement: Supplementary file 14 — Figure EV2 Source Data [file 44319_2025_631_MOESM14_ESM.zip › Figure EV2/EV2D-E/GSEA_Broad Institute_Mh_T11b-high LUSC vs LUAD/HALLMARK_ALLOGRAFT_REJECTION.html]

Details for gene set HALLMARK\_ALLOGRAFT\_REJECTION[GSEA]

|  || Dataset | Ranked list\_DGE\_squamousT11b\_vs\_all adenosadeno\_HSE13-NT copy |
| Phenotype | NoPhenotypeAvailable |
| Upregulated in class | na\_pos |
| GeneSet | HALLMARK\_ALLOGRAFT\_REJECTION |
| Enrichment Score (ES) | 0.4456879 |
| Normalized Enrichment Score (NES) | 1.9513141 |
| Nominal p-value | 0.0 |
| FDR q-value | 0.005058664 |
| FWER p-Value | 0.033 |
Table: GSEA Results Summary

  

Fig 1: Enrichment plot: HALLMARK\_ALLOGRAFT\_REJECTION      
 Profile of the Running ES Score & Positions of GeneSet Members on the Rank Ordered List

  

| SYMBOL | RANK IN GENE LIST | RANK METRIC SCORE | RUNNING ES | CORE ENRICHMENT || 1 | Mmp9 | 114 | 3.578 | 0.0277 | Yes |
| 2 | Igsf6 | 147 | 3.056 | 0.0651 | Yes |
| 3 | Itgb2 | 158 | 2.937 | 0.1054 | Yes |
| 4 | Srgn | 185 | 2.715 | 0.1391 | Yes |
| 5 | Il1b | 240 | 2.351 | 0.1617 | Yes |
| 6 | Ctss | 247 | 2.317 | 0.1939 | Yes |
| 7 | Spi1 | 250 | 2.309 | 0.2268 | Yes |
| 8 | Fcgr2b | 280 | 2.178 | 0.2522 | Yes |
| 9 | Ptprc | 338 | 1.926 | 0.2680 | Yes |
| 10 | Was | 373 | 1.768 | 0.2864 | Yes |
| 11 | Tnf | 391 | 1.722 | 0.3077 | Yes |
| 12 | Lcp2 | 403 | 1.676 | 0.3296 | Yes |
| 13 | Cfp | 433 | 1.599 | 0.3466 | Yes |
| 14 | Gpr65 | 478 | 1.498 | 0.3589 | Yes |
| 15 | Hcls1 | 487 | 1.480 | 0.3786 | Yes |
| 16 | Capg | 574 | 1.263 | 0.3788 | Yes |
| 17 | Irf7 | 617 | 1.174 | 0.3870 | Yes |
| 18 | Il2rg | 648 | 1.091 | 0.3964 | Yes |
| 19 | Tgfb2 | 720 | 0.981 | 0.3957 | Yes |
| 20 | B2m | 794 | 0.876 | 0.3930 | Yes |
| 21 | Lyn | 805 | 0.861 | 0.4034 | Yes |
| 22 | Hif1a | 810 | 0.857 | 0.4149 | Yes |
| 23 | Cd74 | 811 | 0.856 | 0.4273 | Yes |
| 24 | H2-DMb2 | 850 | 0.813 | 0.4310 | Yes |
| 25 | Ets1 | 863 | 0.807 | 0.4402 | Yes |
| 26 | Flna | 905 | 0.755 | 0.4425 | Yes |
| 27 | Tgfb1 | 940 | 0.718 | 0.4457 | Yes |
| 28 | Icam1 | 1070 | 0.586 | 0.4271 | No |
| 29 | Ifnar2 | 1147 | 0.514 | 0.4186 | No |
| 30 | Ifngr1 | 1155 | 0.509 | 0.4245 | No |
| 31 | F2r | 1319 | -0.521 | 0.3978 | No |
| 32 | Akt1 | 1371 | -0.529 | 0.3948 | No |
| 33 | Il4ra | 1455 | -0.542 | 0.3852 | No |
| 34 | Acvr2a | 1588 | -0.565 | 0.3657 | No |
| 35 | Ikbkb | 1797 | -0.599 | 0.3308 | No |
| 36 | Cdkn2a | 2149 | -0.662 | 0.2668 | No |
| 37 | Socs5 | 2503 | -0.728 | 0.2033 | No |
| 38 | Galnt1 | 2630 | -0.754 | 0.1878 | No |
| 39 | Jak2 | 2891 | -0.813 | 0.1450 | No |
| 40 | Inhbb | 3105 | -0.872 | 0.1129 | No |
| 41 | Gcnt1 | 3577 | -1.024 | 0.0290 | No |
| 42 | Traf2 | 3686 | -1.072 | 0.0218 | No |
| 43 | Elf4 | 3767 | -1.109 | 0.0211 | No |
| 44 | Eif3j1 | 4158 | -1.377 | -0.0408 | No |
| 45 | Bcat1 | 4607 | -2.061 | -0.1049 | No |
| 46 | Ccnd2 | 4672 | -2.265 | -0.0856 | No |
| 47 | Tlr2 | 4716 | -2.445 | -0.0594 | No |
| 48 | Ereg | 4731 | -2.541 | -0.0257 | No |
| 49 | Il18 | 4789 | -3.056 | 0.0065 | No |
Table: GSEA details [plain text format]

  

Fig 2: HALLMARK\_ALLOGRAFT\_REJECTION: Random ES distribution      
 Gene set null distribution of ES for **HALLMARK\_ALLOGRAFT\_REJECTION**

  
