# Supplementary material for: TMPRSS11B promotes an acidified microenvironment and immune suppression in squamous lung cancer
Source: EMBO Rep. 2025 Nov 10;26(24):6346–79. doi: 10.1038/s44319-025-00631-1 (PMC12714794; doi:10.1038/s44319-025-00631-1)
Supplement: Supplementary file 14 — Figure EV2 Source Data [file 44319_2025_631_MOESM14_ESM.zip › Figure EV2/EV2D-E/GSEA_Broad Institute_Mh_T11b-high LUSC vs LUAD/HALLMARK_ANDROGEN_RESPONSE.html]

Details for gene set HALLMARK\_ANDROGEN\_RESPONSE[GSEA]

|  || Dataset | Ranked list\_DGE\_squamousT11b\_vs\_all adenosadeno\_HSE13-NT copy |
| Phenotype | NoPhenotypeAvailable |
| Upregulated in class | na\_neg |
| GeneSet | HALLMARK\_ANDROGEN\_RESPONSE |
| Enrichment Score (ES) | -0.2986555 |
| Normalized Enrichment Score (NES) | -1.3716227 |
| Nominal p-value | 0.0890411 |
| FDR q-value | 0.5231148 |
| FWER p-Value | 0.795 |
Table: GSEA Results Summary

  

Fig 1: Enrichment plot: HALLMARK\_ANDROGEN\_RESPONSE      
 Profile of the Running ES Score & Positions of GeneSet Members on the Rank Ordered List

  

| SYMBOL | RANK IN GENE LIST | RANK METRIC SCORE | RUNNING ES | CORE ENRICHMENT || 1 | Aldh1a3 | 48 | 4.574 | 0.0698 | No |
| 2 | Plpp1 | 136 | 3.208 | 0.1076 | No |
| 3 | Mertk | 402 | 1.676 | 0.0814 | No |
| 4 | Maf | 421 | 1.624 | 0.1060 | No |
| 5 | Stk39 | 469 | 1.511 | 0.1226 | No |
| 6 | Fads1 | 568 | 1.289 | 0.1246 | No |
| 7 | Sat1 | 614 | 1.180 | 0.1358 | No |
| 8 | Ndrg1 | 681 | 1.033 | 0.1400 | No |
| 9 | Rrp12 | 753 | 0.927 | 0.1413 | No |
| 10 | B2m | 794 | 0.876 | 0.1482 | No |
| 11 | Elovl5 | 882 | 0.781 | 0.1437 | No |
| 12 | Cdk6 | 1002 | 0.648 | 0.1301 | No |
| 13 | Sord | 1146 | 0.514 | 0.1091 | No |
| 14 | Vapa | 1180 | -0.502 | 0.1110 | No |
| 15 | Akt1 | 1371 | -0.529 | 0.0805 | No |
| 16 | Xrcc6 | 1484 | -0.549 | 0.0666 | No |
| 17 | Herc3 | 1591 | -0.565 | 0.0543 | No |
| 18 | Slc38a2 | 1738 | -0.589 | 0.0341 | No |
| 19 | Slc26a2 | 2768 | -0.784 | -0.1676 | No |
| 20 | Sgk1 | 3151 | -0.885 | -0.2321 | No |
| 21 | Ell2 | 3280 | -0.924 | -0.2427 | No |
| 22 | Xrcc5 | 3481 | -0.991 | -0.2673 | No |
| 23 | Abhd2 | 3632 | -1.044 | -0.2804 | Yes |
| 24 | Pias1 | 3672 | -1.064 | -0.2700 | Yes |
| 25 | Tsc22d1 | 3676 | -1.066 | -0.2520 | Yes |
| 26 | Lifr | 3748 | -1.101 | -0.2476 | Yes |
| 27 | Actn1 | 3808 | -1.132 | -0.2402 | Yes |
| 28 | Tnfaip8 | 3897 | -1.181 | -0.2380 | Yes |
| 29 | Zbtb10 | 3989 | -1.239 | -0.2354 | Yes |
| 30 | B4galt1 | 3994 | -1.244 | -0.2145 | Yes |
| 31 | Pgm3 | 4049 | -1.283 | -0.2034 | Yes |
| 32 | Ccnd1 | 4229 | -1.443 | -0.2157 | Yes |
| 33 | Selenop | 4397 | -1.636 | -0.2221 | Yes |
| 34 | Spdef | 4411 | -1.663 | -0.1958 | Yes |
| 35 | Ptpn21 | 4430 | -1.703 | -0.1698 | Yes |
| 36 | Rab4a | 4465 | -1.760 | -0.1462 | Yes |
| 37 | Krt8 | 4476 | -1.778 | -0.1172 | Yes |
| 38 | Krt19 | 4510 | -1.837 | -0.0920 | Yes |
| 39 | Iqgap2 | 4517 | -1.846 | -0.0611 | Yes |
| 40 | Gpd1l | 4531 | -1.868 | -0.0312 | Yes |
| 41 | Tmprss2 | 4732 | -2.541 | -0.0287 | Yes |
| 42 | Homer2 | 4752 | -2.684 | 0.0142 | Yes |
Table: GSEA details [plain text format]

  

Fig 2: HALLMARK\_ANDROGEN\_RESPONSE: Random ES distribution      
 Gene set null distribution of ES for **HALLMARK\_ANDROGEN\_RESPONSE**

  
