# Supplementary material for: TMPRSS11B promotes an acidified microenvironment and immune suppression in squamous lung cancer
Source: EMBO Rep. 2025 Nov 10;26(24):6346–79. doi: 10.1038/s44319-025-00631-1 (PMC12714794; doi:10.1038/s44319-025-00631-1)
Supplement: Supplementary file 14 — Figure EV2 Source Data [file 44319_2025_631_MOESM14_ESM.zip › Figure EV2/EV2D-E/GSEA_Broad Institute_Mh_T11b-high LUSC vs LUAD/HALLMARK_ANGIOGENESIS.html]

Details for gene set HALLMARK\_ANGIOGENESIS[GSEA]

|  || Dataset | Ranked list\_DGE\_squamousT11b\_vs\_all adenosadeno\_HSE13-NT copy |
| Phenotype | NoPhenotypeAvailable |
| Upregulated in class | na\_pos |
| GeneSet | HALLMARK\_ANGIOGENESIS |
| Enrichment Score (ES) | 0.5224381 |
| Normalized Enrichment Score (NES) | 1.7222518 |
| Nominal p-value | 0.01908397 |
| FDR q-value | 0.029784435 |
| FWER p-Value | 0.256 |
Table: GSEA Results Summary

  

Fig 1: Enrichment plot: HALLMARK\_ANGIOGENESIS      
 Profile of the Running ES Score & Positions of GeneSet Members on the Rank Ordered List

  

| SYMBOL | RANK IN GENE LIST | RANK METRIC SCORE | RUNNING ES | CORE ENRICHMENT || 1 | Spp1 | 69 | 4.139 | 0.1465 | Yes |
| 2 | Cxcl5 | 101 | 3.685 | 0.2833 | Yes |
| 3 | Lpl | 149 | 3.044 | 0.3918 | Yes |
| 4 | Pglyrp1 | 345 | 1.894 | 0.4249 | Yes |
| 5 | S100a4 | 353 | 1.867 | 0.4960 | Yes |
| 6 | Jag2 | 498 | 1.452 | 0.5224 | Yes |
| 7 | Kcnj8 | 898 | 0.760 | 0.4689 | No |
| 8 | Col3a1 | 1034 | 0.619 | 0.4649 | No |
| 9 | Col5a2 | 1080 | 0.573 | 0.4778 | No |
| 10 | App | 1359 | -0.528 | 0.4405 | No |
| 11 | Fgfr1 | 1743 | -0.590 | 0.3837 | No |
| 12 | Pdgfa | 2672 | -0.763 | 0.2202 | No |
| 13 | Ptk2 | 3571 | -1.021 | 0.0730 | No |
| 14 | Vav2 | 3647 | -1.053 | 0.0983 | No |
| 15 | Tnfrsf21 | 4266 | -1.475 | 0.0270 | No |
| 16 | Ccnd2 | 4672 | -2.265 | 0.0308 | No |
Table: GSEA details [plain text format]

  

Fig 2: HALLMARK\_ANGIOGENESIS: Random ES distribution      
 Gene set null distribution of ES for **HALLMARK\_ANGIOGENESIS**

  
