# Supplementary material for: TMPRSS11B promotes an acidified microenvironment and immune suppression in squamous lung cancer
Source: EMBO Rep. 2025 Nov 10;26(24):6346–79. doi: 10.1038/s44319-025-00631-1 (PMC12714794; doi:10.1038/s44319-025-00631-1)
Supplement: Supplementary file 14 — Figure EV2 Source Data [file 44319_2025_631_MOESM14_ESM.zip › Figure EV2/EV2D-E/GSEA_Broad Institute_Mh_T11b-high LUSC vs LUAD/HALLMARK_APICAL_JUNCTION.html]

Details for gene set HALLMARK\_APICAL\_JUNCTION[GSEA]

|  || Dataset | Ranked list\_DGE\_squamousT11b\_vs\_all adenosadeno\_HSE13-NT copy |
| Phenotype | NoPhenotypeAvailable |
| Upregulated in class | na\_pos |
| GeneSet | HALLMARK\_APICAL\_JUNCTION |
| Enrichment Score (ES) | 0.3597492 |
| Normalized Enrichment Score (NES) | 1.7066001 |
| Nominal p-value | 0.006688963 |
| FDR q-value | 0.031200528 |
| FWER p-Value | 0.286 |
Table: GSEA Results Summary

  

Fig 1: Enrichment plot: HALLMARK\_APICAL\_JUNCTION      
 Profile of the Running ES Score & Positions of GeneSet Members on the Rank Ordered List

  

| SYMBOL | RANK IN GENE LIST | RANK METRIC SCORE | RUNNING ES | CORE ENRICHMENT || 1 | Mmp9 | 114 | 3.578 | 0.0183 | Yes |
| 2 | Col17a1 | 154 | 2.992 | 0.0455 | Yes |
| 3 | Cdh3 | 180 | 2.732 | 0.0725 | Yes |
| 4 | Wnk4 | 203 | 2.546 | 0.0980 | Yes |
| 5 | Cd274 | 213 | 2.481 | 0.1255 | Yes |
| 6 | Nectin1 | 233 | 2.387 | 0.1497 | Yes |
| 7 | Sirpa | 268 | 2.242 | 0.1690 | Yes |
| 8 | Cdsn | 318 | 2.017 | 0.1826 | Yes |
| 9 | Ptprc | 338 | 1.926 | 0.2014 | Yes |
| 10 | Nectin4 | 378 | 1.759 | 0.2140 | Yes |
| 11 | Pard6g | 401 | 1.680 | 0.2292 | Yes |
| 12 | Pik3cb | 409 | 1.664 | 0.2474 | Yes |
| 13 | Gnai1 | 414 | 1.654 | 0.2661 | Yes |
| 14 | Msn | 419 | 1.628 | 0.2845 | Yes |
| 15 | Fscn1 | 444 | 1.564 | 0.2980 | Yes |
| 16 | Syk | 459 | 1.531 | 0.3131 | Yes |
| 17 | Tgfbi | 502 | 1.443 | 0.3214 | Yes |
| 18 | Layn | 506 | 1.432 | 0.3377 | Yes |
| 19 | Sdc3 | 550 | 1.335 | 0.3444 | Yes |
| 20 | Jup | 565 | 1.293 | 0.3567 | Yes |
| 21 | Thbs3 | 647 | 1.095 | 0.3527 | Yes |
| 22 | Hras | 719 | 0.981 | 0.3493 | Yes |
| 23 | Cldn4 | 764 | 0.911 | 0.3509 | Yes |
| 24 | Pcdh1 | 824 | 0.840 | 0.3484 | Yes |
| 25 | Map3k20 | 873 | 0.798 | 0.3477 | Yes |
| 26 | Cldn5 | 902 | 0.758 | 0.3508 | Yes |
| 27 | Bmp1 | 903 | 0.757 | 0.3597 | Yes |
| 28 | Cd276 | 1061 | 0.599 | 0.3338 | No |
| 29 | Icam1 | 1070 | 0.586 | 0.3391 | No |
| 30 | Mpzl2 | 1071 | 0.586 | 0.3460 | No |
| 31 | Akt2 | 1349 | -0.525 | 0.2940 | No |
| 32 | Itgb1 | 1391 | -0.531 | 0.2916 | No |
| 33 | Tial1 | 1498 | -0.551 | 0.2758 | No |
| 34 | Nectin2 | 1531 | -0.558 | 0.2757 | No |
| 35 | Cd34 | 1781 | -0.597 | 0.2304 | No |
| 36 | Pbx2 | 1795 | -0.599 | 0.2348 | No |
| 37 | Lima1 | 1819 | -0.604 | 0.2371 | No |
| 38 | Shroom2 | 1911 | -0.620 | 0.2253 | No |
| 39 | Dlg1 | 1932 | -0.623 | 0.2284 | No |
| 40 | Zyx | 2334 | -0.694 | 0.1523 | No |
| 41 | Nf1 | 2379 | -0.701 | 0.1514 | No |
| 42 | Col16a1 | 2564 | -0.740 | 0.1214 | No |
| 43 | Dhx16 | 2626 | -0.753 | 0.1175 | No |
| 44 | Mmp2 | 2903 | -0.814 | 0.0691 | No |
| 45 | Itgb4 | 3162 | -0.888 | 0.0254 | No |
| 46 | Vasp | 3174 | -0.891 | 0.0336 | No |
| 47 | Inppl1 | 3179 | -0.893 | 0.0433 | No |
| 48 | Tsc1 | 3220 | -0.905 | 0.0456 | No |
| 49 | Ptk2 | 3571 | -1.021 | -0.0159 | No |
| 50 | Vav2 | 3647 | -1.053 | -0.0192 | No |
| 51 | Traf1 | 3786 | -1.119 | -0.0350 | No |
| 52 | Actn1 | 3808 | -1.132 | -0.0261 | No |
| 53 | Lamc2 | 3814 | -1.136 | -0.0137 | No |
| 54 | Itga3 | 3850 | -1.157 | -0.0074 | No |
| 55 | Cdh11 | 3879 | -1.171 | 0.0006 | No |
| 56 | Hadh | 3948 | -1.215 | 0.0007 | No |
| 57 | Sgce | 3962 | -1.222 | 0.0124 | No |
| 58 | B4galt1 | 3994 | -1.244 | 0.0206 | No |
| 59 | Mpzl1 | 4114 | -1.339 | 0.0114 | No |
| 60 | Parva | 4173 | -1.387 | 0.0156 | No |
| 61 | Cldn18 | 4346 | -1.570 | -0.0020 | No |
| 62 | Cdh1 | 4436 | -1.716 | -0.0004 | No |
| 63 | Sorbs3 | 4439 | -1.719 | 0.0195 | No |
| 64 | Lamb3 | 4582 | -1.998 | 0.0132 | No |
| 65 | Cdh6 | 4793 | -3.096 | 0.0057 | No |
Table: GSEA details [plain text format]

  

Fig 2: HALLMARK\_APICAL\_JUNCTION: Random ES distribution      
 Gene set null distribution of ES for **HALLMARK\_APICAL\_JUNCTION**

  
