# Supplementary material for: TMPRSS11B promotes an acidified microenvironment and immune suppression in squamous lung cancer
Source: EMBO Rep. 2025 Nov 10;26(24):6346–79. doi: 10.1038/s44319-025-00631-1 (PMC12714794; doi:10.1038/s44319-025-00631-1)
Supplement: Supplementary file 14 — Figure EV2 Source Data [file 44319_2025_631_MOESM14_ESM.zip › Figure EV2/EV2D-E/GSEA_Broad Institute_Mh_T11b-high LUSC vs LUAD/HALLMARK_APICAL_SURFACE.html]

Details for gene set HALLMARK\_APICAL\_SURFACE[GSEA]

|  || Dataset | Ranked list\_DGE\_squamousT11b\_vs\_all adenosadeno\_HSE13-NT copy |
| Phenotype | NoPhenotypeAvailable |
| Upregulated in class | na\_pos |
| GeneSet | HALLMARK\_APICAL\_SURFACE |
| Enrichment Score (ES) | 0.3875113 |
| Normalized Enrichment Score (NES) | 1.3902142 |
| Nominal p-value | 0.11895911 |
| FDR q-value | 0.15516514 |
| FWER p-Value | 0.925 |
Table: GSEA Results Summary

  

Fig 1: Enrichment plot: HALLMARK\_APICAL\_SURFACE      
 Profile of the Running ES Score & Positions of GeneSet Members on the Rank Ordered List

  

| SYMBOL | RANK IN GENE LIST | RANK METRIC SCORE | RUNNING ES | CORE ENRICHMENT || 1 | Lypd3 | 15 | 6.488 | 0.2086 | Yes |
| 2 | Gas1 | 249 | 2.312 | 0.2355 | Yes |
| 3 | Afap1l2 | 270 | 2.236 | 0.3043 | Yes |
| 4 | Hspb1 | 460 | 1.530 | 0.3148 | Yes |
| 5 | Crybg1 | 483 | 1.491 | 0.3589 | Yes |
| 6 | Il2rg | 648 | 1.091 | 0.3603 | Yes |
| 7 | Mal | 733 | 0.961 | 0.3742 | Yes |
| 8 | Lyn | 805 | 0.861 | 0.3875 | Yes |
| 9 | App | 1359 | -0.528 | 0.2895 | No |
| 10 | Shroom2 | 1911 | -0.620 | 0.1949 | No |
| 11 | Adam10 | 1952 | -0.627 | 0.2071 | No |
| 12 | Adipor2 | 2276 | -0.684 | 0.1621 | No |
| 13 | Ncoa6 | 2663 | -0.762 | 0.1065 | No |
| 14 | Sulf2 | 2935 | -0.822 | 0.0769 | No |
| 15 | Gstm5 | 3294 | -0.927 | 0.0326 | No |
| 16 | Efna5 | 3690 | -1.073 | -0.0147 | No |
| 17 | Crocc | 3692 | -1.075 | 0.0202 | No |
| 18 | B4galt1 | 3994 | -1.244 | -0.0020 | No |
| 19 | Atp8b1 | 4315 | -1.523 | -0.0189 | No |
| 20 | Scube1 | 4425 | -1.688 | 0.0134 | No |
| 21 | Pkhd1 | 4626 | -2.104 | 0.0404 | No |
Table: GSEA details [plain text format]

  

Fig 2: HALLMARK\_APICAL\_SURFACE: Random ES distribution      
 Gene set null distribution of ES for **HALLMARK\_APICAL\_SURFACE**

  
