# Supplementary material for: TMPRSS11B promotes an acidified microenvironment and immune suppression in squamous lung cancer
Source: EMBO Rep. 2025 Nov 10;26(24):6346–79. doi: 10.1038/s44319-025-00631-1 (PMC12714794; doi:10.1038/s44319-025-00631-1)
Supplement: Supplementary file 14 — Figure EV2 Source Data [file 44319_2025_631_MOESM14_ESM.zip › Figure EV2/EV2D-E/GSEA_Broad Institute_Mh_T11b-high LUSC vs LUAD/HALLMARK_APOPTOSIS.html]

Details for gene set HALLMARK\_APOPTOSIS[GSEA]

|  || Dataset | Ranked list\_DGE\_squamousT11b\_vs\_all adenosadeno\_HSE13-NT copy |
| Phenotype | NoPhenotypeAvailable |
| Upregulated in class | na\_pos |
| GeneSet | HALLMARK\_APOPTOSIS |
| Enrichment Score (ES) | 0.2653199 |
| Normalized Enrichment Score (NES) | 1.2498802 |
| Nominal p-value | 0.176 |
| FDR q-value | 0.28879714 |
| FWER p-Value | 0.995 |
Table: GSEA Results Summary

  

Fig 1: Enrichment plot: HALLMARK\_APOPTOSIS      
 Profile of the Running ES Score & Positions of GeneSet Members on the Rank Ordered List

  

| SYMBOL | RANK IN GENE LIST | RANK METRIC SCORE | RUNNING ES | CORE ENRICHMENT || 1 | Emp1 | 79 | 4.007 | 0.0292 | Yes |
| 2 | Il1a | 119 | 3.497 | 0.0610 | Yes |
| 3 | Hmox1 | 144 | 3.102 | 0.0915 | Yes |
| 4 | Gna15 | 161 | 2.913 | 0.1214 | Yes |
| 5 | Plat | 188 | 2.690 | 0.1467 | Yes |
| 6 | Anxa1 | 218 | 2.455 | 0.1687 | Yes |
| 7 | Il1b | 240 | 2.351 | 0.1912 | Yes |
| 8 | Cd44 | 329 | 1.976 | 0.1953 | Yes |
| 9 | Tnf | 391 | 1.722 | 0.2021 | Yes |
| 10 | Lgals3 | 447 | 1.559 | 0.2084 | Yes |
| 11 | Hspb1 | 460 | 1.530 | 0.2234 | Yes |
| 12 | Irf1 | 508 | 1.429 | 0.2298 | Yes |
| 13 | Rnasel | 524 | 1.385 | 0.2425 | Yes |
| 14 | Timp2 | 607 | 1.189 | 0.2389 | Yes |
| 15 | Sat1 | 614 | 1.180 | 0.2511 | Yes |
| 16 | Atf3 | 643 | 1.106 | 0.2579 | Yes |
| 17 | Tgfb2 | 720 | 0.981 | 0.2531 | Yes |
| 18 | Birc3 | 745 | 0.942 | 0.2588 | Yes |
| 19 | Gadd45b | 804 | 0.862 | 0.2565 | Yes |
| 20 | Cdkn1a | 861 | 0.808 | 0.2539 | Yes |
| 21 | Mgmt | 922 | 0.739 | 0.2498 | Yes |
| 22 | Cflar | 963 | 0.693 | 0.2493 | Yes |
| 23 | Ier3 | 967 | 0.691 | 0.2565 | Yes |
| 24 | Mcl1 | 969 | 0.689 | 0.2642 | Yes |
| 25 | Casp1 | 1000 | 0.649 | 0.2653 | Yes |
| 26 | Ddit3 | 1038 | 0.616 | 0.2646 | No |
| 27 | Sod2 | 1120 | 0.540 | 0.2537 | No |
| 28 | Ifngr1 | 1155 | 0.509 | 0.2524 | No |
| 29 | Casp3 | 1261 | -0.514 | 0.2362 | No |
| 30 | Erbb3 | 1278 | -0.516 | 0.2387 | No |
| 31 | F2r | 1319 | -0.521 | 0.2362 | No |
| 32 | App | 1359 | -0.528 | 0.2341 | No |
| 33 | Timp3 | 1480 | -0.548 | 0.2151 | No |
| 34 | Bcap31 | 1622 | -0.572 | 0.1920 | No |
| 35 | Bgn | 1714 | -0.584 | 0.1795 | No |
| 36 | Madd | 1824 | -0.604 | 0.1635 | No |
| 37 | Crebbp | 1870 | -0.614 | 0.1610 | No |
| 38 | Ppp2r5b | 1910 | -0.620 | 0.1599 | No |
| 39 | Dnaja1 | 1926 | -0.622 | 0.1639 | No |
| 40 | Rock1 | 1986 | -0.633 | 0.1587 | No |
| 41 | Pak1 | 1990 | -0.634 | 0.1653 | No |
| 42 | Slc20a1 | 2409 | -0.707 | 0.0854 | No |
| 43 | Smad7 | 2594 | -0.746 | 0.0553 | No |
| 44 | Sod1 | 2688 | -0.765 | 0.0444 | No |
| 45 | Mmp2 | 2903 | -0.814 | 0.0087 | No |
| 46 | Fdxr | 2916 | -0.817 | 0.0155 | No |
| 47 | Isg20 | 3115 | -0.875 | -0.0161 | No |
| 48 | Tgfbr3 | 3149 | -0.885 | -0.0129 | No |
| 49 | Erbb2 | 3230 | -0.909 | -0.0194 | No |
| 50 | Casp6 | 3257 | -0.917 | -0.0144 | No |
| 51 | Casp2 | 3441 | -0.978 | -0.0417 | No |
| 52 | Rara | 3514 | -1.003 | -0.0454 | No |
| 53 | Sc5d | 3560 | -1.017 | -0.0432 | No |
| 54 | Ptk2 | 3571 | -1.021 | -0.0336 | No |
| 55 | Pdcd4 | 3741 | -1.099 | -0.0566 | No |
| 56 | Dnajc3 | 3895 | -1.181 | -0.0753 | No |
| 57 | Dcn | 3919 | -1.196 | -0.0664 | No |
| 58 | Bmf | 3943 | -1.211 | -0.0574 | No |
| 59 | Tnfrsf12a | 4008 | -1.254 | -0.0566 | No |
| 60 | Btg2 | 4032 | -1.269 | -0.0469 | No |
| 61 | Bmp2 | 4098 | -1.322 | -0.0454 | No |
| 62 | Ccnd1 | 4229 | -1.443 | -0.0563 | No |
| 63 | Nedd9 | 4238 | -1.453 | -0.0413 | No |
| 64 | Gstm2 | 4466 | -1.761 | -0.0690 | No |
| 65 | Txnip | 4471 | -1.771 | -0.0495 | No |
| 66 | Ccnd2 | 4672 | -2.265 | -0.0657 | No |
| 67 | Ereg | 4731 | -2.541 | -0.0489 | No |
| 68 | Clu | 4775 | -2.816 | -0.0257 | No |
| 69 | Il18 | 4789 | -3.056 | 0.0065 | No |
Table: GSEA details [plain text format]

  

Fig 2: HALLMARK\_APOPTOSIS: Random ES distribution      
 Gene set null distribution of ES for **HALLMARK\_APOPTOSIS**

  
