# Supplementary material for: TMPRSS11B promotes an acidified microenvironment and immune suppression in squamous lung cancer
Source: EMBO Rep. 2025 Nov 10;26(24):6346–79. doi: 10.1038/s44319-025-00631-1 (PMC12714794; doi:10.1038/s44319-025-00631-1)
Supplement: Supplementary file 14 — Figure EV2 Source Data [file 44319_2025_631_MOESM14_ESM.zip › Figure EV2/EV2D-E/GSEA_Broad Institute_Mh_T11b-high LUSC vs LUAD/HALLMARK_BILE_ACID_METABOLISM.html]

Details for gene set HALLMARK\_BILE\_ACID\_METABOLISM[GSEA]

|  || Dataset | Ranked list\_DGE\_squamousT11b\_vs\_all adenosadeno\_HSE13-NT copy |
| Phenotype | NoPhenotypeAvailable |
| Upregulated in class | na\_neg |
| GeneSet | HALLMARK\_BILE\_ACID\_METABOLISM |
| Enrichment Score (ES) | -0.22577034 |
| Normalized Enrichment Score (NES) | -1.0445224 |
| Nominal p-value | 0.39635536 |
| FDR q-value | 1.0 |
| FWER p-Value | 1.0 |
Table: GSEA Results Summary

  

Fig 1: Enrichment plot: HALLMARK\_BILE\_ACID\_METABOLISM      
 Profile of the Running ES Score & Positions of GeneSet Members on the Rank Ordered List

  

| SYMBOL | RANK IN GENE LIST | RANK METRIC SCORE | RUNNING ES | CORE ENRICHMENT || 1 | Abca1 | 172 | 2.809 | 0.0208 | No |
| 2 | Cyp27a1 | 239 | 2.356 | 0.0547 | No |
| 3 | Sult2b1 | 282 | 2.172 | 0.0898 | No |
| 4 | Fads2 | 462 | 1.527 | 0.0832 | No |
| 5 | Fads1 | 568 | 1.289 | 0.0873 | No |
| 6 | Prdx5 | 601 | 1.198 | 0.1049 | No |
| 7 | Cat | 712 | 0.989 | 0.1019 | No |
| 8 | Rbp1 | 1091 | 0.565 | 0.0342 | No |
| 9 | Abca2 | 1364 | -0.528 | -0.0120 | No |
| 10 | Pex19 | 1383 | -0.530 | -0.0051 | No |
| 11 | Hsd3b7 | 2050 | -0.644 | -0.1314 | No |
| 12 | Atxn1 | 2207 | -0.672 | -0.1504 | No |
| 13 | Nedd4 | 2231 | -0.677 | -0.1416 | No |
| 14 | Lonp2 | 2359 | -0.699 | -0.1540 | No |
| 15 | Pex13 | 2371 | -0.700 | -0.1421 | No |
| 16 | Pfkm | 2427 | -0.712 | -0.1392 | No |
| 17 | Idh2 | 2529 | -0.733 | -0.1455 | No |
| 18 | Sod1 | 2688 | -0.765 | -0.1631 | No |
| 19 | Cyp39a1 | 2734 | -0.775 | -0.1569 | No |
| 20 | Gstk1 | 2827 | -0.797 | -0.1600 | No |
| 21 | Fdxr | 2916 | -0.817 | -0.1619 | No |
| 22 | Ephx2 | 2986 | -0.838 | -0.1594 | No |
| 23 | Idh1 | 3183 | -0.894 | -0.1823 | No |
| 24 | Hsd17b4 | 3299 | -0.930 | -0.1876 | No |
| 25 | Acsl1 | 3337 | -0.941 | -0.1763 | No |
| 26 | Pex26 | 3486 | -0.992 | -0.1872 | No |
| 27 | Soat2 | 3567 | -1.020 | -0.1833 | No |
| 28 | Pex7 | 3606 | -1.035 | -0.1703 | No |
| 29 | Optn | 3759 | -1.106 | -0.1797 | No |
| 30 | Hsd17b11 | 3980 | -1.233 | -0.2008 | Yes |
| 31 | Abcd3 | 4034 | -1.270 | -0.1862 | Yes |
| 32 | Pxmp2 | 4099 | -1.324 | -0.1729 | Yes |
| 33 | Mlycd | 4100 | -1.324 | -0.1461 | Yes |
| 34 | Amacr | 4274 | -1.482 | -0.1523 | Yes |
| 35 | Slc22a18 | 4312 | -1.515 | -0.1294 | Yes |
| 36 | Bcar3 | 4336 | -1.548 | -0.1029 | Yes |
| 37 | Nudt12 | 4434 | -1.708 | -0.0887 | Yes |
| 38 | Phyh | 4469 | -1.768 | -0.0601 | Yes |
| 39 | Abca5 | 4609 | -2.066 | -0.0474 | Yes |
| 40 | Sult1b1 | 4616 | -2.083 | -0.0065 | Yes |
| 41 | Tfcp2l1 | 4708 | -2.421 | 0.0234 | Yes |
Table: GSEA details [plain text format]

  

Fig 2: HALLMARK\_BILE\_ACID\_METABOLISM: Random ES distribution      
 Gene set null distribution of ES for **HALLMARK\_BILE\_ACID\_METABOLISM**

  
