# Supplementary material for: TMPRSS11B promotes an acidified microenvironment and immune suppression in squamous lung cancer
Source: EMBO Rep. 2025 Nov 10;26(24):6346–79. doi: 10.1038/s44319-025-00631-1 (PMC12714794; doi:10.1038/s44319-025-00631-1)
Supplement: Supplementary file 14 — Figure EV2 Source Data [file 44319_2025_631_MOESM14_ESM.zip › Figure EV2/EV2D-E/GSEA_Broad Institute_Mh_T11b-high LUSC vs LUAD/HALLMARK_COAGULATION.html]

Details for gene set HALLMARK\_COAGULATION[GSEA]

|  || Dataset | Ranked list\_DGE\_squamousT11b\_vs\_all adenosadeno\_HSE13-NT copy |
| Phenotype | NoPhenotypeAvailable |
| Upregulated in class | na\_pos |
| GeneSet | HALLMARK\_COAGULATION |
| Enrichment Score (ES) | 0.41496757 |
| Normalized Enrichment Score (NES) | 1.7532138 |
| Nominal p-value | 0.012280702 |
| FDR q-value | 0.025054036 |
| FWER p-Value | 0.206 |
Table: GSEA Results Summary

  

Fig 1: Enrichment plot: HALLMARK\_COAGULATION      
 Profile of the Running ES Score & Positions of GeneSet Members on the Rank Ordered List

  

| SYMBOL | RANK IN GENE LIST | RANK METRIC SCORE | RUNNING ES | CORE ENRICHMENT || 1 | Ctsl | 76 | 4.064 | 0.0398 | Yes |
| 2 | Serpinb2 | 89 | 3.864 | 0.0903 | Yes |
| 3 | Ctsk | 111 | 3.616 | 0.1354 | Yes |
| 4 | Mmp9 | 114 | 3.578 | 0.1840 | Yes |
| 5 | Htra1 | 183 | 2.728 | 0.2072 | Yes |
| 6 | Plat | 188 | 2.690 | 0.2432 | Yes |
| 7 | Plau | 210 | 2.513 | 0.2733 | Yes |
| 8 | Anxa1 | 218 | 2.455 | 0.3055 | Yes |
| 9 | Plek | 252 | 2.303 | 0.3301 | Yes |
| 10 | Ctsb | 288 | 2.139 | 0.3521 | Yes |
| 11 | Dusp14 | 324 | 1.988 | 0.3720 | Yes |
| 12 | Apoc1 | 386 | 1.739 | 0.3831 | Yes |
| 13 | Lgmn | 458 | 1.534 | 0.3893 | Yes |
| 14 | C3 | 474 | 1.502 | 0.4067 | Yes |
| 15 | Sh2b2 | 526 | 1.382 | 0.4150 | Yes |
| 16 | Trf | 682 | 1.033 | 0.3967 | No |
| 17 | C1qa | 710 | 0.990 | 0.4046 | No |
| 18 | Bmp1 | 903 | 0.757 | 0.3748 | No |
| 19 | Maff | 1047 | 0.610 | 0.3532 | No |
| 20 | Serpine1 | 1049 | 0.610 | 0.3614 | No |
| 21 | Cfh | 1075 | 0.578 | 0.3641 | No |
| 22 | Timp3 | 1480 | -0.548 | 0.2870 | No |
| 23 | Ctso | 1501 | -0.552 | 0.2904 | No |
| 24 | F3 | 1925 | -0.622 | 0.2104 | No |
| 25 | Pdgfb | 2026 | -0.640 | 0.1982 | No |
| 26 | Pros1 | 2083 | -0.650 | 0.1954 | No |
| 27 | Gnb2 | 2210 | -0.673 | 0.1782 | No |
| 28 | Pef1 | 2385 | -0.703 | 0.1514 | No |
| 29 | S100a13 | 2519 | -0.731 | 0.1336 | No |
| 30 | Mmp2 | 2903 | -0.814 | 0.0646 | No |
| 31 | S100a1 | 3077 | -0.864 | 0.0402 | No |
| 32 | Capn5 | 3263 | -0.919 | 0.0141 | No |
| 33 | Thbs1 | 3344 | -0.945 | 0.0103 | No |
| 34 | Klf7 | 3489 | -0.993 | -0.0062 | No |
| 35 | Cfb | 3801 | -1.129 | -0.0559 | No |
| 36 | Cpq | 4054 | -1.285 | -0.0910 | No |
| 37 | Mmp15 | 4353 | -1.579 | -0.1318 | No |
| 38 | Prss23 | 4568 | -1.964 | -0.1496 | No |
| 39 | Cfi | 4653 | -2.192 | -0.1372 | No |
| 40 | Hpn | 4685 | -2.329 | -0.1117 | No |
| 41 | Hnf4a | 4686 | -2.337 | -0.0797 | No |
| 42 | Hmgcs2 | 4714 | -2.435 | -0.0520 | No |
| 43 | Ctse | 4736 | -2.565 | -0.0212 | No |
| 44 | Clu | 4775 | -2.816 | 0.0094 | No |
Table: GSEA details [plain text format]

  

Fig 2: HALLMARK\_COAGULATION: Random ES distribution      
 Gene set null distribution of ES for **HALLMARK\_COAGULATION**

  
