# Supplementary material for: TMPRSS11B promotes an acidified microenvironment and immune suppression in squamous lung cancer
Source: EMBO Rep. 2025 Nov 10;26(24):6346–79. doi: 10.1038/s44319-025-00631-1 (PMC12714794; doi:10.1038/s44319-025-00631-1)
Supplement: Supplementary file 14 — Figure EV2 Source Data [file 44319_2025_631_MOESM14_ESM.zip › Figure EV2/EV2D-E/GSEA_Broad Institute_Mh_T11b-high LUSC vs LUAD/HALLMARK_COMPLEMENT.html]

Details for gene set HALLMARK\_COMPLEMENT[GSEA]

|  || Dataset | Ranked list\_DGE\_squamousT11b\_vs\_all adenosadeno\_HSE13-NT copy |
| Phenotype | NoPhenotypeAvailable |
| Upregulated in class | na\_pos |
| GeneSet | HALLMARK\_COMPLEMENT |
| Enrichment Score (ES) | 0.516629 |
| Normalized Enrichment Score (NES) | 2.4542263 |
| Nominal p-value | 0.0 |
| FDR q-value | 0.0 |
| FWER p-Value | 0.0 |
Table: GSEA Results Summary

  

Fig 1: Enrichment plot: HALLMARK\_COMPLEMENT      
 Profile of the Running ES Score & Positions of GeneSet Members on the Rank Ordered List

  

| SYMBOL | RANK IN GENE LIST | RANK METRIC SCORE | RUNNING ES | CORE ENRICHMENT || 1 | Mmp12 | 63 | 4.267 | 0.0273 | Yes |
| 2 | Ctsl | 76 | 4.064 | 0.0634 | Yes |
| 3 | Serpinb2 | 89 | 3.864 | 0.0976 | Yes |
| 4 | S100a9 | 110 | 3.624 | 0.1278 | Yes |
| 5 | Itgam | 132 | 3.250 | 0.1543 | Yes |
| 6 | Plat | 188 | 2.690 | 0.1683 | Yes |
| 7 | Pla2g7 | 195 | 2.599 | 0.1918 | Yes |
| 8 | Cd36 | 199 | 2.567 | 0.2155 | Yes |
| 9 | Ctss | 247 | 2.317 | 0.2277 | Yes |
| 10 | Plek | 252 | 2.303 | 0.2487 | Yes |
| 11 | Ltf | 264 | 2.250 | 0.2678 | Yes |
| 12 | Fcer1g | 272 | 2.235 | 0.2875 | Yes |
| 13 | Gngt2 | 273 | 2.235 | 0.3088 | Yes |
| 14 | Ctsd | 274 | 2.219 | 0.3299 | Yes |
| 15 | Dusp5 | 275 | 2.206 | 0.3509 | Yes |
| 16 | F7 | 279 | 2.188 | 0.3710 | Yes |
| 17 | Ctsb | 288 | 2.139 | 0.3897 | Yes |
| 18 | Pim1 | 319 | 2.011 | 0.4025 | Yes |
| 19 | Was | 373 | 1.768 | 0.4081 | Yes |
| 20 | Apoc1 | 386 | 1.739 | 0.4221 | Yes |
| 21 | Lcp2 | 403 | 1.676 | 0.4347 | Yes |
| 22 | Cebpb | 425 | 1.620 | 0.4457 | Yes |
| 23 | Lgals3 | 447 | 1.559 | 0.4561 | Yes |
| 24 | Lgmn | 458 | 1.534 | 0.4685 | Yes |
| 25 | Lipa | 473 | 1.504 | 0.4799 | Yes |
| 26 | C3 | 474 | 1.502 | 0.4942 | Yes |
| 27 | Irf1 | 508 | 1.429 | 0.5008 | Yes |
| 28 | Timp2 | 607 | 1.189 | 0.4915 | Yes |
| 29 | Irf7 | 617 | 1.174 | 0.5007 | Yes |
| 30 | Ehd1 | 637 | 1.118 | 0.5074 | Yes |
| 31 | C1qa | 710 | 0.990 | 0.5016 | Yes |
| 32 | Notch4 | 755 | 0.926 | 0.5011 | Yes |
| 33 | Ctsc | 770 | 0.907 | 0.5068 | Yes |
| 34 | C1qc | 775 | 0.902 | 0.5145 | Yes |
| 35 | Lyn | 805 | 0.861 | 0.5166 | Yes |
| 36 | Rhog | 961 | 0.693 | 0.4906 | No |
| 37 | Msrb1 | 982 | 0.669 | 0.4927 | No |
| 38 | Atox1 | 997 | 0.651 | 0.4960 | No |
| 39 | Casp1 | 1000 | 0.649 | 0.5017 | No |
| 40 | Maff | 1047 | 0.610 | 0.4978 | No |
| 41 | Serpine1 | 1049 | 0.610 | 0.5034 | No |
| 42 | Cfh | 1075 | 0.578 | 0.5037 | No |
| 43 | Fdx1 | 1106 | 0.548 | 0.5026 | No |
| 44 | Car2 | 1171 | -0.500 | 0.4938 | No |
| 45 | Casp3 | 1261 | -0.514 | 0.4800 | No |
| 46 | Ctso | 1501 | -0.552 | 0.4349 | No |
| 47 | Usp8 | 1709 | -0.583 | 0.3969 | No |
| 48 | Brpf3 | 1759 | -0.593 | 0.3922 | No |
| 49 | Calm1 | 1839 | -0.607 | 0.3814 | No |
| 50 | F3 | 1925 | -0.622 | 0.3694 | No |
| 51 | Pdgfb | 2026 | -0.640 | 0.3544 | No |
| 52 | Gpd2 | 2056 | -0.645 | 0.3544 | No |
| 53 | Rnf4 | 2131 | -0.660 | 0.3451 | No |
| 54 | Kif2a | 2174 | -0.665 | 0.3426 | No |
| 55 | Gnb2 | 2210 | -0.673 | 0.3416 | No |
| 56 | Rce1 | 2248 | -0.680 | 0.3403 | No |
| 57 | S100a13 | 2519 | -0.731 | 0.2904 | No |
| 58 | Usp16 | 2546 | -0.735 | 0.2919 | No |
| 59 | Pla2g4a | 2549 | -0.736 | 0.2985 | No |
| 60 | Calm3 | 2555 | -0.737 | 0.3045 | No |
| 61 | Jak2 | 2891 | -0.813 | 0.2416 | No |
| 62 | Dyrk2 | 3562 | -1.017 | 0.1103 | No |
| 63 | Cfb | 3801 | -1.129 | 0.0709 | No |
| 64 | Cpq | 4054 | -1.285 | 0.0301 | No |
| 65 | Lap3 | 4064 | -1.292 | 0.0404 | No |
| 66 | Mmp15 | 4353 | -1.579 | -0.0052 | No |
| 67 | F5 | 4395 | -1.635 | 0.0017 | No |
| 68 | Gca | 4505 | -1.825 | -0.0039 | No |
| 69 | Cdh13 | 4647 | -2.165 | -0.0130 | No |
| 70 | Hnf4a | 4686 | -2.337 | 0.0012 | No |
| 71 | Clu | 4775 | -2.816 | 0.0095 | No |
Table: GSEA details [plain text format]

  

Fig 2: HALLMARK\_COMPLEMENT: Random ES distribution      
 Gene set null distribution of ES for **HALLMARK\_COMPLEMENT**

  
