# Supplementary material for: TMPRSS11B promotes an acidified microenvironment and immune suppression in squamous lung cancer
Source: EMBO Rep. 2025 Nov 10;26(24):6346–79. doi: 10.1038/s44319-025-00631-1 (PMC12714794; doi:10.1038/s44319-025-00631-1)
Supplement: Supplementary file 14 — Figure EV2 Source Data [file 44319_2025_631_MOESM14_ESM.zip › Figure EV2/EV2D-E/GSEA_Broad Institute_Mh_T11b-high LUSC vs LUAD/HALLMARK_DNA_REPAIR.html]

Details for gene set HALLMARK\_DNA\_REPAIR[GSEA]

|  || Dataset | Ranked list\_DGE\_squamousT11b\_vs\_all adenosadeno\_HSE13-NT copy |
| Phenotype | NoPhenotypeAvailable |
| Upregulated in class | na\_neg |
| GeneSet | HALLMARK\_DNA\_REPAIR |
| Enrichment Score (ES) | -0.13840617 |
| Normalized Enrichment Score (NES) | -0.6752001 |
| Nominal p-value | 0.9100529 |
| FDR q-value | 1.0 |
| FWER p-Value | 1.0 |
Table: GSEA Results Summary

  

Fig 1: Enrichment plot: HALLMARK\_DNA\_REPAIR      
 Profile of the Running ES Score & Positions of GeneSet Members on the Rank Ordered List

  

| SYMBOL | RANK IN GENE LIST | RANK METRIC SCORE | RUNNING ES | CORE ENRICHMENT || 1 | Ada | 171 | 2.823 | 0.0266 | No |
| 2 | Hcls1 | 487 | 1.480 | -0.0067 | No |
| 3 | Dut | 1068 | 0.589 | -0.1153 | No |
| 4 | Pnp | 1110 | 0.546 | -0.1118 | No |
| 5 | Sdcbp | 1122 | 0.536 | -0.1022 | No |
| 6 | Rnmt | 1198 | -0.505 | -0.1068 | No |
| 7 | Gmpr2 | 1269 | -0.515 | -0.1101 | No |
| 8 | Vps37b | 1274 | -0.516 | -0.0995 | No |
| 9 | Ak3 | 1352 | -0.526 | -0.1040 | No |
| 10 | Dgcr8 | 1477 | -0.548 | -0.1179 | No |
| 11 | Polr2h | 1576 | -0.563 | -0.1259 | Yes |
| 12 | Bcap31 | 1622 | -0.572 | -0.1227 | Yes |
| 13 | Zwint | 1639 | -0.574 | -0.1134 | Yes |
| 14 | Taf10 | 1704 | -0.583 | -0.1139 | Yes |
| 15 | Brf2 | 1763 | -0.593 | -0.1129 | Yes |
| 16 | Aprt | 1790 | -0.598 | -0.1051 | Yes |
| 17 | Sf3a3 | 1934 | -0.624 | -0.1213 | Yes |
| 18 | Smad5 | 1945 | -0.625 | -0.1095 | Yes |
| 19 | Polr1c | 1959 | -0.629 | -0.0983 | Yes |
| 20 | Nelfcd | 2002 | -0.637 | -0.0931 | Yes |
| 21 | Polr2k | 2071 | -0.648 | -0.0930 | Yes |
| 22 | Gtf3c5 | 2090 | -0.652 | -0.0823 | Yes |
| 23 | Dguok | 2100 | -0.654 | -0.0697 | Yes |
| 24 | Polr2e | 2361 | -0.699 | -0.1088 | Yes |
| 25 | Xpc | 2432 | -0.713 | -0.1077 | Yes |
| 26 | Eif1b | 2510 | -0.729 | -0.1077 | Yes |
| 27 | Polr2c | 2515 | -0.730 | -0.0923 | Yes |
| 28 | Guk1 | 2516 | -0.730 | -0.0762 | Yes |
| 29 | Nt5c | 2556 | -0.737 | -0.0680 | Yes |
| 30 | Nelfb | 2576 | -0.743 | -0.0555 | Yes |
| 31 | Poll | 2648 | -0.759 | -0.0536 | Yes |
| 32 | Polr2i | 2682 | -0.764 | -0.0436 | Yes |
| 33 | Polr3gl | 2763 | -0.783 | -0.0431 | Yes |
| 34 | Pom121 | 2861 | -0.805 | -0.0456 | Yes |
| 35 | Nfx1 | 2926 | -0.819 | -0.0409 | Yes |
| 36 | Tk2 | 2933 | -0.821 | -0.0240 | Yes |
| 37 | Rpa3 | 2978 | -0.836 | -0.0147 | Yes |
| 38 | Mpg | 3068 | -0.859 | -0.0143 | Yes |
| 39 | Snapc4 | 3119 | -0.877 | -0.0054 | Yes |
| 40 | Ercc2 | 3163 | -0.889 | 0.0052 | Yes |
| 41 | Ddb2 | 3225 | -0.907 | 0.0125 | Yes |
| 42 | Surf1 | 3279 | -0.924 | 0.0219 | Yes |
| 43 | Upf3b | 3315 | -0.936 | 0.0352 | Yes |
| 44 | Cant1 | 3316 | -0.936 | 0.0560 | Yes |
| 45 | Bcam | 3563 | -1.018 | 0.0269 | Yes |
| 46 | Nt5c3 | 3583 | -1.026 | 0.0456 | Yes |
| 47 | Rad52 | 3714 | -1.085 | 0.0424 | Yes |
| 48 | Cox17 | 3749 | -1.101 | 0.0597 | Yes |
| 49 | Gsdme | 4065 | -1.295 | 0.0223 | Yes |
| 50 | Gtf2a2 | 4156 | -1.374 | 0.0338 | Yes |
| 51 | Sac3d1 | 4161 | -1.380 | 0.0635 | Yes |
| 52 | Adcy6 | 4381 | -1.614 | 0.0533 | Yes |
| 53 | Umps | 4447 | -1.741 | 0.0782 | Yes |
Table: GSEA details [plain text format]

  

Fig 2: HALLMARK\_DNA\_REPAIR: Random ES distribution      
 Gene set null distribution of ES for **HALLMARK\_DNA\_REPAIR**

  
