# Supplementary material for: TMPRSS11B promotes an acidified microenvironment and immune suppression in squamous lung cancer
Source: EMBO Rep. 2025 Nov 10;26(24):6346–79. doi: 10.1038/s44319-025-00631-1 (PMC12714794; doi:10.1038/s44319-025-00631-1)
Supplement: Supplementary file 14 — Figure EV2 Source Data [file 44319_2025_631_MOESM14_ESM.zip › Figure EV2/EV2D-E/GSEA_Broad Institute_Mh_T11b-high LUSC vs LUAD/HALLMARK_EPITHELIAL_MESENCHYMAL_TRANSITION.html]

Details for gene set HALLMARK\_EPITHELIAL\_MESENCHYMAL\_TRANSITION[GSEA]

|  || Dataset | Ranked list\_DGE\_squamousT11b\_vs\_all adenosadeno\_HSE13-NT copy |
| Phenotype | NoPhenotypeAvailable |
| Upregulated in class | na\_pos |
| GeneSet | HALLMARK\_EPITHELIAL\_MESENCHYMAL\_TRANSITION |
| Enrichment Score (ES) | 0.4242282 |
| Normalized Enrichment Score (NES) | 2.0280616 |
| Nominal p-value | 0.0033112583 |
| FDR q-value | 0.0024903852 |
| FWER p-Value | 0.012 |
Table: GSEA Results Summary

  

Fig 1: Enrichment plot: HALLMARK\_EPITHELIAL\_MESENCHYMAL\_TRANSITION      
 Profile of the Running ES Score & Positions of GeneSet Members on the Rank Ordered List

  

| SYMBOL | RANK IN GENE LIST | RANK METRIC SCORE | RUNNING ES | CORE ENRICHMENT || 1 | Ecm1 | 27 | 5.794 | 0.0496 | Yes |
| 2 | Spp1 | 69 | 4.139 | 0.0804 | Yes |
| 3 | Cxcl5 | 101 | 3.685 | 0.1090 | Yes |
| 4 | Gja1 | 151 | 3.006 | 0.1273 | Yes |
| 5 | Pthlh | 152 | 3.000 | 0.1559 | Yes |
| 6 | Htra1 | 183 | 2.728 | 0.1756 | Yes |
| 7 | Tnc | 191 | 2.672 | 0.1996 | Yes |
| 8 | Gas1 | 249 | 2.312 | 0.2097 | Yes |
| 9 | Vim | 302 | 2.105 | 0.2188 | Yes |
| 10 | Sntb1 | 307 | 2.065 | 0.2376 | Yes |
| 11 | Tpm2 | 308 | 2.054 | 0.2572 | Yes |
| 12 | Aplp1 | 314 | 2.028 | 0.2755 | Yes |
| 13 | Matn2 | 316 | 2.022 | 0.2945 | Yes |
| 14 | Cxcl15 | 326 | 1.980 | 0.3115 | Yes |
| 15 | Cd44 | 329 | 1.976 | 0.3299 | Yes |
| 16 | Lgals1 | 400 | 1.684 | 0.3313 | Yes |
| 17 | Igfbp2 | 408 | 1.668 | 0.3457 | Yes |
| 18 | Dst | 434 | 1.596 | 0.3556 | Yes |
| 19 | Itga5 | 446 | 1.560 | 0.3682 | Yes |
| 20 | Tgfbi | 502 | 1.443 | 0.3704 | Yes |
| 21 | Pmp22 | 517 | 1.400 | 0.3808 | Yes |
| 22 | Emp3 | 537 | 1.365 | 0.3898 | Yes |
| 23 | Capg | 574 | 1.263 | 0.3942 | Yes |
| 24 | Sat1 | 614 | 1.180 | 0.3973 | Yes |
| 25 | Gpc1 | 623 | 1.167 | 0.4067 | Yes |
| 26 | Cadm1 | 633 | 1.126 | 0.4155 | Yes |
| 27 | Igfbp4 | 680 | 1.034 | 0.4157 | Yes |
| 28 | Glipr1 | 687 | 1.026 | 0.4242 | Yes |
| 29 | Gadd45b | 804 | 0.862 | 0.4080 | No |
| 30 | Bmp1 | 903 | 0.757 | 0.3946 | No |
| 31 | Flna | 905 | 0.755 | 0.4016 | No |
| 32 | Tgfb1 | 940 | 0.718 | 0.4013 | No |
| 33 | Col4a1 | 975 | 0.682 | 0.4006 | No |
| 34 | Dab2 | 1031 | 0.623 | 0.3950 | No |
| 35 | Col3a1 | 1034 | 0.619 | 0.4004 | No |
| 36 | Slc6a8 | 1042 | 0.611 | 0.4048 | No |
| 37 | Serpine1 | 1049 | 0.610 | 0.4093 | No |
| 38 | Col5a2 | 1080 | 0.573 | 0.4085 | No |
| 39 | Anpep | 1162 | 0.504 | 0.3962 | No |
| 40 | Tpm1 | 1262 | -0.514 | 0.3803 | No |
| 41 | Itgb5 | 1316 | -0.520 | 0.3741 | No |
| 42 | Itgb1 | 1391 | -0.531 | 0.3635 | No |
| 43 | Timp3 | 1480 | -0.548 | 0.3502 | No |
| 44 | Slit3 | 1568 | -0.563 | 0.3373 | No |
| 45 | Bgn | 1714 | -0.584 | 0.3123 | No |
| 46 | Fuca1 | 1814 | -0.603 | 0.2972 | No |
| 47 | Pdlim4 | 1851 | -0.610 | 0.2954 | No |
| 48 | Pfn2 | 2014 | -0.638 | 0.2674 | No |
| 49 | Col6a3 | 2117 | -0.657 | 0.2522 | No |
| 50 | Sdc4 | 2418 | -0.710 | 0.1957 | No |
| 51 | Pvr | 2520 | -0.731 | 0.1814 | No |
| 52 | Col16a1 | 2564 | -0.740 | 0.1794 | No |
| 53 | Lamc1 | 2789 | -0.788 | 0.1397 | No |
| 54 | Mmp2 | 2903 | -0.814 | 0.1237 | No |
| 55 | Fgf2 | 2942 | -0.823 | 0.1235 | No |
| 56 | Tgm2 | 2996 | -0.839 | 0.1204 | No |
| 57 | Tgfbr3 | 3149 | -0.885 | 0.0968 | No |
| 58 | Thbs1 | 3344 | -0.945 | 0.0649 | No |
| 59 | Pcolce | 3494 | -0.995 | 0.0430 | No |
| 60 | Nt5e | 3535 | -1.010 | 0.0442 | No |
| 61 | Eln | 3761 | -1.107 | 0.0074 | No |
| 62 | Lamc2 | 3814 | -1.136 | 0.0073 | No |
| 63 | Ccn2 | 3829 | -1.143 | 0.0152 | No |
| 64 | Cdh11 | 3879 | -1.171 | 0.0161 | No |
| 65 | Dcn | 3919 | -1.196 | 0.0192 | No |
| 66 | Serpine2 | 3936 | -1.205 | 0.0274 | No |
| 67 | Qsox1 | 3954 | -1.217 | 0.0354 | No |
| 68 | Tnfrsf12a | 4008 | -1.254 | 0.0362 | No |
| 69 | Mgp | 4278 | -1.484 | -0.0063 | No |
| 70 | Basp1 | 4338 | -1.550 | -0.0040 | No |
| 71 | Wnt5a | 4348 | -1.574 | 0.0091 | No |
| 72 | Areg | 4558 | -1.937 | -0.0164 | No |
| 73 | Cdh6 | 4793 | -3.096 | -0.0362 | No |
| 74 | Sgcd | 4820 | -4.373 | -0.0000 | No |
Table: GSEA details [plain text format]

  

Fig 2: HALLMARK\_EPITHELIAL\_MESENCHYMAL\_TRANSITION: Random ES distribution      
 Gene set null distribution of ES for **HALLMARK\_EPITHELIAL\_MESENCHYMAL\_TRANSITION**

  
