# Supplementary material for: TMPRSS11B promotes an acidified microenvironment and immune suppression in squamous lung cancer
Source: EMBO Rep. 2025 Nov 10;26(24):6346–79. doi: 10.1038/s44319-025-00631-1 (PMC12714794; doi:10.1038/s44319-025-00631-1)
Supplement: Supplementary file 14 — Figure EV2 Source Data [file 44319_2025_631_MOESM14_ESM.zip › Figure EV2/EV2D-E/GSEA_Broad Institute_Mh_T11b-high LUSC vs LUAD/HALLMARK_ESTROGEN_RESPONSE_EARLY.html]

Details for gene set HALLMARK\_ESTROGEN\_RESPONSE\_EARLY[GSEA]

|  || Dataset | Ranked list\_DGE\_squamousT11b\_vs\_all adenosadeno\_HSE13-NT copy |
| Phenotype | NoPhenotypeAvailable |
| Upregulated in class | na\_pos |
| GeneSet | HALLMARK\_ESTROGEN\_RESPONSE\_EARLY |
| Enrichment Score (ES) | 0.31283772 |
| Normalized Enrichment Score (NES) | 1.5948567 |
| Nominal p-value | 0.01255887 |
| FDR q-value | 0.058816824 |
| FWER p-Value | 0.538 |
Table: GSEA Results Summary

  

Fig 1: Enrichment plot: HALLMARK\_ESTROGEN\_RESPONSE\_EARLY      
 Profile of the Running ES Score & Positions of GeneSet Members on the Rank Ordered List

  

| SYMBOL | RANK IN GENE LIST | RANK METRIC SCORE | RUNNING ES | CORE ENRICHMENT || 1 | Krt13 | 0 | 7.531 | 0.0530 | Yes |
| 2 | Klk10 | 6 | 6.990 | 0.1011 | Yes |
| 3 | Mreg | 72 | 4.109 | 0.1163 | Yes |
| 4 | Car12 | 95 | 3.743 | 0.1380 | Yes |
| 5 | Aqp3 | 96 | 3.723 | 0.1642 | Yes |
| 6 | Zfp185 | 127 | 3.303 | 0.1811 | Yes |
| 7 | Foxc1 | 146 | 3.072 | 0.1989 | Yes |
| 8 | Gja1 | 151 | 3.006 | 0.2192 | Yes |
| 9 | Anxa9 | 209 | 2.516 | 0.2248 | Yes |
| 10 | P2ry2 | 224 | 2.419 | 0.2389 | Yes |
| 11 | Sult2b1 | 282 | 2.172 | 0.2421 | Yes |
| 12 | Cyp26b1 | 292 | 2.123 | 0.2551 | Yes |
| 13 | Cd44 | 329 | 1.976 | 0.2614 | Yes |
| 14 | Tiam1 | 334 | 1.949 | 0.2743 | Yes |
| 15 | Tubb2b | 422 | 1.621 | 0.2673 | Yes |
| 16 | Klf4 | 448 | 1.555 | 0.2729 | Yes |
| 17 | Rapgefl1 | 463 | 1.527 | 0.2807 | Yes |
| 18 | Gla | 482 | 1.492 | 0.2874 | Yes |
| 19 | Hr | 505 | 1.433 | 0.2928 | Yes |
| 20 | Clic3 | 519 | 1.394 | 0.2999 | Yes |
| 21 | Rab31 | 571 | 1.265 | 0.2980 | Yes |
| 22 | Endod1 | 595 | 1.212 | 0.3017 | Yes |
| 23 | Tpbg | 632 | 1.133 | 0.3020 | Yes |
| 24 | Slc39a6 | 634 | 1.122 | 0.3097 | Yes |
| 25 | Igfbp4 | 680 | 1.034 | 0.3075 | Yes |
| 26 | Celsr2 | 721 | 0.981 | 0.3059 | Yes |
| 27 | Blvrb | 731 | 0.962 | 0.3108 | Yes |
| 28 | Rrp12 | 753 | 0.927 | 0.3128 | Yes |
| 29 | Ccn5 | 784 | 0.883 | 0.3127 | No |
| 30 | Sh3bp5 | 856 | 0.811 | 0.3034 | No |
| 31 | Elovl5 | 882 | 0.781 | 0.3036 | No |
| 32 | Elf3 | 1051 | 0.609 | 0.2723 | No |
| 33 | Fasn | 1054 | 0.606 | 0.2762 | No |
| 34 | Wfs1 | 1079 | 0.574 | 0.2751 | No |
| 35 | Kdm4b | 1306 | -0.519 | 0.2309 | No |
| 36 | Plaat3 | 1505 | -0.552 | 0.1929 | No |
| 37 | Chpt1 | 1556 | -0.561 | 0.1863 | No |
| 38 | Tmem164 | 1726 | -0.588 | 0.1547 | No |
| 39 | Amfr | 1749 | -0.592 | 0.1542 | No |
| 40 | Inpp5f | 1783 | -0.597 | 0.1514 | No |
| 41 | Esrp2 | 1884 | -0.616 | 0.1346 | No |
| 42 | Med24 | 1888 | -0.617 | 0.1383 | No |
| 43 | Frk | 1903 | -0.619 | 0.1397 | No |
| 44 | Itpk1 | 2025 | -0.640 | 0.1185 | No |
| 45 | Il6st | 2082 | -0.650 | 0.1113 | No |
| 46 | Snx24 | 2289 | -0.686 | 0.0725 | No |
| 47 | Ugcg | 2587 | -0.744 | 0.0149 | No |
| 48 | Syngr1 | 2596 | -0.747 | 0.0184 | No |
| 49 | Slc26a2 | 2768 | -0.784 | -0.0122 | No |
| 50 | Jak2 | 2891 | -0.813 | -0.0323 | No |
| 51 | Add3 | 2899 | -0.814 | -0.0281 | No |
| 52 | Tgm2 | 2996 | -0.839 | -0.0425 | No |
| 53 | Lrig1 | 3028 | -0.849 | -0.0431 | No |
| 54 | Adcy9 | 3086 | -0.866 | -0.0491 | No |
| 55 | Inhbb | 3105 | -0.872 | -0.0467 | No |
| 56 | Akap1 | 3110 | -0.874 | -0.0414 | No |
| 57 | Slc19a2 | 3267 | -0.920 | -0.0680 | No |
| 58 | Cant1 | 3316 | -0.936 | -0.0715 | No |
| 59 | Fkbp4 | 3332 | -0.940 | -0.0681 | No |
| 60 | Elf1 | 3360 | -0.950 | -0.0671 | No |
| 61 | Papss2 | 3416 | -0.969 | -0.0720 | No |
| 62 | Fhl2 | 3508 | -1.000 | -0.0842 | No |
| 63 | Rara | 3514 | -1.003 | -0.0782 | No |
| 64 | Fos | 3575 | -1.023 | -0.0837 | No |
| 65 | Ptges | 3605 | -1.035 | -0.0825 | No |
| 66 | Abhd2 | 3632 | -1.044 | -0.0807 | No |
| 67 | Slc37a1 | 3641 | -1.048 | -0.0750 | No |
| 68 | Krt15 | 3685 | -1.071 | -0.0766 | No |
| 69 | Retreg1 | 3700 | -1.080 | -0.0719 | No |
| 70 | Tmprss3 | 3836 | -1.149 | -0.0924 | No |
| 71 | Bag1 | 3870 | -1.165 | -0.0912 | No |
| 72 | Celsr1 | 3907 | -1.189 | -0.0905 | No |
| 73 | Myof | 3964 | -1.223 | -0.0937 | No |
| 74 | Bcl2 | 3987 | -1.239 | -0.0897 | No |
| 75 | B4galt1 | 3994 | -1.244 | -0.0822 | No |
| 76 | Xbp1 | 3997 | -1.246 | -0.0738 | No |
| 77 | Asb13 | 4005 | -1.251 | -0.0665 | No |
| 78 | Tob1 | 4038 | -1.273 | -0.0643 | No |
| 79 | Deptor | 4082 | -1.314 | -0.0642 | No |
| 80 | Syt12 | 4086 | -1.316 | -0.0555 | No |
| 81 | Ttc39a | 4137 | -1.361 | -0.0566 | No |
| 82 | Ccnd1 | 4229 | -1.443 | -0.0657 | No |
| 83 | Ppif | 4262 | -1.472 | -0.0621 | No |
| 84 | Flnb | 4329 | -1.541 | -0.0652 | No |
| 85 | Tbc1d30 | 4359 | -1.589 | -0.0602 | No |
| 86 | Nav2 | 4458 | -1.753 | -0.0686 | No |
| 87 | Krt8 | 4476 | -1.778 | -0.0596 | No |
| 88 | Nadsyn1 | 4491 | -1.796 | -0.0500 | No |
| 89 | Krt19 | 4510 | -1.837 | -0.0409 | No |
| 90 | Muc1 | 4556 | -1.935 | -0.0368 | No |
| 91 | Areg | 4558 | -1.937 | -0.0233 | No |
| 92 | Stc2 | 4564 | -1.949 | -0.0107 | No |
| 93 | Prss23 | 4568 | -1.964 | 0.0025 | No |
| 94 | Tff3 | 4571 | -1.966 | 0.0159 | No |
| 95 | Ovol2 | 4573 | -1.973 | 0.0296 | No |
| 96 | Sema3b | 4798 | -3.196 | 0.0047 | No |
Table: GSEA details [plain text format]

  

Fig 2: HALLMARK\_ESTROGEN\_RESPONSE\_EARLY: Random ES distribution      
 Gene set null distribution of ES for **HALLMARK\_ESTROGEN\_RESPONSE\_EARLY**

  
