# Supplementary material for: TMPRSS11B promotes an acidified microenvironment and immune suppression in squamous lung cancer
Source: EMBO Rep. 2025 Nov 10;26(24):6346–79. doi: 10.1038/s44319-025-00631-1 (PMC12714794; doi:10.1038/s44319-025-00631-1)
Supplement: Supplementary file 14 — Figure EV2 Source Data [file 44319_2025_631_MOESM14_ESM.zip › Figure EV2/EV2D-E/GSEA_Broad Institute_Mh_T11b-high LUSC vs LUAD/HALLMARK_ESTROGEN_RESPONSE_LATE.html]

Details for gene set HALLMARK\_ESTROGEN\_RESPONSE\_LATE[GSEA]

|  || Dataset | Ranked list\_DGE\_squamousT11b\_vs\_all adenosadeno\_HSE13-NT copy |
| Phenotype | NoPhenotypeAvailable |
| Upregulated in class | na\_pos |
| GeneSet | HALLMARK\_ESTROGEN\_RESPONSE\_LATE |
| Enrichment Score (ES) | 0.3592631 |
| Normalized Enrichment Score (NES) | 1.7571822 |
| Nominal p-value | 0.011744967 |
| FDR q-value | 0.026838824 |
| FWER p-Value | 0.2 |
Table: GSEA Results Summary

  

Fig 1: Enrichment plot: HALLMARK\_ESTROGEN\_RESPONSE\_LATE      
 Profile of the Running ES Score & Positions of GeneSet Members on the Rank Ordered List

  

| SYMBOL | RANK IN GENE LIST | RANK METRIC SCORE | RUNNING ES | CORE ENRICHMENT || 1 | Krt13 | 0 | 7.531 | 0.0562 | Yes |
| 2 | Klk10 | 6 | 6.990 | 0.1073 | Yes |
| 3 | Cpe | 77 | 4.024 | 0.1226 | Yes |
| 4 | Klk11 | 90 | 3.861 | 0.1488 | Yes |
| 5 | Car12 | 95 | 3.743 | 0.1759 | Yes |
| 6 | S100a9 | 110 | 3.624 | 0.2000 | Yes |
| 7 | Trim29 | 118 | 3.521 | 0.2248 | Yes |
| 8 | Foxc1 | 146 | 3.072 | 0.2421 | Yes |
| 9 | Anxa9 | 209 | 2.516 | 0.2477 | Yes |
| 10 | Ltf | 264 | 2.250 | 0.2531 | Yes |
| 11 | Sult2b1 | 282 | 2.172 | 0.2658 | Yes |
| 12 | Cyp26b1 | 292 | 2.123 | 0.2797 | Yes |
| 13 | Perp | 322 | 1.994 | 0.2885 | Yes |
| 14 | Cd44 | 329 | 1.976 | 0.3019 | Yes |
| 15 | Tiam1 | 334 | 1.949 | 0.3156 | Yes |
| 16 | Ckb | 372 | 1.769 | 0.3210 | Yes |
| 17 | Klf4 | 448 | 1.555 | 0.3168 | Yes |
| 18 | Rapgefl1 | 463 | 1.527 | 0.3252 | Yes |
| 19 | Gla | 482 | 1.492 | 0.3326 | Yes |
| 20 | Hr | 505 | 1.433 | 0.3386 | Yes |
| 21 | Clic3 | 519 | 1.394 | 0.3463 | Yes |
| 22 | Pkp3 | 529 | 1.375 | 0.3546 | Yes |
| 23 | Rab31 | 571 | 1.265 | 0.3554 | Yes |
| 24 | Tpbg | 632 | 1.133 | 0.3512 | Yes |
| 25 | Igfbp4 | 680 | 1.034 | 0.3490 | Yes |
| 26 | Celsr2 | 721 | 0.981 | 0.3479 | Yes |
| 27 | Blvrb | 731 | 0.962 | 0.3532 | Yes |
| 28 | Prkar2b | 737 | 0.958 | 0.3593 | Yes |
| 29 | Ccn5 | 784 | 0.883 | 0.3561 | No |
| 30 | Elovl5 | 882 | 0.781 | 0.3415 | No |
| 31 | Wfs1 | 1079 | 0.574 | 0.3044 | No |
| 32 | Sord | 1146 | 0.514 | 0.2943 | No |
| 33 | Car2 | 1171 | -0.500 | 0.2930 | No |
| 34 | Plxnb1 | 1212 | -0.507 | 0.2883 | No |
| 35 | Plaat3 | 1505 | -0.552 | 0.2308 | No |
| 36 | Chpt1 | 1556 | -0.561 | 0.2244 | No |
| 37 | Ugdh | 1692 | -0.583 | 0.2003 | No |
| 38 | Amfr | 1749 | -0.592 | 0.1929 | No |
| 39 | Snx10 | 1850 | -0.610 | 0.1763 | No |
| 40 | Frk | 1903 | -0.619 | 0.1699 | No |
| 41 | Itpk1 | 2025 | -0.640 | 0.1492 | No |
| 42 | Il6st | 2082 | -0.650 | 0.1422 | No |
| 43 | Idh2 | 2529 | -0.733 | 0.0535 | No |
| 44 | Fabp5 | 2745 | -0.778 | 0.0139 | No |
| 45 | Slc26a2 | 2768 | -0.784 | 0.0151 | No |
| 46 | Jak2 | 2891 | -0.813 | -0.0045 | No |
| 47 | Add3 | 2899 | -0.814 | 0.0001 | No |
| 48 | Unc13b | 3058 | -0.857 | -0.0269 | No |
| 49 | Isg20 | 3115 | -0.875 | -0.0322 | No |
| 50 | Sgk1 | 3151 | -0.885 | -0.0330 | No |
| 51 | Fkbp4 | 3332 | -0.940 | -0.0640 | No |
| 52 | Rabep1 | 3345 | -0.945 | -0.0594 | No |
| 53 | Papss2 | 3416 | -0.969 | -0.0670 | No |
| 54 | Lsr | 3431 | -0.976 | -0.0627 | No |
| 55 | Fos | 3575 | -1.023 | -0.0852 | No |
| 56 | Ptges | 3605 | -1.035 | -0.0836 | No |
| 57 | Dnajc1 | 3626 | -1.042 | -0.0801 | No |
| 58 | Abhd2 | 3632 | -1.044 | -0.0733 | No |
| 59 | Pdcd4 | 3741 | -1.099 | -0.0879 | No |
| 60 | Lamc2 | 3814 | -1.136 | -0.0946 | No |
| 61 | Tmprss3 | 3836 | -1.149 | -0.0905 | No |
| 62 | Bag1 | 3870 | -1.165 | -0.0888 | No |
| 63 | Myof | 3964 | -1.223 | -0.0993 | No |
| 64 | Dcxr | 3966 | -1.224 | -0.0904 | No |
| 65 | Bcl2 | 3987 | -1.239 | -0.0853 | No |
| 66 | Aldh3a2 | 3988 | -1.239 | -0.0761 | No |
| 67 | Xbp1 | 3997 | -1.246 | -0.0685 | No |
| 68 | Tob1 | 4038 | -1.273 | -0.0674 | No |
| 69 | Tspan13 | 4111 | -1.336 | -0.0727 | No |
| 70 | Ccnd1 | 4229 | -1.443 | -0.0866 | No |
| 71 | Dnajc12 | 4234 | -1.449 | -0.0766 | No |
| 72 | Ppif | 4262 | -1.472 | -0.0713 | No |
| 73 | St6galnac2 | 4289 | -1.494 | -0.0657 | No |
| 74 | Flnb | 4329 | -1.541 | -0.0624 | No |
| 75 | Cdh1 | 4436 | -1.716 | -0.0720 | No |
| 76 | Krt19 | 4510 | -1.837 | -0.0737 | No |
| 77 | Areg | 4558 | -1.937 | -0.0692 | No |
| 78 | Prss23 | 4568 | -1.964 | -0.0564 | No |
| 79 | Tff3 | 4571 | -1.966 | -0.0422 | No |
| 80 | Ovol2 | 4573 | -1.973 | -0.0276 | No |
| 81 | Agr2 | 4671 | -2.265 | -0.0312 | No |
| 82 | Hmgcs2 | 4714 | -2.435 | -0.0219 | No |
| 83 | Homer2 | 4752 | -2.684 | -0.0097 | No |
| 84 | Sema3b | 4798 | -3.196 | 0.0046 | No |
Table: GSEA details [plain text format]

  

Fig 2: HALLMARK\_ESTROGEN\_RESPONSE\_LATE: Random ES distribution      
 Gene set null distribution of ES for **HALLMARK\_ESTROGEN\_RESPONSE\_LATE**

  
