# Supplementary material for: TMPRSS11B promotes an acidified microenvironment and immune suppression in squamous lung cancer
Source: EMBO Rep. 2025 Nov 10;26(24):6346–79. doi: 10.1038/s44319-025-00631-1 (PMC12714794; doi:10.1038/s44319-025-00631-1)
Supplement: Supplementary file 14 — Figure EV2 Source Data [file 44319_2025_631_MOESM14_ESM.zip › Figure EV2/EV2D-E/GSEA_Broad Institute_Mh_T11b-high LUSC vs LUAD/HALLMARK_G2M_CHECKPOINT.html]

Details for gene set HALLMARK\_G2M\_CHECKPOINT[GSEA]

|  || Dataset | Ranked list\_DGE\_squamousT11b\_vs\_all adenosadeno\_HSE13-NT copy |
| Phenotype | NoPhenotypeAvailable |
| Upregulated in class | na\_neg |
| GeneSet | HALLMARK\_G2M\_CHECKPOINT |
| Enrichment Score (ES) | -0.12226891 |
| Normalized Enrichment Score (NES) | -0.617614 |
| Nominal p-value | 0.9575472 |
| FDR q-value | 1.0 |
| FWER p-Value | 1.0 |
Table: GSEA Results Summary

  

Fig 1: Enrichment plot: HALLMARK\_G2M\_CHECKPOINT      
 Profile of the Running ES Score & Positions of GeneSet Members on the Rank Ordered List

  

| SYMBOL | RANK IN GENE LIST | RANK METRIC SCORE | RUNNING ES | CORE ENRICHMENT || 1 | Ccna2 | 582 | 1.243 | -0.0976 | Yes |
| 2 | Cenpa | 604 | 1.193 | -0.0784 | Yes |
| 3 | Cks2 | 729 | 0.967 | -0.0852 | Yes |
| 4 | Mki67 | 758 | 0.922 | -0.0728 | Yes |
| 5 | Hif1a | 810 | 0.857 | -0.0666 | Yes |
| 6 | Cdk1 | 820 | 0.842 | -0.0518 | Yes |
| 7 | Map3k20 | 873 | 0.798 | -0.0469 | Yes |
| 8 | Nusap1 | 907 | 0.752 | -0.0389 | Yes |
| 9 | Incenp | 937 | 0.718 | -0.0307 | Yes |
| 10 | Tgfb1 | 940 | 0.718 | -0.0169 | Yes |
| 11 | Racgap1 | 968 | 0.689 | -0.0089 | Yes |
| 12 | Mad2l1 | 1028 | 0.624 | -0.0089 | Yes |
| 13 | Rbl1 | 1084 | 0.569 | -0.0092 | Yes |
| 14 | Mcm5 | 1087 | 0.567 | 0.0016 | Yes |
| 15 | Stmn1 | 1124 | 0.536 | 0.0047 | Yes |
| 16 | Smc4 | 1134 | 0.523 | 0.0131 | Yes |
| 17 | Smc1a | 1189 | -0.503 | 0.0118 | Yes |
| 18 | Ilf3 | 1356 | -0.527 | -0.0126 | Yes |
| 19 | Srsf1 | 1410 | -0.533 | -0.0132 | Yes |
| 20 | Abl1 | 1424 | -0.536 | -0.0053 | Yes |
| 21 | Cbx1 | 1451 | -0.541 | -0.0000 | Yes |
| 22 | Dtymk | 1507 | -0.552 | -0.0007 | Yes |
| 23 | Cul5 | 1508 | -0.552 | 0.0103 | Yes |
| 24 | Cul3 | 1541 | -0.559 | 0.0147 | Yes |
| 25 | Hira | 1569 | -0.563 | 0.0201 | Yes |
| 26 | Mnat1 | 1645 | -0.575 | 0.0158 | Yes |
| 27 | Rad21 | 1806 | -0.601 | -0.0059 | Yes |
| 28 | Stag1 | 1807 | -0.602 | 0.0060 | Yes |
| 29 | Srsf2 | 1865 | -0.613 | 0.0062 | Yes |
| 30 | Orc5 | 1896 | -0.618 | 0.0122 | Yes |
| 31 | Atrx | 1907 | -0.619 | 0.0223 | Yes |
| 32 | Slc7a1 | 1940 | -0.624 | 0.0280 | Yes |
| 33 | Ctcf | 2004 | -0.637 | 0.0274 | Yes |
| 34 | Mtf2 | 2022 | -0.639 | 0.0365 | Yes |
| 35 | Pura | 2145 | -0.662 | 0.0240 | Yes |
| 36 | Pds5b | 2189 | -0.668 | 0.0282 | Yes |
| 37 | Amd1 | 2241 | -0.679 | 0.0309 | Yes |
| 38 | Sap30 | 2296 | -0.687 | 0.0332 | Yes |
| 39 | Prmt5 | 2299 | -0.688 | 0.0464 | Yes |
| 40 | Hnrnpd | 2328 | -0.694 | 0.0543 | Yes |
| 41 | Numa1 | 2406 | -0.707 | 0.0522 | Yes |
| 42 | Uck2 | 2417 | -0.709 | 0.0641 | Yes |
| 43 | Ythdc1 | 2425 | -0.712 | 0.0768 | Yes |
| 44 | Arid4a | 2439 | -0.715 | 0.0882 | Yes |
| 45 | Nup98 | 2501 | -0.728 | 0.0898 | Yes |
| 46 | Smarcc1 | 2624 | -0.753 | 0.0791 | Yes |
| 47 | Fancc | 2721 | -0.771 | 0.0743 | Yes |
| 48 | Tent4a | 2759 | -0.781 | 0.0820 | Yes |
| 49 | Prpf4b | 2930 | -0.820 | 0.0625 | Yes |
| 50 | Kif5b | 2964 | -0.830 | 0.0721 | Yes |
| 51 | Lig3 | 3109 | -0.874 | 0.0591 | Yes |
| 52 | Cul1 | 3334 | -0.941 | 0.0307 | Yes |
| 53 | Efna5 | 3690 | -1.073 | -0.0226 | Yes |
| 54 | Tle3 | 3809 | -1.133 | -0.0249 | Yes |
| 55 | Smad3 | 3903 | -1.184 | -0.0210 | Yes |
| 56 | Cdc25a | 4011 | -1.255 | -0.0186 | Yes |
| 57 | Kif23 | 4104 | -1.332 | -0.0115 | Yes |
| 58 | Ccnd1 | 4229 | -1.443 | -0.0089 | Yes |
| 59 | Slc38a1 | 4384 | -1.623 | -0.0091 | Yes |
| 60 | Slc12a2 | 4688 | -2.339 | -0.0264 | Yes |
| 61 | Dmd | 4759 | -2.718 | 0.0128 | Yes |
Table: GSEA details [plain text format]

  

Fig 2: HALLMARK\_G2M\_CHECKPOINT: Random ES distribution      
 Gene set null distribution of ES for **HALLMARK\_G2M\_CHECKPOINT**

  
