# Supplementary material for: TMPRSS11B promotes an acidified microenvironment and immune suppression in squamous lung cancer
Source: EMBO Rep. 2025 Nov 10;26(24):6346–79. doi: 10.1038/s44319-025-00631-1 (PMC12714794; doi:10.1038/s44319-025-00631-1)
Supplement: Supplementary file 14 — Figure EV2 Source Data [file 44319_2025_631_MOESM14_ESM.zip › Figure EV2/EV2D-E/GSEA_Broad Institute_Mh_T11b-high LUSC vs LUAD/HALLMARK_HYPOXIA.html]

Details for gene set HALLMARK\_HYPOXIA[GSEA]

|  || Dataset | Ranked list\_DGE\_squamousT11b\_vs\_all adenosadeno\_HSE13-NT copy |
| Phenotype | NoPhenotypeAvailable |
| Upregulated in class | na\_pos |
| GeneSet | HALLMARK\_HYPOXIA |
| Enrichment Score (ES) | 0.3108181 |
| Normalized Enrichment Score (NES) | 1.5083324 |
| Nominal p-value | 0.03937008 |
| FDR q-value | 0.086135864 |
| FWER p-Value | 0.724 |
Table: GSEA Results Summary

  

Fig 1: Enrichment plot: HALLMARK\_HYPOXIA      
 Profile of the Running ES Score & Positions of GeneSet Members on the Rank Ordered List

  

| SYMBOL | RANK IN GENE LIST | RANK METRIC SCORE | RUNNING ES | CORE ENRICHMENT || 1 | Pkp1 | 57 | 4.358 | 0.0352 | Yes |
| 2 | Car12 | 95 | 3.743 | 0.0680 | Yes |
| 3 | Tmem45a | 104 | 3.663 | 0.1060 | Yes |
| 4 | Hmox1 | 144 | 3.102 | 0.1315 | Yes |
| 5 | Sult2b1 | 282 | 2.172 | 0.1261 | Yes |
| 6 | Pim1 | 319 | 2.011 | 0.1404 | Yes |
| 7 | Mif | 323 | 1.991 | 0.1613 | Yes |
| 8 | Dusp1 | 339 | 1.923 | 0.1790 | Yes |
| 9 | S100a4 | 353 | 1.867 | 0.1965 | Yes |
| 10 | Hk2 | 428 | 1.617 | 0.1984 | Yes |
| 11 | Tgfbi | 502 | 1.443 | 0.1987 | Yes |
| 12 | Plin2 | 507 | 1.431 | 0.2134 | Yes |
| 13 | Cited2 | 522 | 1.391 | 0.2255 | Yes |
| 14 | Ppp1r15a | 543 | 1.345 | 0.2359 | Yes |
| 15 | Sdc3 | 550 | 1.335 | 0.2491 | Yes |
| 16 | Prdx5 | 601 | 1.198 | 0.2515 | Yes |
| 17 | Gpc1 | 623 | 1.167 | 0.2597 | Yes |
| 18 | Tpbg | 632 | 1.133 | 0.2703 | Yes |
| 19 | Atf3 | 643 | 1.106 | 0.2802 | Yes |
| 20 | Ndrg1 | 681 | 1.033 | 0.2836 | Yes |
| 21 | Ndst1 | 773 | 0.905 | 0.2743 | Yes |
| 22 | Ccn5 | 784 | 0.883 | 0.2817 | Yes |
| 23 | Ccng2 | 808 | 0.859 | 0.2862 | Yes |
| 24 | Wsb1 | 827 | 0.838 | 0.2915 | Yes |
| 25 | Fam162a | 858 | 0.810 | 0.2939 | Yes |
| 26 | Cdkn1a | 861 | 0.808 | 0.3023 | Yes |
| 27 | Ets1 | 863 | 0.807 | 0.3108 | Yes |
| 28 | Ier3 | 967 | 0.691 | 0.2966 | No |
| 29 | Pgk1 | 1029 | 0.624 | 0.2905 | No |
| 30 | Ddit3 | 1038 | 0.616 | 0.2955 | No |
| 31 | Maff | 1047 | 0.610 | 0.3004 | No |
| 32 | Serpine1 | 1049 | 0.610 | 0.3068 | No |
| 33 | Tgfb3 | 1153 | 0.509 | 0.2906 | No |
| 34 | Ndst2 | 1336 | -0.523 | 0.2579 | No |
| 35 | Selenbp1 | 1398 | -0.532 | 0.2509 | No |
| 36 | Hdlbp | 1479 | -0.548 | 0.2399 | No |
| 37 | Gaa | 1605 | -0.569 | 0.2198 | No |
| 38 | Mt1 | 1658 | -0.576 | 0.2150 | No |
| 39 | Bgn | 1714 | -0.584 | 0.2098 | No |
| 40 | F3 | 1925 | -0.622 | 0.1723 | No |
| 41 | Pnrc1 | 1935 | -0.624 | 0.1771 | No |
| 42 | Pdgfb | 2026 | -0.640 | 0.1651 | No |
| 43 | Grhpr | 2089 | -0.652 | 0.1591 | No |
| 44 | Ugp2 | 2112 | -0.656 | 0.1616 | No |
| 45 | Noct | 2171 | -0.665 | 0.1565 | No |
| 46 | Sap30 | 2296 | -0.687 | 0.1379 | No |
| 47 | Errfi1 | 2313 | -0.690 | 0.1420 | No |
| 48 | Klhl24 | 2386 | -0.704 | 0.1344 | No |
| 49 | Sdc4 | 2418 | -0.710 | 0.1356 | No |
| 50 | Aldoc | 2488 | -0.726 | 0.1289 | No |
| 51 | Ilvbl | 2750 | -0.780 | 0.0823 | No |
| 52 | Gys1 | 2921 | -0.818 | 0.0554 | No |
| 53 | Tgm2 | 2996 | -0.839 | 0.0489 | No |
| 54 | Nr3c1 | 2997 | -0.839 | 0.0580 | No |
| 55 | B3galt6 | 3022 | -0.846 | 0.0621 | No |
| 56 | Isg20 | 3115 | -0.875 | 0.0522 | No |
| 57 | Zfp292 | 3193 | -0.897 | 0.0457 | No |
| 58 | Casp6 | 3257 | -0.917 | 0.0423 | No |
| 59 | Klf7 | 3489 | -0.993 | 0.0044 | No |
| 60 | Prkca | 3544 | -1.013 | 0.0040 | No |
| 61 | Fos | 3575 | -1.023 | 0.0088 | No |
| 62 | Galk1 | 3646 | -1.051 | 0.0054 | No |
| 63 | Pgm2 | 3654 | -1.057 | 0.0154 | No |
| 64 | Cavin1 | 3689 | -1.073 | 0.0199 | No |
| 65 | Ccn2 | 3829 | -1.143 | 0.0030 | No |
| 66 | Dcn | 3919 | -1.196 | -0.0028 | No |
| 67 | Eno3 | 3938 | -1.207 | 0.0065 | No |
| 68 | Slc25a1 | 3974 | -1.228 | 0.0124 | No |
| 69 | Bcl2 | 3987 | -1.239 | 0.0233 | No |
| 70 | Kdelr3 | 4089 | -1.318 | 0.0163 | No |
| 71 | Ackr3 | 4187 | -1.397 | 0.0110 | No |
| 72 | Ankzf1 | 4201 | -1.410 | 0.0235 | No |
| 73 | Nedd4l | 4292 | -1.497 | 0.0208 | No |
| 74 | Csrp2 | 4490 | -1.794 | -0.0013 | No |
| 75 | Stc2 | 4564 | -1.949 | 0.0045 | No |
| 76 | Pam | 4623 | -2.099 | 0.0150 | No |
| 77 | Hs3st1 | 4710 | -2.428 | 0.0232 | No |
Table: GSEA details [plain text format]

  

Fig 2: HALLMARK\_HYPOXIA: Random ES distribution      
 Gene set null distribution of ES for **HALLMARK\_HYPOXIA**

  
