# Supplementary material for: TMPRSS11B promotes an acidified microenvironment and immune suppression in squamous lung cancer
Source: EMBO Rep. 2025 Nov 10;26(24):6346–79. doi: 10.1038/s44319-025-00631-1 (PMC12714794; doi:10.1038/s44319-025-00631-1)
Supplement: Supplementary file 14 — Figure EV2 Source Data [file 44319_2025_631_MOESM14_ESM.zip › Figure EV2/EV2D-E/GSEA_Broad Institute_Mh_T11b-high LUSC vs LUAD/HALLMARK_IL2_STAT5_SIGNALING.html]

Details for gene set HALLMARK\_IL2\_STAT5\_SIGNALING[GSEA]

|  || Dataset | Ranked list\_DGE\_squamousT11b\_vs\_all adenosadeno\_HSE13-NT copy |
| Phenotype | NoPhenotypeAvailable |
| Upregulated in class | na\_pos |
| GeneSet | HALLMARK\_IL2\_STAT5\_SIGNALING |
| Enrichment Score (ES) | 0.34558317 |
| Normalized Enrichment Score (NES) | 1.68828 |
| Nominal p-value | 0.0015748031 |
| FDR q-value | 0.032411166 |
| FWER p-Value | 0.317 |
Table: GSEA Results Summary

  

Fig 1: Enrichment plot: HALLMARK\_IL2\_STAT5\_SIGNALING      
 Profile of the Running ES Score & Positions of GeneSet Members on the Rank Ordered List

  

| SYMBOL | RANK IN GENE LIST | RANK METRIC SCORE | RUNNING ES | CORE ENRICHMENT || 1 | Ecm1 | 27 | 5.794 | 0.0469 | Yes |
| 2 | Il1r2 | 32 | 5.303 | 0.0941 | Yes |
| 3 | Spp1 | 69 | 4.139 | 0.1241 | Yes |
| 4 | Emp1 | 79 | 4.007 | 0.1585 | Yes |
| 5 | Hopx | 134 | 3.244 | 0.1765 | Yes |
| 6 | Plpp1 | 136 | 3.208 | 0.2054 | Yes |
| 7 | Mxd1 | 163 | 2.892 | 0.2262 | Yes |
| 8 | Aplp1 | 314 | 2.028 | 0.2129 | Yes |
| 9 | Pim1 | 319 | 2.011 | 0.2303 | Yes |
| 10 | Cd44 | 329 | 1.976 | 0.2463 | Yes |
| 11 | Tiam1 | 334 | 1.949 | 0.2632 | Yes |
| 12 | Gsto1 | 397 | 1.701 | 0.2655 | Yes |
| 13 | Hk2 | 428 | 1.617 | 0.2739 | Yes |
| 14 | Slc39a8 | 430 | 1.607 | 0.2882 | Yes |
| 15 | Tnfrsf1b | 443 | 1.566 | 0.2999 | Yes |
| 16 | Gpr65 | 478 | 1.498 | 0.3063 | Yes |
| 17 | Ctsz | 493 | 1.463 | 0.3166 | Yes |
| 18 | Plin2 | 507 | 1.431 | 0.3269 | Yes |
| 19 | Ager | 573 | 1.264 | 0.3246 | Yes |
| 20 | Capg | 574 | 1.263 | 0.3361 | Yes |
| 21 | Fah | 583 | 1.236 | 0.3456 | Yes |
| 22 | Ndrg1 | 681 | 1.033 | 0.3345 | No |
| 23 | Phlda1 | 766 | 0.908 | 0.3250 | No |
| 24 | Gbp3 | 801 | 0.866 | 0.3257 | No |
| 25 | Gadd45b | 804 | 0.862 | 0.3331 | No |
| 26 | Prnp | 806 | 0.860 | 0.3407 | No |
| 27 | Il3ra | 859 | 0.808 | 0.3370 | No |
| 28 | Adam19 | 933 | 0.720 | 0.3281 | No |
| 29 | Dennd5a | 936 | 0.719 | 0.3342 | No |
| 30 | Ckap4 | 985 | 0.668 | 0.3302 | No |
| 31 | Fgl2 | 1019 | 0.634 | 0.3290 | No |
| 32 | Maff | 1047 | 0.610 | 0.3288 | No |
| 33 | Ahnak | 1090 | 0.565 | 0.3251 | No |
| 34 | Pnp | 1110 | 0.546 | 0.3260 | No |
| 35 | Hipk2 | 1145 | 0.516 | 0.3235 | No |
| 36 | Ifngr1 | 1155 | 0.509 | 0.3262 | No |
| 37 | Car2 | 1171 | -0.500 | 0.3276 | No |
| 38 | Anxa4 | 1225 | -0.509 | 0.3210 | No |
| 39 | Cdc42se2 | 1251 | -0.513 | 0.3204 | No |
| 40 | Casp3 | 1261 | -0.514 | 0.3232 | No |
| 41 | Swap70 | 1289 | -0.518 | 0.3222 | No |
| 42 | Socs2 | 1448 | -0.541 | 0.2937 | No |
| 43 | Il4ra | 1455 | -0.542 | 0.2974 | No |
| 44 | Ptrh2 | 1845 | -0.609 | 0.2208 | No |
| 45 | Eef1akmt1 | 1880 | -0.616 | 0.2192 | No |
| 46 | Spred2 | 1893 | -0.618 | 0.2223 | No |
| 47 | Enpp1 | 2011 | -0.638 | 0.2034 | No |
| 48 | Pou2f1 | 2355 | -0.698 | 0.1373 | No |
| 49 | Map6 | 2390 | -0.704 | 0.1366 | No |
| 50 | Uck2 | 2417 | -0.709 | 0.1375 | No |
| 51 | Nfkbiz | 2566 | -0.740 | 0.1130 | No |
| 52 | P2rx4 | 2770 | -0.785 | 0.0773 | No |
| 53 | Ttc39b | 2878 | -0.810 | 0.0620 | No |
| 54 | Tgm2 | 2996 | -0.839 | 0.0450 | No |
| 55 | Lrig1 | 3028 | -0.849 | 0.0461 | No |
| 56 | Ncoa3 | 3037 | -0.851 | 0.0521 | No |
| 57 | S100a1 | 3077 | -0.864 | 0.0518 | No |
| 58 | Dcps | 3130 | -0.879 | 0.0488 | No |
| 59 | Lclat1 | 3199 | -0.897 | 0.0425 | No |
| 60 | Wls | 3401 | -0.961 | 0.0089 | No |
| 61 | Slc1a5 | 3499 | -0.997 | -0.0026 | No |
| 62 | Nt5e | 3535 | -1.010 | -0.0008 | No |
| 63 | Sh3bgrl2 | 3772 | -1.111 | -0.0405 | No |
| 64 | Traf1 | 3786 | -1.119 | -0.0331 | No |
| 65 | Pdcd2l | 3851 | -1.158 | -0.0361 | No |
| 66 | Galm | 3869 | -1.165 | -0.0291 | No |
| 67 | Ptch1 | 3920 | -1.197 | -0.0288 | No |
| 68 | Eno3 | 3938 | -1.207 | -0.0215 | No |
| 69 | Serpinb6a | 3957 | -1.219 | -0.0142 | No |
| 70 | Bcl2 | 3987 | -1.239 | -0.0091 | No |
| 71 | Xbp1 | 3997 | -1.246 | 0.0003 | No |
| 72 | Etv4 | 4030 | -1.268 | 0.0051 | No |
| 73 | Bmp2 | 4098 | -1.322 | 0.0029 | No |
| 74 | Tnfrsf21 | 4266 | -1.475 | -0.0190 | No |
| 75 | Amacr | 4274 | -1.482 | -0.0070 | No |
| 76 | Alcam | 4291 | -1.496 | 0.0032 | No |
| 77 | Umps | 4447 | -1.741 | -0.0137 | No |
| 78 | Rabgap1l | 4459 | -1.754 | -0.0001 | No |
| 79 | Etfbkmt | 4483 | -1.782 | 0.0112 | No |
| 80 | Muc1 | 4556 | -1.935 | 0.0135 | No |
| 81 | Ccnd2 | 4672 | -2.265 | 0.0098 | No |
| 82 | Plagl1 | 4687 | -2.337 | 0.0281 | No |
Table: GSEA details [plain text format]

  

Fig 2: HALLMARK\_IL2\_STAT5\_SIGNALING: Random ES distribution      
 Gene set null distribution of ES for **HALLMARK\_IL2\_STAT5\_SIGNALING**

  
