# Supplementary material for: TMPRSS11B promotes an acidified microenvironment and immune suppression in squamous lung cancer
Source: EMBO Rep. 2025 Nov 10;26(24):6346–79. doi: 10.1038/s44319-025-00631-1 (PMC12714794; doi:10.1038/s44319-025-00631-1)
Supplement: Supplementary file 14 — Figure EV2 Source Data [file 44319_2025_631_MOESM14_ESM.zip › Figure EV2/EV2D-E/GSEA_Broad Institute_Mh_T11b-high LUSC vs LUAD/HALLMARK_IL6_JAK_STAT3_SIGNALING.html]

Details for gene set HALLMARK\_IL6\_JAK\_STAT3\_SIGNALING[GSEA]

|  || Dataset | Ranked list\_DGE\_squamousT11b\_vs\_all adenosadeno\_HSE13-NT copy |
| Phenotype | NoPhenotypeAvailable |
| Upregulated in class | na\_pos |
| GeneSet | HALLMARK\_IL6\_JAK\_STAT3\_SIGNALING |
| Enrichment Score (ES) | 0.5494449 |
| Normalized Enrichment Score (NES) | 2.0809844 |
| Nominal p-value | 0.0 |
| FDR q-value | 7.217495E-4 |
| FWER p-Value | 0.003 |
Table: GSEA Results Summary

  

Fig 1: Enrichment plot: HALLMARK\_IL6\_JAK\_STAT3\_SIGNALING      
 Profile of the Running ES Score & Positions of GeneSet Members on the Rank Ordered List

  

| SYMBOL | RANK IN GENE LIST | RANK METRIC SCORE | RUNNING ES | CORE ENRICHMENT || 1 | Il1r2 | 32 | 5.303 | 0.1108 | Yes |
| 2 | Csf3r | 124 | 3.373 | 0.1665 | Yes |
| 3 | Hmox1 | 144 | 3.102 | 0.2312 | Yes |
| 4 | Cd36 | 199 | 2.567 | 0.2768 | Yes |
| 5 | Il1b | 240 | 2.351 | 0.3205 | Yes |
| 6 | Csf2ra | 299 | 2.108 | 0.3551 | Yes |
| 7 | Pim1 | 319 | 2.011 | 0.3957 | Yes |
| 8 | Crlf2 | 325 | 1.984 | 0.4386 | Yes |
| 9 | Cd44 | 329 | 1.976 | 0.4817 | Yes |
| 10 | Tnf | 391 | 1.722 | 0.5071 | Yes |
| 11 | Tnfrsf1b | 443 | 1.566 | 0.5311 | Yes |
| 12 | Irf1 | 508 | 1.429 | 0.5494 | Yes |
| 13 | Il2rg | 648 | 1.091 | 0.5446 | No |
| 14 | Il3ra | 859 | 0.808 | 0.5187 | No |
| 15 | Tgfb1 | 940 | 0.718 | 0.5179 | No |
| 16 | Il1r1 | 1040 | 0.614 | 0.5109 | No |
| 17 | Bak1 | 1066 | 0.590 | 0.5187 | No |
| 18 | Ptpn1 | 1126 | 0.534 | 0.5182 | No |
| 19 | Ifngr1 | 1155 | 0.509 | 0.5237 | No |
| 20 | Stat3 | 1393 | -0.531 | 0.4860 | No |
| 21 | Il4ra | 1455 | -0.542 | 0.4853 | No |
| 22 | Il6st | 2082 | -0.650 | 0.3691 | No |
| 23 | Socs3 | 2116 | -0.657 | 0.3767 | No |
| 24 | Tnfrsf12a | 4008 | -1.254 | 0.0101 | No |
| 25 | Tnfrsf21 | 4266 | -1.475 | -0.0109 | No |
| 26 | Tlr2 | 4716 | -2.445 | -0.0504 | No |
| 27 | Reg1 | 4802 | -3.247 | 0.0038 | No |
Table: GSEA details [plain text format]

  

Fig 2: HALLMARK\_IL6\_JAK\_STAT3\_SIGNALING: Random ES distribution      
 Gene set null distribution of ES for **HALLMARK\_IL6\_JAK\_STAT3\_SIGNALING**

  
