# Supplementary material for: TMPRSS11B promotes an acidified microenvironment and immune suppression in squamous lung cancer
Source: EMBO Rep. 2025 Nov 10;26(24):6346–79. doi: 10.1038/s44319-025-00631-1 (PMC12714794; doi:10.1038/s44319-025-00631-1)
Supplement: Supplementary file 14 — Figure EV2 Source Data [file 44319_2025_631_MOESM14_ESM.zip › Figure EV2/EV2D-E/GSEA_Broad Institute_Mh_T11b-high LUSC vs LUAD/HALLMARK_INFLAMMATORY_RESPONSE.html]

Details for gene set HALLMARK\_INFLAMMATORY\_RESPONSE[GSEA]

|  || Dataset | Ranked list\_DGE\_squamousT11b\_vs\_all adenosadeno\_HSE13-NT copy |
| Phenotype | NoPhenotypeAvailable |
| Upregulated in class | na\_pos |
| GeneSet | HALLMARK\_INFLAMMATORY\_RESPONSE |
| Enrichment Score (ES) | 0.47381657 |
| Normalized Enrichment Score (NES) | 2.2252533 |
| Nominal p-value | 0.0 |
| FDR q-value | 0.0 |
| FWER p-Value | 0.0 |
Table: GSEA Results Summary

  

Fig 1: Enrichment plot: HALLMARK\_INFLAMMATORY\_RESPONSE      
 Profile of the Running ES Score & Positions of GeneSet Members on the Rank Ordered List

  

| SYMBOL | RANK IN GENE LIST | RANK METRIC SCORE | RUNNING ES | CORE ENRICHMENT || 1 | Cxcl5 | 101 | 3.685 | 0.0166 | Yes |
| 2 | Il1a | 119 | 3.497 | 0.0490 | Yes |
| 3 | Csf3r | 124 | 3.373 | 0.0828 | Yes |
| 4 | Gna15 | 161 | 2.913 | 0.1052 | Yes |
| 5 | Mxd1 | 163 | 2.892 | 0.1347 | Yes |
| 6 | Abca1 | 172 | 2.809 | 0.1619 | Yes |
| 7 | Cybb | 173 | 2.805 | 0.1908 | Yes |
| 8 | Msr1 | 196 | 2.587 | 0.2127 | Yes |
| 9 | C3ar1 | 205 | 2.531 | 0.2370 | Yes |
| 10 | P2ry2 | 224 | 2.419 | 0.2581 | Yes |
| 11 | Il1b | 240 | 2.351 | 0.2791 | Yes |
| 12 | Ptafr | 284 | 2.159 | 0.2923 | Yes |
| 13 | Cxcl15 | 326 | 1.980 | 0.3040 | Yes |
| 14 | Rgs1 | 350 | 1.874 | 0.3184 | Yes |
| 15 | Pdpn | 369 | 1.788 | 0.3330 | Yes |
| 16 | Lcp2 | 403 | 1.676 | 0.3433 | Yes |
| 17 | Ifitm1 | 427 | 1.619 | 0.3551 | Yes |
| 18 | Itgb8 | 440 | 1.573 | 0.3688 | Yes |
| 19 | Tnfrsf1b | 443 | 1.566 | 0.3844 | Yes |
| 20 | Itga5 | 446 | 1.560 | 0.4000 | Yes |
| 21 | Lamp3 | 497 | 1.453 | 0.4045 | Yes |
| 22 | Irf1 | 508 | 1.429 | 0.4171 | Yes |
| 23 | Emp3 | 537 | 1.365 | 0.4252 | Yes |
| 24 | Irf7 | 617 | 1.174 | 0.4207 | Yes |
| 25 | Tpbg | 632 | 1.133 | 0.4294 | Yes |
| 26 | Hbegf | 684 | 1.029 | 0.4292 | Yes |
| 27 | Slc31a2 | 706 | 0.996 | 0.4350 | Yes |
| 28 | Rnf144b | 732 | 0.962 | 0.4397 | Yes |
| 29 | Lyn | 805 | 0.861 | 0.4334 | Yes |
| 30 | Hif1a | 810 | 0.857 | 0.4413 | Yes |
| 31 | Cdkn1a | 861 | 0.808 | 0.4391 | Yes |
| 32 | Nfkbia | 887 | 0.772 | 0.4418 | Yes |
| 33 | Sema4d | 912 | 0.749 | 0.4444 | Yes |
| 34 | Slc11a2 | 934 | 0.720 | 0.4474 | Yes |
| 35 | Rhog | 961 | 0.693 | 0.4491 | Yes |
| 36 | Nfkb1 | 972 | 0.685 | 0.4540 | Yes |
| 37 | Kcnj2 | 980 | 0.673 | 0.4595 | Yes |
| 38 | Met | 988 | 0.664 | 0.4648 | Yes |
| 39 | P2rx7 | 992 | 0.661 | 0.4710 | Yes |
| 40 | Il1r1 | 1040 | 0.614 | 0.4674 | Yes |
| 41 | Serpine1 | 1049 | 0.610 | 0.4720 | Yes |
| 42 | Icam1 | 1070 | 0.586 | 0.4738 | Yes |
| 43 | Irak2 | 1214 | -0.507 | 0.4490 | No |
| 44 | Il4ra | 1455 | -0.542 | 0.4040 | No |
| 45 | Calcrl | 1468 | -0.546 | 0.4071 | No |
| 46 | Sgms2 | 1510 | -0.552 | 0.4042 | No |
| 47 | Acvr2a | 1588 | -0.565 | 0.3938 | No |
| 48 | F3 | 1925 | -0.622 | 0.3295 | No |
| 49 | Slc7a1 | 1940 | -0.624 | 0.3330 | No |
| 50 | Slc31a1 | 2381 | -0.702 | 0.2476 | No |
| 51 | Pvr | 2520 | -0.731 | 0.2261 | No |
| 52 | Selenos | 2616 | -0.752 | 0.2139 | No |
| 53 | Ly6e | 2739 | -0.775 | 0.1962 | No |
| 54 | P2rx4 | 2770 | -0.785 | 0.1979 | No |
| 55 | Gabbr1 | 3169 | -0.890 | 0.1234 | No |
| 56 | Scn1b | 3498 | -0.996 | 0.0646 | No |
| 57 | Cd82 | 3518 | -1.004 | 0.0709 | No |
| 58 | Ptger4 | 3558 | -1.016 | 0.0732 | No |
| 59 | Sri | 3726 | -1.092 | 0.0493 | No |
| 60 | Sphk1 | 3794 | -1.125 | 0.0467 | No |
| 61 | Btg2 | 4032 | -1.269 | 0.0099 | No |
| 62 | Lpar1 | 4622 | -2.097 | -0.0924 | No |
| 63 | Hpn | 4685 | -2.329 | -0.0815 | No |
| 64 | Tlr2 | 4716 | -2.445 | -0.0627 | No |
| 65 | Ereg | 4731 | -2.541 | -0.0395 | No |
| 66 | Slc4a4 | 4737 | -2.568 | -0.0142 | No |
| 67 | Il18 | 4789 | -3.056 | 0.0065 | No |
Table: GSEA details [plain text format]

  

Fig 2: HALLMARK\_INFLAMMATORY\_RESPONSE: Random ES distribution      
 Gene set null distribution of ES for **HALLMARK\_INFLAMMATORY\_RESPONSE**

  
