# Supplementary material for: TMPRSS11B promotes an acidified microenvironment and immune suppression in squamous lung cancer
Source: EMBO Rep. 2025 Nov 10;26(24):6346–79. doi: 10.1038/s44319-025-00631-1 (PMC12714794; doi:10.1038/s44319-025-00631-1)
Supplement: Supplementary file 14 — Figure EV2 Source Data [file 44319_2025_631_MOESM14_ESM.zip › Figure EV2/EV2D-E/GSEA_Broad Institute_Mh_T11b-high LUSC vs LUAD/HALLMARK_INTERFERON_GAMMA_RESPONSE.html]

Details for gene set HALLMARK\_INTERFERON\_GAMMA\_RESPONSE[GSEA]

|  || Dataset | Ranked list\_DGE\_squamousT11b\_vs\_all adenosadeno\_HSE13-NT copy |
| Phenotype | NoPhenotypeAvailable |
| Upregulated in class | na\_pos |
| GeneSet | HALLMARK\_INTERFERON\_GAMMA\_RESPONSE |
| Enrichment Score (ES) | 0.3418237 |
| Normalized Enrichment Score (NES) | 1.5738689 |
| Nominal p-value | 0.033274956 |
| FDR q-value | 0.05981818 |
| FWER p-Value | 0.582 |
Table: GSEA Results Summary

  

Fig 1: Enrichment plot: HALLMARK\_INTERFERON\_GAMMA\_RESPONSE      
 Profile of the Running ES Score & Positions of GeneSet Members on the Rank Ordered List

  

| SYMBOL | RANK IN GENE LIST | RANK METRIC SCORE | RUNNING ES | CORE ENRICHMENT || 1 | Tnfaip2 | 83 | 3.933 | 0.0435 | Yes |
| 2 | Cd274 | 213 | 2.481 | 0.0548 | Yes |
| 3 | Slamf7 | 257 | 2.290 | 0.0813 | Yes |
| 4 | Pim1 | 319 | 2.011 | 0.0996 | Yes |
| 5 | Eif4e3 | 385 | 1.739 | 0.1129 | Yes |
| 6 | Irf5 | 394 | 1.709 | 0.1377 | Yes |
| 7 | Lcp2 | 403 | 1.676 | 0.1620 | Yes |
| 8 | Irf1 | 508 | 1.429 | 0.1623 | Yes |
| 9 | Oasl1 | 567 | 1.290 | 0.1701 | Yes |
| 10 | Samhd1 | 599 | 1.201 | 0.1822 | Yes |
| 11 | Irf7 | 617 | 1.174 | 0.1968 | Yes |
| 12 | Trim25 | 627 | 1.149 | 0.2127 | Yes |
| 13 | Ifit3 | 703 | 1.003 | 0.2125 | Yes |
| 14 | B2m | 794 | 0.876 | 0.2072 | Yes |
| 15 | Gbp3 | 801 | 0.866 | 0.2193 | Yes |
| 16 | Hif1a | 810 | 0.857 | 0.2309 | Yes |
| 17 | Cd74 | 811 | 0.856 | 0.2442 | Yes |
| 18 | Cdkn1a | 861 | 0.808 | 0.2464 | Yes |
| 19 | Zbp1 | 870 | 0.800 | 0.2571 | Yes |
| 20 | Nfkbia | 887 | 0.772 | 0.2657 | Yes |
| 21 | Parp12 | 909 | 0.751 | 0.2730 | Yes |
| 22 | Nfkb1 | 972 | 0.685 | 0.2706 | Yes |
| 23 | Casp1 | 1000 | 0.649 | 0.2749 | Yes |
| 24 | Fgl2 | 1019 | 0.634 | 0.2810 | Yes |
| 25 | Psmb8 | 1067 | 0.589 | 0.2802 | Yes |
| 26 | Icam1 | 1070 | 0.586 | 0.2889 | Yes |
| 27 | Cfh | 1075 | 0.578 | 0.2970 | Yes |
| 28 | Ciita | 1094 | 0.561 | 0.3019 | Yes |
| 29 | Nlrc5 | 1099 | 0.555 | 0.3097 | Yes |
| 30 | Pnp | 1110 | 0.546 | 0.3160 | Yes |
| 31 | Sod2 | 1120 | 0.540 | 0.3225 | Yes |
| 32 | Ptpn1 | 1126 | 0.534 | 0.3297 | Yes |
| 33 | Upp1 | 1135 | 0.523 | 0.3362 | Yes |
| 34 | Ifnar2 | 1147 | 0.514 | 0.3418 | Yes |
| 35 | Casp3 | 1261 | -0.514 | 0.3260 | No |
| 36 | Stat3 | 1393 | -0.531 | 0.3067 | No |
| 37 | Il4ra | 1455 | -0.542 | 0.3023 | No |
| 38 | Tor1b | 1483 | -0.548 | 0.3051 | No |
| 39 | Ogfr | 1554 | -0.560 | 0.2991 | No |
| 40 | Epsti1 | 1653 | -0.575 | 0.2874 | No |
| 41 | Rapgef6 | 1950 | -0.627 | 0.2349 | No |
| 42 | Cmtr1 | 1980 | -0.632 | 0.2386 | No |
| 43 | Socs3 | 2116 | -0.657 | 0.2204 | No |
| 44 | Pla2g4a | 2549 | -0.736 | 0.1411 | No |
| 45 | Samd9l | 2699 | -0.767 | 0.1216 | No |
| 46 | Ly6e | 2739 | -0.775 | 0.1254 | No |
| 47 | Jak2 | 2891 | -0.813 | 0.1063 | No |
| 48 | Ifi27 | 2905 | -0.815 | 0.1162 | No |
| 49 | Ncoa3 | 3037 | -0.851 | 0.1019 | No |
| 50 | Helz2 | 3044 | -0.853 | 0.1138 | No |
| 51 | Isg20 | 3115 | -0.875 | 0.1127 | No |
| 52 | Rnf213 | 3273 | -0.921 | 0.0940 | No |
| 53 | Usp18 | 3483 | -0.991 | 0.0654 | No |
| 54 | Tdrd7 | 3625 | -1.042 | 0.0519 | No |
| 55 | Adar | 3724 | -1.091 | 0.0482 | No |
| 56 | Sri | 3726 | -1.092 | 0.0649 | No |
| 57 | Cfb | 3801 | -1.129 | 0.0669 | No |
| 58 | Lap3 | 4064 | -1.292 | 0.0318 | No |
| 59 | Bpgm | 4184 | -1.396 | 0.0284 | No |
| 60 | Lgals3bp | 4189 | -1.402 | 0.0493 | No |
| 61 | Auts2 | 4305 | -1.508 | 0.0485 | No |
| 62 | Txnip | 4471 | -1.771 | 0.0413 | No |
| 63 | St3gal5 | 4602 | -2.054 | 0.0458 | No |
Table: GSEA details [plain text format]

  

Fig 2: HALLMARK\_INTERFERON\_GAMMA\_RESPONSE: Random ES distribution      
 Gene set null distribution of ES for **HALLMARK\_INTERFERON\_GAMMA\_RESPONSE**

  
