# Supplementary material for: TMPRSS11B promotes an acidified microenvironment and immune suppression in squamous lung cancer
Source: EMBO Rep. 2025 Nov 10;26(24):6346–79. doi: 10.1038/s44319-025-00631-1 (PMC12714794; doi:10.1038/s44319-025-00631-1)
Supplement: Supplementary file 14 — Figure EV2 Source Data [file 44319_2025_631_MOESM14_ESM.zip › Figure EV2/EV2D-E/GSEA_Broad Institute_Mh_T11b-high LUSC vs LUAD/HALLMARK_KRAS_SIGNALING_DN.html]

Details for gene set HALLMARK\_KRAS\_SIGNALING\_DN[GSEA]

|  || Dataset | Ranked list\_DGE\_squamousT11b\_vs\_all adenosadeno\_HSE13-NT copy |
| Phenotype | NoPhenotypeAvailable |
| Upregulated in class | na\_pos |
| GeneSet | HALLMARK\_KRAS\_SIGNALING\_DN |
| Enrichment Score (ES) | 0.50783885 |
| Normalized Enrichment Score (NES) | 2.1820056 |
| Nominal p-value | 0.0 |
| FDR q-value | 0.0 |
| FWER p-Value | 0.0 |
Table: GSEA Results Summary

  

Fig 1: Enrichment plot: HALLMARK\_KRAS\_SIGNALING\_DN      
 Profile of the Running ES Score & Positions of GeneSet Members on the Rank Ordered List

  

| SYMBOL | RANK IN GENE LIST | RANK METRIC SCORE | RUNNING ES | CORE ENRICHMENT || 1 | Krt13 | 0 | 7.531 | 0.0714 | Yes |
| 2 | Lypd3 | 15 | 6.488 | 0.1299 | Yes |
| 3 | Sprr3 | 21 | 6.151 | 0.1872 | Yes |
| 4 | Tgm1 | 23 | 6.085 | 0.2447 | Yes |
| 5 | Alox12b | 30 | 5.655 | 0.2970 | Yes |
| 6 | Lgals7 | 44 | 4.762 | 0.3394 | Yes |
| 7 | Pkp1 | 57 | 4.358 | 0.3782 | Yes |
| 8 | Krt5 | 58 | 4.353 | 0.4195 | Yes |
| 9 | Serpinb2 | 89 | 3.864 | 0.4498 | Yes |
| 10 | Slc6a14 | 100 | 3.688 | 0.4827 | Yes |
| 11 | Krt4 | 165 | 2.879 | 0.4966 | Yes |
| 12 | Dlk2 | 222 | 2.426 | 0.5078 | Yes |
| 13 | Igfbp2 | 408 | 1.668 | 0.4849 | No |
| 14 | Tgfb2 | 720 | 0.981 | 0.4290 | No |
| 15 | Celsr2 | 721 | 0.981 | 0.4383 | No |
| 16 | Sptbn2 | 924 | 0.734 | 0.4030 | No |
| 17 | Idua | 1581 | -0.564 | 0.2709 | No |
| 18 | Tcf7l1 | 1746 | -0.590 | 0.2422 | No |
| 19 | Mthfr | 1918 | -0.621 | 0.2122 | No |
| 20 | Gtf3c5 | 2090 | -0.652 | 0.1826 | No |
| 21 | Nr6a1 | 2303 | -0.689 | 0.1447 | No |
| 22 | Copz2 | 2325 | -0.693 | 0.1469 | No |
| 23 | Mast3 | 2443 | -0.716 | 0.1292 | No |
| 24 | Cyp39a1 | 2734 | -0.775 | 0.0758 | No |
| 25 | Tent5c | 3132 | -0.880 | 0.0010 | No |
| 26 | Sgk1 | 3151 | -0.885 | 0.0056 | No |
| 27 | Cdkal1 | 3290 | -0.926 | -0.0146 | No |
| 28 | Msh5 | 3592 | -1.029 | -0.0679 | No |
| 29 | Krt15 | 3685 | -1.071 | -0.0770 | No |
| 30 | Thrb | 3839 | -1.151 | -0.0981 | No |
| 31 | Synpo | 3888 | -1.180 | -0.0970 | No |
| 32 | Coq8a | 3956 | -1.218 | -0.0995 | No |
| 33 | Prodh | 3968 | -1.224 | -0.0902 | No |
| 34 | Btg2 | 4032 | -1.269 | -0.0913 | No |
| 35 | Sidt1 | 4350 | -1.575 | -0.1428 | No |
| 36 | Selenop | 4397 | -1.636 | -0.1369 | No |
| 37 | Pdk2 | 4428 | -1.695 | -0.1272 | No |
| 38 | Prkn | 4472 | -1.772 | -0.1194 | No |
| 39 | Nr4a2 | 4493 | -1.801 | -0.1065 | No |
| 40 | Tff2 | 4597 | -2.036 | -0.1088 | No |
| 41 | Hnf1a | 4638 | -2.142 | -0.0968 | No |
| 42 | Rgs11 | 4652 | -2.187 | -0.0788 | No |
| 43 | Myh7 | 4666 | -2.244 | -0.0603 | No |
| 44 | Ntf3 | 4704 | -2.403 | -0.0453 | No |
| 45 | Slc5a5 | 4706 | -2.415 | -0.0226 | No |
| 46 | Tfcp2l1 | 4708 | -2.421 | 0.0002 | No |
| 47 | Gprc5c | 4715 | -2.435 | 0.0220 | No |
Table: GSEA details [plain text format]

  

Fig 2: HALLMARK\_KRAS\_SIGNALING\_DN: Random ES distribution      
 Gene set null distribution of ES for **HALLMARK\_KRAS\_SIGNALING\_DN**

  
