# Supplementary material for: TMPRSS11B promotes an acidified microenvironment and immune suppression in squamous lung cancer
Source: EMBO Rep. 2025 Nov 10;26(24):6346–79. doi: 10.1038/s44319-025-00631-1 (PMC12714794; doi:10.1038/s44319-025-00631-1)
Supplement: Supplementary file 14 — Figure EV2 Source Data [file 44319_2025_631_MOESM14_ESM.zip › Figure EV2/EV2D-E/GSEA_Broad Institute_Mh_T11b-high LUSC vs LUAD/HALLMARK_KRAS_SIGNALING_UP.html]

Details for gene set HALLMARK\_KRAS\_SIGNALING\_UP[GSEA]

|  || Dataset | Ranked list\_DGE\_squamousT11b\_vs\_all adenosadeno\_HSE13-NT copy |
| Phenotype | NoPhenotypeAvailable |
| Upregulated in class | na\_pos |
| GeneSet | HALLMARK\_KRAS\_SIGNALING\_UP |
| Enrichment Score (ES) | 0.4509821 |
| Normalized Enrichment Score (NES) | 2.2347722 |
| Nominal p-value | 0.0 |
| FDR q-value | 0.0 |
| FWER p-Value | 0.0 |
Table: GSEA Results Summary

  

Fig 1: Enrichment plot: HALLMARK\_KRAS\_SIGNALING\_UP      
 Profile of the Running ES Score & Positions of GeneSet Members on the Rank Ordered List

  

| SYMBOL | RANK IN GENE LIST | RANK METRIC SCORE | RUNNING ES | CORE ENRICHMENT || 1 | Arg1 | 14 | 6.570 | 0.0387 | Yes |
| 2 | Ppbp | 28 | 5.754 | 0.0725 | Yes |
| 3 | Aldh1a3 | 48 | 4.574 | 0.0975 | Yes |
| 4 | Gpnmb | 65 | 4.223 | 0.1209 | Yes |
| 5 | Spp1 | 69 | 4.139 | 0.1466 | Yes |
| 6 | Cpe | 77 | 4.024 | 0.1706 | Yes |
| 7 | Emp1 | 79 | 4.007 | 0.1958 | Yes |
| 8 | Mmp9 | 114 | 3.578 | 0.2113 | Yes |
| 9 | Prdm1 | 116 | 3.558 | 0.2337 | Yes |
| 10 | Adam8 | 138 | 3.205 | 0.2496 | Yes |
| 11 | Mall | 155 | 2.983 | 0.2652 | Yes |
| 12 | Itgb2 | 158 | 2.937 | 0.2834 | Yes |
| 13 | Ammecr1 | 175 | 2.774 | 0.2976 | Yes |
| 14 | Plat | 188 | 2.690 | 0.3121 | Yes |
| 15 | C3ar1 | 205 | 2.531 | 0.3248 | Yes |
| 16 | Plau | 210 | 2.513 | 0.3399 | Yes |
| 17 | Slpi | 220 | 2.439 | 0.3535 | Yes |
| 18 | Il1b | 240 | 2.351 | 0.3644 | Yes |
| 19 | Cd37 | 244 | 2.324 | 0.3785 | Yes |
| 20 | Ctss | 247 | 2.317 | 0.3928 | Yes |
| 21 | Reln | 259 | 2.283 | 0.4049 | Yes |
| 22 | Fcer1g | 272 | 2.235 | 0.4166 | Yes |
| 23 | Csf2ra | 299 | 2.108 | 0.4245 | Yes |
| 24 | Mafb | 303 | 2.104 | 0.4372 | Yes |
| 25 | Il1rl2 | 406 | 1.672 | 0.4262 | Yes |
| 26 | Tnfrsf1b | 443 | 1.566 | 0.4285 | Yes |
| 27 | Klf4 | 448 | 1.555 | 0.4375 | Yes |
| 28 | Dock2 | 467 | 1.518 | 0.4434 | Yes |
| 29 | Lat2 | 477 | 1.499 | 0.4510 | Yes |
| 30 | Ppp1r15a | 543 | 1.345 | 0.4458 | No |
| 31 | Jup | 565 | 1.293 | 0.4495 | No |
| 32 | Il2rg | 648 | 1.091 | 0.4391 | No |
| 33 | Hbegf | 684 | 1.029 | 0.4382 | No |
| 34 | Mmd | 709 | 0.991 | 0.4394 | No |
| 35 | Birc3 | 745 | 0.942 | 0.4380 | No |
| 36 | Lcp1 | 849 | 0.813 | 0.4214 | No |
| 37 | Ets1 | 863 | 0.807 | 0.4238 | No |
| 38 | Cbx8 | 883 | 0.780 | 0.4247 | No |
| 39 | Etv5 | 917 | 0.743 | 0.4224 | No |
| 40 | Psmb8 | 1067 | 0.589 | 0.3946 | No |
| 41 | Mpzl2 | 1071 | 0.586 | 0.3977 | No |
| 42 | Cfh | 1075 | 0.578 | 0.4008 | No |
| 43 | Trib1 | 1112 | 0.544 | 0.3966 | No |
| 44 | Gprc5b | 1161 | 0.504 | 0.3896 | No |
| 45 | Gng11 | 1167 | 0.502 | 0.3918 | No |
| 46 | Car2 | 1171 | -0.500 | 0.3943 | No |
| 47 | Ptcd2 | 1187 | -0.503 | 0.3943 | No |
| 48 | Ccser2 | 1267 | -0.515 | 0.3809 | No |
| 49 | Tmem176a | 1324 | -0.522 | 0.3724 | No |
| 50 | Tmem176b | 1326 | -0.522 | 0.3755 | No |
| 51 | Akt2 | 1349 | -0.525 | 0.3741 | No |
| 52 | Adgra2 | 1366 | -0.529 | 0.3741 | No |
| 53 | Hsd11b1 | 1459 | -0.543 | 0.3581 | No |
| 54 | Sdccag8 | 1612 | -0.571 | 0.3296 | No |
| 55 | Cbr4 | 1696 | -0.583 | 0.3157 | No |
| 56 | Fuca1 | 1814 | -0.603 | 0.2948 | No |
| 57 | Ptbp2 | 1829 | -0.605 | 0.2957 | No |
| 58 | Kcnn4 | 2017 | -0.638 | 0.2601 | No |
| 59 | Vwa5a | 2066 | -0.646 | 0.2541 | No |
| 60 | Wdr33 | 2180 | -0.666 | 0.2344 | No |
| 61 | Fbxo4 | 2374 | -0.701 | 0.1980 | No |
| 62 | Usp12 | 2777 | -0.786 | 0.1180 | No |
| 63 | Rbm4 | 2823 | -0.795 | 0.1135 | No |
| 64 | Laptm5 | 2847 | -0.801 | 0.1137 | No |
| 65 | Galnt3 | 3118 | -0.876 | 0.0622 | No |
| 66 | Il33 | 3253 | -0.916 | 0.0396 | No |
| 67 | Zfp277 | 3310 | -0.934 | 0.0337 | No |
| 68 | Cab39l | 3465 | -0.985 | 0.0074 | No |
| 69 | Scn1b | 3498 | -0.996 | 0.0069 | No |
| 70 | Gadd45g | 3538 | -1.011 | 0.0051 | No |
| 71 | Traf1 | 3786 | -1.119 | -0.0401 | No |
| 72 | Cfb | 3801 | -1.129 | -0.0359 | No |
| 73 | Pigr | 3833 | -1.149 | -0.0351 | No |
| 74 | Trib2 | 3842 | -1.153 | -0.0295 | No |
| 75 | Hkdc1 | 3877 | -1.168 | -0.0293 | No |
| 76 | Spry2 | 3925 | -1.200 | -0.0316 | No |
| 77 | Etv4 | 4030 | -1.268 | -0.0456 | No |
| 78 | Avl9 | 4056 | -1.285 | -0.0427 | No |
| 79 | Bmp2 | 4098 | -1.322 | -0.0430 | No |
| 80 | Tspan13 | 4111 | -1.336 | -0.0370 | No |
| 81 | Plek2 | 4126 | -1.352 | -0.0314 | No |
| 82 | Bpgm | 4184 | -1.396 | -0.0346 | No |
| 83 | Tmem158 | 4387 | -1.624 | -0.0670 | No |
| 84 | Sox9 | 4412 | -1.664 | -0.0616 | No |
| 85 | Adamdec1 | 4451 | -1.746 | -0.0585 | No |
| 86 | Rabgap1l | 4459 | -1.754 | -0.0489 | No |
| 87 | Itgbl1 | 4539 | -1.889 | -0.0536 | No |
| 88 | Ano1 | 4593 | -2.031 | -0.0519 | No |
| 89 | Ccnd2 | 4672 | -2.265 | -0.0540 | No |
| 90 | F2rl1 | 4690 | -2.349 | -0.0427 | No |
| 91 | Anxa10 | 4718 | -2.457 | -0.0328 | No |
| 92 | Ereg | 4731 | -2.541 | -0.0193 | No |
| 93 | Mycn | 4765 | -2.740 | -0.0089 | No |
| 94 | Sema3b | 4798 | -3.196 | 0.0047 | No |
Table: GSEA details [plain text format]

  

Fig 2: HALLMARK\_KRAS\_SIGNALING\_UP: Random ES distribution      
 Gene set null distribution of ES for **HALLMARK\_KRAS\_SIGNALING\_UP**

  
