# Supplementary material for: TMPRSS11B promotes an acidified microenvironment and immune suppression in squamous lung cancer
Source: EMBO Rep. 2025 Nov 10;26(24):6346–79. doi: 10.1038/s44319-025-00631-1 (PMC12714794; doi:10.1038/s44319-025-00631-1)
Supplement: Supplementary file 14 — Figure EV2 Source Data [file 44319_2025_631_MOESM14_ESM.zip › Figure EV2/EV2D-E/GSEA_Broad Institute_Mh_T11b-high LUSC vs LUAD/HALLMARK_MITOTIC_SPINDLE.html]

Details for gene set HALLMARK\_MITOTIC\_SPINDLE[GSEA]

|  || Dataset | Ranked list\_DGE\_squamousT11b\_vs\_all adenosadeno\_HSE13-NT copy |
| Phenotype | NoPhenotypeAvailable |
| Upregulated in class | na\_neg |
| GeneSet | HALLMARK\_MITOTIC\_SPINDLE |
| Enrichment Score (ES) | -0.12169459 |
| Normalized Enrichment Score (NES) | -0.63375777 |
| Nominal p-value | 0.95620435 |
| FDR q-value | 1.0 |
| FWER p-Value | 1.0 |
Table: GSEA Results Summary

  

Fig 1: Enrichment plot: HALLMARK\_MITOTIC\_SPINDLE      
 Profile of the Running ES Score & Positions of GeneSet Members on the Rank Ordered List

  

| SYMBOL | RANK IN GENE LIST | RANK METRIC SCORE | RUNNING ES | CORE ENRICHMENT || 1 | Tiam1 | 334 | 1.949 | -0.0382 | No |
| 2 | Dst | 434 | 1.596 | -0.0328 | No |
| 3 | Fscn1 | 444 | 1.564 | -0.0090 | No |
| 4 | Dock2 | 467 | 1.518 | 0.0113 | No |
| 5 | Sun2 | 598 | 1.204 | 0.0038 | No |
| 6 | Net1 | 654 | 1.078 | 0.0099 | No |
| 7 | Prex1 | 783 | 0.884 | -0.0025 | No |
| 8 | Cdk1 | 820 | 0.842 | 0.0038 | No |
| 9 | Flna | 905 | 0.755 | -0.0014 | No |
| 10 | Nusap1 | 907 | 0.752 | 0.0107 | No |
| 11 | Palld | 927 | 0.725 | 0.0186 | No |
| 12 | Incenp | 937 | 0.718 | 0.0285 | No |
| 13 | Racgap1 | 968 | 0.689 | 0.0335 | No |
| 14 | Arhgap10 | 995 | 0.658 | 0.0389 | No |
| 15 | Arhgap27 | 1048 | 0.610 | 0.0380 | No |
| 16 | Smc4 | 1134 | 0.523 | 0.0287 | No |
| 17 | Smc1a | 1189 | -0.503 | 0.0256 | No |
| 18 | Rabgap1 | 1282 | -0.516 | 0.0148 | No |
| 19 | Cep250 | 1283 | -0.516 | 0.0232 | No |
| 20 | Ckap5 | 1332 | -0.522 | 0.0217 | No |
| 21 | Abl1 | 1424 | -0.536 | 0.0114 | No |
| 22 | Bin1 | 1559 | -0.561 | -0.0076 | No |
| 23 | Cntrl | 1564 | -0.562 | 0.0008 | No |
| 24 | Hdac6 | 1793 | -0.598 | -0.0373 | No |
| 25 | Tlk1 | 1811 | -0.603 | -0.0309 | No |
| 26 | Map1s | 1830 | -0.605 | -0.0248 | No |
| 27 | Shroom2 | 1911 | -0.620 | -0.0314 | No |
| 28 | Dlg1 | 1932 | -0.623 | -0.0254 | No |
| 29 | Rapgef6 | 1950 | -0.627 | -0.0187 | No |
| 30 | Sos1 | 1961 | -0.629 | -0.0104 | No |
| 31 | Rock1 | 1986 | -0.633 | -0.0051 | No |
| 32 | Pcnt | 2095 | -0.653 | -0.0170 | No |
| 33 | Smc3 | 2109 | -0.655 | -0.0090 | No |
| 34 | Trio | 2167 | -0.665 | -0.0101 | No |
| 35 | Nf1 | 2379 | -0.701 | -0.0429 | No |
| 36 | Numa1 | 2406 | -0.707 | -0.0368 | No |
| 37 | Tubgcp3 | 2410 | -0.707 | -0.0258 | No |
| 38 | Stau1 | 2415 | -0.709 | -0.0150 | No |
| 39 | Pkd2 | 2458 | -0.719 | -0.0120 | No |
| 40 | Itsn1 | 2604 | -0.749 | -0.0302 | No |
| 41 | Arfgef1 | 2702 | -0.767 | -0.0380 | No |
| 42 | Kif5b | 2964 | -0.830 | -0.0792 | No |
| 43 | Cd2ap | 3167 | -0.889 | -0.1071 | Yes |
| 44 | Tsc1 | 3220 | -0.905 | -0.1031 | Yes |
| 45 | Plekhg2 | 3242 | -0.911 | -0.0926 | Yes |
| 46 | Kifap3 | 3266 | -0.920 | -0.0823 | Yes |
| 47 | Ppp4r2 | 3390 | -0.958 | -0.0924 | Yes |
| 48 | Cttn | 3403 | -0.961 | -0.0792 | Yes |
| 49 | Bcar1 | 3434 | -0.976 | -0.0694 | Yes |
| 50 | Rasa2 | 3497 | -0.995 | -0.0661 | Yes |
| 51 | Apc | 3622 | -1.041 | -0.0751 | Yes |
| 52 | Atg4b | 3634 | -1.045 | -0.0602 | Yes |
| 53 | Pcgf5 | 3669 | -1.063 | -0.0499 | Yes |
| 54 | Ezr | 3702 | -1.081 | -0.0389 | Yes |
| 55 | Arfip2 | 3827 | -1.141 | -0.0462 | Yes |
| 56 | Cdc42ep1 | 3847 | -1.155 | -0.0312 | Yes |
| 57 | Synpo | 3888 | -1.180 | -0.0202 | Yes |
| 58 | Dynll2 | 3904 | -1.185 | -0.0039 | Yes |
| 59 | Mid1ip1 | 3910 | -1.190 | 0.0146 | Yes |
| 60 | Nck2 | 3935 | -1.205 | 0.0294 | Yes |
| 61 | Pcm1 | 4017 | -1.256 | 0.0330 | Yes |
| 62 | Kif23 | 4104 | -1.332 | 0.0368 | Yes |
| 63 | Sac3d1 | 4161 | -1.380 | 0.0477 | Yes |
| 64 | Nedd9 | 4238 | -1.453 | 0.0556 | Yes |
| 65 | Flnb | 4329 | -1.541 | 0.0620 | Yes |
| 66 | Sorbs2 | 4727 | -2.500 | 0.0196 | Yes |
Table: GSEA details [plain text format]

  

Fig 2: HALLMARK\_MITOTIC\_SPINDLE: Random ES distribution      
 Gene set null distribution of ES for **HALLMARK\_MITOTIC\_SPINDLE**

  
