# Supplementary material for: TMPRSS11B promotes an acidified microenvironment and immune suppression in squamous lung cancer
Source: EMBO Rep. 2025 Nov 10;26(24):6346–79. doi: 10.1038/s44319-025-00631-1 (PMC12714794; doi:10.1038/s44319-025-00631-1)
Supplement: Supplementary file 14 — Figure EV2 Source Data [file 44319_2025_631_MOESM14_ESM.zip › Figure EV2/EV2D-E/GSEA_Broad Institute_Mh_T11b-high LUSC vs LUAD/HALLMARK_MTORC1_SIGNALING.html]

Details for gene set HALLMARK\_MTORC1\_SIGNALING[GSEA]

|  || Dataset | Ranked list\_DGE\_squamousT11b\_vs\_all adenosadeno\_HSE13-NT copy |
| Phenotype | NoPhenotypeAvailable |
| Upregulated in class | na\_pos |
| GeneSet | HALLMARK\_MTORC1\_SIGNALING |
| Enrichment Score (ES) | 0.34521636 |
| Normalized Enrichment Score (NES) | 1.5792516 |
| Nominal p-value | 0.036842104 |
| FDR q-value | 0.0612662 |
| FWER p-Value | 0.565 |
Table: GSEA Results Summary

  

Fig 1: Enrichment plot: HALLMARK\_MTORC1\_SIGNALING      
 Profile of the Running ES Score & Positions of GeneSet Members on the Rank Ordered List

  

| SYMBOL | RANK IN GENE LIST | RANK METRIC SCORE | RUNNING ES | CORE ENRICHMENT || 1 | Itgb2 | 158 | 2.937 | 0.0190 | Yes |
| 2 | Slc7a11 | 277 | 2.198 | 0.0333 | Yes |
| 3 | Hk2 | 428 | 1.617 | 0.0306 | Yes |
| 4 | Cfp | 433 | 1.599 | 0.0581 | Yes |
| 5 | Lgmn | 458 | 1.534 | 0.0803 | Yes |
| 6 | Fads2 | 462 | 1.527 | 0.1068 | Yes |
| 7 | Gla | 482 | 1.492 | 0.1294 | Yes |
| 8 | Sla | 486 | 1.480 | 0.1550 | Yes |
| 9 | Ppp1r15a | 543 | 1.345 | 0.1671 | Yes |
| 10 | Coro1a | 546 | 1.340 | 0.1905 | Yes |
| 11 | Fads1 | 568 | 1.289 | 0.2090 | Yes |
| 12 | Tfrc | 646 | 1.096 | 0.2123 | Yes |
| 13 | Ifrd1 | 671 | 1.048 | 0.2259 | Yes |
| 14 | Map2k3 | 691 | 1.015 | 0.2400 | Yes |
| 15 | Ctsc | 770 | 0.907 | 0.2397 | Yes |
| 16 | Cdkn1a | 861 | 0.808 | 0.2352 | Yes |
| 17 | G6pdx | 869 | 0.804 | 0.2480 | Yes |
| 18 | Elovl5 | 882 | 0.781 | 0.2593 | Yes |
| 19 | Shmt2 | 886 | 0.772 | 0.2724 | Yes |
| 20 | Mllt11 | 944 | 0.710 | 0.2731 | Yes |
| 21 | Psat1 | 954 | 0.702 | 0.2837 | Yes |
| 22 | Tm7sf2 | 1017 | 0.635 | 0.2819 | Yes |
| 23 | Fgl2 | 1019 | 0.634 | 0.2930 | Yes |
| 24 | Pgk1 | 1029 | 0.624 | 0.3022 | Yes |
| 25 | Egln3 | 1032 | 0.623 | 0.3128 | Yes |
| 26 | Ddit3 | 1038 | 0.616 | 0.3227 | Yes |
| 27 | Txnrd1 | 1081 | 0.571 | 0.3240 | Yes |
| 28 | Nupr1 | 1092 | 0.562 | 0.3319 | Yes |
| 29 | Pnp | 1110 | 0.546 | 0.3381 | Yes |
| 30 | Sord | 1146 | 0.514 | 0.3398 | Yes |
| 31 | Edem1 | 1164 | 0.503 | 0.3452 | Yes |
| 32 | Nup205 | 1497 | -0.551 | 0.2854 | No |
| 33 | Qdpr | 1949 | -0.627 | 0.2019 | No |
| 34 | Tbk1 | 1954 | -0.627 | 0.2122 | No |
| 35 | Rpa1 | 2092 | -0.652 | 0.1951 | No |
| 36 | Adipor2 | 2276 | -0.684 | 0.1689 | No |
| 37 | Ppa1 | 2690 | -0.766 | 0.0959 | No |
| 38 | Add3 | 2899 | -0.814 | 0.0667 | No |
| 39 | Fdxr | 2916 | -0.817 | 0.0779 | No |
| 40 | Nufip1 | 3087 | -0.867 | 0.0576 | No |
| 41 | Idh1 | 3183 | -0.894 | 0.0536 | No |
| 42 | Got1 | 3326 | -0.939 | 0.0404 | No |
| 43 | Slc1a5 | 3499 | -0.997 | 0.0221 | No |
| 44 | Sc5d | 3560 | -1.017 | 0.0276 | No |
| 45 | Immt | 3616 | -1.038 | 0.0345 | No |
| 46 | Xbp1 | 3997 | -1.246 | -0.0231 | No |
| 47 | Asns | 4004 | -1.251 | -0.0021 | No |
| 48 | Cdc25a | 4011 | -1.255 | 0.0189 | No |
| 49 | Btg2 | 4032 | -1.269 | 0.0372 | No |
| 50 | Sytl2 | 4596 | -2.034 | -0.0447 | No |
| 51 | Bcat1 | 4607 | -2.061 | -0.0102 | No |
| 52 | Igfbp5 | 4791 | -3.078 | 0.0061 | No |
Table: GSEA details [plain text format]

  

Fig 2: HALLMARK\_MTORC1\_SIGNALING: Random ES distribution      
 Gene set null distribution of ES for **HALLMARK\_MTORC1\_SIGNALING**

  
