# Supplementary material for: TMPRSS11B promotes an acidified microenvironment and immune suppression in squamous lung cancer
Source: EMBO Rep. 2025 Nov 10;26(24):6346–79. doi: 10.1038/s44319-025-00631-1 (PMC12714794; doi:10.1038/s44319-025-00631-1)
Supplement: Supplementary file 14 — Figure EV2 Source Data [file 44319_2025_631_MOESM14_ESM.zip › Figure EV2/EV2D-E/GSEA_Broad Institute_Mh_T11b-high LUSC vs LUAD/HALLMARK_MYOGENESIS.html]

Details for gene set HALLMARK\_MYOGENESIS[GSEA]

|  || Dataset | Ranked list\_DGE\_squamousT11b\_vs\_all adenosadeno\_HSE13-NT copy |
| Phenotype | NoPhenotypeAvailable |
| Upregulated in class | na\_neg |
| GeneSet | HALLMARK\_MYOGENESIS |
| Enrichment Score (ES) | -0.29005656 |
| Normalized Enrichment Score (NES) | -1.4819425 |
| Nominal p-value | 0.040767387 |
| FDR q-value | 0.56162286 |
| FWER p-Value | 0.564 |
Table: GSEA Results Summary

  

Fig 1: Enrichment plot: HALLMARK\_MYOGENESIS      
 Profile of the Running ES Score & Positions of GeneSet Members on the Rank Ordered List

  

| SYMBOL | RANK IN GENE LIST | RANK METRIC SCORE | RUNNING ES | CORE ENRICHMENT || 1 | Cd36 | 199 | 2.567 | -0.0080 | No |
| 2 | Nqo1 | 301 | 2.106 | -0.0015 | No |
| 3 | Tpm2 | 308 | 2.054 | 0.0244 | No |
| 4 | Ckb | 372 | 1.769 | 0.0344 | No |
| 5 | Sorbs1 | 389 | 1.734 | 0.0539 | No |
| 6 | Dapk2 | 593 | 1.218 | 0.0273 | No |
| 7 | Notch1 | 644 | 1.101 | 0.0313 | No |
| 8 | Ifrd1 | 671 | 1.048 | 0.0397 | No |
| 9 | Hbegf | 684 | 1.029 | 0.0507 | No |
| 10 | Wwtr1 | 730 | 0.963 | 0.0540 | No |
| 11 | Cryab | 800 | 0.869 | 0.0509 | No |
| 12 | Gadd45b | 804 | 0.862 | 0.0617 | No |
| 13 | Prnp | 806 | 0.860 | 0.0728 | No |
| 14 | Cdkn1a | 861 | 0.808 | 0.0721 | No |
| 15 | Tgfb1 | 940 | 0.718 | 0.0652 | No |
| 16 | Igf1 | 978 | 0.674 | 0.0663 | No |
| 17 | Lsp1 | 1023 | 0.629 | 0.0653 | No |
| 18 | Col3a1 | 1034 | 0.619 | 0.0714 | No |
| 19 | Slc6a8 | 1042 | 0.611 | 0.0780 | No |
| 20 | Kifc3 | 1046 | 0.611 | 0.0854 | No |
| 21 | Erbb3 | 1278 | -0.516 | 0.0436 | No |
| 22 | Mapre3 | 1300 | -0.519 | 0.0460 | No |
| 23 | Itgb5 | 1316 | -0.520 | 0.0498 | No |
| 24 | Akt2 | 1349 | -0.525 | 0.0499 | No |
| 25 | App | 1359 | -0.528 | 0.0550 | No |
| 26 | Itgb1 | 1391 | -0.531 | 0.0555 | No |
| 27 | Bin1 | 1559 | -0.561 | 0.0278 | No |
| 28 | Gaa | 1605 | -0.569 | 0.0258 | No |
| 29 | Pcx | 1619 | -0.571 | 0.0306 | No |
| 30 | Col6a3 | 2117 | -0.657 | -0.0652 | No |
| 31 | Eif4a2 | 2128 | -0.659 | -0.0586 | No |
| 32 | Tead4 | 2176 | -0.666 | -0.0597 | No |
| 33 | Ephb3 | 2258 | -0.682 | -0.0677 | No |
| 34 | Mapk12 | 2273 | -0.684 | -0.0617 | No |
| 35 | Mras | 2285 | -0.685 | -0.0550 | No |
| 36 | Agrn | 2286 | -0.685 | -0.0459 | No |
| 37 | Pfkm | 2427 | -0.712 | -0.0660 | No |
| 38 | Sh2b1 | 2460 | -0.719 | -0.0632 | No |
| 39 | Fhl1 | 2670 | -0.762 | -0.0971 | No |
| 40 | Fgf2 | 2942 | -0.823 | -0.1432 | No |
| 41 | Adcy9 | 3086 | -0.866 | -0.1619 | No |
| 42 | Pde4dip | 3155 | -0.886 | -0.1645 | No |
| 43 | Itgb4 | 3162 | -0.888 | -0.1540 | No |
| 44 | Acsl1 | 3337 | -0.941 | -0.1782 | No |
| 45 | Col15a1 | 3582 | -1.026 | -0.2160 | No |
| 46 | Pick1 | 3793 | -1.123 | -0.2453 | No |
| 47 | Sphk1 | 3794 | -1.125 | -0.2305 | No |
| 48 | Bag1 | 3870 | -1.165 | -0.2309 | No |
| 49 | Eno3 | 3938 | -1.207 | -0.2290 | No |
| 50 | Hdac5 | 4145 | -1.366 | -0.2543 | No |
| 51 | Foxo4 | 4316 | -1.523 | -0.2700 | Yes |
| 52 | Ptgis | 4321 | -1.530 | -0.2506 | Yes |
| 53 | Itga7 | 4326 | -1.538 | -0.2312 | Yes |
| 54 | Spdef | 4411 | -1.663 | -0.2269 | Yes |
| 55 | Sorbs3 | 4439 | -1.719 | -0.2099 | Yes |
| 56 | Nav2 | 4458 | -1.753 | -0.1906 | Yes |
| 57 | Klf5 | 4543 | -1.893 | -0.1833 | Yes |
| 58 | Tnni2 | 4562 | -1.945 | -0.1615 | Yes |
| 59 | Stc2 | 4564 | -1.949 | -0.1360 | Yes |
| 60 | Cdh13 | 4647 | -2.165 | -0.1247 | Yes |
| 61 | Myh7 | 4666 | -2.244 | -0.0989 | Yes |
| 62 | Dmd | 4759 | -2.718 | -0.0824 | Yes |
| 63 | Clu | 4775 | -2.816 | -0.0484 | Yes |
| 64 | Sgcd | 4820 | -4.373 | -0.0000 | Yes |
Table: GSEA details [plain text format]

  

Fig 2: HALLMARK\_MYOGENESIS: Random ES distribution      
 Gene set null distribution of ES for **HALLMARK\_MYOGENESIS**

  
