# Supplementary material for: TMPRSS11B promotes an acidified microenvironment and immune suppression in squamous lung cancer
Source: EMBO Rep. 2025 Nov 10;26(24):6346–79. doi: 10.1038/s44319-025-00631-1 (PMC12714794; doi:10.1038/s44319-025-00631-1)
Supplement: Supplementary file 14 — Figure EV2 Source Data [file 44319_2025_631_MOESM14_ESM.zip › Figure EV2/EV2D-E/GSEA_Broad Institute_Mh_T11b-high LUSC vs LUAD/HALLMARK_OXIDATIVE_PHOSPHORYLATION.html]

Details for gene set HALLMARK\_OXIDATIVE\_PHOSPHORYLATION[GSEA]

|  || Dataset | Ranked list\_DGE\_squamousT11b\_vs\_all adenosadeno\_HSE13-NT copy |
| Phenotype | NoPhenotypeAvailable |
| Upregulated in class | na\_neg |
| GeneSet | HALLMARK\_OXIDATIVE\_PHOSPHORYLATION |
| Enrichment Score (ES) | -0.18786259 |
| Normalized Enrichment Score (NES) | -0.91215366 |
| Nominal p-value | 0.5952381 |
| FDR q-value | 1.0 |
| FWER p-Value | 1.0 |
Table: GSEA Results Summary

  

Fig 1: Enrichment plot: HALLMARK\_OXIDATIVE\_PHOSPHORYLATION      
 Profile of the Running ES Score & Positions of GeneSet Members on the Rank Ordered List

  

| SYMBOL | RANK IN GENE LIST | RANK METRIC SCORE | RUNNING ES | CORE ENRICHMENT || 1 | Tcirg1 | 638 | 1.112 | -0.1066 | No |
| 2 | Atp6v1e1 | 877 | 0.790 | -0.1372 | No |
| 3 | Atp6v0e | 942 | 0.714 | -0.1333 | No |
| 4 | Fdx1 | 1106 | 0.548 | -0.1541 | No |
| 5 | Atp6v1g1 | 1116 | 0.543 | -0.1428 | No |
| 6 | Ndufb6 | 1150 | 0.512 | -0.1372 | No |
| 7 | Acaa1a | 1177 | -0.501 | -0.1305 | No |
| 8 | Cox15 | 1286 | -0.517 | -0.1405 | No |
| 9 | Ndufv2 | 1437 | -0.539 | -0.1588 | No |
| 10 | Rhot1 | 1458 | -0.543 | -0.1498 | No |
| 11 | Ndufs4 | 1608 | -0.570 | -0.1672 | No |
| 12 | Cyb5r3 | 1675 | -0.579 | -0.1669 | No |
| 13 | Suclg1 | 1762 | -0.593 | -0.1705 | No |
| 14 | Acaa2 | 1846 | -0.609 | -0.1730 | Yes |
| 15 | Vdac3 | 1895 | -0.618 | -0.1681 | Yes |
| 16 | Por | 1937 | -0.624 | -0.1615 | Yes |
| 17 | Cyb5a | 1942 | -0.625 | -0.1471 | Yes |
| 18 | Abcb7 | 1977 | -0.631 | -0.1389 | Yes |
| 19 | Ndufs2 | 2103 | -0.655 | -0.1491 | Yes |
| 20 | Cpt1a | 2123 | -0.658 | -0.1371 | Yes |
| 21 | Acat1 | 2253 | -0.681 | -0.1476 | Yes |
| 22 | Ndufa7 | 2344 | -0.696 | -0.1495 | Yes |
| 23 | Idh3g | 2455 | -0.719 | -0.1550 | Yes |
| 24 | Idh2 | 2529 | -0.733 | -0.1525 | Yes |
| 25 | Sucla2 | 2603 | -0.749 | -0.1496 | Yes |
| 26 | Ndufa9 | 2646 | -0.758 | -0.1399 | Yes |
| 27 | Bckdha | 2848 | -0.802 | -0.1625 | Yes |
| 28 | Acadsb | 2851 | -0.803 | -0.1434 | Yes |
| 29 | Sdha | 2908 | -0.816 | -0.1353 | Yes |
| 30 | Timm8b | 2968 | -0.831 | -0.1274 | Yes |
| 31 | Fh1 | 2972 | -0.834 | -0.1078 | Yes |
| 32 | Idh3b | 3079 | -0.865 | -0.1089 | Yes |
| 33 | Hsd17b10 | 3144 | -0.883 | -0.1009 | Yes |
| 34 | Idh1 | 3183 | -0.894 | -0.0871 | Yes |
| 35 | Ndufb8 | 3274 | -0.922 | -0.0835 | Yes |
| 36 | Surf1 | 3279 | -0.924 | -0.0619 | Yes |
| 37 | Sdhd | 3399 | -0.960 | -0.0634 | Yes |
| 38 | Immt | 3616 | -1.038 | -0.0834 | Yes |
| 39 | Oxa1l | 3651 | -1.054 | -0.0649 | Yes |
| 40 | Decr1 | 3683 | -1.070 | -0.0454 | Yes |
| 41 | Ech1 | 3705 | -1.083 | -0.0234 | Yes |
| 42 | Cox17 | 3749 | -1.101 | -0.0056 | Yes |
| 43 | Hadha | 3788 | -1.119 | 0.0136 | Yes |
| 44 | Acadm | 3866 | -1.164 | 0.0258 | Yes |
| 45 | Atp1b1 | 4241 | -1.458 | -0.0170 | Yes |
| 46 | Aldh6a1 | 4345 | -1.570 | -0.0004 | Yes |
| 47 | Phyh | 4469 | -1.768 | 0.0168 | Yes |
| 48 | Oat | 4683 | -2.321 | 0.0287 | Yes |
Table: GSEA details [plain text format]

  

Fig 2: HALLMARK\_OXIDATIVE\_PHOSPHORYLATION: Random ES distribution      
 Gene set null distribution of ES for **HALLMARK\_OXIDATIVE\_PHOSPHORYLATION**

  
