# Supplementary material for: TMPRSS11B promotes an acidified microenvironment and immune suppression in squamous lung cancer
Source: EMBO Rep. 2025 Nov 10;26(24):6346–79. doi: 10.1038/s44319-025-00631-1 (PMC12714794; doi:10.1038/s44319-025-00631-1)
Supplement: Supplementary file 14 — Figure EV2 Source Data [file 44319_2025_631_MOESM14_ESM.zip › Figure EV2/EV2D-E/GSEA_Broad Institute_Mh_T11b-high LUSC vs LUAD/HALLMARK_P53_PATHWAY.html]

Details for gene set HALLMARK\_P53\_PATHWAY[GSEA]

|  || Dataset | Ranked list\_DGE\_squamousT11b\_vs\_all adenosadeno\_HSE13-NT copy |
| Phenotype | NoPhenotypeAvailable |
| Upregulated in class | na\_pos |
| GeneSet | HALLMARK\_P53\_PATHWAY |
| Enrichment Score (ES) | 0.39051232 |
| Normalized Enrichment Score (NES) | 1.9800013 |
| Nominal p-value | 0.0 |
| FDR q-value | 0.0042050895 |
| FWER p-Value | 0.024 |
Table: GSEA Results Summary

  

Fig 1: Enrichment plot: HALLMARK\_P53\_PATHWAY      
 Profile of the Running ES Score & Positions of GeneSet Members on the Rank Ordered List

  

| SYMBOL | RANK IN GENE LIST | RANK METRIC SCORE | RUNNING ES | CORE ENRICHMENT || 1 | Krt17 | 34 | 5.250 | 0.0356 | Yes |
| 2 | Clca2 | 53 | 4.454 | 0.0681 | Yes |
| 3 | Il1a | 119 | 3.497 | 0.0829 | Yes |
| 4 | Hmox1 | 144 | 3.102 | 0.1031 | Yes |
| 5 | Mxd1 | 163 | 2.892 | 0.1229 | Yes |
| 6 | Ada | 171 | 2.823 | 0.1444 | Yes |
| 7 | Trp63 | 189 | 2.685 | 0.1627 | Yes |
| 8 | S100a10 | 263 | 2.263 | 0.1657 | Yes |
| 9 | Cdkn2b | 266 | 2.248 | 0.1836 | Yes |
| 10 | Ctsd | 274 | 2.219 | 0.2002 | Yes |
| 11 | Slc7a11 | 277 | 2.198 | 0.2177 | Yes |
| 12 | Epha2 | 286 | 2.147 | 0.2335 | Yes |
| 13 | Perp | 322 | 1.994 | 0.2424 | Yes |
| 14 | S100a4 | 353 | 1.867 | 0.2512 | Yes |
| 15 | Plk3 | 392 | 1.721 | 0.2572 | Yes |
| 16 | Rap2b | 398 | 1.689 | 0.2699 | Yes |
| 17 | Def6 | 418 | 1.629 | 0.2792 | Yes |
| 18 | Klf4 | 448 | 1.555 | 0.2857 | Yes |
| 19 | Procr | 451 | 1.548 | 0.2979 | Yes |
| 20 | Dram1 | 494 | 1.458 | 0.3009 | Yes |
| 21 | Jag2 | 498 | 1.452 | 0.3121 | Yes |
| 22 | Eps8l2 | 535 | 1.367 | 0.3157 | Yes |
| 23 | Ppp1r15a | 543 | 1.345 | 0.3252 | Yes |
| 24 | Ier5 | 609 | 1.186 | 0.3211 | Yes |
| 25 | Sat1 | 614 | 1.180 | 0.3299 | Yes |
| 26 | Atf3 | 643 | 1.106 | 0.3329 | Yes |
| 27 | Notch1 | 644 | 1.101 | 0.3419 | Yes |
| 28 | Osgin1 | 663 | 1.064 | 0.3468 | Yes |
| 29 | Stom | 675 | 1.042 | 0.3530 | Yes |
| 30 | Ndrg1 | 681 | 1.033 | 0.3603 | Yes |
| 31 | Hbegf | 684 | 1.029 | 0.3683 | Yes |
| 32 | Hras | 719 | 0.981 | 0.3691 | Yes |
| 33 | Serpinb5 | 728 | 0.969 | 0.3753 | Yes |
| 34 | Phlda3 | 738 | 0.954 | 0.3812 | Yes |
| 35 | Gm2a | 789 | 0.881 | 0.3778 | Yes |
| 36 | Tgfa | 793 | 0.877 | 0.3843 | Yes |
| 37 | Ninj1 | 833 | 0.831 | 0.3828 | Yes |
| 38 | Fam162a | 858 | 0.810 | 0.3844 | Yes |
| 39 | Cdkn1a | 861 | 0.808 | 0.3905 | Yes |
| 40 | Tgfb1 | 940 | 0.718 | 0.3799 | No |
| 41 | Ier3 | 967 | 0.691 | 0.3800 | No |
| 42 | Dgka | 986 | 0.667 | 0.3816 | No |
| 43 | Casp1 | 1000 | 0.649 | 0.3842 | No |
| 44 | Ddit3 | 1038 | 0.616 | 0.3814 | No |
| 45 | Slc3a2 | 1041 | 0.613 | 0.3859 | No |
| 46 | Bak1 | 1066 | 0.590 | 0.3857 | No |
| 47 | Nupr1 | 1092 | 0.562 | 0.3850 | No |
| 48 | Upp1 | 1135 | 0.523 | 0.3803 | No |
| 49 | Rhbdf2 | 1151 | 0.512 | 0.3813 | No |
| 50 | Aen | 1219 | -0.508 | 0.3713 | No |
| 51 | F2r | 1319 | -0.521 | 0.3546 | No |
| 52 | App | 1359 | -0.528 | 0.3507 | No |
| 53 | Dnttip2 | 1724 | -0.587 | 0.2784 | No |
| 54 | Fuca1 | 1814 | -0.603 | 0.2645 | No |
| 55 | Vwa5a | 2066 | -0.646 | 0.2167 | No |
| 56 | Tm7sf3 | 2078 | -0.649 | 0.2196 | No |
| 57 | Traf4 | 2080 | -0.650 | 0.2247 | No |
| 58 | Ei24 | 2132 | -0.660 | 0.2193 | No |
| 59 | Cdkn2a | 2149 | -0.662 | 0.2213 | No |
| 60 | Xpc | 2432 | -0.713 | 0.1675 | No |
| 61 | Pdgfa | 2672 | -0.763 | 0.1231 | No |
| 62 | Fbxw7 | 2677 | -0.763 | 0.1285 | No |
| 63 | Mxd4 | 2713 | -0.770 | 0.1273 | No |
| 64 | Hexim1 | 2775 | -0.786 | 0.1208 | No |
| 65 | Pom121 | 2861 | -0.805 | 0.1094 | No |
| 66 | Pmm1 | 2876 | -0.809 | 0.1131 | No |
| 67 | Fdxr | 2916 | -0.817 | 0.1115 | No |
| 68 | Ptpn14 | 3039 | -0.852 | 0.0926 | No |
| 69 | Inhbb | 3105 | -0.872 | 0.0860 | No |
| 70 | Itgb4 | 3162 | -0.888 | 0.0813 | No |
| 71 | Ddb2 | 3225 | -0.907 | 0.0756 | No |
| 72 | Slc19a2 | 3267 | -0.920 | 0.0744 | No |
| 73 | Wrap73 | 3463 | -0.985 | 0.0412 | No |
| 74 | Kif13b | 3513 | -1.002 | 0.0390 | No |
| 75 | Cd82 | 3518 | -1.004 | 0.0464 | No |
| 76 | Fos | 3575 | -1.023 | 0.0429 | No |
| 77 | Tcn2 | 3584 | -1.026 | 0.0495 | No |
| 78 | Tsc22d1 | 3676 | -1.066 | 0.0390 | No |
| 79 | Wwp1 | 3720 | -1.087 | 0.0387 | No |
| 80 | Ip6k2 | 3754 | -1.104 | 0.0407 | No |
| 81 | Sphk1 | 3794 | -1.125 | 0.0417 | No |
| 82 | Prmt2 | 3921 | -1.198 | 0.0248 | No |
| 83 | Coq8a | 3956 | -1.218 | 0.0275 | No |
| 84 | Dcxr | 3966 | -1.224 | 0.0356 | No |
| 85 | Prkab1 | 4029 | -1.267 | 0.0328 | No |
| 86 | Btg2 | 4032 | -1.269 | 0.0427 | No |
| 87 | Tob1 | 4038 | -1.273 | 0.0520 | No |
| 88 | Bmp2 | 4098 | -1.322 | 0.0503 | No |
| 89 | Tspyl2 | 4124 | -1.351 | 0.0561 | No |
| 90 | Ctsf | 4222 | -1.437 | 0.0472 | No |
| 91 | Tm4sf1 | 4421 | -1.676 | 0.0190 | No |
| 92 | Slc35d1 | 4438 | -1.718 | 0.0296 | No |
| 93 | Txnip | 4471 | -1.771 | 0.0373 | No |
| 94 | Cdh13 | 4647 | -2.165 | 0.0179 | No |
| 95 | Ccnd2 | 4672 | -2.265 | 0.0313 | No |
Table: GSEA details [plain text format]

  

Fig 2: HALLMARK\_P53\_PATHWAY: Random ES distribution      
 Gene set null distribution of ES for **HALLMARK\_P53\_PATHWAY**

  
