# Supplementary material for: TMPRSS11B promotes an acidified microenvironment and immune suppression in squamous lung cancer
Source: EMBO Rep. 2025 Nov 10;26(24):6346–79. doi: 10.1038/s44319-025-00631-1 (PMC12714794; doi:10.1038/s44319-025-00631-1)
Supplement: Supplementary file 14 — Figure EV2 Source Data [file 44319_2025_631_MOESM14_ESM.zip › Figure EV2/EV2D-E/GSEA_Broad Institute_Mh_T11b-high LUSC vs LUAD/HALLMARK_PROTEIN_SECRETION.html]

Details for gene set HALLMARK\_PROTEIN\_SECRETION[GSEA]

|  || Dataset | Ranked list\_DGE\_squamousT11b\_vs\_all adenosadeno\_HSE13-NT copy |
| Phenotype | NoPhenotypeAvailable |
| Upregulated in class | na\_neg |
| GeneSet | HALLMARK\_PROTEIN\_SECRETION |
| Enrichment Score (ES) | -0.24850692 |
| Normalized Enrichment Score (NES) | -1.0370814 |
| Nominal p-value | 0.41784036 |
| FDR q-value | 1.0 |
| FWER p-Value | 1.0 |
Table: GSEA Results Summary

  

Fig 1: Enrichment plot: HALLMARK\_PROTEIN\_SECRETION      
 Profile of the Running ES Score & Positions of GeneSet Members on the Rank Ordered List

  

| SYMBOL | RANK IN GENE LIST | RANK METRIC SCORE | RUNNING ES | CORE ENRICHMENT || 1 | Abca1 | 172 | 2.809 | 0.0479 | No |
| 2 | Dst | 434 | 1.596 | 0.0410 | No |
| 3 | Gla | 482 | 1.492 | 0.0757 | No |
| 4 | Ctsc | 770 | 0.907 | 0.0428 | No |
| 5 | Ap2m1 | 1178 | -0.502 | -0.0272 | No |
| 6 | Mon2 | 1296 | -0.519 | -0.0362 | No |
| 7 | Clcn3 | 1577 | -0.563 | -0.0778 | No |
| 8 | Cltc | 1594 | -0.566 | -0.0643 | No |
| 9 | Cav2 | 1641 | -0.574 | -0.0567 | No |
| 10 | Gosr2 | 1698 | -0.583 | -0.0510 | No |
| 11 | Adam10 | 1952 | -0.627 | -0.0852 | No |
| 12 | Gbf1 | 2047 | -0.643 | -0.0856 | No |
| 13 | Snx2 | 2321 | -0.693 | -0.1219 | No |
| 14 | Cog2 | 2562 | -0.740 | -0.1500 | No |
| 15 | Sod1 | 2688 | -0.765 | -0.1532 | No |
| 16 | Arfgef1 | 2702 | -0.767 | -0.1331 | No |
| 17 | Dop1a | 2881 | -0.810 | -0.1461 | No |
| 18 | Stam | 2980 | -0.837 | -0.1416 | No |
| 19 | Atp1a1 | 3251 | -0.916 | -0.1706 | No |
| 20 | Copb1 | 3395 | -0.959 | -0.1718 | No |
| 21 | Ap2b1 | 3413 | -0.966 | -0.1466 | No |
| 22 | Golga4 | 3485 | -0.991 | -0.1318 | No |
| 23 | Sgms1 | 3493 | -0.994 | -0.1037 | No |
| 24 | Tom1l1 | 4188 | -1.401 | -0.2067 | Yes |
| 25 | Arfgap3 | 4199 | -1.409 | -0.1668 | Yes |
| 26 | Tspan8 | 4446 | -1.740 | -0.1663 | Yes |
| 27 | Ocrl | 4467 | -1.763 | -0.1178 | Yes |
| 28 | Pam | 4623 | -2.099 | -0.0876 | Yes |
| 29 | Ica1 | 4624 | -2.099 | -0.0250 | Yes |
| 30 | Sh3gl2 | 4660 | -2.204 | 0.0334 | Yes |
Table: GSEA details [plain text format]

  

Fig 2: HALLMARK\_PROTEIN\_SECRETION: Random ES distribution      
 Gene set null distribution of ES for **HALLMARK\_PROTEIN\_SECRETION**

  
