# Supplementary material for: TMPRSS11B promotes an acidified microenvironment and immune suppression in squamous lung cancer
Source: EMBO Rep. 2025 Nov 10;26(24):6346–79. doi: 10.1038/s44319-025-00631-1 (PMC12714794; doi:10.1038/s44319-025-00631-1)
Supplement: Supplementary file 14 — Figure EV2 Source Data [file 44319_2025_631_MOESM14_ESM.zip › Figure EV2/EV2D-E/GSEA_Broad Institute_Mh_T11b-high LUSC vs LUAD/HALLMARK_SPERMATOGENESIS.html]

Details for gene set HALLMARK\_SPERMATOGENESIS[GSEA]

|  || Dataset | Ranked list\_DGE\_squamousT11b\_vs\_all adenosadeno\_HSE13-NT copy |
| Phenotype | NoPhenotypeAvailable |
| Upregulated in class | na\_neg |
| GeneSet | HALLMARK\_SPERMATOGENESIS |
| Enrichment Score (ES) | -0.19913657 |
| Normalized Enrichment Score (NES) | -0.69178367 |
| Nominal p-value | 0.8524229 |
| FDR q-value | 1.0 |
| FWER p-Value | 1.0 |
Table: GSEA Results Summary

  

Fig 1: Enrichment plot: HALLMARK\_SPERMATOGENESIS      
 Profile of the Running ES Score & Positions of GeneSet Members on the Rank Ordered List

  

| SYMBOL | RANK IN GENE LIST | RANK METRIC SCORE | RUNNING ES | CORE ENRICHMENT || 1 | Alox15 | 501 | 1.444 | -0.0071 | No |
| 2 | Cdk1 | 820 | 0.842 | -0.0166 | No |
| 3 | Ncaph | 1157 | 0.507 | -0.0524 | No |
| 4 | Pias2 | 1361 | -0.528 | -0.0592 | No |
| 5 | Pebp1 | 1685 | -0.581 | -0.0873 | No |
| 6 | Vdac3 | 1895 | -0.618 | -0.0893 | No |
| 7 | Mast2 | 2154 | -0.662 | -0.0984 | No |
| 8 | Spata6 | 2639 | -0.757 | -0.1482 | Yes |
| 9 | Mllt10 | 2673 | -0.763 | -0.1038 | Yes |
| 10 | Tsn | 2736 | -0.775 | -0.0646 | Yes |
| 11 | Ift88 | 2931 | -0.820 | -0.0498 | Yes |
| 12 | Parp2 | 3201 | -0.899 | -0.0453 | Yes |
| 13 | Gstm5 | 3294 | -0.927 | -0.0021 | Yes |
| 14 | Zc3h14 | 3630 | -1.044 | -0.0016 | Yes |
| 15 | Strbp | 4136 | -1.360 | -0.0152 | Yes |
| 16 | Slc12a2 | 4688 | -2.339 | 0.0275 | Yes |
Table: GSEA details [plain text format]

  

Fig 2: HALLMARK\_SPERMATOGENESIS: Random ES distribution      
 Gene set null distribution of ES for **HALLMARK\_SPERMATOGENESIS**

  
