# Supplementary material for: TMPRSS11B promotes an acidified microenvironment and immune suppression in squamous lung cancer
Source: EMBO Rep. 2025 Nov 10;26(24):6346–79. doi: 10.1038/s44319-025-00631-1 (PMC12714794; doi:10.1038/s44319-025-00631-1)
Supplement: Supplementary file 14 — Figure EV2 Source Data [file 44319_2025_631_MOESM14_ESM.zip › Figure EV2/EV2D-E/GSEA_Broad Institute_Mh_T11b-high LUSC vs LUAD/HALLMARK_TGF_BETA_SIGNALING.html]

Details for gene set HALLMARK\_TGF\_BETA\_SIGNALING[GSEA]

|  || Dataset | Ranked list\_DGE\_squamousT11b\_vs\_all adenosadeno\_HSE13-NT copy |
| Phenotype | NoPhenotypeAvailable |
| Upregulated in class | na\_neg |
| GeneSet | HALLMARK\_TGF\_BETA\_SIGNALING |
| Enrichment Score (ES) | -0.23204908 |
| Normalized Enrichment Score (NES) | -0.9528862 |
| Nominal p-value | 0.5141129 |
| FDR q-value | 1.0 |
| FWER p-Value | 1.0 |
Table: GSEA Results Summary

  

Fig 1: Enrichment plot: HALLMARK\_TGF\_BETA\_SIGNALING      
 Profile of the Running ES Score & Positions of GeneSet Members on the Rank Ordered List

  

| SYMBOL | RANK IN GENE LIST | RANK METRIC SCORE | RUNNING ES | CORE ENRICHMENT || 1 | Ppp1r15a | 543 | 1.345 | -0.0616 | No |
| 2 | Rab31 | 571 | 1.265 | -0.0185 | No |
| 3 | Wwtr1 | 730 | 0.963 | -0.0145 | No |
| 4 | Tgfb1 | 940 | 0.718 | -0.0305 | No |
| 5 | Serpine1 | 1049 | 0.610 | -0.0296 | No |
| 6 | Hipk2 | 1145 | 0.516 | -0.0295 | No |
| 7 | Acvr1 | 1434 | -0.538 | -0.0689 | No |
| 8 | Hdac1 | 1550 | -0.560 | -0.0714 | No |
| 9 | Ltbp2 | 1764 | -0.594 | -0.0930 | No |
| 10 | Slc20a1 | 2409 | -0.707 | -0.2001 | No |
| 11 | Trim33 | 2563 | -0.740 | -0.2036 | Yes |
| 12 | Smad7 | 2594 | -0.746 | -0.1812 | Yes |
| 13 | Fnta | 2665 | -0.762 | -0.1665 | Yes |
| 14 | Smurf2 | 2785 | -0.787 | -0.1611 | Yes |
| 15 | Arid4b | 2924 | -0.818 | -0.1584 | Yes |
| 16 | Id1 | 3000 | -0.840 | -0.1417 | Yes |
| 17 | Fkbp1a | 3188 | -0.895 | -0.1463 | Yes |
| 18 | Thbs1 | 3344 | -0.945 | -0.1424 | Yes |
| 19 | Apc | 3622 | -1.041 | -0.1601 | Yes |
| 20 | Id3 | 3653 | -1.055 | -0.1258 | Yes |
| 21 | Bmpr1a | 3890 | -1.180 | -0.1297 | Yes |
| 22 | Smad3 | 3903 | -1.184 | -0.0867 | Yes |
| 23 | Smad6 | 3973 | -1.227 | -0.0539 | Yes |
| 24 | Bmp2 | 4098 | -1.322 | -0.0290 | Yes |
| 25 | Tgif1 | 4179 | -1.391 | 0.0078 | Yes |
| 26 | Bcar3 | 4336 | -1.548 | 0.0348 | Yes |
| 27 | Cdh1 | 4436 | -1.716 | 0.0801 | Yes |
Table: GSEA details [plain text format]

  

Fig 2: HALLMARK\_TGF\_BETA\_SIGNALING: Random ES distribution      
 Gene set null distribution of ES for **HALLMARK\_TGF\_BETA\_SIGNALING**

  
