# Supplementary material for: TMPRSS11B promotes an acidified microenvironment and immune suppression in squamous lung cancer
Source: EMBO Rep. 2025 Nov 10;26(24):6346–79. doi: 10.1038/s44319-025-00631-1 (PMC12714794; doi:10.1038/s44319-025-00631-1)
Supplement: Supplementary file 14 — Figure EV2 Source Data [file 44319_2025_631_MOESM14_ESM.zip › Figure EV2/EV2D-E/GSEA_Broad Institute_Mh_T11b-high LUSC vs LUAD/HALLMARK_TNFA_SIGNALING_VIA_NFKB.html]

Details for gene set HALLMARK\_TNFA\_SIGNALING\_VIA\_NFKB[GSEA]

|  || Dataset | Ranked list\_DGE\_squamousT11b\_vs\_all adenosadeno\_HSE13-NT copy |
| Phenotype | NoPhenotypeAvailable |
| Upregulated in class | na\_pos |
| GeneSet | HALLMARK\_TNFA\_SIGNALING\_VIA\_NFKB |
| Enrichment Score (ES) | 0.37708947 |
| Normalized Enrichment Score (NES) | 1.9007442 |
| Nominal p-value | 0.0 |
| FDR q-value | 0.008140694 |
| FWER p-Value | 0.06 |
Table: GSEA Results Summary

  

Fig 1: Enrichment plot: HALLMARK\_TNFA\_SIGNALING\_VIA\_NFKB      
 Profile of the Running ES Score & Positions of GeneSet Members on the Rank Ordered List

  

| SYMBOL | RANK IN GENE LIST | RANK METRIC SCORE | RUNNING ES | CORE ENRICHMENT || 1 | Tnfaip2 | 83 | 3.933 | 0.0155 | Yes |
| 2 | Serpinb2 | 89 | 3.864 | 0.0469 | Yes |
| 3 | Cxcl5 | 101 | 3.685 | 0.0756 | Yes |
| 4 | Il1a | 119 | 3.497 | 0.1014 | Yes |
| 5 | Mxd1 | 163 | 2.892 | 0.1166 | Yes |
| 6 | Abca1 | 172 | 2.809 | 0.1385 | Yes |
| 7 | Tnc | 191 | 2.672 | 0.1572 | Yes |
| 8 | Plau | 210 | 2.513 | 0.1745 | Yes |
| 9 | Il1b | 240 | 2.351 | 0.1882 | Yes |
| 10 | Plek | 252 | 2.303 | 0.2052 | Yes |
| 11 | Dusp5 | 275 | 2.206 | 0.2191 | Yes |
| 12 | Cd44 | 329 | 1.976 | 0.2245 | Yes |
| 13 | Dusp1 | 339 | 1.923 | 0.2388 | Yes |
| 14 | Tubb2a | 352 | 1.868 | 0.2519 | Yes |
| 15 | Tnf | 391 | 1.722 | 0.2584 | Yes |
| 16 | Cebpb | 425 | 1.620 | 0.2650 | Yes |
| 17 | Klf4 | 448 | 1.555 | 0.2734 | Yes |
| 18 | Dram1 | 494 | 1.458 | 0.2762 | Yes |
| 19 | Irf1 | 508 | 1.429 | 0.2854 | Yes |
| 20 | Slc16a6 | 538 | 1.357 | 0.2907 | Yes |
| 21 | Ppp1r15a | 543 | 1.345 | 0.3012 | Yes |
| 22 | Ier5 | 609 | 1.186 | 0.2974 | Yes |
| 23 | Sat1 | 614 | 1.180 | 0.3065 | Yes |
| 24 | Ehd1 | 637 | 1.118 | 0.3112 | Yes |
| 25 | Atf3 | 643 | 1.106 | 0.3195 | Yes |
| 26 | Hbegf | 684 | 1.029 | 0.3197 | Yes |
| 27 | Map2k3 | 691 | 1.015 | 0.3269 | Yes |
| 28 | Birc3 | 745 | 0.942 | 0.3237 | Yes |
| 29 | Phlda1 | 766 | 0.908 | 0.3271 | Yes |
| 30 | Gadd45b | 804 | 0.862 | 0.3265 | Yes |
| 31 | B4galt5 | 825 | 0.839 | 0.3293 | Yes |
| 32 | Ninj1 | 833 | 0.831 | 0.3348 | Yes |
| 33 | Plpp3 | 846 | 0.814 | 0.3391 | Yes |
| 34 | Cdkn1a | 861 | 0.808 | 0.3430 | Yes |
| 35 | Zc3h12a | 881 | 0.786 | 0.3456 | Yes |
| 36 | Nfkbia | 887 | 0.772 | 0.3510 | Yes |
| 37 | Nfkbie | 899 | 0.760 | 0.3551 | Yes |
| 38 | Dennd5a | 936 | 0.719 | 0.3535 | Yes |
| 39 | Cflar | 963 | 0.693 | 0.3538 | Yes |
| 40 | Ier3 | 967 | 0.691 | 0.3590 | Yes |
| 41 | Mcl1 | 969 | 0.689 | 0.3646 | Yes |
| 42 | Nfkb1 | 972 | 0.685 | 0.3699 | Yes |
| 43 | Maff | 1047 | 0.610 | 0.3594 | Yes |
| 44 | Serpine1 | 1049 | 0.610 | 0.3643 | Yes |
| 45 | Litaf | 1060 | 0.599 | 0.3672 | Yes |
| 46 | Icam1 | 1070 | 0.586 | 0.3703 | Yes |
| 47 | Serpinb8 | 1074 | 0.582 | 0.3745 | Yes |
| 48 | Trib1 | 1112 | 0.544 | 0.3713 | Yes |
| 49 | Sod2 | 1120 | 0.540 | 0.3743 | Yes |
| 50 | Tank | 1129 | 0.529 | 0.3771 | Yes |
| 51 | Kdm6b | 1191 | -0.504 | 0.3684 | No |
| 52 | Cebpd | 1668 | -0.578 | 0.2727 | No |
| 53 | Birc2 | 1712 | -0.583 | 0.2685 | No |
| 54 | Eif1 | 1859 | -0.612 | 0.2428 | No |
| 55 | F3 | 1925 | -0.622 | 0.2343 | No |
| 56 | Pnrc1 | 1935 | -0.624 | 0.2376 | No |
| 57 | Per1 | 2020 | -0.639 | 0.2252 | No |
| 58 | Il6st | 2082 | -0.650 | 0.2178 | No |
| 59 | Socs3 | 2116 | -0.657 | 0.2163 | No |
| 60 | Sdc4 | 2418 | -0.710 | 0.1587 | No |
| 61 | Egr1 | 2694 | -0.766 | 0.1070 | No |
| 62 | Dnajb4 | 3005 | -0.843 | 0.0486 | No |
| 63 | Trip10 | 3014 | -0.846 | 0.0540 | No |
| 64 | Stat5a | 3062 | -0.858 | 0.0513 | No |
| 65 | Sgk1 | 3151 | -0.885 | 0.0401 | No |
| 66 | Ptger4 | 3558 | -1.016 | -0.0372 | No |
| 67 | Fos | 3575 | -1.023 | -0.0320 | No |
| 68 | Relb | 3640 | -1.048 | -0.0367 | No |
| 69 | Tsc22d1 | 3676 | -1.066 | -0.0351 | No |
| 70 | Traf1 | 3786 | -1.119 | -0.0487 | No |
| 71 | Sphk1 | 3794 | -1.125 | -0.0408 | No |
| 72 | Tnfaip8 | 3897 | -1.181 | -0.0524 | No |
| 73 | Smad3 | 3903 | -1.184 | -0.0435 | No |
| 74 | Clcf1 | 3926 | -1.200 | -0.0381 | No |
| 75 | Rel | 3944 | -1.213 | -0.0315 | No |
| 76 | Nr4a1 | 3978 | -1.233 | -0.0281 | No |
| 77 | Zbtb10 | 3989 | -1.239 | -0.0198 | No |
| 78 | B4galt1 | 3994 | -1.244 | -0.0102 | No |
| 79 | Btg2 | 4032 | -1.269 | -0.0073 | No |
| 80 | Fut4 | 4033 | -1.269 | 0.0034 | No |
| 81 | Bmp2 | 4098 | -1.322 | 0.0009 | No |
| 82 | Tgif1 | 4179 | -1.391 | -0.0043 | No |
| 83 | Ackr3 | 4187 | -1.397 | 0.0060 | No |
| 84 | Ccnd1 | 4229 | -1.443 | 0.0095 | No |
| 85 | Nr4a2 | 4493 | -1.801 | -0.0310 | No |
| 86 | Areg | 4558 | -1.937 | -0.0282 | No |
| 87 | Lamb3 | 4582 | -1.998 | -0.0163 | No |
| 88 | F2rl1 | 4690 | -2.349 | -0.0192 | No |
| 89 | Tlr2 | 4716 | -2.445 | -0.0039 | No |
| 90 | Il18 | 4789 | -3.056 | 0.0066 | No |
Table: GSEA details [plain text format]

  

Fig 2: HALLMARK\_TNFA\_SIGNALING\_VIA\_NFKB: Random ES distribution      
 Gene set null distribution of ES for **HALLMARK\_TNFA\_SIGNALING\_VIA\_NFKB**

  
