# Supplementary material for: TMPRSS11B promotes an acidified microenvironment and immune suppression in squamous lung cancer
Source: EMBO Rep. 2025 Nov 10;26(24):6346–79. doi: 10.1038/s44319-025-00631-1 (PMC12714794; doi:10.1038/s44319-025-00631-1)
Supplement: Supplementary file 14 — Figure EV2 Source Data [file 44319_2025_631_MOESM14_ESM.zip › Figure EV2/EV2D-E/GSEA_Broad Institute_Mh_T11b-high LUSC vs LUAD/HALLMARK_UNFOLDED_PROTEIN_RESPONSE.html]

Details for gene set HALLMARK\_UNFOLDED\_PROTEIN\_RESPONSE[GSEA]

|  || Dataset | Ranked list\_DGE\_squamousT11b\_vs\_all adenosadeno\_HSE13-NT copy |
| Phenotype | NoPhenotypeAvailable |
| Upregulated in class | na\_neg |
| GeneSet | HALLMARK\_UNFOLDED\_PROTEIN\_RESPONSE |
| Enrichment Score (ES) | -0.12927641 |
| Normalized Enrichment Score (NES) | -0.58695215 |
| Nominal p-value | 0.9722864 |
| FDR q-value | 0.96404 |
| FWER p-Value | 1.0 |
Table: GSEA Results Summary

  

Fig 1: Enrichment plot: HALLMARK\_UNFOLDED\_PROTEIN\_RESPONSE      
 Profile of the Running ES Score & Positions of GeneSet Members on the Rank Ordered List

  

| SYMBOL | RANK IN GENE LIST | RANK METRIC SCORE | RUNNING ES | CORE ENRICHMENT || 1 | Tubb2a | 352 | 1.868 | -0.0204 | No |
| 2 | Cebpb | 425 | 1.620 | 0.0108 | No |
| 3 | Atf3 | 643 | 1.106 | -0.0031 | No |
| 4 | Psat1 | 954 | 0.702 | -0.0479 | No |
| 5 | Eif4ebp1 | 966 | 0.691 | -0.0305 | No |
| 6 | Wfs1 | 1079 | 0.574 | -0.0375 | No |
| 7 | Nabp1 | 1119 | 0.540 | -0.0303 | No |
| 8 | Edem1 | 1164 | 0.503 | -0.0251 | No |
| 9 | Cxxc1 | 1174 | -0.501 | -0.0127 | No |
| 10 | Sdad1 | 1223 | -0.508 | -0.0083 | No |
| 11 | Imp3 | 1311 | -0.520 | -0.0116 | No |
| 12 | Dcp2 | 1416 | -0.534 | -0.0182 | No |
| 13 | Nop14 | 1493 | -0.550 | -0.0184 | No |
| 14 | Gosr2 | 1698 | -0.583 | -0.0444 | No |
| 15 | Ddx10 | 1723 | -0.587 | -0.0327 | No |
| 16 | Srprb | 1752 | -0.592 | -0.0217 | No |
| 17 | Exoc2 | 1821 | -0.604 | -0.0187 | No |
| 18 | Slc30a5 | 1906 | -0.619 | -0.0186 | No |
| 19 | Eif4a2 | 2128 | -0.659 | -0.0460 | No |
| 20 | Edc4 | 2261 | -0.682 | -0.0542 | No |
| 21 | Aldh18a1 | 2527 | -0.732 | -0.0887 | No |
| 22 | Preb | 2722 | -0.771 | -0.1073 | Yes |
| 23 | Eif4a3 | 2786 | -0.787 | -0.0980 | Yes |
| 24 | Pop4 | 2798 | -0.790 | -0.0778 | Yes |
| 25 | Cnot4 | 2804 | -0.791 | -0.0563 | Yes |
| 26 | Mtrex | 2918 | -0.817 | -0.0566 | Yes |
| 27 | Dctn1 | 2927 | -0.820 | -0.0349 | Yes |
| 28 | Kif5b | 2964 | -0.830 | -0.0188 | Yes |
| 29 | Paip1 | 3260 | -0.918 | -0.0543 | Yes |
| 30 | Parn | 3492 | -0.994 | -0.0742 | Yes |
| 31 | Hyou1 | 3531 | -1.008 | -0.0534 | Yes |
| 32 | Arfgap1 | 3703 | -1.082 | -0.0584 | Yes |
| 33 | Dnajc3 | 3895 | -1.181 | -0.0646 | Yes |
| 34 | Xbp1 | 3997 | -1.246 | -0.0502 | Yes |
| 35 | Asns | 4004 | -1.251 | -0.0158 | Yes |
| 36 | Kdelr3 | 4089 | -1.318 | 0.0042 | Yes |
| 37 | Tspyl2 | 4124 | -1.351 | 0.0356 | Yes |
| 38 | Pdia5 | 4544 | -1.894 | 0.0020 | Yes |
| 39 | Stc2 | 4564 | -1.949 | 0.0535 | Yes |
Table: GSEA details [plain text format]

  

Fig 2: HALLMARK\_UNFOLDED\_PROTEIN\_RESPONSE: Random ES distribution      
 Gene set null distribution of ES for **HALLMARK\_UNFOLDED\_PROTEIN\_RESPONSE**

  
