# Supplementary material for: TMPRSS11B promotes an acidified microenvironment and immune suppression in squamous lung cancer
Source: EMBO Rep. 2025 Nov 10;26(24):6346–79. doi: 10.1038/s44319-025-00631-1 (PMC12714794; doi:10.1038/s44319-025-00631-1)
Supplement: Supplementary file 14 — Figure EV2 Source Data [file 44319_2025_631_MOESM14_ESM.zip › Figure EV2/EV2D-E/GSEA_Broad Institute_Mh_T11b-high LUSC vs LUAD/HALLMARK_UV_RESPONSE_DN.html]

Details for gene set HALLMARK\_UV\_RESPONSE\_DN[GSEA]

|  || Dataset | Ranked list\_DGE\_squamousT11b\_vs\_all adenosadeno\_HSE13-NT copy |
| Phenotype | NoPhenotypeAvailable |
| Upregulated in class | na\_neg |
| GeneSet | HALLMARK\_UV\_RESPONSE\_DN |
| Enrichment Score (ES) | -0.119621605 |
| Normalized Enrichment Score (NES) | -0.6332741 |
| Nominal p-value | 0.9321149 |
| FDR q-value | 1.0 |
| FWER p-Value | 1.0 |
Table: GSEA Results Summary

  

Fig 1: Enrichment plot: HALLMARK\_UV\_RESPONSE\_DN      
 Profile of the Running ES Score & Positions of GeneSet Members on the Rank Ordered List

  

| SYMBOL | RANK IN GENE LIST | RANK METRIC SCORE | RUNNING ES | CORE ENRICHMENT || 1 | Gja1 | 151 | 3.006 | 0.0108 | No |
| 2 | Dusp1 | 339 | 1.923 | -0.0013 | No |
| 3 | Pik3cd | 516 | 1.406 | -0.0185 | No |
| 4 | Pmp22 | 517 | 1.400 | 0.0014 | No |
| 5 | Cited2 | 522 | 1.391 | 0.0202 | No |
| 6 | Adgrl2 | 575 | 1.262 | 0.0272 | No |
| 7 | Prkar2b | 737 | 0.958 | 0.0068 | No |
| 8 | Mgll | 752 | 0.929 | 0.0170 | No |
| 9 | Rnd3 | 756 | 0.924 | 0.0295 | No |
| 10 | Plpp3 | 846 | 0.814 | 0.0223 | No |
| 11 | Syne1 | 854 | 0.811 | 0.0323 | No |
| 12 | Mgmt | 922 | 0.739 | 0.0287 | No |
| 13 | Nfkb1 | 972 | 0.685 | 0.0281 | No |
| 14 | Met | 988 | 0.664 | 0.0343 | No |
| 15 | Dab2 | 1031 | 0.623 | 0.0343 | No |
| 16 | Col3a1 | 1034 | 0.619 | 0.0427 | No |
| 17 | Serpine1 | 1049 | 0.610 | 0.0484 | No |
| 18 | Col5a2 | 1080 | 0.573 | 0.0502 | No |
| 19 | Fzd2 | 1114 | 0.544 | 0.0509 | No |
| 20 | Pias3 | 1215 | -0.507 | 0.0371 | No |
| 21 | Anxa4 | 1225 | -0.509 | 0.0424 | No |
| 22 | Ltbp1 | 1378 | -0.530 | 0.0179 | No |
| 23 | Dyrk1a | 1405 | -0.533 | 0.0200 | No |
| 24 | Acvr2a | 1588 | -0.565 | -0.0103 | No |
| 25 | Mt1 | 1658 | -0.576 | -0.0167 | No |
| 26 | Nr1d2 | 1730 | -0.589 | -0.0233 | No |
| 27 | Scaf8 | 1754 | -0.592 | -0.0198 | No |
| 28 | Atrx | 1907 | -0.619 | -0.0430 | No |
| 29 | F3 | 1925 | -0.622 | -0.0378 | No |
| 30 | Dlg1 | 1932 | -0.623 | -0.0302 | No |
| 31 | Slc7a1 | 1940 | -0.624 | -0.0228 | No |
| 32 | Nfib | 2205 | -0.672 | -0.0689 | No |
| 33 | Atxn1 | 2207 | -0.672 | -0.0596 | No |
| 34 | Rbpms | 2228 | -0.676 | -0.0542 | No |
| 35 | Sipa1l1 | 2250 | -0.680 | -0.0490 | No |
| 36 | Nek7 | 2304 | -0.689 | -0.0504 | No |
| 37 | Ythdc1 | 2425 | -0.712 | -0.0656 | No |
| 38 | Togaram1 | 2438 | -0.715 | -0.0579 | No |
| 39 | Aggf1 | 2533 | -0.734 | -0.0673 | No |
| 40 | Smad7 | 2594 | -0.746 | -0.0694 | No |
| 41 | Tent4a | 2759 | -0.781 | -0.0929 | No |
| 42 | Lamc1 | 2789 | -0.788 | -0.0878 | No |
| 43 | Add3 | 2899 | -0.814 | -0.0992 | No |
| 44 | Nr3c1 | 2997 | -0.839 | -0.1077 | Yes |
| 45 | Id1 | 3000 | -0.840 | -0.0963 | Yes |
| 46 | Tgfbr2 | 3104 | -0.872 | -0.1056 | Yes |
| 47 | Tgfbr3 | 3149 | -0.885 | -0.1023 | Yes |
| 48 | Dbp | 3176 | -0.892 | -0.0951 | Yes |
| 49 | Erbb2 | 3230 | -0.909 | -0.0934 | Yes |
| 50 | Atrn | 3278 | -0.923 | -0.0902 | Yes |
| 51 | Phf3 | 3289 | -0.926 | -0.0792 | Yes |
| 52 | Prdm2 | 3454 | -0.983 | -0.0998 | Yes |
| 53 | Rasa2 | 3497 | -0.995 | -0.0946 | Yes |
| 54 | Fhl2 | 3508 | -1.000 | -0.0825 | Yes |
| 55 | Prkca | 3544 | -1.013 | -0.0755 | Yes |
| 56 | Mios | 3573 | -1.022 | -0.0670 | Yes |
| 57 | Gcnt1 | 3577 | -1.024 | -0.0531 | Yes |
| 58 | Vav2 | 3647 | -1.053 | -0.0527 | Yes |
| 59 | Sri | 3726 | -1.092 | -0.0536 | Yes |
| 60 | Bmpr1a | 3890 | -1.180 | -0.0712 | Yes |
| 61 | Smad3 | 3903 | -1.184 | -0.0570 | Yes |
| 62 | Efemp1 | 4096 | -1.322 | -0.0787 | Yes |
| 63 | Plcb4 | 4260 | -1.472 | -0.0922 | Yes |
| 64 | Slc22a18 | 4312 | -1.515 | -0.0814 | Yes |
| 65 | Pparg | 4408 | -1.658 | -0.0779 | Yes |
| 66 | Ptpn21 | 4430 | -1.703 | -0.0582 | Yes |
| 67 | Lpar1 | 4622 | -2.097 | -0.0687 | Yes |
| 68 | Ica1 | 4624 | -2.099 | -0.0392 | Yes |
| 69 | Kit | 4740 | -2.572 | -0.0270 | Yes |
| 70 | Igfbp5 | 4791 | -3.078 | 0.0061 | Yes |
Table: GSEA details [plain text format]

  

Fig 2: HALLMARK\_UV\_RESPONSE\_DN: Random ES distribution      
 Gene set null distribution of ES for **HALLMARK\_UV\_RESPONSE\_DN**

  
