# Supplementary figures and images for: TMPRSS11B promotes an acidified microenvironment and immune suppression in squamous lung cancer (part 4 of 6)
Source: EMBO Rep. 2025 Nov 10;26(24):6346–79. doi: 10.1038/s44319-025-00631-1 (PMC12714794; doi:10.1038/s44319-025-00631-1)

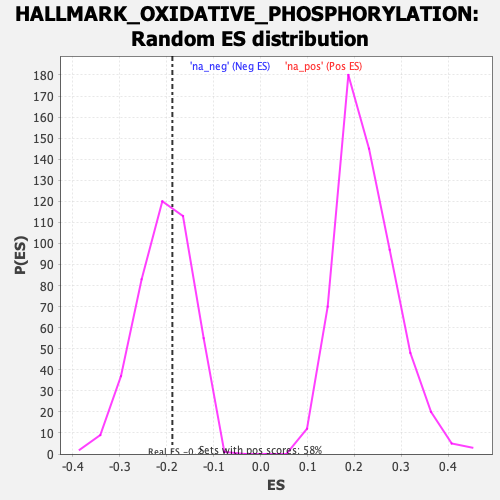

Supplement: Supplementary file 14 — Figure EV2 Source Data [file 44319_2025_631_MOESM14_ESM.zip › Figure EV2/EV2D-E/GSEA_Broad Institute_Mh_T11b-high LUSC vs LUAD/gset_rnd_es_dist_926.png]

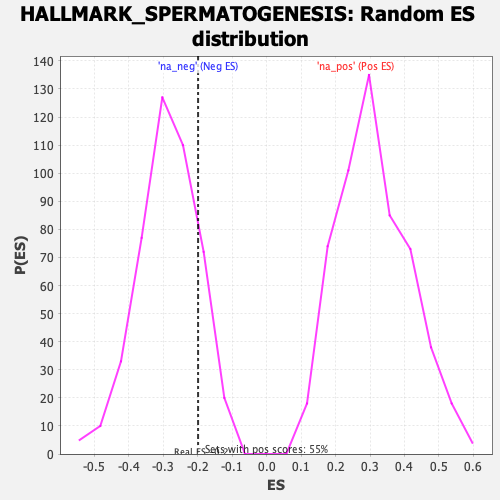

Supplement: Supplementary file 14 — Figure EV2 Source Data [file 44319_2025_631_MOESM14_ESM.zip › Figure EV2/EV2D-E/GSEA_Broad Institute_Mh_T11b-high LUSC vs LUAD/gset_rnd_es_dist_928.png]

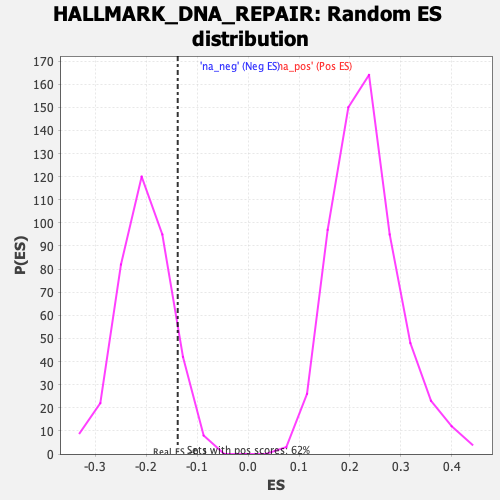

Supplement: Supplementary file 14 — Figure EV2 Source Data [file 44319_2025_631_MOESM14_ESM.zip › Figure EV2/EV2D-E/GSEA_Broad Institute_Mh_T11b-high LUSC vs LUAD/gset_rnd_es_dist_930.png]

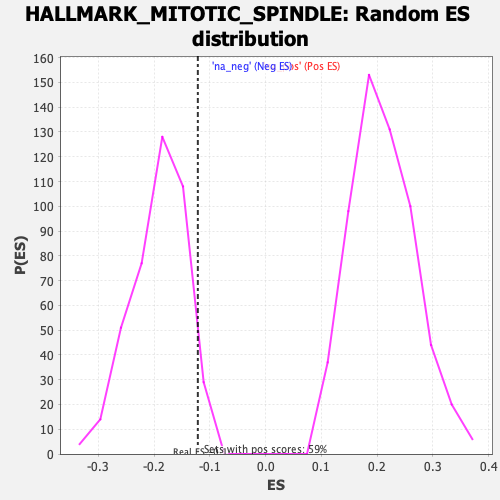

Supplement: Supplementary file 14 — Figure EV2 Source Data [file 44319_2025_631_MOESM14_ESM.zip › Figure EV2/EV2D-E/GSEA_Broad Institute_Mh_T11b-high LUSC vs LUAD/gset_rnd_es_dist_932.png]

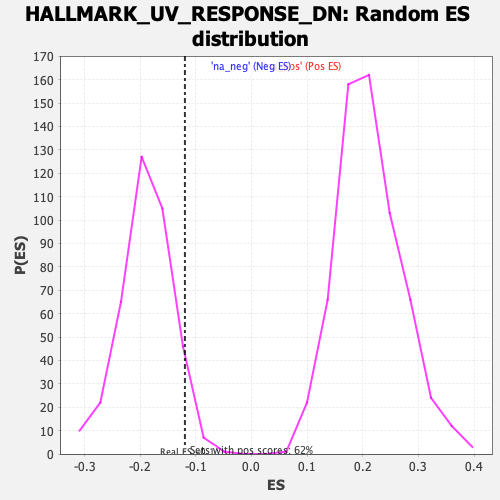

Supplement: Supplementary file 14 — Figure EV2 Source Data [file 44319_2025_631_MOESM14_ESM.zip › Figure EV2/EV2D-E/GSEA_Broad Institute_Mh_T11b-high LUSC vs LUAD/gset_rnd_es_dist_934.png]

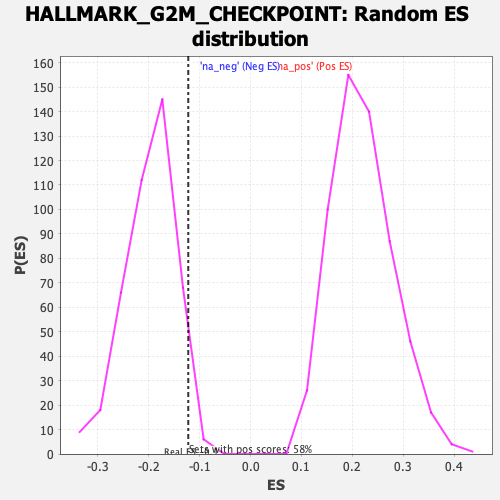

Supplement: Supplementary file 14 — Figure EV2 Source Data [file 44319_2025_631_MOESM14_ESM.zip › Figure EV2/EV2D-E/GSEA_Broad Institute_Mh_T11b-high LUSC vs LUAD/gset_rnd_es_dist_936.png]

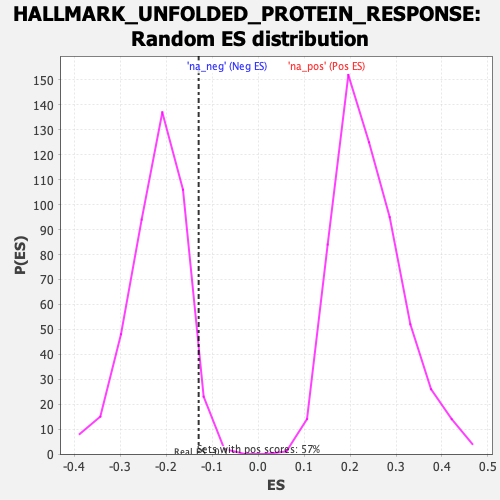

Supplement: Supplementary file 14 — Figure EV2 Source Data [file 44319_2025_631_MOESM14_ESM.zip › Figure EV2/EV2D-E/GSEA_Broad Institute_Mh_T11b-high LUSC vs LUAD/gset_rnd_es_dist_938.png]

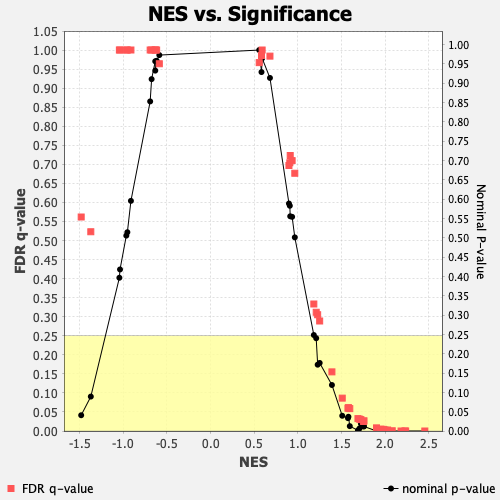

Supplement: Supplementary file 14 — Figure EV2 Source Data [file 44319_2025_631_MOESM14_ESM.zip › Figure EV2/EV2D-E/GSEA_Broad Institute_Mh_T11b-high LUSC vs LUAD/pvalues_vs_nes_plot.png]

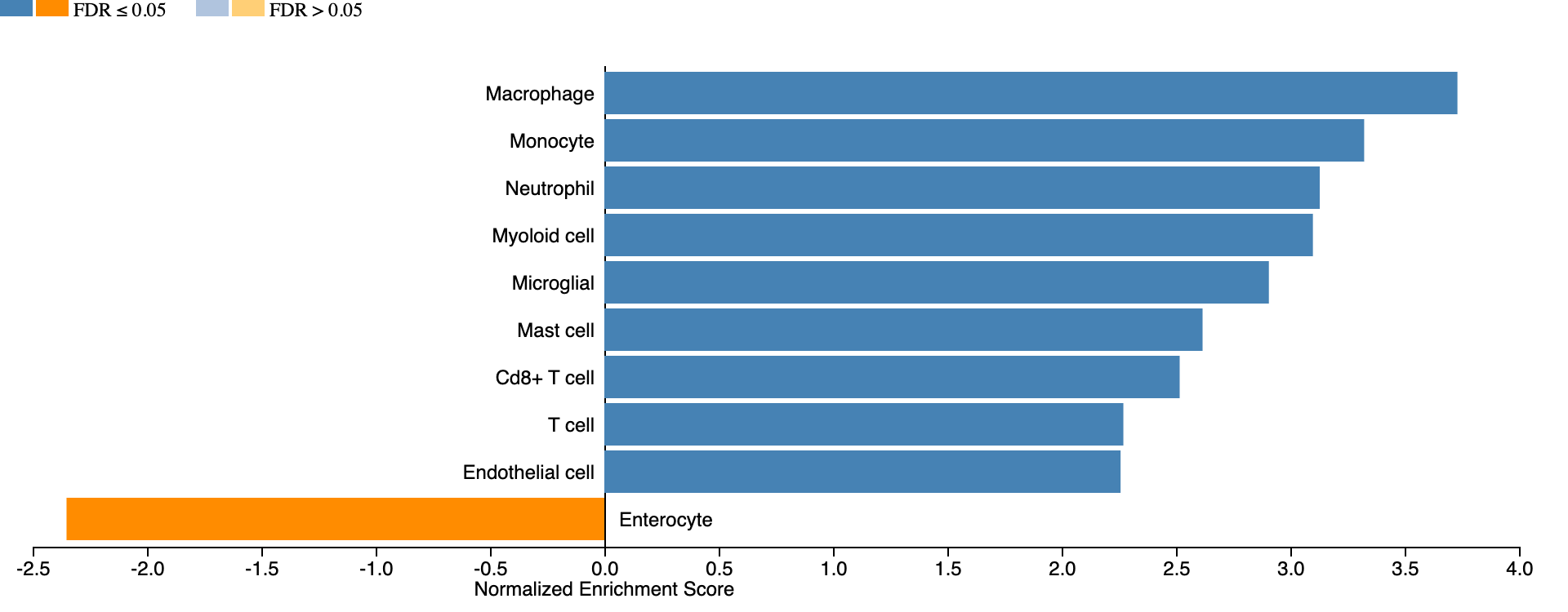

Supplement: Supplementary file 17 — Figure EV5 Source Data [file 44319_2025_631_MOESM17_ESM.zip › Figure EV5/EV5A/GSEA T11b high LUSC vs LUAD/wg_bar (2).png]

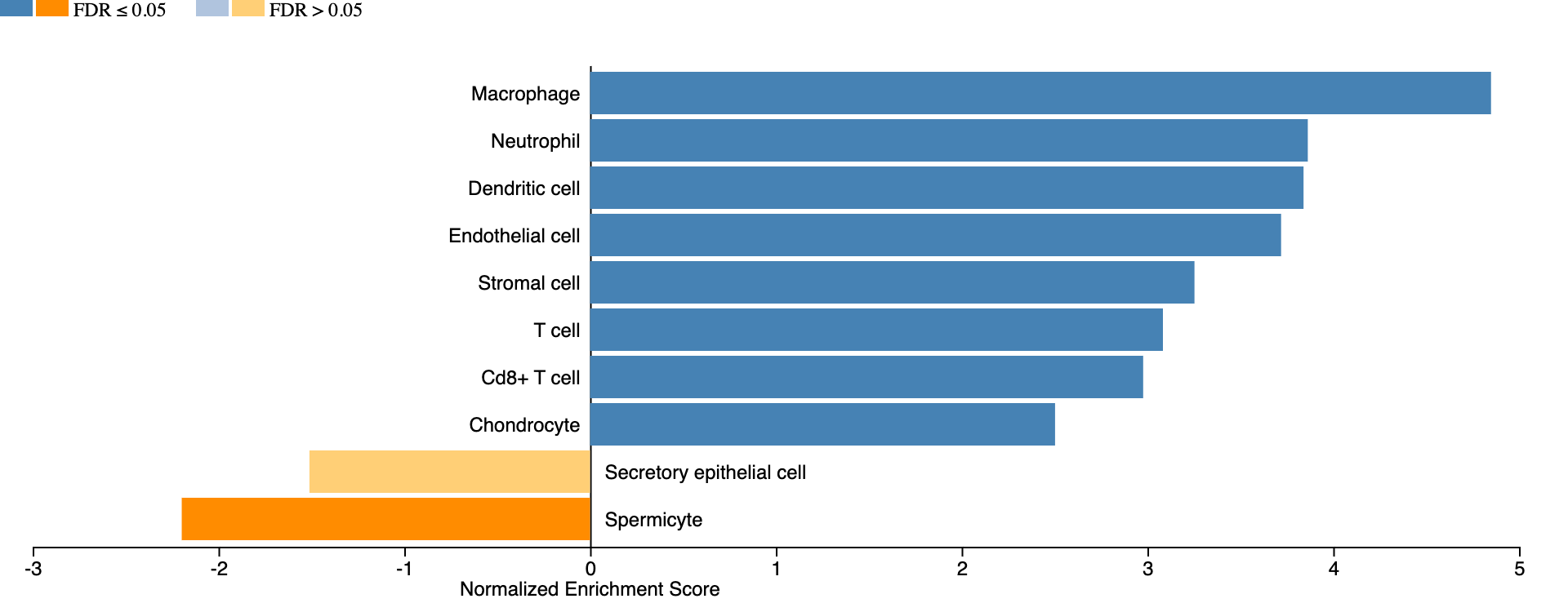

Supplement: Supplementary file 17 — Figure EV5 Source Data [file 44319_2025_631_MOESM17_ESM.zip › Figure EV5/EV5A/GSEA T11b high LUSC vs T11b low LUSC/wg_bar (2).png]

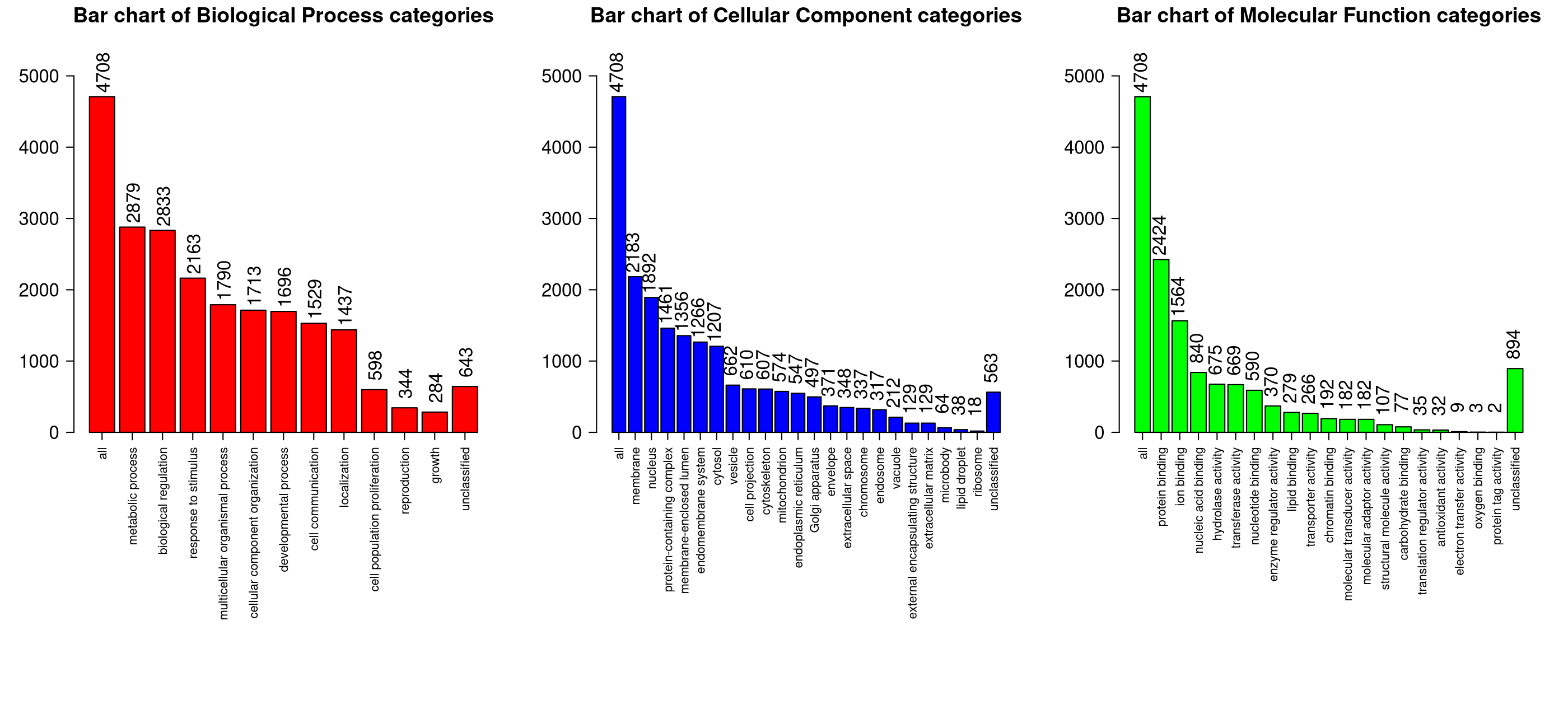

Supplement: Supplementary file 17 — Figure EV5 Source Data [file 44319_2025_631_MOESM17_ESM.zip › Figure EV5/EV5A/GSEA T11b high LUSC vs LUAD/Project_wg_result1731453835/goslim_summary_wg_result1731453835.png]

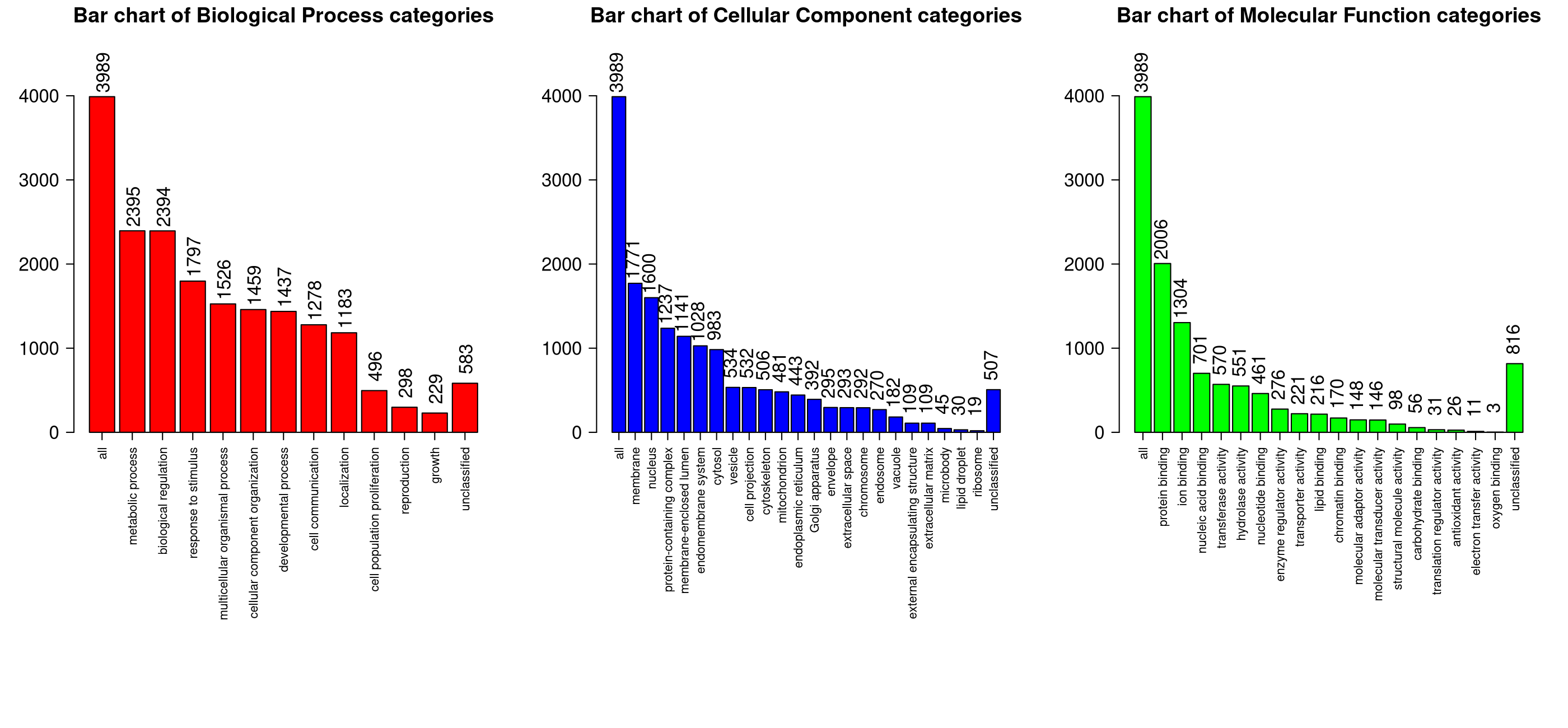

Supplement: Supplementary file 17 — Figure EV5 Source Data [file 44319_2025_631_MOESM17_ESM.zip › Figure EV5/EV5A/GSEA T11b high LUSC vs T11b low LUSC/Project_wg_result1729116948/goslim_summary_wg_result1729116948.png]

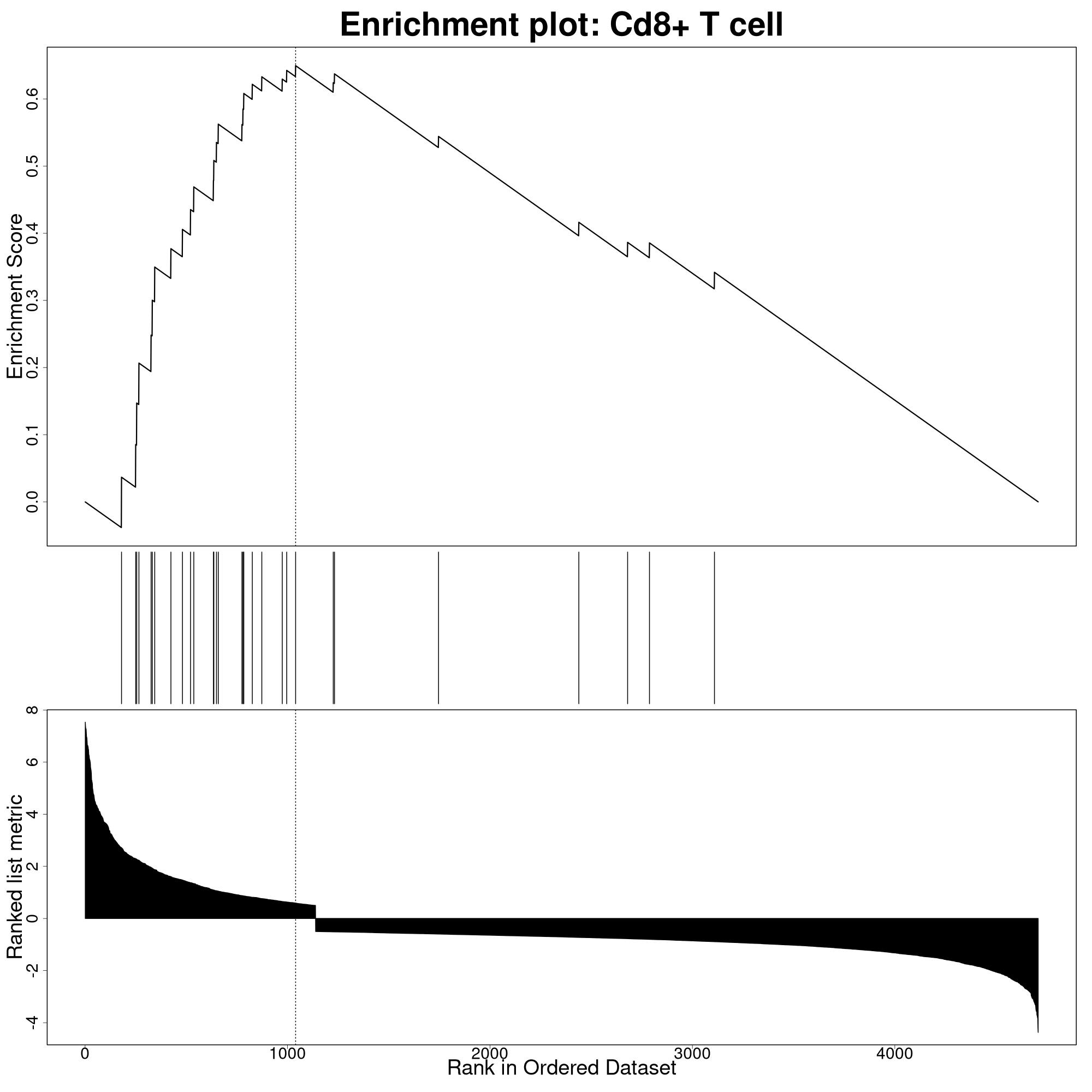

Supplement: Supplementary file 17 — Figure EV5 Source Data [file 44319_2025_631_MOESM17_ESM.zip › Figure EV5/EV5A/GSEA T11b high LUSC vs LUAD/Project_wg_result1731453835/Project_wg_result1731453835_GSEA/Cd8_ T cell.png]

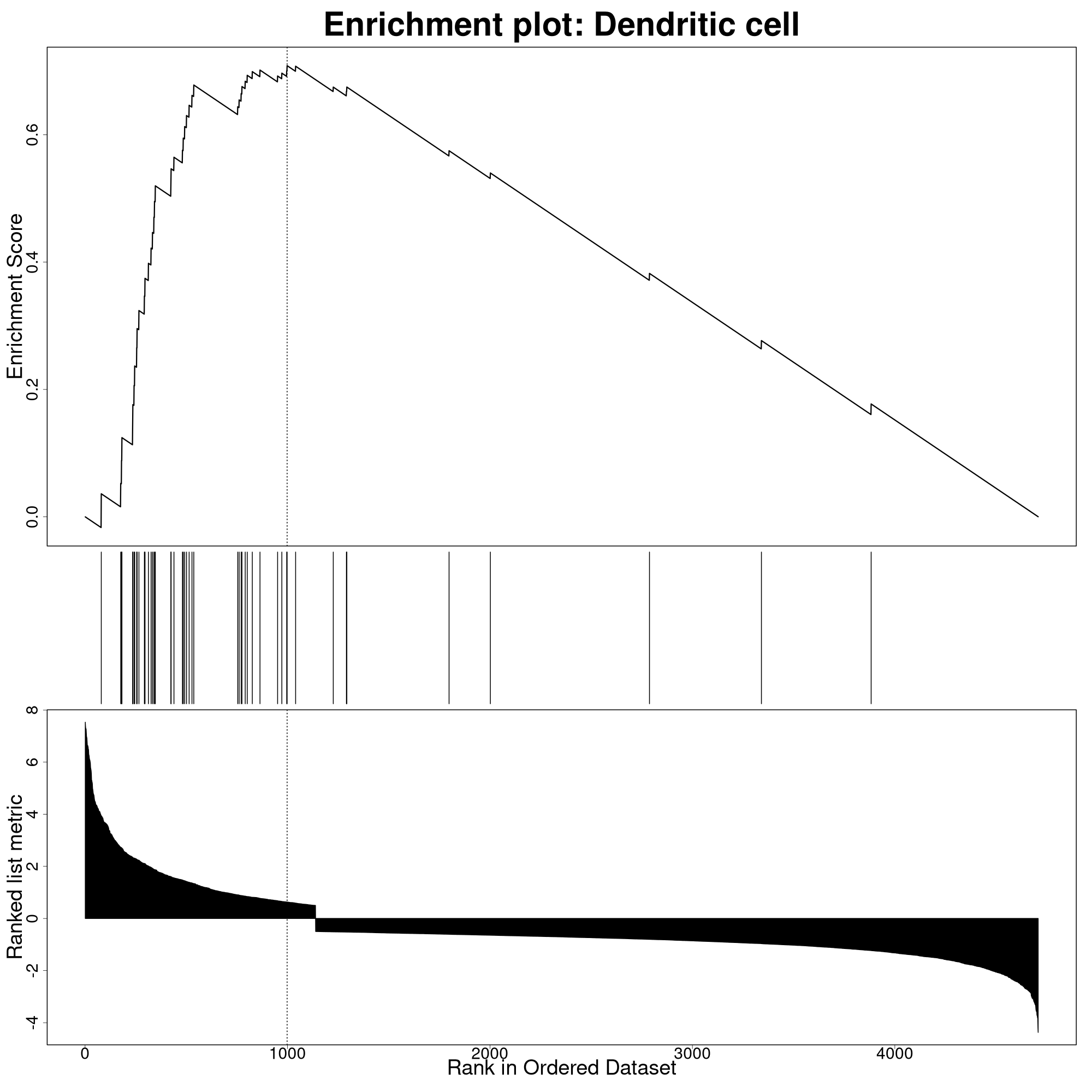

Supplement: Supplementary file 17 — Figure EV5 Source Data [file 44319_2025_631_MOESM17_ESM.zip › Figure EV5/EV5A/GSEA T11b high LUSC vs LUAD/Project_wg_result1731453835/Project_wg_result1731453835_GSEA/Dendritic cell.png]

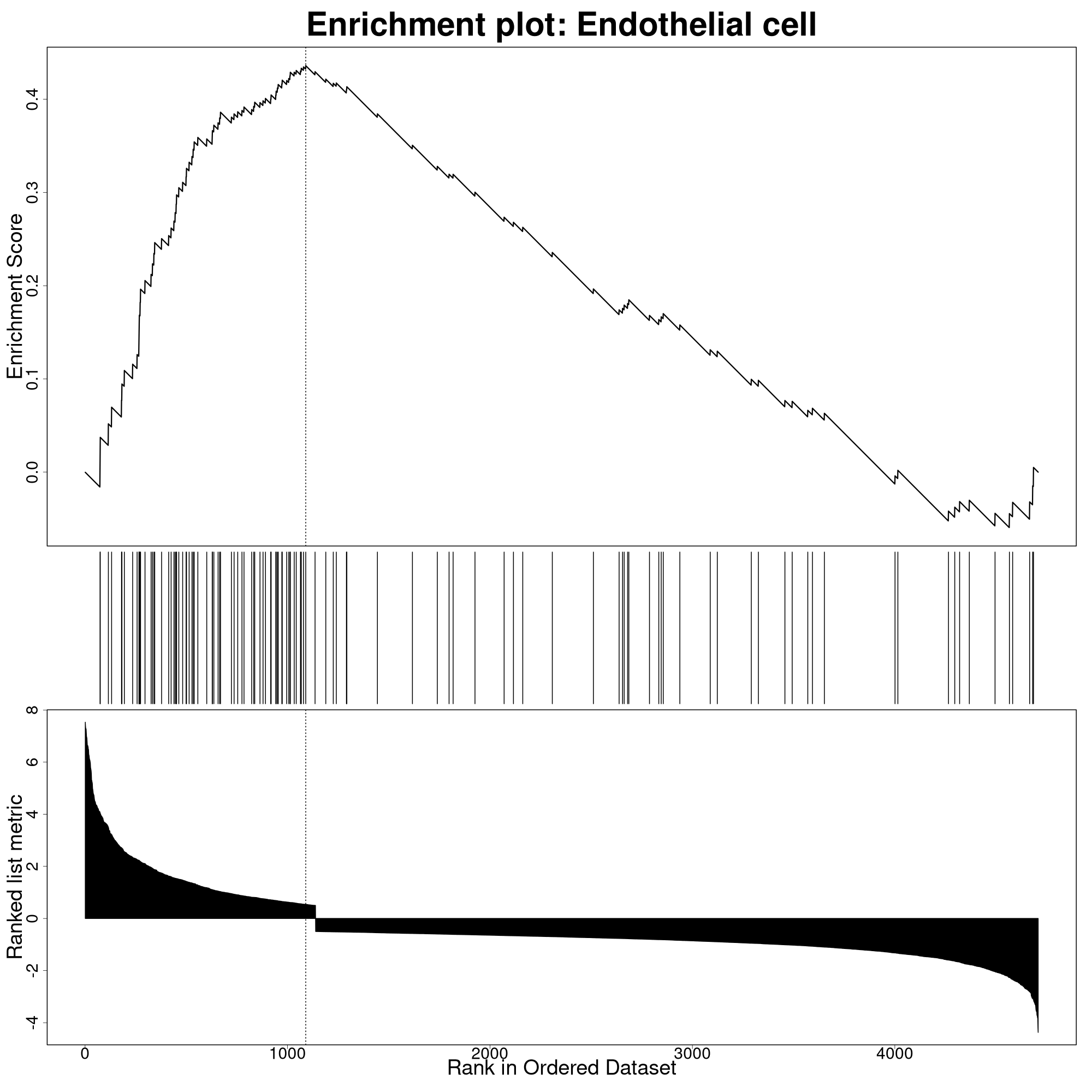

Supplement: Supplementary file 17 — Figure EV5 Source Data [file 44319_2025_631_MOESM17_ESM.zip › Figure EV5/EV5A/GSEA T11b high LUSC vs LUAD/Project_wg_result1731453835/Project_wg_result1731453835_GSEA/Endothelial cell.png]

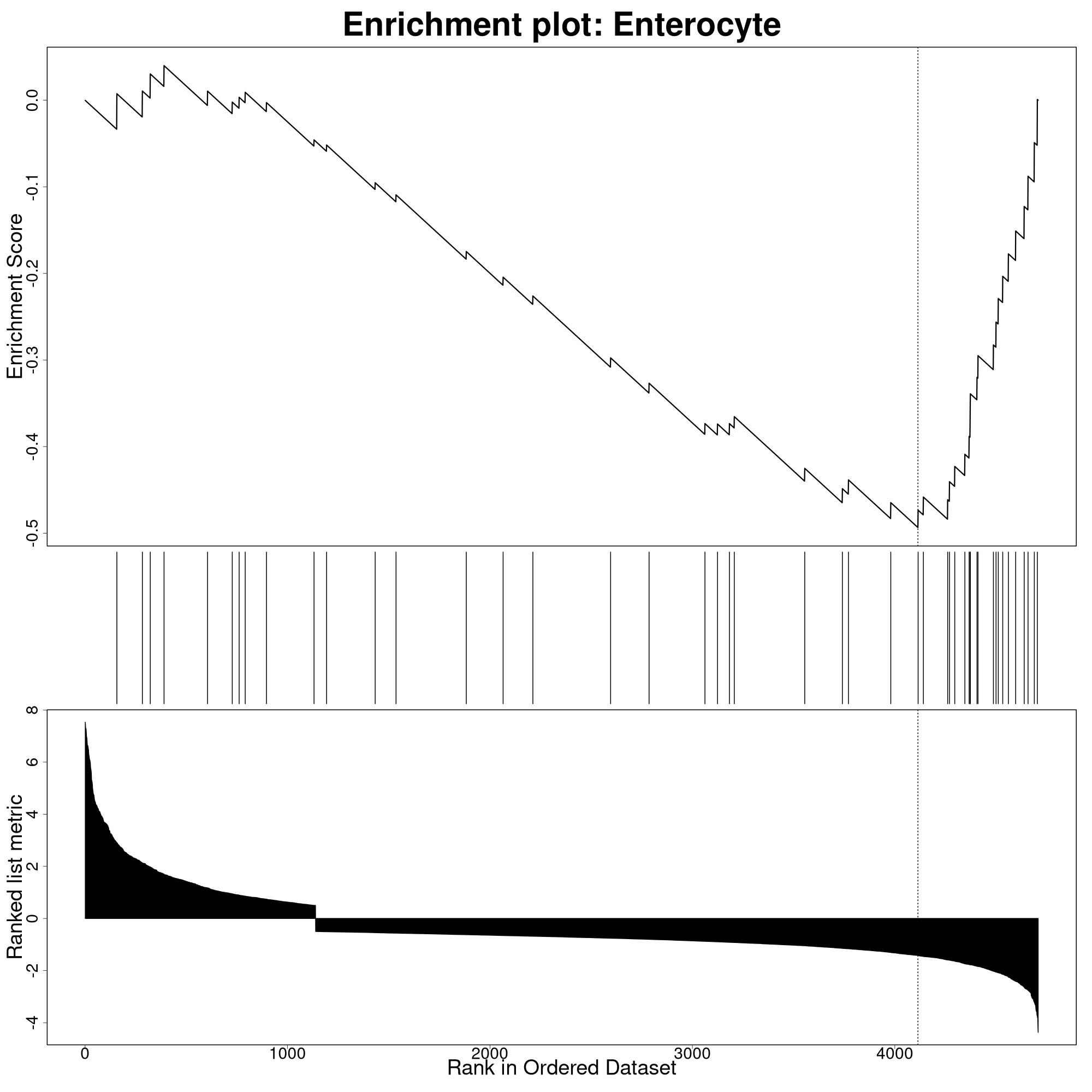

Supplement: Supplementary file 17 — Figure EV5 Source Data [file 44319_2025_631_MOESM17_ESM.zip › Figure EV5/EV5A/GSEA T11b high LUSC vs LUAD/Project_wg_result1731453835/Project_wg_result1731453835_GSEA/Enterocyte.png]

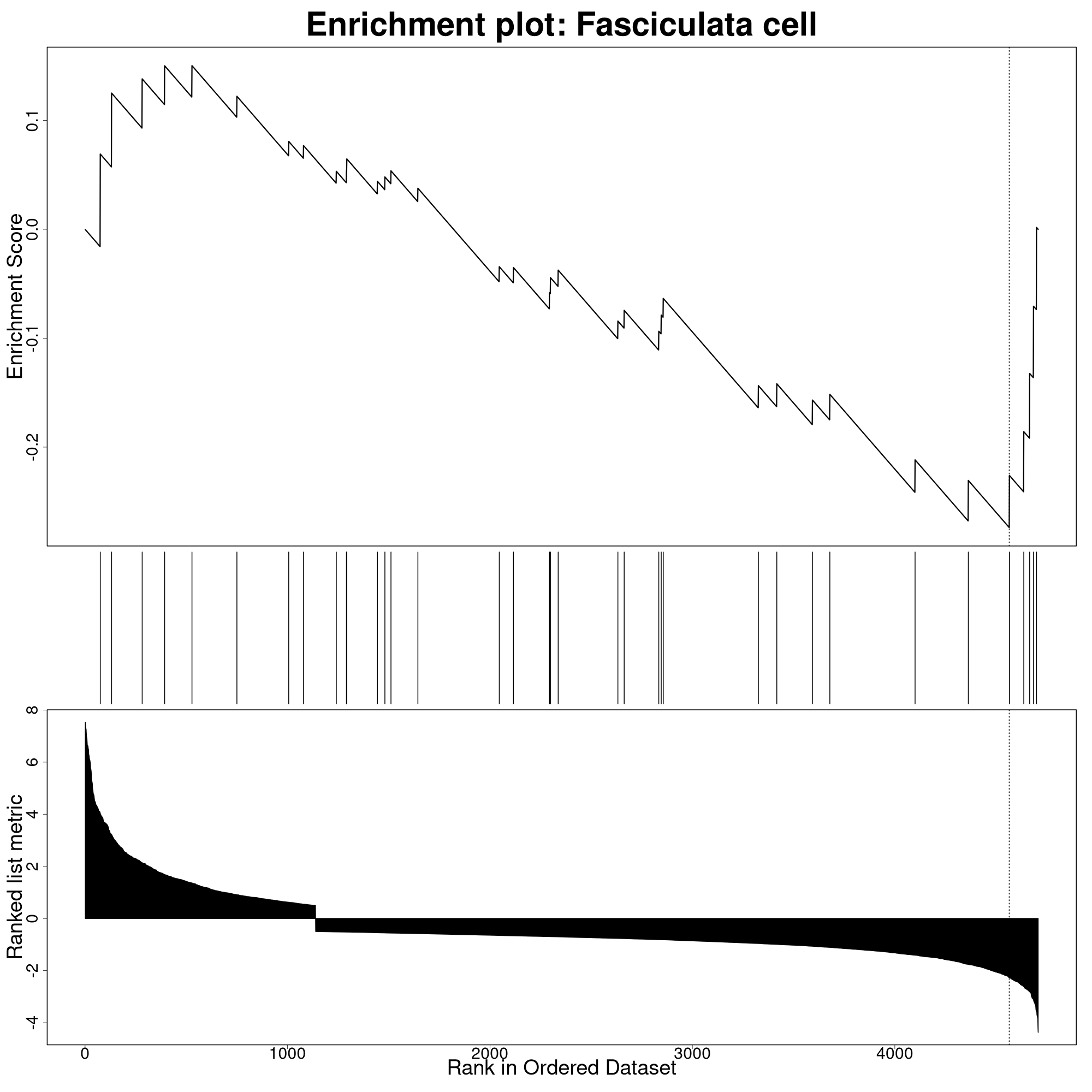

Supplement: Supplementary file 17 — Figure EV5 Source Data [file 44319_2025_631_MOESM17_ESM.zip › Figure EV5/EV5A/GSEA T11b high LUSC vs LUAD/Project_wg_result1731453835/Project_wg_result1731453835_GSEA/Fasciculata cell.png]

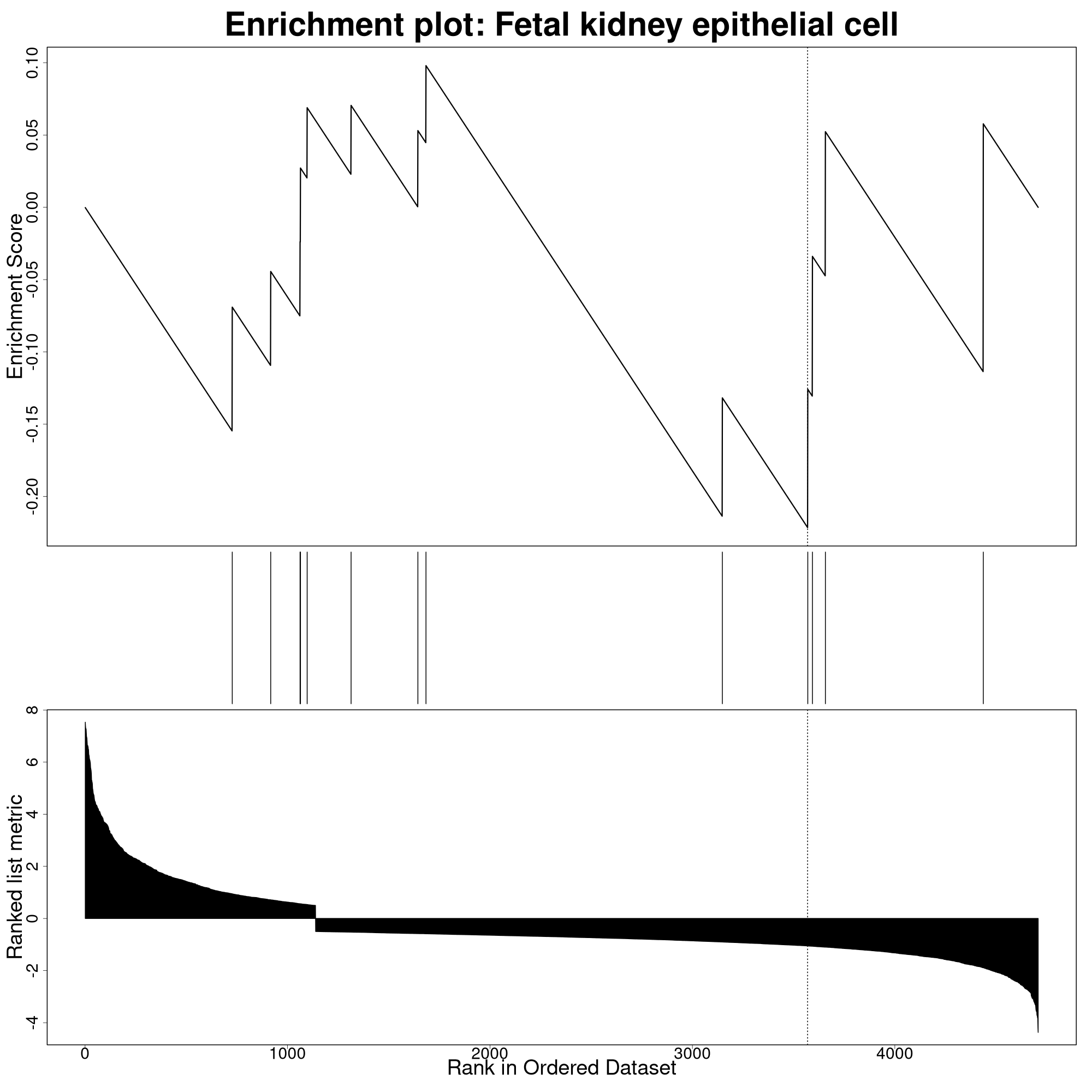

Supplement: Supplementary file 17 — Figure EV5 Source Data [file 44319_2025_631_MOESM17_ESM.zip › Figure EV5/EV5A/GSEA T11b high LUSC vs LUAD/Project_wg_result1731453835/Project_wg_result1731453835_GSEA/Fetal kidney epithelial cell.png]

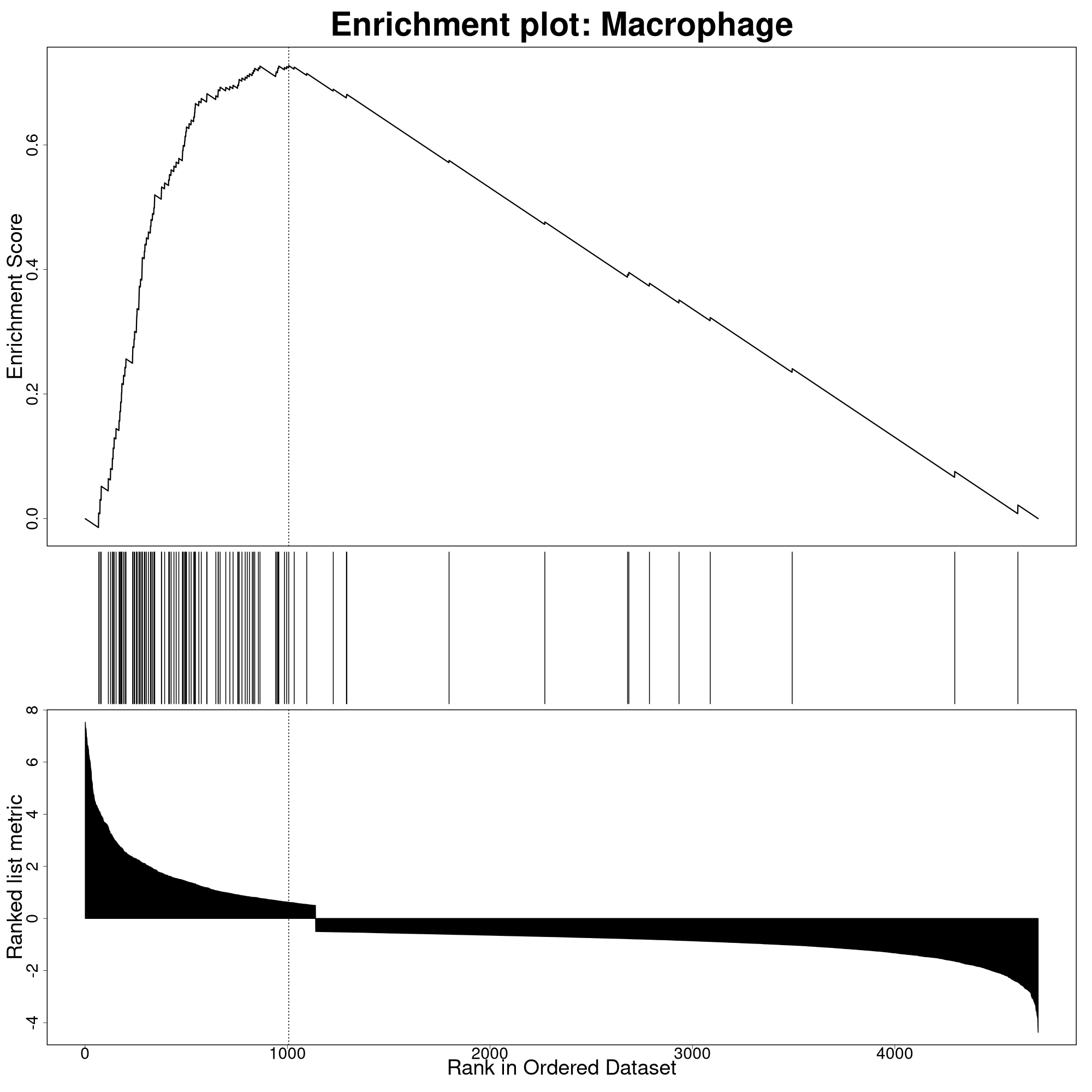

Supplement: Supplementary file 17 — Figure EV5 Source Data [file 44319_2025_631_MOESM17_ESM.zip › Figure EV5/EV5A/GSEA T11b high LUSC vs LUAD/Project_wg_result1731453835/Project_wg_result1731453835_GSEA/Macrophage.png]

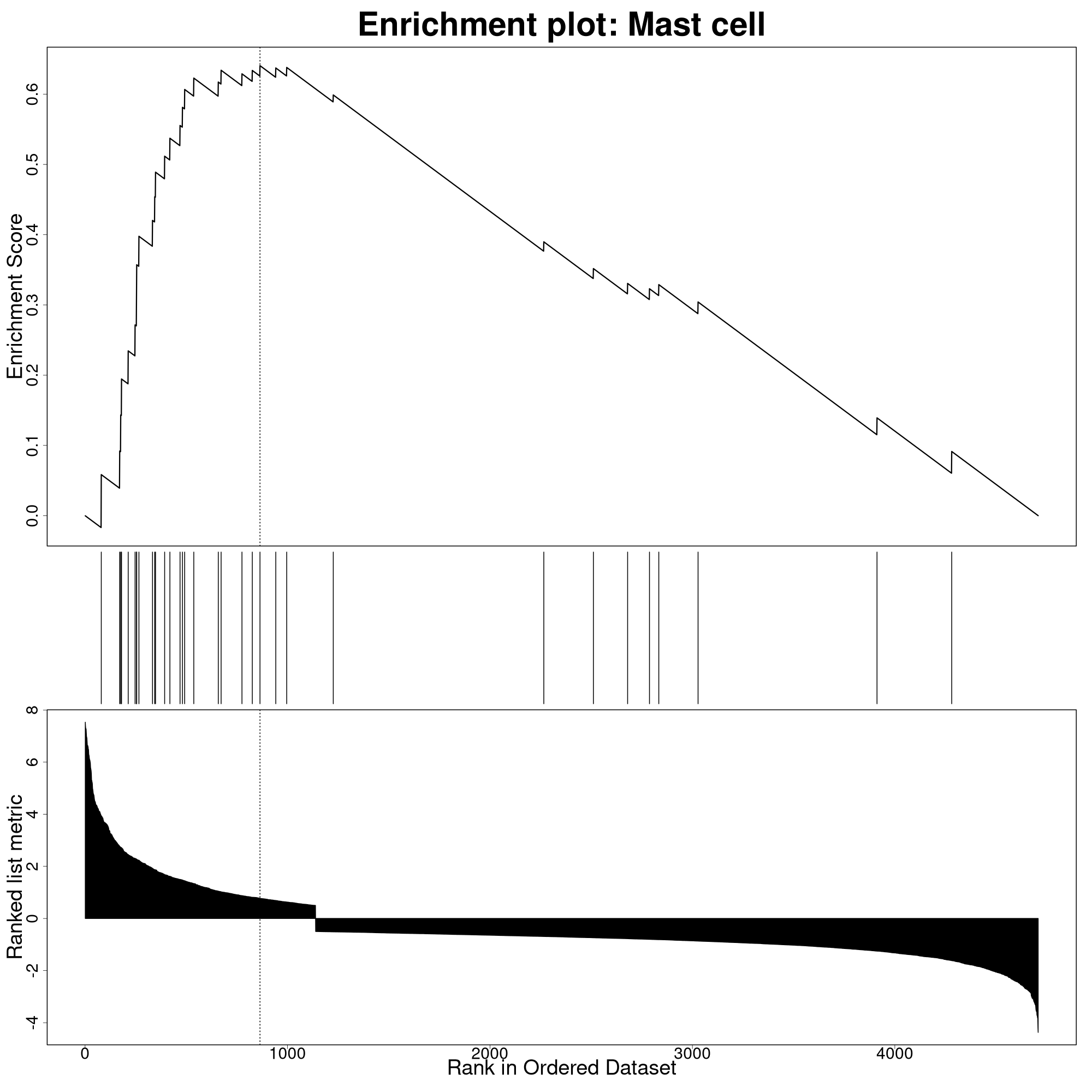

Supplement: Supplementary file 17 — Figure EV5 Source Data [file 44319_2025_631_MOESM17_ESM.zip › Figure EV5/EV5A/GSEA T11b high LUSC vs LUAD/Project_wg_result1731453835/Project_wg_result1731453835_GSEA/Mast cell.png]

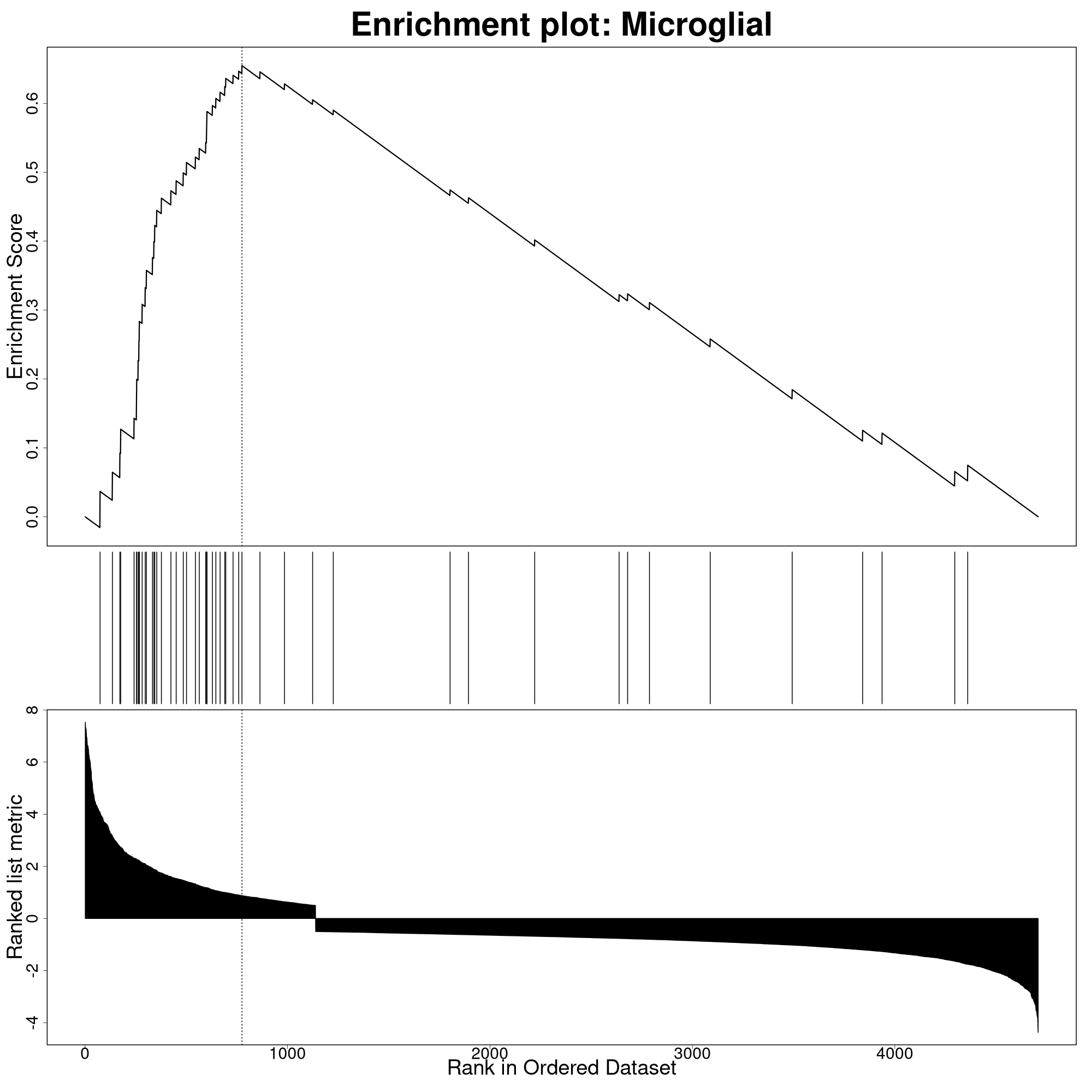

Supplement: Supplementary file 17 — Figure EV5 Source Data [file 44319_2025_631_MOESM17_ESM.zip › Figure EV5/EV5A/GSEA T11b high LUSC vs LUAD/Project_wg_result1731453835/Project_wg_result1731453835_GSEA/Microglial.png]

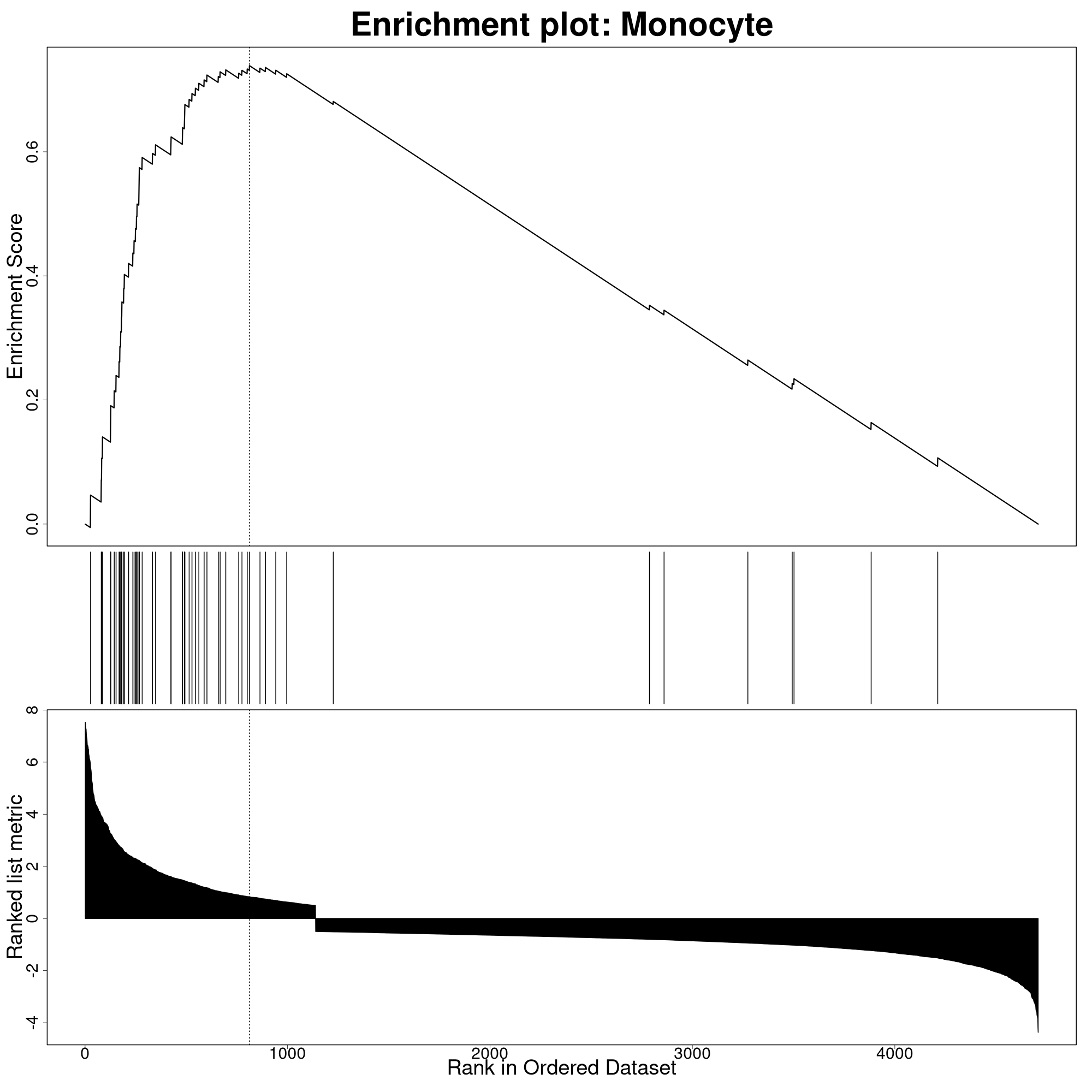

Supplement: Supplementary file 17 — Figure EV5 Source Data [file 44319_2025_631_MOESM17_ESM.zip › Figure EV5/EV5A/GSEA T11b high LUSC vs LUAD/Project_wg_result1731453835/Project_wg_result1731453835_GSEA/Monocyte.png]

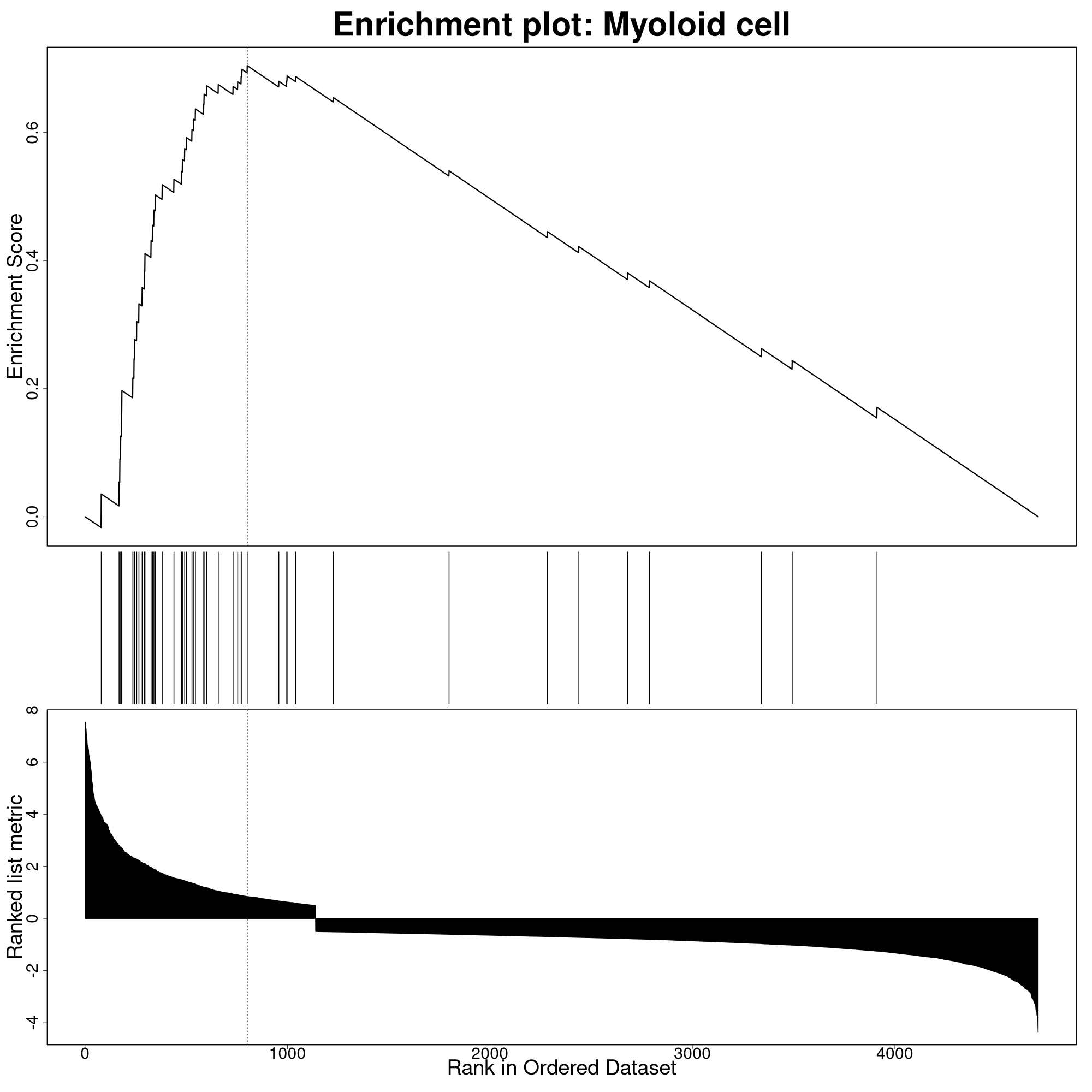

Supplement: Supplementary file 17 — Figure EV5 Source Data [file 44319_2025_631_MOESM17_ESM.zip › Figure EV5/EV5A/GSEA T11b high LUSC vs LUAD/Project_wg_result1731453835/Project_wg_result1731453835_GSEA/Myoloid cell.png]

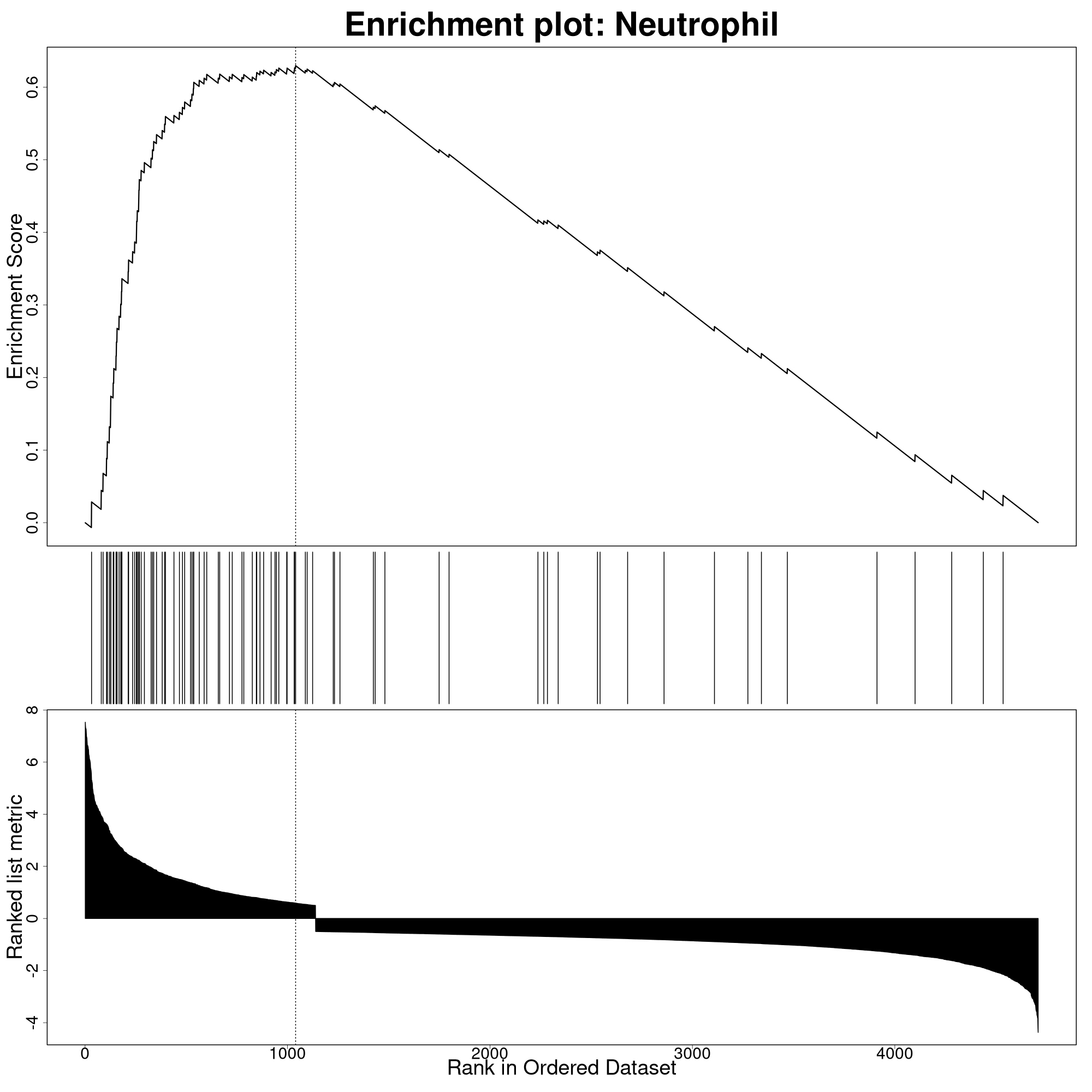

Supplement: Supplementary file 17 — Figure EV5 Source Data [file 44319_2025_631_MOESM17_ESM.zip › Figure EV5/EV5A/GSEA T11b high LUSC vs LUAD/Project_wg_result1731453835/Project_wg_result1731453835_GSEA/Neutrophil.png]

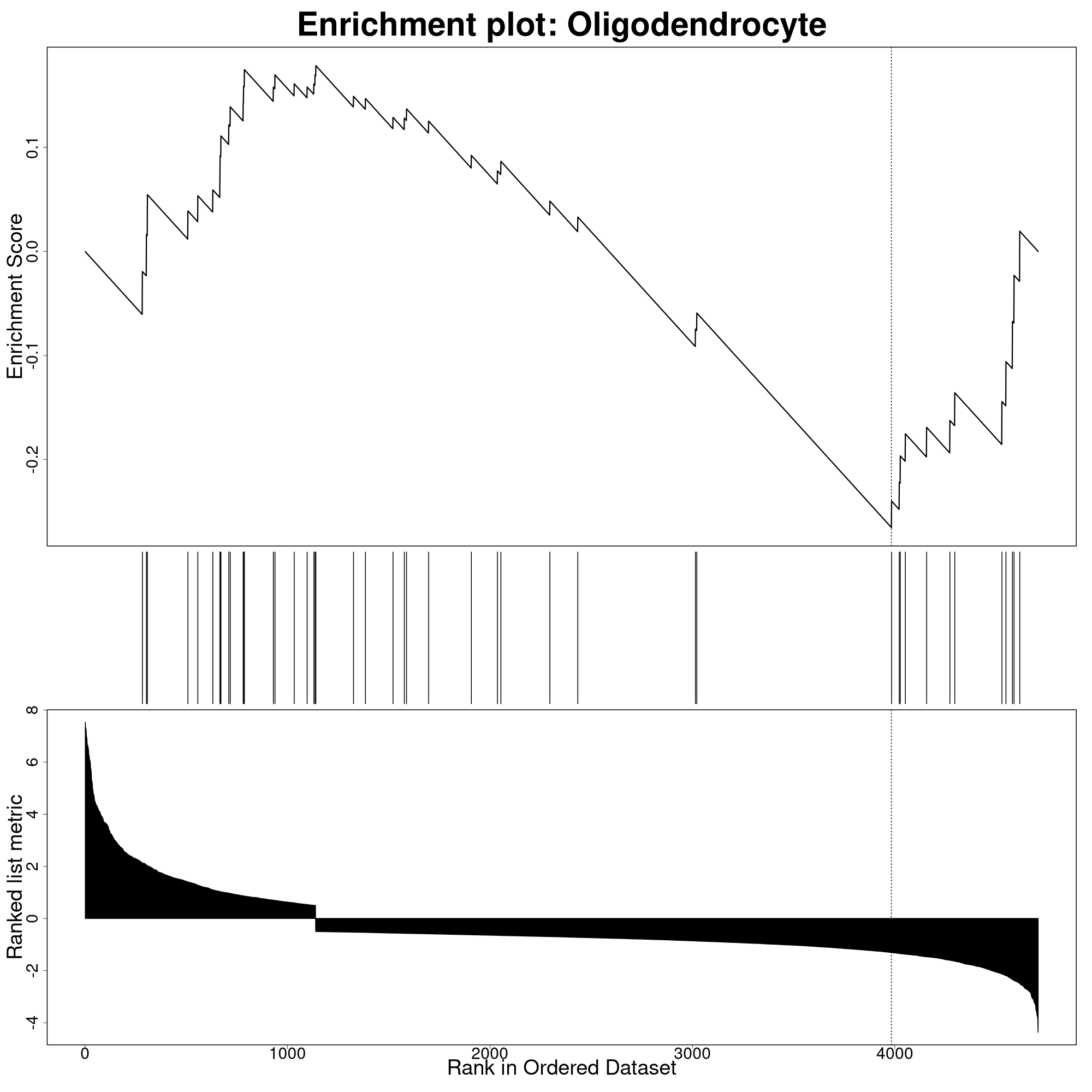

Supplement: Supplementary file 17 — Figure EV5 Source Data [file 44319_2025_631_MOESM17_ESM.zip › Figure EV5/EV5A/GSEA T11b high LUSC vs LUAD/Project_wg_result1731453835/Project_wg_result1731453835_GSEA/Oligodendrocyte.png]

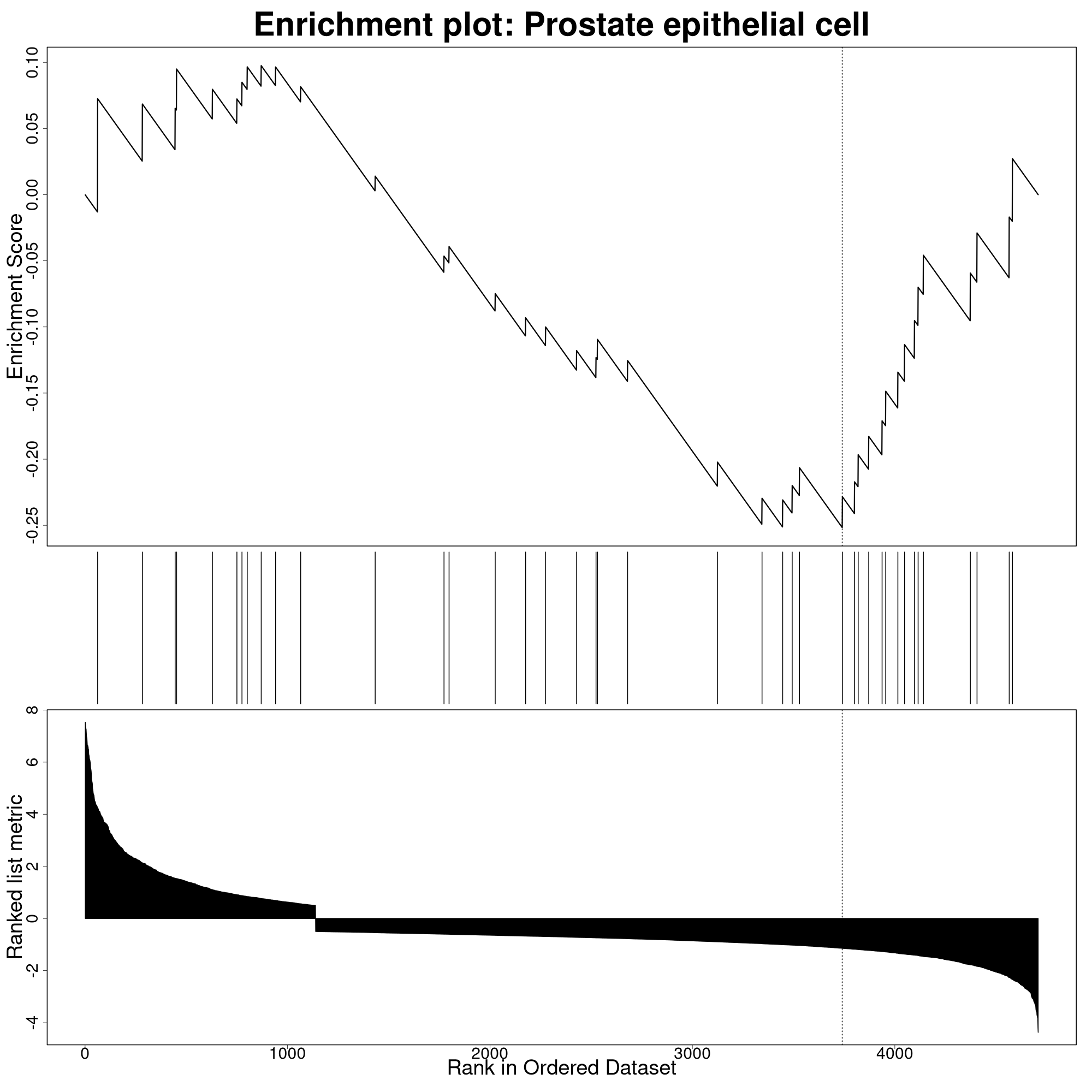

Supplement: Supplementary file 17 — Figure EV5 Source Data [file 44319_2025_631_MOESM17_ESM.zip › Figure EV5/EV5A/GSEA T11b high LUSC vs LUAD/Project_wg_result1731453835/Project_wg_result1731453835_GSEA/Prostate epithelial cell.png]

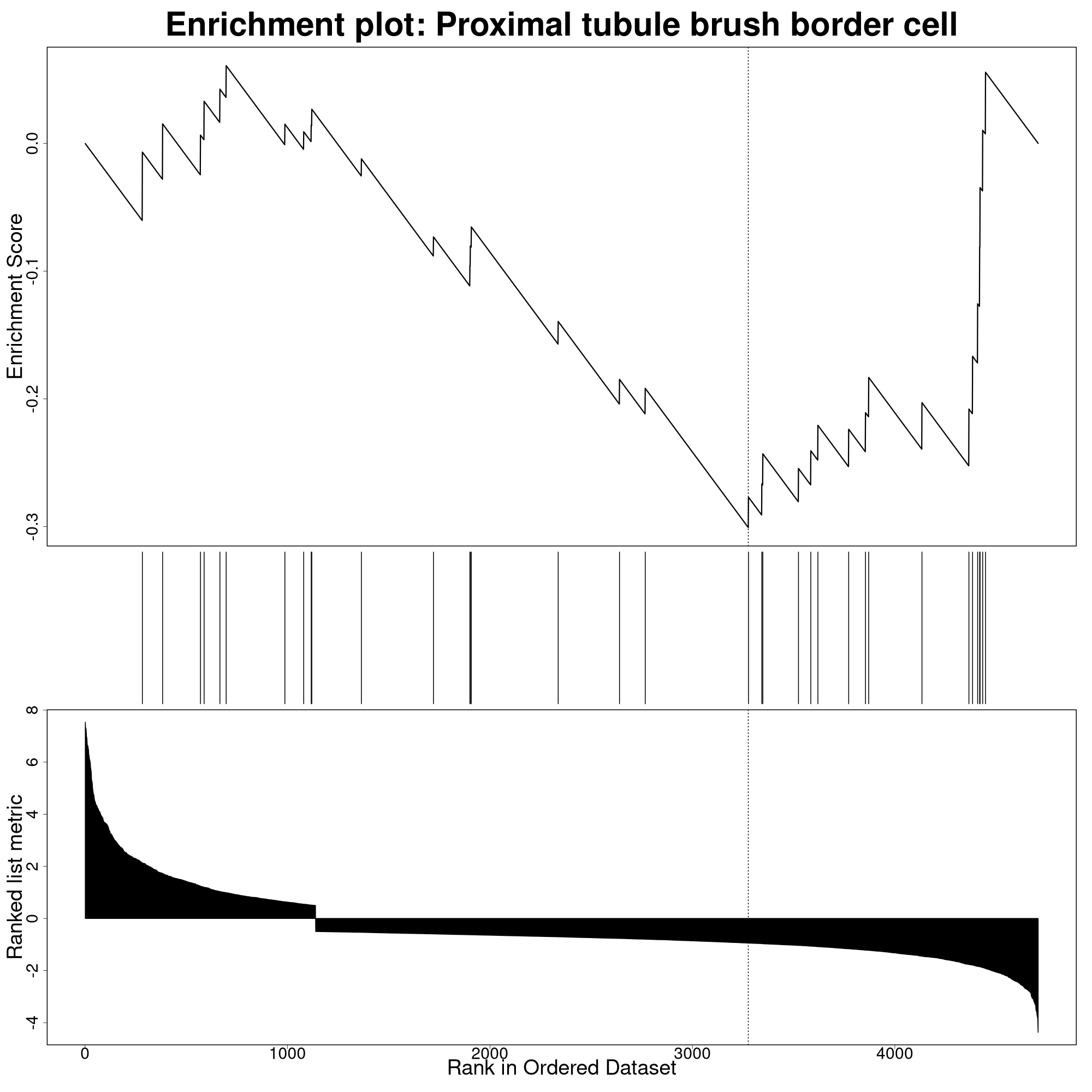

Supplement: Supplementary file 17 — Figure EV5 Source Data [file 44319_2025_631_MOESM17_ESM.zip › Figure EV5/EV5A/GSEA T11b high LUSC vs LUAD/Project_wg_result1731453835/Project_wg_result1731453835_GSEA/Proximal tubule brush border cell.png]

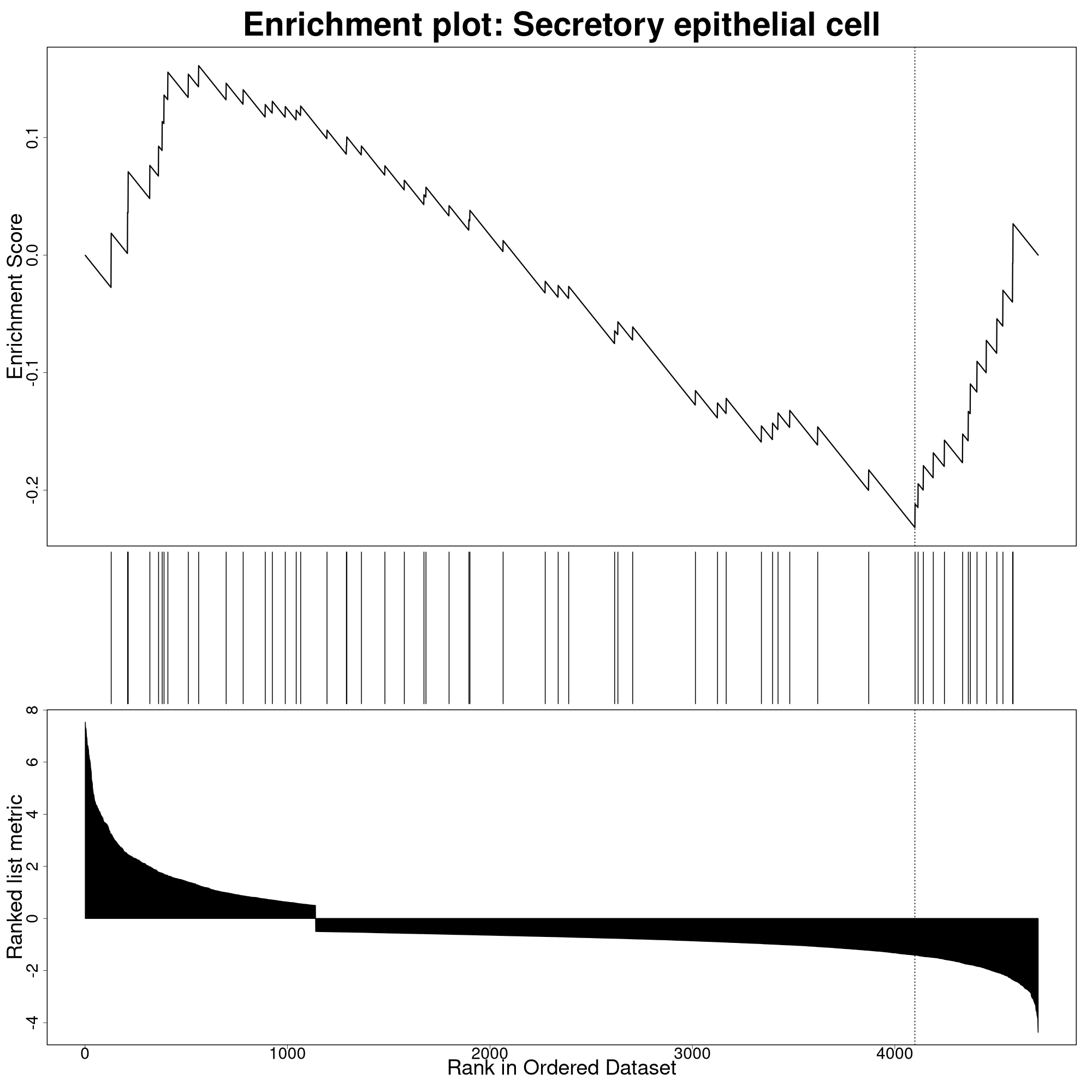

Supplement: Supplementary file 17 — Figure EV5 Source Data [file 44319_2025_631_MOESM17_ESM.zip › Figure EV5/EV5A/GSEA T11b high LUSC vs LUAD/Project_wg_result1731453835/Project_wg_result1731453835_GSEA/Secretory epithelial cell.png]

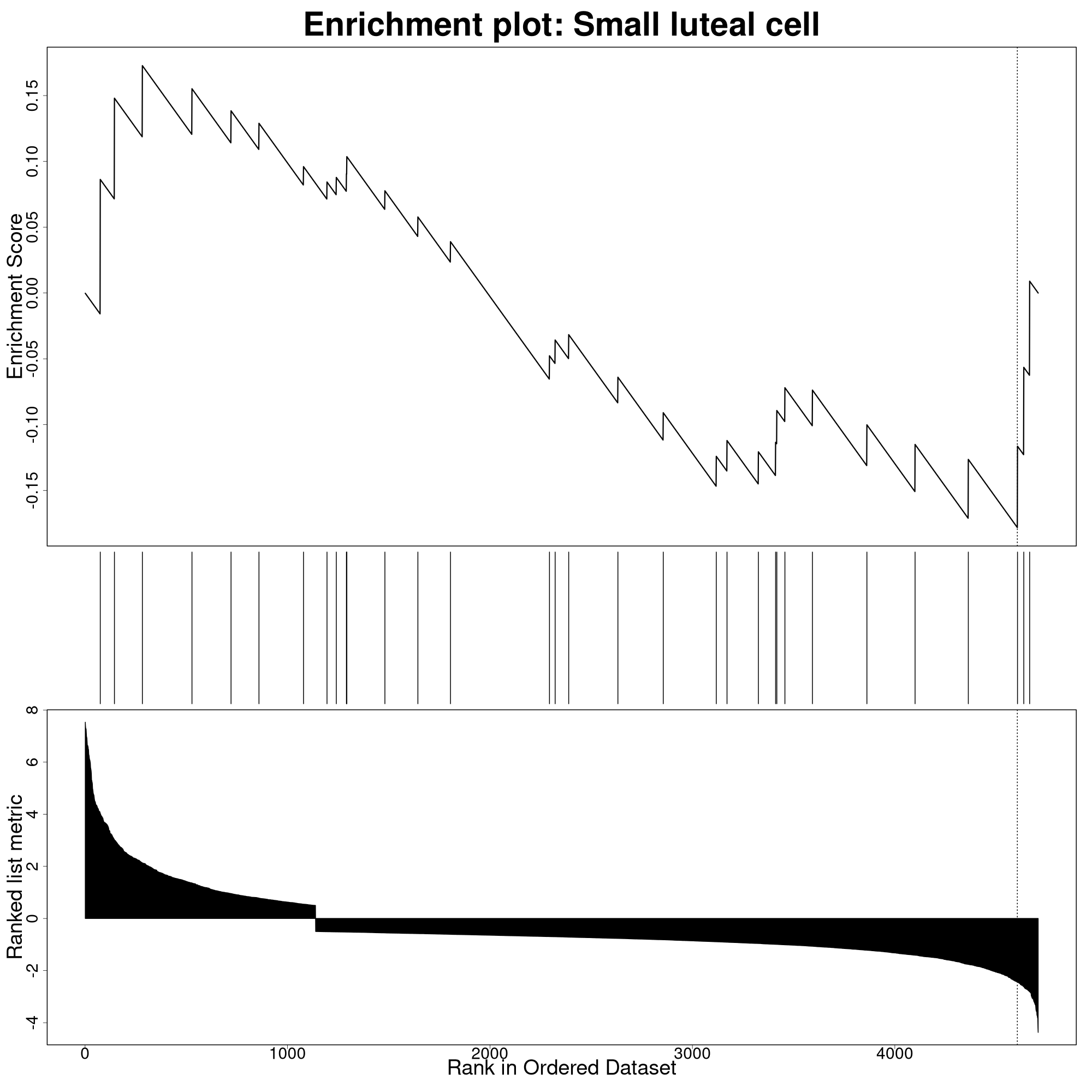

Supplement: Supplementary file 17 — Figure EV5 Source Data [file 44319_2025_631_MOESM17_ESM.zip › Figure EV5/EV5A/GSEA T11b high LUSC vs LUAD/Project_wg_result1731453835/Project_wg_result1731453835_GSEA/Small luteal cell.png]

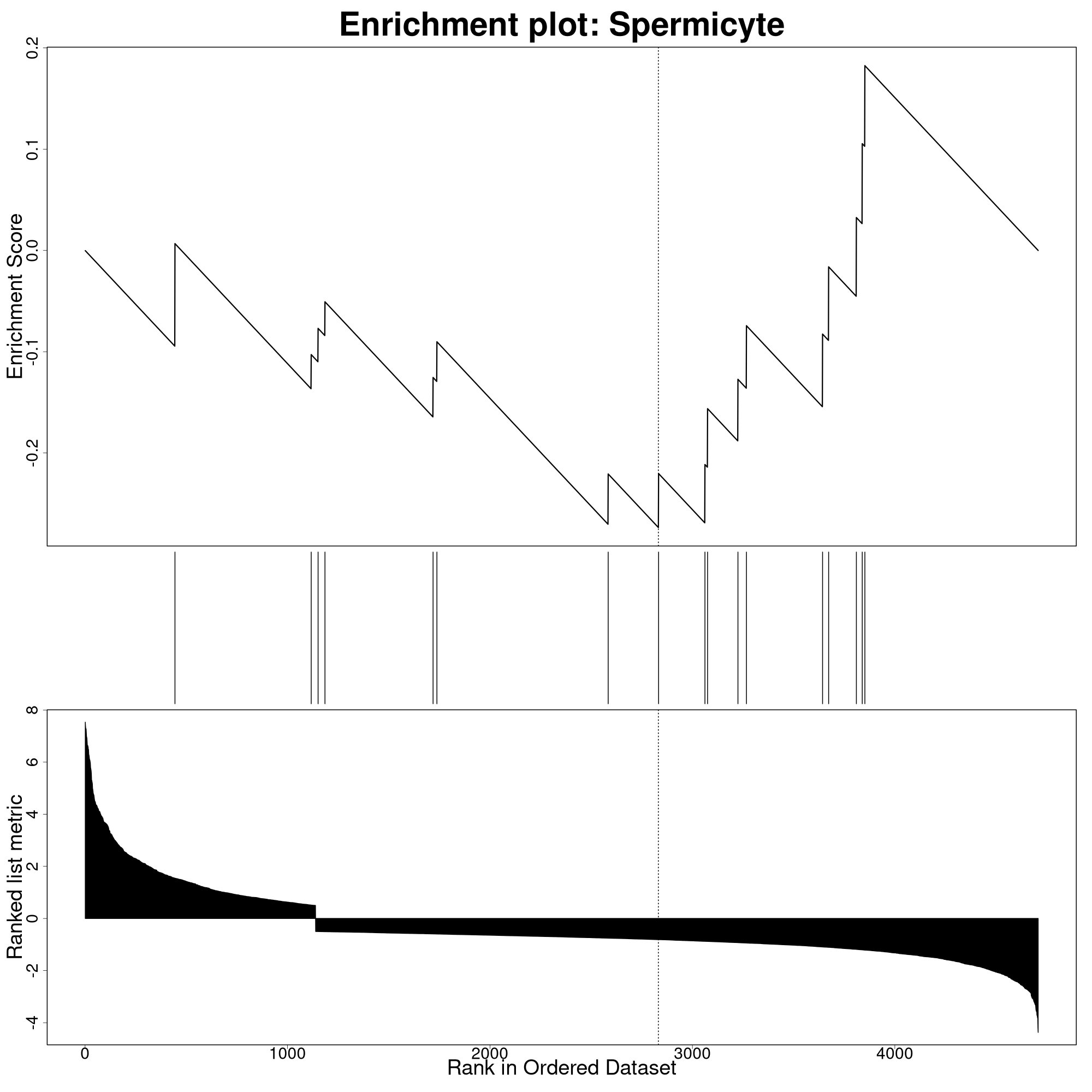

Supplement: Supplementary file 17 — Figure EV5 Source Data [file 44319_2025_631_MOESM17_ESM.zip › Figure EV5/EV5A/GSEA T11b high LUSC vs LUAD/Project_wg_result1731453835/Project_wg_result1731453835_GSEA/Spermicyte.png]

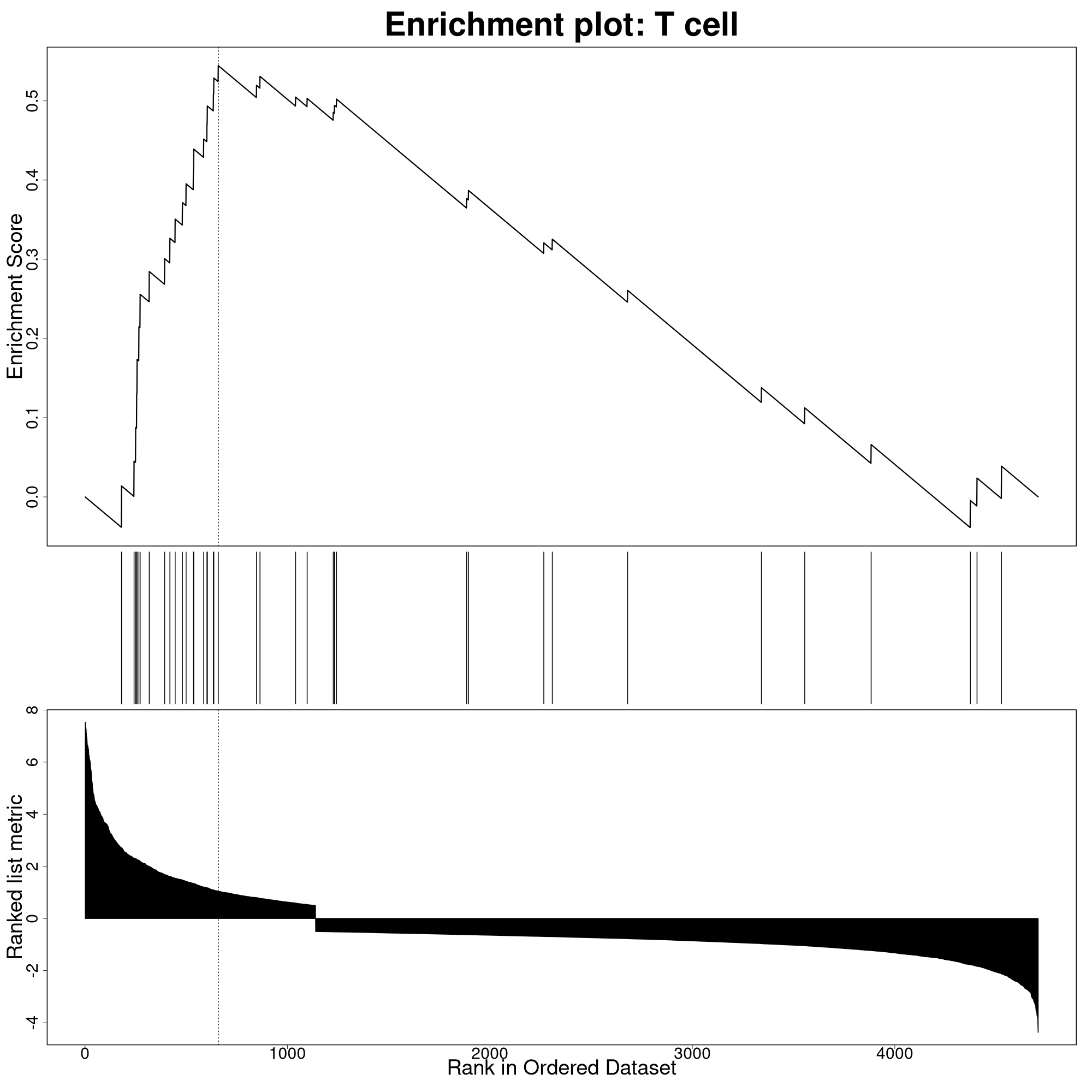

Supplement: Supplementary file 17 — Figure EV5 Source Data [file 44319_2025_631_MOESM17_ESM.zip › Figure EV5/EV5A/GSEA T11b high LUSC vs LUAD/Project_wg_result1731453835/Project_wg_result1731453835_GSEA/T cell.png]

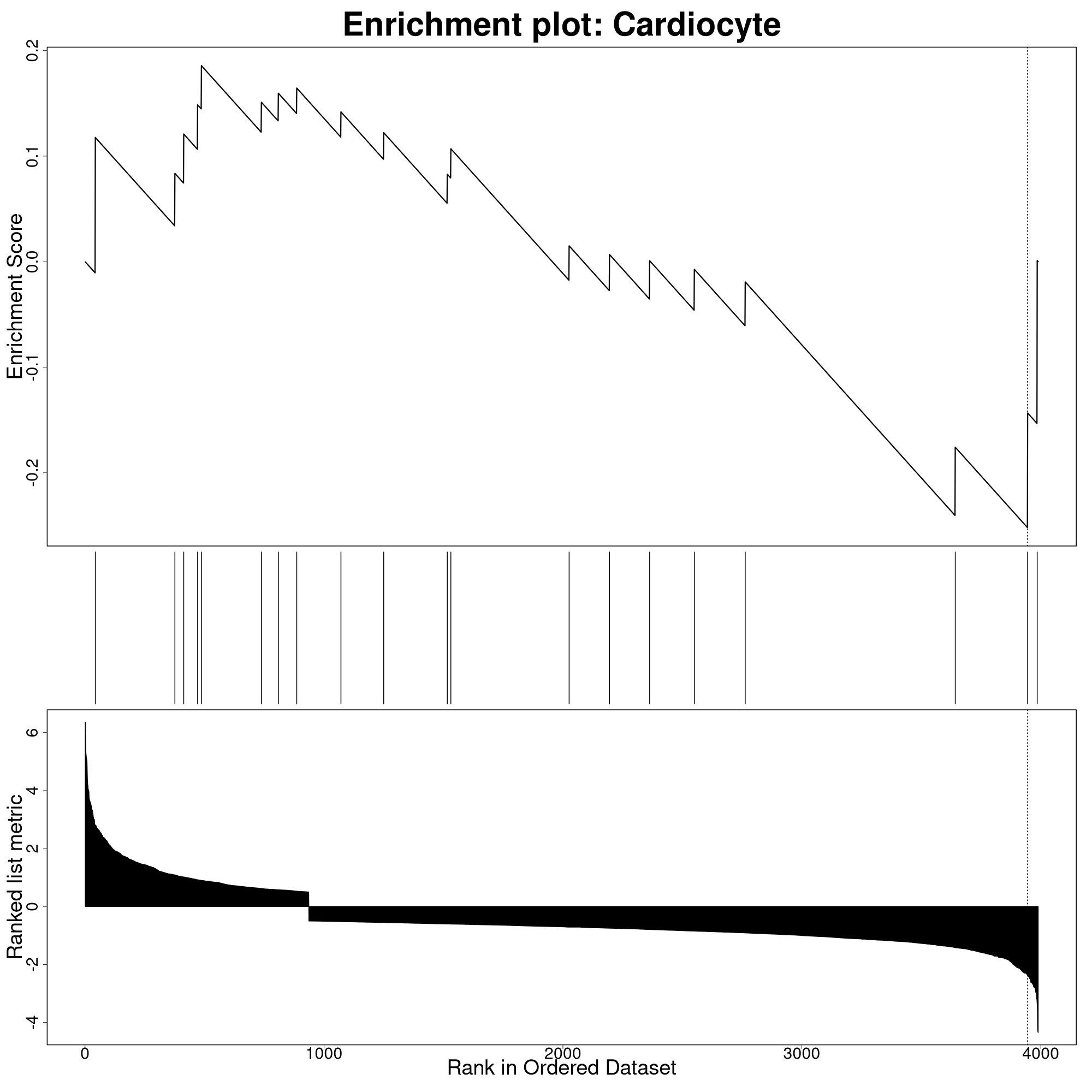

Supplement: Supplementary file 17 — Figure EV5 Source Data [file 44319_2025_631_MOESM17_ESM.zip › Figure EV5/EV5A/GSEA T11b high LUSC vs T11b low LUSC/Project_wg_result1729116948/Project_wg_result1729116948_GSEA/Cardiocyte.png]

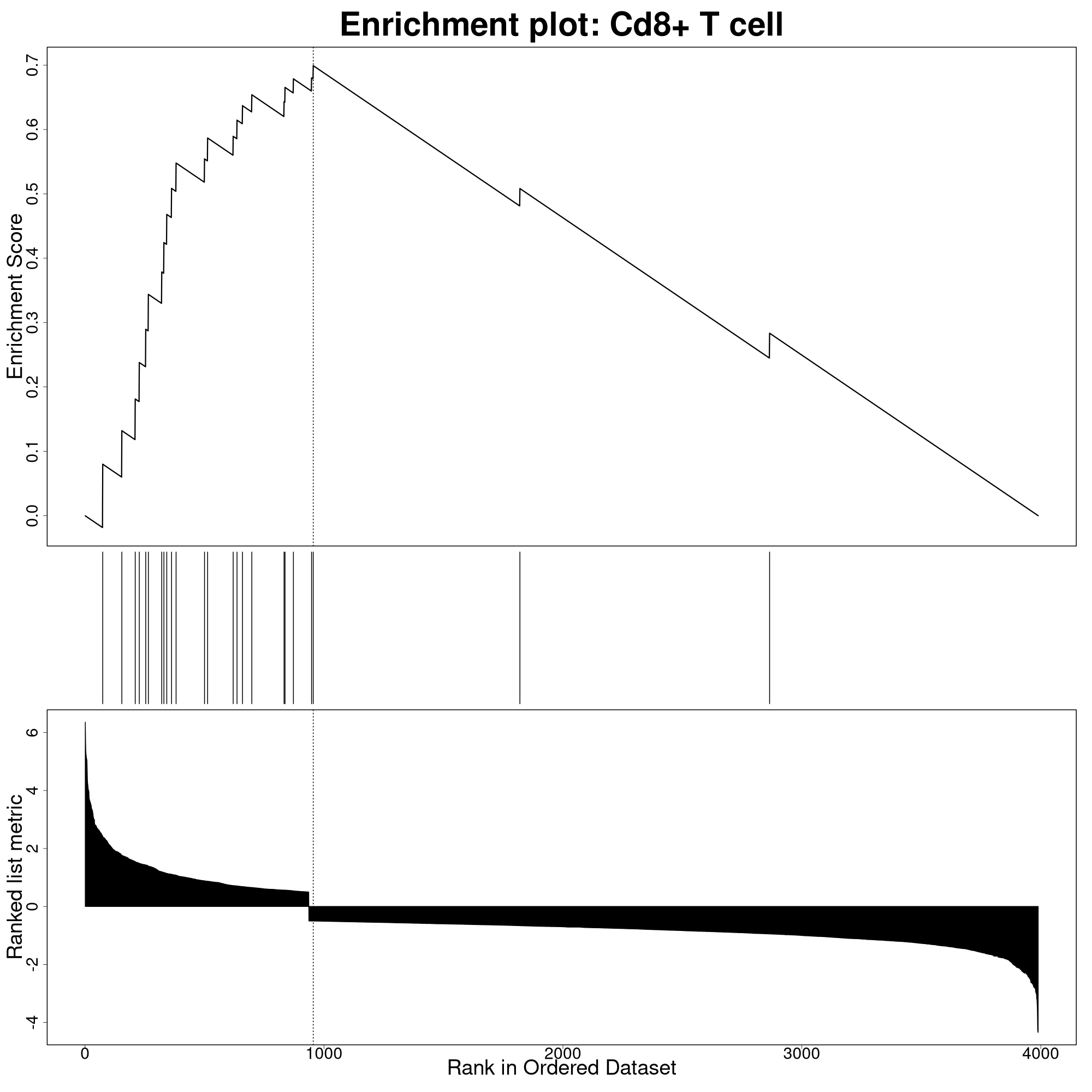

Supplement: Supplementary file 17 — Figure EV5 Source Data [file 44319_2025_631_MOESM17_ESM.zip › Figure EV5/EV5A/GSEA T11b high LUSC vs T11b low LUSC/Project_wg_result1729116948/Project_wg_result1729116948_GSEA/Cd8_ T cell.png]

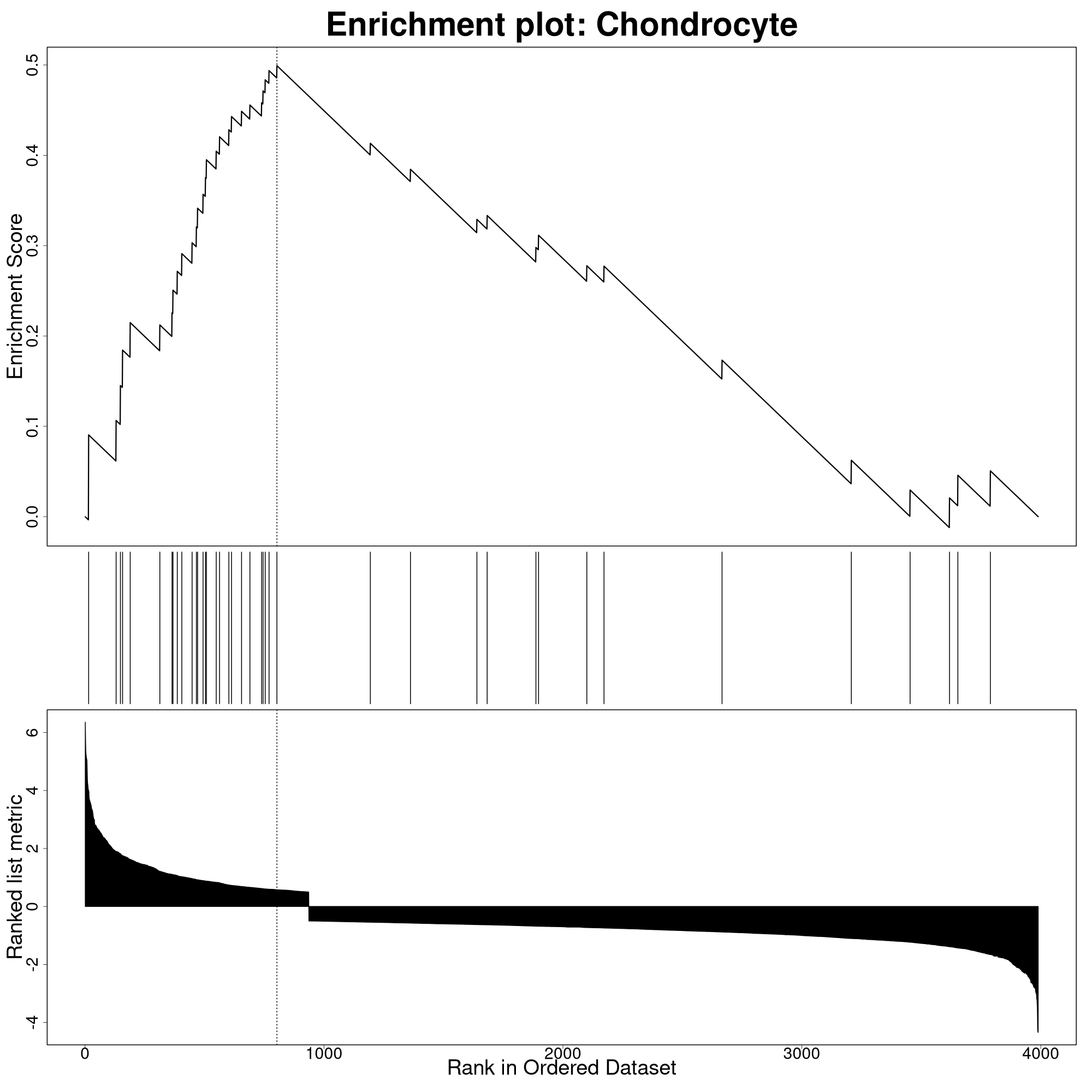

Supplement: Supplementary file 17 — Figure EV5 Source Data [file 44319_2025_631_MOESM17_ESM.zip › Figure EV5/EV5A/GSEA T11b high LUSC vs T11b low LUSC/Project_wg_result1729116948/Project_wg_result1729116948_GSEA/Chondrocyte.png]

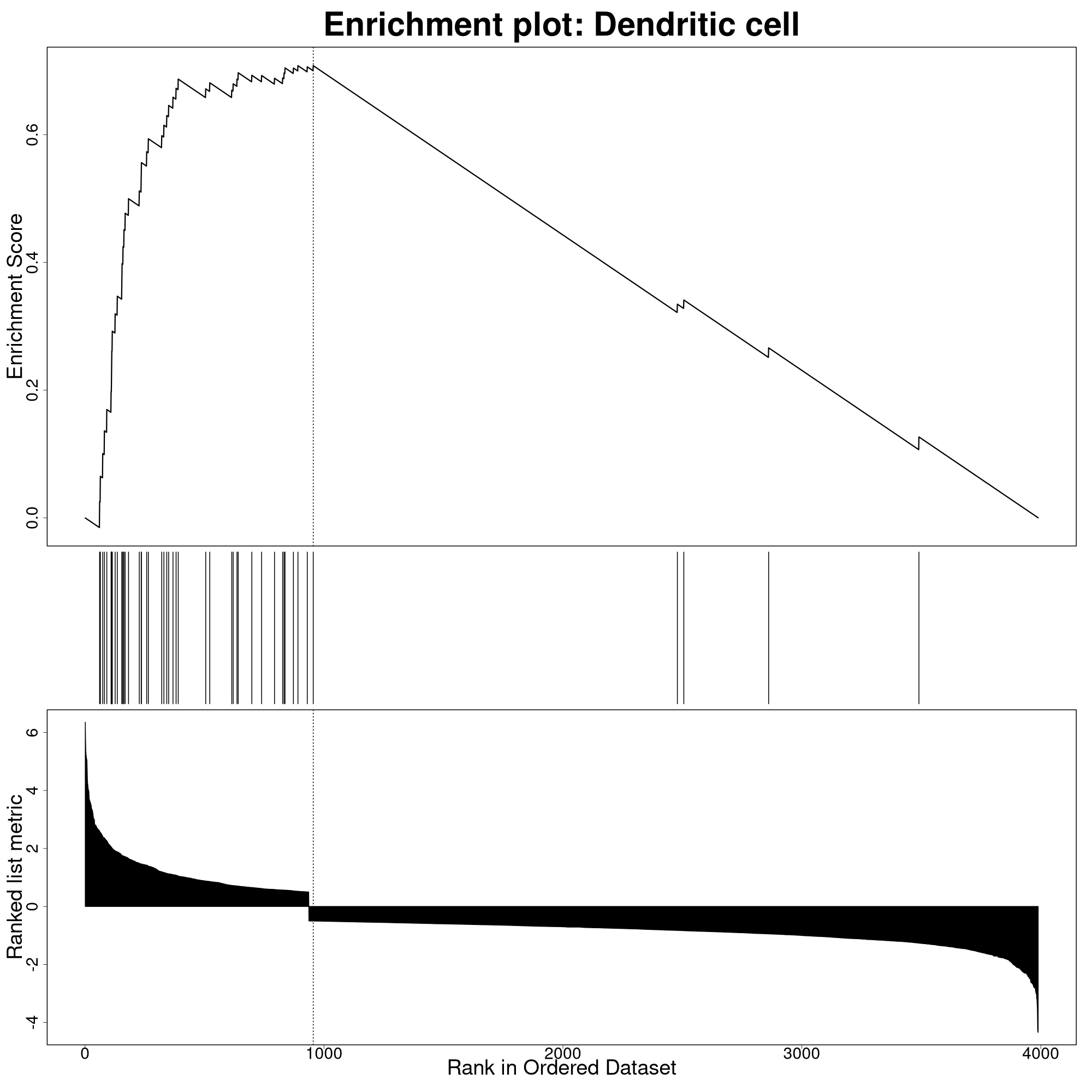

Supplement: Supplementary file 17 — Figure EV5 Source Data [file 44319_2025_631_MOESM17_ESM.zip › Figure EV5/EV5A/GSEA T11b high LUSC vs T11b low LUSC/Project_wg_result1729116948/Project_wg_result1729116948_GSEA/Dendritic cell.png]

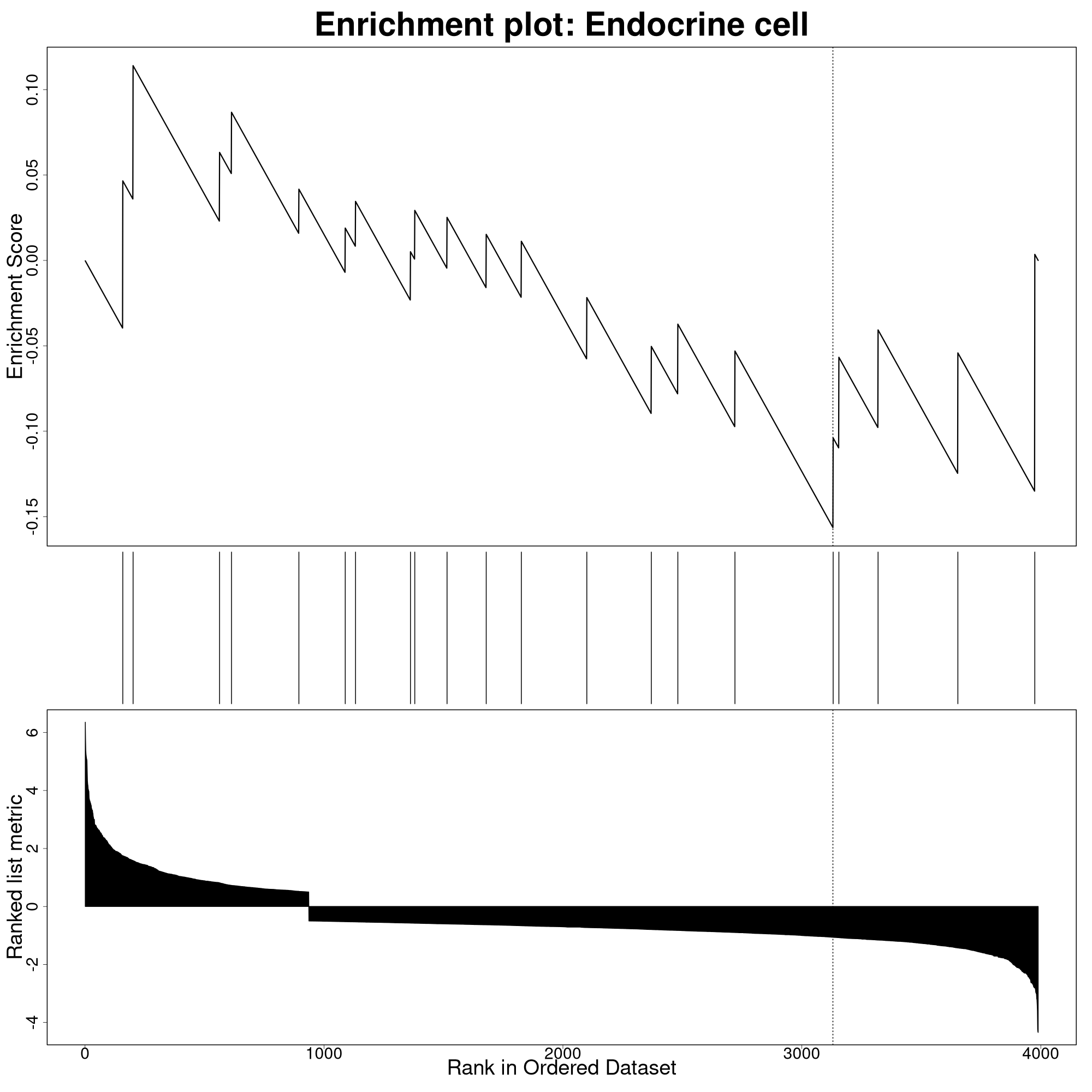

Supplement: Supplementary file 17 — Figure EV5 Source Data [file 44319_2025_631_MOESM17_ESM.zip › Figure EV5/EV5A/GSEA T11b high LUSC vs T11b low LUSC/Project_wg_result1729116948/Project_wg_result1729116948_GSEA/Endocrine cell.png]

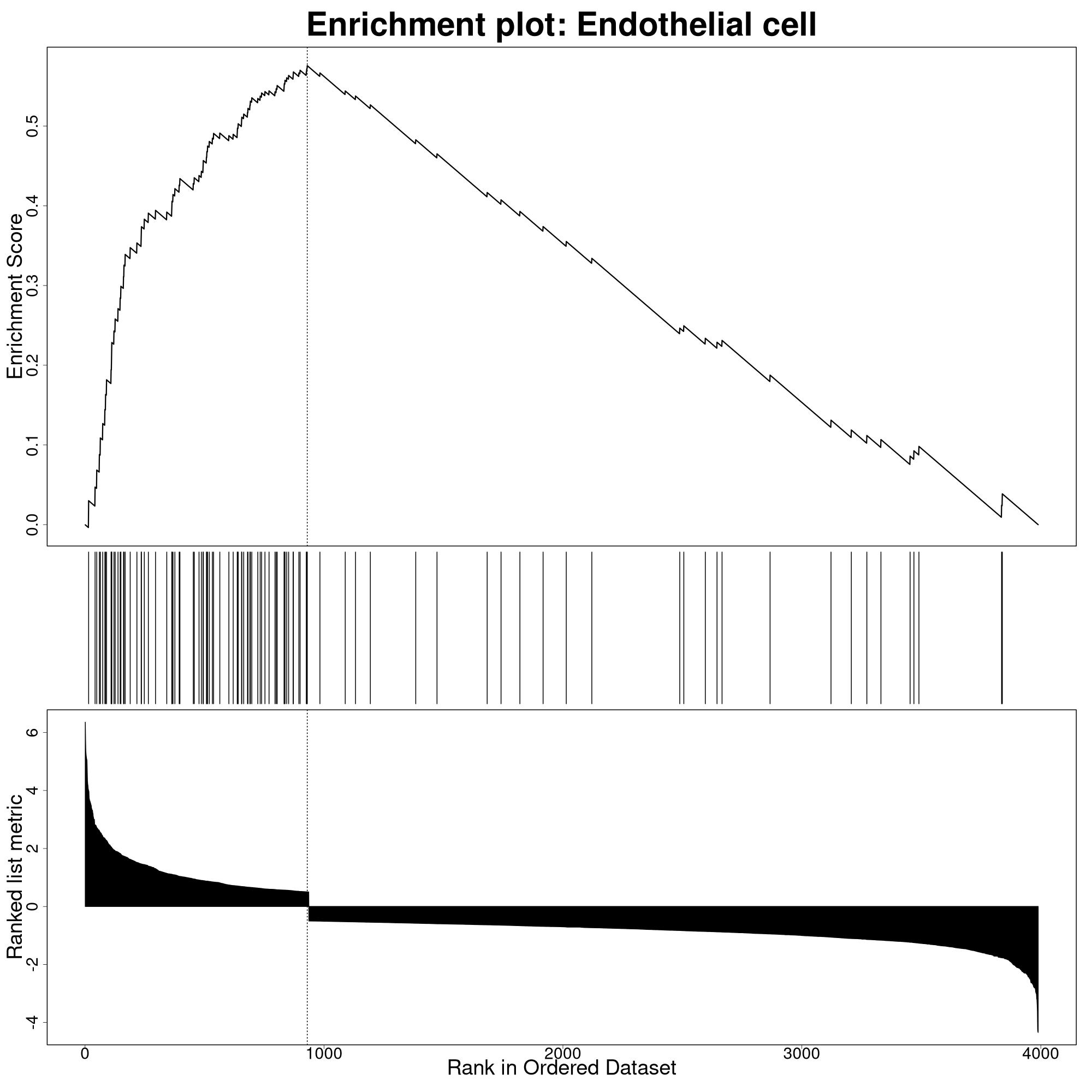

Supplement: Supplementary file 17 — Figure EV5 Source Data [file 44319_2025_631_MOESM17_ESM.zip › Figure EV5/EV5A/GSEA T11b high LUSC vs T11b low LUSC/Project_wg_result1729116948/Project_wg_result1729116948_GSEA/Endothelial cell.png]

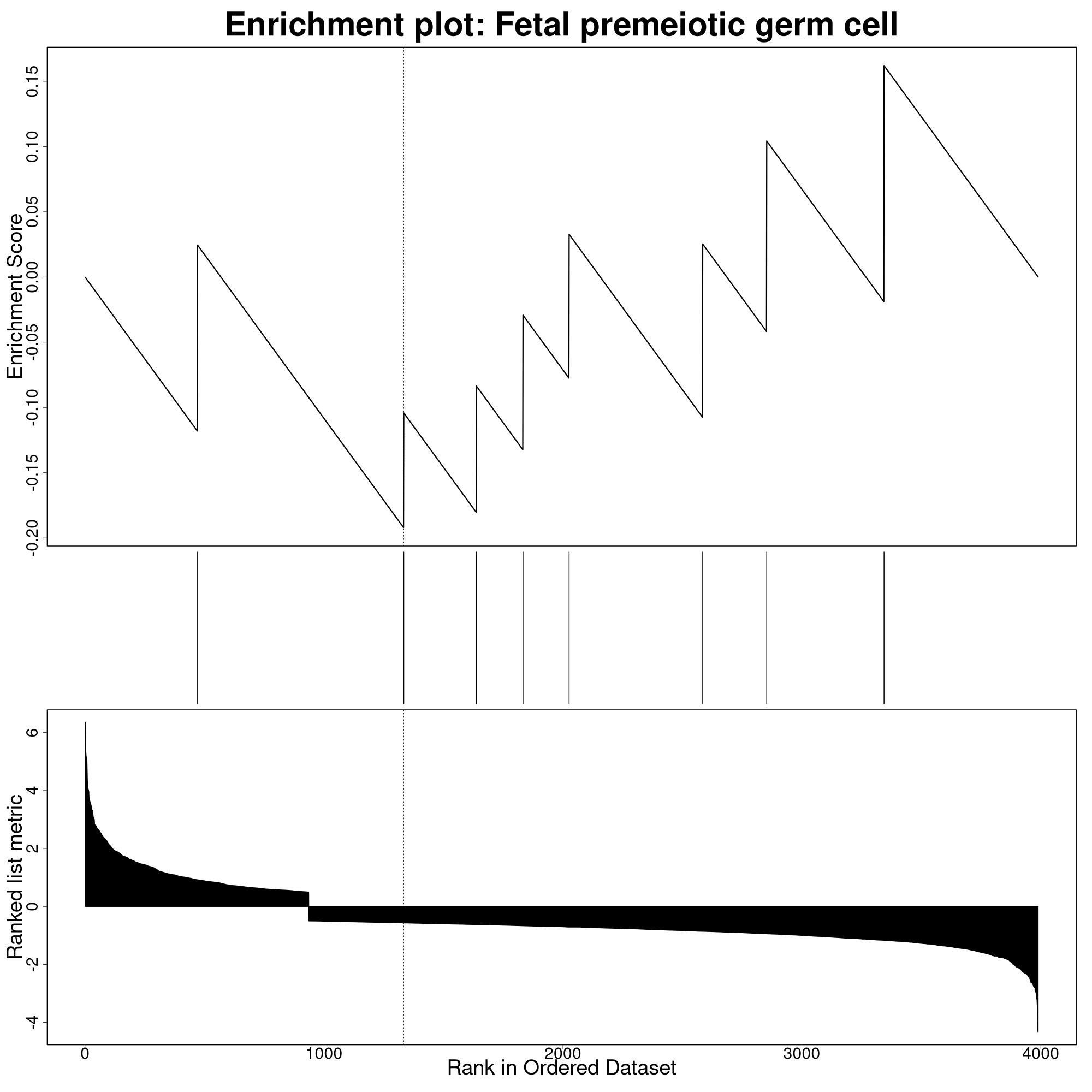

Supplement: Supplementary file 17 — Figure EV5 Source Data [file 44319_2025_631_MOESM17_ESM.zip › Figure EV5/EV5A/GSEA T11b high LUSC vs T11b low LUSC/Project_wg_result1729116948/Project_wg_result1729116948_GSEA/Fetal premeiotic germ cell.png]

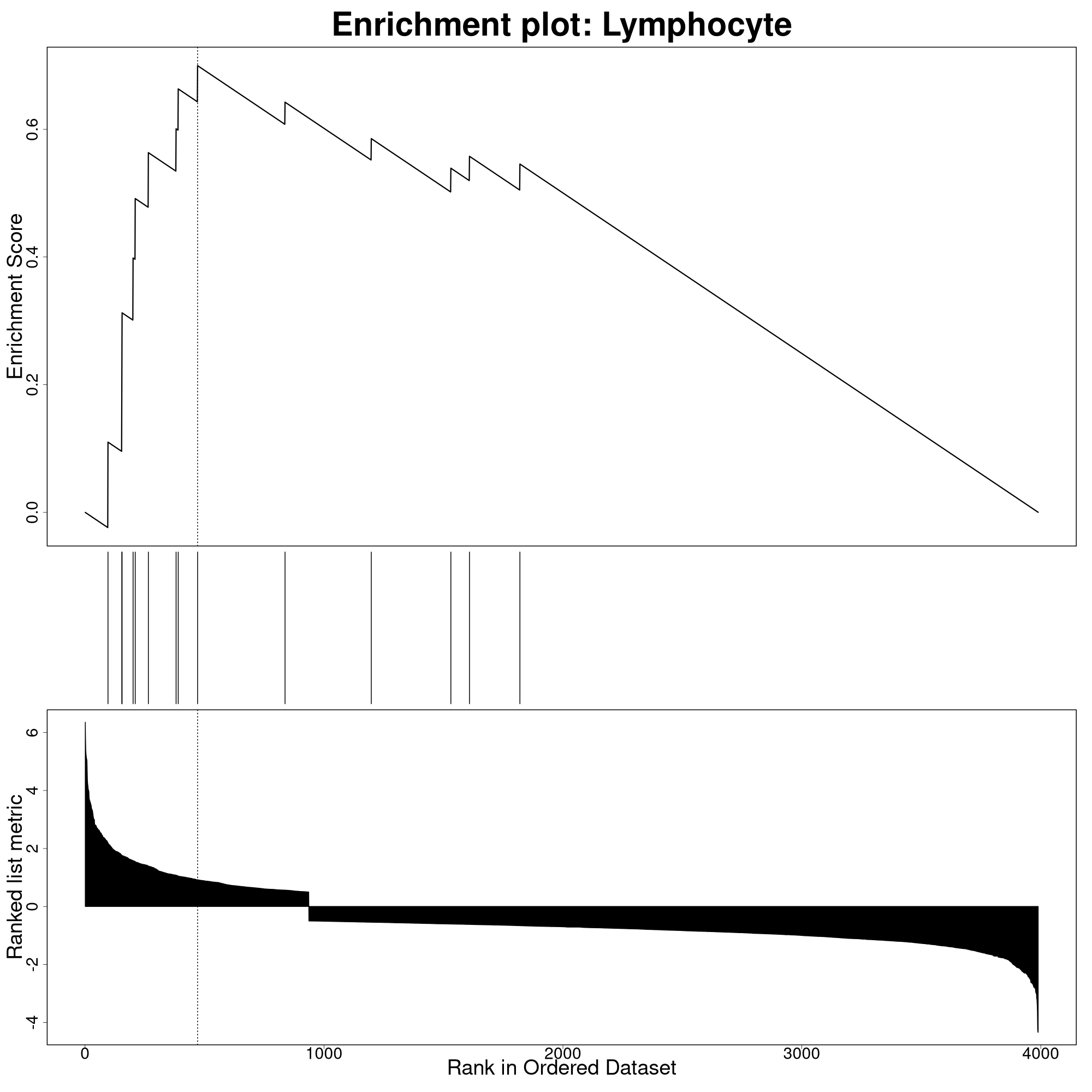

Supplement: Supplementary file 17 — Figure EV5 Source Data [file 44319_2025_631_MOESM17_ESM.zip › Figure EV5/EV5A/GSEA T11b high LUSC vs T11b low LUSC/Project_wg_result1729116948/Project_wg_result1729116948_GSEA/Lymphocyte.png]

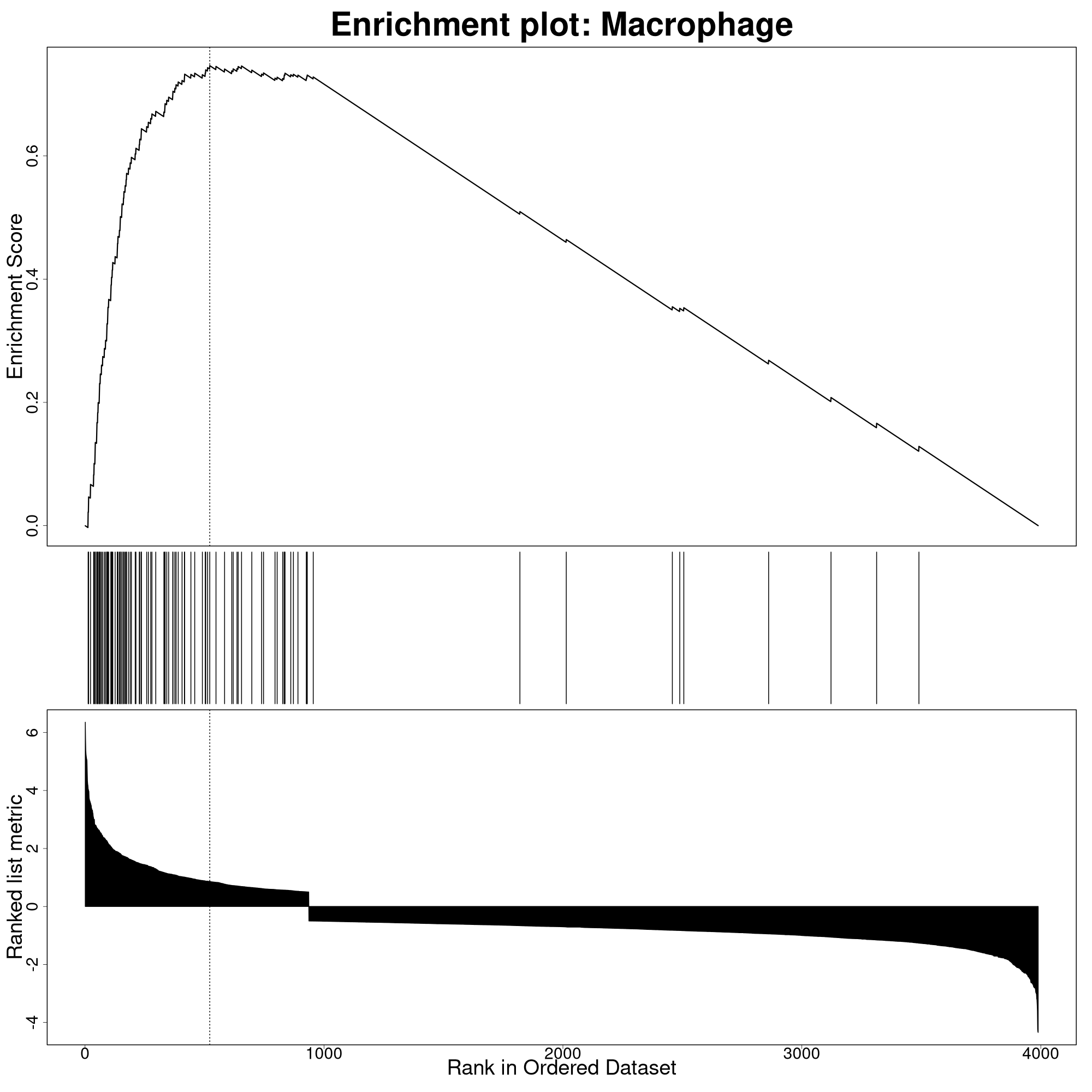

Supplement: Supplementary file 17 — Figure EV5 Source Data [file 44319_2025_631_MOESM17_ESM.zip › Figure EV5/EV5A/GSEA T11b high LUSC vs T11b low LUSC/Project_wg_result1729116948/Project_wg_result1729116948_GSEA/Macrophage.png]

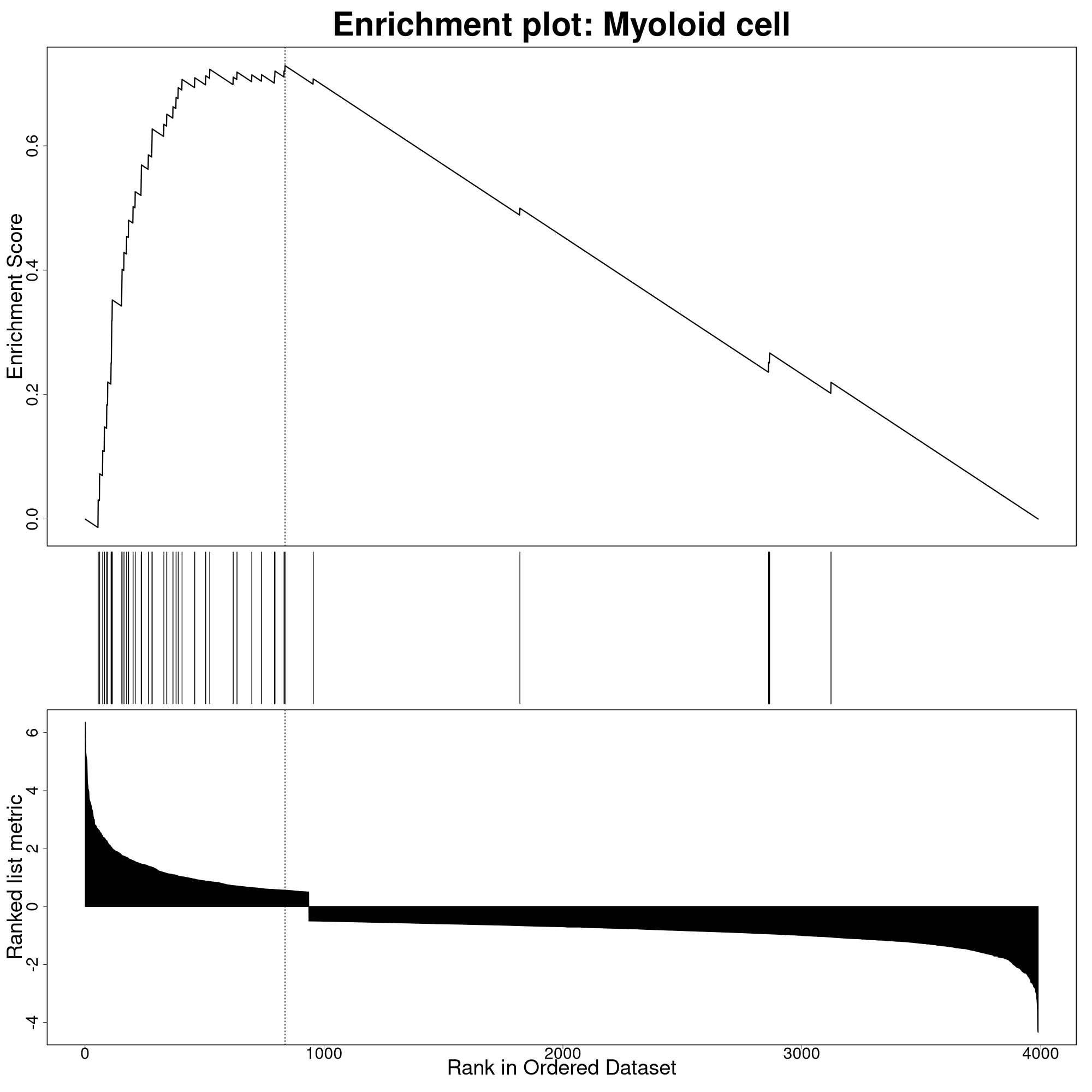

Supplement: Supplementary file 17 — Figure EV5 Source Data [file 44319_2025_631_MOESM17_ESM.zip › Figure EV5/EV5A/GSEA T11b high LUSC vs T11b low LUSC/Project_wg_result1729116948/Project_wg_result1729116948_GSEA/Myoloid cell.png]

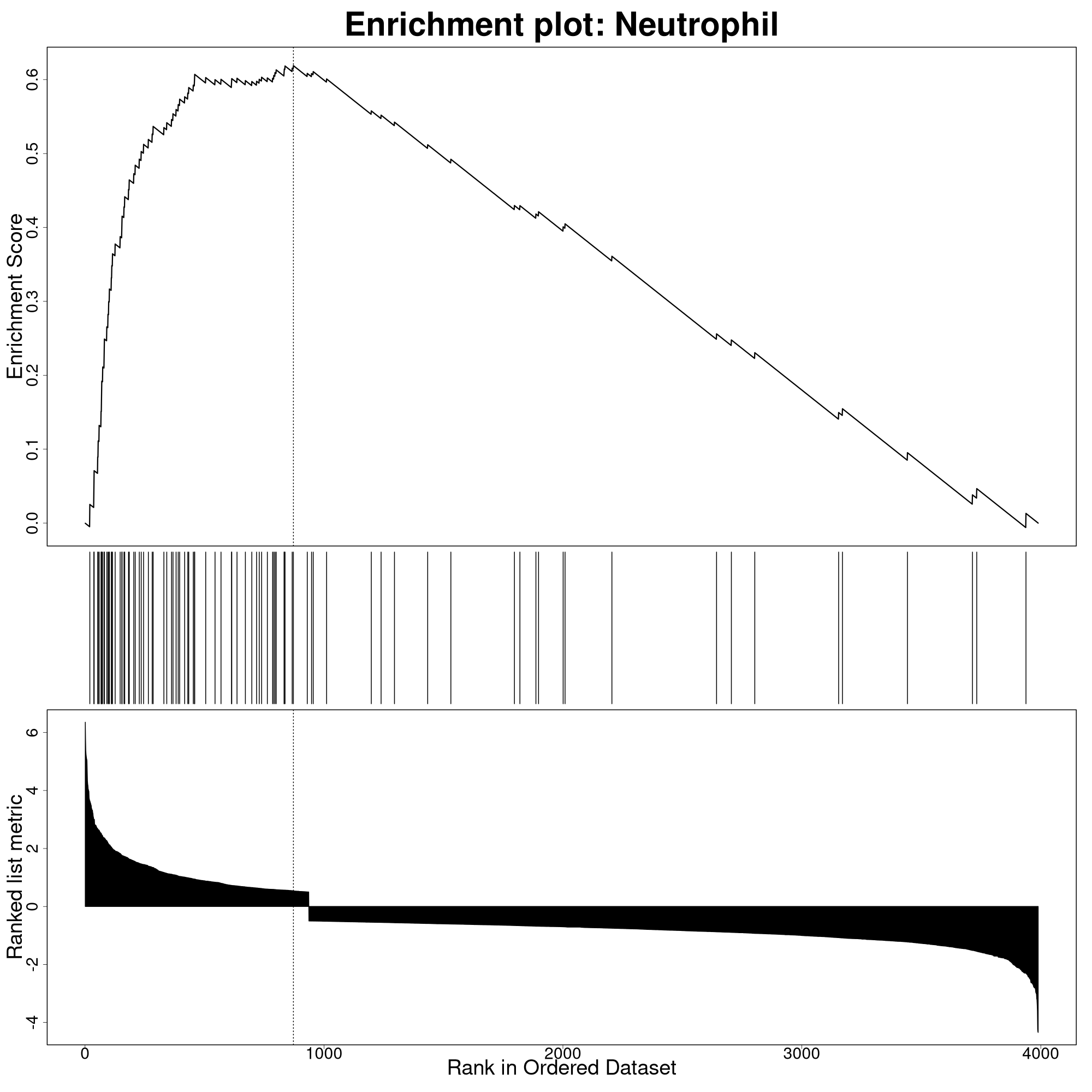

Supplement: Supplementary file 17 — Figure EV5 Source Data [file 44319_2025_631_MOESM17_ESM.zip › Figure EV5/EV5A/GSEA T11b high LUSC vs T11b low LUSC/Project_wg_result1729116948/Project_wg_result1729116948_GSEA/Neutrophil.png]

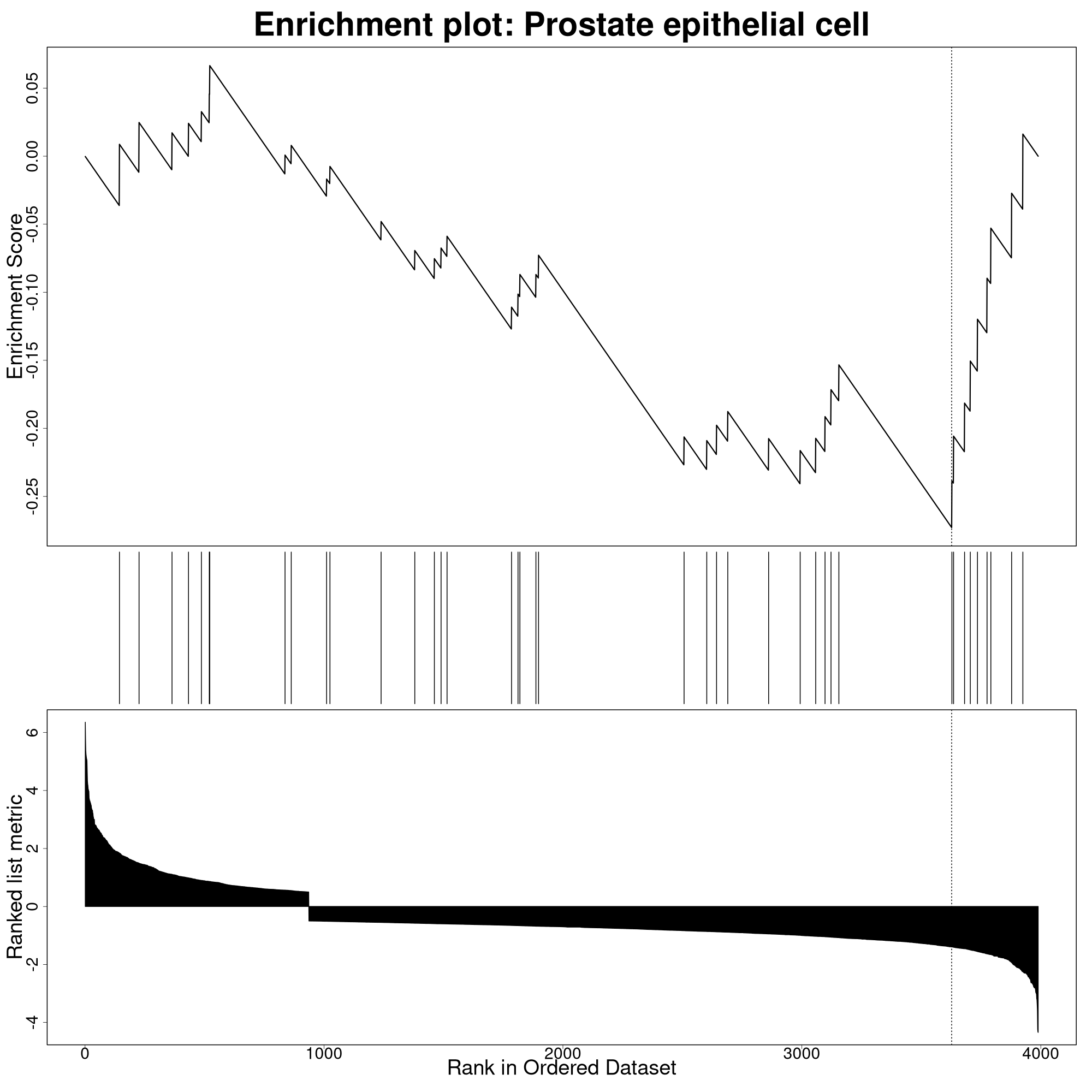

Supplement: Supplementary file 17 — Figure EV5 Source Data [file 44319_2025_631_MOESM17_ESM.zip › Figure EV5/EV5A/GSEA T11b high LUSC vs T11b low LUSC/Project_wg_result1729116948/Project_wg_result1729116948_GSEA/Prostate epithelial cell.png]

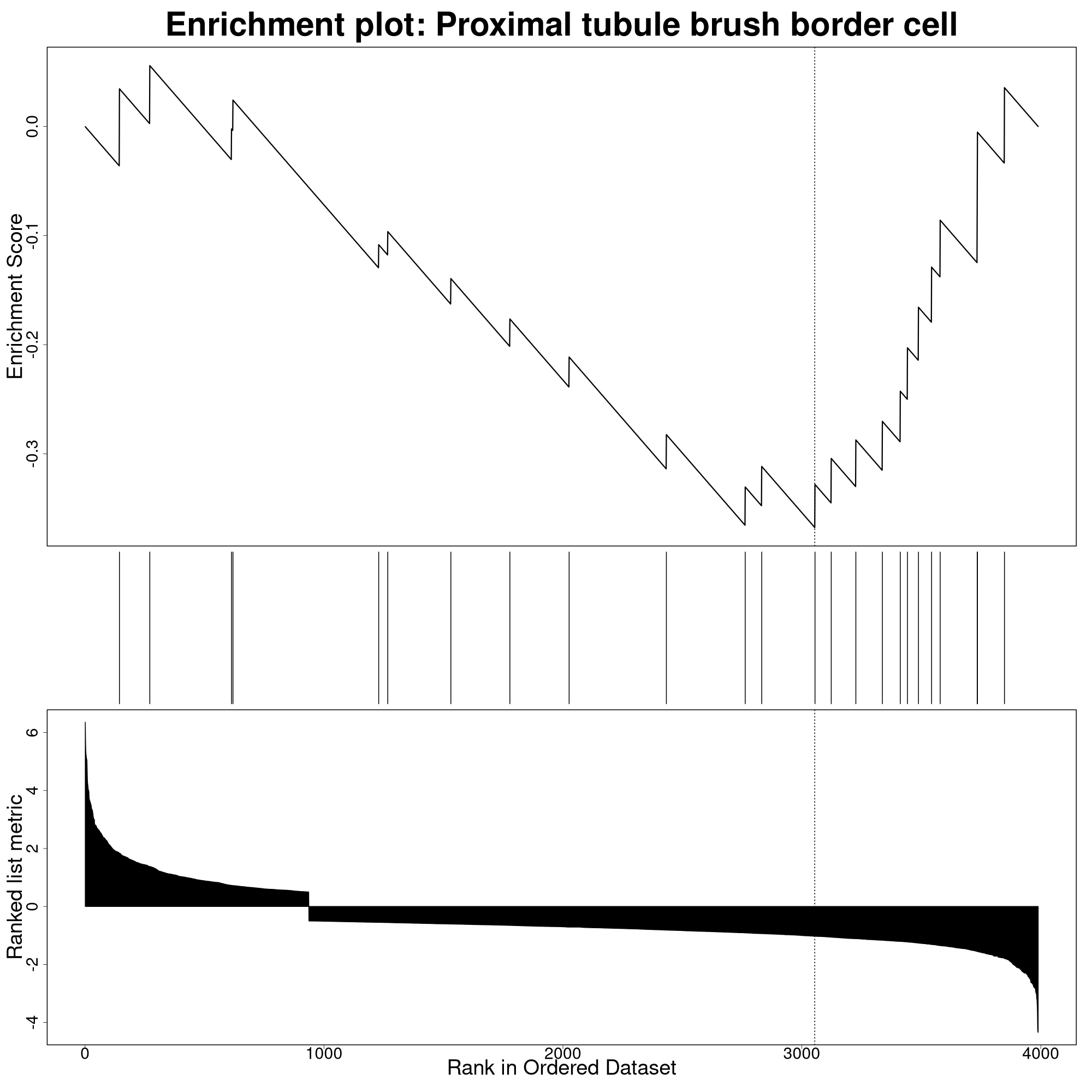

Supplement: Supplementary file 17 — Figure EV5 Source Data [file 44319_2025_631_MOESM17_ESM.zip › Figure EV5/EV5A/GSEA T11b high LUSC vs T11b low LUSC/Project_wg_result1729116948/Project_wg_result1729116948_GSEA/Proximal tubule brush border cell.png]

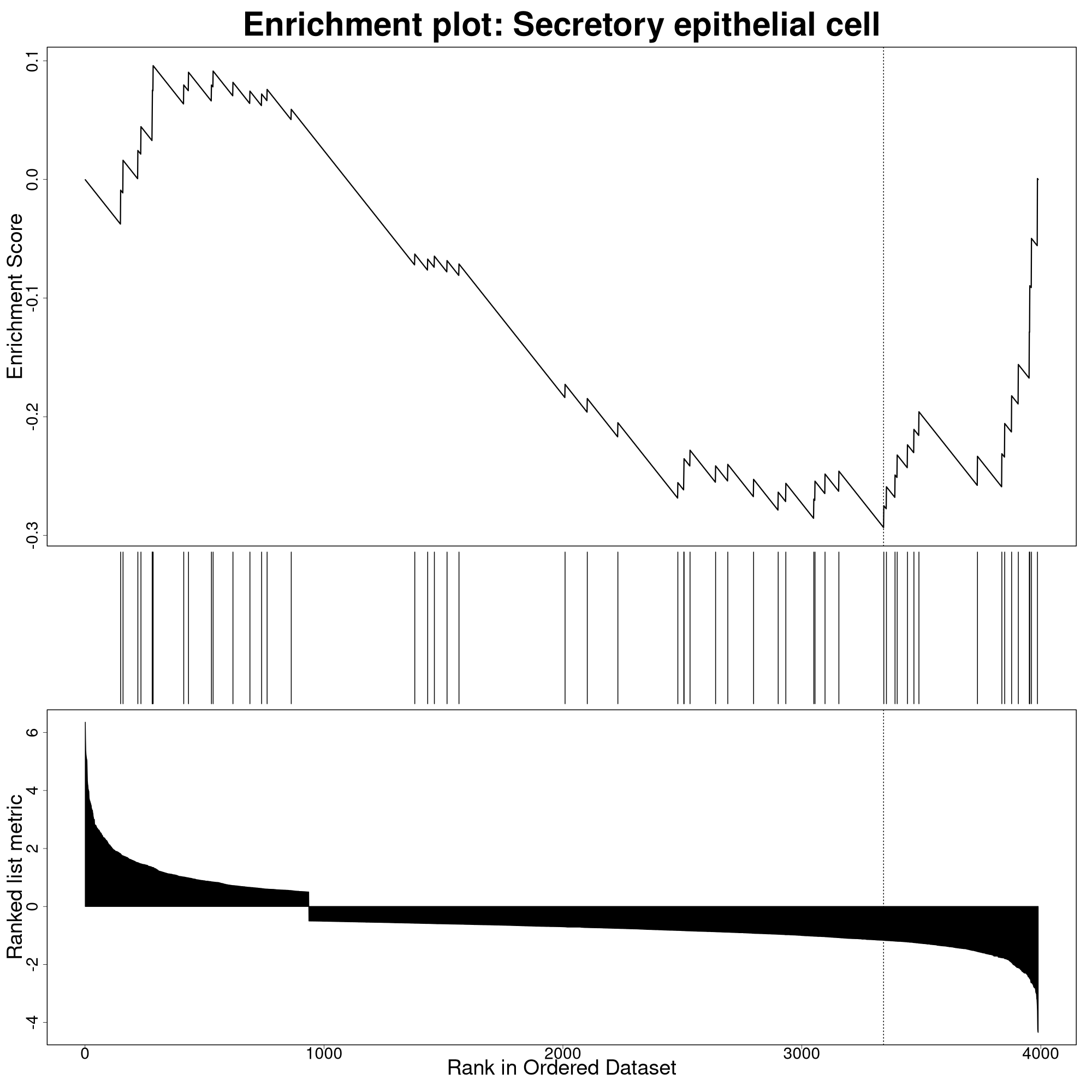

Supplement: Supplementary file 17 — Figure EV5 Source Data [file 44319_2025_631_MOESM17_ESM.zip › Figure EV5/EV5A/GSEA T11b high LUSC vs T11b low LUSC/Project_wg_result1729116948/Project_wg_result1729116948_GSEA/Secretory epithelial cell.png]

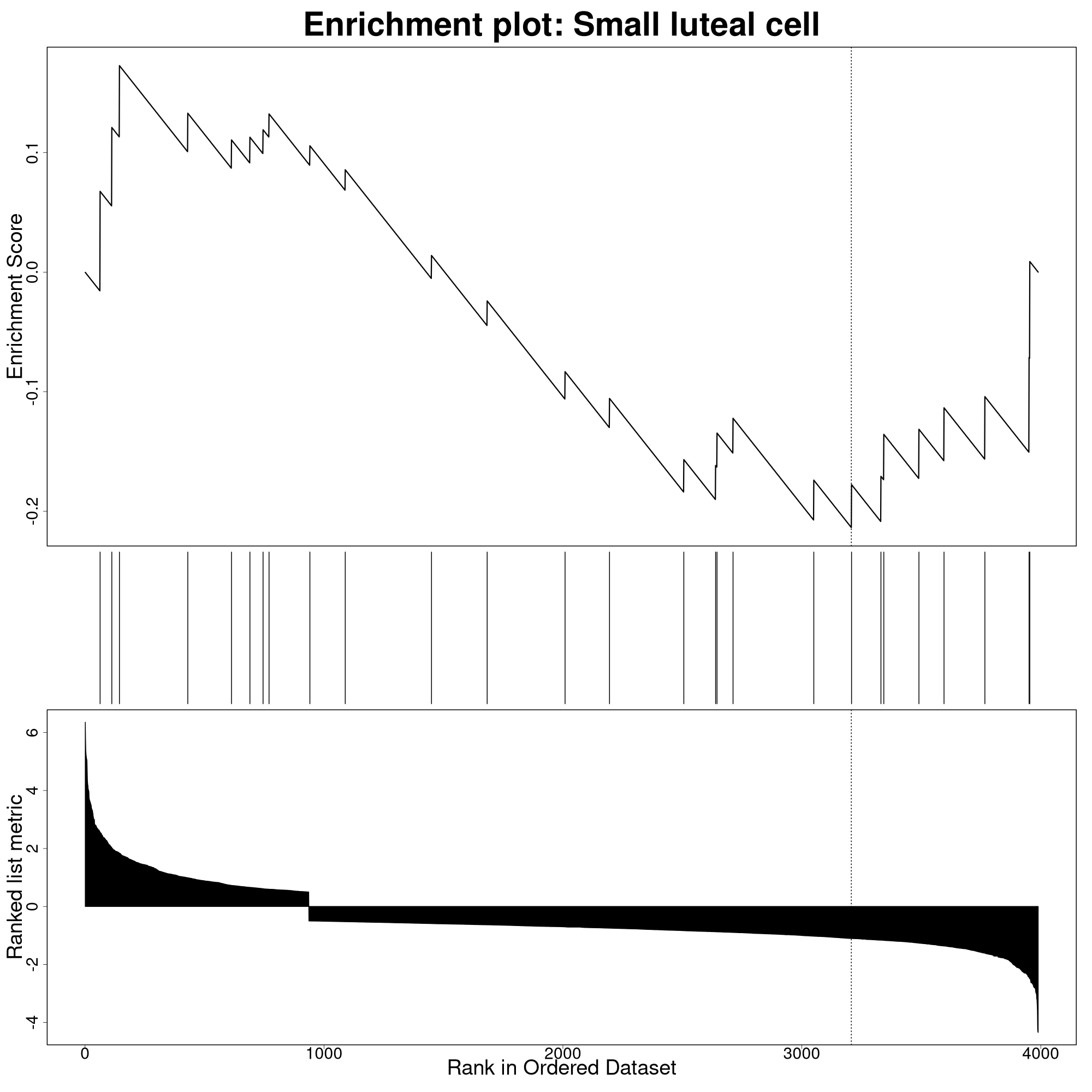

Supplement: Supplementary file 17 — Figure EV5 Source Data [file 44319_2025_631_MOESM17_ESM.zip › Figure EV5/EV5A/GSEA T11b high LUSC vs T11b low LUSC/Project_wg_result1729116948/Project_wg_result1729116948_GSEA/Small luteal cell.png]

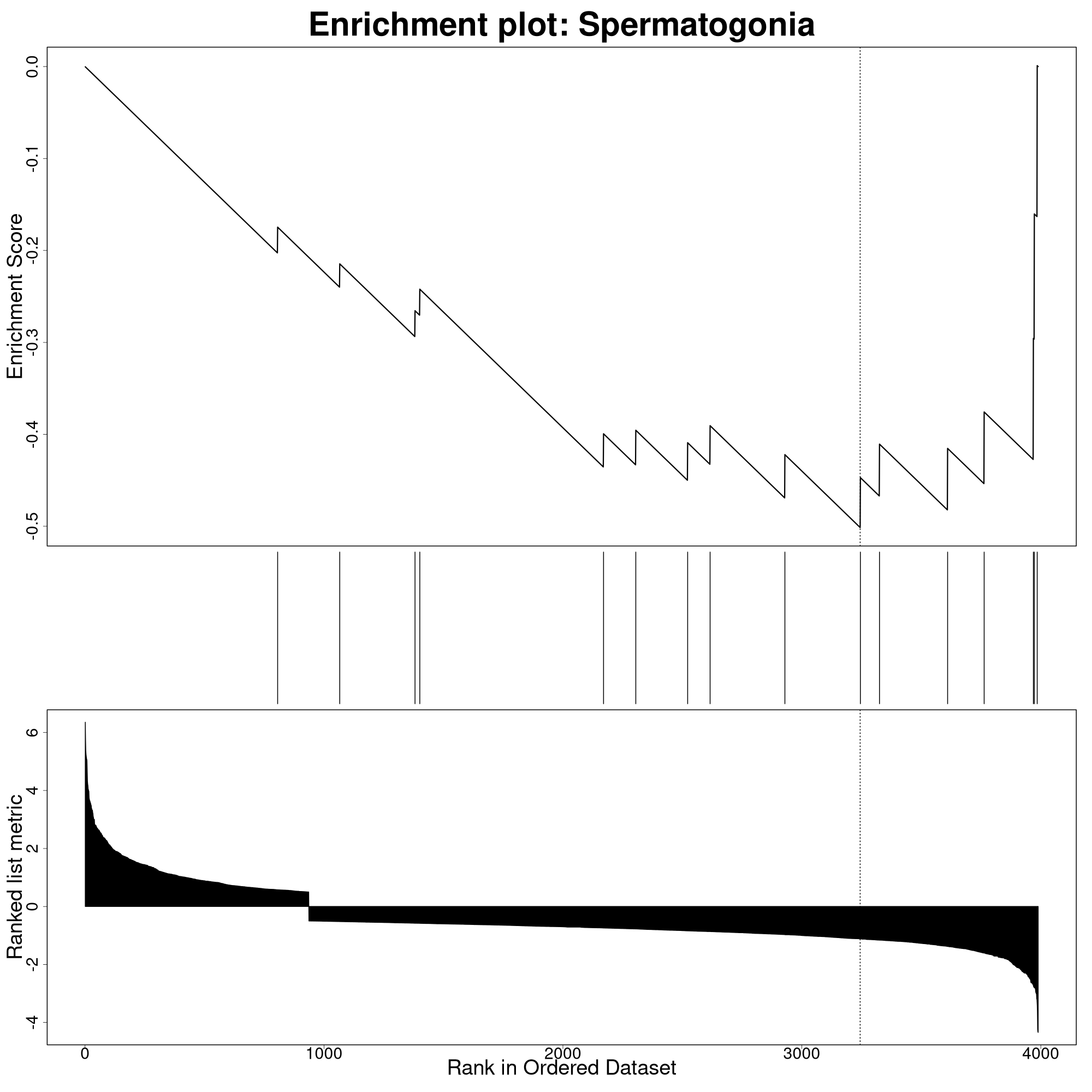

Supplement: Supplementary file 17 — Figure EV5 Source Data [file 44319_2025_631_MOESM17_ESM.zip › Figure EV5/EV5A/GSEA T11b high LUSC vs T11b low LUSC/Project_wg_result1729116948/Project_wg_result1729116948_GSEA/Spermatogonia.png]

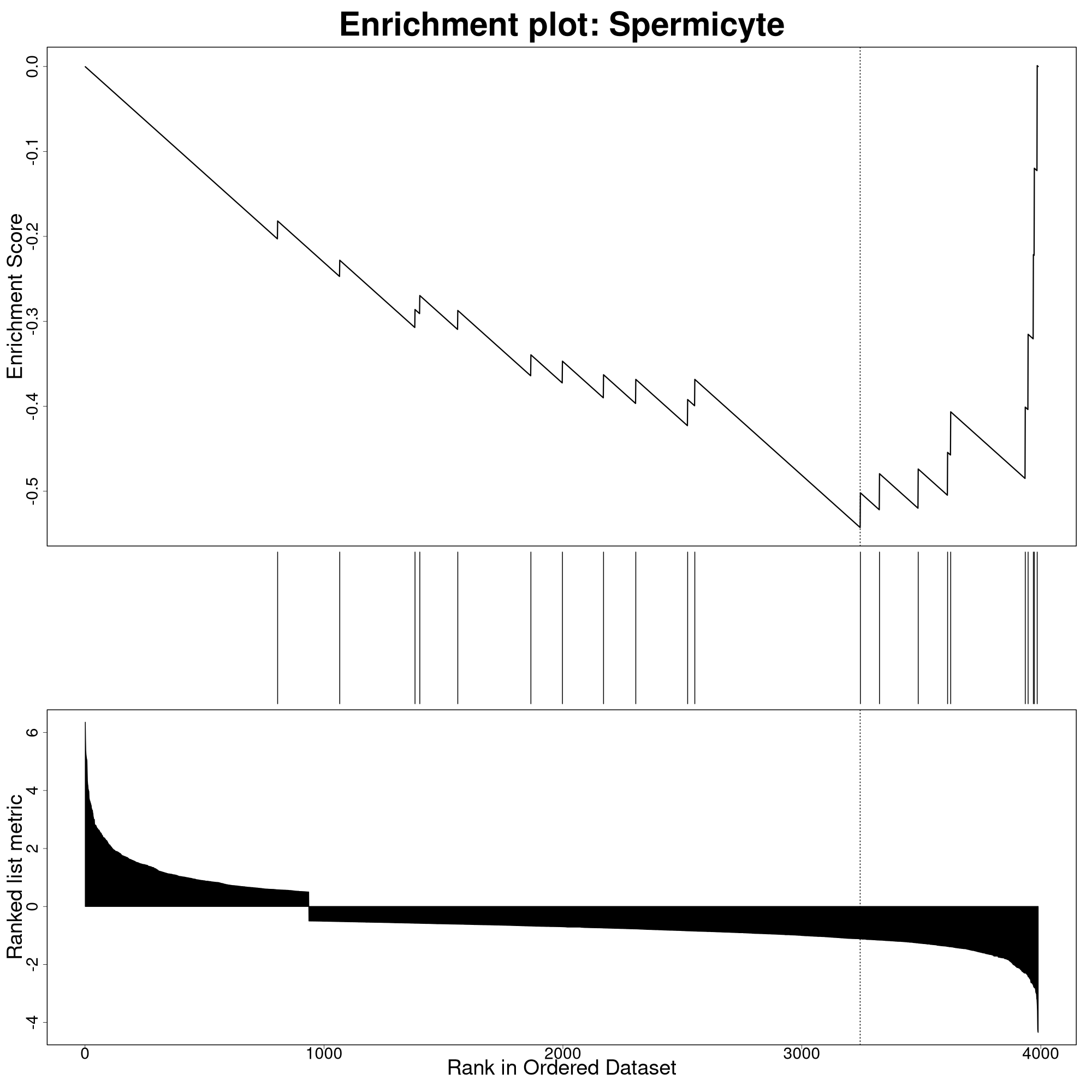

Supplement: Supplementary file 17 — Figure EV5 Source Data [file 44319_2025_631_MOESM17_ESM.zip › Figure EV5/EV5A/GSEA T11b high LUSC vs T11b low LUSC/Project_wg_result1729116948/Project_wg_result1729116948_GSEA/Spermicyte.png]

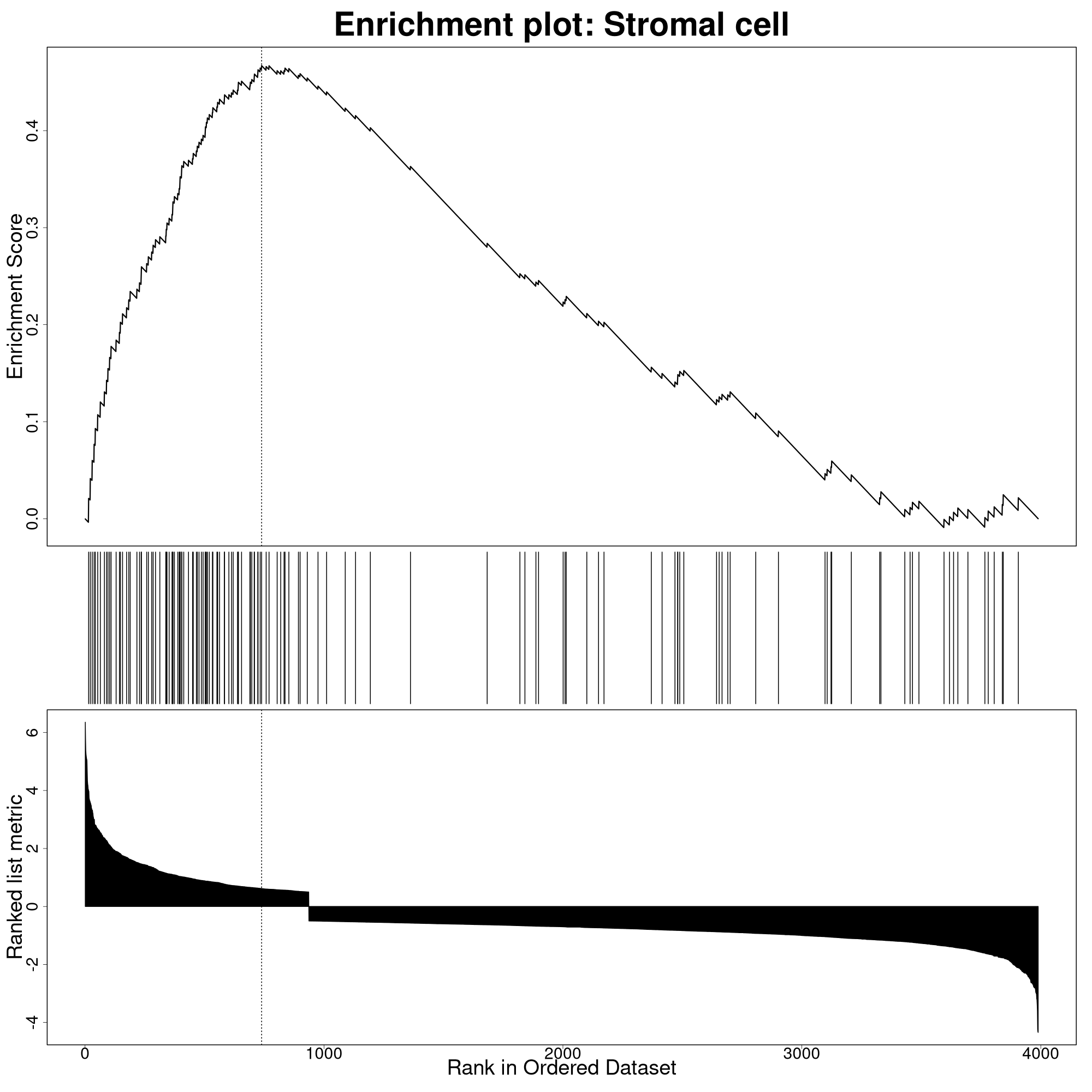

Supplement: Supplementary file 17 — Figure EV5 Source Data [file 44319_2025_631_MOESM17_ESM.zip › Figure EV5/EV5A/GSEA T11b high LUSC vs T11b low LUSC/Project_wg_result1729116948/Project_wg_result1729116948_GSEA/Stromal cell.png]

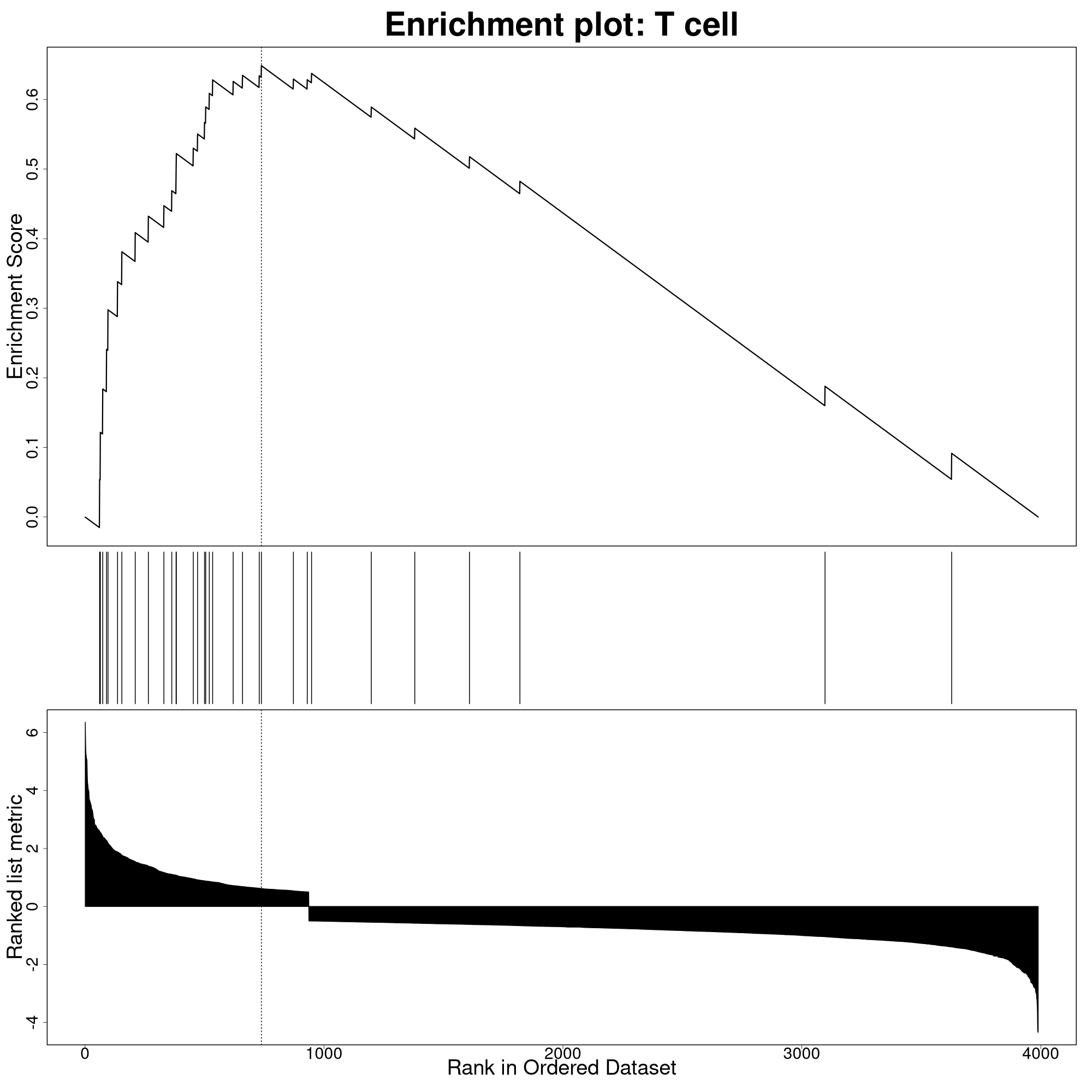

Supplement: Supplementary file 17 — Figure EV5 Source Data [file 44319_2025_631_MOESM17_ESM.zip › Figure EV5/EV5A/GSEA T11b high LUSC vs T11b low LUSC/Project_wg_result1729116948/Project_wg_result1729116948_GSEA/T cell.png]

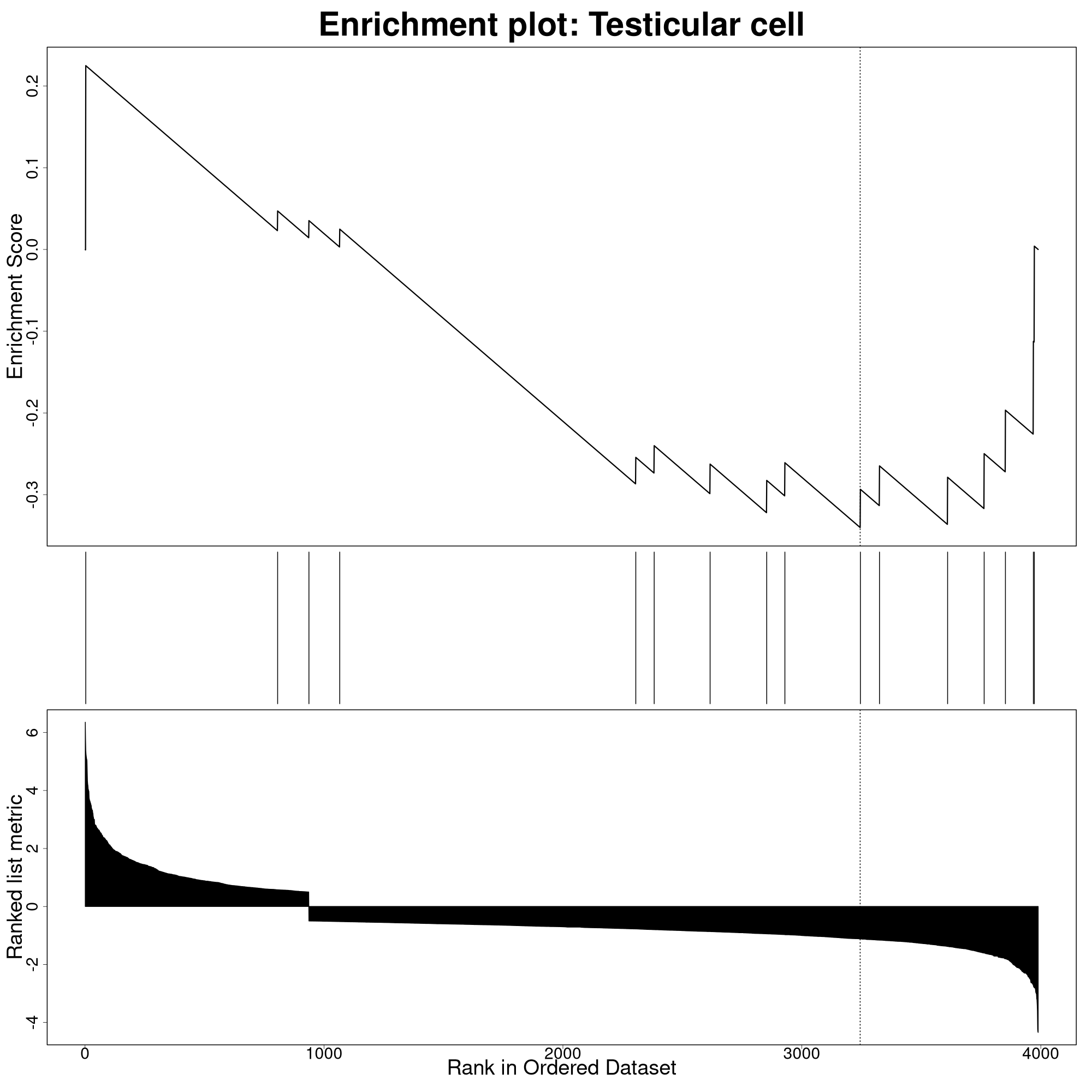

Supplement: Supplementary file 17 — Figure EV5 Source Data [file 44319_2025_631_MOESM17_ESM.zip › Figure EV5/EV5A/GSEA T11b high LUSC vs T11b low LUSC/Project_wg_result1729116948/Project_wg_result1729116948_GSEA/Testicular cell.png]

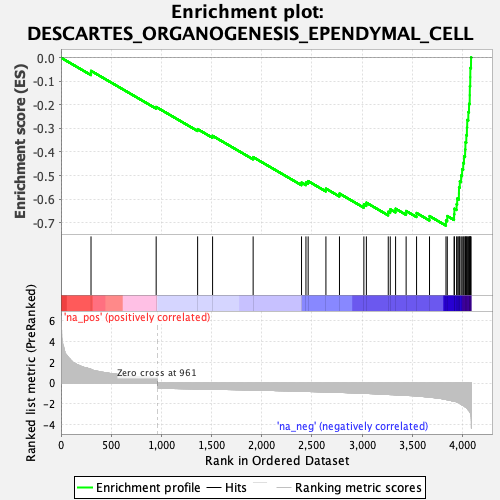

Supplement: Supplementary file 18 — Figure EV6 Source Data [file 44319_2025_631_MOESM18_ESM.zip › Figure EV6/EV6C-D/GSEA_Broad Institute_M8_T11b high vs low LUSC/enplot_DESCARTES_ORGANOGENESIS_EPENDYMAL_CELL_833.png]

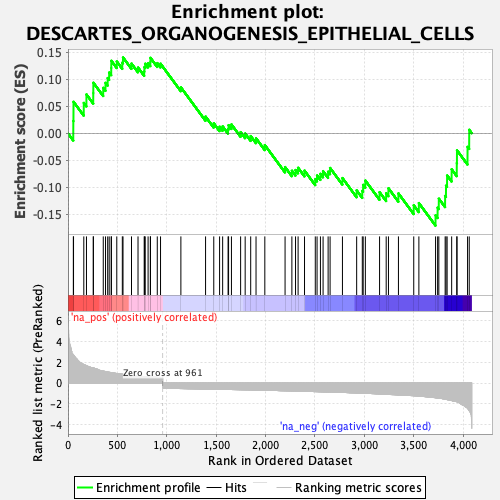

Supplement: Supplementary file 18 — Figure EV6 Source Data [file 44319_2025_631_MOESM18_ESM.zip › Figure EV6/EV6C-D/GSEA_Broad Institute_M8_T11b high vs low LUSC/enplot_DESCARTES_ORGANOGENESIS_EPITHELIAL_CELLS_851.png]

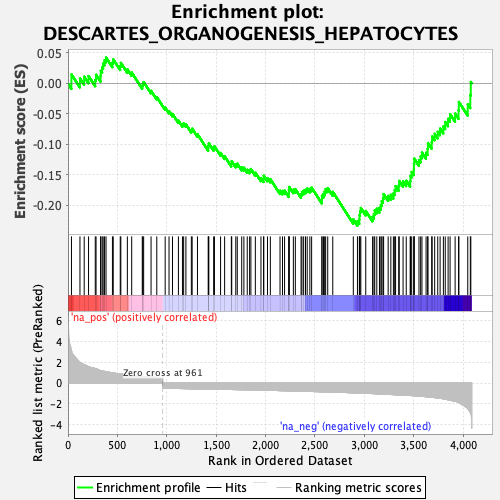

Supplement: Supplementary file 18 — Figure EV6 Source Data [file 44319_2025_631_MOESM18_ESM.zip › Figure EV6/EV6C-D/GSEA_Broad Institute_M8_T11b high vs low LUSC/enplot_DESCARTES_ORGANOGENESIS_HEPATOCYTES_839.png]

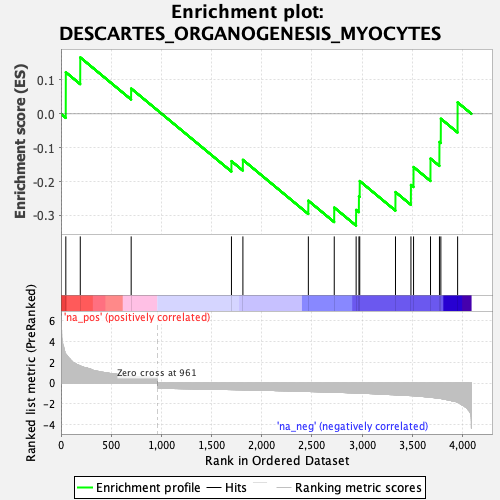

Supplement: Supplementary file 18 — Figure EV6 Source Data [file 44319_2025_631_MOESM18_ESM.zip › Figure EV6/EV6C-D/GSEA_Broad Institute_M8_T11b high vs low LUSC/enplot_DESCARTES_ORGANOGENESIS_MYOCYTES_845.png]

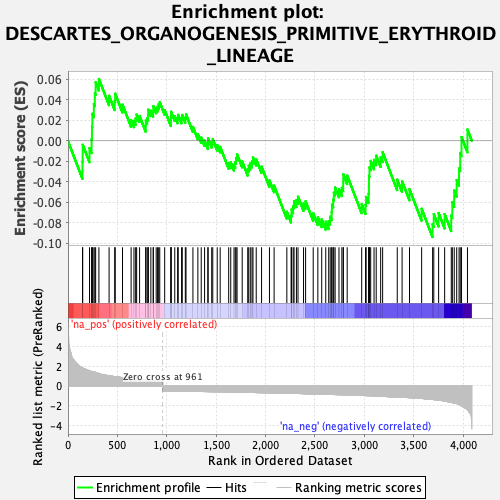

Supplement: Supplementary file 18 — Figure EV6 Source Data [file 44319_2025_631_MOESM18_ESM.zip › Figure EV6/EV6C-D/GSEA_Broad Institute_M8_T11b high vs low LUSC/enplot_DESCARTES_ORGANOGENESIS_PRIMITIVE_ERYTHROID_LINEAGE_867.png]

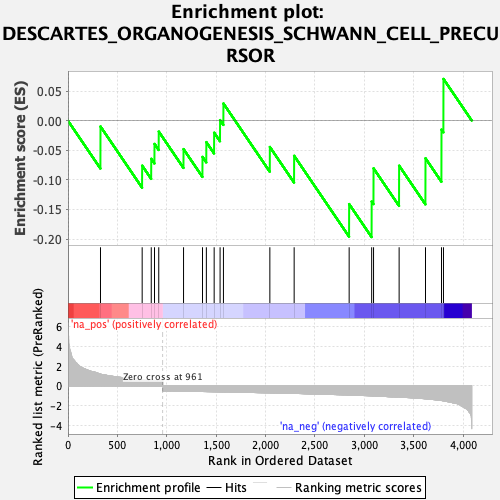

Supplement: Supplementary file 18 — Figure EV6 Source Data [file 44319_2025_631_MOESM18_ESM.zip › Figure EV6/EV6C-D/GSEA_Broad Institute_M8_T11b high vs low LUSC/enplot_DESCARTES_ORGANOGENESIS_SCHWANN_CELL_PRECURSOR_863.png]

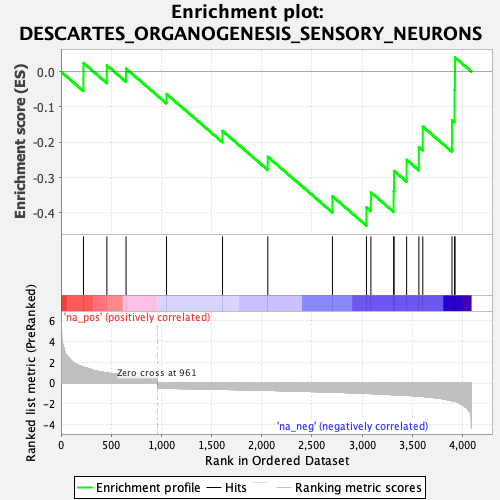

Supplement: Supplementary file 18 — Figure EV6 Source Data [file 44319_2025_631_MOESM18_ESM.zip › Figure EV6/EV6C-D/GSEA_Broad Institute_M8_T11b high vs low LUSC/enplot_DESCARTES_ORGANOGENESIS_SENSORY_NEURONS_835.png]

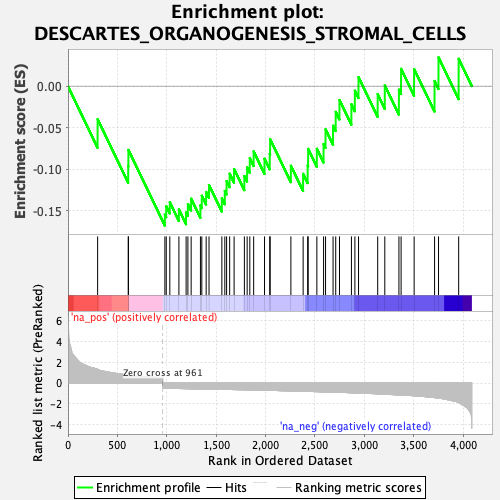

Supplement: Supplementary file 18 — Figure EV6 Source Data [file 44319_2025_631_MOESM18_ESM.zip › Figure EV6/EV6C-D/GSEA_Broad Institute_M8_T11b high vs low LUSC/enplot_DESCARTES_ORGANOGENESIS_STROMAL_CELLS_855.png]

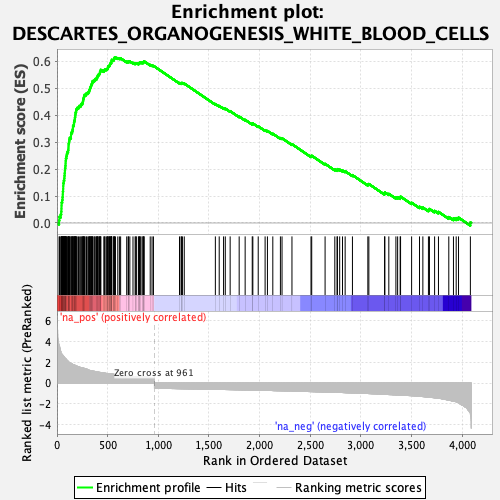

Supplement: Supplementary file 18 — Figure EV6 Source Data [file 44319_2025_631_MOESM18_ESM.zip › Figure EV6/EV6C-D/GSEA_Broad Institute_M8_T11b high vs low LUSC/enplot_DESCARTES_ORGANOGENESIS_WHITE_BLOOD_CELLS_797.png]

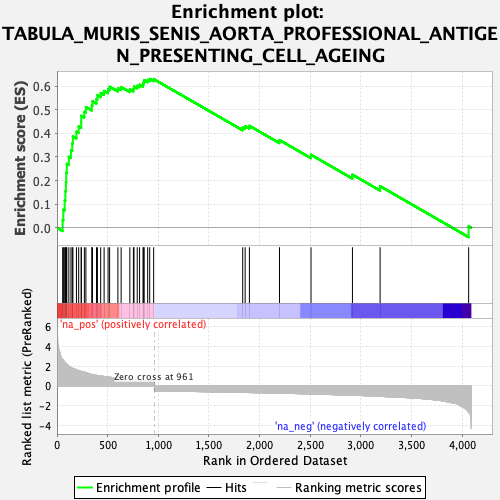

Supplement: Supplementary file 18 — Figure EV6 Source Data [file 44319_2025_631_MOESM18_ESM.zip › Figure EV6/EV6C-D/GSEA_Broad Institute_M8_T11b high vs low LUSC/enplot_TABULA_MURIS_SENIS_AORTA_PROFESSIONAL_ANTIGEN_PRESENTING_CELL_AGEING_817.png]

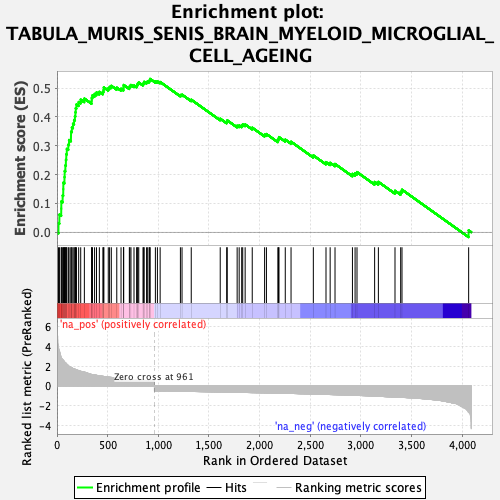

Supplement: Supplementary file 18 — Figure EV6 Source Data [file 44319_2025_631_MOESM18_ESM.zip › Figure EV6/EV6C-D/GSEA_Broad Institute_M8_T11b high vs low LUSC/enplot_TABULA_MURIS_SENIS_BRAIN_MYELOID_MICROGLIAL_CELL_AGEING_811.png]

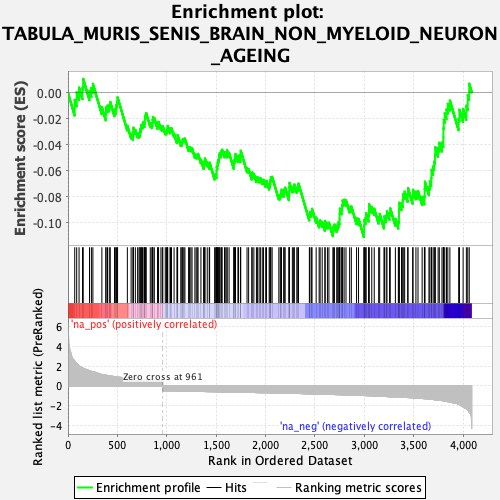

Supplement: Supplementary file 18 — Figure EV6 Source Data [file 44319_2025_631_MOESM18_ESM.zip › Figure EV6/EV6C-D/GSEA_Broad Institute_M8_T11b high vs low LUSC/enplot_TABULA_MURIS_SENIS_BRAIN_NON_MYELOID_NEURON_AGEING_857.png]

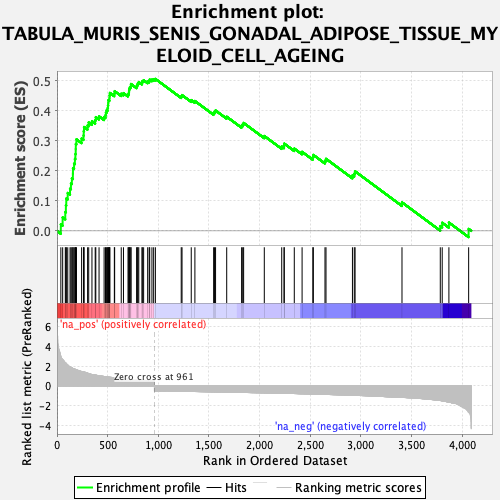

Supplement: Supplementary file 18 — Figure EV6 Source Data [file 44319_2025_631_MOESM18_ESM.zip › Figure EV6/EV6C-D/GSEA_Broad Institute_M8_T11b high vs low LUSC/enplot_TABULA_MURIS_SENIS_GONADAL_ADIPOSE_TISSUE_MYELOID_CELL_AGEING_821.png]

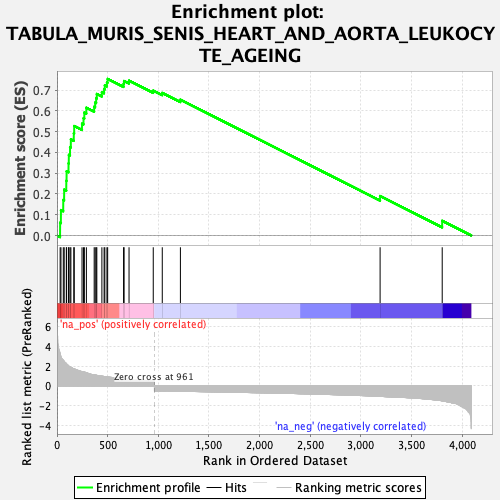

Supplement: Supplementary file 18 — Figure EV6 Source Data [file 44319_2025_631_MOESM18_ESM.zip › Figure EV6/EV6C-D/GSEA_Broad Institute_M8_T11b high vs low LUSC/enplot_TABULA_MURIS_SENIS_HEART_AND_AORTA_LEUKOCYTE_AGEING_805.png]

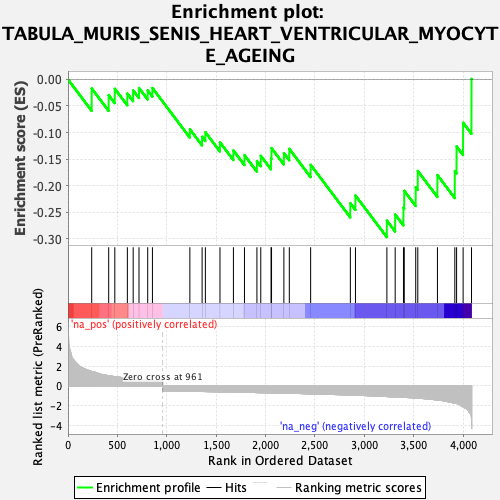

Supplement: Supplementary file 18 — Figure EV6 Source Data [file 44319_2025_631_MOESM18_ESM.zip › Figure EV6/EV6C-D/GSEA_Broad Institute_M8_T11b high vs low LUSC/enplot_TABULA_MURIS_SENIS_HEART_VENTRICULAR_MYOCYTE_AGEING_843.png]

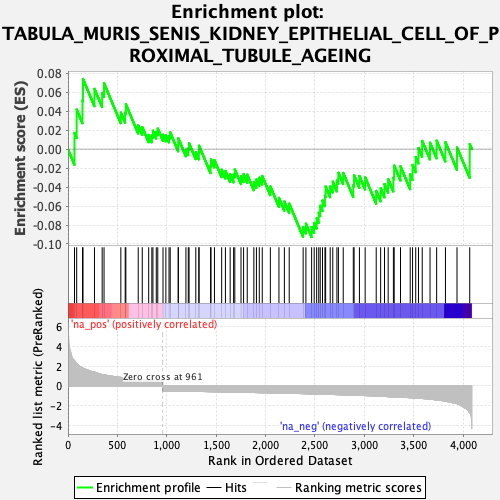

Supplement: Supplementary file 18 — Figure EV6 Source Data [file 44319_2025_631_MOESM18_ESM.zip › Figure EV6/EV6C-D/GSEA_Broad Institute_M8_T11b high vs low LUSC/enplot_TABULA_MURIS_SENIS_KIDNEY_EPITHELIAL_CELL_OF_PROXIMAL_TUBULE_AGEING_869.png]

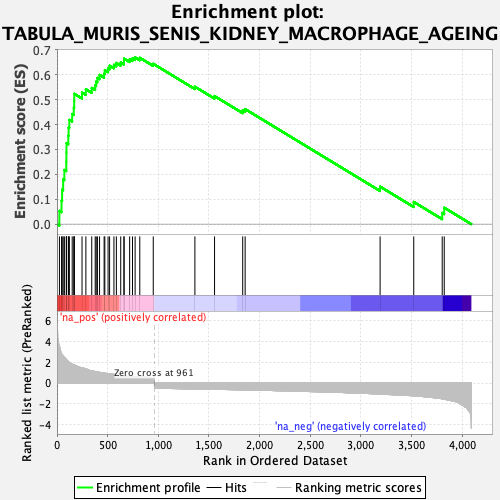

Supplement: Supplementary file 18 — Figure EV6 Source Data [file 44319_2025_631_MOESM18_ESM.zip › Figure EV6/EV6C-D/GSEA_Broad Institute_M8_T11b high vs low LUSC/enplot_TABULA_MURIS_SENIS_KIDNEY_MACROPHAGE_AGEING_809.png]

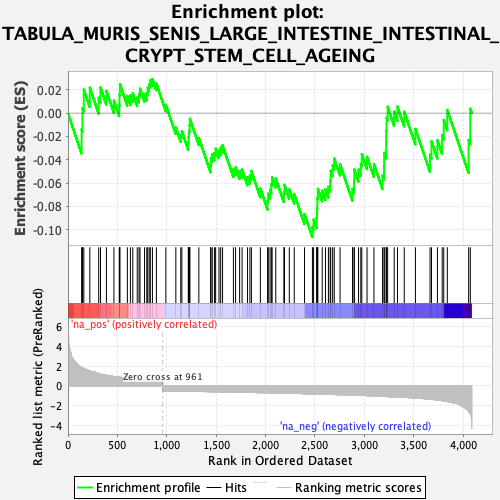

Supplement: Supplementary file 18 — Figure EV6 Source Data [file 44319_2025_631_MOESM18_ESM.zip › Figure EV6/EV6C-D/GSEA_Broad Institute_M8_T11b high vs low LUSC/enplot_TABULA_MURIS_SENIS_LARGE_INTESTINE_INTESTINAL_CRYPT_STEM_CELL_AGEING_865.png]

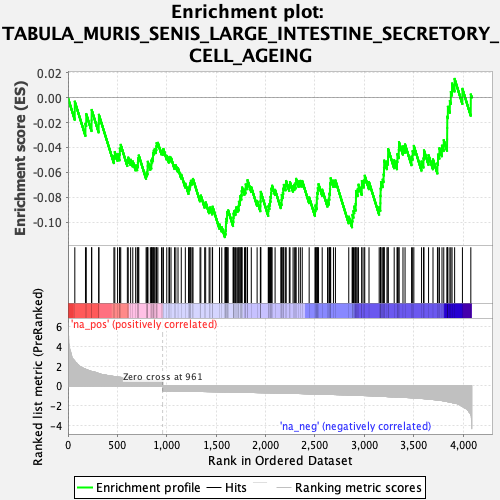

Supplement: Supplementary file 18 — Figure EV6 Source Data [file 44319_2025_631_MOESM18_ESM.zip › Figure EV6/EV6C-D/GSEA_Broad Institute_M8_T11b high vs low LUSC/enplot_TABULA_MURIS_SENIS_LARGE_INTESTINE_SECRETORY_CELL_AGEING_859.png]

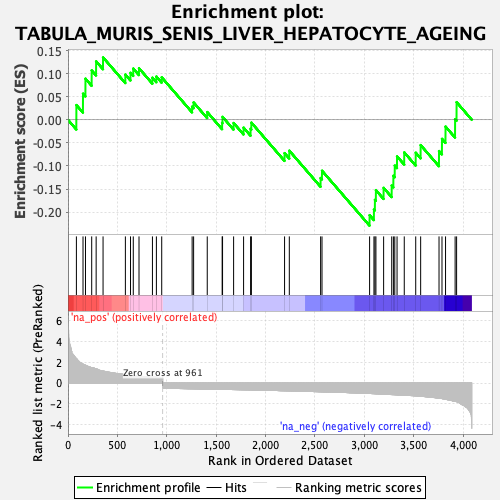

Supplement: Supplementary file 18 — Figure EV6 Source Data [file 44319_2025_631_MOESM18_ESM.zip › Figure EV6/EV6C-D/GSEA_Broad Institute_M8_T11b high vs low LUSC/enplot_TABULA_MURIS_SENIS_LIVER_HEPATOCYTE_AGEING_847.png]

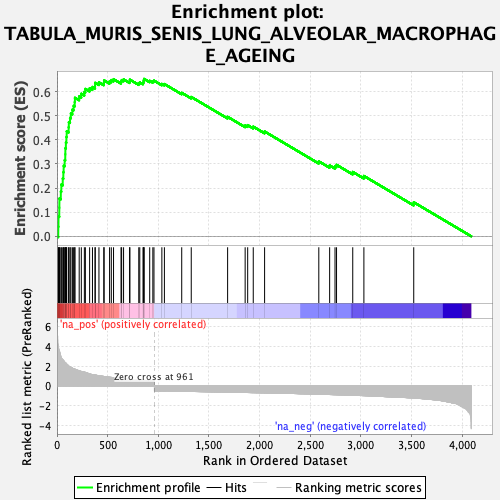

Supplement: Supplementary file 18 — Figure EV6 Source Data [file 44319_2025_631_MOESM18_ESM.zip › Figure EV6/EV6C-D/GSEA_Broad Institute_M8_T11b high vs low LUSC/enplot_TABULA_MURIS_SENIS_LUNG_ALVEOLAR_MACROPHAGE_AGEING_803.png]

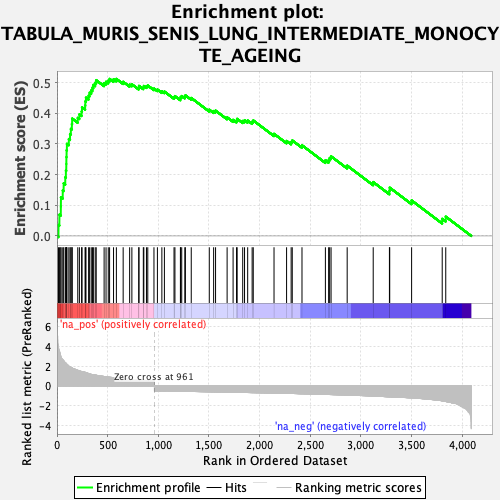

Supplement: Supplementary file 18 — Figure EV6 Source Data [file 44319_2025_631_MOESM18_ESM.zip › Figure EV6/EV6C-D/GSEA_Broad Institute_M8_T11b high vs low LUSC/enplot_TABULA_MURIS_SENIS_LUNG_INTERMEDIATE_MONOCYTE_AGEING_825.png]

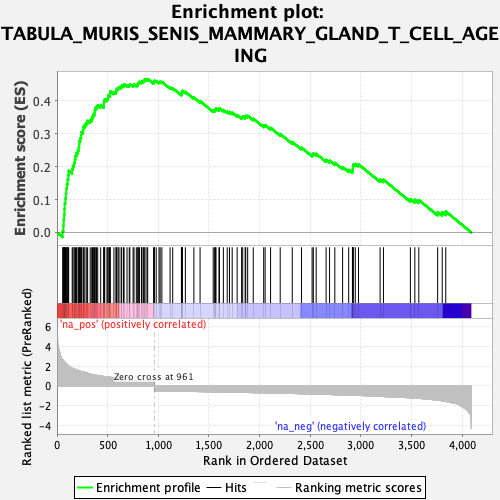

Supplement: Supplementary file 18 — Figure EV6 Source Data [file 44319_2025_631_MOESM18_ESM.zip › Figure EV6/EV6C-D/GSEA_Broad Institute_M8_T11b high vs low LUSC/enplot_TABULA_MURIS_SENIS_MAMMARY_GLAND_T_CELL_AGEING_823.png]

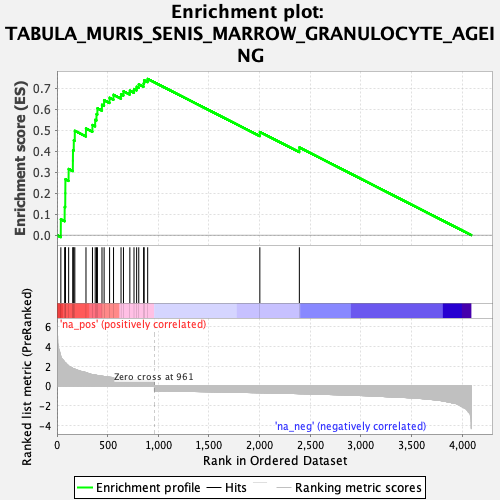

Supplement: Supplementary file 18 — Figure EV6 Source Data [file 44319_2025_631_MOESM18_ESM.zip › Figure EV6/EV6C-D/GSEA_Broad Institute_M8_T11b high vs low LUSC/enplot_TABULA_MURIS_SENIS_MARROW_GRANULOCYTE_AGEING_819.png]

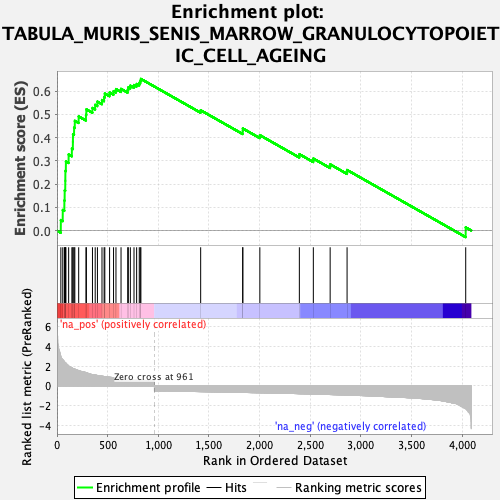

Supplement: Supplementary file 18 — Figure EV6 Source Data [file 44319_2025_631_MOESM18_ESM.zip › Figure EV6/EV6C-D/GSEA_Broad Institute_M8_T11b high vs low LUSC/enplot_TABULA_MURIS_SENIS_MARROW_GRANULOCYTOPOIETIC_CELL_AGEING_815.png]

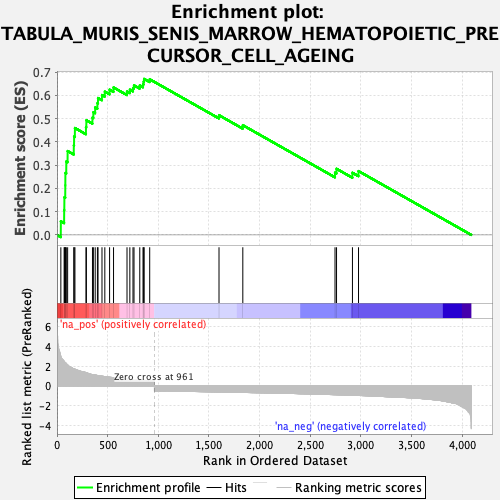

Supplement: Supplementary file 18 — Figure EV6 Source Data [file 44319_2025_631_MOESM18_ESM.zip › Figure EV6/EV6C-D/GSEA_Broad Institute_M8_T11b high vs low LUSC/enplot_TABULA_MURIS_SENIS_MARROW_HEMATOPOIETIC_PRECURSOR_CELL_AGEING_813.png]

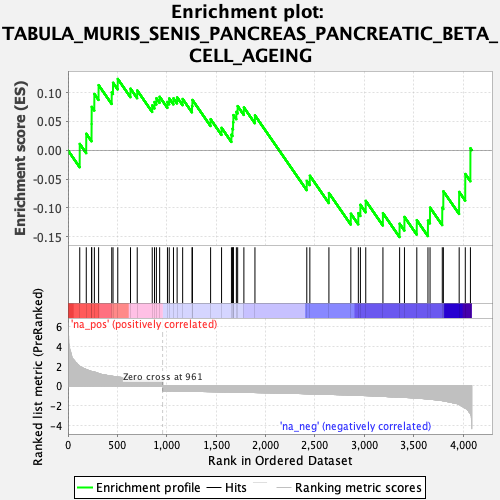

Supplement: Supplementary file 18 — Figure EV6 Source Data [file 44319_2025_631_MOESM18_ESM.zip › Figure EV6/EV6C-D/GSEA_Broad Institute_M8_T11b high vs low LUSC/enplot_TABULA_MURIS_SENIS_PANCREAS_PANCREATIC_BETA_CELL_AGEING_861.png]

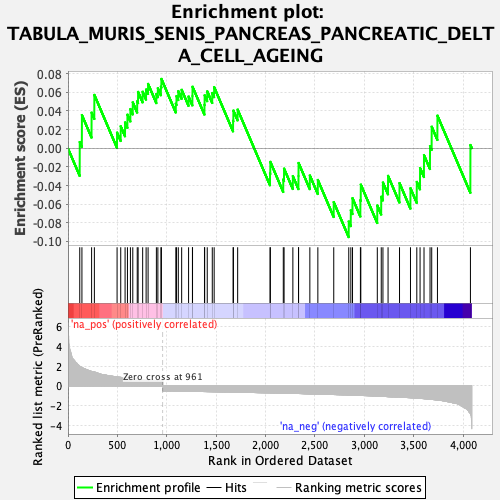

Supplement: Supplementary file 18 — Figure EV6 Source Data [file 44319_2025_631_MOESM18_ESM.zip › Figure EV6/EV6C-D/GSEA_Broad Institute_M8_T11b high vs low LUSC/enplot_TABULA_MURIS_SENIS_PANCREAS_PANCREATIC_DELTA_CELL_AGEING_871.png]

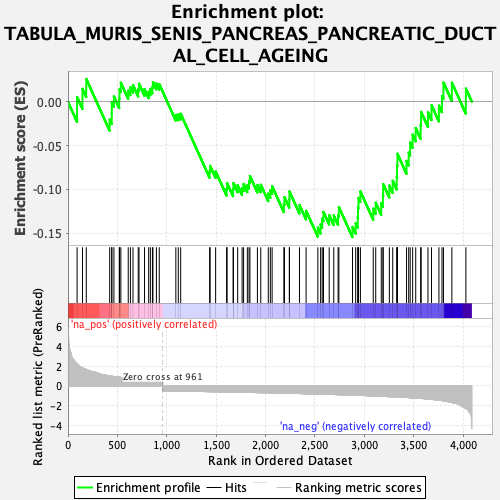

Supplement: Supplementary file 18 — Figure EV6 Source Data [file 44319_2025_631_MOESM18_ESM.zip › Figure EV6/EV6C-D/GSEA_Broad Institute_M8_T11b high vs low LUSC/enplot_TABULA_MURIS_SENIS_PANCREAS_PANCREATIC_DUCTAL_CELL_AGEING_853.png]

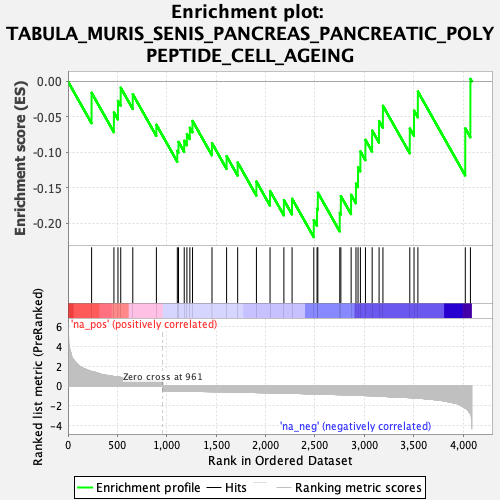

Supplement: Supplementary file 18 — Figure EV6 Source Data [file 44319_2025_631_MOESM18_ESM.zip › Figure EV6/EV6C-D/GSEA_Broad Institute_M8_T11b high vs low LUSC/enplot_TABULA_MURIS_SENIS_PANCREAS_PANCREATIC_POLYPEPTIDE_CELL_AGEING_849.png]

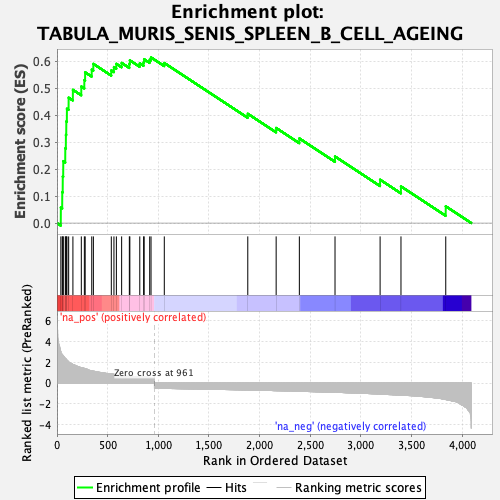

Supplement: Supplementary file 18 — Figure EV6 Source Data [file 44319_2025_631_MOESM18_ESM.zip › Figure EV6/EV6C-D/GSEA_Broad Institute_M8_T11b high vs low LUSC/enplot_TABULA_MURIS_SENIS_SPLEEN_B_CELL_AGEING_827.png]

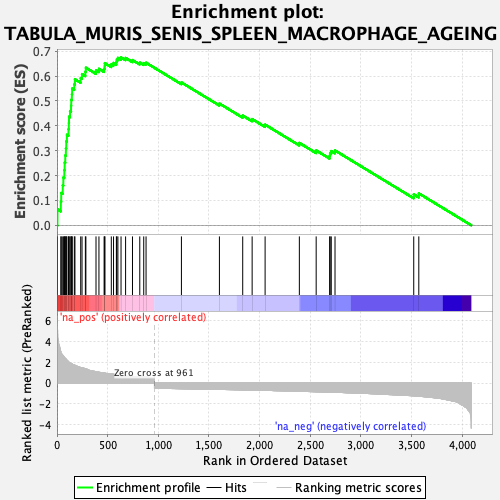

Supplement: Supplementary file 18 — Figure EV6 Source Data [file 44319_2025_631_MOESM18_ESM.zip › Figure EV6/EV6C-D/GSEA_Broad Institute_M8_T11b high vs low LUSC/enplot_TABULA_MURIS_SENIS_SPLEEN_MACROPHAGE_AGEING_801.png]

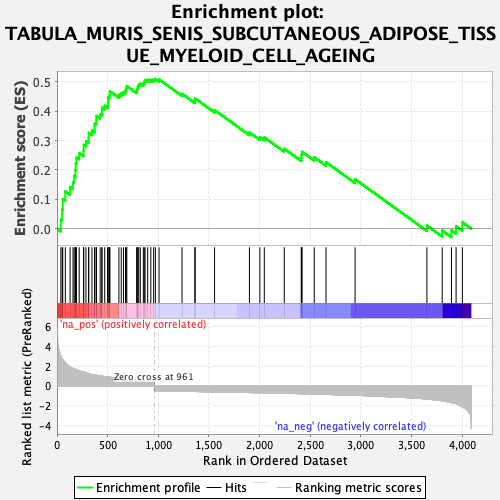

Supplement: Supplementary file 18 — Figure EV6 Source Data [file 44319_2025_631_MOESM18_ESM.zip › Figure EV6/EV6C-D/GSEA_Broad Institute_M8_T11b high vs low LUSC/enplot_TABULA_MURIS_SENIS_SUBCUTANEOUS_ADIPOSE_TISSUE_MYELOID_CELL_AGEING_829.png]

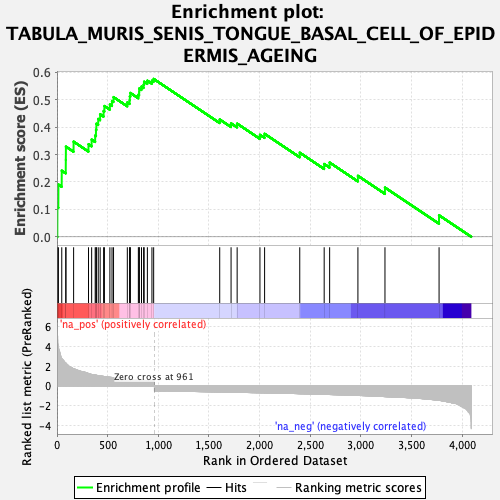

Supplement: Supplementary file 18 — Figure EV6 Source Data [file 44319_2025_631_MOESM18_ESM.zip › Figure EV6/EV6C-D/GSEA_Broad Institute_M8_T11b high vs low LUSC/enplot_TABULA_MURIS_SENIS_TONGUE_BASAL_CELL_OF_EPIDERMIS_AGEING_831.png]

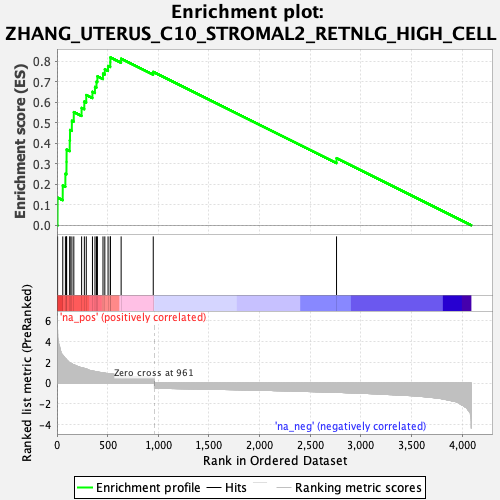

Supplement: Supplementary file 18 — Figure EV6 Source Data [file 44319_2025_631_MOESM18_ESM.zip › Figure EV6/EV6C-D/GSEA_Broad Institute_M8_T11b high vs low LUSC/enplot_ZHANG_UTERUS_C10_STROMAL2_RETNLG_HIGH_CELL_807.png]

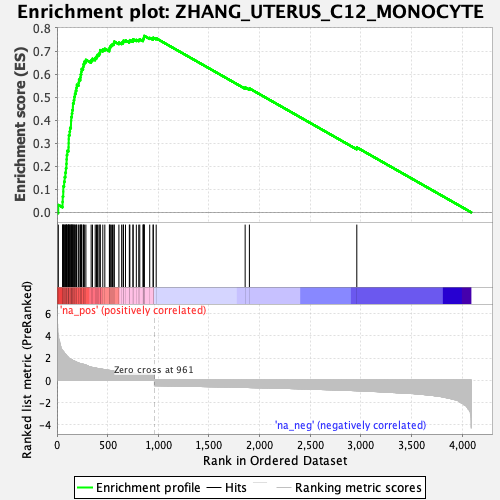

Supplement: Supplementary file 18 — Figure EV6 Source Data [file 44319_2025_631_MOESM18_ESM.zip › Figure EV6/EV6C-D/GSEA_Broad Institute_M8_T11b high vs low LUSC/enplot_ZHANG_UTERUS_C12_MONOCYTE_793.png]

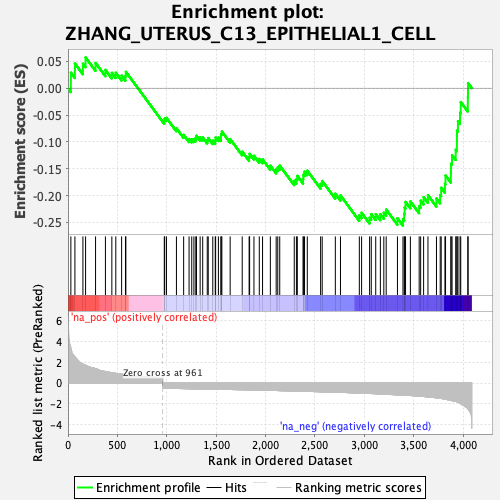

Supplement: Supplementary file 18 — Figure EV6 Source Data [file 44319_2025_631_MOESM18_ESM.zip › Figure EV6/EV6C-D/GSEA_Broad Institute_M8_T11b high vs low LUSC/enplot_ZHANG_UTERUS_C13_EPITHELIAL1_CELL_837.png]

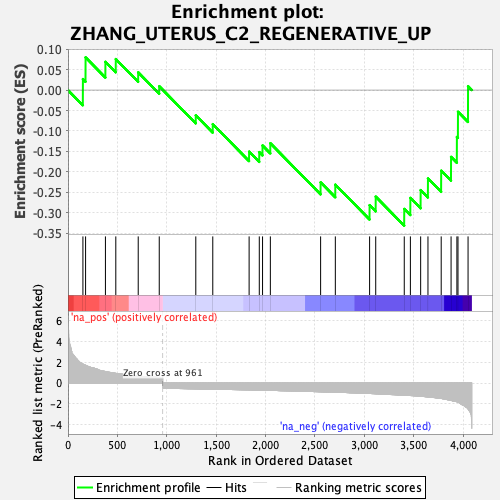

Supplement: Supplementary file 18 — Figure EV6 Source Data [file 44319_2025_631_MOESM18_ESM.zip › Figure EV6/EV6C-D/GSEA_Broad Institute_M8_T11b high vs low LUSC/enplot_ZHANG_UTERUS_C2_REGENERATIVE_UP_841.png]

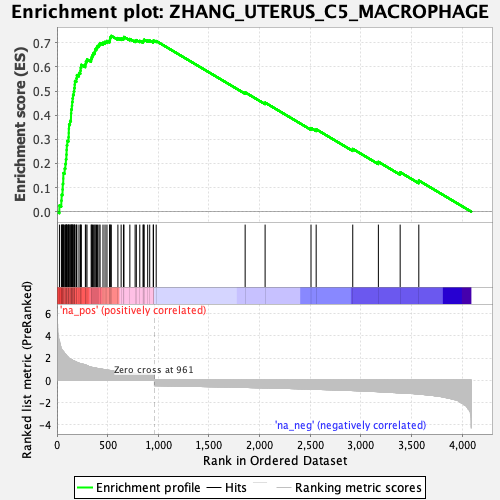

Supplement: Supplementary file 18 — Figure EV6 Source Data [file 44319_2025_631_MOESM18_ESM.zip › Figure EV6/EV6C-D/GSEA_Broad Institute_M8_T11b high vs low LUSC/enplot_ZHANG_UTERUS_C5_MACROPHAGE_795.png]

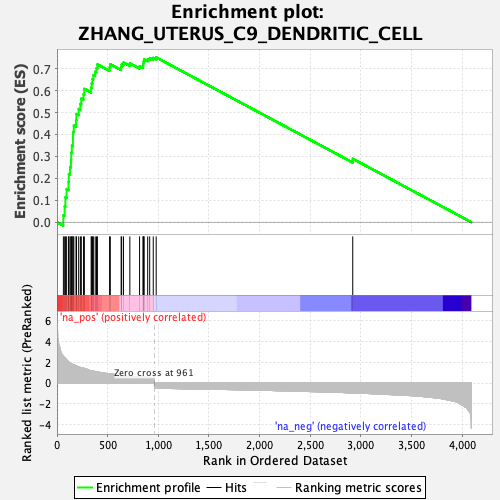

Supplement: Supplementary file 18 — Figure EV6 Source Data [file 44319_2025_631_MOESM18_ESM.zip › Figure EV6/EV6C-D/GSEA_Broad Institute_M8_T11b high vs low LUSC/enplot_ZHANG_UTERUS_C9_DENDRITIC_CELL_799.png]

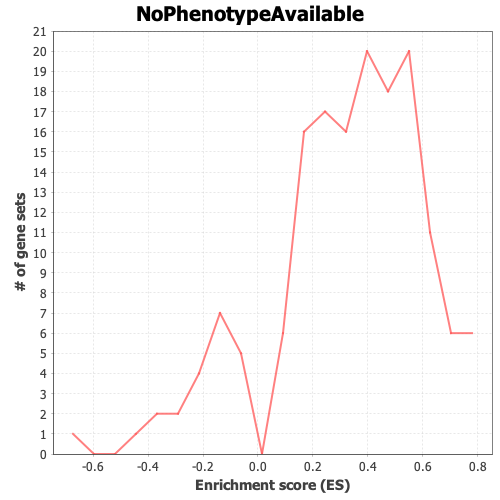

Supplement: Supplementary file 18 — Figure EV6 Source Data [file 44319_2025_631_MOESM18_ESM.zip › Figure EV6/EV6C-D/GSEA_Broad Institute_M8_T11b high vs low LUSC/global_es_histogram.png]

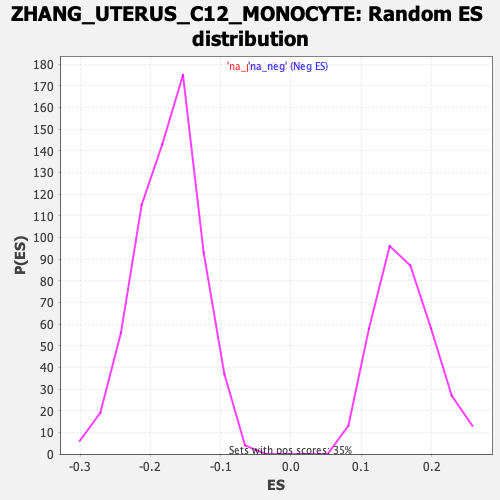

Supplement: Supplementary file 18 — Figure EV6 Source Data [file 44319_2025_631_MOESM18_ESM.zip › Figure EV6/EV6C-D/GSEA_Broad Institute_M8_T11b high vs low LUSC/gset_rnd_es_dist_794.png]

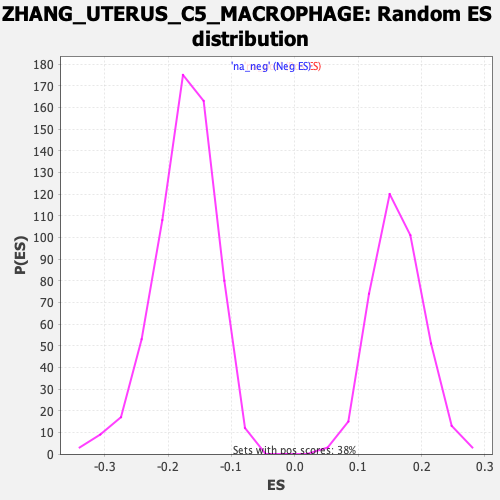

Supplement: Supplementary file 18 — Figure EV6 Source Data [file 44319_2025_631_MOESM18_ESM.zip › Figure EV6/EV6C-D/GSEA_Broad Institute_M8_T11b high vs low LUSC/gset_rnd_es_dist_796.png]

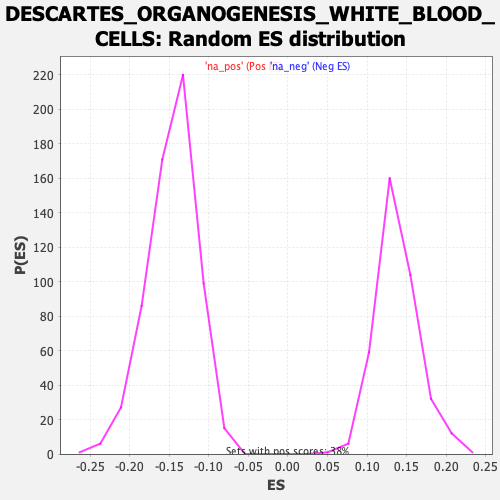

Supplement: Supplementary file 18 — Figure EV6 Source Data [file 44319_2025_631_MOESM18_ESM.zip › Figure EV6/EV6C-D/GSEA_Broad Institute_M8_T11b high vs low LUSC/gset_rnd_es_dist_798.png]

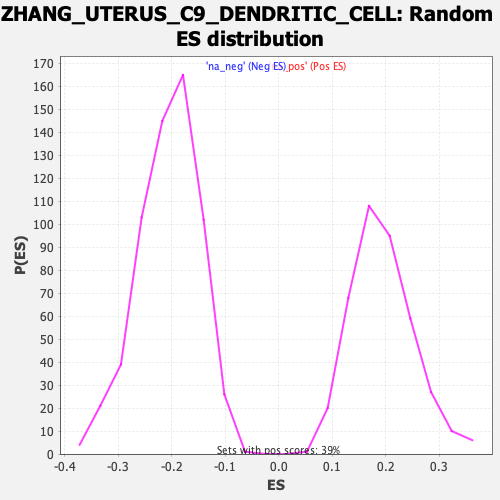

Supplement: Supplementary file 18 — Figure EV6 Source Data [file 44319_2025_631_MOESM18_ESM.zip › Figure EV6/EV6C-D/GSEA_Broad Institute_M8_T11b high vs low LUSC/gset_rnd_es_dist_800.png]

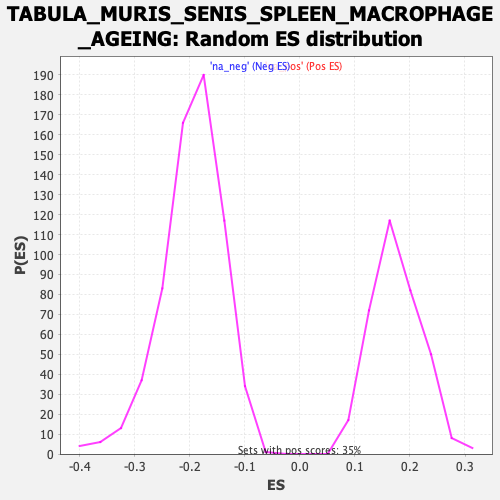

Supplement: Supplementary file 18 — Figure EV6 Source Data [file 44319_2025_631_MOESM18_ESM.zip › Figure EV6/EV6C-D/GSEA_Broad Institute_M8_T11b high vs low LUSC/gset_rnd_es_dist_802.png]

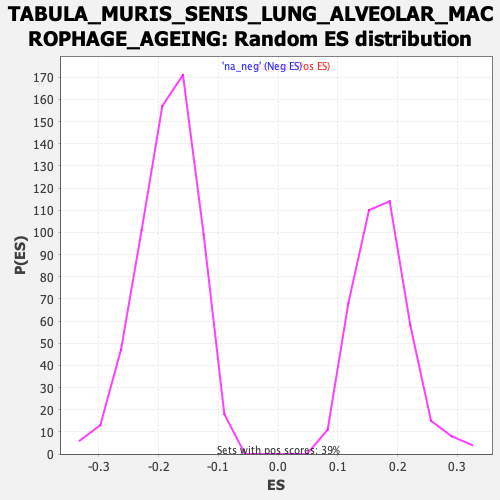

Supplement: Supplementary file 18 — Figure EV6 Source Data [file 44319_2025_631_MOESM18_ESM.zip › Figure EV6/EV6C-D/GSEA_Broad Institute_M8_T11b high vs low LUSC/gset_rnd_es_dist_804.png]

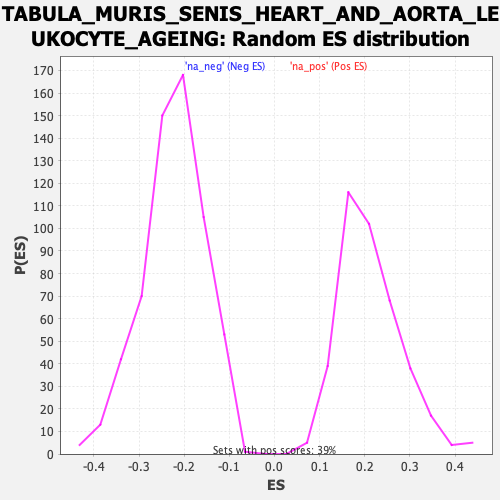

Supplement: Supplementary file 18 — Figure EV6 Source Data [file 44319_2025_631_MOESM18_ESM.zip › Figure EV6/EV6C-D/GSEA_Broad Institute_M8_T11b high vs low LUSC/gset_rnd_es_dist_806.png]

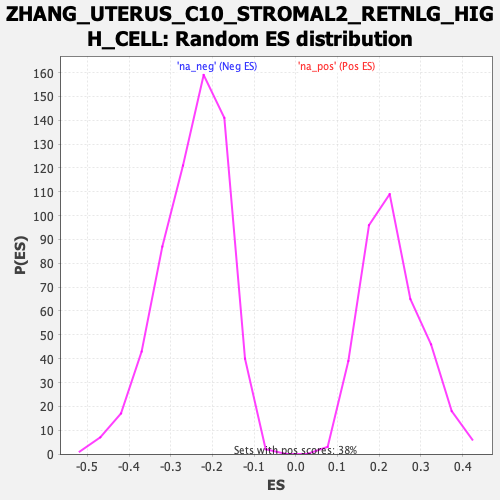

Supplement: Supplementary file 18 — Figure EV6 Source Data [file 44319_2025_631_MOESM18_ESM.zip › Figure EV6/EV6C-D/GSEA_Broad Institute_M8_T11b high vs low LUSC/gset_rnd_es_dist_808.png]
