# Supplementary material for: TMPRSS11B promotes an acidified microenvironment and immune suppression in squamous lung cancer
Source: EMBO Rep. 2025 Nov 10;26(24):6346–79. doi: 10.1038/s44319-025-00631-1 (PMC12714794; doi:10.1038/s44319-025-00631-1)
Supplement: Supplementary file 15 — Figure EV3 Source Data [file 44319_2025_631_MOESM15_ESM.zip › Figure EV3/EV3A/Read Me.rtf]

The RNA sequencing data has been deposited to GEO and accession number is included in the manuscript. The processed differential gene expression analysis files are included here. 
