# Supplementary material for: TMPRSS11B promotes an acidified microenvironment and immune suppression in squamous lung cancer
Source: EMBO Rep. 2025 Nov 10;26(24):6346–79. doi: 10.1038/s44319-025-00631-1 (PMC12714794; doi:10.1038/s44319-025-00631-1)
Supplement: Supplementary file 15 — Figure EV3 Source Data [file 44319_2025_631_MOESM15_ESM.zip › Figure EV3/EV3B-C/Read Me.rtf]

The spatial transcriptomics data has been deposited to GEO and accession number is included in the manuscript. The differential gene expression analysis files are provided in the source data for Fig 3D-E and Fig 5C-D.
