# Supplementary material for: TMPRSS11B promotes an acidified microenvironment and immune suppression in squamous lung cancer
Source: EMBO Rep. 2025 Nov 10;26(24):6346–79. doi: 10.1038/s44319-025-00631-1 (PMC12714794; doi:10.1038/s44319-025-00631-1)
Supplement: Supplementary file 16 — Figure EV4 Source Data [file 44319_2025_631_MOESM16_ESM.zip › Figure EV4/EV4A-B/Read Me.rtf]

The spatial transcriptomics data used for this analysis has been deposited to GEO and accession number is included in the manuscript. The marker genes used for the analysis have been provided in Table EV2.
