# Supplementary material for: TMPRSS11B promotes an acidified microenvironment and immune suppression in squamous lung cancer
Source: EMBO Rep. 2025 Nov 10;26(24):6346–79. doi: 10.1038/s44319-025-00631-1 (PMC12714794; doi:10.1038/s44319-025-00631-1)
Supplement: Supplementary file 17 — Figure EV5 Source Data [file 44319_2025_631_MOESM17_ESM.zip › Figure EV5/EV5A/Read Me.rtf]

GSEA has been performed using WEB-based Gene Set Analysis Toolkit. https://www.webgestalt.org/The “T11b high vs low squamous_Analysis.xlsx” file representing the differential gene expression analysis (DEG) results and the .rnk file used for the pre-ranked gene set enrichment analysis (GSEA), generated using the average log2FC as the ranking metric, have been provided in source data for Figure 3D-E.The “T11b high LUSC vs LUAD_Analysis.xlsx” file representing the differential gene expression analysis (DEG) results and the .rnk file used for the pre-ranked gene set enrichment analysis (GSEA), generated using the average log2FC as the ranking metric, have been provided in source data for Figure 5C-D.
