# Supplementary material for: TMPRSS11B promotes an acidified microenvironment and immune suppression in squamous lung cancer
Source: EMBO Rep. 2025 Nov 10;26(24):6346–79. doi: 10.1038/s44319-025-00631-1 (PMC12714794; doi:10.1038/s44319-025-00631-1)
Supplement: Supplementary file 17 — Figure EV5 Source Data [file 44319_2025_631_MOESM17_ESM.zip › Figure EV5/EV5B/Read Me.rtf]

The spatial transcriptomics data used for this analysis has been deposited to GEO and accession number is included in the manuscript. 
