# Supplementary material for: TMPRSS11B promotes an acidified microenvironment and immune suppression in squamous lung cancer
Source: EMBO Rep. 2025 Nov 10;26(24):6346–79. doi: 10.1038/s44319-025-00631-1 (PMC12714794; doi:10.1038/s44319-025-00631-1)
Supplement: Supplementary file 18 — Figure EV6 Source Data [file 44319_2025_631_MOESM18_ESM.zip › Figure EV6/EV6C-D/GSEA_Broad Institute_M8_T11b high vs low LUSC/DESCARTES_ORGANOGENESIS_EPENDYMAL_CELL.html]

Details for gene set DESCARTES\_ORGANOGENESIS\_EPENDYMAL\_CELL[GSEA]

|  || Dataset | T11b high vs low squamous\_GSEA\_Ranked |
| Phenotype | NoPhenotypeAvailable |
| Upregulated in class | na\_neg |
| GeneSet | DESCARTES\_ORGANOGENESIS\_EPENDYMAL\_CELL |
| Enrichment Score (ES) | -0.7124724 |
| Normalized Enrichment Score (NES) | -3.5286777 |
| Nominal p-value | 0.0 |
| FDR q-value | 0.0 |
| FWER p-Value | 0.0 |
Table: GSEA Results Summary

  

Fig 1: Enrichment plot: DESCARTES\_ORGANOGENESIS\_EPENDYMAL\_CELL      
 Profile of the Running ES Score & Positions of GeneSet Members on the Rank Ordered List

  

| SYMBOL | RANK IN GENE LIST | RANK METRIC SCORE | RUNNING ES | CORE ENRICHMENT || 1 | Pthlh | 299 | 1.307 | -0.0560 | No |
| 2 | Dusp14 | 948 | 0.506 | -0.2092 | No |
| 3 | Ezr | 1361 | -0.567 | -0.3033 | No |
| 4 | Tmem267 | 1510 | -0.597 | -0.3317 | No |
| 5 | Scrn2 | 1914 | -0.677 | -0.4220 | No |
| 6 | Shroom3 | 2396 | -0.790 | -0.5301 | No |
| 7 | Esyt3 | 2440 | -0.801 | -0.5298 | No |
| 8 | Cfap69 | 2462 | -0.807 | -0.5239 | No |
| 9 | Pidd1 | 2639 | -0.858 | -0.5557 | No |
| 10 | Rph3al | 2774 | -0.898 | -0.5765 | No |
| 11 | Ccdc149 | 3017 | -0.982 | -0.6229 | No |
| 12 | Zkscan7 | 3042 | -0.991 | -0.6153 | No |
| 13 | Clmn | 3260 | -1.099 | -0.6539 | No |
| 14 | Slc2a12 | 3280 | -1.106 | -0.6435 | No |
| 15 | Ttll3 | 3333 | -1.127 | -0.6410 | No |
| 16 | Cfap43 | 3438 | -1.179 | -0.6506 | No |
| 17 | Dkk3 | 3542 | -1.238 | -0.6591 | No |
| 18 | Dnaaf3 | 3671 | -1.356 | -0.6722 | No |
| 19 | Bbs9 | 3835 | -1.579 | -0.6909 | Yes |
| 20 | Ccdc157 | 3848 | -1.601 | -0.6720 | Yes |
| 21 | Wdr19 | 3916 | -1.754 | -0.6646 | Yes |
| 22 | 1700088E04Rik | 3919 | -1.759 | -0.6410 | Yes |
| 23 | Ccdc30 | 3941 | -1.802 | -0.6216 | Yes |
| 24 | Ccdc162 | 3947 | -1.815 | -0.5980 | Yes |
| 25 | Fam161a | 3964 | -1.898 | -0.5761 | Yes |
| 26 | Foxj1 | 3965 | -1.910 | -0.5500 | Yes |
| 27 | Vwa3b | 3973 | -1.964 | -0.5249 | Yes |
| 28 | Cfap126 | 3986 | -2.034 | -0.5000 | Yes |
| 29 | Nme5 | 3996 | -2.104 | -0.4735 | Yes |
| 30 | Cfap54 | 4007 | -2.127 | -0.4469 | Yes |
| 31 | Pih1d2 | 4015 | -2.197 | -0.4186 | Yes |
| 32 | Saxo2 | 4026 | -2.285 | -0.3899 | Yes |
| 33 | Capsl | 4029 | -2.297 | -0.3590 | Yes |
| 34 | Drc3 | 4038 | -2.344 | -0.3289 | Yes |
| 35 | Spata18 | 4044 | -2.423 | -0.2971 | Yes |
| 36 | Slc5a3 | 4047 | -2.434 | -0.2643 | Yes |
| 37 | Dynlrb2 | 4058 | -2.630 | -0.2308 | Yes |
| 38 | Ppil6 | 4065 | -2.661 | -0.1960 | Yes |
| 39 | Cfap65 | 4072 | -2.790 | -0.1593 | Yes |
| 40 | Rsph1 | 4073 | -2.805 | -0.1210 | Yes |
| 41 | Ttc21a | 4077 | -2.827 | -0.0831 | Yes |
| 42 | Cfap206 | 4078 | -2.882 | -0.0437 | Yes |
| 43 | Mlf1 | 4085 | -3.382 | 0.0010 | Yes |
Table: GSEA details [plain text format]

  

Fig 2: DESCARTES\_ORGANOGENESIS\_EPENDYMAL\_CELL: Random ES distribution      
 Gene set null distribution of ES for **DESCARTES\_ORGANOGENESIS\_EPENDYMAL\_CELL**

  
