# Supplementary material for: TMPRSS11B promotes an acidified microenvironment and immune suppression in squamous lung cancer
Source: EMBO Rep. 2025 Nov 10;26(24):6346–79. doi: 10.1038/s44319-025-00631-1 (PMC12714794; doi:10.1038/s44319-025-00631-1)
Supplement: Supplementary file 18 — Figure EV6 Source Data [file 44319_2025_631_MOESM18_ESM.zip › Figure EV6/EV6C-D/GSEA_Broad Institute_M8_T11b high vs low LUSC/DESCARTES_ORGANOGENESIS_EPITHELIAL_CELLS.html]

Details for gene set DESCARTES\_ORGANOGENESIS\_EPITHELIAL\_CELLS[GSEA]

|  || Dataset | T11b high vs low squamous\_GSEA\_Ranked |
| Phenotype | NoPhenotypeAvailable |
| Upregulated in class | na\_neg |
| GeneSet | DESCARTES\_ORGANOGENESIS\_EPITHELIAL\_CELLS |
| Enrichment Score (ES) | -0.17121424 |
| Normalized Enrichment Score (NES) | -0.9574786 |
| Nominal p-value | 0.52689874 |
| FDR q-value | 1.0 |
| FWER p-Value | 1.0 |
Table: GSEA Results Summary

  

Fig 1: Enrichment plot: DESCARTES\_ORGANOGENESIS\_EPITHELIAL\_CELLS      
 Profile of the Running ES Score & Positions of GeneSet Members on the Rank Ordered List

  

| SYMBOL | RANK IN GENE LIST | RANK METRIC SCORE | RUNNING ES | CORE ENRICHMENT || 1 | Gjb6 | 54 | 2.692 | 0.0228 | No |
| 2 | Krt14 | 56 | 2.659 | 0.0584 | No |
| 3 | Mfge8 | 160 | 1.751 | 0.0563 | No |
| 4 | Gjb2 | 187 | 1.644 | 0.0720 | No |
| 5 | Cxcl14 | 256 | 1.437 | 0.0744 | No |
| 6 | Pkp1 | 257 | 1.434 | 0.0937 | No |
| 7 | Grhl3 | 357 | 1.128 | 0.0843 | No |
| 8 | Barx2 | 380 | 1.095 | 0.0936 | No |
| 9 | Pkp3 | 402 | 1.044 | 0.1024 | No |
| 10 | Cdh3 | 417 | 1.023 | 0.1127 | No |
| 11 | Ppl | 438 | 0.994 | 0.1211 | No |
| 12 | Galnt18 | 439 | 0.992 | 0.1345 | No |
| 13 | Scnn1b | 494 | 0.907 | 0.1332 | No |
| 14 | Mal2 | 550 | 0.845 | 0.1309 | No |
| 15 | Plcd3 | 557 | 0.838 | 0.1407 | No |
| 16 | Crybg1 | 643 | 0.719 | 0.1293 | No |
| 17 | Krt5 | 709 | 0.661 | 0.1220 | No |
| 18 | Elf5 | 772 | 0.607 | 0.1147 | No |
| 19 | Gpr87 | 774 | 0.606 | 0.1227 | No |
| 20 | Nectin4 | 782 | 0.601 | 0.1290 | No |
| 21 | Cldn4 | 811 | 0.589 | 0.1300 | No |
| 22 | Sfn | 834 | 0.573 | 0.1322 | No |
| 23 | Col17a1 | 835 | 0.573 | 0.1399 | No |
| 24 | Fat2 | 904 | 0.529 | 0.1301 | No |
| 25 | Tacstd2 | 937 | 0.512 | 0.1291 | No |
| 26 | Esrp1 | 1143 | -0.530 | 0.0852 | No |
| 27 | Ildr1 | 1393 | -0.573 | 0.0310 | No |
| 28 | Esrp2 | 1476 | -0.588 | 0.0185 | No |
| 29 | Itga3 | 1535 | -0.600 | 0.0121 | No |
| 30 | Exph5 | 1566 | -0.606 | 0.0128 | No |
| 31 | Arhgef19 | 1621 | -0.614 | 0.0077 | No |
| 32 | Pwwp2b | 1626 | -0.616 | 0.0150 | No |
| 33 | Wnt4 | 1655 | -0.622 | 0.0164 | No |
| 34 | Arhgap8 | 1748 | -0.639 | 0.0021 | No |
| 35 | Galnt3 | 1794 | -0.648 | -0.0004 | No |
| 36 | Kdf1 | 1850 | -0.660 | -0.0052 | No |
| 37 | Sh3rf2 | 1904 | -0.674 | -0.0093 | No |
| 38 | Lnx1 | 1993 | -0.692 | -0.0218 | No |
| 39 | Patj | 2198 | -0.738 | -0.0627 | No |
| 40 | Tmem30b | 2267 | -0.755 | -0.0694 | No |
| 41 | Atp2c2 | 2304 | -0.763 | -0.0681 | No |
| 42 | Ttll10 | 2330 | -0.770 | -0.0640 | No |
| 43 | Fermt1 | 2395 | -0.789 | -0.0693 | No |
| 44 | Pof1b | 2504 | -0.819 | -0.0851 | No |
| 45 | Lsr | 2522 | -0.825 | -0.0782 | No |
| 46 | Fzd6 | 2556 | -0.834 | -0.0752 | No |
| 47 | Myo5c | 2583 | -0.843 | -0.0703 | No |
| 48 | Tmco6 | 2634 | -0.857 | -0.0712 | No |
| 49 | Kcnk1 | 2654 | -0.863 | -0.0643 | No |
| 50 | Arhgef38 | 2779 | -0.900 | -0.0831 | No |
| 51 | Mid1 | 2923 | -0.946 | -0.1059 | No |
| 52 | Grhl2 | 2980 | -0.967 | -0.1068 | No |
| 53 | Cdh1 | 2989 | -0.975 | -0.0957 | No |
| 54 | Lama5 | 3011 | -0.980 | -0.0877 | No |
| 55 | Lypd6b | 3155 | -1.039 | -0.1093 | No |
| 56 | Spint2 | 3222 | -1.076 | -0.1112 | No |
| 57 | Adrb1 | 3245 | -1.091 | -0.1020 | No |
| 58 | Rbbp8 | 3346 | -1.138 | -0.1115 | No |
| 59 | Tmprss2 | 3501 | -1.211 | -0.1335 | No |
| 60 | Ovol2 | 3553 | -1.249 | -0.1294 | No |
| 61 | Gabrp | 3722 | -1.409 | -0.1522 | Yes |
| 62 | Cftr | 3743 | -1.432 | -0.1379 | Yes |
| 63 | Ntf5 | 3755 | -1.444 | -0.1212 | Yes |
| 64 | Tmem220 | 3819 | -1.542 | -0.1161 | Yes |
| 65 | Unc5cl | 3828 | -1.565 | -0.0970 | Yes |
| 66 | Klf5 | 3839 | -1.583 | -0.0782 | Yes |
| 67 | Paqr5 | 3885 | -1.675 | -0.0668 | Yes |
| 68 | Cyp2s1 | 3937 | -1.783 | -0.0555 | Yes |
| 69 | Wfdc2 | 3939 | -1.799 | -0.0315 | Yes |
| 70 | Frem2 | 4046 | -2.432 | -0.0252 | Yes |
| 71 | Krt15 | 4063 | -2.644 | 0.0065 | Yes |
Table: GSEA details [plain text format]

  

Fig 2: DESCARTES\_ORGANOGENESIS\_EPITHELIAL\_CELLS: Random ES distribution      
 Gene set null distribution of ES for **DESCARTES\_ORGANOGENESIS\_EPITHELIAL\_CELLS**

  
