# Supplementary material for: TMPRSS11B promotes an acidified microenvironment and immune suppression in squamous lung cancer
Source: EMBO Rep. 2025 Nov 10;26(24):6346–79. doi: 10.1038/s44319-025-00631-1 (PMC12714794; doi:10.1038/s44319-025-00631-1)
Supplement: Supplementary file 18 — Figure EV6 Source Data [file 44319_2025_631_MOESM18_ESM.zip › Figure EV6/EV6C-D/GSEA_Broad Institute_M8_T11b high vs low LUSC/DESCARTES_ORGANOGENESIS_HEPATOCYTES.html]

Details for gene set DESCARTES\_ORGANOGENESIS\_HEPATOCYTES[GSEA]

|  || Dataset | T11b high vs low squamous\_GSEA\_Ranked |
| Phenotype | NoPhenotypeAvailable |
| Upregulated in class | na\_neg |
| GeneSet | DESCARTES\_ORGANOGENESIS\_HEPATOCYTES |
| Enrichment Score (ES) | -0.23322515 |
| Normalized Enrichment Score (NES) | -1.4769179 |
| Nominal p-value | 0.023183925 |
| FDR q-value | 0.2661988 |
| FWER p-Value | 0.994 |
Table: GSEA Results Summary

  

Fig 1: Enrichment plot: DESCARTES\_ORGANOGENESIS\_HEPATOCYTES      
 Profile of the Running ES Score & Positions of GeneSet Members on the Rank Ordered List

  

| SYMBOL | RANK IN GENE LIST | RANK METRIC SCORE | RUNNING ES | CORE ENRICHMENT || 1 | Stra6l | 35 | 3.108 | 0.0149 | No |
| 2 | Mmp19 | 121 | 1.964 | 0.0084 | No |
| 3 | Apoc1 | 164 | 1.742 | 0.0111 | No |
| 4 | Pygl | 208 | 1.570 | 0.0122 | No |
| 5 | Ndrg1 | 276 | 1.380 | 0.0058 | No |
| 6 | Slc36a2 | 284 | 1.352 | 0.0144 | No |
| 7 | F7 | 330 | 1.190 | 0.0121 | No |
| 8 | Slc25a48 | 332 | 1.186 | 0.0209 | No |
| 9 | Cd302 | 346 | 1.152 | 0.0264 | No |
| 10 | Ikbke | 356 | 1.129 | 0.0328 | No |
| 11 | Slc7a2 | 370 | 1.114 | 0.0380 | No |
| 12 | Fabp5 | 385 | 1.088 | 0.0427 | No |
| 13 | Cpm | 450 | 0.977 | 0.0340 | No |
| 14 | Plekhg6 | 458 | 0.964 | 0.0396 | No |
| 15 | Dhrs9 | 529 | 0.869 | 0.0286 | No |
| 16 | Tmem37 | 535 | 0.861 | 0.0339 | No |
| 17 | Trf | 601 | 0.771 | 0.0234 | No |
| 18 | Kifc3 | 645 | 0.717 | 0.0180 | No |
| 19 | Adk | 752 | 0.626 | -0.0040 | No |
| 20 | Itih4 | 757 | 0.622 | -0.0003 | No |
| 21 | Psat1 | 766 | 0.613 | 0.0024 | No |
| 22 | Fkbp11 | 841 | 0.570 | -0.0119 | No |
| 23 | Mocos | 899 | 0.534 | -0.0223 | No |
| 24 | Hdlbp | 984 | -0.503 | -0.0396 | No |
| 25 | Tmem205 | 1023 | -0.509 | -0.0453 | No |
| 26 | Baiap2l1 | 1058 | -0.515 | -0.0500 | No |
| 27 | Gldc | 1118 | -0.526 | -0.0609 | No |
| 28 | Lmbrd2 | 1159 | -0.531 | -0.0669 | No |
| 29 | Dgat2 | 1171 | -0.534 | -0.0656 | No |
| 30 | 2310039H08Rik | 1194 | -0.538 | -0.0671 | No |
| 31 | R3hdm2 | 1249 | -0.548 | -0.0765 | No |
| 32 | Fah | 1257 | -0.549 | -0.0741 | No |
| 33 | Ngef | 1311 | -0.558 | -0.0832 | No |
| 34 | Slc39a4 | 1420 | -0.579 | -0.1061 | No |
| 35 | Plekhg3 | 1422 | -0.580 | -0.1019 | No |
| 36 | Slc35d2 | 1426 | -0.580 | -0.0982 | No |
| 37 | Hnf4a | 1475 | -0.588 | -0.1059 | No |
| 38 | Tst | 1483 | -0.590 | -0.1031 | No |
| 39 | Ugt2b34 | 1545 | -0.601 | -0.1139 | No |
| 40 | Pgm3 | 1586 | -0.609 | -0.1194 | No |
| 41 | Chmp4c | 1654 | -0.621 | -0.1316 | No |
| 42 | Nostrin | 1658 | -0.622 | -0.1276 | No |
| 43 | Aldh3a2 | 1699 | -0.630 | -0.1329 | No |
| 44 | Akr1c19 | 1714 | -0.632 | -0.1316 | No |
| 45 | Ttc38 | 1759 | -0.641 | -0.1378 | No |
| 46 | Siah2 | 1781 | -0.644 | -0.1382 | No |
| 47 | Bphl | 1815 | -0.651 | -0.1415 | No |
| 48 | Hjurp | 1839 | -0.659 | -0.1423 | No |
| 49 | Rassf6 | 1852 | -0.661 | -0.1403 | No |
| 50 | Acnat1 | 1897 | -0.672 | -0.1463 | No |
| 51 | Chdh | 1954 | -0.686 | -0.1552 | No |
| 52 | Aqp11 | 1981 | -0.689 | -0.1565 | No |
| 53 | Pctp | 1982 | -0.690 | -0.1512 | No |
| 54 | Acad11 | 2020 | -0.696 | -0.1553 | No |
| 55 | Gstm1 | 2049 | -0.704 | -0.1570 | No |
| 56 | Cyp3a13 | 2147 | -0.728 | -0.1759 | No |
| 57 | 0610040J01Rik | 2172 | -0.733 | -0.1763 | No |
| 58 | Fpgs | 2193 | -0.737 | -0.1758 | No |
| 59 | Ccdc125 | 2236 | -0.748 | -0.1807 | No |
| 60 | Pcyt2 | 2237 | -0.748 | -0.1750 | No |
| 61 | Acaa2 | 2240 | -0.748 | -0.1698 | No |
| 62 | Nipsnap1 | 2282 | -0.758 | -0.1743 | No |
| 63 | 2810459M11Rik | 2302 | -0.763 | -0.1733 | No |
| 64 | Ctps2 | 2360 | -0.776 | -0.1818 | No |
| 65 | Fggy | 2369 | -0.779 | -0.1778 | No |
| 66 | Agmo | 2386 | -0.786 | -0.1759 | No |
| 67 | Insr | 2405 | -0.793 | -0.1744 | No |
| 68 | Bche | 2422 | -0.797 | -0.1723 | No |
| 69 | Hadh | 2449 | -0.804 | -0.1728 | No |
| 70 | Gstz1 | 2467 | -0.810 | -0.1709 | No |
| 71 | Slc19a2 | 2571 | -0.840 | -0.1905 | No |
| 72 | Zfp395 | 2572 | -0.841 | -0.1841 | No |
| 73 | Cbx7 | 2587 | -0.844 | -0.1812 | No |
| 74 | Slc25a15 | 2599 | -0.848 | -0.1775 | No |
| 75 | Acsl1 | 2609 | -0.850 | -0.1732 | No |
| 76 | Snd1 | 2630 | -0.856 | -0.1718 | No |
| 77 | Ddt | 2681 | -0.869 | -0.1778 | No |
| 78 | Cryz | 2889 | -0.936 | -0.2229 | No |
| 79 | Ly75 | 2931 | -0.950 | -0.2260 | Yes |
| 80 | Cfi | 2950 | -0.956 | -0.2232 | Yes |
| 81 | Rhpn2 | 2952 | -0.957 | -0.2162 | Yes |
| 82 | Ocln | 2957 | -0.959 | -0.2099 | Yes |
| 83 | Mettl26 | 2965 | -0.961 | -0.2043 | Yes |
| 84 | Grk3 | 3015 | -0.981 | -0.2092 | Yes |
| 85 | Mst1 | 3084 | -1.012 | -0.2186 | Yes |
| 86 | Asl | 3098 | -1.016 | -0.2142 | Yes |
| 87 | Tmem143 | 3106 | -1.020 | -0.2081 | Yes |
| 88 | Inpp5f | 3127 | -1.029 | -0.2053 | Yes |
| 89 | Pik3c2g | 3154 | -1.039 | -0.2040 | Yes |
| 90 | Lactb2 | 3168 | -1.047 | -0.1993 | Yes |
| 91 | Galm | 3177 | -1.050 | -0.1933 | Yes |
| 92 | Gstt2 | 3190 | -1.059 | -0.1882 | Yes |
| 93 | Slc25a10 | 3196 | -1.061 | -0.1814 | Yes |
| 94 | Tcea3 | 3242 | -1.088 | -0.1844 | Yes |
| 95 | Dop1b | 3269 | -1.102 | -0.1826 | Yes |
| 96 | Ass1 | 3295 | -1.111 | -0.1804 | Yes |
| 97 | Sfxn2 | 3308 | -1.115 | -0.1750 | Yes |
| 98 | Abcd3 | 3317 | -1.119 | -0.1684 | Yes |
| 99 | Atp8b1 | 3349 | -1.140 | -0.1676 | Yes |
| 100 | Stard10 | 3354 | -1.141 | -0.1599 | Yes |
| 101 | Slc22a18 | 3393 | -1.158 | -0.1606 | Yes |
| 102 | Sirt3 | 3425 | -1.174 | -0.1595 | Yes |
| 103 | Gsta3 | 3465 | -1.196 | -0.1602 | Yes |
| 104 | Pdia5 | 3468 | -1.197 | -0.1516 | Yes |
| 105 | Aldh6a1 | 3480 | -1.202 | -0.1452 | Yes |
| 106 | Gpd1 | 3502 | -1.211 | -0.1412 | Yes |
| 107 | Cutc | 3503 | -1.212 | -0.1320 | Yes |
| 108 | Mcrip2 | 3506 | -1.215 | -0.1232 | Yes |
| 109 | Steap2 | 3554 | -1.250 | -0.1256 | Yes |
| 110 | Tmem176a | 3571 | -1.266 | -0.1199 | Yes |
| 111 | Faah | 3584 | -1.275 | -0.1132 | Yes |
| 112 | Phyh | 3626 | -1.310 | -0.1136 | Yes |
| 113 | Abcc10 | 3642 | -1.323 | -0.1073 | Yes |
| 114 | Echdc2 | 3645 | -1.326 | -0.0977 | Yes |
| 115 | Nadsyn1 | 3683 | -1.364 | -0.0966 | Yes |
| 116 | Tmem51 | 3687 | -1.367 | -0.0869 | Yes |
| 117 | Akr1c13 | 3713 | -1.397 | -0.0826 | Yes |
| 118 | Aspa | 3744 | -1.432 | -0.0792 | Yes |
| 119 | Hpn | 3768 | -1.456 | -0.0739 | Yes |
| 120 | Chka | 3801 | -1.515 | -0.0704 | Yes |
| 121 | Mgst2 | 3820 | -1.548 | -0.0632 | Yes |
| 122 | Slc44a3 | 3849 | -1.605 | -0.0580 | Yes |
| 123 | Itih2 | 3869 | -1.646 | -0.0502 | Yes |
| 124 | Hgfac | 3920 | -1.761 | -0.0494 | Yes |
| 125 | Tfcp2l1 | 3955 | -1.839 | -0.0440 | Yes |
| 126 | Sytl5 | 3958 | -1.852 | -0.0303 | Yes |
| 127 | Hmgcs2 | 4048 | -2.436 | -0.0342 | Yes |
| 128 | Krt20 | 4074 | -2.807 | -0.0191 | Yes |
| 129 | Baiap2l2 | 4079 | -2.967 | 0.0025 | Yes |
Table: GSEA details [plain text format]

  

Fig 2: DESCARTES\_ORGANOGENESIS\_HEPATOCYTES: Random ES distribution      
 Gene set null distribution of ES for **DESCARTES\_ORGANOGENESIS\_HEPATOCYTES**

  
