# Supplementary material for: TMPRSS11B promotes an acidified microenvironment and immune suppression in squamous lung cancer
Source: EMBO Rep. 2025 Nov 10;26(24):6346–79. doi: 10.1038/s44319-025-00631-1 (PMC12714794; doi:10.1038/s44319-025-00631-1)
Supplement: Supplementary file 18 — Figure EV6 Source Data [file 44319_2025_631_MOESM18_ESM.zip › Figure EV6/EV6C-D/GSEA_Broad Institute_M8_T11b high vs low LUSC/DESCARTES_ORGANOGENESIS_MYOCYTES.html]

Details for gene set DESCARTES\_ORGANOGENESIS\_MYOCYTES[GSEA]

|  || Dataset | T11b high vs low squamous\_GSEA\_Ranked |
| Phenotype | NoPhenotypeAvailable |
| Upregulated in class | na\_neg |
| GeneSet | DESCARTES\_ORGANOGENESIS\_MYOCYTES |
| Enrichment Score (ES) | -0.32987937 |
| Normalized Enrichment Score (NES) | -1.2069505 |
| Nominal p-value | 0.25378153 |
| FDR q-value | 0.64670455 |
| FWER p-Value | 1.0 |
Table: GSEA Results Summary

  

Fig 1: Enrichment plot: DESCARTES\_ORGANOGENESIS\_MYOCYTES      
 Profile of the Running ES Score & Positions of GeneSet Members on the Rank Ordered List

  

| SYMBOL | RANK IN GENE LIST | RANK METRIC SCORE | RUNNING ES | CORE ENRICHMENT || 1 | Frmpd1 | 48 | 2.773 | 0.1229 | No |
| 2 | Cdkn1a | 192 | 1.625 | 0.1667 | No |
| 3 | Gramd1b | 699 | 0.668 | 0.0749 | No |
| 4 | Clcn5 | 1698 | -0.630 | -0.1396 | No |
| 5 | Prss36 | 1812 | -0.651 | -0.1357 | No |
| 6 | Ptgis | 2464 | -0.808 | -0.2563 | No |
| 7 | Cdkn1c | 2722 | -0.882 | -0.2766 | No |
| 8 | Ncoa1 | 2940 | -0.953 | -0.2836 | Yes |
| 9 | Cep41 | 2968 | -0.962 | -0.2435 | Yes |
| 10 | Cd82 | 2976 | -0.965 | -0.1984 | Yes |
| 11 | Macrod1 | 3332 | -1.127 | -0.2308 | Yes |
| 12 | Rnf217 | 3486 | -1.204 | -0.2099 | Yes |
| 13 | Igdcc4 | 3512 | -1.217 | -0.1570 | Yes |
| 14 | Adh1 | 3681 | -1.362 | -0.1321 | Yes |
| 15 | Vgll3 | 3770 | -1.457 | -0.0829 | Yes |
| 16 | Sytl2 | 3784 | -1.480 | -0.0143 | Yes |
| 17 | Tmem38a | 3951 | -1.831 | 0.0339 | Yes |
Table: GSEA details [plain text format]

  

Fig 2: DESCARTES\_ORGANOGENESIS\_MYOCYTES: Random ES distribution      
 Gene set null distribution of ES for **DESCARTES\_ORGANOGENESIS\_MYOCYTES**

  
