# Supplementary material for: TMPRSS11B promotes an acidified microenvironment and immune suppression in squamous lung cancer
Source: EMBO Rep. 2025 Nov 10;26(24):6346–79. doi: 10.1038/s44319-025-00631-1 (PMC12714794; doi:10.1038/s44319-025-00631-1)
Supplement: Supplementary file 18 — Figure EV6 Source Data [file 44319_2025_631_MOESM18_ESM.zip › Figure EV6/EV6C-D/GSEA_Broad Institute_M8_T11b high vs low LUSC/DESCARTES_ORGANOGENESIS_PRIMITIVE_ERYTHROID_LINEAGE.html]

Details for gene set DESCARTES\_ORGANOGENESIS\_PRIMITIVE\_ERYTHROID\_LINEAGE[GSEA]

|  || Dataset | T11b high vs low squamous\_GSEA\_Ranked |
| Phenotype | NoPhenotypeAvailable |
| Upregulated in class | na\_neg |
| GeneSet | DESCARTES\_ORGANOGENESIS\_PRIMITIVE\_ERYTHROID\_LINEAGE |
| Enrichment Score (ES) | -0.093958765 |
| Normalized Enrichment Score (NES) | -0.5900492 |
| Nominal p-value | 0.9754501 |
| FDR q-value | 1.0 |
| FWER p-Value | 1.0 |
Table: GSEA Results Summary

  

Fig 1: Enrichment plot: DESCARTES\_ORGANOGENESIS\_PRIMITIVE\_ERYTHROID\_LINEAGE      
 Profile of the Running ES Score & Positions of GeneSet Members on the Rank Ordered List

  

| SYMBOL | RANK IN GENE LIST | RANK METRIC SCORE | RUNNING ES | CORE ENRICHMENT || 1 | Fth1 | 147 | 1.835 | -0.0203 | No |
| 2 | Pik3cb | 149 | 1.832 | -0.0038 | No |
| 3 | Spns2 | 218 | 1.525 | -0.0070 | No |
| 4 | Dusp1 | 239 | 1.468 | 0.0014 | No |
| 5 | Csf2rb | 243 | 1.458 | 0.0140 | No |
| 6 | Acp5 | 247 | 1.450 | 0.0265 | No |
| 7 | Pim1 | 262 | 1.425 | 0.0360 | No |
| 8 | Lpcat1 | 272 | 1.389 | 0.0464 | No |
| 9 | Creg1 | 280 | 1.373 | 0.0572 | No |
| 10 | Ehd1 | 313 | 1.237 | 0.0604 | No |
| 11 | St3gal6 | 416 | 1.025 | 0.0441 | No |
| 12 | Gadd45a | 473 | 0.944 | 0.0386 | No |
| 13 | Mindy2 | 478 | 0.927 | 0.0461 | No |
| 14 | Ppp1r15a | 552 | 0.843 | 0.0354 | No |
| 15 | Cat | 639 | 0.721 | 0.0203 | No |
| 16 | Tfrc | 668 | 0.696 | 0.0196 | No |
| 17 | Tinagl1 | 685 | 0.683 | 0.0218 | No |
| 18 | Cited2 | 695 | 0.672 | 0.0257 | No |
| 19 | Hipk2 | 725 | 0.651 | 0.0243 | No |
| 20 | Shmt2 | 787 | 0.598 | 0.0144 | No |
| 21 | Ccdc71l | 788 | 0.597 | 0.0199 | No |
| 22 | Zc3hav1 | 802 | 0.592 | 0.0220 | No |
| 23 | Cks2 | 812 | 0.588 | 0.0251 | No |
| 24 | Ptp4a3 | 813 | 0.588 | 0.0305 | No |
| 25 | Rexo2 | 839 | 0.571 | 0.0294 | No |
| 26 | Fam117a | 863 | 0.563 | 0.0288 | No |
| 27 | Nmnat3 | 864 | 0.561 | 0.0339 | No |
| 28 | Eif2ak1 | 893 | 0.541 | 0.0318 | No |
| 29 | Gtpbp2 | 907 | 0.529 | 0.0333 | No |
| 30 | Ackr3 | 918 | 0.524 | 0.0356 | No |
| 31 | Cpeb4 | 929 | 0.516 | 0.0378 | No |
| 32 | Ucp2 | 979 | -0.502 | 0.0301 | No |
| 33 | Brpf3 | 1042 | -0.512 | 0.0191 | No |
| 34 | Ppm1g | 1043 | -0.512 | 0.0238 | No |
| 35 | Pfkfb2 | 1044 | -0.513 | 0.0285 | No |
| 36 | Iars2 | 1081 | -0.519 | 0.0242 | No |
| 37 | Mpv17l2 | 1110 | -0.524 | 0.0219 | No |
| 38 | Sri | 1116 | -0.525 | 0.0254 | No |
| 39 | Urod | 1150 | -0.530 | 0.0220 | No |
| 40 | Ankrd54 | 1157 | -0.531 | 0.0253 | No |
| 41 | Hipk1 | 1188 | -0.537 | 0.0227 | No |
| 42 | Ino80c | 1195 | -0.538 | 0.0261 | No |
| 43 | Pnpo | 1265 | -0.549 | 0.0137 | No |
| 44 | Tmem131 | 1315 | -0.559 | 0.0065 | No |
| 45 | Ndufs3 | 1349 | -0.565 | 0.0033 | No |
| 46 | Rfesd | 1383 | -0.571 | 0.0002 | No |
| 47 | Ogfod2 | 1417 | -0.579 | -0.0028 | No |
| 48 | Hmbs | 1418 | -0.579 | 0.0025 | No |
| 49 | Uevld | 1455 | -0.586 | -0.0012 | No |
| 50 | Prdx6 | 1466 | -0.587 | 0.0016 | No |
| 51 | Cd59a | 1511 | -0.597 | -0.0040 | No |
| 52 | Kif2a | 1541 | -0.600 | -0.0058 | No |
| 53 | Ddx27 | 1627 | -0.616 | -0.0216 | No |
| 54 | Telo2 | 1648 | -0.620 | -0.0210 | No |
| 55 | Btrc | 1681 | -0.627 | -0.0233 | No |
| 56 | Slc25a39 | 1694 | -0.629 | -0.0206 | No |
| 57 | Taf10 | 1702 | -0.630 | -0.0166 | No |
| 58 | Fech | 1712 | -0.632 | -0.0131 | No |
| 59 | Cited4 | 1764 | -0.642 | -0.0201 | No |
| 60 | Piezo1 | 1820 | -0.653 | -0.0280 | No |
| 61 | Nans | 1830 | -0.656 | -0.0242 | No |
| 62 | Clp1 | 1846 | -0.660 | -0.0220 | No |
| 63 | Cox17 | 1863 | -0.664 | -0.0199 | No |
| 64 | Sgk3 | 1873 | -0.667 | -0.0161 | No |
| 65 | Irs2 | 1906 | -0.674 | -0.0180 | No |
| 66 | Abhd4 | 1960 | -0.686 | -0.0251 | No |
| 67 | Ccnd3 | 2040 | -0.700 | -0.0386 | No |
| 68 | Ift140 | 2087 | -0.712 | -0.0437 | No |
| 69 | Cops6 | 2216 | -0.743 | -0.0692 | No |
| 70 | Spty2d1 | 2259 | -0.753 | -0.0729 | No |
| 71 | Dhrs11 | 2264 | -0.754 | -0.0670 | No |
| 72 | Afg3l2 | 2280 | -0.757 | -0.0638 | No |
| 73 | Polr1b | 2289 | -0.759 | -0.0589 | No |
| 74 | Tmem238 | 2313 | -0.766 | -0.0577 | No |
| 75 | Chac2 | 2329 | -0.769 | -0.0545 | No |
| 76 | Tmem184a | 2385 | -0.785 | -0.0611 | No |
| 77 | Rhbdd1 | 2406 | -0.793 | -0.0589 | No |
| 78 | Galnt10 | 2483 | -0.815 | -0.0706 | No |
| 79 | Bsg | 2530 | -0.827 | -0.0747 | No |
| 80 | Vangl1 | 2569 | -0.839 | -0.0766 | No |
| 81 | Stradb | 2610 | -0.850 | -0.0789 | No |
| 82 | Fam241a | 2640 | -0.858 | -0.0783 | No |
| 83 | Eif3f | 2655 | -0.863 | -0.0740 | No |
| 84 | Reep6 | 2670 | -0.867 | -0.0696 | No |
| 85 | Tnfrsf21 | 2673 | -0.868 | -0.0622 | No |
| 86 | Tatdn3 | 2686 | -0.871 | -0.0572 | No |
| 87 | Akip1 | 2692 | -0.874 | -0.0505 | No |
| 88 | Tnrc6b | 2705 | -0.877 | -0.0455 | No |
| 89 | Rabac1 | 2744 | -0.888 | -0.0470 | No |
| 90 | Golph3l | 2773 | -0.897 | -0.0458 | No |
| 91 | Tmem147 | 2787 | -0.902 | -0.0408 | No |
| 92 | Slc6a9 | 2788 | -0.902 | -0.0326 | No |
| 93 | Pdik1l | 2827 | -0.915 | -0.0338 | No |
| 94 | Tspan33 | 2974 | -0.965 | -0.0618 | No |
| 95 | Zfp524 | 3013 | -0.981 | -0.0624 | No |
| 96 | Lcmt2 | 3019 | -0.982 | -0.0547 | No |
| 97 | Prmt3 | 3044 | -0.991 | -0.0517 | No |
| 98 | Arrdc2 | 3047 | -0.994 | -0.0431 | No |
| 99 | Maz | 3048 | -0.994 | -0.0340 | No |
| 100 | Ano1 | 3053 | -0.995 | -0.0259 | No |
| 101 | Cep70 | 3065 | -1.003 | -0.0195 | No |
| 102 | Mrm1 | 3099 | -1.017 | -0.0185 | No |
| 103 | Cyb5a | 3121 | -1.027 | -0.0144 | No |
| 104 | Aldh9a1 | 3166 | -1.046 | -0.0160 | No |
| 105 | Ttc39a | 3186 | -1.056 | -0.0111 | No |
| 106 | Zfpm1 | 3334 | -1.127 | -0.0378 | No |
| 107 | Plek2 | 3383 | -1.154 | -0.0394 | No |
| 108 | Mthfd2 | 3458 | -1.191 | -0.0471 | No |
| 109 | Taf5l | 3581 | -1.273 | -0.0662 | No |
| 110 | Nanp | 3692 | -1.373 | -0.0814 | Yes |
| 111 | Flt3l | 3704 | -1.390 | -0.0715 | Yes |
| 112 | Klhdc2 | 3753 | -1.442 | -0.0704 | Yes |
| 113 | Asb1 | 3814 | -1.535 | -0.0715 | Yes |
| 114 | Tmc6 | 3881 | -1.660 | -0.0729 | Yes |
| 115 | Gcnt1 | 3892 | -1.706 | -0.0599 | Yes |
| 116 | Dhrs13 | 3911 | -1.745 | -0.0484 | Yes |
| 117 | Slc26a2 | 3936 | -1.780 | -0.0382 | Yes |
| 118 | St3gal5 | 3960 | -1.876 | -0.0269 | Yes |
| 119 | Hdac11 | 3972 | -1.950 | -0.0118 | Yes |
| 120 | Tent5c | 3985 | -2.032 | 0.0037 | Yes |
| 121 | Gm867 | 4045 | -2.432 | 0.0111 | Yes |
Table: GSEA details [plain text format]

  

Fig 2: DESCARTES\_ORGANOGENESIS\_PRIMITIVE\_ERYTHROID\_LINEAGE: Random ES distribution      
 Gene set null distribution of ES for **DESCARTES\_ORGANOGENESIS\_PRIMITIVE\_ERYTHROID\_LINEAGE**

  
