# Supplementary material for: TMPRSS11B promotes an acidified microenvironment and immune suppression in squamous lung cancer
Source: EMBO Rep. 2025 Nov 10;26(24):6346–79. doi: 10.1038/s44319-025-00631-1 (PMC12714794; doi:10.1038/s44319-025-00631-1)
Supplement: Supplementary file 18 — Figure EV6 Source Data [file 44319_2025_631_MOESM18_ESM.zip › Figure EV6/EV6C-D/GSEA_Broad Institute_M8_T11b high vs low LUSC/DESCARTES_ORGANOGENESIS_SCHWANN_CELL_PRECURSOR.html]

Details for gene set DESCARTES\_ORGANOGENESIS\_SCHWANN\_CELL\_PRECURSOR[GSEA]

|  || Dataset | T11b high vs low squamous\_GSEA\_Ranked |
| Phenotype | NoPhenotypeAvailable |
| Upregulated in class | na\_neg |
| GeneSet | DESCARTES\_ORGANOGENESIS\_SCHWANN\_CELL\_PRECURSOR |
| Enrichment Score (ES) | -0.19668911 |
| Normalized Enrichment Score (NES) | -0.7649728 |
| Nominal p-value | 0.7575251 |
| FDR q-value | 1.0 |
| FWER p-Value | 1.0 |
Table: GSEA Results Summary

  

Fig 1: Enrichment plot: DESCARTES\_ORGANOGENESIS\_SCHWANN\_CELL\_PRECURSOR      
 Profile of the Running ES Score & Positions of GeneSet Members on the Rank Ordered List

  

| SYMBOL | RANK IN GENE LIST | RANK METRIC SCORE | RUNNING ES | CORE ENRICHMENT || 1 | Mal | 329 | 1.191 | -0.0099 | No |
| 2 | Ubl3 | 751 | 0.627 | -0.0760 | No |
| 3 | Afap1l2 | 843 | 0.569 | -0.0644 | No |
| 4 | Matn2 | 876 | 0.554 | -0.0393 | No |
| 5 | Tspan7 | 919 | 0.523 | -0.0184 | No |
| 6 | Fhdc1 | 1170 | -0.534 | -0.0481 | No |
| 7 | Erbb3 | 1362 | -0.568 | -0.0612 | No |
| 8 | Stard13 | 1401 | -0.575 | -0.0363 | No |
| 9 | Wdfy1 | 1480 | -0.589 | -0.0203 | No |
| 10 | Btbd3 | 1540 | -0.600 | 0.0009 | No |
| 11 | Atp10b | 1574 | -0.607 | 0.0290 | No |
| 12 | Itprid2 | 2044 | -0.702 | -0.0444 | No |
| 13 | Otud1 | 2290 | -0.760 | -0.0593 | No |
| 14 | Rasa2 | 2847 | -0.924 | -0.1409 | Yes |
| 15 | Tmem117 | 3075 | -1.008 | -0.1366 | Yes |
| 16 | Cyp2j6 | 3094 | -1.015 | -0.0806 | Yes |
| 17 | Ednrb | 3353 | -1.140 | -0.0760 | Yes |
| 18 | Dagla | 3620 | -1.306 | -0.0636 | Yes |
| 19 | Olfml2a | 3782 | -1.478 | -0.0151 | Yes |
| 20 | Lmo4 | 3802 | -1.515 | 0.0705 | Yes |
Table: GSEA details [plain text format]

  

Fig 2: DESCARTES\_ORGANOGENESIS\_SCHWANN\_CELL\_PRECURSOR: Random ES distribution      
 Gene set null distribution of ES for **DESCARTES\_ORGANOGENESIS\_SCHWANN\_CELL\_PRECURSOR**

  
