# Supplementary material for: TMPRSS11B promotes an acidified microenvironment and immune suppression in squamous lung cancer
Source: EMBO Rep. 2025 Nov 10;26(24):6346–79. doi: 10.1038/s44319-025-00631-1 (PMC12714794; doi:10.1038/s44319-025-00631-1)
Supplement: Supplementary file 18 — Figure EV6 Source Data [file 44319_2025_631_MOESM18_ESM.zip › Figure EV6/EV6C-D/GSEA_Broad Institute_M8_T11b high vs low LUSC/DESCARTES_ORGANOGENESIS_SENSORY_NEURONS.html]

Details for gene set DESCARTES\_ORGANOGENESIS\_SENSORY\_NEURONS[GSEA]

|  || Dataset | T11b high vs low squamous\_GSEA\_Ranked |
| Phenotype | NoPhenotypeAvailable |
| Upregulated in class | na\_neg |
| GeneSet | DESCARTES\_ORGANOGENESIS\_SENSORY\_NEURONS |
| Enrichment Score (ES) | -0.43631387 |
| Normalized Enrichment Score (NES) | -1.6166675 |
| Nominal p-value | 0.03350084 |
| FDR q-value | 0.2129139 |
| FWER p-Value | 0.852 |
Table: GSEA Results Summary

  

Fig 1: Enrichment plot: DESCARTES\_ORGANOGENESIS\_SENSORY\_NEURONS      
 Profile of the Running ES Score & Positions of GeneSet Members on the Rank Ordered List

  

| SYMBOL | RANK IN GENE LIST | RANK METRIC SCORE | RUNNING ES | CORE ENRICHMENT || 1 | Mreg | 224 | 1.514 | 0.0242 | No |
| 2 | Rnf150 | 457 | 0.967 | 0.0178 | No |
| 3 | Tbc1d9 | 648 | 0.714 | 0.0085 | No |
| 4 | Csnk1g1 | 1051 | -0.513 | -0.0633 | No |
| 5 | Prkce | 1609 | -0.612 | -0.1681 | No |
| 6 | Pde4dip | 2060 | -0.712 | -0.2413 | No |
| 7 | Tacc2 | 2704 | -0.876 | -0.3533 | No |
| 8 | Ankrd24 | 3043 | -0.991 | -0.3845 | Yes |
| 9 | Rapgef6 | 3087 | -1.012 | -0.3421 | Yes |
| 10 | B4galnt3 | 3314 | -1.118 | -0.3392 | Yes |
| 11 | Kif13b | 3318 | -1.119 | -0.2814 | Yes |
| 12 | Mapk8ip1 | 3443 | -1.182 | -0.2500 | Yes |
| 13 | Ccdc92 | 3565 | -1.257 | -0.2140 | Yes |
| 14 | Synpo | 3604 | -1.290 | -0.1558 | Yes |
| 15 | Kif19a | 3895 | -1.712 | -0.1375 | Yes |
| 16 | Cerkl | 3921 | -1.762 | -0.0515 | Yes |
| 17 | Tesc | 3925 | -1.768 | 0.0403 | Yes |
Table: GSEA details [plain text format]

  

Fig 2: DESCARTES\_ORGANOGENESIS\_SENSORY\_NEURONS: Random ES distribution      
 Gene set null distribution of ES for **DESCARTES\_ORGANOGENESIS\_SENSORY\_NEURONS**

  
