# Supplementary material for: TMPRSS11B promotes an acidified microenvironment and immune suppression in squamous lung cancer
Source: EMBO Rep. 2025 Nov 10;26(24):6346–79. doi: 10.1038/s44319-025-00631-1 (PMC12714794; doi:10.1038/s44319-025-00631-1)
Supplement: Supplementary file 18 — Figure EV6 Source Data [file 44319_2025_631_MOESM18_ESM.zip › Figure EV6/EV6C-D/GSEA_Broad Institute_M8_T11b high vs low LUSC/DESCARTES_ORGANOGENESIS_STROMAL_CELLS.html]

Details for gene set DESCARTES\_ORGANOGENESIS\_STROMAL\_CELLS[GSEA]

|  || Dataset | T11b high vs low squamous\_GSEA\_Ranked |
| Phenotype | NoPhenotypeAvailable |
| Upregulated in class | na\_neg |
| GeneSet | DESCARTES\_ORGANOGENESIS\_STROMAL\_CELLS |
| Enrichment Score (ES) | -0.16778296 |
| Normalized Enrichment Score (NES) | -0.8458407 |
| Nominal p-value | 0.6871069 |
| FDR q-value | 1.0 |
| FWER p-Value | 1.0 |
Table: GSEA Results Summary

  

Fig 1: Enrichment plot: DESCARTES\_ORGANOGENESIS\_STROMAL\_CELLS      
 Profile of the Running ES Score & Positions of GeneSet Members on the Rank Ordered List

  

| SYMBOL | RANK IN GENE LIST | RANK METRIC SCORE | RUNNING ES | CORE ENRICHMENT || 1 | Zswim4 | 300 | 1.306 | -0.0398 | No |
| 2 | Oaz2 | 610 | 0.756 | -0.0963 | No |
| 3 | D16Ertd472e | 612 | 0.752 | -0.0768 | No |
| 4 | Terf2ip | 981 | -0.503 | -0.1545 | Yes |
| 5 | Tmem11 | 995 | -0.504 | -0.1445 | Yes |
| 6 | Nacc1 | 1031 | -0.510 | -0.1397 | Yes |
| 7 | Trp53bp2 | 1123 | -0.526 | -0.1483 | Yes |
| 8 | Rdh14 | 1196 | -0.538 | -0.1520 | Yes |
| 9 | Ercc8 | 1214 | -0.542 | -0.1419 | Yes |
| 10 | Fbxo4 | 1247 | -0.547 | -0.1354 | Yes |
| 11 | Prpf38a | 1341 | -0.564 | -0.1435 | Yes |
| 12 | Rbbp6 | 1355 | -0.566 | -0.1318 | Yes |
| 13 | Avil | 1399 | -0.575 | -0.1273 | Yes |
| 14 | Cand1 | 1428 | -0.580 | -0.1190 | Yes |
| 15 | Arpc5l | 1558 | -0.604 | -0.1350 | Yes |
| 16 | Rab5a | 1588 | -0.609 | -0.1261 | Yes |
| 17 | Ppa1 | 1606 | -0.612 | -0.1142 | Yes |
| 18 | Mycbp | 1637 | -0.618 | -0.1053 | Yes |
| 19 | Cnot6 | 1682 | -0.627 | -0.0997 | Yes |
| 20 | Ythdc1 | 1786 | -0.646 | -0.1082 | Yes |
| 21 | Ints12 | 1814 | -0.651 | -0.0977 | Yes |
| 22 | Golph3 | 1841 | -0.659 | -0.0868 | Yes |
| 23 | Lrrc41 | 1880 | -0.670 | -0.0785 | Yes |
| 24 | Togaram1 | 1990 | -0.692 | -0.0872 | Yes |
| 25 | Rtca | 2043 | -0.702 | -0.0816 | Yes |
| 26 | Zfp664 | 2047 | -0.703 | -0.0639 | Yes |
| 27 | Snx33 | 2257 | -0.753 | -0.0957 | Yes |
| 28 | Srsf3 | 2381 | -0.784 | -0.1055 | Yes |
| 29 | Fam83e | 2427 | -0.799 | -0.0956 | Yes |
| 30 | Zfp212 | 2432 | -0.799 | -0.0755 | Yes |
| 31 | Pcnp | 2520 | -0.824 | -0.0753 | Yes |
| 32 | 4833439L19Rik | 2588 | -0.844 | -0.0697 | Yes |
| 33 | Mfsd9 | 2608 | -0.850 | -0.0520 | Yes |
| 34 | Kpna6 | 2684 | -0.870 | -0.0476 | Yes |
| 35 | Coa7 | 2711 | -0.878 | -0.0309 | Yes |
| 36 | Asf1a | 2749 | -0.889 | -0.0167 | Yes |
| 37 | Gabpb1 | 2870 | -0.931 | -0.0218 | Yes |
| 38 | Ddx52 | 2905 | -0.942 | -0.0054 | Yes |
| 39 | Bri3 | 2942 | -0.954 | 0.0108 | Yes |
| 40 | Psen1 | 3136 | -1.034 | -0.0097 | Yes |
| 41 | Exog | 3208 | -1.068 | 0.0009 | Yes |
| 42 | Taf7 | 3351 | -1.140 | -0.0042 | Yes |
| 43 | Nmral1 | 3373 | -1.148 | 0.0208 | Yes |
| 44 | Orc4 | 3505 | -1.215 | 0.0204 | Yes |
| 45 | Smad4 | 3712 | -1.395 | 0.0062 | Yes |
| 46 | Gabpa | 3752 | -1.442 | 0.0346 | Yes |
| 47 | Cep95 | 3956 | -1.843 | 0.0329 | Yes |
Table: GSEA details [plain text format]

  

Fig 2: DESCARTES\_ORGANOGENESIS\_STROMAL\_CELLS: Random ES distribution      
 Gene set null distribution of ES for **DESCARTES\_ORGANOGENESIS\_STROMAL\_CELLS**

  
