# Supplementary material for: TMPRSS11B promotes an acidified microenvironment and immune suppression in squamous lung cancer
Source: EMBO Rep. 2025 Nov 10;26(24):6346–79. doi: 10.1038/s44319-025-00631-1 (PMC12714794; doi:10.1038/s44319-025-00631-1)
Supplement: Supplementary file 18 — Figure EV6 Source Data [file 44319_2025_631_MOESM18_ESM.zip › Figure EV6/EV6C-D/GSEA_Broad Institute_M8_T11b high vs low LUSC/DESCARTES_ORGANOGENESIS_WHITE_BLOOD_CELLS.html]

Details for gene set DESCARTES\_ORGANOGENESIS\_WHITE\_BLOOD\_CELLS[GSEA]

|  || Dataset | T11b high vs low squamous\_GSEA\_Ranked |
| Phenotype | NoPhenotypeAvailable |
| Upregulated in class | na\_pos |
| GeneSet | DESCARTES\_ORGANOGENESIS\_WHITE\_BLOOD\_CELLS |
| Enrichment Score (ES) | 0.6144981 |
| Normalized Enrichment Score (NES) | 4.450143 |
| Nominal p-value | 0.0 |
| FDR q-value | 0.0 |
| FWER p-Value | 0.0 |
Table: GSEA Results Summary

  

Fig 1: Enrichment plot: DESCARTES\_ORGANOGENESIS\_WHITE\_BLOOD\_CELLS      
 Profile of the Running ES Score & Positions of GeneSet Members on the Rank Ordered List

  

| SYMBOL | RANK IN GENE LIST | RANK METRIC SCORE | RUNNING ES | CORE ENRICHMENT || 1 | Cd300lf | 21 | 3.644 | 0.0092 | Yes |
| 2 | Trem2 | 24 | 3.569 | 0.0230 | Yes |
| 3 | Clec4n | 37 | 3.016 | 0.0321 | Yes |
| 4 | Cd300c2 | 42 | 2.822 | 0.0424 | Yes |
| 5 | Il1a | 44 | 2.812 | 0.0534 | Yes |
| 6 | Cd84 | 45 | 2.807 | 0.0646 | Yes |
| 7 | C3ar1 | 46 | 2.795 | 0.0759 | Yes |
| 8 | Evi2a | 52 | 2.706 | 0.0854 | Yes |
| 9 | Hmox1 | 55 | 2.684 | 0.0957 | Yes |
| 10 | Cybb | 57 | 2.654 | 0.1061 | Yes |
| 11 | Nfam1 | 58 | 2.646 | 0.1167 | Yes |
| 12 | Itgam | 61 | 2.631 | 0.1267 | Yes |
| 13 | Fcgr2b | 62 | 2.610 | 0.1372 | Yes |
| 14 | Ctss | 63 | 2.582 | 0.1476 | Yes |
| 15 | Slc15a3 | 67 | 2.536 | 0.1570 | Yes |
| 16 | Ncf1 | 72 | 2.475 | 0.1659 | Yes |
| 17 | Cd37 | 74 | 2.458 | 0.1755 | Yes |
| 18 | Fcer1g | 76 | 2.415 | 0.1849 | Yes |
| 19 | Msr1 | 78 | 2.391 | 0.1942 | Yes |
| 20 | Slc11a1 | 80 | 2.375 | 0.2035 | Yes |
| 21 | Tyrobp | 83 | 2.366 | 0.2125 | Yes |
| 22 | Cd36 | 85 | 2.334 | 0.2216 | Yes |
| 23 | Clec2g | 86 | 2.330 | 0.2309 | Yes |
| 24 | Apoe | 88 | 2.296 | 0.2399 | Yes |
| 25 | Ccl9 | 93 | 2.271 | 0.2480 | Yes |
| 26 | Cd68 | 97 | 2.215 | 0.2561 | Yes |
| 27 | Fcgr3 | 102 | 2.142 | 0.2637 | Yes |
| 28 | Hk3 | 110 | 2.075 | 0.2702 | Yes |
| 29 | Csf3r | 113 | 2.052 | 0.2779 | Yes |
| 30 | Mpeg1 | 114 | 2.045 | 0.2861 | Yes |
| 31 | Emp3 | 115 | 2.020 | 0.2942 | Yes |
| 32 | C1qa | 119 | 1.990 | 0.3014 | Yes |
| 33 | Cd300a | 122 | 1.959 | 0.3088 | Yes |
| 34 | C5ar1 | 125 | 1.945 | 0.3161 | Yes |
| 35 | Ctsz | 138 | 1.884 | 0.3206 | Yes |
| 36 | C1qb | 139 | 1.882 | 0.3281 | Yes |
| 37 | Lgmn | 141 | 1.877 | 0.3354 | Yes |
| 38 | Ctsd | 150 | 1.830 | 0.3407 | Yes |
| 39 | Sirpa | 154 | 1.794 | 0.3471 | Yes |
| 40 | Fxyd5 | 157 | 1.767 | 0.3537 | Yes |
| 41 | Spi1 | 158 | 1.765 | 0.3608 | Yes |
| 42 | Ccl6 | 166 | 1.733 | 0.3659 | Yes |
| 43 | Cd33 | 168 | 1.727 | 0.3726 | Yes |
| 44 | Cfp | 171 | 1.717 | 0.3790 | Yes |
| 45 | Hexb | 175 | 1.703 | 0.3850 | Yes |
| 46 | Ctsb | 177 | 1.695 | 0.3916 | Yes |
| 47 | Mctp1 | 180 | 1.688 | 0.3978 | Yes |
| 48 | Crlf2 | 182 | 1.670 | 0.4043 | Yes |
| 49 | Pik3ap1 | 184 | 1.660 | 0.4107 | Yes |
| 50 | Slc37a2 | 189 | 1.627 | 0.4162 | Yes |
| 51 | Ptafr | 191 | 1.626 | 0.4224 | Yes |
| 52 | Gusb | 199 | 1.597 | 0.4271 | Yes |
| 53 | Myo1f | 212 | 1.559 | 0.4302 | Yes |
| 54 | Cyth4 | 220 | 1.521 | 0.4345 | Yes |
| 55 | C1qc | 234 | 1.480 | 0.4371 | Yes |
| 56 | Psap | 240 | 1.466 | 0.4417 | Yes |
| 57 | Mertk | 250 | 1.448 | 0.4452 | Yes |
| 58 | Jdp2 | 255 | 1.437 | 0.4500 | Yes |
| 59 | Ptprc | 258 | 1.430 | 0.4552 | Yes |
| 60 | Arhgap9 | 260 | 1.428 | 0.4607 | Yes |
| 61 | Apbb1ip | 264 | 1.423 | 0.4656 | Yes |
| 62 | Npl | 268 | 1.398 | 0.4705 | Yes |
| 63 | Dock2 | 271 | 1.390 | 0.4755 | Yes |
| 64 | Grn | 285 | 1.352 | 0.4776 | Yes |
| 65 | Tnfrsf26 | 291 | 1.342 | 0.4817 | Yes |
| 66 | Pik3cd | 304 | 1.288 | 0.4838 | Yes |
| 67 | Hck | 312 | 1.246 | 0.4870 | Yes |
| 68 | Myo5a | 318 | 1.220 | 0.4906 | Yes |
| 69 | Pstpip1 | 321 | 1.210 | 0.4950 | Yes |
| 70 | Gna15 | 326 | 1.197 | 0.4987 | Yes |
| 71 | Selplg | 328 | 1.195 | 0.5033 | Yes |
| 72 | Fnip2 | 334 | 1.179 | 0.5067 | Yes |
| 73 | Cd53 | 337 | 1.171 | 0.5109 | Yes |
| 74 | Plxdc1 | 341 | 1.162 | 0.5148 | Yes |
| 75 | Fmnl1 | 345 | 1.153 | 0.5187 | Yes |
| 76 | Cd52 | 350 | 1.140 | 0.5222 | Yes |
| 77 | Il17ra | 351 | 1.140 | 0.5268 | Yes |
| 78 | Havcr2 | 364 | 1.121 | 0.5282 | Yes |
| 79 | Irf1 | 372 | 1.111 | 0.5309 | Yes |
| 80 | Lgals3 | 377 | 1.096 | 0.5342 | Yes |
| 81 | Coro1a | 390 | 1.079 | 0.5355 | Yes |
| 82 | Ptgs1 | 392 | 1.072 | 0.5395 | Yes |
| 83 | Alox5ap | 399 | 1.051 | 0.5422 | Yes |
| 84 | Gpr65 | 401 | 1.047 | 0.5462 | Yes |
| 85 | Vsir | 405 | 1.036 | 0.5495 | Yes |
| 86 | Csf1r | 414 | 1.025 | 0.5516 | Yes |
| 87 | Zc3h12a | 419 | 1.020 | 0.5547 | Yes |
| 88 | Apobec1 | 424 | 1.015 | 0.5577 | Yes |
| 89 | Ctsa | 425 | 1.013 | 0.5618 | Yes |
| 90 | Lpxn | 431 | 1.004 | 0.5645 | Yes |
| 91 | Nckap1l | 432 | 1.004 | 0.5686 | Yes |
| 92 | Tnfrsf1b | 463 | 0.955 | 0.5647 | Yes |
| 93 | Il10rb | 467 | 0.949 | 0.5677 | Yes |
| 94 | Ehd4 | 474 | 0.943 | 0.5700 | Yes |
| 95 | Inpp5d | 487 | 0.918 | 0.5706 | Yes |
| 96 | Rab32 | 496 | 0.903 | 0.5722 | Yes |
| 97 | Gbp7 | 501 | 0.898 | 0.5747 | Yes |
| 98 | Hcls1 | 503 | 0.897 | 0.5781 | Yes |
| 99 | Cebpb | 504 | 0.897 | 0.5817 | Yes |
| 100 | Rasa4 | 514 | 0.886 | 0.5829 | Yes |
| 101 | Cyba | 519 | 0.875 | 0.5854 | Yes |
| 102 | Stab1 | 523 | 0.873 | 0.5881 | Yes |
| 103 | St6galnac4 | 525 | 0.873 | 0.5914 | Yes |
| 104 | Il3ra | 530 | 0.869 | 0.5938 | Yes |
| 105 | Tmem104 | 537 | 0.860 | 0.5957 | Yes |
| 106 | Abr | 538 | 0.860 | 0.5992 | Yes |
| 107 | Runx3 | 539 | 0.860 | 0.6026 | Yes |
| 108 | Tcirg1 | 541 | 0.853 | 0.6058 | Yes |
| 109 | Ptk2b | 559 | 0.835 | 0.6048 | Yes |
| 110 | Cd44 | 562 | 0.834 | 0.6076 | Yes |
| 111 | Rab20 | 565 | 0.831 | 0.6105 | Yes |
| 112 | Ifnar2 | 567 | 0.830 | 0.6135 | Yes |
| 113 | Snx8 | 577 | 0.816 | 0.6145 | Yes |
| 114 | Slc29a3 | 600 | 0.773 | 0.6120 | No |
| 115 | Blnk | 616 | 0.747 | 0.6111 | No |
| 116 | Gmip | 628 | 0.732 | 0.6112 | No |
| 117 | Mlkl | 688 | 0.680 | 0.5988 | No |
| 118 | Dtx3l | 702 | 0.665 | 0.5982 | No |
| 119 | Tmem106a | 706 | 0.663 | 0.6001 | No |
| 120 | H2-D1 | 719 | 0.654 | 0.5996 | No |
| 121 | Slfn2 | 750 | 0.628 | 0.5944 | No |
| 122 | Ms4a7 | 771 | 0.610 | 0.5918 | No |
| 123 | Def6 | 777 | 0.604 | 0.5929 | No |
| 124 | Arrb2 | 786 | 0.600 | 0.5933 | No |
| 125 | Vps18 | 805 | 0.591 | 0.5910 | No |
| 126 | Fam111a | 809 | 0.590 | 0.5926 | No |
| 127 | Gm2a | 815 | 0.585 | 0.5937 | No |
| 128 | Sat1 | 817 | 0.584 | 0.5958 | No |
| 129 | Maf | 826 | 0.576 | 0.5960 | No |
| 130 | Lcp2 | 840 | 0.570 | 0.5950 | No |
| 131 | Cd74 | 849 | 0.567 | 0.5952 | No |
| 132 | H2-K1 | 855 | 0.565 | 0.5962 | No |
| 133 | Litaf | 856 | 0.564 | 0.5985 | No |
| 134 | B2m | 860 | 0.563 | 0.5999 | No |
| 135 | Casp8 | 920 | 0.520 | 0.5869 | No |
| 136 | Blvra | 938 | 0.512 | 0.5846 | No |
| 137 | Dab2 | 952 | 0.504 | 0.5833 | No |
| 138 | Hfe | 1209 | -0.542 | 0.5198 | No |
| 139 | Cebpzos | 1225 | -0.544 | 0.5182 | No |
| 140 | Cmtm7 | 1228 | -0.545 | 0.5198 | No |
| 141 | Apobec3 | 1238 | -0.546 | 0.5197 | No |
| 142 | Madd | 1256 | -0.548 | 0.5176 | No |
| 143 | Fgd2 | 1563 | -0.605 | 0.4416 | No |
| 144 | Ppm1h | 1601 | -0.611 | 0.4345 | No |
| 145 | Tnfrsf11a | 1644 | -0.619 | 0.4262 | No |
| 146 | Trim65 | 1661 | -0.623 | 0.4246 | No |
| 147 | Tmem141 | 1709 | -0.631 | 0.4151 | No |
| 148 | Aga | 1798 | -0.648 | 0.3952 | No |
| 149 | Ly6e | 1857 | -0.663 | 0.3830 | No |
| 150 | H2-M3 | 1927 | -0.681 | 0.3680 | No |
| 151 | Il15 | 1933 | -0.681 | 0.3694 | No |
| 152 | Morc3 | 1986 | -0.691 | 0.3589 | No |
| 153 | Selenop | 2054 | -0.707 | 0.3446 | No |
| 154 | Lrmda | 2077 | -0.712 | 0.3418 | No |
| 155 | Rnf213 | 2130 | -0.724 | 0.3313 | No |
| 156 | Manba | 2205 | -0.739 | 0.3153 | No |
| 157 | Ocel1 | 2221 | -0.744 | 0.3145 | No |
| 158 | Tbc1d5 | 2319 | -0.767 | 0.2927 | No |
| 159 | Snx2 | 2507 | -0.820 | 0.2480 | No |
| 160 | Slc46a3 | 2514 | -0.823 | 0.2498 | No |
| 161 | Epsti1 | 2646 | -0.860 | 0.2197 | No |
| 162 | Il18r1 | 2742 | -0.887 | 0.1989 | No |
| 163 | Tlr4 | 2764 | -0.895 | 0.1971 | No |
| 164 | Fchsd2 | 2768 | -0.896 | 0.1999 | No |
| 165 | Gsdmd | 2790 | -0.904 | 0.1982 | No |
| 166 | Plcg2 | 2818 | -0.911 | 0.1949 | No |
| 167 | Snx6 | 2844 | -0.920 | 0.1922 | No |
| 168 | Shisa5 | 2916 | -0.946 | 0.1778 | No |
| 169 | Tap1 | 3068 | -1.004 | 0.1431 | No |
| 170 | Il18 | 3078 | -1.010 | 0.1448 | No |
| 171 | Casp4 | 3233 | -1.084 | 0.1097 | No |
| 172 | Glb1 | 3236 | -1.084 | 0.1135 | No |
| 173 | Wwp1 | 3275 | -1.103 | 0.1082 | No |
| 174 | Fcgrt | 3345 | -1.138 | 0.0951 | No |
| 175 | Sp100 | 3362 | -1.143 | 0.0956 | No |
| 176 | Tlr2 | 3387 | -1.156 | 0.0941 | No |
| 177 | Tcn2 | 3391 | -1.158 | 0.0979 | No |
| 178 | Rel | 3500 | -1.210 | 0.0751 | No |
| 179 | Map3k5 | 3578 | -1.271 | 0.0605 | No |
| 180 | Myb | 3611 | -1.297 | 0.0575 | No |
| 181 | Cela1 | 3666 | -1.349 | 0.0490 | No |
| 182 | Ptpn22 | 3676 | -1.360 | 0.0522 | No |
| 183 | Pparg | 3727 | -1.412 | 0.0450 | No |
| 184 | Reps2 | 3765 | -1.454 | 0.0414 | No |
| 185 | Cracr2b | 3867 | -1.641 | 0.0221 | No |
| 186 | Nfatc2 | 3913 | -1.752 | 0.0176 | No |
| 187 | Arid5a | 3940 | -1.802 | 0.0181 | No |
| 188 | Oas2 | 3963 | -1.896 | 0.0201 | No |
| 189 | Adgb | 4080 | -2.975 | 0.0023 | No |
Table: GSEA details [plain text format]

  

Fig 2: DESCARTES\_ORGANOGENESIS\_WHITE\_BLOOD\_CELLS: Random ES distribution      
 Gene set null distribution of ES for **DESCARTES\_ORGANOGENESIS\_WHITE\_BLOOD\_CELLS**

  
