# Supplementary material for: TMPRSS11B promotes an acidified microenvironment and immune suppression in squamous lung cancer
Source: EMBO Rep. 2025 Nov 10;26(24):6346–79. doi: 10.1038/s44319-025-00631-1 (PMC12714794; doi:10.1038/s44319-025-00631-1)
Supplement: Supplementary file 18 — Figure EV6 Source Data [file 44319_2025_631_MOESM18_ESM.zip › Figure EV6/EV6C-D/GSEA_Broad Institute_M8_T11b high vs low LUSC/gsea_report_for_na_neg_1723673728644.html]

Report for na\_neg 1723673728644 [GSEA]

| GS  follow link to MSigDB | GS DETAILS | SIZE | ES | NES | NOM p-val | FDR q-val | FWER p-val | RANK AT MAX | LEADING EDGE || 1 | DESCARTES\_ORGANOGENESIS\_EPENDYMAL\_CELL | Details ... | 43 | -0.71 | -3.53 | 0.000 | 0.000 | 0.000 | 256 | tags=58%, list=6%, signal=61% |
| 2 | DESCARTES\_ORGANOGENESIS\_SENSORY\_NEURONS | Details ... | 17 | -0.44 | -1.62 | 0.034 | 0.213 | 0.852 | 1048 | tags=59%, list=26%, signal=79% |
| 3 | ZHANG\_UTERUS\_C13\_EPITHELIAL1\_CELL | Details ... | 88 | -0.26 | -1.49 | 0.040 | 0.335 | 0.990 | 699 | tags=28%, list=17%, signal=34% |
| 4 | DESCARTES\_ORGANOGENESIS\_HEPATOCYTES | Details ... | 129 | -0.23 | -1.48 | 0.023 | 0.266 | 0.994 | 1160 | tags=40%, list=28%, signal=53% |
| 5 | ZHANG\_UTERUS\_C2\_REGENERATIVE\_UP | Details ... | 25 | -0.33 | -1.38 | 0.115 | 0.371 | 1.000 | 686 | tags=36%, list=17%, signal=43% |
| 6 | TABULA\_MURIS\_SENIS\_HEART\_VENTRICULAR\_MYOCYTE\_AGEING | Details ... | 34 | -0.30 | -1.38 | 0.096 | 0.320 | 1.000 | 862 | tags=32%, list=21%, signal=41% |
| 7 | DESCARTES\_ORGANOGENESIS\_MYOCYTES | Details ... | 17 | -0.33 | -1.21 | 0.254 | 0.647 | 1.000 | 1151 | tags=59%, list=28%, signal=82% |
| 8 | TABULA\_MURIS\_SENIS\_LIVER\_HEPATOCYTE\_AGEING | Details ... | 43 | -0.23 | -1.13 | 0.301 | 0.810 | 1.000 | 1037 | tags=40%, list=25%, signal=52% |
| 9 | TABULA\_MURIS\_SENIS\_PANCREAS\_PANCREATIC\_POLYPEPTIDE\_CELL\_AGEING | Details ... | 37 | -0.22 | -1.04 | 0.409 | 1.000 | 1.000 | 1602 | tags=49%, list=39%, signal=79% |
| 10 | DESCARTES\_ORGANOGENESIS\_EPITHELIAL\_CELLS | Details ... | 71 | -0.17 | -0.96 | 0.527 | 1.000 | 1.000 | 369 | tags=15%, list=9%, signal=17% |
| 11 | TABULA\_MURIS\_SENIS\_PANCREAS\_PANCREATIC\_DUCTAL\_CELL\_AGEING | Details ... | 87 | -0.15 | -0.90 | 0.648 | 1.000 | 1.000 | 1210 | tags=34%, list=30%, signal=48% |
| 12 | DESCARTES\_ORGANOGENESIS\_STROMAL\_CELLS | Details ... | 47 | -0.17 | -0.85 | 0.687 | 1.000 | 1.000 | 3110 | tags=94%, list=76%, signal=386% |
| 13 | TABULA\_MURIS\_SENIS\_BRAIN\_NON\_MYELOID\_NEURON\_AGEING | Details ... | 239 | -0.11 | -0.78 | 0.872 | 1.000 | 1.000 | 1094 | tags=26%, list=27%, signal=33% |
| 14 | TABULA\_MURIS\_SENIS\_LARGE\_INTESTINE\_SECRETORY\_CELL\_AGEING | Details ... | 201 | -0.11 | -0.78 | 0.871 | 1.000 | 1.000 | 2502 | tags=66%, list=61%, signal=162% |
| 15 | TABULA\_MURIS\_SENIS\_PANCREAS\_PANCREATIC\_BETA\_CELL\_AGEING | Details ... | 50 | -0.15 | -0.77 | 0.806 | 1.000 | 1.000 | 733 | tags=20%, list=18%, signal=24% |
| 16 | DESCARTES\_ORGANOGENESIS\_SCHWANN\_CELL\_PRECURSOR | Details ... | 20 | -0.20 | -0.76 | 0.758 | 1.000 | 1.000 | 1016 | tags=30%, list=25%, signal=40% |
| 17 | TABULA\_MURIS\_SENIS\_LARGE\_INTESTINE\_INTESTINAL\_CRYPT\_STEM\_CELL\_AGEING | Details ... | 102 | -0.11 | -0.64 | 0.945 | 1.000 | 1.000 | 1616 | tags=40%, list=40%, signal=65% |
| 18 | DESCARTES\_ORGANOGENESIS\_PRIMITIVE\_ERYTHROID\_LINEAGE | Details ... | 121 | -0.09 | -0.59 | 0.975 | 1.000 | 1.000 | 399 | tags=10%, list=10%, signal=11% |
| 19 | TABULA\_MURIS\_SENIS\_KIDNEY\_EPITHELIAL\_CELL\_OF\_PROXIMAL\_TUBULE\_AGEING | Details ... | 84 | -0.09 | -0.54 | 0.979 | 1.000 | 1.000 | 1624 | tags=40%, list=40%, signal=66% |
| 20 | TABULA\_MURIS\_SENIS\_PANCREAS\_PANCREATIC\_DELTA\_CELL\_AGEING | Details ... | 62 | -0.10 | -0.52 | 0.982 | 1.000 | 1.000 | 1248 | tags=29%, list=31%, signal=41% |
| 21 | TABULA\_MURIS\_SENIS\_LARGE\_INTESTINE\_LARGE\_INTESTINE\_GOBLET\_CELL\_AGEING |  | 217 | -0.06 | -0.45 | 1.000 | 1.000 | 1.000 | 2608 | tags=65%, list=64%, signal=169% |
| 22 | TABULA\_MURIS\_SENIS\_LARGE\_INTESTINE\_ENTEROCYTE\_OF\_EPITHELIUM\_OF\_LARGE\_INTESTINE\_AGEING |  | 276 | -0.05 | -0.35 | 1.000 | 1.000 | 1.000 | 3400 | tags=91%, list=83%, signal=505% |
Table: Gene sets enriched in phenotype **na**[plain text format]****

  
