# Supplementary material for: TMPRSS11B promotes an acidified microenvironment and immune suppression in squamous lung cancer
Source: EMBO Rep. 2025 Nov 10;26(24):6346–79. doi: 10.1038/s44319-025-00631-1 (PMC12714794; doi:10.1038/s44319-025-00631-1)
Supplement: Supplementary file 18 — Figure EV6 Source Data [file 44319_2025_631_MOESM18_ESM.zip › Figure EV6/EV6C-D/GSEA_Broad Institute_M8_T11b high vs low LUSC/gsea_report_for_na_pos_1723673728644.html]

Report for na\_pos 1723673728644 [GSEA]

| GS  follow link to MSigDB | GS DETAILS | SIZE | ES | NES | NOM p-val | FDR q-val | FWER p-val | RANK AT MAX | LEADING EDGE || 1 | ZHANG\_UTERUS\_C12\_MONOCYTE | Details ... | 83 | 0.77 | 4.74 | 0.000 | 0.000 | 0.000 | 861 | tags=92%, list=21%, signal=114% |
| 2 | ZHANG\_UTERUS\_C5\_MACROPHAGE | Details ... | 80 | 0.73 | 4.50 | 0.000 | 0.000 | 0.000 | 536 | tags=70%, list=13%, signal=79% |
| 3 | DESCARTES\_ORGANOGENESIS\_WHITE\_BLOOD\_CELLS | Details ... | 189 | 0.61 | 4.45 | 0.000 | 0.000 | 0.000 | 577 | tags=60%, list=14%, signal=66% |
| 4 | ZHANG\_UTERUS\_C9\_DENDRITIC\_CELL | Details ... | 43 | 0.75 | 3.87 | 0.000 | 0.000 | 0.000 | 979 | tags=98%, list=24%, signal=127% |
| 5 | TABULA\_MURIS\_SENIS\_SPLEEN\_MACROPHAGE\_AGEING | Details ... | 54 | 0.68 | 3.85 | 0.000 | 0.000 | 0.000 | 632 | tags=67%, list=15%, signal=78% |
| 6 | TABULA\_MURIS\_SENIS\_LUNG\_ALVEOLAR\_MACROPHAGE\_AGEING | Details ... | 67 | 0.65 | 3.77 | 0.000 | 0.000 | 0.000 | 860 | tags=72%, list=21%, signal=89% |
| 7 | TABULA\_MURIS\_SENIS\_HEART\_AND\_AORTA\_LEUKOCYTE\_AGEING | Details ... | 33 | 0.75 | 3.56 | 0.000 | 0.000 | 0.000 | 500 | tags=76%, list=12%, signal=86% |
| 8 | ZHANG\_UTERUS\_C10\_STROMAL2\_RETNLG\_HIGH\_CELL | Details ... | 24 | 0.82 | 3.51 | 0.000 | 0.000 | 0.000 | 527 | tags=88%, list=13%, signal=100% |
| 9 | TABULA\_MURIS\_SENIS\_KIDNEY\_MACROPHAGE\_AGEING | Details ... | 44 | 0.67 | 3.48 | 0.000 | 0.000 | 0.000 | 771 | tags=77%, list=19%, signal=94% |
| 10 | TABULA\_MURIS\_SENIS\_BRAIN\_MYELOID\_MICROGLIAL\_CELL\_AGEING | Details ... | 95 | 0.53 | 3.40 | 0.000 | 0.000 | 0.000 | 919 | tags=63%, list=22%, signal=80% |
| 11 | TABULA\_MURIS\_SENIS\_MARROW\_HEMATOPOIETIC\_PRECURSOR\_CELL\_AGEING | Details ... | 36 | 0.67 | 3.38 | 0.000 | 0.000 | 0.000 | 860 | tags=81%, list=21%, signal=101% |
| 12 | TABULA\_MURIS\_SENIS\_MARROW\_GRANULOCYTOPOIETIC\_CELL\_AGEING | Details ... | 43 | 0.65 | 3.38 | 0.000 | 0.000 | 0.000 | 828 | tags=79%, list=20%, signal=98% |
| 13 | TABULA\_MURIS\_SENIS\_AORTA\_PROFESSIONAL\_ANTIGEN\_PRESENTING\_CELL\_AGEING | Details ... | 46 | 0.63 | 3.33 | 0.000 | 0.000 | 0.000 | 915 | tags=80%, list=22%, signal=102% |
| 14 | TABULA\_MURIS\_SENIS\_MARROW\_GRANULOCYTE\_AGEING | Details ... | 29 | 0.74 | 3.32 | 0.000 | 0.000 | 0.000 | 895 | tags=93%, list=22%, signal=118% |
| 15 | TABULA\_MURIS\_SENIS\_GONADAL\_ADIPOSE\_TISSUE\_MYELOID\_CELL\_AGEING | Details ... | 91 | 0.51 | 3.26 | 0.000 | 0.000 | 0.000 | 971 | tags=68%, list=24%, signal=87% |
| 16 | TABULA\_MURIS\_SENIS\_MAMMARY\_GLAND\_T\_CELL\_AGEING | Details ... | 139 | 0.47 | 3.19 | 0.000 | 0.000 | 0.000 | 895 | tags=56%, list=22%, signal=69% |
| 17 | TABULA\_MURIS\_SENIS\_LUNG\_INTERMEDIATE\_MONOCYTE\_AGEING | Details ... | 86 | 0.51 | 3.15 | 0.000 | 0.000 | 0.000 | 586 | tags=43%, list=14%, signal=49% |
| 18 | TABULA\_MURIS\_SENIS\_SPLEEN\_B\_CELL\_AGEING | Details ... | 34 | 0.61 | 3.03 | 0.000 | 0.000 | 0.000 | 928 | tags=76%, list=23%, signal=98% |
| 19 | TABULA\_MURIS\_SENIS\_SUBCUTANEOUS\_ADIPOSE\_TISSUE\_MYELOID\_CELL\_AGEING | Details ... | 68 | 0.51 | 3.01 | 0.000 | 0.000 | 0.000 | 971 | tags=71%, list=24%, signal=91% |
| 20 | TABULA\_MURIS\_SENIS\_TONGUE\_BASAL\_CELL\_OF\_EPIDERMIS\_AGEING | Details ... | 42 | 0.58 | 2.99 | 0.000 | 0.000 | 0.000 | 954 | tags=74%, list=23%, signal=95% |
| 21 | TABULA\_MURIS\_SENIS\_MARROW\_PROMONOCYTE\_AGEING |  | 32 | 0.63 | 2.99 | 0.000 | 0.000 | 0.000 | 566 | tags=56%, list=14%, signal=65% |
| 22 | TABULA\_MURIS\_SENIS\_HEART\_MONOCYTE\_AGEING |  | 44 | 0.56 | 2.94 | 0.000 | 0.000 | 0.000 | 519 | tags=48%, list=13%, signal=54% |
| 23 | ZHANG\_UTERUS\_C8\_NK\_CELL |  | 41 | 0.56 | 2.94 | 0.000 | 0.000 | 0.000 | 896 | tags=71%, list=22%, signal=90% |
| 24 | TABULA\_MURIS\_SENIS\_SPLEEN\_CD8\_POSITIVE\_ALPHA\_BETA\_T\_CELL\_AGEING |  | 43 | 0.56 | 2.93 | 0.000 | 0.000 | 0.000 | 971 | tags=77%, list=24%, signal=100% |
| 25 | TABULA\_MURIS\_SENIS\_SPLEEN\_T\_CELL\_AGEING |  | 70 | 0.49 | 2.88 | 0.000 | 0.000 | 0.000 | 860 | tags=57%, list=21%, signal=71% |
| 26 | TABULA\_MURIS\_SENIS\_LUNG\_NON\_CLASSICAL\_MONOCYTE\_AGEING |  | 18 | 0.75 | 2.84 | 0.000 | 0.000 | 0.000 | 586 | tags=78%, list=14%, signal=90% |
| 27 | TABULA\_MURIS\_SENIS\_LIMB\_MUSCLE\_MESENCHYMAL\_STEM\_CELL\_AGEING |  | 50 | 0.52 | 2.84 | 0.000 | 0.000 | 0.000 | 860 | tags=70%, list=21%, signal=88% |
| 28 | TABULA\_MURIS\_SENIS\_MARROW\_NAIVE\_T\_CELL\_AGEING |  | 20 | 0.72 | 2.83 | 0.000 | 0.000 | 0.000 | 719 | tags=80%, list=18%, signal=97% |
| 29 | TABULA\_MURIS\_SENIS\_MAMMARY\_GLAND\_B\_CELL\_AGEING |  | 87 | 0.45 | 2.83 | 0.000 | 0.000 | 0.000 | 869 | tags=55%, list=21%, signal=69% |
| 30 | TABULA\_MURIS\_SENIS\_SUBCUTANEOUS\_ADIPOSE\_TISSUE\_B\_CELL\_AGEING |  | 49 | 0.51 | 2.80 | 0.000 | 0.000 | 0.000 | 950 | tags=67%, list=23%, signal=87% |
| 31 | TABULA\_MURIS\_SENIS\_LUNG\_CD4\_POSITIVE\_ALPHA\_BETA\_T\_CELL\_AGEING |  | 44 | 0.54 | 2.80 | 0.000 | 0.000 | 0.000 | 860 | tags=66%, list=21%, signal=83% |
| 32 | ZHANG\_UTERUS\_C4\_MYOFIBROBLAST |  | 111 | 0.42 | 2.78 | 0.000 | 0.000 | 0.000 | 800 | tags=52%, list=20%, signal=63% |
| 33 | TABULA\_MURIS\_SENIS\_MAMMARY\_GLAND\_MACROPHAGE\_AGEING |  | 26 | 0.63 | 2.78 | 0.000 | 0.000 | 0.000 | 760 | tags=73%, list=19%, signal=89% |
| 34 | TABULA\_MURIS\_SENIS\_SPLEEN\_CD4\_POSITIVE\_ALPHA\_BETA\_T\_CELL\_AGEING |  | 51 | 0.50 | 2.77 | 0.000 | 0.000 | 0.000 | 895 | tags=67%, list=22%, signal=84% |
| 35 | TABULA\_MURIS\_SENIS\_MARROW\_MACROPHAGE\_AGEING |  | 50 | 0.51 | 2.73 | 0.000 | 0.000 | 0.000 | 895 | tags=62%, list=22%, signal=78% |
| 36 | ZHANG\_UTERUS\_C1\_PROLIFERATIVE\_STROMAL1\_MGP\_HIGH\_CELL |  | 93 | 0.43 | 2.72 | 0.000 | 0.000 | 0.000 | 792 | tags=55%, list=19%, signal=66% |
| 37 | TABULA\_MURIS\_SENIS\_LUNG\_B\_CELL\_AGEING |  | 31 | 0.57 | 2.67 | 0.000 | 0.000 | 0.000 | 928 | tags=71%, list=23%, signal=91% |
| 38 | TABULA\_MURIS\_SENIS\_LUNG\_CLASSICAL\_MONOCYTE\_AGEING |  | 72 | 0.44 | 2.65 | 0.000 | 0.000 | 0.000 | 586 | tags=33%, list=14%, signal=38% |
| 39 | TABULA\_MURIS\_SENIS\_BLADDER\_BLADDER\_UROTHELIAL\_CELL\_AGEING |  | 54 | 0.48 | 2.65 | 0.000 | 0.000 | 0.000 | 895 | tags=65%, list=22%, signal=82% |
| 40 | TABULA\_MURIS\_SENIS\_HEART\_ENDOTHELIAL\_CELL\_OF\_CORONARY\_ARTERY\_AGEING |  | 41 | 0.51 | 2.65 | 0.000 | 0.000 | 0.000 | 915 | tags=71%, list=22%, signal=90% |
| 41 | TABULA\_MURIS\_SENIS\_MARROW\_PRECURSOR\_B\_CELL\_AGEING |  | 31 | 0.55 | 2.61 | 0.000 | 0.000 | 0.000 | 691 | tags=55%, list=17%, signal=65% |
| 42 | TABULA\_MURIS\_SENIS\_LIMB\_MUSCLE\_MACROPHAGE\_AGEING |  | 34 | 0.53 | 2.59 | 0.000 | 0.000 | 0.000 | 949 | tags=71%, list=23%, signal=91% |
| 43 | TABULA\_MURIS\_SENIS\_TRACHEA\_FIBROBLAST\_AGEING |  | 33 | 0.54 | 2.57 | 0.000 | 0.000 | 0.000 | 719 | tags=58%, list=18%, signal=69% |
| 44 | TABULA\_MURIS\_SENIS\_MESENTERIC\_ADIPOSE\_TISSUE\_MACROPHAGE\_AGEING |  | 25 | 0.59 | 2.54 | 0.000 | 0.000 | 0.000 | 938 | tags=76%, list=23%, signal=98% |
| 45 | TABULA\_MURIS\_SENIS\_BLADDER\_BLADDER\_CELL\_AGEING |  | 83 | 0.41 | 2.54 | 0.000 | 0.000 | 0.000 | 895 | tags=59%, list=22%, signal=74% |
| 46 | ZHANG\_UTERUS\_C0\_SECRETORY\_STROMAL3\_NPPC\_HIGH\_CELL |  | 106 | 0.39 | 2.54 | 0.000 | 0.000 | 0.000 | 792 | tags=52%, list=19%, signal=63% |
| 47 | TABULA\_MURIS\_SENIS\_SPLEEN\_MATURE\_NK\_T\_CELL\_AGEING |  | 19 | 0.61 | 2.53 | 0.000 | 0.000 | 0.000 | 115 | tags=37%, list=3%, signal=38% |
| 48 | TABULA\_MURIS\_SENIS\_SUBCUTANEOUS\_ADIPOSE\_TISSUE\_MESENCHYMAL\_STEM\_CELL\_OF\_ADIPOSE\_AGEING |  | 84 | 0.41 | 2.51 | 0.000 | 0.000 | 0.000 | 661 | tags=43%, list=16%, signal=50% |
| 49 | TABULA\_MURIS\_SENIS\_LIVER\_ENDOTHELIAL\_CELL\_OF\_HEPATIC\_SINUSOID\_AGEING |  | 108 | 0.38 | 2.51 | 0.000 | 0.000 | 0.000 | 924 | tags=52%, list=23%, signal=65% |
| 50 | ZHANG\_UTERUS\_C2\_SECRETORY\_STROMAL3\_RAMP3\_HIGH\_CELL |  | 69 | 0.43 | 2.50 | 0.000 | 0.000 | 0.000 | 792 | tags=57%, list=19%, signal=69% |
| 51 | TABULA\_MURIS\_SENIS\_MESENTERIC\_ADIPOSE\_TISSUE\_CD4\_POSITIVE\_ALPHA\_BETA\_T\_CELL\_AGEING |  | 25 | 0.57 | 2.47 | 0.000 | 0.000 | 0.002 | 954 | tags=72%, list=23%, signal=93% |
| 52 | TABULA\_MURIS\_SENIS\_BRAIN\_MYELOID\_MACROPHAGE\_AGEING |  | 15 | 0.67 | 2.45 | 0.000 | 0.000 | 0.002 | 895 | tags=87%, list=22%, signal=111% |
| 53 | TABULA\_MURIS\_SENIS\_MESENTERIC\_ADIPOSE\_TISSUE\_B\_CELL\_AGEING |  | 26 | 0.56 | 2.44 | 0.000 | 0.000 | 0.003 | 910 | tags=77%, list=22%, signal=98% |
| 54 | ZHANG\_UTERUS\_C3\_PROLIFERATIVE\_STROMAL1\_STROCXCL14\_HIGH\_CELL |  | 21 | 0.59 | 2.44 | 0.000 | 0.000 | 0.003 | 924 | tags=76%, list=23%, signal=98% |
| 55 | TABULA\_MURIS\_SENIS\_LIMB\_MUSCLE\_T\_CELL\_AGEING |  | 76 | 0.40 | 2.42 | 0.000 | 0.000 | 0.003 | 915 | tags=57%, list=22%, signal=72% |
| 56 | ZHANG\_UTERUS\_C6\_ENDOTHELIAL\_PLVAP\_HIGH\_CELL |  | 39 | 0.48 | 2.41 | 0.000 | 0.000 | 0.003 | 875 | tags=69%, list=21%, signal=87% |
| 57 | TABULA\_MURIS\_SENIS\_MARROW\_ERYTHROBLAST\_AGEING |  | 28 | 0.53 | 2.40 | 0.000 | 0.000 | 0.003 | 839 | tags=64%, list=21%, signal=80% |
| 58 | TABULA\_MURIS\_SENIS\_BROWN\_ADIPOSE\_TISSUE\_B\_CELL\_AGEING |  | 101 | 0.37 | 2.40 | 0.000 | 0.000 | 0.003 | 895 | tags=50%, list=22%, signal=63% |
| 59 | TABULA\_MURIS\_SENIS\_MARROW\_NAIVE\_B\_CELL\_AGEING |  | 80 | 0.39 | 2.40 | 0.000 | 0.000 | 0.003 | 895 | tags=50%, list=22%, signal=63% |
| 60 | TABULA\_MURIS\_SENIS\_MARROW\_MONOCYTE\_AGEING |  | 19 | 0.61 | 2.39 | 0.002 | 0.000 | 0.004 | 506 | tags=53%, list=12%, signal=60% |
| 61 | TABULA\_MURIS\_SENIS\_LIMB\_MUSCLE\_B\_CELL\_AGEING |  | 58 | 0.41 | 2.39 | 0.000 | 0.000 | 0.004 | 652 | tags=40%, list=16%, signal=47% |
| 62 | TABULA\_MURIS\_SENIS\_TONGUE\_KERATINOCYTE\_AGEING |  | 16 | 0.63 | 2.38 | 0.000 | 0.000 | 0.004 | 164 | tags=44%, list=4%, signal=45% |
| 63 | DESCARTES\_ORGANOGENESIS\_MEGAKARYOCYTES |  | 35 | 0.47 | 2.32 | 0.000 | 0.000 | 0.008 | 731 | tags=49%, list=18%, signal=59% |
| 64 | TABULA\_MURIS\_SENIS\_BROWN\_ADIPOSE\_TISSUE\_T\_CELL\_AGEING |  | 34 | 0.48 | 2.32 | 0.000 | 0.000 | 0.008 | 519 | tags=41%, list=13%, signal=47% |
| 65 | TABULA\_MURIS\_SENIS\_HEART\_AND\_AORTA\_ENDOTHELIAL\_CELL\_OF\_CORONARY\_ARTERY\_AGEING |  | 75 | 0.38 | 2.29 | 0.000 | 0.000 | 0.009 | 915 | tags=56%, list=22%, signal=71% |
| 66 | TABULA\_MURIS\_SENIS\_LIMB\_MUSCLE\_ENDOTHELIAL\_CELL\_AGEING |  | 36 | 0.46 | 2.28 | 0.003 | 0.000 | 0.009 | 915 | tags=64%, list=22%, signal=82% |
| 67 | ZHANG\_UTERUS\_C1\_REGENERATIVE\_UP |  | 102 | 0.35 | 2.27 | 0.000 | 0.000 | 0.009 | 954 | tags=53%, list=23%, signal=67% |
| 68 | TABULA\_MURIS\_SENIS\_THYMUS\_DN4\_THYMOCYTE\_AGEING |  | 56 | 0.40 | 2.24 | 0.000 | 0.000 | 0.015 | 915 | tags=50%, list=22%, signal=64% |
| 69 | TABULA\_MURIS\_SENIS\_MARROW\_IMMATURE\_B\_CELL\_AGEING |  | 51 | 0.41 | 2.23 | 0.000 | 0.001 | 0.016 | 991 | tags=59%, list=24%, signal=77% |
| 70 | TABULA\_MURIS\_SENIS\_BROWN\_ADIPOSE\_TISSUE\_MYELOID\_CELL\_AGEING |  | 71 | 0.37 | 2.22 | 0.000 | 0.001 | 0.017 | 971 | tags=54%, list=24%, signal=69% |
| 71 | TABULA\_MURIS\_SENIS\_LIMB\_MUSCLE\_SKELETAL\_MUSCLE\_SATELLITE\_CELL\_AGEING |  | 34 | 0.45 | 2.22 | 0.000 | 0.001 | 0.017 | 522 | tags=41%, list=13%, signal=47% |
| 72 | TABULA\_MURIS\_SENIS\_AORTA\_AORTIC\_ENDOTHELIAL\_CELL\_AGEING |  | 80 | 0.35 | 2.18 | 0.000 | 0.001 | 0.019 | 919 | tags=55%, list=22%, signal=70% |
| 73 | TABULA\_MURIS\_SENIS\_KIDNEY\_FENESTRATED\_CELL\_AGEING |  | 25 | 0.50 | 2.18 | 0.000 | 0.001 | 0.019 | 387 | tags=36%, list=9%, signal=40% |
| 74 | TABULA\_MURIS\_SENIS\_TRACHEA\_MACROPHAGE\_AGEING |  | 21 | 0.52 | 2.18 | 0.000 | 0.001 | 0.020 | 895 | tags=67%, list=22%, signal=85% |
| 75 | TABULA\_MURIS\_SENIS\_MARROW\_PROERYTHROBLAST\_AGEING |  | 19 | 0.53 | 2.18 | 0.000 | 0.001 | 0.021 | 892 | tags=68%, list=22%, signal=87% |
| 76 | TABULA\_MURIS\_SENIS\_LIVER\_NK\_CELL\_AGEING |  | 67 | 0.37 | 2.17 | 0.000 | 0.001 | 0.021 | 849 | tags=48%, list=21%, signal=59% |
| 77 | TABULA\_MURIS\_SENIS\_LUNG\_BRONCHIAL\_SMOOTH\_MUSCLE\_CELL\_AGEING |  | 50 | 0.39 | 2.12 | 0.000 | 0.001 | 0.032 | 915 | tags=60%, list=22%, signal=76% |
| 78 | TABULA\_MURIS\_SENIS\_KIDNEY\_T\_CELL\_AGEING |  | 18 | 0.53 | 2.11 | 0.002 | 0.001 | 0.035 | 586 | tags=50%, list=14%, signal=58% |
| 79 | TABULA\_MURIS\_SENIS\_MAMMARY\_GLAND\_BASAL\_CELL\_AGEING |  | 125 | 0.30 | 2.08 | 0.000 | 0.001 | 0.040 | 895 | tags=46%, list=22%, signal=58% |
| 80 | TABULA\_MURIS\_SENIS\_GONADAL\_ADIPOSE\_TISSUE\_B\_CELL\_AGEING |  | 28 | 0.45 | 2.07 | 0.000 | 0.001 | 0.045 | 895 | tags=57%, list=22%, signal=73% |
| 81 | TABULA\_MURIS\_SENIS\_MAMMARY\_GLAND\_STROMAL\_CELL\_AGEING |  | 100 | 0.32 | 2.04 | 0.000 | 0.002 | 0.053 | 662 | tags=37%, list=16%, signal=43% |
| 82 | TABULA\_MURIS\_SENIS\_HEART\_AND\_AORTA\_FIBROBLAST\_OF\_CARDIAC\_TISSUE\_AGEING |  | 74 | 0.34 | 2.03 | 0.000 | 0.002 | 0.061 | 860 | tags=51%, list=21%, signal=64% |
| 83 | TABULA\_MURIS\_SENIS\_GONADAL\_ADIPOSE\_TISSUE\_MESENCHYMAL\_STEM\_CELL\_OF\_ADIPOSE\_AGEING |  | 192 | 0.28 | 2.02 | 0.000 | 0.002 | 0.063 | 915 | tags=44%, list=22%, signal=54% |
| 84 | TABULA\_MURIS\_SENIS\_MAMMARY\_GLAND\_LUMINAL\_EPITHELIAL\_CELL\_OF\_MAMMARY\_GLAND\_AGEING |  | 135 | 0.29 | 1.97 | 0.000 | 0.003 | 0.103 | 950 | tags=49%, list=23%, signal=62% |
| 85 | TABULA\_MURIS\_SENIS\_HEART\_FIBROBLAST\_OF\_CARDIAC\_TISSUE\_AGEING |  | 77 | 0.32 | 1.97 | 0.002 | 0.003 | 0.107 | 915 | tags=52%, list=22%, signal=66% |
| 86 | TABULA\_MURIS\_SENIS\_SUBCUTANEOUS\_ADIPOSE\_TISSUE\_ENDOTHELIAL\_CELL\_AGEING |  | 54 | 0.35 | 1.95 | 0.000 | 0.004 | 0.122 | 937 | tags=50%, list=23%, signal=64% |
| 87 | TABULA\_MURIS\_SENIS\_DIAPHRAGM\_SKELETAL\_MUSCLE\_SATELLITE\_CELL\_AGEING |  | 38 | 0.38 | 1.91 | 0.010 | 0.005 | 0.159 | 997 | tags=58%, list=24%, signal=76% |
| 88 | TABULA\_MURIS\_SENIS\_LUNG\_FIBROBLAST\_OF\_LUNG\_AGEING |  | 76 | 0.32 | 1.91 | 0.005 | 0.005 | 0.166 | 915 | tags=45%, list=22%, signal=57% |
| 89 | TABULA\_MURIS\_SENIS\_BLADDER\_ENDOTHELIAL\_CELL\_AGEING |  | 125 | 0.28 | 1.87 | 0.000 | 0.007 | 0.220 | 689 | tags=35%, list=17%, signal=41% |
| 90 | DESCARTES\_ORGANOGENESIS\_ENDOTHELIAL\_CELLS |  | 76 | 0.30 | 1.84 | 0.003 | 0.009 | 0.284 | 875 | tags=50%, list=21%, signal=62% |
| 91 | TABULA\_MURIS\_SENIS\_LIMB\_MUSCLE\_SMOOTH\_MUSCLE\_CELL\_AGEING |  | 29 | 0.38 | 1.75 | 0.021 | 0.015 | 0.454 | 968 | tags=66%, list=24%, signal=85% |
| 92 | TABULA\_MURIS\_SENIS\_MARROW\_NK\_CELL\_AGEING |  | 134 | 0.25 | 1.72 | 0.006 | 0.019 | 0.523 | 1018 | tags=41%, list=25%, signal=53% |
| 93 | TABULA\_MURIS\_SENIS\_KIDNEY\_PODOCYTE\_AGEING |  | 58 | 0.31 | 1.72 | 0.008 | 0.019 | 0.539 | 860 | tags=43%, list=21%, signal=54% |
| 94 | DESCARTES\_ORGANOGENESIS\_MELANOCYTES |  | 15 | 0.47 | 1.71 | 0.037 | 0.019 | 0.545 | 411 | tags=40%, list=10%, signal=44% |
| 95 | TABULA\_MURIS\_SENIS\_DIAPHRAGM\_MESENCHYMAL\_STEM\_CELL\_AGEING |  | 77 | 0.27 | 1.65 | 0.014 | 0.028 | 0.682 | 1035 | tags=49%, list=25%, signal=65% |
| 96 | TABULA\_MURIS\_SENIS\_LUNG\_ADVENTITIAL\_CELL\_AGEING |  | 58 | 0.29 | 1.62 | 0.022 | 0.033 | 0.753 | 962 | tags=50%, list=24%, signal=64% |
| 97 | TABULA\_MURIS\_SENIS\_SKIN\_EPIDERMAL\_CELL\_AGEING |  | 194 | 0.21 | 1.55 | 0.003 | 0.049 | 0.881 | 1023 | tags=40%, list=25%, signal=50% |
| 98 | TABULA\_MURIS\_SENIS\_BROWN\_ADIPOSE\_TISSUE\_ENDOTHELIAL\_CELL\_AGEING |  | 68 | 0.27 | 1.54 | 0.034 | 0.051 | 0.892 | 759 | tags=35%, list=19%, signal=43% |
| 99 | TABULA\_MURIS\_SENIS\_KIDNEY\_KIDNEY\_DISTAL\_CONVOLUTED\_TUBULE\_EPITHELIAL\_CELL\_AGEING |  | 30 | 0.32 | 1.52 | 0.060 | 0.058 | 0.921 | 895 | tags=50%, list=22%, signal=64% |
| 100 | TABULA\_MURIS\_SENIS\_MARROW\_LATE\_PRO\_B\_CELL\_AGEING |  | 51 | 0.27 | 1.47 | 0.059 | 0.074 | 0.972 | 1927 | tags=76%, list=47%, signal=143% |
| 101 | TABULA\_MURIS\_SENIS\_LIVER\_MATURE\_NK\_T\_CELL\_AGEING |  | 31 | 0.31 | 1.47 | 0.078 | 0.073 | 0.972 | 219 | tags=19%, list=5%, signal=20% |
| 102 | TABULA\_MURIS\_SENIS\_PANCREAS\_LEUKOCYTE\_AGEING |  | 17 | 0.37 | 1.45 | 0.075 | 0.079 | 0.977 | 971 | tags=65%, list=24%, signal=84% |
| 103 | TABULA\_MURIS\_SENIS\_HEART\_VALVE\_CELL\_AGEING |  | 34 | 0.30 | 1.44 | 0.094 | 0.081 | 0.979 | 910 | tags=47%, list=22%, signal=60% |
| 104 | TABULA\_MURIS\_SENIS\_KIDNEY\_KIDNEY\_LOOP\_OF\_HENLE\_THICK\_ASCENDING\_LIMB\_EPITHELIAL\_CELL\_AGEING |  | 62 | 0.24 | 1.37 | 0.070 | 0.120 | 0.998 | 923 | tags=45%, list=23%, signal=57% |
| 105 | TABULA\_MURIS\_SENIS\_GONADAL\_ADIPOSE\_TISSUE\_ENDOTHELIAL\_CELL\_AGEING |  | 277 | 0.17 | 1.36 | 0.033 | 0.127 | 0.999 | 1035 | tags=37%, list=25%, signal=46% |
| 106 | ZHANG\_UTERUS\_C15\_B\_CELL |  | 24 | 0.30 | 1.32 | 0.153 | 0.149 | 1.000 | 391 | tags=29%, list=10%, signal=32% |
| 107 | TABULA\_MURIS\_SENIS\_MESENTERIC\_ADIPOSE\_TISSUE\_MESENCHYMAL\_STEM\_CELL\_OF\_ADIPOSE\_AGEING |  | 186 | 0.18 | 1.32 | 0.064 | 0.148 | 1.000 | 916 | tags=39%, list=22%, signal=48% |
| 108 | TABULA\_MURIS\_SENIS\_SKIN\_BULGE\_KERATINOCYTE\_AGEING |  | 233 | 0.17 | 1.31 | 0.056 | 0.153 | 1.000 | 867 | tags=31%, list=21%, signal=37% |
| 109 | TABULA\_MURIS\_SENIS\_PANCREAS\_PANCREATIC\_ALPHA\_CELL\_AGEING |  | 41 | 0.25 | 1.29 | 0.166 | 0.173 | 1.000 | 239 | tags=15%, list=6%, signal=15% |
| 110 | TABULA\_MURIS\_SENIS\_BRAIN\_NON\_MYELOID\_ENDOTHELIAL\_CELL\_AGEING |  | 88 | 0.21 | 1.27 | 0.150 | 0.184 | 1.000 | 1018 | tags=43%, list=25%, signal=56% |
| 111 | TABULA\_MURIS\_SENIS\_HEART\_ATRIAL\_MYOCYTE\_AGEING |  | 44 | 0.24 | 1.26 | 0.198 | 0.197 | 1.000 | 390 | tags=20%, list=10%, signal=22% |
| 112 | TABULA\_MURIS\_SENIS\_MARROW\_GRANULOCYTE\_MONOCYTE\_PROGENITOR\_CELL\_AGEING |  | 50 | 0.22 | 1.18 | 0.233 | 0.280 | 1.000 | 264 | tags=14%, list=6%, signal=15% |
| 113 | TABULA\_MURIS\_SENIS\_BRAIN\_NON\_MYELOID\_BRAIN\_PERICYTE\_AGEING |  | 183 | 0.16 | 1.17 | 0.149 | 0.282 | 1.000 | 719 | tags=25%, list=18%, signal=29% |
| 114 | TABULA\_MURIS\_SENIS\_SKIN\_BASAL\_CELL\_OF\_EPIDERMIS\_AGEING |  | 254 | 0.15 | 1.16 | 0.159 | 0.292 | 1.000 | 1287 | tags=45%, list=31%, signal=61% |
| 115 | TABULA\_MURIS\_SENIS\_SPLEEN\_GRANULOCYTE\_AGEING |  | 48 | 0.21 | 1.15 | 0.265 | 0.300 | 1.000 | 724 | tags=29%, list=18%, signal=35% |
| 116 | TABULA\_MURIS\_SENIS\_MARROW\_MATURE\_ALPHA\_BETA\_T\_CELL\_AGEING |  | 125 | 0.17 | 1.13 | 0.247 | 0.329 | 1.000 | 1116 | tags=42%, list=27%, signal=57% |
| 117 | TABULA\_MURIS\_SENIS\_THYMUS\_THYMOCYTE\_AGEING |  | 101 | 0.17 | 1.08 | 0.306 | 0.399 | 1.000 | 1883 | tags=70%, list=46%, signal=127% |
| 118 | TABULA\_MURIS\_SENIS\_KIDNEY\_BRUSH\_CELL\_AGEING |  | 17 | 0.27 | 1.05 | 0.362 | 0.437 | 1.000 | 88 | tags=12%, list=2%, signal=12% |
| 119 | TABULA\_MURIS\_SENIS\_MESENTERIC\_ADIPOSE\_TISSUE\_ENDOTHELIAL\_CELL\_AGEING |  | 20 | 0.26 | 1.05 | 0.365 | 0.434 | 1.000 | 915 | tags=45%, list=22%, signal=58% |
| 120 | TABULA\_MURIS\_SENIS\_KIDNEY\_KIDNEY\_PROXIMAL\_CONVOLUTED\_TUBULE\_EPITHELIAL\_CELL\_AGEING |  | 48 | 0.20 | 1.05 | 0.392 | 0.444 | 1.000 | 280 | tags=17%, list=7%, signal=18% |
| 121 | ZHANG\_UTERUS\_C7\_EPITHELIAL2\_CELL |  | 181 | 0.14 | 1.04 | 0.379 | 0.446 | 1.000 | 1015 | tags=35%, list=25%, signal=44% |
| 122 | TABULA\_MURIS\_SENIS\_BRAIN\_NON\_MYELOID\_OLIGODENDROCYTE\_PRECURSOR\_CELL\_AGEING |  | 111 | 0.16 | 1.04 | 0.385 | 0.448 | 1.000 | 1007 | tags=37%, list=25%, signal=48% |
| 123 | TABULA\_MURIS\_SENIS\_SUBCUTANEOUS\_ADIPOSE\_TISSUE\_EPITHELIAL\_CELL\_AGEING |  | 174 | 0.14 | 1.00 | 0.448 | 0.506 | 1.000 | 639 | tags=21%, list=16%, signal=23% |
| 124 | TABULA\_MURIS\_SENIS\_AORTA\_FIBROBLAST\_OF\_CARDIAC\_TISSUE\_AGEING |  | 208 | 0.14 | 1.00 | 0.510 | 0.514 | 1.000 | 736 | tags=24%, list=18%, signal=28% |
| 125 | TABULA\_MURIS\_SENIS\_BRAIN\_NON\_MYELOID\_ASTROCYTE\_AGEING |  | 127 | 0.14 | 0.98 | 0.516 | 0.533 | 1.000 | 895 | tags=30%, list=22%, signal=37% |
| 126 | TABULA\_MURIS\_SENIS\_MAMMARY\_GLAND\_ENDOTHELIAL\_CELL\_AGEING |  | 155 | 0.14 | 0.98 | 0.505 | 0.538 | 1.000 | 919 | tags=33%, list=22%, signal=41% |
| 127 | TABULA\_MURIS\_SENIS\_KIDNEY\_KIDNEY\_COLLECTING\_DUCT\_PRINCIPAL\_CELL\_AGEING |  | 115 | 0.14 | 0.97 | 0.516 | 0.550 | 1.000 | 979 | tags=37%, list=24%, signal=48% |
| 128 | DESCARTES\_ORGANOGENESIS\_CARDIAC\_MUSCLE\_LINEAGES |  | 23 | 0.22 | 0.93 | 0.539 | 0.603 | 1.000 | 759 | tags=43%, list=19%, signal=53% |
| 129 | TABULA\_MURIS\_SENIS\_TRACHEA\_T\_CELL\_AGEING |  | 134 | 0.13 | 0.86 | 0.734 | 0.725 | 1.000 | 3397 | tags=97%, list=83%, signal=554% |
| 130 | DESCARTES\_ORGANOGENESIS\_CHONDROCYTE\_PROGENITORS |  | 16 | 0.22 | 0.84 | 0.655 | 0.745 | 1.000 | 335 | tags=19%, list=8%, signal=20% |
| 131 | TABULA\_MURIS\_SENIS\_MARROW\_HEMATOPOIETIC\_STEM\_CELL\_AGEING |  | 186 | 0.12 | 0.83 | 0.807 | 0.756 | 1.000 | 701 | tags=20%, list=17%, signal=24% |
| 132 | TABULA\_MURIS\_SENIS\_PANCREAS\_ENDOTHELIAL\_CELL\_AGEING |  | 68 | 0.14 | 0.79 | 0.791 | 0.813 | 1.000 | 651 | tags=22%, list=16%, signal=26% |
| 133 | TABULA\_MURIS\_SENIS\_SPLEEN\_PROERYTHROBLAST\_AGEING |  | 221 | 0.10 | 0.75 | 0.929 | 0.845 | 1.000 | 2143 | tags=69%, list=52%, signal=137% |
| 134 | TABULA\_MURIS\_SENIS\_BROWN\_ADIPOSE\_TISSUE\_MESENCHYMAL\_STEM\_CELL\_OF\_ADIPOSE\_AGEING |  | 290 | 0.09 | 0.71 | 0.984 | 0.881 | 1.000 | 916 | tags=29%, list=22%, signal=34% |
| 135 | TABULA\_MURIS\_SENIS\_TRACHEA\_ENDOTHELIAL\_CELL\_AGEING |  | 55 | 0.12 | 0.68 | 0.884 | 0.906 | 1.000 | 1286 | tags=49%, list=31%, signal=71% |
| 136 | TABULA\_MURIS\_SENIS\_BRAIN\_NON\_MYELOID\_OLIGODENDROCYTE\_AGEING |  | 268 | 0.06 | 0.44 | 1.000 | 0.995 | 1.000 | 3421 | tags=94%, list=84%, signal=535% |
Table: Gene sets enriched in phenotype **na**[plain text format]****

  
