# Supplementary material for: TMPRSS11B promotes an acidified microenvironment and immune suppression in squamous lung cancer
Source: EMBO Rep. 2025 Nov 10;26(24):6346–79. doi: 10.1038/s44319-025-00631-1 (PMC12714794; doi:10.1038/s44319-025-00631-1)
Supplement: Supplementary file 18 — Figure EV6 Source Data [file 44319_2025_631_MOESM18_ESM.zip › Figure EV6/EV6C-D/GSEA_Broad Institute_M8_T11b high vs low LUSC/TABULA_MURIS_SENIS_AORTA_PROFESSIONAL_ANTIGEN_PRESENTING_CELL_AGEING.html]

Details for gene set TABULA\_MURIS\_SENIS\_AORTA\_PROFESSIONAL\_ANTIGEN\_PRESENTING\_CELL\_AGEING[GSEA]

|  || Dataset | T11b high vs low squamous\_GSEA\_Ranked |
| Phenotype | NoPhenotypeAvailable |
| Upregulated in class | na\_pos |
| GeneSet | TABULA\_MURIS\_SENIS\_AORTA\_PROFESSIONAL\_ANTIGEN\_PRESENTING\_CELL\_AGEING |
| Enrichment Score (ES) | 0.6308809 |
| Normalized Enrichment Score (NES) | 3.3279507 |
| Nominal p-value | 0.0 |
| FDR q-value | 0.0 |
| FWER p-Value | 0.0 |
Table: GSEA Results Summary

  

Fig 1: Enrichment plot: TABULA\_MURIS\_SENIS\_AORTA\_PROFESSIONAL\_ANTIGEN\_PRESENTING\_CELL\_AGEING      
 Profile of the Running ES Score & Positions of GeneSet Members on the Rank Ordered List

  

| SYMBOL | RANK IN GENE LIST | RANK METRIC SCORE | RUNNING ES | CORE ENRICHMENT || 1 | Krt14 | 56 | 2.659 | 0.0325 | Yes |
| 2 | Ctss | 63 | 2.582 | 0.0761 | Yes |
| 3 | Fcer1g | 76 | 2.415 | 0.1153 | Yes |
| 4 | Tyrobp | 83 | 2.366 | 0.1550 | Yes |
| 5 | Apoe | 88 | 2.296 | 0.1941 | Yes |
| 6 | Ly6a | 92 | 2.274 | 0.2330 | Yes |
| 7 | Cd68 | 97 | 2.215 | 0.2707 | Yes |
| 8 | Wfdc17 | 117 | 1.998 | 0.3008 | Yes |
| 9 | Ctsz | 138 | 1.884 | 0.3288 | Yes |
| 10 | Tgfbi | 151 | 1.825 | 0.3576 | Yes |
| 11 | Fxyd5 | 157 | 1.767 | 0.3872 | Yes |
| 12 | Cdkn1a | 192 | 1.625 | 0.4072 | Yes |
| 13 | Arhgdib | 214 | 1.549 | 0.4290 | Yes |
| 14 | Dusp1 | 239 | 1.468 | 0.4487 | Yes |
| 15 | Psap | 240 | 1.466 | 0.4743 | Yes |
| 16 | Srgn | 270 | 1.392 | 0.4914 | Yes |
| 17 | Grn | 285 | 1.352 | 0.5115 | Yes |
| 18 | Capg | 343 | 1.160 | 0.5176 | Yes |
| 19 | Cd52 | 350 | 1.140 | 0.5360 | Yes |
| 20 | Coro1a | 390 | 1.079 | 0.5452 | Yes |
| 21 | Alox5ap | 399 | 1.051 | 0.5616 | Yes |
| 22 | Lpxn | 431 | 1.004 | 0.5714 | Yes |
| 23 | Ifitm2 | 465 | 0.952 | 0.5799 | Yes |
| 24 | Cebpb | 504 | 0.897 | 0.5861 | Yes |
| 25 | Cyba | 519 | 0.875 | 0.5979 | Yes |
| 26 | Trf | 601 | 0.771 | 0.5913 | Yes |
| 27 | Actb | 633 | 0.726 | 0.5963 | Yes |
| 28 | H2-D1 | 719 | 0.654 | 0.5867 | Yes |
| 29 | Timp3 | 754 | 0.625 | 0.5892 | Yes |
| 30 | Anxa2 | 760 | 0.620 | 0.5988 | Yes |
| 31 | Sparc | 792 | 0.595 | 0.6015 | Yes |
| 32 | Gm2a | 815 | 0.585 | 0.6063 | Yes |
| 33 | Cd74 | 849 | 0.567 | 0.6080 | Yes |
| 34 | H2-K1 | 855 | 0.565 | 0.6166 | Yes |
| 35 | B2m | 860 | 0.563 | 0.6254 | Yes |
| 36 | Cfl1 | 895 | 0.538 | 0.6264 | Yes |
| 37 | H2-Ab1 | 915 | 0.525 | 0.6309 | Yes |
| 38 | Nfkbia | 954 | 0.502 | 0.6302 | No |
| 39 | Reep5 | 1833 | -0.657 | 0.4246 | No |
| 40 | Ly6e | 1857 | -0.663 | 0.4305 | No |
| 41 | Tgif1 | 1899 | -0.673 | 0.4321 | No |
| 42 | Oas1a | 2196 | -0.737 | 0.3717 | No |
| 43 | Snx2 | 2507 | -0.820 | 0.3094 | No |
| 44 | Shisa5 | 2916 | -0.946 | 0.2250 | No |
| 45 | Fos | 3189 | -1.058 | 0.1762 | No |
| 46 | Krt15 | 4063 | -2.644 | 0.0064 | No |
Table: GSEA details [plain text format]

  

Fig 2: TABULA\_MURIS\_SENIS\_AORTA\_PROFESSIONAL\_ANTIGEN\_PRESENTING\_CELL\_AGEING: Random ES distribution      
 Gene set null distribution of ES for **TABULA\_MURIS\_SENIS\_AORTA\_PROFESSIONAL\_ANTIGEN\_PRESENTING\_CELL\_AGEING**

  
