# Supplementary material for: TMPRSS11B promotes an acidified microenvironment and immune suppression in squamous lung cancer
Source: EMBO Rep. 2025 Nov 10;26(24):6346–79. doi: 10.1038/s44319-025-00631-1 (PMC12714794; doi:10.1038/s44319-025-00631-1)
Supplement: Supplementary file 18 — Figure EV6 Source Data [file 44319_2025_631_MOESM18_ESM.zip › Figure EV6/EV6C-D/GSEA_Broad Institute_M8_T11b high vs low LUSC/TABULA_MURIS_SENIS_BRAIN_MYELOID_MICROGLIAL_CELL_AGEING.html]

Details for gene set TABULA\_MURIS\_SENIS\_BRAIN\_MYELOID\_MICROGLIAL\_CELL\_AGEING[GSEA]

|  || Dataset | T11b high vs low squamous\_GSEA\_Ranked |
| Phenotype | NoPhenotypeAvailable |
| Upregulated in class | na\_pos |
| GeneSet | TABULA\_MURIS\_SENIS\_BRAIN\_MYELOID\_MICROGLIAL\_CELL\_AGEING |
| Enrichment Score (ES) | 0.5316557 |
| Normalized Enrichment Score (NES) | 3.395433 |
| Nominal p-value | 0.0 |
| FDR q-value | 0.0 |
| FWER p-Value | 0.0 |
Table: GSEA Results Summary

  

Fig 1: Enrichment plot: TABULA\_MURIS\_SENIS\_BRAIN\_MYELOID\_MICROGLIAL\_CELL\_AGEING      
 Profile of the Running ES Score & Positions of GeneSet Members on the Rank Ordered List

  

| SYMBOL | RANK IN GENE LIST | RANK METRIC SCORE | RUNNING ES | CORE ENRICHMENT || 1 | Ctsl | 15 | 3.988 | 0.0315 | Yes |
| 2 | Trem2 | 24 | 3.569 | 0.0611 | Yes |
| 3 | Cd300c2 | 42 | 2.822 | 0.0818 | Yes |
| 4 | Lpl | 43 | 2.812 | 0.1067 | Yes |
| 5 | Krt14 | 56 | 2.659 | 0.1272 | Yes |
| 6 | Fcgr2b | 62 | 2.610 | 0.1491 | Yes |
| 7 | Ctss | 63 | 2.582 | 0.1719 | Yes |
| 8 | Cd37 | 74 | 2.458 | 0.1912 | Yes |
| 9 | Fcer1g | 76 | 2.415 | 0.2123 | Yes |
| 10 | Tyrobp | 83 | 2.366 | 0.2317 | Yes |
| 11 | Apoe | 88 | 2.296 | 0.2510 | Yes |
| 12 | Ly6a | 92 | 2.274 | 0.2704 | Yes |
| 13 | Cd68 | 97 | 2.215 | 0.2890 | Yes |
| 14 | Vim | 112 | 2.054 | 0.3037 | Yes |
| 15 | C1qa | 119 | 1.990 | 0.3198 | Yes |
| 16 | Ctsz | 138 | 1.884 | 0.3319 | Yes |
| 17 | C1qb | 139 | 1.882 | 0.3486 | Yes |
| 18 | Fth1 | 147 | 1.835 | 0.3631 | Yes |
| 19 | Spi1 | 158 | 1.765 | 0.3762 | Yes |
| 20 | Gpsm3 | 169 | 1.725 | 0.3890 | Yes |
| 21 | Ctsb | 177 | 1.695 | 0.4022 | Yes |
| 22 | Crlf2 | 182 | 1.670 | 0.4160 | Yes |
| 23 | Csf2ra | 185 | 1.657 | 0.4302 | Yes |
| 24 | Cdkn1a | 192 | 1.625 | 0.4430 | Yes |
| 25 | Arhgdib | 214 | 1.549 | 0.4515 | Yes |
| 26 | C1qc | 234 | 1.480 | 0.4598 | Yes |
| 27 | Srgn | 270 | 1.392 | 0.4634 | Yes |
| 28 | Cxcl16 | 342 | 1.161 | 0.4559 | Yes |
| 29 | Capg | 343 | 1.160 | 0.4661 | Yes |
| 30 | Cd52 | 350 | 1.140 | 0.4747 | Yes |
| 31 | Ppp1r18 | 371 | 1.111 | 0.4795 | Yes |
| 32 | Coro1a | 390 | 1.079 | 0.4846 | Yes |
| 33 | Plekho1 | 418 | 1.020 | 0.4868 | Yes |
| 34 | Trim35 | 453 | 0.972 | 0.4869 | Yes |
| 35 | Tubb6 | 460 | 0.961 | 0.4939 | Yes |
| 36 | Timp2 | 462 | 0.955 | 0.5022 | Yes |
| 37 | Prelid1 | 506 | 0.892 | 0.4993 | Yes |
| 38 | Cyba | 519 | 0.875 | 0.5040 | Yes |
| 39 | Npc2 | 536 | 0.861 | 0.5076 | Yes |
| 40 | Atp6v0e | 590 | 0.795 | 0.5014 | Yes |
| 41 | Cd63 | 632 | 0.727 | 0.4976 | Yes |
| 42 | Cotl1 | 656 | 0.709 | 0.4981 | Yes |
| 43 | Fabp4 | 657 | 0.708 | 0.5043 | Yes |
| 44 | Ddah2 | 658 | 0.706 | 0.5106 | Yes |
| 45 | Lat2 | 714 | 0.657 | 0.5026 | Yes |
| 46 | H2-D1 | 719 | 0.654 | 0.5074 | Yes |
| 47 | Serping1 | 730 | 0.645 | 0.5106 | Yes |
| 48 | Anxa2 | 760 | 0.620 | 0.5089 | Yes |
| 49 | Arrb2 | 786 | 0.600 | 0.5079 | Yes |
| 50 | Sparc | 792 | 0.595 | 0.5119 | Yes |
| 51 | Arpc4 | 798 | 0.593 | 0.5159 | Yes |
| 52 | Pkm | 807 | 0.591 | 0.5191 | Yes |
| 53 | Cd74 | 849 | 0.567 | 0.5139 | Yes |
| 54 | H2-K1 | 855 | 0.565 | 0.5176 | Yes |
| 55 | B2m | 860 | 0.563 | 0.5216 | Yes |
| 56 | Atp6v0b | 884 | 0.550 | 0.5207 | Yes |
| 57 | Cfl1 | 895 | 0.538 | 0.5230 | Yes |
| 58 | Dpysl2 | 910 | 0.527 | 0.5241 | Yes |
| 59 | H2-Ab1 | 915 | 0.525 | 0.5278 | Yes |
| 60 | Tspan7 | 919 | 0.523 | 0.5317 | Yes |
| 61 | Limd2 | 971 | -0.501 | 0.5233 | No |
| 62 | Eef1d | 991 | -0.504 | 0.5230 | No |
| 63 | Vps28 | 1018 | -0.509 | 0.5210 | No |
| 64 | Cox7a2l | 1218 | -0.543 | 0.4760 | No |
| 65 | Emc10 | 1234 | -0.546 | 0.4771 | No |
| 66 | Eif3k | 1325 | -0.562 | 0.4595 | No |
| 67 | Ppp1r35 | 1611 | -0.612 | 0.3936 | No |
| 68 | Tmed9 | 1675 | -0.626 | 0.3834 | No |
| 69 | Pabpn1 | 1680 | -0.626 | 0.3879 | No |
| 70 | Ifi27 | 1778 | -0.644 | 0.3693 | No |
| 71 | Aga | 1798 | -0.648 | 0.3703 | No |
| 72 | Naxe | 1823 | -0.654 | 0.3701 | No |
| 73 | Reep5 | 1833 | -0.657 | 0.3737 | No |
| 74 | Ly6e | 1857 | -0.663 | 0.3738 | No |
| 75 | H2-M3 | 1927 | -0.681 | 0.3625 | No |
| 76 | Gstm1 | 2049 | -0.704 | 0.3385 | No |
| 77 | Gadd45gip1 | 2067 | -0.712 | 0.3405 | No |
| 78 | Ddhd2 | 2180 | -0.735 | 0.3190 | No |
| 79 | Kdelr1 | 2186 | -0.736 | 0.3242 | No |
| 80 | Vkorc1 | 2191 | -0.737 | 0.3297 | No |
| 81 | Ypel3 | 2253 | -0.751 | 0.3211 | No |
| 82 | Scn1b | 2310 | -0.764 | 0.3139 | No |
| 83 | Bsg | 2530 | -0.827 | 0.2664 | No |
| 84 | Eif3f | 2655 | -0.863 | 0.2430 | No |
| 85 | Tmed3 | 2696 | -0.874 | 0.2407 | No |
| 86 | Rabac1 | 2744 | -0.888 | 0.2368 | No |
| 87 | Shisa5 | 2916 | -0.946 | 0.2024 | No |
| 88 | Bri3 | 2942 | -0.954 | 0.2045 | No |
| 89 | Selenos | 2961 | -0.960 | 0.2085 | No |
| 90 | Dok1 | 3135 | -1.034 | 0.1744 | No |
| 91 | Lgals3bp | 3172 | -1.049 | 0.1746 | No |
| 92 | Sf3b4 | 3336 | -1.127 | 0.1438 | No |
| 93 | Mid1ip1 | 3392 | -1.158 | 0.1403 | No |
| 94 | Clu | 3405 | -1.162 | 0.1476 | No |
| 95 | Krt15 | 4063 | -2.644 | 0.0065 | No |
Table: GSEA details [plain text format]

  

Fig 2: TABULA\_MURIS\_SENIS\_BRAIN\_MYELOID\_MICROGLIAL\_CELL\_AGEING: Random ES distribution      
 Gene set null distribution of ES for **TABULA\_MURIS\_SENIS\_BRAIN\_MYELOID\_MICROGLIAL\_CELL\_AGEING**

  
