# Supplementary material for: TMPRSS11B promotes an acidified microenvironment and immune suppression in squamous lung cancer
Source: EMBO Rep. 2025 Nov 10;26(24):6346–79. doi: 10.1038/s44319-025-00631-1 (PMC12714794; doi:10.1038/s44319-025-00631-1)
Supplement: Supplementary file 18 — Figure EV6 Source Data [file 44319_2025_631_MOESM18_ESM.zip › Figure EV6/EV6C-D/GSEA_Broad Institute_M8_T11b high vs low LUSC/TABULA_MURIS_SENIS_BRAIN_NON_MYELOID_NEURON_AGEING.html]

Details for gene set TABULA\_MURIS\_SENIS\_BRAIN\_NON\_MYELOID\_NEURON\_AGEING[GSEA]

|  || Dataset | T11b high vs low squamous\_GSEA\_Ranked |
| Phenotype | NoPhenotypeAvailable |
| Upregulated in class | na\_neg |
| GeneSet | TABULA\_MURIS\_SENIS\_BRAIN\_NON\_MYELOID\_NEURON\_AGEING |
| Enrichment Score (ES) | -0.11116566 |
| Normalized Enrichment Score (NES) | -0.78490466 |
| Nominal p-value | 0.87240356 |
| FDR q-value | 1.0 |
| FWER p-Value | 1.0 |
Table: GSEA Results Summary

  

Fig 1: Enrichment plot: TABULA\_MURIS\_SENIS\_BRAIN\_NON\_MYELOID\_NEURON\_AGEING      
 Profile of the Running ES Score & Positions of GeneSet Members on the Rank Ordered List

  

| SYMBOL | RANK IN GENE LIST | RANK METRIC SCORE | RUNNING ES | CORE ENRICHMENT || 1 | Mamdc2 | 68 | 2.532 | -0.0058 | No |
| 2 | Apoe | 88 | 2.296 | 0.0001 | No |
| 3 | Vim | 112 | 2.054 | 0.0038 | No |
| 4 | Fth1 | 147 | 1.835 | 0.0036 | No |
| 5 | Sirpa | 154 | 1.794 | 0.0105 | No |
| 6 | Spns2 | 218 | 1.525 | 0.0013 | No |
| 7 | 5031439G07Rik | 237 | 1.473 | 0.0035 | No |
| 8 | Cldn5 | 252 | 1.447 | 0.0067 | No |
| 9 | Capg | 343 | 1.160 | -0.0112 | No |
| 10 | Col3a1 | 383 | 1.092 | -0.0162 | No |
| 11 | Fabp5 | 385 | 1.088 | -0.0114 | No |
| 12 | Abcc5 | 400 | 1.047 | -0.0101 | No |
| 13 | Plekho1 | 418 | 1.020 | -0.0097 | No |
| 14 | Nectin1 | 428 | 1.008 | -0.0073 | No |
| 15 | Flna | 471 | 0.946 | -0.0138 | No |
| 16 | Col1a1 | 483 | 0.921 | -0.0123 | No |
| 17 | Plpp3 | 490 | 0.910 | -0.0096 | No |
| 18 | Sult2b1 | 497 | 0.901 | -0.0069 | No |
| 19 | Rnf149 | 502 | 0.897 | -0.0037 | No |
| 20 | Trf | 601 | 0.771 | -0.0256 | No |
| 21 | Cat | 639 | 0.721 | -0.0318 | No |
| 22 | Ddah2 | 658 | 0.706 | -0.0331 | No |
| 23 | Pltp | 661 | 0.702 | -0.0304 | No |
| 24 | Klf4 | 662 | 0.701 | -0.0271 | No |
| 25 | Smpdl3b | 682 | 0.684 | -0.0288 | No |
| 26 | Wwtr1 | 705 | 0.663 | -0.0314 | No |
| 27 | H2-D1 | 719 | 0.654 | -0.0317 | No |
| 28 | Col1a2 | 729 | 0.645 | -0.0310 | No |
| 29 | Rsu1 | 731 | 0.645 | -0.0282 | No |
| 30 | 2310022B05Rik | 740 | 0.637 | -0.0273 | No |
| 31 | Csrp1 | 743 | 0.636 | -0.0248 | No |
| 32 | Gatm | 755 | 0.624 | -0.0248 | No |
| 33 | Anxa2 | 760 | 0.620 | -0.0229 | No |
| 34 | S100a16 | 775 | 0.605 | -0.0237 | No |
| 35 | B4galt5 | 778 | 0.602 | -0.0214 | No |
| 36 | Tgfb1i1 | 779 | 0.601 | -0.0185 | No |
| 37 | Arrb2 | 786 | 0.600 | -0.0173 | No |
| 38 | Sparc | 792 | 0.595 | -0.0158 | No |
| 39 | Slc36a1 | 833 | 0.573 | -0.0235 | No |
| 40 | Cd74 | 849 | 0.567 | -0.0247 | No |
| 41 | H2-K1 | 855 | 0.565 | -0.0234 | No |
| 42 | Snf8 | 857 | 0.564 | -0.0210 | No |
| 43 | B2m | 860 | 0.563 | -0.0188 | No |
| 44 | Ppp1r12c | 874 | 0.557 | -0.0196 | No |
| 45 | Gtpbp2 | 907 | 0.529 | -0.0254 | No |
| 46 | Arhgap27 | 911 | 0.526 | -0.0237 | No |
| 47 | Mtln | 917 | 0.524 | -0.0226 | No |
| 48 | Wipi1 | 941 | 0.510 | -0.0261 | No |
| 49 | Dgkz | 955 | 0.502 | -0.0272 | No |
| 50 | Tmem41a | 960 | 0.500 | -0.0258 | No |
| 51 | Sfxn3 | 985 | -0.503 | -0.0297 | No |
| 52 | Txndc11 | 996 | -0.504 | -0.0299 | No |
| 53 | Mblac1 | 1001 | -0.506 | -0.0286 | No |
| 54 | Gnb2 | 1007 | -0.506 | -0.0275 | No |
| 55 | Ado | 1010 | -0.507 | -0.0257 | No |
| 56 | Yeats2 | 1033 | -0.510 | -0.0290 | No |
| 57 | Haghl | 1038 | -0.511 | -0.0276 | No |
| 58 | Clec16a | 1048 | -0.513 | -0.0275 | No |
| 59 | Agpat5 | 1074 | -0.517 | -0.0316 | No |
| 60 | Mon2 | 1103 | -0.523 | -0.0364 | No |
| 61 | C1qtnf1 | 1109 | -0.524 | -0.0352 | No |
| 62 | Mpv17l2 | 1110 | -0.524 | -0.0328 | No |
| 63 | Nt5c | 1142 | -0.530 | -0.0383 | No |
| 64 | Mecr | 1153 | -0.530 | -0.0385 | No |
| 65 | Ankrd54 | 1157 | -0.531 | -0.0367 | No |
| 66 | Cbl | 1164 | -0.533 | -0.0358 | No |
| 67 | Map1s | 1174 | -0.535 | -0.0356 | No |
| 68 | Hsd17b11 | 1184 | -0.536 | -0.0354 | No |
| 69 | Prrg2 | 1221 | -0.544 | -0.0422 | No |
| 70 | Trappc6a | 1231 | -0.545 | -0.0420 | No |
| 71 | Vgll4 | 1244 | -0.547 | -0.0425 | No |
| 72 | Madd | 1256 | -0.548 | -0.0428 | No |
| 73 | Terf1 | 1285 | -0.553 | -0.0475 | No |
| 74 | Ctc1 | 1298 | -0.554 | -0.0480 | No |
| 75 | Mtmr10 | 1309 | -0.558 | -0.0480 | No |
| 76 | Pla2g4e | 1317 | -0.560 | -0.0472 | No |
| 77 | Thoc6 | 1346 | -0.565 | -0.0518 | No |
| 78 | Zfp827 | 1374 | -0.570 | -0.0561 | No |
| 79 | Slc35d1 | 1376 | -0.570 | -0.0537 | No |
| 80 | Pick1 | 1384 | -0.571 | -0.0528 | No |
| 81 | Gpatch8 | 1387 | -0.571 | -0.0507 | No |
| 82 | Krt8 | 1410 | -0.578 | -0.0537 | No |
| 83 | Ehmt2 | 1430 | -0.581 | -0.0559 | No |
| 84 | Nr2c2ap | 1434 | -0.582 | -0.0539 | No |
| 85 | Foxj3 | 1485 | -0.591 | -0.0641 | No |
| 86 | Cep164 | 1492 | -0.593 | -0.0629 | No |
| 87 | AU040320 | 1504 | -0.595 | -0.0630 | No |
| 88 | Crtc3 | 1505 | -0.597 | -0.0602 | No |
| 89 | Nfat5 | 1506 | -0.597 | -0.0573 | No |
| 90 | Zfp821 | 1513 | -0.597 | -0.0561 | No |
| 91 | N4bp2 | 1516 | -0.597 | -0.0538 | No |
| 92 | Trak1 | 1520 | -0.597 | -0.0518 | No |
| 93 | Gtf3c5 | 1528 | -0.599 | -0.0508 | No |
| 94 | Fam98c | 1529 | -0.599 | -0.0480 | No |
| 95 | Wdr59 | 1536 | -0.600 | -0.0467 | No |
| 96 | Cystm1 | 1549 | -0.602 | -0.0470 | No |
| 97 | Commd9 | 1553 | -0.602 | -0.0450 | No |
| 98 | Gpt | 1561 | -0.605 | -0.0439 | No |
| 99 | Slc9a6 | 1585 | -0.608 | -0.0470 | No |
| 100 | Acvrl1 | 1592 | -0.610 | -0.0457 | No |
| 101 | Prkce | 1609 | -0.612 | -0.0470 | No |
| 102 | Ppp1r35 | 1611 | -0.612 | -0.0444 | No |
| 103 | Asb6 | 1631 | -0.617 | -0.0464 | No |
| 104 | Tmem208 | 1679 | -0.626 | -0.0557 | No |
| 105 | Pabpn1 | 1680 | -0.626 | -0.0527 | No |
| 106 | Fh1 | 1687 | -0.627 | -0.0514 | No |
| 107 | Tchh | 1693 | -0.628 | -0.0497 | No |
| 108 | Tmem259 | 1696 | -0.629 | -0.0473 | No |
| 109 | Ift43 | 1721 | -0.634 | -0.0505 | No |
| 110 | Tigd5 | 1726 | -0.634 | -0.0486 | No |
| 111 | Gpr137 | 1746 | -0.639 | -0.0505 | No |
| 112 | Ptrhd1 | 1747 | -0.639 | -0.0475 | No |
| 113 | 1700025G04Rik | 1749 | -0.639 | -0.0448 | No |
| 114 | Tsc1 | 1813 | -0.651 | -0.0581 | No |
| 115 | Glt8d1 | 1829 | -0.656 | -0.0589 | No |
| 116 | Ankrd13d | 1861 | -0.664 | -0.0638 | No |
| 117 | Auts2 | 1865 | -0.665 | -0.0615 | No |
| 118 | Grcc10 | 1882 | -0.670 | -0.0625 | No |
| 119 | Ppa2 | 1908 | -0.675 | -0.0658 | No |
| 120 | Arl3 | 1918 | -0.678 | -0.0649 | No |
| 121 | Zfp362 | 1935 | -0.681 | -0.0659 | No |
| 122 | Endog | 1948 | -0.685 | -0.0658 | No |
| 123 | Camk2n1 | 1967 | -0.688 | -0.0672 | No |
| 124 | Ccdc9 | 1980 | -0.689 | -0.0671 | No |
| 125 | Cebpd | 2002 | -0.693 | -0.0693 | No |
| 126 | Clk2 | 2010 | -0.695 | -0.0679 | No |
| 127 | Rnf138 | 2037 | -0.700 | -0.0713 | No |
| 128 | Pus10 | 2045 | -0.702 | -0.0698 | No |
| 129 | Gstm1 | 2049 | -0.704 | -0.0673 | No |
| 130 | Selenop | 2054 | -0.707 | -0.0650 | No |
| 131 | Gadd45gip1 | 2067 | -0.712 | -0.0648 | No |
| 132 | Usp20 | 2135 | -0.726 | -0.0788 | No |
| 133 | Ring1 | 2149 | -0.729 | -0.0787 | No |
| 134 | Iqcc | 2156 | -0.731 | -0.0769 | No |
| 135 | Epg5 | 2162 | -0.732 | -0.0747 | No |
| 136 | Trrap | 2184 | -0.735 | -0.0767 | No |
| 137 | Nfkbil1 | 2190 | -0.737 | -0.0746 | No |
| 138 | Usp31 | 2199 | -0.738 | -0.0732 | No |
| 139 | Pcyt2 | 2237 | -0.748 | -0.0793 | No |
| 140 | Acaa2 | 2240 | -0.748 | -0.0763 | No |
| 141 | Spr | 2241 | -0.749 | -0.0727 | No |
| 142 | Kpna4 | 2243 | -0.749 | -0.0695 | No |
| 143 | Taco1 | 2272 | -0.755 | -0.0732 | No |
| 144 | Pnpla6 | 2285 | -0.759 | -0.0728 | No |
| 145 | Eif2b4 | 2292 | -0.760 | -0.0707 | No |
| 146 | Sco2 | 2317 | -0.767 | -0.0734 | No |
| 147 | Pik3r4 | 2327 | -0.769 | -0.0721 | No |
| 148 | Hook2 | 2334 | -0.771 | -0.0700 | No |
| 149 | Acp6 | 2444 | -0.802 | -0.0946 | No |
| 150 | Hadh | 2449 | -0.804 | -0.0918 | No |
| 151 | Ptgis | 2464 | -0.808 | -0.0917 | No |
| 152 | Gramd4 | 2471 | -0.812 | -0.0894 | No |
| 153 | Prrc1 | 2513 | -0.822 | -0.0962 | No |
| 154 | Rmdn1 | 2543 | -0.829 | -0.0998 | No |
| 155 | Zfp692 | 2553 | -0.834 | -0.0982 | No |
| 156 | Dynll2 | 2574 | -0.841 | -0.0995 | No |
| 157 | Hdac5 | 2601 | -0.848 | -0.1022 | No |
| 158 | Tmc7 | 2604 | -0.850 | -0.0988 | No |
| 159 | Sap130 | 2627 | -0.856 | -0.1005 | No |
| 160 | Syt7 | 2642 | -0.859 | -0.1001 | No |
| 161 | Elp2 | 2683 | -0.870 | -0.1064 | No |
| 162 | Srcin1 | 2687 | -0.871 | -0.1031 | No |
| 163 | Pcgf2 | 2698 | -0.876 | -0.1015 | No |
| 164 | L3mbtl2 | 2721 | -0.882 | -0.1031 | No |
| 165 | Them6 | 2731 | -0.885 | -0.1013 | No |
| 166 | Dnajc1 | 2741 | -0.887 | -0.0995 | No |
| 167 | Armc9 | 2750 | -0.889 | -0.0974 | No |
| 168 | Aldh5a1 | 2751 | -0.889 | -0.0932 | No |
| 169 | Per1 | 2753 | -0.890 | -0.0892 | No |
| 170 | Tmem39b | 2771 | -0.896 | -0.0895 | No |
| 171 | Rbm7 | 2777 | -0.899 | -0.0865 | No |
| 172 | Senp6 | 2781 | -0.900 | -0.0831 | No |
| 173 | Adgrb1 | 2796 | -0.905 | -0.0825 | No |
| 174 | Sidt1 | 2815 | -0.910 | -0.0829 | No |
| 175 | Zranb1 | 2852 | -0.925 | -0.0879 | No |
| 176 | Nr2c1 | 2869 | -0.931 | -0.0876 | No |
| 177 | Nln | 2922 | -0.946 | -0.0967 | No |
| 178 | Bri3 | 2942 | -0.954 | -0.0971 | No |
| 179 | Polr3e | 2997 | -0.975 | -0.1066 | Yes |
| 180 | Mettl1 | 2999 | -0.975 | -0.1023 | Yes |
| 181 | Ube2o | 3001 | -0.975 | -0.0979 | Yes |
| 182 | Bahcc1 | 3014 | -0.981 | -0.0964 | Yes |
| 183 | Lcmt2 | 3019 | -0.982 | -0.0929 | Yes |
| 184 | Prmt3 | 3044 | -0.991 | -0.0944 | Yes |
| 185 | Maz | 3048 | -0.994 | -0.0906 | Yes |
| 186 | Vps26c | 3049 | -0.994 | -0.0859 | Yes |
| 187 | G2e3 | 3076 | -1.009 | -0.0879 | Yes |
| 188 | Dusp23 | 3101 | -1.019 | -0.0893 | Yes |
| 189 | Mfn1 | 3146 | -1.037 | -0.0959 | Yes |
| 190 | 1700109H08Rik | 3156 | -1.040 | -0.0933 | Yes |
| 191 | Rdh13 | 3200 | -1.066 | -0.0995 | Yes |
| 192 | Ncmap | 3204 | -1.068 | -0.0953 | Yes |
| 193 | Spint2 | 3222 | -1.076 | -0.0946 | Yes |
| 194 | Akr1e1 | 3230 | -1.081 | -0.0914 | Yes |
| 195 | Sorbs3 | 3256 | -1.096 | -0.0927 | Yes |
| 196 | Chd3 | 3263 | -1.100 | -0.0891 | Yes |
| 197 | C1qtnf4 | 3315 | -1.118 | -0.0971 | Yes |
| 198 | Fcgrt | 3345 | -1.138 | -0.0993 | Yes |
| 199 | Taf7 | 3351 | -1.140 | -0.0952 | Yes |
| 200 | Ppcs | 3352 | -1.140 | -0.0898 | Yes |
| 201 | Stard10 | 3354 | -1.141 | -0.0847 | Yes |
| 202 | Zfp324 | 3377 | -1.149 | -0.0850 | Yes |
| 203 | Vps13d | 3390 | -1.158 | -0.0827 | Yes |
| 204 | Kctd17 | 3394 | -1.159 | -0.0781 | Yes |
| 205 | Polr2i | 3409 | -1.163 | -0.0762 | Yes |
| 206 | Kif21b | 3440 | -1.180 | -0.0785 | Yes |
| 207 | Mapk8ip1 | 3443 | -1.182 | -0.0734 | Yes |
| 208 | Zfp787 | 3490 | -1.205 | -0.0797 | Yes |
| 209 | C2cd3 | 3494 | -1.208 | -0.0748 | Yes |
| 210 | Elac1 | 3523 | -1.227 | -0.0763 | Yes |
| 211 | Kansl1l | 3544 | -1.243 | -0.0757 | Yes |
| 212 | Kiz | 3586 | -1.278 | -0.0803 | Yes |
| 213 | Slc25a35 | 3609 | -1.297 | -0.0799 | Yes |
| 214 | Ulk2 | 3612 | -1.297 | -0.0744 | Yes |
| 215 | Med19 | 3614 | -1.297 | -0.0685 | Yes |
| 216 | Cul9 | 3655 | -1.343 | -0.0726 | Yes |
| 217 | Trim68 | 3664 | -1.349 | -0.0683 | Yes |
| 218 | Zc3h6 | 3679 | -1.362 | -0.0656 | Yes |
| 219 | Adh1 | 3681 | -1.362 | -0.0594 | Yes |
| 220 | Pycr1 | 3697 | -1.381 | -0.0568 | Yes |
| 221 | Zfp771 | 3709 | -1.390 | -0.0532 | Yes |
| 222 | Epb41l4b | 3716 | -1.399 | -0.0481 | Yes |
| 223 | Dbp | 3719 | -1.400 | -0.0421 | Yes |
| 224 | Slc25a42 | 3748 | -1.439 | -0.0426 | Yes |
| 225 | Gpatch1 | 3760 | -1.449 | -0.0386 | Yes |
| 226 | Qsox1 | 3787 | -1.486 | -0.0384 | Yes |
| 227 | Chka | 3801 | -1.515 | -0.0347 | Yes |
| 228 | Lmo4 | 3802 | -1.515 | -0.0275 | Yes |
| 229 | Ppp1r1b | 3805 | -1.518 | -0.0209 | Yes |
| 230 | Tmem67 | 3815 | -1.535 | -0.0161 | Yes |
| 231 | B9d2 | 3833 | -1.575 | -0.0131 | Yes |
| 232 | Sec14l1 | 3846 | -1.601 | -0.0087 | Yes |
| 233 | Cracr2b | 3867 | -1.641 | -0.0061 | Yes |
| 234 | Cep95 | 3956 | -1.843 | -0.0203 | Yes |
| 235 | Fam161a | 3964 | -1.898 | -0.0132 | Yes |
| 236 | Tppp3 | 4001 | -2.112 | -0.0127 | Yes |
| 237 | Cd177 | 4034 | -2.310 | -0.0101 | Yes |
| 238 | Hmgcs2 | 4048 | -2.436 | -0.0020 | Yes |
| 239 | Krt15 | 4063 | -2.644 | 0.0068 | Yes |
Table: GSEA details [plain text format]

  

Fig 2: TABULA\_MURIS\_SENIS\_BRAIN\_NON\_MYELOID\_NEURON\_AGEING: Random ES distribution      
 Gene set null distribution of ES for **TABULA\_MURIS\_SENIS\_BRAIN\_NON\_MYELOID\_NEURON\_AGEING**

  
