# Supplementary material for: TMPRSS11B promotes an acidified microenvironment and immune suppression in squamous lung cancer
Source: EMBO Rep. 2025 Nov 10;26(24):6346–79. doi: 10.1038/s44319-025-00631-1 (PMC12714794; doi:10.1038/s44319-025-00631-1)
Supplement: Supplementary file 18 — Figure EV6 Source Data [file 44319_2025_631_MOESM18_ESM.zip › Figure EV6/EV6C-D/GSEA_Broad Institute_M8_T11b high vs low LUSC/TABULA_MURIS_SENIS_GONADAL_ADIPOSE_TISSUE_MYELOID_CELL_AGEING.html]

Details for gene set TABULA\_MURIS\_SENIS\_GONADAL\_ADIPOSE\_TISSUE\_MYELOID\_CELL\_AGEING[GSEA]

|  || Dataset | T11b high vs low squamous\_GSEA\_Ranked |
| Phenotype | NoPhenotypeAvailable |
| Upregulated in class | na\_pos |
| GeneSet | TABULA\_MURIS\_SENIS\_GONADAL\_ADIPOSE\_TISSUE\_MYELOID\_CELL\_AGEING |
| Enrichment Score (ES) | 0.5067424 |
| Normalized Enrichment Score (NES) | 3.255468 |
| Nominal p-value | 0.0 |
| FDR q-value | 0.0 |
| FWER p-Value | 0.0 |
Table: GSEA Results Summary

  

Fig 1: Enrichment plot: TABULA\_MURIS\_SENIS\_GONADAL\_ADIPOSE\_TISSUE\_MYELOID\_CELL\_AGEING      
 Profile of the Running ES Score & Positions of GeneSet Members on the Rank Ordered List

  

| SYMBOL | RANK IN GENE LIST | RANK METRIC SCORE | RUNNING ES | CORE ENRICHMENT || 1 | S100a8 | 38 | 3.013 | 0.0215 | Yes |
| 2 | Krt14 | 56 | 2.659 | 0.0446 | Yes |
| 3 | S100a9 | 82 | 2.366 | 0.0627 | Yes |
| 4 | Apoe | 88 | 2.296 | 0.0851 | Yes |
| 5 | Ly6a | 92 | 2.274 | 0.1078 | Yes |
| 6 | Htra1 | 106 | 2.122 | 0.1264 | Yes |
| 7 | Il1b | 129 | 1.912 | 0.1406 | Yes |
| 8 | Ctsz | 138 | 1.884 | 0.1580 | Yes |
| 9 | Fth1 | 147 | 1.835 | 0.1749 | Yes |
| 10 | Fxyd5 | 157 | 1.767 | 0.1908 | Yes |
| 11 | Spi1 | 158 | 1.765 | 0.2090 | Yes |
| 12 | Gpsm3 | 169 | 1.725 | 0.2242 | Yes |
| 13 | Ctsb | 177 | 1.695 | 0.2400 | Yes |
| 14 | Crlf2 | 182 | 1.670 | 0.2561 | Yes |
| 15 | Csf2ra | 185 | 1.657 | 0.2727 | Yes |
| 16 | Gja1 | 186 | 1.653 | 0.2897 | Yes |
| 17 | Cdkn1a | 192 | 1.625 | 0.3052 | Yes |
| 18 | Csf2rb | 243 | 1.458 | 0.3077 | Yes |
| 19 | Pim1 | 262 | 1.425 | 0.3179 | Yes |
| 20 | Apbb1ip | 264 | 1.423 | 0.3323 | Yes |
| 21 | Ckb | 267 | 1.414 | 0.3463 | Yes |
| 22 | Hilpda | 302 | 1.302 | 0.3512 | Yes |
| 23 | Ehd1 | 313 | 1.237 | 0.3615 | Yes |
| 24 | Fmnl1 | 345 | 1.153 | 0.3656 | Yes |
| 25 | Lgals3 | 377 | 1.096 | 0.3691 | Yes |
| 26 | Col3a1 | 383 | 1.092 | 0.3791 | Yes |
| 27 | Col6a1 | 415 | 1.025 | 0.3819 | Yes |
| 28 | Ifitm2 | 465 | 0.952 | 0.3794 | Yes |
| 29 | Col6a2 | 479 | 0.927 | 0.3857 | Yes |
| 30 | Col1a1 | 483 | 0.921 | 0.3945 | Yes |
| 31 | Plpp3 | 490 | 0.910 | 0.4023 | Yes |
| 32 | Hspb1 | 500 | 0.898 | 0.4093 | Yes |
| 33 | Hcls1 | 503 | 0.897 | 0.4181 | Yes |
| 34 | Prelid1 | 506 | 0.892 | 0.4267 | Yes |
| 35 | Fstl1 | 507 | 0.892 | 0.4359 | Yes |
| 36 | Rcn1 | 518 | 0.877 | 0.4425 | Yes |
| 37 | Cyba | 519 | 0.875 | 0.4515 | Yes |
| 38 | Gadd45b | 522 | 0.873 | 0.4599 | Yes |
| 39 | Dmkn | 566 | 0.830 | 0.4577 | Yes |
| 40 | Fbn1 | 569 | 0.830 | 0.4658 | Yes |
| 41 | Dusp3 | 634 | 0.725 | 0.4572 | Yes |
| 42 | Cotl1 | 656 | 0.709 | 0.4593 | Yes |
| 43 | Ece1 | 701 | 0.665 | 0.4551 | Yes |
| 44 | Krt5 | 709 | 0.661 | 0.4602 | Yes |
| 45 | Igfbp7 | 711 | 0.661 | 0.4667 | Yes |
| 46 | Myl12a | 713 | 0.657 | 0.4733 | Yes |
| 47 | H2-D1 | 719 | 0.654 | 0.4787 | Yes |
| 48 | Col1a2 | 729 | 0.645 | 0.4831 | Yes |
| 49 | Serping1 | 730 | 0.645 | 0.4898 | Yes |
| 50 | Arrb2 | 786 | 0.600 | 0.4822 | Yes |
| 51 | Sparc | 792 | 0.595 | 0.4871 | Yes |
| 52 | Arpc4 | 798 | 0.593 | 0.4919 | Yes |
| 53 | Pkm | 807 | 0.591 | 0.4960 | Yes |
| 54 | Rexo2 | 839 | 0.571 | 0.4941 | Yes |
| 55 | Dtnbp1 | 842 | 0.569 | 0.4995 | Yes |
| 56 | H2-K1 | 855 | 0.565 | 0.5023 | Yes |
| 57 | Cfl1 | 895 | 0.538 | 0.4981 | Yes |
| 58 | Dpysl2 | 910 | 0.527 | 0.5000 | Yes |
| 59 | H2-Ab1 | 915 | 0.525 | 0.5044 | Yes |
| 60 | Tacstd2 | 937 | 0.512 | 0.5044 | Yes |
| 61 | Nfkbia | 954 | 0.502 | 0.5056 | Yes |
| 62 | Limd2 | 971 | -0.501 | 0.5067 | Yes |
| 63 | Dhrs4 | 1226 | -0.545 | 0.4488 | No |
| 64 | Emc10 | 1234 | -0.546 | 0.4527 | No |
| 65 | Eif3k | 1325 | -0.562 | 0.4360 | No |
| 66 | Ezr | 1361 | -0.567 | 0.4331 | No |
| 67 | Tbcb | 1546 | -0.602 | 0.3933 | No |
| 68 | Cmip | 1555 | -0.603 | 0.3975 | No |
| 69 | Etfb | 1565 | -0.605 | 0.4014 | No |
| 70 | Tmed9 | 1675 | -0.626 | 0.3806 | No |
| 71 | Smim14 | 1822 | -0.654 | 0.3509 | No |
| 72 | Reep5 | 1833 | -0.657 | 0.3551 | No |
| 73 | Bcl7c | 1842 | -0.659 | 0.3599 | No |
| 74 | Mospd3 | 2046 | -0.703 | 0.3164 | No |
| 75 | Gpc3 | 2218 | -0.743 | 0.2813 | No |
| 76 | Spr | 2241 | -0.749 | 0.2835 | No |
| 77 | Kpna4 | 2243 | -0.749 | 0.2909 | No |
| 78 | Zfp703 | 2342 | -0.773 | 0.2744 | No |
| 79 | Kmt2b | 2419 | -0.795 | 0.2636 | No |
| 80 | Psmc4 | 2525 | -0.826 | 0.2458 | No |
| 81 | Bsg | 2530 | -0.827 | 0.2533 | No |
| 82 | Spag7 | 2645 | -0.860 | 0.2337 | No |
| 83 | Eif3f | 2655 | -0.863 | 0.2403 | No |
| 84 | Shisa5 | 2916 | -0.946 | 0.1850 | No |
| 85 | Commd10 | 2934 | -0.952 | 0.1906 | No |
| 86 | Bri3 | 2942 | -0.954 | 0.1986 | No |
| 87 | Clu | 3405 | -1.162 | 0.0951 | No |
| 88 | Inmt | 3783 | -1.479 | 0.0160 | No |
| 89 | Lmo4 | 3802 | -1.515 | 0.0271 | No |
| 90 | Clec3b | 3868 | -1.644 | 0.0278 | No |
| 91 | Krt15 | 4063 | -2.644 | 0.0065 | No |
Table: GSEA details [plain text format]

  

Fig 2: TABULA\_MURIS\_SENIS\_GONADAL\_ADIPOSE\_TISSUE\_MYELOID\_CELL\_AGEING: Random ES distribution      
 Gene set null distribution of ES for **TABULA\_MURIS\_SENIS\_GONADAL\_ADIPOSE\_TISSUE\_MYELOID\_CELL\_AGEING**

  
