# Supplementary material for: TMPRSS11B promotes an acidified microenvironment and immune suppression in squamous lung cancer
Source: EMBO Rep. 2025 Nov 10;26(24):6346–79. doi: 10.1038/s44319-025-00631-1 (PMC12714794; doi:10.1038/s44319-025-00631-1)
Supplement: Supplementary file 18 — Figure EV6 Source Data [file 44319_2025_631_MOESM18_ESM.zip › Figure EV6/EV6C-D/GSEA_Broad Institute_M8_T11b high vs low LUSC/TABULA_MURIS_SENIS_HEART_AND_AORTA_LEUKOCYTE_AGEING.html]

Details for gene set TABULA\_MURIS\_SENIS\_HEART\_AND\_AORTA\_LEUKOCYTE\_AGEING[GSEA]

|  || Dataset | T11b high vs low squamous\_GSEA\_Ranked |
| Phenotype | NoPhenotypeAvailable |
| Upregulated in class | na\_pos |
| GeneSet | TABULA\_MURIS\_SENIS\_HEART\_AND\_AORTA\_LEUKOCYTE\_AGEING |
| Enrichment Score (ES) | 0.75374496 |
| Normalized Enrichment Score (NES) | 3.557271 |
| Nominal p-value | 0.0 |
| FDR q-value | 0.0 |
| FWER p-Value | 0.0 |
Table: GSEA Results Summary

  

Fig 1: Enrichment plot: TABULA\_MURIS\_SENIS\_HEART\_AND\_AORTA\_LEUKOCYTE\_AGEING      
 Profile of the Running ES Score & Positions of GeneSet Members on the Rank Ordered List

  

| SYMBOL | RANK IN GENE LIST | RANK METRIC SCORE | RUNNING ES | CORE ENRICHMENT || 1 | Ecm1 | 31 | 3.325 | 0.0615 | Yes |
| 2 | Clec4d | 39 | 3.005 | 0.1222 | Yes |
| 3 | Fcgr2b | 62 | 2.610 | 0.1710 | Yes |
| 4 | Itgb2 | 71 | 2.481 | 0.2207 | Yes |
| 5 | Ly6a | 92 | 2.274 | 0.2630 | Yes |
| 6 | Plek | 95 | 2.237 | 0.3090 | Yes |
| 7 | Mpeg1 | 114 | 2.045 | 0.3471 | Yes |
| 8 | Wfdc17 | 117 | 1.998 | 0.3881 | Yes |
| 9 | Il1b | 129 | 1.912 | 0.4252 | Yes |
| 10 | Pirb | 137 | 1.894 | 0.4628 | Yes |
| 11 | Ccl6 | 166 | 1.733 | 0.4919 | Yes |
| 12 | Sirpb1c | 170 | 1.719 | 0.5269 | Yes |
| 13 | Acp5 | 247 | 1.450 | 0.5383 | Yes |
| 14 | Pim1 | 262 | 1.425 | 0.5645 | Yes |
| 15 | Srgn | 270 | 1.392 | 0.5917 | Yes |
| 16 | Anxa1 | 290 | 1.344 | 0.6150 | Yes |
| 17 | Xdh | 367 | 1.116 | 0.6194 | Yes |
| 18 | Lgals3 | 377 | 1.096 | 0.6400 | Yes |
| 19 | Tnfaip2 | 387 | 1.086 | 0.6604 | Yes |
| 20 | Metrnl | 393 | 1.071 | 0.6814 | Yes |
| 21 | Nupr1 | 443 | 0.988 | 0.6898 | Yes |
| 22 | Ifitm2 | 465 | 0.952 | 0.7044 | Yes |
| 23 | Msrb1 | 472 | 0.945 | 0.7226 | Yes |
| 24 | Ier5 | 493 | 0.908 | 0.7365 | Yes |
| 25 | Hspb1 | 500 | 0.898 | 0.7537 | Yes |
| 26 | Fabp4 | 657 | 0.708 | 0.7300 | No |
| 27 | Klf4 | 662 | 0.701 | 0.7436 | No |
| 28 | Igfbp7 | 711 | 0.661 | 0.7455 | No |
| 29 | Mcl1 | 950 | 0.505 | 0.6973 | No |
| 30 | Synj1 | 1039 | -0.512 | 0.6863 | No |
| 31 | Cox7a2l | 1218 | -0.543 | 0.6537 | No |
| 32 | Fos | 3189 | -1.058 | 0.1901 | No |
| 33 | Lmo4 | 3802 | -1.515 | 0.0707 | No |
Table: GSEA details [plain text format]

  

Fig 2: TABULA\_MURIS\_SENIS\_HEART\_AND\_AORTA\_LEUKOCYTE\_AGEING: Random ES distribution      
 Gene set null distribution of ES for **TABULA\_MURIS\_SENIS\_HEART\_AND\_AORTA\_LEUKOCYTE\_AGEING**

  
