# Supplementary material for: TMPRSS11B promotes an acidified microenvironment and immune suppression in squamous lung cancer
Source: EMBO Rep. 2025 Nov 10;26(24):6346–79. doi: 10.1038/s44319-025-00631-1 (PMC12714794; doi:10.1038/s44319-025-00631-1)
Supplement: Supplementary file 18 — Figure EV6 Source Data [file 44319_2025_631_MOESM18_ESM.zip › Figure EV6/EV6C-D/GSEA_Broad Institute_M8_T11b high vs low LUSC/TABULA_MURIS_SENIS_HEART_VENTRICULAR_MYOCYTE_AGEING.html]

Details for gene set TABULA\_MURIS\_SENIS\_HEART\_VENTRICULAR\_MYOCYTE\_AGEING[GSEA]

|  || Dataset | T11b high vs low squamous\_GSEA\_Ranked |
| Phenotype | NoPhenotypeAvailable |
| Upregulated in class | na\_neg |
| GeneSet | TABULA\_MURIS\_SENIS\_HEART\_VENTRICULAR\_MYOCYTE\_AGEING |
| Enrichment Score (ES) | -0.29656497 |
| Normalized Enrichment Score (NES) | -1.3763902 |
| Nominal p-value | 0.096283786 |
| FDR q-value | 0.31984892 |
| FWER p-Value | 1.0 |
Table: GSEA Results Summary

  

Fig 1: Enrichment plot: TABULA\_MURIS\_SENIS\_HEART\_VENTRICULAR\_MYOCYTE\_AGEING      
 Profile of the Running ES Score & Positions of GeneSet Members on the Rank Ordered List

  

| SYMBOL | RANK IN GENE LIST | RANK METRIC SCORE | RUNNING ES | CORE ENRICHMENT || 1 | Psap | 240 | 1.466 | -0.0171 | No |
| 2 | Smtn | 412 | 1.030 | -0.0298 | No |
| 3 | Ehd4 | 474 | 0.943 | -0.0177 | No |
| 4 | Trf | 601 | 0.771 | -0.0267 | No |
| 5 | Map7d1 | 660 | 0.704 | -0.0208 | No |
| 6 | H2-D1 | 719 | 0.654 | -0.0164 | No |
| 7 | Pkm | 807 | 0.591 | -0.0209 | No |
| 8 | H2-K1 | 855 | 0.565 | -0.0163 | No |
| 9 | Emc10 | 1234 | -0.546 | -0.0939 | No |
| 10 | Amfr | 1358 | -0.567 | -0.1079 | No |
| 11 | Maged1 | 1391 | -0.573 | -0.0994 | No |
| 12 | Gaa | 1539 | -0.600 | -0.1184 | No |
| 13 | Tmed9 | 1675 | -0.626 | -0.1338 | No |
| 14 | Med25 | 1787 | -0.646 | -0.1426 | No |
| 15 | Psmd1 | 1913 | -0.677 | -0.1540 | No |
| 16 | Nectin2 | 1952 | -0.686 | -0.1438 | No |
| 17 | Prpf19 | 2056 | -0.709 | -0.1488 | No |
| 18 | Sec23ip | 2061 | -0.712 | -0.1294 | No |
| 19 | Kdelr1 | 2186 | -0.736 | -0.1389 | No |
| 20 | Spr | 2241 | -0.749 | -0.1307 | No |
| 21 | Bckdha | 2457 | -0.806 | -0.1606 | No |
| 22 | Ric8a | 2859 | -0.928 | -0.2329 | No |
| 23 | Mtus2 | 2911 | -0.944 | -0.2184 | No |
| 24 | Lamb2 | 3229 | -1.081 | -0.2656 | Yes |
| 25 | Dnaja4 | 3313 | -1.117 | -0.2540 | Yes |
| 26 | Btbd1 | 3397 | -1.161 | -0.2412 | Yes |
| 27 | Clu | 3405 | -1.162 | -0.2096 | Yes |
| 28 | Aldh2 | 3521 | -1.226 | -0.2028 | Yes |
| 29 | Dkk3 | 3542 | -1.238 | -0.1723 | Yes |
| 30 | Ptov1 | 3741 | -1.430 | -0.1801 | Yes |
| 31 | Pam | 3917 | -1.755 | -0.1729 | Yes |
| 32 | Oat | 3935 | -1.777 | -0.1262 | Yes |
| 33 | Tppp3 | 4001 | -2.112 | -0.0817 | Yes |
| 34 | Ces1d | 4086 | -3.597 | 0.0007 | Yes |
Table: GSEA details [plain text format]

  

Fig 2: TABULA\_MURIS\_SENIS\_HEART\_VENTRICULAR\_MYOCYTE\_AGEING: Random ES distribution      
 Gene set null distribution of ES for **TABULA\_MURIS\_SENIS\_HEART\_VENTRICULAR\_MYOCYTE\_AGEING**

  
