# Supplementary material for: TMPRSS11B promotes an acidified microenvironment and immune suppression in squamous lung cancer
Source: EMBO Rep. 2025 Nov 10;26(24):6346–79. doi: 10.1038/s44319-025-00631-1 (PMC12714794; doi:10.1038/s44319-025-00631-1)
Supplement: Supplementary file 18 — Figure EV6 Source Data [file 44319_2025_631_MOESM18_ESM.zip › Figure EV6/EV6C-D/GSEA_Broad Institute_M8_T11b high vs low LUSC/TABULA_MURIS_SENIS_KIDNEY_EPITHELIAL_CELL_OF_PROXIMAL_TUBULE_AGEING.html]

Details for gene set TABULA\_MURIS\_SENIS\_KIDNEY\_EPITHELIAL\_CELL\_OF\_PROXIMAL\_TUBULE\_AGEING[GSEA]

|  || Dataset | T11b high vs low squamous\_GSEA\_Ranked |
| Phenotype | NoPhenotypeAvailable |
| Upregulated in class | na\_neg |
| GeneSet | TABULA\_MURIS\_SENIS\_KIDNEY\_EPITHELIAL\_CELL\_OF\_PROXIMAL\_TUBULE\_AGEING |
| Enrichment Score (ES) | -0.09272604 |
| Normalized Enrichment Score (NES) | -0.53851146 |
| Nominal p-value | 0.9792 |
| FDR q-value | 1.0 |
| FWER p-Value | 1.0 |
Table: GSEA Results Summary

  

Fig 1: Enrichment plot: TABULA\_MURIS\_SENIS\_KIDNEY\_EPITHELIAL\_CELL\_OF\_PROXIMAL\_TUBULE\_AGEING      
 Profile of the Running ES Score & Positions of GeneSet Members on the Rank Ordered List

  

| SYMBOL | RANK IN GENE LIST | RANK METRIC SCORE | RUNNING ES | CORE ENRICHMENT || 1 | S100a10 | 66 | 2.555 | 0.0169 | No |
| 2 | Apoe | 88 | 2.296 | 0.0417 | No |
| 3 | Fth1 | 147 | 1.835 | 0.0512 | No |
| 4 | Scd1 | 152 | 1.815 | 0.0739 | No |
| 5 | Npl | 268 | 1.398 | 0.0635 | No |
| 6 | Cd302 | 346 | 1.152 | 0.0593 | No |
| 7 | Bnip3 | 365 | 1.118 | 0.0694 | No |
| 8 | Tmem37 | 535 | 0.861 | 0.0385 | No |
| 9 | Txndc17 | 579 | 0.812 | 0.0384 | No |
| 10 | Txn1 | 586 | 0.800 | 0.0473 | No |
| 11 | Igfbp7 | 711 | 0.661 | 0.0250 | No |
| 12 | Adk | 752 | 0.626 | 0.0232 | No |
| 13 | Sat1 | 817 | 0.584 | 0.0148 | No |
| 14 | Cd74 | 849 | 0.567 | 0.0145 | No |
| 15 | B2m | 860 | 0.563 | 0.0194 | No |
| 16 | Mgll | 894 | 0.540 | 0.0182 | No |
| 17 | Fam162a | 909 | 0.527 | 0.0216 | No |
| 18 | Echs1 | 962 | -0.501 | 0.0152 | No |
| 19 | Eef1d | 991 | -0.504 | 0.0147 | No |
| 20 | Tmem205 | 1023 | -0.509 | 0.0137 | No |
| 21 | Ssr4 | 1035 | -0.511 | 0.0176 | No |
| 22 | Sri | 1116 | -0.525 | 0.0045 | No |
| 23 | Tmem59 | 1117 | -0.525 | 0.0113 | No |
| 24 | 2310039H08Rik | 1194 | -0.538 | -0.0006 | No |
| 25 | Cox7a2l | 1218 | -0.543 | 0.0008 | No |
| 26 | Dhrs4 | 1226 | -0.545 | 0.0061 | No |
| 27 | Iah1 | 1294 | -0.554 | -0.0034 | No |
| 28 | Eif3k | 1325 | -0.562 | -0.0035 | No |
| 29 | Sephs2 | 1327 | -0.563 | 0.0036 | No |
| 30 | Guk1 | 1444 | -0.584 | -0.0177 | No |
| 31 | Hsd17b10 | 1448 | -0.584 | -0.0108 | No |
| 32 | Tst | 1483 | -0.590 | -0.0116 | No |
| 33 | Ccdc107 | 1556 | -0.604 | -0.0217 | No |
| 34 | Gm5617 | 1594 | -0.610 | -0.0230 | No |
| 35 | Nop10 | 1642 | -0.619 | -0.0266 | No |
| 36 | Mpst | 1677 | -0.626 | -0.0269 | No |
| 37 | Bola3 | 1689 | -0.628 | -0.0215 | No |
| 38 | Gemin7 | 1752 | -0.640 | -0.0286 | No |
| 39 | Ifi27 | 1778 | -0.644 | -0.0264 | No |
| 40 | Bphl | 1815 | -0.651 | -0.0269 | No |
| 41 | Grcc10 | 1882 | -0.670 | -0.0346 | No |
| 42 | Ppa2 | 1908 | -0.675 | -0.0320 | No |
| 43 | Krtcap2 | 1937 | -0.682 | -0.0301 | No |
| 44 | Camk2n1 | 1967 | -0.688 | -0.0284 | No |
| 45 | Gstm1 | 2049 | -0.704 | -0.0394 | No |
| 46 | Tmco1 | 2136 | -0.726 | -0.0514 | No |
| 47 | Vkorc1 | 2191 | -0.737 | -0.0552 | No |
| 48 | Acaa2 | 2240 | -0.748 | -0.0574 | No |
| 49 | Srsf3 | 2381 | -0.784 | -0.0821 | No |
| 50 | Chchd7 | 2409 | -0.793 | -0.0785 | No |
| 51 | Gstz1 | 2467 | -0.810 | -0.0821 | Yes |
| 52 | Ascc1 | 2493 | -0.818 | -0.0777 | Yes |
| 53 | Hmgb1 | 2518 | -0.823 | -0.0729 | Yes |
| 54 | Sgk1 | 2539 | -0.829 | -0.0671 | Yes |
| 55 | Eci1 | 2554 | -0.834 | -0.0597 | Yes |
| 56 | Fbp2 | 2577 | -0.842 | -0.0542 | Yes |
| 57 | Hcfc1r1 | 2602 | -0.848 | -0.0491 | Yes |
| 58 | Acsl1 | 2609 | -0.850 | -0.0395 | Yes |
| 59 | Eif3f | 2655 | -0.863 | -0.0394 | Yes |
| 60 | Ddt | 2681 | -0.869 | -0.0343 | Yes |
| 61 | Sult1c2 | 2723 | -0.883 | -0.0330 | Yes |
| 62 | Atraid | 2738 | -0.886 | -0.0249 | Yes |
| 63 | Tmem147 | 2787 | -0.902 | -0.0251 | Yes |
| 64 | Cryz | 2889 | -0.936 | -0.0381 | Yes |
| 65 | Akr7a5 | 2897 | -0.939 | -0.0276 | Yes |
| 66 | Krcc1 | 2951 | -0.956 | -0.0283 | Yes |
| 67 | Fmc1 | 3009 | -0.979 | -0.0298 | Yes |
| 68 | Cyb5a | 3121 | -1.027 | -0.0440 | Yes |
| 69 | Aldh9a1 | 3166 | -1.046 | -0.0414 | Yes |
| 70 | Ggact | 3205 | -1.068 | -0.0369 | Yes |
| 71 | Tcea3 | 3242 | -1.088 | -0.0317 | Yes |
| 72 | Ass1 | 3295 | -1.111 | -0.0301 | Yes |
| 73 | Ccnd1 | 3303 | -1.112 | -0.0173 | Yes |
| 74 | Npnt | 3367 | -1.146 | -0.0181 | Yes |
| 75 | Gsta3 | 3465 | -1.196 | -0.0267 | Yes |
| 76 | Dnajc12 | 3489 | -1.204 | -0.0167 | Yes |
| 77 | Aldh2 | 3521 | -1.226 | -0.0084 | Yes |
| 78 | Cyp4b1 | 3549 | -1.246 | 0.0011 | Yes |
| 79 | Pts | 3587 | -1.278 | 0.0086 | Yes |
| 80 | Cela1 | 3666 | -1.349 | 0.0068 | Yes |
| 81 | Tstd1 | 3733 | -1.423 | 0.0089 | Yes |
| 82 | Rida | 3822 | -1.553 | 0.0072 | Yes |
| 83 | Wfdc2 | 3939 | -1.799 | 0.0018 | Yes |
| 84 | Cyp2a4 | 4068 | -2.712 | 0.0052 | Yes |
Table: GSEA details [plain text format]

  

Fig 2: TABULA\_MURIS\_SENIS\_KIDNEY\_EPITHELIAL\_CELL\_OF\_PROXIMAL\_TUBULE\_AGEING: Random ES distribution      
 Gene set null distribution of ES for **TABULA\_MURIS\_SENIS\_KIDNEY\_EPITHELIAL\_CELL\_OF\_PROXIMAL\_TUBULE\_AGEING**

  
