# Supplementary material for: TMPRSS11B promotes an acidified microenvironment and immune suppression in squamous lung cancer
Source: EMBO Rep. 2025 Nov 10;26(24):6346–79. doi: 10.1038/s44319-025-00631-1 (PMC12714794; doi:10.1038/s44319-025-00631-1)
Supplement: Supplementary file 18 — Figure EV6 Source Data [file 44319_2025_631_MOESM18_ESM.zip › Figure EV6/EV6C-D/GSEA_Broad Institute_M8_T11b high vs low LUSC/TABULA_MURIS_SENIS_KIDNEY_MACROPHAGE_AGEING.html]

Details for gene set TABULA\_MURIS\_SENIS\_KIDNEY\_MACROPHAGE\_AGEING[GSEA]

|  || Dataset | T11b high vs low squamous\_GSEA\_Ranked |
| Phenotype | NoPhenotypeAvailable |
| Upregulated in class | na\_pos |
| GeneSet | TABULA\_MURIS\_SENIS\_KIDNEY\_MACROPHAGE\_AGEING |
| Enrichment Score (ES) | 0.6699623 |
| Normalized Enrichment Score (NES) | 3.4811463 |
| Nominal p-value | 0.0 |
| FDR q-value | 0.0 |
| FWER p-Value | 0.0 |
Table: GSEA Results Summary

  

Fig 1: Enrichment plot: TABULA\_MURIS\_SENIS\_KIDNEY\_MACROPHAGE\_AGEING      
 Profile of the Running ES Score & Positions of GeneSet Members on the Rank Ordered List

  

| SYMBOL | RANK IN GENE LIST | RANK METRIC SCORE | RUNNING ES | CORE ENRICHMENT || 1 | Trem2 | 24 | 3.569 | 0.0531 | Yes |
| 2 | Cd84 | 45 | 2.807 | 0.0945 | Yes |
| 3 | Gngt2 | 51 | 2.724 | 0.1383 | Yes |
| 4 | Itgam | 61 | 2.631 | 0.1796 | Yes |
| 5 | Lyz1 | 73 | 2.460 | 0.2175 | Yes |
| 6 | Ly6a | 92 | 2.274 | 0.2507 | Yes |
| 7 | Ccl9 | 93 | 2.271 | 0.2882 | Yes |
| 8 | Pla2g7 | 94 | 2.250 | 0.3254 | Yes |
| 9 | Vim | 112 | 2.054 | 0.3552 | Yes |
| 10 | Emp3 | 115 | 2.020 | 0.3881 | Yes |
| 11 | Cd300a | 122 | 1.959 | 0.4190 | Yes |
| 12 | Ctsd | 150 | 1.830 | 0.4425 | Yes |
| 13 | Ccl6 | 166 | 1.733 | 0.4675 | Yes |
| 14 | Sirpb1c | 170 | 1.719 | 0.4952 | Yes |
| 15 | Cfp | 171 | 1.717 | 0.5235 | Yes |
| 16 | Acp5 | 247 | 1.450 | 0.5290 | Yes |
| 17 | Grn | 285 | 1.352 | 0.5422 | Yes |
| 18 | Capg | 343 | 1.160 | 0.5472 | Yes |
| 19 | Lgals3 | 377 | 1.096 | 0.5572 | Yes |
| 20 | Tnfaip2 | 387 | 1.086 | 0.5729 | Yes |
| 21 | Alox5ap | 399 | 1.051 | 0.5876 | Yes |
| 22 | Zeb2 | 420 | 1.020 | 0.5995 | Yes |
| 23 | Ifitm2 | 465 | 0.952 | 0.6044 | Yes |
| 24 | Msrb1 | 472 | 0.945 | 0.6185 | Yes |
| 25 | Cebpb | 504 | 0.897 | 0.6256 | Yes |
| 26 | Cyba | 519 | 0.875 | 0.6366 | Yes |
| 27 | Cd44 | 562 | 0.834 | 0.6400 | Yes |
| 28 | Txn1 | 586 | 0.800 | 0.6476 | Yes |
| 29 | Rap2b | 629 | 0.730 | 0.6493 | Yes |
| 30 | Pltp | 661 | 0.702 | 0.6532 | Yes |
| 31 | Klf4 | 662 | 0.701 | 0.6648 | Yes |
| 32 | Pgk1 | 717 | 0.655 | 0.6623 | Yes |
| 33 | Atp1b3 | 745 | 0.632 | 0.6661 | Yes |
| 34 | Ms4a7 | 771 | 0.610 | 0.6700 | Yes |
| 35 | Sat1 | 817 | 0.584 | 0.6685 | No |
| 36 | Mcl1 | 950 | 0.505 | 0.6442 | No |
| 37 | Ezr | 1361 | -0.567 | 0.5523 | No |
| 38 | Cmip | 1555 | -0.603 | 0.5145 | No |
| 39 | Reep5 | 1833 | -0.657 | 0.4569 | No |
| 40 | Ly6e | 1857 | -0.663 | 0.4622 | No |
| 41 | Fos | 3189 | -1.058 | 0.1507 | No |
| 42 | Aldh2 | 3521 | -1.226 | 0.0892 | No |
| 43 | Lmo4 | 3802 | -1.515 | 0.0450 | No |
| 44 | Rida | 3822 | -1.553 | 0.0660 | No |
Table: GSEA details [plain text format]

  

Fig 2: TABULA\_MURIS\_SENIS\_KIDNEY\_MACROPHAGE\_AGEING: Random ES distribution      
 Gene set null distribution of ES for **TABULA\_MURIS\_SENIS\_KIDNEY\_MACROPHAGE\_AGEING**

  
