# Supplementary material for: TMPRSS11B promotes an acidified microenvironment and immune suppression in squamous lung cancer
Source: EMBO Rep. 2025 Nov 10;26(24):6346–79. doi: 10.1038/s44319-025-00631-1 (PMC12714794; doi:10.1038/s44319-025-00631-1)
Supplement: Supplementary file 18 — Figure EV6 Source Data [file 44319_2025_631_MOESM18_ESM.zip › Figure EV6/EV6C-D/GSEA_Broad Institute_M8_T11b high vs low LUSC/TABULA_MURIS_SENIS_LARGE_INTESTINE_INTESTINAL_CRYPT_STEM_CELL_AGEING.html]

Details for gene set TABULA\_MURIS\_SENIS\_LARGE\_INTESTINE\_INTESTINAL\_CRYPT\_STEM\_CELL\_AGEING[GSEA]

|  || Dataset | T11b high vs low squamous\_GSEA\_Ranked |
| Phenotype | NoPhenotypeAvailable |
| Upregulated in class | na\_neg |
| GeneSet | TABULA\_MURIS\_SENIS\_LARGE\_INTESTINE\_INTESTINAL\_CRYPT\_STEM\_CELL\_AGEING |
| Enrichment Score (ES) | -0.106292546 |
| Normalized Enrichment Score (NES) | -0.63940006 |
| Nominal p-value | 0.9448819 |
| FDR q-value | 1.0 |
| FWER p-Value | 1.0 |
Table: GSEA Results Summary

  

Fig 1: Enrichment plot: TABULA\_MURIS\_SENIS\_LARGE\_INTESTINE\_INTESTINAL\_CRYPT\_STEM\_CELL\_AGEING      
 Profile of the Running ES Score & Positions of GeneSet Members on the Rank Ordered List

  

| SYMBOL | RANK IN GENE LIST | RANK METRIC SCORE | RUNNING ES | CORE ENRICHMENT || 1 | Ctsz | 138 | 1.884 | -0.0140 | No |
| 2 | Fth1 | 147 | 1.835 | 0.0041 | No |
| 3 | Mfge8 | 160 | 1.751 | 0.0202 | No |
| 4 | Marcksl1 | 221 | 1.520 | 0.0218 | No |
| 5 | S100a14 | 311 | 1.260 | 0.0133 | No |
| 6 | Mal | 329 | 1.191 | 0.0220 | No |
| 7 | Lypd3 | 389 | 1.080 | 0.0191 | No |
| 8 | Ifitm2 | 465 | 0.952 | 0.0107 | No |
| 9 | Cyba | 519 | 0.875 | 0.0070 | No |
| 10 | Gadd45b | 522 | 0.873 | 0.0160 | No |
| 11 | Rgs1 | 526 | 0.873 | 0.0248 | No |
| 12 | Trf | 601 | 0.771 | 0.0147 | No |
| 13 | Cd63 | 632 | 0.727 | 0.0151 | No |
| 14 | Cotl1 | 656 | 0.709 | 0.0171 | No |
| 15 | Ece1 | 701 | 0.665 | 0.0133 | No |
| 16 | H2-D1 | 719 | 0.654 | 0.0162 | No |
| 17 | Col1a2 | 729 | 0.645 | 0.0210 | No |
| 18 | S100a16 | 775 | 0.605 | 0.0164 | No |
| 19 | Arpc4 | 798 | 0.593 | 0.0173 | No |
| 20 | Pkm | 807 | 0.591 | 0.0218 | No |
| 21 | Rgcc | 822 | 0.579 | 0.0246 | No |
| 22 | Sfn | 834 | 0.573 | 0.0281 | No |
| 23 | H2-K1 | 855 | 0.565 | 0.0293 | No |
| 24 | Cfl1 | 895 | 0.538 | 0.0254 | No |
| 25 | Eef1d | 991 | -0.504 | 0.0071 | No |
| 26 | Elof1 | 1092 | -0.521 | -0.0123 | No |
| 27 | Nt5c | 1142 | -0.530 | -0.0188 | No |
| 28 | Mecr | 1153 | -0.530 | -0.0155 | No |
| 29 | Cox7a2l | 1218 | -0.543 | -0.0256 | No |
| 30 | Prrg2 | 1221 | -0.544 | -0.0202 | No |
| 31 | Dhrs4 | 1226 | -0.545 | -0.0152 | No |
| 32 | Trappc6a | 1231 | -0.545 | -0.0102 | No |
| 33 | Emc10 | 1234 | -0.546 | -0.0048 | No |
| 34 | Eif3k | 1325 | -0.562 | -0.0212 | No |
| 35 | Guk1 | 1444 | -0.584 | -0.0444 | No |
| 36 | Hsd17b10 | 1448 | -0.584 | -0.0388 | No |
| 37 | Fam241b | 1461 | -0.586 | -0.0354 | No |
| 38 | Tst | 1483 | -0.590 | -0.0342 | No |
| 39 | Cldn3 | 1495 | -0.593 | -0.0304 | No |
| 40 | Fam98c | 1529 | -0.599 | -0.0322 | No |
| 41 | Tbcb | 1546 | -0.602 | -0.0296 | No |
| 42 | Etfb | 1565 | -0.605 | -0.0275 | No |
| 43 | Tmed9 | 1675 | -0.626 | -0.0480 | No |
| 44 | Nt5c3b | 1697 | -0.630 | -0.0463 | No |
| 45 | Emg1 | 1738 | -0.637 | -0.0494 | No |
| 46 | 2610528J11Rik | 1763 | -0.642 | -0.0484 | No |
| 47 | Tmed4 | 1818 | -0.653 | -0.0548 | No |
| 48 | Bcl7c | 1842 | -0.659 | -0.0534 | No |
| 49 | Ly6e | 1857 | -0.663 | -0.0496 | No |
| 50 | Endog | 1948 | -0.685 | -0.0647 | No |
| 51 | Zmat5 | 2021 | -0.696 | -0.0751 | No |
| 52 | Vps72 | 2028 | -0.697 | -0.0690 | No |
| 53 | Mospd3 | 2046 | -0.703 | -0.0656 | No |
| 54 | Ppp4c | 2059 | -0.712 | -0.0608 | No |
| 55 | Gadd45gip1 | 2067 | -0.712 | -0.0548 | No |
| 56 | Cdpf1 | 2104 | -0.712 | -0.0560 | No |
| 57 | Kdelr1 | 2186 | -0.736 | -0.0683 | No |
| 58 | Cdc42ep5 | 2192 | -0.737 | -0.0615 | No |
| 59 | Spr | 2241 | -0.749 | -0.0653 | No |
| 60 | 2510002D24Rik | 2291 | -0.760 | -0.0693 | No |
| 61 | Fermt1 | 2395 | -0.789 | -0.0865 | No |
| 62 | Aarsd1 | 2475 | -0.813 | -0.0974 | Yes |
| 63 | Noxo1 | 2487 | -0.817 | -0.0912 | Yes |
| 64 | Hmgb1 | 2518 | -0.823 | -0.0897 | Yes |
| 65 | Lsr | 2522 | -0.825 | -0.0815 | Yes |
| 66 | Psmc4 | 2525 | -0.826 | -0.0729 | Yes |
| 67 | Bsg | 2530 | -0.827 | -0.0649 | Yes |
| 68 | Dynll2 | 2574 | -0.841 | -0.0665 | Yes |
| 69 | Fahd1 | 2607 | -0.850 | -0.0652 | Yes |
| 70 | Pmm1 | 2637 | -0.858 | -0.0631 | Yes |
| 71 | Eif3f | 2655 | -0.863 | -0.0579 | Yes |
| 72 | Dcps | 2660 | -0.864 | -0.0495 | Yes |
| 73 | Psmg3 | 2680 | -0.869 | -0.0447 | Yes |
| 74 | Tmed3 | 2696 | -0.874 | -0.0389 | Yes |
| 75 | Hes6 | 2755 | -0.892 | -0.0437 | Yes |
| 76 | Smco4 | 2881 | -0.934 | -0.0648 | Yes |
| 77 | Akr7a5 | 2897 | -0.939 | -0.0583 | Yes |
| 78 | Cdk5rap3 | 2899 | -0.939 | -0.0483 | Yes |
| 79 | Bri3 | 2942 | -0.954 | -0.0484 | Yes |
| 80 | Mettl26 | 2965 | -0.961 | -0.0434 | Yes |
| 81 | Rfc2 | 2975 | -0.965 | -0.0351 | Yes |
| 82 | Krtcap3 | 3028 | -0.985 | -0.0374 | Yes |
| 83 | Asl | 3098 | -1.016 | -0.0436 | Yes |
| 84 | Qtrt1 | 3185 | -1.056 | -0.0536 | Yes |
| 85 | Pafah1b3 | 3201 | -1.066 | -0.0457 | Yes |
| 86 | Nudt22 | 3202 | -1.066 | -0.0340 | Yes |
| 87 | Spint2 | 3222 | -1.076 | -0.0270 | Yes |
| 88 | Sgf29 | 3223 | -1.076 | -0.0152 | Yes |
| 89 | Osgep | 3226 | -1.080 | -0.0039 | Yes |
| 90 | Ptgr1 | 3237 | -1.085 | 0.0054 | Yes |
| 91 | Ccnd1 | 3303 | -1.112 | 0.0013 | Yes |
| 92 | Sf3b4 | 3336 | -1.127 | 0.0056 | Yes |
| 93 | Clu | 3405 | -1.162 | 0.0013 | Yes |
| 94 | Sdsl | 3518 | -1.220 | -0.0135 | Yes |
| 95 | Cela1 | 3666 | -1.349 | -0.0356 | Yes |
| 96 | Adh1 | 3681 | -1.362 | -0.0242 | Yes |
| 97 | Ptov1 | 3741 | -1.430 | -0.0233 | Yes |
| 98 | Rnf186 | 3789 | -1.490 | -0.0188 | Yes |
| 99 | Ppp1r1b | 3805 | -1.518 | -0.0060 | Yes |
| 100 | Bbc3 | 3841 | -1.590 | 0.0026 | Yes |
| 101 | Kcne3 | 4057 | -2.590 | -0.0229 | Yes |
| 102 | Krt20 | 4074 | -2.807 | 0.0038 | Yes |
Table: GSEA details [plain text format]

  

Fig 2: TABULA\_MURIS\_SENIS\_LARGE\_INTESTINE\_INTESTINAL\_CRYPT\_STEM\_CELL\_AGEING: Random ES distribution      
 Gene set null distribution of ES for **TABULA\_MURIS\_SENIS\_LARGE\_INTESTINE\_INTESTINAL\_CRYPT\_STEM\_CELL\_AGEING**

  
