# Supplementary material for: TMPRSS11B promotes an acidified microenvironment and immune suppression in squamous lung cancer
Source: EMBO Rep. 2025 Nov 10;26(24):6346–79. doi: 10.1038/s44319-025-00631-1 (PMC12714794; doi:10.1038/s44319-025-00631-1)
Supplement: Supplementary file 18 — Figure EV6 Source Data [file 44319_2025_631_MOESM18_ESM.zip › Figure EV6/EV6C-D/GSEA_Broad Institute_M8_T11b high vs low LUSC/TABULA_MURIS_SENIS_LARGE_INTESTINE_SECRETORY_CELL_AGEING.html]

Details for gene set TABULA\_MURIS\_SENIS\_LARGE\_INTESTINE\_SECRETORY\_CELL\_AGEING[GSEA]

|  || Dataset | T11b high vs low squamous\_GSEA\_Ranked |
| Phenotype | NoPhenotypeAvailable |
| Upregulated in class | na\_neg |
| GeneSet | TABULA\_MURIS\_SENIS\_LARGE\_INTESTINE\_SECRETORY\_CELL\_AGEING |
| Enrichment Score (ES) | -0.112070456 |
| Normalized Enrichment Score (NES) | -0.7798393 |
| Nominal p-value | 0.8708709 |
| FDR q-value | 1.0 |
| FWER p-Value | 1.0 |
Table: GSEA Results Summary

  

Fig 1: Enrichment plot: TABULA\_MURIS\_SENIS\_LARGE\_INTESTINE\_SECRETORY\_CELL\_AGEING      
 Profile of the Running ES Score & Positions of GeneSet Members on the Rank Ordered List

  

| SYMBOL | RANK IN GENE LIST | RANK METRIC SCORE | RUNNING ES | CORE ENRICHMENT || 1 | Mxd1 | 69 | 2.527 | -0.0032 | No |
| 2 | Ctsb | 177 | 1.695 | -0.0209 | No |
| 3 | Csf2ra | 185 | 1.657 | -0.0132 | No |
| 4 | Dusp1 | 239 | 1.468 | -0.0183 | No |
| 5 | Psap | 240 | 1.466 | -0.0099 | No |
| 6 | S100a14 | 311 | 1.260 | -0.0206 | No |
| 7 | Ehd1 | 313 | 1.237 | -0.0138 | No |
| 8 | Ifitm2 | 465 | 0.952 | -0.0471 | No |
| 9 | Ehd4 | 474 | 0.943 | -0.0437 | No |
| 10 | Rnf149 | 502 | 0.897 | -0.0455 | No |
| 11 | Gadd45b | 522 | 0.873 | -0.0454 | No |
| 12 | St6galnac4 | 525 | 0.873 | -0.0409 | No |
| 13 | Dnajb1 | 534 | 0.862 | -0.0379 | No |
| 14 | Trf | 601 | 0.771 | -0.0505 | No |
| 15 | Oaz2 | 610 | 0.756 | -0.0482 | No |
| 16 | Dusp3 | 634 | 0.725 | -0.0499 | No |
| 17 | Cotl1 | 656 | 0.709 | -0.0512 | No |
| 18 | Tinagl1 | 685 | 0.683 | -0.0545 | No |
| 19 | Ece1 | 701 | 0.665 | -0.0545 | No |
| 20 | Ehbp1l1 | 708 | 0.661 | -0.0522 | No |
| 21 | Ssr1 | 710 | 0.661 | -0.0487 | No |
| 22 | Pgk1 | 717 | 0.655 | -0.0465 | No |
| 23 | Ctnnbip1 | 791 | 0.597 | -0.0618 | No |
| 24 | Arpc4 | 798 | 0.593 | -0.0599 | No |
| 25 | Slc48a1 | 806 | 0.591 | -0.0583 | No |
| 26 | Pkm | 807 | 0.591 | -0.0549 | No |
| 27 | Ube2v1 | 808 | 0.590 | -0.0515 | No |
| 28 | Sfn | 834 | 0.573 | -0.0546 | No |
| 29 | Acat2 | 844 | 0.569 | -0.0537 | No |
| 30 | Stap2 | 845 | 0.568 | -0.0504 | No |
| 31 | Prnp | 853 | 0.565 | -0.0489 | No |
| 32 | Dusp5 | 861 | 0.563 | -0.0475 | No |
| 33 | Psmd2 | 862 | 0.563 | -0.0443 | No |
| 34 | Csrnp1 | 869 | 0.559 | -0.0426 | No |
| 35 | Bcl2l1 | 878 | 0.552 | -0.0415 | No |
| 36 | Fam110a | 891 | 0.544 | -0.0414 | No |
| 37 | Cfl1 | 895 | 0.538 | -0.0391 | No |
| 38 | Sh3gl1 | 898 | 0.535 | -0.0365 | No |
| 39 | Dpysl2 | 910 | 0.527 | -0.0363 | No |
| 40 | Xpnpep1 | 947 | 0.507 | -0.0426 | No |
| 41 | Calm3 | 961 | -0.500 | -0.0431 | No |
| 42 | Cln6 | 966 | -0.501 | -0.0413 | No |
| 43 | Usp22 | 1000 | -0.506 | -0.0468 | No |
| 44 | Slc35a2 | 1022 | -0.509 | -0.0493 | No |
| 45 | Fbxw2 | 1028 | -0.510 | -0.0476 | No |
| 46 | Ppm1g | 1043 | -0.512 | -0.0483 | No |
| 47 | Trabd | 1079 | -0.518 | -0.0543 | No |
| 48 | Elof1 | 1092 | -0.521 | -0.0544 | No |
| 49 | Eml2 | 1114 | -0.525 | -0.0568 | No |
| 50 | Polr3d | 1147 | -0.530 | -0.0619 | No |
| 51 | Hipk1 | 1188 | -0.537 | -0.0691 | No |
| 52 | Prrg2 | 1221 | -0.544 | -0.0742 | No |
| 53 | Dhrs4 | 1226 | -0.545 | -0.0721 | No |
| 54 | Emc10 | 1234 | -0.546 | -0.0708 | No |
| 55 | Apobec3 | 1238 | -0.546 | -0.0684 | No |
| 56 | Farsb | 1245 | -0.547 | -0.0668 | No |
| 57 | Ung | 1258 | -0.549 | -0.0667 | No |
| 58 | Tbc1d17 | 1266 | -0.549 | -0.0653 | No |
| 59 | Btbd2 | 1337 | -0.564 | -0.0801 | No |
| 60 | Atad3a | 1345 | -0.565 | -0.0786 | No |
| 61 | Pick1 | 1384 | -0.571 | -0.0851 | No |
| 62 | Efcab14 | 1394 | -0.574 | -0.0841 | No |
| 63 | Tmem161a | 1429 | -0.580 | -0.0895 | No |
| 64 | Egln2 | 1438 | -0.583 | -0.0882 | No |
| 65 | Fam241b | 1461 | -0.586 | -0.0905 | No |
| 66 | Ppil1 | 1464 | -0.586 | -0.0876 | No |
| 67 | Pak1 | 1534 | -0.600 | -0.1019 | No |
| 68 | Rnf44 | 1557 | -0.604 | -0.1041 | No |
| 69 | Abcb8 | 1589 | -0.609 | -0.1086 | Yes |
| 70 | Eif2a | 1596 | -0.610 | -0.1066 | Yes |
| 71 | Tmbim6 | 1599 | -0.611 | -0.1036 | Yes |
| 72 | Tfg | 1600 | -0.611 | -0.1001 | Yes |
| 73 | Pkp2 | 1604 | -0.611 | -0.0973 | Yes |
| 74 | Ppp1r35 | 1611 | -0.612 | -0.0953 | Yes |
| 75 | Lias | 1612 | -0.612 | -0.0918 | Yes |
| 76 | Arfgef3 | 1622 | -0.615 | -0.0906 | Yes |
| 77 | Inpp4a | 1672 | -0.625 | -0.0996 | Yes |
| 78 | Tmed9 | 1675 | -0.626 | -0.0965 | Yes |
| 79 | Mpst | 1677 | -0.626 | -0.0931 | Yes |
| 80 | Foxp1 | 1684 | -0.627 | -0.0911 | Yes |
| 81 | Nt5c3b | 1697 | -0.630 | -0.0905 | Yes |
| 82 | Kmt5c | 1703 | -0.631 | -0.0882 | Yes |
| 83 | Tsc22d1 | 1718 | -0.633 | -0.0881 | Yes |
| 84 | Actr2 | 1729 | -0.634 | -0.0871 | Yes |
| 85 | Brix1 | 1731 | -0.635 | -0.0837 | Yes |
| 86 | Bub3 | 1742 | -0.637 | -0.0826 | Yes |
| 87 | Nr1h2 | 1744 | -0.638 | -0.0791 | Yes |
| 88 | Sap18 | 1756 | -0.640 | -0.0783 | Yes |
| 89 | Ttc38 | 1759 | -0.641 | -0.0751 | Yes |
| 90 | 2610528J11Rik | 1763 | -0.642 | -0.0722 | Yes |
| 91 | Med25 | 1787 | -0.646 | -0.0744 | Yes |
| 92 | Aga | 1798 | -0.648 | -0.0732 | Yes |
| 93 | Nup62 | 1800 | -0.649 | -0.0697 | Yes |
| 94 | Tcf7l2 | 1816 | -0.651 | -0.0698 | Yes |
| 95 | Tmed4 | 1818 | -0.653 | -0.0663 | Yes |
| 96 | Slc30a6 | 1856 | -0.663 | -0.0720 | Yes |
| 97 | Lmf2 | 1915 | -0.678 | -0.0830 | Yes |
| 98 | Endog | 1948 | -0.685 | -0.0873 | Yes |
| 99 | Slc9a1 | 1949 | -0.685 | -0.0834 | Yes |
| 100 | Inava | 1951 | -0.685 | -0.0797 | Yes |
| 101 | Nectin2 | 1952 | -0.686 | -0.0757 | Yes |
| 102 | Vps72 | 2028 | -0.697 | -0.0910 | Yes |
| 103 | Coasy | 2030 | -0.698 | -0.0872 | Yes |
| 104 | Ccnd3 | 2040 | -0.700 | -0.0855 | Yes |
| 105 | Mospd3 | 2046 | -0.703 | -0.0827 | Yes |
| 106 | Snx17 | 2051 | -0.705 | -0.0797 | Yes |
| 107 | Prpf19 | 2056 | -0.709 | -0.0767 | Yes |
| 108 | Ppp4c | 2059 | -0.712 | -0.0731 | Yes |
| 109 | Gadd45gip1 | 2067 | -0.712 | -0.0708 | Yes |
| 110 | Foxa2 | 2097 | -0.712 | -0.0741 | Yes |
| 111 | Tnk1 | 2155 | -0.730 | -0.0846 | Yes |
| 112 | Tcf4 | 2163 | -0.732 | -0.0822 | Yes |
| 113 | Traf3ip2 | 2165 | -0.732 | -0.0782 | Yes |
| 114 | Tmub1 | 2176 | -0.734 | -0.0765 | Yes |
| 115 | Ddhd2 | 2180 | -0.735 | -0.0731 | Yes |
| 116 | Kdelr1 | 2186 | -0.736 | -0.0701 | Yes |
| 117 | Atg4b | 2204 | -0.739 | -0.0702 | Yes |
| 118 | Itpk1 | 2209 | -0.740 | -0.0670 | Yes |
| 119 | Spr | 2241 | -0.749 | -0.0707 | Yes |
| 120 | Ppif | 2248 | -0.750 | -0.0679 | Yes |
| 121 | Elavl1 | 2279 | -0.756 | -0.0712 | Yes |
| 122 | Eif2b4 | 2292 | -0.760 | -0.0699 | Yes |
| 123 | Cnp | 2305 | -0.763 | -0.0686 | Yes |
| 124 | Scn1b | 2310 | -0.764 | -0.0652 | Yes |
| 125 | Pnkp | 2336 | -0.771 | -0.0672 | Yes |
| 126 | Bzw2 | 2353 | -0.775 | -0.0669 | Yes |
| 127 | Klf16 | 2372 | -0.781 | -0.0670 | Yes |
| 128 | Tagap1 | 2442 | -0.801 | -0.0801 | Yes |
| 129 | Ep400 | 2501 | -0.819 | -0.0903 | Yes |
| 130 | Tmem109 | 2506 | -0.820 | -0.0866 | Yes |
| 131 | Inafm2 | 2519 | -0.824 | -0.0850 | Yes |
| 132 | Pcnp | 2520 | -0.824 | -0.0802 | Yes |
| 133 | Psmc4 | 2525 | -0.826 | -0.0765 | Yes |
| 134 | Bsg | 2530 | -0.827 | -0.0728 | Yes |
| 135 | Ruvbl2 | 2537 | -0.828 | -0.0695 | Yes |
| 136 | Dynll2 | 2574 | -0.841 | -0.0740 | Yes |
| 137 | Mcat | 2628 | -0.856 | -0.0826 | Yes |
| 138 | Syt7 | 2642 | -0.859 | -0.0810 | Yes |
| 139 | Ecsit | 2647 | -0.860 | -0.0771 | Yes |
| 140 | Vmac | 2653 | -0.861 | -0.0734 | Yes |
| 141 | Eif3f | 2655 | -0.863 | -0.0687 | Yes |
| 142 | Dcps | 2660 | -0.864 | -0.0648 | Yes |
| 143 | Dus1l | 2688 | -0.871 | -0.0667 | Yes |
| 144 | Nfia | 2708 | -0.877 | -0.0665 | Yes |
| 145 | Tmem263 | 2843 | -0.920 | -0.0957 | Yes |
| 146 | Lzts2 | 2877 | -0.933 | -0.0988 | Yes |
| 147 | Smco4 | 2881 | -0.934 | -0.0942 | Yes |
| 148 | Mrtfb | 2891 | -0.937 | -0.0911 | Yes |
| 149 | Cdk5rap3 | 2899 | -0.939 | -0.0875 | Yes |
| 150 | Paip1 | 2913 | -0.944 | -0.0854 | Yes |
| 151 | Shisa5 | 2916 | -0.946 | -0.0804 | Yes |
| 152 | Gpr180 | 2917 | -0.946 | -0.0750 | Yes |
| 153 | Commd10 | 2934 | -0.952 | -0.0736 | Yes |
| 154 | Bri3 | 2942 | -0.954 | -0.0699 | Yes |
| 155 | Rfc2 | 2975 | -0.965 | -0.0726 | Yes |
| 156 | Cd82 | 2976 | -0.965 | -0.0670 | Yes |
| 157 | Polr3e | 2997 | -0.975 | -0.0666 | Yes |
| 158 | Bag3 | 3005 | -0.975 | -0.0627 | Yes |
| 159 | Maz | 3048 | -0.994 | -0.0678 | Yes |
| 160 | Sh2b1 | 3151 | -1.038 | -0.0881 | Yes |
| 161 | Krt19 | 3163 | -1.044 | -0.0849 | Yes |
| 162 | Rgmb | 3164 | -1.045 | -0.0789 | Yes |
| 163 | Aldh9a1 | 3166 | -1.046 | -0.0731 | Yes |
| 164 | Btbd6 | 3170 | -1.047 | -0.0678 | Yes |
| 165 | Vamp2 | 3187 | -1.056 | -0.0659 | Yes |
| 166 | Slc25a10 | 3196 | -1.061 | -0.0618 | Yes |
| 167 | Rdh13 | 3200 | -1.066 | -0.0564 | Yes |
| 168 | Nudt22 | 3202 | -1.066 | -0.0505 | Yes |
| 169 | Akr1e1 | 3230 | -1.081 | -0.0513 | Yes |
| 170 | Sil1 | 3241 | -1.088 | -0.0476 | Yes |
| 171 | Tcea3 | 3242 | -1.088 | -0.0413 | Yes |
| 172 | Ccnd1 | 3303 | -1.112 | -0.0503 | Yes |
| 173 | Macrod1 | 3332 | -1.127 | -0.0510 | Yes |
| 174 | Sf3b4 | 3336 | -1.127 | -0.0453 | Yes |
| 175 | Msi2 | 3350 | -1.140 | -0.0421 | Yes |
| 176 | Ppcs | 3352 | -1.140 | -0.0358 | Yes |
| 177 | Mid1ip1 | 3392 | -1.158 | -0.0391 | Yes |
| 178 | Prkab1 | 3413 | -1.165 | -0.0376 | Yes |
| 179 | Pheta1 | 3479 | -1.202 | -0.0473 | Yes |
| 180 | Zfp787 | 3490 | -1.205 | -0.0430 | Yes |
| 181 | Gpd1 | 3502 | -1.211 | -0.0388 | Yes |
| 182 | Arfip2 | 3580 | -1.272 | -0.0513 | Yes |
| 183 | Fundc1 | 3600 | -1.286 | -0.0488 | Yes |
| 184 | Eri3 | 3605 | -1.290 | -0.0424 | Yes |
| 185 | Rbm38 | 3651 | -1.338 | -0.0462 | Yes |
| 186 | Hspa2 | 3696 | -1.378 | -0.0496 | Yes |
| 187 | Ptov1 | 3741 | -1.430 | -0.0527 | Yes |
| 188 | 2410002F23Rik | 3747 | -1.438 | -0.0457 | Yes |
| 189 | Fut2 | 3761 | -1.450 | -0.0407 | Yes |
| 190 | Qsox1 | 3787 | -1.486 | -0.0385 | Yes |
| 191 | Ppp1r1b | 3805 | -1.518 | -0.0342 | Yes |
| 192 | Klf5 | 3839 | -1.583 | -0.0335 | Yes |
| 193 | Ica1 | 3840 | -1.586 | -0.0244 | Yes |
| 194 | Bbc3 | 3841 | -1.590 | -0.0152 | Yes |
| 195 | Cnot9 | 3847 | -1.601 | -0.0073 | Yes |
| 196 | Cracr2b | 3867 | -1.641 | -0.0027 | Yes |
| 197 | Sox9 | 3877 | -1.658 | 0.0045 | Yes |
| 198 | Slc1a5 | 3889 | -1.685 | 0.0114 | Yes |
| 199 | Hid1 | 3915 | -1.752 | 0.0151 | Yes |
| 200 | D630039A03Rik | 3994 | -2.095 | 0.0071 | Yes |
| 201 | Baiap2l2 | 4079 | -2.967 | 0.0026 | Yes |
Table: GSEA details [plain text format]

  

Fig 2: TABULA\_MURIS\_SENIS\_LARGE\_INTESTINE\_SECRETORY\_CELL\_AGEING: Random ES distribution      
 Gene set null distribution of ES for **TABULA\_MURIS\_SENIS\_LARGE\_INTESTINE\_SECRETORY\_CELL\_AGEING**

  
