# Supplementary material for: TMPRSS11B promotes an acidified microenvironment and immune suppression in squamous lung cancer
Source: EMBO Rep. 2025 Nov 10;26(24):6346–79. doi: 10.1038/s44319-025-00631-1 (PMC12714794; doi:10.1038/s44319-025-00631-1)
Supplement: Supplementary file 18 — Figure EV6 Source Data [file 44319_2025_631_MOESM18_ESM.zip › Figure EV6/EV6C-D/GSEA_Broad Institute_M8_T11b high vs low LUSC/TABULA_MURIS_SENIS_LIVER_HEPATOCYTE_AGEING.html]

Details for gene set TABULA\_MURIS\_SENIS\_LIVER\_HEPATOCYTE\_AGEING[GSEA]

|  || Dataset | T11b high vs low squamous\_GSEA\_Ranked |
| Phenotype | NoPhenotypeAvailable |
| Upregulated in class | na\_neg |
| GeneSet | TABULA\_MURIS\_SENIS\_LIVER\_HEPATOCYTE\_AGEING |
| Enrichment Score (ES) | -0.22926746 |
| Normalized Enrichment Score (NES) | -1.1253976 |
| Nominal p-value | 0.30097088 |
| FDR q-value | 0.80964893 |
| FWER p-Value | 1.0 |
Table: GSEA Results Summary

  

Fig 1: Enrichment plot: TABULA\_MURIS\_SENIS\_LIVER\_HEPATOCYTE\_AGEING      
 Profile of the Running ES Score & Positions of GeneSet Members on the Rank Ordered List

  

| SYMBOL | RANK IN GENE LIST | RANK METRIC SCORE | RUNNING ES | CORE ENRICHMENT || 1 | Cd36 | 85 | 2.334 | 0.0318 | No |
| 2 | Scd1 | 152 | 1.815 | 0.0566 | No |
| 3 | Ctsb | 177 | 1.695 | 0.0890 | No |
| 4 | Psap | 240 | 1.466 | 0.1069 | No |
| 5 | Grn | 285 | 1.352 | 0.1266 | No |
| 6 | Rnf125 | 355 | 1.131 | 0.1351 | No |
| 7 | Col18a1 | 581 | 0.812 | 0.0979 | No |
| 8 | Actb | 633 | 0.726 | 0.1017 | No |
| 9 | Pltp | 661 | 0.702 | 0.1109 | No |
| 10 | H2-D1 | 719 | 0.654 | 0.1116 | No |
| 11 | H2-K1 | 855 | 0.565 | 0.0910 | No |
| 12 | Cfl1 | 895 | 0.538 | 0.0936 | No |
| 13 | Got1 | 949 | 0.506 | 0.0919 | No |
| 14 | Fah | 1257 | -0.549 | 0.0285 | No |
| 15 | Srebf1 | 1272 | -0.551 | 0.0375 | No |
| 16 | Krt8 | 1410 | -0.578 | 0.0167 | No |
| 17 | Gpt | 1561 | -0.605 | -0.0067 | No |
| 18 | Etfb | 1565 | -0.605 | 0.0063 | No |
| 19 | Mpst | 1677 | -0.626 | -0.0070 | No |
| 20 | Ifi27 | 1778 | -0.644 | -0.0172 | No |
| 21 | Gstt3 | 1849 | -0.660 | -0.0195 | No |
| 22 | Ly6e | 1857 | -0.663 | -0.0062 | No |
| 23 | Fpgs | 2193 | -0.737 | -0.0723 | No |
| 24 | Spr | 2241 | -0.749 | -0.0670 | No |
| 25 | Tmem176b | 2558 | -0.834 | -0.1262 | No |
| 26 | Zfp395 | 2572 | -0.841 | -0.1104 | No |
| 27 | Pigr | 3054 | -0.996 | -0.2067 | Yes |
| 28 | Asl | 3098 | -1.016 | -0.1944 | Yes |
| 29 | Coq8a | 3108 | -1.021 | -0.1735 | Yes |
| 30 | Sardh | 3118 | -1.026 | -0.1525 | Yes |
| 31 | Slc25a10 | 3196 | -1.061 | -0.1475 | Yes |
| 32 | Adh5 | 3278 | -1.105 | -0.1425 | Yes |
| 33 | Ass1 | 3295 | -1.111 | -0.1213 | Yes |
| 34 | Sult1a1 | 3309 | -1.116 | -0.0993 | Yes |
| 35 | Macrod1 | 3332 | -1.127 | -0.0792 | Yes |
| 36 | Clu | 3405 | -1.162 | -0.0707 | Yes |
| 37 | Aldh2 | 3521 | -1.226 | -0.0714 | Yes |
| 38 | Tmem176a | 3571 | -1.266 | -0.0549 | Yes |
| 39 | Clec2d | 3757 | -1.449 | -0.0678 | Yes |
| 40 | Qsox1 | 3787 | -1.486 | -0.0414 | Yes |
| 41 | Dcxr | 3823 | -1.555 | -0.0148 | Yes |
| 42 | Hgfac | 3920 | -1.761 | 0.0013 | Yes |
| 43 | Oat | 3935 | -1.777 | 0.0381 | Yes |
Table: GSEA details [plain text format]

  

Fig 2: TABULA\_MURIS\_SENIS\_LIVER\_HEPATOCYTE\_AGEING: Random ES distribution      
 Gene set null distribution of ES for **TABULA\_MURIS\_SENIS\_LIVER\_HEPATOCYTE\_AGEING**

  
