# Supplementary material for: TMPRSS11B promotes an acidified microenvironment and immune suppression in squamous lung cancer
Source: EMBO Rep. 2025 Nov 10;26(24):6346–79. doi: 10.1038/s44319-025-00631-1 (PMC12714794; doi:10.1038/s44319-025-00631-1)
Supplement: Supplementary file 18 — Figure EV6 Source Data [file 44319_2025_631_MOESM18_ESM.zip › Figure EV6/EV6C-D/GSEA_Broad Institute_M8_T11b high vs low LUSC/TABULA_MURIS_SENIS_LUNG_ALVEOLAR_MACROPHAGE_AGEING.html]

Details for gene set TABULA\_MURIS\_SENIS\_LUNG\_ALVEOLAR\_MACROPHAGE\_AGEING[GSEA]

|  || Dataset | T11b high vs low squamous\_GSEA\_Ranked |
| Phenotype | NoPhenotypeAvailable |
| Upregulated in class | na\_pos |
| GeneSet | TABULA\_MURIS\_SENIS\_LUNG\_ALVEOLAR\_MACROPHAGE\_AGEING |
| Enrichment Score (ES) | 0.6523547 |
| Normalized Enrichment Score (NES) | 3.774636 |
| Nominal p-value | 0.0 |
| FDR q-value | 0.0 |
| FWER p-Value | 0.0 |
Table: GSEA Results Summary

  

Fig 1: Enrichment plot: TABULA\_MURIS\_SENIS\_LUNG\_ALVEOLAR\_MACROPHAGE\_AGEING      
 Profile of the Running ES Score & Positions of GeneSet Members on the Rank Ordered List

  

| SYMBOL | RANK IN GENE LIST | RANK METRIC SCORE | RUNNING ES | CORE ENRICHMENT || 1 | Spp1 | 13 | 4.054 | 0.0399 | Yes |
| 2 | Ctsl | 15 | 3.988 | 0.0822 | Yes |
| 3 | Ctsk | 23 | 3.576 | 0.1185 | Yes |
| 4 | Trem2 | 24 | 3.569 | 0.1565 | Yes |
| 5 | S100a8 | 38 | 3.013 | 0.1854 | Yes |
| 6 | Lpl | 43 | 2.812 | 0.2143 | Yes |
| 7 | Cybb | 57 | 2.654 | 0.2394 | Yes |
| 8 | Ctss | 63 | 2.582 | 0.2656 | Yes |
| 9 | S100a10 | 66 | 2.555 | 0.2923 | Yes |
| 10 | Fcer1g | 76 | 2.415 | 0.3158 | Yes |
| 11 | S100a9 | 82 | 2.366 | 0.3398 | Yes |
| 12 | Tyrobp | 83 | 2.366 | 0.3650 | Yes |
| 13 | Apoe | 88 | 2.296 | 0.3885 | Yes |
| 14 | Ccl9 | 93 | 2.271 | 0.4117 | Yes |
| 15 | Cd274 | 96 | 2.227 | 0.4349 | Yes |
| 16 | Emp3 | 115 | 2.020 | 0.4519 | Yes |
| 17 | Wfdc17 | 117 | 1.998 | 0.4729 | Yes |
| 18 | Il1b | 129 | 1.912 | 0.4906 | Yes |
| 19 | Pirb | 137 | 1.894 | 0.5090 | Yes |
| 20 | Scd1 | 152 | 1.815 | 0.5249 | Yes |
| 21 | Apoc1 | 164 | 1.742 | 0.5407 | Yes |
| 22 | Hexb | 175 | 1.703 | 0.5563 | Yes |
| 23 | Ctsb | 177 | 1.695 | 0.5741 | Yes |
| 24 | Msn | 219 | 1.522 | 0.5802 | Yes |
| 25 | Psap | 240 | 1.466 | 0.5908 | Yes |
| 26 | Srgn | 270 | 1.392 | 0.5984 | Yes |
| 27 | Creg1 | 280 | 1.373 | 0.6108 | Yes |
| 28 | Fcgr4 | 322 | 1.209 | 0.6135 | Yes |
| 29 | Cd52 | 350 | 1.140 | 0.6189 | Yes |
| 30 | Igf1 | 376 | 1.100 | 0.6244 | Yes |
| 31 | Lgals3 | 377 | 1.096 | 0.6361 | Yes |
| 32 | Csf1r | 414 | 1.025 | 0.6381 | Yes |
| 33 | Timp2 | 462 | 0.955 | 0.6366 | Yes |
| 34 | Ifitm2 | 465 | 0.952 | 0.6462 | Yes |
| 35 | Cyba | 519 | 0.875 | 0.6423 | Yes |
| 36 | Npc2 | 536 | 0.861 | 0.6475 | Yes |
| 37 | Slpi | 558 | 0.836 | 0.6512 | Yes |
| 38 | Cd63 | 632 | 0.727 | 0.6408 | Yes |
| 39 | Crip1 | 638 | 0.721 | 0.6472 | Yes |
| 40 | Fabp4 | 657 | 0.708 | 0.6503 | Yes |
| 41 | Pgk1 | 717 | 0.655 | 0.6426 | Yes |
| 42 | H2-D1 | 719 | 0.654 | 0.6493 | Yes |
| 43 | Pkm | 807 | 0.591 | 0.6340 | Yes |
| 44 | Sat1 | 817 | 0.584 | 0.6380 | Yes |
| 45 | Cd74 | 849 | 0.567 | 0.6363 | Yes |
| 46 | H2-K1 | 855 | 0.565 | 0.6411 | Yes |
| 47 | Litaf | 856 | 0.564 | 0.6471 | Yes |
| 48 | B2m | 860 | 0.563 | 0.6524 | Yes |
| 49 | H2-Ab1 | 915 | 0.525 | 0.6445 | No |
| 50 | Rtn4 | 946 | 0.508 | 0.6425 | No |
| 51 | Ccdc12 | 957 | 0.501 | 0.6453 | No |
| 52 | Ssr4 | 1035 | -0.511 | 0.6316 | No |
| 53 | Uqcc3 | 1060 | -0.515 | 0.6311 | No |
| 54 | Trappc6a | 1231 | -0.545 | 0.5947 | No |
| 55 | Eif3k | 1325 | -0.562 | 0.5776 | No |
| 56 | Foxp1 | 1684 | -0.627 | 0.4952 | No |
| 57 | Ly6e | 1857 | -0.663 | 0.4596 | No |
| 58 | Grcc10 | 1882 | -0.670 | 0.4607 | No |
| 59 | Krtcap2 | 1937 | -0.682 | 0.4546 | No |
| 60 | Gstm1 | 2049 | -0.704 | 0.4345 | No |
| 61 | Alcam | 2584 | -0.843 | 0.3107 | No |
| 62 | Sod1 | 2691 | -0.874 | 0.2937 | No |
| 63 | Rabac1 | 2744 | -0.888 | 0.2902 | No |
| 64 | Cd24a | 2759 | -0.893 | 0.2962 | No |
| 65 | Rgs2 | 2919 | -0.946 | 0.2668 | No |
| 66 | Cd2ap | 3029 | -0.985 | 0.2502 | No |
| 67 | Aldh2 | 3521 | -1.226 | 0.1412 | No |
Table: GSEA details [plain text format]

  

Fig 2: TABULA\_MURIS\_SENIS\_LUNG\_ALVEOLAR\_MACROPHAGE\_AGEING: Random ES distribution      
 Gene set null distribution of ES for **TABULA\_MURIS\_SENIS\_LUNG\_ALVEOLAR\_MACROPHAGE\_AGEING**

  
