# Supplementary material for: TMPRSS11B promotes an acidified microenvironment and immune suppression in squamous lung cancer
Source: EMBO Rep. 2025 Nov 10;26(24):6346–79. doi: 10.1038/s44319-025-00631-1 (PMC12714794; doi:10.1038/s44319-025-00631-1)
Supplement: Supplementary file 18 — Figure EV6 Source Data [file 44319_2025_631_MOESM18_ESM.zip › Figure EV6/EV6C-D/GSEA_Broad Institute_M8_T11b high vs low LUSC/TABULA_MURIS_SENIS_LUNG_INTERMEDIATE_MONOCYTE_AGEING.html]

Details for gene set TABULA\_MURIS\_SENIS\_LUNG\_INTERMEDIATE\_MONOCYTE\_AGEING[GSEA]

|  || Dataset | T11b high vs low squamous\_GSEA\_Ranked |
| Phenotype | NoPhenotypeAvailable |
| Upregulated in class | na\_pos |
| GeneSet | TABULA\_MURIS\_SENIS\_LUNG\_INTERMEDIATE\_MONOCYTE\_AGEING |
| Enrichment Score (ES) | 0.51216674 |
| Normalized Enrichment Score (NES) | 3.1534595 |
| Nominal p-value | 0.0 |
| FDR q-value | 0.0 |
| FWER p-Value | 0.0 |
Table: GSEA Results Summary

  

Fig 1: Enrichment plot: TABULA\_MURIS\_SENIS\_LUNG\_INTERMEDIATE\_MONOCYTE\_AGEING      
 Profile of the Running ES Score & Positions of GeneSet Members on the Rank Ordered List

  

| SYMBOL | RANK IN GENE LIST | RANK METRIC SCORE | RUNNING ES | CORE ENRICHMENT || 1 | Ctsl | 15 | 3.988 | 0.0359 | Yes |
| 2 | Trem2 | 24 | 3.569 | 0.0694 | Yes |
| 3 | S100a8 | 38 | 3.013 | 0.0961 | Yes |
| 4 | Clec4d | 39 | 3.005 | 0.1260 | Yes |
| 5 | Cybb | 57 | 2.654 | 0.1481 | Yes |
| 6 | S100a10 | 66 | 2.555 | 0.1715 | Yes |
| 7 | S100a9 | 82 | 2.366 | 0.1913 | Yes |
| 8 | Apoe | 88 | 2.296 | 0.2129 | Yes |
| 9 | Ly6a | 92 | 2.274 | 0.2347 | Yes |
| 10 | Ccl9 | 93 | 2.271 | 0.2573 | Yes |
| 11 | Plek | 95 | 2.237 | 0.2793 | Yes |
| 12 | Lgals1 | 98 | 2.178 | 0.3004 | Yes |
| 13 | Wfdc17 | 117 | 1.998 | 0.3158 | Yes |
| 14 | Il1b | 129 | 1.912 | 0.3321 | Yes |
| 15 | Pirb | 137 | 1.894 | 0.3492 | Yes |
| 16 | Fth1 | 147 | 1.835 | 0.3651 | Yes |
| 17 | Ctsd | 150 | 1.830 | 0.3828 | Yes |
| 18 | Emb | 205 | 1.583 | 0.3851 | Yes |
| 19 | Marcksl1 | 221 | 1.520 | 0.3965 | Yes |
| 20 | Csf2rb | 243 | 1.458 | 0.4057 | Yes |
| 21 | Acp5 | 247 | 1.450 | 0.4194 | Yes |
| 22 | Syk | 278 | 1.375 | 0.4256 | Yes |
| 23 | Creg1 | 280 | 1.373 | 0.4390 | Yes |
| 24 | Hp | 286 | 1.351 | 0.4511 | Yes |
| 25 | Hck | 312 | 1.246 | 0.4573 | Yes |
| 26 | Fcgr4 | 322 | 1.209 | 0.4670 | Yes |
| 27 | Rassf4 | 338 | 1.171 | 0.4749 | Yes |
| 28 | Cd52 | 350 | 1.140 | 0.4835 | Yes |
| 29 | Bcl2a1b | 359 | 1.126 | 0.4927 | Yes |
| 30 | Lgals3 | 377 | 1.096 | 0.4994 | Yes |
| 31 | Tnfaip2 | 387 | 1.086 | 0.5079 | Yes |
| 32 | Ifitm2 | 465 | 0.952 | 0.4982 | Yes |
| 33 | Mif | 484 | 0.921 | 0.5028 | Yes |
| 34 | Prelid1 | 506 | 0.892 | 0.5064 | Yes |
| 35 | Cyba | 519 | 0.875 | 0.5121 | Yes |
| 36 | Slpi | 558 | 0.836 | 0.5110 | Yes |
| 37 | Txn1 | 586 | 0.800 | 0.5122 | Yes |
| 38 | Esd | 653 | 0.710 | 0.5027 | No |
| 39 | Pgk1 | 717 | 0.655 | 0.4935 | No |
| 40 | Aprt | 739 | 0.638 | 0.4946 | No |
| 41 | Pkm | 807 | 0.591 | 0.4838 | No |
| 42 | Fam111a | 809 | 0.590 | 0.4894 | No |
| 43 | Rilpl2 | 852 | 0.566 | 0.4845 | No |
| 44 | Snf8 | 857 | 0.564 | 0.4891 | No |
| 45 | Macf1 | 883 | 0.550 | 0.4883 | No |
| 46 | Jak1 | 896 | 0.537 | 0.4907 | No |
| 47 | Ccdc12 | 957 | 0.501 | 0.4807 | No |
| 48 | Eef1d | 991 | -0.504 | 0.4774 | No |
| 49 | Ssr4 | 1035 | -0.511 | 0.4718 | No |
| 50 | Uqcc3 | 1060 | -0.515 | 0.4709 | No |
| 51 | Dnm2 | 1155 | -0.531 | 0.4527 | No |
| 52 | Cbl | 1164 | -0.533 | 0.4560 | No |
| 53 | Cox7a2l | 1218 | -0.543 | 0.4482 | No |
| 54 | Eif3c | 1220 | -0.544 | 0.4533 | No |
| 55 | Trappc6a | 1231 | -0.545 | 0.4562 | No |
| 56 | Rexo1 | 1262 | -0.549 | 0.4542 | No |
| 57 | Sec11c | 1267 | -0.550 | 0.4587 | No |
| 58 | Eif3k | 1325 | -0.562 | 0.4500 | No |
| 59 | Xpa | 1503 | -0.595 | 0.4117 | No |
| 60 | Tbcb | 1546 | -0.602 | 0.4072 | No |
| 61 | Etfb | 1565 | -0.605 | 0.4087 | No |
| 62 | Tmem208 | 1679 | -0.626 | 0.3867 | No |
| 63 | Emg1 | 1738 | -0.637 | 0.3786 | No |
| 64 | Usp34 | 1772 | -0.643 | 0.3767 | No |
| 65 | Ifi27 | 1778 | -0.644 | 0.3819 | No |
| 66 | Reep5 | 1833 | -0.657 | 0.3749 | No |
| 67 | Nol7 | 1851 | -0.661 | 0.3773 | No |
| 68 | Grcc10 | 1882 | -0.670 | 0.3764 | No |
| 69 | P4hb | 1926 | -0.681 | 0.3725 | No |
| 70 | Krtcap2 | 1937 | -0.682 | 0.3767 | No |
| 71 | Cnpy2 | 2142 | -0.727 | 0.3330 | No |
| 72 | Rere | 2266 | -0.755 | 0.3098 | No |
| 73 | Fas | 2311 | -0.764 | 0.3064 | No |
| 74 | Ciao2a | 2322 | -0.768 | 0.3115 | No |
| 75 | Son | 2418 | -0.795 | 0.2957 | No |
| 76 | Spata13 | 2649 | -0.860 | 0.2468 | No |
| 77 | Ddt | 2681 | -0.869 | 0.2477 | No |
| 78 | Sod1 | 2691 | -0.874 | 0.2542 | No |
| 79 | Tnrc6b | 2705 | -0.877 | 0.2596 | No |
| 80 | Rbm39 | 2864 | -0.930 | 0.2294 | No |
| 81 | Cyb5a | 3121 | -1.027 | 0.1757 | No |
| 82 | Kif5b | 3281 | -1.107 | 0.1470 | No |
| 83 | Smim11 | 3284 | -1.107 | 0.1575 | No |
| 84 | Rel | 3500 | -1.210 | 0.1158 | No |
| 85 | Lmo4 | 3802 | -1.515 | 0.0557 | No |
| 86 | Tnfaip8 | 3837 | -1.581 | 0.0629 | No |
Table: GSEA details [plain text format]

  

Fig 2: TABULA\_MURIS\_SENIS\_LUNG\_INTERMEDIATE\_MONOCYTE\_AGEING: Random ES distribution      
 Gene set null distribution of ES for **TABULA\_MURIS\_SENIS\_LUNG\_INTERMEDIATE\_MONOCYTE\_AGEING**

  
