# Supplementary material for: TMPRSS11B promotes an acidified microenvironment and immune suppression in squamous lung cancer
Source: EMBO Rep. 2025 Nov 10;26(24):6346–79. doi: 10.1038/s44319-025-00631-1 (PMC12714794; doi:10.1038/s44319-025-00631-1)
Supplement: Supplementary file 18 — Figure EV6 Source Data [file 44319_2025_631_MOESM18_ESM.zip › Figure EV6/EV6C-D/GSEA_Broad Institute_M8_T11b high vs low LUSC/TABULA_MURIS_SENIS_MAMMARY_GLAND_T_CELL_AGEING.html]

Details for gene set TABULA\_MURIS\_SENIS\_MAMMARY\_GLAND\_T\_CELL\_AGEING[GSEA]

|  || Dataset | T11b high vs low squamous\_GSEA\_Ranked |
| Phenotype | NoPhenotypeAvailable |
| Upregulated in class | na\_pos |
| GeneSet | TABULA\_MURIS\_SENIS\_MAMMARY\_GLAND\_T\_CELL\_AGEING |
| Enrichment Score (ES) | 0.4661027 |
| Normalized Enrichment Score (NES) | 3.1900249 |
| Nominal p-value | 0.0 |
| FDR q-value | 0.0 |
| FWER p-Value | 0.0 |
Table: GSEA Results Summary

  

Fig 1: Enrichment plot: TABULA\_MURIS\_SENIS\_MAMMARY\_GLAND\_T\_CELL\_AGEING      
 Profile of the Running ES Score & Positions of GeneSet Members on the Rank Ordered List

  

| SYMBOL | RANK IN GENE LIST | RANK METRIC SCORE | RUNNING ES | CORE ENRICHMENT || 1 | Krt14 | 56 | 2.659 | 0.0044 | Yes |
| 2 | Ctss | 63 | 2.582 | 0.0210 | Yes |
| 3 | S100a10 | 66 | 2.555 | 0.0384 | Yes |
| 4 | Itgb2 | 71 | 2.481 | 0.0547 | Yes |
| 5 | Cd37 | 74 | 2.458 | 0.0714 | Yes |
| 6 | Fcer1g | 76 | 2.415 | 0.0881 | Yes |
| 7 | Tyrobp | 83 | 2.366 | 0.1031 | Yes |
| 8 | Apoe | 88 | 2.296 | 0.1182 | Yes |
| 9 | Ly6a | 92 | 2.274 | 0.1333 | Yes |
| 10 | Lgals1 | 98 | 2.178 | 0.1473 | Yes |
| 11 | Glrx | 105 | 2.127 | 0.1607 | Yes |
| 12 | Vim | 112 | 2.054 | 0.1735 | Yes |
| 13 | Emp3 | 115 | 2.020 | 0.1872 | Yes |
| 14 | Ctsd | 150 | 1.830 | 0.1914 | Yes |
| 15 | Fxyd5 | 157 | 1.767 | 0.2022 | Yes |
| 16 | Gpsm3 | 169 | 1.725 | 0.2115 | Yes |
| 17 | Ctsb | 177 | 1.695 | 0.2216 | Yes |
| 18 | Crlf2 | 182 | 1.670 | 0.2323 | Yes |
| 19 | Cdkn1a | 192 | 1.625 | 0.2414 | Yes |
| 20 | Sla | 207 | 1.573 | 0.2488 | Yes |
| 21 | Arhgdib | 214 | 1.549 | 0.2582 | Yes |
| 22 | Msn | 219 | 1.522 | 0.2678 | Yes |
| 23 | Marcksl1 | 221 | 1.520 | 0.2782 | Yes |
| 24 | Lcp1 | 231 | 1.490 | 0.2863 | Yes |
| 25 | Dusp1 | 239 | 1.468 | 0.2948 | Yes |
| 26 | Psap | 240 | 1.466 | 0.3051 | Yes |
| 27 | Ptprc | 258 | 1.430 | 0.3108 | Yes |
| 28 | Arhgap9 | 260 | 1.428 | 0.3206 | Yes |
| 29 | Pxdc1 | 274 | 1.384 | 0.3270 | Yes |
| 30 | Cxcr4 | 289 | 1.347 | 0.3328 | Yes |
| 31 | Hilpda | 302 | 1.302 | 0.3389 | Yes |
| 32 | Selplg | 328 | 1.195 | 0.3409 | Yes |
| 33 | Capg | 343 | 1.160 | 0.3455 | Yes |
| 34 | Cd52 | 350 | 1.140 | 0.3520 | Yes |
| 35 | Bcl2a1b | 359 | 1.126 | 0.3578 | Yes |
| 36 | Ppp1r18 | 371 | 1.111 | 0.3628 | Yes |
| 37 | Atf3 | 373 | 1.107 | 0.3703 | Yes |
| 38 | Lgals3 | 377 | 1.096 | 0.3772 | Yes |
| 39 | Coro1a | 390 | 1.079 | 0.3818 | Yes |
| 40 | Pkp3 | 402 | 1.044 | 0.3863 | Yes |
| 41 | Lpxn | 431 | 1.004 | 0.3862 | Yes |
| 42 | Timp2 | 462 | 0.955 | 0.3853 | Yes |
| 43 | Tnfrsf1b | 463 | 0.955 | 0.3920 | Yes |
| 44 | Ifitm2 | 465 | 0.952 | 0.3984 | Yes |
| 45 | Flna | 471 | 0.946 | 0.4038 | Yes |
| 46 | Ier5 | 493 | 0.908 | 0.4048 | Yes |
| 47 | Hcls1 | 503 | 0.897 | 0.4088 | Yes |
| 48 | Cebpb | 504 | 0.897 | 0.4151 | Yes |
| 49 | Cyba | 519 | 0.875 | 0.4177 | Yes |
| 50 | Gadd45b | 522 | 0.873 | 0.4233 | Yes |
| 51 | Rgs1 | 526 | 0.873 | 0.4286 | Yes |
| 52 | Cd44 | 562 | 0.834 | 0.4256 | Yes |
| 53 | 1810037I17Rik | 582 | 0.811 | 0.4264 | Yes |
| 54 | Txn1 | 586 | 0.800 | 0.4313 | Yes |
| 55 | Atp6v0e | 590 | 0.795 | 0.4361 | Yes |
| 56 | Anxa6 | 603 | 0.769 | 0.4384 | Yes |
| 57 | D16Ertd472e | 612 | 0.752 | 0.4417 | Yes |
| 58 | Actb | 633 | 0.726 | 0.4417 | Yes |
| 59 | Crip1 | 638 | 0.721 | 0.4457 | Yes |
| 60 | Cotl1 | 656 | 0.709 | 0.4464 | Yes |
| 61 | Klf4 | 662 | 0.701 | 0.4500 | Yes |
| 62 | Ostf1 | 691 | 0.673 | 0.4477 | Yes |
| 63 | Myl12a | 713 | 0.657 | 0.4469 | Yes |
| 64 | H2-D1 | 719 | 0.654 | 0.4502 | Yes |
| 65 | Ubl3 | 751 | 0.627 | 0.4468 | Yes |
| 66 | Anxa2 | 760 | 0.620 | 0.4491 | Yes |
| 67 | Kctd12 | 783 | 0.601 | 0.4477 | Yes |
| 68 | Arpc4 | 798 | 0.593 | 0.4483 | Yes |
| 69 | Celf2 | 799 | 0.593 | 0.4525 | Yes |
| 70 | Pkm | 807 | 0.591 | 0.4549 | Yes |
| 71 | Gm2a | 815 | 0.585 | 0.4572 | Yes |
| 72 | Sfn | 834 | 0.573 | 0.4566 | Yes |
| 73 | Rexo2 | 839 | 0.571 | 0.4596 | Yes |
| 74 | H2-K1 | 855 | 0.565 | 0.4598 | Yes |
| 75 | B2m | 860 | 0.563 | 0.4627 | Yes |
| 76 | Csrnp1 | 869 | 0.559 | 0.4646 | Yes |
| 77 | Wipf1 | 888 | 0.546 | 0.4639 | Yes |
| 78 | Cfl1 | 895 | 0.538 | 0.4661 | Yes |
| 79 | Nfkbia | 954 | 0.502 | 0.4549 | No |
| 80 | Ccdc12 | 957 | 0.501 | 0.4579 | No |
| 81 | Calm3 | 961 | -0.500 | 0.4607 | No |
| 82 | Ucp2 | 979 | -0.502 | 0.4599 | No |
| 83 | Gnb2 | 1007 | -0.506 | 0.4566 | No |
| 84 | Vps28 | 1018 | -0.509 | 0.4576 | No |
| 85 | Ssr4 | 1035 | -0.511 | 0.4571 | No |
| 86 | Sri | 1116 | -0.525 | 0.4406 | No |
| 87 | Nt5c | 1142 | -0.530 | 0.4380 | No |
| 88 | Cmtm7 | 1228 | -0.545 | 0.4203 | No |
| 89 | Trappc6a | 1231 | -0.545 | 0.4236 | No |
| 90 | Emc10 | 1234 | -0.546 | 0.4269 | No |
| 91 | Apobec3 | 1238 | -0.546 | 0.4299 | No |
| 92 | Sec11c | 1267 | -0.550 | 0.4267 | No |
| 93 | Tram1 | 1351 | -0.566 | 0.4096 | No |
| 94 | Psma1 | 1412 | -0.578 | 0.3985 | No |
| 95 | Odc1 | 1542 | -0.601 | 0.3701 | No |
| 96 | Ccdc107 | 1556 | -0.604 | 0.3710 | No |
| 97 | Etfb | 1565 | -0.605 | 0.3732 | No |
| 98 | Ddost | 1569 | -0.606 | 0.3767 | No |
| 99 | Tmbim6 | 1599 | -0.611 | 0.3736 | No |
| 100 | Plaat3 | 1603 | -0.611 | 0.3771 | No |
| 101 | Nop10 | 1642 | -0.619 | 0.3719 | No |
| 102 | Pabpn1 | 1680 | -0.626 | 0.3669 | No |
| 103 | Taf10 | 1702 | -0.630 | 0.3660 | No |
| 104 | Actr2 | 1729 | -0.634 | 0.3638 | No |
| 105 | Ifi27 | 1778 | -0.644 | 0.3562 | No |
| 106 | Smim14 | 1822 | -0.654 | 0.3499 | No |
| 107 | Reep5 | 1833 | -0.657 | 0.3520 | No |
| 108 | Ly6e | 1857 | -0.663 | 0.3508 | No |
| 109 | Cox17 | 1863 | -0.664 | 0.3542 | No |
| 110 | Grcc10 | 1882 | -0.670 | 0.3543 | No |
| 111 | Krtcap2 | 1937 | -0.682 | 0.3454 | No |
| 112 | Ccnd3 | 2040 | -0.700 | 0.3245 | No |
| 113 | Selenop | 2054 | -0.707 | 0.3261 | No |
| 114 | Cox7c | 2108 | -0.712 | 0.3177 | No |
| 115 | Lsm7 | 2203 | -0.739 | 0.2991 | No |
| 116 | Ciao2a | 2322 | -0.768 | 0.2746 | No |
| 117 | Spn | 2413 | -0.794 | 0.2574 | No |
| 118 | Hmgb1 | 2518 | -0.823 | 0.2368 | No |
| 119 | Bsg | 2530 | -0.827 | 0.2398 | No |
| 120 | Tmem176b | 2558 | -0.834 | 0.2388 | No |
| 121 | Pigp | 2656 | -0.864 | 0.2203 | No |
| 122 | F2r | 2690 | -0.872 | 0.2181 | No |
| 123 | Il18r1 | 2742 | -0.887 | 0.2114 | No |
| 124 | Ech1 | 2819 | -0.911 | 0.1985 | No |
| 125 | BC031181 | 2879 | -0.933 | 0.1901 | No |
| 126 | Shisa5 | 2916 | -0.946 | 0.1876 | No |
| 127 | Rgs2 | 2919 | -0.946 | 0.1937 | No |
| 128 | Nr4a2 | 2921 | -0.946 | 0.2001 | No |
| 129 | Txnip | 2924 | -0.947 | 0.2062 | No |
| 130 | Ppp1r12a | 2944 | -0.954 | 0.2081 | No |
| 131 | Cd82 | 2976 | -0.965 | 0.2070 | No |
| 132 | Fos | 3189 | -1.058 | 0.1608 | No |
| 133 | Spint2 | 3222 | -1.076 | 0.1602 | No |
| 134 | Pdcd4 | 3488 | -1.204 | 0.1015 | No |
| 135 | Egr1 | 3532 | -1.234 | 0.0993 | No |
| 136 | Tmem176a | 3571 | -1.266 | 0.0985 | No |
| 137 | Clec2d | 3757 | -1.449 | 0.0619 | No |
| 138 | Lmo4 | 3802 | -1.515 | 0.0613 | No |
| 139 | Tnfaip8 | 3837 | -1.581 | 0.0638 | No |
Table: GSEA details [plain text format]

  

Fig 2: TABULA\_MURIS\_SENIS\_MAMMARY\_GLAND\_T\_CELL\_AGEING: Random ES distribution      
 Gene set null distribution of ES for **TABULA\_MURIS\_SENIS\_MAMMARY\_GLAND\_T\_CELL\_AGEING**

  
