# Supplementary material for: TMPRSS11B promotes an acidified microenvironment and immune suppression in squamous lung cancer
Source: EMBO Rep. 2025 Nov 10;26(24):6346–79. doi: 10.1038/s44319-025-00631-1 (PMC12714794; doi:10.1038/s44319-025-00631-1)
Supplement: Supplementary file 18 — Figure EV6 Source Data [file 44319_2025_631_MOESM18_ESM.zip › Figure EV6/EV6C-D/GSEA_Broad Institute_M8_T11b high vs low LUSC/TABULA_MURIS_SENIS_MARROW_GRANULOCYTE_AGEING.html]

Details for gene set TABULA\_MURIS\_SENIS\_MARROW\_GRANULOCYTE\_AGEING[GSEA]

|  || Dataset | T11b high vs low squamous\_GSEA\_Ranked |
| Phenotype | NoPhenotypeAvailable |
| Upregulated in class | na\_pos |
| GeneSet | TABULA\_MURIS\_SENIS\_MARROW\_GRANULOCYTE\_AGEING |
| Enrichment Score (ES) | 0.7443518 |
| Normalized Enrichment Score (NES) | 3.3246467 |
| Nominal p-value | 0.0 |
| FDR q-value | 0.0 |
| FWER p-Value | 0.0 |
Table: GSEA Results Summary

  

Fig 1: Enrichment plot: TABULA\_MURIS\_SENIS\_MARROW\_GRANULOCYTE\_AGEING      
 Profile of the Running ES Score & Positions of GeneSet Members on the Rank Ordered List

  

| SYMBOL | RANK IN GENE LIST | RANK METRIC SCORE | RUNNING ES | CORE ENRICHMENT || 1 | S100a8 | 38 | 3.013 | 0.0754 | Yes |
| 2 | Fcer1g | 76 | 2.415 | 0.1342 | Yes |
| 3 | S100a9 | 82 | 2.366 | 0.1995 | Yes |
| 4 | Tyrobp | 83 | 2.366 | 0.2661 | Yes |
| 5 | Emp3 | 115 | 2.020 | 0.3152 | Yes |
| 6 | Fxyd5 | 157 | 1.767 | 0.3549 | Yes |
| 7 | Spi1 | 158 | 1.765 | 0.4045 | Yes |
| 8 | Ccl6 | 166 | 1.733 | 0.4515 | Yes |
| 9 | Orm1 | 176 | 1.697 | 0.4971 | Yes |
| 10 | Hp | 286 | 1.351 | 0.5082 | Yes |
| 11 | Cd52 | 350 | 1.140 | 0.5248 | Yes |
| 12 | Lgals3 | 377 | 1.096 | 0.5492 | Yes |
| 13 | Coro1a | 390 | 1.079 | 0.5766 | Yes |
| 14 | Alox5ap | 399 | 1.051 | 0.6042 | Yes |
| 15 | Lcn2 | 444 | 0.985 | 0.6210 | Yes |
| 16 | Ifitm2 | 465 | 0.952 | 0.6429 | Yes |
| 17 | Cyba | 519 | 0.875 | 0.6544 | Yes |
| 18 | Slpi | 558 | 0.836 | 0.6686 | Yes |
| 19 | Cd63 | 632 | 0.727 | 0.6711 | Yes |
| 20 | Cotl1 | 656 | 0.709 | 0.6853 | Yes |
| 21 | H2-D1 | 719 | 0.654 | 0.6884 | Yes |
| 22 | Anxa2 | 760 | 0.620 | 0.6960 | Yes |
| 23 | Arrb2 | 786 | 0.600 | 0.7068 | Yes |
| 24 | Pkm | 807 | 0.591 | 0.7184 | Yes |
| 25 | H2-K1 | 855 | 0.565 | 0.7227 | Yes |
| 26 | B2m | 860 | 0.563 | 0.7376 | Yes |
| 27 | Cfl1 | 895 | 0.538 | 0.7444 | Yes |
| 28 | Cebpd | 2002 | -0.693 | 0.4915 | No |
| 29 | Jchain | 2392 | -0.788 | 0.4179 | No |
Table: GSEA details [plain text format]

  

Fig 2: TABULA\_MURIS\_SENIS\_MARROW\_GRANULOCYTE\_AGEING: Random ES distribution      
 Gene set null distribution of ES for **TABULA\_MURIS\_SENIS\_MARROW\_GRANULOCYTE\_AGEING**

  
