# Supplementary material for: TMPRSS11B promotes an acidified microenvironment and immune suppression in squamous lung cancer
Source: EMBO Rep. 2025 Nov 10;26(24):6346–79. doi: 10.1038/s44319-025-00631-1 (PMC12714794; doi:10.1038/s44319-025-00631-1)
Supplement: Supplementary file 18 — Figure EV6 Source Data [file 44319_2025_631_MOESM18_ESM.zip › Figure EV6/EV6C-D/GSEA_Broad Institute_M8_T11b high vs low LUSC/TABULA_MURIS_SENIS_MARROW_GRANULOCYTOPOIETIC_CELL_AGEING.html]

Details for gene set TABULA\_MURIS\_SENIS\_MARROW\_GRANULOCYTOPOIETIC\_CELL\_AGEING[GSEA]

|  || Dataset | T11b high vs low squamous\_GSEA\_Ranked |
| Phenotype | NoPhenotypeAvailable |
| Upregulated in class | na\_pos |
| GeneSet | TABULA\_MURIS\_SENIS\_MARROW\_GRANULOCYTOPOIETIC\_CELL\_AGEING |
| Enrichment Score (ES) | 0.6532081 |
| Normalized Enrichment Score (NES) | 3.37994 |
| Nominal p-value | 0.0 |
| FDR q-value | 0.0 |
| FWER p-Value | 0.0 |
Table: GSEA Results Summary

  

Fig 1: Enrichment plot: TABULA\_MURIS\_SENIS\_MARROW\_GRANULOCYTOPOIETIC\_CELL\_AGEING      
 Profile of the Running ES Score & Positions of GeneSet Members on the Rank Ordered List

  

| SYMBOL | RANK IN GENE LIST | RANK METRIC SCORE | RUNNING ES | CORE ENRICHMENT || 1 | S100a8 | 38 | 3.013 | 0.0451 | Yes |
| 2 | Cybb | 57 | 2.654 | 0.0886 | Yes |
| 3 | Lyz1 | 73 | 2.460 | 0.1294 | Yes |
| 4 | Fcer1g | 76 | 2.415 | 0.1726 | Yes |
| 5 | S100a9 | 82 | 2.366 | 0.2141 | Yes |
| 6 | Tyrobp | 83 | 2.366 | 0.2569 | Yes |
| 7 | Apoe | 88 | 2.296 | 0.2975 | Yes |
| 8 | Emp3 | 115 | 2.020 | 0.3276 | Yes |
| 9 | Fth1 | 147 | 1.835 | 0.3531 | Yes |
| 10 | Fxyd5 | 157 | 1.767 | 0.3828 | Yes |
| 11 | Spi1 | 158 | 1.765 | 0.4147 | Yes |
| 12 | Gpsm3 | 169 | 1.725 | 0.4435 | Yes |
| 13 | Orm1 | 176 | 1.697 | 0.4727 | Yes |
| 14 | Arhgdib | 214 | 1.549 | 0.4915 | Yes |
| 15 | Hp | 286 | 1.351 | 0.4984 | Yes |
| 16 | Anxa1 | 290 | 1.344 | 0.5220 | Yes |
| 17 | Cd52 | 350 | 1.140 | 0.5280 | Yes |
| 18 | Lgals3 | 377 | 1.096 | 0.5414 | Yes |
| 19 | Alox5ap | 399 | 1.051 | 0.5552 | Yes |
| 20 | Lcn2 | 444 | 0.985 | 0.5622 | Yes |
| 21 | Ifitm2 | 465 | 0.952 | 0.5744 | Yes |
| 22 | Msrb1 | 472 | 0.945 | 0.5900 | Yes |
| 23 | Cyba | 519 | 0.875 | 0.5945 | Yes |
| 24 | Slpi | 558 | 0.836 | 0.6002 | Yes |
| 25 | 1810037I17Rik | 582 | 0.811 | 0.6092 | Yes |
| 26 | Cd63 | 632 | 0.727 | 0.6102 | Yes |
| 27 | Cdkn2d | 698 | 0.668 | 0.6063 | Yes |
| 28 | Spc25 | 704 | 0.664 | 0.6170 | Yes |
| 29 | Cdk1 | 724 | 0.651 | 0.6241 | Yes |
| 30 | Anxa2 | 760 | 0.620 | 0.6267 | Yes |
| 31 | Arrb2 | 786 | 0.600 | 0.6313 | Yes |
| 32 | Cks2 | 812 | 0.588 | 0.6358 | Yes |
| 33 | Rgcc | 822 | 0.579 | 0.6440 | Yes |
| 34 | AA467197 | 828 | 0.576 | 0.6532 | Yes |
| 35 | Hmbs | 1418 | -0.579 | 0.5181 | No |
| 36 | Reep5 | 1833 | -0.657 | 0.4277 | No |
| 37 | Ltf | 1834 | -0.657 | 0.4396 | No |
| 38 | Cebpd | 2002 | -0.693 | 0.4109 | No |
| 39 | Jchain | 2392 | -0.788 | 0.3290 | No |
| 40 | Bsg | 2530 | -0.827 | 0.3101 | No |
| 41 | Tmed3 | 2696 | -0.874 | 0.2851 | No |
| 42 | Ap3s1 | 2863 | -0.930 | 0.2609 | No |
| 43 | Cd177 | 4034 | -2.310 | 0.0136 | No |
Table: GSEA details [plain text format]

  

Fig 2: TABULA\_MURIS\_SENIS\_MARROW\_GRANULOCYTOPOIETIC\_CELL\_AGEING: Random ES distribution      
 Gene set null distribution of ES for **TABULA\_MURIS\_SENIS\_MARROW\_GRANULOCYTOPOIETIC\_CELL\_AGEING**

  
