# Supplementary material for: TMPRSS11B promotes an acidified microenvironment and immune suppression in squamous lung cancer
Source: EMBO Rep. 2025 Nov 10;26(24):6346–79. doi: 10.1038/s44319-025-00631-1 (PMC12714794; doi:10.1038/s44319-025-00631-1)
Supplement: Supplementary file 18 — Figure EV6 Source Data [file 44319_2025_631_MOESM18_ESM.zip › Figure EV6/EV6C-D/GSEA_Broad Institute_M8_T11b high vs low LUSC/TABULA_MURIS_SENIS_MARROW_HEMATOPOIETIC_PRECURSOR_CELL_AGEING.html]

Details for gene set TABULA\_MURIS\_SENIS\_MARROW\_HEMATOPOIETIC\_PRECURSOR\_CELL\_AGEING[GSEA]

|  || Dataset | T11b high vs low squamous\_GSEA\_Ranked |
| Phenotype | NoPhenotypeAvailable |
| Upregulated in class | na\_pos |
| GeneSet | TABULA\_MURIS\_SENIS\_MARROW\_HEMATOPOIETIC\_PRECURSOR\_CELL\_AGEING |
| Enrichment Score (ES) | 0.67113733 |
| Normalized Enrichment Score (NES) | 3.380071 |
| Nominal p-value | 0.0 |
| FDR q-value | 0.0 |
| FWER p-Value | 0.0 |
Table: GSEA Results Summary

  

Fig 1: Enrichment plot: TABULA\_MURIS\_SENIS\_MARROW\_HEMATOPOIETIC\_PRECURSOR\_CELL\_AGEING      
 Profile of the Running ES Score & Positions of GeneSet Members on the Rank Ordered List

  

| SYMBOL | RANK IN GENE LIST | RANK METRIC SCORE | RUNNING ES | CORE ENRICHMENT || 1 | S100a8 | 38 | 3.013 | 0.0585 | Yes |
| 2 | Itgb2 | 71 | 2.481 | 0.1066 | Yes |
| 3 | Lyz1 | 73 | 2.460 | 0.1618 | Yes |
| 4 | S100a9 | 82 | 2.366 | 0.2131 | Yes |
| 5 | Tyrobp | 83 | 2.366 | 0.2665 | Yes |
| 6 | Ly6a | 92 | 2.274 | 0.3157 | Yes |
| 7 | Glrx | 105 | 2.127 | 0.3607 | Yes |
| 8 | Ccl6 | 166 | 1.733 | 0.3850 | Yes |
| 9 | Gpsm3 | 169 | 1.725 | 0.4234 | Yes |
| 10 | Ctsb | 177 | 1.695 | 0.4599 | Yes |
| 11 | Hp | 286 | 1.351 | 0.4637 | Yes |
| 12 | Anxa1 | 290 | 1.344 | 0.4933 | Yes |
| 13 | Cd52 | 350 | 1.140 | 0.5044 | Yes |
| 14 | Bcl2a1b | 359 | 1.126 | 0.5278 | Yes |
| 15 | Lgals3 | 377 | 1.096 | 0.5483 | Yes |
| 16 | Alox5ap | 399 | 1.051 | 0.5668 | Yes |
| 17 | Vsir | 405 | 1.036 | 0.5889 | Yes |
| 18 | Lcn2 | 444 | 0.985 | 0.6018 | Yes |
| 19 | Msrb1 | 472 | 0.945 | 0.6164 | Yes |
| 20 | Cyba | 519 | 0.875 | 0.6248 | Yes |
| 21 | Slpi | 558 | 0.836 | 0.6342 | Yes |
| 22 | Ostf1 | 691 | 0.673 | 0.6168 | Yes |
| 23 | H2-D1 | 719 | 0.654 | 0.6249 | Yes |
| 24 | Slfn2 | 750 | 0.628 | 0.6317 | Yes |
| 25 | Anxa2 | 760 | 0.620 | 0.6434 | Yes |
| 26 | Sat1 | 817 | 0.584 | 0.6428 | Yes |
| 27 | Cd74 | 849 | 0.567 | 0.6479 | Yes |
| 28 | H2-K1 | 855 | 0.565 | 0.6594 | Yes |
| 29 | B2m | 860 | 0.563 | 0.6711 | Yes |
| 30 | H2-Ab1 | 915 | 0.525 | 0.6696 | No |
| 31 | Tmbim6 | 1599 | -0.611 | 0.5149 | No |
| 32 | Ltf | 1834 | -0.657 | 0.4720 | No |
| 33 | Rabac1 | 2744 | -0.888 | 0.2678 | No |
| 34 | Cd24a | 2759 | -0.893 | 0.2845 | No |
| 35 | Shisa5 | 2916 | -0.946 | 0.2673 | No |
| 36 | Cd82 | 2976 | -0.965 | 0.2745 | No |
Table: GSEA details [plain text format]

  

Fig 2: TABULA\_MURIS\_SENIS\_MARROW\_HEMATOPOIETIC\_PRECURSOR\_CELL\_AGEING: Random ES distribution      
 Gene set null distribution of ES for **TABULA\_MURIS\_SENIS\_MARROW\_HEMATOPOIETIC\_PRECURSOR\_CELL\_AGEING**

  
