# Supplementary material for: TMPRSS11B promotes an acidified microenvironment and immune suppression in squamous lung cancer
Source: EMBO Rep. 2025 Nov 10;26(24):6346–79. doi: 10.1038/s44319-025-00631-1 (PMC12714794; doi:10.1038/s44319-025-00631-1)
Supplement: Supplementary file 18 — Figure EV6 Source Data [file 44319_2025_631_MOESM18_ESM.zip › Figure EV6/EV6C-D/GSEA_Broad Institute_M8_T11b high vs low LUSC/TABULA_MURIS_SENIS_PANCREAS_PANCREATIC_BETA_CELL_AGEING.html]

Details for gene set TABULA\_MURIS\_SENIS\_PANCREAS\_PANCREATIC\_BETA\_CELL\_AGEING[GSEA]

|  || Dataset | T11b high vs low squamous\_GSEA\_Ranked |
| Phenotype | NoPhenotypeAvailable |
| Upregulated in class | na\_neg |
| GeneSet | TABULA\_MURIS\_SENIS\_PANCREAS\_PANCREATIC\_BETA\_CELL\_AGEING |
| Enrichment Score (ES) | -0.15054457 |
| Normalized Enrichment Score (NES) | -0.7651919 |
| Nominal p-value | 0.8056042 |
| FDR q-value | 1.0 |
| FWER p-Value | 1.0 |
Table: GSEA Results Summary

  

Fig 1: Enrichment plot: TABULA\_MURIS\_SENIS\_PANCREAS\_PANCREATIC\_BETA\_CELL\_AGEING      
 Profile of the Running ES Score & Positions of GeneSet Members on the Rank Ordered List

  

| SYMBOL | RANK IN GENE LIST | RANK METRIC SCORE | RUNNING ES | CORE ENRICHMENT || 1 | C1qa | 119 | 1.990 | 0.0110 | No |
| 2 | Csf2ra | 185 | 1.657 | 0.0287 | No |
| 3 | Dusp1 | 239 | 1.468 | 0.0454 | No |
| 4 | Psap | 240 | 1.466 | 0.0753 | No |
| 5 | Ckb | 267 | 1.414 | 0.0976 | No |
| 6 | Wfs1 | 310 | 1.262 | 0.1129 | No |
| 7 | Nupr1 | 443 | 0.988 | 0.1003 | No |
| 8 | Emilin1 | 456 | 0.969 | 0.1170 | No |
| 9 | Cebpb | 504 | 0.897 | 0.1237 | No |
| 10 | Actb | 633 | 0.726 | 0.1067 | No |
| 11 | Ece1 | 701 | 0.665 | 0.1037 | No |
| 12 | Prnp | 853 | 0.565 | 0.0778 | No |
| 13 | Matn2 | 876 | 0.554 | 0.0837 | No |
| 14 | Cfl1 | 895 | 0.538 | 0.0901 | No |
| 15 | Gns | 928 | 0.516 | 0.0927 | No |
| 16 | Gnb2 | 1007 | -0.506 | 0.0837 | No |
| 17 | Tshz1 | 1025 | -0.510 | 0.0899 | No |
| 18 | Ttc3 | 1068 | -0.517 | 0.0900 | No |
| 19 | Zc3h3 | 1105 | -0.523 | 0.0917 | No |
| 20 | Tuba1a | 1161 | -0.531 | 0.0889 | No |
| 21 | Madd | 1256 | -0.548 | 0.0768 | No |
| 22 | Arglu1 | 1260 | -0.549 | 0.0873 | No |
| 23 | Guk1 | 1444 | -0.584 | 0.0538 | No |
| 24 | Cmip | 1555 | -0.603 | 0.0389 | No |
| 25 | Wnt4 | 1655 | -0.622 | 0.0270 | No |
| 26 | Bet1l | 1666 | -0.625 | 0.0373 | No |
| 27 | Inpp4a | 1672 | -0.625 | 0.0487 | No |
| 28 | Tmed9 | 1675 | -0.626 | 0.0610 | No |
| 29 | Zfp516 | 1705 | -0.631 | 0.0666 | No |
| 30 | Tsc22d1 | 1718 | -0.633 | 0.0765 | No |
| 31 | Siah2 | 1781 | -0.644 | 0.0743 | No |
| 32 | Gmppa | 1893 | -0.672 | 0.0605 | No |
| 33 | Son | 2418 | -0.795 | -0.0530 | No |
| 34 | Hadh | 2449 | -0.804 | -0.0441 | No |
| 35 | Syt7 | 2642 | -0.859 | -0.0741 | No |
| 36 | Rbm39 | 2864 | -0.930 | -0.1099 | No |
| 37 | Ncoa1 | 2940 | -0.953 | -0.1091 | No |
| 38 | Selenos | 2961 | -0.960 | -0.0945 | No |
| 39 | Grk3 | 3015 | -0.981 | -0.0877 | No |
| 40 | Fos | 3189 | -1.058 | -0.1090 | No |
| 41 | Prkca | 3358 | -1.142 | -0.1273 | Yes |
| 42 | Efna5 | 3407 | -1.163 | -0.1155 | Yes |
| 43 | Egr1 | 3532 | -1.234 | -0.1211 | Yes |
| 44 | Echdc2 | 3645 | -1.326 | -0.1219 | Yes |
| 45 | Cela1 | 3666 | -1.349 | -0.0993 | Yes |
| 46 | Ttc28 | 3790 | -1.490 | -0.0995 | Yes |
| 47 | Chka | 3801 | -1.515 | -0.0711 | Yes |
| 48 | Ptprn2 | 3961 | -1.882 | -0.0722 | Yes |
| 49 | Rab3a | 4023 | -2.268 | -0.0411 | Yes |
| 50 | Clps | 4075 | -2.812 | 0.0035 | Yes |
Table: GSEA details [plain text format]

  

Fig 2: TABULA\_MURIS\_SENIS\_PANCREAS\_PANCREATIC\_BETA\_CELL\_AGEING: Random ES distribution      
 Gene set null distribution of ES for **TABULA\_MURIS\_SENIS\_PANCREAS\_PANCREATIC\_BETA\_CELL\_AGEING**

  
