# Supplementary material for: TMPRSS11B promotes an acidified microenvironment and immune suppression in squamous lung cancer
Source: EMBO Rep. 2025 Nov 10;26(24):6346–79. doi: 10.1038/s44319-025-00631-1 (PMC12714794; doi:10.1038/s44319-025-00631-1)
Supplement: Supplementary file 18 — Figure EV6 Source Data [file 44319_2025_631_MOESM18_ESM.zip › Figure EV6/EV6C-D/GSEA_Broad Institute_M8_T11b high vs low LUSC/TABULA_MURIS_SENIS_PANCREAS_PANCREATIC_DELTA_CELL_AGEING.html]

Details for gene set TABULA\_MURIS\_SENIS\_PANCREAS\_PANCREATIC\_DELTA\_CELL\_AGEING[GSEA]

|  || Dataset | T11b high vs low squamous\_GSEA\_Ranked |
| Phenotype | NoPhenotypeAvailable |
| Upregulated in class | na\_neg |
| GeneSet | TABULA\_MURIS\_SENIS\_PANCREAS\_PANCREATIC\_DELTA\_CELL\_AGEING |
| Enrichment Score (ES) | -0.09531735 |
| Normalized Enrichment Score (NES) | -0.5157389 |
| Nominal p-value | 0.9817276 |
| FDR q-value | 1.0 |
| FWER p-Value | 1.0 |
Table: GSEA Results Summary

  

Fig 1: Enrichment plot: TABULA\_MURIS\_SENIS\_PANCREAS\_PANCREATIC\_DELTA\_CELL\_AGEING      
 Profile of the Running ES Score & Positions of GeneSet Members on the Rank Ordered List

  

| SYMBOL | RANK IN GENE LIST | RANK METRIC SCORE | RUNNING ES | CORE ENRICHMENT || 1 | C1qa | 119 | 1.990 | 0.0067 | No |
| 2 | Lgmn | 141 | 1.877 | 0.0356 | No |
| 3 | Dusp1 | 239 | 1.468 | 0.0382 | No |
| 4 | Ckb | 267 | 1.414 | 0.0573 | No |
| 5 | Sult2b1 | 497 | 0.901 | 0.0168 | No |
| 6 | Dnajb1 | 534 | 0.862 | 0.0236 | No |
| 7 | Nnat | 578 | 0.815 | 0.0277 | No |
| 8 | Il11ra1 | 602 | 0.771 | 0.0360 | No |
| 9 | Cd63 | 632 | 0.727 | 0.0421 | No |
| 10 | Cotl1 | 656 | 0.709 | 0.0493 | No |
| 11 | Ece1 | 701 | 0.665 | 0.0504 | No |
| 12 | Ssr1 | 710 | 0.661 | 0.0605 | No |
| 13 | Tubb2b | 756 | 0.624 | 0.0606 | No |
| 14 | Ctnnbip1 | 791 | 0.597 | 0.0631 | No |
| 15 | Cldn4 | 811 | 0.589 | 0.0691 | No |
| 16 | Cfl1 | 895 | 0.538 | 0.0582 | No |
| 17 | Dpysl2 | 910 | 0.527 | 0.0643 | No |
| 18 | Wipi1 | 941 | 0.510 | 0.0662 | No |
| 19 | Rtn4 | 946 | 0.508 | 0.0744 | No |
| 20 | Elof1 | 1092 | -0.521 | 0.0479 | No |
| 21 | Mapre3 | 1099 | -0.522 | 0.0559 | No |
| 22 | Tmem59 | 1117 | -0.525 | 0.0613 | No |
| 23 | Txnl4a | 1151 | -0.530 | 0.0627 | No |
| 24 | Prrg2 | 1221 | -0.544 | 0.0555 | No |
| 25 | Arglu1 | 1260 | -0.549 | 0.0560 | No |
| 26 | Alkbh6 | 1261 | -0.549 | 0.0660 | No |
| 27 | Fxyd6 | 1382 | -0.571 | 0.0466 | No |
| 28 | Pick1 | 1384 | -0.571 | 0.0567 | No |
| 29 | Krt8 | 1410 | -0.578 | 0.0611 | No |
| 30 | Fam241b | 1461 | -0.586 | 0.0593 | No |
| 31 | Wdfy1 | 1480 | -0.589 | 0.0656 | No |
| 32 | Inpp4a | 1672 | -0.625 | 0.0295 | No |
| 33 | Tmed9 | 1675 | -0.626 | 0.0404 | No |
| 34 | Tsc22d1 | 1718 | -0.633 | 0.0415 | No |
| 35 | Mospd3 | 2046 | -0.703 | -0.0269 | No |
| 36 | Gstm1 | 2049 | -0.704 | -0.0146 | No |
| 37 | Ddhd2 | 2180 | -0.735 | -0.0335 | No |
| 38 | Depp1 | 2188 | -0.736 | -0.0218 | No |
| 39 | Nsmce3 | 2277 | -0.756 | -0.0299 | No |
| 40 | Hook2 | 2334 | -0.771 | -0.0298 | No |
| 41 | Zfyve21 | 2335 | -0.771 | -0.0158 | No |
| 42 | Hadh | 2449 | -0.804 | -0.0292 | No |
| 43 | Bsg | 2530 | -0.827 | -0.0340 | No |
| 44 | Sod1 | 2691 | -0.874 | -0.0578 | No |
| 45 | Tmem263 | 2843 | -0.920 | -0.0786 | Yes |
| 46 | Rbm39 | 2864 | -0.930 | -0.0666 | Yes |
| 47 | Smco4 | 2881 | -0.934 | -0.0536 | Yes |
| 48 | Selenos | 2961 | -0.960 | -0.0557 | Yes |
| 49 | Mettl26 | 2965 | -0.961 | -0.0390 | Yes |
| 50 | Mob2 | 3132 | -1.032 | -0.0614 | Yes |
| 51 | Lgals3bp | 3172 | -1.049 | -0.0520 | Yes |
| 52 | Fos | 3189 | -1.058 | -0.0367 | Yes |
| 53 | Sil1 | 3241 | -1.088 | -0.0296 | Yes |
| 54 | Ogfod3 | 3357 | -1.142 | -0.0374 | Yes |
| 55 | Pdia5 | 3468 | -1.197 | -0.0429 | Yes |
| 56 | Egr1 | 3532 | -1.234 | -0.0361 | Yes |
| 57 | Ccdc92 | 3565 | -1.257 | -0.0212 | Yes |
| 58 | Eri3 | 3605 | -1.290 | -0.0074 | Yes |
| 59 | Cela1 | 3666 | -1.349 | 0.0023 | Yes |
| 60 | Nadsyn1 | 3683 | -1.364 | 0.0231 | Yes |
| 61 | Ptov1 | 3741 | -1.430 | 0.0350 | Yes |
| 62 | Clps | 4075 | -2.812 | 0.0035 | Yes |
Table: GSEA details [plain text format]

  

Fig 2: TABULA\_MURIS\_SENIS\_PANCREAS\_PANCREATIC\_DELTA\_CELL\_AGEING: Random ES distribution      
 Gene set null distribution of ES for **TABULA\_MURIS\_SENIS\_PANCREAS\_PANCREATIC\_DELTA\_CELL\_AGEING**

  
