# Supplementary material for: TMPRSS11B promotes an acidified microenvironment and immune suppression in squamous lung cancer
Source: EMBO Rep. 2025 Nov 10;26(24):6346–79. doi: 10.1038/s44319-025-00631-1 (PMC12714794; doi:10.1038/s44319-025-00631-1)
Supplement: Supplementary file 18 — Figure EV6 Source Data [file 44319_2025_631_MOESM18_ESM.zip › Figure EV6/EV6C-D/GSEA_Broad Institute_M8_T11b high vs low LUSC/TABULA_MURIS_SENIS_PANCREAS_PANCREATIC_DUCTAL_CELL_AGEING.html]

Details for gene set TABULA\_MURIS\_SENIS\_PANCREAS\_PANCREATIC\_DUCTAL\_CELL\_AGEING[GSEA]

|  || Dataset | T11b high vs low squamous\_GSEA\_Ranked |
| Phenotype | NoPhenotypeAvailable |
| Upregulated in class | na\_neg |
| GeneSet | TABULA\_MURIS\_SENIS\_PANCREAS\_PANCREATIC\_DUCTAL\_CELL\_AGEING |
| Enrichment Score (ES) | -0.15447353 |
| Normalized Enrichment Score (NES) | -0.90142715 |
| Nominal p-value | 0.6483871 |
| FDR q-value | 1.0 |
| FWER p-Value | 1.0 |
Table: GSEA Results Summary

  

Fig 1: Enrichment plot: TABULA\_MURIS\_SENIS\_PANCREAS\_PANCREATIC\_DUCTAL\_CELL\_AGEING      
 Profile of the Running ES Score & Positions of GeneSet Members on the Rank Ordered List

  

| SYMBOL | RANK IN GENE LIST | RANK METRIC SCORE | RUNNING ES | CORE ENRICHMENT || 1 | Ly6a | 92 | 2.274 | 0.0053 | No |
| 2 | Fth1 | 147 | 1.835 | 0.0146 | No |
| 3 | Csf2ra | 185 | 1.657 | 0.0260 | No |
| 4 | Cryab | 422 | 1.017 | -0.0204 | No |
| 5 | Nupr1 | 443 | 0.988 | -0.0131 | No |
| 6 | Lcn2 | 444 | 0.985 | -0.0008 | No |
| 7 | Ifitm2 | 465 | 0.952 | 0.0060 | No |
| 8 | Cyba | 519 | 0.875 | 0.0036 | No |
| 9 | Gadd45b | 522 | 0.873 | 0.0140 | No |
| 10 | Tmem37 | 535 | 0.861 | 0.0217 | No |
| 11 | Oaz2 | 610 | 0.756 | 0.0126 | No |
| 12 | Cd63 | 632 | 0.727 | 0.0164 | No |
| 13 | Ddah2 | 658 | 0.706 | 0.0189 | No |
| 14 | Igfbp7 | 711 | 0.661 | 0.0142 | No |
| 15 | H2-D1 | 719 | 0.654 | 0.0205 | No |
| 16 | S100a16 | 775 | 0.605 | 0.0143 | No |
| 17 | Sat1 | 817 | 0.584 | 0.0113 | No |
| 18 | Sfn | 834 | 0.573 | 0.0145 | No |
| 19 | H2-K1 | 855 | 0.565 | 0.0165 | No |
| 20 | B2m | 860 | 0.563 | 0.0225 | No |
| 21 | Cfl1 | 895 | 0.538 | 0.0207 | No |
| 22 | Rbp1 | 924 | 0.518 | 0.0201 | No |
| 23 | Elof1 | 1092 | -0.521 | -0.0151 | No |
| 24 | Tmem59 | 1117 | -0.525 | -0.0146 | No |
| 25 | Kdsr | 1141 | -0.529 | -0.0138 | No |
| 26 | Nr2c2ap | 1434 | -0.582 | -0.0795 | No |
| 27 | Ift27 | 1439 | -0.583 | -0.0732 | No |
| 28 | Cldn3 | 1495 | -0.593 | -0.0796 | No |
| 29 | Ppa1 | 1606 | -0.612 | -0.0995 | No |
| 30 | Ppp1r35 | 1611 | -0.612 | -0.0929 | No |
| 31 | Inpp4a | 1672 | -0.625 | -0.1001 | No |
| 32 | Tmed9 | 1675 | -0.626 | -0.0928 | No |
| 33 | Tsc22d1 | 1718 | -0.633 | -0.0954 | No |
| 34 | 2610528J11Rik | 1763 | -0.642 | -0.0984 | No |
| 35 | Ifi27 | 1778 | -0.644 | -0.0939 | No |
| 36 | Tcf7l2 | 1816 | -0.651 | -0.0951 | No |
| 37 | Reep5 | 1833 | -0.657 | -0.0909 | No |
| 38 | Bcl7c | 1842 | -0.659 | -0.0847 | No |
| 39 | Arl3 | 1918 | -0.678 | -0.0950 | No |
| 40 | Nectin2 | 1952 | -0.686 | -0.0948 | No |
| 41 | Vps72 | 2028 | -0.697 | -0.1048 | No |
| 42 | Gstm1 | 2049 | -0.704 | -0.1011 | No |
| 43 | Gadd45gip1 | 2067 | -0.712 | -0.0965 | No |
| 44 | Kdelr1 | 2186 | -0.736 | -0.1168 | No |
| 45 | Cdc42ep5 | 2192 | -0.737 | -0.1089 | No |
| 46 | Spr | 2241 | -0.749 | -0.1116 | No |
| 47 | Kpna4 | 2243 | -0.749 | -0.1025 | No |
| 48 | Thap3 | 2344 | -0.773 | -0.1179 | No |
| 49 | Cic | 2411 | -0.794 | -0.1245 | No |
| 50 | Bsg | 2530 | -0.827 | -0.1437 | Yes |
| 51 | Tmem176b | 2558 | -0.834 | -0.1401 | Yes |
| 52 | Dynll2 | 2574 | -0.841 | -0.1334 | Yes |
| 53 | Cbx7 | 2587 | -0.844 | -0.1259 | Yes |
| 54 | Spag7 | 2645 | -0.860 | -0.1294 | Yes |
| 55 | Sod1 | 2691 | -0.874 | -0.1298 | Yes |
| 56 | Zfp704 | 2735 | -0.886 | -0.1295 | Yes |
| 57 | Jag1 | 2743 | -0.887 | -0.1202 | Yes |
| 58 | Smco4 | 2881 | -0.934 | -0.1429 | Yes |
| 59 | Paip1 | 2913 | -0.944 | -0.1389 | Yes |
| 60 | Commd10 | 2934 | -0.952 | -0.1320 | Yes |
| 61 | Cfap298 | 2937 | -0.953 | -0.1207 | Yes |
| 62 | Bri3 | 2942 | -0.954 | -0.1098 | Yes |
| 63 | Selenos | 2961 | -0.960 | -0.1024 | Yes |
| 64 | Igsf5 | 3091 | -1.015 | -0.1220 | Yes |
| 65 | Gstm2 | 3116 | -1.026 | -0.1153 | Yes |
| 66 | Lgals3bp | 3172 | -1.049 | -0.1160 | Yes |
| 67 | Fos | 3189 | -1.058 | -0.1068 | Yes |
| 68 | Ssbp3 | 3192 | -1.060 | -0.0941 | Yes |
| 69 | Spry2 | 3254 | -1.095 | -0.0958 | Yes |
| 70 | Marf1 | 3288 | -1.108 | -0.0902 | Yes |
| 71 | Smim19 | 3329 | -1.127 | -0.0862 | Yes |
| 72 | Macrod1 | 3332 | -1.127 | -0.0727 | Yes |
| 73 | Sf3b4 | 3336 | -1.127 | -0.0594 | Yes |
| 74 | Fuz | 3428 | -1.175 | -0.0676 | Yes |
| 75 | Nr2f2 | 3450 | -1.187 | -0.0581 | Yes |
| 76 | Gsta3 | 3465 | -1.196 | -0.0467 | Yes |
| 77 | Zfp787 | 3490 | -1.205 | -0.0377 | Yes |
| 78 | Aldh2 | 3521 | -1.226 | -0.0300 | Yes |
| 79 | Tmem176a | 3571 | -1.266 | -0.0265 | Yes |
| 80 | Chd6 | 3576 | -1.268 | -0.0117 | Yes |
| 81 | Echdc2 | 3645 | -1.326 | -0.0122 | Yes |
| 82 | Adh1 | 3681 | -1.362 | -0.0040 | Yes |
| 83 | Arhgap44 | 3756 | -1.446 | -0.0045 | Yes |
| 84 | Qsox1 | 3787 | -1.486 | 0.0064 | Yes |
| 85 | Lmo4 | 3802 | -1.515 | 0.0218 | Yes |
| 86 | Lrrc75a | 3887 | -1.679 | 0.0217 | Yes |
| 87 | Capsl | 4029 | -2.297 | 0.0150 | Yes |
Table: GSEA details [plain text format]

  

Fig 2: TABULA\_MURIS\_SENIS\_PANCREAS\_PANCREATIC\_DUCTAL\_CELL\_AGEING: Random ES distribution      
 Gene set null distribution of ES for **TABULA\_MURIS\_SENIS\_PANCREAS\_PANCREATIC\_DUCTAL\_CELL\_AGEING**

  
