# Supplementary material for: TMPRSS11B promotes an acidified microenvironment and immune suppression in squamous lung cancer
Source: EMBO Rep. 2025 Nov 10;26(24):6346–79. doi: 10.1038/s44319-025-00631-1 (PMC12714794; doi:10.1038/s44319-025-00631-1)
Supplement: Supplementary file 18 — Figure EV6 Source Data [file 44319_2025_631_MOESM18_ESM.zip › Figure EV6/EV6C-D/GSEA_Broad Institute_M8_T11b high vs low LUSC/TABULA_MURIS_SENIS_PANCREAS_PANCREATIC_POLYPEPTIDE_CELL_AGEING.html]

Details for gene set TABULA\_MURIS\_SENIS\_PANCREAS\_PANCREATIC\_POLYPEPTIDE\_CELL\_AGEING[GSEA]

|  || Dataset | T11b high vs low squamous\_GSEA\_Ranked |
| Phenotype | NoPhenotypeAvailable |
| Upregulated in class | na\_neg |
| GeneSet | TABULA\_MURIS\_SENIS\_PANCREAS\_PANCREATIC\_POLYPEPTIDE\_CELL\_AGEING |
| Enrichment Score (ES) | -0.21931215 |
| Normalized Enrichment Score (NES) | -1.0368735 |
| Nominal p-value | 0.40946168 |
| FDR q-value | 1.0 |
| FWER p-Value | 1.0 |
Table: GSEA Results Summary

  

Fig 1: Enrichment plot: TABULA\_MURIS\_SENIS\_PANCREAS\_PANCREATIC\_POLYPEPTIDE\_CELL\_AGEING      
 Profile of the Running ES Score & Positions of GeneSet Members on the Rank Ordered List

  

| SYMBOL | RANK IN GENE LIST | RANK METRIC SCORE | RUNNING ES | CORE ENRICHMENT || 1 | Dusp1 | 239 | 1.468 | -0.0160 | No |
| 2 | Ifitm2 | 465 | 0.952 | -0.0437 | No |
| 3 | Prelid1 | 506 | 0.892 | -0.0275 | No |
| 4 | Dnajb1 | 534 | 0.862 | -0.0090 | No |
| 5 | Cotl1 | 656 | 0.709 | -0.0181 | No |
| 6 | Cfl1 | 895 | 0.538 | -0.0611 | No |
| 7 | Chd1 | 1107 | -0.524 | -0.0978 | No |
| 8 | Ctnnbl1 | 1119 | -0.526 | -0.0852 | No |
| 9 | Trappc5 | 1177 | -0.536 | -0.0836 | No |
| 10 | Gal3st1 | 1204 | -0.542 | -0.0741 | No |
| 11 | Emc10 | 1234 | -0.546 | -0.0653 | No |
| 12 | Alkbh6 | 1261 | -0.549 | -0.0557 | No |
| 13 | Mmadhc | 1458 | -0.586 | -0.0869 | No |
| 14 | Ppa1 | 1606 | -0.612 | -0.1053 | No |
| 15 | Tsc22d1 | 1718 | -0.633 | -0.1142 | No |
| 16 | Ppa2 | 1908 | -0.675 | -0.1411 | No |
| 17 | Mospd3 | 2046 | -0.703 | -0.1543 | No |
| 18 | Kdelr1 | 2186 | -0.736 | -0.1671 | No |
| 19 | Ilkap | 2269 | -0.755 | -0.1653 | No |
| 20 | Casz1 | 2489 | -0.817 | -0.1954 | Yes |
| 21 | Lsr | 2522 | -0.825 | -0.1792 | Yes |
| 22 | Bsg | 2530 | -0.827 | -0.1567 | Yes |
| 23 | Agtrap | 2752 | -0.889 | -0.1853 | Yes |
| 24 | Tmem219 | 2763 | -0.894 | -0.1616 | Yes |
| 25 | Klc3 | 2867 | -0.930 | -0.1598 | Yes |
| 26 | Shisa5 | 2916 | -0.946 | -0.1440 | Yes |
| 27 | Cfap298 | 2937 | -0.953 | -0.1210 | Yes |
| 28 | Selenos | 2961 | -0.960 | -0.0986 | Yes |
| 29 | Zbtb7c | 3012 | -0.981 | -0.0823 | Yes |
| 30 | Pofut1 | 3080 | -1.012 | -0.0692 | Yes |
| 31 | Sdr39u1 | 3150 | -1.038 | -0.0559 | Yes |
| 32 | Fos | 3189 | -1.058 | -0.0344 | Yes |
| 33 | Bace2 | 3461 | -1.193 | -0.0664 | Yes |
| 34 | Erlec1 | 3504 | -1.214 | -0.0412 | Yes |
| 35 | Lrig1 | 3543 | -1.241 | -0.0143 | Yes |
| 36 | Rab3a | 4023 | -2.268 | -0.0662 | Yes |
| 37 | Clps | 4075 | -2.812 | 0.0035 | Yes |
Table: GSEA details [plain text format]

  

Fig 2: TABULA\_MURIS\_SENIS\_PANCREAS\_PANCREATIC\_POLYPEPTIDE\_CELL\_AGEING: Random ES distribution      
 Gene set null distribution of ES for **TABULA\_MURIS\_SENIS\_PANCREAS\_PANCREATIC\_POLYPEPTIDE\_CELL\_AGEING**

  
