# Supplementary material for: TMPRSS11B promotes an acidified microenvironment and immune suppression in squamous lung cancer
Source: EMBO Rep. 2025 Nov 10;26(24):6346–79. doi: 10.1038/s44319-025-00631-1 (PMC12714794; doi:10.1038/s44319-025-00631-1)
Supplement: Supplementary file 18 — Figure EV6 Source Data [file 44319_2025_631_MOESM18_ESM.zip › Figure EV6/EV6C-D/GSEA_Broad Institute_M8_T11b high vs low LUSC/TABULA_MURIS_SENIS_SPLEEN_B_CELL_AGEING.html]

Details for gene set TABULA\_MURIS\_SENIS\_SPLEEN\_B\_CELL\_AGEING[GSEA]

|  || Dataset | T11b high vs low squamous\_GSEA\_Ranked |
| Phenotype | NoPhenotypeAvailable |
| Upregulated in class | na\_pos |
| GeneSet | TABULA\_MURIS\_SENIS\_SPLEEN\_B\_CELL\_AGEING |
| Enrichment Score (ES) | 0.6133975 |
| Normalized Enrichment Score (NES) | 3.0292358 |
| Nominal p-value | 0.0 |
| FDR q-value | 0.0 |
| FWER p-Value | 0.0 |
Table: GSEA Results Summary

  

Fig 1: Enrichment plot: TABULA\_MURIS\_SENIS\_SPLEEN\_B\_CELL\_AGEING      
 Profile of the Running ES Score & Positions of GeneSet Members on the Rank Ordered List

  

| SYMBOL | RANK IN GENE LIST | RANK METRIC SCORE | RUNNING ES | CORE ENRICHMENT || 1 | S100a8 | 38 | 3.013 | 0.0575 | Yes |
| 2 | Evi2a | 52 | 2.706 | 0.1143 | Yes |
| 3 | Cybb | 57 | 2.654 | 0.1722 | Yes |
| 4 | Fcgr2b | 62 | 2.610 | 0.2291 | Yes |
| 5 | S100a9 | 82 | 2.366 | 0.2769 | Yes |
| 6 | Apoe | 88 | 2.296 | 0.3266 | Yes |
| 7 | Ly6a | 92 | 2.274 | 0.3763 | Yes |
| 8 | Lgals1 | 98 | 2.178 | 0.4234 | Yes |
| 9 | Emp3 | 115 | 2.020 | 0.4643 | Yes |
| 10 | Fxyd5 | 157 | 1.767 | 0.4934 | Yes |
| 11 | Psap | 240 | 1.466 | 0.5057 | Yes |
| 12 | Srgn | 270 | 1.392 | 0.5294 | Yes |
| 13 | Syk | 278 | 1.375 | 0.5582 | Yes |
| 14 | Capg | 343 | 1.160 | 0.5682 | Yes |
| 15 | Bcl2a1b | 359 | 1.126 | 0.5895 | Yes |
| 16 | Npc2 | 536 | 0.861 | 0.5652 | Yes |
| 17 | Cd44 | 562 | 0.834 | 0.5775 | Yes |
| 18 | Txn1 | 586 | 0.800 | 0.5896 | Yes |
| 19 | Crip1 | 638 | 0.721 | 0.5930 | Yes |
| 20 | Lat2 | 714 | 0.657 | 0.5891 | Yes |
| 21 | H2-D1 | 719 | 0.654 | 0.6026 | Yes |
| 22 | Sat1 | 817 | 0.584 | 0.5917 | Yes |
| 23 | H2-K1 | 855 | 0.565 | 0.5951 | Yes |
| 24 | B2m | 860 | 0.563 | 0.6066 | Yes |
| 25 | H2-Ab1 | 915 | 0.525 | 0.6049 | Yes |
| 26 | Gns | 928 | 0.516 | 0.6134 | Yes |
| 27 | Txndc5 | 1059 | -0.515 | 0.5928 | No |
| 28 | Nap1l1 | 1883 | -0.670 | 0.4047 | No |
| 29 | Tcf4 | 2163 | -0.732 | 0.3522 | No |
| 30 | Jchain | 2392 | -0.788 | 0.3134 | No |
| 31 | Rabac1 | 2744 | -0.888 | 0.2466 | No |
| 32 | Fos | 3189 | -1.058 | 0.1606 | No |
| 33 | Zbtb20 | 3395 | -1.159 | 0.1358 | No |
| 34 | Tnfaip8 | 3837 | -1.581 | 0.0621 | No |
Table: GSEA details [plain text format]

  

Fig 2: TABULA\_MURIS\_SENIS\_SPLEEN\_B\_CELL\_AGEING: Random ES distribution      
 Gene set null distribution of ES for **TABULA\_MURIS\_SENIS\_SPLEEN\_B\_CELL\_AGEING**

  
