# Supplementary material for: TMPRSS11B promotes an acidified microenvironment and immune suppression in squamous lung cancer
Source: EMBO Rep. 2025 Nov 10;26(24):6346–79. doi: 10.1038/s44319-025-00631-1 (PMC12714794; doi:10.1038/s44319-025-00631-1)
Supplement: Supplementary file 18 — Figure EV6 Source Data [file 44319_2025_631_MOESM18_ESM.zip › Figure EV6/EV6C-D/GSEA_Broad Institute_M8_T11b high vs low LUSC/TABULA_MURIS_SENIS_SPLEEN_MACROPHAGE_AGEING.html]

Details for gene set TABULA\_MURIS\_SENIS\_SPLEEN\_MACROPHAGE\_AGEING[GSEA]

|  || Dataset | T11b high vs low squamous\_GSEA\_Ranked |
| Phenotype | NoPhenotypeAvailable |
| Upregulated in class | na\_pos |
| GeneSet | TABULA\_MURIS\_SENIS\_SPLEEN\_MACROPHAGE\_AGEING |
| Enrichment Score (ES) | 0.6750092 |
| Normalized Enrichment Score (NES) | 3.8532424 |
| Nominal p-value | 0.0 |
| FDR q-value | 0.0 |
| FWER p-Value | 0.0 |
Table: GSEA Results Summary

  

Fig 1: Enrichment plot: TABULA\_MURIS\_SENIS\_SPLEEN\_MACROPHAGE\_AGEING      
 Profile of the Running ES Score & Positions of GeneSet Members on the Rank Ordered List

  

| SYMBOL | RANK IN GENE LIST | RANK METRIC SCORE | RUNNING ES | CORE ENRICHMENT || 1 | Ppbp | 5 | 5.111 | 0.0641 | Yes |
| 2 | S100a8 | 38 | 3.013 | 0.0947 | Yes |
| 3 | Cd300c2 | 42 | 2.822 | 0.1301 | Yes |
| 4 | Cybb | 57 | 2.654 | 0.1605 | Yes |
| 5 | Ctss | 63 | 2.582 | 0.1923 | Yes |
| 6 | Lyz1 | 73 | 2.460 | 0.2215 | Yes |
| 7 | Fcer1g | 76 | 2.415 | 0.2519 | Yes |
| 8 | S100a9 | 82 | 2.366 | 0.2809 | Yes |
| 9 | Apoe | 88 | 2.296 | 0.3091 | Yes |
| 10 | Ly6a | 92 | 2.274 | 0.3374 | Yes |
| 11 | Lgals1 | 98 | 2.178 | 0.3640 | Yes |
| 12 | Mpeg1 | 114 | 2.045 | 0.3865 | Yes |
| 13 | Wfdc17 | 117 | 1.998 | 0.4115 | Yes |
| 14 | C1qa | 119 | 1.990 | 0.4367 | Yes |
| 15 | Il1b | 129 | 1.912 | 0.4589 | Yes |
| 16 | C1qb | 139 | 1.882 | 0.4808 | Yes |
| 17 | Lgmn | 141 | 1.877 | 0.5045 | Yes |
| 18 | Fth1 | 147 | 1.835 | 0.5268 | Yes |
| 19 | Ctsd | 150 | 1.830 | 0.5497 | Yes |
| 20 | Cfp | 171 | 1.717 | 0.5667 | Yes |
| 21 | Ctsb | 177 | 1.695 | 0.5871 | Yes |
| 22 | C1qc | 234 | 1.480 | 0.5922 | Yes |
| 23 | Acp5 | 247 | 1.450 | 0.6077 | Yes |
| 24 | Creg1 | 280 | 1.373 | 0.6174 | Yes |
| 25 | Grn | 285 | 1.352 | 0.6337 | Yes |
| 26 | Fabp5 | 385 | 1.088 | 0.6230 | Yes |
| 27 | Csf1r | 414 | 1.025 | 0.6292 | Yes |
| 28 | Ifitm2 | 465 | 0.952 | 0.6290 | Yes |
| 29 | Flna | 471 | 0.946 | 0.6398 | Yes |
| 30 | Msrb1 | 472 | 0.945 | 0.6519 | Yes |
| 31 | Npc2 | 536 | 0.861 | 0.6473 | Yes |
| 32 | Slpi | 558 | 0.836 | 0.6528 | Yes |
| 33 | Txn1 | 586 | 0.800 | 0.6563 | Yes |
| 34 | Atp6v0e | 590 | 0.795 | 0.6658 | Yes |
| 35 | Trf | 601 | 0.771 | 0.6731 | Yes |
| 36 | Cd63 | 632 | 0.727 | 0.6750 | Yes |
| 37 | Gng11 | 677 | 0.687 | 0.6729 | No |
| 38 | Atp1b3 | 745 | 0.632 | 0.6644 | No |
| 39 | Sat1 | 817 | 0.584 | 0.6543 | No |
| 40 | Litaf | 856 | 0.564 | 0.6521 | No |
| 41 | Psma5 | 879 | 0.552 | 0.6537 | No |
| 42 | Cmtm7 | 1228 | -0.545 | 0.5744 | No |
| 43 | Plaat3 | 1603 | -0.611 | 0.4896 | No |
| 44 | Reep5 | 1833 | -0.657 | 0.4412 | No |
| 45 | P4hb | 1926 | -0.681 | 0.4271 | No |
| 46 | Selenop | 2054 | -0.707 | 0.4047 | No |
| 47 | Jchain | 2392 | -0.788 | 0.3313 | No |
| 48 | Tmem176b | 2558 | -0.834 | 0.3011 | No |
| 49 | Sod1 | 2691 | -0.874 | 0.2795 | No |
| 50 | Tmed3 | 2696 | -0.874 | 0.2897 | No |
| 51 | Lamp2 | 2707 | -0.877 | 0.2985 | No |
| 52 | Rabac1 | 2744 | -0.888 | 0.3009 | No |
| 53 | Aldh2 | 3521 | -1.226 | 0.1243 | No |
| 54 | Tmem176a | 3571 | -1.266 | 0.1283 | No |
Table: GSEA details [plain text format]

  

Fig 2: TABULA\_MURIS\_SENIS\_SPLEEN\_MACROPHAGE\_AGEING: Random ES distribution      
 Gene set null distribution of ES for **TABULA\_MURIS\_SENIS\_SPLEEN\_MACROPHAGE\_AGEING**

  
