# Supplementary material for: TMPRSS11B promotes an acidified microenvironment and immune suppression in squamous lung cancer
Source: EMBO Rep. 2025 Nov 10;26(24):6346–79. doi: 10.1038/s44319-025-00631-1 (PMC12714794; doi:10.1038/s44319-025-00631-1)
Supplement: Supplementary file 18 — Figure EV6 Source Data [file 44319_2025_631_MOESM18_ESM.zip › Figure EV6/EV6C-D/GSEA_Broad Institute_M8_T11b high vs low LUSC/TABULA_MURIS_SENIS_SUBCUTANEOUS_ADIPOSE_TISSUE_MYELOID_CELL_AGEING.html]

Details for gene set TABULA\_MURIS\_SENIS\_SUBCUTANEOUS\_ADIPOSE\_TISSUE\_MYELOID\_CELL\_AGEING[GSEA]

|  || Dataset | T11b high vs low squamous\_GSEA\_Ranked |
| Phenotype | NoPhenotypeAvailable |
| Upregulated in class | na\_pos |
| GeneSet | TABULA\_MURIS\_SENIS\_SUBCUTANEOUS\_ADIPOSE\_TISSUE\_MYELOID\_CELL\_AGEING |
| Enrichment Score (ES) | 0.5094662 |
| Normalized Enrichment Score (NES) | 3.0058424 |
| Nominal p-value | 0.0 |
| FDR q-value | 0.0 |
| FWER p-Value | 0.0 |
Table: GSEA Results Summary

  

Fig 1: Enrichment plot: TABULA\_MURIS\_SENIS\_SUBCUTANEOUS\_ADIPOSE\_TISSUE\_MYELOID\_CELL\_AGEING      
 Profile of the Running ES Score & Positions of GeneSet Members on the Rank Ordered List

  

| SYMBOL | RANK IN GENE LIST | RANK METRIC SCORE | RUNNING ES | CORE ENRICHMENT || 1 | S100a8 | 38 | 3.013 | 0.0317 | Yes |
| 2 | Gngt2 | 51 | 2.724 | 0.0659 | Yes |
| 3 | Cybb | 57 | 2.654 | 0.1008 | Yes |
| 4 | S100a9 | 82 | 2.366 | 0.1272 | Yes |
| 5 | Il1b | 129 | 1.912 | 0.1418 | Yes |
| 6 | Spi1 | 158 | 1.765 | 0.1590 | Yes |
| 7 | Sirpb1c | 170 | 1.719 | 0.1797 | Yes |
| 8 | Crlf2 | 182 | 1.670 | 0.1998 | Yes |
| 9 | Csf2ra | 185 | 1.657 | 0.2219 | Yes |
| 10 | Cdkn1a | 192 | 1.625 | 0.2426 | Yes |
| 11 | Msn | 219 | 1.522 | 0.2569 | Yes |
| 12 | Pim1 | 262 | 1.425 | 0.2659 | Yes |
| 13 | Apbb1ip | 264 | 1.423 | 0.2850 | Yes |
| 14 | Hp | 286 | 1.351 | 0.2983 | Yes |
| 15 | Hck | 312 | 1.246 | 0.3090 | Yes |
| 16 | Ehd1 | 313 | 1.237 | 0.3259 | Yes |
| 17 | Fmnl1 | 345 | 1.153 | 0.3340 | Yes |
| 18 | Ppp1r18 | 371 | 1.111 | 0.3429 | Yes |
| 19 | Irf1 | 372 | 1.111 | 0.3581 | Yes |
| 20 | Tnfaip2 | 387 | 1.086 | 0.3694 | Yes |
| 21 | Coro1a | 390 | 1.079 | 0.3836 | Yes |
| 22 | Plaur | 426 | 1.012 | 0.3887 | Yes |
| 23 | Nupr1 | 443 | 0.988 | 0.3982 | Yes |
| 24 | Lcn2 | 444 | 0.985 | 0.4117 | Yes |
| 25 | Flna | 471 | 0.946 | 0.4181 | Yes |
| 26 | Rnf149 | 502 | 0.897 | 0.4229 | Yes |
| 27 | Hcls1 | 503 | 0.897 | 0.4352 | Yes |
| 28 | Cebpb | 504 | 0.897 | 0.4474 | Yes |
| 29 | Cyba | 519 | 0.875 | 0.4559 | Yes |
| 30 | Gadd45b | 522 | 0.873 | 0.4673 | Yes |
| 31 | Emilin2 | 611 | 0.755 | 0.4557 | Yes |
| 32 | Dusp3 | 634 | 0.725 | 0.4601 | Yes |
| 33 | Cotl1 | 656 | 0.709 | 0.4646 | Yes |
| 34 | Ifrd1 | 676 | 0.688 | 0.4692 | Yes |
| 35 | Smpdl3b | 682 | 0.684 | 0.4773 | Yes |
| 36 | Slc16a3 | 689 | 0.680 | 0.4851 | Yes |
| 37 | Arrb2 | 786 | 0.600 | 0.4694 | Yes |
| 38 | Sparc | 792 | 0.595 | 0.4763 | Yes |
| 39 | Arpc4 | 798 | 0.593 | 0.4832 | Yes |
| 40 | Pkm | 807 | 0.591 | 0.4892 | Yes |
| 41 | Rgcc | 822 | 0.579 | 0.4936 | Yes |
| 42 | Rilpl2 | 852 | 0.566 | 0.4942 | Yes |
| 43 | Dusp5 | 861 | 0.563 | 0.4999 | Yes |
| 44 | Csrnp1 | 869 | 0.559 | 0.5057 | Yes |
| 45 | Cfl1 | 895 | 0.538 | 0.5069 | Yes |
| 46 | Cdc42ep2 | 926 | 0.517 | 0.5065 | Yes |
| 47 | Nfkbia | 954 | 0.502 | 0.5066 | Yes |
| 48 | Limd2 | 971 | -0.501 | 0.5095 | Yes |
| 49 | Gnb2 | 1007 | -0.506 | 0.5077 | No |
| 50 | Emc10 | 1234 | -0.546 | 0.4589 | No |
| 51 | Ezr | 1361 | -0.567 | 0.4353 | No |
| 52 | Map3k14 | 1363 | -0.568 | 0.4428 | No |
| 53 | Cmip | 1555 | -0.603 | 0.4036 | No |
| 54 | Tgif1 | 1899 | -0.673 | 0.3275 | No |
| 55 | Cebpd | 2002 | -0.693 | 0.3116 | No |
| 56 | Mospd3 | 2046 | -0.703 | 0.3105 | No |
| 57 | Kpna4 | 2243 | -0.749 | 0.2720 | No |
| 58 | Cic | 2411 | -0.794 | 0.2413 | No |
| 59 | Spn | 2413 | -0.794 | 0.2519 | No |
| 60 | Kmt2b | 2419 | -0.795 | 0.2615 | No |
| 61 | Sgk1 | 2539 | -0.829 | 0.2432 | No |
| 62 | Eif3f | 2655 | -0.863 | 0.2264 | No |
| 63 | Bri3 | 2942 | -0.954 | 0.1683 | No |
| 64 | Rbm38 | 3651 | -1.338 | 0.0105 | No |
| 65 | Lmo4 | 3802 | -1.515 | -0.0061 | No |
| 66 | Pim3 | 3893 | -1.707 | -0.0051 | No |
| 67 | Wfdc2 | 3939 | -1.799 | 0.0082 | No |
| 68 | Tppp3 | 4001 | -2.112 | 0.0219 | No |
Table: GSEA details [plain text format]

  

Fig 2: TABULA\_MURIS\_SENIS\_SUBCUTANEOUS\_ADIPOSE\_TISSUE\_MYELOID\_CELL\_AGEING: Random ES distribution      
 Gene set null distribution of ES for **TABULA\_MURIS\_SENIS\_SUBCUTANEOUS\_ADIPOSE\_TISSUE\_MYELOID\_CELL\_AGEING**

  
