# Supplementary material for: TMPRSS11B promotes an acidified microenvironment and immune suppression in squamous lung cancer
Source: EMBO Rep. 2025 Nov 10;26(24):6346–79. doi: 10.1038/s44319-025-00631-1 (PMC12714794; doi:10.1038/s44319-025-00631-1)
Supplement: Supplementary file 18 — Figure EV6 Source Data [file 44319_2025_631_MOESM18_ESM.zip › Figure EV6/EV6C-D/GSEA_Broad Institute_M8_T11b high vs low LUSC/TABULA_MURIS_SENIS_TONGUE_BASAL_CELL_OF_EPIDERMIS_AGEING.html]

Details for gene set TABULA\_MURIS\_SENIS\_TONGUE\_BASAL\_CELL\_OF\_EPIDERMIS\_AGEING[GSEA]

|  || Dataset | T11b high vs low squamous\_GSEA\_Ranked |
| Phenotype | NoPhenotypeAvailable |
| Upregulated in class | na\_pos |
| GeneSet | TABULA\_MURIS\_SENIS\_TONGUE\_BASAL\_CELL\_OF\_EPIDERMIS\_AGEING |
| Enrichment Score (ES) | 0.5757945 |
| Normalized Enrichment Score (NES) | 2.9907084 |
| Nominal p-value | 0.0 |
| FDR q-value | 0.0 |
| FWER p-Value | 0.0 |
Table: GSEA Results Summary

  

Fig 1: Enrichment plot: TABULA\_MURIS\_SENIS\_TONGUE\_BASAL\_CELL\_OF\_EPIDERMIS\_AGEING      
 Profile of the Running ES Score & Positions of GeneSet Members on the Rank Ordered List

  

| SYMBOL | RANK IN GENE LIST | RANK METRIC SCORE | RUNNING ES | CORE ENRICHMENT || 1 | Tgm3 | 4 | 5.180 | 0.1080 | Yes |
| 2 | Krt16 | 14 | 4.032 | 0.1906 | Yes |
| 3 | Krt6b | 47 | 2.784 | 0.2413 | Yes |
| 4 | Krtdap | 87 | 2.327 | 0.2806 | Yes |
| 5 | Apoe | 88 | 2.296 | 0.3289 | Yes |
| 6 | Apoc1 | 164 | 1.742 | 0.3470 | Yes |
| 7 | S100a14 | 311 | 1.260 | 0.3375 | Yes |
| 8 | Cxcl16 | 342 | 1.161 | 0.3545 | Yes |
| 9 | Lgals3 | 377 | 1.096 | 0.3691 | Yes |
| 10 | Fabp5 | 385 | 1.088 | 0.3903 | Yes |
| 11 | Lypd3 | 389 | 1.080 | 0.4123 | Yes |
| 12 | Stx11 | 407 | 1.035 | 0.4298 | Yes |
| 13 | Plaur | 426 | 1.012 | 0.4467 | Yes |
| 14 | Tubb6 | 460 | 0.961 | 0.4588 | Yes |
| 15 | Cysrt1 | 468 | 0.947 | 0.4770 | Yes |
| 16 | Gadd45b | 522 | 0.873 | 0.4822 | Yes |
| 17 | Gsto1 | 543 | 0.850 | 0.4952 | Yes |
| 18 | Slpi | 558 | 0.836 | 0.5093 | Yes |
| 19 | Ccna2 | 694 | 0.673 | 0.4901 | Yes |
| 20 | Pgk1 | 717 | 0.655 | 0.4984 | Yes |
| 21 | H2-D1 | 719 | 0.654 | 0.5119 | Yes |
| 22 | Cdk1 | 724 | 0.651 | 0.5246 | Yes |
| 23 | Ovol1 | 803 | 0.592 | 0.5178 | Yes |
| 24 | Cldn4 | 811 | 0.589 | 0.5285 | Yes |
| 25 | Cks2 | 812 | 0.588 | 0.5409 | Yes |
| 26 | Sfn | 834 | 0.573 | 0.5477 | Yes |
| 27 | H2-K1 | 855 | 0.565 | 0.5547 | Yes |
| 28 | B2m | 860 | 0.563 | 0.5655 | Yes |
| 29 | Cenpa | 892 | 0.542 | 0.5693 | Yes |
| 30 | Tacstd2 | 937 | 0.512 | 0.5692 | Yes |
| 31 | Nfkbia | 954 | 0.502 | 0.5758 | Yes |
| 32 | Ppa1 | 1606 | -0.612 | 0.4278 | No |
| 33 | Tsc22d1 | 1718 | -0.633 | 0.4137 | No |
| 34 | Ifi27 | 1778 | -0.644 | 0.4127 | No |
| 35 | Ehf | 2003 | -0.694 | 0.3720 | No |
| 36 | Gstm1 | 2049 | -0.704 | 0.3757 | No |
| 37 | Shroom3 | 2396 | -0.790 | 0.3068 | No |
| 38 | Pmm1 | 2637 | -0.858 | 0.2656 | No |
| 39 | Sod1 | 2691 | -0.874 | 0.2708 | No |
| 40 | Aldh3a1 | 2970 | -0.964 | 0.2225 | No |
| 41 | Ptgr1 | 3237 | -1.085 | 0.1796 | No |
| 42 | Adh7 | 3771 | -1.457 | 0.0786 | No |
Table: GSEA details [plain text format]

  

Fig 2: TABULA\_MURIS\_SENIS\_TONGUE\_BASAL\_CELL\_OF\_EPIDERMIS\_AGEING: Random ES distribution      
 Gene set null distribution of ES for **TABULA\_MURIS\_SENIS\_TONGUE\_BASAL\_CELL\_OF\_EPIDERMIS\_AGEING**

  
