# Supplementary material for: TMPRSS11B promotes an acidified microenvironment and immune suppression in squamous lung cancer
Source: EMBO Rep. 2025 Nov 10;26(24):6346–79. doi: 10.1038/s44319-025-00631-1 (PMC12714794; doi:10.1038/s44319-025-00631-1)
Supplement: Supplementary file 18 — Figure EV6 Source Data [file 44319_2025_631_MOESM18_ESM.zip › Figure EV6/EV6C-D/GSEA_Broad Institute_M8_T11b high vs low LUSC/ZHANG_UTERUS_C10_STROMAL2_RETNLG_HIGH_CELL.html]

Details for gene set ZHANG\_UTERUS\_C10\_STROMAL2\_RETNLG\_HIGH\_CELL[GSEA]

|  || Dataset | T11b high vs low squamous\_GSEA\_Ranked |
| Phenotype | NoPhenotypeAvailable |
| Upregulated in class | na\_pos |
| GeneSet | ZHANG\_UTERUS\_C10\_STROMAL2\_RETNLG\_HIGH\_CELL |
| Enrichment Score (ES) | 0.81830347 |
| Normalized Enrichment Score (NES) | 3.5129163 |
| Nominal p-value | 0.0 |
| FDR q-value | 0.0 |
| FWER p-Value | 0.0 |
Table: GSEA Results Summary

  

Fig 1: Enrichment plot: ZHANG\_UTERUS\_C10\_STROMAL2\_RETNLG\_HIGH\_CELL      
 Profile of the Running ES Score & Positions of GeneSet Members on the Rank Ordered List

  

| SYMBOL | RANK IN GENE LIST | RANK METRIC SCORE | RUNNING ES | CORE ENRICHMENT || 1 | Cxcl3 | 6 | 5.077 | 0.1348 | Yes |
| 2 | Cybb | 57 | 2.654 | 0.1937 | Yes |
| 3 | Tyrobp | 83 | 2.366 | 0.2511 | Yes |
| 4 | Pla2g7 | 94 | 2.250 | 0.3090 | Yes |
| 5 | Plek | 95 | 2.237 | 0.3690 | Yes |
| 6 | Slc7a11 | 126 | 1.940 | 0.4137 | Yes |
| 7 | Il1b | 129 | 1.912 | 0.4646 | Yes |
| 8 | Fth1 | 147 | 1.835 | 0.5096 | Yes |
| 9 | Ccl6 | 166 | 1.733 | 0.5517 | Yes |
| 10 | Csf2rb | 243 | 1.458 | 0.5722 | Yes |
| 11 | Srgn | 270 | 1.392 | 0.6031 | Yes |
| 12 | Cxcr4 | 289 | 1.347 | 0.6349 | Yes |
| 13 | Cd52 | 350 | 1.140 | 0.6507 | Yes |
| 14 | Adam8 | 375 | 1.104 | 0.6745 | Yes |
| 15 | Coro1a | 390 | 1.079 | 0.7000 | Yes |
| 16 | Alox5ap | 399 | 1.051 | 0.7262 | Yes |
| 17 | Il1rn | 454 | 0.972 | 0.7390 | Yes |
| 18 | Msrb1 | 472 | 0.945 | 0.7602 | Yes |
| 19 | Cebpb | 504 | 0.897 | 0.7766 | Yes |
| 20 | Rgs1 | 526 | 0.873 | 0.7949 | Yes |
| 21 | Serpinb2 | 527 | 0.873 | 0.8183 | Yes |
| 22 | Actb | 633 | 0.726 | 0.8120 | No |
| 23 | Mcl1 | 950 | 0.505 | 0.7478 | No |
| 24 | Cd24a | 2759 | -0.893 | 0.3271 | No |
Table: GSEA details [plain text format]

  

Fig 2: ZHANG\_UTERUS\_C10\_STROMAL2\_RETNLG\_HIGH\_CELL: Random ES distribution      
 Gene set null distribution of ES for **ZHANG\_UTERUS\_C10\_STROMAL2\_RETNLG\_HIGH\_CELL**

  
