# Supplementary material for: TMPRSS11B promotes an acidified microenvironment and immune suppression in squamous lung cancer
Source: EMBO Rep. 2025 Nov 10;26(24):6346–79. doi: 10.1038/s44319-025-00631-1 (PMC12714794; doi:10.1038/s44319-025-00631-1)
Supplement: Supplementary file 18 — Figure EV6 Source Data [file 44319_2025_631_MOESM18_ESM.zip › Figure EV6/EV6C-D/GSEA_Broad Institute_M8_T11b high vs low LUSC/ZHANG_UTERUS_C12_MONOCYTE.html]

Details for gene set ZHANG\_UTERUS\_C12\_MONOCYTE[GSEA]

|  || Dataset | T11b high vs low squamous\_GSEA\_Ranked |
| Phenotype | NoPhenotypeAvailable |
| Upregulated in class | na\_pos |
| GeneSet | ZHANG\_UTERUS\_C12\_MONOCYTE |
| Enrichment Score (ES) | 0.7658258 |
| Normalized Enrichment Score (NES) | 4.742664 |
| Nominal p-value | 0.0 |
| FDR q-value | 0.0 |
| FWER p-Value | 0.0 |
Table: GSEA Results Summary

  

Fig 1: Enrichment plot: ZHANG\_UTERUS\_C12\_MONOCYTE      
 Profile of the Running ES Score & Positions of GeneSet Members on the Rank Ordered List

  

| SYMBOL | RANK IN GENE LIST | RANK METRIC SCORE | RUNNING ES | CORE ENRICHMENT || 1 | Spp1 | 13 | 4.054 | 0.0323 | Yes |
| 2 | Hmox1 | 55 | 2.684 | 0.0456 | Yes |
| 3 | Cybb | 57 | 2.654 | 0.0687 | Yes |
| 4 | Fcgr2b | 62 | 2.610 | 0.0906 | Yes |
| 5 | Ctss | 63 | 2.582 | 0.1132 | Yes |
| 6 | Itgb2 | 71 | 2.481 | 0.1332 | Yes |
| 7 | Fcer1g | 76 | 2.415 | 0.1534 | Yes |
| 8 | Tyrobp | 83 | 2.366 | 0.1727 | Yes |
| 9 | Apoe | 88 | 2.296 | 0.1918 | Yes |
| 10 | Ccl9 | 93 | 2.271 | 0.2108 | Yes |
| 11 | Plek | 95 | 2.237 | 0.2301 | Yes |
| 12 | Cd68 | 97 | 2.215 | 0.2493 | Yes |
| 13 | Fcgr3 | 102 | 2.142 | 0.2671 | Yes |
| 14 | Mpeg1 | 114 | 2.045 | 0.2823 | Yes |
| 15 | Emp3 | 115 | 2.020 | 0.3000 | Yes |
| 16 | Mafb | 116 | 2.017 | 0.3177 | Yes |
| 17 | Wfdc17 | 117 | 1.998 | 0.3353 | Yes |
| 18 | C5ar1 | 125 | 1.945 | 0.3506 | Yes |
| 19 | Il1b | 129 | 1.912 | 0.3666 | Yes |
| 20 | Ctsz | 138 | 1.884 | 0.3811 | Yes |
| 21 | C1qb | 139 | 1.882 | 0.3977 | Yes |
| 22 | Lgmn | 141 | 1.877 | 0.4139 | Yes |
| 23 | Fth1 | 147 | 1.835 | 0.4287 | Yes |
| 24 | Tgfbi | 151 | 1.825 | 0.4440 | Yes |
| 25 | Fxyd5 | 157 | 1.767 | 0.4583 | Yes |
| 26 | Spi1 | 158 | 1.765 | 0.4737 | Yes |
| 27 | Ccl6 | 166 | 1.733 | 0.4872 | Yes |
| 28 | Cfp | 171 | 1.717 | 0.5013 | Yes |
| 29 | Ctsb | 177 | 1.695 | 0.5149 | Yes |
| 30 | Csf2ra | 185 | 1.657 | 0.5277 | Yes |
| 31 | Cdkn1a | 192 | 1.625 | 0.5404 | Yes |
| 32 | Sdc3 | 196 | 1.610 | 0.5538 | Yes |
| 33 | Arhgdib | 214 | 1.549 | 0.5632 | Yes |
| 34 | Plin2 | 217 | 1.534 | 0.5761 | Yes |
| 35 | Lcp1 | 231 | 1.490 | 0.5860 | Yes |
| 36 | C1qc | 234 | 1.480 | 0.5984 | Yes |
| 37 | Psap | 240 | 1.466 | 0.6101 | Yes |
| 38 | Csf2rb | 243 | 1.458 | 0.6223 | Yes |
| 39 | Ptprc | 258 | 1.430 | 0.6314 | Yes |
| 40 | Pim1 | 262 | 1.425 | 0.6432 | Yes |
| 41 | Srgn | 270 | 1.392 | 0.6536 | Yes |
| 42 | Grn | 285 | 1.352 | 0.6620 | Yes |
| 43 | Cd53 | 337 | 1.171 | 0.6595 | Yes |
| 44 | Cd52 | 350 | 1.140 | 0.6665 | Yes |
| 45 | Lgals3 | 377 | 1.096 | 0.6697 | Yes |
| 46 | Coro1a | 390 | 1.079 | 0.6761 | Yes |
| 47 | Alox5ap | 399 | 1.051 | 0.6834 | Yes |
| 48 | Csf1r | 414 | 1.025 | 0.6889 | Yes |
| 49 | Ctsa | 425 | 1.013 | 0.6952 | Yes |
| 50 | Plaur | 426 | 1.012 | 0.7041 | Yes |
| 51 | Rab8b | 452 | 0.975 | 0.7064 | Yes |
| 52 | Msrb1 | 472 | 0.945 | 0.7100 | Yes |
| 53 | Ftl1-ps1 | 517 | 0.881 | 0.7067 | Yes |
| 54 | Cyba | 519 | 0.875 | 0.7142 | Yes |
| 55 | Rgs1 | 526 | 0.873 | 0.7203 | Yes |
| 56 | Npc2 | 536 | 0.861 | 0.7256 | Yes |
| 57 | Irf7 | 548 | 0.847 | 0.7303 | Yes |
| 58 | Cd44 | 562 | 0.834 | 0.7344 | Yes |
| 59 | Rab20 | 565 | 0.831 | 0.7412 | Yes |
| 60 | Emilin2 | 611 | 0.755 | 0.7366 | Yes |
| 61 | Crip1 | 638 | 0.721 | 0.7364 | Yes |
| 62 | Esd | 653 | 0.710 | 0.7391 | Yes |
| 63 | Cotl1 | 656 | 0.709 | 0.7449 | Yes |
| 64 | Ifrd1 | 676 | 0.688 | 0.7461 | Yes |
| 65 | Lat2 | 714 | 0.657 | 0.7427 | Yes |
| 66 | H2-D1 | 719 | 0.654 | 0.7474 | Yes |
| 67 | Slfn2 | 750 | 0.628 | 0.7454 | Yes |
| 68 | Ubl3 | 751 | 0.627 | 0.7509 | Yes |
| 69 | Kctd12 | 783 | 0.601 | 0.7485 | Yes |
| 70 | Pkm | 807 | 0.591 | 0.7479 | Yes |
| 71 | Sat1 | 817 | 0.584 | 0.7508 | Yes |
| 72 | Cd74 | 849 | 0.567 | 0.7480 | Yes |
| 73 | Rilpl2 | 852 | 0.566 | 0.7525 | Yes |
| 74 | Litaf | 856 | 0.564 | 0.7567 | Yes |
| 75 | B2m | 860 | 0.563 | 0.7609 | Yes |
| 76 | Dusp5 | 861 | 0.563 | 0.7658 | Yes |
| 77 | H2-Ab1 | 915 | 0.525 | 0.7572 | No |
| 78 | Got1 | 949 | 0.506 | 0.7534 | No |
| 79 | Mcl1 | 950 | 0.505 | 0.7578 | No |
| 80 | Ucp2 | 979 | -0.502 | 0.7553 | No |
| 81 | Ly6e | 1857 | -0.663 | 0.5422 | No |
| 82 | Tgif1 | 1899 | -0.673 | 0.5379 | No |
| 83 | Por | 2959 | -0.960 | 0.2820 | No |
Table: GSEA details [plain text format]

  

Fig 2: ZHANG\_UTERUS\_C12\_MONOCYTE: Random ES distribution      
 Gene set null distribution of ES for **ZHANG\_UTERUS\_C12\_MONOCYTE**

  
