# Supplementary material for: TMPRSS11B promotes an acidified microenvironment and immune suppression in squamous lung cancer
Source: EMBO Rep. 2025 Nov 10;26(24):6346–79. doi: 10.1038/s44319-025-00631-1 (PMC12714794; doi:10.1038/s44319-025-00631-1)
Supplement: Supplementary file 18 — Figure EV6 Source Data [file 44319_2025_631_MOESM18_ESM.zip › Figure EV6/EV6C-D/GSEA_Broad Institute_M8_T11b high vs low LUSC/ZHANG_UTERUS_C13_EPITHELIAL1_CELL.html]

Details for gene set ZHANG\_UTERUS\_C13\_EPITHELIAL1\_CELL[GSEA]

|  || Dataset | T11b high vs low squamous\_GSEA\_Ranked |
| Phenotype | NoPhenotypeAvailable |
| Upregulated in class | na\_neg |
| GeneSet | ZHANG\_UTERUS\_C13\_EPITHELIAL1\_CELL |
| Enrichment Score (ES) | -0.25595102 |
| Normalized Enrichment Score (NES) | -1.485154 |
| Nominal p-value | 0.039938558 |
| FDR q-value | 0.33467123 |
| FWER p-Value | 0.99 |
Table: GSEA Results Summary

  

Fig 1: Enrichment plot: ZHANG\_UTERUS\_C13\_EPITHELIAL1\_CELL      
 Profile of the Running ES Score & Positions of GeneSet Members on the Rank Ordered List

  

| SYMBOL | RANK IN GENE LIST | RANK METRIC SCORE | RUNNING ES | CORE ENRICHMENT || 1 | Plat | 29 | 3.372 | 0.0289 | No |
| 2 | Sprr2f | 70 | 2.525 | 0.0460 | No |
| 3 | Tgfbi | 151 | 1.825 | 0.0456 | No |
| 4 | S100g | 178 | 1.695 | 0.0573 | No |
| 5 | Pdlim4 | 279 | 1.375 | 0.0471 | No |
| 6 | Serpinb11 | 379 | 1.095 | 0.0341 | No |
| 7 | Lcn2 | 444 | 0.985 | 0.0287 | No |
| 8 | Mif | 484 | 0.921 | 0.0288 | No |
| 9 | Gsto1 | 543 | 0.850 | 0.0235 | No |
| 10 | 1810037I17Rik | 582 | 0.811 | 0.0227 | No |
| 11 | Txn1 | 586 | 0.800 | 0.0305 | No |
| 12 | Coa3 | 975 | -0.502 | -0.0611 | No |
| 13 | Ucp2 | 979 | -0.502 | -0.0564 | No |
| 14 | Ndufc2 | 997 | -0.504 | -0.0553 | No |
| 15 | Ndufab1 | 1098 | -0.522 | -0.0747 | No |
| 16 | Dgat2 | 1171 | -0.534 | -0.0869 | No |
| 17 | Dhrs4 | 1226 | -0.545 | -0.0946 | No |
| 18 | Bola1 | 1253 | -0.548 | -0.0952 | No |
| 19 | Ndufv2 | 1275 | -0.552 | -0.0945 | No |
| 20 | Iah1 | 1294 | -0.554 | -0.0931 | No |
| 21 | Pmf1 | 1301 | -0.556 | -0.0886 | No |
| 22 | Gtf3c6 | 1338 | -0.564 | -0.0916 | No |
| 23 | Mphosph8 | 1364 | -0.568 | -0.0917 | No |
| 24 | Krt8 | 1410 | -0.578 | -0.0968 | No |
| 25 | Slc39a4 | 1420 | -0.579 | -0.0928 | No |
| 26 | Prdx6 | 1466 | -0.587 | -0.0977 | No |
| 27 | Cib1 | 1491 | -0.592 | -0.0974 | No |
| 28 | Cldn3 | 1495 | -0.593 | -0.0918 | No |
| 29 | Fxyd3 | 1523 | -0.598 | -0.0921 | No |
| 30 | Cystm1 | 1549 | -0.602 | -0.0919 | No |
| 31 | Acadl | 1550 | -0.602 | -0.0854 | No |
| 32 | Arpc5l | 1558 | -0.604 | -0.0807 | No |
| 33 | Nop10 | 1642 | -0.619 | -0.0948 | No |
| 34 | Cited4 | 1764 | -0.642 | -0.1181 | No |
| 35 | Ltf | 1834 | -0.657 | -0.1283 | No |
| 36 | Hjurp | 1839 | -0.659 | -0.1223 | No |
| 37 | Nap1l1 | 1883 | -0.670 | -0.1258 | No |
| 38 | Krtcap2 | 1937 | -0.682 | -0.1317 | No |
| 39 | Stx18 | 1970 | -0.688 | -0.1324 | No |
| 40 | Gstm1 | 2049 | -0.704 | -0.1443 | No |
| 41 | Cox7c | 2108 | -0.712 | -0.1511 | No |
| 42 | Knop1 | 2124 | -0.720 | -0.1472 | No |
| 43 | Sftpd | 2144 | -0.727 | -0.1441 | No |
| 44 | 2510002D24Rik | 2291 | -0.760 | -0.1724 | No |
| 45 | Tmem238 | 2313 | -0.766 | -0.1695 | No |
| 46 | Ciao2a | 2322 | -0.768 | -0.1632 | No |
| 47 | Srsf3 | 2381 | -0.784 | -0.1693 | No |
| 48 | Yipf1 | 2383 | -0.785 | -0.1611 | No |
| 49 | Fermt1 | 2395 | -0.789 | -0.1554 | No |
| 50 | Nudt14 | 2423 | -0.797 | -0.1536 | No |
| 51 | Tmem176b | 2558 | -0.834 | -0.1782 | No |
| 52 | Dynll2 | 2574 | -0.841 | -0.1729 | No |
| 53 | Lamp2 | 2707 | -0.877 | -0.1965 | No |
| 54 | Cd24a | 2759 | -0.893 | -0.1996 | No |
| 55 | Cfi | 2950 | -0.956 | -0.2368 | No |
| 56 | Rpa3 | 2973 | -0.965 | -0.2320 | No |
| 57 | Pigr | 3054 | -0.996 | -0.2413 | No |
| 58 | Cbx6 | 3071 | -1.005 | -0.2345 | No |
| 59 | Gstm2 | 3116 | -1.026 | -0.2345 | No |
| 60 | Krt19 | 3163 | -1.044 | -0.2348 | No |
| 61 | Smim22 | 3198 | -1.064 | -0.2319 | No |
| 62 | Spint2 | 3222 | -1.076 | -0.2261 | No |
| 63 | Sf3b4 | 3336 | -1.127 | -0.2422 | No |
| 64 | Mid1ip1 | 3392 | -1.158 | -0.2435 | Yes |
| 65 | Clu | 3405 | -1.162 | -0.2341 | Yes |
| 66 | Polr2i | 3409 | -1.163 | -0.2223 | Yes |
| 67 | Hat1 | 3418 | -1.169 | -0.2118 | Yes |
| 68 | Ivns1abp | 3467 | -1.197 | -0.2109 | Yes |
| 69 | Rbbp7 | 3556 | -1.252 | -0.2195 | Yes |
| 70 | Tmem176a | 3571 | -1.266 | -0.2094 | Yes |
| 71 | Aamdc | 3601 | -1.288 | -0.2028 | Yes |
| 72 | Echdc2 | 3645 | -1.326 | -0.1993 | Yes |
| 73 | Paics | 3731 | -1.423 | -0.2053 | Yes |
| 74 | Adi1 | 3769 | -1.457 | -0.1989 | Yes |
| 75 | Kctd14 | 3779 | -1.474 | -0.1854 | Yes |
| 76 | Gtf2a2 | 3817 | -1.542 | -0.1781 | Yes |
| 77 | Dcxr | 3823 | -1.555 | -0.1626 | Yes |
| 78 | Sox9 | 3877 | -1.658 | -0.1581 | Yes |
| 79 | Cldn10 | 3879 | -1.659 | -0.1405 | Yes |
| 80 | Slc1a5 | 3889 | -1.685 | -0.1247 | Yes |
| 81 | Muc1 | 3926 | -1.769 | -0.1147 | Yes |
| 82 | Sult1d1 | 3938 | -1.797 | -0.0982 | Yes |
| 83 | Wfdc2 | 3939 | -1.799 | -0.0789 | Yes |
| 84 | Gstm7 | 3949 | -1.827 | -0.0615 | Yes |
| 85 | Cbr2 | 3970 | -1.937 | -0.0457 | Yes |
| 86 | Tmem158 | 3977 | -2.002 | -0.0258 | Yes |
| 87 | Cxcl17 | 4049 | -2.472 | -0.0170 | Yes |
| 88 | Aldh1a1 | 4051 | -2.492 | 0.0095 | Yes |
Table: GSEA details [plain text format]

  

Fig 2: ZHANG\_UTERUS\_C13\_EPITHELIAL1\_CELL: Random ES distribution      
 Gene set null distribution of ES for **ZHANG\_UTERUS\_C13\_EPITHELIAL1\_CELL**

  
