# Supplementary material for: TMPRSS11B promotes an acidified microenvironment and immune suppression in squamous lung cancer
Source: EMBO Rep. 2025 Nov 10;26(24):6346–79. doi: 10.1038/s44319-025-00631-1 (PMC12714794; doi:10.1038/s44319-025-00631-1)
Supplement: Supplementary file 18 — Figure EV6 Source Data [file 44319_2025_631_MOESM18_ESM.zip › Figure EV6/EV6C-D/GSEA_Broad Institute_M8_T11b high vs low LUSC/ZHANG_UTERUS_C2_REGENERATIVE_UP.html]

Details for gene set ZHANG\_UTERUS\_C2\_REGENERATIVE\_UP[GSEA]

|  || Dataset | T11b high vs low squamous\_GSEA\_Ranked |
| Phenotype | NoPhenotypeAvailable |
| Upregulated in class | na\_neg |
| GeneSet | ZHANG\_UTERUS\_C2\_REGENERATIVE\_UP |
| Enrichment Score (ES) | -0.33159044 |
| Normalized Enrichment Score (NES) | -1.3826224 |
| Nominal p-value | 0.11456628 |
| FDR q-value | 0.3711699 |
| FWER p-Value | 1.0 |
Table: GSEA Results Summary

  

Fig 1: Enrichment plot: ZHANG\_UTERUS\_C2\_REGENERATIVE\_UP      
 Profile of the Running ES Score & Positions of GeneSet Members on the Rank Ordered List

  

| SYMBOL | RANK IN GENE LIST | RANK METRIC SCORE | RUNNING ES | CORE ENRICHMENT || 1 | Tgfbi | 151 | 1.825 | 0.0268 | No |
| 2 | S100g | 178 | 1.695 | 0.0799 | No |
| 3 | Serpinb11 | 379 | 1.095 | 0.0691 | No |
| 4 | Mif | 484 | 0.921 | 0.0758 | No |
| 5 | Igfbp7 | 711 | 0.661 | 0.0433 | No |
| 6 | Rbp1 | 924 | 0.518 | 0.0093 | No |
| 7 | Iah1 | 1294 | -0.554 | -0.0620 | No |
| 8 | Prdx6 | 1466 | -0.587 | -0.0835 | No |
| 9 | Ltf | 1834 | -0.657 | -0.1507 | No |
| 10 | Krtcap2 | 1937 | -0.682 | -0.1519 | No |
| 11 | Stx18 | 1970 | -0.688 | -0.1356 | No |
| 12 | Gstm1 | 2049 | -0.704 | -0.1301 | No |
| 13 | Tmem176b | 2558 | -0.834 | -0.2258 | No |
| 14 | Lamp2 | 2707 | -0.877 | -0.2315 | No |
| 15 | Pigr | 3054 | -0.996 | -0.2817 | No |
| 16 | Gstm2 | 3116 | -1.026 | -0.2607 | No |
| 17 | Clu | 3405 | -1.162 | -0.2908 | Yes |
| 18 | Ivns1abp | 3467 | -1.197 | -0.2639 | Yes |
| 19 | Tmem176a | 3571 | -1.266 | -0.2448 | Yes |
| 20 | Echdc2 | 3645 | -1.326 | -0.2163 | Yes |
| 21 | Kctd14 | 3779 | -1.474 | -0.1973 | Yes |
| 22 | Cldn10 | 3879 | -1.659 | -0.1635 | Yes |
| 23 | Sult1d1 | 3938 | -1.797 | -0.1148 | Yes |
| 24 | Gstm7 | 3949 | -1.827 | -0.0532 | Yes |
| 25 | Aldh1a1 | 4051 | -2.492 | 0.0093 | Yes |
Table: GSEA details [plain text format]

  

Fig 2: ZHANG\_UTERUS\_C2\_REGENERATIVE\_UP: Random ES distribution      
 Gene set null distribution of ES for **ZHANG\_UTERUS\_C2\_REGENERATIVE\_UP**

  
