# Supplementary material for: TMPRSS11B promotes an acidified microenvironment and immune suppression in squamous lung cancer
Source: EMBO Rep. 2025 Nov 10;26(24):6346–79. doi: 10.1038/s44319-025-00631-1 (PMC12714794; doi:10.1038/s44319-025-00631-1)
Supplement: Supplementary file 18 — Figure EV6 Source Data [file 44319_2025_631_MOESM18_ESM.zip › Figure EV6/EV6C-D/GSEA_Broad Institute_M8_T11b high vs low LUSC/ZHANG_UTERUS_C5_MACROPHAGE.html]

Details for gene set ZHANG\_UTERUS\_C5\_MACROPHAGE[GSEA]

|  || Dataset | T11b high vs low squamous\_GSEA\_Ranked |
| Phenotype | NoPhenotypeAvailable |
| Upregulated in class | na\_pos |
| GeneSet | ZHANG\_UTERUS\_C5\_MACROPHAGE |
| Enrichment Score (ES) | 0.7289074 |
| Normalized Enrichment Score (NES) | 4.4982915 |
| Nominal p-value | 0.0 |
| FDR q-value | 0.0 |
| FWER p-Value | 0.0 |
Table: GSEA Results Summary

  

Fig 1: Enrichment plot: ZHANG\_UTERUS\_C5\_MACROPHAGE      
 Profile of the Running ES Score & Positions of GeneSet Members on the Rank Ordered List

  

| SYMBOL | RANK IN GENE LIST | RANK METRIC SCORE | RUNNING ES | CORE ENRICHMENT || 1 | Trem2 | 24 | 3.569 | 0.0256 | Yes |
| 2 | Lpl | 43 | 2.812 | 0.0460 | Yes |
| 3 | C3ar1 | 46 | 2.795 | 0.0703 | Yes |
| 4 | Hmox1 | 55 | 2.684 | 0.0921 | Yes |
| 5 | Cybb | 57 | 2.654 | 0.1153 | Yes |
| 6 | Fcgr2b | 62 | 2.610 | 0.1374 | Yes |
| 7 | Ctss | 63 | 2.582 | 0.1603 | Yes |
| 8 | Fcer1g | 76 | 2.415 | 0.1787 | Yes |
| 9 | Tyrobp | 83 | 2.366 | 0.1982 | Yes |
| 10 | Apoe | 88 | 2.296 | 0.2175 | Yes |
| 11 | Ccl9 | 93 | 2.271 | 0.2366 | Yes |
| 12 | Plek | 95 | 2.237 | 0.2562 | Yes |
| 13 | Cd68 | 97 | 2.215 | 0.2755 | Yes |
| 14 | Fcgr3 | 102 | 2.142 | 0.2935 | Yes |
| 15 | Mpeg1 | 114 | 2.045 | 0.3089 | Yes |
| 16 | Mafb | 116 | 2.017 | 0.3265 | Yes |
| 17 | Wfdc17 | 117 | 1.998 | 0.3442 | Yes |
| 18 | C1qa | 119 | 1.990 | 0.3616 | Yes |
| 19 | Il1b | 129 | 1.912 | 0.3763 | Yes |
| 20 | Ctsz | 138 | 1.884 | 0.3909 | Yes |
| 21 | C1qb | 139 | 1.882 | 0.4076 | Yes |
| 22 | Lgmn | 141 | 1.877 | 0.4240 | Yes |
| 23 | Fth1 | 147 | 1.835 | 0.4390 | Yes |
| 24 | Ctsd | 150 | 1.830 | 0.4547 | Yes |
| 25 | Sirpa | 154 | 1.794 | 0.4699 | Yes |
| 26 | Spi1 | 158 | 1.765 | 0.4847 | Yes |
| 27 | Ccl6 | 166 | 1.733 | 0.4983 | Yes |
| 28 | Cfp | 171 | 1.717 | 0.5126 | Yes |
| 29 | Hexb | 175 | 1.703 | 0.5269 | Yes |
| 30 | Ctsb | 177 | 1.695 | 0.5417 | Yes |
| 31 | Cdkn1a | 192 | 1.625 | 0.5525 | Yes |
| 32 | Sdc3 | 196 | 1.610 | 0.5661 | Yes |
| 33 | Plin2 | 217 | 1.534 | 0.5747 | Yes |
| 34 | Lcp1 | 231 | 1.490 | 0.5846 | Yes |
| 35 | C1qc | 234 | 1.480 | 0.5972 | Yes |
| 36 | Psap | 240 | 1.466 | 0.6090 | Yes |
| 37 | Creg1 | 280 | 1.373 | 0.6114 | Yes |
| 38 | Grn | 285 | 1.352 | 0.6224 | Yes |
| 39 | Lipa | 296 | 1.318 | 0.6315 | Yes |
| 40 | Cd53 | 337 | 1.171 | 0.6319 | Yes |
| 41 | Cxcl16 | 342 | 1.161 | 0.6412 | Yes |
| 42 | Cd52 | 350 | 1.140 | 0.6496 | Yes |
| 43 | Bcl2a1b | 359 | 1.126 | 0.6576 | Yes |
| 44 | Atf3 | 373 | 1.107 | 0.6641 | Yes |
| 45 | Lgals3 | 377 | 1.096 | 0.6731 | Yes |
| 46 | Coro1a | 390 | 1.079 | 0.6796 | Yes |
| 47 | Alox5ap | 399 | 1.051 | 0.6869 | Yes |
| 48 | Csf1r | 414 | 1.025 | 0.6925 | Yes |
| 49 | Ctsa | 425 | 1.013 | 0.6990 | Yes |
| 50 | Il1rn | 454 | 0.972 | 0.7006 | Yes |
| 51 | Ehd4 | 474 | 0.943 | 0.7042 | Yes |
| 52 | Ier5 | 493 | 0.908 | 0.7078 | Yes |
| 53 | Cyba | 519 | 0.875 | 0.7093 | Yes |
| 54 | Stab1 | 523 | 0.873 | 0.7163 | Yes |
| 55 | Rgs1 | 526 | 0.873 | 0.7235 | Yes |
| 56 | Npc2 | 536 | 0.861 | 0.7289 | Yes |
| 57 | Trf | 601 | 0.771 | 0.7198 | No |
| 58 | Actb | 633 | 0.726 | 0.7185 | No |
| 59 | Cotl1 | 656 | 0.709 | 0.7193 | No |
| 60 | Pltp | 661 | 0.702 | 0.7245 | No |
| 61 | H2-D1 | 719 | 0.654 | 0.7161 | No |
| 62 | Ms4a7 | 771 | 0.610 | 0.7087 | No |
| 63 | Kctd12 | 783 | 0.601 | 0.7113 | No |
| 64 | Sat1 | 817 | 0.584 | 0.7083 | No |
| 65 | Cd74 | 849 | 0.567 | 0.7056 | No |
| 66 | H2-K1 | 855 | 0.565 | 0.7093 | No |
| 67 | B2m | 860 | 0.563 | 0.7133 | No |
| 68 | Cfl1 | 895 | 0.538 | 0.7096 | No |
| 69 | H2-Ab1 | 915 | 0.525 | 0.7095 | No |
| 70 | Mcl1 | 950 | 0.505 | 0.7055 | No |
| 71 | Dab2 | 952 | 0.504 | 0.7097 | No |
| 72 | Ucp2 | 979 | -0.502 | 0.7077 | No |
| 73 | Ly6e | 1857 | -0.663 | 0.4948 | No |
| 74 | Selenop | 2054 | -0.707 | 0.4522 | No |
| 75 | Snx2 | 2507 | -0.820 | 0.3468 | No |
| 76 | Tmem176b | 2558 | -0.834 | 0.3417 | No |
| 77 | Rgs2 | 2919 | -0.946 | 0.2603 | No |
| 78 | Lgals3bp | 3172 | -1.049 | 0.2067 | No |
| 79 | Tlr2 | 3387 | -1.156 | 0.1636 | No |
| 80 | Tmem176a | 3571 | -1.266 | 0.1292 | No |
Table: GSEA details [plain text format]

  

Fig 2: ZHANG\_UTERUS\_C5\_MACROPHAGE: Random ES distribution      
 Gene set null distribution of ES for **ZHANG\_UTERUS\_C5\_MACROPHAGE**

  
