# Supplementary material for: TMPRSS11B promotes an acidified microenvironment and immune suppression in squamous lung cancer
Source: EMBO Rep. 2025 Nov 10;26(24):6346–79. doi: 10.1038/s44319-025-00631-1 (PMC12714794; doi:10.1038/s44319-025-00631-1)
Supplement: Supplementary file 18 — Figure EV6 Source Data [file 44319_2025_631_MOESM18_ESM.zip › Figure EV6/EV6C-D/GSEA_Broad Institute_M8_T11b high vs low LUSC/ZHANG_UTERUS_C9_DENDRITIC_CELL.html]

Details for gene set ZHANG\_UTERUS\_C9\_DENDRITIC\_CELL[GSEA]

|  || Dataset | T11b high vs low squamous\_GSEA\_Ranked |
| Phenotype | NoPhenotypeAvailable |
| Upregulated in class | na\_pos |
| GeneSet | ZHANG\_UTERUS\_C9\_DENDRITIC\_CELL |
| Enrichment Score (ES) | 0.75108916 |
| Normalized Enrichment Score (NES) | 3.8734698 |
| Nominal p-value | 0.0 |
| FDR q-value | 0.0 |
| FWER p-Value | 0.0 |
Table: GSEA Results Summary

  

Fig 1: Enrichment plot: ZHANG\_UTERUS\_C9\_DENDRITIC\_CELL      
 Profile of the Running ES Score & Positions of GeneSet Members on the Rank Ordered List

  

| SYMBOL | RANK IN GENE LIST | RANK METRIC SCORE | RUNNING ES | CORE ENRICHMENT || 1 | Ctss | 63 | 2.582 | 0.0312 | Yes |
| 2 | Fcer1g | 76 | 2.415 | 0.0719 | Yes |
| 3 | Tyrobp | 83 | 2.366 | 0.1133 | Yes |
| 4 | Plek | 95 | 2.237 | 0.1511 | Yes |
| 5 | Mpeg1 | 114 | 2.045 | 0.1836 | Yes |
| 6 | Wfdc17 | 117 | 1.998 | 0.2193 | Yes |
| 7 | Il1b | 129 | 1.912 | 0.2512 | Yes |
| 8 | Ctsz | 138 | 1.884 | 0.2834 | Yes |
| 9 | Lgmn | 141 | 1.877 | 0.3168 | Yes |
| 10 | Fth1 | 147 | 1.835 | 0.3488 | Yes |
| 11 | Fxyd5 | 157 | 1.767 | 0.3786 | Yes |
| 12 | Spi1 | 158 | 1.765 | 0.4106 | Yes |
| 13 | Ccl6 | 166 | 1.733 | 0.4402 | Yes |
| 14 | Il1r2 | 188 | 1.630 | 0.4645 | Yes |
| 15 | Cdkn1a | 192 | 1.625 | 0.4932 | Yes |
| 16 | Arhgdib | 214 | 1.549 | 0.5161 | Yes |
| 17 | Lcp1 | 231 | 1.490 | 0.5391 | Yes |
| 18 | Psap | 240 | 1.466 | 0.5636 | Yes |
| 19 | Pim1 | 262 | 1.425 | 0.5843 | Yes |
| 20 | Srgn | 270 | 1.392 | 0.6077 | Yes |
| 21 | Cd53 | 337 | 1.171 | 0.6126 | Yes |
| 22 | Cxcl16 | 342 | 1.161 | 0.6327 | Yes |
| 23 | Cd52 | 350 | 1.140 | 0.6516 | Yes |
| 24 | Bcl2a1b | 359 | 1.126 | 0.6700 | Yes |
| 25 | Lgals3 | 377 | 1.096 | 0.6856 | Yes |
| 26 | Coro1a | 390 | 1.079 | 0.7022 | Yes |
| 27 | Alox5ap | 399 | 1.051 | 0.7192 | Yes |
| 28 | Cyba | 519 | 0.875 | 0.7056 | Yes |
| 29 | Rgs1 | 526 | 0.873 | 0.7200 | Yes |
| 30 | Actb | 633 | 0.726 | 0.7069 | Yes |
| 31 | Crip1 | 638 | 0.721 | 0.7190 | Yes |
| 32 | Cotl1 | 656 | 0.709 | 0.7276 | Yes |
| 33 | H2-D1 | 719 | 0.654 | 0.7241 | Yes |
| 34 | Gm2a | 815 | 0.585 | 0.7112 | Yes |
| 35 | Cd74 | 849 | 0.567 | 0.7134 | Yes |
| 36 | Rilpl2 | 852 | 0.566 | 0.7231 | Yes |
| 37 | H2-K1 | 855 | 0.565 | 0.7328 | Yes |
| 38 | B2m | 860 | 0.563 | 0.7420 | Yes |
| 39 | Cfl1 | 895 | 0.538 | 0.7434 | Yes |
| 40 | H2-Ab1 | 915 | 0.525 | 0.7482 | Yes |
| 41 | Mcl1 | 950 | 0.505 | 0.7489 | Yes |
| 42 | Ucp2 | 979 | -0.502 | 0.7511 | Yes |
| 43 | Rgs2 | 2919 | -0.946 | 0.2891 | No |
Table: GSEA details [plain text format]

  

Fig 2: ZHANG\_UTERUS\_C9\_DENDRITIC\_CELL: Random ES distribution      
 Gene set null distribution of ES for **ZHANG\_UTERUS\_C9\_DENDRITIC\_CELL**

  
