# Supplementary material for: TMPRSS11B promotes an acidified microenvironment and immune suppression in squamous lung cancer
Source: EMBO Rep. 2025 Nov 10;26(24):6346–79. doi: 10.1038/s44319-025-00631-1 (PMC12714794; doi:10.1038/s44319-025-00631-1)
Supplement: Supplementary file 19 — Appendix Figure S1 Source Data [file 44319_2025_631_MOESM19_ESM.zip › Appendix Figure S1/S1D/Read Me.rtf]

The spatial transcriptomics data used to generate the plots have been deposited to GEO and the accession number included in the manuscript. Also, the marker genes used for defining the immune cell populations are provided in the source data for Fig EV6A-B (and also in Table EV3).
