# Supplementary material for: TMPRSS11B promotes an acidified microenvironment and immune suppression in squamous lung cancer
Source: EMBO Rep. 2025 Nov 10;26(24):6346–79. doi: 10.1038/s44319-025-00631-1 (PMC12714794; doi:10.1038/s44319-025-00631-1)
Supplement: Supplementary file 19 — Appendix Figure S1 Source Data [file 44319_2025_631_MOESM19_ESM.zip › Appendix Figure S1/S1C/GSEA Broad Institute_low pH vs rest of the regions (high pH)_Mh/gsea_report_for_na_neg_1723672247527.html]

Report for na\_neg 1723672247527 [GSEA]

| GS  follow link to MSigDB | GS DETAILS | SIZE | ES | NES | NOM p-val | FDR q-val | FWER p-val | RANK AT MAX | LEADING EDGE || 1 | HALLMARK\_KRAS\_SIGNALING\_DN | Details ... | 30 | -0.70 | -2.92 | 0.000 | 0.000 | 0.000 | 379 | tags=63%, list=12%, signal=72% |
| 2 | HALLMARK\_ESTROGEN\_RESPONSE\_LATE | Details ... | 65 | -0.43 | -2.12 | 0.000 | 0.003 | 0.009 | 690 | tags=45%, list=23%, signal=56% |
| 3 | HALLMARK\_ESTROGEN\_RESPONSE\_EARLY | Details ... | 80 | -0.37 | -1.96 | 0.000 | 0.010 | 0.047 | 589 | tags=36%, list=19%, signal=44% |
| 4 | HALLMARK\_E2F\_TARGETS | Details ... | 15 | -0.45 | -1.56 | 0.042 | 0.209 | 0.710 | 1374 | tags=87%, list=45%, signal=157% |
| 5 | HALLMARK\_MYC\_TARGETS\_V1 | Details ... | 30 | -0.36 | -1.52 | 0.051 | 0.200 | 0.775 | 1947 | tags=100%, list=64%, signal=275% |
| 6 | HALLMARK\_G2M\_CHECKPOINT | Details ... | 27 | -0.37 | -1.48 | 0.084 | 0.220 | 0.863 | 1363 | tags=74%, list=45%, signal=133% |
| 7 | HALLMARK\_ANDROGEN\_RESPONSE | Details ... | 29 | -0.34 | -1.41 | 0.102 | 0.260 | 0.936 | 1000 | tags=59%, list=33%, signal=87% |
| 8 | HALLMARK\_GLYCOLYSIS | Details ... | 66 | -0.26 | -1.29 | 0.159 | 0.420 | 0.997 | 690 | tags=35%, list=23%, signal=44% |
| 9 | HALLMARK\_BILE\_ACID\_METABOLISM | Details ... | 24 | -0.28 | -1.09 | 0.350 | 0.777 | 1.000 | 836 | tags=46%, list=28%, signal=63% |
| 10 | HALLMARK\_XENOBIOTIC\_METABOLISM | Details ... | 58 | -0.23 | -1.08 | 0.365 | 0.725 | 1.000 | 770 | tags=38%, list=25%, signal=50% |
| 11 | HALLMARK\_MYC\_TARGETS\_V2 | Details ... | 16 | -0.30 | -1.02 | 0.429 | 0.791 | 1.000 | 1902 | tags=94%, list=63%, signal=249% |
| 12 | HALLMARK\_HYPOXIA | Details ... | 63 | -0.20 | -1.00 | 0.475 | 0.765 | 1.000 | 346 | tags=16%, list=11%, signal=18% |
| 13 | HALLMARK\_UNFOLDED\_PROTEIN\_RESPONSE | Details ... | 27 | -0.24 | -0.97 | 0.493 | 0.771 | 1.000 | 626 | tags=30%, list=21%, signal=37% |
| 14 | HALLMARK\_FATTY\_ACID\_METABOLISM | Details ... | 43 | -0.20 | -0.91 | 0.604 | 0.833 | 1.000 | 1203 | tags=56%, list=40%, signal=91% |
| 15 | HALLMARK\_OXIDATIVE\_PHOSPHORYLATION | Details ... | 30 | -0.20 | -0.83 | 0.666 | 0.918 | 1.000 | 2153 | tags=93%, list=71%, signal=317% |
| 16 | HALLMARK\_MTORC1\_SIGNALING | Details ... | 46 | -0.16 | -0.75 | 0.790 | 0.987 | 1.000 | 726 | tags=33%, list=24%, signal=42% |
| 17 | HALLMARK\_INTERFERON\_ALPHA\_RESPONSE | Details ... | 15 | -0.23 | -0.75 | 0.738 | 0.933 | 1.000 | 649 | tags=33%, list=21%, signal=42% |
| 18 | HALLMARK\_P53\_PATHWAY | Details ... | 55 | -0.14 | -0.70 | 0.853 | 0.939 | 1.000 | 818 | tags=29%, list=27%, signal=39% |
| 19 | HALLMARK\_HEME\_METABOLISM | Details ... | 42 | -0.15 | -0.68 | 0.841 | 0.913 | 1.000 | 404 | tags=17%, list=13%, signal=19% |
| 20 | HALLMARK\_MITOTIC\_SPINDLE | Details ... | 38 | -0.12 | -0.51 | 0.968 | 0.974 | 1.000 | 2334 | tags=92%, list=77%, signal=392% |
Table: Gene sets enriched in phenotype **na**[plain text format]****

  
