# Supplementary material for: TMPRSS11B promotes an acidified microenvironment and immune suppression in squamous lung cancer
Source: EMBO Rep. 2025 Nov 10;26(24):6346–79. doi: 10.1038/s44319-025-00631-1 (PMC12714794; doi:10.1038/s44319-025-00631-1)
Supplement: Supplementary file 19 — Appendix Figure S1 Source Data [file 44319_2025_631_MOESM19_ESM.zip › Appendix Figure S1/S1C/GSEA Broad Institute_low pH vs rest of the regions (high pH)_Mh/gsea_report_for_na_pos_1723672247527.html]

Report for na\_pos 1723672247527 [GSEA]

| GS  follow link to MSigDB | GS DETAILS | SIZE | ES | NES | NOM p-val | FDR q-val | FWER p-val | RANK AT MAX | LEADING EDGE || 1 | HALLMARK\_ALLOGRAFT\_REJECTION | Details ... | 45 | 0.54 | 3.07 | 0.000 | 0.000 | 0.000 | 740 | tags=71%, list=24%, signal=93% |
| 2 | HALLMARK\_EPITHELIAL\_MESENCHYMAL\_TRANSITION | Details ... | 79 | 0.46 | 2.98 | 0.000 | 0.000 | 0.000 | 870 | tags=71%, list=29%, signal=97% |
| 3 | HALLMARK\_COMPLEMENT | Details ... | 64 | 0.44 | 2.76 | 0.000 | 0.000 | 0.000 | 525 | tags=47%, list=17%, signal=55% |
| 4 | HALLMARK\_COAGULATION | Details ... | 47 | 0.42 | 2.38 | 0.000 | 0.001 | 0.002 | 658 | tags=55%, list=22%, signal=70% |
| 5 | HALLMARK\_IL6\_JAK\_STAT3\_SIGNALING | Details ... | 25 | 0.47 | 2.22 | 0.000 | 0.002 | 0.007 | 954 | tags=84%, list=31%, signal=121% |
| 6 | HALLMARK\_INTERFERON\_GAMMA\_RESPONSE | Details ... | 45 | 0.31 | 1.77 | 0.004 | 0.038 | 0.110 | 749 | tags=49%, list=25%, signal=64% |
| 7 | HALLMARK\_KRAS\_SIGNALING\_UP | Details ... | 79 | 0.27 | 1.75 | 0.005 | 0.037 | 0.125 | 778 | tags=48%, list=26%, signal=63% |
| 8 | HALLMARK\_APICAL\_JUNCTION | Details ... | 52 | 0.28 | 1.67 | 0.027 | 0.054 | 0.202 | 907 | tags=58%, list=30%, signal=81% |
| 9 | HALLMARK\_INFLAMMATORY\_RESPONSE | Details ... | 49 | 0.28 | 1.63 | 0.020 | 0.063 | 0.256 | 751 | tags=47%, list=25%, signal=61% |
| 10 | HALLMARK\_ADIPOGENESIS | Details ... | 56 | 0.24 | 1.46 | 0.072 | 0.145 | 0.531 | 752 | tags=36%, list=25%, signal=47% |
| 11 | HALLMARK\_ANGIOGENESIS | Details ... | 15 | 0.35 | 1.37 | 0.116 | 0.201 | 0.686 | 728 | tags=67%, list=24%, signal=87% |
| 12 | HALLMARK\_CHOLESTEROL\_HOMEOSTASIS | Details ... | 24 | 0.28 | 1.33 | 0.132 | 0.227 | 0.761 | 161 | tags=21%, list=5%, signal=22% |
| 13 | HALLMARK\_MYOGENESIS | Details ... | 49 | 0.21 | 1.21 | 0.208 | 0.343 | 0.909 | 934 | tags=53%, list=31%, signal=75% |
| 14 | HALLMARK\_IL2\_STAT5\_SIGNALING | Details ... | 68 | 0.18 | 1.15 | 0.290 | 0.421 | 0.959 | 907 | tags=44%, list=30%, signal=61% |
| 15 | HALLMARK\_UV\_RESPONSE\_DN | Details ... | 47 | 0.20 | 1.13 | 0.311 | 0.419 | 0.968 | 710 | tags=38%, list=23%, signal=49% |
| 16 | HALLMARK\_DNA\_REPAIR | Details ... | 22 | 0.23 | 1.02 | 0.421 | 0.587 | 0.994 | 2349 | tags=100%, list=77%, signal=437% |
| 17 | HALLMARK\_UV\_RESPONSE\_UP | Details ... | 39 | 0.14 | 0.80 | 0.760 | 0.978 | 1.000 | 524 | tags=23%, list=17%, signal=28% |
| 18 | HALLMARK\_PI3K\_AKT\_MTOR\_SIGNALING | Details ... | 22 | 0.16 | 0.72 | 0.831 | 1.000 | 1.000 | 973 | tags=50%, list=32%, signal=73% |
| 19 | HALLMARK\_TNFA\_SIGNALING\_VIA\_NFKB | Details ... | 54 | 0.11 | 0.68 | 0.908 | 1.000 | 1.000 | 1098 | tags=48%, list=36%, signal=74% |
| 20 | HALLMARK\_APOPTOSIS | Details ... | 48 | 0.12 | 0.67 | 0.910 | 0.986 | 1.000 | 494 | tags=19%, list=16%, signal=22% |
| 21 | HALLMARK\_PROTEIN\_SECRETION |  | 18 | 0.15 | 0.63 | 0.935 | 0.976 | 1.000 | 2292 | tags=94%, list=75%, signal=382% |
| 22 | HALLMARK\_PEROXISOME |  | 19 | 0.14 | 0.61 | 0.928 | 0.942 | 1.000 | 815 | tags=37%, list=27%, signal=50% |
Table: Gene sets enriched in phenotype **na**[plain text format]****

  
