# Supplementary material for: TMPRSS11B promotes an acidified microenvironment and immune suppression in squamous lung cancer
Source: EMBO Rep. 2025 Nov 10;26(24):6346–79. doi: 10.1038/s44319-025-00631-1 (PMC12714794; doi:10.1038/s44319-025-00631-1)
Supplement: Supplementary file 19 — Appendix Figure S1 Source Data [file 44319_2025_631_MOESM19_ESM.zip › Appendix Figure S1/S1C/GSEA Broad Institute_low pH vs rest of the regions (high pH)_Mh/HALLMARK_ADIPOGENESIS.html]

Details for gene set HALLMARK\_ADIPOGENESIS[GSEA]

|  || Dataset | Lactate high vs low\_Ranked |
| Phenotype | NoPhenotypeAvailable |
| Upregulated in class | na\_pos |
| GeneSet | HALLMARK\_ADIPOGENESIS |
| Enrichment Score (ES) | 0.23939516 |
| Normalized Enrichment Score (NES) | 1.4553547 |
| Nominal p-value | 0.072072074 |
| FDR q-value | 0.14502762 |
| FWER p-Value | 0.531 |
Table: GSEA Results Summary

  

Fig 1: Enrichment plot: HALLMARK\_ADIPOGENESIS      
 Profile of the Running ES Score & Positions of GeneSet Members on the Rank Ordered List

  

| SYMBOL | RANK IN GENE LIST | RANK METRIC SCORE | RUNNING ES | CORE ENRICHMENT || 1 | Apoe | 6 | 2.177 | 0.0409 | Yes |
| 2 | Fabp4 | 12 | 2.100 | 0.0807 | Yes |
| 3 | Lpl | 34 | 1.833 | 0.1099 | Yes |
| 4 | Cd36 | 52 | 1.748 | 0.1387 | Yes |
| 5 | Plin2 | 90 | 1.607 | 0.1580 | Yes |
| 6 | Abca1 | 206 | 1.367 | 0.1464 | Yes |
| 7 | Cd302 | 283 | 1.237 | 0.1454 | Yes |
| 8 | Col4a1 | 286 | 1.235 | 0.1691 | Yes |
| 9 | Col15a1 | 289 | 1.234 | 0.1928 | Yes |
| 10 | Cat | 297 | 1.225 | 0.2146 | Yes |
| 11 | Mgll | 316 | 1.208 | 0.2324 | Yes |
| 12 | Enpp2 | 553 | 0.948 | 0.1720 | Yes |
| 13 | Sparcl1 | 566 | 0.938 | 0.1865 | Yes |
| 14 | Mylk | 588 | 0.905 | 0.1973 | Yes |
| 15 | Cavin2 | 626 | 0.870 | 0.2021 | Yes |
| 16 | Esyt1 | 678 | 0.829 | 0.2014 | Yes |
| 17 | Lama4 | 698 | 0.811 | 0.2110 | Yes |
| 18 | Scarb1 | 738 | 0.772 | 0.2132 | Yes |
| 19 | Ifngr1 | 740 | 0.770 | 0.2280 | Yes |
| 20 | Dhrs7 | 752 | 0.762 | 0.2394 | Yes |
| 21 | Rab34 | 891 | 0.637 | 0.2057 | No |
| 22 | Slc27a1 | 896 | 0.632 | 0.2169 | No |
| 23 | Itga7 | 899 | 0.630 | 0.2286 | No |
| 24 | C3 | 948 | 0.602 | 0.2244 | No |
| 25 | Ywhag | 981 | 0.575 | 0.2250 | No |
| 26 | Dgat1 | 987 | 0.571 | 0.2346 | No |
| 27 | Gpx3 | 1070 | 0.527 | 0.2176 | No |
| 28 | Stom | 1085 | 0.517 | 0.2231 | No |
| 29 | Sowahc | 1089 | 0.509 | 0.2321 | No |
| 30 | Slc25a1 | 1126 | -0.504 | 0.2300 | No |
| 31 | Coq9 | 1192 | -0.518 | 0.2184 | No |
| 32 | Idh3g | 1205 | -0.521 | 0.2247 | No |
| 33 | Slc25a10 | 1343 | -0.551 | 0.1897 | No |
| 34 | Mccc1 | 1402 | -0.563 | 0.1813 | No |
| 35 | Idh3a | 1408 | -0.565 | 0.1908 | No |
| 36 | Gpd2 | 1481 | -0.582 | 0.1782 | No |
| 37 | Phyh | 1502 | -0.587 | 0.1830 | No |
| 38 | Gpat4 | 1643 | -0.631 | 0.1486 | No |
| 39 | Sqor | 1649 | -0.632 | 0.1594 | No |
| 40 | Ephx2 | 1664 | -0.637 | 0.1673 | No |
| 41 | Retsat | 1742 | -0.669 | 0.1547 | No |
| 42 | Elmod3 | 1847 | -0.706 | 0.1338 | No |
| 43 | Hadh | 1869 | -0.711 | 0.1408 | No |
| 44 | Tob1 | 1942 | -0.737 | 0.1312 | No |
| 45 | Acadm | 1975 | -0.750 | 0.1352 | No |
| 46 | Atl2 | 1993 | -0.757 | 0.1445 | No |
| 47 | Gphn | 2003 | -0.762 | 0.1565 | No |
| 48 | Cmbl | 2054 | -0.787 | 0.1553 | No |
| 49 | Pim3 | 2306 | -0.930 | 0.0895 | No |
| 50 | Acadl | 2329 | -0.941 | 0.1007 | No |
| 51 | Slc1a5 | 2375 | -0.989 | 0.1052 | No |
| 52 | Por | 2395 | -1.005 | 0.1186 | No |
| 53 | Dbt | 2469 | -1.064 | 0.1152 | No |
| 54 | Hspb8 | 2503 | -1.097 | 0.1258 | No |
| 55 | Reep6 | 2570 | -1.171 | 0.1268 | No |
| 56 | Tst | 2769 | -1.524 | 0.0905 | No |
Table: GSEA details [plain text format]

  

Fig 2: HALLMARK\_ADIPOGENESIS: Random ES distribution      
 Gene set null distribution of ES for **HALLMARK\_ADIPOGENESIS**

  
