# Supplementary material for: TMPRSS11B promotes an acidified microenvironment and immune suppression in squamous lung cancer
Source: EMBO Rep. 2025 Nov 10;26(24):6346–79. doi: 10.1038/s44319-025-00631-1 (PMC12714794; doi:10.1038/s44319-025-00631-1)
Supplement: Supplementary file 19 — Appendix Figure S1 Source Data [file 44319_2025_631_MOESM19_ESM.zip › Appendix Figure S1/S1C/GSEA Broad Institute_low pH vs rest of the regions (high pH)_Mh/HALLMARK_ALLOGRAFT_REJECTION.html]

Details for gene set HALLMARK\_ALLOGRAFT\_REJECTION[GSEA]

|  || Dataset | Lactate high vs low\_Ranked |
| Phenotype | NoPhenotypeAvailable |
| Upregulated in class | na\_pos |
| GeneSet | HALLMARK\_ALLOGRAFT\_REJECTION |
| Enrichment Score (ES) | 0.5387431 |
| Normalized Enrichment Score (NES) | 3.0663192 |
| Nominal p-value | 0.0 |
| FDR q-value | 0.0 |
| FWER p-Value | 0.0 |
Table: GSEA Results Summary

  

Fig 1: Enrichment plot: HALLMARK\_ALLOGRAFT\_REJECTION      
 Profile of the Running ES Score & Positions of GeneSet Members on the Rank Ordered List

  

| SYMBOL | RANK IN GENE LIST | RANK METRIC SCORE | RUNNING ES | CORE ENRICHMENT || 1 | Ctss | 14 | 2.088 | 0.0367 | Yes |
| 2 | Ly86 | 66 | 1.677 | 0.0529 | Yes |
| 3 | Spi1 | 86 | 1.616 | 0.0786 | Yes |
| 4 | Was | 89 | 1.612 | 0.1098 | Yes |
| 5 | Fcgr2b | 102 | 1.572 | 0.1370 | Yes |
| 6 | Itgb2 | 138 | 1.498 | 0.1550 | Yes |
| 7 | Gpr65 | 151 | 1.461 | 0.1799 | Yes |
| 8 | Hcls1 | 156 | 1.451 | 0.2073 | Yes |
| 9 | Cfp | 168 | 1.424 | 0.2319 | Yes |
| 10 | Ccr5 | 173 | 1.410 | 0.2585 | Yes |
| 11 | Igsf6 | 198 | 1.379 | 0.2778 | Yes |
| 12 | St8sia4 | 225 | 1.343 | 0.2957 | Yes |
| 13 | Ptprc | 255 | 1.283 | 0.3115 | Yes |
| 14 | Prkcb | 268 | 1.263 | 0.3325 | Yes |
| 15 | Ccr2 | 288 | 1.234 | 0.3506 | Yes |
| 16 | Fgr | 296 | 1.227 | 0.3726 | Yes |
| 17 | Cd74 | 376 | 1.133 | 0.3686 | Yes |
| 18 | Il2rg | 380 | 1.128 | 0.3900 | Yes |
| 19 | B2m | 402 | 1.097 | 0.4047 | Yes |
| 20 | Nos2 | 407 | 1.092 | 0.4250 | Yes |
| 21 | H2-DMa | 418 | 1.083 | 0.4431 | Yes |
| 22 | Lcp2 | 449 | 1.047 | 0.4538 | Yes |
| 23 | Fas | 457 | 1.039 | 0.4721 | Yes |
| 24 | H2-Aa | 465 | 1.035 | 0.4903 | Yes |
| 25 | Icam1 | 492 | 0.999 | 0.5014 | Yes |
| 26 | Mmp9 | 499 | 0.993 | 0.5191 | Yes |
| 27 | H2-DMb2 | 630 | 0.867 | 0.4928 | Yes |
| 28 | Irf8 | 663 | 0.840 | 0.4988 | Yes |
| 29 | Tgfb1 | 664 | 0.839 | 0.5154 | Yes |
| 30 | Ets1 | 671 | 0.831 | 0.5299 | Yes |
| 31 | Flna | 707 | 0.805 | 0.5342 | Yes |
| 32 | Ifngr1 | 740 | 0.770 | 0.5387 | Yes |
| 33 | Ltb | 902 | 0.628 | 0.4974 | No |
| 34 | Ifnar2 | 958 | 0.597 | 0.4909 | No |
| 35 | F2r | 1002 | 0.562 | 0.4877 | No |
| 36 | Ly75 | 1042 | 0.542 | 0.4854 | No |
| 37 | Ptpn6 | 1096 | 0.505 | 0.4777 | No |
| 38 | Egfr | 1252 | -0.532 | 0.4365 | No |
| 39 | Bcl3 | 1709 | -0.657 | 0.2973 | No |
| 40 | Cdkn2a | 2223 | -0.874 | 0.1433 | No |
| 41 | Ccnd2 | 2242 | -0.887 | 0.1548 | No |
| 42 | Il18 | 2586 | -1.191 | 0.0639 | No |
| 43 | Irf7 | 2638 | -1.265 | 0.0719 | No |
| 44 | Gcnt1 | 2743 | -1.463 | 0.0662 | No |
| 45 | Tlr2 | 2819 | -1.629 | 0.0735 | No |
Table: GSEA details [plain text format]

  

Fig 2: HALLMARK\_ALLOGRAFT\_REJECTION: Random ES distribution      
 Gene set null distribution of ES for **HALLMARK\_ALLOGRAFT\_REJECTION**

  
