# Supplementary material for: TMPRSS11B promotes an acidified microenvironment and immune suppression in squamous lung cancer
Source: EMBO Rep. 2025 Nov 10;26(24):6346–79. doi: 10.1038/s44319-025-00631-1 (PMC12714794; doi:10.1038/s44319-025-00631-1)
Supplement: Supplementary file 19 — Appendix Figure S1 Source Data [file 44319_2025_631_MOESM19_ESM.zip › Appendix Figure S1/S1C/GSEA Broad Institute_low pH vs rest of the regions (high pH)_Mh/HALLMARK_ANDROGEN_RESPONSE.html]

Details for gene set HALLMARK\_ANDROGEN\_RESPONSE[GSEA]

|  || Dataset | Lactate high vs low\_Ranked |
| Phenotype | NoPhenotypeAvailable |
| Upregulated in class | na\_neg |
| GeneSet | HALLMARK\_ANDROGEN\_RESPONSE |
| Enrichment Score (ES) | -0.34423843 |
| Normalized Enrichment Score (NES) | -1.4140304 |
| Nominal p-value | 0.10164425 |
| FDR q-value | 0.25980195 |
| FWER p-Value | 0.936 |
Table: GSEA Results Summary

  

Fig 1: Enrichment plot: HALLMARK\_ANDROGEN\_RESPONSE      
 Profile of the Running ES Score & Positions of GeneSet Members on the Rank Ordered List

  

| SYMBOL | RANK IN GENE LIST | RANK METRIC SCORE | RUNNING ES | CORE ENRICHMENT || 1 | Mertk | 24 | 1.934 | 0.0576 | No |
| 2 | B2m | 402 | 1.097 | -0.0305 | No |
| 3 | Ncoa4 | 683 | 0.825 | -0.0955 | No |
| 4 | Akap12 | 713 | 0.801 | -0.0780 | No |
| 5 | Adamts1 | 723 | 0.793 | -0.0541 | No |
| 6 | Gucy1a1 | 759 | 0.752 | -0.0403 | No |
| 7 | Fkbp5 | 935 | 0.606 | -0.0779 | No |
| 8 | Krt8 | 1193 | -0.519 | -0.1457 | No |
| 9 | Hmgcr | 1281 | -0.538 | -0.1563 | No |
| 10 | Pmepa1 | 1426 | -0.570 | -0.1848 | No |
| 11 | Appbp2 | 1480 | -0.581 | -0.1827 | No |
| 12 | Ank | 1878 | -0.713 | -0.2904 | No |
| 13 | Ngly1 | 2041 | -0.779 | -0.3178 | Yes |
| 14 | Tnfaip8 | 2050 | -0.785 | -0.2939 | Yes |
| 15 | Iqgap2 | 2051 | -0.786 | -0.2673 | Yes |
| 16 | Hmgcs1 | 2251 | -0.894 | -0.3031 | Yes |
| 17 | Slc38a2 | 2296 | -0.923 | -0.2864 | Yes |
| 18 | Dhcr24 | 2316 | -0.935 | -0.2611 | Yes |
| 19 | Gpd1l | 2397 | -1.006 | -0.2536 | Yes |
| 20 | Pgm3 | 2398 | -1.006 | -0.2195 | Yes |
| 21 | Slc26a2 | 2411 | -1.012 | -0.1892 | Yes |
| 22 | Krt19 | 2476 | -1.067 | -0.1743 | Yes |
| 23 | Acsl3 | 2510 | -1.103 | -0.1479 | Yes |
| 24 | Tmprss2 | 2557 | -1.159 | -0.1239 | Yes |
| 25 | Ell2 | 2637 | -1.264 | -0.1073 | Yes |
| 26 | Spdef | 2763 | -1.514 | -0.0975 | Yes |
| 27 | Abcc4 | 2791 | -1.569 | -0.0533 | Yes |
| 28 | Aldh1a3 | 2868 | -1.845 | -0.0160 | Yes |
| 29 | Homer2 | 2918 | -2.140 | 0.0402 | Yes |
Table: GSEA details [plain text format]

  

Fig 2: HALLMARK\_ANDROGEN\_RESPONSE: Random ES distribution      
 Gene set null distribution of ES for **HALLMARK\_ANDROGEN\_RESPONSE**

  
