# Supplementary material for: TMPRSS11B promotes an acidified microenvironment and immune suppression in squamous lung cancer
Source: EMBO Rep. 2025 Nov 10;26(24):6346–79. doi: 10.1038/s44319-025-00631-1 (PMC12714794; doi:10.1038/s44319-025-00631-1)
Supplement: Supplementary file 19 — Appendix Figure S1 Source Data [file 44319_2025_631_MOESM19_ESM.zip › Appendix Figure S1/S1C/GSEA Broad Institute_low pH vs rest of the regions (high pH)_Mh/HALLMARK_ANGIOGENESIS.html]

Details for gene set HALLMARK\_ANGIOGENESIS[GSEA]

|  || Dataset | Lactate high vs low\_Ranked |
| Phenotype | NoPhenotypeAvailable |
| Upregulated in class | na\_pos |
| GeneSet | HALLMARK\_ANGIOGENESIS |
| Enrichment Score (ES) | 0.350368 |
| Normalized Enrichment Score (NES) | 1.3727691 |
| Nominal p-value | 0.116094984 |
| FDR q-value | 0.20059662 |
| FWER p-Value | 0.686 |
Table: GSEA Results Summary

  

Fig 1: Enrichment plot: HALLMARK\_ANGIOGENESIS      
 Profile of the Running ES Score & Positions of GeneSet Members on the Rank Ordered List

  

| SYMBOL | RANK IN GENE LIST | RANK METRIC SCORE | RUNNING ES | CORE ENRICHMENT || 1 | Lpl | 34 | 1.833 | 0.0841 | Yes |
| 2 | Spp1 | 232 | 1.327 | 0.0880 | Yes |
| 3 | Thbd | 306 | 1.215 | 0.1271 | Yes |
| 4 | Nrp1 | 353 | 1.158 | 0.1722 | Yes |
| 5 | Col5a2 | 387 | 1.116 | 0.2193 | Yes |
| 6 | Postn | 400 | 1.098 | 0.2725 | Yes |
| 7 | Col3a1 | 501 | 0.992 | 0.2910 | Yes |
| 8 | Fstl1 | 573 | 0.928 | 0.3158 | Yes |
| 9 | Vegfa | 650 | 0.852 | 0.3350 | Yes |
| 10 | Slco2a1 | 728 | 0.785 | 0.3504 | Yes |
| 11 | Tnfrsf21 | 1702 | -0.654 | 0.0627 | No |
| 12 | Jag1 | 1979 | -0.750 | 0.0105 | No |
| 13 | Ccnd2 | 2242 | -0.887 | -0.0300 | No |
| 14 | Pglyrp1 | 2963 | -2.492 | -0.1383 | No |
| 15 | Cxcl5 | 2999 | -3.136 | 0.0132 | No |
Table: GSEA details [plain text format]

  

Fig 2: HALLMARK\_ANGIOGENESIS: Random ES distribution      
 Gene set null distribution of ES for **HALLMARK\_ANGIOGENESIS**

  
