# Supplementary material for: TMPRSS11B promotes an acidified microenvironment and immune suppression in squamous lung cancer
Source: EMBO Rep. 2025 Nov 10;26(24):6346–79. doi: 10.1038/s44319-025-00631-1 (PMC12714794; doi:10.1038/s44319-025-00631-1)
Supplement: Supplementary file 19 — Appendix Figure S1 Source Data [file 44319_2025_631_MOESM19_ESM.zip › Appendix Figure S1/S1C/GSEA Broad Institute_low pH vs rest of the regions (high pH)_Mh/HALLMARK_APICAL_JUNCTION.html]

Details for gene set HALLMARK\_APICAL\_JUNCTION[GSEA]

|  || Dataset | Lactate high vs low\_Ranked |
| Phenotype | NoPhenotypeAvailable |
| Upregulated in class | na\_pos |
| GeneSet | HALLMARK\_APICAL\_JUNCTION |
| Enrichment Score (ES) | 0.27776787 |
| Normalized Enrichment Score (NES) | 1.6734371 |
| Nominal p-value | 0.027131783 |
| FDR q-value | 0.05418867 |
| FWER p-Value | 0.202 |
Table: GSEA Results Summary

  

Fig 1: Enrichment plot: HALLMARK\_APICAL\_JUNCTION      
 Profile of the Running ES Score & Positions of GeneSet Members on the Rank Ordered List

  

| SYMBOL | RANK IN GENE LIST | RANK METRIC SCORE | RUNNING ES | CORE ENRICHMENT || 1 | Sirpa | 56 | 1.702 | 0.0123 | Yes |
| 2 | Speg | 82 | 1.620 | 0.0335 | Yes |
| 3 | Cd274 | 104 | 1.569 | 0.0551 | Yes |
| 4 | Sdc3 | 106 | 1.564 | 0.0834 | Yes |
| 5 | Layn | 187 | 1.395 | 0.0821 | Yes |
| 6 | Syk | 200 | 1.373 | 0.1031 | Yes |
| 7 | Evl | 204 | 1.369 | 0.1271 | Yes |
| 8 | Cldn5 | 227 | 1.338 | 0.1442 | Yes |
| 9 | Ptprc | 255 | 1.283 | 0.1585 | Yes |
| 10 | Msn | 280 | 1.243 | 0.1732 | Yes |
| 11 | Vwf | 343 | 1.170 | 0.1738 | Yes |
| 12 | Icam2 | 355 | 1.156 | 0.1912 | Yes |
| 13 | Itga9 | 454 | 1.043 | 0.1775 | Yes |
| 14 | Icam1 | 492 | 0.999 | 0.1833 | Yes |
| 15 | Mmp9 | 499 | 0.993 | 0.1995 | Yes |
| 16 | Thbs3 | 537 | 0.959 | 0.2046 | Yes |
| 17 | Fbn1 | 568 | 0.935 | 0.2116 | Yes |
| 18 | Bmp1 | 619 | 0.873 | 0.2108 | Yes |
| 19 | Tspan4 | 625 | 0.870 | 0.2250 | Yes |
| 20 | Tgfbi | 651 | 0.851 | 0.2322 | Yes |
| 21 | Pecam1 | 658 | 0.845 | 0.2456 | Yes |
| 22 | Cnn2 | 727 | 0.789 | 0.2373 | Yes |
| 23 | Rras | 788 | 0.717 | 0.2303 | Yes |
| 24 | Fscn1 | 817 | 0.694 | 0.2336 | Yes |
| 25 | Epb41l2 | 827 | 0.683 | 0.2430 | Yes |
| 26 | Cd34 | 835 | 0.679 | 0.2531 | Yes |
| 27 | Lama3 | 849 | 0.672 | 0.2610 | Yes |
| 28 | Actb | 856 | 0.664 | 0.2711 | Yes |
| 29 | Cldn18 | 889 | 0.637 | 0.2720 | Yes |
| 30 | Traf1 | 907 | 0.626 | 0.2778 | Yes |
| 31 | Adam15 | 974 | 0.580 | 0.2663 | No |
| 32 | Exoc4 | 995 | 0.567 | 0.2699 | No |
| 33 | Rsu1 | 1079 | 0.519 | 0.2516 | No |
| 34 | Egfr | 1252 | -0.532 | 0.2038 | No |
| 35 | Wasl | 1306 | -0.542 | 0.1959 | No |
| 36 | Cldn4 | 1430 | -0.572 | 0.1652 | No |
| 37 | Nectin2 | 1479 | -0.581 | 0.1597 | No |
| 38 | Gtf2f1 | 1622 | -0.623 | 0.1236 | No |
| 39 | Pcdh1 | 1696 | -0.652 | 0.1111 | No |
| 40 | Hadh | 1869 | -0.711 | 0.0665 | No |
| 41 | Mpzl1 | 2081 | -0.800 | 0.0105 | No |
| 42 | Nectin1 | 2239 | -0.886 | -0.0259 | No |
| 43 | Jup | 2337 | -0.950 | -0.0410 | No |
| 44 | Tnfrsf11b | 2433 | -1.037 | -0.0539 | No |
| 45 | Itgb4 | 2445 | -1.047 | -0.0384 | No |
| 46 | Grb7 | 2550 | -1.150 | -0.0523 | No |
| 47 | Cldn7 | 2642 | -1.269 | -0.0595 | No |
| 48 | Cdh1 | 2655 | -1.294 | -0.0399 | No |
| 49 | Mpzl2 | 2710 | -1.388 | -0.0327 | No |
| 50 | Nectin4 | 2786 | -1.558 | -0.0293 | No |
| 51 | Pard6g | 2829 | -1.663 | -0.0130 | No |
| 52 | Col17a1 | 3036 | -4.544 | 0.0010 | No |
Table: GSEA details [plain text format]

  

Fig 2: HALLMARK\_APICAL\_JUNCTION: Random ES distribution      
 Gene set null distribution of ES for **HALLMARK\_APICAL\_JUNCTION**

  
