# Supplementary material for: TMPRSS11B promotes an acidified microenvironment and immune suppression in squamous lung cancer
Source: EMBO Rep. 2025 Nov 10;26(24):6346–79. doi: 10.1038/s44319-025-00631-1 (PMC12714794; doi:10.1038/s44319-025-00631-1)
Supplement: Supplementary file 19 — Appendix Figure S1 Source Data [file 44319_2025_631_MOESM19_ESM.zip › Appendix Figure S1/S1C/GSEA Broad Institute_low pH vs rest of the regions (high pH)_Mh/HALLMARK_APOPTOSIS.html]

Details for gene set HALLMARK\_APOPTOSIS[GSEA]

|  || Dataset | Lactate high vs low\_Ranked |
| Phenotype | NoPhenotypeAvailable |
| Upregulated in class | na\_pos |
| GeneSet | HALLMARK\_APOPTOSIS |
| Enrichment Score (ES) | 0.11915209 |
| Normalized Enrichment Score (NES) | 0.6737978 |
| Nominal p-value | 0.9102564 |
| FDR q-value | 0.985926 |
| FWER p-Value | 1.0 |
Table: GSEA Results Summary

  

Fig 1: Enrichment plot: HALLMARK\_APOPTOSIS      
 Profile of the Running ES Score & Positions of GeneSet Members on the Rank Ordered List

  

| SYMBOL | RANK IN GENE LIST | RANK METRIC SCORE | RUNNING ES | CORE ENRICHMENT || 1 | Hmox1 | 16 | 2.051 | 0.0437 | Yes |
| 2 | Cav1 | 107 | 1.564 | 0.0511 | Yes |
| 3 | Atf3 | 161 | 1.444 | 0.0680 | Yes |
| 4 | Hgf | 247 | 1.294 | 0.0705 | Yes |
| 5 | Timp2 | 334 | 1.180 | 0.0700 | Yes |
| 6 | Lgals3 | 344 | 1.170 | 0.0950 | Yes |
| 7 | Fas | 457 | 1.039 | 0.0825 | Yes |
| 8 | Psen2 | 474 | 1.024 | 0.1017 | Yes |
| 9 | Pdgfrb | 494 | 0.996 | 0.1192 | Yes |
| 10 | Gpx1 | 603 | 0.888 | 0.1043 | No |
| 11 | Ifngr1 | 740 | 0.770 | 0.0773 | No |
| 12 | Cdkn1a | 749 | 0.765 | 0.0929 | No |
| 13 | Mcl1 | 861 | 0.655 | 0.0715 | No |
| 14 | Bgn | 870 | 0.650 | 0.0844 | No |
| 15 | Bcl2l11 | 885 | 0.641 | 0.0951 | No |
| 16 | Timp3 | 959 | 0.596 | 0.0849 | No |
| 17 | F2r | 1002 | 0.562 | 0.0844 | No |
| 18 | Gpx3 | 1070 | 0.527 | 0.0746 | No |
| 19 | Cd44 | 1072 | 0.523 | 0.0868 | No |
| 20 | Bnip3l | 1074 | 0.520 | 0.0989 | No |
| 21 | Madd | 1135 | -0.506 | 0.0909 | No |
| 22 | Hspb1 | 1190 | -0.517 | 0.0853 | No |
| 23 | Erbb3 | 1223 | -0.527 | 0.0872 | No |
| 24 | Gch1 | 1277 | -0.537 | 0.0823 | No |
| 25 | Anxa1 | 1347 | -0.552 | 0.0725 | No |
| 26 | Casp6 | 1431 | -0.572 | 0.0584 | No |
| 27 | Btg2 | 1440 | -0.573 | 0.0694 | No |
| 28 | Pmaip1 | 1451 | -0.575 | 0.0799 | No |
| 29 | Casp4 | 1495 | -0.585 | 0.0795 | No |
| 30 | Crebbp | 1505 | -0.588 | 0.0906 | No |
| 31 | Dap | 1535 | -0.595 | 0.0951 | No |
| 32 | Bid | 1565 | -0.606 | 0.0999 | No |
| 33 | Casp7 | 1587 | -0.613 | 0.1076 | No |
| 34 | Retsat | 1742 | -0.669 | 0.0721 | No |
| 35 | Gstm2 | 1848 | -0.706 | 0.0539 | No |
| 36 | Ank | 1878 | -0.713 | 0.0613 | No |
| 37 | Rara | 1927 | -0.731 | 0.0628 | No |
| 38 | Pak1 | 2001 | -0.762 | 0.0566 | No |
| 39 | Dnajc3 | 2133 | -0.820 | 0.0324 | No |
| 40 | Plat | 2156 | -0.832 | 0.0450 | No |
| 41 | Ccnd2 | 2242 | -0.887 | 0.0378 | No |
| 42 | Sc5d | 2421 | -1.024 | 0.0028 | No |
| 43 | Bik | 2425 | -1.027 | 0.0264 | No |
| 44 | Il18 | 2586 | -1.191 | 0.0015 | No |
| 45 | Erbb2 | 2600 | -1.214 | 0.0262 | No |
| 46 | Dcn | 2701 | -1.362 | 0.0254 | No |
| 47 | Isg20 | 2853 | -1.758 | 0.0170 | No |
| 48 | Clu | 2876 | -1.874 | 0.0545 | No |
Table: GSEA details [plain text format]

  

Fig 2: HALLMARK\_APOPTOSIS: Random ES distribution      
 Gene set null distribution of ES for **HALLMARK\_APOPTOSIS**

  
