# Supplementary material for: TMPRSS11B promotes an acidified microenvironment and immune suppression in squamous lung cancer
Source: EMBO Rep. 2025 Nov 10;26(24):6346–79. doi: 10.1038/s44319-025-00631-1 (PMC12714794; doi:10.1038/s44319-025-00631-1)
Supplement: Supplementary file 19 — Appendix Figure S1 Source Data [file 44319_2025_631_MOESM19_ESM.zip › Appendix Figure S1/S1C/GSEA Broad Institute_low pH vs rest of the regions (high pH)_Mh/HALLMARK_BILE_ACID_METABOLISM.html]

Details for gene set HALLMARK\_BILE\_ACID\_METABOLISM[GSEA]

|  || Dataset | Lactate high vs low\_Ranked |
| Phenotype | NoPhenotypeAvailable |
| Upregulated in class | na\_neg |
| GeneSet | HALLMARK\_BILE\_ACID\_METABOLISM |
| Enrichment Score (ES) | -0.27769154 |
| Normalized Enrichment Score (NES) | -1.0946629 |
| Nominal p-value | 0.3497823 |
| FDR q-value | 0.7771715 |
| FWER p-Value | 1.0 |
Table: GSEA Results Summary

  

Fig 1: Enrichment plot: HALLMARK\_BILE\_ACID\_METABOLISM      
 Profile of the Running ES Score & Positions of GeneSet Members on the Rank Ordered List

  

| SYMBOL | RANK IN GENE LIST | RANK METRIC SCORE | RUNNING ES | CORE ENRICHMENT || 1 | Abca1 | 206 | 1.367 | -0.0062 | No |
| 2 | Cat | 297 | 1.225 | 0.0196 | No |
| 3 | Npc1 | 638 | 0.859 | -0.0542 | No |
| 4 | Hsd3b7 | 754 | 0.760 | -0.0578 | No |
| 5 | Abcd1 | 815 | 0.697 | -0.0460 | No |
| 6 | Pecr | 1110 | -0.502 | -0.1207 | No |
| 7 | Abca5 | 1482 | -0.582 | -0.2173 | No |
| 8 | Phyh | 1502 | -0.587 | -0.1969 | No |
| 9 | Slc22a18 | 1519 | -0.591 | -0.1754 | No |
| 10 | Ephx2 | 1664 | -0.637 | -0.1942 | No |
| 11 | Nudt12 | 1698 | -0.652 | -0.1755 | No |
| 12 | Retsat | 1742 | -0.669 | -0.1594 | No |
| 13 | Gclm | 2000 | -0.761 | -0.2101 | No |
| 14 | Aldh1a1 | 2205 | -0.861 | -0.2386 | Yes |
| 15 | Amacr | 2285 | -0.916 | -0.2232 | Yes |
| 16 | Dhcr24 | 2316 | -0.935 | -0.1907 | Yes |
| 17 | Crot | 2333 | -0.945 | -0.1531 | Yes |
| 18 | Cyp27a1 | 2463 | -1.060 | -0.1478 | Yes |
| 19 | Tfcp2l1 | 2528 | -1.125 | -0.1179 | Yes |
| 20 | Pex26 | 2545 | -1.145 | -0.0713 | Yes |
| 21 | Rbp1 | 2599 | -1.213 | -0.0338 | Yes |
| 22 | Pex7 | 2649 | -1.281 | 0.0081 | Yes |
| 23 | Cyp39a1 | 2663 | -1.308 | 0.0632 | Yes |
| 24 | Acsl1 | 2687 | -1.346 | 0.1167 | Yes |
Table: GSEA details [plain text format]

  

Fig 2: HALLMARK\_BILE\_ACID\_METABOLISM: Random ES distribution      
 Gene set null distribution of ES for **HALLMARK\_BILE\_ACID\_METABOLISM**

  
