# Supplementary material for: TMPRSS11B promotes an acidified microenvironment and immune suppression in squamous lung cancer
Source: EMBO Rep. 2025 Nov 10;26(24):6346–79. doi: 10.1038/s44319-025-00631-1 (PMC12714794; doi:10.1038/s44319-025-00631-1)
Supplement: Supplementary file 19 — Appendix Figure S1 Source Data [file 44319_2025_631_MOESM19_ESM.zip › Appendix Figure S1/S1C/GSEA Broad Institute_low pH vs rest of the regions (high pH)_Mh/HALLMARK_CHOLESTEROL_HOMEOSTASIS.html]

Details for gene set HALLMARK\_CHOLESTEROL\_HOMEOSTASIS[GSEA]

|  || Dataset | Lactate high vs low\_Ranked |
| Phenotype | NoPhenotypeAvailable |
| Upregulated in class | na\_pos |
| GeneSet | HALLMARK\_CHOLESTEROL\_HOMEOSTASIS |
| Enrichment Score (ES) | 0.2793164 |
| Normalized Enrichment Score (NES) | 1.3266494 |
| Nominal p-value | 0.13157895 |
| FDR q-value | 0.22668849 |
| FWER p-Value | 0.761 |
Table: GSEA Results Summary

  

Fig 1: Enrichment plot: HALLMARK\_CHOLESTEROL\_HOMEOSTASIS      
 Profile of the Running ES Score & Positions of GeneSet Members on the Rank Ordered List

  

| SYMBOL | RANK IN GENE LIST | RANK METRIC SCORE | RUNNING ES | CORE ENRICHMENT || 1 | Lgmn | 28 | 1.878 | 0.0655 | Yes |
| 2 | Lpl | 34 | 1.833 | 0.1369 | Yes |
| 3 | Antxr2 | 77 | 1.632 | 0.1880 | Yes |
| 4 | Gusb | 123 | 1.530 | 0.2340 | Yes |
| 5 | Atf3 | 161 | 1.444 | 0.2793 | Yes |
| 6 | Lgals3 | 344 | 1.170 | 0.2656 | No |
| 7 | Cxcl16 | 479 | 1.013 | 0.2615 | No |
| 8 | Anxa5 | 743 | 0.769 | 0.2049 | No |
| 9 | Fasn | 884 | 0.641 | 0.1840 | No |
| 10 | Plaur | 1098 | 0.504 | 0.1335 | No |
| 11 | Hmgcr | 1281 | -0.538 | 0.0946 | No |
| 12 | S100a11 | 1339 | -0.550 | 0.0976 | No |
| 13 | Fabp5 | 1603 | -0.617 | 0.0350 | No |
| 14 | Aldoc | 1714 | -0.659 | 0.0248 | No |
| 15 | Fdft1 | 1952 | -0.741 | -0.0243 | No |
| 16 | Jag1 | 1979 | -0.750 | -0.0030 | No |
| 17 | Cyp51 | 2098 | -0.806 | -0.0101 | No |
| 18 | Acss2 | 2126 | -0.816 | 0.0135 | No |
| 19 | Lss | 2249 | -0.890 | 0.0085 | No |
| 20 | Hmgcs1 | 2251 | -0.894 | 0.0438 | No |
| 21 | Sc5d | 2421 | -1.024 | 0.0286 | No |
| 22 | Sqle | 2622 | -1.234 | 0.0114 | No |
| 23 | Hsd17b7 | 2654 | -1.293 | 0.0526 | No |
| 24 | Clu | 2876 | -1.874 | 0.0540 | No |
Table: GSEA details [plain text format]

  

Fig 2: HALLMARK\_CHOLESTEROL\_HOMEOSTASIS: Random ES distribution      
 Gene set null distribution of ES for **HALLMARK\_CHOLESTEROL\_HOMEOSTASIS**

  
