# Supplementary material for: TMPRSS11B promotes an acidified microenvironment and immune suppression in squamous lung cancer
Source: EMBO Rep. 2025 Nov 10;26(24):6346–79. doi: 10.1038/s44319-025-00631-1 (PMC12714794; doi:10.1038/s44319-025-00631-1)
Supplement: Supplementary file 19 — Appendix Figure S1 Source Data [file 44319_2025_631_MOESM19_ESM.zip › Appendix Figure S1/S1C/GSEA Broad Institute_low pH vs rest of the regions (high pH)_Mh/HALLMARK_COAGULATION.html]

Details for gene set HALLMARK\_COAGULATION[GSEA]

|  || Dataset | Lactate high vs low\_Ranked |
| Phenotype | NoPhenotypeAvailable |
| Upregulated in class | na\_pos |
| GeneSet | HALLMARK\_COAGULATION |
| Enrichment Score (ES) | 0.41685614 |
| Normalized Enrichment Score (NES) | 2.38285 |
| Nominal p-value | 0.0 |
| FDR q-value | 8.8141026E-4 |
| FWER p-Value | 0.002 |
Table: GSEA Results Summary

  

Fig 1: Enrichment plot: HALLMARK\_COAGULATION      
 Profile of the Running ES Score & Positions of GeneSet Members on the Rank Ordered List

  

| SYMBOL | RANK IN GENE LIST | RANK METRIC SCORE | RUNNING ES | CORE ENRICHMENT || 1 | Ctsl | 8 | 2.153 | 0.0358 | Yes |
| 2 | Lgmn | 28 | 1.878 | 0.0630 | Yes |
| 3 | C1qa | 29 | 1.862 | 0.0963 | Yes |
| 4 | Mmp3 | 30 | 1.861 | 0.1296 | Yes |
| 5 | F10 | 33 | 1.837 | 0.1617 | Yes |
| 6 | Apoc1 | 40 | 1.811 | 0.1921 | Yes |
| 7 | Ctsb | 46 | 1.778 | 0.2222 | Yes |
| 8 | Ctsk | 71 | 1.668 | 0.2440 | Yes |
| 9 | Dpp4 | 121 | 1.532 | 0.2550 | Yes |
| 10 | Plek | 135 | 1.509 | 0.2776 | Yes |
| 11 | Lrp1 | 183 | 1.402 | 0.2869 | Yes |
| 12 | Fgg | 221 | 1.352 | 0.2987 | Yes |
| 13 | Thbd | 306 | 1.215 | 0.2924 | Yes |
| 14 | Sh2b2 | 308 | 1.214 | 0.3137 | Yes |
| 15 | Mmp11 | 313 | 1.209 | 0.3340 | Yes |
| 16 | Vwf | 343 | 1.170 | 0.3452 | Yes |
| 17 | Htra1 | 363 | 1.150 | 0.3594 | Yes |
| 18 | Sparc | 460 | 1.038 | 0.3459 | Yes |
| 19 | Mmp9 | 499 | 0.993 | 0.3509 | Yes |
| 20 | Itgb3 | 516 | 0.975 | 0.3630 | Yes |
| 21 | Serping1 | 519 | 0.973 | 0.3797 | Yes |
| 22 | Fn1 | 520 | 0.973 | 0.3971 | Yes |
| 23 | Trf | 541 | 0.957 | 0.4075 | Yes |
| 24 | Fbn1 | 568 | 0.935 | 0.4156 | Yes |
| 25 | Bmp1 | 619 | 0.873 | 0.4145 | Yes |
| 26 | Pecam1 | 658 | 0.845 | 0.4169 | Yes |
| 27 | Ctsh | 890 | 0.637 | 0.3511 | No |
| 28 | Iscu | 905 | 0.626 | 0.3576 | No |
| 29 | C3 | 948 | 0.602 | 0.3543 | No |
| 30 | Timp3 | 959 | 0.596 | 0.3616 | No |
| 31 | Cfh | 1030 | 0.548 | 0.3480 | No |
| 32 | Plau | 1059 | 0.534 | 0.3482 | No |
| 33 | Maff | 1259 | -0.533 | 0.2912 | No |
| 34 | Anxa1 | 1347 | -0.552 | 0.2720 | No |
| 35 | Rac1 | 1404 | -0.564 | 0.2634 | No |
| 36 | Ctse | 1586 | -0.612 | 0.2139 | No |
| 37 | Plat | 2156 | -0.832 | 0.0386 | No |
| 38 | Hnf4a | 2271 | -0.910 | 0.0168 | No |
| 39 | Gda | 2280 | -0.913 | 0.0304 | No |
| 40 | F3 | 2372 | -0.985 | 0.0176 | No |
| 41 | Mmp15 | 2405 | -1.010 | 0.0250 | No |
| 42 | Prss23 | 2623 | -1.238 | -0.0254 | No |
| 43 | Hpn | 2653 | -1.291 | -0.0120 | No |
| 44 | Cfb | 2772 | -1.529 | -0.0241 | No |
| 45 | Capn5 | 2830 | -1.667 | -0.0134 | No |
| 46 | Clu | 2876 | -1.874 | 0.0051 | No |
| 47 | Cfi | 2977 | -2.744 | 0.0207 | No |
Table: GSEA details [plain text format]

  

Fig 2: HALLMARK\_COAGULATION: Random ES distribution      
 Gene set null distribution of ES for **HALLMARK\_COAGULATION**

  
