# Supplementary material for: TMPRSS11B promotes an acidified microenvironment and immune suppression in squamous lung cancer
Source: EMBO Rep. 2025 Nov 10;26(24):6346–79. doi: 10.1038/s44319-025-00631-1 (PMC12714794; doi:10.1038/s44319-025-00631-1)
Supplement: Supplementary file 19 — Appendix Figure S1 Source Data [file 44319_2025_631_MOESM19_ESM.zip › Appendix Figure S1/S1C/GSEA Broad Institute_low pH vs rest of the regions (high pH)_Mh/HALLMARK_COMPLEMENT.html]

Details for gene set HALLMARK\_COMPLEMENT[GSEA]

|  || Dataset | Lactate high vs low\_Ranked |
| Phenotype | NoPhenotypeAvailable |
| Upregulated in class | na\_pos |
| GeneSet | HALLMARK\_COMPLEMENT |
| Enrichment Score (ES) | 0.44041333 |
| Normalized Enrichment Score (NES) | 2.7563283 |
| Nominal p-value | 0.0 |
| FDR q-value | 0.0 |
| FWER p-Value | 0.0 |
Table: GSEA Results Summary

  

Fig 1: Enrichment plot: HALLMARK\_COMPLEMENT      
 Profile of the Running ES Score & Positions of GeneSet Members on the Rank Ordered List

  

| SYMBOL | RANK IN GENE LIST | RANK METRIC SCORE | RUNNING ES | CORE ENRICHMENT || 1 | Ctsl | 8 | 2.153 | 0.0251 | Yes |
| 2 | F7 | 10 | 2.132 | 0.0523 | Yes |
| 3 | Ctss | 14 | 2.088 | 0.0783 | Yes |
| 4 | C1qc | 20 | 1.988 | 0.1023 | Yes |
| 5 | Lgmn | 28 | 1.878 | 0.1242 | Yes |
| 6 | C1qa | 29 | 1.862 | 0.1483 | Yes |
| 7 | Spock2 | 31 | 1.859 | 0.1720 | Yes |
| 8 | F10 | 33 | 1.837 | 0.1954 | Yes |
| 9 | Apoc1 | 40 | 1.811 | 0.2168 | Yes |
| 10 | Ctsb | 46 | 1.778 | 0.2381 | Yes |
| 11 | Cd36 | 52 | 1.748 | 0.2590 | Yes |
| 12 | Lipa | 54 | 1.721 | 0.2809 | Yes |
| 13 | Gngt2 | 58 | 1.698 | 0.3018 | Yes |
| 14 | Pik3r5 | 63 | 1.685 | 0.3222 | Yes |
| 15 | Pla2g7 | 65 | 1.683 | 0.3437 | Yes |
| 16 | Was | 89 | 1.612 | 0.3567 | Yes |
| 17 | Fcer1g | 95 | 1.597 | 0.3757 | Yes |
| 18 | Dpp4 | 121 | 1.532 | 0.3871 | Yes |
| 19 | Plek | 135 | 1.509 | 0.4022 | Yes |
| 20 | Ctsd | 136 | 1.507 | 0.4217 | Yes |
| 21 | Lrp1 | 183 | 1.402 | 0.4244 | Yes |
| 22 | Mmp12 | 218 | 1.354 | 0.4304 | Yes |
| 23 | Col4a2 | 331 | 1.182 | 0.4081 | Yes |
| 24 | Timp2 | 334 | 1.180 | 0.4227 | Yes |
| 25 | Lgals3 | 344 | 1.170 | 0.4347 | Yes |
| 26 | Dock10 | 440 | 1.057 | 0.4165 | Yes |
| 27 | Lcp2 | 449 | 1.047 | 0.4273 | Yes |
| 28 | Serping1 | 519 | 0.973 | 0.4167 | Yes |
| 29 | Fn1 | 520 | 0.973 | 0.4293 | Yes |
| 30 | Ctsc | 525 | 0.966 | 0.4404 | Yes |
| 31 | Msrb1 | 629 | 0.868 | 0.4170 | No |
| 32 | Cblb | 702 | 0.809 | 0.4033 | No |
| 33 | Anxa5 | 743 | 0.769 | 0.3998 | No |
| 34 | Notch4 | 766 | 0.746 | 0.4020 | No |
| 35 | Cebpb | 773 | 0.736 | 0.4095 | No |
| 36 | Grb2 | 816 | 0.695 | 0.4044 | No |
| 37 | Prcp | 842 | 0.676 | 0.4047 | No |
| 38 | Pim1 | 859 | 0.656 | 0.4078 | No |
| 39 | Ctsh | 890 | 0.637 | 0.4060 | No |
| 40 | Rhog | 932 | 0.609 | 0.4001 | No |
| 41 | C3 | 948 | 0.602 | 0.4028 | No |
| 42 | Prkcd | 984 | 0.574 | 0.3985 | No |
| 43 | Sh2b3 | 991 | 0.568 | 0.4038 | No |
| 44 | Cfh | 1030 | 0.548 | 0.3981 | No |
| 45 | Plaur | 1098 | 0.504 | 0.3821 | No |
| 46 | Cpm | 1180 | -0.515 | 0.3615 | No |
| 47 | Calm3 | 1189 | -0.517 | 0.3655 | No |
| 48 | Maff | 1259 | -0.533 | 0.3492 | No |
| 49 | Rnf4 | 1284 | -0.538 | 0.3481 | No |
| 50 | Gpd2 | 1481 | -0.582 | 0.2898 | No |
| 51 | Casp4 | 1495 | -0.585 | 0.2929 | No |
| 52 | Brpf3 | 1526 | -0.593 | 0.2905 | No |
| 53 | Casp7 | 1587 | -0.613 | 0.2783 | No |
| 54 | Usp16 | 1669 | -0.639 | 0.2593 | No |
| 55 | Gnb4 | 1779 | -0.681 | 0.2315 | No |
| 56 | Plat | 2156 | -0.832 | 0.1159 | No |
| 57 | Hnf4a | 2271 | -0.910 | 0.0893 | No |
| 58 | F3 | 2372 | -0.985 | 0.0685 | No |
| 59 | Mmp15 | 2405 | -1.010 | 0.0708 | No |
| 60 | Irf7 | 2638 | -1.265 | 0.0092 | No |
| 61 | Cfb | 2772 | -1.529 | -0.0158 | No |
| 62 | Cdh13 | 2854 | -1.759 | -0.0203 | No |
| 63 | Clu | 2876 | -1.874 | -0.0031 | No |
| 64 | Ltf | 3035 | -4.454 | 0.0013 | No |
Table: GSEA details [plain text format]

  

Fig 2: HALLMARK\_COMPLEMENT: Random ES distribution      
 Gene set null distribution of ES for **HALLMARK\_COMPLEMENT**

  
