# Supplementary material for: TMPRSS11B promotes an acidified microenvironment and immune suppression in squamous lung cancer
Source: EMBO Rep. 2025 Nov 10;26(24):6346–79. doi: 10.1038/s44319-025-00631-1 (PMC12714794; doi:10.1038/s44319-025-00631-1)
Supplement: Supplementary file 19 — Appendix Figure S1 Source Data [file 44319_2025_631_MOESM19_ESM.zip › Appendix Figure S1/S1C/GSEA Broad Institute_low pH vs rest of the regions (high pH)_Mh/HALLMARK_DNA_REPAIR.html]

Details for gene set HALLMARK\_DNA\_REPAIR[GSEA]

|  || Dataset | Lactate high vs low\_Ranked |
| Phenotype | NoPhenotypeAvailable |
| Upregulated in class | na\_pos |
| GeneSet | HALLMARK\_DNA\_REPAIR |
| Enrichment Score (ES) | 0.22862817 |
| Normalized Enrichment Score (NES) | 1.0233078 |
| Nominal p-value | 0.4207317 |
| FDR q-value | 0.58683985 |
| FWER p-Value | 0.994 |
Table: GSEA Results Summary

  

Fig 1: Enrichment plot: HALLMARK\_DNA\_REPAIR      
 Profile of the Running ES Score & Positions of GeneSet Members on the Rank Ordered List

  

| SYMBOL | RANK IN GENE LIST | RANK METRIC SCORE | RUNNING ES | CORE ENRICHMENT || 1 | Hcls1 | 156 | 1.451 | 0.0405 | Yes |
| 2 | Npr2 | 320 | 1.203 | 0.0630 | Yes |
| 3 | Sdcbp | 945 | 0.602 | -0.1055 | Yes |
| 4 | Arl6ip1 | 961 | 0.595 | -0.0726 | Yes |
| 5 | Aaas | 1052 | 0.536 | -0.0683 | Yes |
| 6 | Stx3 | 1053 | 0.536 | -0.0343 | Yes |
| 7 | Ddb2 | 1209 | -0.523 | -0.0524 | Yes |
| 8 | Upf3b | 1309 | -0.542 | -0.0507 | Yes |
| 9 | Dguok | 1359 | -0.554 | -0.0317 | Yes |
| 10 | Surf1 | 1396 | -0.562 | -0.0080 | Yes |
| 11 | Polr2h | 1511 | -0.589 | -0.0083 | Yes |
| 12 | Nelfe | 1569 | -0.607 | 0.0114 | Yes |
| 13 | Ak3 | 1592 | -0.614 | 0.0431 | Yes |
| 14 | Gtf2f1 | 1622 | -0.623 | 0.0731 | Yes |
| 15 | Rpa2 | 1783 | -0.681 | 0.0634 | Yes |
| 16 | Xpc | 1812 | -0.692 | 0.0981 | Yes |
| 17 | Zwint | 1875 | -0.712 | 0.1228 | Yes |
| 18 | Tyms | 1908 | -0.724 | 0.1582 | Yes |
| 19 | Gtf2h3 | 1910 | -0.725 | 0.2040 | Yes |
| 20 | Gtf2a2 | 2095 | -0.804 | 0.1941 | Yes |
| 21 | Pom121 | 2253 | -0.895 | 0.1990 | Yes |
| 22 | Vps37b | 2349 | -0.962 | 0.2286 | Yes |
Table: GSEA details [plain text format]

  

Fig 2: HALLMARK\_DNA\_REPAIR: Random ES distribution      
 Gene set null distribution of ES for **HALLMARK\_DNA\_REPAIR**

  
