# Supplementary material for: TMPRSS11B promotes an acidified microenvironment and immune suppression in squamous lung cancer
Source: EMBO Rep. 2025 Nov 10;26(24):6346–79. doi: 10.1038/s44319-025-00631-1 (PMC12714794; doi:10.1038/s44319-025-00631-1)
Supplement: Supplementary file 19 — Appendix Figure S1 Source Data [file 44319_2025_631_MOESM19_ESM.zip › Appendix Figure S1/S1C/GSEA Broad Institute_low pH vs rest of the regions (high pH)_Mh/HALLMARK_E2F_TARGETS.html]

Details for gene set HALLMARK\_E2F\_TARGETS[GSEA]

|  || Dataset | Lactate high vs low\_Ranked |
| Phenotype | NoPhenotypeAvailable |
| Upregulated in class | na\_neg |
| GeneSet | HALLMARK\_E2F\_TARGETS |
| Enrichment Score (ES) | -0.45449716 |
| Normalized Enrichment Score (NES) | -1.5563226 |
| Nominal p-value | 0.041800644 |
| FDR q-value | 0.20873465 |
| FWER p-Value | 0.71 |
Table: GSEA Results Summary

  

Fig 1: Enrichment plot: HALLMARK\_E2F\_TARGETS      
 Profile of the Running ES Score & Positions of GeneSet Members on the Rank Ordered List

  

| SYMBOL | RANK IN GENE LIST | RANK METRIC SCORE | RUNNING ES | CORE ENRICHMENT || 1 | Cdkn1a | 749 | 0.765 | -0.1898 | No |
| 2 | Ctcf | 1124 | -0.504 | -0.2753 | No |
| 3 | Paics | 1667 | -0.638 | -0.4063 | Yes |
| 4 | Rpa2 | 1783 | -0.681 | -0.3928 | Yes |
| 5 | Nop56 | 1822 | -0.696 | -0.3527 | Yes |
| 6 | Nbn | 1876 | -0.712 | -0.3164 | Yes |
| 7 | Nap1l1 | 1905 | -0.723 | -0.2709 | Yes |
| 8 | Pop7 | 2037 | -0.777 | -0.2555 | Yes |
| 9 | Cdkn2a | 2223 | -0.874 | -0.2506 | Yes |
| 10 | Mlh1 | 2260 | -0.899 | -0.1946 | Yes |
| 11 | Pold2 | 2301 | -0.928 | -0.1376 | Yes |
| 12 | Cdc25a | 2319 | -0.935 | -0.0725 | Yes |
| 13 | Psip1 | 2410 | -1.012 | -0.0257 | Yes |
| 14 | Rbbp7 | 2609 | -1.222 | 0.0012 | Yes |
| 15 | Shmt1 | 2871 | -1.860 | 0.0555 | Yes |
Table: GSEA details [plain text format]

  

Fig 2: HALLMARK\_E2F\_TARGETS: Random ES distribution      
 Gene set null distribution of ES for **HALLMARK\_E2F\_TARGETS**

  
