# Supplementary material for: TMPRSS11B promotes an acidified microenvironment and immune suppression in squamous lung cancer
Source: EMBO Rep. 2025 Nov 10;26(24):6346–79. doi: 10.1038/s44319-025-00631-1 (PMC12714794; doi:10.1038/s44319-025-00631-1)
Supplement: Supplementary file 19 — Appendix Figure S1 Source Data [file 44319_2025_631_MOESM19_ESM.zip › Appendix Figure S1/S1C/GSEA Broad Institute_low pH vs rest of the regions (high pH)_Mh/HALLMARK_EPITHELIAL_MESENCHYMAL_TRANSITION.html]

Details for gene set HALLMARK\_EPITHELIAL\_MESENCHYMAL\_TRANSITION[GSEA]

|  || Dataset | Lactate high vs low\_Ranked |
| Phenotype | NoPhenotypeAvailable |
| Upregulated in class | na\_pos |
| GeneSet | HALLMARK\_EPITHELIAL\_MESENCHYMAL\_TRANSITION |
| Enrichment Score (ES) | 0.45754868 |
| Normalized Enrichment Score (NES) | 2.982232 |
| Nominal p-value | 0.0 |
| FDR q-value | 0.0 |
| FWER p-Value | 0.0 |
Table: GSEA Results Summary

  

Fig 1: Enrichment plot: HALLMARK\_EPITHELIAL\_MESENCHYMAL\_TRANSITION      
 Profile of the Running ES Score & Positions of GeneSet Members on the Rank Ordered List

  

| SYMBOL | RANK IN GENE LIST | RANK METRIC SCORE | RUNNING ES | CORE ENRICHMENT || 1 | Mmp3 | 30 | 1.861 | 0.0129 | Yes |
| 2 | Lgals1 | 45 | 1.781 | 0.0303 | Yes |
| 3 | Itga5 | 85 | 1.616 | 0.0372 | Yes |
| 4 | Vim | 128 | 1.521 | 0.0418 | Yes |
| 5 | Col6a3 | 175 | 1.408 | 0.0438 | Yes |
| 6 | Lrp1 | 183 | 1.402 | 0.0588 | Yes |
| 7 | Pcolce2 | 184 | 1.402 | 0.0762 | Yes |
| 8 | Cxcl15 | 190 | 1.391 | 0.0917 | Yes |
| 9 | Emp3 | 191 | 1.389 | 0.1090 | Yes |
| 10 | Glipr1 | 208 | 1.364 | 0.1205 | Yes |
| 11 | Anpep | 212 | 1.361 | 0.1363 | Yes |
| 12 | Spp1 | 232 | 1.327 | 0.1464 | Yes |
| 13 | Col6a2 | 237 | 1.316 | 0.1613 | Yes |
| 14 | Pmp22 | 266 | 1.263 | 0.1675 | Yes |
| 15 | Fbln5 | 275 | 1.258 | 0.1804 | Yes |
| 16 | Col4a1 | 286 | 1.235 | 0.1924 | Yes |
| 17 | Gja1 | 314 | 1.208 | 0.1982 | Yes |
| 18 | Col4a2 | 331 | 1.182 | 0.2075 | Yes |
| 19 | Htra1 | 363 | 1.150 | 0.2113 | Yes |
| 20 | Fstl3 | 379 | 1.129 | 0.2202 | Yes |
| 21 | Col5a2 | 387 | 1.116 | 0.2317 | Yes |
| 22 | Postn | 400 | 1.098 | 0.2412 | Yes |
| 23 | Fas | 457 | 1.039 | 0.2352 | Yes |
| 24 | Sparc | 460 | 1.038 | 0.2474 | Yes |
| 25 | Cxcl12 | 488 | 1.003 | 0.2507 | Yes |
| 26 | Pdgfrb | 494 | 0.996 | 0.2614 | Yes |
| 27 | Plod1 | 497 | 0.995 | 0.2731 | Yes |
| 28 | Col3a1 | 501 | 0.992 | 0.2844 | Yes |
| 29 | Col5a1 | 508 | 0.983 | 0.2945 | Yes |
| 30 | Itgb3 | 516 | 0.975 | 0.3043 | Yes |
| 31 | Fn1 | 520 | 0.973 | 0.3153 | Yes |
| 32 | Plod3 | 539 | 0.958 | 0.3211 | Yes |
| 33 | Serpinh1 | 546 | 0.953 | 0.3309 | Yes |
| 34 | Dpysl3 | 551 | 0.949 | 0.3413 | Yes |
| 35 | Lox | 559 | 0.942 | 0.3506 | Yes |
| 36 | Fbn1 | 568 | 0.935 | 0.3595 | Yes |
| 37 | Fstl1 | 573 | 0.928 | 0.3697 | Yes |
| 38 | Mylk | 588 | 0.905 | 0.3762 | Yes |
| 39 | Bmp1 | 619 | 0.873 | 0.3769 | Yes |
| 40 | Col1a1 | 643 | 0.856 | 0.3797 | Yes |
| 41 | Vegfa | 650 | 0.852 | 0.3882 | Yes |
| 42 | Tgfbi | 651 | 0.851 | 0.3988 | Yes |
| 43 | Crlf1 | 653 | 0.849 | 0.4090 | Yes |
| 44 | Tgfb1 | 664 | 0.839 | 0.4160 | Yes |
| 45 | Col1a2 | 704 | 0.807 | 0.4128 | Yes |
| 46 | Flna | 707 | 0.805 | 0.4221 | Yes |
| 47 | Gpc1 | 721 | 0.794 | 0.4276 | Yes |
| 48 | Efemp2 | 767 | 0.743 | 0.4216 | Yes |
| 49 | Prrx1 | 778 | 0.727 | 0.4272 | Yes |
| 50 | Wipf1 | 781 | 0.724 | 0.4356 | Yes |
| 51 | Rgs4 | 791 | 0.714 | 0.4414 | Yes |
| 52 | Sfrp1 | 833 | 0.680 | 0.4359 | Yes |
| 53 | Lama3 | 849 | 0.672 | 0.4392 | Yes |
| 54 | Fermt2 | 853 | 0.668 | 0.4465 | Yes |
| 55 | Dab2 | 867 | 0.651 | 0.4502 | Yes |
| 56 | Bgn | 870 | 0.650 | 0.4575 | Yes |
| 57 | Mgp | 898 | 0.630 | 0.4562 | No |
| 58 | Timp3 | 959 | 0.596 | 0.4434 | No |
| 59 | Serpine2 | 967 | 0.590 | 0.4483 | No |
| 60 | Fgf2 | 1014 | 0.555 | 0.4397 | No |
| 61 | Slc6a8 | 1041 | 0.543 | 0.4376 | No |
| 62 | Cald1 | 1066 | 0.529 | 0.4361 | No |
| 63 | P3h1 | 1068 | 0.528 | 0.4423 | No |
| 64 | Cd44 | 1072 | 0.523 | 0.4478 | No |
| 65 | Loxl1 | 1091 | 0.506 | 0.4480 | No |
| 66 | Plaur | 1098 | 0.504 | 0.4522 | No |
| 67 | Sdc1 | 1228 | -0.527 | 0.4151 | No |
| 68 | Pmepa1 | 1426 | -0.570 | 0.3557 | No |
| 69 | Nt5e | 1516 | -0.591 | 0.3330 | No |
| 70 | Dst | 2035 | -0.777 | 0.1676 | No |
| 71 | Tpm2 | 2092 | -0.804 | 0.1587 | No |
| 72 | Basp1 | 2191 | -0.855 | 0.1362 | No |
| 73 | Tnfrsf11b | 2433 | -1.037 | 0.0677 | No |
| 74 | Dcn | 2701 | -1.362 | -0.0056 | No |
| 75 | Ecm1 | 2738 | -1.455 | 0.0003 | No |
| 76 | Igfbp3 | 2753 | -1.492 | 0.0140 | No |
| 77 | Areg | 2775 | -1.538 | 0.0260 | No |
| 78 | Qsox1 | 2881 | -1.902 | 0.0141 | No |
| 79 | Cxcl5 | 2999 | -3.136 | 0.0135 | No |
Table: GSEA details [plain text format]

  

Fig 2: HALLMARK\_EPITHELIAL\_MESENCHYMAL\_TRANSITION: Random ES distribution      
 Gene set null distribution of ES for **HALLMARK\_EPITHELIAL\_MESENCHYMAL\_TRANSITION**

  
