# Supplementary material for: TMPRSS11B promotes an acidified microenvironment and immune suppression in squamous lung cancer
Source: EMBO Rep. 2025 Nov 10;26(24):6346–79. doi: 10.1038/s44319-025-00631-1 (PMC12714794; doi:10.1038/s44319-025-00631-1)
Supplement: Supplementary file 19 — Appendix Figure S1 Source Data [file 44319_2025_631_MOESM19_ESM.zip › Appendix Figure S1/S1C/GSEA Broad Institute_low pH vs rest of the regions (high pH)_Mh/HALLMARK_ESTROGEN_RESPONSE_EARLY.html]

Details for gene set HALLMARK\_ESTROGEN\_RESPONSE\_EARLY[GSEA]

|  || Dataset | Lactate high vs low\_Ranked |
| Phenotype | NoPhenotypeAvailable |
| Upregulated in class | na\_neg |
| GeneSet | HALLMARK\_ESTROGEN\_RESPONSE\_EARLY |
| Enrichment Score (ES) | -0.3706426 |
| Normalized Enrichment Score (NES) | -1.9572785 |
| Nominal p-value | 0.0 |
| FDR q-value | 0.010139499 |
| FWER p-Value | 0.047 |
Table: GSEA Results Summary

  

Fig 1: Enrichment plot: HALLMARK\_ESTROGEN\_RESPONSE\_EARLY      
 Profile of the Running ES Score & Positions of GeneSet Members on the Rank Ordered List

  

| SYMBOL | RANK IN GENE LIST | RANK METRIC SCORE | RUNNING ES | CORE ENRICHMENT || 1 | Gla | 181 | 1.403 | -0.0454 | No |
| 2 | Gja1 | 314 | 1.208 | -0.0765 | No |
| 3 | Cbfa2t3 | 394 | 1.107 | -0.0908 | No |
| 4 | Podxl | 420 | 1.081 | -0.0871 | No |
| 5 | Cxcl12 | 488 | 1.003 | -0.0985 | No |
| 6 | Ccn5 | 542 | 0.955 | -0.1057 | No |
| 7 | Rab31 | 543 | 0.955 | -0.0950 | No |
| 8 | Dlc1 | 576 | 0.923 | -0.0955 | No |
| 9 | Clic3 | 594 | 0.901 | -0.0911 | No |
| 10 | Wfs1 | 639 | 0.858 | -0.0964 | No |
| 11 | Dhrs3 | 703 | 0.807 | -0.1086 | No |
| 12 | Olfm1 | 716 | 0.798 | -0.1037 | No |
| 13 | Scarb1 | 738 | 0.772 | -0.1022 | No |
| 14 | Slc7a2 | 795 | 0.711 | -0.1131 | No |
| 15 | Sh3bp5 | 881 | 0.642 | -0.1346 | No |
| 16 | Fasn | 884 | 0.641 | -0.1281 | No |
| 17 | Syngr1 | 911 | 0.622 | -0.1299 | No |
| 18 | Fkbp5 | 935 | 0.606 | -0.1309 | No |
| 19 | Cd44 | 1072 | 0.523 | -0.1710 | No |
| 20 | Slc1a1 | 1093 | 0.505 | -0.1721 | No |
| 21 | Akap1 | 1129 | -0.505 | -0.1783 | No |
| 22 | Med24 | 1187 | -0.516 | -0.1917 | No |
| 23 | Krt8 | 1193 | -0.519 | -0.1876 | No |
| 24 | Bag1 | 1264 | -0.534 | -0.2053 | No |
| 25 | Tsku | 1373 | -0.558 | -0.2355 | No |
| 26 | Bcl2 | 1390 | -0.561 | -0.2346 | No |
| 27 | Pmaip1 | 1451 | -0.575 | -0.2485 | No |
| 28 | Isg20l2 | 1544 | -0.599 | -0.2728 | No |
| 29 | Retreg1 | 1612 | -0.619 | -0.2885 | No |
| 30 | Ovol2 | 1648 | -0.632 | -0.2933 | No |
| 31 | Fhl2 | 1756 | -0.673 | -0.3219 | No |
| 32 | Mast4 | 1762 | -0.675 | -0.3160 | No |
| 33 | Itpk1 | 1784 | -0.682 | -0.3155 | No |
| 34 | Rhod | 1804 | -0.688 | -0.3142 | No |
| 35 | Siah2 | 1909 | -0.724 | -0.3412 | No |
| 36 | Rara | 1927 | -0.731 | -0.3388 | No |
| 37 | Tob1 | 1942 | -0.737 | -0.3352 | No |
| 38 | Fdft1 | 1952 | -0.741 | -0.3300 | No |
| 39 | Xbp1 | 1976 | -0.750 | -0.3293 | No |
| 40 | Hr | 1982 | -0.751 | -0.3226 | No |
| 41 | Mlph | 1995 | -0.757 | -0.3182 | No |
| 42 | Nrip1 | 2104 | -0.807 | -0.3456 | No |
| 43 | Fkbp4 | 2117 | -0.814 | -0.3406 | No |
| 44 | Frk | 2131 | -0.819 | -0.3358 | No |
| 45 | Ppif | 2171 | -0.845 | -0.3395 | No |
| 46 | Slc39a6 | 2185 | -0.852 | -0.3343 | No |
| 47 | Flnb | 2221 | -0.871 | -0.3364 | No |
| 48 | Tbc1d30 | 2231 | -0.882 | -0.3295 | No |
| 49 | Hes1 | 2255 | -0.896 | -0.3273 | No |
| 50 | Tiam1 | 2352 | -0.967 | -0.3489 | No |
| 51 | Slc26a2 | 2411 | -1.012 | -0.3571 | No |
| 52 | Ptges | 2452 | -1.053 | -0.3588 | Yes |
| 53 | Slc7a5 | 2465 | -1.061 | -0.3510 | Yes |
| 54 | Celsr1 | 2472 | -1.065 | -0.3411 | Yes |
| 55 | Krt19 | 2476 | -1.067 | -0.3301 | Yes |
| 56 | Tjp3 | 2478 | -1.070 | -0.3185 | Yes |
| 57 | Fos | 2491 | -1.083 | -0.3104 | Yes |
| 58 | Hspb8 | 2503 | -1.097 | -0.3018 | Yes |
| 59 | Elf3 | 2533 | -1.130 | -0.2990 | Yes |
| 60 | Esrp2 | 2535 | -1.132 | -0.2866 | Yes |
| 61 | Nadsyn1 | 2576 | -1.177 | -0.2869 | Yes |
| 62 | Tpbg | 2601 | -1.216 | -0.2814 | Yes |
| 63 | Slc37a1 | 2618 | -1.230 | -0.2730 | Yes |
| 64 | Prss23 | 2623 | -1.238 | -0.2605 | Yes |
| 65 | Cldn7 | 2642 | -1.269 | -0.2524 | Yes |
| 66 | Lad1 | 2675 | -1.325 | -0.2483 | Yes |
| 67 | Areg | 2775 | -1.538 | -0.2645 | Yes |
| 68 | Muc1 | 2794 | -1.579 | -0.2529 | Yes |
| 69 | Ttc39a | 2798 | -1.588 | -0.2362 | Yes |
| 70 | Aqp3 | 2802 | -1.594 | -0.2193 | Yes |
| 71 | Igf1r | 2859 | -1.811 | -0.2179 | Yes |
| 72 | Foxc1 | 2895 | -1.983 | -0.2075 | Yes |
| 73 | Lrig1 | 2913 | -2.103 | -0.1897 | Yes |
| 74 | Reep1 | 2914 | -2.104 | -0.1661 | Yes |
| 75 | Stc2 | 2919 | -2.148 | -0.1434 | Yes |
| 76 | Krt15 | 2935 | -2.256 | -0.1232 | Yes |
| 77 | Celsr2 | 2961 | -2.404 | -0.1047 | Yes |
| 78 | Car12 | 3022 | -3.775 | -0.0827 | Yes |
| 79 | Krt13 | 3025 | -3.823 | -0.0405 | Yes |
| 80 | Klk10 | 3029 | -4.008 | 0.0034 | Yes |
Table: GSEA details [plain text format]

  

Fig 2: HALLMARK\_ESTROGEN\_RESPONSE\_EARLY: Random ES distribution      
 Gene set null distribution of ES for **HALLMARK\_ESTROGEN\_RESPONSE\_EARLY**

  
