# Supplementary material for: TMPRSS11B promotes an acidified microenvironment and immune suppression in squamous lung cancer
Source: EMBO Rep. 2025 Nov 10;26(24):6346–79. doi: 10.1038/s44319-025-00631-1 (PMC12714794; doi:10.1038/s44319-025-00631-1)
Supplement: Supplementary file 19 — Appendix Figure S1 Source Data [file 44319_2025_631_MOESM19_ESM.zip › Appendix Figure S1/S1C/GSEA Broad Institute_low pH vs rest of the regions (high pH)_Mh/HALLMARK_ESTROGEN_RESPONSE_LATE.html]

Details for gene set HALLMARK\_ESTROGEN\_RESPONSE\_LATE[GSEA]

|  || Dataset | Lactate high vs low\_Ranked |
| Phenotype | NoPhenotypeAvailable |
| Upregulated in class | na\_neg |
| GeneSet | HALLMARK\_ESTROGEN\_RESPONSE\_LATE |
| Enrichment Score (ES) | -0.43035284 |
| Normalized Enrichment Score (NES) | -2.1199775 |
| Nominal p-value | 0.0 |
| FDR q-value | 0.002977357 |
| FWER p-Value | 0.009 |
Table: GSEA Results Summary

  

Fig 1: Enrichment plot: HALLMARK\_ESTROGEN\_RESPONSE\_LATE      
 Profile of the Running ES Score & Positions of GeneSet Members on the Rank Ordered List

  

| SYMBOL | RANK IN GENE LIST | RANK METRIC SCORE | RUNNING ES | CORE ENRICHMENT || 1 | Cav1 | 107 | 1.564 | -0.0166 | No |
| 2 | Gla | 181 | 1.403 | -0.0238 | No |
| 3 | Ckb | 422 | 1.078 | -0.0911 | No |
| 4 | Cxcl12 | 488 | 1.003 | -0.1006 | No |
| 5 | Ccn5 | 542 | 0.955 | -0.1065 | No |
| 6 | Rab31 | 543 | 0.955 | -0.0947 | No |
| 7 | Clic3 | 594 | 0.901 | -0.1004 | No |
| 8 | Wfs1 | 639 | 0.858 | -0.1046 | No |
| 9 | Olfm1 | 716 | 0.798 | -0.1202 | No |
| 10 | Scarb1 | 738 | 0.772 | -0.1177 | No |
| 11 | Emp2 | 915 | 0.618 | -0.1692 | No |
| 12 | Fkbp5 | 935 | 0.606 | -0.1681 | No |
| 13 | Jak1 | 1040 | 0.543 | -0.1964 | No |
| 14 | Cd44 | 1072 | 0.523 | -0.2003 | No |
| 15 | Ptpn6 | 1096 | 0.505 | -0.2018 | No |
| 16 | Mettl3 | 1241 | -0.530 | -0.2437 | No |
| 17 | Bag1 | 1264 | -0.534 | -0.2444 | No |
| 18 | Llgl2 | 1344 | -0.551 | -0.2642 | No |
| 19 | Bcl2 | 1390 | -0.561 | -0.2724 | No |
| 20 | Fabp5 | 1603 | -0.617 | -0.3360 | No |
| 21 | Ovol2 | 1648 | -0.632 | -0.3430 | No |
| 22 | Itpk1 | 1784 | -0.682 | -0.3799 | No |
| 23 | Lsr | 1792 | -0.685 | -0.3738 | No |
| 24 | Siah2 | 1909 | -0.724 | -0.4038 | No |
| 25 | Tob1 | 1942 | -0.737 | -0.4054 | No |
| 26 | Fdft1 | 1952 | -0.741 | -0.3993 | No |
| 27 | Dnajc1 | 1957 | -0.742 | -0.3915 | No |
| 28 | Xbp1 | 1976 | -0.750 | -0.3882 | No |
| 29 | Hr | 1982 | -0.751 | -0.3806 | No |
| 30 | Dnajc12 | 2024 | -0.771 | -0.3849 | No |
| 31 | Nrip1 | 2104 | -0.807 | -0.4014 | No |
| 32 | Fkbp4 | 2117 | -0.814 | -0.3954 | No |
| 33 | Frk | 2131 | -0.819 | -0.3896 | No |
| 34 | Ppif | 2171 | -0.845 | -0.3923 | No |
| 35 | Unc13b | 2200 | -0.859 | -0.3911 | No |
| 36 | Flnb | 2221 | -0.871 | -0.3870 | No |
| 37 | Gale | 2351 | -0.965 | -0.4184 | Yes |
| 38 | Tiam1 | 2352 | -0.967 | -0.4064 | Yes |
| 39 | Slc26a2 | 2411 | -1.012 | -0.4134 | Yes |
| 40 | Dcxr | 2434 | -1.037 | -0.4080 | Yes |
| 41 | Ptges | 2452 | -1.053 | -0.4006 | Yes |
| 42 | Slc7a5 | 2465 | -1.061 | -0.3916 | Yes |
| 43 | Krt19 | 2476 | -1.067 | -0.3817 | Yes |
| 44 | Tjp3 | 2478 | -1.070 | -0.3688 | Yes |
| 45 | Fos | 2491 | -1.083 | -0.3594 | Yes |
| 46 | Hspb8 | 2503 | -1.097 | -0.3495 | Yes |
| 47 | Trim29 | 2540 | -1.140 | -0.3475 | Yes |
| 48 | Tpbg | 2601 | -1.216 | -0.3526 | Yes |
| 49 | Prss23 | 2623 | -1.238 | -0.3444 | Yes |
| 50 | St14 | 2628 | -1.245 | -0.3303 | Yes |
| 51 | Cdh1 | 2655 | -1.294 | -0.3230 | Yes |
| 52 | Fgfr3 | 2662 | -1.308 | -0.3089 | Yes |
| 53 | Pkp3 | 2706 | -1.377 | -0.3063 | Yes |
| 54 | Tst | 2769 | -1.524 | -0.3083 | Yes |
| 55 | Areg | 2775 | -1.538 | -0.2909 | Yes |
| 56 | Isg20 | 2853 | -1.758 | -0.2950 | Yes |
| 57 | Foxc1 | 2895 | -1.983 | -0.2843 | Yes |
| 58 | Homer2 | 2918 | -2.140 | -0.2652 | Yes |
| 59 | Agr2 | 2938 | -2.260 | -0.2436 | Yes |
| 60 | Celsr2 | 2961 | -2.404 | -0.2213 | Yes |
| 61 | Car12 | 3022 | -3.775 | -0.1947 | Yes |
| 62 | Cpe | 3023 | -3.802 | -0.1477 | Yes |
| 63 | Krt13 | 3025 | -3.823 | -0.1007 | Yes |
| 64 | Klk10 | 3029 | -4.008 | -0.0521 | Yes |
| 65 | Ltf | 3035 | -4.454 | 0.0013 | Yes |
Table: GSEA details [plain text format]

  

Fig 2: HALLMARK\_ESTROGEN\_RESPONSE\_LATE: Random ES distribution      
 Gene set null distribution of ES for **HALLMARK\_ESTROGEN\_RESPONSE\_LATE**

  
