# Supplementary material for: TMPRSS11B promotes an acidified microenvironment and immune suppression in squamous lung cancer
Source: EMBO Rep. 2025 Nov 10;26(24):6346–79. doi: 10.1038/s44319-025-00631-1 (PMC12714794; doi:10.1038/s44319-025-00631-1)
Supplement: Supplementary file 19 — Appendix Figure S1 Source Data [file 44319_2025_631_MOESM19_ESM.zip › Appendix Figure S1/S1C/GSEA Broad Institute_low pH vs rest of the regions (high pH)_Mh/HALLMARK_FATTY_ACID_METABOLISM.html]

Details for gene set HALLMARK\_FATTY\_ACID\_METABOLISM[GSEA]

|  || Dataset | Lactate high vs low\_Ranked |
| Phenotype | NoPhenotypeAvailable |
| Upregulated in class | na\_neg |
| GeneSet | HALLMARK\_FATTY\_ACID\_METABOLISM |
| Enrichment Score (ES) | -0.20087957 |
| Normalized Enrichment Score (NES) | -0.91104126 |
| Nominal p-value | 0.60385144 |
| FDR q-value | 0.8329803 |
| FWER p-Value | 1.0 |
Table: GSEA Results Summary

  

Fig 1: Enrichment plot: HALLMARK\_FATTY\_ACID\_METABOLISM      
 Profile of the Running ES Score & Positions of GeneSet Members on the Rank Ordered List

  

| SYMBOL | RANK IN GENE LIST | RANK METRIC SCORE | RUNNING ES | CORE ENRICHMENT || 1 | Lgals1 | 45 | 1.781 | 0.0319 | No |
| 2 | Cd36 | 52 | 1.748 | 0.0759 | No |
| 3 | Fmo1 | 281 | 1.243 | 0.0325 | No |
| 4 | Mgll | 316 | 1.208 | 0.0530 | No |
| 5 | Blvra | 612 | 0.878 | -0.0223 | No |
| 6 | Acsl4 | 699 | 0.810 | -0.0297 | No |
| 7 | Maoa | 805 | 0.703 | -0.0462 | No |
| 8 | G0s2 | 878 | 0.645 | -0.0532 | No |
| 9 | Fasn | 884 | 0.641 | -0.0380 | No |
| 10 | S100a10 | 965 | 0.592 | -0.0491 | No |
| 11 | Erp29 | 1003 | 0.562 | -0.0467 | No |
| 12 | Gabarapl1 | 1061 | 0.533 | -0.0517 | No |
| 13 | Idh3g | 1205 | -0.521 | -0.0857 | No |
| 14 | Idh3b | 1206 | -0.521 | -0.0719 | No |
| 15 | Acss1 | 1367 | -0.557 | -0.1107 | No |
| 16 | Ostc | 1457 | -0.576 | -0.1252 | No |
| 17 | Gpd2 | 1481 | -0.582 | -0.1175 | No |
| 18 | Bckdhb | 1694 | -0.651 | -0.1711 | No |
| 19 | Retsat | 1742 | -0.669 | -0.1692 | No |
| 20 | Trp53inp2 | 1838 | -0.703 | -0.1824 | Yes |
| 21 | Aldh3a1 | 1866 | -0.710 | -0.1727 | Yes |
| 22 | Hadh | 1869 | -0.711 | -0.1546 | Yes |
| 23 | Eci1 | 1871 | -0.711 | -0.1362 | Yes |
| 24 | Nbn | 1876 | -0.712 | -0.1188 | Yes |
| 25 | Fh1 | 1923 | -0.730 | -0.1149 | Yes |
| 26 | Hsp90aa1 | 1965 | -0.745 | -0.1090 | Yes |
| 27 | Acadm | 1975 | -0.750 | -0.0923 | Yes |
| 28 | Sucla2 | 1992 | -0.757 | -0.0777 | Yes |
| 29 | Pts | 2017 | -0.768 | -0.0655 | Yes |
| 30 | Apex1 | 2125 | -0.816 | -0.0797 | Yes |
| 31 | Cbr3 | 2160 | -0.834 | -0.0690 | Yes |
| 32 | Aldh1a1 | 2205 | -0.861 | -0.0611 | Yes |
| 33 | Hmgcs1 | 2251 | -0.894 | -0.0525 | Yes |
| 34 | Grhpr | 2258 | -0.898 | -0.0309 | Yes |
| 35 | Dhcr24 | 2316 | -0.935 | -0.0253 | Yes |
| 36 | Acadl | 2329 | -0.941 | -0.0046 | Yes |
| 37 | D2hgdh | 2344 | -0.954 | 0.0159 | Yes |
| 38 | Gpd1 | 2364 | -0.978 | 0.0353 | Yes |
| 39 | Cryz | 2436 | -1.039 | 0.0390 | Yes |
| 40 | Reep6 | 2570 | -1.171 | 0.0254 | Yes |
| 41 | Hsd17b7 | 2654 | -1.293 | 0.0318 | Yes |
| 42 | Acsl1 | 2687 | -1.346 | 0.0565 | Yes |
| 43 | Adh7 | 2943 | -2.301 | 0.0320 | Yes |
Table: GSEA details [plain text format]

  

Fig 2: HALLMARK\_FATTY\_ACID\_METABOLISM: Random ES distribution      
 Gene set null distribution of ES for **HALLMARK\_FATTY\_ACID\_METABOLISM**

  
