# Supplementary material for: TMPRSS11B promotes an acidified microenvironment and immune suppression in squamous lung cancer
Source: EMBO Rep. 2025 Nov 10;26(24):6346–79. doi: 10.1038/s44319-025-00631-1 (PMC12714794; doi:10.1038/s44319-025-00631-1)
Supplement: Supplementary file 19 — Appendix Figure S1 Source Data [file 44319_2025_631_MOESM19_ESM.zip › Appendix Figure S1/S1C/GSEA Broad Institute_low pH vs rest of the regions (high pH)_Mh/HALLMARK_G2M_CHECKPOINT.html]

Details for gene set HALLMARK\_G2M\_CHECKPOINT[GSEA]

|  || Dataset | Lactate high vs low\_Ranked |
| Phenotype | NoPhenotypeAvailable |
| Upregulated in class | na\_neg |
| GeneSet | HALLMARK\_G2M\_CHECKPOINT |
| Enrichment Score (ES) | -0.36606595 |
| Normalized Enrichment Score (NES) | -1.4751811 |
| Nominal p-value | 0.083577715 |
| FDR q-value | 0.22010465 |
| FWER p-Value | 0.863 |
Table: GSEA Results Summary

  

Fig 1: Enrichment plot: HALLMARK\_G2M\_CHECKPOINT      
 Profile of the Running ES Score & Positions of GeneSet Members on the Rank Ordered List

  

| SYMBOL | RANK IN GENE LIST | RANK METRIC SCORE | RUNNING ES | CORE ENRICHMENT || 1 | Cenpa | 655 | 0.847 | -0.1809 | No |
| 2 | Tgfb1 | 664 | 0.839 | -0.1474 | No |
| 3 | Ccna2 | 1094 | 0.505 | -0.2680 | No |
| 4 | Ctcf | 1124 | -0.504 | -0.2559 | No |
| 5 | Ythdc1 | 1358 | -0.554 | -0.3094 | No |
| 6 | Kif5b | 1372 | -0.558 | -0.2897 | No |
| 7 | Mnat1 | 1421 | -0.568 | -0.2811 | No |
| 8 | Prmt5 | 1678 | -0.642 | -0.3384 | Yes |
| 9 | Bcl3 | 1709 | -0.657 | -0.3200 | Yes |
| 10 | Rpa2 | 1783 | -0.681 | -0.3149 | Yes |
| 11 | Smarcc1 | 1816 | -0.695 | -0.2956 | Yes |
| 12 | Katna1 | 1911 | -0.726 | -0.2955 | Yes |
| 13 | Tfdp1 | 2061 | -0.789 | -0.3110 | Yes |
| 14 | Sap30 | 2116 | -0.814 | -0.2938 | Yes |
| 15 | Lig3 | 2159 | -0.834 | -0.2718 | Yes |
| 16 | Tent4a | 2176 | -0.847 | -0.2406 | Yes |
| 17 | G3bp1 | 2307 | -0.930 | -0.2437 | Yes |
| 18 | Cdc25a | 2319 | -0.935 | -0.2070 | Yes |
| 19 | Fancc | 2323 | -0.938 | -0.1676 | Yes |
| 20 | Uck2 | 2365 | -0.978 | -0.1390 | Yes |
| 21 | Kif23 | 2458 | -1.058 | -0.1240 | Yes |
| 22 | Dkc1 | 2460 | -1.059 | -0.0787 | Yes |
| 23 | Slc7a5 | 2465 | -1.061 | -0.0343 | Yes |
| 24 | Slc7a1 | 2531 | -1.128 | -0.0073 | Yes |
| 25 | Slc12a2 | 2621 | -1.233 | 0.0163 | Yes |
| 26 | Sqle | 2622 | -1.234 | 0.0695 | Yes |
| 27 | Tle3 | 2801 | -1.591 | 0.0790 | Yes |
Table: GSEA details [plain text format]

  

Fig 2: HALLMARK\_G2M\_CHECKPOINT: Random ES distribution      
 Gene set null distribution of ES for **HALLMARK\_G2M\_CHECKPOINT**

  
