# Supplementary material for: TMPRSS11B promotes an acidified microenvironment and immune suppression in squamous lung cancer
Source: EMBO Rep. 2025 Nov 10;26(24):6346–79. doi: 10.1038/s44319-025-00631-1 (PMC12714794; doi:10.1038/s44319-025-00631-1)
Supplement: Supplementary file 19 — Appendix Figure S1 Source Data [file 44319_2025_631_MOESM19_ESM.zip › Appendix Figure S1/S1C/GSEA Broad Institute_low pH vs rest of the regions (high pH)_Mh/HALLMARK_GLYCOLYSIS.html]

Details for gene set HALLMARK\_GLYCOLYSIS[GSEA]

|  || Dataset | Lactate high vs low\_Ranked |
| Phenotype | NoPhenotypeAvailable |
| Upregulated in class | na\_neg |
| GeneSet | HALLMARK\_GLYCOLYSIS |
| Enrichment Score (ES) | -0.25777847 |
| Normalized Enrichment Score (NES) | -1.2883451 |
| Nominal p-value | 0.15949367 |
| FDR q-value | 0.41952363 |
| FWER p-Value | 0.997 |
Table: GSEA Results Summary

  

Fig 1: Enrichment plot: HALLMARK\_GLYCOLYSIS      
 Profile of the Running ES Score & Positions of GeneSet Members on the Rank Ordered List

  

| SYMBOL | RANK IN GENE LIST | RANK METRIC SCORE | RUNNING ES | CORE ENRICHMENT || 1 | Mertk | 24 | 1.934 | 0.0207 | No |
| 2 | Sdc3 | 106 | 1.564 | 0.0168 | No |
| 3 | Gusb | 123 | 1.530 | 0.0342 | No |
| 4 | Nol3 | 273 | 1.260 | 0.0028 | No |
| 5 | Chst12 | 357 | 1.155 | -0.0079 | No |
| 6 | Plod1 | 497 | 0.995 | -0.0398 | No |
| 7 | Col5a1 | 508 | 0.983 | -0.0286 | No |
| 8 | Hs2st1 | 615 | 0.876 | -0.0512 | No |
| 9 | Pygl | 633 | 0.865 | -0.0440 | No |
| 10 | Vegfa | 650 | 0.852 | -0.0367 | No |
| 11 | Tgfbi | 651 | 0.851 | -0.0241 | No |
| 12 | Cenpa | 655 | 0.847 | -0.0125 | No |
| 13 | Txn1 | 656 | 0.846 | 0.0001 | No |
| 14 | P4ha1 | 693 | 0.817 | 0.0002 | No |
| 15 | Gpc1 | 721 | 0.794 | 0.0029 | No |
| 16 | Cxcr4 | 847 | 0.673 | -0.0291 | No |
| 17 | Pgam1 | 880 | 0.643 | -0.0303 | No |
| 18 | Ext1 | 1064 | 0.531 | -0.0839 | No |
| 19 | Cd44 | 1072 | 0.523 | -0.0785 | No |
| 20 | Med24 | 1187 | -0.516 | -0.1091 | No |
| 21 | Sdc1 | 1228 | -0.527 | -0.1147 | No |
| 22 | Egfr | 1252 | -0.532 | -0.1145 | No |
| 23 | Fam162a | 1258 | -0.533 | -0.1083 | No |
| 24 | Got2 | 1317 | -0.545 | -0.1197 | No |
| 25 | Slc25a10 | 1343 | -0.551 | -0.1199 | No |
| 26 | Casp6 | 1431 | -0.572 | -0.1406 | No |
| 27 | Irs2 | 1443 | -0.574 | -0.1358 | No |
| 28 | Nt5e | 1516 | -0.591 | -0.1512 | No |
| 29 | Ak3 | 1592 | -0.614 | -0.1673 | No |
| 30 | Mpi | 1642 | -0.631 | -0.1744 | No |
| 31 | Pgk1 | 1680 | -0.642 | -0.1773 | No |
| 32 | Pgm2 | 1899 | -0.721 | -0.2398 | No |
| 33 | Gmppa | 1915 | -0.727 | -0.2341 | No |
| 34 | G6pdx | 1934 | -0.733 | -0.2292 | No |
| 35 | Fut8 | 1949 | -0.740 | -0.2229 | No |
| 36 | Met | 1981 | -0.751 | -0.2221 | No |
| 37 | Cog2 | 2046 | -0.783 | -0.2320 | No |
| 38 | Cited2 | 2055 | -0.788 | -0.2230 | No |
| 39 | Sap30 | 2116 | -0.814 | -0.2310 | No |
| 40 | Fkbp4 | 2117 | -0.814 | -0.2189 | No |
| 41 | B3gnt3 | 2195 | -0.856 | -0.2320 | No |
| 42 | Sox9 | 2201 | -0.860 | -0.2209 | No |
| 43 | Gmppb | 2217 | -0.868 | -0.2131 | No |
| 44 | Gale | 2351 | -0.965 | -0.2434 | Yes |
| 45 | Cldn3 | 2379 | -0.991 | -0.2377 | Yes |
| 46 | B3galt6 | 2406 | -1.010 | -0.2314 | Yes |
| 47 | Kdelr3 | 2415 | -1.016 | -0.2190 | Yes |
| 48 | Bik | 2425 | -1.027 | -0.2067 | Yes |
| 49 | Gal3st1 | 2470 | -1.064 | -0.2057 | Yes |
| 50 | Slc25a13 | 2489 | -1.082 | -0.1956 | Yes |
| 51 | Gfpt1 | 2493 | -1.086 | -0.1805 | Yes |
| 52 | Glrx | 2526 | -1.119 | -0.1746 | Yes |
| 53 | Elf3 | 2533 | -1.130 | -0.1598 | Yes |
| 54 | Tpbg | 2601 | -1.216 | -0.1642 | Yes |
| 55 | Slc35a3 | 2644 | -1.272 | -0.1594 | Yes |
| 56 | Dcn | 2701 | -1.362 | -0.1579 | Yes |
| 57 | Pam | 2741 | -1.459 | -0.1493 | Yes |
| 58 | Fbp2 | 2748 | -1.486 | -0.1292 | Yes |
| 59 | Igfbp3 | 2753 | -1.492 | -0.1084 | Yes |
| 60 | Pygb | 2757 | -1.505 | -0.0870 | Yes |
| 61 | Dsc2 | 2797 | -1.583 | -0.0765 | Yes |
| 62 | Capn5 | 2830 | -1.667 | -0.0625 | Yes |
| 63 | Isg20 | 2853 | -1.758 | -0.0437 | Yes |
| 64 | Qsox1 | 2881 | -1.902 | -0.0245 | Yes |
| 65 | Stc2 | 2919 | -2.148 | -0.0050 | Yes |
| 66 | Gpr87 | 2992 | -3.022 | 0.0158 | Yes |
Table: GSEA details [plain text format]

  

Fig 2: HALLMARK\_GLYCOLYSIS: Random ES distribution      
 Gene set null distribution of ES for **HALLMARK\_GLYCOLYSIS**

  
