# Supplementary material for: TMPRSS11B promotes an acidified microenvironment and immune suppression in squamous lung cancer
Source: EMBO Rep. 2025 Nov 10;26(24):6346–79. doi: 10.1038/s44319-025-00631-1 (PMC12714794; doi:10.1038/s44319-025-00631-1)
Supplement: Supplementary file 19 — Appendix Figure S1 Source Data [file 44319_2025_631_MOESM19_ESM.zip › Appendix Figure S1/S1C/GSEA Broad Institute_low pH vs rest of the regions (high pH)_Mh/HALLMARK_HEME_METABOLISM.html]

Details for gene set HALLMARK\_HEME\_METABOLISM[GSEA]

|  || Dataset | Lactate high vs low\_Ranked |
| Phenotype | NoPhenotypeAvailable |
| Upregulated in class | na\_neg |
| GeneSet | HALLMARK\_HEME\_METABOLISM |
| Enrichment Score (ES) | -0.153033 |
| Normalized Enrichment Score (NES) | -0.680307 |
| Nominal p-value | 0.8413793 |
| FDR q-value | 0.9126704 |
| FWER p-Value | 1.0 |
Table: GSEA Results Summary

  

Fig 1: Enrichment plot: HALLMARK\_HEME\_METABOLISM      
 Profile of the Running ES Score & Positions of GeneSet Members on the Rank Ordered List

  

| SYMBOL | RANK IN GENE LIST | RANK METRIC SCORE | RUNNING ES | CORE ENRICHMENT || 1 | Ctsb | 46 | 1.778 | 0.0318 | No |
| 2 | Hebp1 | 224 | 1.345 | 0.0084 | No |
| 3 | Cat | 297 | 1.225 | 0.0168 | No |
| 4 | Tns1 | 373 | 1.134 | 0.0219 | No |
| 5 | Acp5 | 483 | 1.008 | 0.0123 | No |
| 6 | Bmp2k | 604 | 0.888 | -0.0042 | No |
| 7 | Blvra | 612 | 0.878 | 0.0167 | No |
| 8 | Tmcc2 | 634 | 0.864 | 0.0326 | No |
| 9 | Ncoa4 | 683 | 0.825 | 0.0385 | No |
| 10 | Atp6v0a1 | 784 | 0.720 | 0.0242 | No |
| 11 | Mfhas1 | 793 | 0.712 | 0.0404 | No |
| 12 | Mpp1 | 928 | 0.611 | 0.0119 | No |
| 13 | Sdcbp | 945 | 0.602 | 0.0226 | No |
| 14 | C3 | 948 | 0.602 | 0.0378 | No |
| 15 | Kdm7a | 964 | 0.592 | 0.0485 | No |
| 16 | Picalm | 993 | 0.568 | 0.0542 | No |
| 17 | Lpin2 | 1035 | 0.546 | 0.0550 | No |
| 18 | Slc6a8 | 1041 | 0.543 | 0.0678 | No |
| 19 | Bnip3l | 1074 | 0.520 | 0.0709 | No |
| 20 | Nr3c1 | 1076 | 0.519 | 0.0843 | No |
| 21 | Agpat4 | 1086 | 0.517 | 0.0950 | No |
| 22 | Rap1gap | 1140 | -0.507 | 0.0908 | No |
| 23 | Xpo7 | 1314 | -0.544 | 0.0475 | No |
| 24 | Btg2 | 1440 | -0.573 | 0.0210 | No |
| 25 | Cir1 | 1528 | -0.593 | 0.0077 | No |
| 26 | Bsg | 1560 | -0.604 | 0.0133 | No |
| 27 | Ctse | 1586 | -0.612 | 0.0212 | No |
| 28 | Minpp1 | 1593 | -0.614 | 0.0355 | No |
| 29 | Vezf1 | 1786 | -0.682 | -0.0104 | No |
| 30 | Igsf3 | 1971 | -0.748 | -0.0520 | No |
| 31 | Gclm | 2000 | -0.761 | -0.0411 | No |
| 32 | Sec14l1 | 2112 | -0.812 | -0.0566 | No |
| 33 | Klf3 | 2248 | -0.890 | -0.0781 | No |
| 34 | Smox | 2377 | -0.990 | -0.0945 | No |
| 35 | Nudt4 | 2437 | -1.040 | -0.0867 | No |
| 36 | Ell2 | 2637 | -1.264 | -0.1195 | Yes |
| 37 | Slc6a9 | 2685 | -1.342 | -0.0996 | Yes |
| 38 | Asns | 2703 | -1.371 | -0.0689 | Yes |
| 39 | Aqp3 | 2802 | -1.594 | -0.0594 | Yes |
| 40 | Pdzk1ip1 | 2812 | -1.614 | -0.0196 | Yes |
| 41 | Tent5c | 2850 | -1.746 | 0.0144 | Yes |
| 42 | Mboat2 | 2864 | -1.824 | 0.0584 | Yes |
Table: GSEA details [plain text format]

  

Fig 2: HALLMARK\_HEME\_METABOLISM: Random ES distribution      
 Gene set null distribution of ES for **HALLMARK\_HEME\_METABOLISM**

  
