# Supplementary material for: TMPRSS11B promotes an acidified microenvironment and immune suppression in squamous lung cancer
Source: EMBO Rep. 2025 Nov 10;26(24):6346–79. doi: 10.1038/s44319-025-00631-1 (PMC12714794; doi:10.1038/s44319-025-00631-1)
Supplement: Supplementary file 19 — Appendix Figure S1 Source Data [file 44319_2025_631_MOESM19_ESM.zip › Appendix Figure S1/S1C/GSEA Broad Institute_low pH vs rest of the regions (high pH)_Mh/HALLMARK_HYPOXIA.html]

Details for gene set HALLMARK\_HYPOXIA[GSEA]

|  || Dataset | Lactate high vs low\_Ranked |
| Phenotype | NoPhenotypeAvailable |
| Upregulated in class | na\_neg |
| GeneSet | HALLMARK\_HYPOXIA |
| Enrichment Score (ES) | -0.2002304 |
| Normalized Enrichment Score (NES) | -1.0031741 |
| Nominal p-value | 0.47543162 |
| FDR q-value | 0.7651616 |
| FWER p-Value | 1.0 |
Table: GSEA Results Summary

  

Fig 1: Enrichment plot: HALLMARK\_HYPOXIA      
 Profile of the Running ES Score & Positions of GeneSet Members on the Rank Ordered List

  

| SYMBOL | RANK IN GENE LIST | RANK METRIC SCORE | RUNNING ES | CORE ENRICHMENT || 1 | Hmox1 | 16 | 2.051 | 0.0262 | No |
| 2 | Plin2 | 90 | 1.607 | 0.0265 | No |
| 3 | Sdc3 | 106 | 1.564 | 0.0455 | No |
| 4 | Cav1 | 107 | 1.564 | 0.0696 | No |
| 5 | Atf3 | 161 | 1.444 | 0.0741 | No |
| 6 | Cdkn1c | 446 | 1.049 | -0.0052 | No |
| 7 | Hexa | 476 | 1.021 | 0.0008 | No |
| 8 | Col5a1 | 508 | 0.983 | 0.0056 | No |
| 9 | Ccn5 | 542 | 0.955 | 0.0092 | No |
| 10 | Lox | 559 | 0.942 | 0.0183 | No |
| 11 | Dusp1 | 569 | 0.935 | 0.0297 | No |
| 12 | Vegfa | 650 | 0.852 | 0.0160 | No |
| 13 | Tgfbi | 651 | 0.851 | 0.0291 | No |
| 14 | Ets1 | 671 | 0.831 | 0.0355 | No |
| 15 | P4ha1 | 693 | 0.817 | 0.0410 | No |
| 16 | Akap12 | 713 | 0.801 | 0.0470 | No |
| 17 | Gpc1 | 721 | 0.794 | 0.0569 | No |
| 18 | Wsb1 | 730 | 0.780 | 0.0662 | No |
| 19 | Scarb1 | 738 | 0.772 | 0.0757 | No |
| 20 | Cdkn1a | 749 | 0.765 | 0.0842 | No |
| 21 | Cxcr4 | 847 | 0.673 | 0.0619 | No |
| 22 | Pim1 | 859 | 0.656 | 0.0684 | No |
| 23 | Bgn | 870 | 0.650 | 0.0750 | No |
| 24 | Atp7a | 874 | 0.648 | 0.0840 | No |
| 25 | Pklr | 949 | 0.602 | 0.0684 | No |
| 26 | Ndst1 | 1019 | 0.553 | 0.0537 | No |
| 27 | Ext1 | 1064 | 0.531 | 0.0472 | No |
| 28 | Bnip3l | 1074 | 0.520 | 0.0521 | No |
| 29 | Nr3c1 | 1076 | 0.519 | 0.0598 | No |
| 30 | Plaur | 1098 | 0.504 | 0.0605 | No |
| 31 | Slc25a1 | 1126 | -0.504 | 0.0592 | No |
| 32 | Egfr | 1252 | -0.532 | 0.0254 | No |
| 33 | Fam162a | 1258 | -0.533 | 0.0320 | No |
| 34 | Maff | 1259 | -0.533 | 0.0402 | No |
| 35 | Fosl2 | 1283 | -0.538 | 0.0407 | No |
| 36 | Pdk1 | 1331 | -0.549 | 0.0334 | No |
| 37 | Bcl2 | 1390 | -0.561 | 0.0226 | No |
| 38 | Casp6 | 1431 | -0.572 | 0.0179 | No |
| 39 | Irs2 | 1443 | -0.574 | 0.0231 | No |
| 40 | Pgk1 | 1680 | -0.642 | -0.0463 | No |
| 41 | Aldoc | 1714 | -0.659 | -0.0472 | No |
| 42 | Pgm2 | 1899 | -0.721 | -0.0979 | No |
| 43 | Siah2 | 1909 | -0.724 | -0.0898 | No |
| 44 | Cited2 | 2055 | -0.788 | -0.1264 | No |
| 45 | Klhl24 | 2080 | -0.799 | -0.1221 | No |
| 46 | Sap30 | 2116 | -0.814 | -0.1213 | No |
| 47 | Grhpr | 2258 | -0.898 | -0.1549 | No |
| 48 | F3 | 2372 | -0.985 | -0.1776 | No |
| 49 | B3galt6 | 2406 | -1.010 | -0.1732 | No |
| 50 | Kdelr3 | 2415 | -1.016 | -0.1602 | No |
| 51 | Fos | 2491 | -1.083 | -0.1687 | No |
| 52 | Glrx | 2526 | -1.119 | -0.1629 | No |
| 53 | Tpbg | 2601 | -1.216 | -0.1690 | No |
| 54 | Plac8 | 2695 | -1.353 | -0.1794 | Yes |
| 55 | Dcn | 2701 | -1.362 | -0.1601 | Yes |
| 56 | Pam | 2741 | -1.459 | -0.1507 | Yes |
| 57 | Igfbp3 | 2753 | -1.492 | -0.1314 | Yes |
| 58 | Hs3st1 | 2816 | -1.624 | -0.1272 | Yes |
| 59 | Isg20 | 2853 | -1.758 | -0.1122 | Yes |
| 60 | Stc2 | 2919 | -2.148 | -0.1009 | Yes |
| 61 | Tmem45a | 2934 | -2.244 | -0.0711 | Yes |
| 62 | Pkp1 | 2996 | -3.083 | -0.0441 | Yes |
| 63 | Car12 | 3022 | -3.775 | 0.0057 | Yes |
Table: GSEA details [plain text format]

  

Fig 2: HALLMARK\_HYPOXIA: Random ES distribution      
 Gene set null distribution of ES for **HALLMARK\_HYPOXIA**

  
