# Supplementary material for: TMPRSS11B promotes an acidified microenvironment and immune suppression in squamous lung cancer
Source: EMBO Rep. 2025 Nov 10;26(24):6346–79. doi: 10.1038/s44319-025-00631-1 (PMC12714794; doi:10.1038/s44319-025-00631-1)
Supplement: Supplementary file 19 — Appendix Figure S1 Source Data [file 44319_2025_631_MOESM19_ESM.zip › Appendix Figure S1/S1C/GSEA Broad Institute_low pH vs rest of the regions (high pH)_Mh/HALLMARK_IL2_STAT5_SIGNALING.html]

Details for gene set HALLMARK\_IL2\_STAT5\_SIGNALING[GSEA]

|  || Dataset | Lactate high vs low\_Ranked |
| Phenotype | NoPhenotypeAvailable |
| Upregulated in class | na\_pos |
| GeneSet | HALLMARK\_IL2\_STAT5\_SIGNALING |
| Enrichment Score (ES) | 0.17784809 |
| Normalized Enrichment Score (NES) | 1.1463549 |
| Nominal p-value | 0.28959277 |
| FDR q-value | 0.4205365 |
| FWER p-Value | 0.959 |
Table: GSEA Results Summary

  

Fig 1: Enrichment plot: HALLMARK\_IL2\_STAT5\_SIGNALING      
 Profile of the Running ES Score & Positions of GeneSet Members on the Rank Ordered List

  

| SYMBOL | RANK IN GENE LIST | RANK METRIC SCORE | RUNNING ES | CORE ENRICHMENT || 1 | Ager | 41 | 1.803 | 0.0132 | Yes |
| 2 | Plin2 | 90 | 1.607 | 0.0212 | Yes |
| 3 | Cd83 | 101 | 1.575 | 0.0415 | Yes |
| 4 | Cd48 | 146 | 1.476 | 0.0488 | Yes |
| 5 | Gpr65 | 151 | 1.461 | 0.0694 | Yes |
| 6 | Tnfrsf1b | 171 | 1.412 | 0.0842 | Yes |
| 7 | Spp1 | 232 | 1.327 | 0.0839 | Yes |
| 8 | Ctsz | 242 | 1.303 | 0.1004 | Yes |
| 9 | Col6a1 | 246 | 1.297 | 0.1188 | Yes |
| 10 | Lrrc8c | 287 | 1.235 | 0.1239 | Yes |
| 11 | Adam19 | 312 | 1.209 | 0.1340 | Yes |
| 12 | Nrp1 | 353 | 1.158 | 0.1379 | Yes |
| 13 | Fgl2 | 428 | 1.073 | 0.1291 | Yes |
| 14 | She | 444 | 1.051 | 0.1398 | Yes |
| 15 | Cdkn1c | 446 | 1.049 | 0.1552 | Yes |
| 16 | Dennd5a | 521 | 0.973 | 0.1449 | Yes |
| 17 | Il3ra | 609 | 0.883 | 0.1288 | Yes |
| 18 | Irf8 | 663 | 0.840 | 0.1236 | Yes |
| 19 | Slc39a8 | 674 | 0.830 | 0.1327 | Yes |
| 20 | Gbp3 | 686 | 0.821 | 0.1413 | Yes |
| 21 | P4ha1 | 693 | 0.817 | 0.1515 | Yes |
| 22 | Dhrs3 | 703 | 0.807 | 0.1606 | Yes |
| 23 | Ifngr1 | 740 | 0.770 | 0.1601 | Yes |
| 24 | Hipk2 | 746 | 0.766 | 0.1699 | Yes |
| 25 | Spry4 | 794 | 0.711 | 0.1647 | Yes |
| 26 | Map6 | 814 | 0.697 | 0.1688 | Yes |
| 27 | Prnp | 820 | 0.690 | 0.1775 | Yes |
| 28 | Pim1 | 859 | 0.656 | 0.1745 | Yes |
| 29 | Ltb | 902 | 0.628 | 0.1698 | Yes |
| 30 | Traf1 | 907 | 0.626 | 0.1778 | Yes |
| 31 | Myo1e | 1010 | 0.558 | 0.1519 | No |
| 32 | Gabarapl1 | 1061 | 0.533 | 0.1431 | No |
| 33 | Cd44 | 1072 | 0.523 | 0.1475 | No |
| 34 | Etfbkmt | 1194 | -0.519 | 0.1146 | No |
| 35 | Rabgap1l | 1215 | -0.525 | 0.1158 | No |
| 36 | Maff | 1259 | -0.533 | 0.1093 | No |
| 37 | Dcps | 1357 | -0.554 | 0.0850 | No |
| 38 | Bcl2 | 1390 | -0.561 | 0.0826 | No |
| 39 | Pdcd2l | 1401 | -0.563 | 0.0877 | No |
| 40 | Pus1 | 1450 | -0.575 | 0.0802 | No |
| 41 | Nt5e | 1516 | -0.591 | 0.0671 | No |
| 42 | Spred2 | 1590 | -0.614 | 0.0518 | No |
| 43 | St3gal4 | 1629 | -0.626 | 0.0484 | No |
| 44 | Ckap4 | 1631 | -0.627 | 0.0575 | No |
| 45 | Nop2 | 1663 | -0.637 | 0.0566 | No |
| 46 | Tnfrsf21 | 1702 | -0.654 | 0.0536 | No |
| 47 | Galm | 1846 | -0.705 | 0.0161 | No |
| 48 | Xbp1 | 1976 | -0.750 | -0.0161 | No |
| 49 | Mxd1 | 2005 | -0.763 | -0.0141 | No |
| 50 | Ptrh2 | 2096 | -0.805 | -0.0323 | No |
| 51 | Ccnd2 | 2242 | -0.887 | -0.0678 | No |
| 52 | Sh3bgrl2 | 2257 | -0.897 | -0.0590 | No |
| 53 | Amacr | 2285 | -0.916 | -0.0544 | No |
| 54 | Phlda1 | 2291 | -0.920 | -0.0423 | No |
| 55 | Tiam1 | 2352 | -0.967 | -0.0479 | No |
| 56 | Uck2 | 2365 | -0.978 | -0.0373 | No |
| 57 | Ahr | 2374 | -0.988 | -0.0252 | No |
| 58 | Slc1a5 | 2375 | -0.989 | -0.0103 | No |
| 59 | Hopx | 2384 | -0.995 | 0.0019 | No |
| 60 | Nfkbiz | 2444 | -1.047 | -0.0023 | No |
| 61 | Irf6 | 2657 | -1.295 | -0.0542 | No |
| 62 | Cdcp1 | 2704 | -1.374 | -0.0491 | No |
| 63 | Ecm1 | 2738 | -1.455 | -0.0383 | No |
| 64 | Muc1 | 2794 | -1.579 | -0.0332 | No |
| 65 | Ikzf2 | 2807 | -1.599 | -0.0132 | No |
| 66 | Igf1r | 2859 | -1.811 | -0.0032 | No |
| 67 | Gsto1 | 2912 | -2.103 | 0.0108 | No |
| 68 | Lrig1 | 2913 | -2.103 | 0.0424 | No |
Table: GSEA details [plain text format]

  

Fig 2: HALLMARK\_IL2\_STAT5\_SIGNALING: Random ES distribution      
 Gene set null distribution of ES for **HALLMARK\_IL2\_STAT5\_SIGNALING**

  
