# Supplementary material for: TMPRSS11B promotes an acidified microenvironment and immune suppression in squamous lung cancer
Source: EMBO Rep. 2025 Nov 10;26(24):6346–79. doi: 10.1038/s44319-025-00631-1 (PMC12714794; doi:10.1038/s44319-025-00631-1)
Supplement: Supplementary file 19 — Appendix Figure S1 Source Data [file 44319_2025_631_MOESM19_ESM.zip › Appendix Figure S1/S1C/GSEA Broad Institute_low pH vs rest of the regions (high pH)_Mh/HALLMARK_IL6_JAK_STAT3_SIGNALING.html]

Details for gene set HALLMARK\_IL6\_JAK\_STAT3\_SIGNALING[GSEA]

|  || Dataset | Lactate high vs low\_Ranked |
| Phenotype | NoPhenotypeAvailable |
| Upregulated in class | na\_pos |
| GeneSet | HALLMARK\_IL6\_JAK\_STAT3\_SIGNALING |
| Enrichment Score (ES) | 0.46882418 |
| Normalized Enrichment Score (NES) | 2.2173038 |
| Nominal p-value | 0.0 |
| FDR q-value | 0.002427747 |
| FWER p-Value | 0.007 |
Table: GSEA Results Summary

  

Fig 1: Enrichment plot: HALLMARK\_IL6\_JAK\_STAT3\_SIGNALING      
 Profile of the Running ES Score & Positions of GeneSet Members on the Rank Ordered List

  

| SYMBOL | RANK IN GENE LIST | RANK METRIC SCORE | RUNNING ES | CORE ENRICHMENT || 1 | Hmox1 | 16 | 2.051 | 0.0679 | Yes |
| 2 | Cd36 | 52 | 1.748 | 0.1188 | Yes |
| 3 | Pik3r5 | 63 | 1.685 | 0.1756 | Yes |
| 4 | Csf2ra | 143 | 1.478 | 0.2022 | Yes |
| 5 | Tnfrsf1b | 171 | 1.412 | 0.2437 | Yes |
| 6 | Crlf2 | 352 | 1.164 | 0.2255 | Yes |
| 7 | Il2rg | 380 | 1.128 | 0.2569 | Yes |
| 8 | Fas | 457 | 1.039 | 0.2688 | Yes |
| 9 | Acvrl1 | 462 | 1.038 | 0.3045 | Yes |
| 10 | Il10rb | 469 | 1.031 | 0.3394 | Yes |
| 11 | Itgb3 | 516 | 0.975 | 0.3590 | Yes |
| 12 | Il3ra | 609 | 0.883 | 0.3600 | Yes |
| 13 | Tgfb1 | 664 | 0.839 | 0.3720 | Yes |
| 14 | Ifngr1 | 740 | 0.770 | 0.3747 | Yes |
| 15 | Osmr | 751 | 0.762 | 0.3986 | Yes |
| 16 | Grb2 | 816 | 0.695 | 0.4022 | Yes |
| 17 | Pim1 | 859 | 0.656 | 0.4117 | Yes |
| 18 | Ltb | 902 | 0.628 | 0.4202 | Yes |
| 19 | Csf3r | 924 | 0.615 | 0.4352 | Yes |
| 20 | Ptpn1 | 940 | 0.604 | 0.4518 | Yes |
| 21 | Tyk2 | 954 | 0.598 | 0.4688 | Yes |
| 22 | Cd44 | 1072 | 0.523 | 0.4487 | No |
| 23 | Tnfrsf21 | 1702 | -0.654 | 0.2634 | No |
| 24 | Tlr2 | 2819 | -1.629 | -0.0485 | No |
| 25 | Reg1 | 3012 | -3.392 | 0.0090 | No |
Table: GSEA details [plain text format]

  

Fig 2: HALLMARK\_IL6\_JAK\_STAT3\_SIGNALING: Random ES distribution      
 Gene set null distribution of ES for **HALLMARK\_IL6\_JAK\_STAT3\_SIGNALING**

  
