# Supplementary material for: TMPRSS11B promotes an acidified microenvironment and immune suppression in squamous lung cancer
Source: EMBO Rep. 2025 Nov 10;26(24):6346–79. doi: 10.1038/s44319-025-00631-1 (PMC12714794; doi:10.1038/s44319-025-00631-1)
Supplement: Supplementary file 19 — Appendix Figure S1 Source Data [file 44319_2025_631_MOESM19_ESM.zip › Appendix Figure S1/S1C/GSEA Broad Institute_low pH vs rest of the regions (high pH)_Mh/HALLMARK_INFLAMMATORY_RESPONSE.html]

Details for gene set HALLMARK\_INFLAMMATORY\_RESPONSE[GSEA]

|  || Dataset | Lactate high vs low\_Ranked |
| Phenotype | NoPhenotypeAvailable |
| Upregulated in class | na\_pos |
| GeneSet | HALLMARK\_INFLAMMATORY\_RESPONSE |
| Enrichment Score (ES) | 0.2750859 |
| Normalized Enrichment Score (NES) | 1.629107 |
| Nominal p-value | 0.01984127 |
| FDR q-value | 0.062735125 |
| FWER p-Value | 0.256 |
Table: GSEA Results Summary

  

Fig 1: Enrichment plot: HALLMARK\_INFLAMMATORY\_RESPONSE      
 Profile of the Running ES Score & Positions of GeneSet Members on the Rank Ordered List

  

| SYMBOL | RANK IN GENE LIST | RANK METRIC SCORE | RUNNING ES | CORE ENRICHMENT || 1 | C3ar1 | 4 | 2.207 | 0.0388 | Yes |
| 2 | Pik3r5 | 63 | 1.685 | 0.0501 | Yes |
| 3 | Itga5 | 85 | 1.616 | 0.0725 | Yes |
| 4 | Rgs1 | 105 | 1.567 | 0.0946 | Yes |
| 5 | Lamp3 | 109 | 1.560 | 0.1220 | Yes |
| 6 | Cybb | 119 | 1.535 | 0.1470 | Yes |
| 7 | Cd48 | 146 | 1.476 | 0.1651 | Yes |
| 8 | Tnfrsf1b | 171 | 1.412 | 0.1828 | Yes |
| 9 | Cxcl15 | 190 | 1.391 | 0.2021 | Yes |
| 10 | Emp3 | 191 | 1.389 | 0.2274 | Yes |
| 11 | Abca1 | 206 | 1.367 | 0.2476 | Yes |
| 12 | Msr1 | 252 | 1.288 | 0.2560 | Yes |
| 13 | Ptafr | 395 | 1.106 | 0.2286 | Yes |
| 14 | Pdpn | 429 | 1.071 | 0.2371 | Yes |
| 15 | Lcp2 | 449 | 1.047 | 0.2498 | Yes |
| 16 | Icam1 | 492 | 0.999 | 0.2539 | Yes |
| 17 | Itgb3 | 516 | 0.975 | 0.2639 | Yes |
| 18 | Ptpre | 623 | 0.871 | 0.2444 | Yes |
| 19 | Calcrl | 631 | 0.867 | 0.2578 | Yes |
| 20 | Axl | 729 | 0.782 | 0.2396 | Yes |
| 21 | Bst2 | 736 | 0.772 | 0.2516 | Yes |
| 22 | Cdkn1a | 749 | 0.765 | 0.2615 | Yes |
| 23 | Osmr | 751 | 0.762 | 0.2751 | Yes |
| 24 | Slc7a2 | 795 | 0.711 | 0.2736 | No |
| 25 | Slc31a2 | 832 | 0.680 | 0.2740 | No |
| 26 | Csf3r | 924 | 0.615 | 0.2548 | No |
| 27 | Rhog | 932 | 0.609 | 0.2635 | No |
| 28 | P2rx7 | 951 | 0.599 | 0.2684 | No |
| 29 | Plaur | 1098 | 0.504 | 0.2287 | No |
| 30 | Gabbr1 | 1173 | -0.513 | 0.2133 | No |
| 31 | Selenos | 1201 | -0.520 | 0.2138 | No |
| 32 | Gch1 | 1277 | -0.537 | 0.1985 | No |
| 33 | Btg2 | 1440 | -0.573 | 0.1547 | No |
| 34 | Hbegf | 1860 | -0.709 | 0.0275 | No |
| 35 | Met | 1981 | -0.751 | 0.0011 | No |
| 36 | Mxd1 | 2005 | -0.763 | 0.0073 | No |
| 37 | F3 | 2372 | -0.985 | -0.0972 | No |
| 38 | Ahr | 2374 | -0.988 | -0.0795 | No |
| 39 | Ly6e | 2392 | -1.001 | -0.0670 | No |
| 40 | Atp2a2 | 2399 | -1.006 | -0.0507 | No |
| 41 | Slc7a1 | 2531 | -1.128 | -0.0740 | No |
| 42 | Il18 | 2586 | -1.191 | -0.0703 | No |
| 43 | Tpbg | 2601 | -1.216 | -0.0529 | No |
| 44 | Irf7 | 2638 | -1.265 | -0.0419 | No |
| 45 | Hpn | 2653 | -1.291 | -0.0231 | No |
| 46 | Slc4a4 | 2742 | -1.460 | -0.0260 | No |
| 47 | Tlr2 | 2819 | -1.629 | -0.0217 | No |
| 48 | Ifitm1 | 2906 | -2.063 | -0.0129 | No |
| 49 | Cxcl5 | 2999 | -3.136 | 0.0134 | No |
Table: GSEA details [plain text format]

  

Fig 2: HALLMARK\_INFLAMMATORY\_RESPONSE: Random ES distribution      
 Gene set null distribution of ES for **HALLMARK\_INFLAMMATORY\_RESPONSE**

  
