# Supplementary material for: TMPRSS11B promotes an acidified microenvironment and immune suppression in squamous lung cancer
Source: EMBO Rep. 2025 Nov 10;26(24):6346–79. doi: 10.1038/s44319-025-00631-1 (PMC12714794; doi:10.1038/s44319-025-00631-1)
Supplement: Supplementary file 19 — Appendix Figure S1 Source Data [file 44319_2025_631_MOESM19_ESM.zip › Appendix Figure S1/S1C/GSEA Broad Institute_low pH vs rest of the regions (high pH)_Mh/HALLMARK_INTERFERON_ALPHA_RESPONSE.html]

Details for gene set HALLMARK\_INTERFERON\_ALPHA\_RESPONSE[GSEA]

|  || Dataset | Lactate high vs low\_Ranked |
| Phenotype | NoPhenotypeAvailable |
| Upregulated in class | na\_neg |
| GeneSet | HALLMARK\_INTERFERON\_ALPHA\_RESPONSE |
| Enrichment Score (ES) | -0.22610866 |
| Normalized Enrichment Score (NES) | -0.7499286 |
| Nominal p-value | 0.7379845 |
| FDR q-value | 0.93283194 |
| FWER p-Value | 1.0 |
Table: GSEA Results Summary

  

Fig 1: Enrichment plot: HALLMARK\_INTERFERON\_ALPHA\_RESPONSE      
 Profile of the Running ES Score & Positions of GeneSet Members on the Rank Ordered List

  

| SYMBOL | RANK IN GENE LIST | RANK METRIC SCORE | RUNNING ES | CORE ENRICHMENT || 1 | Lamp3 | 109 | 1.560 | 0.0604 | No |
| 2 | Cd74 | 376 | 1.133 | 0.0425 | No |
| 3 | B2m | 402 | 1.097 | 0.1020 | No |
| 4 | Tmem140 | 495 | 0.995 | 0.1331 | No |
| 5 | Gbp3 | 686 | 0.821 | 0.1211 | No |
| 6 | Bst2 | 736 | 0.772 | 0.1526 | No |
| 7 | Psmb8 | 838 | 0.678 | 0.1611 | No |
| 8 | Tdrd7 | 1333 | -0.549 | 0.0318 | No |
| 9 | Usp18 | 1621 | -0.622 | -0.0247 | No |
| 10 | Oas1a | 2188 | -0.854 | -0.1590 | No |
| 11 | Ly6e | 2392 | -1.001 | -0.1642 | Yes |
| 12 | Oasl1 | 2408 | -1.010 | -0.1067 | Yes |
| 13 | Irf7 | 2638 | -1.265 | -0.1043 | Yes |
| 14 | Isg20 | 2853 | -1.758 | -0.0664 | Yes |
| 15 | Ifitm1 | 2906 | -2.063 | 0.0440 | Yes |
Table: GSEA details [plain text format]

  

Fig 2: HALLMARK\_INTERFERON\_ALPHA\_RESPONSE: Random ES distribution      
 Gene set null distribution of ES for **HALLMARK\_INTERFERON\_ALPHA\_RESPONSE**

  
