# Supplementary material for: TMPRSS11B promotes an acidified microenvironment and immune suppression in squamous lung cancer
Source: EMBO Rep. 2025 Nov 10;26(24):6346–79. doi: 10.1038/s44319-025-00631-1 (PMC12714794; doi:10.1038/s44319-025-00631-1)
Supplement: Supplementary file 19 — Appendix Figure S1 Source Data [file 44319_2025_631_MOESM19_ESM.zip › Appendix Figure S1/S1C/GSEA Broad Institute_low pH vs rest of the regions (high pH)_Mh/HALLMARK_INTERFERON_GAMMA_RESPONSE.html]

Details for gene set HALLMARK\_INTERFERON\_GAMMA\_RESPONSE[GSEA]

|  || Dataset | Lactate high vs low\_Ranked |
| Phenotype | NoPhenotypeAvailable |
| Upregulated in class | na\_pos |
| GeneSet | HALLMARK\_INTERFERON\_GAMMA\_RESPONSE |
| Enrichment Score (ES) | 0.31425297 |
| Normalized Enrichment Score (NES) | 1.7705812 |
| Nominal p-value | 0.004048583 |
| FDR q-value | 0.03797622 |
| FWER p-Value | 0.11 |
Table: GSEA Results Summary

  

Fig 1: Enrichment plot: HALLMARK\_INTERFERON\_GAMMA\_RESPONSE      
 Profile of the Running ES Score & Positions of GeneSet Members on the Rank Ordered List

  

| SYMBOL | RANK IN GENE LIST | RANK METRIC SCORE | RUNNING ES | CORE ENRICHMENT || 1 | Slamf7 | 25 | 1.910 | 0.0358 | Yes |
| 2 | Fcgr1 | 38 | 1.818 | 0.0738 | Yes |
| 3 | Cd274 | 104 | 1.569 | 0.0883 | Yes |
| 4 | St8sia4 | 225 | 1.343 | 0.0793 | Yes |
| 5 | Nlrc5 | 374 | 1.134 | 0.0560 | Yes |
| 6 | Cd74 | 376 | 1.133 | 0.0819 | Yes |
| 7 | B2m | 402 | 1.097 | 0.0989 | Yes |
| 8 | H2-DMa | 418 | 1.083 | 0.1189 | Yes |
| 9 | Fgl2 | 428 | 1.073 | 0.1407 | Yes |
| 10 | Lcp2 | 449 | 1.047 | 0.1582 | Yes |
| 11 | Fas | 457 | 1.039 | 0.1799 | Yes |
| 12 | H2-Aa | 465 | 1.035 | 0.2014 | Yes |
| 13 | Icam1 | 492 | 0.999 | 0.2158 | Yes |
| 14 | Samhd1 | 505 | 0.985 | 0.2346 | Yes |
| 15 | Serping1 | 519 | 0.973 | 0.2527 | Yes |
| 16 | Ciita | 536 | 0.959 | 0.2695 | Yes |
| 17 | Irf5 | 624 | 0.871 | 0.2606 | Yes |
| 18 | Il18bp | 640 | 0.858 | 0.2754 | Yes |
| 19 | Irf8 | 663 | 0.840 | 0.2875 | Yes |
| 20 | Gbp3 | 686 | 0.821 | 0.2991 | Yes |
| 21 | Bst2 | 736 | 0.772 | 0.3006 | Yes |
| 22 | Cdkn1a | 749 | 0.765 | 0.3143 | Yes |
| 23 | Psmb8 | 838 | 0.678 | 0.3005 | No |
| 24 | Pim1 | 859 | 0.656 | 0.3090 | No |
| 25 | Ptpn1 | 940 | 0.604 | 0.2963 | No |
| 26 | Ifnar2 | 958 | 0.597 | 0.3044 | No |
| 27 | Cfh | 1030 | 0.548 | 0.2933 | No |
| 28 | Eif4e3 | 1044 | 0.541 | 0.3015 | No |
| 29 | Ptpn6 | 1096 | 0.505 | 0.2961 | No |
| 30 | Gch1 | 1277 | -0.537 | 0.2484 | No |
| 31 | Tdrd7 | 1333 | -0.549 | 0.2427 | No |
| 32 | Casp4 | 1495 | -0.585 | 0.2025 | No |
| 33 | Rnf213 | 1561 | -0.604 | 0.1947 | No |
| 34 | Casp7 | 1587 | -0.613 | 0.2005 | No |
| 35 | Rapgef6 | 1614 | -0.620 | 0.2062 | No |
| 36 | Usp18 | 1621 | -0.622 | 0.2186 | No |
| 37 | Zbp1 | 1962 | -0.745 | 0.1223 | No |
| 38 | Upp1 | 2304 | -0.929 | 0.0299 | No |
| 39 | Ly6e | 2392 | -1.001 | 0.0239 | No |
| 40 | Oasl1 | 2408 | -1.010 | 0.0423 | No |
| 41 | St3gal5 | 2575 | -1.175 | 0.0140 | No |
| 42 | Irf7 | 2638 | -1.265 | 0.0225 | No |
| 43 | Auts2 | 2751 | -1.491 | 0.0196 | No |
| 44 | Cfb | 2772 | -1.529 | 0.0482 | No |
| 45 | Isg20 | 2853 | -1.758 | 0.0621 | No |
Table: GSEA details [plain text format]

  

Fig 2: HALLMARK\_INTERFERON\_GAMMA\_RESPONSE: Random ES distribution      
 Gene set null distribution of ES for **HALLMARK\_INTERFERON\_GAMMA\_RESPONSE**

  
