# Supplementary material for: TMPRSS11B promotes an acidified microenvironment and immune suppression in squamous lung cancer
Source: EMBO Rep. 2025 Nov 10;26(24):6346–79. doi: 10.1038/s44319-025-00631-1 (PMC12714794; doi:10.1038/s44319-025-00631-1)
Supplement: Supplementary file 19 — Appendix Figure S1 Source Data [file 44319_2025_631_MOESM19_ESM.zip › Appendix Figure S1/S1C/GSEA Broad Institute_low pH vs rest of the regions (high pH)_Mh/HALLMARK_KRAS_SIGNALING_DN.html]

Details for gene set HALLMARK\_KRAS\_SIGNALING\_DN[GSEA]

|  || Dataset | Lactate high vs low\_Ranked |
| Phenotype | NoPhenotypeAvailable |
| Upregulated in class | na\_neg |
| GeneSet | HALLMARK\_KRAS\_SIGNALING\_DN |
| Enrichment Score (ES) | -0.70125526 |
| Normalized Enrichment Score (NES) | -2.9203906 |
| Nominal p-value | 0.0 |
| FDR q-value | 0.0 |
| FWER p-Value | 0.0 |
Table: GSEA Results Summary

  

Fig 1: Enrichment plot: HALLMARK\_KRAS\_SIGNALING\_DN      
 Profile of the Running ES Score & Positions of GeneSet Members on the Rank Ordered List

  

| SYMBOL | RANK IN GENE LIST | RANK METRIC SCORE | RUNNING ES | CORE ENRICHMENT || 1 | Slc29a3 | 219 | 1.353 | -0.0488 | No |
| 2 | Zbtb16 | 311 | 1.211 | -0.0576 | No |
| 3 | Snn | 534 | 0.960 | -0.1143 | No |
| 4 | Hc | 562 | 0.941 | -0.1066 | No |
| 5 | Tcf7l1 | 810 | 0.700 | -0.1762 | No |
| 6 | Btg2 | 1440 | -0.573 | -0.3750 | No |
| 7 | Cdkal1 | 1961 | -0.745 | -0.5346 | No |
| 8 | Camk1d | 2130 | -0.818 | -0.5759 | No |
| 9 | Prodh | 2180 | -0.848 | -0.5772 | No |
| 10 | Thrb | 2186 | -0.852 | -0.5637 | No |
| 11 | Tfcp2l1 | 2528 | -1.125 | -0.6571 | No |
| 12 | Fgfr3 | 2662 | -1.308 | -0.6781 | Yes |
| 13 | Cyp39a1 | 2663 | -1.308 | -0.6549 | Yes |
| 14 | Nr4a2 | 2700 | -1.361 | -0.6427 | Yes |
| 15 | Sptbn2 | 2707 | -1.377 | -0.6203 | Yes |
| 16 | Sidt1 | 2729 | -1.424 | -0.6020 | Yes |
| 17 | Mfsd6 | 2773 | -1.532 | -0.5892 | Yes |
| 18 | Tent5c | 2850 | -1.746 | -0.5835 | Yes |
| 19 | Tgm1 | 2866 | -1.841 | -0.5558 | Yes |
| 20 | Gprc5c | 2909 | -2.080 | -0.5329 | Yes |
| 21 | Krt15 | 2935 | -2.256 | -0.5012 | Yes |
| 22 | Celsr2 | 2961 | -2.404 | -0.4669 | Yes |
| 23 | Krt4 | 2983 | -2.885 | -0.4227 | Yes |
| 24 | Pkp1 | 2996 | -3.083 | -0.3721 | Yes |
| 25 | Lypd3 | 3005 | -3.177 | -0.3184 | Yes |
| 26 | Sprr3 | 3007 | -3.316 | -0.2600 | Yes |
| 27 | Krt5 | 3013 | -3.435 | -0.2007 | Yes |
| 28 | Krt13 | 3025 | -3.823 | -0.1366 | Yes |
| 29 | Lgals7 | 3028 | -3.903 | -0.0681 | Yes |
| 30 | Tff2 | 3031 | -4.030 | 0.0027 | Yes |
Table: GSEA details [plain text format]

  

Fig 2: HALLMARK\_KRAS\_SIGNALING\_DN: Random ES distribution      
 Gene set null distribution of ES for **HALLMARK\_KRAS\_SIGNALING\_DN**

  
