# Supplementary material for: TMPRSS11B promotes an acidified microenvironment and immune suppression in squamous lung cancer
Source: EMBO Rep. 2025 Nov 10;26(24):6346–79. doi: 10.1038/s44319-025-00631-1 (PMC12714794; doi:10.1038/s44319-025-00631-1)
Supplement: Supplementary file 19 — Appendix Figure S1 Source Data [file 44319_2025_631_MOESM19_ESM.zip › Appendix Figure S1/S1C/GSEA Broad Institute_low pH vs rest of the regions (high pH)_Mh/HALLMARK_KRAS_SIGNALING_UP.html]

Details for gene set HALLMARK\_KRAS\_SIGNALING\_UP[GSEA]

|  || Dataset | Lactate high vs low\_Ranked |
| Phenotype | NoPhenotypeAvailable |
| Upregulated in class | na\_pos |
| GeneSet | HALLMARK\_KRAS\_SIGNALING\_UP |
| Enrichment Score (ES) | 0.27275234 |
| Normalized Enrichment Score (NES) | 1.7525212 |
| Nominal p-value | 0.0048076925 |
| FDR q-value | 0.03668281 |
| FWER p-Value | 0.125 |
Table: GSEA Results Summary

  

Fig 1: Enrichment plot: HALLMARK\_KRAS\_SIGNALING\_UP      
 Profile of the Running ES Score & Positions of GeneSet Members on the Rank Ordered List

  

| SYMBOL | RANK IN GENE LIST | RANK METRIC SCORE | RUNNING ES | CORE ENRICHMENT || 1 | Gpnmb | 3 | 2.213 | 0.0237 | Yes |
| 2 | C3ar1 | 4 | 2.207 | 0.0483 | Yes |
| 3 | Ctss | 14 | 2.088 | 0.0685 | Yes |
| 4 | Mafb | 22 | 1.943 | 0.0878 | Yes |
| 5 | Cd37 | 49 | 1.763 | 0.0987 | Yes |
| 6 | Lat2 | 64 | 1.685 | 0.1128 | Yes |
| 7 | Fcer1g | 95 | 1.597 | 0.1204 | Yes |
| 8 | Itgb2 | 138 | 1.498 | 0.1230 | Yes |
| 9 | Etv5 | 142 | 1.482 | 0.1385 | Yes |
| 10 | Csf2ra | 143 | 1.478 | 0.1549 | Yes |
| 11 | Tnfrsf1b | 171 | 1.412 | 0.1616 | Yes |
| 12 | Adam8 | 222 | 1.351 | 0.1598 | Yes |
| 13 | Dock2 | 229 | 1.333 | 0.1726 | Yes |
| 14 | Spp1 | 232 | 1.327 | 0.1867 | Yes |
| 15 | Tmem100 | 258 | 1.277 | 0.1925 | Yes |
| 16 | Ikzf1 | 285 | 1.236 | 0.1975 | Yes |
| 17 | Mmp11 | 313 | 1.209 | 0.2019 | Yes |
| 18 | Ace | 329 | 1.185 | 0.2100 | Yes |
| 19 | Mmd | 349 | 1.166 | 0.2166 | Yes |
| 20 | Nrp1 | 353 | 1.158 | 0.2285 | Yes |
| 21 | Nin | 359 | 1.153 | 0.2397 | Yes |
| 22 | Il2rg | 380 | 1.128 | 0.2455 | Yes |
| 23 | Lcp1 | 416 | 1.084 | 0.2458 | Yes |
| 24 | Tspan7 | 487 | 1.004 | 0.2333 | Yes |
| 25 | Mmp9 | 499 | 0.993 | 0.2407 | Yes |
| 26 | Plvap | 512 | 0.977 | 0.2475 | Yes |
| 27 | Adgra2 | 558 | 0.942 | 0.2429 | Yes |
| 28 | Sparcl1 | 566 | 0.938 | 0.2510 | Yes |
| 29 | Pecam1 | 658 | 0.845 | 0.2296 | Yes |
| 30 | Laptm5 | 659 | 0.844 | 0.2391 | Yes |
| 31 | Gng11 | 662 | 0.840 | 0.2478 | Yes |
| 32 | Irf8 | 663 | 0.840 | 0.2571 | Yes |
| 33 | Ets1 | 671 | 0.831 | 0.2640 | Yes |
| 34 | Adgrl4 | 692 | 0.817 | 0.2664 | Yes |
| 35 | Akap12 | 713 | 0.801 | 0.2686 | Yes |
| 36 | Eng | 733 | 0.776 | 0.2708 | Yes |
| 37 | Gucy1a1 | 759 | 0.752 | 0.2707 | Yes |
| 38 | Prrx1 | 778 | 0.727 | 0.2728 | Yes |
| 39 | Psmb8 | 838 | 0.678 | 0.2604 | No |
| 40 | Cxcr4 | 847 | 0.673 | 0.2652 | No |
| 41 | G0s2 | 878 | 0.645 | 0.2622 | No |
| 42 | Traf1 | 907 | 0.626 | 0.2598 | No |
| 43 | Gprc5b | 927 | 0.612 | 0.2602 | No |
| 44 | Cfh | 1030 | 0.548 | 0.2318 | No |
| 45 | Plau | 1059 | 0.534 | 0.2283 | No |
| 46 | Plaur | 1098 | 0.504 | 0.2211 | No |
| 47 | Mtmr10 | 1182 | -0.515 | 0.1988 | No |
| 48 | Btbd3 | 1191 | -0.517 | 0.2019 | No |
| 49 | Rabgap1l | 1215 | -0.525 | 0.2000 | No |
| 50 | Dnmbp | 1239 | -0.530 | 0.1981 | No |
| 51 | Ano1 | 1324 | -0.547 | 0.1758 | No |
| 52 | Ptcd2 | 1568 | -0.607 | 0.1005 | No |
| 53 | Yrdc | 1600 | -0.616 | 0.0969 | No |
| 54 | Prdm1 | 1634 | -0.628 | 0.0928 | No |
| 55 | Rbm4 | 1734 | -0.666 | 0.0668 | No |
| 56 | Hbegf | 1860 | -0.709 | 0.0325 | No |
| 57 | Ank | 1878 | -0.713 | 0.0347 | No |
| 58 | Slpi | 2011 | -0.766 | -0.0013 | No |
| 59 | Plat | 2156 | -0.832 | -0.0407 | No |
| 60 | Sox9 | 2201 | -0.860 | -0.0460 | No |
| 61 | Cbr4 | 2234 | -0.884 | -0.0469 | No |
| 62 | Ccnd2 | 2242 | -0.887 | -0.0394 | No |
| 63 | Crot | 2333 | -0.945 | -0.0593 | No |
| 64 | Jup | 2337 | -0.950 | -0.0497 | No |
| 65 | Btc | 2354 | -0.969 | -0.0443 | No |
| 66 | Tspan1 | 2419 | -1.017 | -0.0545 | No |
| 67 | Glrx | 2526 | -1.119 | -0.0779 | No |
| 68 | Plek2 | 2648 | -1.279 | -0.1045 | No |
| 69 | Trib2 | 2678 | -1.331 | -0.0994 | No |
| 70 | Mpzl2 | 2710 | -1.388 | -0.0944 | No |
| 71 | Cab39l | 2750 | -1.490 | -0.0910 | No |
| 72 | Igfbp3 | 2753 | -1.492 | -0.0750 | No |
| 73 | Cfb | 2772 | -1.529 | -0.0640 | No |
| 74 | Galnt3 | 2845 | -1.733 | -0.0690 | No |
| 75 | Aldh1a3 | 2868 | -1.845 | -0.0559 | No |
| 76 | Adamdec1 | 2890 | -1.956 | -0.0412 | No |
| 77 | Pigr | 2910 | -2.092 | -0.0242 | No |
| 78 | Tmem158 | 2930 | -2.218 | -0.0059 | No |
| 79 | Cpe | 3023 | -3.802 | 0.0054 | No |
Table: GSEA details [plain text format]

  

Fig 2: HALLMARK\_KRAS\_SIGNALING\_UP: Random ES distribution      
 Gene set null distribution of ES for **HALLMARK\_KRAS\_SIGNALING\_UP**

  
