# Supplementary material for: TMPRSS11B promotes an acidified microenvironment and immune suppression in squamous lung cancer
Source: EMBO Rep. 2025 Nov 10;26(24):6346–79. doi: 10.1038/s44319-025-00631-1 (PMC12714794; doi:10.1038/s44319-025-00631-1)
Supplement: Supplementary file 19 — Appendix Figure S1 Source Data [file 44319_2025_631_MOESM19_ESM.zip › Appendix Figure S1/S1C/GSEA Broad Institute_low pH vs rest of the regions (high pH)_Mh/HALLMARK_MITOTIC_SPINDLE.html]

Details for gene set HALLMARK\_MITOTIC\_SPINDLE[GSEA]

|  || Dataset | Lactate high vs low\_Ranked |
| Phenotype | NoPhenotypeAvailable |
| Upregulated in class | na\_neg |
| GeneSet | HALLMARK\_MITOTIC\_SPINDLE |
| Enrichment Score (ES) | -0.11669238 |
| Normalized Enrichment Score (NES) | -0.5148202 |
| Nominal p-value | 0.9675141 |
| FDR q-value | 0.9740158 |
| FWER p-Value | 1.0 |
Table: GSEA Results Summary

  

Fig 1: Enrichment plot: HALLMARK\_MITOTIC\_SPINDLE      
 Profile of the Running ES Score & Positions of GeneSet Members on the Rank Ordered List

  

| SYMBOL | RANK IN GENE LIST | RANK METRIC SCORE | RUNNING ES | CORE ENRICHMENT || 1 | Dock2 | 229 | 1.333 | -0.0321 | No |
| 2 | Nin | 359 | 1.153 | -0.0368 | No |
| 3 | Prex1 | 435 | 1.066 | -0.0264 | No |
| 4 | Flna | 707 | 0.805 | -0.0900 | Yes |
| 5 | Abr | 764 | 0.747 | -0.0838 | Yes |
| 6 | Cdc42ep2 | 787 | 0.717 | -0.0674 | Yes |
| 7 | Fscn1 | 817 | 0.694 | -0.0540 | Yes |
| 8 | Epb41l2 | 827 | 0.683 | -0.0344 | Yes |
| 9 | Arhgap29 | 840 | 0.678 | -0.0159 | Yes |
| 10 | Arhgef2 | 866 | 0.652 | -0.0026 | Yes |
| 11 | Bcl2l11 | 885 | 0.641 | 0.0127 | Yes |
| 12 | Rapgef5 | 903 | 0.627 | 0.0278 | Yes |
| 13 | Arap3 | 941 | 0.604 | 0.0355 | Yes |
| 14 | Myo9b | 983 | 0.575 | 0.0409 | Yes |
| 15 | Arl8a | 992 | 0.568 | 0.0571 | Yes |
| 16 | Myo1e | 1010 | 0.558 | 0.0700 | Yes |
| 17 | Net1 | 1229 | -0.528 | 0.0148 | Yes |
| 18 | Wasl | 1306 | -0.542 | 0.0075 | Yes |
| 19 | Kif5b | 1372 | -0.558 | 0.0043 | Yes |
| 20 | Arfip2 | 1400 | -0.562 | 0.0140 | Yes |
| 21 | Kif3b | 1532 | -0.595 | -0.0099 | Yes |
| 22 | Nck2 | 1589 | -0.613 | -0.0082 | Yes |
| 23 | Rapgef6 | 1614 | -0.620 | 0.0043 | Yes |
| 24 | Stk38l | 1697 | -0.652 | -0.0013 | Yes |
| 25 | Arfgef1 | 1700 | -0.653 | 0.0196 | Yes |
| 26 | Cttn | 1883 | -0.716 | -0.0172 | Yes |
| 27 | Katna1 | 1911 | -0.726 | -0.0021 | Yes |
| 28 | Rabgap1 | 1988 | -0.755 | -0.0024 | Yes |
| 29 | Dst | 2035 | -0.777 | 0.0080 | Yes |
| 30 | Pcgf5 | 2083 | -0.801 | 0.0189 | Yes |
| 31 | Cd2ap | 2149 | -0.827 | 0.0247 | Yes |
| 32 | Flnb | 2221 | -0.871 | 0.0300 | Yes |
| 33 | Ezr | 2297 | -0.926 | 0.0357 | Yes |
| 34 | Tiam1 | 2352 | -0.967 | 0.0498 | Yes |
| 35 | Palld | 2424 | -1.027 | 0.0602 | Yes |
| 36 | Kif23 | 2458 | -1.058 | 0.0843 | Yes |
| 37 | Rasa2 | 2689 | -1.349 | 0.0524 | Yes |
| 38 | Sorbs2 | 2886 | -1.925 | 0.0510 | Yes |
Table: GSEA details [plain text format]

  

Fig 2: HALLMARK\_MITOTIC\_SPINDLE: Random ES distribution      
 Gene set null distribution of ES for **HALLMARK\_MITOTIC\_SPINDLE**

  
