# Supplementary material for: TMPRSS11B promotes an acidified microenvironment and immune suppression in squamous lung cancer
Source: EMBO Rep. 2025 Nov 10;26(24):6346–79. doi: 10.1038/s44319-025-00631-1 (PMC12714794; doi:10.1038/s44319-025-00631-1)
Supplement: Supplementary file 19 — Appendix Figure S1 Source Data [file 44319_2025_631_MOESM19_ESM.zip › Appendix Figure S1/S1C/GSEA Broad Institute_low pH vs rest of the regions (high pH)_Mh/HALLMARK_MTORC1_SIGNALING.html]

Details for gene set HALLMARK\_MTORC1\_SIGNALING[GSEA]

|  || Dataset | Lactate high vs low\_Ranked |
| Phenotype | NoPhenotypeAvailable |
| Upregulated in class | na\_neg |
| GeneSet | HALLMARK\_MTORC1\_SIGNALING |
| Enrichment Score (ES) | -0.1618196 |
| Normalized Enrichment Score (NES) | -0.75240463 |
| Nominal p-value | 0.79038715 |
| FDR q-value | 0.987149 |
| FWER p-Value | 1.0 |
Table: GSEA Results Summary

  

Fig 1: Enrichment plot: HALLMARK\_MTORC1\_SIGNALING      
 Profile of the Running ES Score & Positions of GeneSet Members on the Rank Ordered List

  

| SYMBOL | RANK IN GENE LIST | RANK METRIC SCORE | RUNNING ES | CORE ENRICHMENT || 1 | Lgmn | 28 | 1.878 | 0.0329 | No |
| 2 | Itgb2 | 138 | 1.498 | 0.0303 | No |
| 3 | Cfp | 168 | 1.424 | 0.0526 | No |
| 4 | Gla | 181 | 1.403 | 0.0802 | No |
| 5 | Sla | 256 | 1.280 | 0.0843 | No |
| 6 | Fgl2 | 428 | 1.073 | 0.0514 | No |
| 7 | Coro1a | 522 | 0.970 | 0.0421 | No |
| 8 | Ctsc | 525 | 0.966 | 0.0632 | No |
| 9 | Serpinh1 | 546 | 0.953 | 0.0780 | No |
| 10 | Actr3 | 665 | 0.838 | 0.0575 | No |
| 11 | P4ha1 | 693 | 0.817 | 0.0669 | No |
| 12 | Gga2 | 747 | 0.766 | 0.0664 | No |
| 13 | Cdkn1a | 749 | 0.765 | 0.0833 | No |
| 14 | Ccng1 | 823 | 0.687 | 0.0744 | No |
| 15 | Cxcr4 | 847 | 0.673 | 0.0818 | No |
| 16 | Hmgcr | 1281 | -0.538 | -0.0507 | No |
| 17 | Pdk1 | 1331 | -0.549 | -0.0547 | No |
| 18 | Stip1 | 1374 | -0.558 | -0.0562 | No |
| 19 | Shmt2 | 1410 | -0.565 | -0.0551 | No |
| 20 | Pno1 | 1423 | -0.568 | -0.0463 | No |
| 21 | Btg2 | 1440 | -0.573 | -0.0388 | No |
| 22 | Pgk1 | 1680 | -0.642 | -0.1042 | No |
| 23 | Dapp1 | 1681 | -0.643 | -0.0897 | No |
| 24 | Ppa1 | 1719 | -0.661 | -0.0872 | No |
| 25 | Hspa9 | 1743 | -0.670 | -0.0798 | No |
| 26 | Rpn1 | 1925 | -0.730 | -0.1238 | No |
| 27 | G6pdx | 1934 | -0.733 | -0.1099 | No |
| 28 | Xbp1 | 1976 | -0.750 | -0.1067 | No |
| 29 | Cyp51 | 2098 | -0.806 | -0.1290 | No |
| 30 | Arpc5l | 2141 | -0.824 | -0.1245 | No |
| 31 | Hmgcs1 | 2251 | -0.894 | -0.1408 | No |
| 32 | Psph | 2315 | -0.934 | -0.1408 | Yes |
| 33 | Dhcr24 | 2316 | -0.935 | -0.1197 | Yes |
| 34 | Cdc25a | 2319 | -0.935 | -0.0993 | Yes |
| 35 | Slc1a5 | 2375 | -0.989 | -0.0954 | Yes |
| 36 | Atp2a2 | 2399 | -1.006 | -0.0805 | Yes |
| 37 | Sc5d | 2421 | -1.024 | -0.0644 | Yes |
| 38 | Slc7a5 | 2465 | -1.061 | -0.0549 | Yes |
| 39 | Acsl3 | 2510 | -1.103 | -0.0447 | Yes |
| 40 | Glrx | 2526 | -1.119 | -0.0246 | Yes |
| 41 | Igfbp5 | 2556 | -1.159 | -0.0082 | Yes |
| 42 | Nupr1 | 2565 | -1.167 | 0.0155 | Yes |
| 43 | Sqle | 2622 | -1.234 | 0.0245 | Yes |
| 44 | Asns | 2703 | -1.371 | 0.0287 | Yes |
| 45 | Psat1 | 2790 | -1.568 | 0.0353 | Yes |
| 46 | Srd5a1 | 2916 | -2.112 | 0.0411 | Yes |
Table: GSEA details [plain text format]

  

Fig 2: HALLMARK\_MTORC1\_SIGNALING: Random ES distribution      
 Gene set null distribution of ES for **HALLMARK\_MTORC1\_SIGNALING**

  
