# Supplementary material for: TMPRSS11B promotes an acidified microenvironment and immune suppression in squamous lung cancer
Source: EMBO Rep. 2025 Nov 10;26(24):6346–79. doi: 10.1038/s44319-025-00631-1 (PMC12714794; doi:10.1038/s44319-025-00631-1)
Supplement: Supplementary file 19 — Appendix Figure S1 Source Data [file 44319_2025_631_MOESM19_ESM.zip › Appendix Figure S1/S1C/GSEA Broad Institute_low pH vs rest of the regions (high pH)_Mh/HALLMARK_MYC_TARGETS_V1.html]

Details for gene set HALLMARK\_MYC\_TARGETS\_V1[GSEA]

|  || Dataset | Lactate high vs low\_Ranked |
| Phenotype | NoPhenotypeAvailable |
| Upregulated in class | na\_neg |
| GeneSet | HALLMARK\_MYC\_TARGETS\_V1 |
| Enrichment Score (ES) | -0.36345515 |
| Normalized Enrichment Score (NES) | -1.5240096 |
| Nominal p-value | 0.05087209 |
| FDR q-value | 0.20019151 |
| FWER p-Value | 0.775 |
Table: GSEA Results Summary

  

Fig 1: Enrichment plot: HALLMARK\_MYC\_TARGETS\_V1      
 Profile of the Running ES Score & Positions of GeneSet Members on the Rank Ordered List

  

| SYMBOL | RANK IN GENE LIST | RANK METRIC SCORE | RUNNING ES | CORE ENRICHMENT || 1 | Ccna2 | 1094 | 0.505 | -0.3392 | Yes |
| 2 | Cstf2 | 1113 | -0.502 | -0.3211 | Yes |
| 3 | Ddx18 | 1139 | -0.507 | -0.3051 | Yes |
| 4 | Fbl | 1188 | -0.517 | -0.2963 | Yes |
| 5 | Rack1 | 1195 | -0.519 | -0.2733 | Yes |
| 6 | Got2 | 1317 | -0.545 | -0.2874 | Yes |
| 7 | Snrpa1 | 1335 | -0.549 | -0.2667 | Yes |
| 8 | Eef1b2 | 1352 | -0.553 | -0.2455 | Yes |
| 9 | Acp1 | 1433 | -0.572 | -0.2446 | Yes |
| 10 | Glo1 | 1573 | -0.608 | -0.2616 | Yes |
| 11 | Aimp2 | 1605 | -0.617 | -0.2423 | Yes |
| 12 | Nhp2 | 1637 | -0.629 | -0.2224 | Yes |
| 13 | Ppm1g | 1660 | -0.637 | -0.1992 | Yes |
| 14 | Pgk1 | 1680 | -0.642 | -0.1747 | Yes |
| 15 | Vdac3 | 1705 | -0.657 | -0.1512 | Yes |
| 16 | Rsl1d1 | 1733 | -0.666 | -0.1282 | Yes |
| 17 | Nop16 | 1748 | -0.672 | -0.1006 | Yes |
| 18 | Smarcc1 | 1816 | -0.695 | -0.0896 | Yes |
| 19 | Nop56 | 1822 | -0.696 | -0.0578 | Yes |
| 20 | Ruvbl2 | 1892 | -0.719 | -0.0462 | Yes |
| 21 | Nap1l1 | 1905 | -0.723 | -0.0155 | Yes |
| 22 | Tyms | 1908 | -0.724 | 0.0185 | Yes |
| 23 | Hnrnpc | 1958 | -0.742 | 0.0379 | Yes |
| 24 | Hdac2 | 2053 | -0.786 | 0.0444 | Yes |
| 25 | Tfdp1 | 2061 | -0.789 | 0.0799 | Yes |
| 26 | Apex1 | 2125 | -0.816 | 0.0981 | Yes |
| 27 | Pold2 | 2301 | -0.928 | 0.0845 | Yes |
| 28 | G3bp1 | 2307 | -0.930 | 0.1275 | Yes |
| 29 | Srsf3 | 2496 | -1.090 | 0.1173 | Yes |
| 30 | Cad | 2664 | -1.309 | 0.1246 | Yes |
Table: GSEA details [plain text format]

  

Fig 2: HALLMARK\_MYC\_TARGETS\_V1: Random ES distribution      
 Gene set null distribution of ES for **HALLMARK\_MYC\_TARGETS\_V1**

  
