# Supplementary material for: TMPRSS11B promotes an acidified microenvironment and immune suppression in squamous lung cancer
Source: EMBO Rep. 2025 Nov 10;26(24):6346–79. doi: 10.1038/s44319-025-00631-1 (PMC12714794; doi:10.1038/s44319-025-00631-1)
Supplement: Supplementary file 19 — Appendix Figure S1 Source Data [file 44319_2025_631_MOESM19_ESM.zip › Appendix Figure S1/S1C/GSEA Broad Institute_low pH vs rest of the regions (high pH)_Mh/HALLMARK_MYC_TARGETS_V2.html]

Details for gene set HALLMARK\_MYC\_TARGETS\_V2[GSEA]

|  || Dataset | Lactate high vs low\_Ranked |
| Phenotype | NoPhenotypeAvailable |
| Upregulated in class | na\_neg |
| GeneSet | HALLMARK\_MYC\_TARGETS\_V2 |
| Enrichment Score (ES) | -0.3012629 |
| Normalized Enrichment Score (NES) | -1.0230021 |
| Nominal p-value | 0.42901236 |
| FDR q-value | 0.7905485 |
| FWER p-Value | 1.0 |
Table: GSEA Results Summary

  

Fig 1: Enrichment plot: HALLMARK\_MYC\_TARGETS\_V2      
 Profile of the Running ES Score & Positions of GeneSet Members on the Rank Ordered List

  

| SYMBOL | RANK IN GENE LIST | RANK METRIC SCORE | RUNNING ES | CORE ENRICHMENT || 1 | Map3k6 | 627 | 0.869 | -0.1323 | No |
| 2 | Ddx18 | 1139 | -0.507 | -0.2575 | Yes |
| 3 | Wdr43 | 1214 | -0.524 | -0.2366 | Yes |
| 4 | Tfb2m | 1293 | -0.539 | -0.2158 | Yes |
| 5 | Nip7 | 1334 | -0.549 | -0.1816 | Yes |
| 6 | Bysl | 1437 | -0.572 | -0.1659 | Yes |
| 7 | Pus1 | 1450 | -0.575 | -0.1202 | Yes |
| 8 | Aimp2 | 1605 | -0.617 | -0.1178 | Yes |
| 9 | Nop2 | 1663 | -0.637 | -0.0816 | Yes |
| 10 | Nop16 | 1748 | -0.672 | -0.0513 | Yes |
| 11 | Nop56 | 1822 | -0.696 | -0.0153 | Yes |
| 12 | Mphosph10 | 1889 | -0.719 | 0.0250 | Yes |
| 13 | Ppan | 2047 | -0.784 | 0.0409 | Yes |
| 14 | Pprc1 | 2211 | -0.865 | 0.0617 | Yes |
| 15 | Las1l | 2604 | -1.217 | 0.0372 | Yes |
| 16 | Wdr74 | 2617 | -1.230 | 0.1396 | Yes |
Table: GSEA details [plain text format]

  

Fig 2: HALLMARK\_MYC\_TARGETS\_V2: Random ES distribution      
 Gene set null distribution of ES for **HALLMARK\_MYC\_TARGETS\_V2**

  
