# Supplementary material for: TMPRSS11B promotes an acidified microenvironment and immune suppression in squamous lung cancer
Source: EMBO Rep. 2025 Nov 10;26(24):6346–79. doi: 10.1038/s44319-025-00631-1 (PMC12714794; doi:10.1038/s44319-025-00631-1)
Supplement: Supplementary file 19 — Appendix Figure S1 Source Data [file 44319_2025_631_MOESM19_ESM.zip › Appendix Figure S1/S1C/GSEA Broad Institute_low pH vs rest of the regions (high pH)_Mh/HALLMARK_MYOGENESIS.html]

Details for gene set HALLMARK\_MYOGENESIS[GSEA]

|  || Dataset | Lactate high vs low\_Ranked |
| Phenotype | NoPhenotypeAvailable |
| Upregulated in class | na\_pos |
| GeneSet | HALLMARK\_MYOGENESIS |
| Enrichment Score (ES) | 0.21209669 |
| Normalized Enrichment Score (NES) | 1.2132454 |
| Nominal p-value | 0.20754717 |
| FDR q-value | 0.3428826 |
| FWER p-Value | 0.909 |
Table: GSEA Results Summary

  

Fig 1: Enrichment plot: HALLMARK\_MYOGENESIS      
 Profile of the Running ES Score & Positions of GeneSet Members on the Rank Ordered List

  

| SYMBOL | RANK IN GENE LIST | RANK METRIC SCORE | RUNNING ES | CORE ENRICHMENT || 1 | Cd36 | 52 | 1.748 | 0.0167 | Yes |
| 2 | Igf1 | 60 | 1.691 | 0.0473 | Yes |
| 3 | Speg | 82 | 1.620 | 0.0718 | Yes |
| 4 | Cryab | 158 | 1.449 | 0.0750 | Yes |
| 5 | Col6a3 | 175 | 1.408 | 0.0971 | Yes |
| 6 | Col6a2 | 237 | 1.316 | 0.1023 | Yes |
| 7 | Col15a1 | 289 | 1.234 | 0.1093 | Yes |
| 8 | Col4a2 | 331 | 1.182 | 0.1187 | Yes |
| 9 | Ckb | 422 | 1.078 | 0.1096 | Yes |
| 10 | Lsp1 | 459 | 1.039 | 0.1178 | Yes |
| 11 | Sparc | 460 | 1.038 | 0.1380 | Yes |
| 12 | Psen2 | 474 | 1.024 | 0.1536 | Yes |
| 13 | Col3a1 | 501 | 0.992 | 0.1643 | Yes |
| 14 | Mylk | 588 | 0.905 | 0.1532 | Yes |
| 15 | Col1a1 | 643 | 0.856 | 0.1518 | Yes |
| 16 | Ptp4a3 | 644 | 0.854 | 0.1685 | Yes |
| 17 | Tgfb1 | 664 | 0.839 | 0.1785 | Yes |
| 18 | Fhl1 | 688 | 0.820 | 0.1868 | Yes |
| 19 | Cdkn1a | 749 | 0.765 | 0.1816 | Yes |
| 20 | Sod3 | 806 | 0.702 | 0.1766 | Yes |
| 21 | Prnp | 820 | 0.690 | 0.1857 | Yes |
| 22 | Mras | 843 | 0.676 | 0.1915 | Yes |
| 23 | Dapk2 | 858 | 0.658 | 0.1996 | Yes |
| 24 | Igfbp7 | 860 | 0.655 | 0.2121 | Yes |
| 25 | Itga7 | 899 | 0.630 | 0.2116 | Yes |
| 26 | Lpin1 | 934 | 0.607 | 0.2121 | Yes |
| 27 | Fgf2 | 1014 | 0.555 | 0.1965 | No |
| 28 | Slc6a8 | 1041 | 0.543 | 0.1984 | No |
| 29 | Gpx3 | 1070 | 0.527 | 0.1993 | No |
| 30 | Mapk12 | 1154 | -0.510 | 0.1815 | No |
| 31 | Erbb3 | 1223 | -0.527 | 0.1690 | No |
| 32 | Bag1 | 1264 | -0.534 | 0.1660 | No |
| 33 | Cnn3 | 1470 | -0.579 | 0.1088 | No |
| 34 | Pde4dip | 1538 | -0.597 | 0.0980 | No |
| 35 | Foxo4 | 1653 | -0.633 | 0.0722 | No |
| 36 | Hbegf | 1860 | -0.709 | 0.0172 | No |
| 37 | Pick1 | 2025 | -0.771 | -0.0226 | No |
| 38 | Tpm2 | 2092 | -0.804 | -0.0290 | No |
| 39 | Itgb4 | 2445 | -1.047 | -0.1263 | No |
| 40 | Hspb8 | 2503 | -1.097 | -0.1240 | No |
| 41 | Acsl1 | 2687 | -1.346 | -0.1589 | No |
| 42 | Igfbp3 | 2753 | -1.492 | -0.1516 | No |
| 43 | Spdef | 2763 | -1.514 | -0.1251 | No |
| 44 | Ephb3 | 2765 | -1.522 | -0.0958 | No |
| 45 | Klf5 | 2823 | -1.644 | -0.0828 | No |
| 46 | Cdh13 | 2854 | -1.759 | -0.0585 | No |
| 47 | Clu | 2876 | -1.874 | -0.0290 | No |
| 48 | Reep1 | 2914 | -2.104 | -0.0004 | No |
| 49 | Stc2 | 2919 | -2.148 | 0.0401 | No |
Table: GSEA details [plain text format]

  

Fig 2: HALLMARK\_MYOGENESIS: Random ES distribution      
 Gene set null distribution of ES for **HALLMARK\_MYOGENESIS**

  
