# Supplementary material for: TMPRSS11B promotes an acidified microenvironment and immune suppression in squamous lung cancer
Source: EMBO Rep. 2025 Nov 10;26(24):6346–79. doi: 10.1038/s44319-025-00631-1 (PMC12714794; doi:10.1038/s44319-025-00631-1)
Supplement: Supplementary file 19 — Appendix Figure S1 Source Data [file 44319_2025_631_MOESM19_ESM.zip › Appendix Figure S1/S1C/GSEA Broad Institute_low pH vs rest of the regions (high pH)_Mh/HALLMARK_OXIDATIVE_PHOSPHORYLATION.html]

Details for gene set HALLMARK\_OXIDATIVE\_PHOSPHORYLATION[GSEA]

|  || Dataset | Lactate high vs low\_Ranked |
| Phenotype | NoPhenotypeAvailable |
| Upregulated in class | na\_neg |
| GeneSet | HALLMARK\_OXIDATIVE\_PHOSPHORYLATION |
| Enrichment Score (ES) | -0.20163988 |
| Normalized Enrichment Score (NES) | -0.83126795 |
| Nominal p-value | 0.66618496 |
| FDR q-value | 0.91833717 |
| FWER p-Value | 1.0 |
Table: GSEA Results Summary

  

Fig 1: Enrichment plot: HALLMARK\_OXIDATIVE\_PHOSPHORYLATION      
 Profile of the Running ES Score & Positions of GeneSet Members on the Rank Ordered List

  

| SYMBOL | RANK IN GENE LIST | RANK METRIC SCORE | RUNNING ES | CORE ENRICHMENT || 1 | Tcirg1 | 509 | 0.983 | -0.1224 | No |
| 2 | Atp6v1c1 | 524 | 0.967 | -0.0810 | No |
| 3 | Atp6v0e | 888 | 0.638 | -0.1713 | Yes |
| 4 | Iscu | 905 | 0.626 | -0.1468 | Yes |
| 5 | Atp6v0c | 960 | 0.595 | -0.1365 | Yes |
| 6 | Atp6v1g1 | 1020 | 0.553 | -0.1298 | Yes |
| 7 | Idh3g | 1205 | -0.521 | -0.1662 | Yes |
| 8 | Idh3b | 1206 | -0.521 | -0.1414 | Yes |
| 9 | Ndufs2 | 1297 | -0.540 | -0.1456 | Yes |
| 10 | Got2 | 1317 | -0.545 | -0.1260 | Yes |
| 11 | Surf1 | 1396 | -0.562 | -0.1252 | Yes |
| 12 | Idh3a | 1408 | -0.565 | -0.1020 | Yes |
| 13 | Pdhx | 1453 | -0.575 | -0.0893 | Yes |
| 14 | Phyh | 1502 | -0.587 | -0.0773 | Yes |
| 15 | Ndufa9 | 1524 | -0.592 | -0.0561 | Yes |
| 16 | Casp7 | 1587 | -0.613 | -0.0476 | Yes |
| 17 | Ndufv2 | 1610 | -0.618 | -0.0255 | Yes |
| 18 | Vdac3 | 1705 | -0.657 | -0.0255 | Yes |
| 19 | Retsat | 1742 | -0.669 | -0.0057 | Yes |
| 20 | Hspa9 | 1743 | -0.670 | 0.0262 | Yes |
| 21 | Acat1 | 1859 | -0.709 | 0.0216 | Yes |
| 22 | Pmpca | 1863 | -0.710 | 0.0544 | Yes |
| 23 | Eci1 | 1871 | -0.711 | 0.0859 | Yes |
| 24 | Fh1 | 1923 | -0.730 | 0.1036 | Yes |
| 25 | Acadm | 1975 | -0.750 | 0.1223 | Yes |
| 26 | Sucla2 | 1992 | -0.757 | 0.1530 | Yes |
| 27 | Atp1b1 | 2139 | -0.823 | 0.1436 | Yes |
| 28 | Por | 2395 | -1.005 | 0.1067 | Yes |
| 29 | Timm9 | 2416 | -1.016 | 0.1484 | Yes |
| 30 | Oat | 2615 | -1.226 | 0.1409 | Yes |
Table: GSEA details [plain text format]

  

Fig 2: HALLMARK\_OXIDATIVE\_PHOSPHORYLATION: Random ES distribution      
 Gene set null distribution of ES for **HALLMARK\_OXIDATIVE\_PHOSPHORYLATION**

  
