# Supplementary material for: TMPRSS11B promotes an acidified microenvironment and immune suppression in squamous lung cancer
Source: EMBO Rep. 2025 Nov 10;26(24):6346–79. doi: 10.1038/s44319-025-00631-1 (PMC12714794; doi:10.1038/s44319-025-00631-1)
Supplement: Supplementary file 19 — Appendix Figure S1 Source Data [file 44319_2025_631_MOESM19_ESM.zip › Appendix Figure S1/S1C/GSEA Broad Institute_low pH vs rest of the regions (high pH)_Mh/HALLMARK_P53_PATHWAY.html]

Details for gene set HALLMARK\_P53\_PATHWAY[GSEA]

|  || Dataset | Lactate high vs low\_Ranked |
| Phenotype | NoPhenotypeAvailable |
| Upregulated in class | na\_neg |
| GeneSet | HALLMARK\_P53\_PATHWAY |
| Enrichment Score (ES) | -0.14459942 |
| Normalized Enrichment Score (NES) | -0.7015693 |
| Nominal p-value | 0.8530997 |
| FDR q-value | 0.93943524 |
| FWER p-Value | 1.0 |
Table: GSEA Results Summary

  

Fig 1: Enrichment plot: HALLMARK\_P53\_PATHWAY      
 Profile of the Running ES Score & Positions of GeneSet Members on the Rank Ordered List

  

| SYMBOL | RANK IN GENE LIST | RANK METRIC SCORE | RUNNING ES | CORE ENRICHMENT || 1 | Hmox1 | 16 | 2.051 | 0.0317 | No |
| 2 | Ctsd | 136 | 1.507 | 0.0190 | No |
| 3 | Atf3 | 161 | 1.444 | 0.0371 | No |
| 4 | Abcc5 | 162 | 1.441 | 0.0631 | No |
| 5 | Steap3 | 262 | 1.267 | 0.0528 | No |
| 6 | Dram1 | 263 | 1.266 | 0.0757 | No |
| 7 | Gm2a | 274 | 1.259 | 0.0950 | No |
| 8 | Zbtb16 | 311 | 1.211 | 0.1048 | No |
| 9 | Rap2b | 338 | 1.178 | 0.1174 | No |
| 10 | Fas | 457 | 1.039 | 0.0967 | No |
| 11 | Ptpre | 623 | 0.871 | 0.0571 | No |
| 12 | Tgfb1 | 664 | 0.839 | 0.0589 | No |
| 13 | Cdkn1a | 749 | 0.765 | 0.0445 | No |
| 14 | Ccng1 | 823 | 0.687 | 0.0325 | No |
| 15 | Iscu | 905 | 0.626 | 0.0167 | No |
| 16 | Rhbdf2 | 922 | 0.616 | 0.0224 | No |
| 17 | Cebpa | 923 | 0.615 | 0.0335 | No |
| 18 | S100a10 | 965 | 0.592 | 0.0305 | No |
| 19 | F2r | 1002 | 0.562 | 0.0286 | No |
| 20 | Ninj1 | 1025 | 0.551 | 0.0312 | No |
| 21 | Stom | 1085 | 0.517 | 0.0207 | No |
| 22 | Osgin1 | 1138 | -0.506 | 0.0125 | No |
| 23 | Rack1 | 1195 | -0.519 | 0.0031 | No |
| 24 | Ddb2 | 1209 | -0.523 | 0.0082 | No |
| 25 | Sdc1 | 1228 | -0.527 | 0.0117 | No |
| 26 | Fam162a | 1258 | -0.533 | 0.0116 | No |
| 27 | Tax1bp3 | 1383 | -0.560 | -0.0199 | No |
| 28 | Btg2 | 1440 | -0.573 | -0.0283 | No |
| 29 | Tcn2 | 1491 | -0.583 | -0.0345 | No |
| 30 | Retsat | 1742 | -0.669 | -0.1061 | No |
| 31 | Eps8l2 | 1793 | -0.685 | -0.1105 | No |
| 32 | Xpc | 1812 | -0.692 | -0.1041 | No |
| 33 | Dgka | 1813 | -0.694 | -0.0915 | No |
| 34 | Hbegf | 1860 | -0.709 | -0.0941 | No |
| 35 | Pmm1 | 1922 | -0.729 | -0.1014 | No |
| 36 | Tob1 | 1942 | -0.737 | -0.0945 | No |
| 37 | Epha2 | 1997 | -0.758 | -0.0989 | No |
| 38 | Mxd1 | 2005 | -0.763 | -0.0874 | No |
| 39 | Hexim1 | 2163 | -0.842 | -0.1248 | No |
| 40 | Cdkn2a | 2223 | -0.874 | -0.1288 | Yes |
| 41 | Ptpn14 | 2228 | -0.878 | -0.1143 | Yes |
| 42 | Ccnd2 | 2242 | -0.887 | -0.1027 | Yes |
| 43 | Pom121 | 2253 | -0.895 | -0.0899 | Yes |
| 44 | Upp1 | 2304 | -0.929 | -0.0898 | Yes |
| 45 | Slc35d1 | 2339 | -0.951 | -0.0840 | Yes |
| 46 | Dcxr | 2434 | -1.037 | -0.0968 | Yes |
| 47 | Itgb4 | 2445 | -1.047 | -0.0812 | Yes |
| 48 | Fos | 2491 | -1.083 | -0.0768 | Yes |
| 49 | Nupr1 | 2565 | -1.167 | -0.0801 | Yes |
| 50 | Gpx2 | 2598 | -1.211 | -0.0690 | Yes |
| 51 | St14 | 2628 | -1.245 | -0.0562 | Yes |
| 52 | Prkab1 | 2736 | -1.451 | -0.0659 | Yes |
| 53 | Cdh13 | 2854 | -1.759 | -0.0733 | Yes |
| 54 | Krt17 | 3006 | -3.210 | -0.0659 | Yes |
| 55 | Trp63 | 3034 | -4.244 | 0.0017 | Yes |
Table: GSEA details [plain text format]

  

Fig 2: HALLMARK\_P53\_PATHWAY: Random ES distribution      
 Gene set null distribution of ES for **HALLMARK\_P53\_PATHWAY**

  
