# Supplementary material for: TMPRSS11B promotes an acidified microenvironment and immune suppression in squamous lung cancer
Source: EMBO Rep. 2025 Nov 10;26(24):6346–79. doi: 10.1038/s44319-025-00631-1 (PMC12714794; doi:10.1038/s44319-025-00631-1)
Supplement: Supplementary file 19 — Appendix Figure S1 Source Data [file 44319_2025_631_MOESM19_ESM.zip › Appendix Figure S1/S1C/GSEA Broad Institute_low pH vs rest of the regions (high pH)_Mh/HALLMARK_PI3K_AKT_MTOR_SIGNALING.html]

Details for gene set HALLMARK\_PI3K\_AKT\_MTOR\_SIGNALING[GSEA]

|  || Dataset | Lactate high vs low\_Ranked |
| Phenotype | NoPhenotypeAvailable |
| Upregulated in class | na\_pos |
| GeneSet | HALLMARK\_PI3K\_AKT\_MTOR\_SIGNALING |
| Enrichment Score (ES) | 0.1645873 |
| Normalized Enrichment Score (NES) | 0.7242108 |
| Nominal p-value | 0.83076924 |
| FDR q-value | 1.0 |
| FWER p-Value | 1.0 |
Table: GSEA Results Summary

  

Fig 1: Enrichment plot: HALLMARK\_PI3K\_AKT\_MTOR\_SIGNALING      
 Profile of the Running ES Score & Positions of GeneSet Members on the Rank Ordered List

  

| SYMBOL | RANK IN GENE LIST | RANK METRIC SCORE | RUNNING ES | CORE ENRICHMENT || 1 | Dusp3 | 244 | 1.299 | -0.0173 | Yes |
| 2 | Sla | 256 | 1.280 | 0.0416 | Yes |
| 3 | Prkcb | 268 | 1.263 | 0.0997 | Yes |
| 4 | Il2rg | 380 | 1.128 | 0.1181 | Yes |
| 5 | Actr3 | 665 | 0.838 | 0.0650 | Yes |
| 6 | Cdkn1a | 749 | 0.765 | 0.0749 | Yes |
| 7 | Plcb1 | 760 | 0.752 | 0.1083 | Yes |
| 8 | Grb2 | 816 | 0.695 | 0.1241 | Yes |
| 9 | Cxcr4 | 847 | 0.673 | 0.1471 | Yes |
| 10 | Mknk1 | 921 | 0.617 | 0.1531 | Yes |
| 11 | Cfl1 | 973 | 0.581 | 0.1646 | Yes |
| 12 | Egfr | 1252 | -0.532 | 0.0985 | No |
| 13 | Pdk1 | 1331 | -0.549 | 0.0995 | No |
| 14 | Rac1 | 1404 | -0.564 | 0.1032 | No |
| 15 | Dapp1 | 1681 | -0.643 | 0.0432 | No |
| 16 | Mapkap1 | 2256 | -0.896 | -0.1032 | No |
| 17 | Pak4 | 2266 | -0.904 | -0.0620 | No |
| 18 | Ppp2r1b | 2268 | -0.905 | -0.0180 | No |
| 19 | Tiam1 | 2352 | -0.967 | 0.0018 | No |
| 20 | Prkag1 | 2400 | -1.006 | 0.0354 | No |
| 21 | Cab39l | 2750 | -1.490 | -0.0074 | No |
| 22 | Gna14 | 2911 | -2.102 | 0.0424 | No |
Table: GSEA details [plain text format]

  

Fig 2: HALLMARK\_PI3K\_AKT\_MTOR\_SIGNALING: Random ES distribution      
 Gene set null distribution of ES for **HALLMARK\_PI3K\_AKT\_MTOR\_SIGNALING**

  
