# Supplementary material for: TMPRSS11B promotes an acidified microenvironment and immune suppression in squamous lung cancer
Source: EMBO Rep. 2025 Nov 10;26(24):6346–79. doi: 10.1038/s44319-025-00631-1 (PMC12714794; doi:10.1038/s44319-025-00631-1)
Supplement: Supplementary file 19 — Appendix Figure S1 Source Data [file 44319_2025_631_MOESM19_ESM.zip › Appendix Figure S1/S1C/GSEA Broad Institute_low pH vs rest of the regions (high pH)_Mh/HALLMARK_TNFA_SIGNALING_VIA_NFKB.html]

Details for gene set HALLMARK\_TNFA\_SIGNALING\_VIA\_NFKB[GSEA]

|  || Dataset | Lactate high vs low\_Ranked |
| Phenotype | NoPhenotypeAvailable |
| Upregulated in class | na\_pos |
| GeneSet | HALLMARK\_TNFA\_SIGNALING\_VIA\_NFKB |
| Enrichment Score (ES) | 0.11274885 |
| Normalized Enrichment Score (NES) | 0.68078977 |
| Nominal p-value | 0.908 |
| FDR q-value | 1.0 |
| FWER p-Value | 1.0 |
Table: GSEA Results Summary

  

Fig 1: Enrichment plot: HALLMARK\_TNFA\_SIGNALING\_VIA\_NFKB      
 Profile of the Running ES Score & Positions of GeneSet Members on the Rank Ordered List

  

| SYMBOL | RANK IN GENE LIST | RANK METRIC SCORE | RUNNING ES | CORE ENRICHMENT || 1 | Cd83 | 101 | 1.575 | -0.0016 | Yes |
| 2 | Plek | 135 | 1.509 | 0.0182 | Yes |
| 3 | Atf3 | 161 | 1.444 | 0.0394 | Yes |
| 4 | Abca1 | 206 | 1.367 | 0.0527 | Yes |
| 5 | Dram1 | 263 | 1.266 | 0.0598 | Yes |
| 6 | B4galt5 | 385 | 1.117 | 0.0422 | Yes |
| 7 | Klf2 | 412 | 1.087 | 0.0557 | Yes |
| 8 | Icam1 | 492 | 0.999 | 0.0497 | Yes |
| 9 | Dennd5a | 521 | 0.973 | 0.0603 | Yes |
| 10 | Snn | 534 | 0.960 | 0.0759 | Yes |
| 11 | Dusp1 | 569 | 0.935 | 0.0836 | Yes |
| 12 | Ptpre | 623 | 0.871 | 0.0837 | Yes |
| 13 | Vegfa | 650 | 0.852 | 0.0924 | Yes |
| 14 | Slc16a6 | 668 | 0.834 | 0.1038 | Yes |
| 15 | Cdkn1a | 749 | 0.765 | 0.0927 | Yes |
| 16 | Cebpb | 773 | 0.736 | 0.1000 | Yes |
| 17 | Mcl1 | 861 | 0.655 | 0.0843 | Yes |
| 18 | G0s2 | 878 | 0.645 | 0.0921 | Yes |
| 19 | Traf1 | 907 | 0.626 | 0.0956 | Yes |
| 20 | Rcan1 | 920 | 0.617 | 0.1042 | Yes |
| 21 | Plpp3 | 938 | 0.605 | 0.1109 | Yes |
| 22 | Ninj1 | 1025 | 0.551 | 0.0933 | Yes |
| 23 | Plau | 1059 | 0.534 | 0.0932 | Yes |
| 24 | Cd44 | 1072 | 0.523 | 0.0999 | Yes |
| 25 | Litaf | 1084 | 0.517 | 0.1068 | Yes |
| 26 | Plaur | 1098 | 0.504 | 0.1127 | Yes |
| 27 | Tgif1 | 1220 | -0.526 | 0.0830 | No |
| 28 | Maff | 1259 | -0.533 | 0.0812 | No |
| 29 | Gch1 | 1277 | -0.537 | 0.0865 | No |
| 30 | Fosl2 | 1283 | -0.538 | 0.0958 | No |
| 31 | Pmepa1 | 1426 | -0.570 | 0.0599 | No |
| 32 | Btg2 | 1440 | -0.573 | 0.0673 | No |
| 33 | Irs2 | 1443 | -0.574 | 0.0784 | No |
| 34 | Ier2 | 1527 | -0.593 | 0.0627 | No |
| 35 | Yrdc | 1600 | -0.616 | 0.0512 | No |
| 36 | Nr4a1 | 1632 | -0.627 | 0.0536 | No |
| 37 | Trip10 | 1641 | -0.630 | 0.0639 | No |
| 38 | Bcl3 | 1709 | -0.657 | 0.0549 | No |
| 39 | Fut4 | 1729 | -0.665 | 0.0621 | No |
| 40 | Hbegf | 1860 | -0.709 | 0.0331 | No |
| 41 | Jag1 | 1979 | -0.750 | 0.0089 | No |
| 42 | Mxd1 | 2005 | -0.763 | 0.0162 | No |
| 43 | Tnfaip8 | 2050 | -0.785 | 0.0175 | No |
| 44 | Hes1 | 2255 | -0.896 | -0.0325 | No |
| 45 | Phlda1 | 2291 | -0.920 | -0.0254 | No |
| 46 | Serpinb8 | 2355 | -0.969 | -0.0267 | No |
| 47 | F3 | 2372 | -0.985 | -0.0118 | No |
| 48 | Fos | 2491 | -1.083 | -0.0292 | No |
| 49 | Il18 | 2586 | -1.191 | -0.0363 | No |
| 50 | Nr4a2 | 2700 | -1.361 | -0.0463 | No |
| 51 | Egr1 | 2733 | -1.440 | -0.0275 | No |
| 52 | Areg | 2775 | -1.538 | -0.0098 | No |
| 53 | Tlr2 | 2819 | -1.629 | 0.0092 | No |
| 54 | Cxcl5 | 2999 | -3.136 | 0.0134 | No |
Table: GSEA details [plain text format]

  

Fig 2: HALLMARK\_TNFA\_SIGNALING\_VIA\_NFKB: Random ES distribution      
 Gene set null distribution of ES for **HALLMARK\_TNFA\_SIGNALING\_VIA\_NFKB**

  
