# Supplementary material for: TMPRSS11B promotes an acidified microenvironment and immune suppression in squamous lung cancer
Source: EMBO Rep. 2025 Nov 10;26(24):6346–79. doi: 10.1038/s44319-025-00631-1 (PMC12714794; doi:10.1038/s44319-025-00631-1)
Supplement: Supplementary file 19 — Appendix Figure S1 Source Data [file 44319_2025_631_MOESM19_ESM.zip › Appendix Figure S1/S1C/GSEA Broad Institute_low pH vs rest of the regions (high pH)_Mh/HALLMARK_UNFOLDED_PROTEIN_RESPONSE.html]

Details for gene set HALLMARK\_UNFOLDED\_PROTEIN\_RESPONSE[GSEA]

|  || Dataset | Lactate high vs low\_Ranked |
| Phenotype | NoPhenotypeAvailable |
| Upregulated in class | na\_neg |
| GeneSet | HALLMARK\_UNFOLDED\_PROTEIN\_RESPONSE |
| Enrichment Score (ES) | -0.24372908 |
| Normalized Enrichment Score (NES) | -0.9716677 |
| Nominal p-value | 0.49271137 |
| FDR q-value | 0.7710928 |
| FWER p-Value | 1.0 |
Table: GSEA Results Summary

  

Fig 1: Enrichment plot: HALLMARK\_UNFOLDED\_PROTEIN\_RESPONSE      
 Profile of the Running ES Score & Positions of GeneSet Members on the Rank Ordered List

  

| SYMBOL | RANK IN GENE LIST | RANK METRIC SCORE | RUNNING ES | CORE ENRICHMENT || 1 | Atf3 | 161 | 1.444 | 0.0049 | No |
| 2 | Wfs1 | 639 | 0.858 | -0.1188 | No |
| 3 | Vegfa | 650 | 0.852 | -0.0877 | No |
| 4 | Cebpb | 773 | 0.736 | -0.0984 | No |
| 5 | Atp6v0d1 | 1017 | 0.554 | -0.1567 | No |
| 6 | Slc30a5 | 1132 | -0.505 | -0.1742 | No |
| 7 | Dctn1 | 1291 | -0.539 | -0.2048 | No |
| 8 | Kif5b | 1372 | -0.558 | -0.2089 | Yes |
| 9 | Wipi1 | 1385 | -0.560 | -0.1902 | Yes |
| 10 | Dnaja4 | 1411 | -0.565 | -0.1757 | Yes |
| 11 | Parn | 1543 | -0.598 | -0.1950 | Yes |
| 12 | Nhp2 | 1637 | -0.629 | -0.2005 | Yes |
| 13 | Nop14 | 1711 | -0.658 | -0.1981 | Yes |
| 14 | Hspa9 | 1743 | -0.670 | -0.1814 | Yes |
| 15 | Nop56 | 1822 | -0.696 | -0.1791 | Yes |
| 16 | Hyou1 | 1969 | -0.747 | -0.1974 | Yes |
| 17 | Xbp1 | 1976 | -0.750 | -0.1691 | Yes |
| 18 | Dnajc3 | 2133 | -0.820 | -0.1878 | Yes |
| 19 | Herpud1 | 2283 | -0.915 | -0.2003 | Yes |
| 20 | Kdelr3 | 2415 | -1.016 | -0.2027 | Yes |
| 21 | Dkc1 | 2460 | -1.059 | -0.1745 | Yes |
| 22 | Slc7a5 | 2465 | -1.061 | -0.1330 | Yes |
| 23 | Aldh18a1 | 2546 | -1.145 | -0.1133 | Yes |
| 24 | Asns | 2703 | -1.371 | -0.1097 | Yes |
| 25 | Psat1 | 2790 | -1.568 | -0.0749 | Yes |
| 26 | Pdia5 | 2847 | -1.737 | -0.0234 | Yes |
| 27 | Stc2 | 2919 | -2.148 | 0.0398 | Yes |
Table: GSEA details [plain text format]

  

Fig 2: HALLMARK\_UNFOLDED\_PROTEIN\_RESPONSE: Random ES distribution      
 Gene set null distribution of ES for **HALLMARK\_UNFOLDED\_PROTEIN\_RESPONSE**

  
