# Supplementary material for: TMPRSS11B promotes an acidified microenvironment and immune suppression in squamous lung cancer
Source: EMBO Rep. 2025 Nov 10;26(24):6346–79. doi: 10.1038/s44319-025-00631-1 (PMC12714794; doi:10.1038/s44319-025-00631-1)
Supplement: Supplementary file 19 — Appendix Figure S1 Source Data [file 44319_2025_631_MOESM19_ESM.zip › Appendix Figure S1/S1C/GSEA Broad Institute_low pH vs rest of the regions (high pH)_Mh/HALLMARK_UV_RESPONSE_DN.html]

Details for gene set HALLMARK\_UV\_RESPONSE\_DN[GSEA]

|  || Dataset | Lactate high vs low\_Ranked |
| Phenotype | NoPhenotypeAvailable |
| Upregulated in class | na\_pos |
| GeneSet | HALLMARK\_UV\_RESPONSE\_DN |
| Enrichment Score (ES) | 0.19937101 |
| Normalized Enrichment Score (NES) | 1.1321365 |
| Nominal p-value | 0.31111112 |
| FDR q-value | 0.4188123 |
| FWER p-Value | 0.968 |
Table: GSEA Results Summary

  

Fig 1: Enrichment plot: HALLMARK\_UV\_RESPONSE\_DN      
 Profile of the Running ES Score & Positions of GeneSet Members on the Rank Ordered List

  

| SYMBOL | RANK IN GENE LIST | RANK METRIC SCORE | RUNNING ES | CORE ENRICHMENT || 1 | Cav1 | 107 | 1.564 | -0.0023 | Yes |
| 2 | Pik3cd | 144 | 1.476 | 0.0173 | Yes |
| 3 | Ptprm | 223 | 1.346 | 0.0200 | Yes |
| 4 | Pmp22 | 266 | 1.263 | 0.0330 | Yes |
| 5 | Fbln5 | 275 | 1.258 | 0.0572 | Yes |
| 6 | Celf2 | 276 | 1.256 | 0.0841 | Yes |
| 7 | Gja1 | 314 | 1.208 | 0.0976 | Yes |
| 8 | Mgll | 316 | 1.208 | 0.1231 | Yes |
| 9 | Nrp1 | 353 | 1.158 | 0.1359 | Yes |
| 10 | Col5a2 | 387 | 1.116 | 0.1487 | Yes |
| 11 | Pdgfrb | 494 | 0.996 | 0.1346 | Yes |
| 12 | Col3a1 | 501 | 0.992 | 0.1538 | Yes |
| 13 | Itgb3 | 516 | 0.975 | 0.1700 | Yes |
| 14 | Dusp1 | 569 | 0.935 | 0.1726 | Yes |
| 15 | Dlc1 | 576 | 0.923 | 0.1904 | Yes |
| 16 | Col1a1 | 643 | 0.856 | 0.1867 | Yes |
| 17 | Col1a2 | 704 | 0.807 | 0.1839 | Yes |
| 18 | Syne1 | 710 | 0.803 | 0.1994 | Yes |
| 19 | Rgs4 | 791 | 0.714 | 0.1879 | No |
| 20 | Apbb2 | 854 | 0.668 | 0.1815 | No |
| 21 | Dab2 | 867 | 0.651 | 0.1914 | No |
| 22 | Plpp3 | 938 | 0.605 | 0.1810 | No |
| 23 | Sfmbt1 | 1073 | 0.523 | 0.1474 | No |
| 24 | Nr3c1 | 1076 | 0.519 | 0.1578 | No |
| 25 | Ythdc1 | 1358 | -0.554 | 0.0758 | No |
| 26 | Slc22a18 | 1519 | -0.591 | 0.0350 | No |
| 27 | Bckdhb | 1694 | -0.651 | -0.0092 | No |
| 28 | Fhl2 | 1756 | -0.673 | -0.0152 | No |
| 29 | Dbp | 1844 | -0.704 | -0.0292 | No |
| 30 | Synj2 | 1849 | -0.707 | -0.0154 | No |
| 31 | Met | 1981 | -0.751 | -0.0431 | No |
| 32 | Runx1 | 2029 | -0.774 | -0.0423 | No |
| 33 | Cited2 | 2055 | -0.788 | -0.0338 | No |
| 34 | Bmpr1a | 2065 | -0.792 | -0.0198 | No |
| 35 | Tent4a | 2176 | -0.847 | -0.0385 | No |
| 36 | Sipa1l1 | 2327 | -0.941 | -0.0685 | No |
| 37 | Abcc1 | 2340 | -0.951 | -0.0521 | No |
| 38 | F3 | 2372 | -0.985 | -0.0414 | No |
| 39 | Id1 | 2455 | -1.056 | -0.0462 | No |
| 40 | Slc7a1 | 2531 | -1.128 | -0.0471 | No |
| 41 | Igfbp5 | 2556 | -1.159 | -0.0304 | No |
| 42 | Erbb2 | 2600 | -1.214 | -0.0188 | No |
| 43 | Rnd3 | 2679 | -1.331 | -0.0163 | No |
| 44 | Rasa2 | 2689 | -1.349 | 0.0095 | No |
| 45 | Gcnt1 | 2743 | -1.463 | 0.0231 | No |
| 46 | Kit | 2838 | -1.701 | 0.0281 | No |
| 47 | Igf1r | 2859 | -1.811 | 0.0601 | No |
Table: GSEA details [plain text format]

  

Fig 2: HALLMARK\_UV\_RESPONSE\_DN: Random ES distribution      
 Gene set null distribution of ES for **HALLMARK\_UV\_RESPONSE\_DN**

  
