# Supplementary material for: TMPRSS11B promotes an acidified microenvironment and immune suppression in squamous lung cancer
Source: EMBO Rep. 2025 Nov 10;26(24):6346–79. doi: 10.1038/s44319-025-00631-1 (PMC12714794; doi:10.1038/s44319-025-00631-1)
Supplement: Supplementary file 19 — Appendix Figure S1 Source Data [file 44319_2025_631_MOESM19_ESM.zip › Appendix Figure S1/S1C/GSEA Broad Institute_low pH vs rest of the regions (high pH)_Mh/HALLMARK_UV_RESPONSE_UP.html]

Details for gene set HALLMARK\_UV\_RESPONSE\_UP[GSEA]

|  || Dataset | Lactate high vs low\_Ranked |
| Phenotype | NoPhenotypeAvailable |
| Upregulated in class | na\_pos |
| GeneSet | HALLMARK\_UV\_RESPONSE\_UP |
| Enrichment Score (ES) | 0.14289613 |
| Normalized Enrichment Score (NES) | 0.7997105 |
| Nominal p-value | 0.75974023 |
| FDR q-value | 0.9776208 |
| FWER p-Value | 1.0 |
Table: GSEA Results Summary

  

Fig 1: Enrichment plot: HALLMARK\_UV\_RESPONSE\_UP      
 Profile of the Running ES Score & Positions of GeneSet Members on the Rank Ordered List

  

| SYMBOL | RANK IN GENE LIST | RANK METRIC SCORE | RUNNING ES | CORE ENRICHMENT || 1 | Ctsl | 8 | 2.153 | 0.0537 | Yes |
| 2 | Hmox1 | 16 | 2.051 | 0.1050 | Yes |
| 3 | Atf3 | 161 | 1.444 | 0.0948 | Yes |
| 4 | Fmo1 | 281 | 1.243 | 0.0877 | Yes |
| 5 | Cdo1 | 362 | 1.150 | 0.0911 | Yes |
| 6 | Cdkn1c | 446 | 1.049 | 0.0909 | Yes |
| 7 | Icam1 | 492 | 0.999 | 0.1021 | Yes |
| 8 | Creg1 | 517 | 0.973 | 0.1196 | Yes |
| 9 | Atp6v1c1 | 524 | 0.967 | 0.1429 | Yes |
| 10 | Olfm1 | 716 | 0.798 | 0.1001 | No |
| 11 | Arrb2 | 718 | 0.796 | 0.1206 | No |
| 12 | Grina | 726 | 0.791 | 0.1390 | No |
| 13 | Maoa | 805 | 0.703 | 0.1314 | No |
| 14 | Bcl2l11 | 885 | 0.641 | 0.1219 | No |
| 15 | Prkcd | 984 | 0.574 | 0.1042 | No |
| 16 | Dgat1 | 987 | 0.571 | 0.1185 | No |
| 17 | Slc6a8 | 1041 | 0.543 | 0.1151 | No |
| 18 | Gpx3 | 1070 | 0.527 | 0.1195 | No |
| 19 | Gch1 | 1277 | -0.537 | 0.0649 | No |
| 20 | Stip1 | 1374 | -0.558 | 0.0475 | No |
| 21 | Btg2 | 1440 | -0.573 | 0.0409 | No |
| 22 | Polr2h | 1511 | -0.589 | 0.0329 | No |
| 23 | Rab27a | 1534 | -0.595 | 0.0412 | No |
| 24 | Bsg | 1560 | -0.604 | 0.0486 | No |
| 25 | Bid | 1565 | -0.606 | 0.0632 | No |
| 26 | Nr4a1 | 1632 | -0.627 | 0.0576 | No |
| 27 | Ppat | 1764 | -0.676 | 0.0316 | No |
| 28 | Rpn1 | 1925 | -0.730 | -0.0026 | No |
| 29 | Fkbp4 | 2117 | -0.814 | -0.0449 | No |
| 30 | Dnajb1 | 2127 | -0.818 | -0.0265 | No |
| 31 | Ppif | 2171 | -0.845 | -0.0187 | No |
| 32 | Clcn2 | 2448 | -1.052 | -0.0832 | No |
| 33 | Ggh | 2480 | -1.071 | -0.0654 | No |
| 34 | Fos | 2491 | -1.083 | -0.0404 | No |
| 35 | Asns | 2703 | -1.371 | -0.0749 | No |
| 36 | Tst | 2769 | -1.524 | -0.0566 | No |
| 37 | Aqp3 | 2802 | -1.594 | -0.0256 | No |
| 38 | Epcam | 2813 | -1.616 | 0.0134 | No |
| 39 | Tyro3 | 2950 | -2.353 | 0.0297 | No |
Table: GSEA details [plain text format]

  

Fig 2: HALLMARK\_UV\_RESPONSE\_UP: Random ES distribution      
 Gene set null distribution of ES for **HALLMARK\_UV\_RESPONSE\_UP**

  
