# Supplementary material for: TMPRSS11B promotes an acidified microenvironment and immune suppression in squamous lung cancer
Source: EMBO Rep. 2025 Nov 10;26(24):6346–79. doi: 10.1038/s44319-025-00631-1 (PMC12714794; doi:10.1038/s44319-025-00631-1)
Supplement: Supplementary file 19 — Appendix Figure S1 Source Data [file 44319_2025_631_MOESM19_ESM.zip › Appendix Figure S1/S1C/GSEA Broad Institute_low pH vs rest of the regions (high pH)_Mh/HALLMARK_XENOBIOTIC_METABOLISM.html]

Details for gene set HALLMARK\_XENOBIOTIC\_METABOLISM[GSEA]

|  || Dataset | Lactate high vs low\_Ranked |
| Phenotype | NoPhenotypeAvailable |
| Upregulated in class | na\_neg |
| GeneSet | HALLMARK\_XENOBIOTIC\_METABOLISM |
| Enrichment Score (ES) | -0.22750123 |
| Normalized Enrichment Score (NES) | -1.08345 |
| Nominal p-value | 0.36455697 |
| FDR q-value | 0.7249234 |
| FWER p-Value | 1.0 |
Table: GSEA Results Summary

  

Fig 1: Enrichment plot: HALLMARK\_XENOBIOTIC\_METABOLISM      
 Profile of the Running ES Score & Positions of GeneSet Members on the Rank Ordered List

  

| SYMBOL | RANK IN GENE LIST | RANK METRIC SCORE | RUNNING ES | CORE ENRICHMENT || 1 | Apoe | 6 | 2.177 | 0.0318 | No |
| 2 | Hmox1 | 16 | 2.051 | 0.0606 | No |
| 3 | F10 | 33 | 1.837 | 0.0838 | No |
| 4 | Cd36 | 52 | 1.748 | 0.1049 | No |
| 5 | Igf1 | 60 | 1.691 | 0.1288 | No |
| 6 | Enpep | 235 | 1.322 | 0.0910 | No |
| 7 | Fmo1 | 281 | 1.243 | 0.0952 | No |
| 8 | Cat | 297 | 1.225 | 0.1092 | No |
| 9 | Arg2 | 303 | 1.219 | 0.1264 | No |
| 10 | Cdo1 | 362 | 1.150 | 0.1249 | No |
| 11 | Fas | 457 | 1.039 | 0.1095 | No |
| 12 | Slc12a4 | 635 | 0.862 | 0.0635 | No |
| 13 | Npc1 | 638 | 0.859 | 0.0762 | No |
| 14 | Irf8 | 663 | 0.840 | 0.0812 | No |
| 15 | Xdh | 717 | 0.797 | 0.0758 | No |
| 16 | Dhrs7 | 752 | 0.762 | 0.0762 | No |
| 17 | Acox3 | 758 | 0.754 | 0.0862 | No |
| 18 | Ddah2 | 800 | 0.706 | 0.0834 | No |
| 19 | Maoa | 805 | 0.703 | 0.0930 | No |
| 20 | Acp2 | 1015 | 0.554 | 0.0316 | No |
| 21 | Ninj1 | 1025 | 0.551 | 0.0371 | No |
| 22 | Lpin2 | 1035 | 0.546 | 0.0426 | No |
| 23 | Gabarapl1 | 1061 | 0.533 | 0.0424 | No |
| 24 | Rap1gap | 1140 | -0.507 | 0.0242 | No |
| 25 | Gch1 | 1277 | -0.537 | -0.0131 | No |
| 26 | Shmt2 | 1410 | -0.565 | -0.0486 | No |
| 27 | Casp6 | 1431 | -0.572 | -0.0464 | No |
| 28 | Acp1 | 1433 | -0.572 | -0.0379 | No |
| 29 | Atoh8 | 1704 | -0.656 | -0.1182 | No |
| 30 | Retsat | 1742 | -0.669 | -0.1202 | No |
| 31 | Gart | 1796 | -0.686 | -0.1274 | No |
| 32 | Aldh3a1 | 1866 | -0.710 | -0.1395 | No |
| 33 | Mccc2 | 1879 | -0.713 | -0.1324 | No |
| 34 | Pmm1 | 1922 | -0.729 | -0.1352 | No |
| 35 | Epha2 | 1997 | -0.758 | -0.1482 | No |
| 36 | Pts | 2017 | -0.768 | -0.1427 | No |
| 37 | Hnf4a | 2271 | -0.910 | -0.2134 | Yes |
| 38 | Entpd5 | 2299 | -0.927 | -0.2080 | Yes |
| 39 | Upp1 | 2304 | -0.929 | -0.1949 | Yes |
| 40 | Ptgr1 | 2332 | -0.943 | -0.1894 | Yes |
| 41 | Crot | 2333 | -0.945 | -0.1747 | Yes |
| 42 | Jup | 2337 | -0.950 | -0.1609 | Yes |
| 43 | Slc35d1 | 2339 | -0.951 | -0.1465 | Yes |
| 44 | Slc1a5 | 2375 | -0.989 | -0.1429 | Yes |
| 45 | Smox | 2377 | -0.990 | -0.1278 | Yes |
| 46 | Por | 2395 | -1.005 | -0.1179 | Yes |
| 47 | Atp2a2 | 2399 | -1.006 | -0.1033 | Yes |
| 48 | Dcxr | 2434 | -1.037 | -0.0986 | Yes |
| 49 | Ptges | 2452 | -1.053 | -0.0879 | Yes |
| 50 | Cyp27a1 | 2463 | -1.060 | -0.0748 | Yes |
| 51 | Spint2 | 2539 | -1.139 | -0.0823 | Yes |
| 52 | Cfb | 2772 | -1.529 | -0.1364 | Yes |
| 53 | Ces1d | 2822 | -1.643 | -0.1273 | Yes |
| 54 | Hgfac | 2903 | -2.055 | -0.1222 | Yes |
| 55 | Gsto1 | 2912 | -2.103 | -0.0922 | Yes |
| 56 | Adh7 | 2943 | -2.301 | -0.0665 | Yes |
| 57 | Fetub | 2987 | -2.921 | -0.0356 | Yes |
| 58 | Reg1 | 3012 | -3.392 | 0.0091 | Yes |
Table: GSEA details [plain text format]

  

Fig 2: HALLMARK\_XENOBIOTIC\_METABOLISM: Random ES distribution      
 Gene set null distribution of ES for **HALLMARK\_XENOBIOTIC\_METABOLISM**

  
