# Supplementary figures and images for: TMPRSS11B promotes an acidified microenvironment and immune suppression in squamous lung cancer (part 6 of 6)
Source: EMBO Rep. 2025 Nov 10;26(24):6346–79. doi: 10.1038/s44319-025-00631-1 (PMC12714794; doi:10.1038/s44319-025-00631-1)

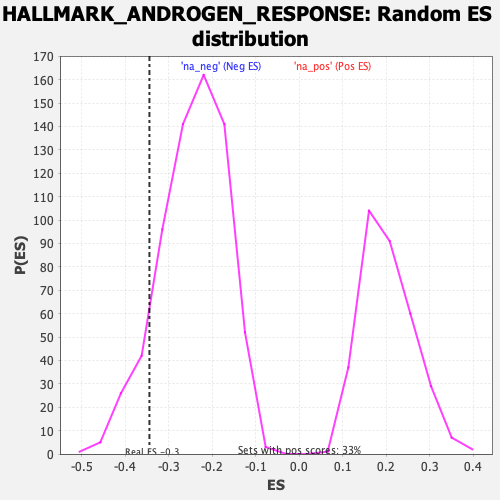

Supplement: Supplementary file 19 — Appendix Figure S1 Source Data [file 44319_2025_631_MOESM19_ESM.zip › Appendix Figure S1/S1C/GSEA Broad Institute_low pH vs rest of the regions (high pH)_Mh/gset_rnd_es_dist_214.png]

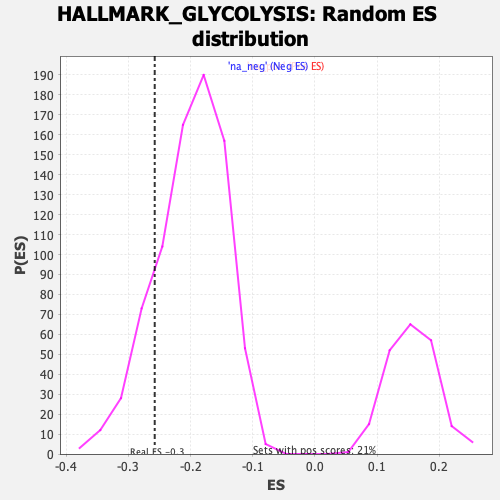

Supplement: Supplementary file 19 — Appendix Figure S1 Source Data [file 44319_2025_631_MOESM19_ESM.zip › Appendix Figure S1/S1C/GSEA Broad Institute_low pH vs rest of the regions (high pH)_Mh/gset_rnd_es_dist_216.png]

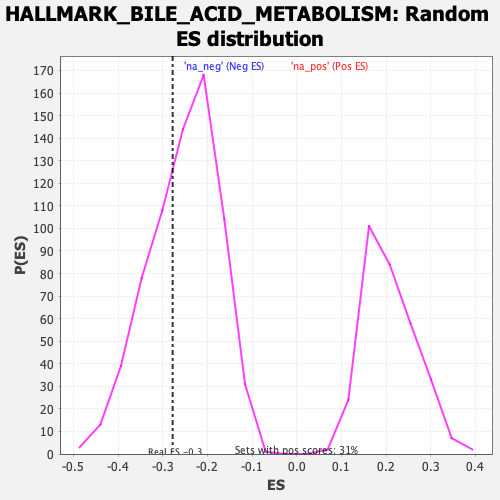

Supplement: Supplementary file 19 — Appendix Figure S1 Source Data [file 44319_2025_631_MOESM19_ESM.zip › Appendix Figure S1/S1C/GSEA Broad Institute_low pH vs rest of the regions (high pH)_Mh/gset_rnd_es_dist_218.png]

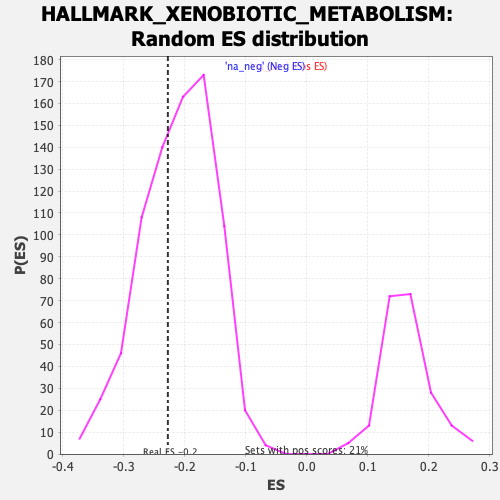

Supplement: Supplementary file 19 — Appendix Figure S1 Source Data [file 44319_2025_631_MOESM19_ESM.zip › Appendix Figure S1/S1C/GSEA Broad Institute_low pH vs rest of the regions (high pH)_Mh/gset_rnd_es_dist_220.png]

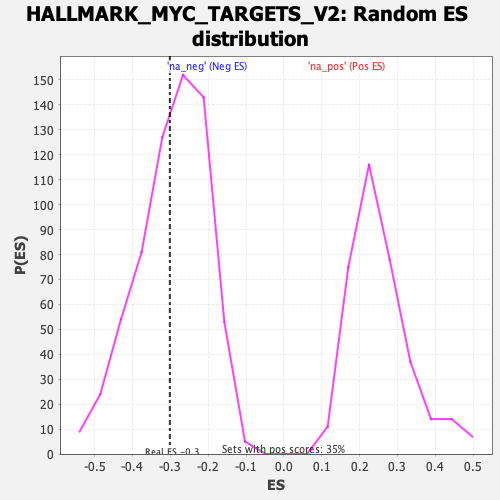

Supplement: Supplementary file 19 — Appendix Figure S1 Source Data [file 44319_2025_631_MOESM19_ESM.zip › Appendix Figure S1/S1C/GSEA Broad Institute_low pH vs rest of the regions (high pH)_Mh/gset_rnd_es_dist_222.png]

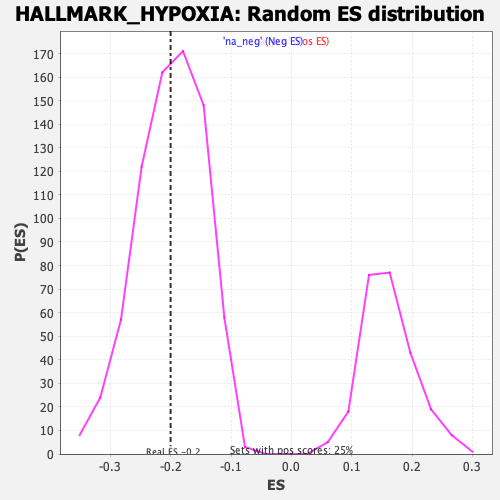

Supplement: Supplementary file 19 — Appendix Figure S1 Source Data [file 44319_2025_631_MOESM19_ESM.zip › Appendix Figure S1/S1C/GSEA Broad Institute_low pH vs rest of the regions (high pH)_Mh/gset_rnd_es_dist_224.png]

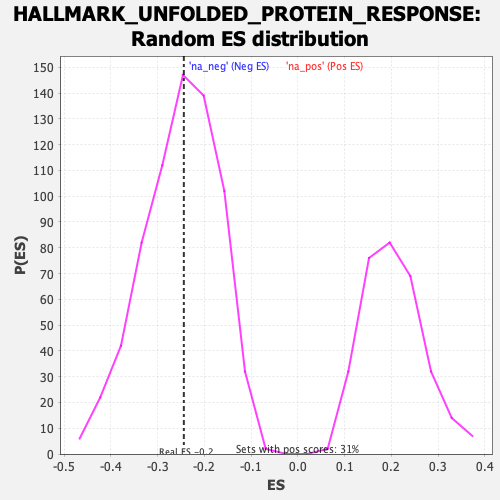

Supplement: Supplementary file 19 — Appendix Figure S1 Source Data [file 44319_2025_631_MOESM19_ESM.zip › Appendix Figure S1/S1C/GSEA Broad Institute_low pH vs rest of the regions (high pH)_Mh/gset_rnd_es_dist_226.png]

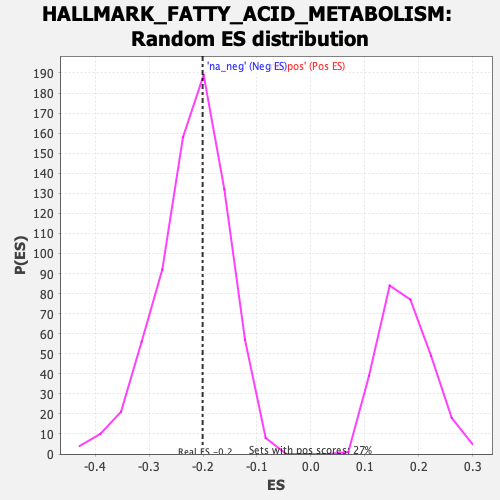

Supplement: Supplementary file 19 — Appendix Figure S1 Source Data [file 44319_2025_631_MOESM19_ESM.zip › Appendix Figure S1/S1C/GSEA Broad Institute_low pH vs rest of the regions (high pH)_Mh/gset_rnd_es_dist_228.png]

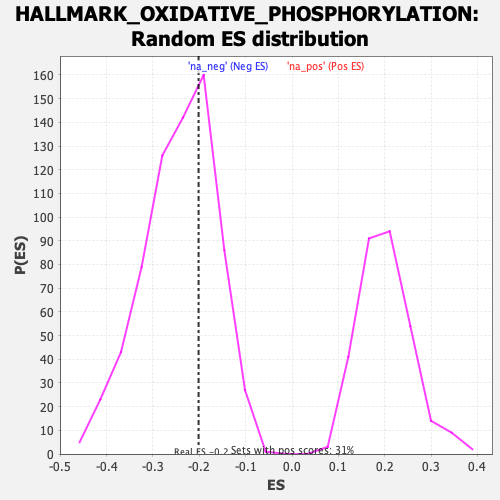

Supplement: Supplementary file 19 — Appendix Figure S1 Source Data [file 44319_2025_631_MOESM19_ESM.zip › Appendix Figure S1/S1C/GSEA Broad Institute_low pH vs rest of the regions (high pH)_Mh/gset_rnd_es_dist_230.png]

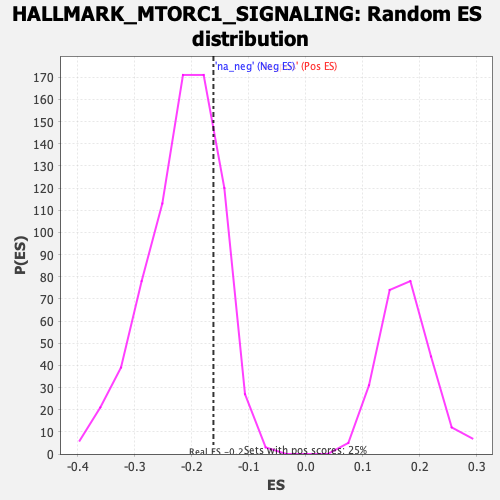

Supplement: Supplementary file 19 — Appendix Figure S1 Source Data [file 44319_2025_631_MOESM19_ESM.zip › Appendix Figure S1/S1C/GSEA Broad Institute_low pH vs rest of the regions (high pH)_Mh/gset_rnd_es_dist_232.png]

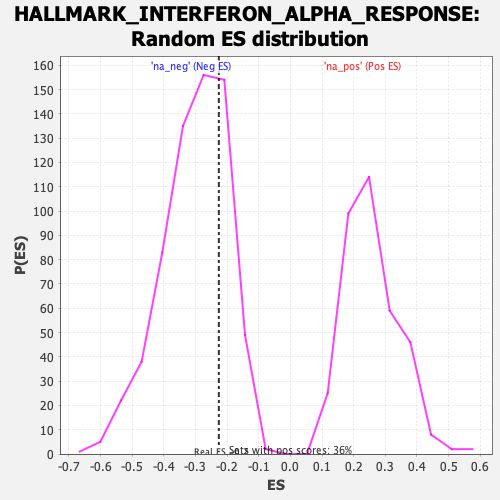

Supplement: Supplementary file 19 — Appendix Figure S1 Source Data [file 44319_2025_631_MOESM19_ESM.zip › Appendix Figure S1/S1C/GSEA Broad Institute_low pH vs rest of the regions (high pH)_Mh/gset_rnd_es_dist_234.png]

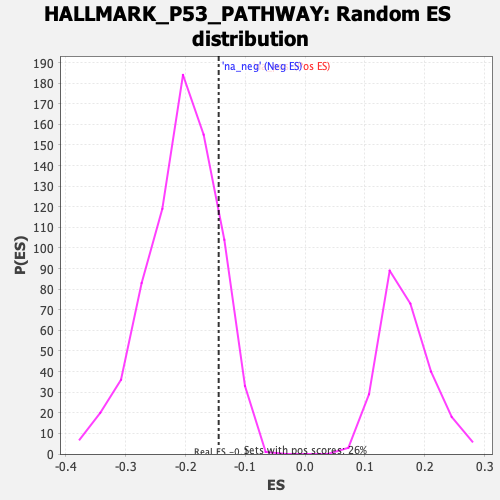

Supplement: Supplementary file 19 — Appendix Figure S1 Source Data [file 44319_2025_631_MOESM19_ESM.zip › Appendix Figure S1/S1C/GSEA Broad Institute_low pH vs rest of the regions (high pH)_Mh/gset_rnd_es_dist_236.png]

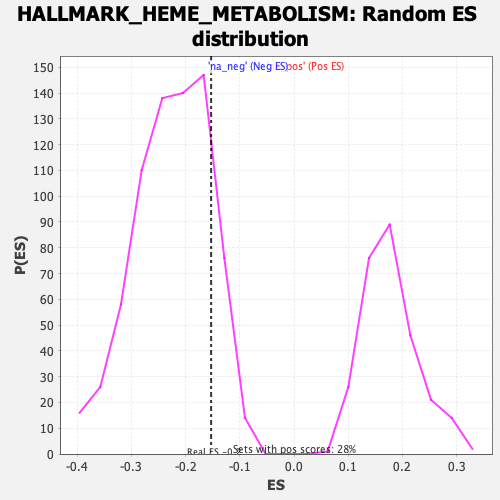

Supplement: Supplementary file 19 — Appendix Figure S1 Source Data [file 44319_2025_631_MOESM19_ESM.zip › Appendix Figure S1/S1C/GSEA Broad Institute_low pH vs rest of the regions (high pH)_Mh/gset_rnd_es_dist_238.png]

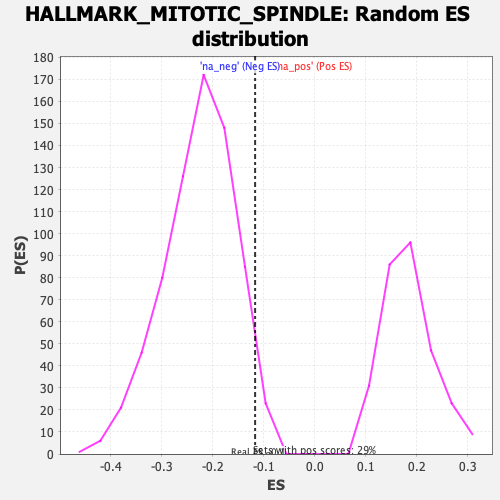

Supplement: Supplementary file 19 — Appendix Figure S1 Source Data [file 44319_2025_631_MOESM19_ESM.zip › Appendix Figure S1/S1C/GSEA Broad Institute_low pH vs rest of the regions (high pH)_Mh/gset_rnd_es_dist_240.png]

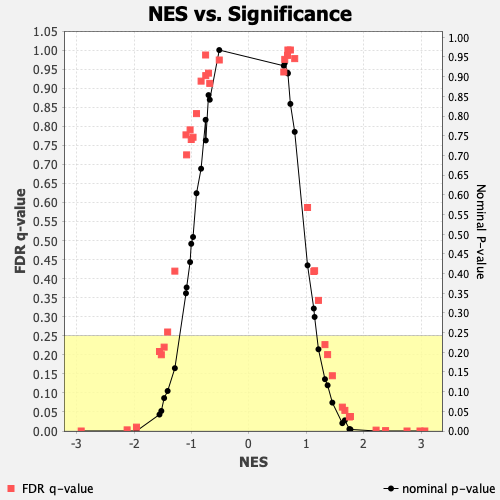

Supplement: Supplementary file 19 — Appendix Figure S1 Source Data [file 44319_2025_631_MOESM19_ESM.zip › Appendix Figure S1/S1C/GSEA Broad Institute_low pH vs rest of the regions (high pH)_Mh/pvalues_vs_nes_plot.png]

# Percentage overlap with tumors

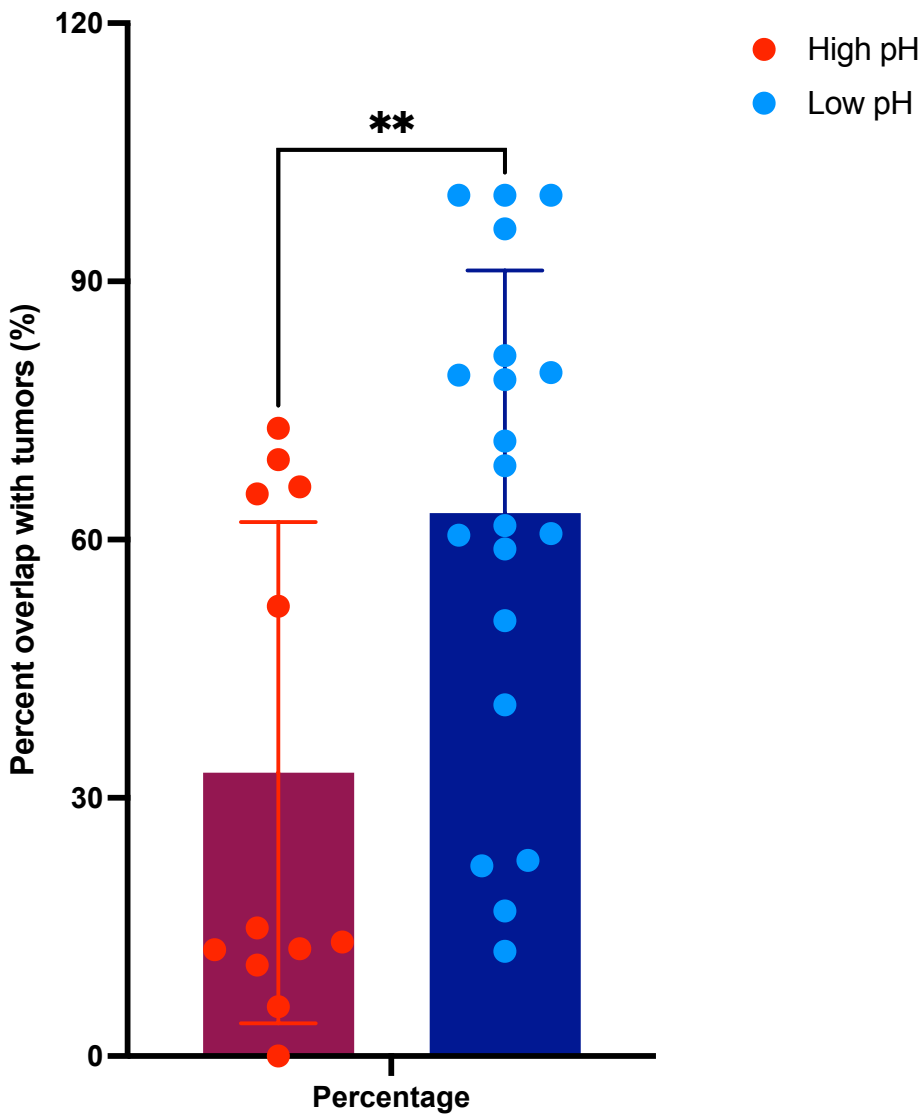

Supplement: Supplementary file 20 — Appendix Figure S2 Source Data [file 44319_2025_631_MOESM20_ESM.zip › Appendix Figure S2/S2B/Percentage overlap with tumors.pdf]
