# Supplementary material for: TMPRSS11B promotes an acidified microenvironment and immune suppression in squamous lung cancer
Source: EMBO Rep. 2025 Nov 10;26(24):6346–79. doi: 10.1038/s44319-025-00631-1 (PMC12714794; doi:10.1038/s44319-025-00631-1)
Supplement: Supplementary file 21 — Appendix Figure S3 Source Data [file 44319_2025_631_MOESM21_ESM.zip › Appendix Figure S3/S3A-B/Read Me.rtf]

The spatial transcriptomics data used for this analysis has been deposited to GEO and accession number is included in the manuscript. The markers used for the analysis have been provided in Table EV2.
