# Supplementary material for: TMPRSS11B promotes an acidified microenvironment and immune suppression in squamous lung cancer
Source: EMBO Rep. 2025 Nov 10;26(24):6346–79. doi: 10.1038/s44319-025-00631-1 (PMC12714794; doi:10.1038/s44319-025-00631-1)
Supplement: Supplementary file 22 — Expanded View Figures [file 44319_2025_631_MOESM22_ESM.pdf]

Expanded View Figures

Figure EV1. *Tmprss11b* depletion inhibits tumor burden in a syngeneic mouse model of LUSC.

(**A**) Agarose gel electrophoresis images of PCR amplified products from the Surveyor assay performed on the genomic DNA isolated from KLN205 cells expressing control or *Tmprss11b* sg1 or *Tmprss11b* sg2. The assay was repeated two times with different surveyor primers to confirm the observations (biological replicates). (**B**) Image showing the resected tumors at endpoint from the syngeneic experiment in Fig. 1C. (**C**) qRT-PCR analysis of *Tmprss11b* mRNA in KLN205 cells expressing doxycycline-inducible control shRNA or two independent shRNA sequences targeting *Tmprss11b*. Brown–Forsythe and Welch ANOVA test with Dunnett's T3 multiple comparisons test was used for the statistical analysis, ****$P < 0.0001$. Plot represents mean ± SD; $n = 4$ per group (technical replicates). Experiment was repeated two times for confirmation (biological replicates). (**D**) Quantification of tumor volumes of KLN205 cells expressing doxycycline-inducible control or *Tmprss11b* shRNA on day 50 (terminal measurement) post injection in syngeneic DBA/2 wild-type mice ($n = 10$ control shRNA mice; $n = 8$ *Tmprss11b* sh1 mice; $n = 10$ *Tmprss11b* sh2 mice, biological replicates). Ordinary one-way ANOVA with Dunnett's multiple comparisons test was used for the statistical analysis, $P = 0.0235$. Plot represents mean ± SD. (**E**) Image showing the resected tumors from the syngeneic experiment in Fig. 1D. (**F**) qRT-PCR analysis of *Tmprss11b* mRNA in the resected KLN205 tumors from D). Brown–Forsythe and Welch ANOVA test with Dunnett's T3 multiple comparisons test was used for the statistical analysis, $P = 0.0055$ (*T11b* shRNA1), $P = 0.0041$(*T11b* shRNA2). Plot represents mean ± SD; $n = 3$ technical replicates, $n = 3$–6 tumors per group, biological replicates. (**G**) Quantification of CD8a staining from fluorescent immunohistochemistry (IHC-F) performed on KLN205 tumor sections. An unpaired t test with Welch's correction was used for the statistical analysis ($n = 10$–14 fields per tumor section, 3 tumors per group, biological replicates). Plot represents mean ± SD. Source data are available online for this figure.

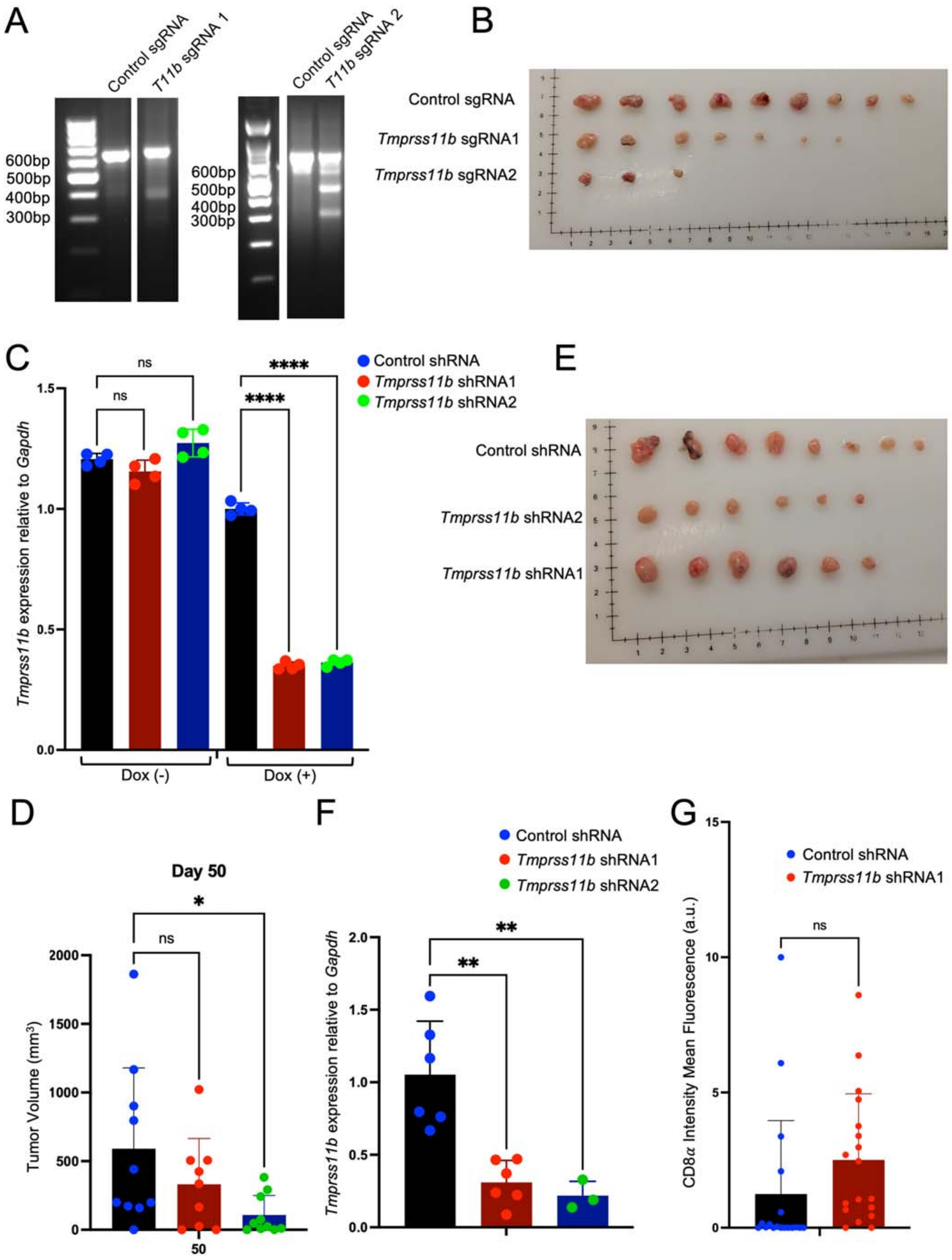
A
Control sgRNA
T11b sgRNA 1
Control sgRNA
T11b sgRNA 2
600bp
500bp
400bp
300bp
600bp
500bp
400bp
300bp
B
Control sgRNA
Tmprss11b sgRNA1
Tmprss11b sgRNA2
C
Control shRNA
Tmprss11b shRNA1
Tmprss11b shRNA2
Tmprss11b expression relative to Gapdh
ns
ns
****
****
Dox (-)
Dox (+)
E
Control shRNA
Tmprss11b shRNA2
Tmprss11b shRNA1
D
Day 50
Tumor Volume (mm3)
ns
*
50
F
Control shRNA
Tmprss11b shRNA1
Tmprss11b shRNA2
Tmprss11b expression relative to Gapdh
**
**
G
Control shRNA
Tmprss11b shRNA1
CD8α Intensity Mean Fluorescence (a.u.)
ns

 © The Author(s)

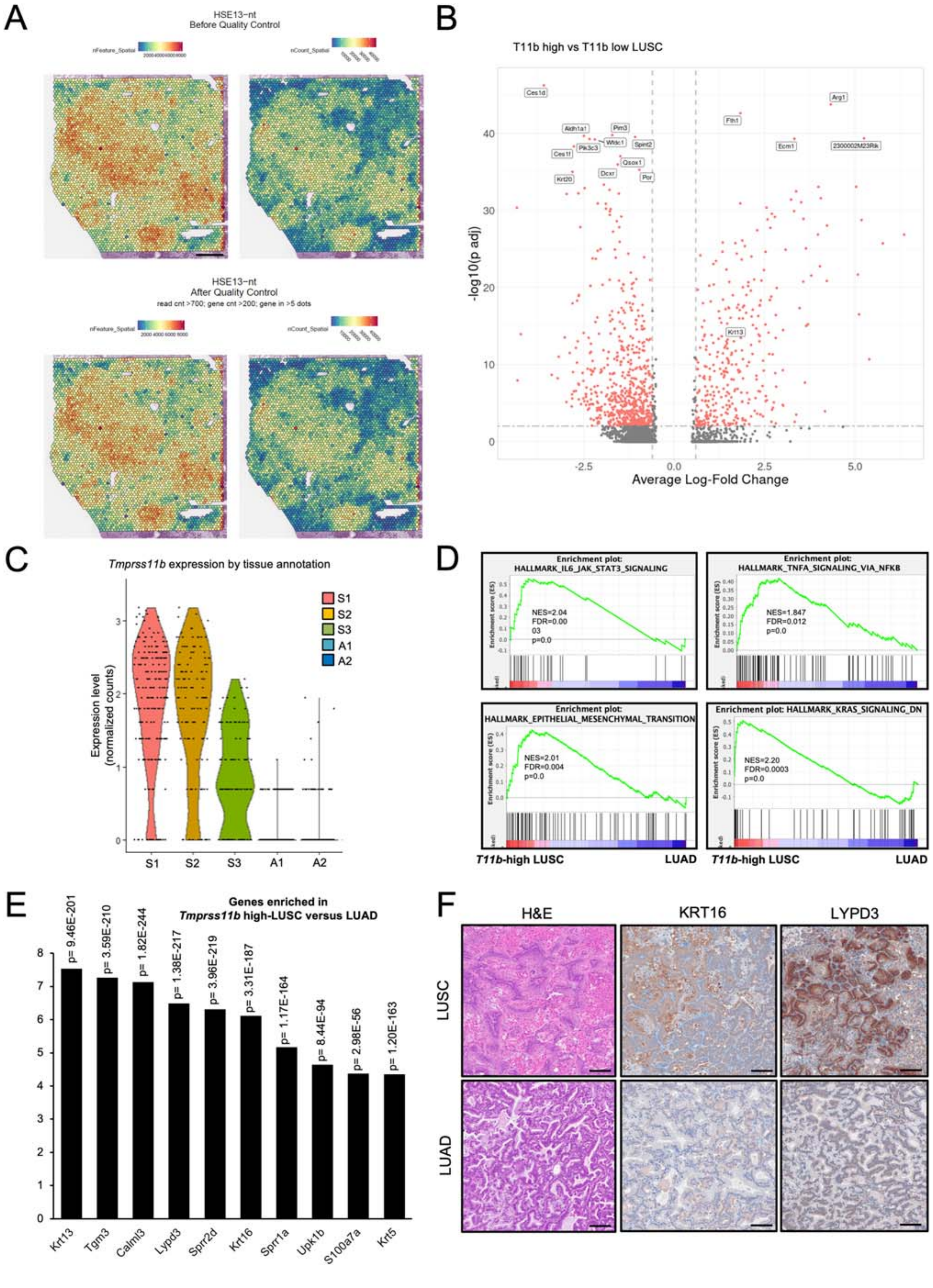
A
HSE13-nt
Before Quality Control
HSE13-nt
After Quality Control
read cnt >700; gene cnt >200; gene in >5 dots
B
T11b high vs T11b low LUSC
-log10(p adj)
Average Log-Fold Change
C
Tmprss11b expression by tissue annotation
Expression level (normalized counts)
S1
S2
S3
A1
A2
D
Enrichment plot: HALLMARK_IL6_JAK_STAT3_SIGNALING
NES=2.04
Enrichment plot: HALLMARK_TNFA_SIGNALING_VIA_NFKB
NES=1.847
FDR=0.012
p=0.0
Enrichment plot: HALLMARK_EPITHELIAL_MESENCHYMAL_TRANSITION
NES=2.01
FDR=0.004
p=0.0
Enrichment plot: HALLMARK_KRAS_SIGNALING_DN
NES=2.20
FDR=0.0003
p=0.0
T11b-high LUSC
LUAD
E
Genes enriched in Tmprss11b high-LUSC versus LUAD
p= 9.46E-201
p= 3.59E-210
p= 1.82E-244
p= 1.38E-217
p= 3.96E-219
p= 3.31E-187
p= 1.17E-164
p= 8.44E-94
p= 2.98E-56
p= 1.20E-163
Krt13
Tgm3
Calml3
Lypd3
Sprr2d
Krt16
Sprr1a
Upk1b
S100a7a
Krt5
F
H&E
KRT16
LYPD3
LUSC
LUAD

Figure EV2. *Tmprss11b*-high squamous tumors have increased expression of oncogenes and squamous markers.

(**A**) Spatial plots depicting the read counts before and after the quality control process. Scale bar, 1 mm. (**B**) Volcano plot showing the top differentially expressed genes in the *Tmprss11b*-high versus low LUSCs spatial data. A total of 4090 genes are plotted, of which 970 pass filter (colored). A two-tailed Wilcoxon rank-sum test with Bonferroni correction was used for the statistical analysis ($n = 4280$ Visium spots post-QC, biological replicates). (**C**) Violin plot depicting normalized counts for *Tmprss11b* transcript in the annotated regions (from Fig. 2B) ($n = 1597$, biological replicates). (**D**) Gene set enrichment analysis (GSEA) of the *Tmprss11b*-high LUSC versus LUAD spatial data with normalized enrichment scores (NES), false discovery rate (FDR) and *P* values for the indicated gene signatures. The nominal *P* and FDR values were obtained from the "GSEA Preranked" tool (from Broad Institute) using a weighted scoring scheme. Gene sets were evaluated based on the default normalized enrichment score method, and statistical significance was determined by bootstrapping with 1000 permutations. (**E**) Top squamous markers and known oncogenes from the differential gene expression (DEG) analysis of the *Tmprss11b*-high LUSC versus LUAD spatial data. Differential gene expression was calculated using Seurat's FindAllMarkers function, and direct comparisons between two classes and corresponding *P* values were obtained using Seurat's FindMarkers function with the Wilcoxon rank-sum test with Bonferroni *P* value correction. (**F**) Representative H&E and immunohistochemistry (IHC) validation for KRT16 and LYPD3 in LUSC (top) and mucinous LUAD (bottom). The staining was repeated with lung sections from different mice ($n = 2$–3, biological replicates). Scale bar, 100 µm. Source data are available online for this figure

© The Author(s)

A

Keratin genes showing reduced expression in *Tmprss11b* knockdown KLN205 tumors

Gene	*T11b* sh1 log2FC	FDR	*T11b* sh2 log2FC	FDR
Krt1	-5.13	0.195	-8.66	0.015
Krt5	-3.51	0.353	-7.48	0.015
Krt6b	-3.52	0.33	-4.76	0.13
Krt10	-2.64	0.195	-2.13	0.54
Krt16	-3.02	0.013	-2.7	0.051
Krtdap	-3.78	0.69 (p=0.0204)	-7.37	0.09

B

Keratin genes enriched in *T11b*-high versus *T11b*-low LUSCs spatial data

Gene	log2FC	Adj p -value
Krt16	4.03	1.85E-23
Krt6b	2.78	7.03E-30
Krt14	2.66	1.85E-28
Krtdap	2.33	5.02E-17
Krt17	1.59	3.20E-12
Krt4	1.58	5.50E-12
Krt13	1.48	5.72E-14
Krt6a	1.405	6.74E-13

C

Keratin genes enriched in *T11b*-high LUSCs versus LUADs spatial data

Gene	log2FC	Adj p -value
Krt13	7.53	9.46E-201
Krtdap	7.39	1.07E-231
Krt6b	7.29	7.74E-227
Krt16	6.12	3.31E-187
Krt6a	5.71	1.07E-190
Krt14	5.55	1.21E-185
Krt17	5.25	1.39E-197
Krt5	4.35	1.20E-163

D

Keratin genes enriched in *T11B*-high versus *T11B*-low LUSCs TCGA data

Gene	log ratio	T-test p -value
KRT13	4.85	1.30715E-33
KRT24	4.17	1.47289E-19
KRTDAP	3.96	4.58479E-20
KRT78	3.44	1.44602E-31
KRT16	2.92	2.58855E-27
KRT6C	2.83	4.80952E-33
KRT10	2.81	1.24801E-11
KRT6B	2.79	3.89382E-31
KRT14	2.69	2.48345E-12
KRT6A	2.50	3.68511E-28

E

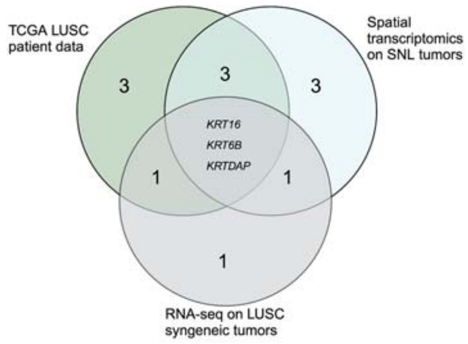

Figure EV3. Modulation of TMPRSS11B expression is accompanied by coordinated changes in expression of Keratin genes.

(**A**) Top downregulated Keratin genes from differential gene expression analysis of control shRNA versus *Tmprss11b* shRNA bulk RNA sequencing from the KLN205 syngeneic experiment in Fig. 1D. The log2FC change depicts the reduction in expression of the indicated genes in the *Tmprss11b* knockdown tumors compared to the control. (**B**) Top Keratin genes from the differential gene expression (DEG) analysis of the *Tmprss11b*-high versus low in LUSC spatial data from SNL lung tumors. (**C**) Top keratin genes from the differential gene expression (DEG) analysis of the *Tmprss11b*-high LUSC versus LUAD spatial data from SNL lung tumors. (**D**) Top Keratin genes from the differential gene expression (DEG) analysis of *TMPRSS11B*-high versus low LUSC human tumors from TCGA. (**E**) Venn diagram depicting overlapping Keratin genes from the gene lists in (**A–D**). Source data are available online for this figure

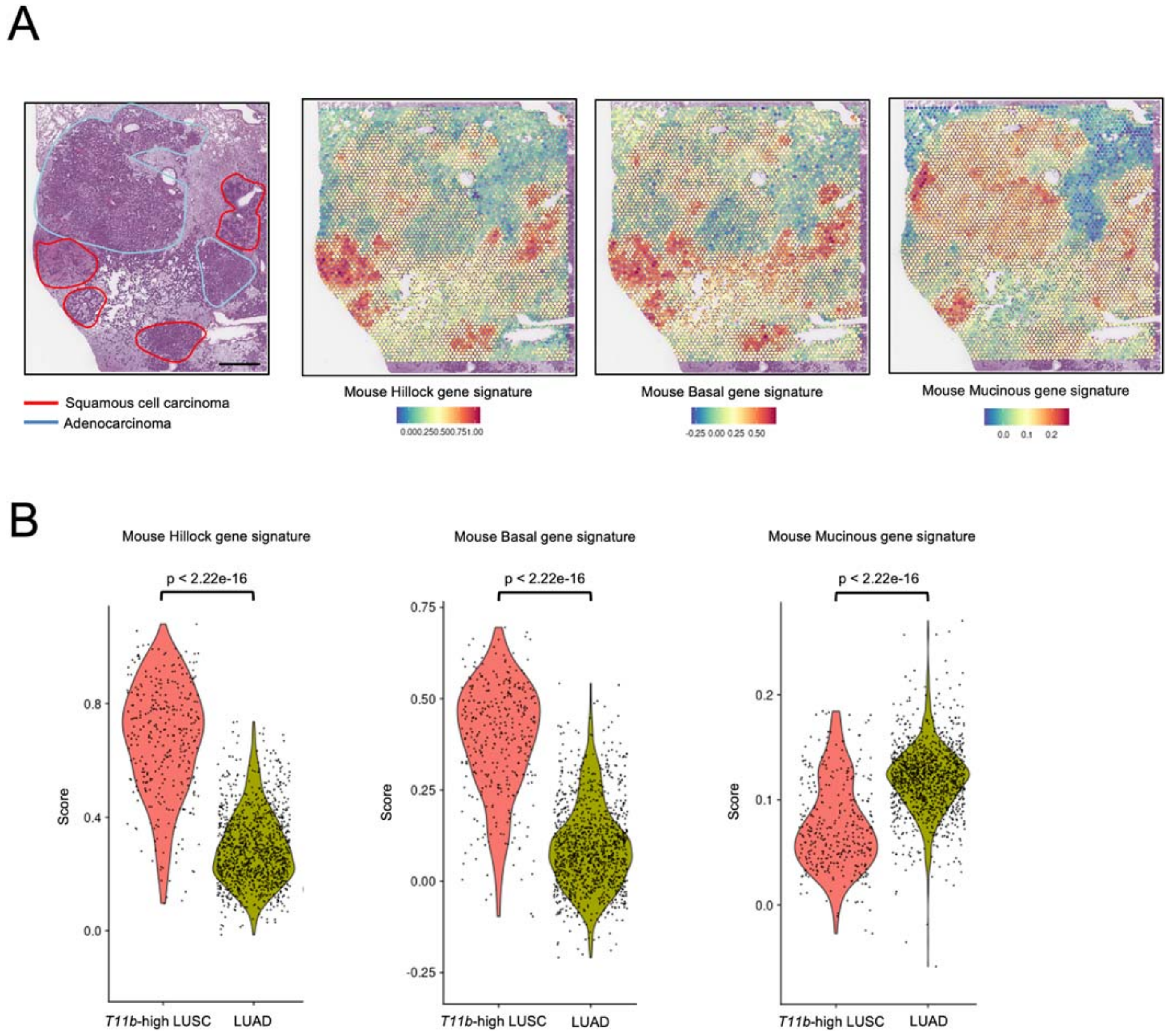

Figure EV4. *Tmprss11b*-high squamous tumors show enrichment for hillock and basal gene signatures.

(**A**) H&E image of the lung section from 3 A), annotated with regions of LUSC and LUAD, (left) and spatial plots from the transcriptomic data depicting the distribution of the indicated gene signatures (right). Scale bar, 1 mm. (**B**) Violin plots representing the enrichment for the indicated gene signatures in *Tmprss11b*-high LUSC and LUAD. A two-tailed Wilcoxon rank-sum test was used for the statistical analysis ($n = 1492$, biological replicates). $P <$ 2.22e-16 for all comparisons. Source data are available online for this figure

 © The Author(s)

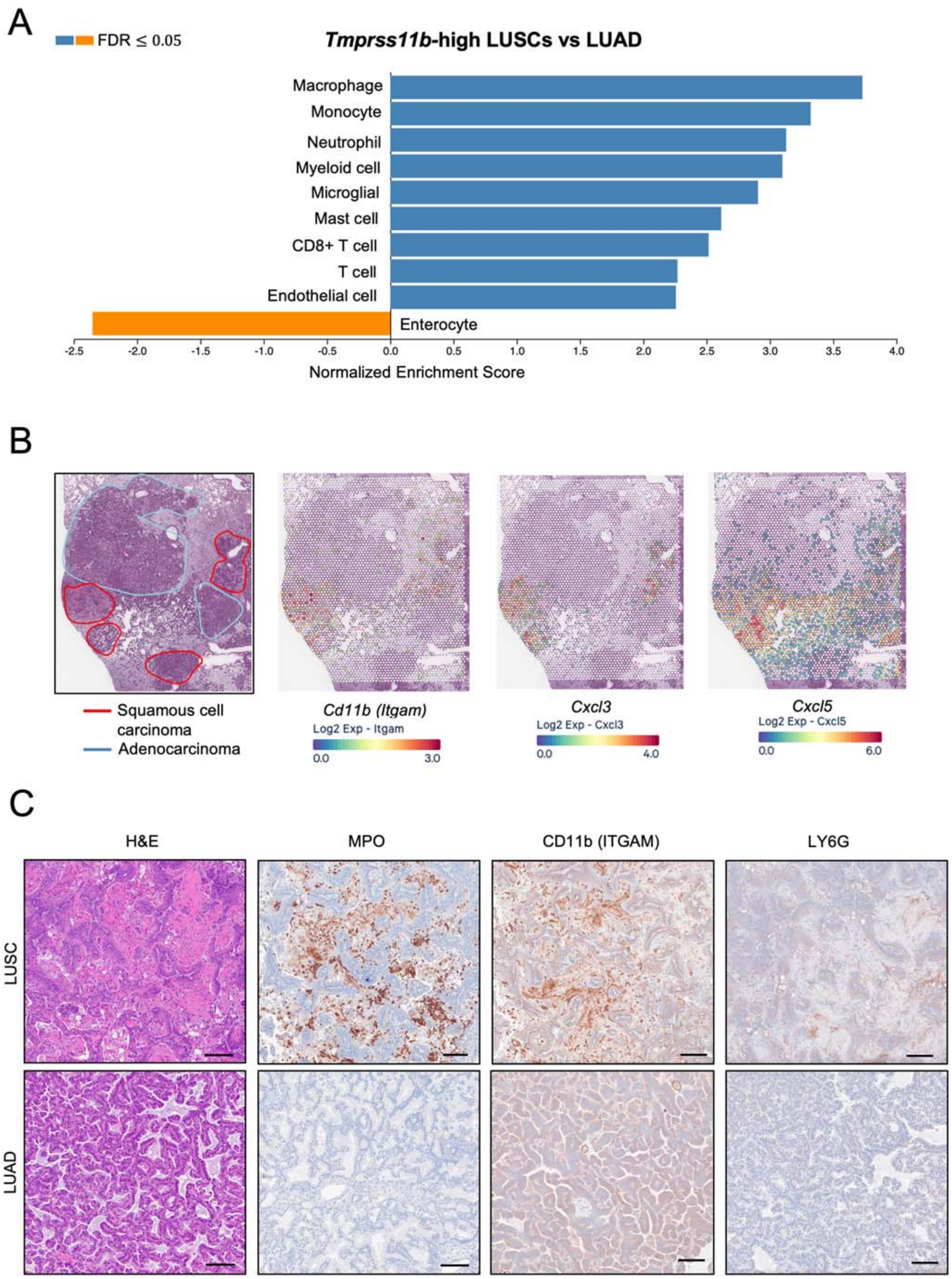
A
FDR ≤ 0.05
Tmprss11b-high LUSCs vs LUAD
Macrophage
Monocyte
Neutrophil
Myeloid cell
Microglial
Mast cell
CD8+ T cell
T cell
Endothelial cell
Enterocyte
-2.5
-2.0
-1.5
-1.0
-0.5
0.0
0.5
1.0
1.5
2.0
2.5
3.0
3.5
4.0
Normalized Enrichment Score
B
Squamous cell carcinoma
Adenocarcinoma
Cd11b (Itgam)
Log2 Exp - Itgam
0.0
3.0
Cxcl3
Log2 Exp - Cxcl3
0.0
4.0
Cxcl5
Log2 Exp - Cxcl5
0.0
6.0
C
H&E
MPO
CD11b (ITGAM)
LY6G
LUSC
LUAD

Figure EV5. *Tmprss11b*-high squamous tumors have higher infiltration of neutrophils.

(**A**) Gene set enrichment analysis (GSEA) of *Tmprss11b*-high LUSC versus LUAD spatial transcriptomics data with normalized enrichment scores (NES) and false discovery rate (FDR) for the indicated gene signatures; analysis performed using WEB-based Gene Set Analysis Toolkit. (**B**) H&E image of the lung section from Fig. 3A), annotated with regions of LUSC and LUAD, and corresponding spatial plots depicting the distribution of the indicated mRNAs (neutrophil markers). (**C**) Representative H&E and immunohistochemistry (IHC) validation for MPO, CD11b (ITGAM) and LY6G in LUSC (top) and mucinous LUAD (bottom). The staining was repeated with lung sections from different mice ($n = 2$, biological replicates). Scale bar, 100 µm. Source data are available online for this figure

 © The Author(s)

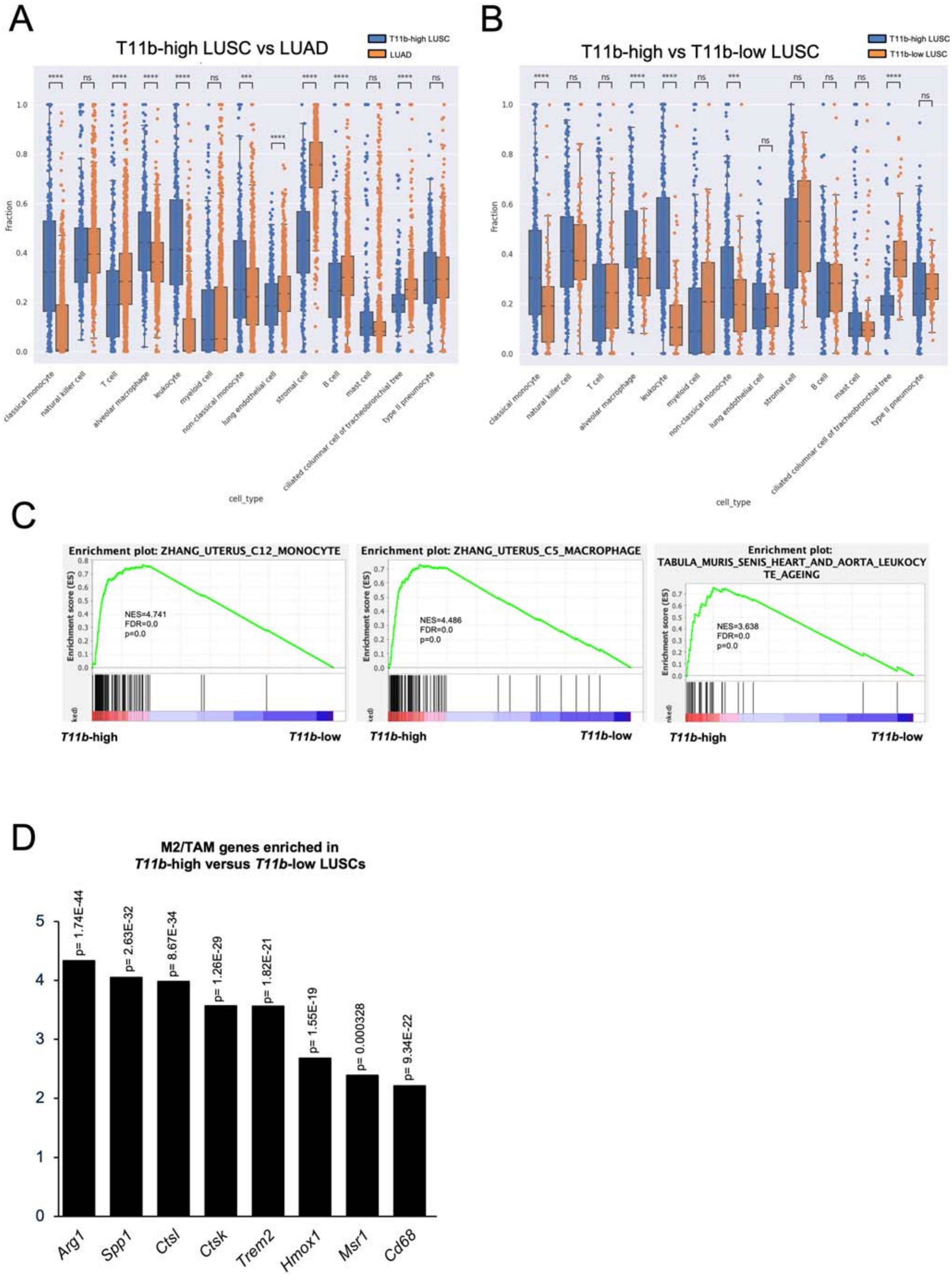
A
T11b-high LUSC vs LUAD
T11b-high LUSC
LUAD
Fraction
cell_type
B
T11b-high vs T11b-low LUSC
T11b-high LUSC
T11b-low LUSC
Fraction
cell_type
classical monocyte
natural killer cell
T cell
alveolar macrophage
leukocyte
myeloid cell
non-classical monocyte
lung endothelial cell
stromal cell
B cell
mast cell
ciliated columnar cell of tracheobronchial tree
type II pneumocyte
C
Enrichment plot: ZHANG_UTERUS_C12_MONOCYTE
NES=4.741
FDR=0.0
p=0.0
Enrichment plot: ZHANG_UTERUS_C5_MACROPHAGE
NES=4.486
FDR=0.0
p=0.0
Enrichment plot: TABULA_MURIS_SENIS_HEART_AND_AORTA_LEUKOCYTE_AGEING
NES=3.638
FDR=0.0
p=0.0
Enrichment score (ES)
T11b-high
T11b-low
D
M2/TAM genes enriched in T11b-high versus T11b-low LUSCs
p= 1.74E-44
p= 2.63E-32
p= 8.67E-34
p= 1.26E-29
p= 1.82E-21
p= 1.55E-19
p= 0.000328
p= 9.34E-22
Arg1
Spp1
Ctsl
Ctsk
Trem2
Hmox1
Msr1
Cd68

Figure EV6. *Tmprss11b*-high squamous tumors show enrichment for M2-like macrophage markers.

(**A**) Quantification of immune cell populations in *Tmprss11b*-high LUSC vs. LUAD using cell deconvolution analysis of the spatial data. A two-sided Mann–Whitney–Wilcoxon test was used for the statistical analysis (*T11b*-high LUSC *n* = 344, LUAD *n* = 1148, biological replicates), ****P = 7.96E-78 (Classical monocyte), ****P = 7.63E-16 (T cell), ****P = 4.74E-17 (Alveolar macrophage), ****P = 6.04E-133 (Leukocyte), ***P = 2.31E-05 (Non-classical monocyte), ****P = 2.84E-10 (Lung endothelial cell), ****P = 1.28E-111 (Stromal cell), ****P = 3.92E-09 (B cell), ****P = 4.41E-44 (Ciliated columnar cell of tracheobronchial tree). The box plots represent the distribution of values for each group, extending from the 25th percentile to the 75th percentile; *T11b*-high LUSC: Classical monocyte (minima=0.00035, median=0.32265, maxima=1, Q1 = 0.16193, Q3 = 0.52806), Natural killer cell (minima=0.04763, median=0.37114, maxima=0.82722, Q1 = 0.28091, Q3 = 0.49943), T cell (minima=0, median=0.18974, maxima=0.72977, Q1 = 0.06047, Q3 = 0.32819), Alveolar macrophages (minima=0.01914, median=0.44117, maxima=0.91769, Q1 = 0.32846, Q3 = 0.56415), Leukocyte (minima=0.9.87E-05, median=0.41385, maxima=1, Q1 = 0.27138, Q3 = 0.61336), Myeloid cell (minima=0.00012, median=0.04951, maxima=0.61895, Q1 = 0.00328, Q3 = 0.24955), Non-classical monocyte (minima=0.00036, median=0.25014, maxima=0.92007, Q1 = 0.13621, Q3 = 0.44976), Lung endothelial cell (minima=0, median=0.18464, maxima=0.52498, Q1 = 0.10807, Q3 = 0.27484), Stromal cell (minima=0, median=0.44905, maxima=0.94097, Q1 = 0.31863, Q3 = 0.56757), B cell (minima=0, median=0.24525, maxima=0.69056, Q1 = 0.13767, Q3 = 0.35883), Mast cell (minima=0.00046, median=0.09658, maxima=0.30038, Q1 = 0.06603, Q3 = 0.15977), Ciliated columnar cell of tracheobronchial tree (minima=0.05124, median=0.18792, maxima=0.34003, Q1 = 0.15953, Q3 = 0.23173), Type II pneumocyte (minima=0, median=0.28766, maxima=0.71305, Q1 = 0.19541, Q3 = 0.40246); LUAD: Classical monocyte (minima=0, median=0.00788, maxima=0.47288, Q1 = 0.00108, Q3 = 0.18980), Natural killer cell (minima=0.04933, median=0.39471, maxima=0.76680, Q1 = 0.31838, Q3 = 0.49774), T cell (minima=0.00029, median=0.28494, maxima=0.70883, Q1 = 0.19111, Q3 = 0.39820), Alveolar macrophages (minima=0.04437, median=0.36127, maxima=0.67967, Q1 = 0.28261, Q3 = 0.44143), Leukocyte (minima=0, median=0.00264, maxima=0.33064, Q1 = 0.00058, Q3 = 0.13260), Myeloid cell (minima=0, median=0.05014, maxima=0.64748, Q1 = 0.00252, Q3 = 0.26050), Non-classical monocyte (minima=0, median=0.22165, maxima=0.68314, Q1 = 0.10763, Q3 = 0.33783), Lung endothelial cell (minima=0, median=0.23560, maxima=0.51640, Q1 = 0.16346, Q3 = 0.30464), Stromal cell (minima=0.39036, median=0.75595, maxima=1, Q1 = 0.66414, Q3 = 0.84667), B cell (minima=0, median=0.30153, maxima=0.62486, Q1 = 0.22758, Q3 = 0.38650), Mast cell (minima=0, median=0.08179, maxima=0.20366, Q1 = 0.06479, Q3 = 0.12034), Ciliated columnar cell of tracheobronchial tree (minima=0.08851, median=0.25076, maxima=0.41768, Q1 = 0.21195, Q3 = 0.29424), Type II pneumocyte (minima=0, median=0.29401, maxima=0.63087, Q1 = 0.21685, Q3 = 0.38246). (**B**) Quantification of immune cell populations in *Tmprss11b*-high vs. *Tmprss11b*-low LUSC using cell deconvolution analysis of the spatial data. A two-sided Mann–Whitney-Wilcoxon test was used for the statistical analysis (*T11b*-high LUSC *n* = 344, *Tmprss11b*-low LUSC *n* = 104, biological replicates), ****P = 8.89E-08 (Classical monocyte), ****P = 3.22E-15 (Alveolar macrophage), ****P = 1.25E-32 (Leukocyte), ***P = 6.54E-05 (Non-classical monocyte), ****P = 2.42E-36 (Ciliated columnar cell of tracheobronchial tree). The box plots represent the distribution of values for each group, extending from the 25th percentile to the 75th percentile; *T11b*-high LUSC: Classical monocyte (minima=0.00029, median=0.30549, maxima=1, Q1 = 0.15744, Q3 = 0.49527), Natural killer cell (minima=0, median=0.41140, maxima=0.97370, Q1 = 0.26653, Q3 = 0.5490), T cell (minima=0, median=0.18983, maxima=0.81820, Q1 = 0.05084, Q3 = 0.35778), Alveolar macrophages (minima=0.00293, median=0.43913, maxima=0.91408, Q1 = 0.34461, Q3 = 0.57240), Leukocyte (minima=0.00068, median=0.40974, maxima=1, Q1 = 0.26157, Q3 = 0.62635), Myeloid cell (minima=4.55E-05, median=0.09014, maxima=0.65331, Q1 = 0.00217, Q3 = 0.26263), Non-classical monocyte (minima=1.67E-05, median=0.26527, maxima=0.85779, Q1 = 0.14324, Q3 = 0.42906), Lung endothelial cell (minima=0, median=0.17896, maxima=0.55746, Q1 = 0.09927, Q3 = 0.28255), Stromal cell (minima=0, median=0.44430, maxima=1, Q1 = 0.26373, Q3 = 0.62252), B cell (minima=0, median=0.24471, maxima=0.69114, Q1 = 0.14794, Q3 = 0.36522), Mast cell (minima=0, median=0.10069, maxima=0.30702, Q1 = 0.06993, Q3 = 0.16477), Ciliated columnar cell of tracheobronchial tree (minima=0.03638, median=0.19021, maxima=0.35185, Q1 = 0.15468, Q3 = 0.23355), Type II pneumocyte (minima=0, median=0.24117, maxima=0.67966, Q1 = 0.1531, Q3 = 0.36374); *Tmprss11b*-low LUSC: Classical monocyte (minima=0.00020, median=0.19138, maxima=0.60530, Q1 = 0.04595, Q3 = 0.26969), Natural killer cell (minima=0, median=0.37341, maxima=0.84796, Q1 = 0.29730, Q3 = 0.51756), T cell (minima=7.77E-05, median=0.24393, maxima=0.74889, Q1 = 0.10171, Q3 = 0.36058), Alveolar macrophages (minima=0.08051, median=0.30265, maxima=0.60637, Q1 = 0.23290, Q3 = 0.38229), Leukocyte (minima=0, median=0.10504, maxima=0.43837, Q1 = 0.03357, Q3 = 0.19549), Myeloid cell (minima=0.00048, median=0.20803, maxima=0.90453, Q1 = 0.00571, Q3 = 0.36524), Non-classical monocyte (minima=0.00013, median=0.19676, maxima=0.60907, Q1 = 0.08942, Q3 = 0.29728), Lung endothelial cell (minima=0, median=0.18352, maxima=0.39994, Q1 = 0.10900, Q3 = 0.23955), Stromal cell (minima=0.10516, median=0.53043, maxima=0.88675, Q1 = 0.32928, Q3 = 0.69341), B cell (minima=0.00077, median=0.28140, maxima=0.64062, Q1 = 0.17045, Q3 = 0.35852), Mast cell (minima=0, median=0.09511, maxima=0.21496, Q1 = 0.06911, Q3 = 0.12745), Ciliated columnar cell of tracheobronchial tree (minima=0.13684, median=0.37548, maxima=0.66299, Q1 = 0.30862, Q3 = 0.45037), Type II pneumocyte (minima=0.06470, median=0.26157, maxima=0.47461, Q1 = 0.21841, Q3 = 0.32089). (**C**) Gene set enrichment analysis (GSEA) of the *Tmprss11b*-high versus low LUSC spatial data with normalized enrichment scores (NES), false discovery rate (FDR) and *p* values for the indicated immune cell gene signatures. The nominal *P* and FDR values were obtained from the "GSEA Preranked" tool (from Broad Institute) using a weighted scoring scheme. Gene sets were evaluated based on the default normalized enrichment score method, and statistical significance was determined by bootstrapping with 1000 permutations. (**D**) Bar graph representing top M2-like/TAM genes from the differential gene expression (DEG) analysis of *Tmprss11b*-high versus low LUSC spatial transcriptomics data. Differential gene expression was calculated using Seurat's FindAllMarkers function, and direct comparisons between two classes and corresponding *P* values were obtained using Seurat's FindMarkers function with the Wilcoxon rank-sum test with Bonferroni *P* value correction. Source data are available online for this figure

 © The Author(s)
